# Plant‐Based Food Preferences Rich in Polyphenols and Their Causal Effects on Inflammatory Bowel Disease

**DOI:** 10.1002/fsn3.71453

**Published:** 2026-01-14

**Authors:** Tinghui Yue, Qiang Su, Song He, Yuhua Liu, Dan Huang, Zhenxiang An, Yuanli He

**Affiliations:** ^1^ The First Clinical Medical College Guizhou University of Traditional Chinese Medicine Guiyang Guizhou China; ^2^ Department of Gastroenterology, First Affiliated Hospital of Guizhou University of Traditional Chinese Medicine Guiyang Guizhou China; ^3^ Department of Traditional Chinese Medicine Classics, First Affiliated Hospital of Guizhou University of Traditional Chinese Medicine Guiyang Guizhou China; ^4^ Department of Cadre Health Care, First Affiliated Hospital of Guizhou University of Traditional Chinese Medicine Guiyang Guizhou China

**Keywords:** Crohn's disease, dietary polyphenols, disease prevention, inflammatory bowel disease, mendelian randomization, non‐communicable chronic diseases, polyphenol‐rich plant foods, ulcerative colitis

## Abstract

Inflammatory bowel disease (IBD), including ulcerative colitis (UC) and Crohn's disease (CD), represents a major non‐communicable chronic disease with increasing global prevalence. Dietary polyphenols from plant‐based foods have demonstrated potent anti‐inflammatory, antioxidant, and immunomodulatory properties that may play a crucial role in preventing and managing chronic inflammatory conditions. While dietary modifications are frequently advocated in the management of IBD, the intrinsic role of dietary preferences for polyphenol‐rich plant foods remains insufficiently understood. By exploring these links, researchers can develop targeted dietary plans that offer new ways to prevent IBD onset and improve treatment outcomes. This study utilized genome‐wide association study (GWAS) summary statistics pertaining to 65 plant‐based food preferences (sample size ranging from 115,868 to 159,579) and two subtypes of IBD from the most recent FinnGen database (Release 11): UC (*n* = 438,538) and CD (*n* = 434,250). The principal causal inference was performed using inverse variance weighted (IVW) in the two‐sample Mendelian randomization (MR), with additional analyses conducted using MR‐Egger and weighted median methods. To ensure robustness, we conducted extensive sensitivity analyses, incorporating Cochran's *Q* test, the MR‐Egger intercept test, the MR‐PRESSO test, and leave‐one‐out analysis. The directionality of the identified associations was confirmed through Steiger filtering. Additionally, we utilized multivariable Mendelian randomization (MVMR) to evaluate the direct effects of dietary factors on IBD. Colocalization analysis was also employed to identify shared genetic architecture between preferences for polyphenol‐rich edible plants and both UC and CD. Following the application of the Bonferroni correction, this MR analysis identified significant genetic associations between preferences for polyphenol‐rich plant foods and IBD risk. For CD, preferences for orange juice (rich in flavonoids such as hesperidin and naringin, OR: 0.329, 95% CI: 0.146–0.743, *p* = 0.007) and lentils/beans (abundant in polyphenols and flavonoids, OR: 0.178, 95% CI: 0.048–0.658, *p* = 0.010) demonstrated strong protective effects. Using MVMR techniques, we found that UC risk increased among those with aniseed preferences (OR: 3.942, 95% CI: 1.075–14.451, *p* = 0.039), while individuals who favored melon (rich in cucurbitacins and antioxidants, OR: 0.168, 95% CI: 0.031–0.910, *p* = 0.039) showed decreased likelihood of developing CD. Through colocalization analysis, we identified shared genetic signals between UC and aniseed preference (PP.H4 = 99.52%), supporting their biological connection. These findings suggest that the anti‐inflammatory and antioxidant properties of dietary polyphenols may mediate protective effects through modulation of gut microbiota, enhancement of intestinal barrier function, and regulation of immune responses. Our study provides robust genetic evidence for causal relationships between preferences for polyphenol‐rich plant foods and IBD risk, highlighting the potential role of dietary polyphenols in preventing non‐communicable chronic inflammatory diseases. These biological insights suggest new directions for improving disease screening and developing targeted dietary interventions for IBD prevention and management.

## Graphical abstract

This Mendelian randomization study investigated causal relationships between polyphenol‐rich plant food preferences and inflammatory bowel disease (IBD) risk using genome‐wide association study data. Six dietary preferences were significantly associated with IBD risk. Notably, multivariable Mendelian randomization analysis revealed that genetically predicted preference for aniseed independently increased ulcerative colitis risk, while preferences for orange juice, lentils/beans, and melon were inversely associated with Crohn's disease risk, suggesting their potential role in IBD pathogenesis and prevention.

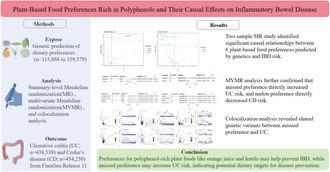

## 1 Introduction

Inflammatory bowel disease (IBD) has emerged as a mounting global health challenge and represents a significant non‐communicable chronic disease, with its impact extending far beyond the gut (Zhong et al. 2024). The two main forms—ulcerative colitis (UC) and Crohn's disease (CD)—can transform patients' lives, leading to chronic disability and sharply reduced quality of life. Recent global burden studies reveal that IBD now affects over 6.8 million people worldwide, with an alarming 83.8% increase in prevalence (Park et al. 2023). The disease rates have climbed significantly in emerging industrial nations, indicating that environmental elements shape its progression. Despite significant advances in understanding IBD, its underlying mechanisms remain surprisingly elusive. Research has established that genes, environmental exposures, and immune function work together in disease development, but identifying the key triggers that lead to illness remains a scientific challenge. This knowledge gap is particularly concerning given the growing burden of IBD on healthcare systems and its peak onset during people's most productive year (Jairath and Feagan 2020).

Recent research has turned the spotlight on diet as a key environmental factor in IBD pathogenesis. Dietary polyphenols, predominantly found in plant‐based foods such as fruits, vegetables, legumes, and whole grains, are bioactive compounds with well‐documented anti‐inflammatory, antioxidant, and immunomodulatory properties. Growing evidence links specific dietary patterns to IBD risk, with ultra‐processed foods and pro‐inflammatory diets increasing disease susceptibility (Marino et al. 2021; Vissers et al. 2022). Polyphenolic compounds have been shown to modulate multiple pathways implicated in IBD pathogenesis, including the suppression of pro‐inflammatory cytokines, enhancement of intestinal barrier function, and beneficial modulation of gut microbiota composition. However, most studies have focused on broad dietary patterns rather than individual food preferences. Even more intriguing is the emerging evidence of gene–diet interactions in IBD development (Magro et al. 2024), suggesting that genetic factors might influence both food choices and disease susceptibility through shared pathways. The relationship between food preferences and IBD risk takes on new significance when we consider the role of early‐life dietary habits.

Our decision to focus on “food preference” rather than “actual intake” as the primary exposure was driven by both methodological considerations and current data constraints. While actual intake provides a direct measure of consumption, quantifying long‐term dietary habits relies heavily on self‐reported questionnaires, which are often prone to measurement error and recall bias. More importantly, from a genetic epidemiology perspective, the availability of high‐quality data is a deciding factor. Currently, large‐scale GWAS summary statistics representing the precise quantitative intake of specific polyphenol‐rich foods are limited. In contrast, GWAS data for food preferences, such as those derived from massive cohorts like the UK Biobank, offer the robust sample size and statistical power required for reliable Mendelian randomization (MR) analysis. Therefore, utilizing food preference serves as a valid and accessible genetic proxy that reflects an individual's long‐term dietary inclination.

The relationship between dietary preferences for polyphenol‐rich plant foods and IBD risk takes on new significance when we consider their potential role in chronic disease prevention. These preferences, shaped by both genetic and environmental factors, could serve as early indicators of disease susceptibility. Understanding this connection could revolutionize our approach to IBD prevention and early intervention, especially given the current limitations of traditional screening methods. This complex interplay between genetics, preferences for polyphenol‐rich edible plants, and IBD risk raises intriguing questions about causality. While we know that dietary polyphenols can influence gut inflammation through antioxidant and anti‐inflammatory mechanisms (Christensen et al. 2024), the direction of causality between edible plant preferences and IBD development remains unclear. Could genetic variants that influence edible plant preferences also affect IBD susceptibility? Or do early inflammatory processes shape edible plant preferences before clinical symptoms appear?

Traditional observational studies exploring these diet‐disease connections face inherent limitations. Confounding factors—from lifestyle habits to socioeconomic status—can mask true relationships, while reverse causation remains a persistent concern (Li 2023). For instance, patients might change their eating habits in response to early, subtle disease symptoms, making it challenging to determine whether dietary preferences contribute to disease onset or merely reflect its early manifestations. Recent advances in genetic epidemiology offer a promising solution to these methodological challenges. MR, often described as nature's randomized controlled trial, provides a powerful tool for probing causal relationships (Su et al. 2024). This approach leverages genetic variants as instrumental variables, capitalizing on the random allocation of genes at conception—a process unaffected by typical confounders or disease status.

Building on this foundation, we employed a sophisticated multi‐pronged approach. Two‐sample MR allowed us to examine the causal effects of specific edible plant preferences on IBD subtypes using separate GWAS datasets, enhancing statistical power while minimizing bias (Davey Smith and Hemani 2014). To capture the complex nature of these relationships, we complemented this with multivariable Mendelian randomization (MVMR), which helped disentangle the effects of correlated dietary preferences (Cui et al. 2024). Recognizing that genetic signals often cluster in specific genomic regions, we further employed colocalization analysis. This method helps distinguish between shared genetic effects and mere proximity, providing crucial insights into the biological mechanisms linking edible plant preferences to IBD (Yun et al. 2023). By integrating these complementary approaches, we aimed to build a more complete picture of how genetic variants might influence both dietary choices and IBD risk through shared biological pathways.

Here, we set out to unravel the complex relationship between edible plant preferences and IBD risk through a comprehensive genetic approach. Our study had three key aims: First, to identify potential causal links between specific edible plant preferences and IBD development using two‐sample MR. Second, to explore how different dietary preferences might work together to influence disease risk through MVMR analysis. Finally, to pinpoint shared genetic architecture between edible plant choices and IBD through colocalization analysis, potentially revealing new biological pathways.

This work represents a crucial step toward understanding how our genetic makeup shapes both our preferences for polyphenol‐rich foods and disease susceptibility. Beyond advancing our theoretical understanding of how dietary polyphenols contribute to non‐communicable chronic disease prevention, our findings could open new avenues for personalized IBD prevention strategies. By identifying individuals at higher risk based on their genetically influenced preferences for polyphenol‐rich plant foods, we might finally move from reactive treatment to proactive prevention in IBD care.

## 2 Methods and Materials

### 2.1 Study Design

**FIGURE 1:**
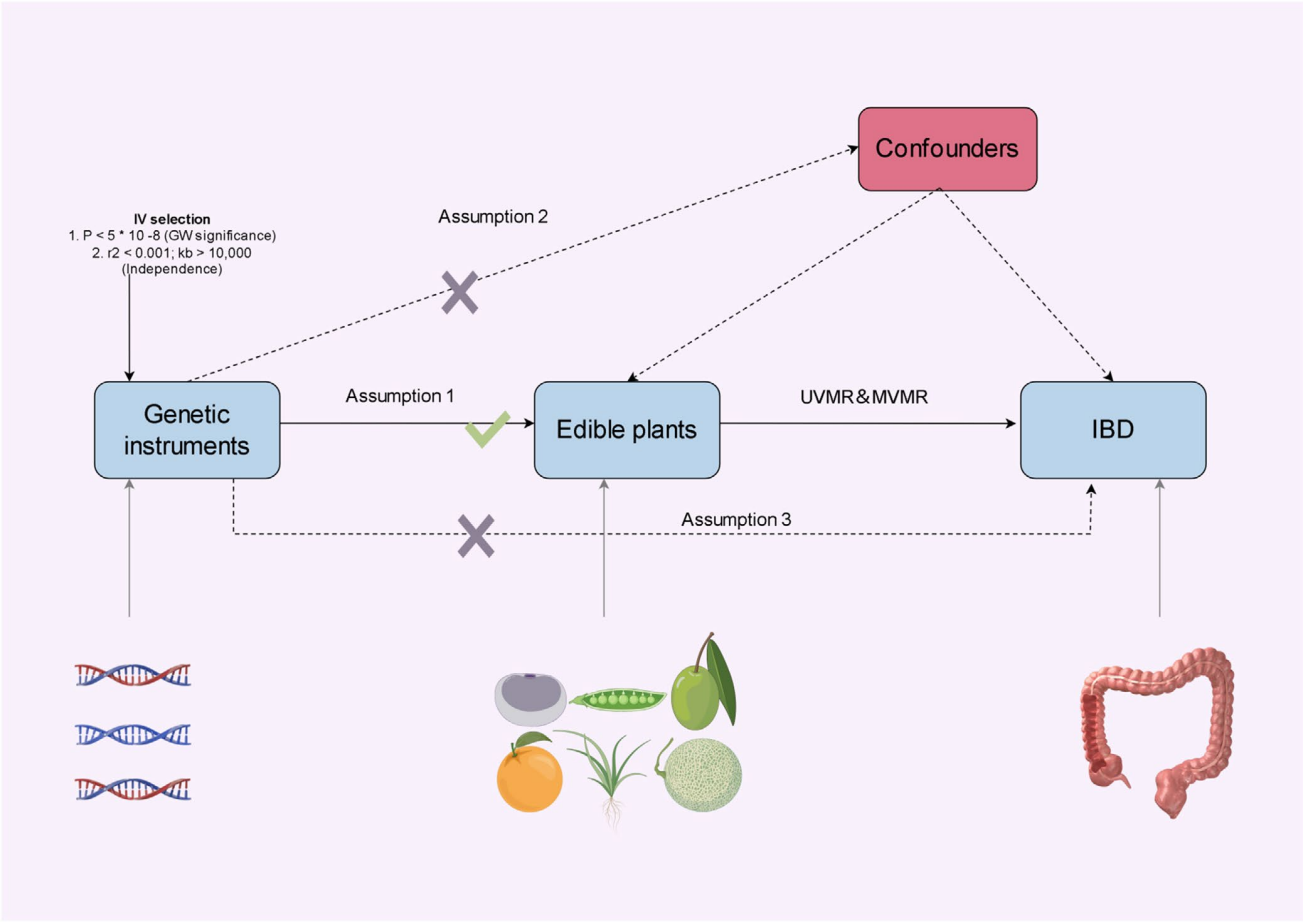
The flowchart presented in this study delineates the core assumptions underpinning the MR analysis. The principal aim of performing two‐sample MR and MVMR analyses is to examine the potential causal relationship between edible plant preferences and the susceptibility to IBD (UC and CD). The three key assumptions include (1) strong instrumental variables: Genetic instruments (SNPs) must have strong associations with the studied plant preferences; (2) independence from confounders: Instruments should be independent of potential confounding variables; and (3) exclusion restriction: The influence on outcomes must occur solely through food preference pathways.

To investigate potential causal relationships between edible plant preferences and IBD subtypes (UC and CD), we developed a comprehensive analytical framework combining two‐sample MR (UVMR), MVMR, and colocalization analyses. Our approach rests on three fundamental assumptions essential for valid MR inference (Yun et al. 2023). The genetic markers (SNPs) chosen as instrumental variables needed to meet three criteria: strong associations with the studied plant preferences, independence from potential confounding variables, and influence on outcomes strictly through food preference pathways rather than other biological mechanisms (Figure 1). We leveraged publicly available GWAS summary statistics, implementing our analytical pipeline in R (version 4.2.1). Specifically, we employed the TwoSampleMR package for primary analyses, supplemented by MR‐PRESSO for horizontal pleiotropy assessment, and the coloc package for shared genetic architecture investigation (Burgess et al. 2019). The study's reliance on published GWAS summary data meant that further ethical clearance was not necessary. This methodological approach allows for robust causal inference while accounting for potential genetic confounding and pleiotropy.

### 2.2 GWAS Data of Edible Plant Preferences

Recent GWAS have identified genetic associations for 65 distinct edible plant preference phenotypes (May‐Wilson et al. 2022), offering new insights into how dietary choices connect with specific IBD manifestations (UC and CD). The genetic data analyzed came from 11 combined cohorts, including 161,625 participants of European descent. This GWAS dataset, now accessible in the GWAS Catalog with complete documentation in its original publication, marks the most extensive genetic investigation of food preferences conducted so far. We list the complete set of 65 plant‐based food preferences, their standardized names, GWAS identifiers, and abbreviated forms in Table S1.

### 2.3 GWAS Data for UC and CD

Starting in 2017, FinnGen emerged as a collaborative venture between public institutions and private partners, connecting genetic data from Finnish biobanks to national health registries. This large‐scale project harnesses Finland's genetically distinct population structure to identify disease‐linked genetic variants (Kurki et al. 2023). For our analysis, we utilized summary‐level genetic association estimates for UC and CD from the latest publicly available FinnGen release (R11). The dataset comprised 261 UC cases (ICD‐10: K51) and 438,277 controls, alongside 1870 CD cases (ICD‐10: K50) and 432,380 controls (Kurki et al. 2023). The statistical models for genetic associations accounted for various factors: participant demographics (age and sex), genetic ancestry markers, and technical variation across sample processing groups.

### 2.4 IVs Selection

To validate assumption (1), we implemented a rigorous IV selection protocol for edible plant preferences. We applied a genome‐wide significance threshold of 5 × 10^−8^ for SNP selection to ensure robust genetic instruments. Linkage disequilibrium (LD) pruning was performed to remove correlated SNPs (*R* ^2^ > 0.001, kb > 10,000), following established protocols (Zheng et al. 2020). To ensure robust genetic instruments, we computed *r* ^2^ values and *F*‐statistics for individual SNPs, filtering out those with *F*‐statistics under 10 to maintain analytical validity. The genetic instrument selection process excluded SNPs that showed outcome associations at *p* < 1 × 10^−5^, reducing potential cross‐trait effects. Data quality control included removing palindromic variants and SNPs with allele frequency inconsistencies between datasets (Richardson et al. 2019; Zheng et al. 2020). The selection process removed SNPs showing outcome associations at *p* < 1 × 10^−5^. We restricted our causal estimates to metabolites with a minimum of two valid genetic markers, ensuring analytical reliability (Gill et al. 2019).

### 2.5 Statistical Analysis and Secondary Analysis

The primary statistical approach utilized inverse‐variance weighted (IVW) methods to examine how plant food choices affect UC and CD development. Heterogeneity testing guided our model selection: fixed‐effects models for homogeneous results, and random‐effects models when significant variation existed. The analysis combined individual genetic variant effects through Wald ratio meta‐analysis, assuming the genetic markers influenced outcomes only through dietary preferences (Yavorska and Burgess 2017). To validate our initial findings, we analyzed metabolites with significant IVW results (*p* < 0.05) using three additional statistical methods: MR‐Egger regression, weighted median, and weighted mode approaches. The weighted median analysis provides reliable estimates even when half of the genetic markers fail to meet validity criteria. The MR‐Egger approach serves two key functions: detecting direct genetic effects on outcomes and generating reliable estimates when the InSIDE criterion (Instrument Strength Independent of Direct Effect) holds (Chen et al. 2022). This method remains effective even in scenarios where conventional instrument validity is questioned. The weighted mode approach examines the distribution of genetic effects, focusing on the most frequently observed estimate. This method produces valid results when the largest group of genetic markers yielding similar estimates represents true causal relationships, providing an additional safeguard against direct genetic effects (Yang et al. 2024).

Further analytical steps evaluated the consistency and reliability of our findings. Using Cochran's *Q* test, we examined variation among the identified genetic markers, with *Q* statistics and *I* ^2^ values providing quantitative measures of result heterogeneity (Burgess et al. 2015). Variation across genetic markers was quantified using *I* ^2^, calculated as [*Q*‐ (*K*‐1)]/*Q*, where *K* represents the total genetic variants and *Q* the corresponding test statistic. To examine potential direct genetic influences, we employed two complementary approaches: the MR‐Egger intercept method and MR‐PRESSO global test. Intercept values near zero suggested pathway‐specific genetic effects. Using MR‐PRESSO methodology, we identified and removed genetic variants showing unusual effect patterns. The analytical process was then repeated with the refined dataset, with additional testing rounds confirming the effectiveness of outlier removal (Verbanck et al. 2018). Sensitivity analyses incorporated leave‐one‐out (LOO) assessments, systematically removing individual SNPs to evaluate their influence on overall causal estimates (Flatby et al. 2023). Cross‐method comparisons were conducted to verify the robustness of our findings. Agreement among different analytical approaches provided additional confidence in the validity of our conclusions.

The application of Steiger directionality tests helped validate our analytical framework. This testing approach confirmed that the selected genetic markers exhibited stronger associations with exposure than outcome variables, strengthening the foundation of our causal analyses. Implementation of the Steiger filtering procedure strengthened our analytical framework by validating the proposed causal direction. This methodological step helped minimize potential reverse causation effects, enhancing the robustness of our findings (Hemani et al. 2017).

In summary, we implemented a comprehensive, multi‐step analytical framework to identify edible plant preferences with potential causal influences on UC and CD development. We established four essential criteria for result validation: (1) evidence of statistical significance through IVW analysis (*p* < 0.05); (2) directional and quantitative consistency across analytical approaches; (3) absence of significant heterogeneity and horizontal pleiotropy in effect estimates; and (4) result stability following sequential removal of individual genetic variants. Following initial analyses, positive findings underwent multiple testing correction according to the previously described methodologies and protocols. Statistical significance was determined using Bonferroni‐adjusted *p*‐values, calculated as *p* < 0.05/*N*, where *N* represents the number of tests performed (Wu et al. 2020). *N* = 4 represents the four complementary MR analytical methods employed (IVW, MR‐Egger, Weighted Median, and Weighted Mode). Consequently, the threshold for robust statistical significance was set at *p* < 0.0125 (0.05/4). Associations with PP‐values between 0.0125 and 0.05 were considered nominally significant, suggesting potential causal links warranting further verification.

### 2.6 Confounding Analysis and MVMR Analysis

Our analytical validation process included a comprehensive assessment of horizontal pleiotropy and the identification of genetic variants potentially violating core assumptions. While our methodology was thorough, we acknowledge that some genetic markers might introduce residual confounding effects. To evaluate this possibility, we conducted systematic searches through the GWAS Catalog (https://www.ebi.ac.uk/gwas/), examining potential associations between our selected instrumental variables and known risk factors for UC and CD. These factors included smoking patterns, alcohol intake, educational attainment, post‐appendectomy status (Le Berre et al. 2023), and the diseases themselves. To assess result robustness, we conducted additional analyses after excluding genetic variants that demonstrated significant associations (*p* < 1 × 10^−5^) with either the identified confounding factors or primary outcomes.

Key MR assumptions stipulate that genetic variants should influence a single risk factor exclusively. However, certain variants demonstrate associations with multiple phenotypic traits, a genetic phenomenon referred to as pleiotropy. For these complex scenarios, MVMR methodology provides an analytical framework that addresses the interplay between genetic variants and their multiple associated exposures (Sanderson 2021). The MVMR framework offers methodological advantages over UVMR by addressing fundamental challenges related to independence, dominance, and effect comparability. This advanced approach enables the differentiation of individual exposure effects, contrasting with UVMR's assessment of aggregate exposure impacts. The analytical framework integrates MVMR methodology with LASSO regression techniques to evaluate potential interactions among characterized plant‐based dietary patterns (Xiao et al. 2022). In the context of MVMR analysis, the IVW methodology incorporates a regression framework where genetic variants associated with exposures are analyzed against outcomes, weighted by inverse outcome variance. Potential pleiotropic effects within instrumental variables were addressed through outlier detection and removal using the MR‐PRESSO analytical framework. The application of LASSO regression methodology enabled the identification and removal of collinear exposures, enhancing the reliability of causal estimates (Grant and Burgess 2021).

### 2.7 Colocalization Analysis

The coloc R package was employed for colocalization analyses to investigate the relationship between edible plant preferences identified in UC and CD (Liu et al. 2019). This analytical strategy enabled the detection of shared causal variants across specific genomic regions, potentially explaining the mechanistic links between these factors and both phenotypic manifestations. Using a Bayesian statistical framework, the colocalization analysis computes posterior probabilities for five hypotheses (H0–H4): absence of trait associations (H0); association limited to trait 1 (H1); association limited to trait 2 (H2); associations with both traits through independent causal variants (H3); and associations with both traits mediated by a shared causal variant (H4) (Foley et al. 2021). All analyses were executed using default prior probability parameters (p1 = 1 × 10^−4^, p2 = 1 × 10^−4^, p12 = 1 × 10^−5^). Posterior probability H4 exceeding 80% in our colocalization analyses provided robust evidence for shared causal variants within specific genomic regions that influence both gene expression and disease susceptibility in UC and CD (Yun et al. 2023).

## 3 Results

### 3.1 Preliminary Analysis

#### 3.1.1 Identification of SNPs and Initial Screening

**FIGURE 2:**
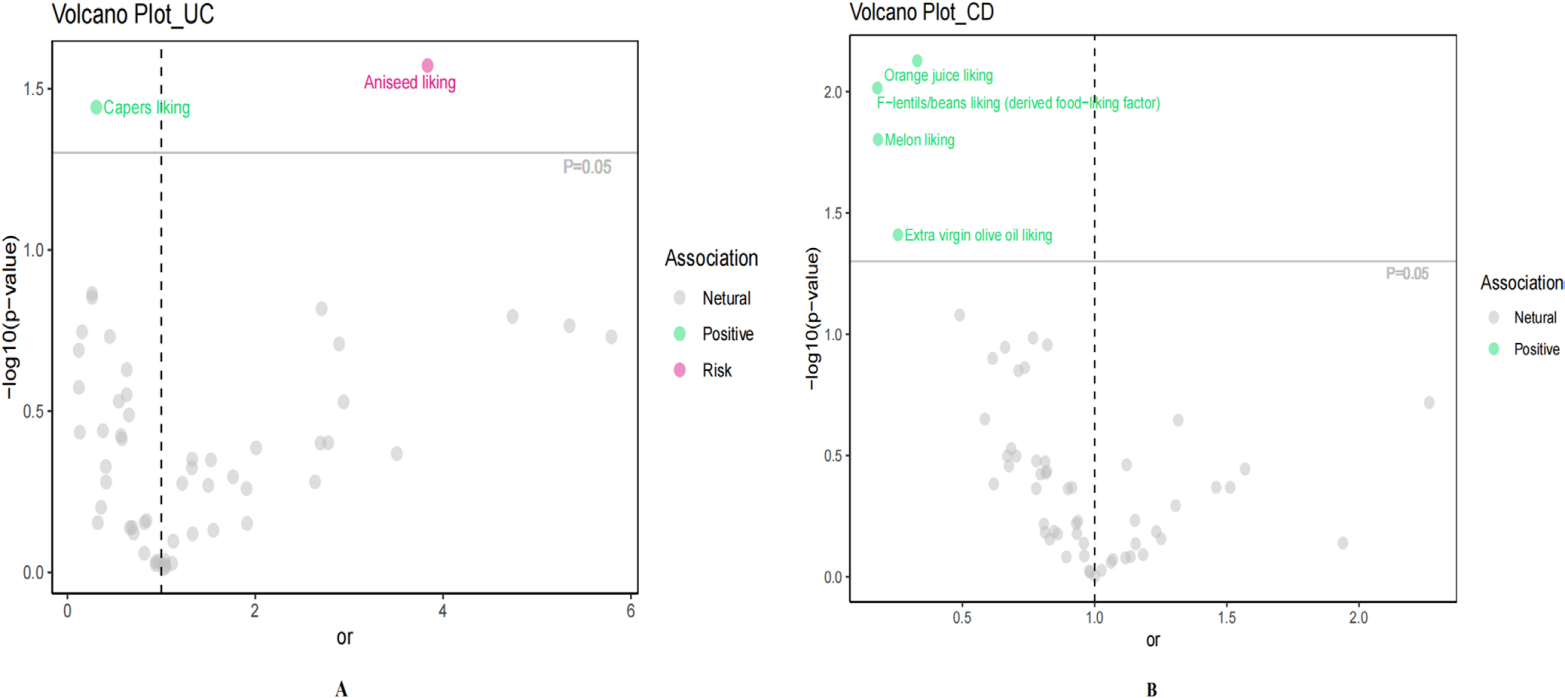
Volcano plot of edible plant preferences associated with the risk of developing UC and CD.

**FIGURE 3:**
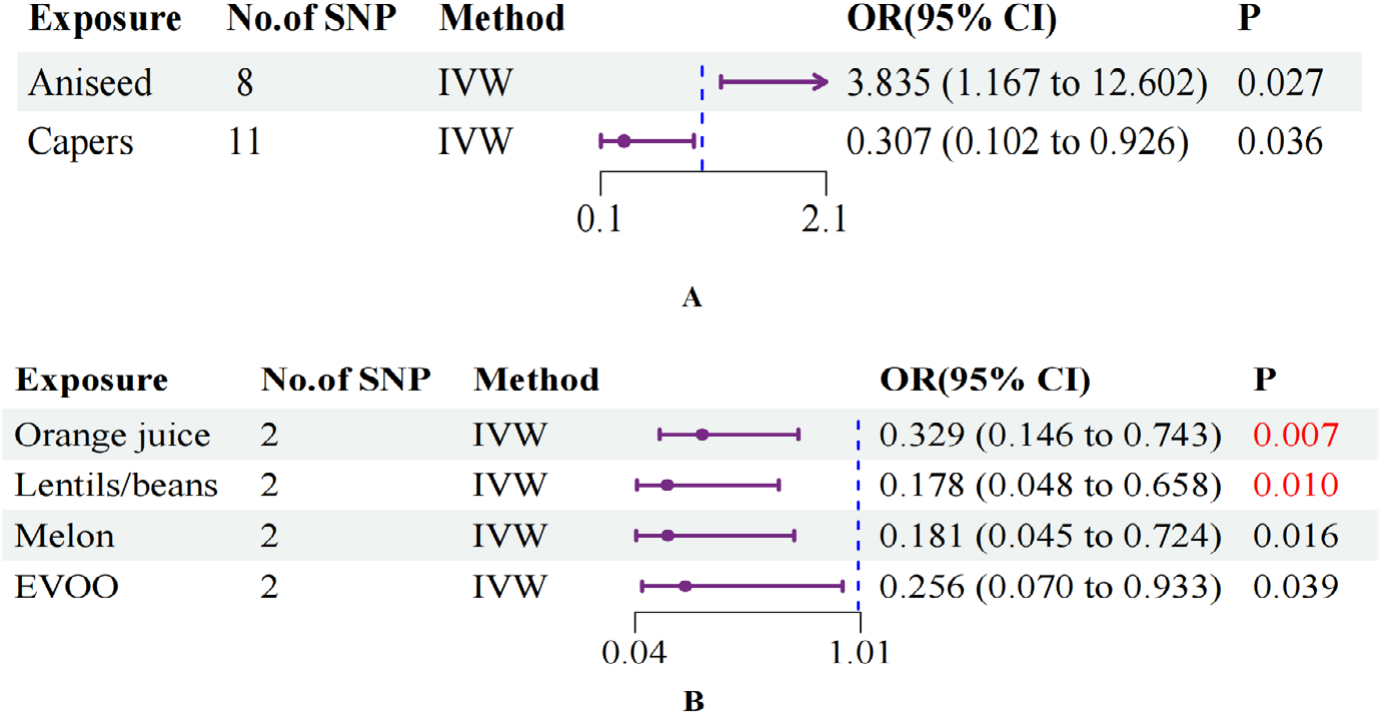
Forest plot of edible plant preferences versus UC (A) and CD (B) risk. aniseed, aniseed liking; Capers, Capers liking; orange juice, orange juice liking; CI, confidence interval; EVOO, extra virgin olive oil liking; IVW, inverse variance weighted; lentils/beans, *F*‐lentils/beans liking (derived food‐liking factor); OR, odds ratio.

A genome‐wide significance threshold of *p* < 5 × 10^−8^ yielded 808 SNPs associated with edible plant preferences. The suitability of these variants as IVs for MR analyses was supported by robust *F*‐statistics (range: 40.67–1794.67). Comprehensive IV characteristics are documented in Table S2. Prior to implementing MR analyses, we performed systematic outlier screening through confounding factor evaluation and MR‐PRESSO application, with results detailed in Table S3. In this systematic MR investigation exploring the relationships between 65 edible plant preference phenotypes and UC/CD manifestations, we identified significant causal relationships using the IVW method. After applying stringent statistical corrections, including Bonferroni adjustment (*p* < 0.0125), two edible plant preferences demonstrated strong causal associations with UC development, while four showed robust causal relationships with CD development (Table S4; Figures 2 and 3).

#### 3.1.2 Mendelian Randomization Analysis and Sensitivity Tests

Our genetic analyses uncovered significant correlations between specific food preferences and IBD susceptibility. In UC analyses, genetic disposition toward Aniseed preference exhibited a positive correlation (odds ratio [OR]: 3.835, 95% confidence interval [CI]: 1.167–12.602, *p* = 0.027), whereas Capers preference showed a protective association (OR: 0.307, 95% CI: 0.102–0.926, *p* = 0.036) (Figure 3A). For CD, following multiple testing adjustment, two dietary preferences demonstrated strong protective associations: Orange juice preference (OR: 0.329, 95% CI: 0.146–0.743, *p* = 0.007 < 0.0125) and the *F*‐lentils/beans preference factor (OR: 0.178, 95% CI: 0.048–0.658, *p* = 0.010 < 0.0125). Two additional preferences showed potentially protective effects, though not surviving multiple testing correction: Melon preference (OR: 0.181, 95% CI: 0.045–0.724, *p* = 0.016) and Extra virgin olive oil (EVOO) preference (OR: 0.256, 95% CI: 0.070–0.933, *p* = 0.039) (Figure 3B). Results from complementary MR approaches (MR‐Egger, Weighted Median, and Weighted Mode) showed strong alignment with IVW estimates, providing additional validation for our findings across diverse analytical frameworks.

To provide a clearer clinical interpretation of the OR, we calculated the proportion of food preference variance explained by the genetic instrumental variables (*R* ^2^), which ranged from 0.000255 to 0.011 (Table S2). These values indicate that a small but significant portion of the variance in food preferences can be attributed to genetic influences. For instance, an OR of 3.842 for aniseed preference suggests that individuals with a genetic predisposition to prefer aniseed are more than three times as likely to develop UC compared with those without this predisposition. This translates into a meaningful difference in actual preference as influenced by the underlying genetic architecture. Understanding these effect sizes can help contextualize the clinical implications of our findings and guide further dietary recommendations.

Multiple sensitivity assessments validated the robustness of our results. Pleiotropy analyses and MR‐PRESSO evaluations indicated the absence of horizontal pleiotropy across all SNPs (Table S5), validating our IV selection. The reliability of our findings was further supported by heterogeneity assessments, which showed no significant variation in MR outcomes (Table S6). LOO analyses revealed no substantial bias sources (Figure S1), and Steiger directionality testing of all IVs confirmed the absence of reverse causality (Table S7). Figures S2–S4 present additional visualization analyses.

### 3.2 MVMR Analysis

To disentangle the complex relationships among edible plant preferences, we employed MVMR methodology. This analytical framework enabled assessment of their independent contributions to UC and CD susceptibility. The incorporation of LASSO regression within the MVMR approach strengthened result reliability while effectively controlling for potential confounding factors.

**FIGURE 4:**
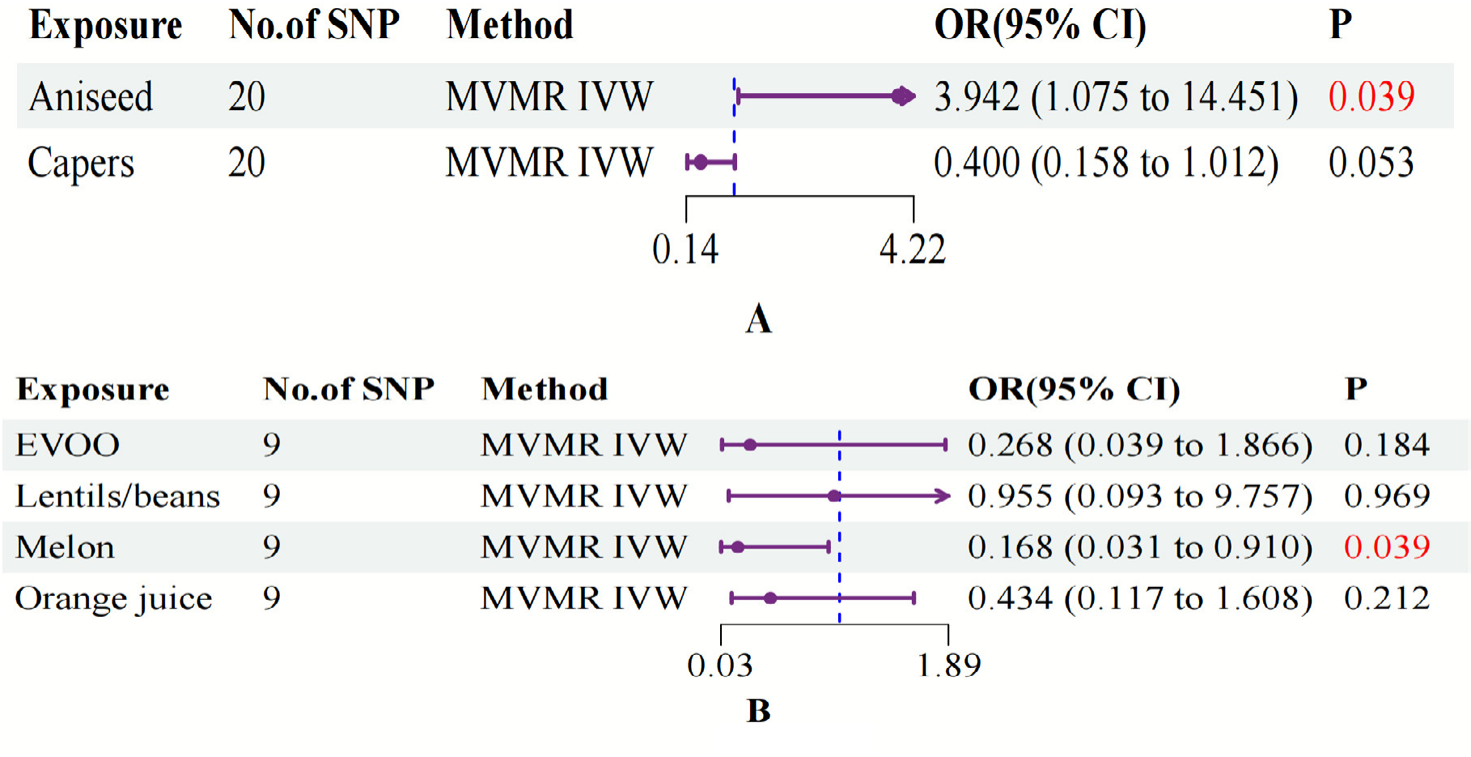
Forest plot illustrating the outcomes of the MVMR analysis (A: UC; B: CD). Capers, Capers liking; CI, confidence interval; aniseed, aniseed liking; EVOO, extra virgin olive oil liking; lentils/beans, F‐lentils/beans liking (derived food‐liking factor); MVMR IVW, multivariable Mendelian randomization inverse variance weighted; OR, odds ratio; orange juice, orange juice liking.

MVMR implementation revealed two edible plant preference factors that demonstrated robust associations with IBD susceptibility, persisting after adjustment for other dietary preferences. Genetic predisposition to aniseed liking showed a positive association with UC risk (OR: 3.842, 95% CI: 1.075–14.451, *p* = 0.039) (Figure 4A), while genetic predisposition to melon liking demonstrated an inverse relationship with CD risk (OR: 0.168, 95% CI: 0.031–0.910, *p* = 0.039) (Figure 4B). These findings suggest independent effects of genetic predispositions to aniseed and melon preferences on UC and CD susceptibility, respectively. The odds ratios indicate that individuals with genetic tendencies toward these specific food preferences may exhibit altered disease risk profiles (Table S8, Figure 4).

### 3.3 Colocalization Analysis

**FIGURE 5:**
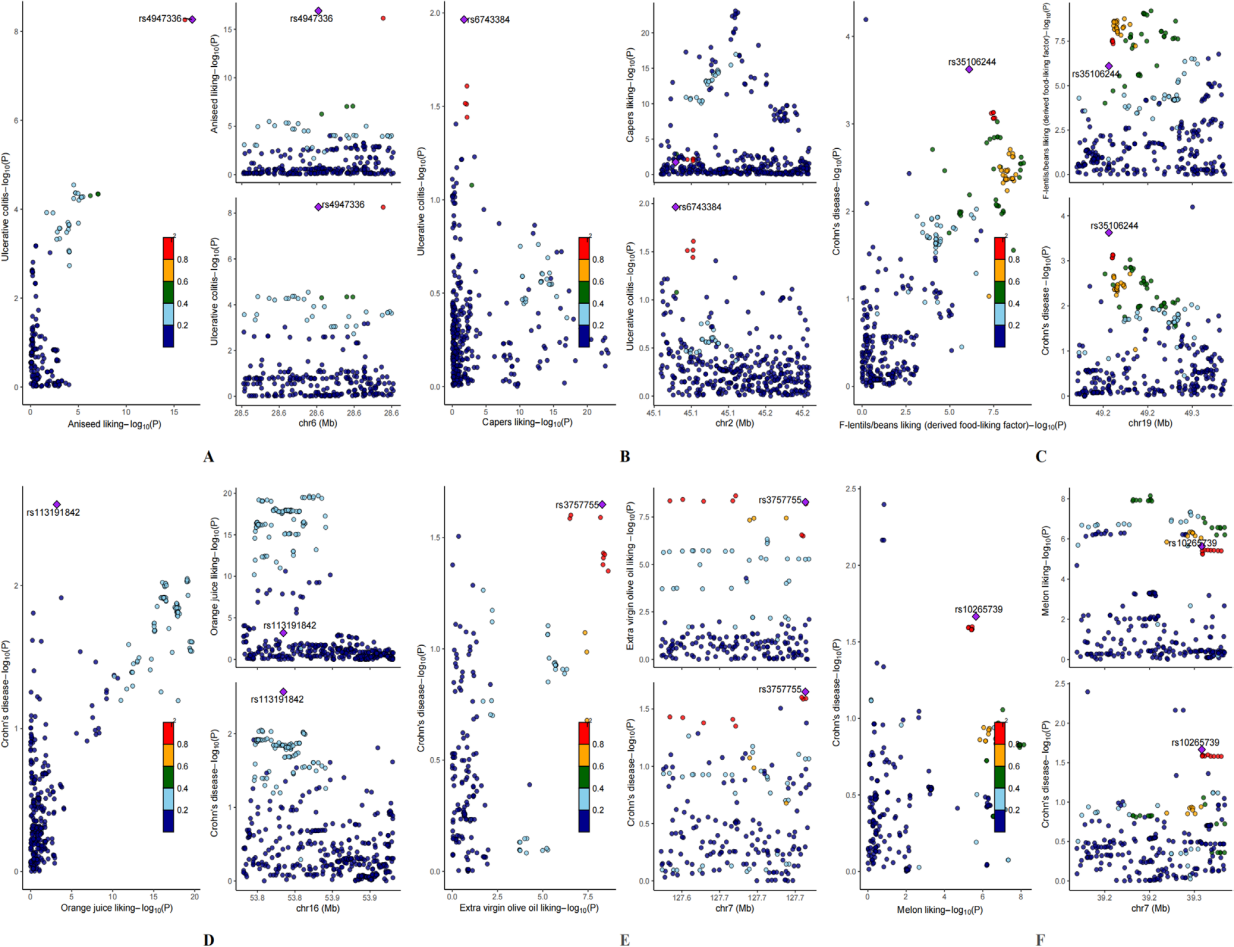
Regional association plot and linkage disequilibrium (LD) analysis of significant genetic loci. The lead SNP (rsid), identified by the lowest combined P‐value from both datasets, is highlighted.

This colocalization investigation yielded strong evidence for shared genetic foundations between aniseed preference and UC susceptibility, with a posterior probability of 99.52% (PP.H4). The lead SNP rs4947336, located in the 6p21.33 region, demonstrated strong colocalization signals, suggesting a common genetic basis for both traits. This genomic region encompasses several immune‐related genes and has been previously implicated in IBD susceptibility (Goyette et al. 2015). The identified variant may influence disease risk through modulation of immune response pathways and inflammatory mediators (Cho and Feldman 2015). In contrast, colocalization analyses for other edible plant preferences did not support shared genetic determinants with either UC or CD risk (Table S9, Figure 5).

## 4 Discussion

### 4.1 Dietary Polyphenols in Plant Foods and IBD Risk

IBD manifests as a sophisticated autoimmune condition characterized by complex pathogenic cascades. While expanding evidence establishes the importance of dietary polyphenols from plant‐based foods in UC and CD pathogenesis, the precise mechanistic relationships and underlying molecular pathways await full characterization. This analytical approach integrated SNPs associated with preferences for polyphenol‐rich edible plants and FinnGen database resources, incorporating detailed locus evaluation, to examine potential causal pathways influencing UC and CD susceptibility through MR methodology. Our multi‐layered analytical approach, encompassing UVMR, MVMR, and colocalization analyses, revealed compelling evidence for causal relationships between specific preferences for polyphenol‐rich edible plants and IBD development.

Our comprehensive MR analyses revealed significant causal relationships between genetically predicted food preferences and IBD risk. For UC, UVMR analysis demonstrated that one standard deviation (SD) increase in aniseed preference was associated with a 283.5% increased risk (subsequently confirmed by MVMR showing a 294.2% increase), while increased capers preference corresponded to a 69.3% reduced risk. For CD susceptibility, UVMR identified several polyphenol‐rich dietary preferences with protective effects: orange juice (67.1% risk reduction), lentils/beans (82.2% reduction), melon (81.9% reduction), and EVOO (74.4% reduction). Among these associations, melon preference maintained significance in MVMR analysis, showing an 83.2% risk reduction per SD increase. Notably, the causal relationship between aniseed preference and UC risk was further supported by colocalization analysis, revealing shared causal variants.

This study is innovative in several key aspects: First, it employs a comprehensive MR approach to investigate the causal link between dietary preferences for polyphenol‐rich plant foods and the risk of IBD, specifically UC and CD. Second, it integrates advanced multi‐variant methodologies, including MVMR and colocalization analyses, to uncover shared genetic determinants of dietary choices and inflammatory disease risk. Third, by identifying specific food preferences that significantly influence IBD susceptibility, this research paves the way for personalized dietary recommendations aimed at disease prevention and management.

These findings illuminate novel pathways through which dietary polyphenols may influence IBD pathogenesis. The identification of these causal relationships not only enhances our understanding of how bioactive polyphenolic compounds contribute to IBD etiology but also suggests potential therapeutic targets for disease management through dietary interventions rich in these compounds. Further investigation of the molecular mechanisms underlying these associations could inform personalized dietary interventions and preventive strategies, potentially reducing future IBD incidence.

### 4.2 The Causal Relationship Between Edible Plant Preferences and UC and CD

In our MR analysis, the positive association between aniseed preference and UC risk reveals a novel perspective in IBD pathogenesis. While aniseed contains various polyphenolic compounds with documented bioactive properties, our findings demonstrated that genetically predicted increased aniseed preference was associated with a 283.5% higher UC risk, with this causal relationship further supported by colocalization analysis (PP.H4 = 99.52%). This observation appears paradoxical to previous findings, where anethole, the primary bioactive compound in aniseed, has demonstrated anti‐inflammatory properties by alleviating oxidative stress and inflammatory responses in acetic acid‐induced colitis in mice (Ghasemi‐Dehnoo et al. 2022). Aniseed contains multiple bioactive compounds, with trans‐anethole (93.9%) being the predominant component in its essential oil, followed by estragole (2.4%). The chemical profile also includes other compounds such as (E)–methyleugenol, α‐cuparene, α‐himachalene, β‐bisabolene, and p‐anisaldehyde (Shojaii and Abdollahi Fard 2012). Despite the documented anti‐inflammatory properties of trans‐anethole, our findings suggest a complex relationship between aniseed preference and UC risk. This apparent paradox might be explained by the presence of other bioactive compounds or their metabolites that could influence intestinal immune responses (Peterson and Artis 2014). The strong genetic correlation identified through colocalization analysis suggests that genetic variants determining aniseed preference might independently affect UC risk through shared immunological pathways, or that dietary patterns associated with aniseed preference might constitute risk factors for UC development. Further mechanistic studies are needed to elucidate whether specific aniseed components directly influence UC pathogenesis or if the observed association is mediated through other biological mechanisms. Notably, this colocalization analysis identified significant signals with the lead SNP rs4947336 showing strong evidence of shared genetic effects between aniseed preference and UC risk (PP.H4 = 99.52%). This SNP is located in the 6p21.33 region, which encompasses the HLA class II region, a genomic area consistently implicated in UC pathogenesis (de Lange et al. 2017). The HLA region harbors genes encoding major histocompatibility complex (MHC) molecules that are crucial for immune response regulation and antigen presentation (Goyette et al. 2015). This shared genetic architecture suggests a mechanism of biological pleiotropy, where rs4947336 might simultaneously influence sensory perception and immune reactivity. It is plausible that variants in this region regulate the expression of receptors involved in both taste signal transduction (influencing food preference) and pathogen recognition (modulating inflammatory responses). Consequently, an individual's genetic predisposition to prefer specific flavors like aniseed could naturally co‐occur with a heightened susceptibility to autoimmune inflammation, driven by the same underlying immunogenetic profile.

This MR analysis revealed a protective association between capers preference and UC risk, with genetically predicted increased capers preference corresponding to a 69.3% reduction in UC risk. These observations parallel established findings regarding the therapeutic properties of *Capparis spinosa* L., characterized by its rich polyphenolic profile including specific flavonoids (quercetin and rutin) and glucosinolate compounds (Sun et al. 2023). These polyphenolic compounds have demonstrated significant anti‐inflammatory and antioxidant properties in experimental studies. Emerging evidence suggests that the protective effect of capers might be partially mediated through modulation of the gut microbiota. The polyphenolic compounds in capers, particularly quercetin and rutin, have been shown to act as prebiotics, promoting the growth of beneficial bacteria such as *Akkermansia muciniphila* and *Faecalibacterium prausnitzii* , which are typically depleted in UC patients (Parada Venegas et al. 2019). These bacteria are crucial producers of short‐chain fatty acids (SCFAs), particularly butyrate, which serves as an energy source for colonocytes and exhibits anti‐inflammatory properties (Lavelle and Sokol 2020). Furthermore, the glucosinolates can be metabolized by gut microbiota to form bioactive isothiocyanates, which have been demonstrated to enhance intestinal barrier function and reduce inflammatory responses (Lloyd‐Price et al. 2019). Recent metabolomic studies have shown that polyphenol consumption leads to increased levels of beneficial metabolites, including SCFAs and tryptophan metabolites, which are known to promote intestinal homeostasis and regulate immune responses (Franzosa et al. 2019). These biological mechanisms could potentially explain the protective effect observed in our genetic analysis. However, further investigation is needed to determine whether the genetic variants influencing capers preference directly affect UC risk through these pathways or through other biological mechanisms.

The relationship between dietary preferences and CD risk has long been a subject of interest in IBD research. Our MR analysis provides novel evidence for a protective role of orange juice preference against CD development, where genetically predicted increased orange juice preference was associated with a significant reduction in CD risk. Orange juice is a particularly rich source of citrus flavonoids, including hesperidin, naringin, and other polyphenolic compounds, along with vitamin C, carotenoids, and pectin (Lv et al. 2015). These polyphenolic compounds exhibit potent antioxidant and anti‐inflammatory properties that may contribute to intestinal health. Notably, citrus flavonoids have been demonstrated to modulate inflammatory responses through multiple pathways, including the suppression of pro‐inflammatory cytokine production and regulation of immune cell function (Testai and Calderone 2017). The protective association observed in our study might be explained through several biological mechanisms. Orange juice consumption has been associated with beneficial modulation of the gut microbiota composition and function. The pectin and flavonoids in orange juice can act as prebiotics, promoting the growth of beneficial bacteria and enhancing the production of short‐chain fatty acids (SCFAs) (Parada Venegas et al. 2019). These SCFAs, particularly butyrate, play crucial roles in maintaining intestinal barrier integrity and regulating immune responses, both of which are compromised in CD (Lavelle and Sokol 2020). Moreover, the antioxidant compounds in orange juice have been shown to reduce oxidative stress and inflammation, key factors in CD pathogenesis. Recent metabolomic studies have demonstrated that regular consumption of citrus fruits can modulate tryptophan metabolism and aryl hydrocarbon receptor signaling, pathways that are crucial for intestinal immune homeostasis (Franzosa et al. 2019). While these mechanistic insights provide biological plausibility for our findings, it is important to note that the relationship between orange juice preference and CD risk may be more complex than direct causation. The genetic variants influencing orange juice preference might affect CD risk through multiple pathways, including taste receptor‐mediated immune responses or broader dietary patterns associated with citrus preferences. Further research is warranted to fully elucidate the molecular mechanisms underlying this protective association and to explore potential therapeutic implications for CD prevention or management.

Legumes are rich sources of dietary polyphenols and other bioactive compounds that exhibit potent anti‐inflammatory properties (Singh et al. 2017). The polyphenols and flavonoids found in lentils and beans have been shown to suppress pro‐inflammatory cytokines (including TNF‐α, IL‐1β, and IL‐6) and inhibit the NF‐κB signaling pathway, which is hyperactivated in CD (Parada Venegas et al. 2019). Additionally, legume‐derived bioactive peptides can activate the AMPK pathway and enhance the expression of antioxidant enzymes, thereby reducing oxidative stress and inflammation in intestinal tissues (Lavelle and Sokol 2020). Lentils and beans are also rich in dietary fiber, resistant starch, and oligosaccharides (So et al. 2018). These components contribute to intestinal health through multiple pathways: the soluble fiber components promote the production of short‐chain fatty acids (SCFAs), particularly butyrate, which has been shown to strengthen intestinal barrier function by upregulating tight junction proteins and reducing intestinal permeability (Rooks and Garrett 2016). Furthermore, butyrate exhibits direct anti‐inflammatory effects by inhibiting histone deacetylases (HDACs) in immune cells, leading to the suppression of pro‐inflammatory mediators and the promotion of regulatory T cell development (Campbell et al. 2020). The genetic variants influencing legume preference might affect CD risk through these anti‐inflammatory and barrier‐protective pathways, potentially mediated by taste perception‐influenced dietary choices. Further research is needed to elucidate the specific molecular mechanisms underlying this protective association.

Melons contain unique bioactive compounds including cucurbitacins and various polyphenolic antioxidants (Alghasham 2013). Specifically, cucurbitacin B has been shown to inhibit the JAK/STAT3 signaling pathway and suppress the production of pro‐inflammatory cytokines including IL‐6 and TNF‐α, which are key mediators in CD pathogenesis (Zhang et al. 2011). Melons also contain substantial amounts of polyphenolic antioxidants and superoxide dismutase (SOD), particularly in muskmelon varieties, which play a crucial role in cellular antioxidant defense (Zhang et al. 2018). The SOD activity, combined with high levels of vitamin C and β‐carotene, provides significant protection against oxidative stress, a key factor in intestinal inflammation. Recent studies have demonstrated that these antioxidant compounds can activate the Nrf2/ARE pathway, enhancing the expression of antioxidant enzymes and protecting intestinal epithelial cells from inflammatory damage (Thiruvengadam et al. 2021). Furthermore, melons contain unique proteolytic enzymes, such as cucumisin, that may aid in protein digestion and reduce antigenic protein load (Rao et al. 1998). The high water content and soluble fiber in melons also contribute to proper hydration and intestinal barrier function, as demonstrated by recent metabolomic studies (Schirmer et al. 2016). The genetic variants influencing melon preference might modulate CD risk through these anti‐inflammatory and antioxidant pathways.

This MR analysis revealed a significant inverse association between genetic predisposition to EVOO and CD risk, where increased genetic preference for EVOO was associated with reduced CD susceptibility. This finding provides genetic evidence supporting the potential protective effects of EVOO in CD pathogenesis. The protective association might be explained by EVOO's unique polyphenolic compounds and their molecular mechanisms. EVOO is particularly rich in phenolic compounds, especially hydroxytyrosol and oleuropein, which demonstrate potent anti‐inflammatory properties (Aparicio‐Soto et al. 2016). These polyphenolic compounds have been shown to directly inhibit the NLRP3 inflammasome, a key mediator of intestinal inflammation in CD, thereby reducing the production of pro‐inflammatory cytokines IL‐1β and IL‐18 (Santangelo et al. 2018). EVOO's oleocanthal, another phenolic compound, exhibits mechanisms similar to ibuprofen by inhibiting both COX‐1 and COX‐2 enzymes, thus reducing prostaglandin synthesis and inflammatory responses (Peyrol et al. 2017). Recent studies have demonstrated that oleocanthal can specifically target the mTOR/NF‐κB signaling pathway, which is frequently dysregulated in CD patients (Neurath 2019). Furthermore, EVOO is characterized by a high proportion of monounsaturated fatty acids, particularly oleic acid (C18:1n‐9), which has been shown to modulate intestinal barrier function. Oleic acid activates the PPAR‐γ pathway, enhancing tight junction protein expression and reducing intestinal permeability (Meital et al. 2020). Additionally, EVOO's squalene and tocopherols exhibit antioxidant properties, protecting intestinal epithelial cells from oxidative stress‐induced damage through activation of the Nrf2/HO‐1 pathway (Aparicio‐Soto et al. 2016). The genetic variants influencing EVOO preference might affect CD risk through these anti‐inflammatory and barrier‐protective pathways. Further investigation is needed to fully elucidate the molecular mechanisms underlying this protective association.

### 4.3 Strengths, Limitations, and Future Directions

The methodological framework of this MR investigation provides significant analytical advantages. It represents the most comprehensive assessment to date examining causality between preferences for polyphenol‐rich edible plants and IBD manifestations (UC and CD), contributing to our understanding of how dietary polyphenols may play a role in non‐communicable chronic disease prevention. Moreover, our sophisticated MR implementation effectively addresses key methodological considerations, including reverse causation and potential confounding factors. Multiple analytical approaches were implemented to validate MR assumptions and minimize potential biases. The consistency in directional effects across diverse MR estimators, supported by extensive sensitivity testing, demonstrates result stability. The integration of MVMR and colocalization analyses further strengthens our conclusions by addressing potential pleiotropic effects among plant preference traits.

Several methodological considerations warrant discussion in interpreting these findings. The demographic scope, limited to European‐ancestry populations, potentially constrains result generalizability across diverse ethnic backgrounds. While unmeasured pleiotropic effects in MR analyses represent a potential source of bias, the validity of our instrumental variables is supported by robust *F*‐statistic values (> 10) across all selected SNPs. Third, it is important to note that genetic predisposition to food preference does not strictly equate to actual polyphenol intake. Since individual consumption levels were not quantified, we cannot determine the precise dose–response relationship, although preference is generally considered a strong predictor of dietary habits. Additionally, the low number of cases, with only 261 UC and 1870 CD patients among significantly larger exposure sample sizes, may substantially compromise the statistical power of our findings, affecting the robustness of associations drawn in this investigation. Finally, while our study focused on preferences for polyphenol‐rich edible plants, the complexity of dietary patterns, polyphenol bioavailability, food preparation methods that may affect polyphenol content, and interactions between different plant‐based bioactive compounds could not be fully captured in this analysis. The empirical foundation supporting relationships between preferences for polyphenol‐rich plants and IBD requires further strengthening through mechanistic investigations and robust clinical validation via adequately powered randomized controlled trials examining UC and CD outcomes.

Looking ahead, future research needs to prioritize interventional clinical trials to validate the causal relationships identified in our genetic analysis. Simultaneously, basic experiments can be conducted to elucidate the mechanisms by which dietary polyphenols from these plant foods exert their effects, designing research plans from multiple perspectives and levels. This allows for the mechanistic study of polyphenol‐mediated multi‐signals, multi‐pathways, and multi‐targets, particularly focusing on inflammatory pathways and oxidative stress mechanisms implicated in both UC and CD pathogenesis. The molecular mechanisms comprise several interconnected signaling cascades, including NF‐κB‐mediated regulation, JAK/STAT signal transduction, NLRP3 inflammasome activation, TNF‐dependent pathways, MAPK‐regulated processes, and mTOR signaling networks. Additionally, investigation of pathways related to polyphenol metabolism, intestinal barrier function, mucosal immunity, and gut microbiota‐polyphenol interactions would provide valuable insights into how polyphenol‐rich plant‐based foods differentially affect UC and CD. Such mechanistic studies focusing on the bioactive properties of dietary polyphenols would provide scientific and effective data to support clinical research and potentially inform dietary recommendations for IBD prevention and management, aligning with the broader goal of utilizing dietary polyphenols in the prevention of non‐communicable chronic diseases, with specific considerations for the distinct pathophysiology of UC and CD.

## 5 Conclusion

This systematic MR investigation establishes causal relationships between genetic determinants of preferences for polyphenol‐rich plant foods and IBD susceptibility patterns in both UC and CD. The identified associations highlight the potential role of dietary polyphenols in preventing non‐communicable chronic inflammatory diseases. The preference‐associated factors present significant implications for IBD risk assessment, preventive interventions, and therapeutic strategies, while informing the design of subsequent clinical investigations.

Overall, the innovative approach taken in this study not only bridges the gap between diet and genetics but also lays a foundation for future clinical trials exploring dietary interventions in IBD management. The identification of specific food preferences linked to IBD risk warrants further research into personalized nutrition strategies that could mitigate disease incidence and improve patient outcomes.

These findings additionally provide a methodological framework for examining IBD pathogenic mechanisms and etiological factors. These findings not only advance our understanding of the genetic architecture underlying dietary preferences for polyphenol‐rich foods in IBD but also highlight promising avenues for therapeutic intervention through dietary modification rich in bioactive polyphenolic compounds.

## Author Contributions

**Tinghui Yue:** conceptualization, formal analysis, and writing – original draft preparation. **Qiang Su:** conceptualization, formal analysis, and writing – original draft preparation. **Song He:** data curation. **Yuhua Liu:**visualization. **Dan Huang:** methodology. **Zhenxiang An:** supervision and writing – review and editing. **Yuanli He:** supervision and writing – review and editing. All authors have read and agreed to the published version of the manuscript.

## Funding

This study was supported by the Fifth Batch of National Clinical Excellence Training Program for Traditional Chinese Medicine Practitioners, National Administration of Traditional Chinese Medicine of China (Grant No. State TCM Education Letter [2022] No. 1); the Traditional Chinese Medicine Spleen and Stomach Disease Scientific and Technological Innovation Talent Team Construction Project, Guizhou University of Traditional Chinese Medicine (Grant No. Gui TCM TD [2022] No. 005); the Traditional Chinese Medicine and Ethnic Medicine Scientific and Technological Research Project, Guizhou Provincial Administration of Traditional Chinese Medicine (Grant No. QZYY‐2025‐026); the Postgraduate Education Innovation Program, Guizhou University of Traditional Chinese Medicine (Grant Nos. YCXKYB2024004 and YCXKYB2025015); and the Guizhou Clinical Research Center for Digestive Diseases (Grant No. Qian Ke He Platform‐LCZX [2025] 001).

## Ethics Statement

The authors have nothing to report.

## Conflicts of Interest

The authors declare no conflicts of interest.

## Supporting information


**Figures S1–S4:** fsn371453‐sup‐0001‐FiguresS1‐S4.pdf.

**Tables S1–S9:** fsn371453‐sup‐0002‐TablesS1‐S9.xlsx.

## Data Availability

All data generated or analyzed during this study are included in this published article [and its Supporting Information files].
