# Supplementary material for: Plant‐Based Food Preferences Rich in Polyphenols and Their Causal Effects on Inflammatory Bowel Disease
Source: Food Sci Nutr. 2026 Jan 14;14(1):e71453. doi: 10.1002/fsn3.71453 (PMC12800916; doi:10.1002/fsn3.71453)
Supplement: Supplementary file 1 — Figures S1–S4: fsn371453‐sup‐0001‐FiguresS1‐S4.pdf. [file FSN3-14-e71453-s002.pdf]

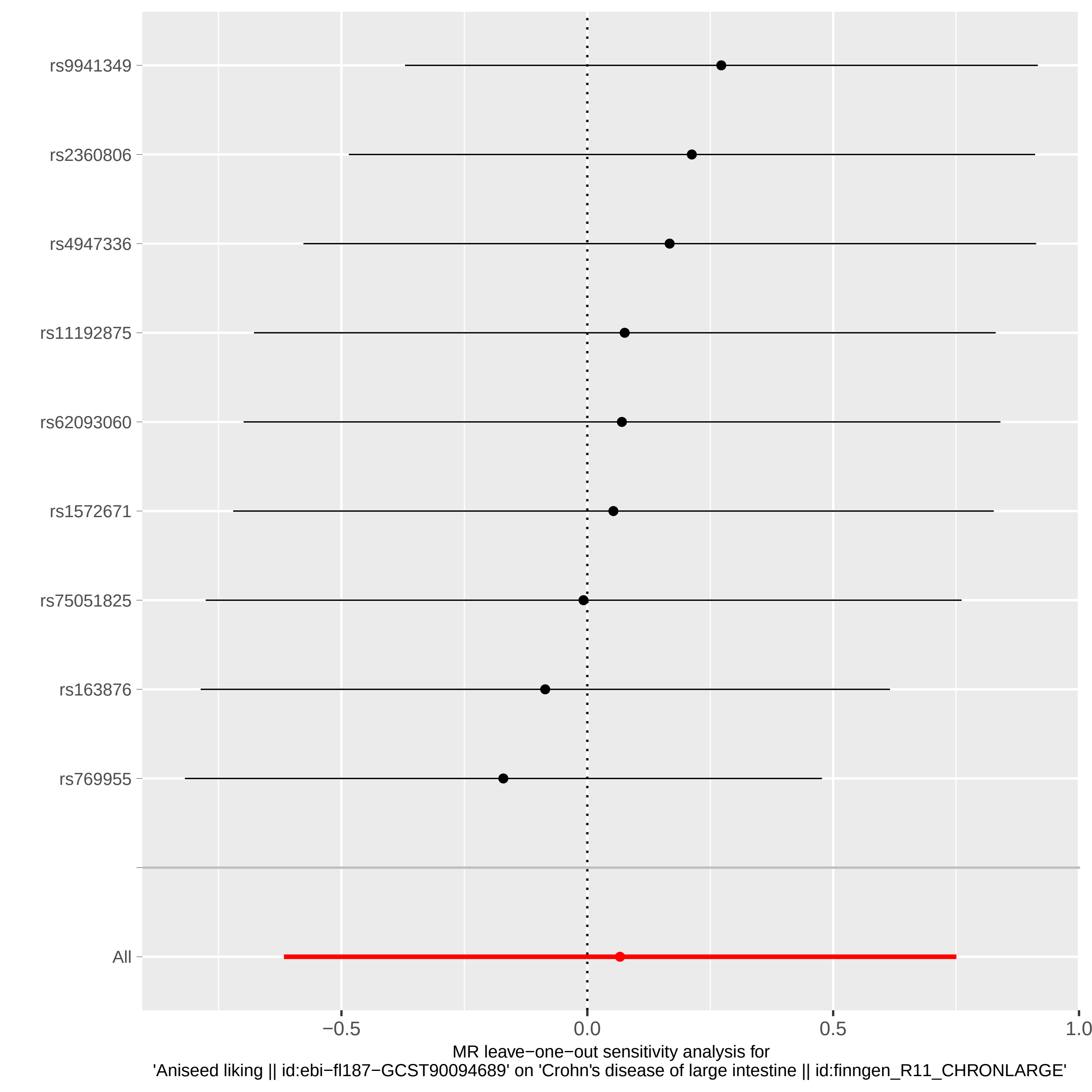
rs9941349
rs2360806
rs4947336
rs11192875
rs62093060
rs1572671
rs75051825
rs163876
rs769955
All
−0.5
0.0
0.5
1.0
MR leave−one−out sensitivity analysis for
'Aniseed liking || id:ebi−fl187−GCST90094689' on 'Crohn's disease of large intestine || id:finngen_R11_CHRONLARGE'

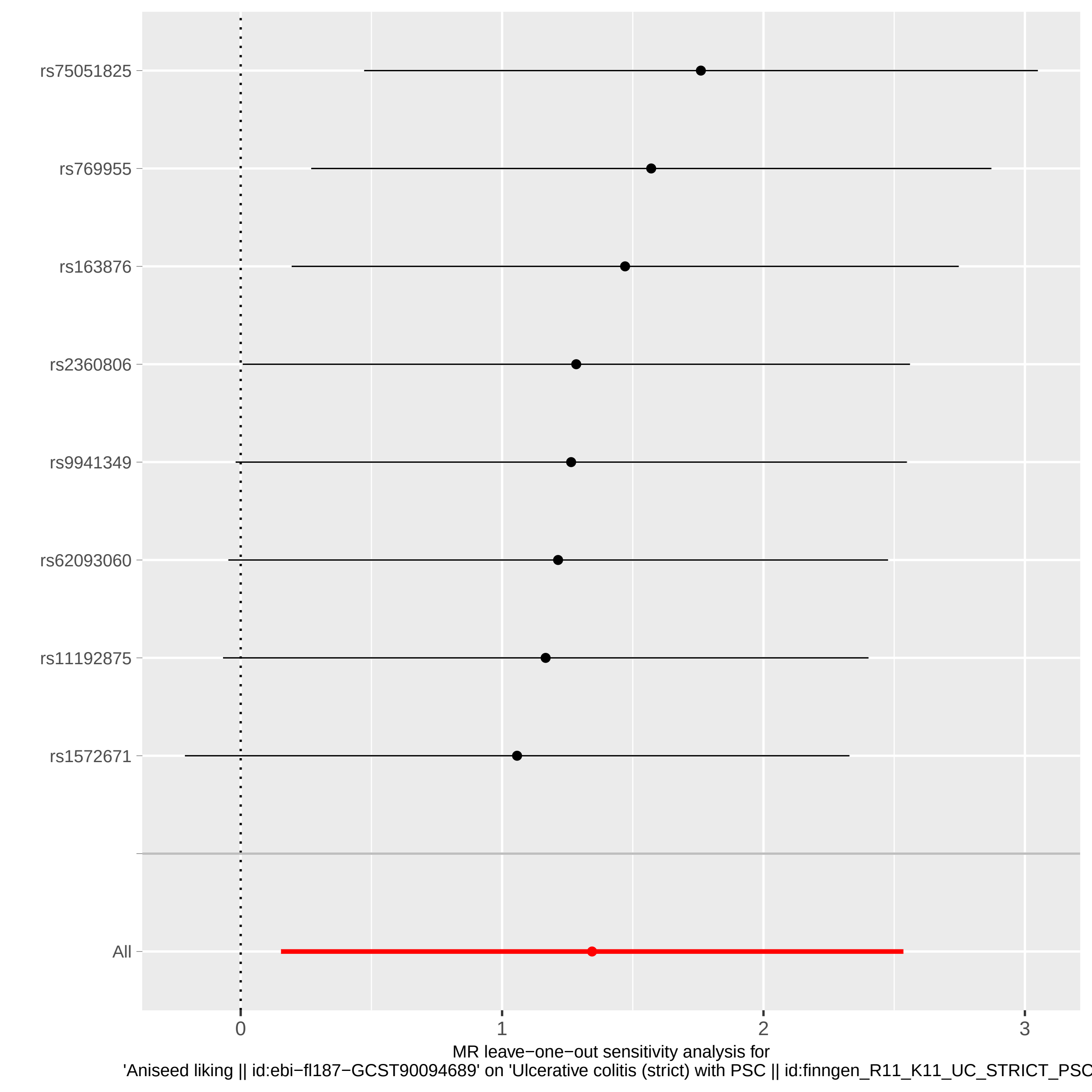

rs75051825
rs769955
rs163876
rs2360806
rs9941349
rs62093060
rs11192875
rs1572671
All
0
1
2
3
MR leave−one−out sensitivity analysis for
'Aniseed liking || id:ebi−fl187−GCST90094689' on 'Ulcerative colitis (strict) with PSC || id:finngen_R11_K11_UC_STRICT_PSC

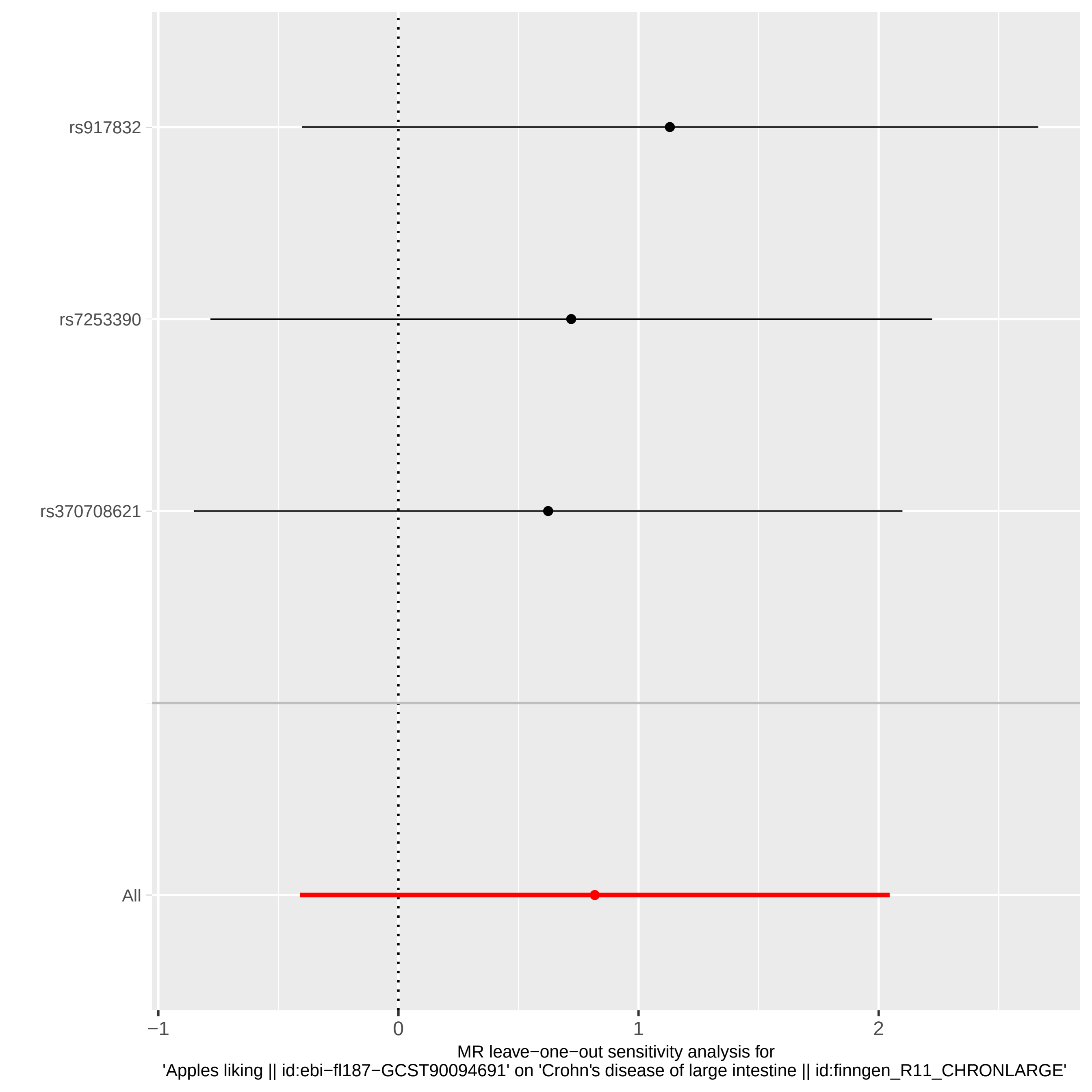
rs917832
rs7253390
rs370708621
All
−1
0
1
2
MR leave−one−out sensitivity analysis for
'Apples liking || id:ebi−fl187−GCST90094691' on 'Crohn's disease of large intestine || id:finngen_R11_CHRONLARGE'

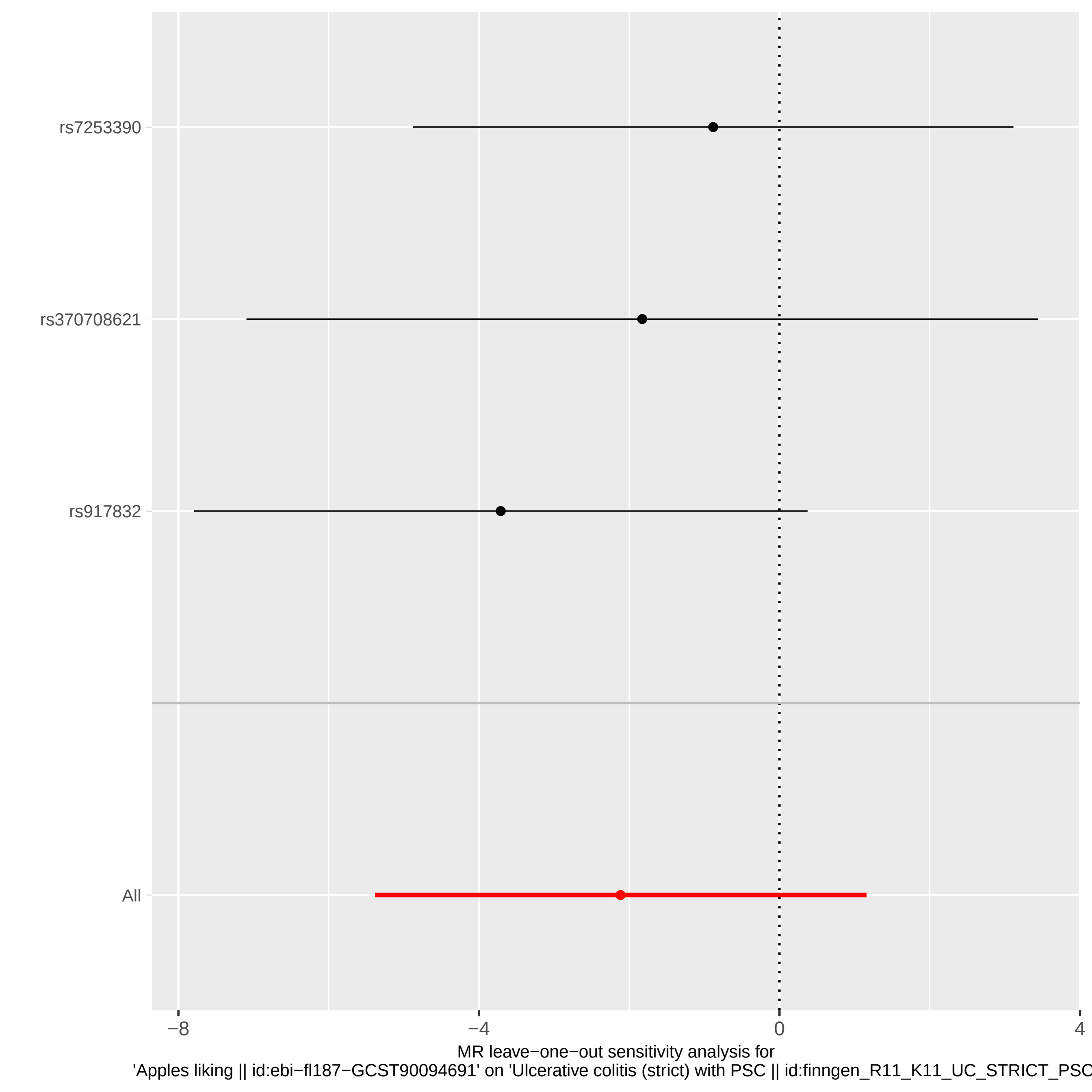
rs7253390
rs370708621
rs917832
All
−8
−4
0
4
MR leave−one−out sensitivity analysis for
'Apples liking || id:ebi−fl187−GCST90094691' on 'Ulcerative colitis (strict) with PSC || id:finngen_R11_K11_UC_STRICT_PSC

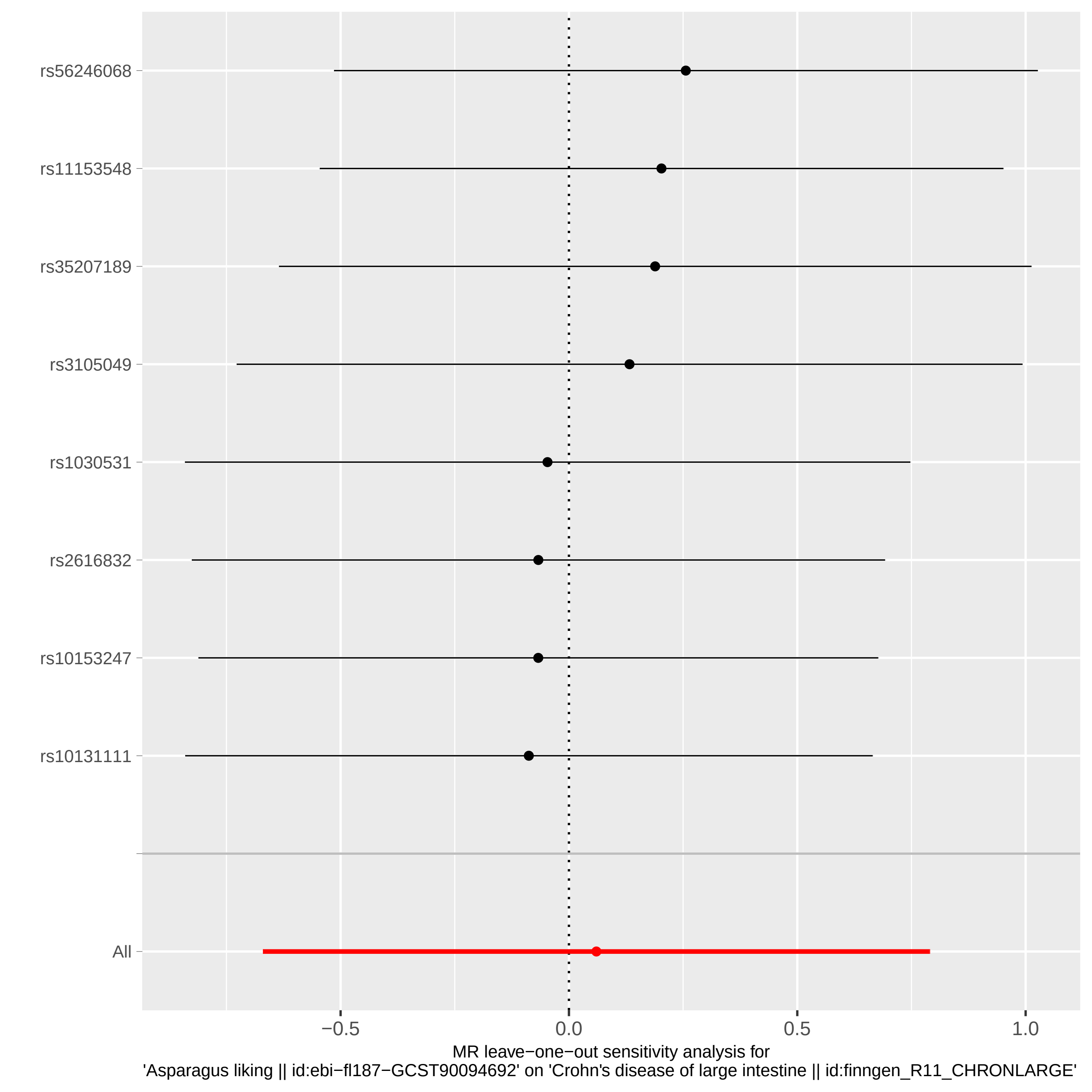
rs56246068
rs11153548
rs35207189
rs3105049
rs1030531
rs2616832
rs10153247
rs10131111
All
−0.5
0.0
0.5
1.0
MR leave−one−out sensitivity analysis for
'Asparagus liking || id:ebi−fl187−GCST90094692' on 'Crohn's disease of large intestine || id:finngen_R11_CHRONLARGE'

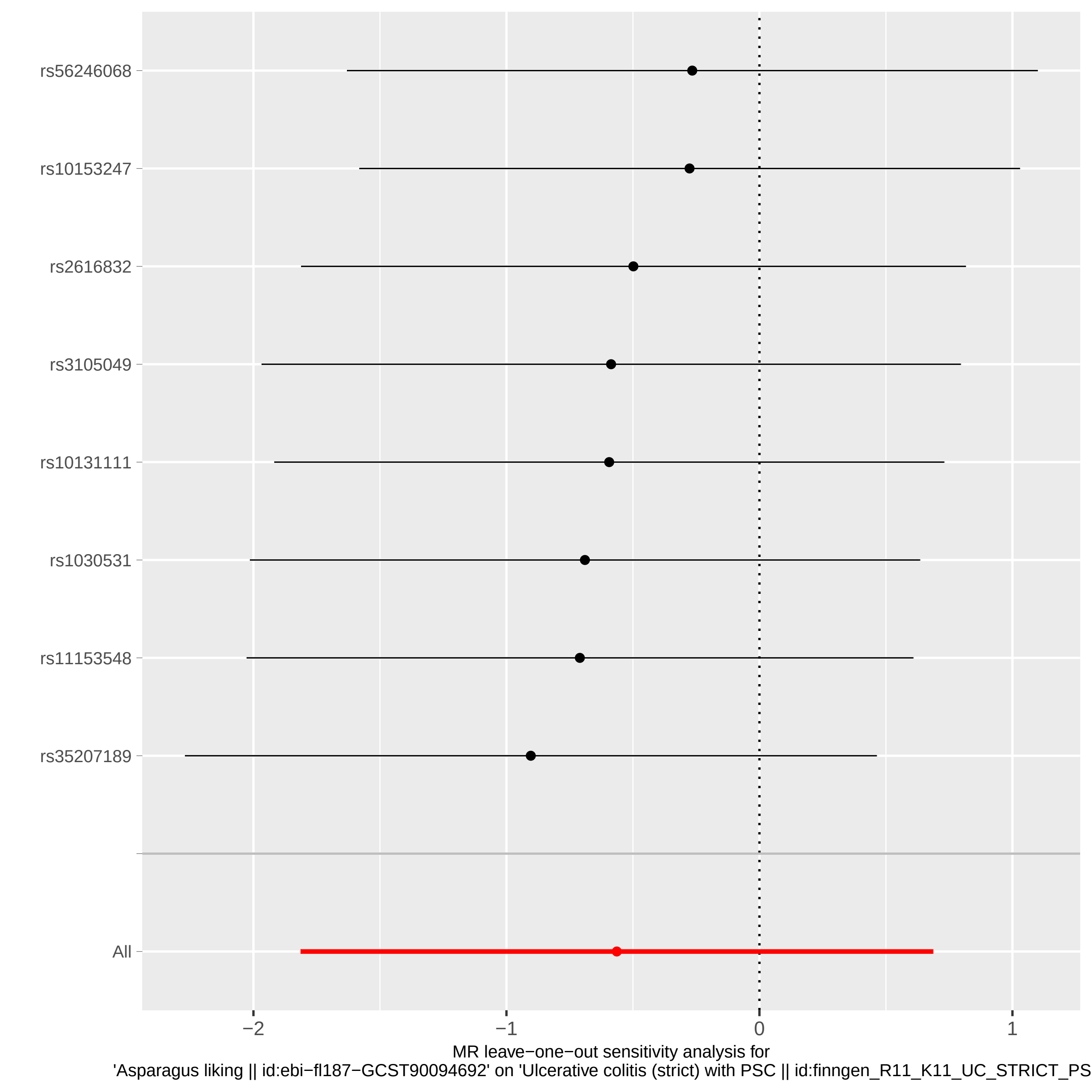
rs56246068
rs10153247
rs2616832
rs3105049
rs10131111
rs1030531
rs11153548
rs35207189
All
−2
−1
0
1
MR leave−one−out sensitivity analysis for
'Asparagus liking || id:ebi−fl187−GCST90094692' on 'Ulcerative colitis (strict) with PSC || id:finngen_R11_K11_UC_STRICT_PS

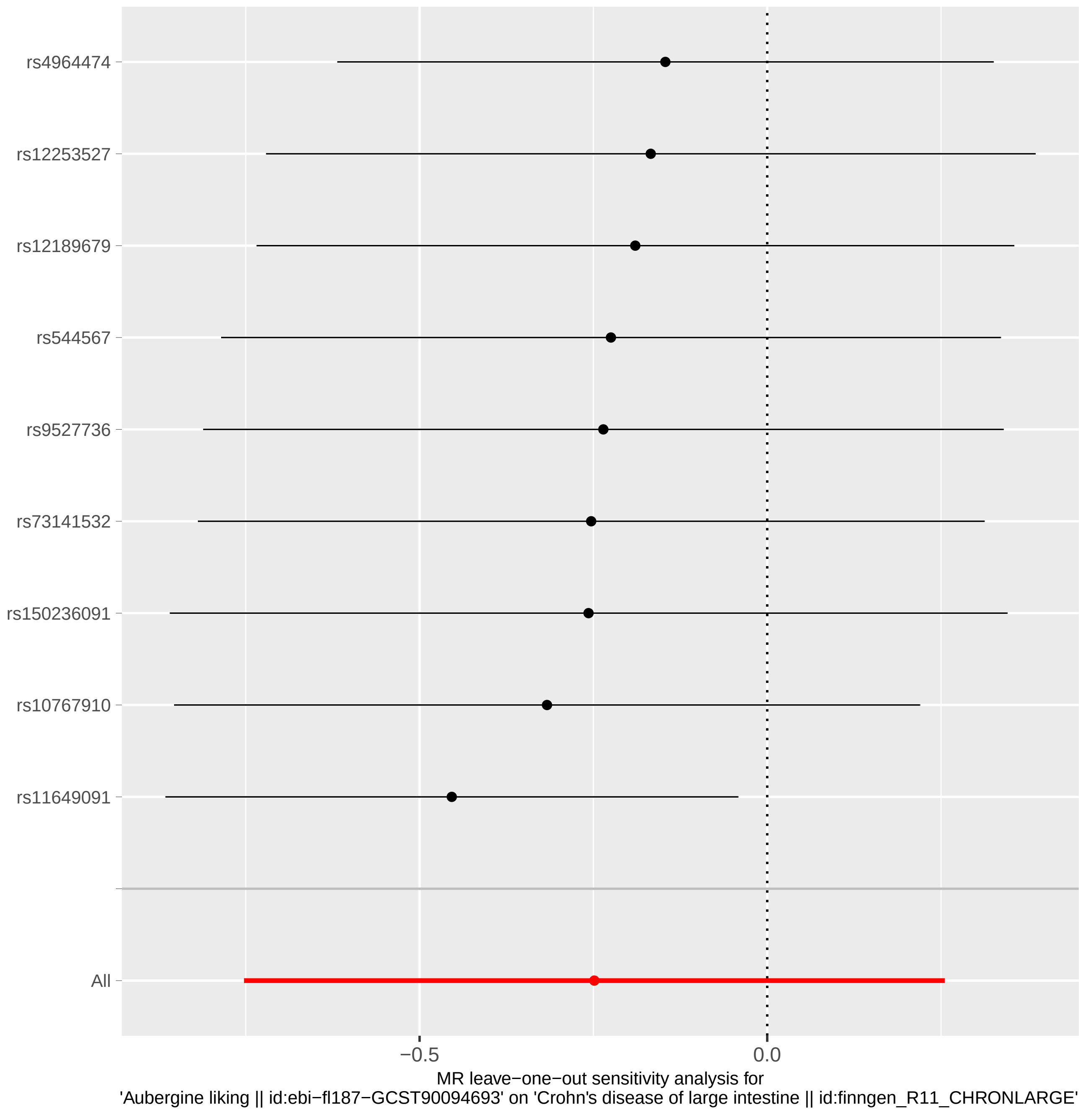
rs4964474
rs12253527
rs12189679
rs544567
rs9527736
rs73141532
rs150236091
rs10767910
rs11649091
All
−0.5
0.0
MR leave−one−out sensitivity analysis for
'Aubergine liking || id:ebi−fl187−GCST90094693' on 'Crohn's disease of large intestine || id:finngen_R11_CHRONLARGE'

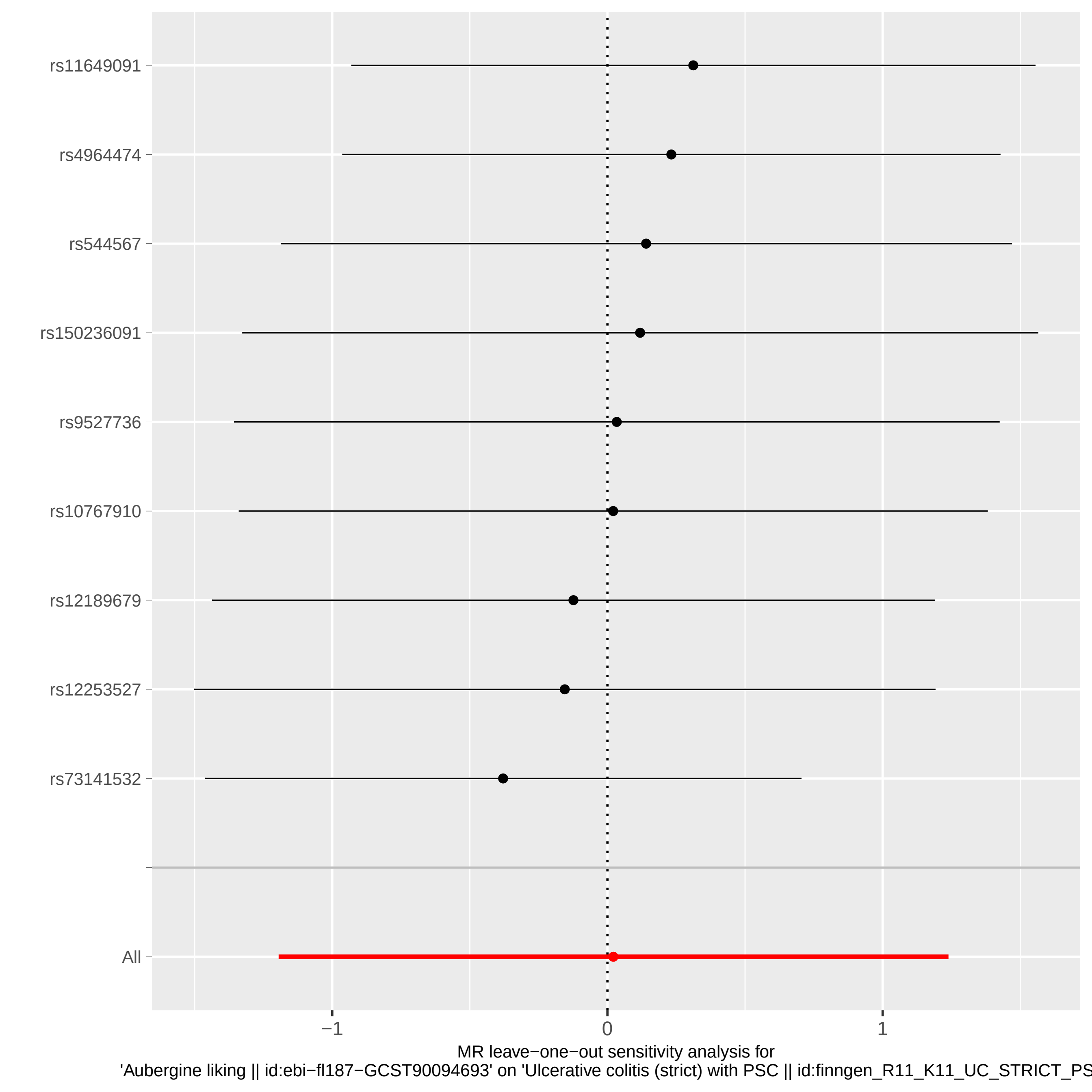
rs11649091
rs4964474
rs544567
rs150236091
rs9527736
rs10767910
rs12189679
rs12253527
rs73141532
All
−1
0
1
MR leave−one−out sensitivity analysis for
'Aubergine liking || id:ebi−fl187−GCST90094693' on 'Ulcerative colitis (strict) with PSC || id:finngen_R11_K11_UC_STRICT_PS

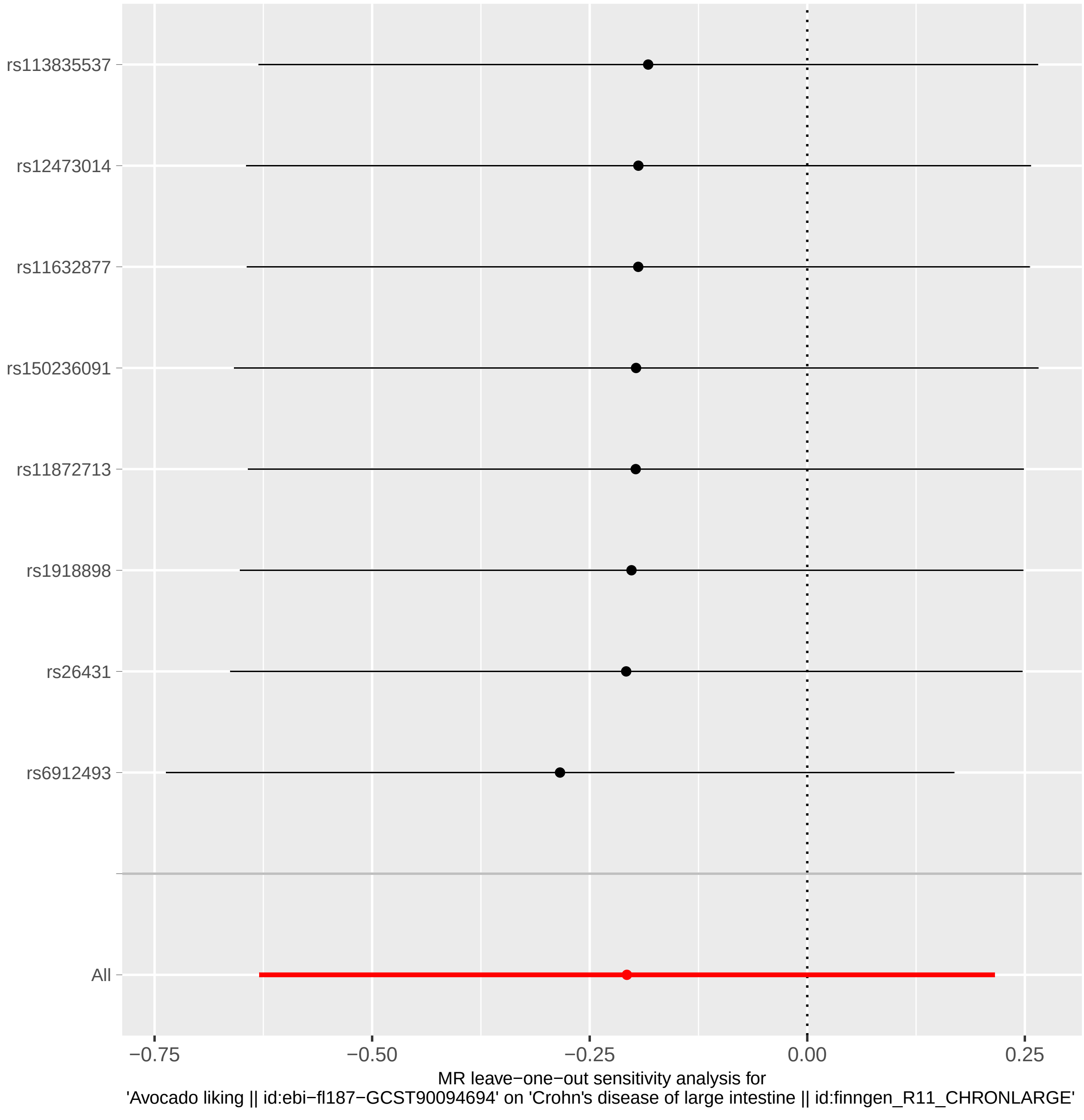
rs113835537
rs12473014
rs11632877
rs150236091
rs11872713
rs1918898
rs26431
rs6912493
All
−0.75
−0.50
−0.25
0.00
0.25
MR leave−one−out sensitivity analysis for
'Avocado liking || id:ebi−fl187−GCST90094694' on 'Crohn's disease of large intestine || id:finngen_R11_CHRONLARGE'

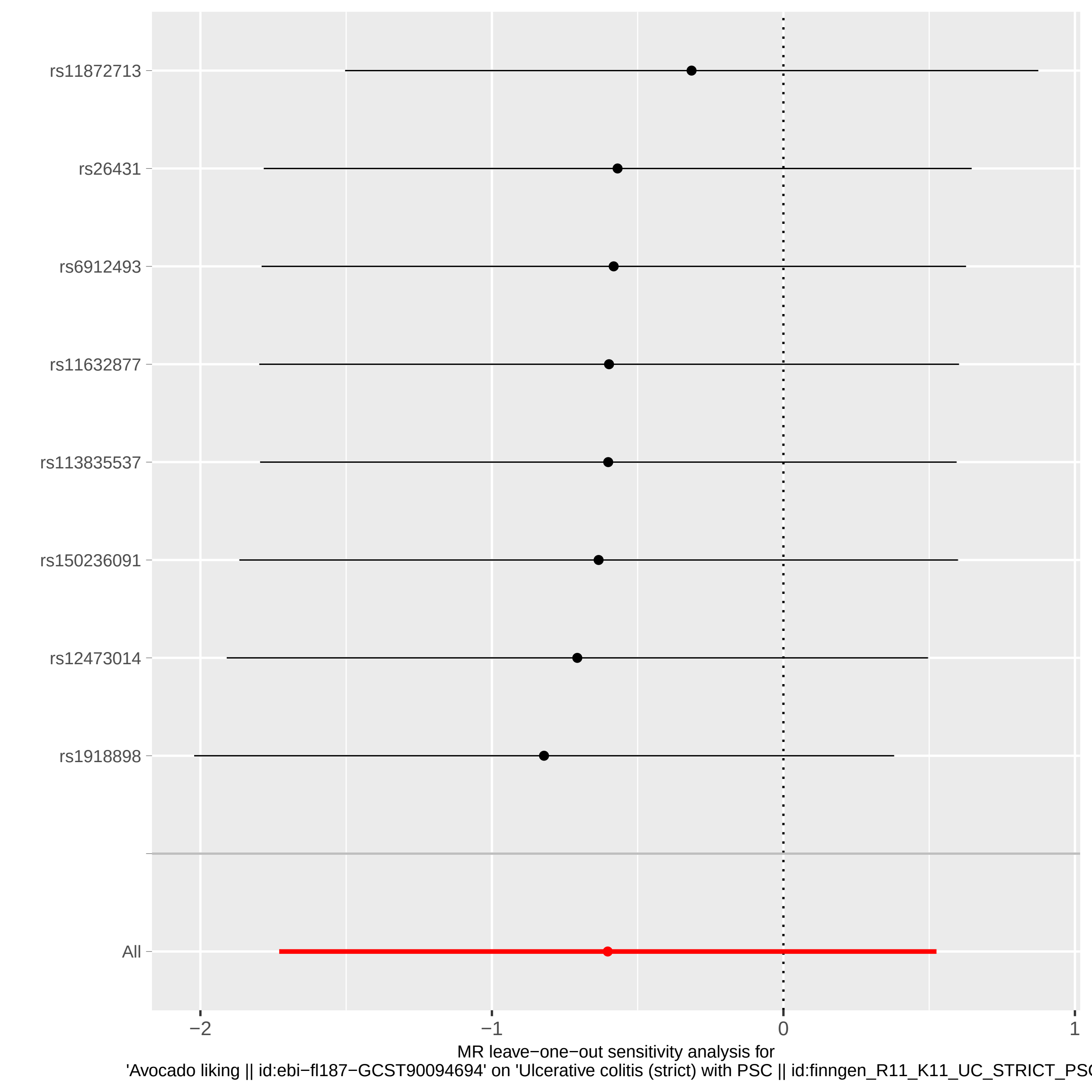

rs11872713
rs26431
rs6912493
rs11632877
rs113835537
rs150236091
rs12473014
rs1918898
All
−2
−1
0
1
MR leave−one−out sensitivity analysis for
'Avocado liking || id:ebi−fl187−GCST90094694' on 'Ulcerative colitis (strict) with PSC || id:finngen_R11_K11_UC_STRICT_PS

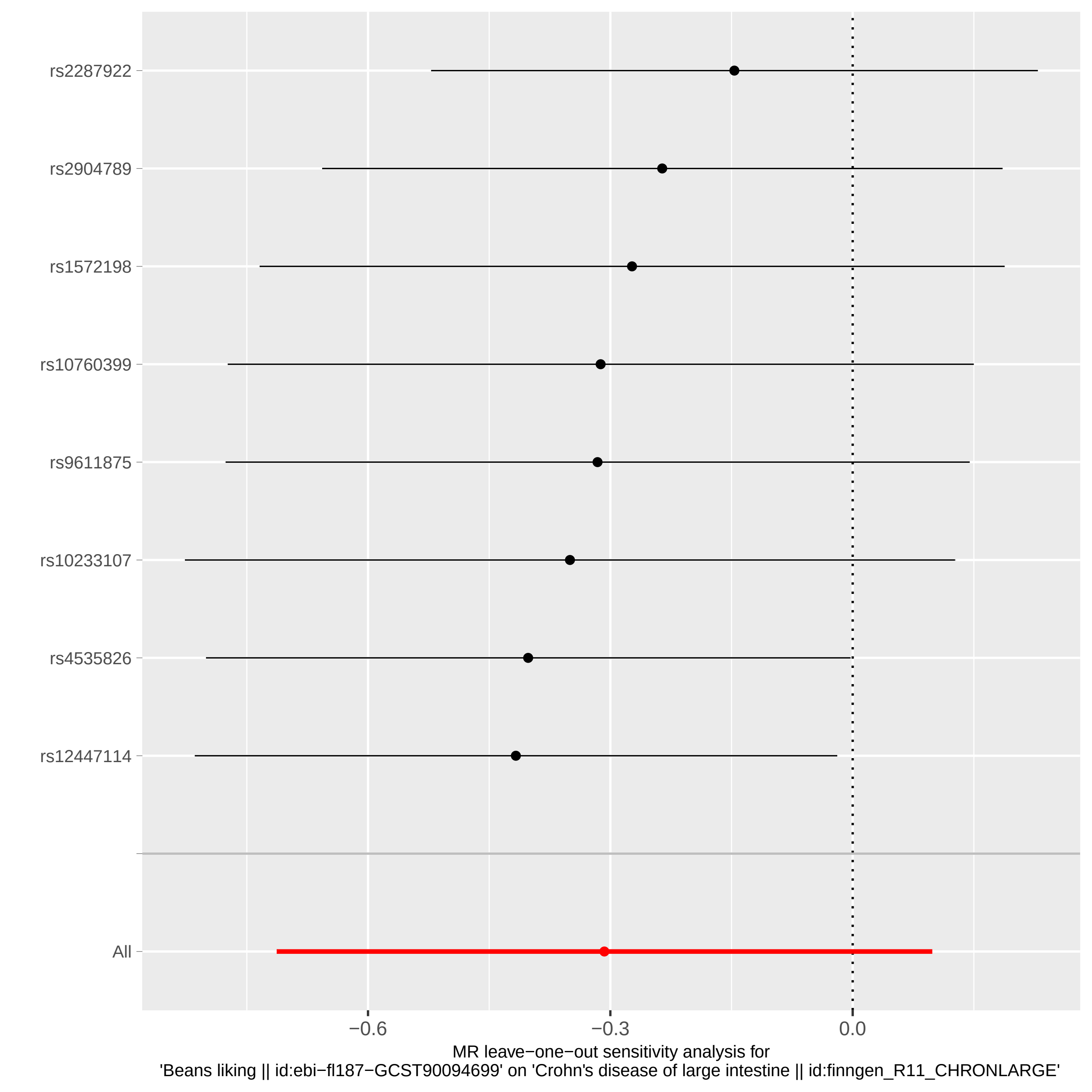
rs2287922
rs2904789
rs1572198
rs10760399
rs9611875
rs10233107
rs4535826
rs12447114
All
−0.6
−0.3
0.0
MR leave−one−out sensitivity analysis for
'Beans liking || id:ebi−fl187−GCST90094699' on 'Crohn's disease of large intestine || id:finngen_R11_CHRONLARGE'

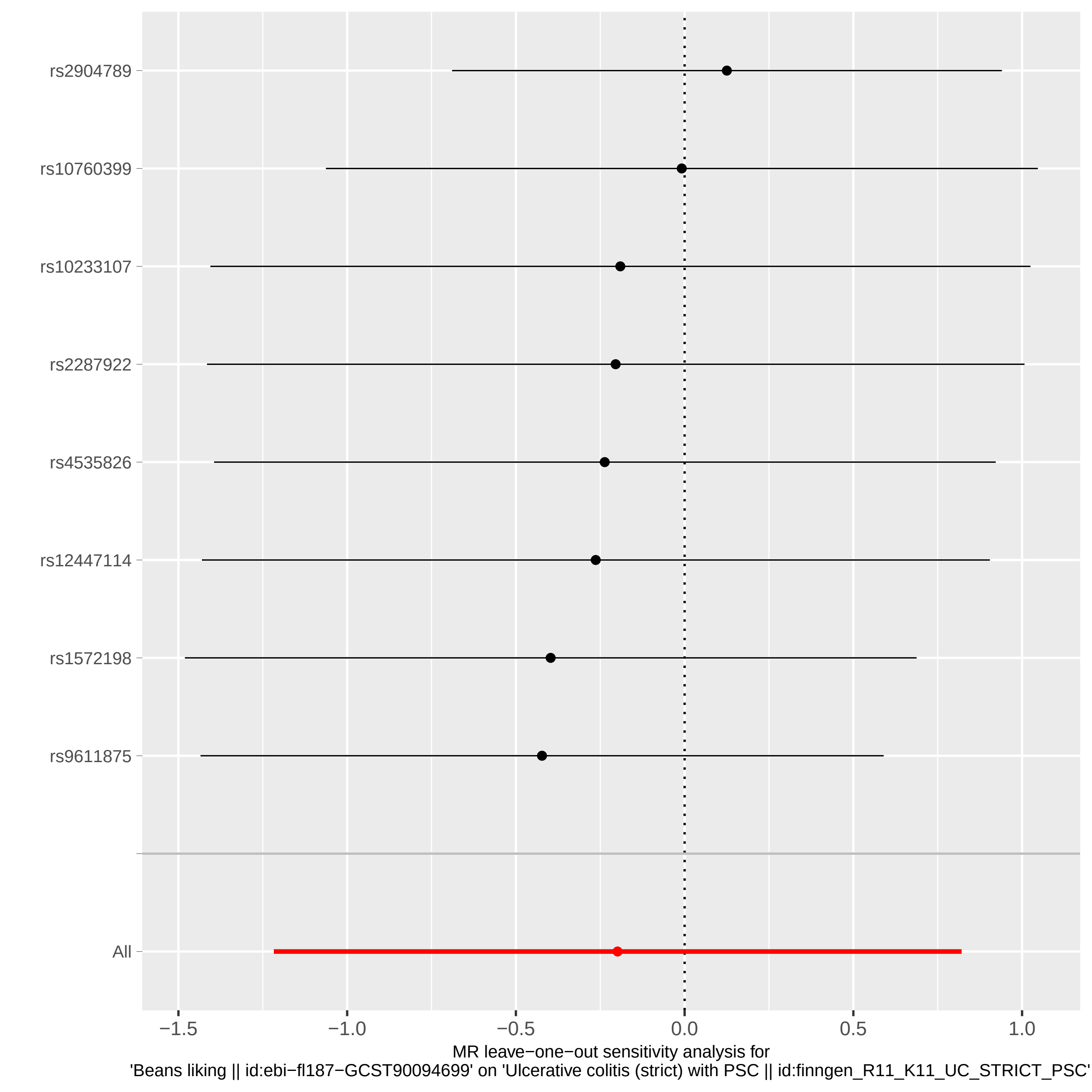
rs2904789
rs10760399
rs10233107
rs2287922
rs4535826
rs12447114
rs1572198
rs9611875
All
−1.5
−1.0
−0.5
0.0
0.5
1.0
MR leave−one−out sensitivity analysis for
'Beans liking || id:ebi−fl187−GCST90094699' on 'Ulcerative colitis (strict) with PSC || id:finngen_R11_K11_UC_STRICT_PSC'

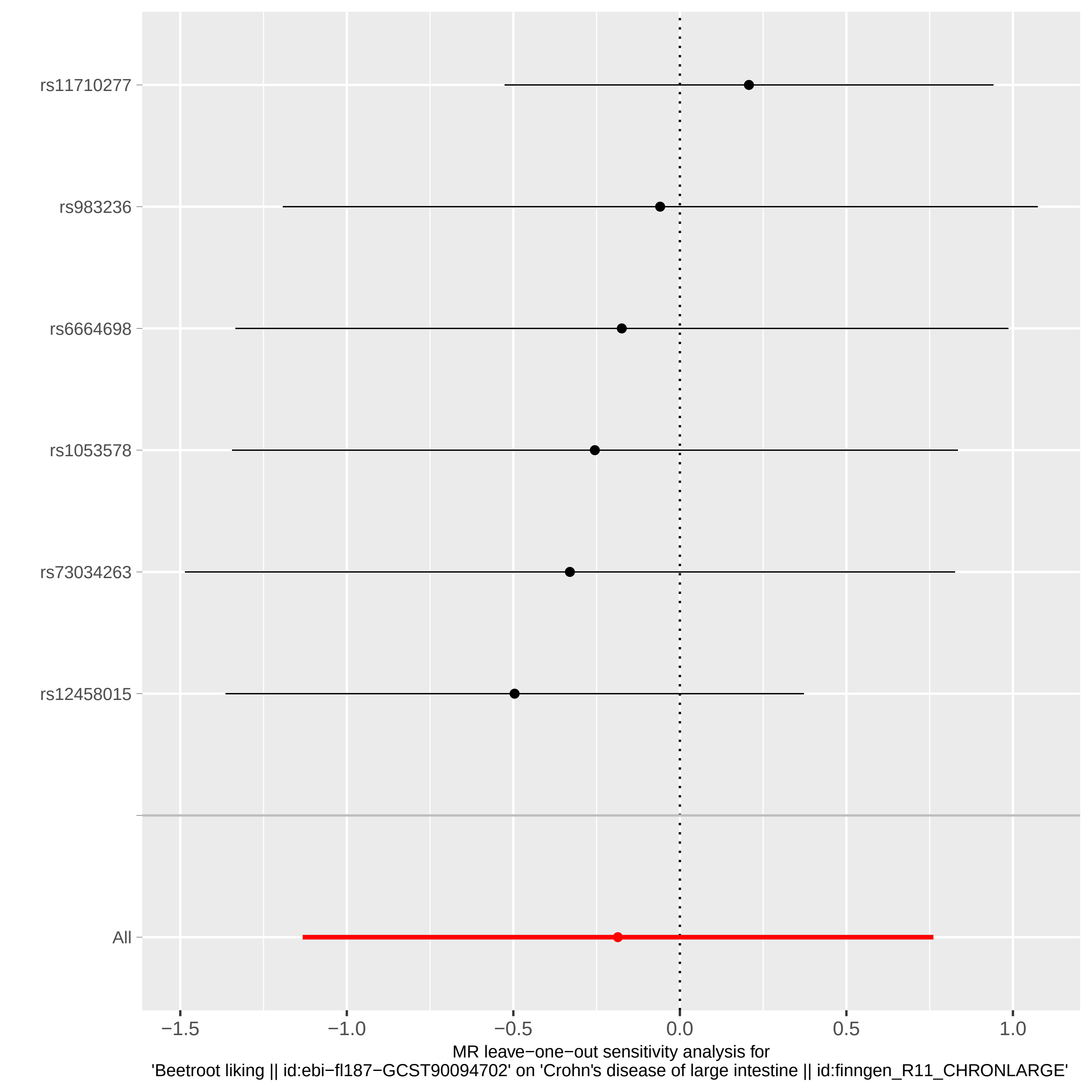
rs11710277
rs983236
rs6664698
rs1053578
rs73034263
rs12458015
All
−1.5
−1.0
−0.5
0.0
0.5
1.0
MR leave−one−out sensitivity analysis for
'Beetroot liking || id:ebi−fl187−GCST90094702' on 'Crohn's disease of large intestine || id:finngen_R11_CHRONLARGE'

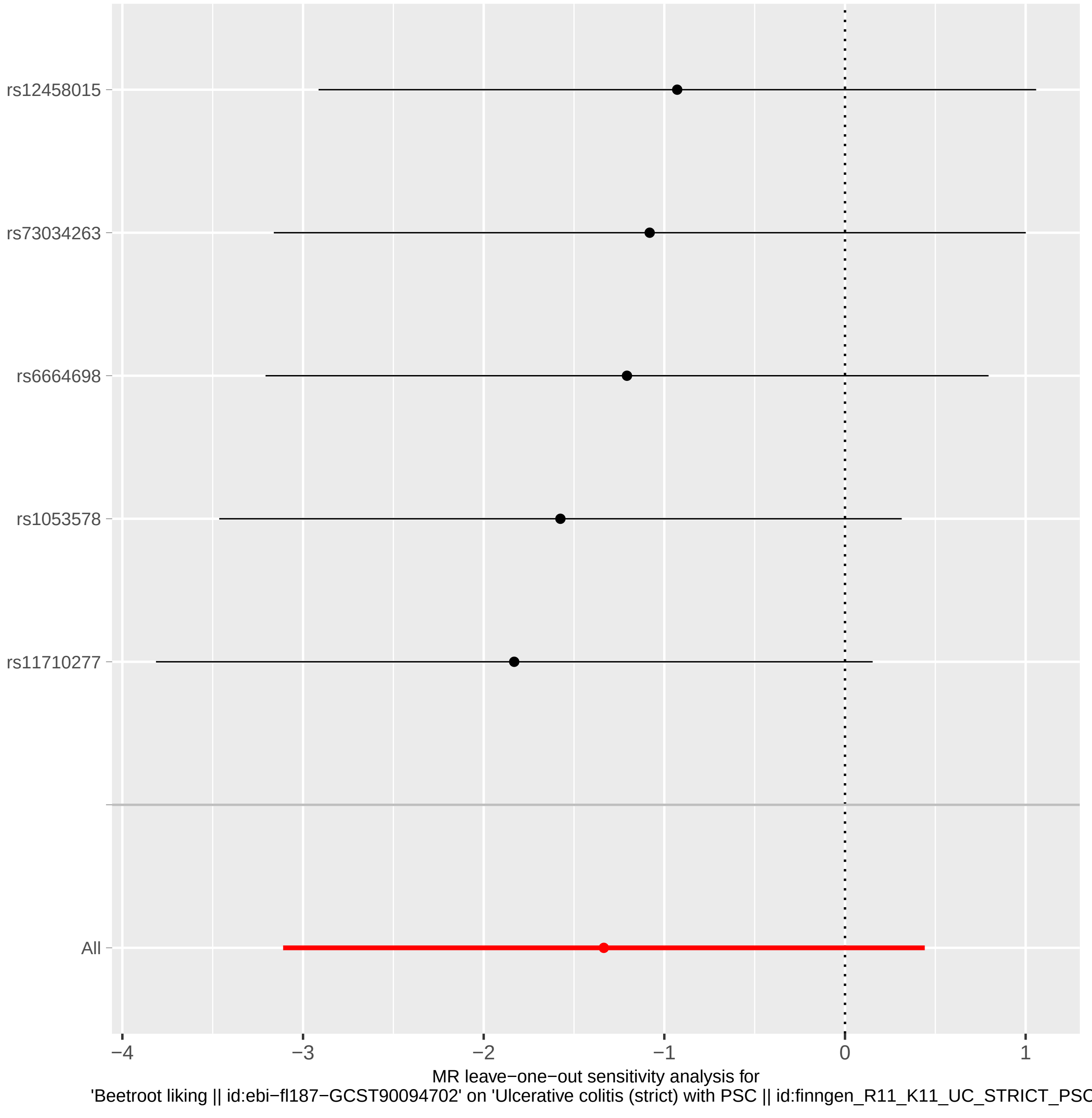

rs12458015
rs73034263
rs6664698
rs1053578
rs11710277
All
−4
−3
−2
−1
0
1
MR leave−one−out sensitivity analysis for
'Beetroot liking || id:ebi−fl187−GCST90094702' on 'Ulcerative colitis (strict) with PSC || id:finngen_R11_K11_UC_STRICT_PSC

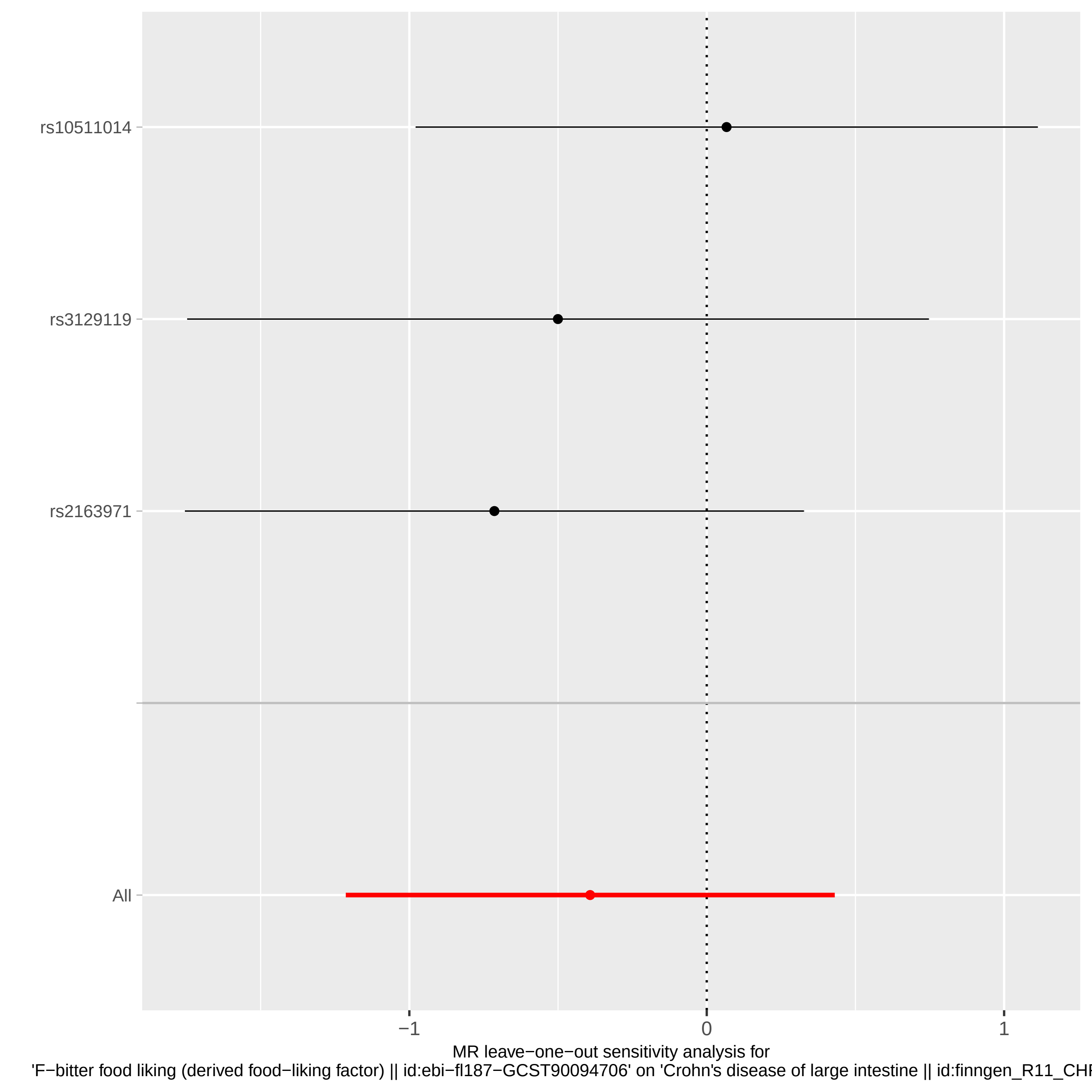
rs10511014
rs3129119
rs2163971
All
−1
0
1
MR leave−one−out sensitivity analysis for
'F−bitter food liking (derived food−liking factor) || id:ebi−fl187−GCST90094706' on 'Crohn's disease of large intestine || id:finngen_R11_CH

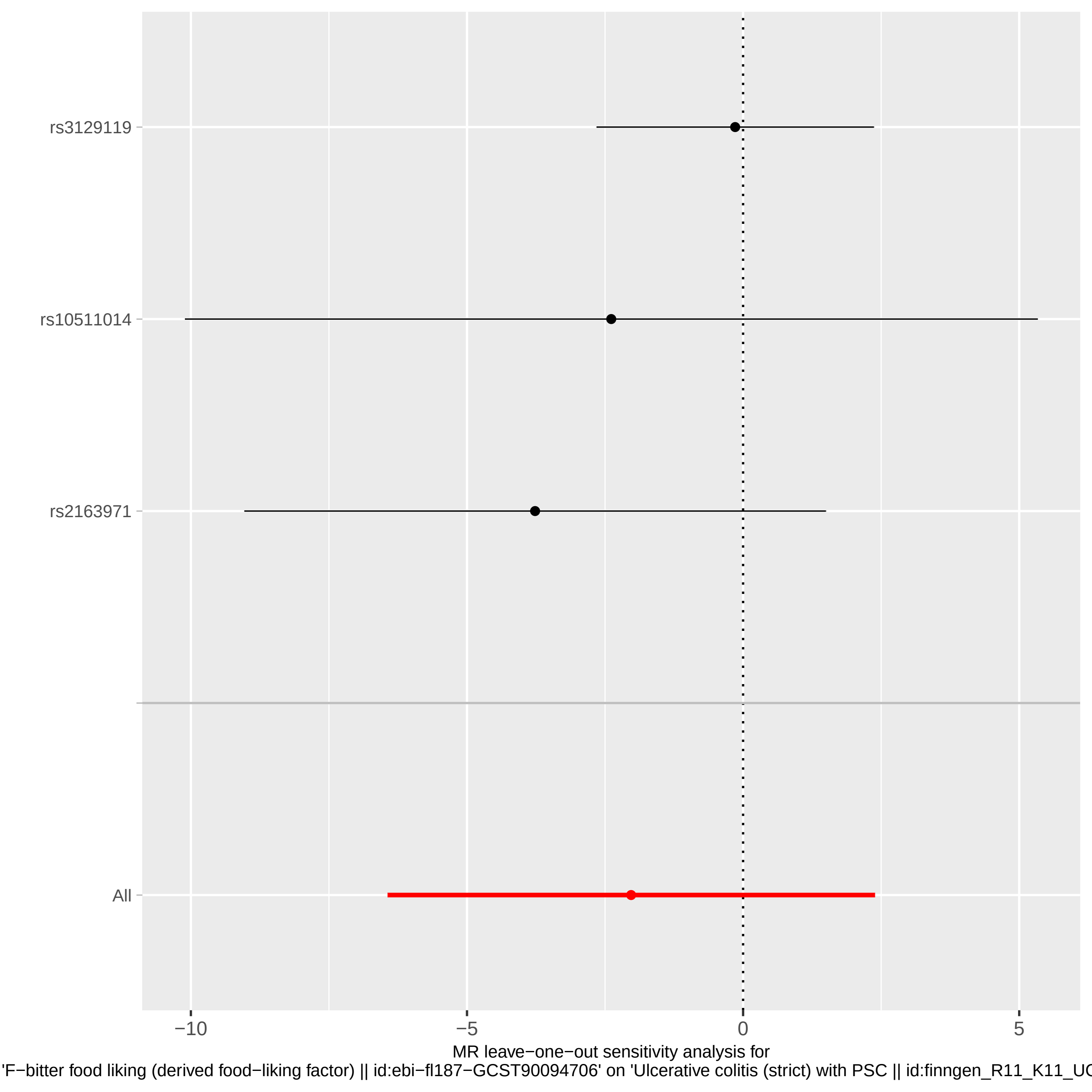
rs3129119
rs10511014
rs2163971
All
−10
−5
0
5
MR leave−one−out sensitivity analysis for
'F−bitter food liking (derived food−liking factor) || id:ebi−fl187−GCST90094706' on 'Ulcerative colitis (strict) with PSC || id:finngen_R11_K11_UC

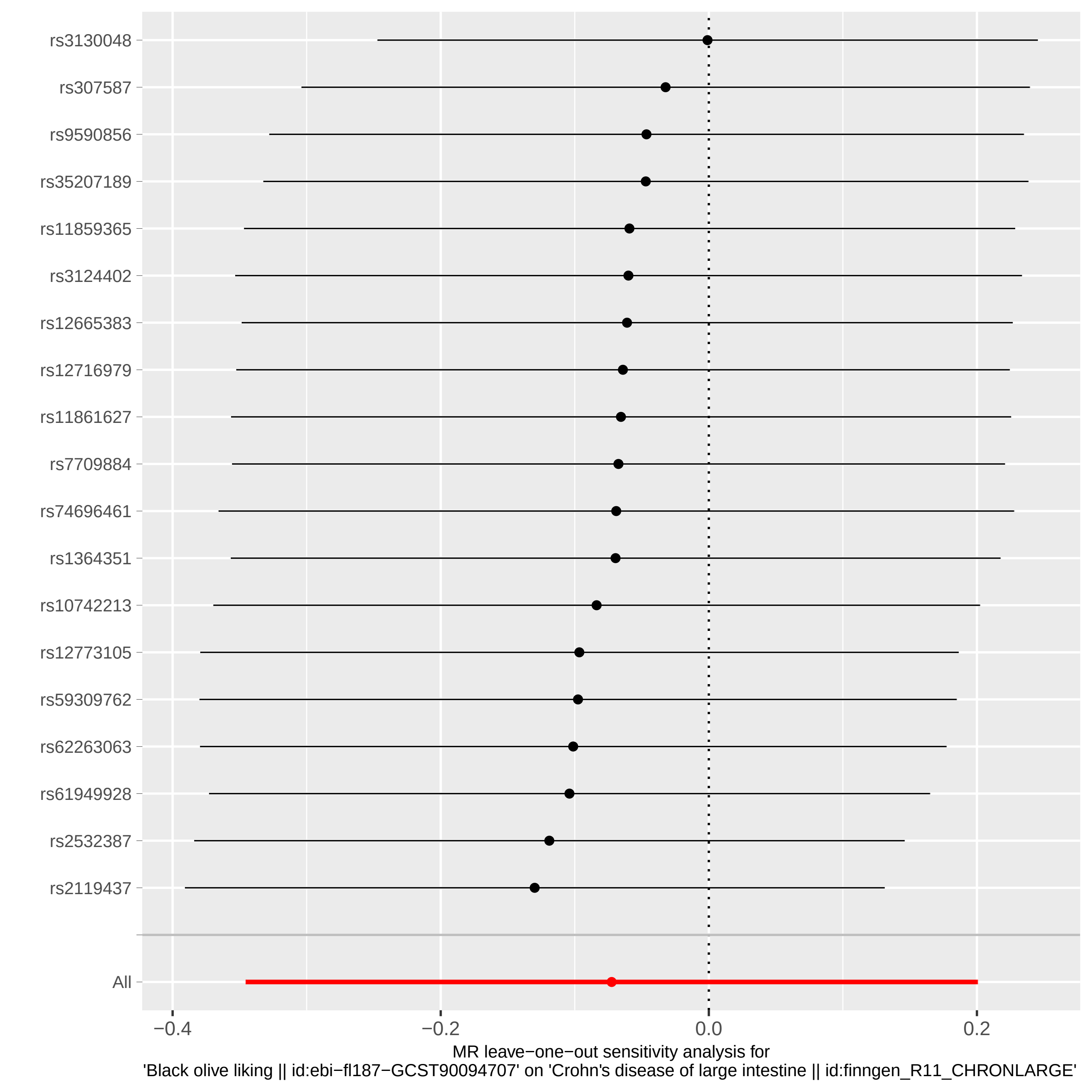
rs3130048
rs307587
rs9590856
rs35207189
rs11859365
rs3124402
rs12665383
rs12716979
rs11861627
rs7709884
rs74696461
rs1364351
rs10742213
rs12773105
rs59309762
rs62263063
rs61949928
rs2532387
rs2119437
All
−0.4
−0.2
0.0
0.2
MR leave−one−out sensitivity analysis for
'Black olive liking || id:ebi−fl187−GCST90094707' on 'Crohn's disease of large intestine || id:finngen_R11_CHRONLARGE'

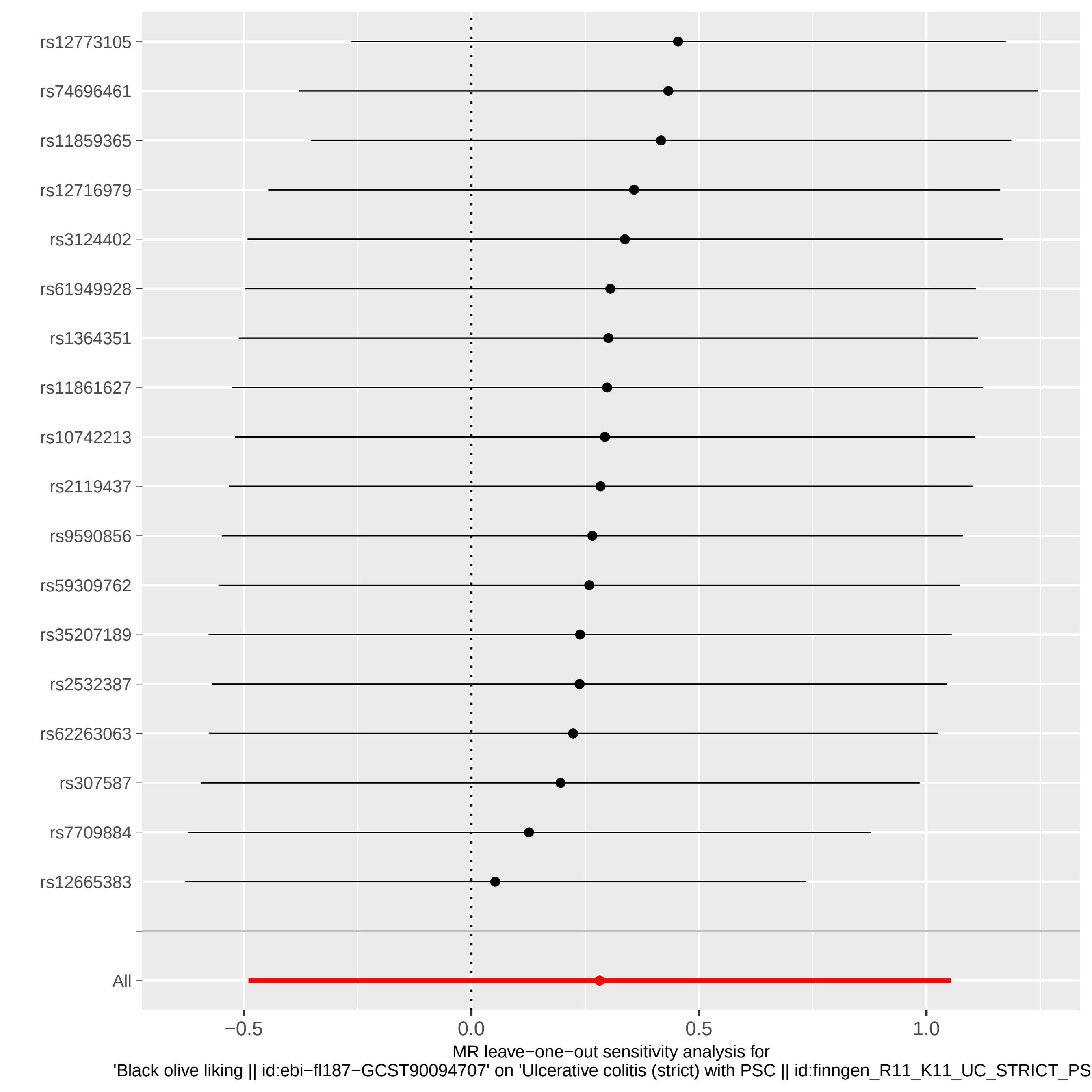
rs12773105
rs74696461
rs11859365
rs12716979
rs3124402
rs61949928
rs1364351
rs11861627
rs10742213
rs2119437
rs9590856
rs59309762
rs35207189
rs2532387
rs62263063
rs307587
rs7709884
rs12665383
All
−0.5
0.0
0.5
1.0
MR leave−one−out sensitivity analysis for
'Black olive liking || id:ebi−fl187−GCST90094707' on 'Ulcerative colitis (strict) with PSC || id:finngen_R11_K11_UC_STRICT_PS

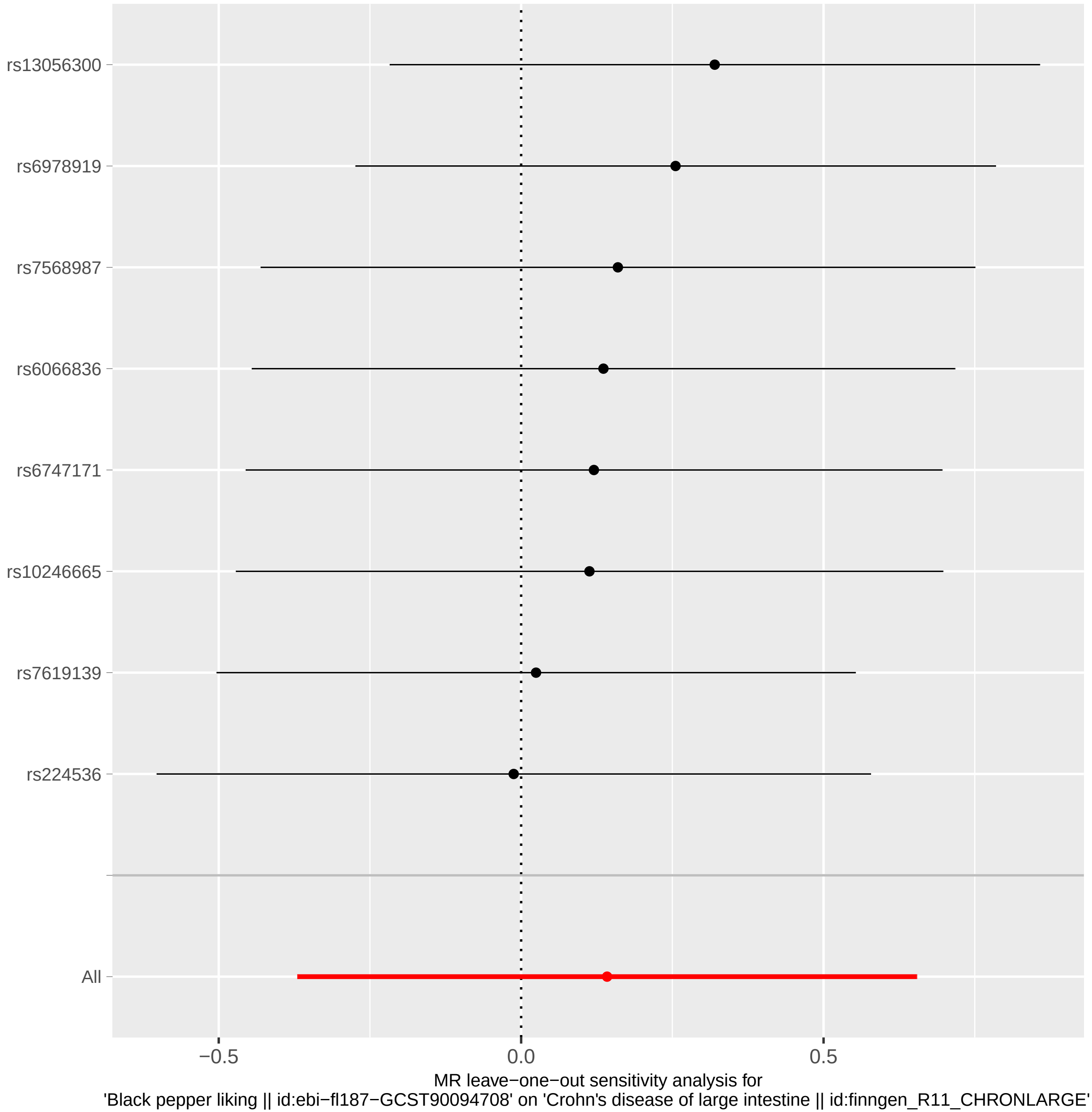
rs13056300
rs6978919
rs7568987
rs6066836
rs6747171
rs10246665
rs7619139
rs224536
All
−0.5
0.0
0.5
MR leave−one−out sensitivity analysis for
'Black pepper liking || id:ebi−fl187−GCST90094708' on 'Crohn's disease of large intestine || id:finngen_R11_CHRONLARGE'

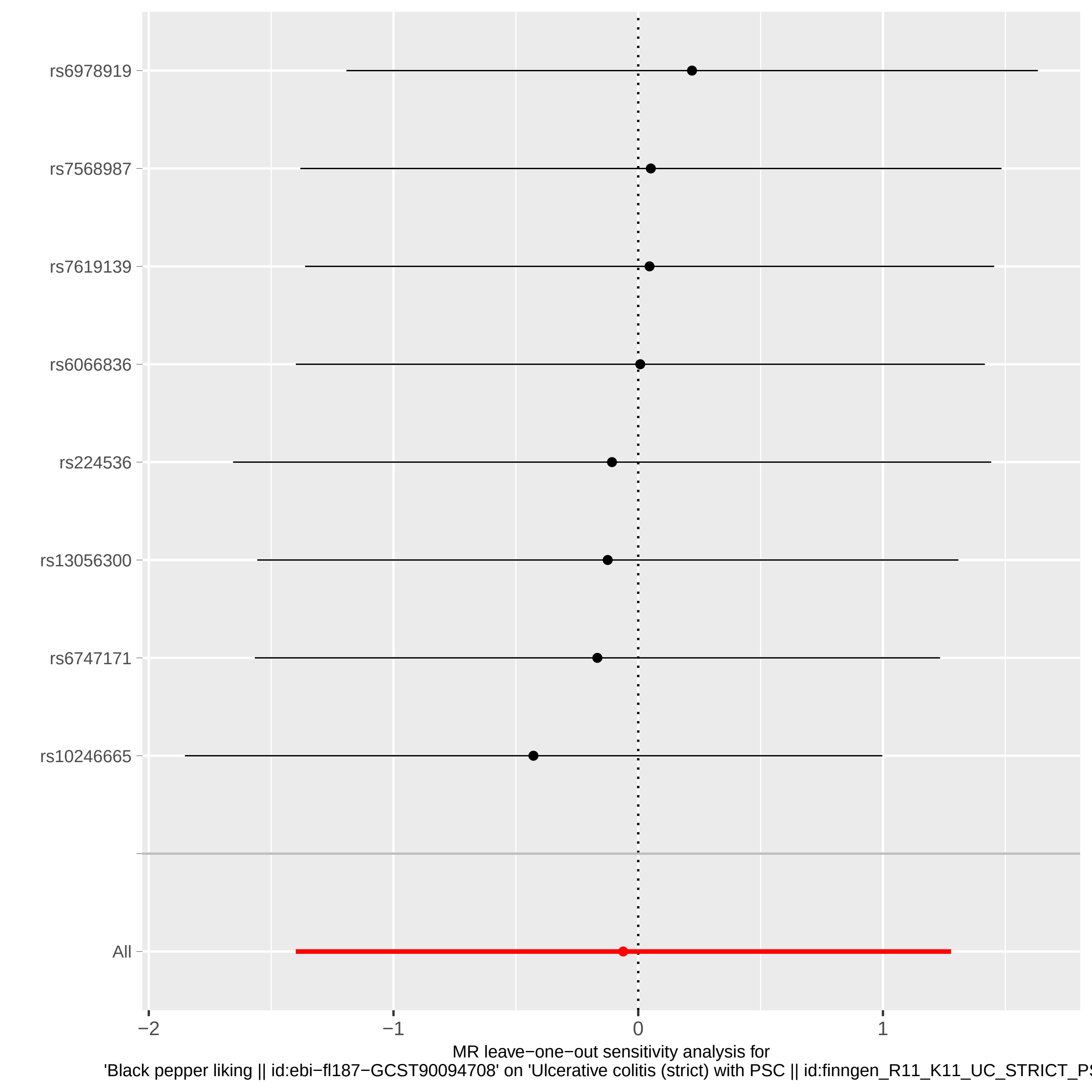
rs6978919
rs7568987
rs7619139
rs6066836
rs224536
rs13056300
rs6747171
rs10246665
All
−2
−1
0
1
MR leave−one−out sensitivity analysis for
'Black pepper liking || id:ebi−fl187−GCST90094708' on 'Ulcerative colitis (strict) with PSC || id:finngen_R11_K11_UC_STRICT_PS

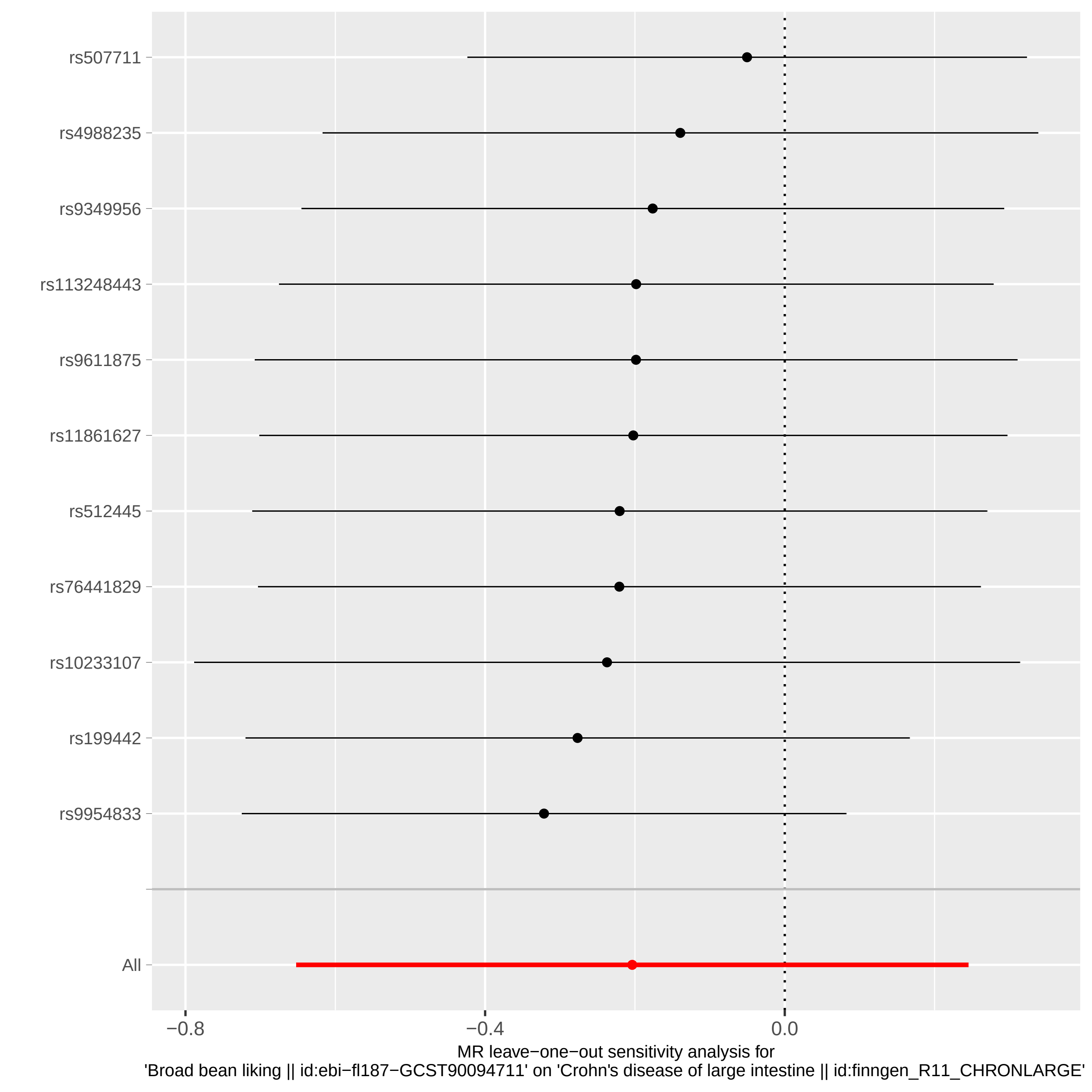

rs507711
rs4988235
rs9349956
rs113248443
rs9611875
rs11861627
rs512445
rs76441829
rs10233107
rs199442
rs9954833
All
−0.8
−0.4
0.0
MR leave−one−out sensitivity analysis for
'Broad bean liking || id:ebi−fl187−GCST90094711' on 'Crohn's disease of large intestine || id:finngen_R11_CHRONLARGE'

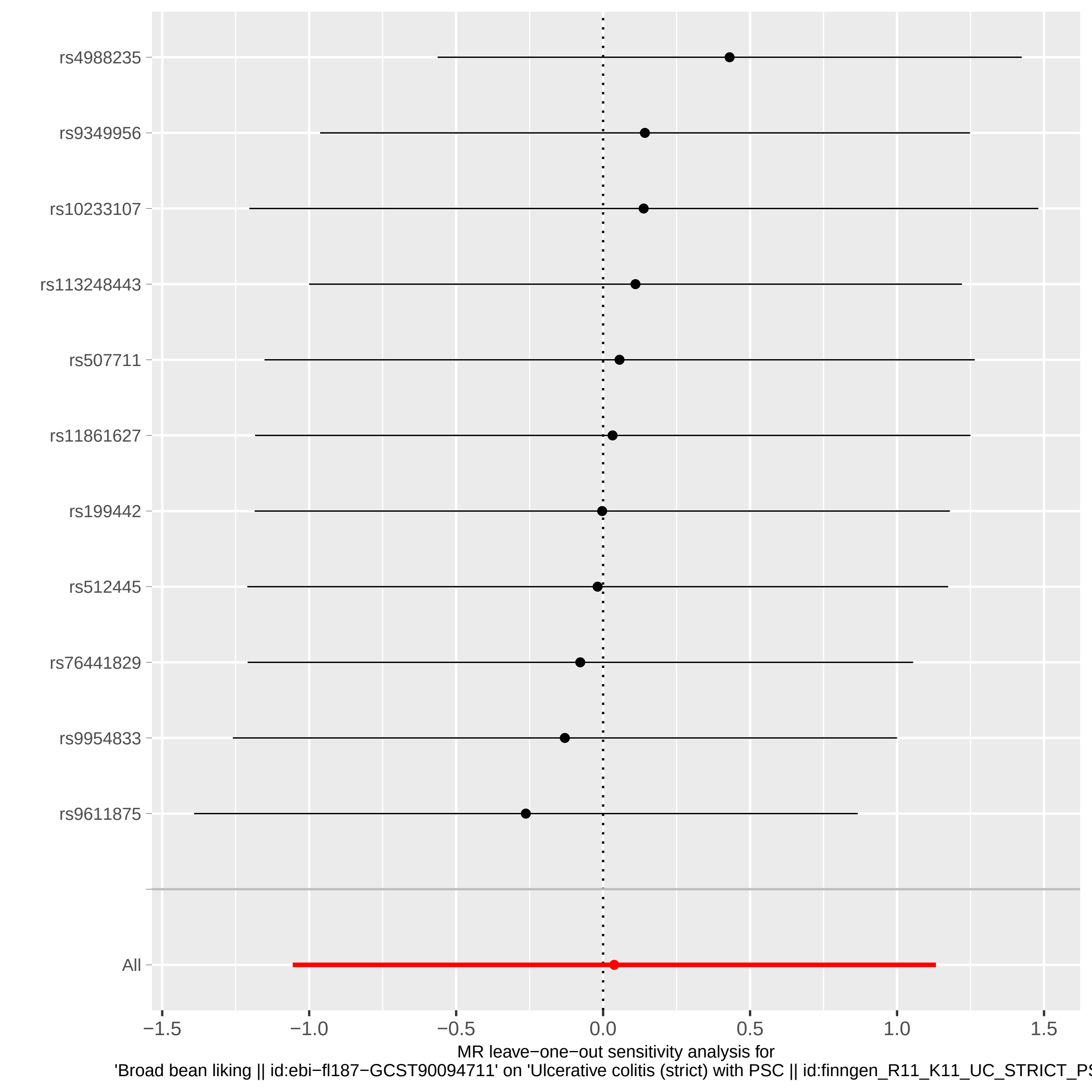
rs4988235
rs9349956
rs10233107
rs113248443
rs507711
rs11861627
rs199442
rs512445
rs76441829
rs9954833
rs9611875
All
−1.5
−1.0
−0.5
0.0
0.5
1.0
1.5
MR leave−one−out sensitivity analysis for
'Broad bean liking || id:ebi−fl187−GCST90094711' on 'Ulcerative colitis (strict) with PSC || id:finngen_R11_K11_UC_STRICT_PS

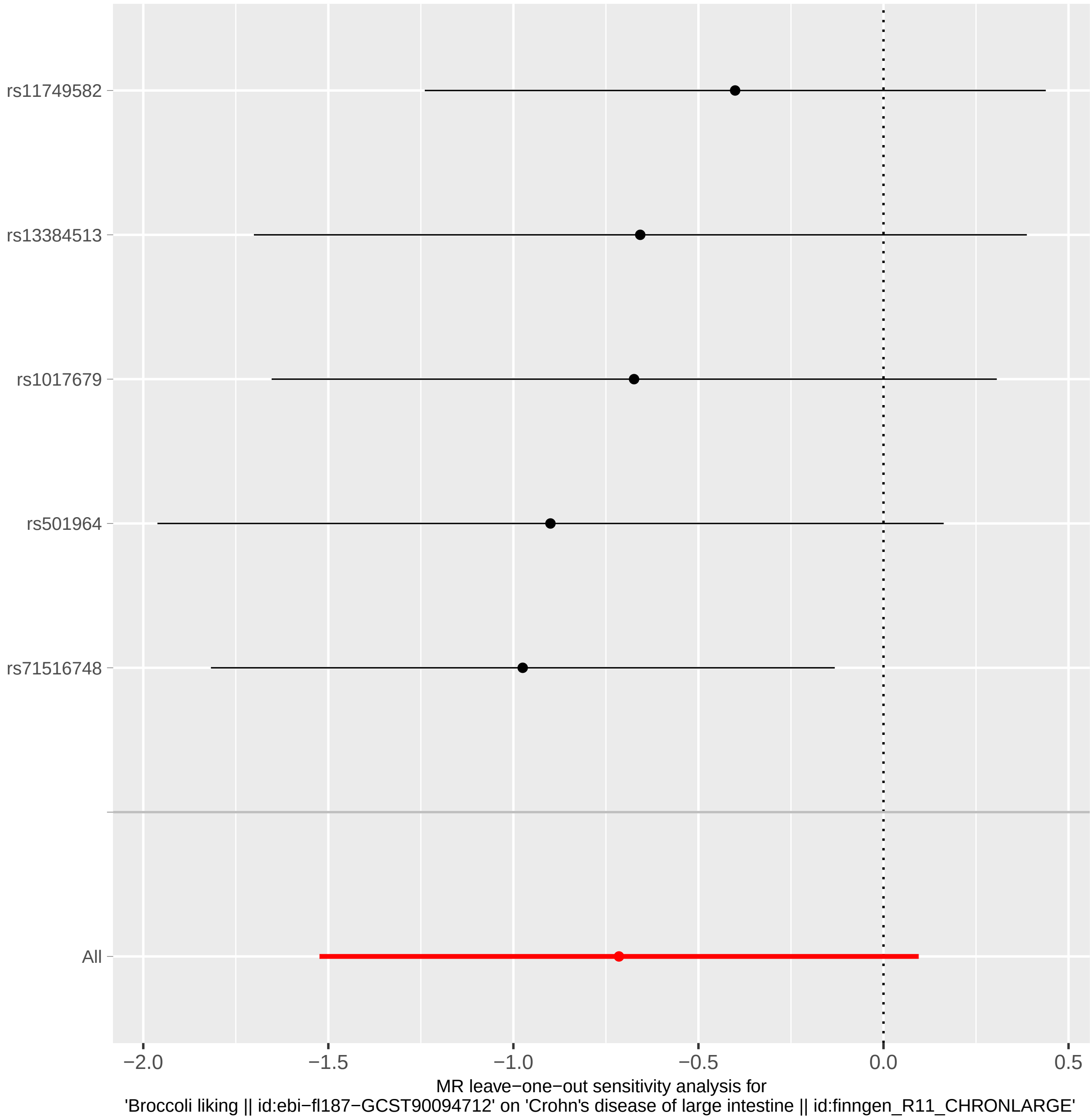
rs11749582
rs13384513
rs1017679
rs501964
rs71516748
All
−2.0
−1.5
−1.0
−0.5
0.0
0.5
MR leave−one−out sensitivity analysis for
'Broccoli liking || id:ebi−fl187−GCST90094712' on 'Crohn's disease of large intestine || id:finngen_R11_CHRONLARGE'

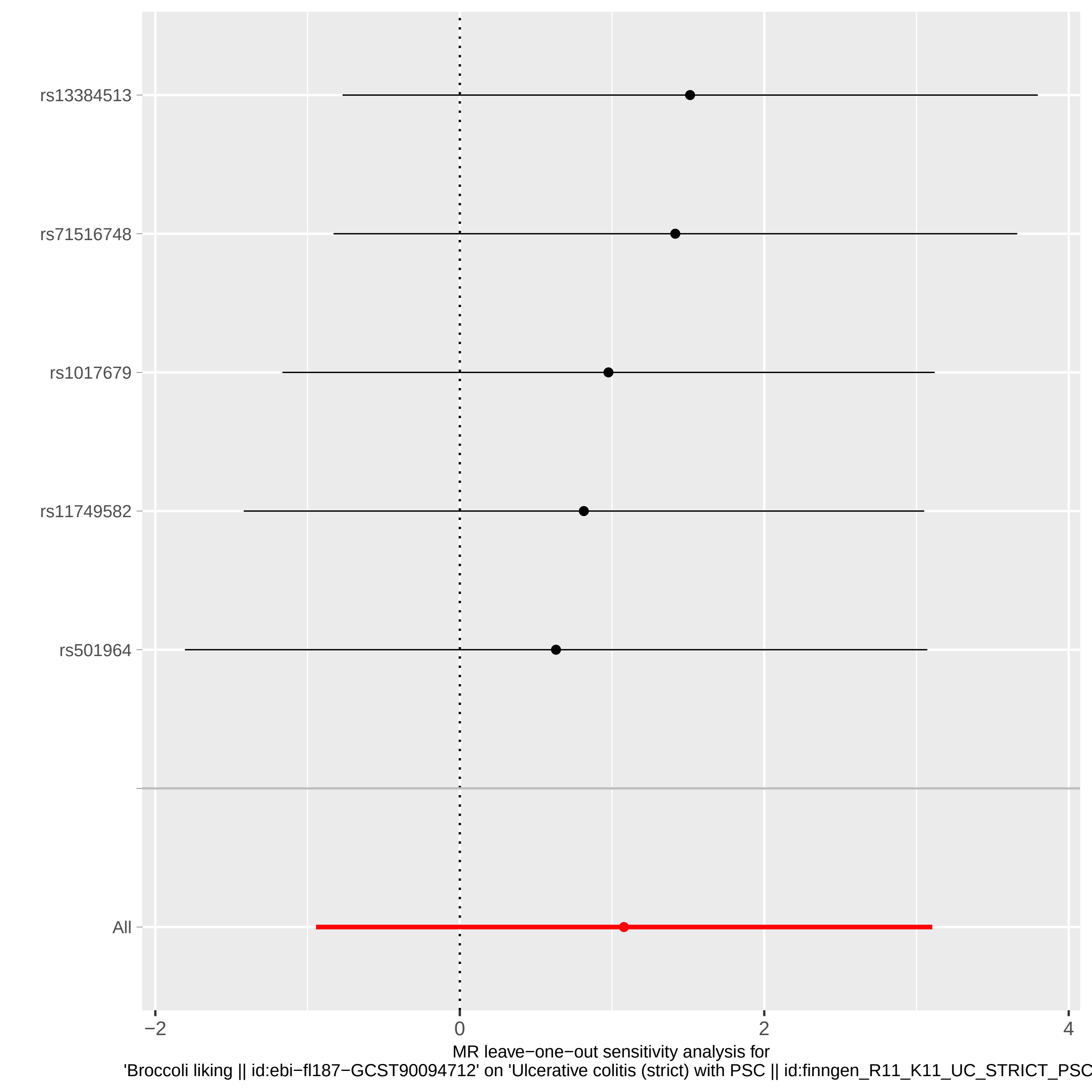
rs13384513
rs71516748
rs1017679
rs11749582
rs501964
All
−2
0
2
4
MR leave−one−out sensitivity analysis for
'Broccoli liking || id:ebi−fl187−GCST90094712' on 'Ulcerative colitis (strict) with PSC || id:finngen_R11_K11_UC_STRICT_PSC

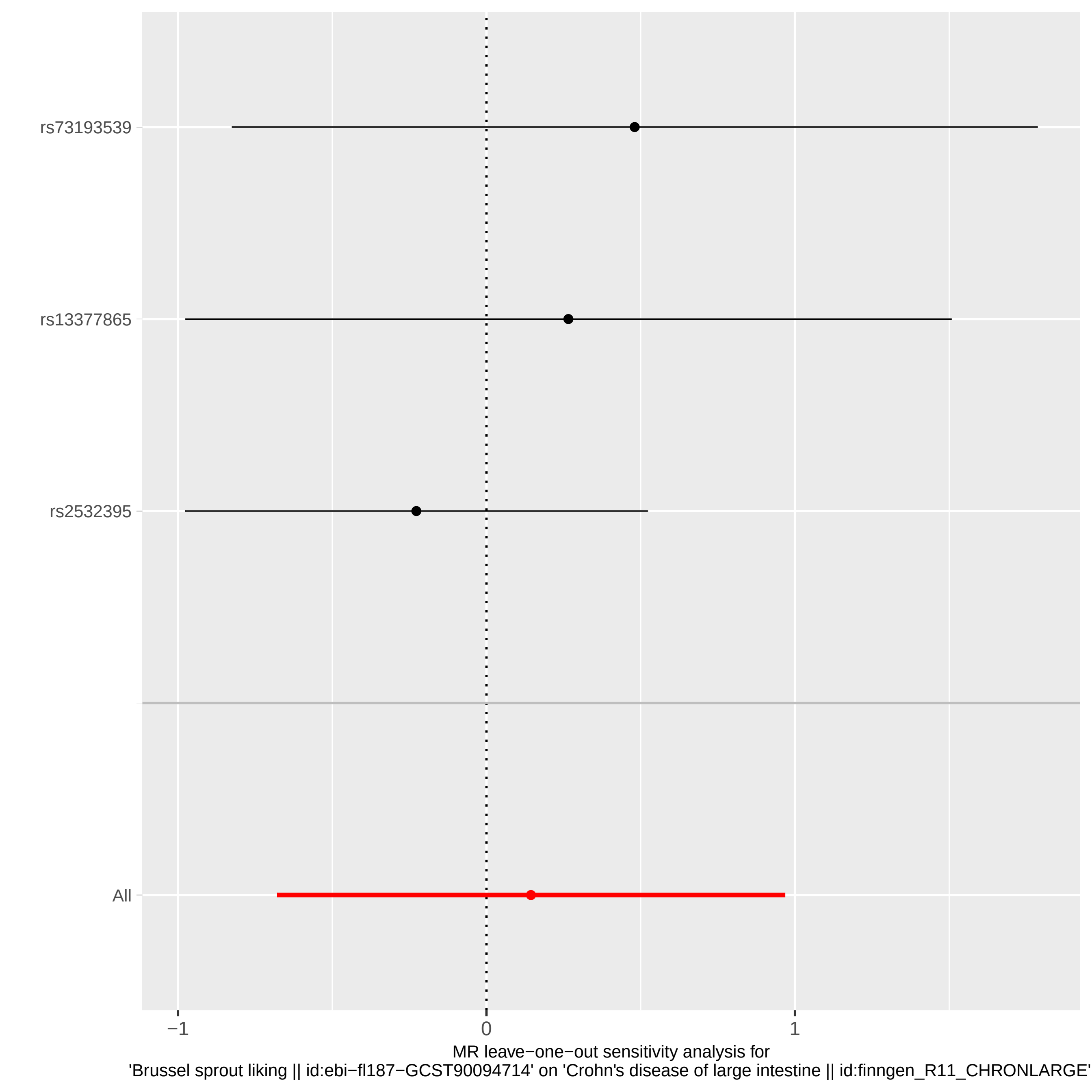
rs73193539
rs13377865
rs2532395
All
−1
0
1
MR leave−one−out sensitivity analysis for
'Brussel sprout liking || id:ebi−fl187−GCST90094714' on 'Crohn's disease of large intestine || id:finngen_R11_CHRONLARGE'

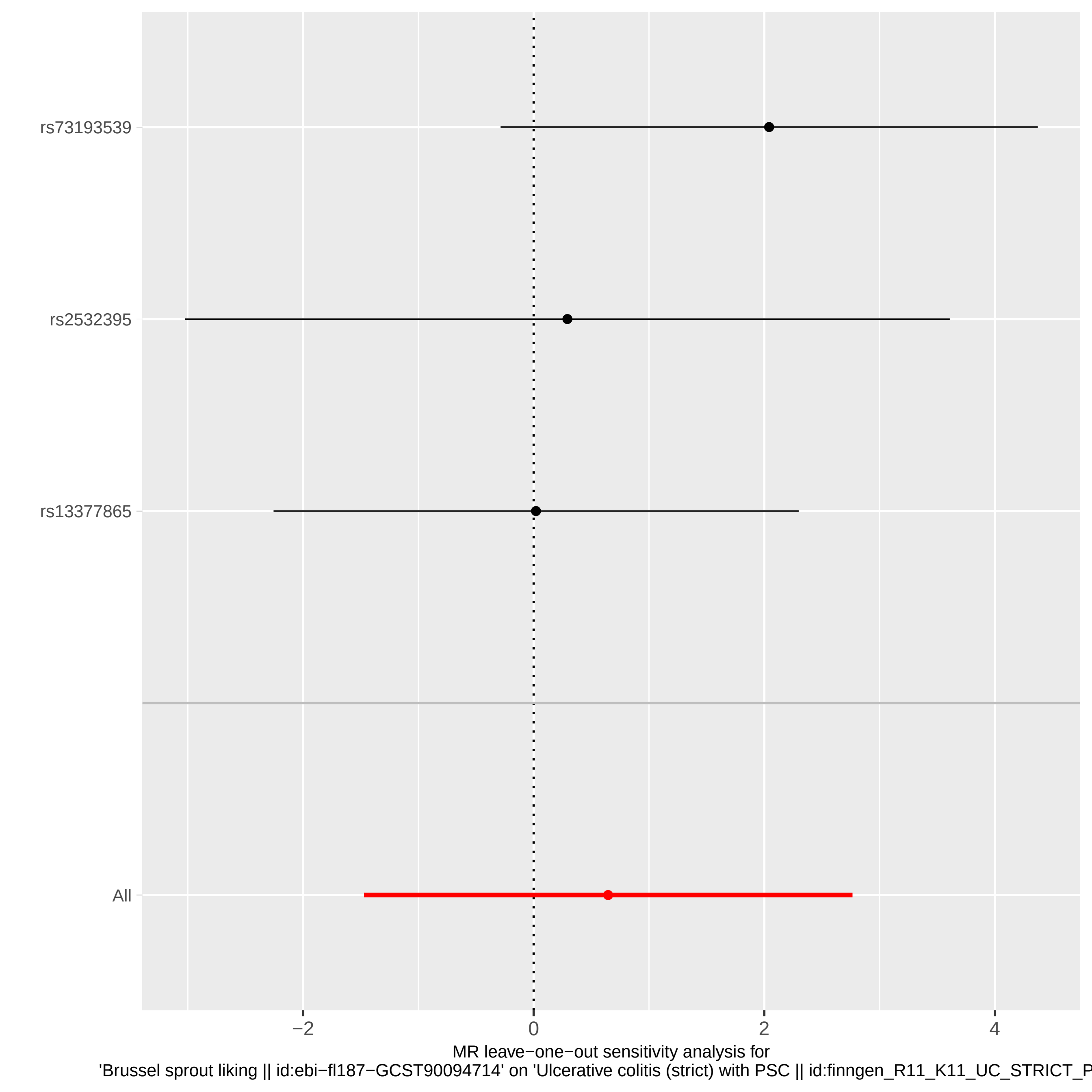

rs73193539
rs2532395
rs13377865
All
−2
0
2
4
MR leave−one−out sensitivity analysis for
'Brussel sprout liking || id:ebi−fl187−GCST90094714' on 'Ulcerative colitis (strict) with PSC || id:finngen_R11_K11_UC_STRICT_P

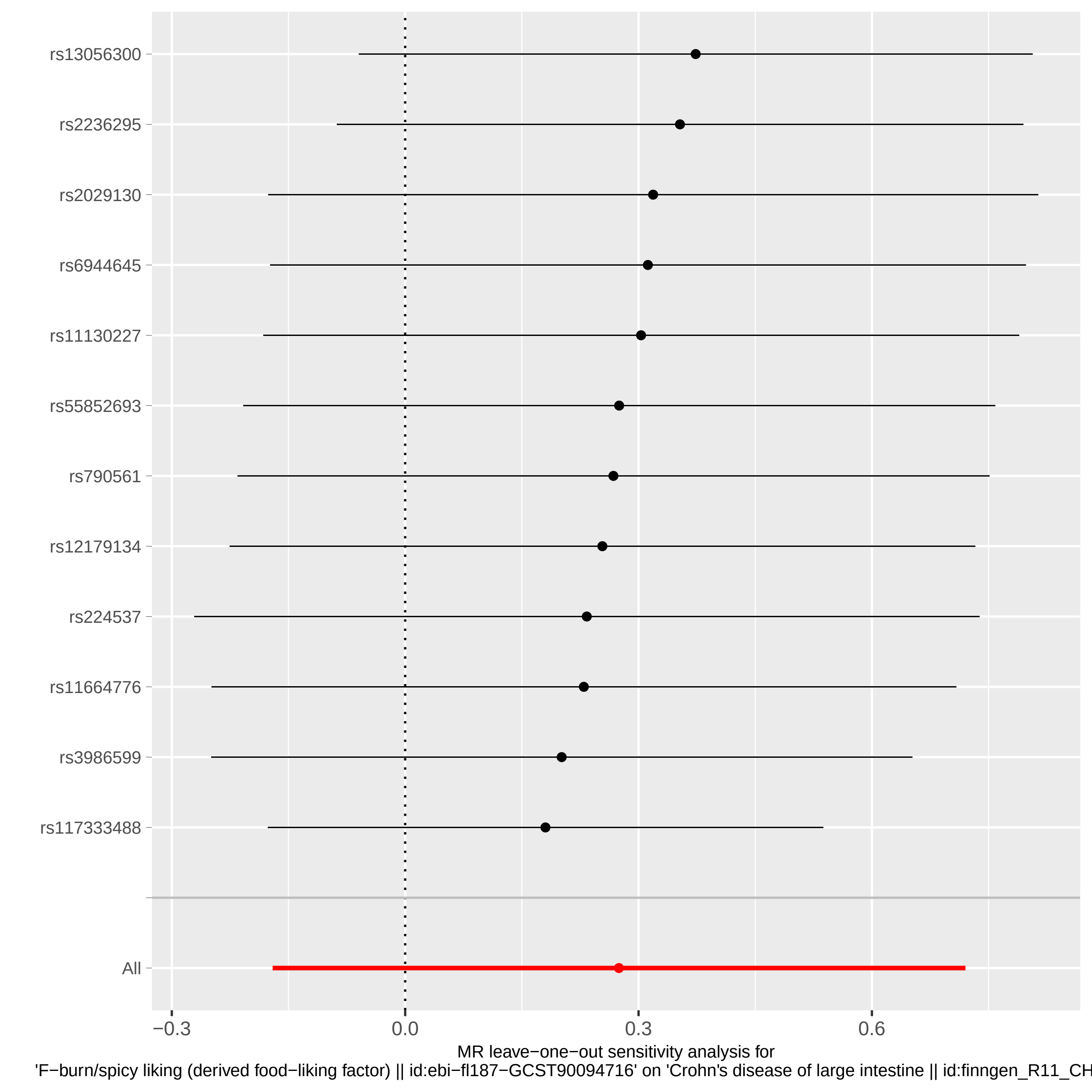
rs13056300
rs2236295
rs2029130
rs6944645
rs11130227
rs55852693
rs790561
rs12179134
rs224537
rs11664776
rs3986599
rs117333488
All
−0.3
0.0
0.3
0.6
MR leave−one−out sensitivity analysis for
'F−burn/spicy liking (derived food−liking factor) || id:ebi−fl187−GCST90094716' on 'Crohn's disease of large intestine || id:finngen_R11_CH

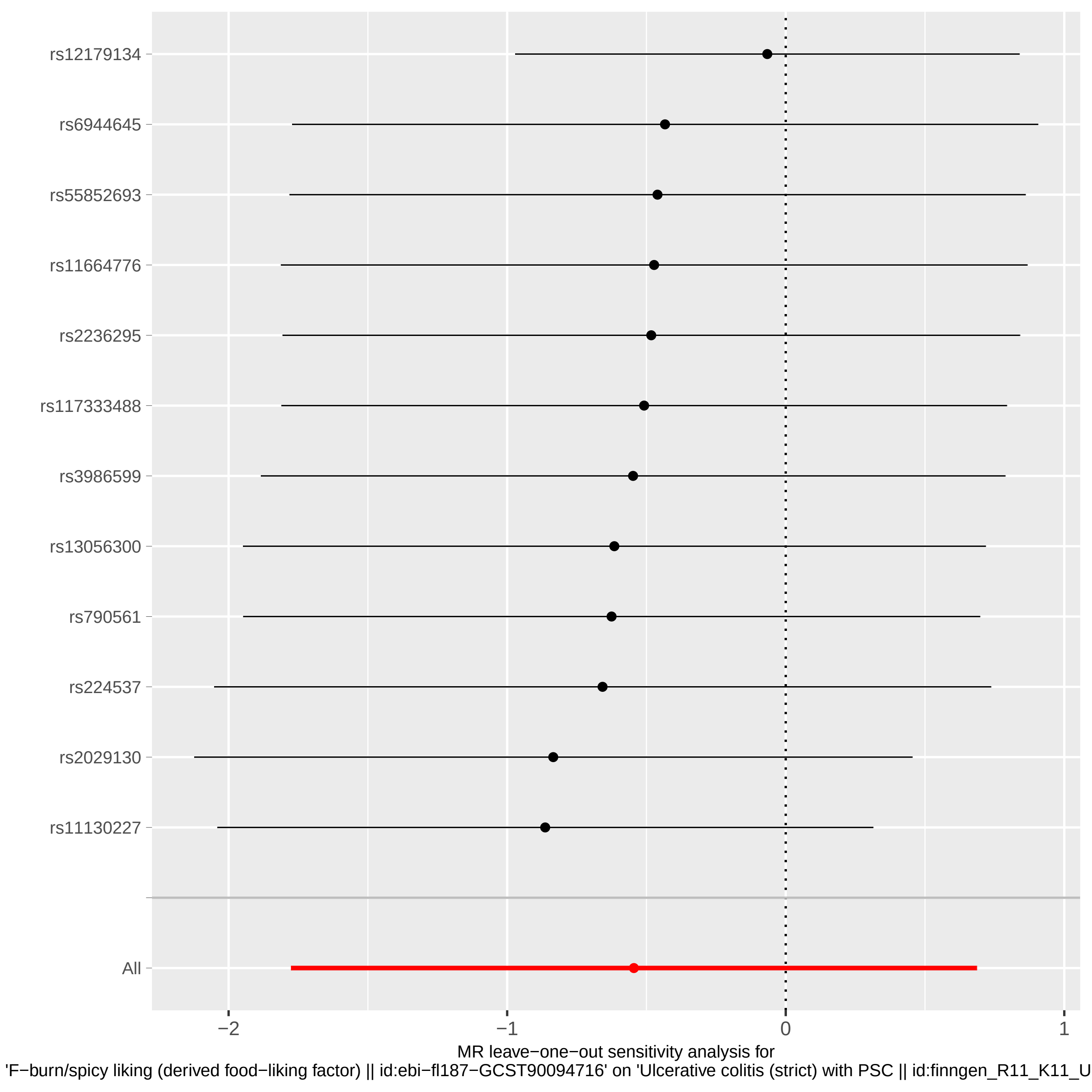

rs12179134
rs6944645
rs55852693
rs11664776
rs2236295
rs117333488
rs3986599
rs13056300
rs790561
rs224537
rs2029130
rs11130227
All
−2
−1
0
1
MR leave−one−out sensitivity analysis for
'F−burn/spicy liking (derived food−liking factor) || id:ebi−fl187−GCST90094716' on 'Ulcerative colitis (strict) with PSC || id:finngen_R11_K11_U

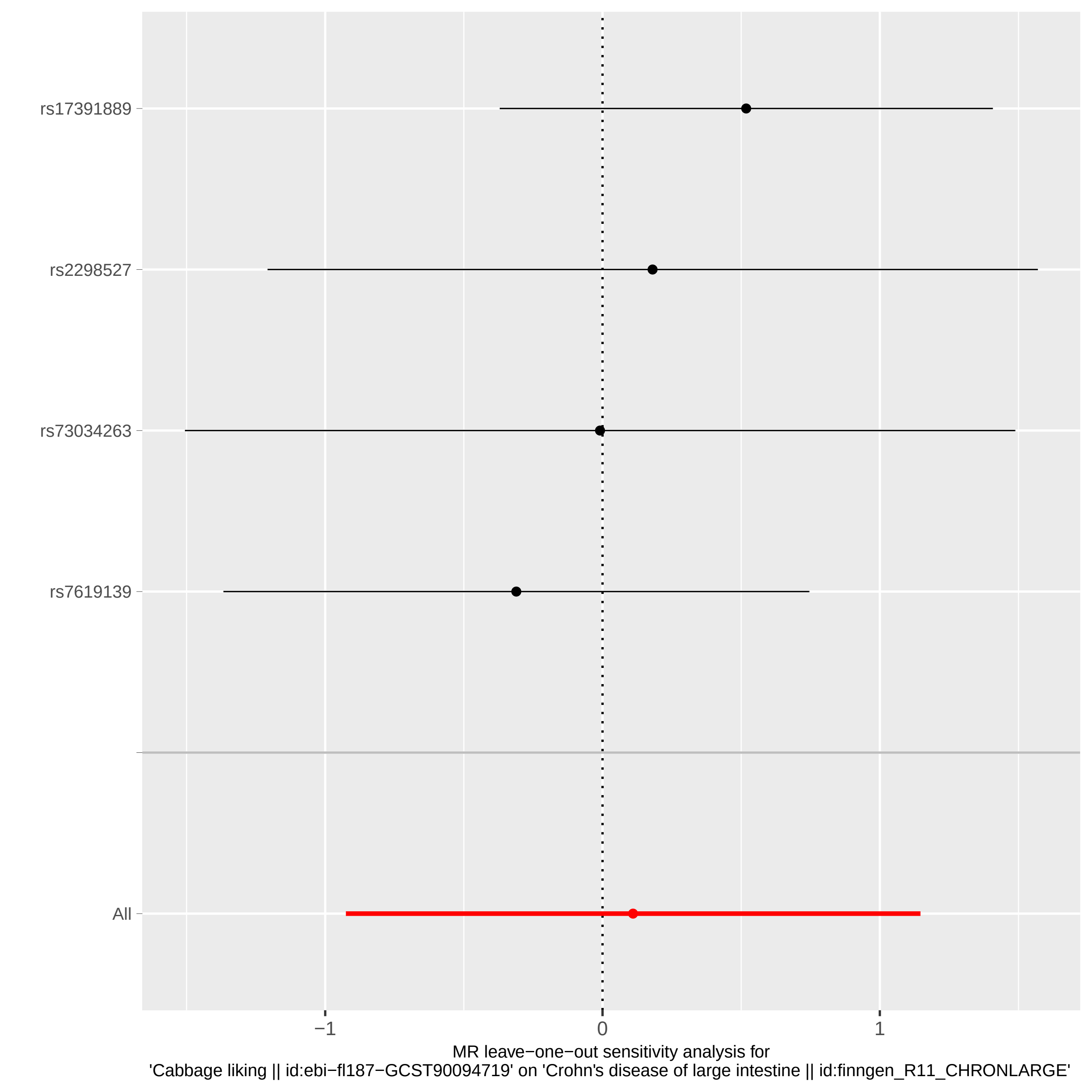
rs17391889
rs2298527
rs73034263
rs7619139
All
−1
0
1
MR leave−one−out sensitivity analysis for
'Cabbage liking || id:ebi−fl187−GCST90094719' on 'Crohn's disease of large intestine || id:finngen_R11_CHRONLARGE'

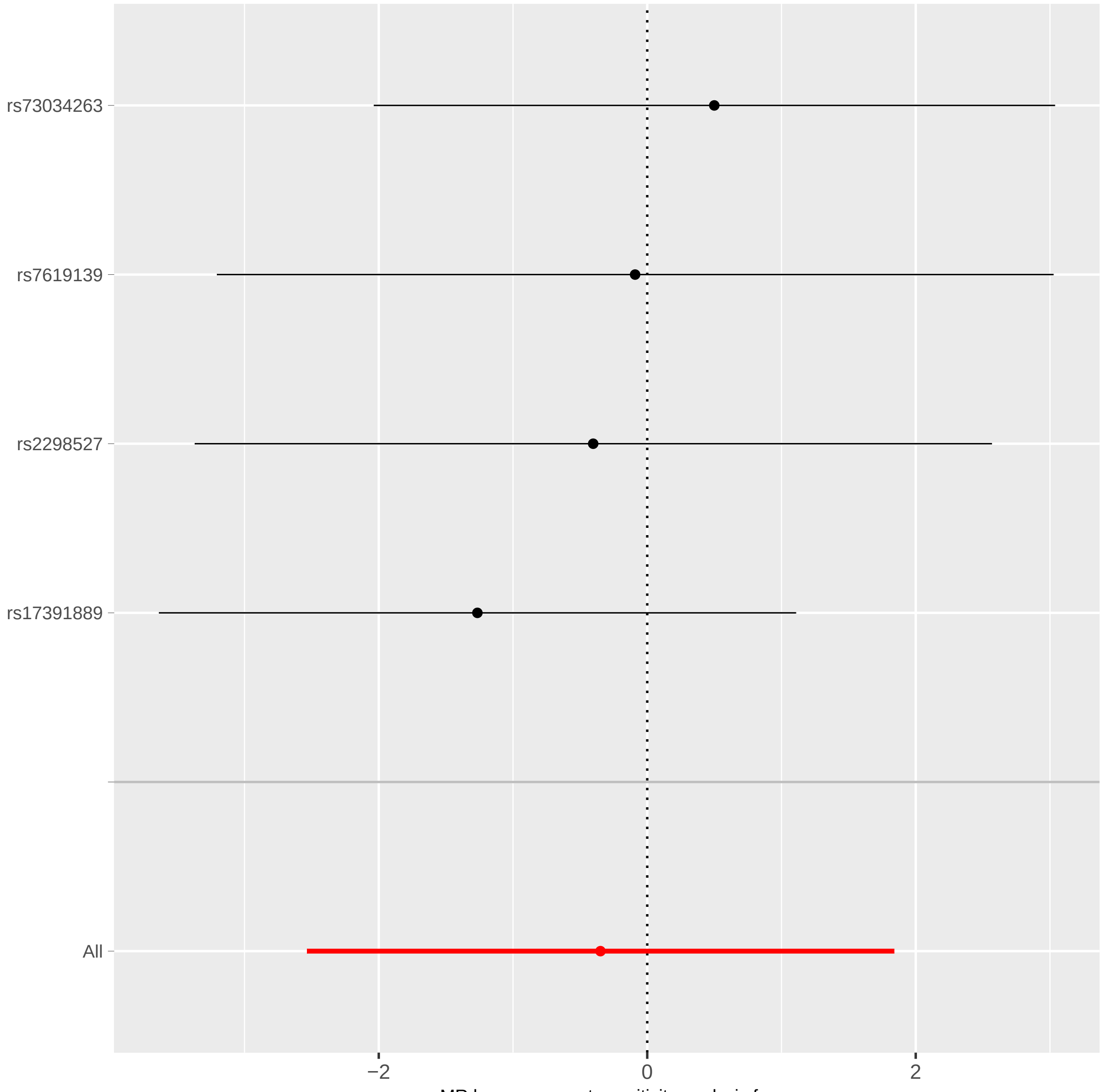

MR leave−one−out sensitivity analysis for
'Cabbage liking || id:ebi−fl187−GCST90094719' on 'Ulcerative colitis (strict) with PSC || id:finngen_R11_K11_UC_STRICT_PSC

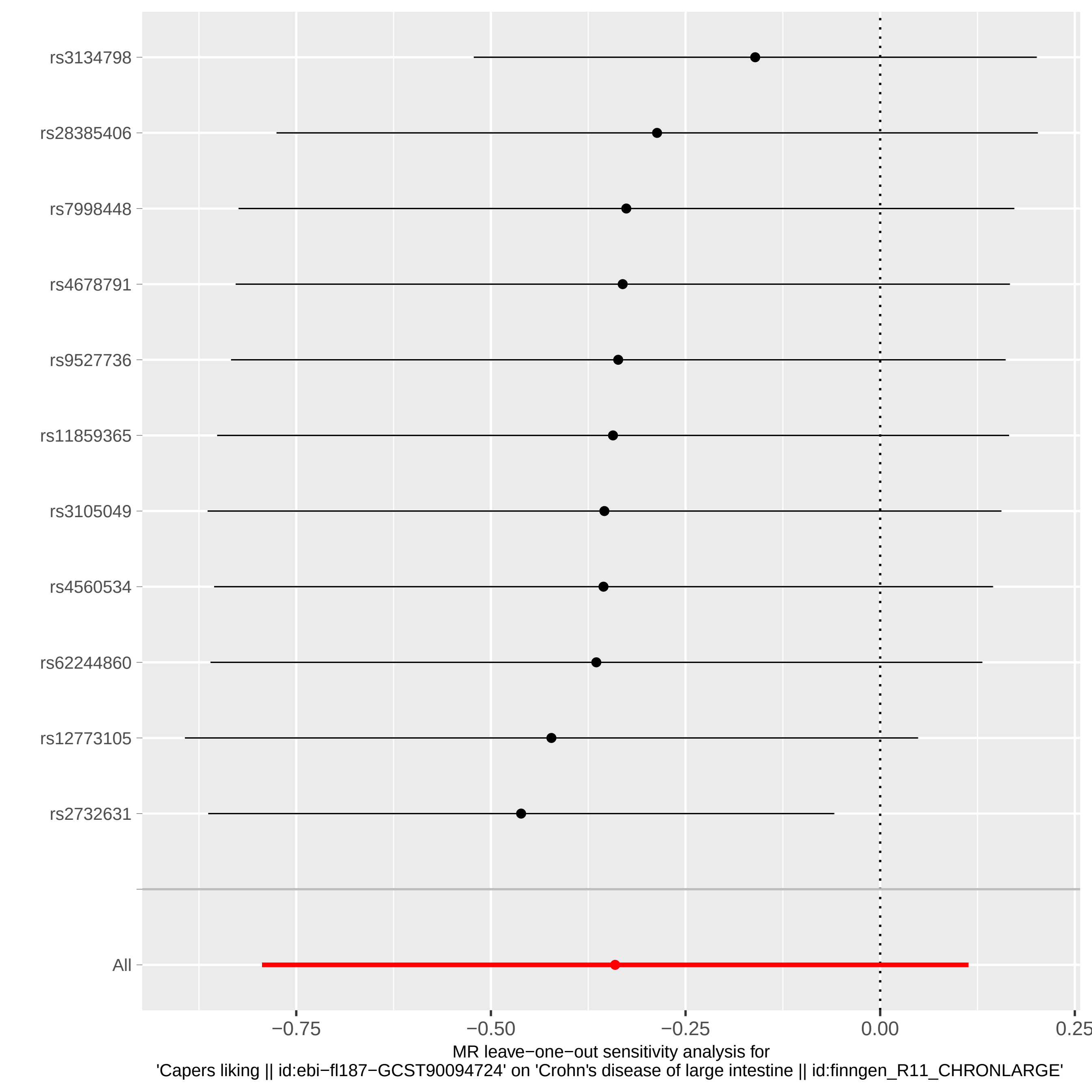

rs3134798
rs28385406
rs7998448
rs4678791
rs9527736
rs11859365
rs3105049
rs4560534
rs62244860
rs12773105
rs2732631
All
−0.75
−0.50
−0.25
0.00
0.25
MR leave−one−out sensitivity analysis for
'Capers liking || id:ebi−fl187−GCST90094724' on 'Crohn's disease of large intestine || id:finngen_R11_CHRONLARGE'

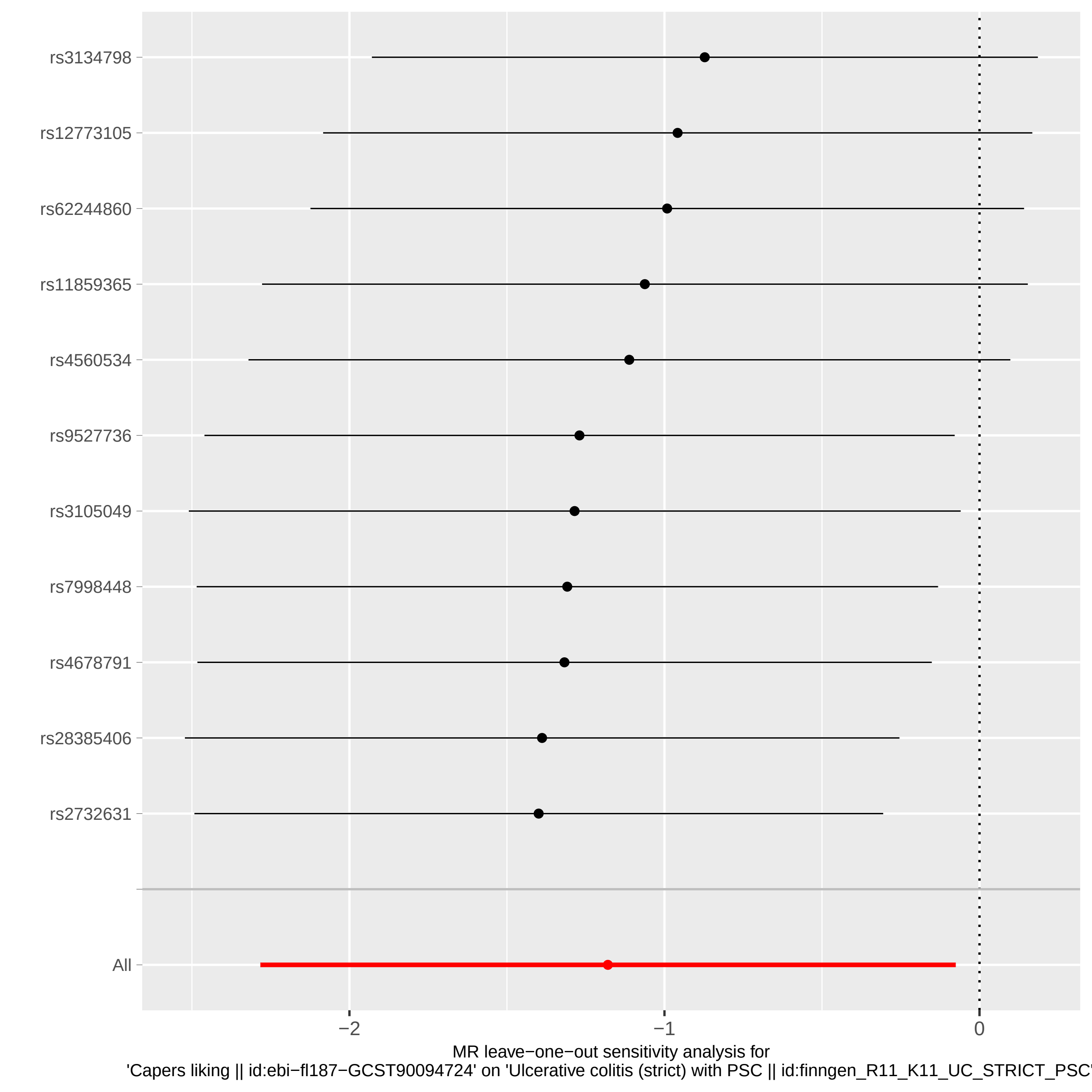
rs3134798
rs12773105
rs62244860
rs11859365
rs4560534
rs9527736
rs3105049
rs7998448
rs4678791
rs28385406
rs2732631
All
−2
−1
0
MR leave−one−out sensitivity analysis for
'Capers liking || id:ebi−fl187−GCST90094724' on 'Ulcerative colitis (strict) with PSC || id:finngen_R11_K11_UC_STRICT_PSC

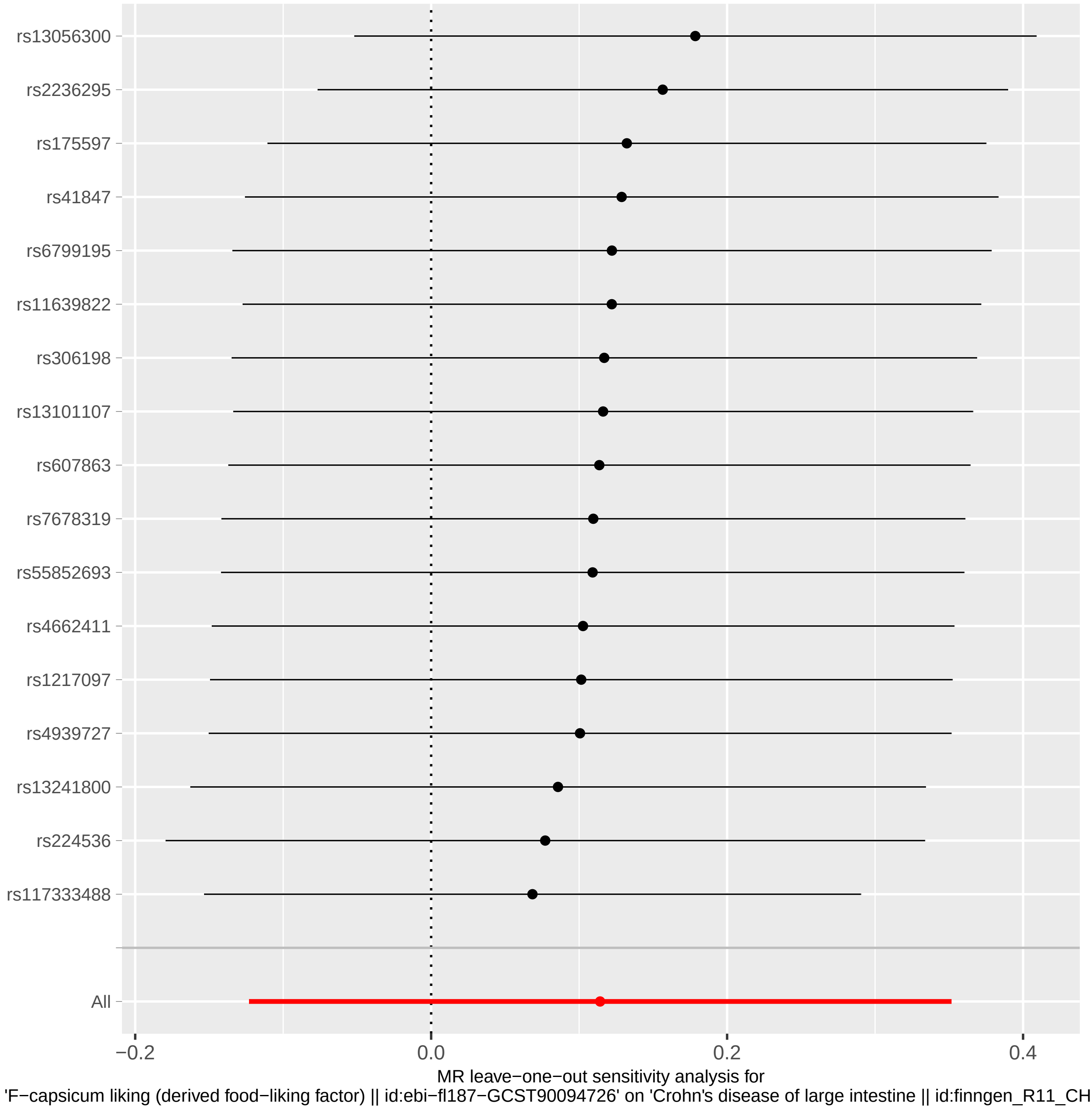

rs13056300
rs2236295
rs175597
rs41847
rs6799195
rs11639822
rs306198
rs13101107
rs607863
rs7678319
rs55852693
rs4662411
rs1217097
rs4939727
rs13241800
rs224536
rs117333488
All
−0.2
0.0
0.2
0.4
MR leave−one−out sensitivity analysis for
'F−capsicum liking (derived food−liking factor) || id:ebi−fl187−GCST90094726' on 'Crohn's disease of large intestine || id:finngen_R11_CH

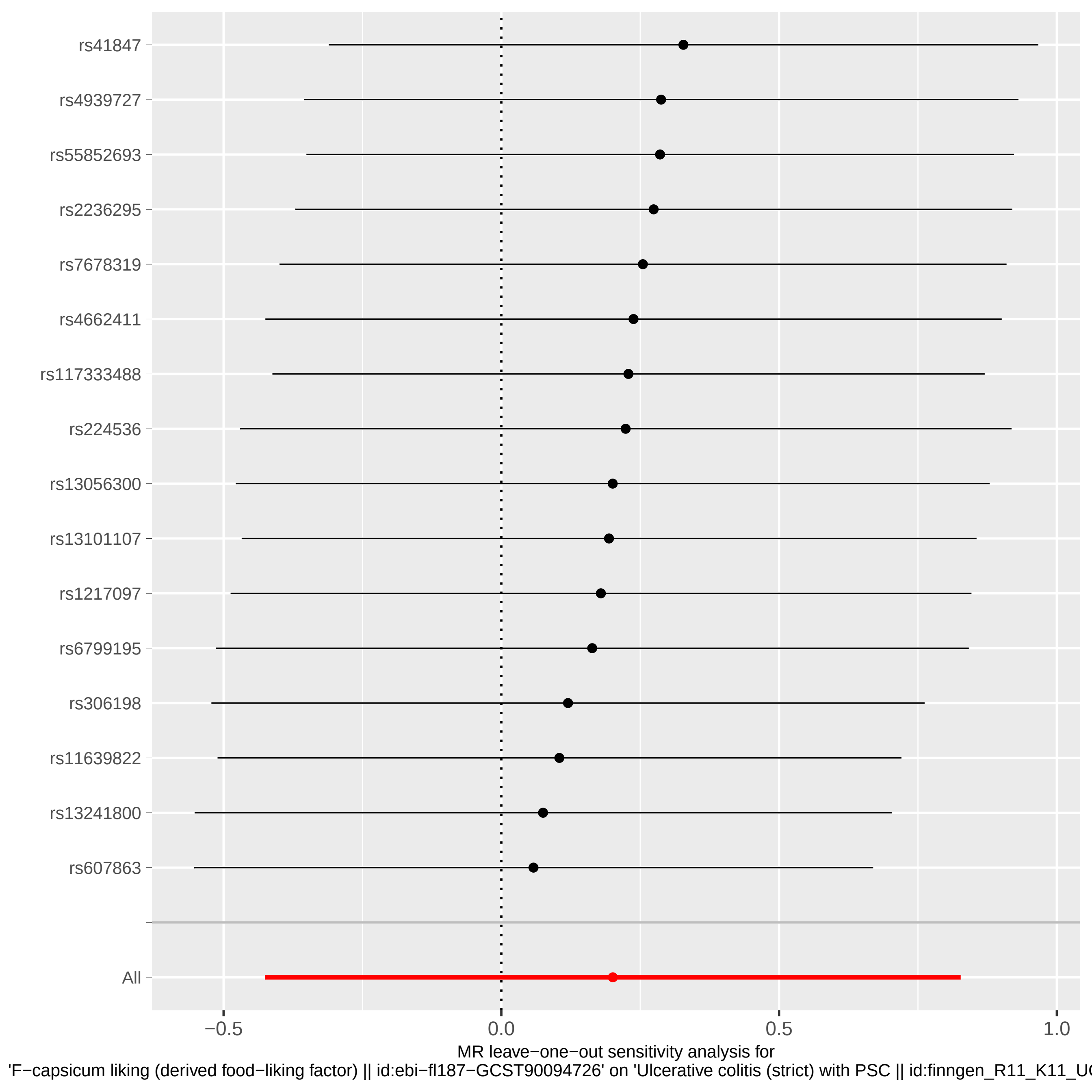

rs41847
rs4939727
rs55852693
rs2236295
rs7678319
rs4662411
rs117333488
rs224536
rs13056300
rs13101107
rs1217097
rs6799195
rs306198
rs11639822
rs13241800
rs607863
All
−0.5
0.0
0.5
1.0
MR leave−one−out sensitivity analysis for
'F−capsicum liking (derived food−liking factor) || id:ebi−fl187−GCST90094726' on 'Ulcerative colitis (strict) with PSC || id:finngen_R11_K11_U

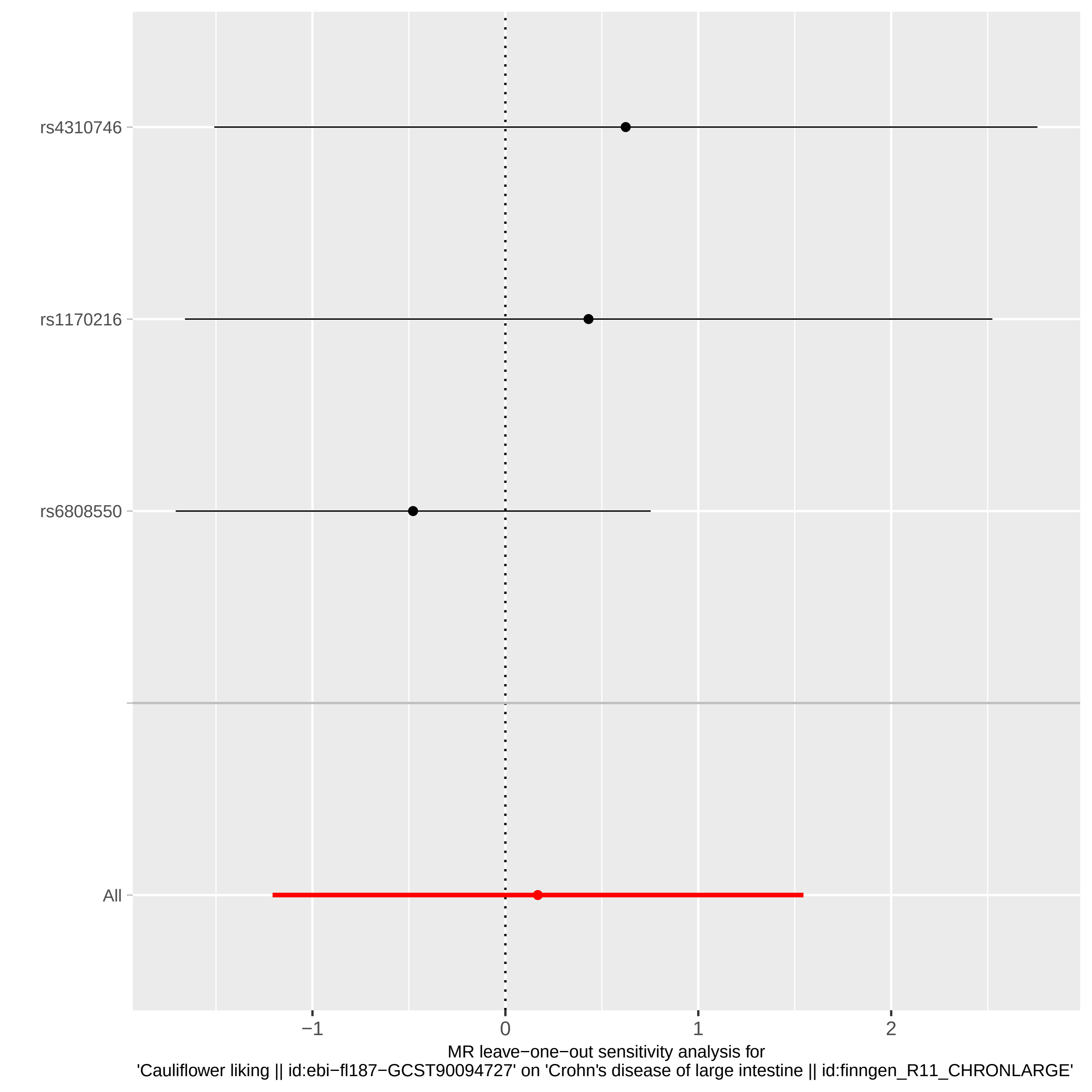
rs4310746
rs1170216
rs6808550
All
−1
0
1
2
MR leave−one−out sensitivity analysis for
'Cauliflower liking || id:ebi−fl187−GCST90094727' on 'Crohn's disease of large intestine || id:finngen_R11_CHRONLARGE'

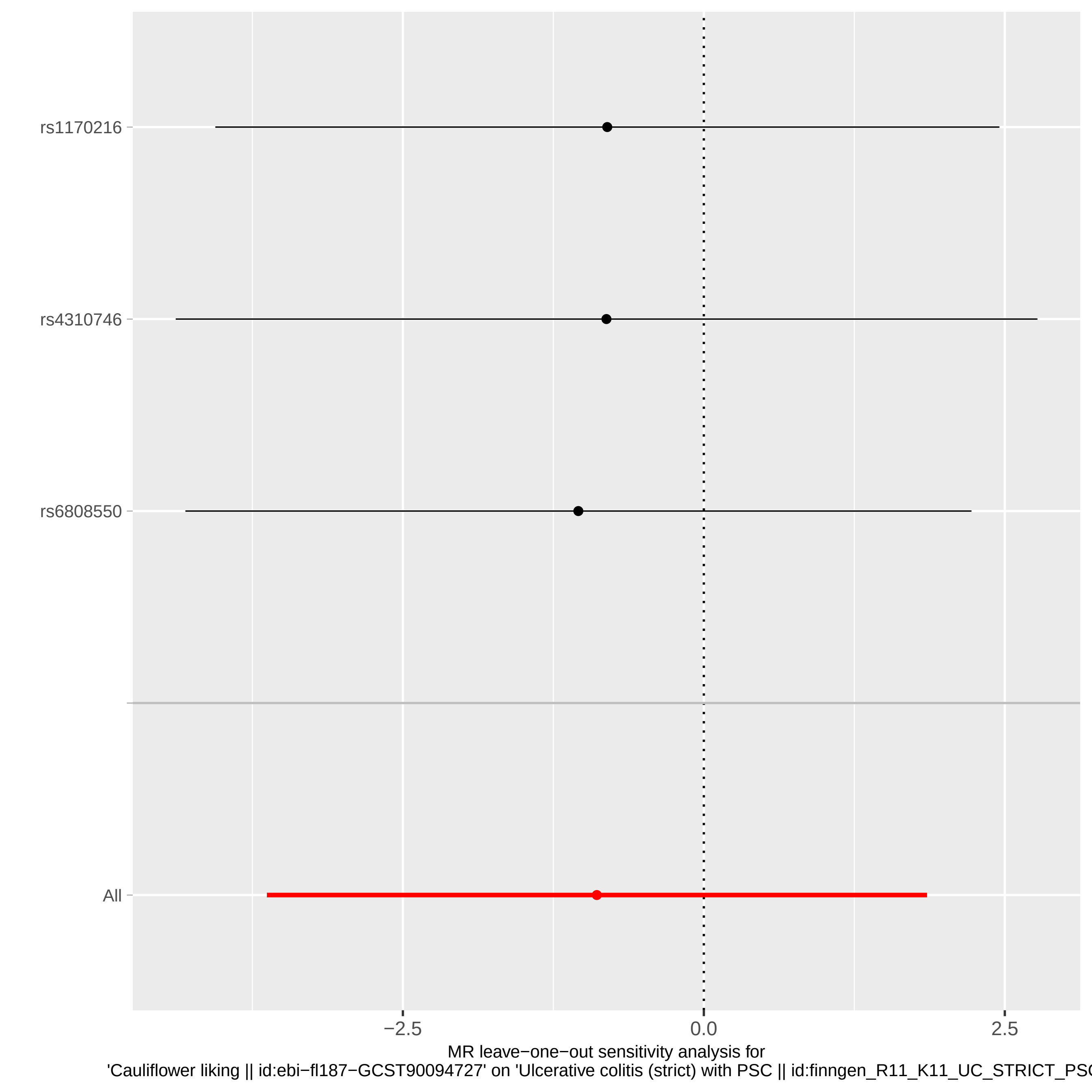
rs1170216
rs4310746
rs6808550
All
−2.5
0.0
2.5
MR leave−one−out sensitivity analysis for
'Cauliflower liking || id:ebi−fl187−GCST90094727' on 'Ulcerative colitis (strict) with PSC || id:finngen_R11_K11_UC_STRICT_PS

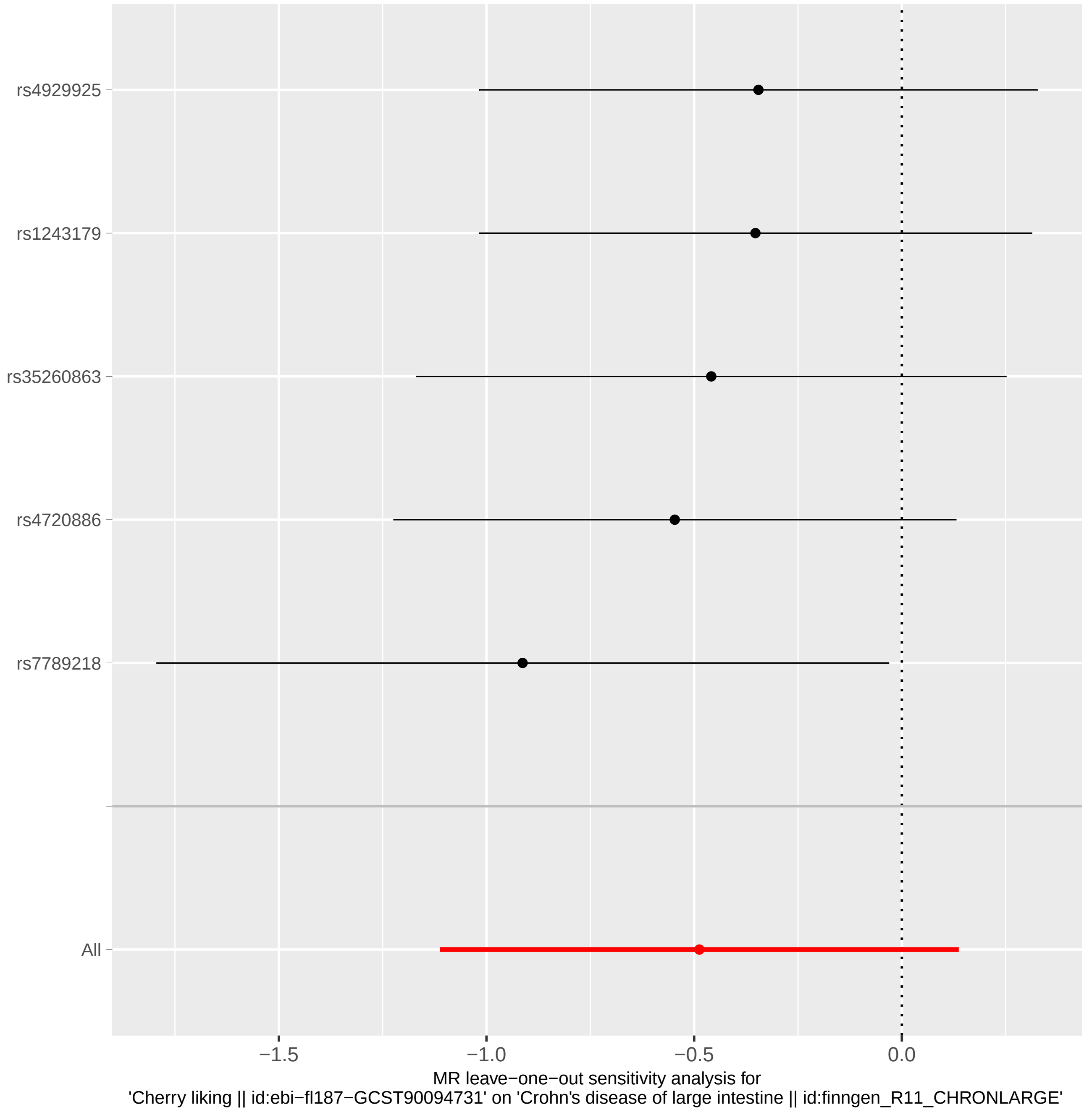
rs4929925
rs1243179
rs35260863
rs4720886
rs7789218
All
−1.5
−1.0
−0.5
0.0
MR leave−one−out sensitivity analysis for
'Cherry liking || id:ebi−fl187−GCST90094731' on 'Crohn's disease of large intestine || id:finngen_R11_CHRONLARGE'

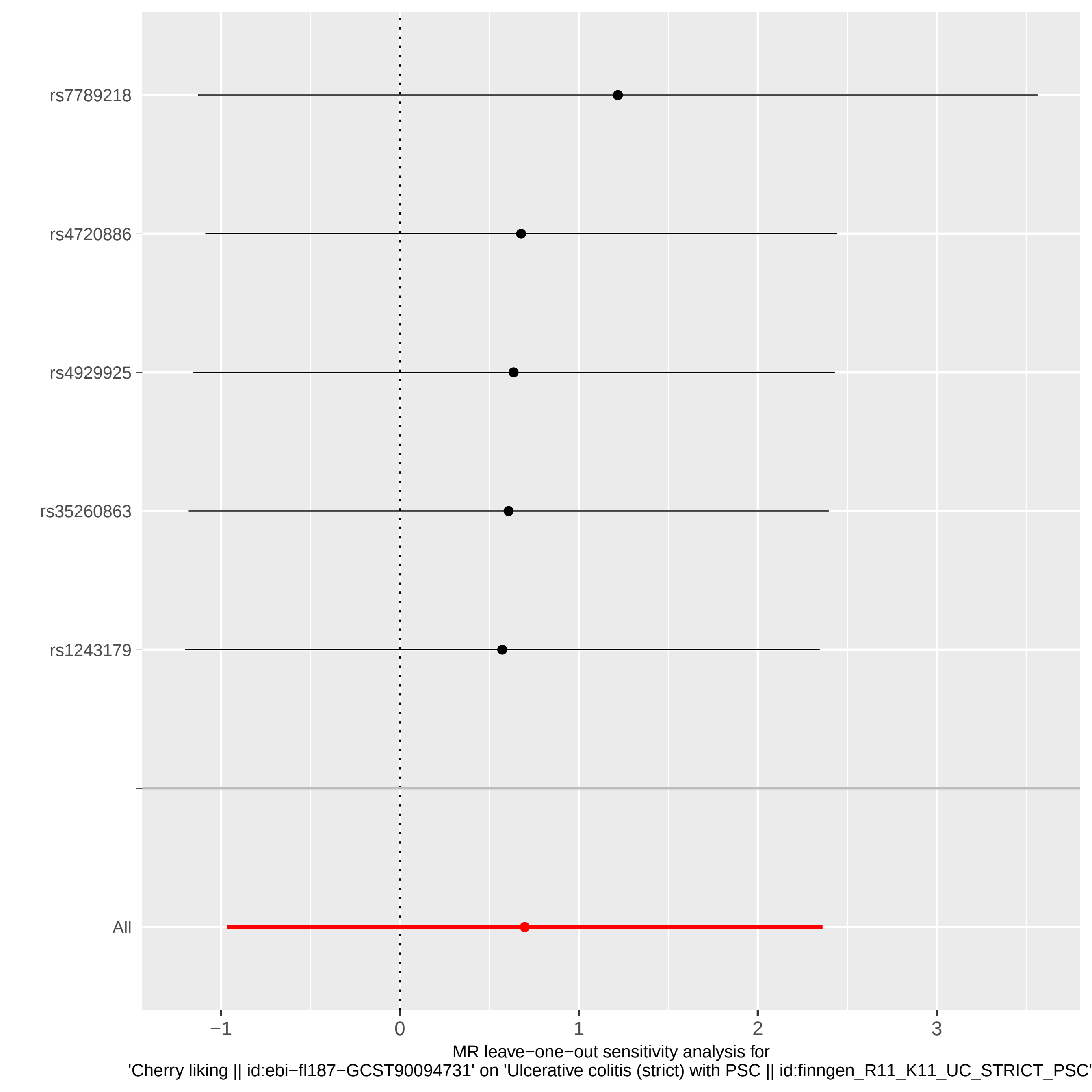

rs7789218
rs4720886
rs4929925
rs35260863
rs1243179
All
−1
0
1
2
3
MR leave−one−out sensitivity analysis for
'Cherry liking || id:ebi−fl187−GCST90094731' on 'Ulcerative colitis (strict) with PSC || id:finngen_R11_K11_UC_STRICT_PSC'

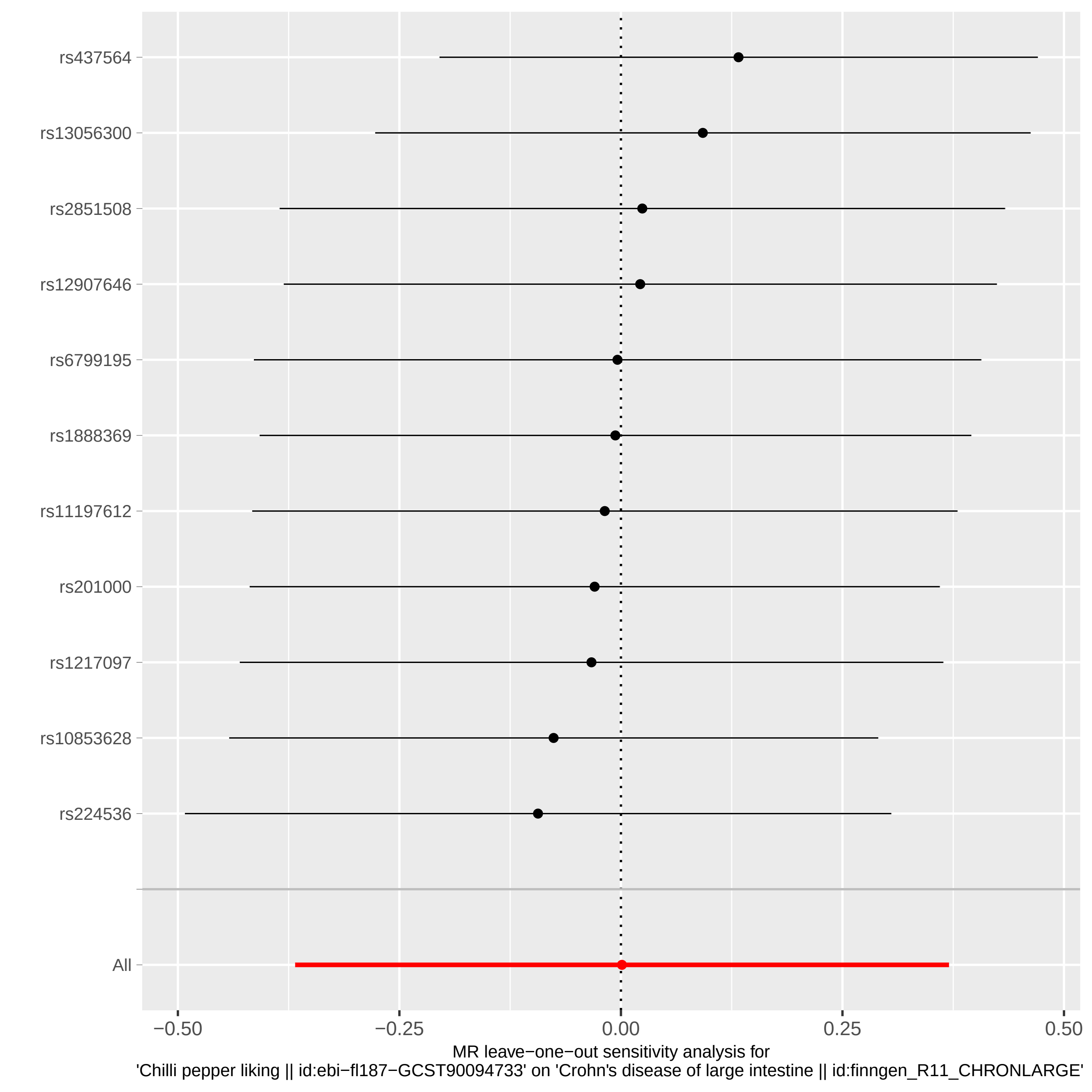
rs437564
rs13056300
rs2851508
rs12907646
rs6799195
rs1888369
rs11197612
rs201000
rs1217097
rs10853628
rs224536
All
−0.50
−0.25
0.00
0.25
0.50
MR leave−one−out sensitivity analysis for
'Chilli pepper liking || id:ebi−fl187−GCST90094733' on 'Crohn's disease of large intestine || id:finngen_R11_CHRONLARGE'

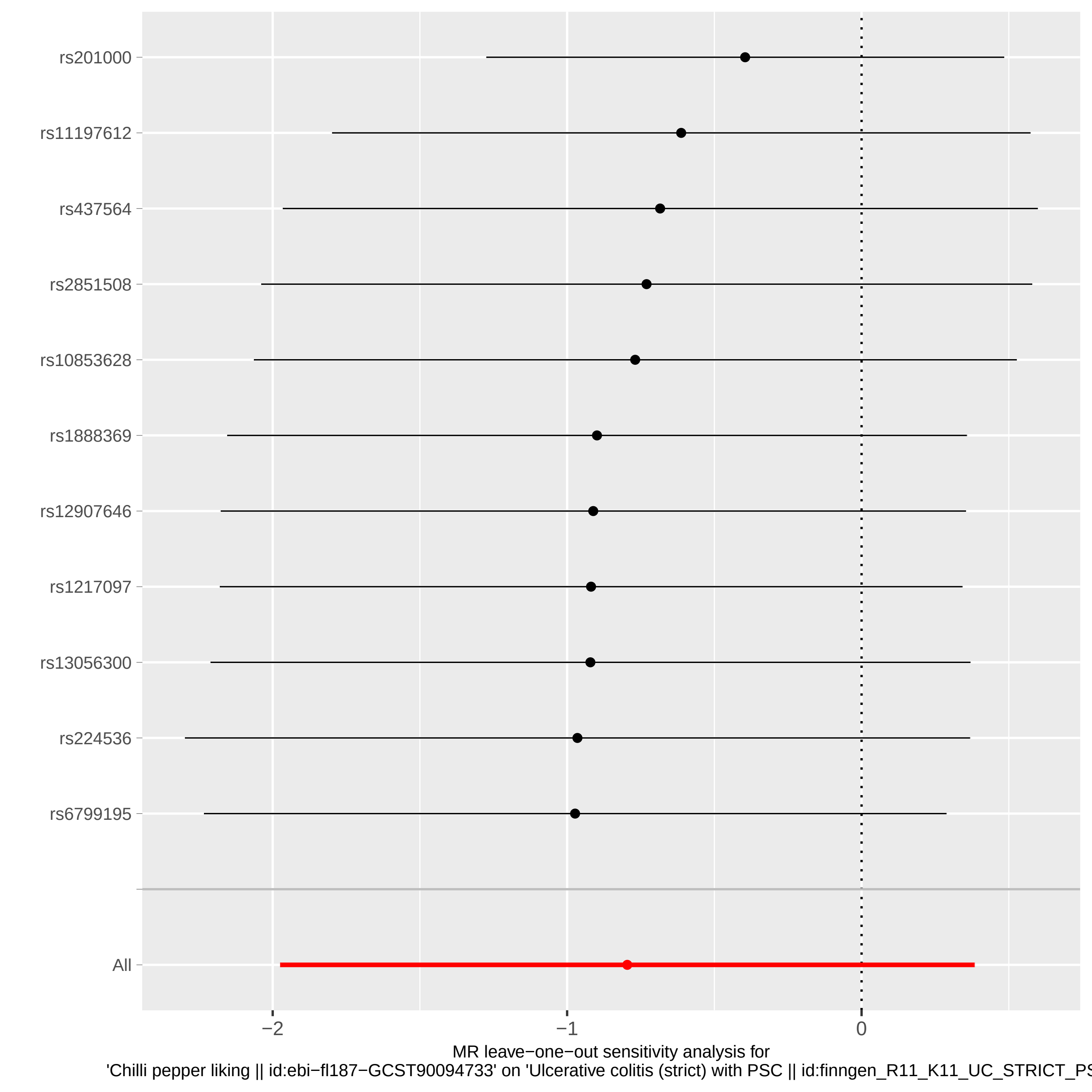
rs201000
rs11197612
rs437564
rs2851508
rs10853628
rs1888369
rs12907646
rs1217097
rs13056300
rs224536
rs6799195
All
−2
−1
0
MR leave−one−out sensitivity analysis for
'Chilli pepper liking || id:ebi−fl187−GCST90094733' on 'Ulcerative colitis (strict) with PSC || id:finngen_R11_K11_UC_STRICT_PS

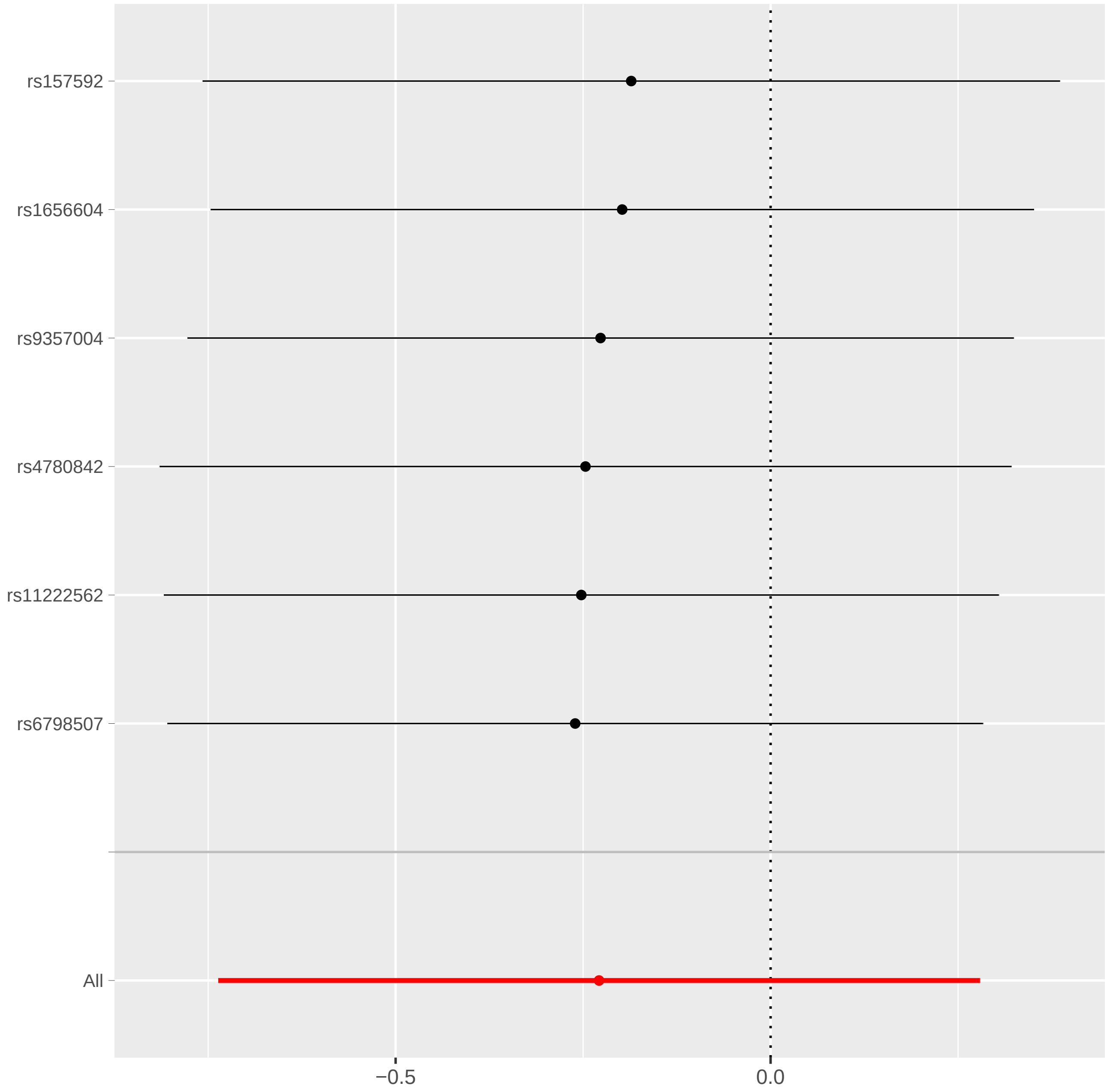

MR leave−one−out sensitivity analysis for
'Corn flake liking || id:ebi−fl187−GCST90094745' on 'Crohn's disease of large intestine || id:finngen_R11_CHRONLARGE'

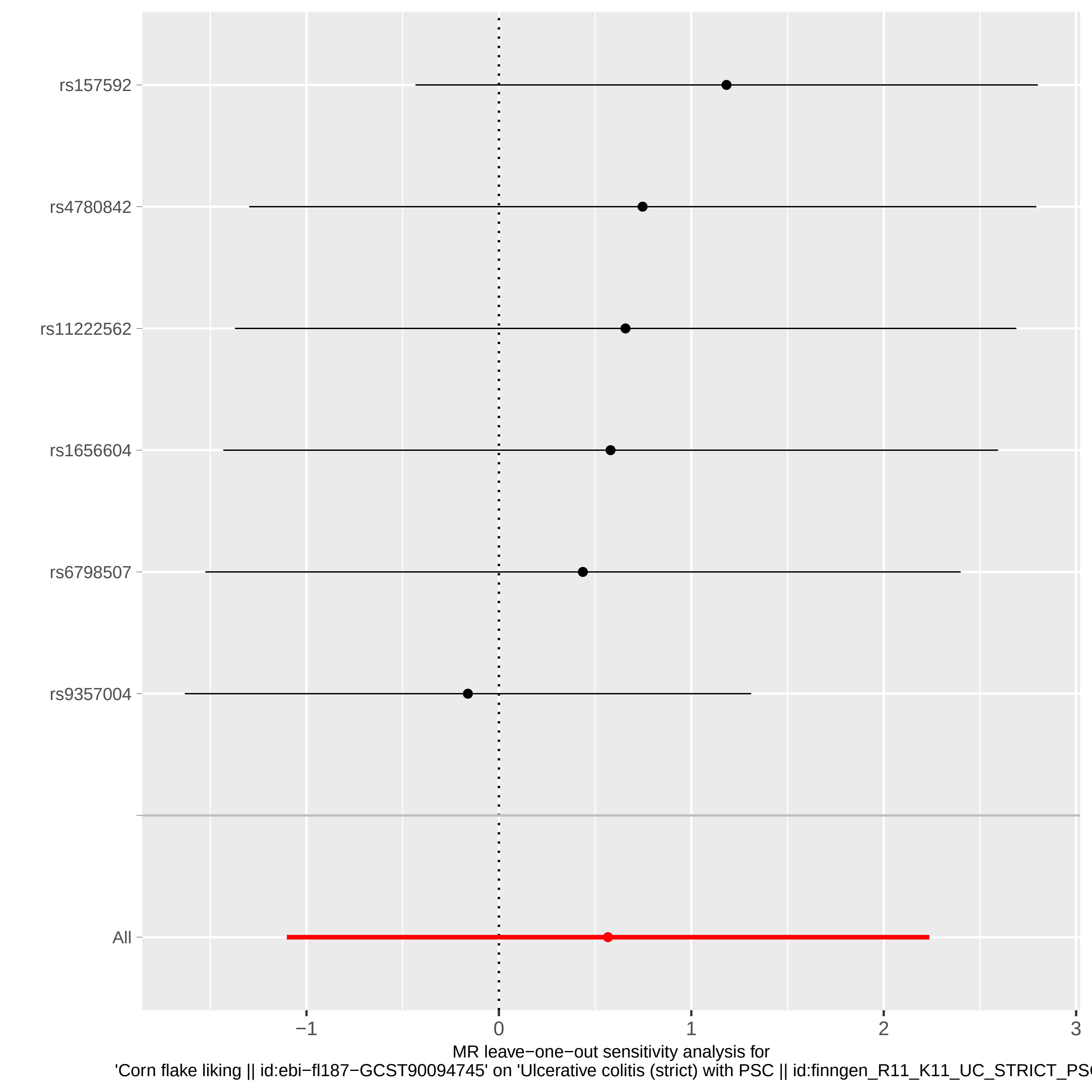
rs157592
rs4780842
rs11222562
rs1656604
rs6798507
rs9357004
All
−1
0
1
2
3
MR leave−one−out sensitivity analysis for
'Corn flake liking || id:ebi−fl187−GCST90094745' on 'Ulcerative colitis (strict) with PSC || id:finngen_R11_K11_UC_STRICT_PS

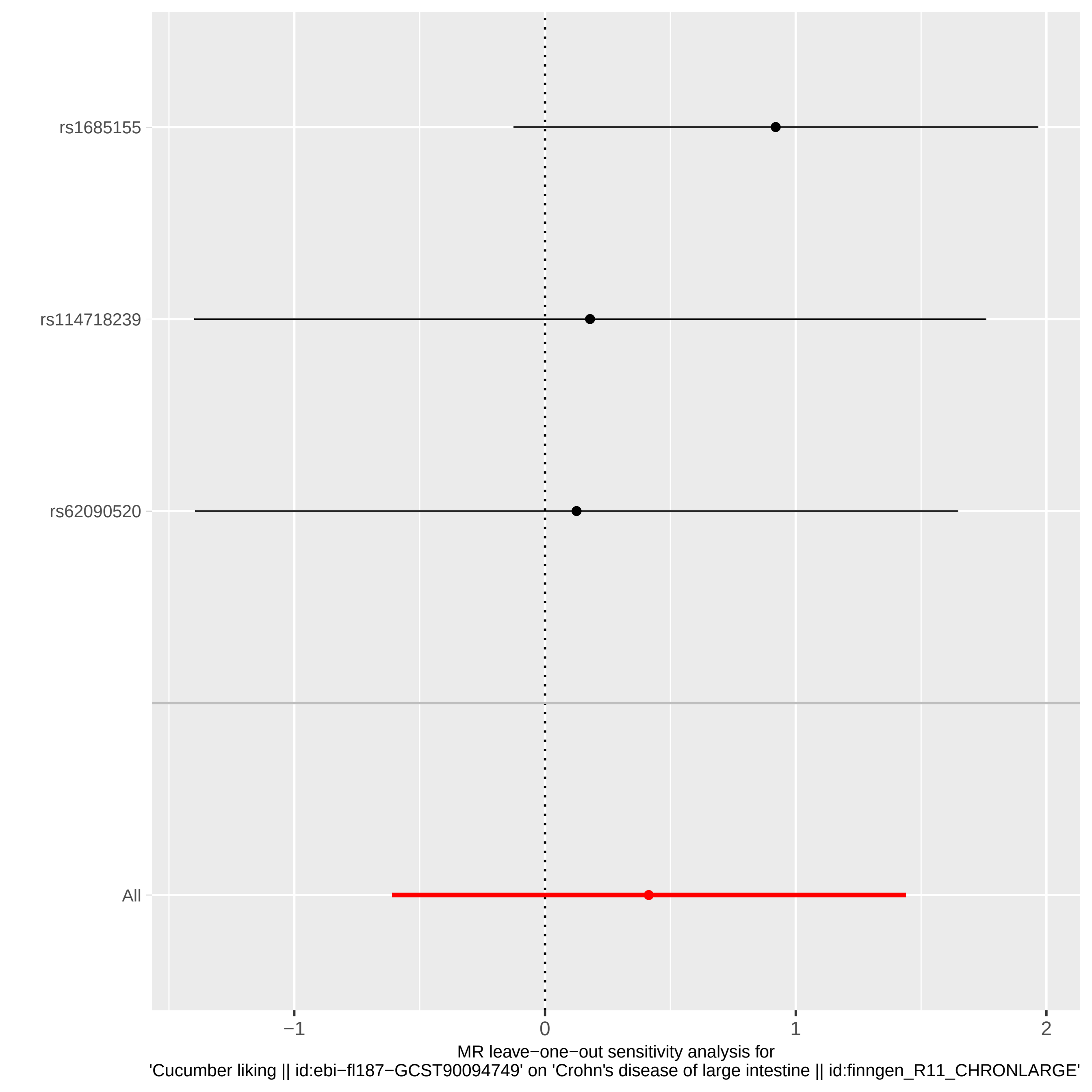
rs1685155
rs114718239
rs62090520
All
−1
0
1
2
MR leave−one−out sensitivity analysis for
'Cucumber liking || id:ebi−fl187−GCST90094749' on 'Crohn's disease of large intestine || id:finngen_R11_CHRONLARGE'

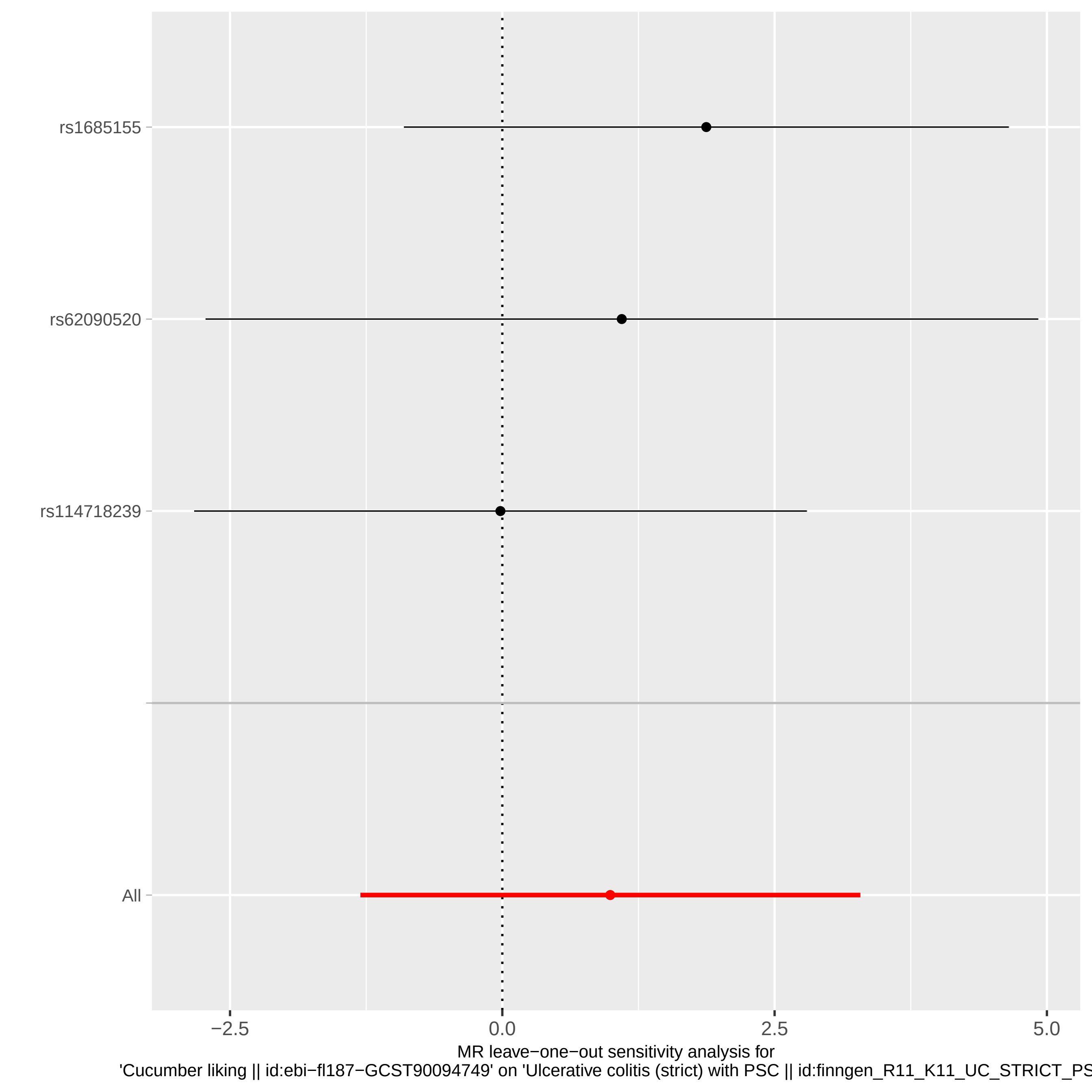
rs1685155
rs62090520
rs114718239
All
−2.5
0.0
2.5
5.0
MR leave−one−out sensitivity analysis for
'Cucumber liking || id:ebi−fl187−GCST90094749' on 'Ulcerative colitis (strict) with PSC || id:finngen_R11_K11_UC_STRICT_PS

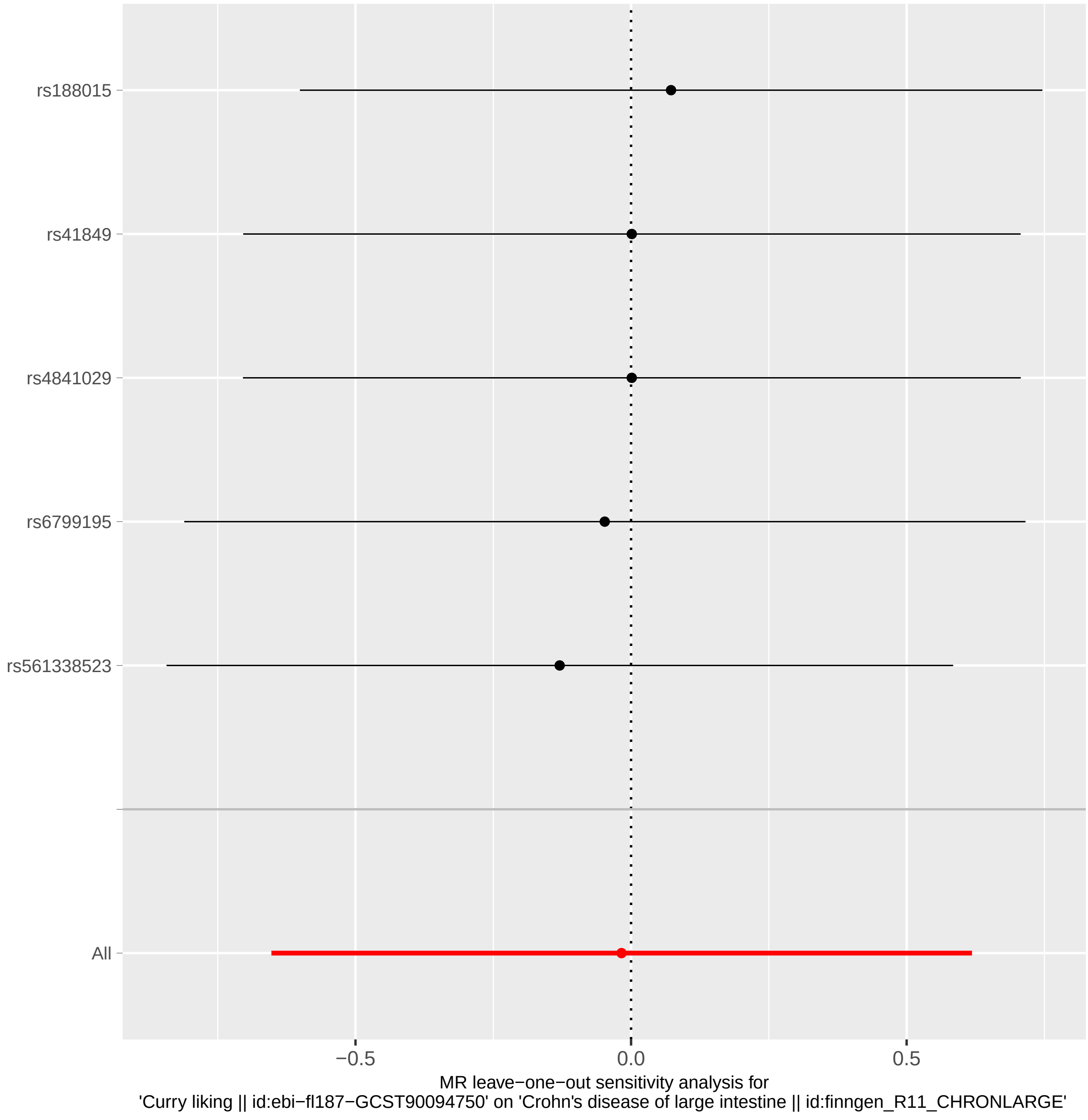
rs188015
rs41849
rs4841029
rs6799195
rs561338523
All
−0.5
0.0
0.5
MR leave−one−out sensitivity analysis for
'Curry liking || id:ebi−fl187−GCST90094750' on 'Crohn's disease of large intestine || id:finngen_R11_CHRONLARGE'

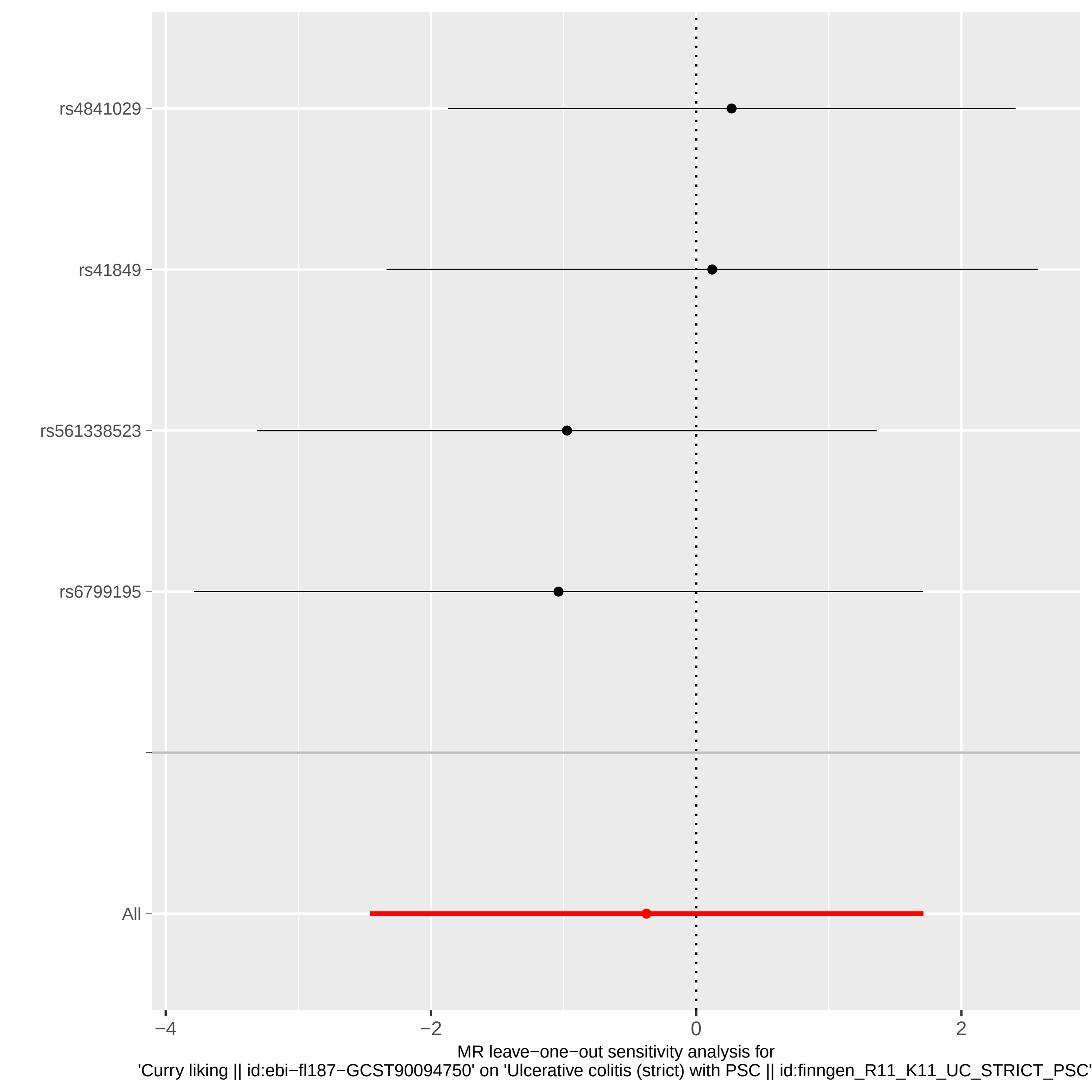
rs4841029
rs41849
rs561338523
rs6799195
All
−4
−2
0
2
MR leave−one−out sensitivity analysis for
'Curry liking || id:ebi−fl187−GCST90094750' on 'Ulcerative colitis (strict) with PSC || id:finngen_R11_K11_UC_STRICT_PSC'

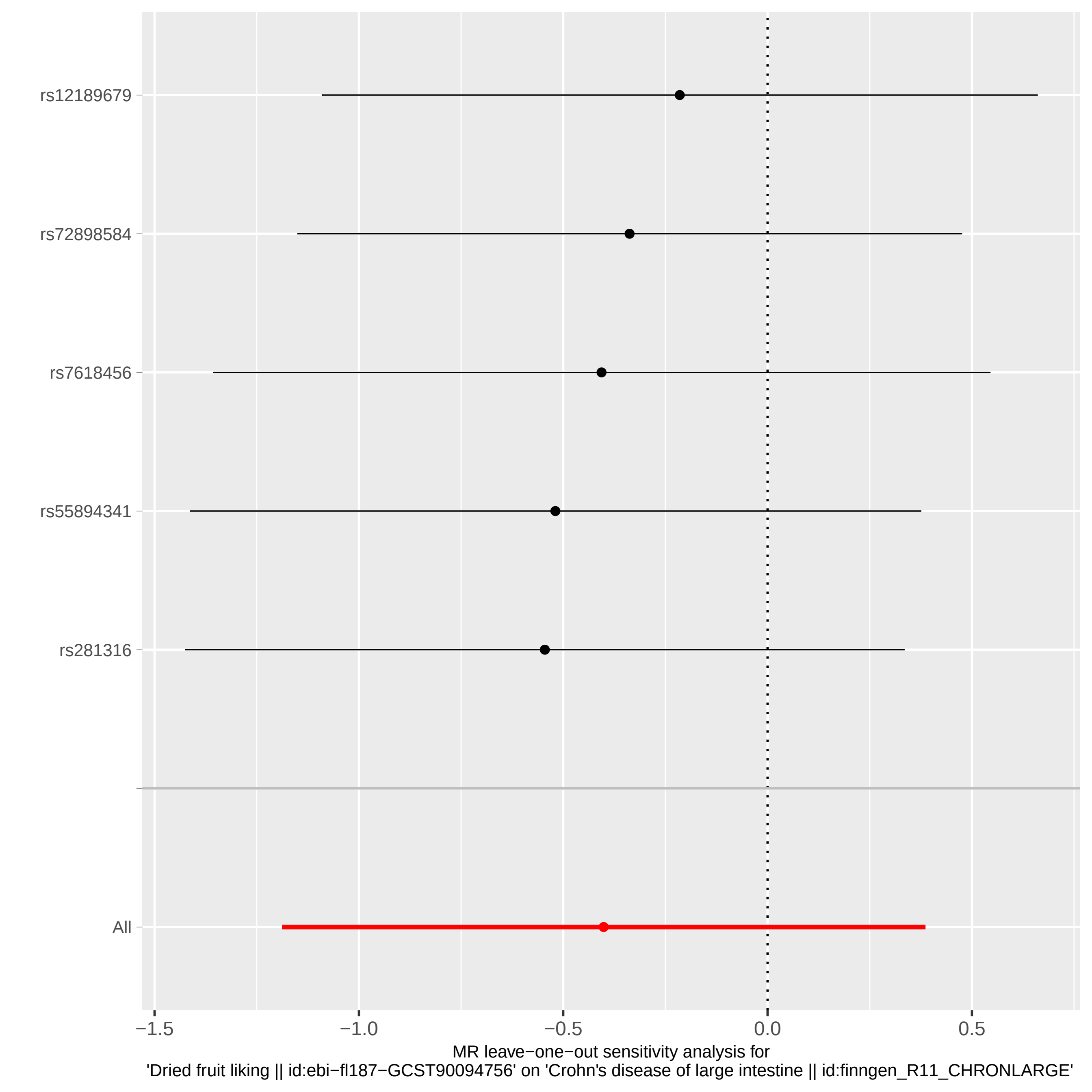
rs12189679
rs72898584
rs7618456
rs55894341
rs281316
All
−1.5
−1.0
−0.5
0.0
0.5
MR leave−one−out sensitivity analysis for
'Dried fruit liking || id:ebi−fl187−GCST90094756' on 'Crohn's disease of large intestine || id:finngen_R11_CHRONLARGE'

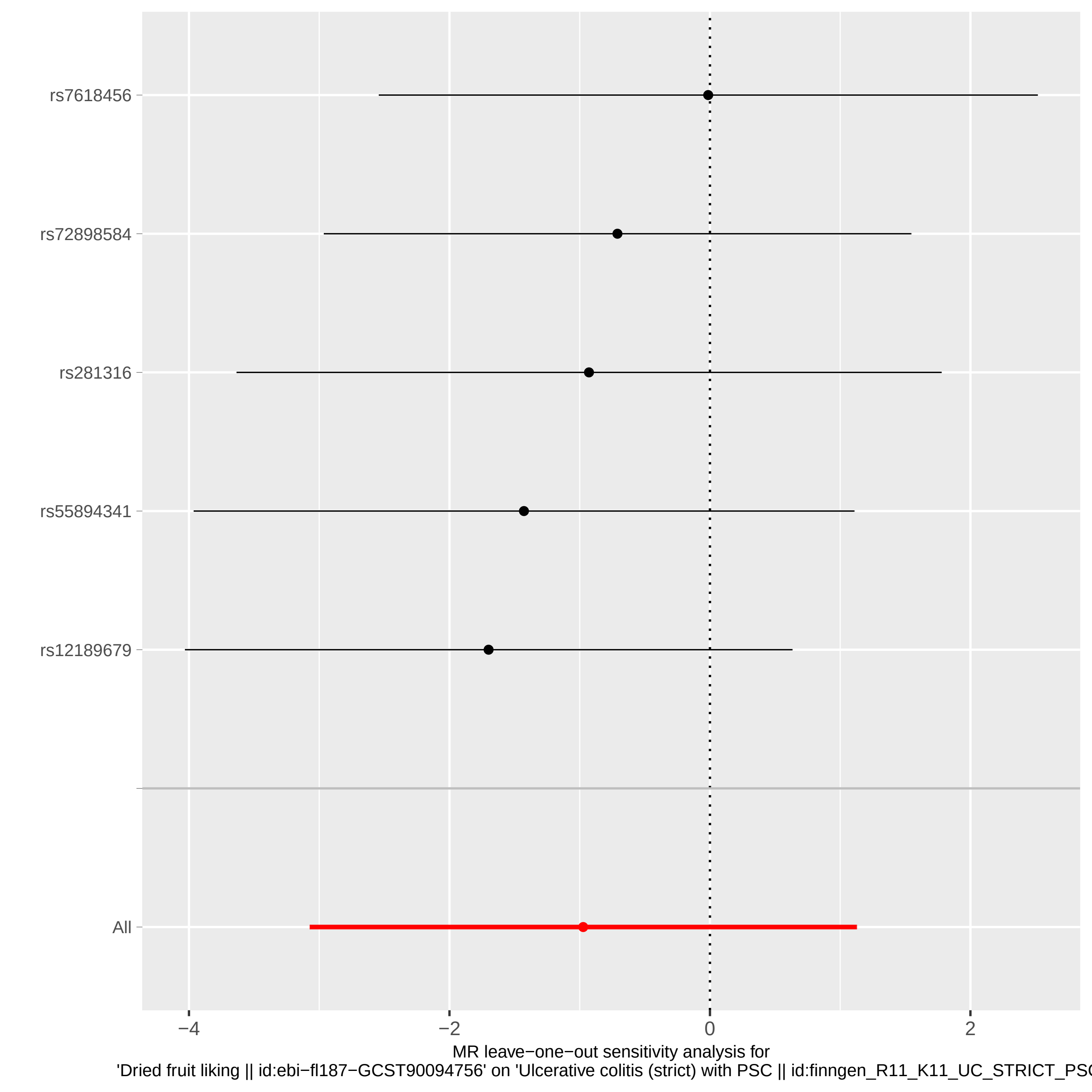
rs7618456
rs72898584
rs281316
rs55894341
rs12189679
All
−4
−2
0
2
MR leave−one−out sensitivity analysis for
'Dried fruit liking || id:ebi−fl187−GCST90094756' on 'Ulcerative colitis (strict) with PSC || id:finngen_R11_K11_UC_STRICT_PS

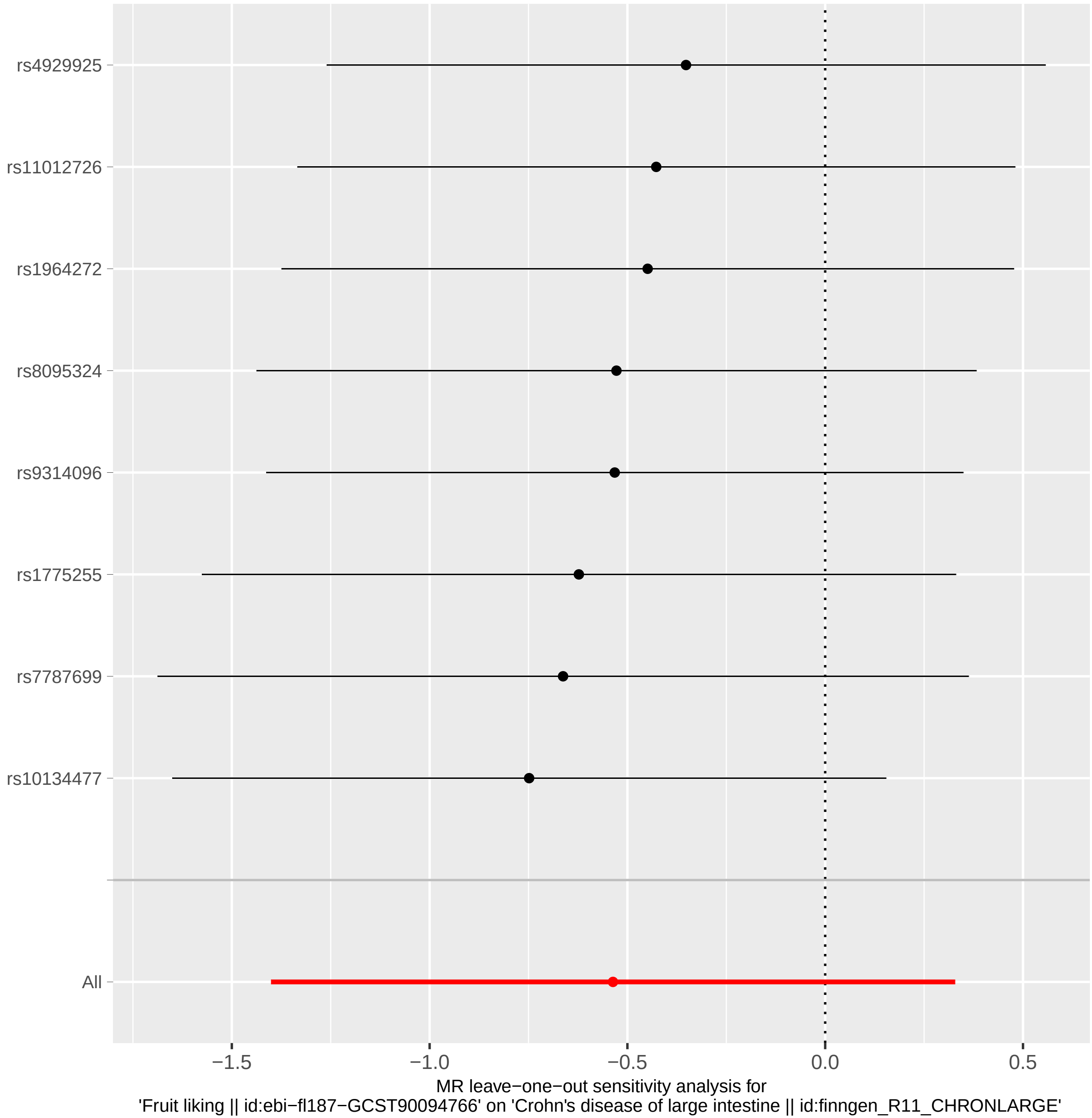
rs4929925
rs11012726
rs1964272
rs8095324
rs9314096
rs1775255
rs7787699
rs10134477
All
−1.5
−1.0
−0.5
0.0
0.5
MR leave−one−out sensitivity analysis for
'Fruit liking || id:ebi−fl187−GCST90094766' on 'Crohn's disease of large intestine || id:finngen_R11_CHRONLARGE'

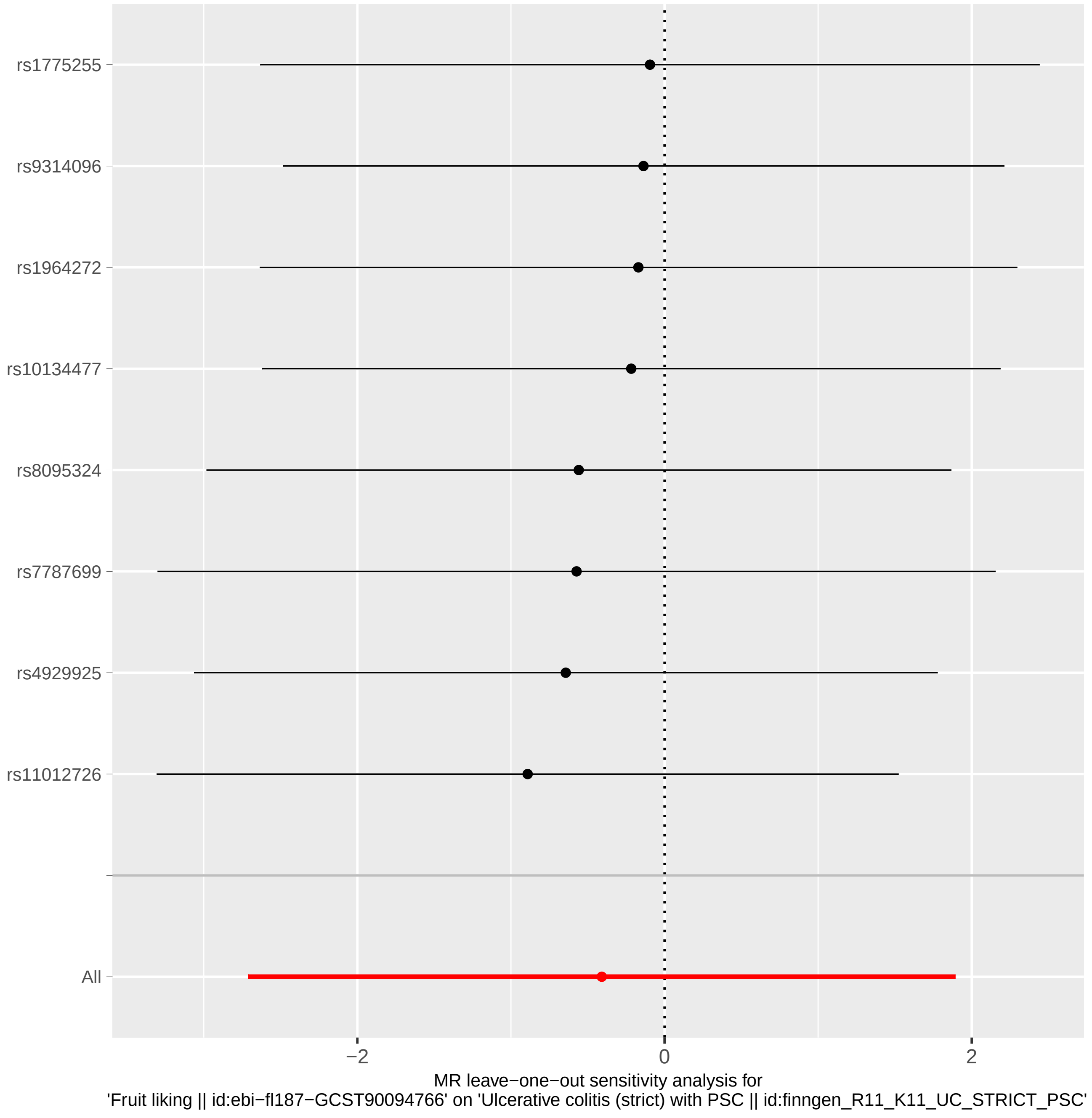
rs1775255
rs9314096
rs1964272
rs10134477
rs8095324
rs7787699
rs4929925
rs11012726
All
−2
0
2
MR leave−one−out sensitivity analysis for
'Fruit liking || id:ebi−fl187−GCST90094766' on 'Ulcerative colitis (strict) with PSC || id:finngen_R11_K11_UC_STRICT_PSC'

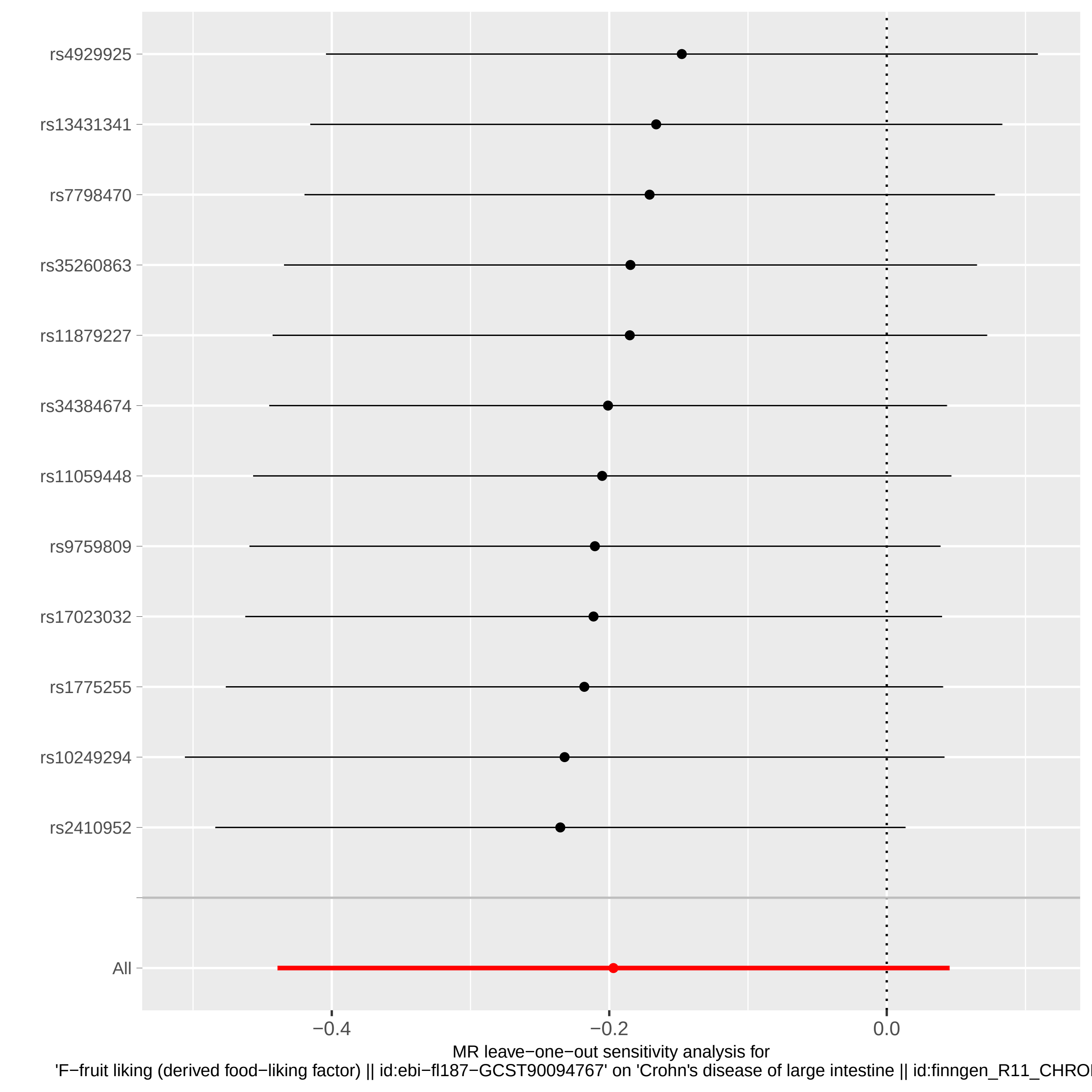

rs4929925
rs13431341
rs7798470
rs35260863
rs11879227
rs34384674
rs11059448
rs9759809
rs17023032
rs1775255
rs10249294
rs2410952
All
−0.4
−0.2
0.0
MR leave−one−out sensitivity analysis for
'F−fruit liking (derived food−liking factor) || id:ebi−fl187−GCST90094767' on 'Crohn's disease of large intestine || id:finngen_R11_CHRO

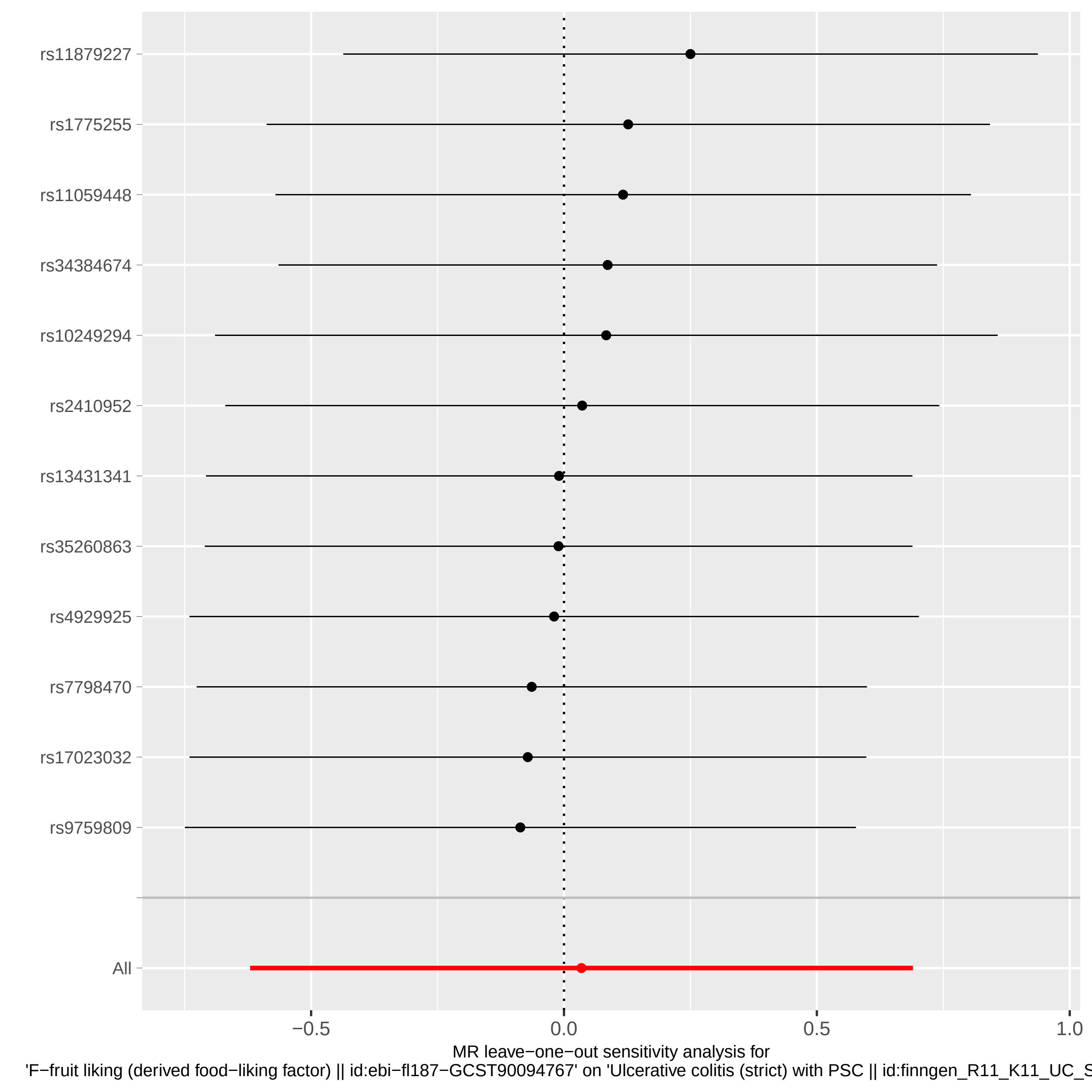
rs11879227
rs1775255
rs11059448
rs34384674
rs10249294
rs2410952
rs13431341
rs35260863
rs4929925
rs7798470
rs17023032
rs9759809
All
−0.5
0.0
0.5
1.0
MR leave−one−out sensitivity analysis for
'F−fruit liking (derived food−liking factor) || id:ebi−fl187−GCST90094767' on 'Ulcerative colitis (strict) with PSC || id:finngen_R11_K11_UC_S

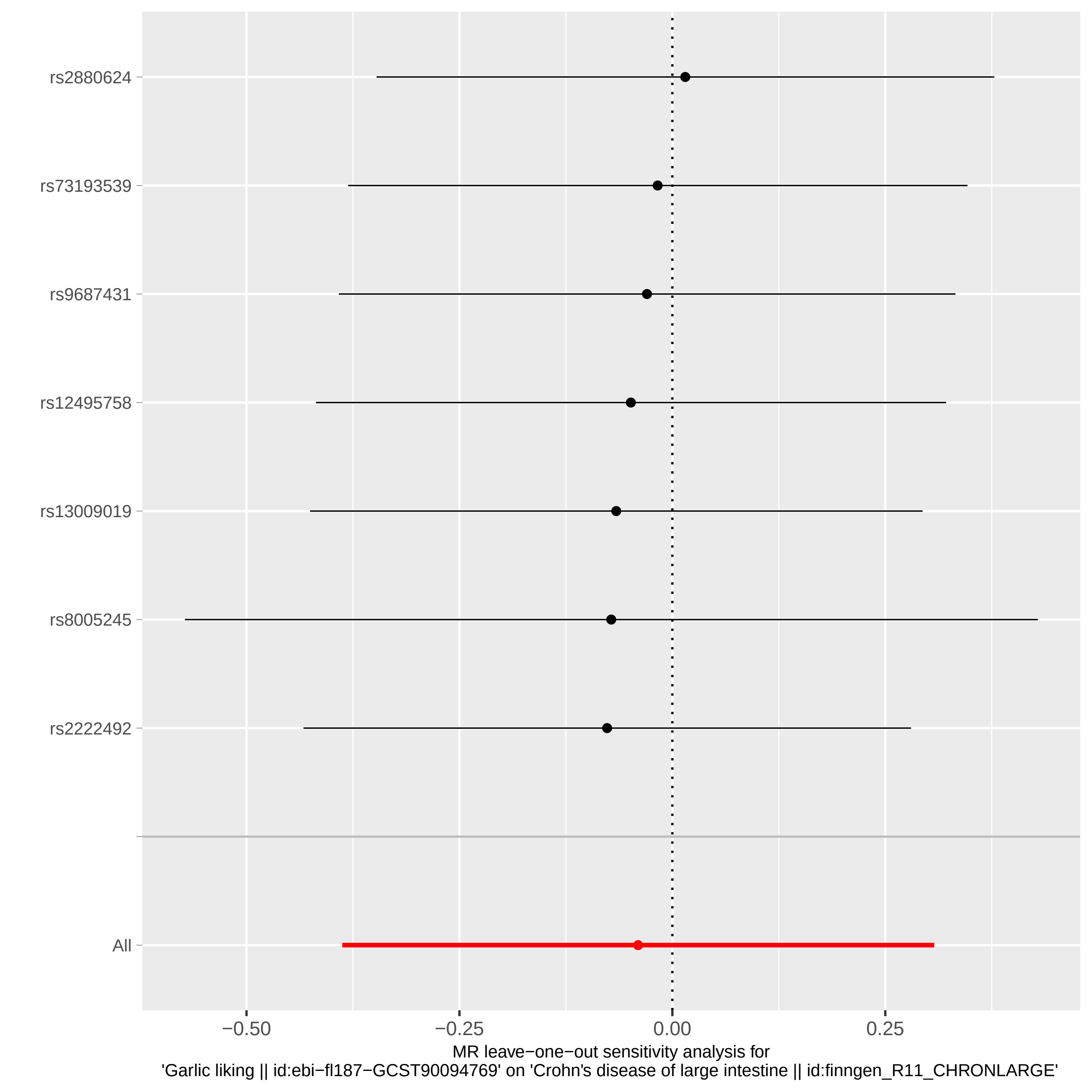
rs2880624
rs73193539
rs9687431
rs12495758
rs13009019
rs8005245
rs2222492
All
−0.50
−0.25
0.00
0.25
MR leave−one−out sensitivity analysis for
'Garlic liking || id:ebi−fl187−GCST90094769' on 'Crohn's disease of large intestine || id:finngen_R11_CHRONLARGE'

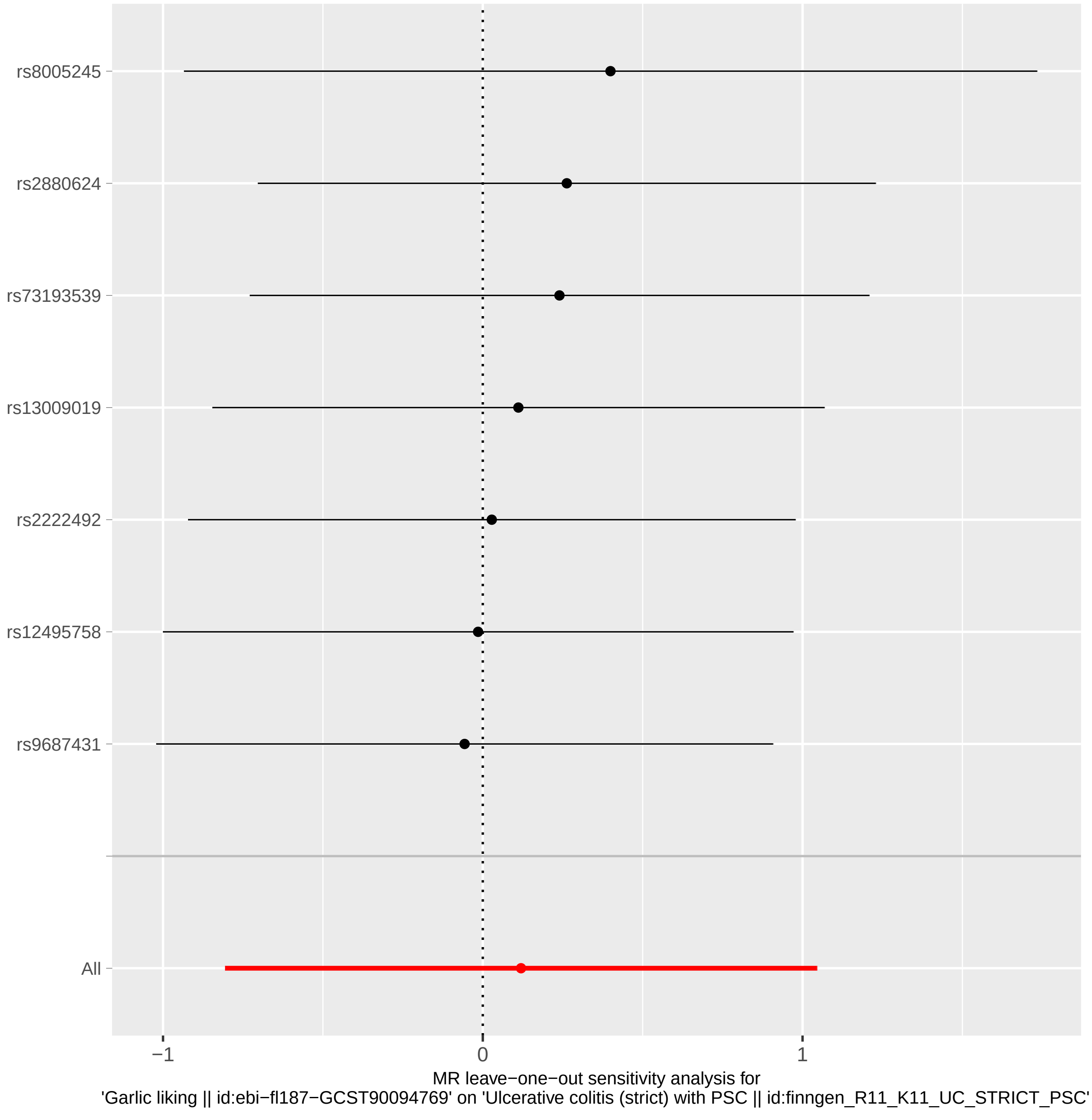
rs8005245
rs2880624
rs73193539
rs13009019
rs2222492
rs12495758
rs9687431
All
−1
0
1
MR leave−one−out sensitivity analysis for
'Garlic liking || id:ebi−fl187−GCST90094769' on 'Ulcerative colitis (strict) with PSC || id:finngen_R11_K11_UC_STRICT_PSC'

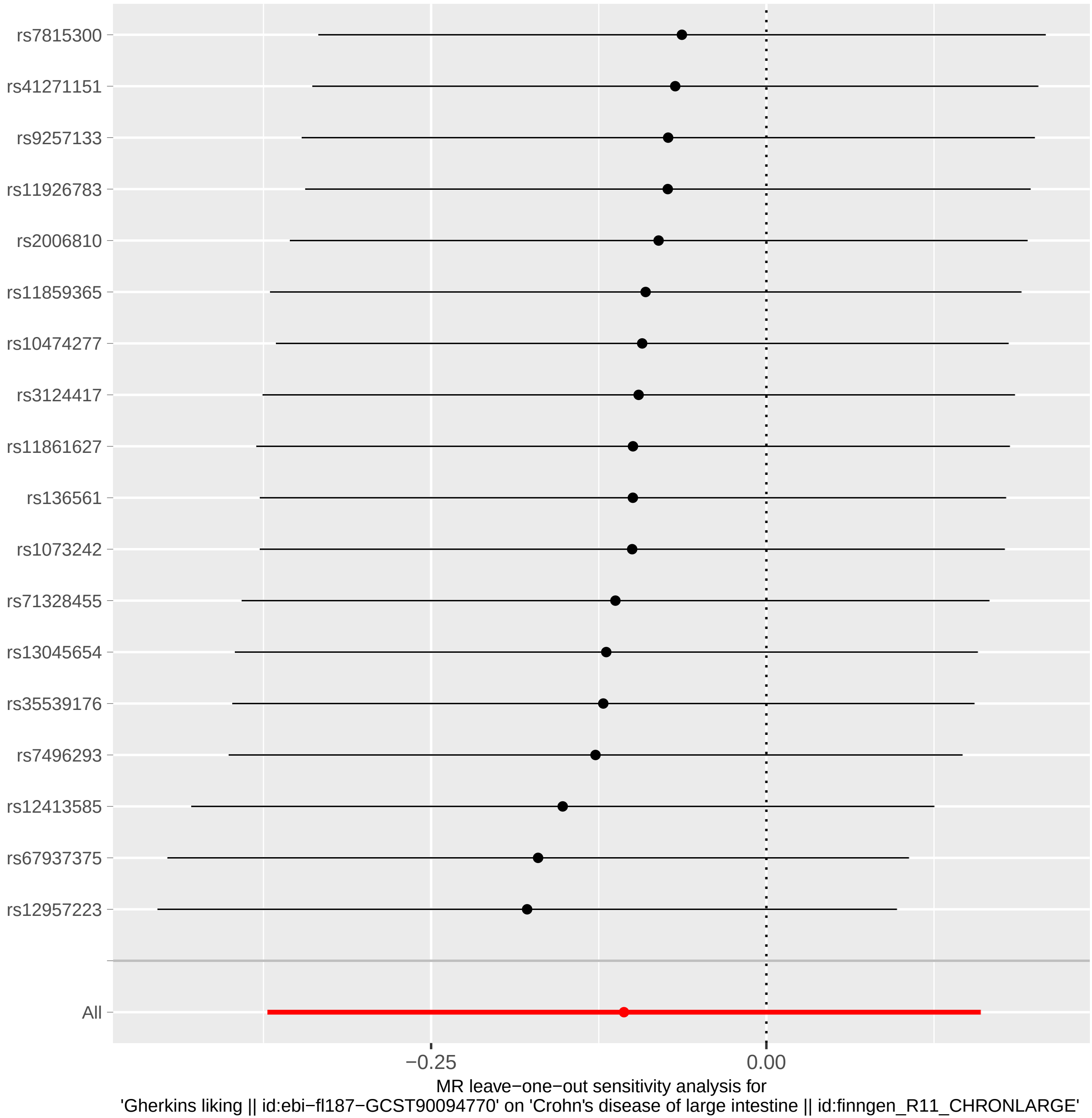
rs7815300
rs41271151
rs9257133
rs11926783
rs2006810
rs11859365
rs10474277
rs3124417
rs11861627
rs136561
rs1073242
rs71328455
rs13045654
rs35539176
rs7496293
rs12413585
rs67937375
rs12957223
All
−0.25
0.00
MR leave−one−out sensitivity analysis for
'Gherkins liking || id:ebi−fl187−GCST90094770' on 'Crohn's disease of large intestine || id:finngen_R11_CHRONLARGE'

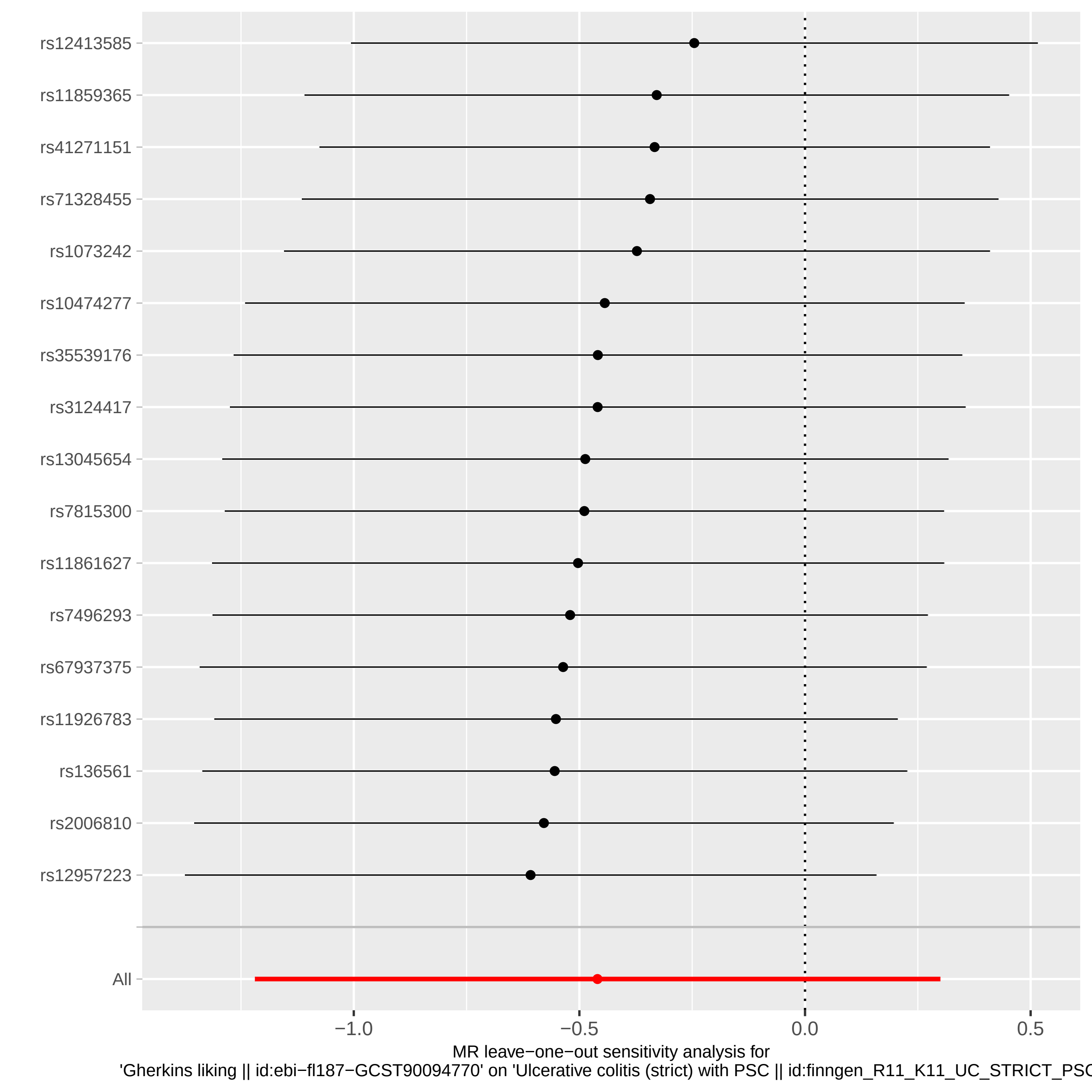
rs12413585
rs11859365
rs41271151
rs71328455
rs1073242
rs10474277
rs35539176
rs3124417
rs13045654
rs7815300
rs11861627
rs7496293
rs67937375
rs11926783
rs136561
rs2006810
rs12957223
All
−1.0
−0.5
0.0
0.5
MR leave−one−out sensitivity analysis for
'Gherkins liking || id:ebi−fl187−GCST90094770' on 'Ulcerative colitis (strict) with PSC || id:finngen_R11_K11_UC_STRICT_PSC

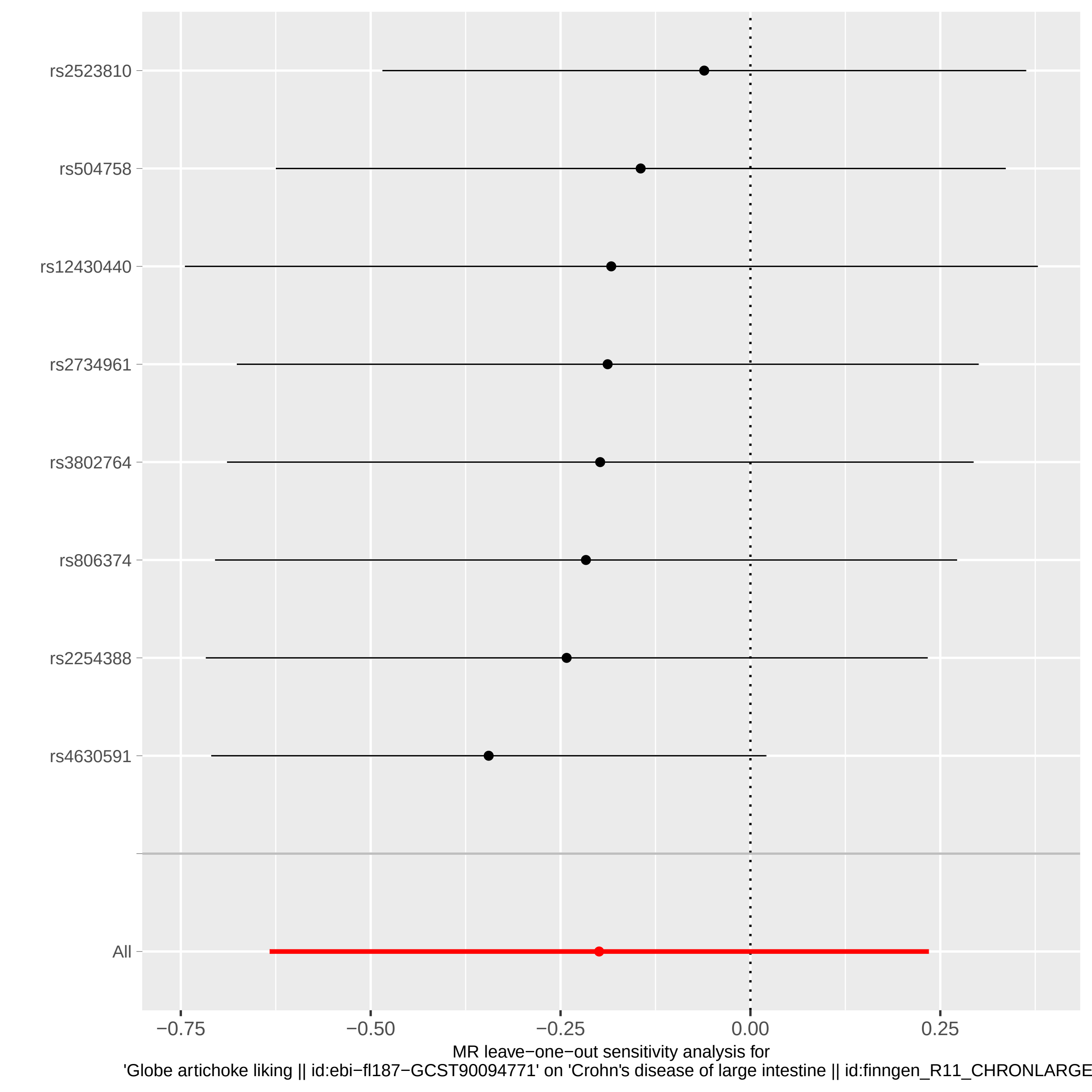
rs2523810
rs504758
rs12430440
rs2734961
rs3802764
rs806374
rs2254388
rs4630591
All
−0.75
−0.50
−0.25
0.00
0.25
MR leave−one−out sensitivity analysis for
'Globe artichoke liking || id:ebi−fl187−GCST90094771' on 'Crohn's disease of large intestine || id:finngen_R11_CHRONLARGE

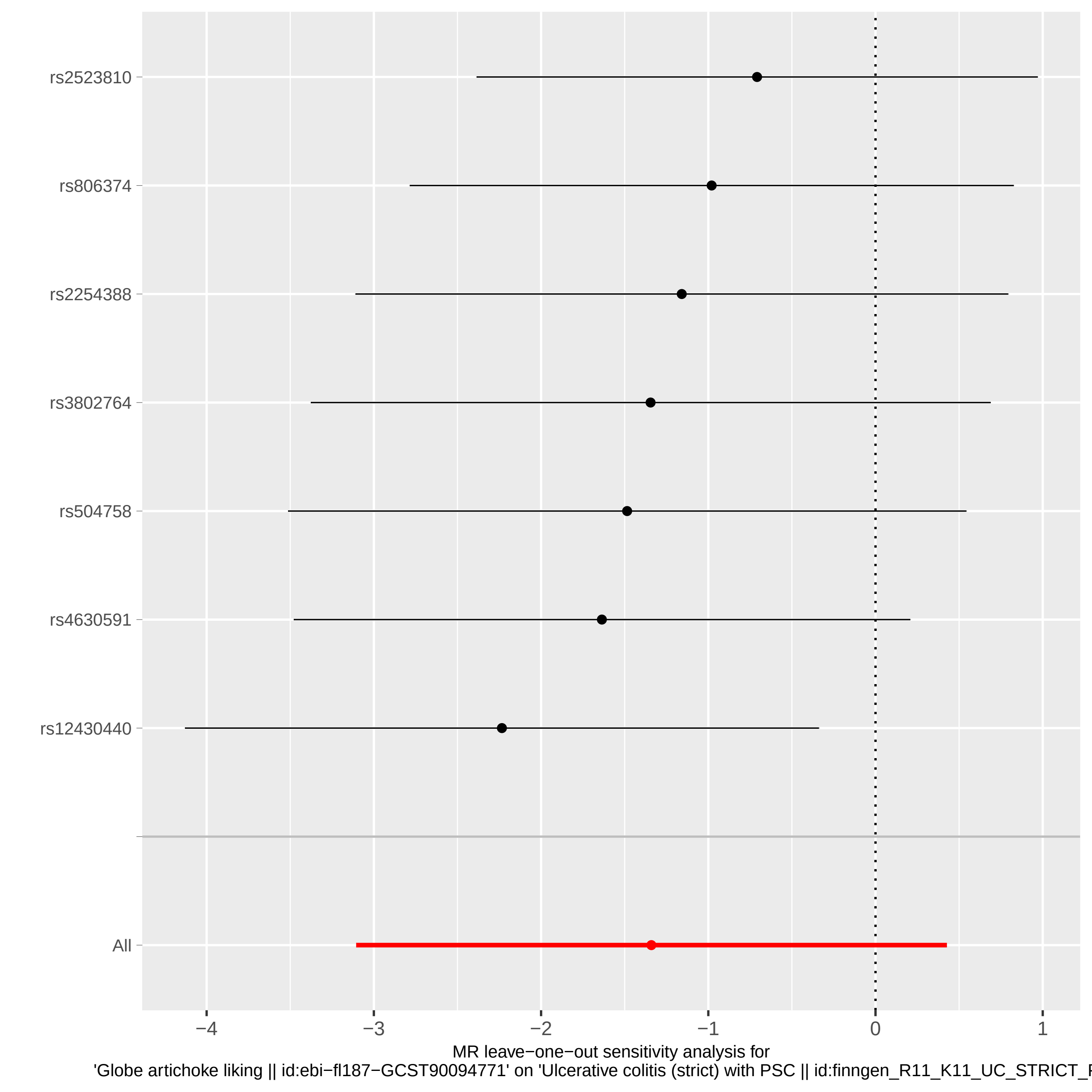
rs2523810
rs806374
rs2254388
rs3802764
rs504758
rs4630591
rs12430440
All
−4
−3
−2
−1
0
1
MR leave−one−out sensitivity analysis for
'Globe artichoke liking || id:ebi−fl187−GCST90094771' on 'Ulcerative colitis (strict) with PSC || id:finngen_R11_K11_UC_STRICT_

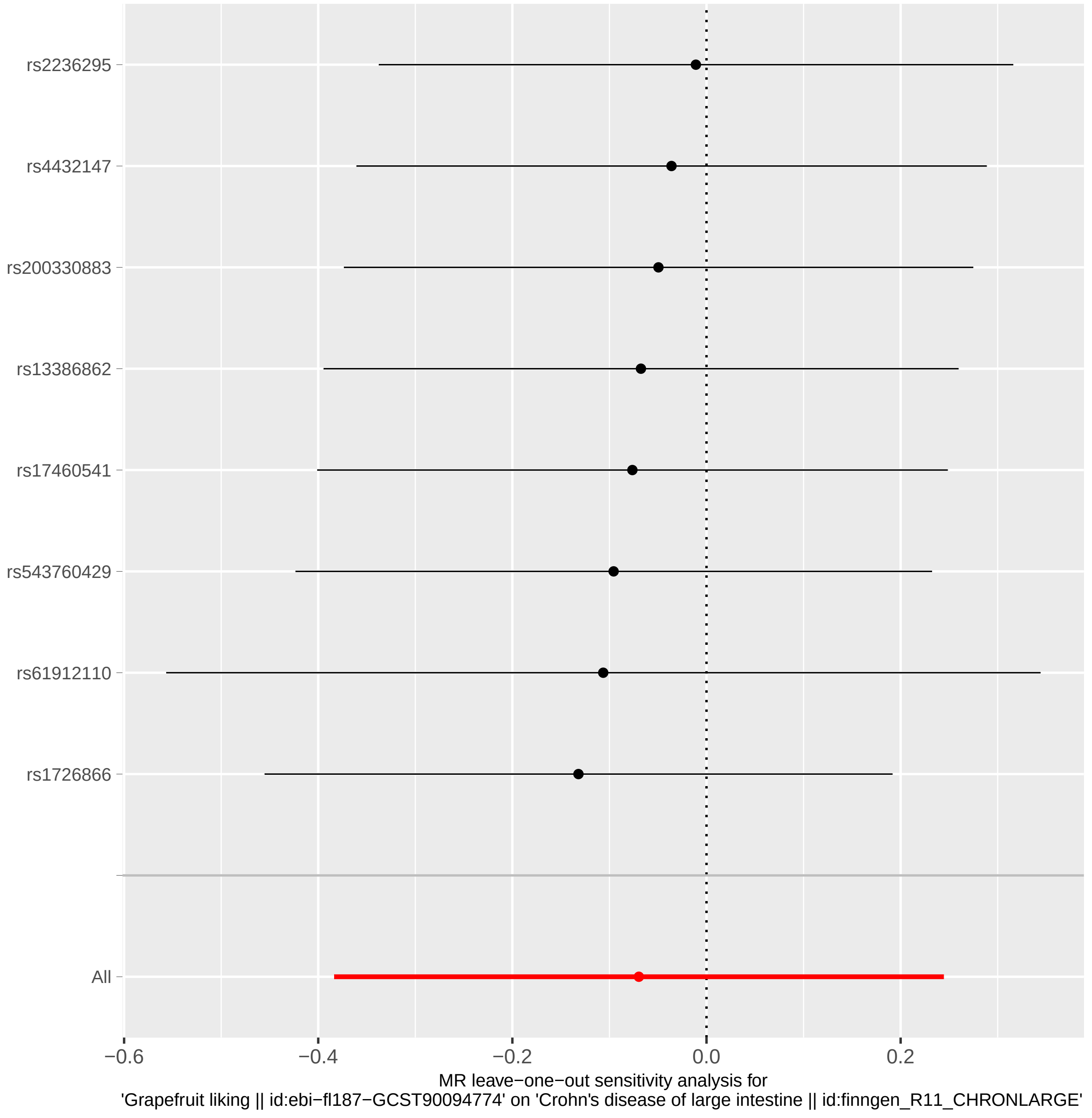
rs2236295
rs4432147
rs200330883
rs13386862
rs17460541
rs543760429
rs61912110
rs1726866
All
−0.6
−0.4
−0.2
0.0
0.2
MR leave−one−out sensitivity analysis for
'Grapefruit liking || id:ebi−fl187−GCST90094774' on 'Crohn's disease of large intestine || id:finngen_R11_CHRONLARGE'

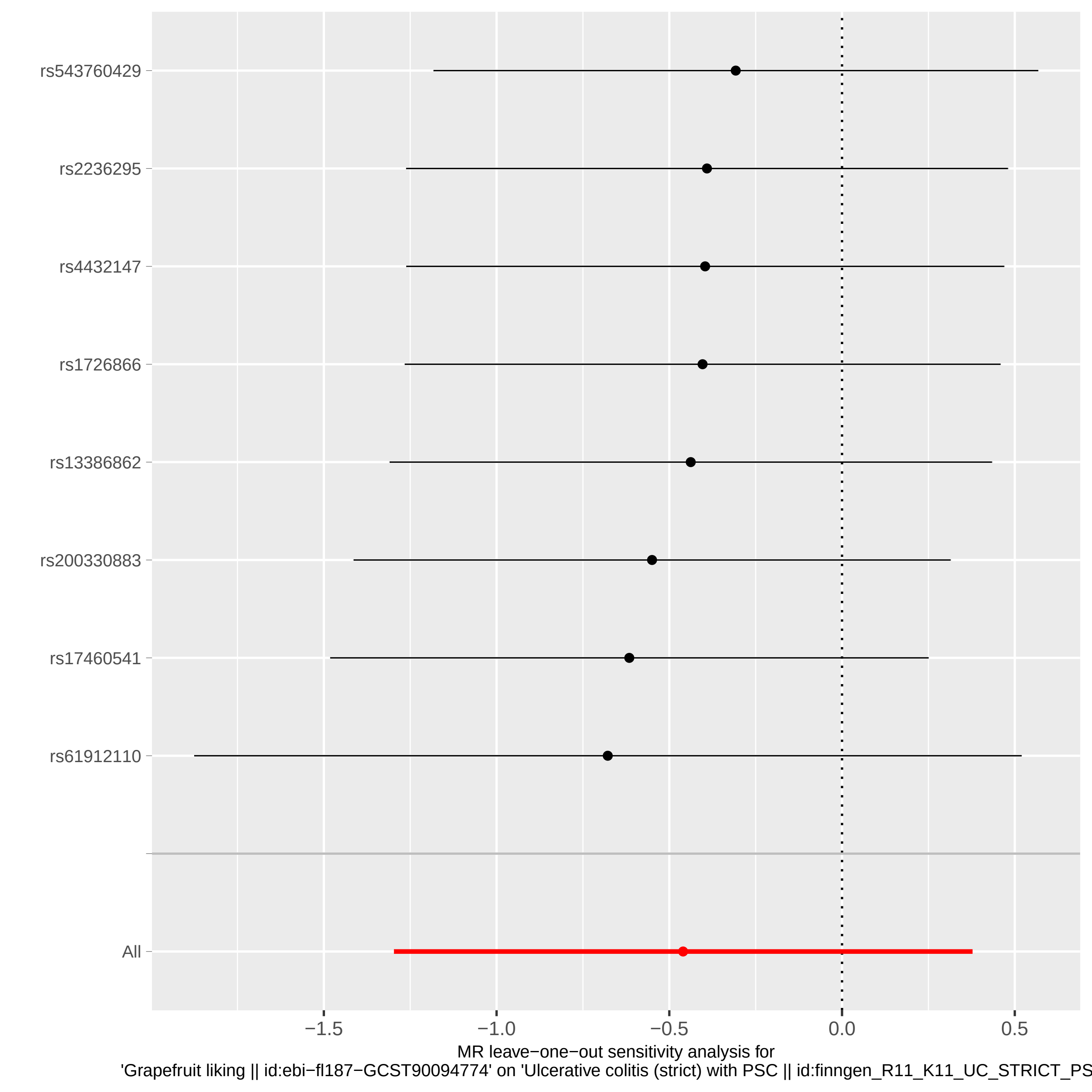
rs543760429
rs2236295
rs4432147
rs1726866
rs13386862
rs200330883
rs17460541
rs61912110
All
−1.5
−1.0
−0.5
0.0
0.5
MR leave−one−out sensitivity analysis for
'Grapefruit liking || id:ebi−fl187−GCST90094774' on 'Ulcerative colitis (strict) with PSC || id:finngen_R11_K11_UC_STRICT_PS

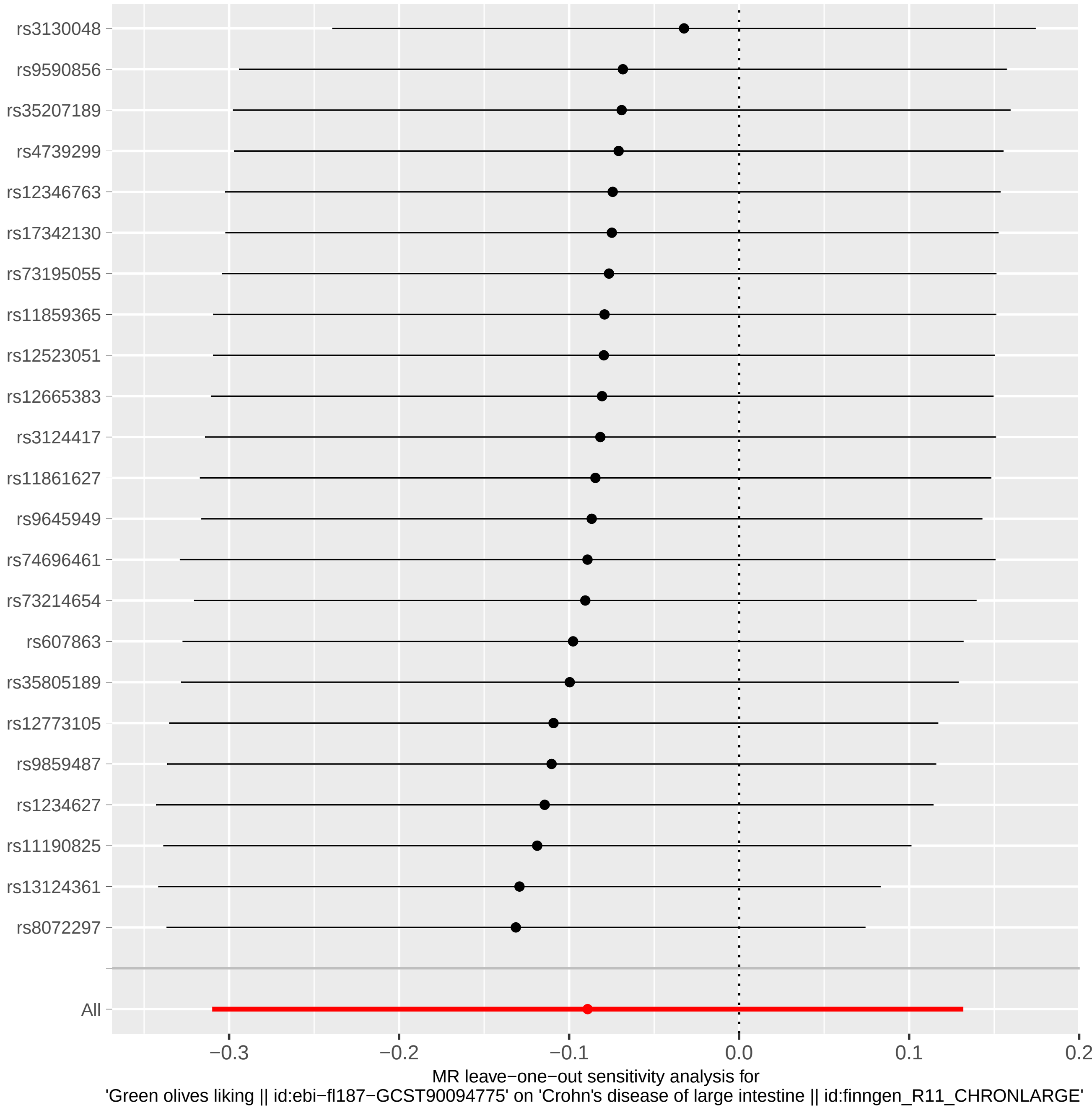
rs3130048
rs9590856
rs35207189
rs4739299
rs12346763
rs17342130
rs73195055
rs11859365
rs12523051
rs12665383
rs3124417
rs11861627
rs9645949
rs74696461
rs73214654
rs607863
rs35805189
rs12773105
rs9859487
rs1234627
rs11190825
rs13124361
rs8072297
All
−0.3
−0.2
−0.1
0.0
0.1
0.2
MR leave−one−out sensitivity analysis for
'Green olives liking || id:ebi−fl187−GCST90094775' on 'Crohn's disease of large intestine || id:finngen_R11_CHRONLARGE'

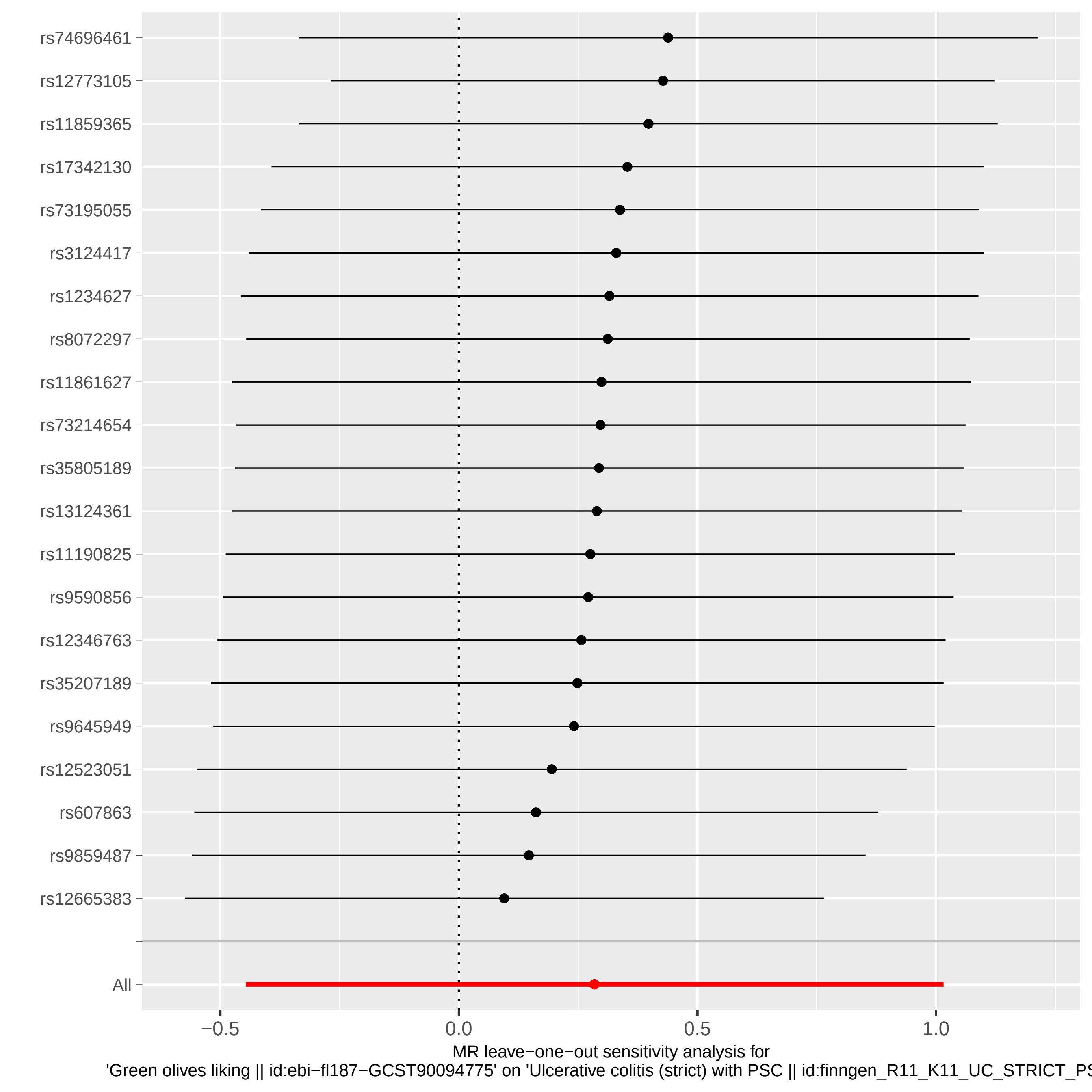

rs74696461
rs12773105
rs11859365
rs17342130
rs73195055
rs3124417
rs1234627
rs8072297
rs11861627
rs73214654
rs35805189
rs13124361
rs11190825
rs9590856
rs12346763
rs35207189
rs9645949
rs12523051
rs607863
rs9859487
rs12665383
All
−0.5
0.0
0.5
1.0
MR leave−one−out sensitivity analysis for
'Green olives liking || id:ebi−fl187−GCST90094775' on 'Ulcerative colitis (strict) with PSC || id:finngen_R11_K11_UC_STRICT_PS

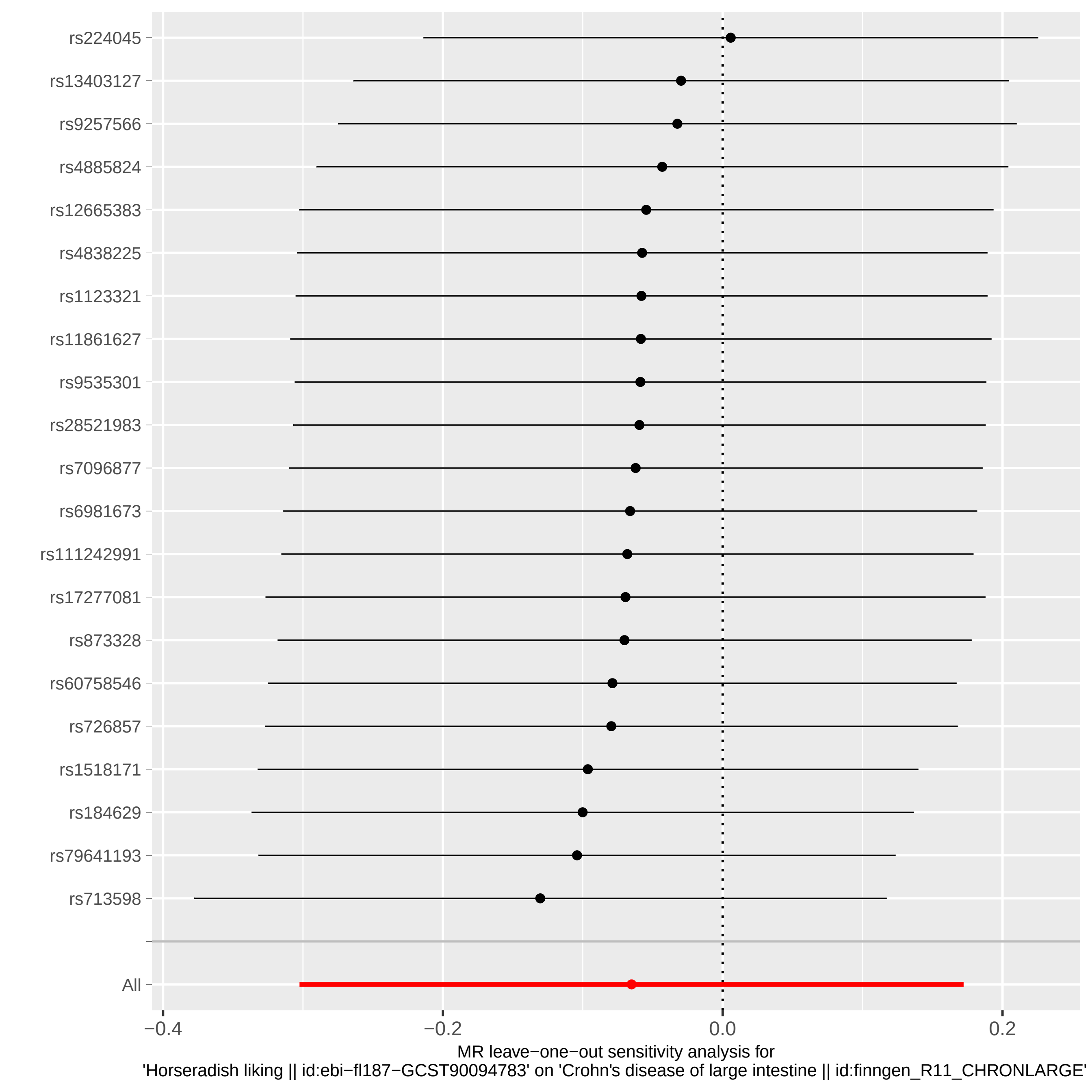
rs224045
rs13403127
rs9257566
rs4885824
rs12665383
rs4838225
rs1123321
rs11861627
rs9535301
rs28521983
rs7096877
rs6981673
rs111242991
rs17277081
rs873328
rs60758546
rs726857
rs1518171
rs184629
rs79641193
rs713598
All
−0.4
−0.2
0.0
0.2
MR leave−one−out sensitivity analysis for
'Horseradish liking || id:ebi−fl187−GCST90094783' on 'Crohn's disease of large intestine || id:finngen_R11_CHRONLARGE'

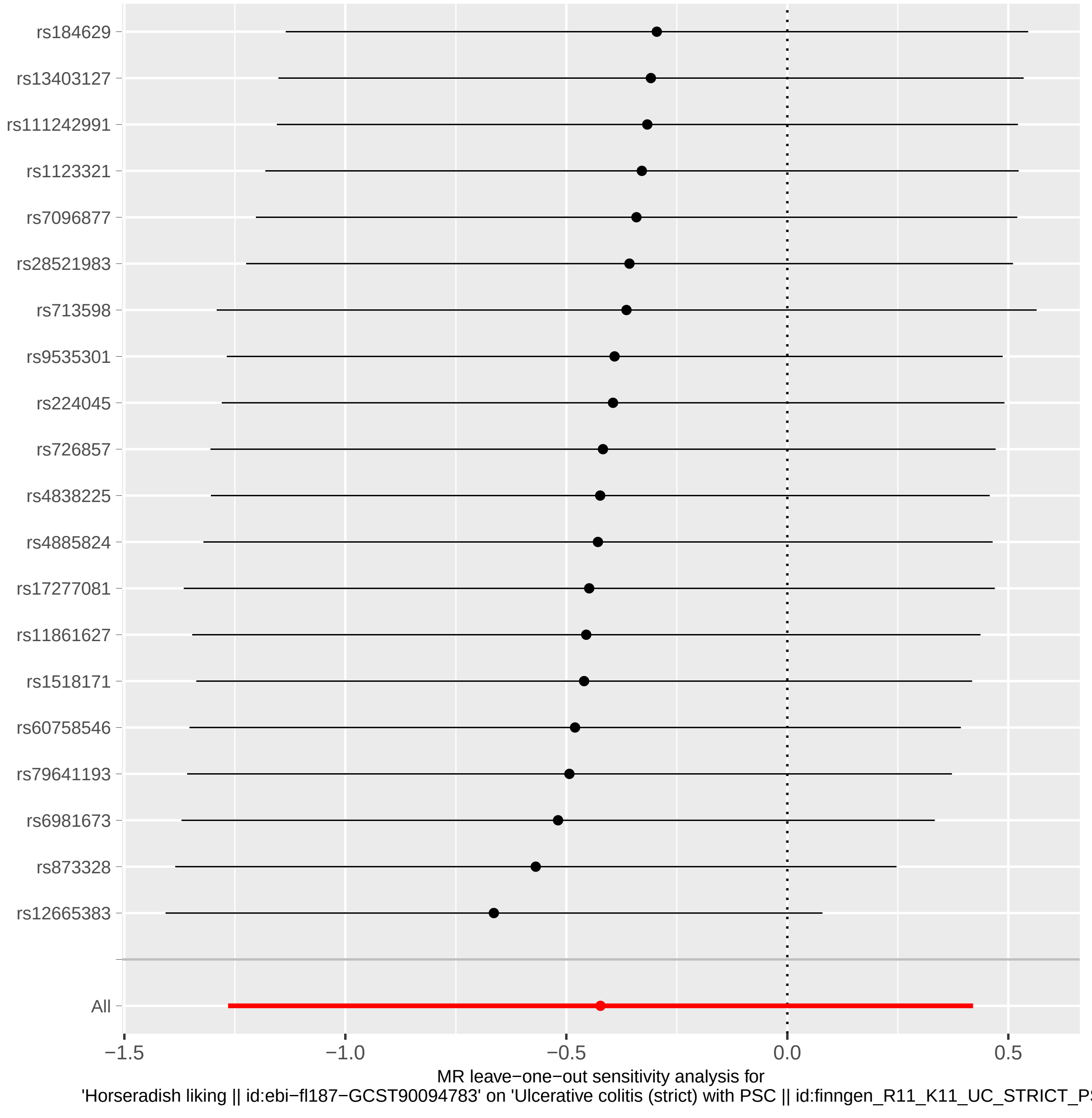

rs184629
rs13403127
rs111242991
rs1123321
rs7096877
rs28521983
rs713598
rs9535301
rs224045
rs726857
rs4838225
rs4885824
rs17277081
rs11861627
rs1518171
rs60758546
rs79641193
rs6981673
rs873328
rs12665383
All
−1.5
−1.0
−0.5
0.0
0.5
MR leave−one−out sensitivity analysis for
'Horseradish liking || id:ebi−fl187−GCST90094783' on 'Ulcerative colitis (strict) with PSC || id:finngen_R11_K11_UC_STRICT_P

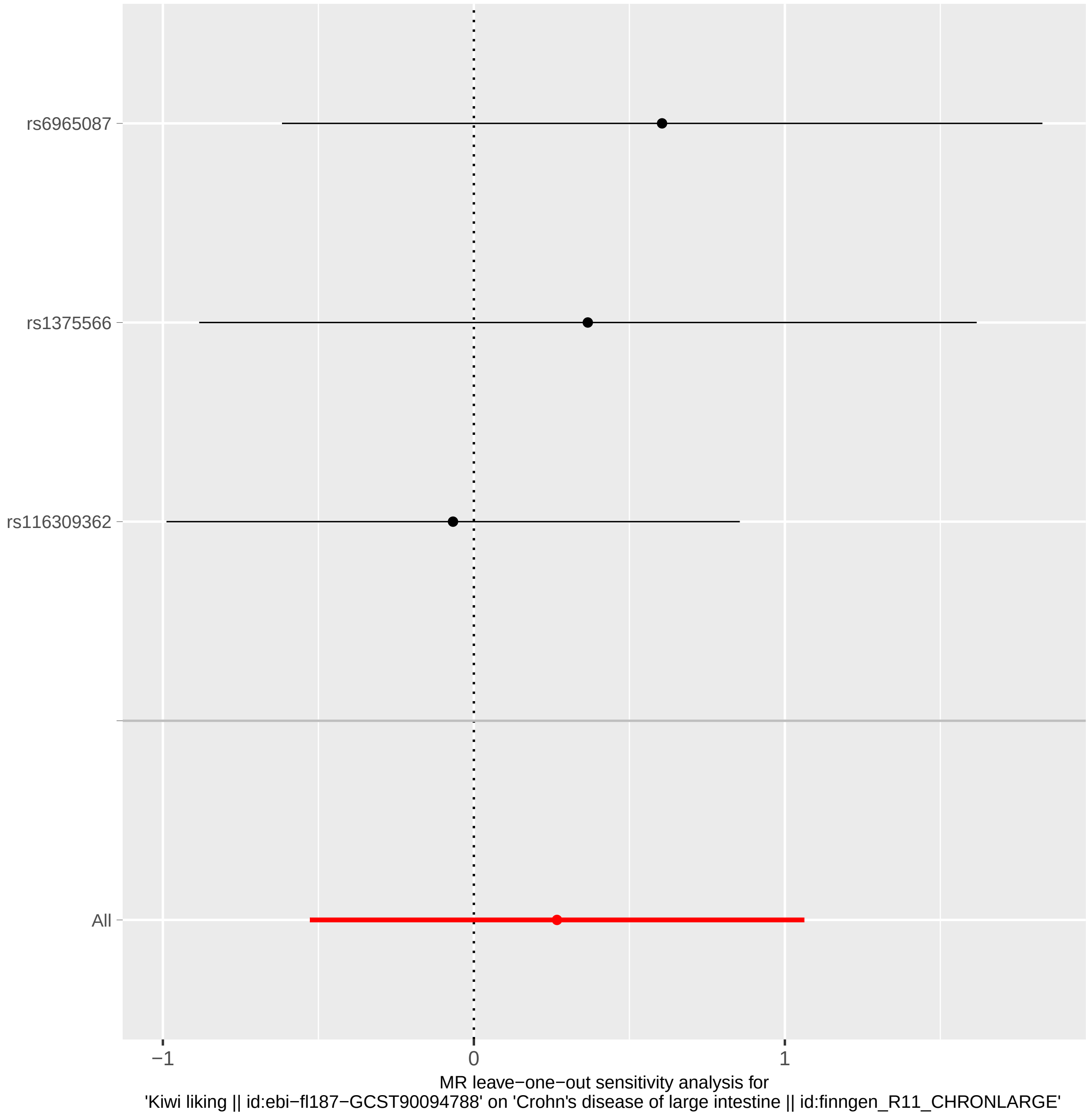

rs6965087
rs1375566
rs116309362
All
−1
0
1
MR leave−one−out sensitivity analysis for
'Kiwi liking || id:ebi−fl187−GCST90094788' on 'Crohn's disease of large intestine || id:finngen_R11_CHRONLARGE'

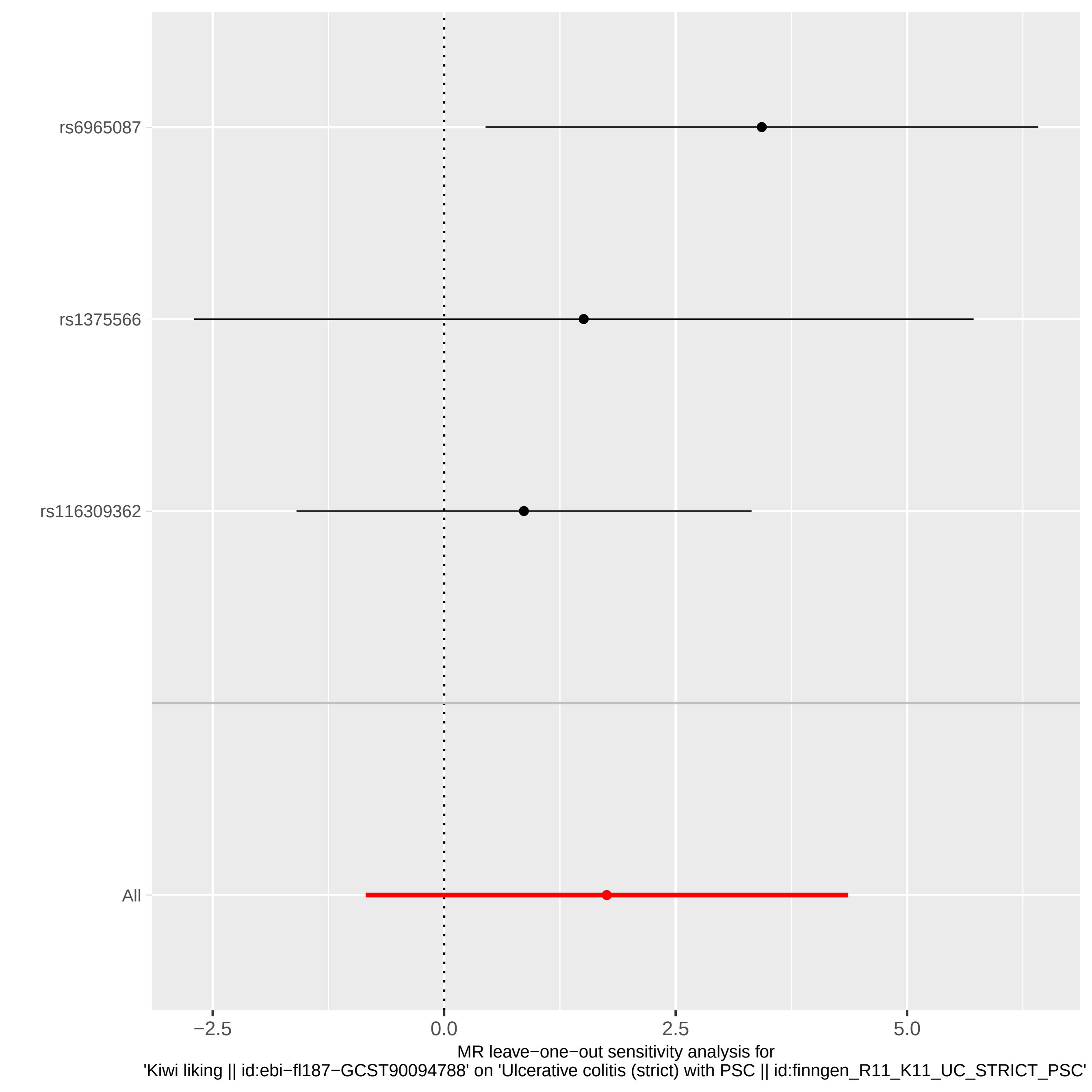
rs6965087
rs1375566
rs116309362
All
−2.5
0.0
2.5
5.0
MR leave−one−out sensitivity analysis for
'Kiwi liking || id:ebi−fl187−GCST90094788' on 'Ulcerative colitis (strict) with PSC || id:finngen_R11_K11_UC_STRICT_PSC'

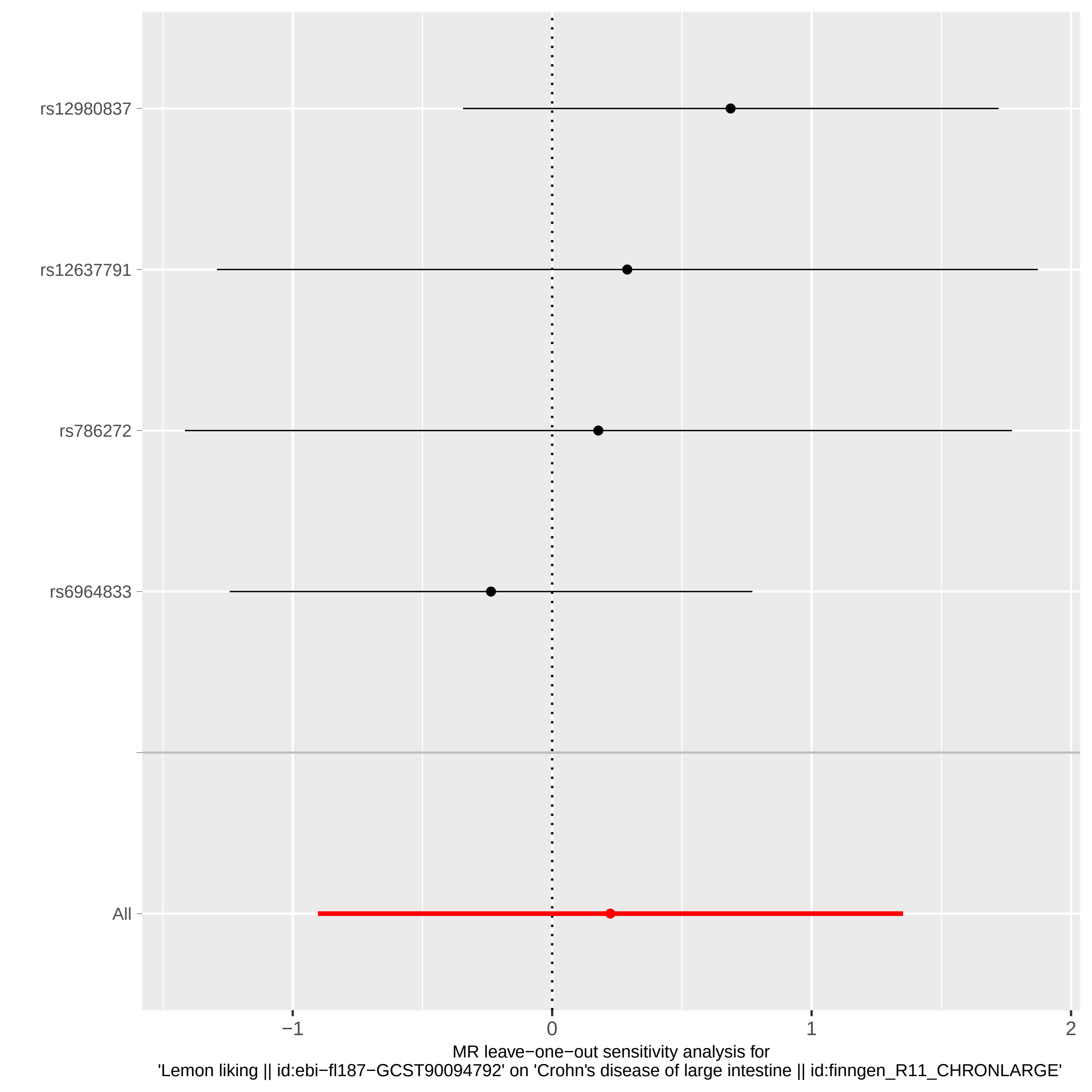

rs12980837
rs12637791
rs786272
rs6964833
All
−1
0
1
2
MR leave−one−out sensitivity analysis for
'Lemon liking || id:ebi−fl187−GCST90094792' on 'Crohn's disease of large intestine || id:finngen_R11_CHRONLARGE'

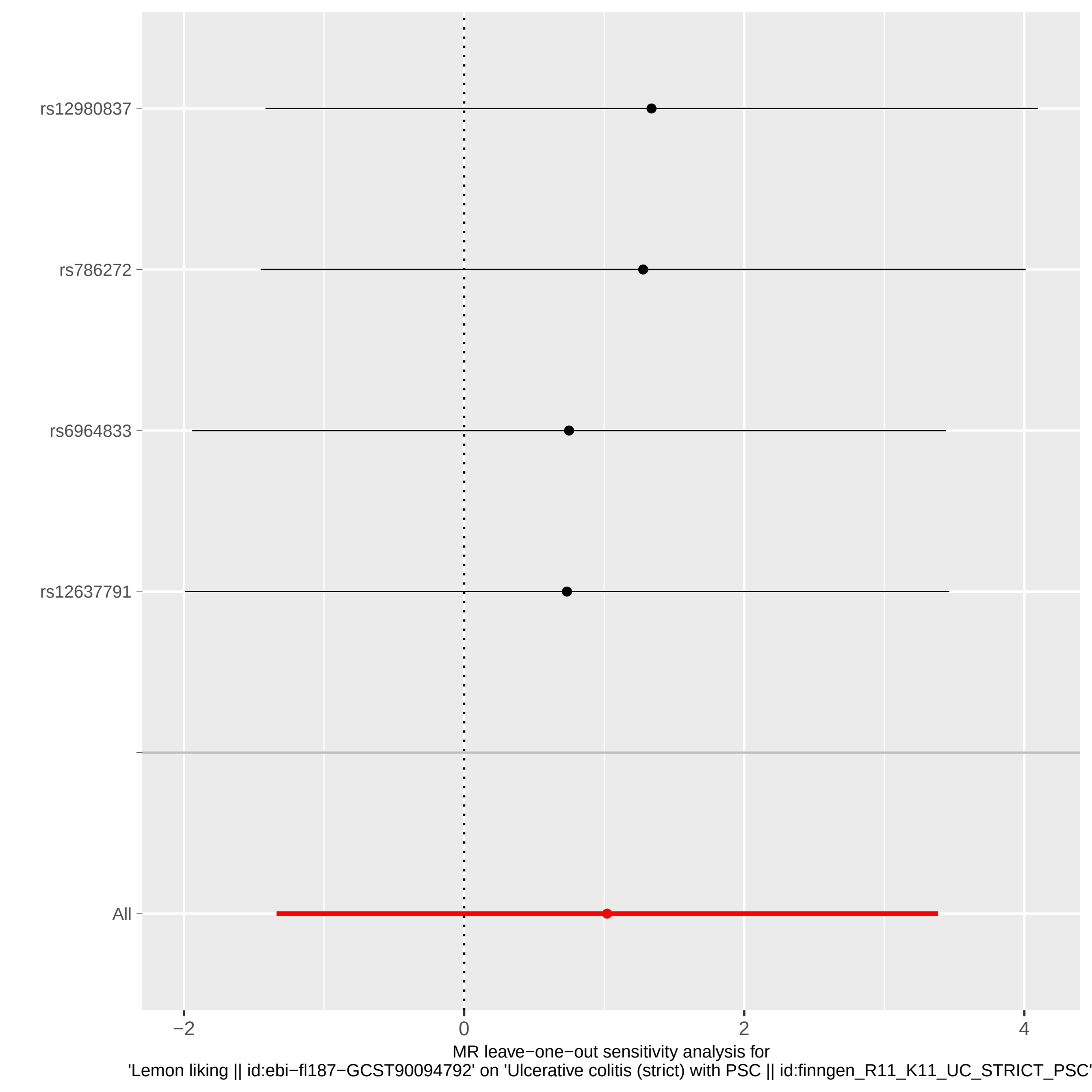
rs12980837
rs786272
rs6964833
rs12637791
All
−2
0
2
4
MR leave−one−out sensitivity analysis for
'Lemon liking || id:ebi−fl187−GCST90094792' on 'Ulcerative colitis (strict) with PSC || id:finngen_R11_K11_UC_STRICT_PSC'

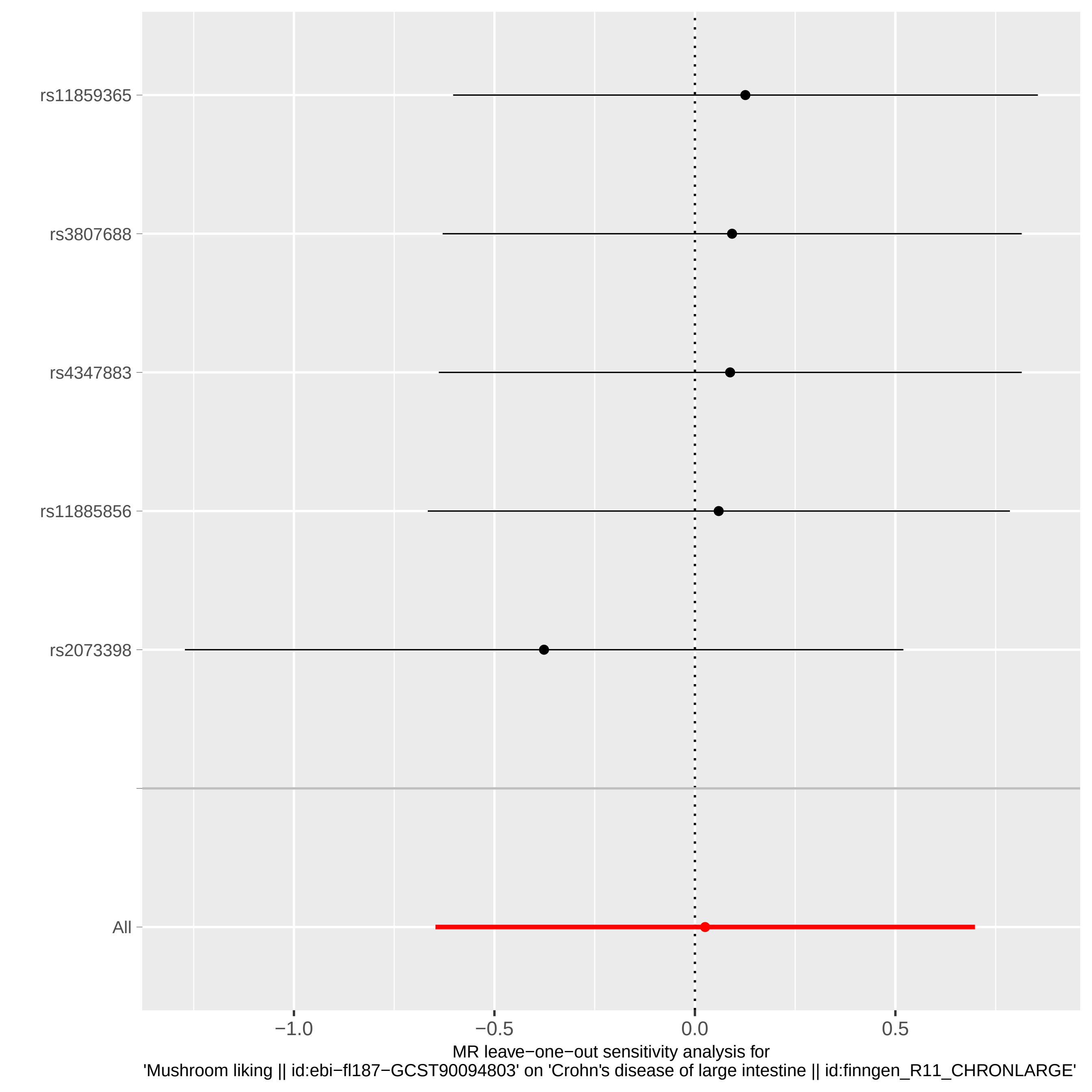

rs11859365
rs3807688
rs4347883
rs11885856
rs2073398
All
−1.0
−0.5
0.0
0.5
MR leave−one−out sensitivity analysis for
'Mushroom liking || id:ebi−fl187−GCST90094803' on 'Crohn's disease of large intestine || id:finngen_R11_CHRONLARGE'

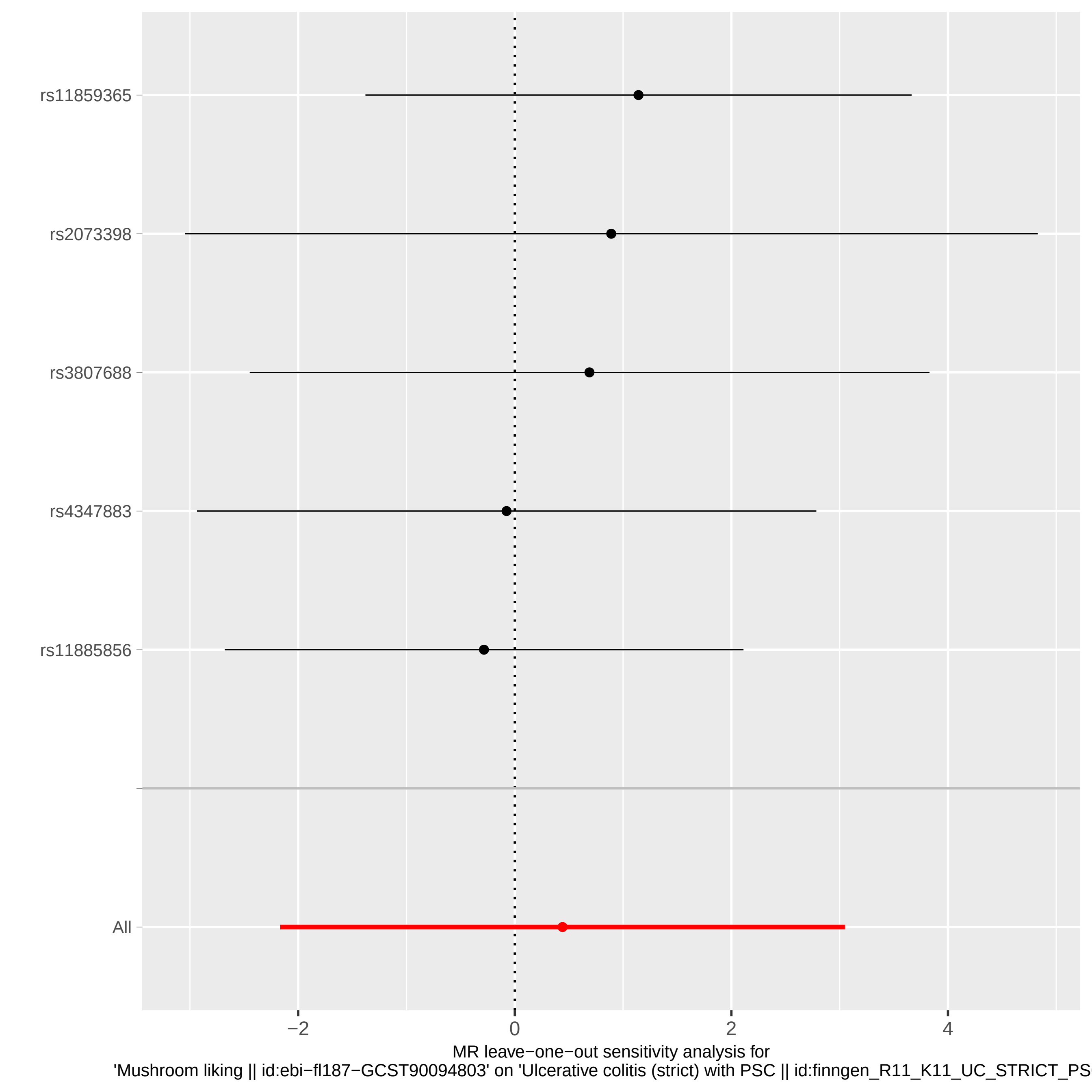
rs11859365
rs2073398
rs3807688
rs4347883
rs11885856
All
−2
0
2
4
MR leave−one−out sensitivity analysis for
'Mushroom liking || id:ebi−fl187−GCST90094803' on 'Ulcerative colitis (strict) with PSC || id:finngen_R11_K11_UC_STRICT_PS

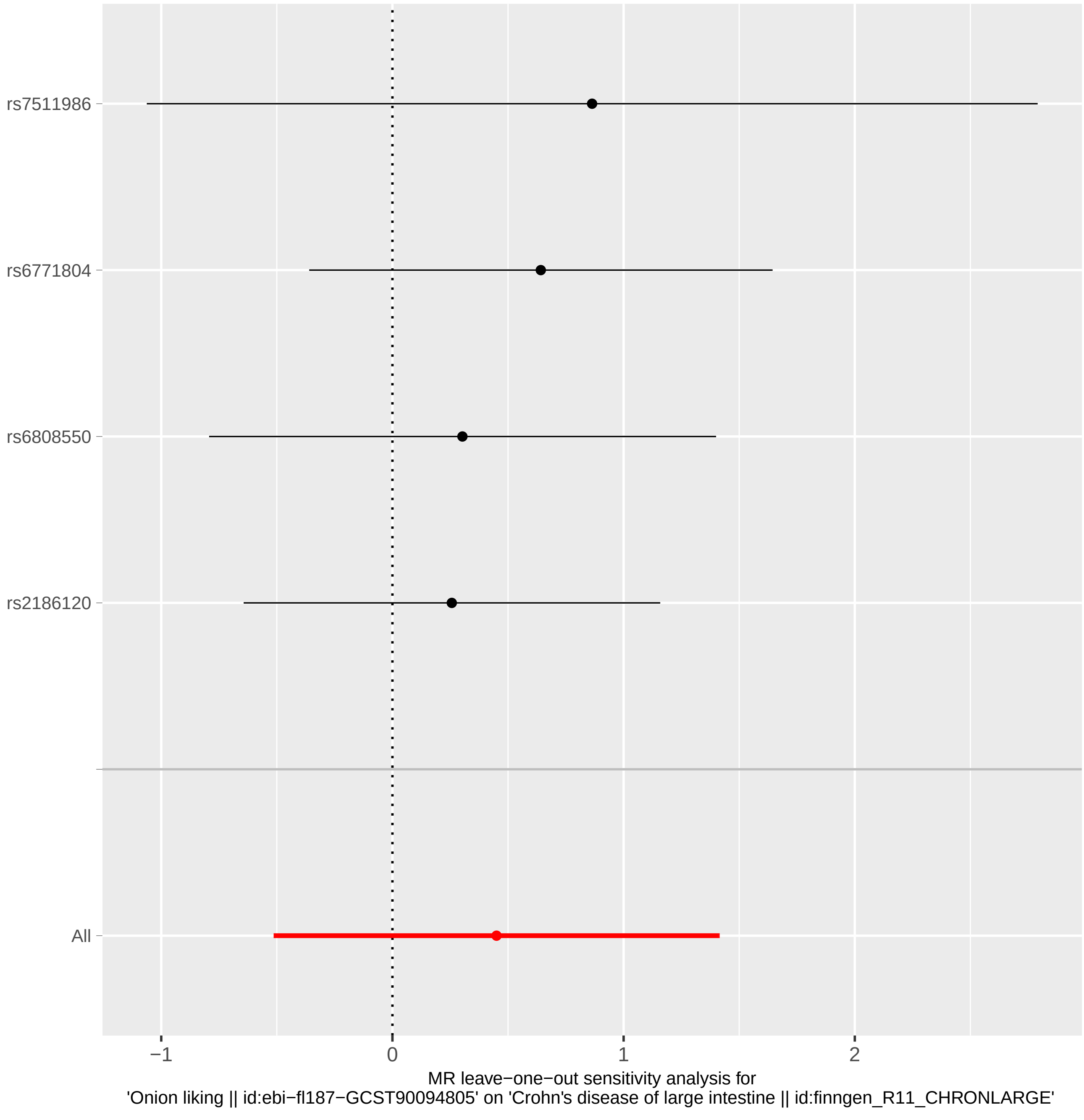
rs7511986
rs6771804
rs6808550
rs2186120
All
−1
0
1
2
MR leave−one−out sensitivity analysis for
'Onion liking || id:ebi−fl187−GCST90094805' on 'Crohn's disease of large intestine || id:finngen_R11_CHRONLARGE'

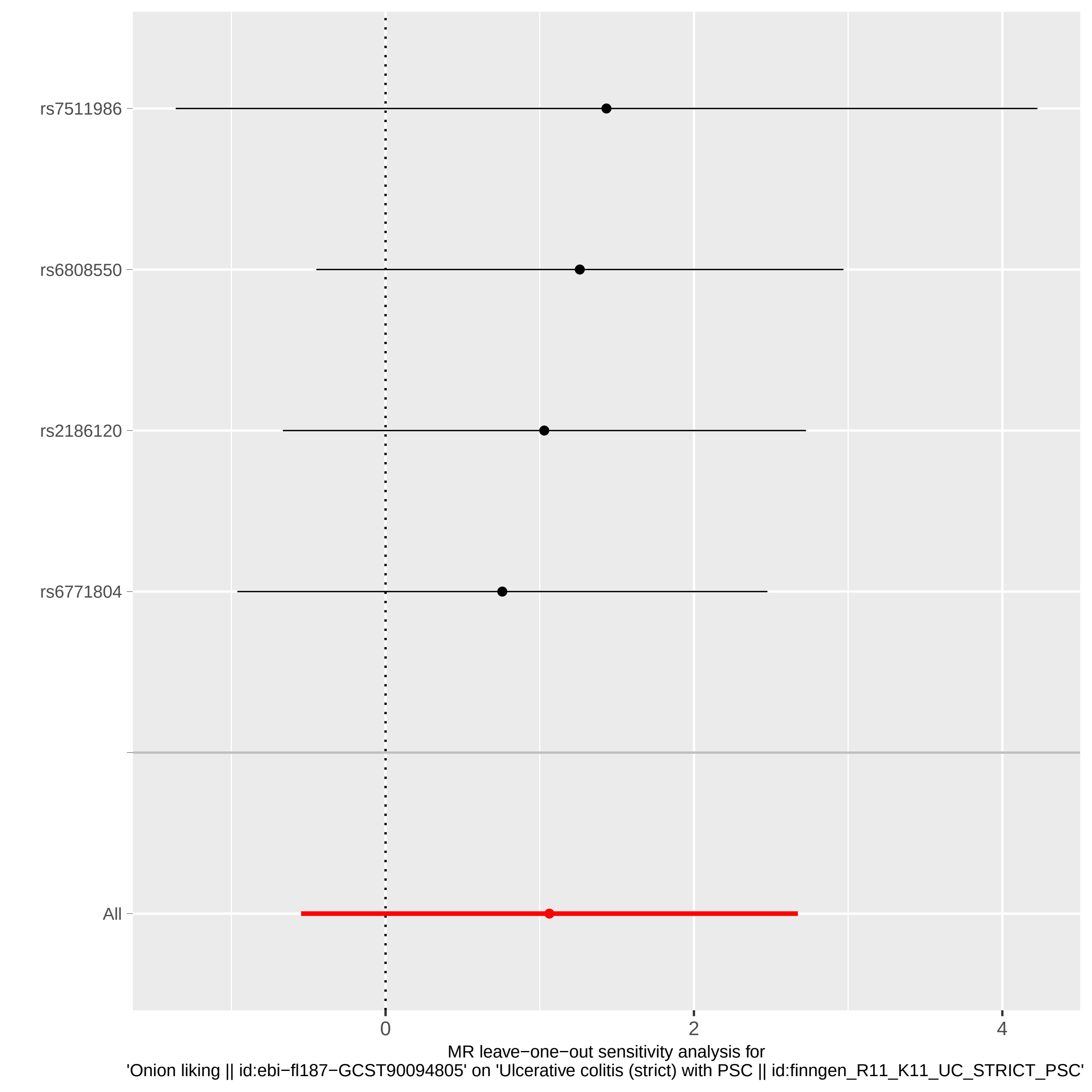
rs7511986
rs6808550
rs2186120
rs6771804
All
0
2
4
MR leave−one−out sensitivity analysis for
'Onion liking || id:ebi−fl187−GCST90094805' on 'Ulcerative colitis (strict) with PSC || id:finngen_R11_K11_UC_STRICT_PSC'

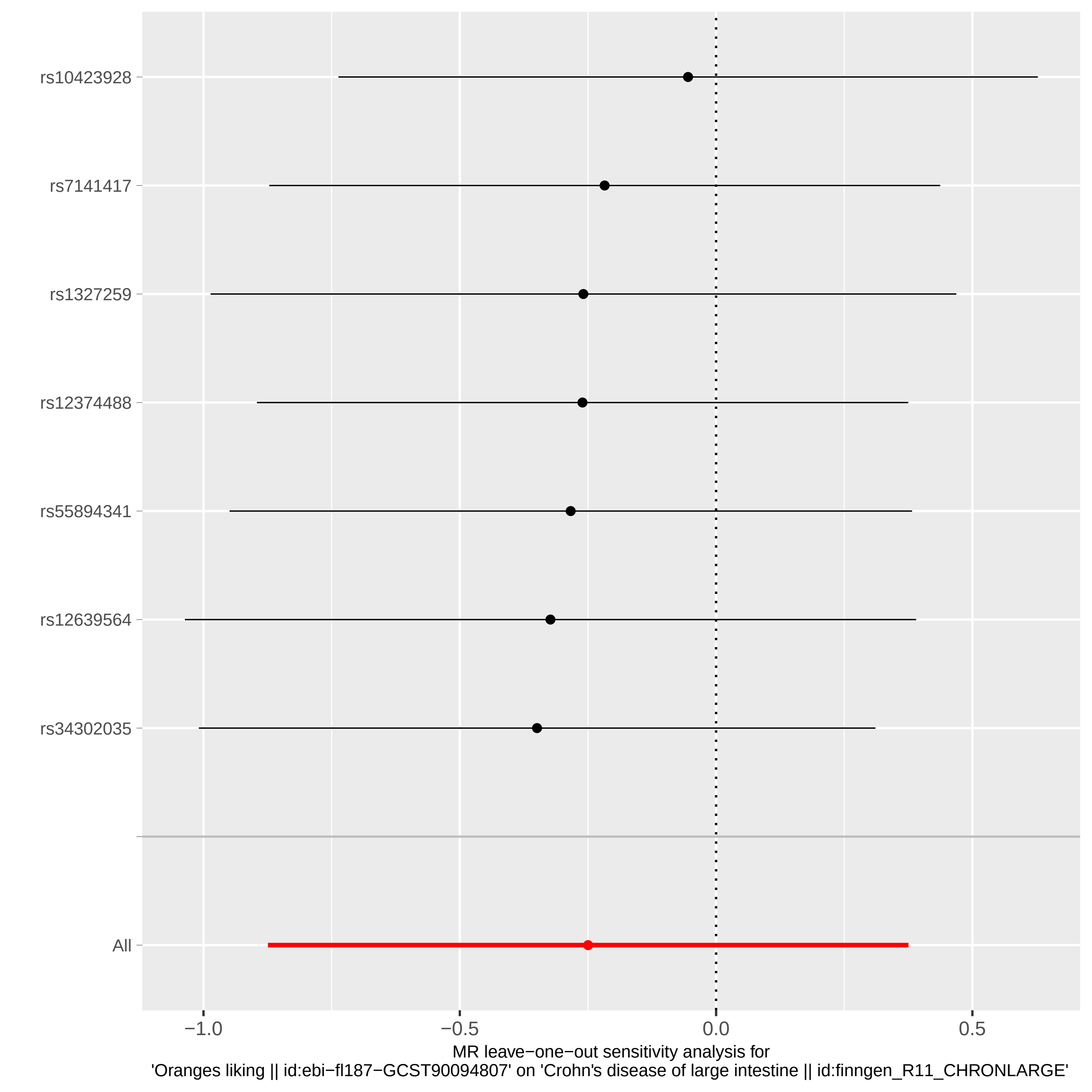
rs10423928
rs7141417
rs1327259
rs12374488
rs55894341
rs12639564
rs34302035
All
−1.0
−0.5
0.0
0.5
MR leave−one−out sensitivity analysis for
'Oranges liking || id:ebi−fl187−GCST90094807' on 'Crohn's disease of large intestine || id:finngen_R11_CHRONLARGE'

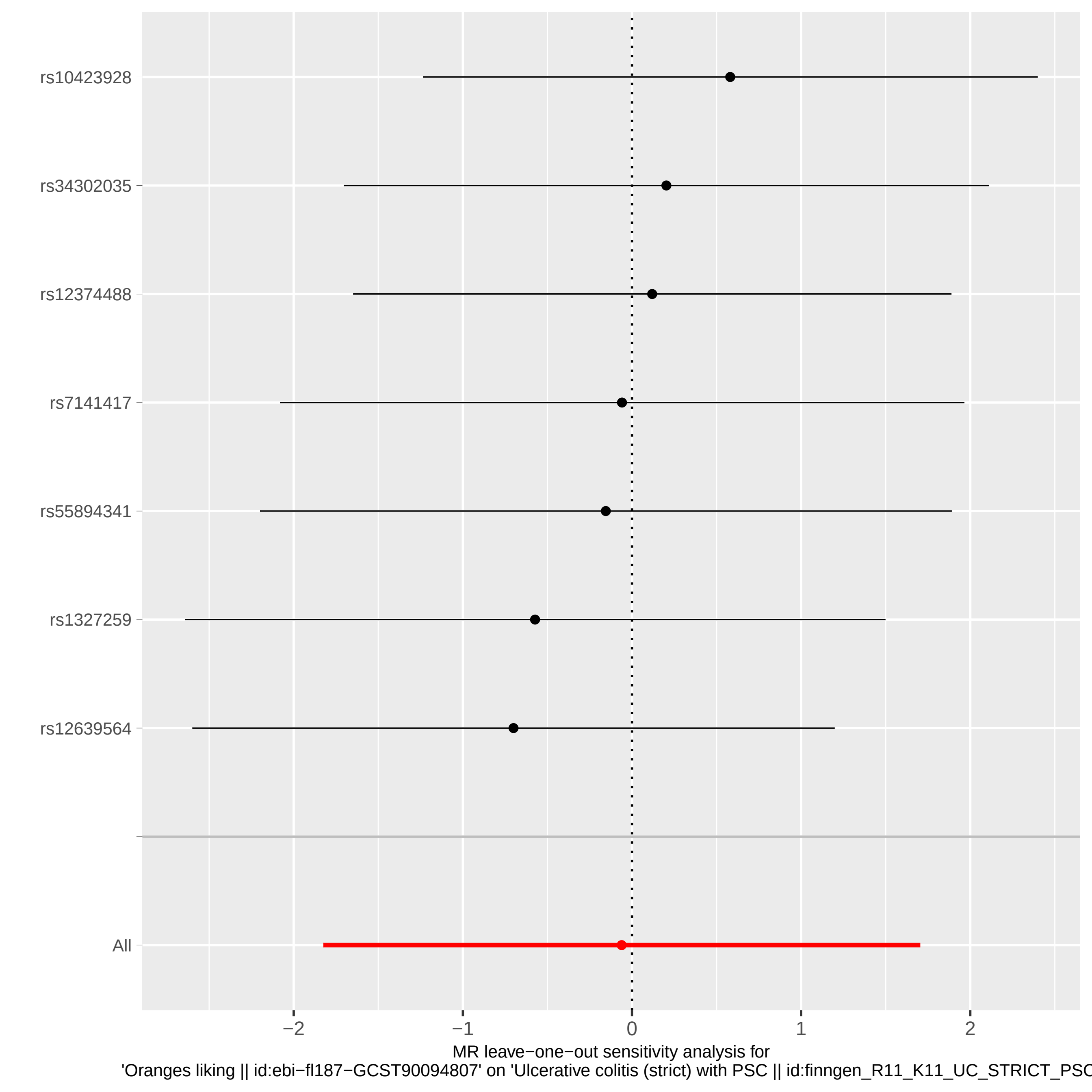
rs10423928
rs34302035
rs12374488
rs7141417
rs55894341
rs1327259
rs12639564
All
−2
−1
0
1
2
MR leave−one−out sensitivity analysis for
'Oranges liking || id:ebi−fl187−GCST90094807' on 'Ulcerative colitis (strict) with PSC || id:finngen_R11_K11_UC_STRICT_PSC

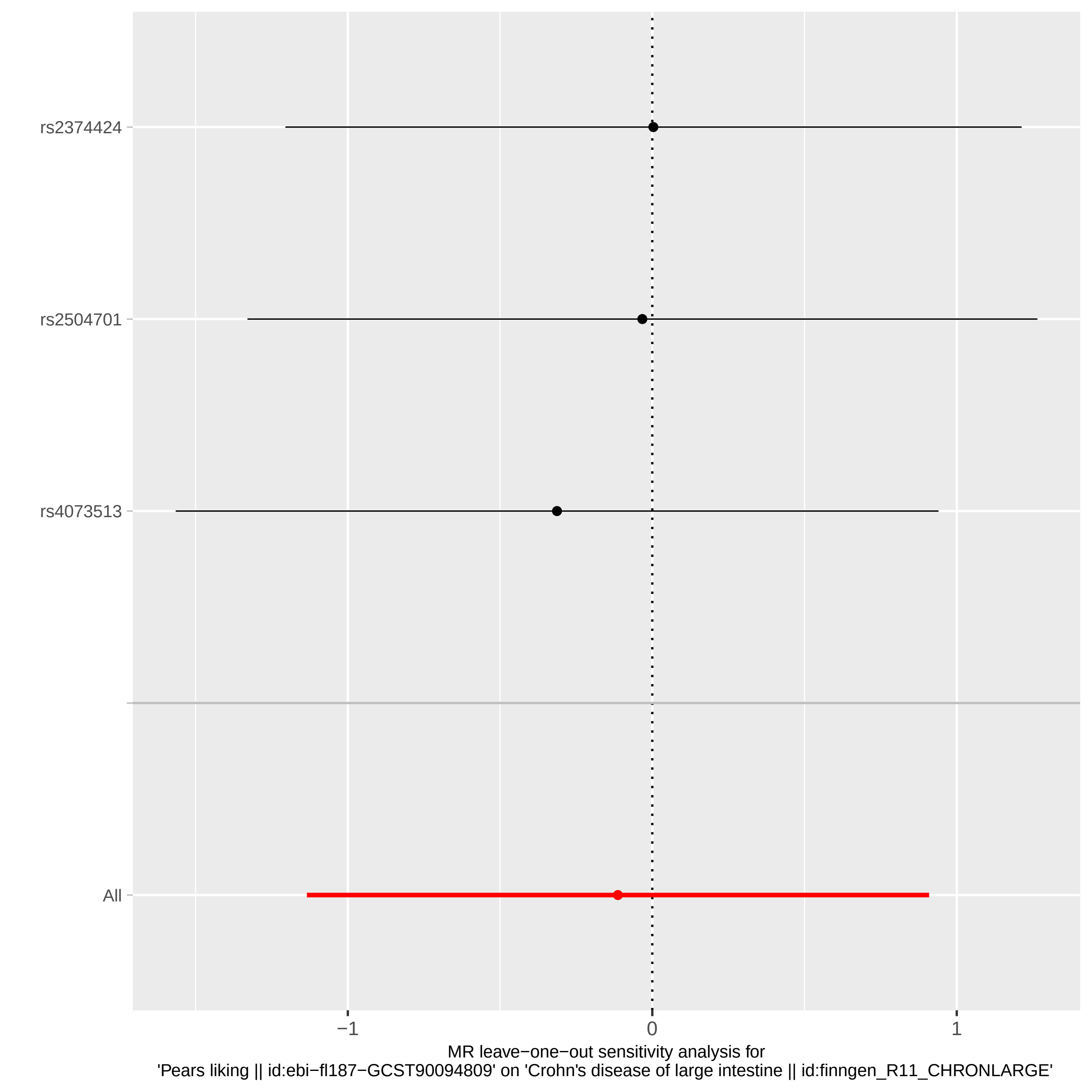
rs2374424
rs2504701
rs4073513
All
−1
0
1
MR leave−one−out sensitivity analysis for
'Pears liking || id:ebi−fl187−GCST90094809' on 'Crohn's disease of large intestine || id:finngen_R11_CHRONLARGE'

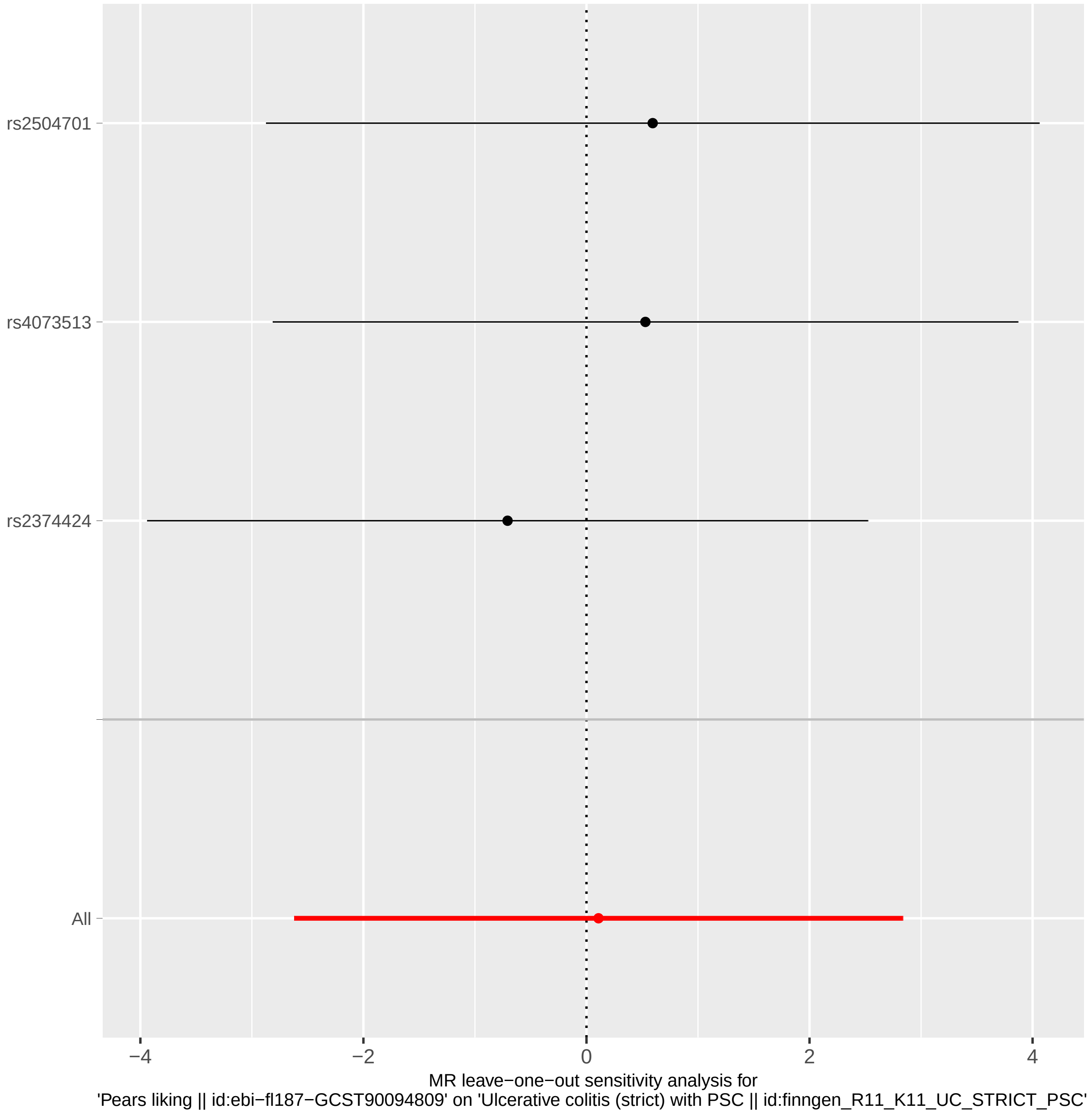
rs2504701
rs4073513
rs2374424
All
−4
−2
0
2
4
MR leave−one−out sensitivity analysis for
'Pears liking || id:ebi−fl187−GCST90094809' on 'Ulcerative colitis (strict) with PSC || id:finngen_R11_K11_UC_STRICT_PSC'

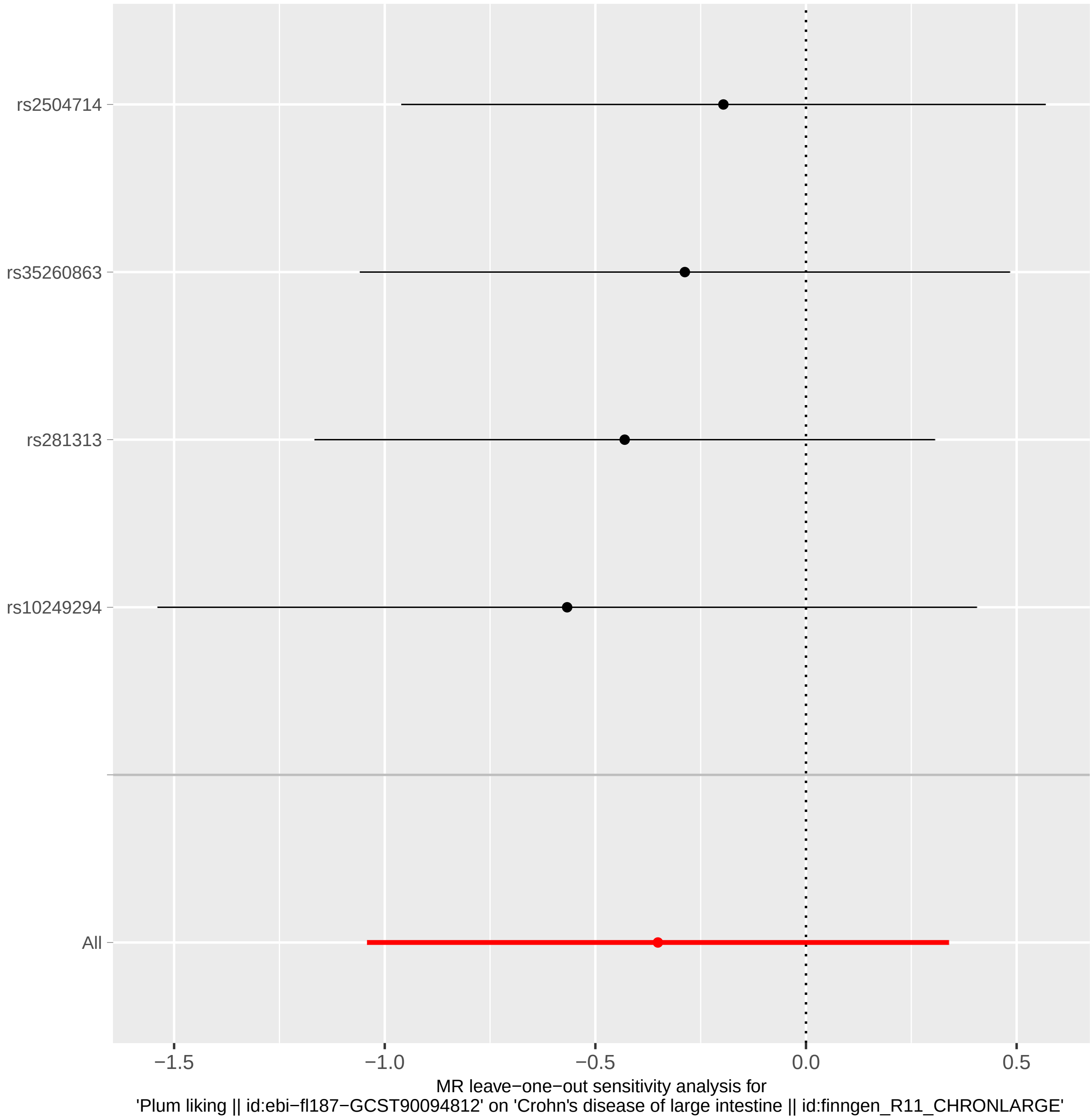
rs2504714
rs35260863
rs281313
rs10249294
All
−1.5
−1.0
−0.5
0.0
0.5
MR leave−one−out sensitivity analysis for
'Plum liking || id:ebi−fl187−GCST90094812' on 'Crohn's disease of large intestine || id:finngen_R11_CHRONLARGE'

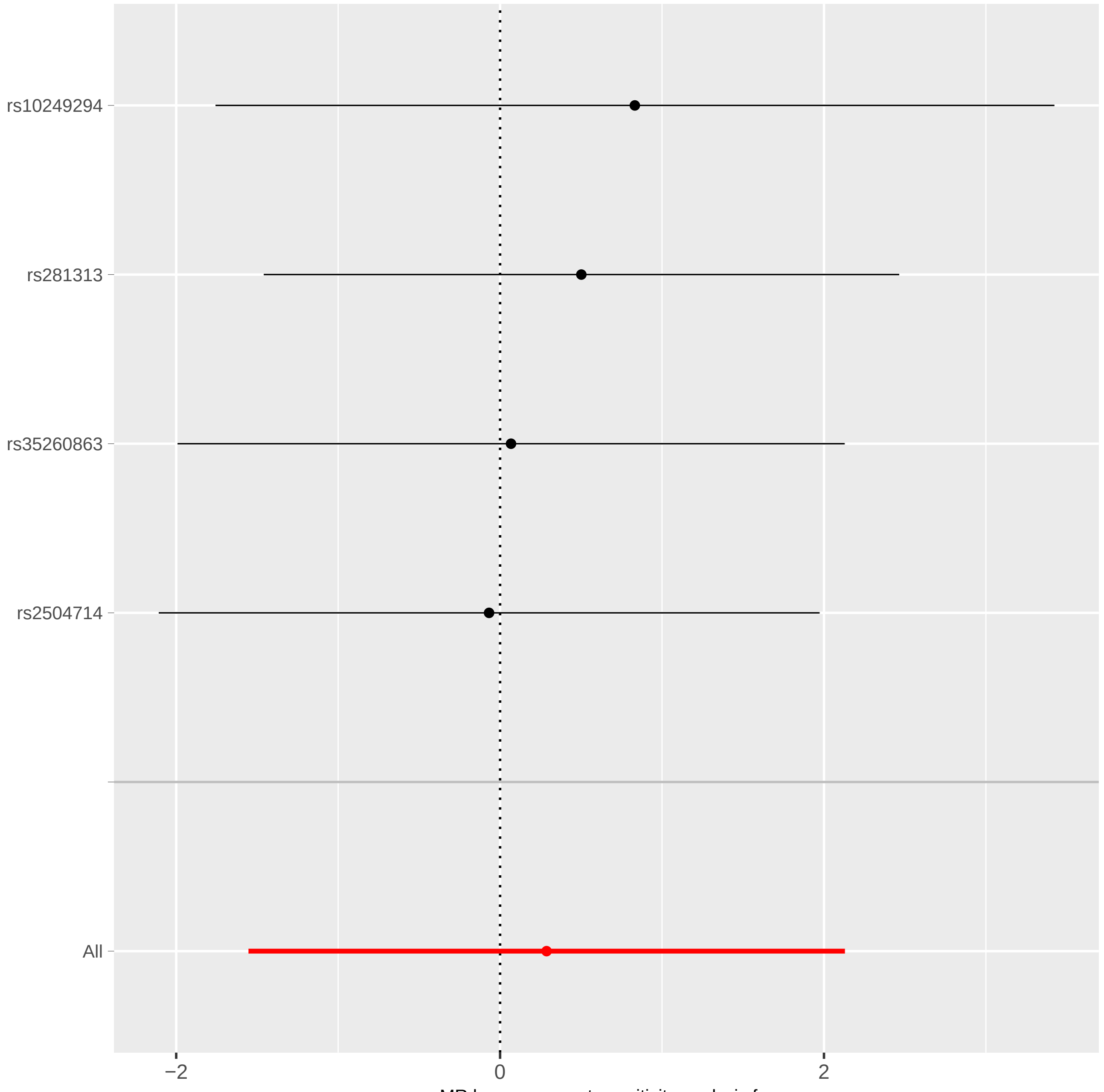

MR leave−one−out sensitivity analysis for
'Plum liking || id:ebi−fl187−GCST90094812' on 'Ulcerative colitis (strict) with PSC || id:finngen_R11_K11_UC_STRICT_PSC'

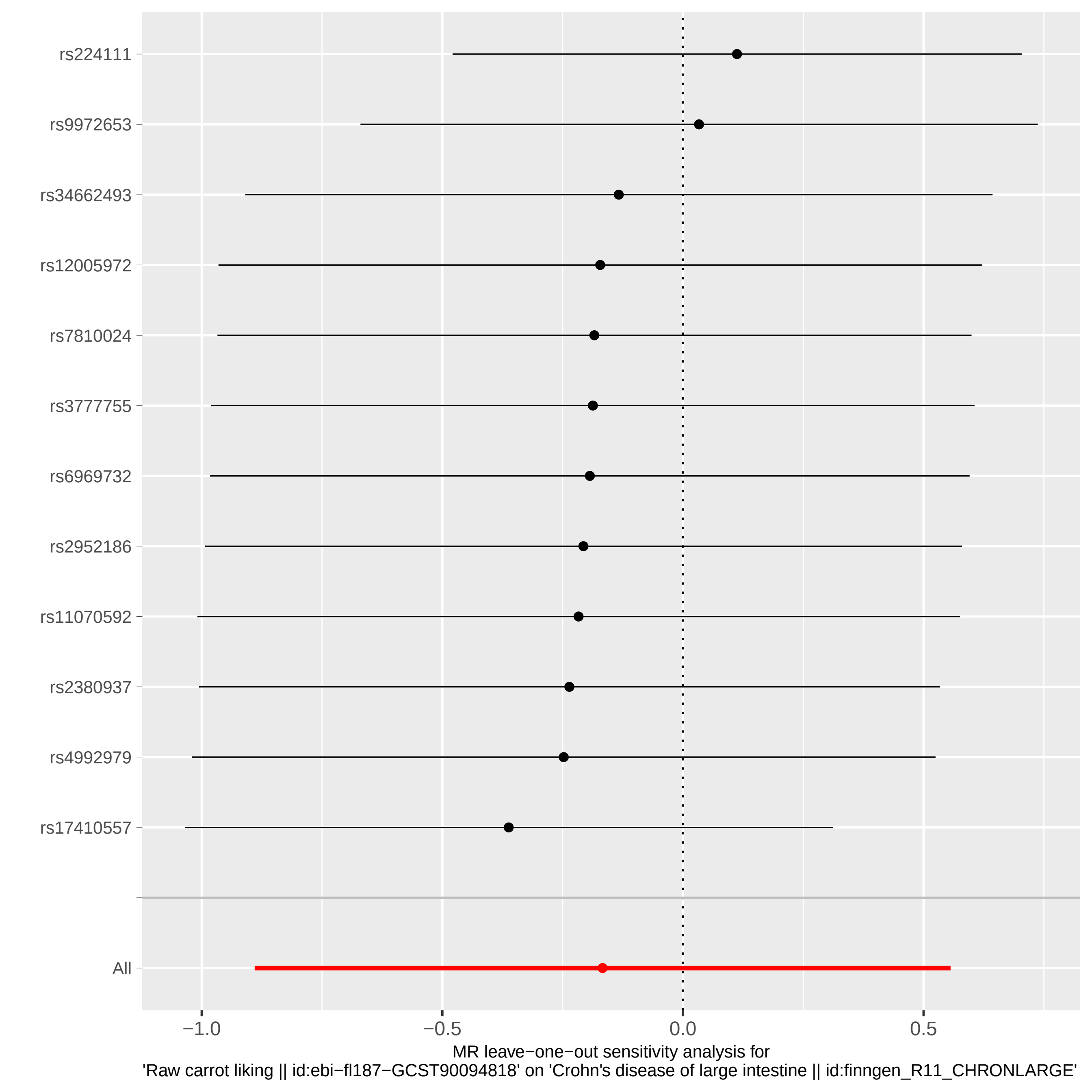
rs224111
rs9972653
rs34662493
rs12005972
rs7810024
rs3777755
rs6969732
rs2952186
rs11070592
rs2380937
rs4992979
rs17410557
All
−1.0
−0.5
0.0
0.5
MR leave−one−out sensitivity analysis for
'Raw carrot liking || id:ebi−fl187−GCST90094818' on 'Crohn's disease of large intestine || id:finngen_R11_CHRONLARGE'

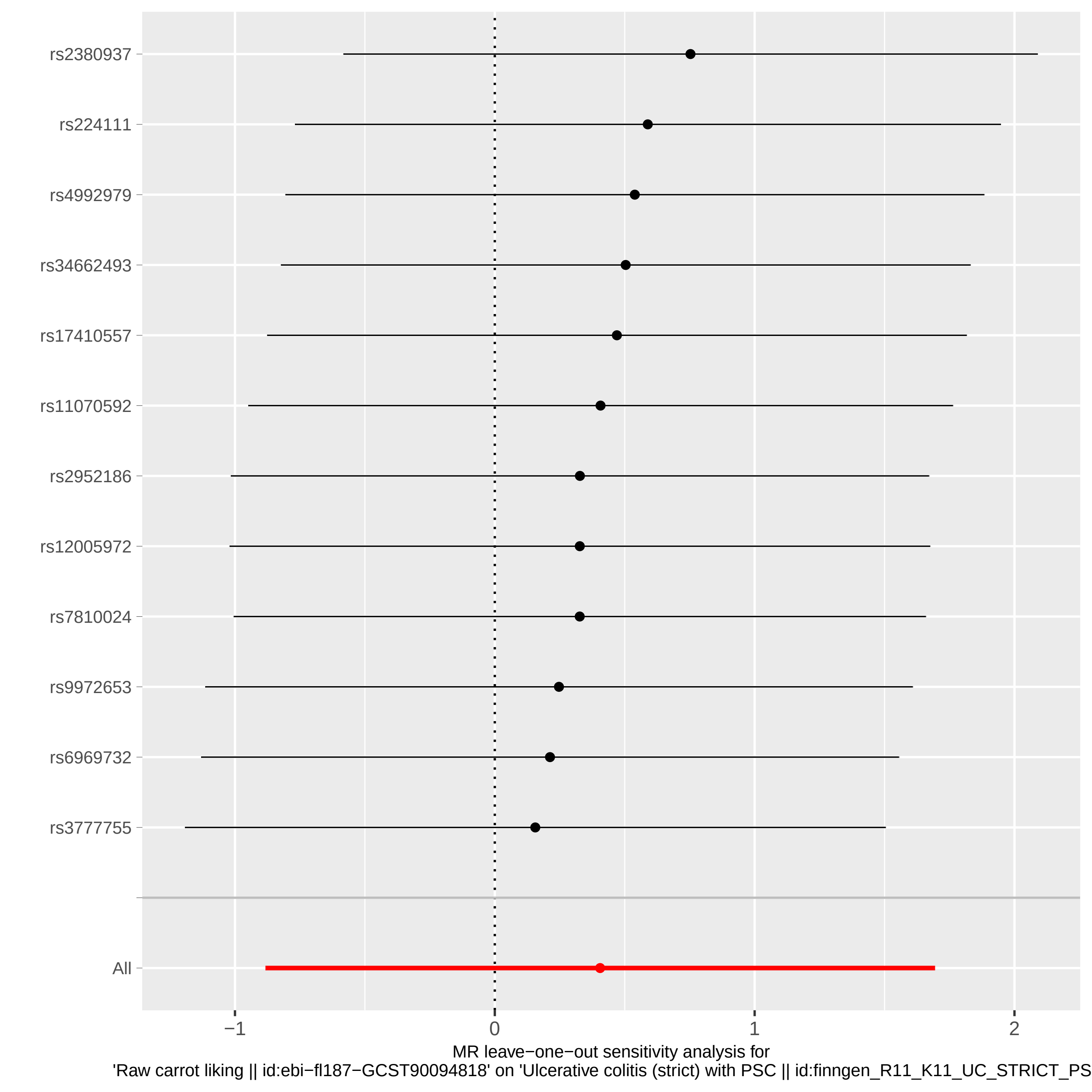
rs2380937
rs224111
rs4992979
rs34662493
rs17410557
rs11070592
rs2952186
rs12005972
rs7810024
rs9972653
rs6969732
rs3777755
All
−1
0
1
2
MR leave−one−out sensitivity analysis for
'Raw carrot liking || id:ebi−fl187−GCST90094818' on 'Ulcerative colitis (strict) with PSC || id:finngen_R11_K11_UC_STRICT_PS

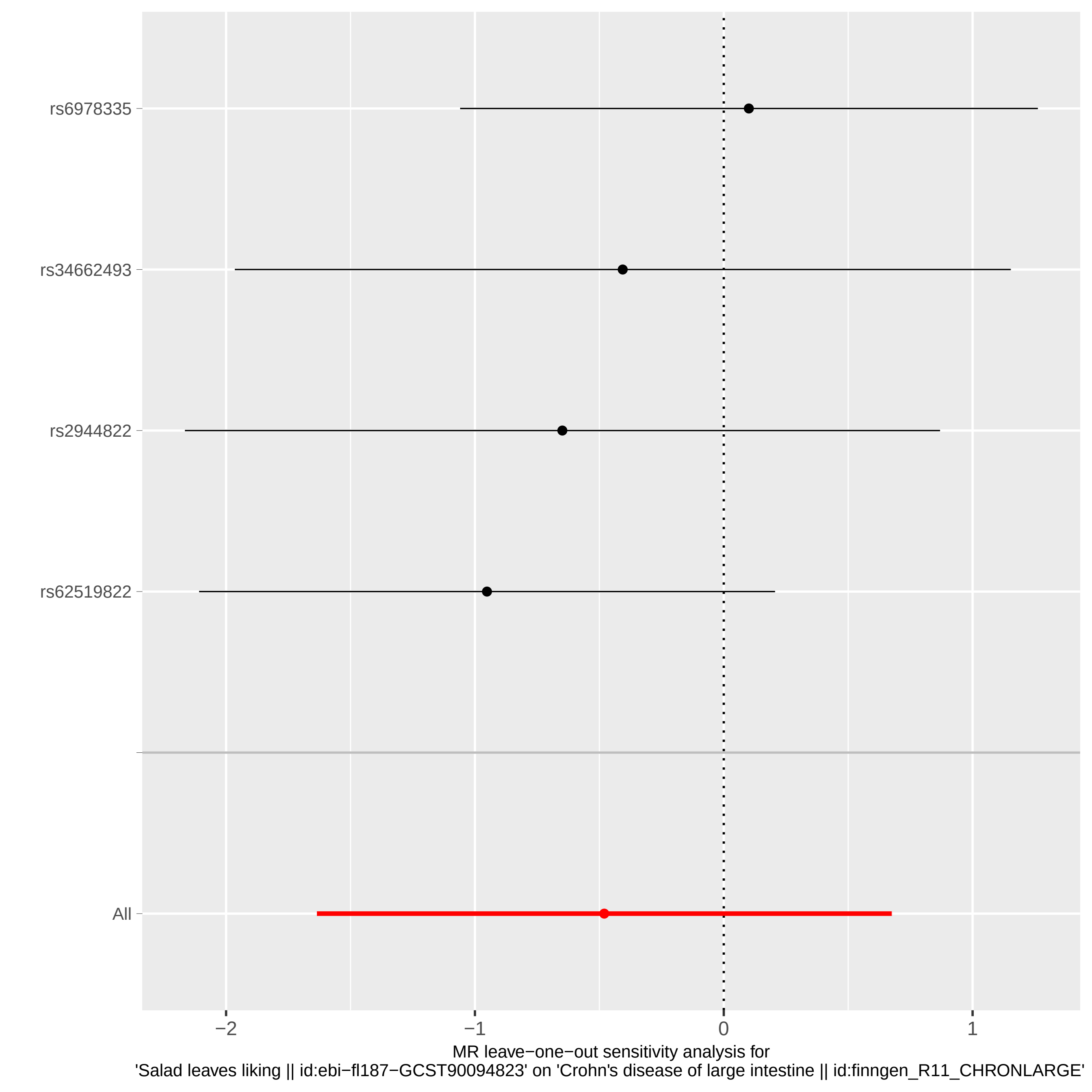
rs6978335
rs34662493
rs2944822
rs62519822
All
−2
−1
0
1
MR leave−one−out sensitivity analysis for
'Salad leaves liking || id:ebi−fl187−GCST90094823' on 'Crohn's disease of large intestine || id:finngen_R11_CHRONLARGE'

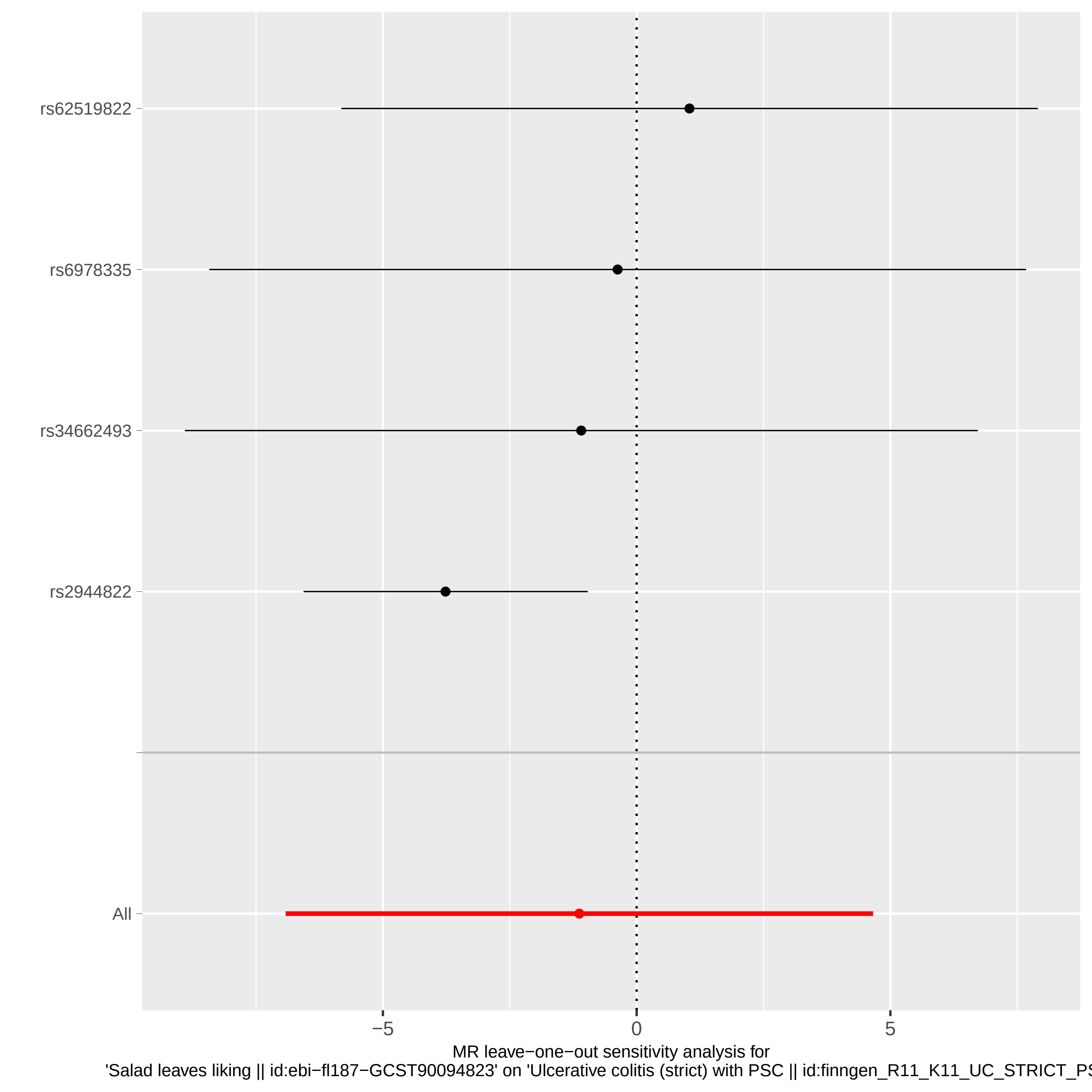

rs62519822
rs6978335
rs34662493
rs2944822
All
−5
0
5
MR leave−one−out sensitivity analysis for
'Salad leaves liking || id:ebi−fl187−GCST90094823' on 'Ulcerative colitis (strict) with PSC || id:finngen_R11_K11_UC_STRICT_PS

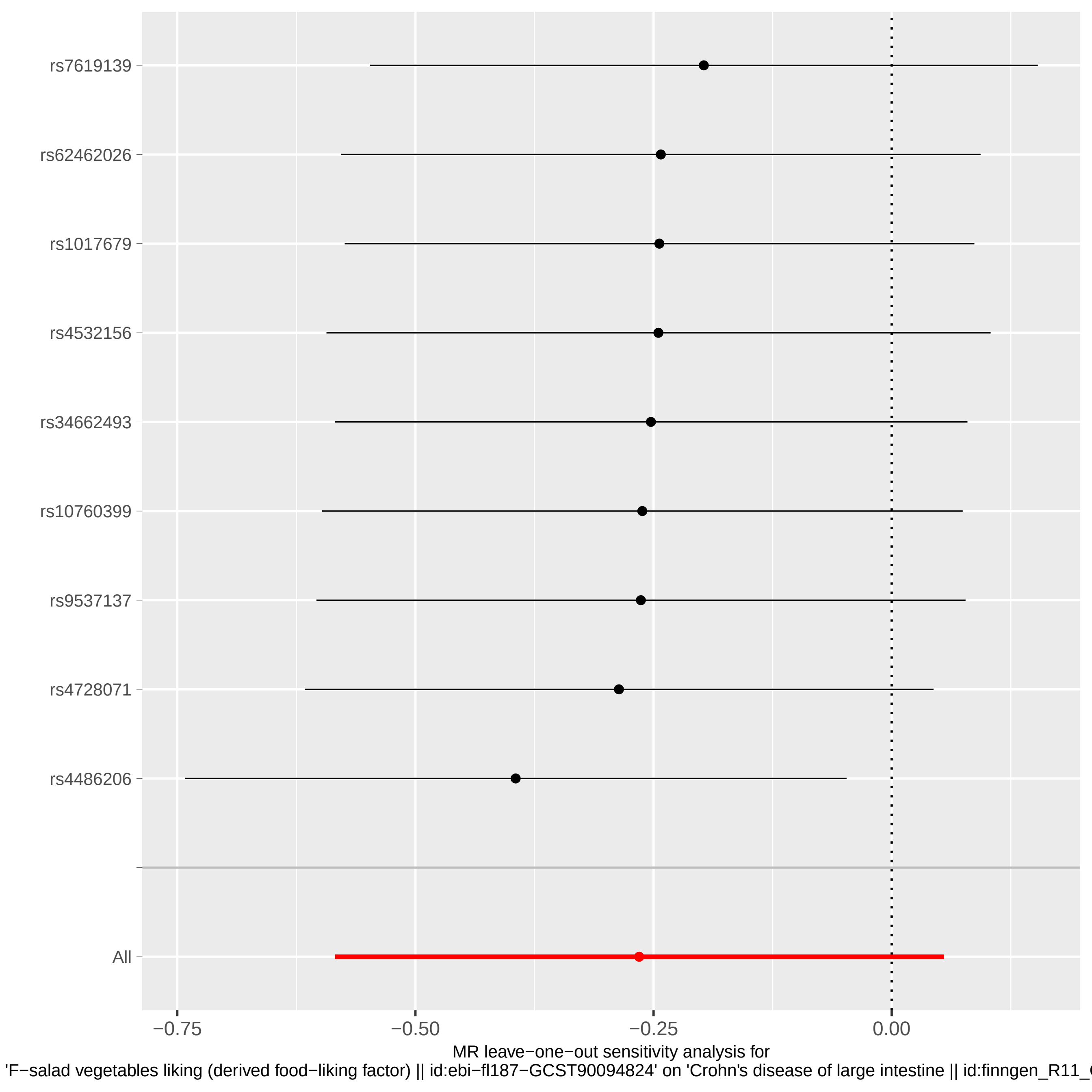

rs7619139
rs62462026
rs1017679
rs4532156
rs34662493
rs10760399
rs9537137
rs4728071
rs4486206
All
−0.75
−0.50
−0.25
0.00
MR leave−one−out sensitivity analysis for
'F−salad vegetables liking (derived food−liking factor) || id:ebi−fl187−GCST90094824' on 'Crohn's disease of large intestine || id:finngen_R11_

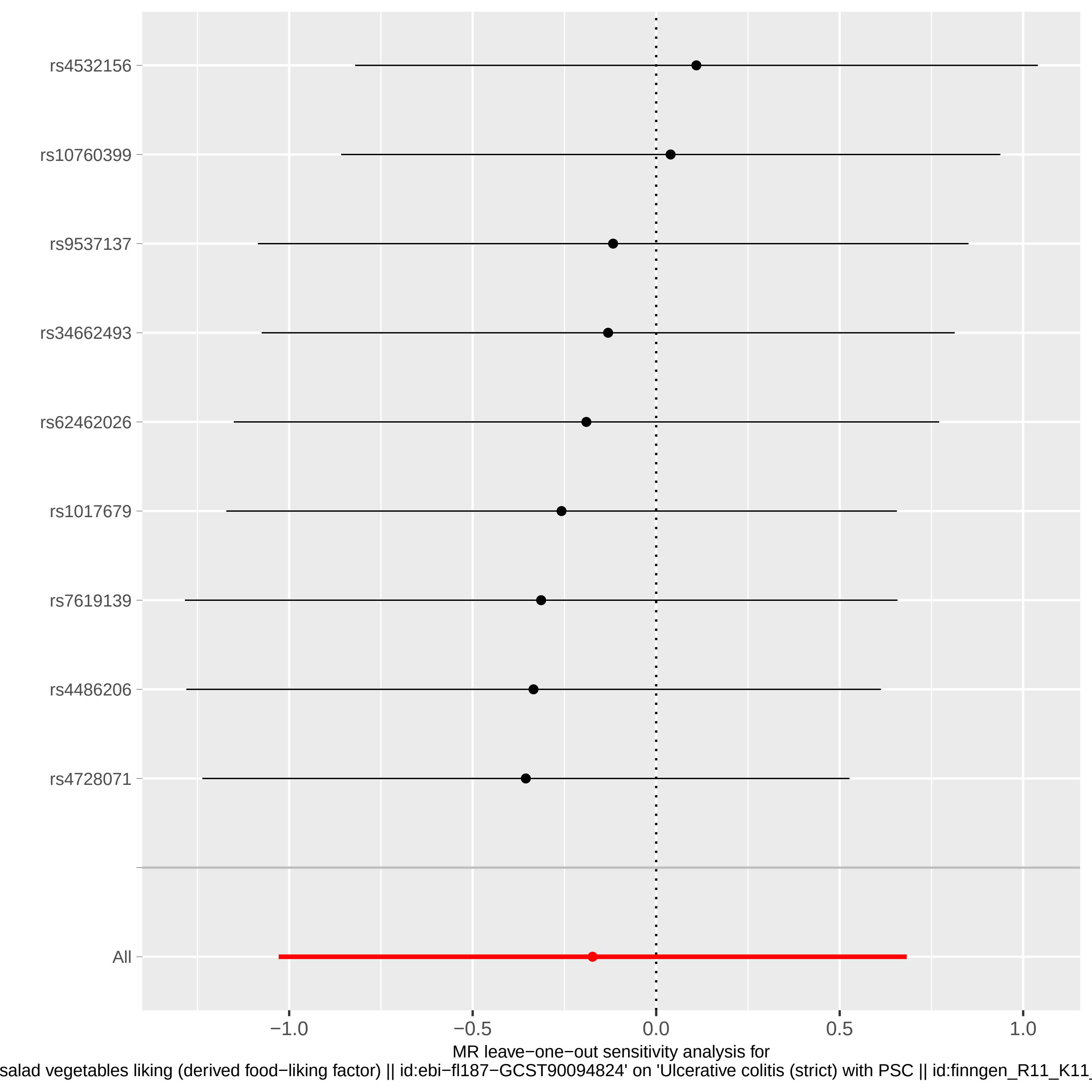
rs4532156
rs10760399
rs9537137
rs34662493
rs62462026
rs1017679
rs7619139
rs4486206
rs4728071
All
−1.0
−0.5
0.0
0.5
1.0
MR leave−one−out sensitivity analysis for
salad vegetables liking (derived food−liking factor) || id:ebi−fl187−GCST90094824' on 'Ulcerative colitis (strict) with PSC || id:finngen_R11_K11_

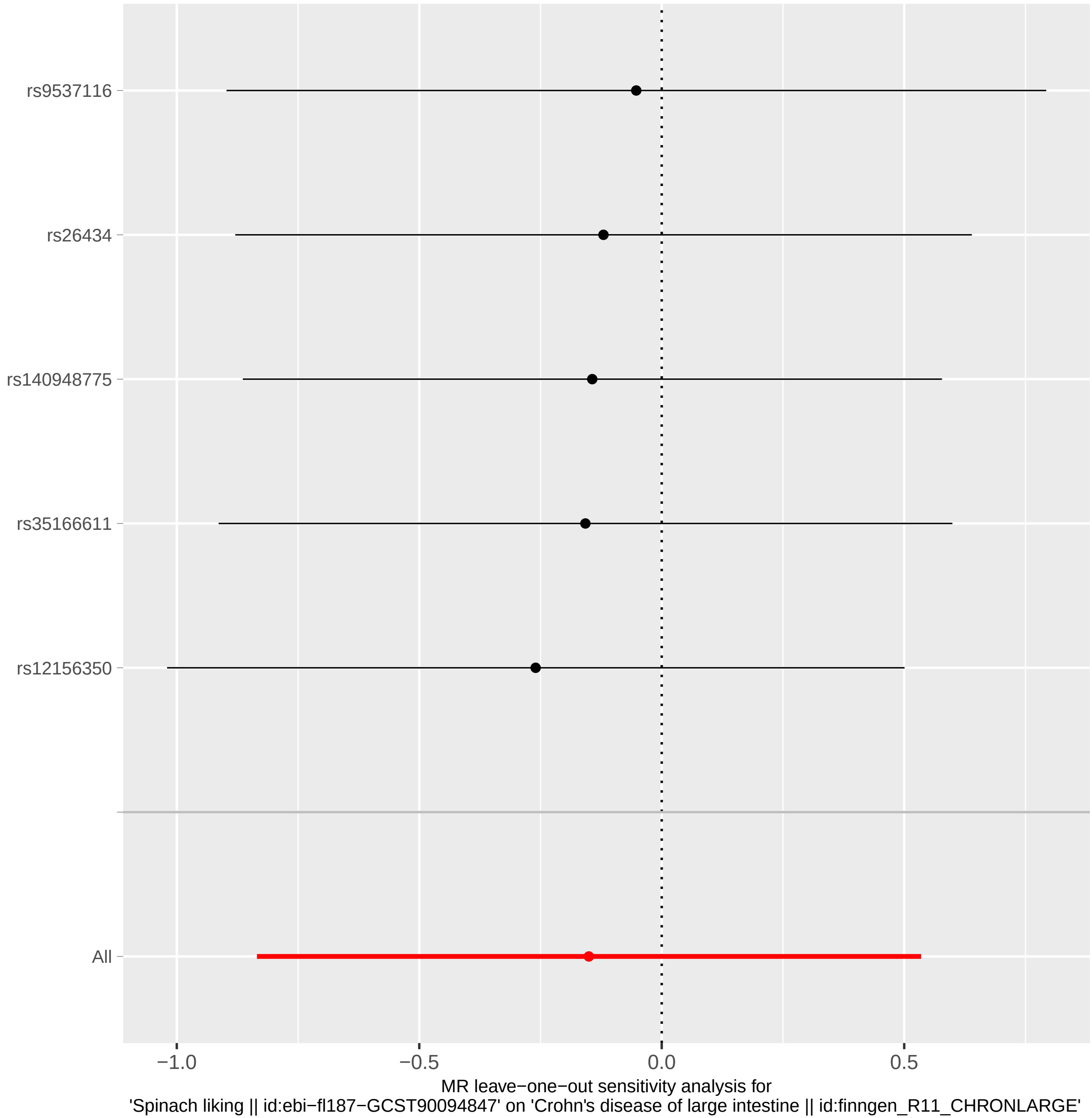
rs9537116
rs26434
rs140948775
rs35166611
rs12156350
All
−1.0
−0.5
0.0
0.5
MR leave−one−out sensitivity analysis for
'Spinach liking || id:ebi−fl187−GCST90094847' on 'Crohn's disease of large intestine || id:finngen_R11_CHRONLARGE'

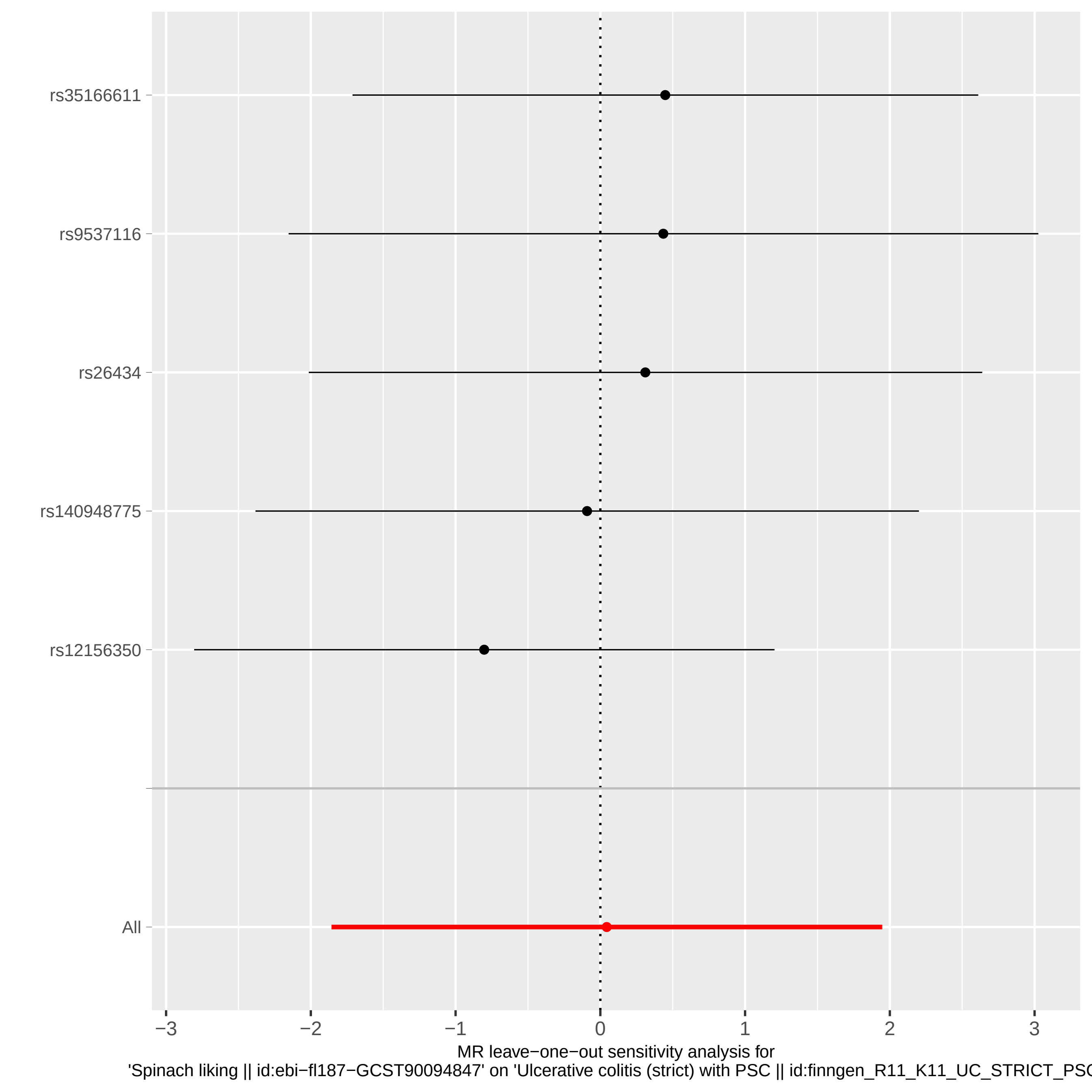
rs35166611
rs9537116
rs26434
rs140948775
rs12156350
All
−3
−2
−1
0
1
2
3
MR leave−one−out sensitivity analysis for
'Spinach liking || id:ebi−fl187−GCST90094847' on 'Ulcerative colitis (strict) with PSC || id:finngen_R11_K11_UC_STRICT_PSC

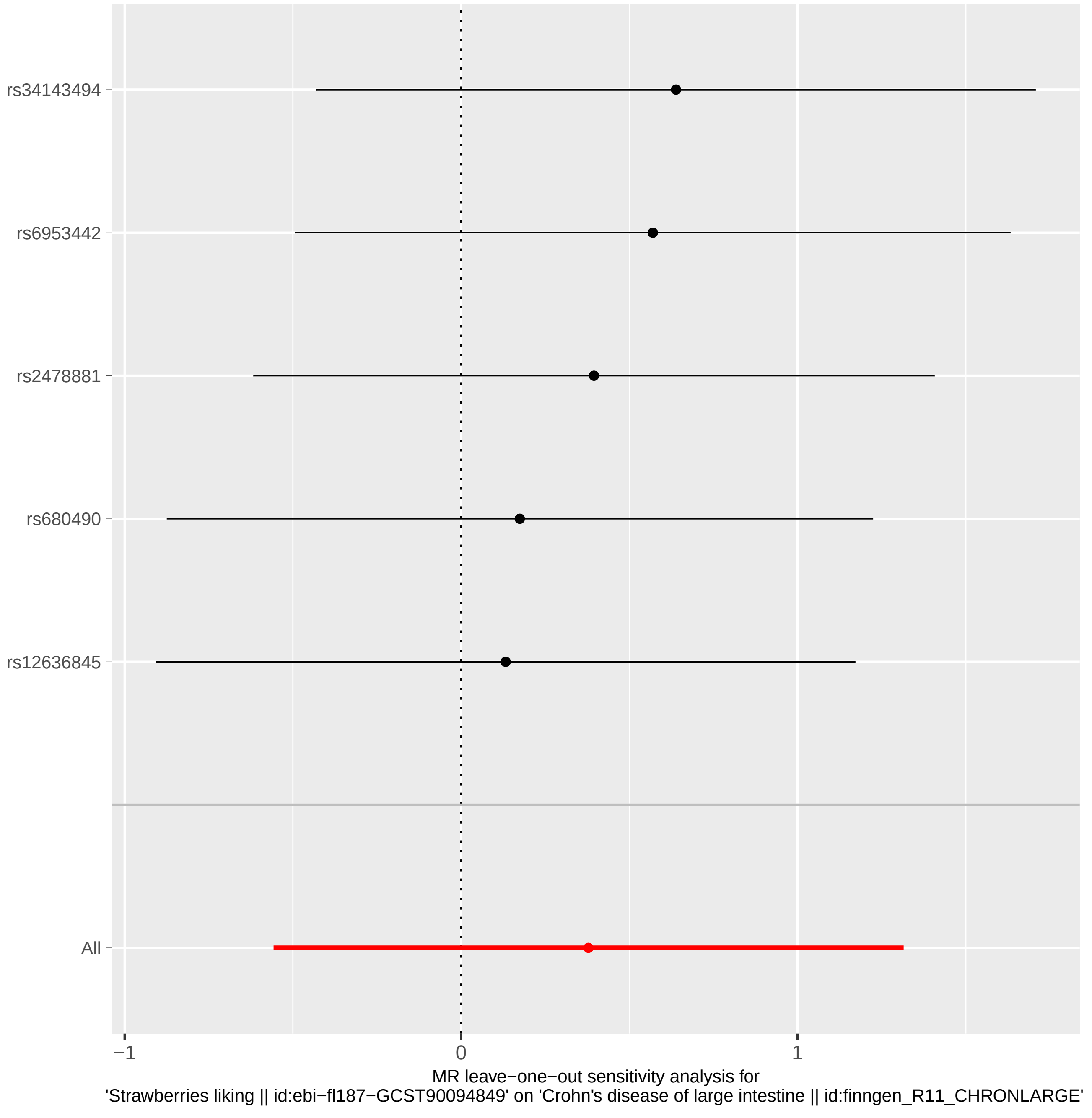
rs34143494
rs6953442
rs2478881
rs680490
rs12636845
All
−1
0
1
MR leave−one−out sensitivity analysis for
'Strawberries liking || id:ebi−fl187−GCST90094849' on 'Crohn's disease of large intestine || id:finngen_R11_CHRONLARGE'

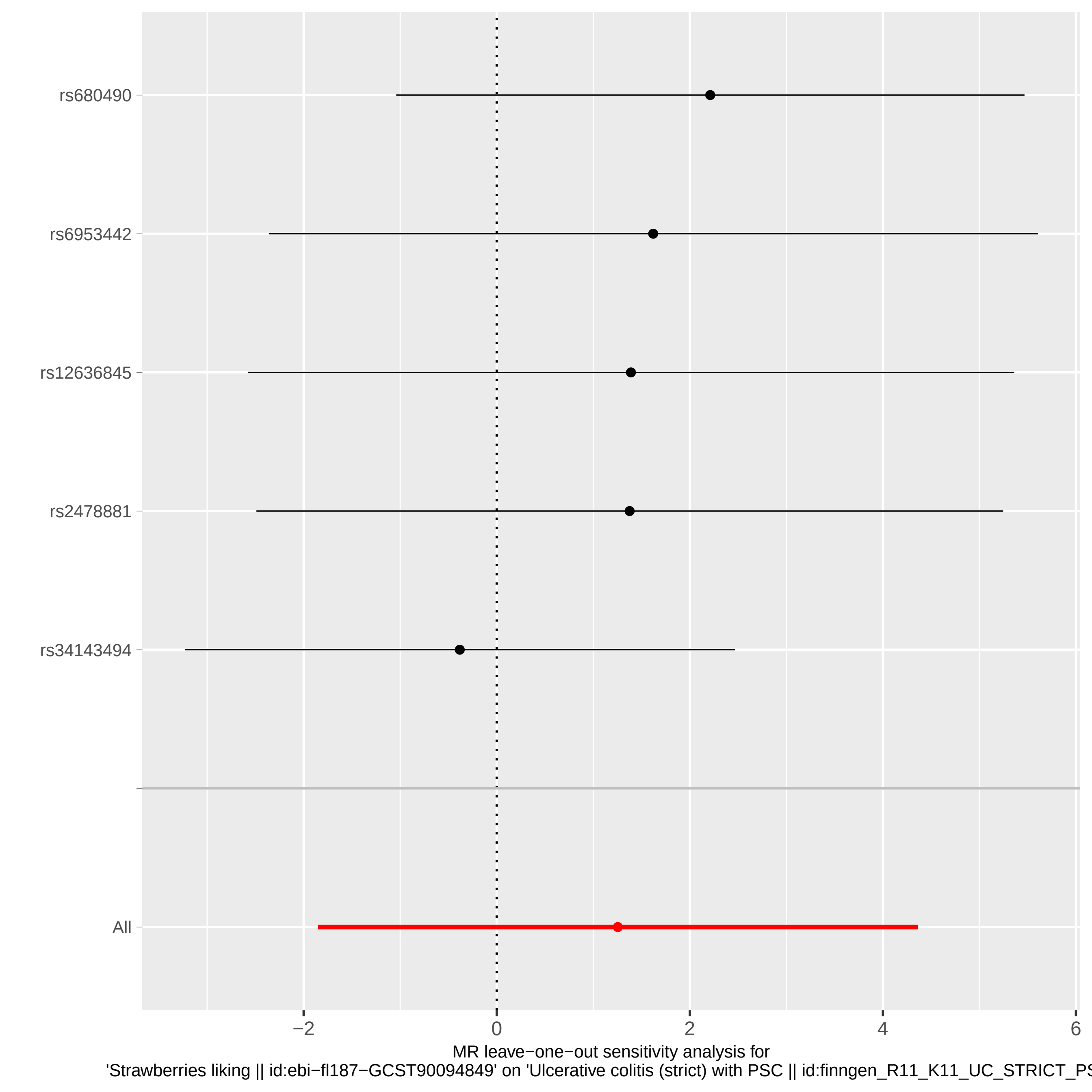
rs680490
rs6953442
rs12636845
rs2478881
rs34143494
All
−2
0
2
4
6
MR leave−one−out sensitivity analysis for
'Strawberries liking || id:ebi−fl187−GCST90094849' on 'Ulcerative colitis (strict) with PSC || id:finngen_R11_K11_UC_STRICT_PS

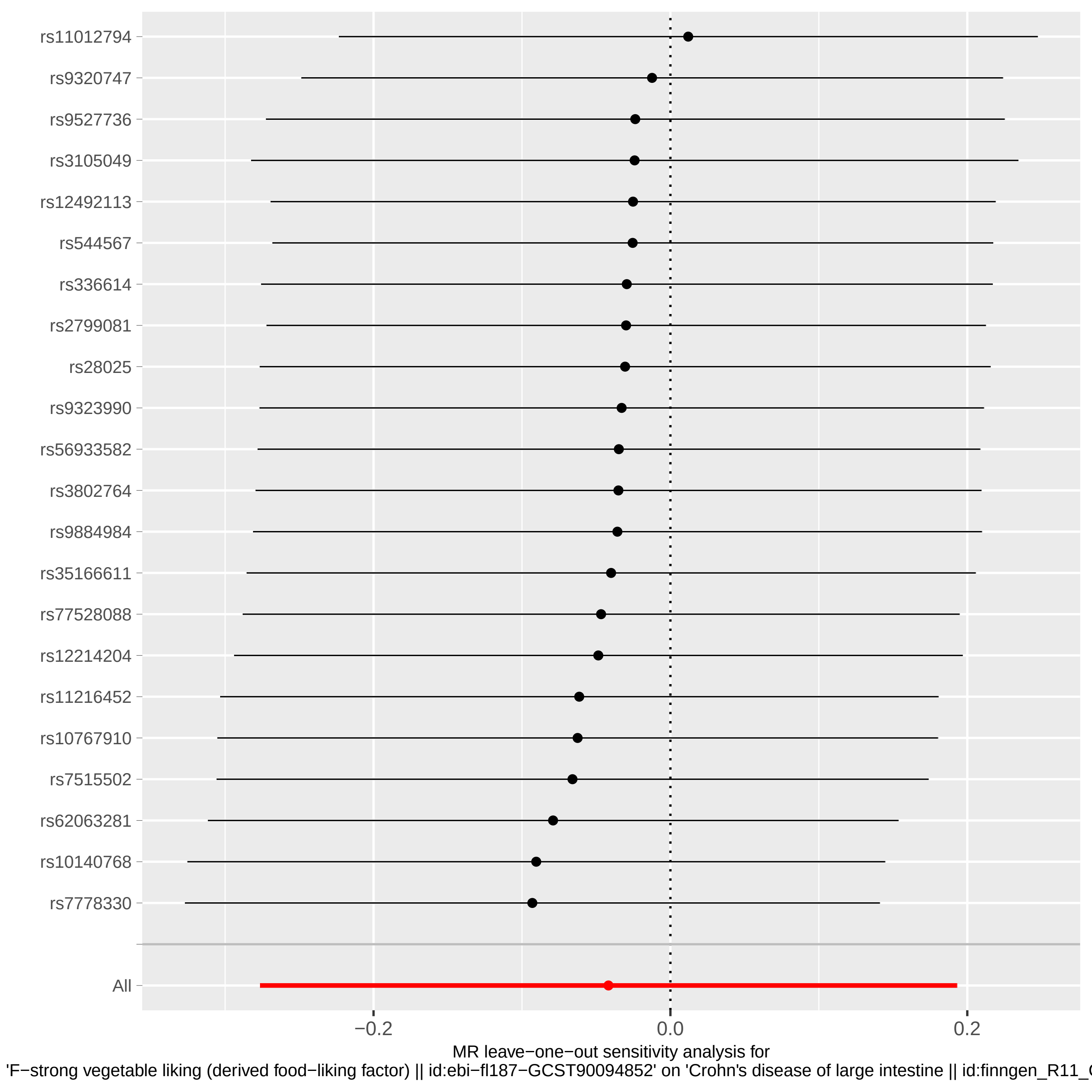
rs11012794
rs9320747
rs9527736
rs3105049
rs12492113
rs544567
rs336614
rs2799081
rs28025
rs9323990
rs56933582
rs3802764
rs9884984
rs35166611
rs77528088
rs12214204
rs11216452
rs10767910
rs7515502
rs62063281
rs10140768
rs7778330
All
−0.2
0.0
0.2
MR leave−one−out sensitivity analysis for
'F−strong vegetable liking (derived food−liking factor) || id:ebi−fl187−GCST90094852' on 'Crohn's disease of large intestine || id:finngen_R11_

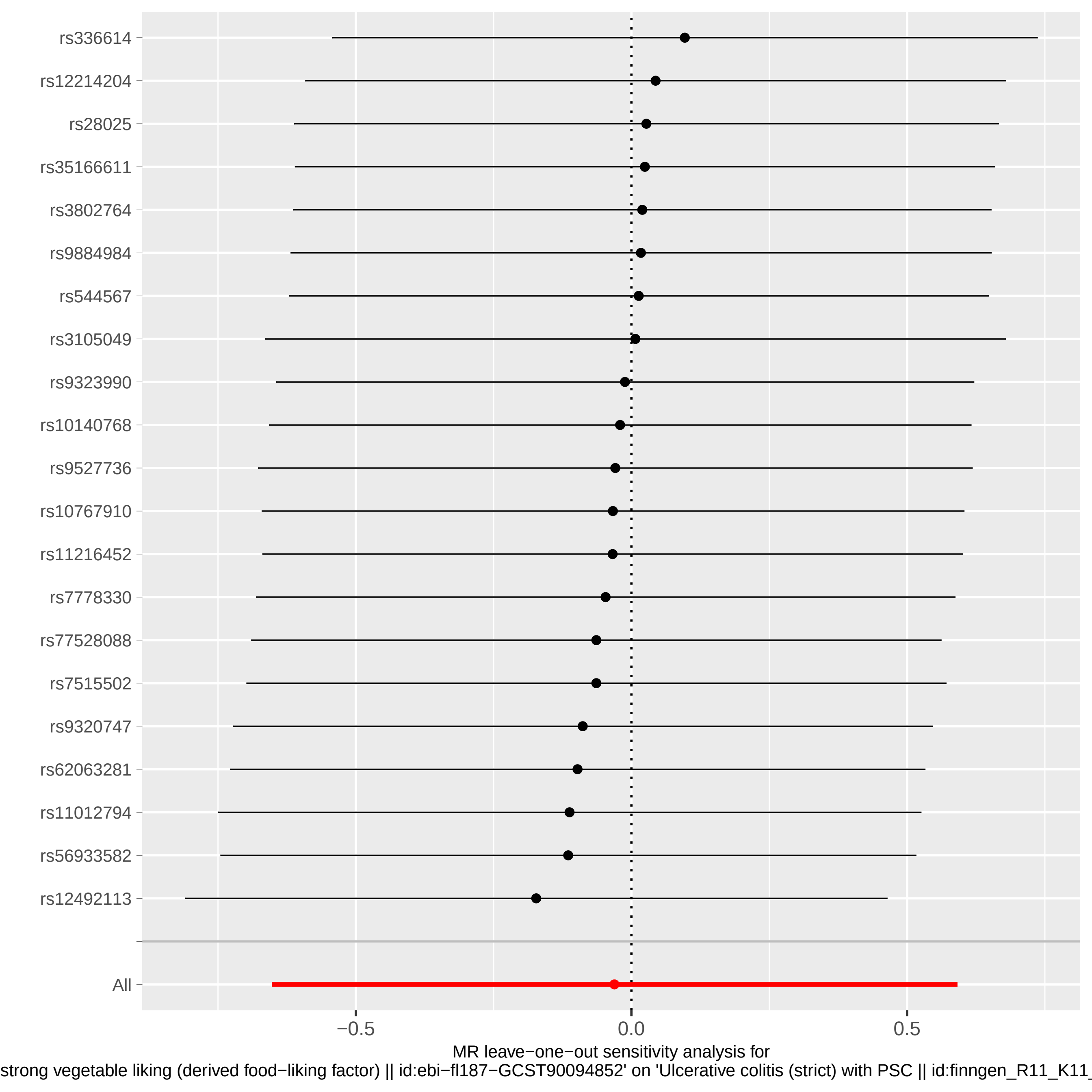
rs336614
rs12214204
rs28025
rs35166611
rs3802764
rs9884984
rs544567
rs3105049
rs9323990
rs10140768
rs9527736
rs10767910
rs11216452
rs7778330
rs77528088
rs7515502
rs9320747
rs62063281
rs11012794
rs56933582
rs12492113
All
−0.5
0.0
0.5
MR leave−one−out sensitivity analysis for
strong vegetable liking (derived food−liking factor) || id:ebi−fl187−GCST90094852' on 'Ulcerative colitis (strict) with PSC || id:finngen_R11_K11_

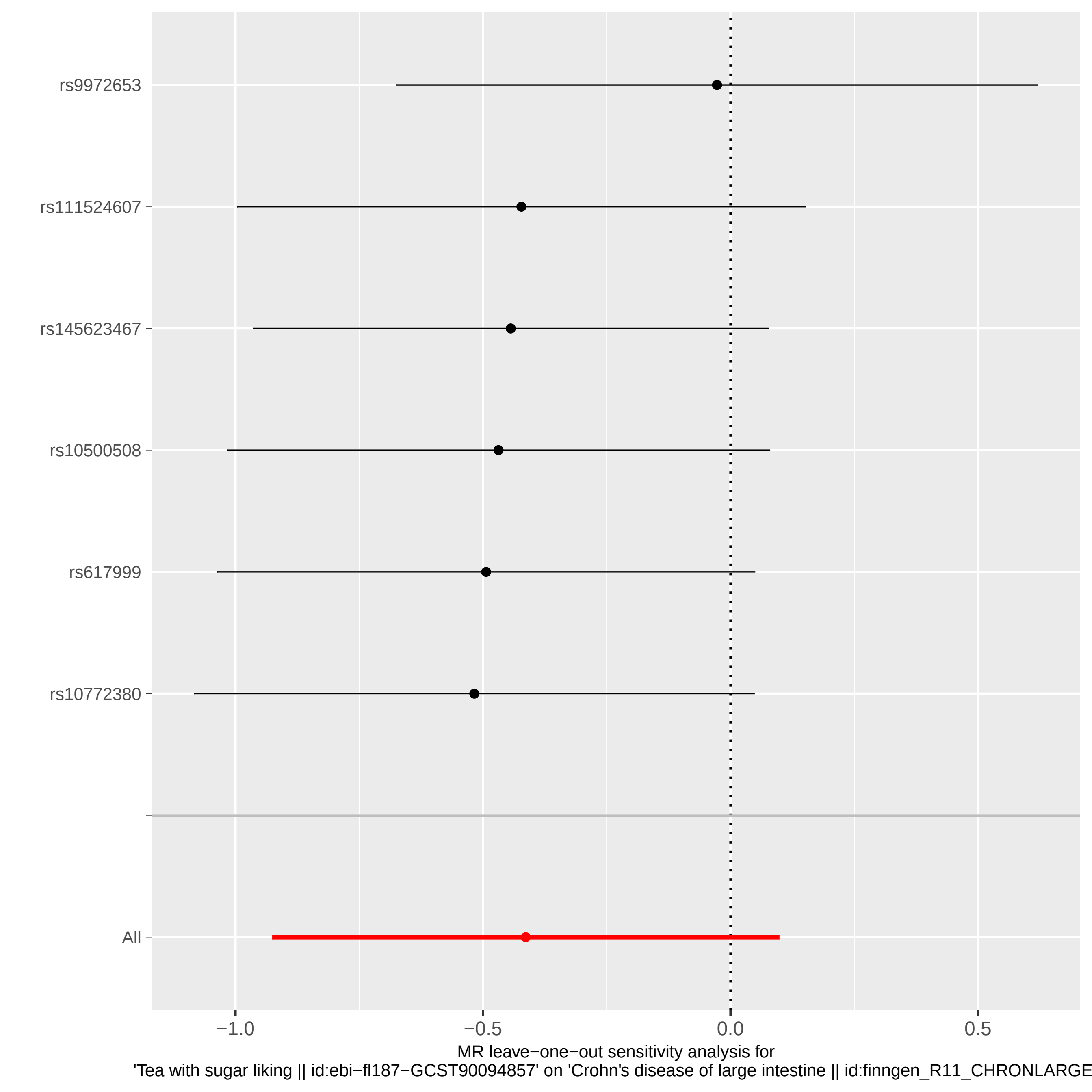

rs9972653
rs111524607
rs145623467
rs10500508
rs617999
rs10772380
All
−1.0
−0.5
0.0
0.5
MR leave−one−out sensitivity analysis for
'Tea with sugar liking || id:ebi−fl187−GCST90094857' on 'Crohn's disease of large intestine || id:finngen_R11_CHRONLARGE

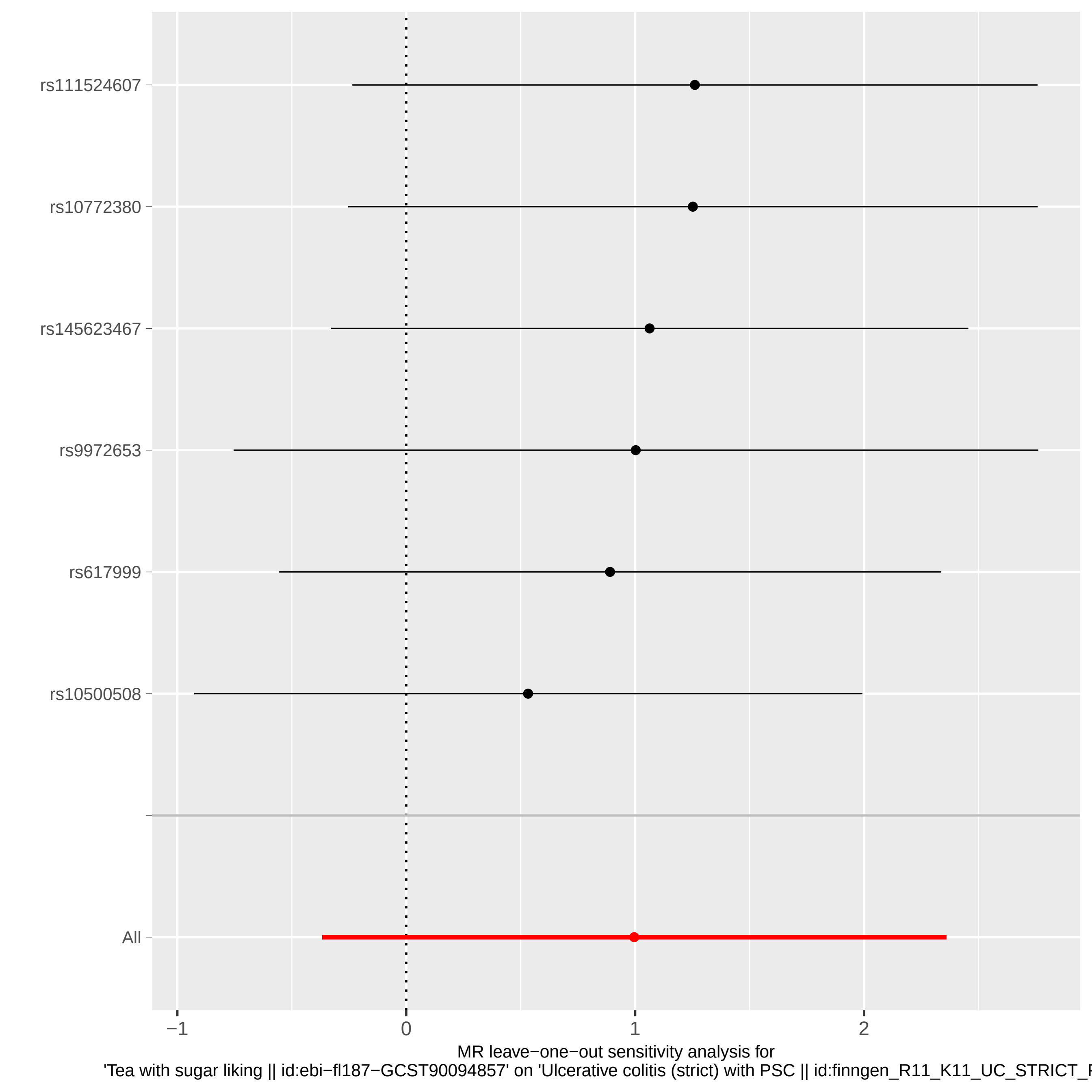
rs111524607
rs10772380
rs145623467
rs9972653
rs617999
rs10500508
All
−1
0
1
2
MR leave−one−out sensitivity analysis for
'Tea with sugar liking || id:ebi−fl187−GCST90094857' on 'Ulcerative colitis (strict) with PSC || id:finngen_R11_K11_UC_STRICT_F

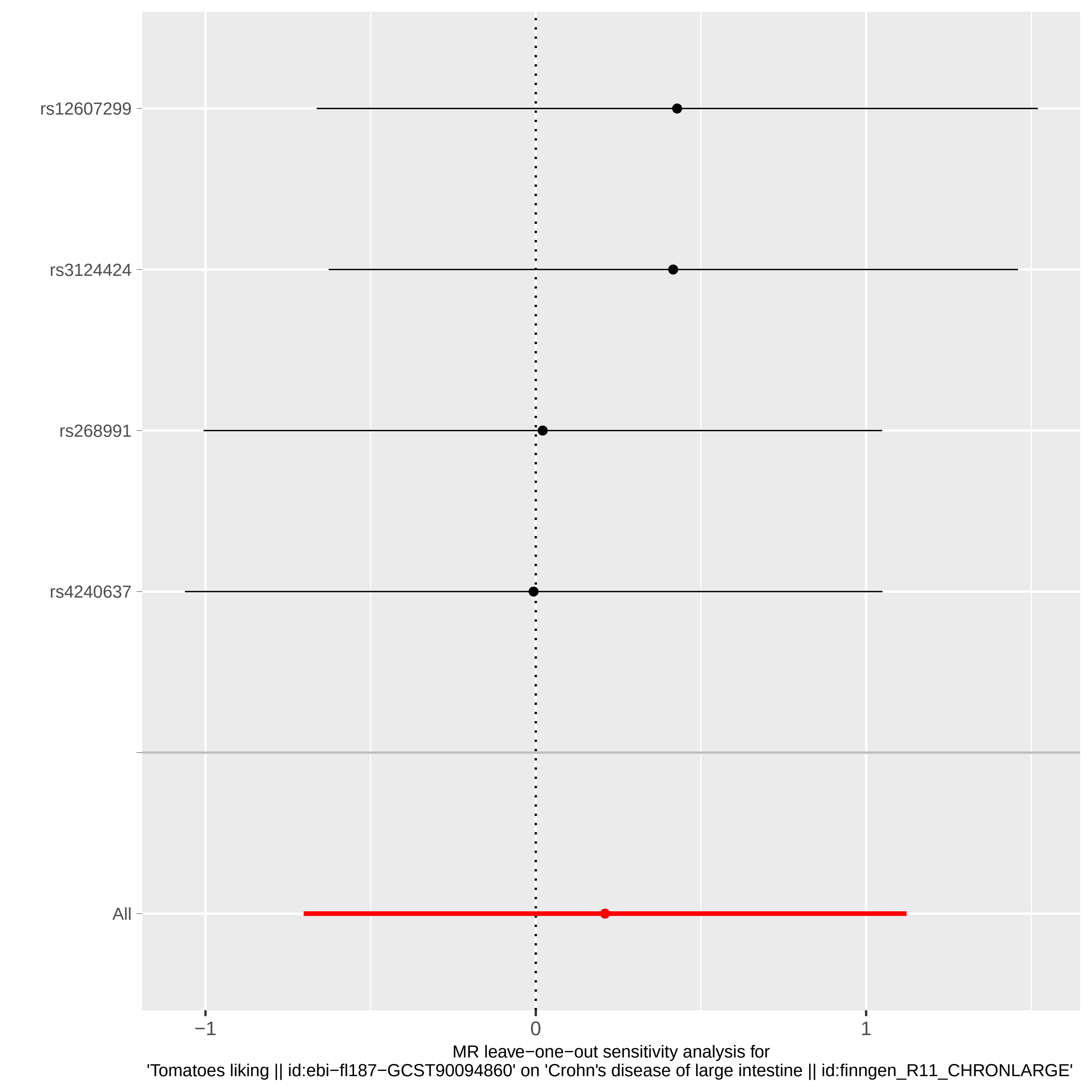

rs12607299
rs3124424
rs268991
rs4240637
All
−1
0
1
MR leave−one−out sensitivity analysis for
'Tomatoes liking || id:ebi−fl187−GCST90094860' on 'Crohn's disease of large intestine || id:finngen_R11_CHRONLARGE'

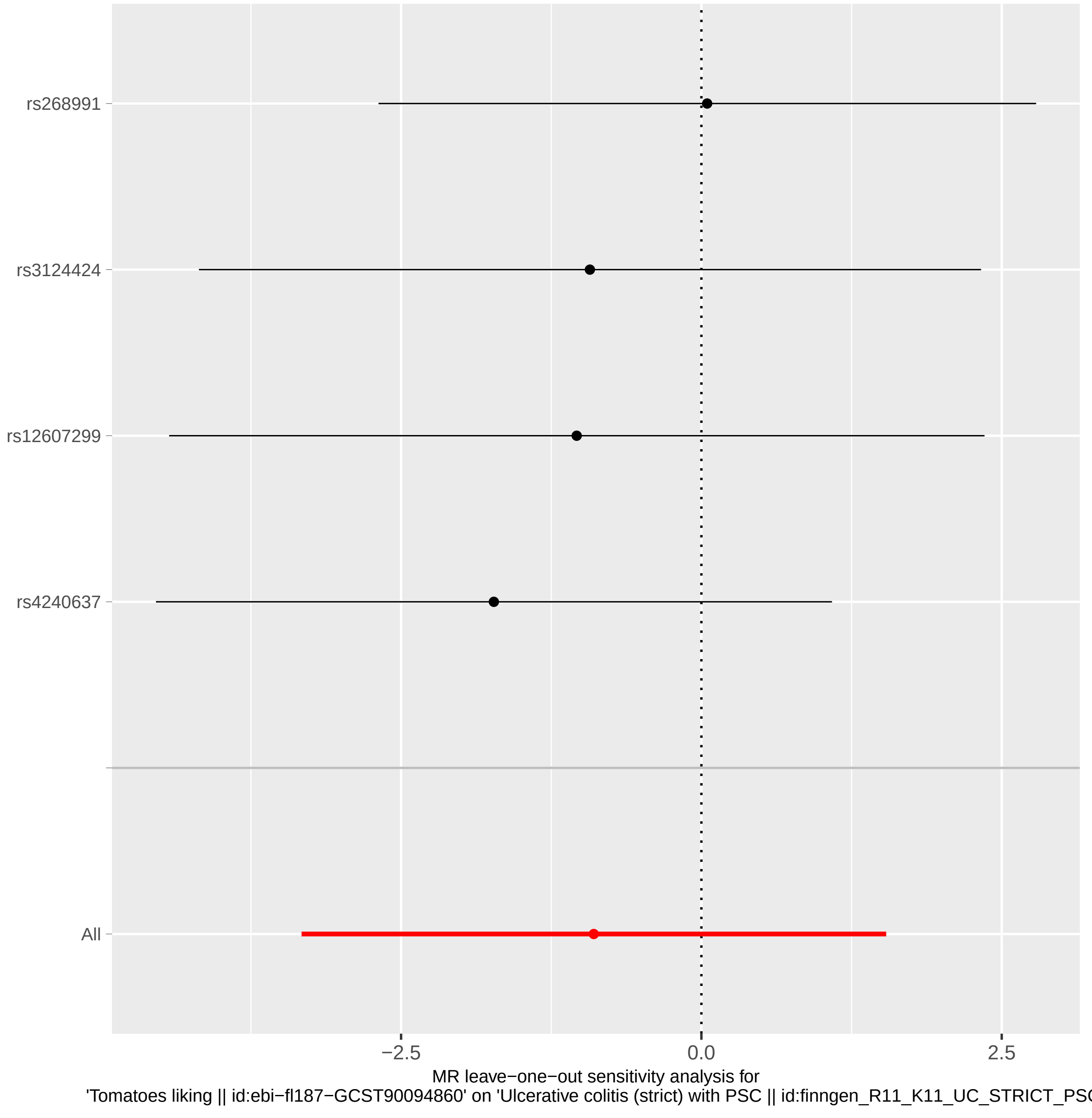
rs268991
rs3124424
rs12607299
rs4240637
All
−2.5
0.0
2.5
MR leave−one−out sensitivity analysis for
'Tomatoes liking || id:ebi−fl187−GCST90094860' on 'Ulcerative colitis (strict) with PSC || id:finngen_R11_K11_UC_STRICT_PSC

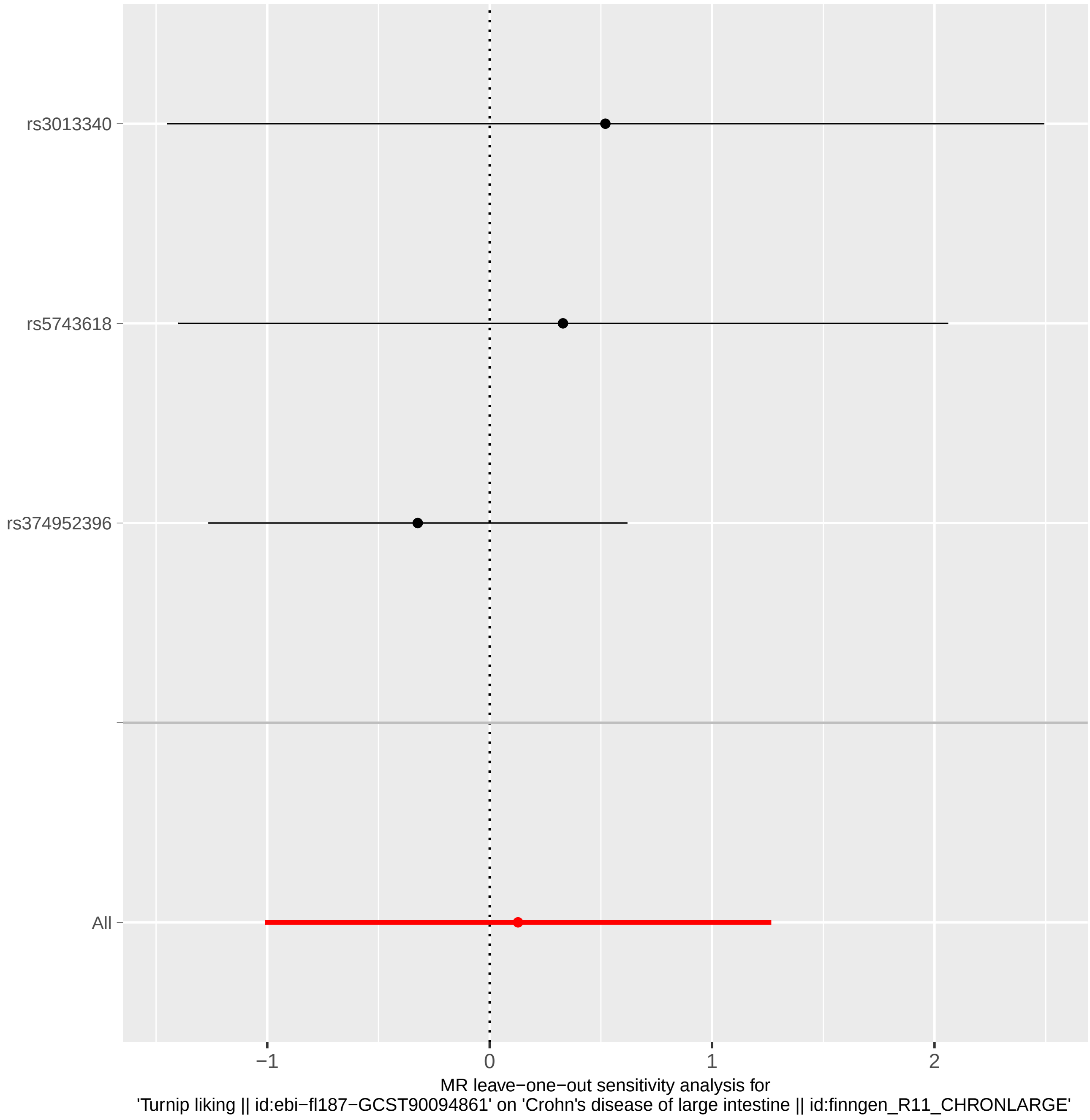

rs3013340
rs5743618
rs374952396
All
−1
0
1
2
MR leave−one−out sensitivity analysis for
'Turnip liking || id:ebi−fl187−GCST90094861' on 'Crohn's disease of large intestine || id:finngen_R11_CHRONLARGE'

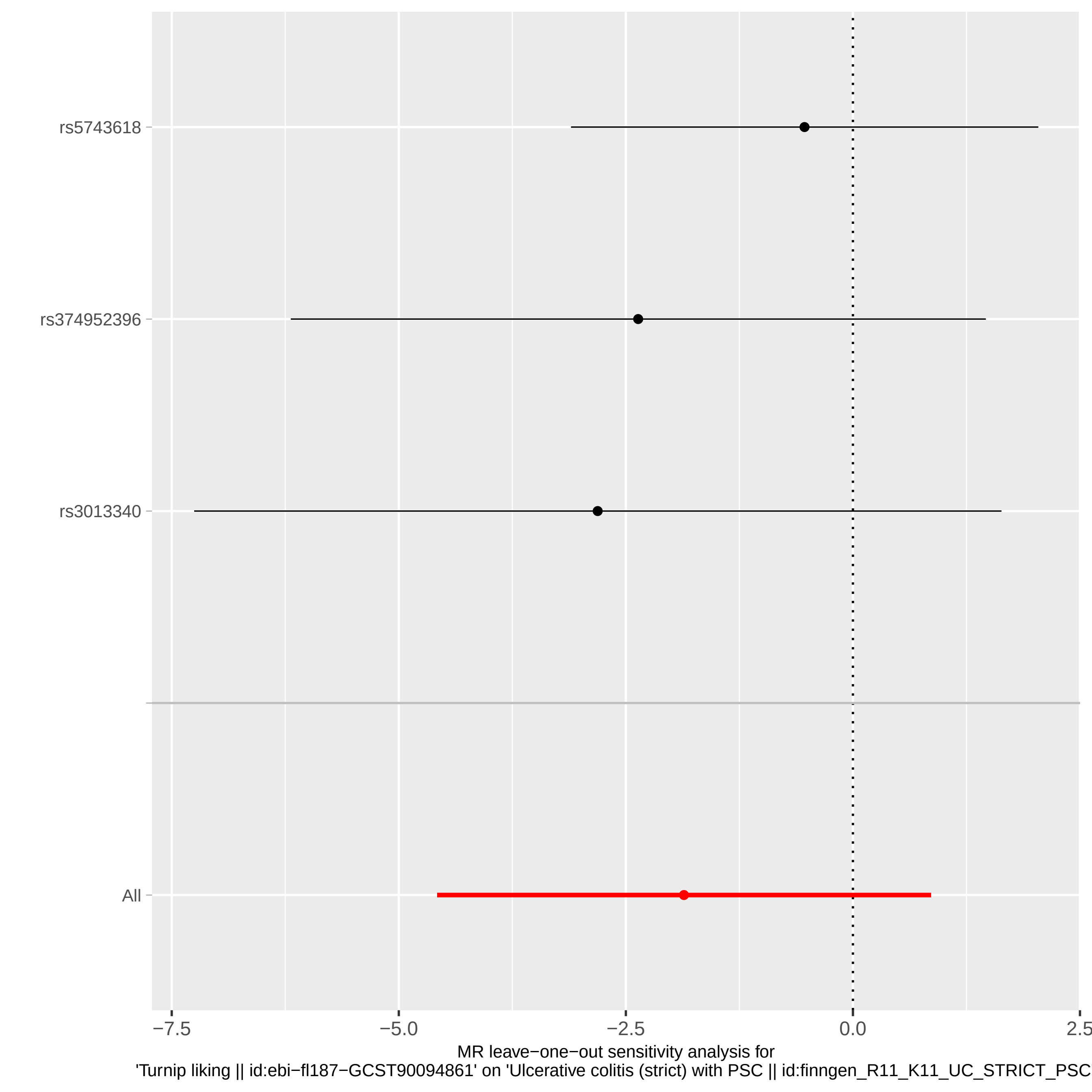
rs5743618
rs374952396
rs3013340
All
−7.5
−5.0
−2.5
0.0
2.5
MR leave−one−out sensitivity analysis for
'Turnip liking || id:ebi−fl187−GCST90094861' on 'Ulcerative colitis (strict) with PSC || id:finngen_R11_K11_UC_STRICT_PSC

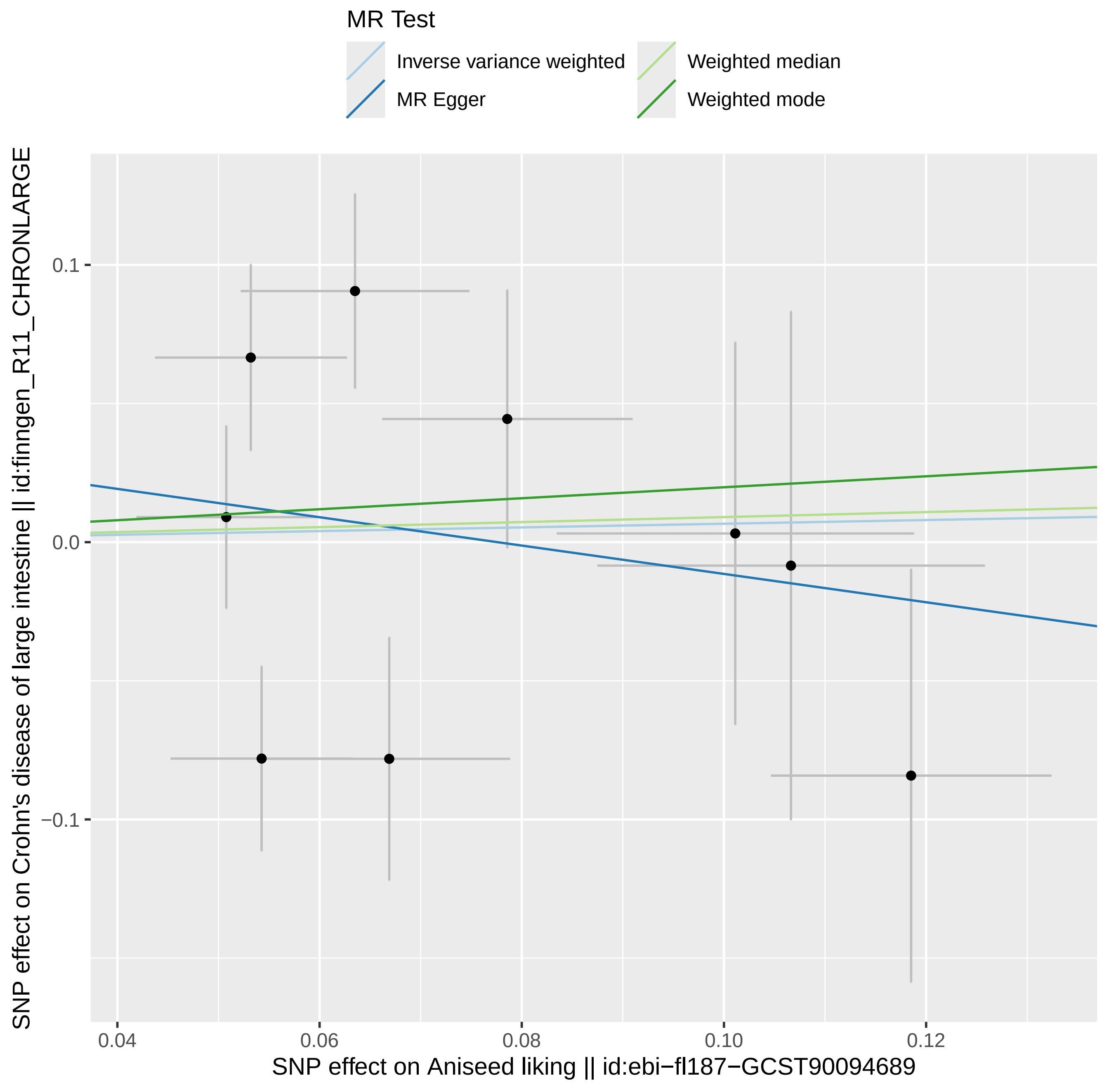

MR Test
Inverse variance weighted
MR Egger
Weighted median
Weighted mode
0.1
0.0
−0.1
0.04
0.06
0.08
0.10
0.12
SNP effect on Aniseed liking || id:ebi−fl187−GCST90094689
SNP effect on Crohn's disease of large intestine || id:finngen_R11_CHRONLARGE

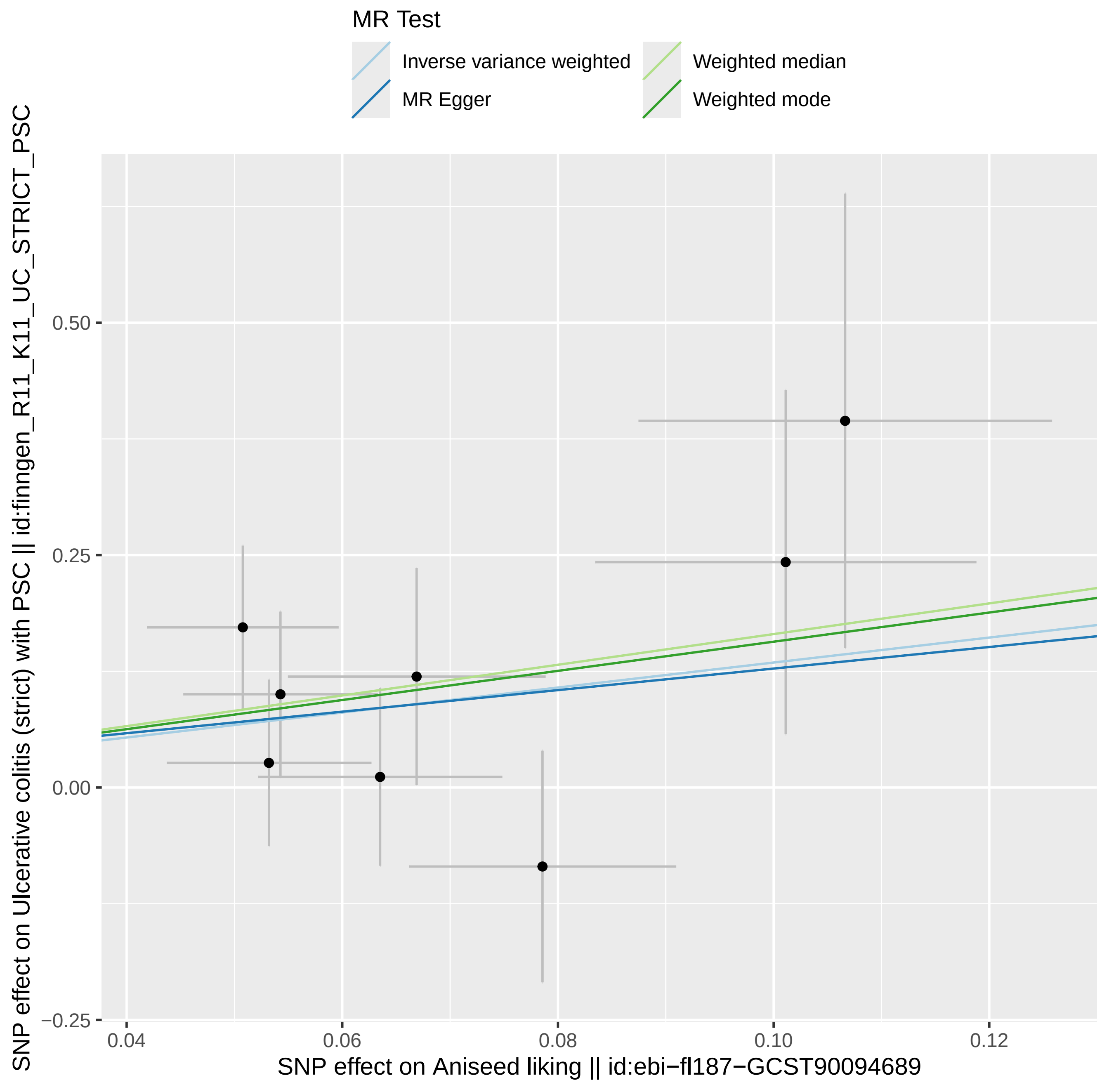

MR Test
Inverse variance weighted
MR Egger
Weighted median
Weighted mode
0.50
0.25
0.00
−0.25
0.04
0.06
0.08
0.10
0.12
SNP effect on Aniseed liking || id:ebi−fl187−GCST90094689
SNP effect on Ulcerative colitis (strict) with PSC || id:finngen_R11_K11_UC_STRICT_PSC

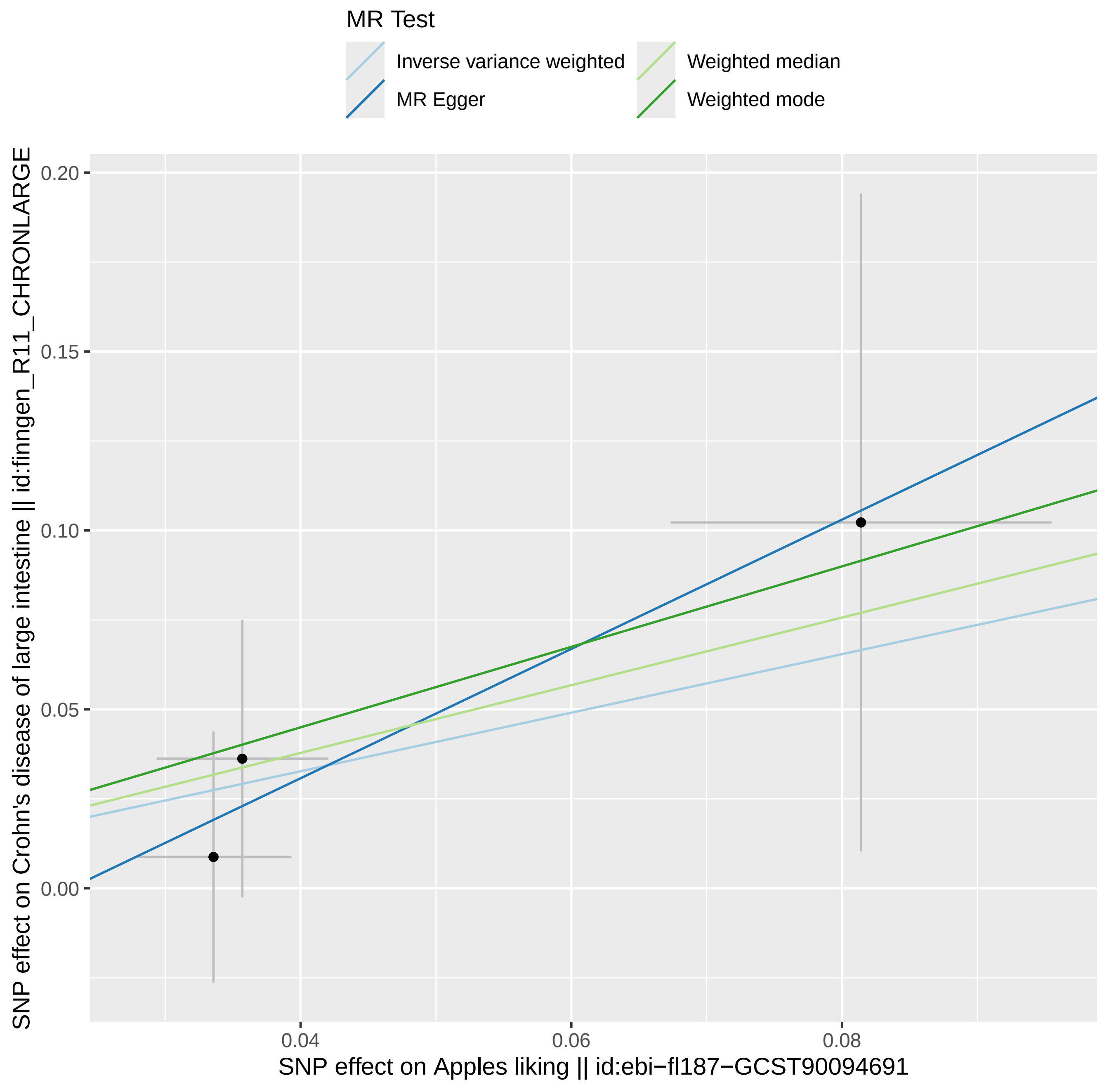

MR Test
Inverse variance weighted
MR Egger
Weighted median
Weighted mode
SNP effect on Crohn's disease of large intestine || id:finngen_R11_CHRONLARGE
0.20
0.15
0.10
0.05
0.00
0.04
0.06
0.08
SNP effect on Apples liking || id:ebi−fl187−GCST90094691

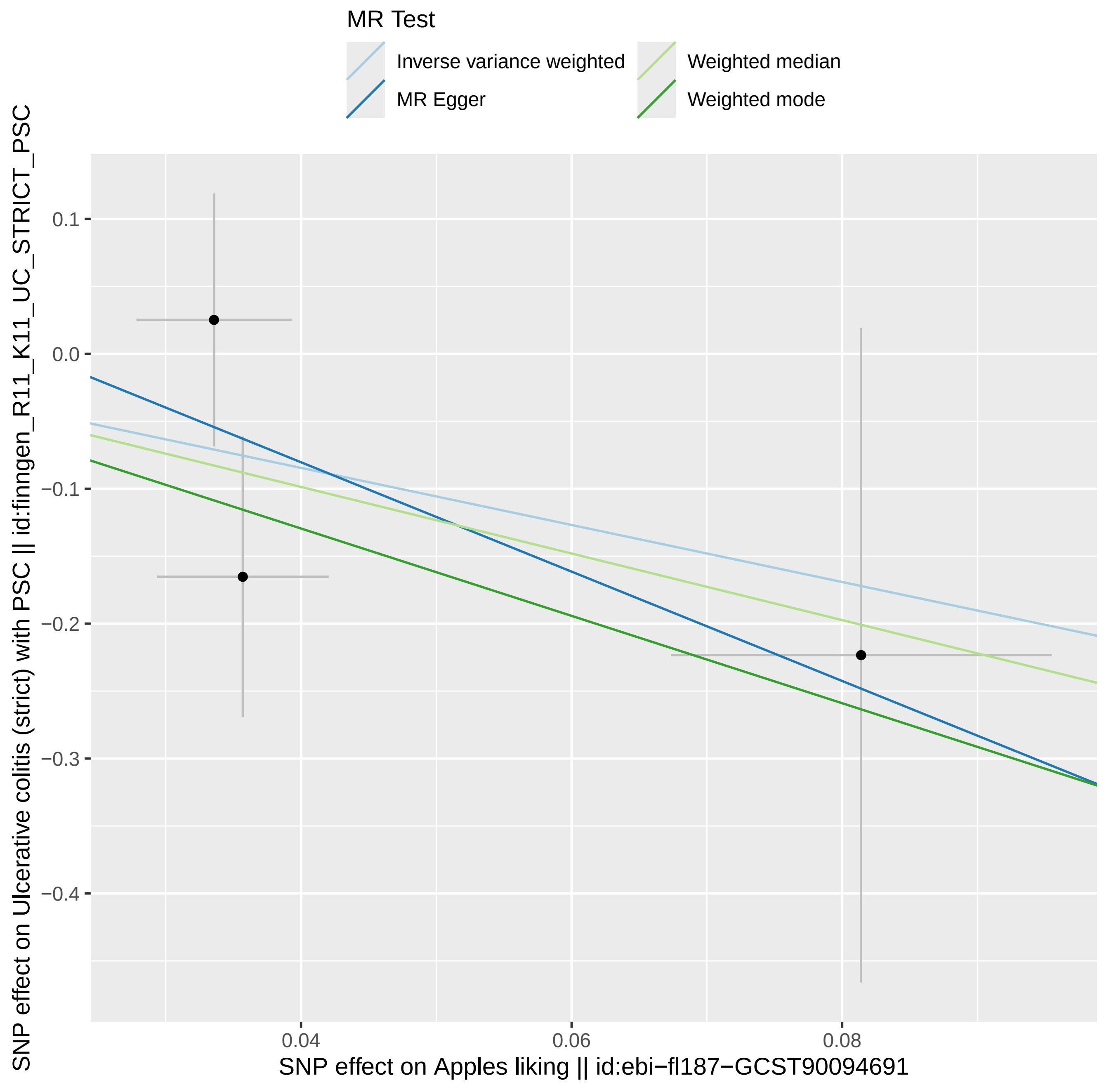
MR Test
Inverse variance weighted
MR Egger
Weighted median
Weighted mode
0.1
0.0
−0.1
−0.2
−0.3
−0.4
0.04
0.06
0.08
SNP effect on Ulcerative colitis (strict) with PSC || id:finngen_R11_K11_UC_STRICT_PSC
SNP effect on Apples liking || id:ebi−fl187−GCST90094691

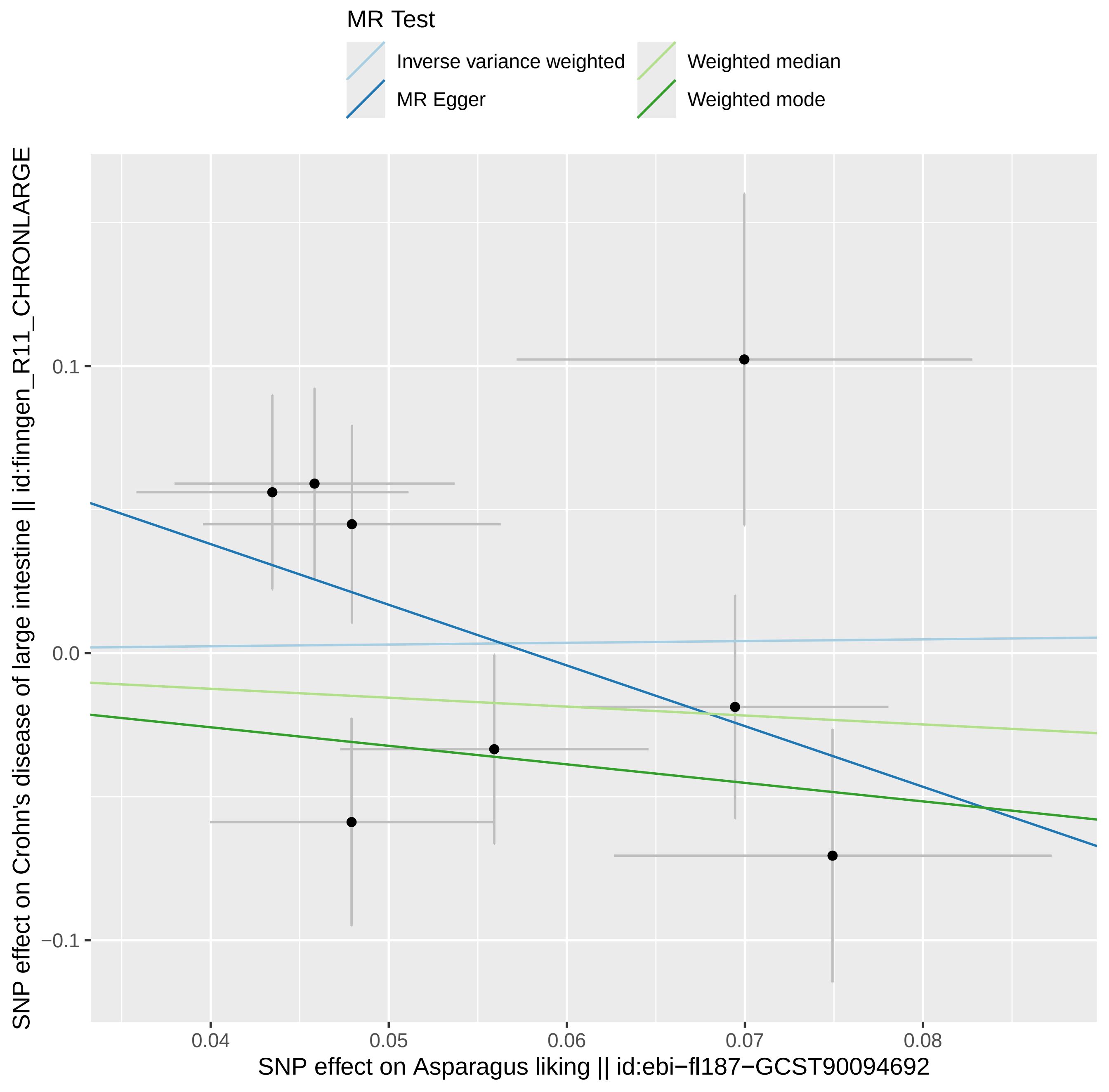

MR Test
Inverse variance weighted
MR Egger
Weighted median
Weighted mode
SNP effect on Crohn's disease of large intestine || id:finngen_R11_CHRONLARGE
0.1
0.0
−0.1
0.04
0.05
0.06
0.07
0.08
SNP effect on Asparagus liking || id:ebi−fl187−GCST90094692

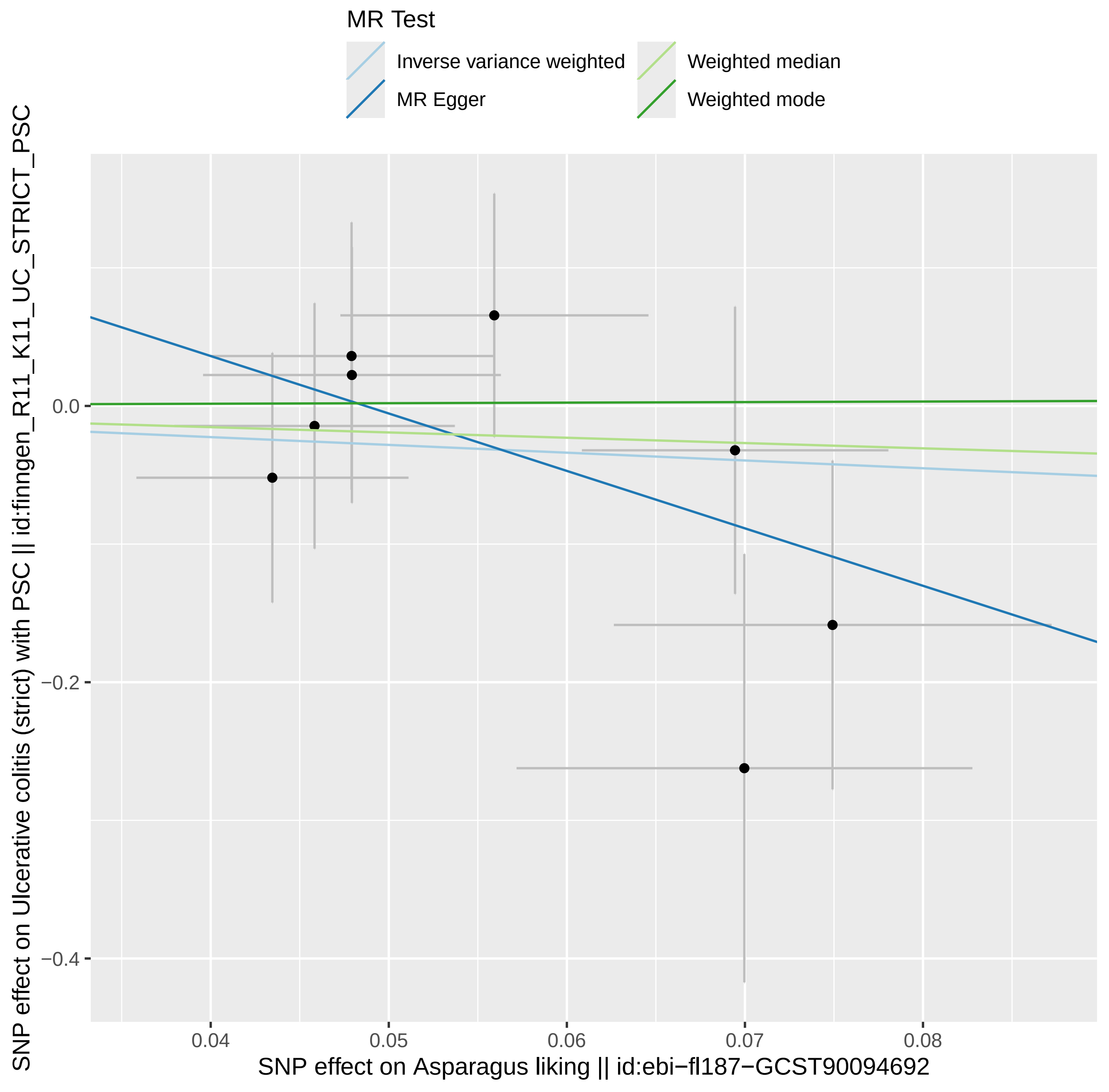
MR Test
Inverse variance weighted
MR Egger
Weighted median
Weighted mode
0.0
−0.2
−0.4
0.04
0.05
0.06
0.07
0.08
SNP effect on Asparagus liking || id:ebi−fl187−GCST90094692
SNP effect on Ulcerative colitis (strict) with PSC || id:finngen_R11_K11_UC_STRICT_PSC

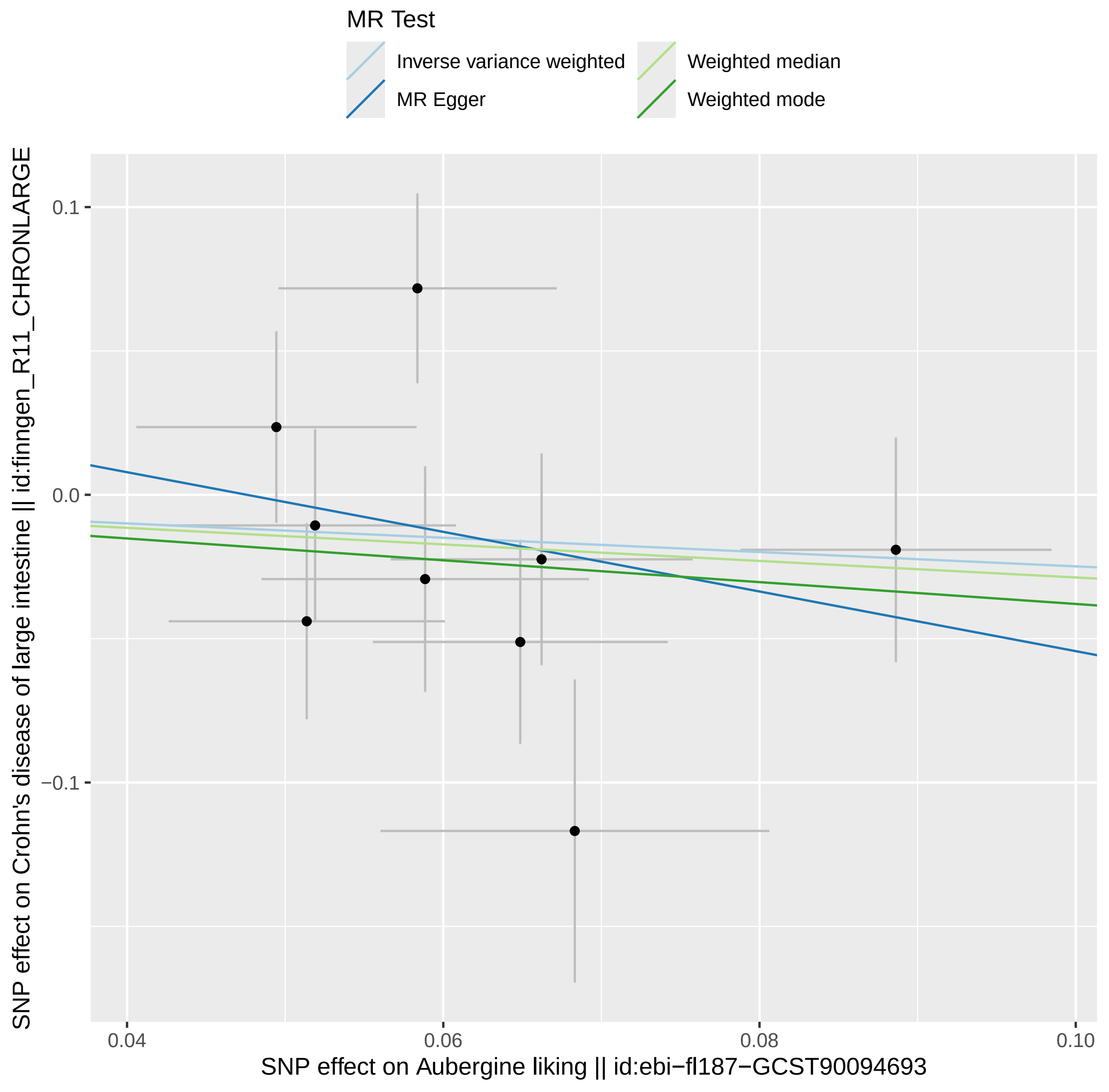
MR Test
Inverse variance weighted
MR Egger
Weighted median
Weighted mode
0.1
0.0
−0.1
0.04
0.06
0.08
0.10
SNP effect on Crohn's disease of large intestine || id:finngen_R11_CHRONLARGE
SNP effect on Aubergine liking || id:ebi−fl187−GCST90094693

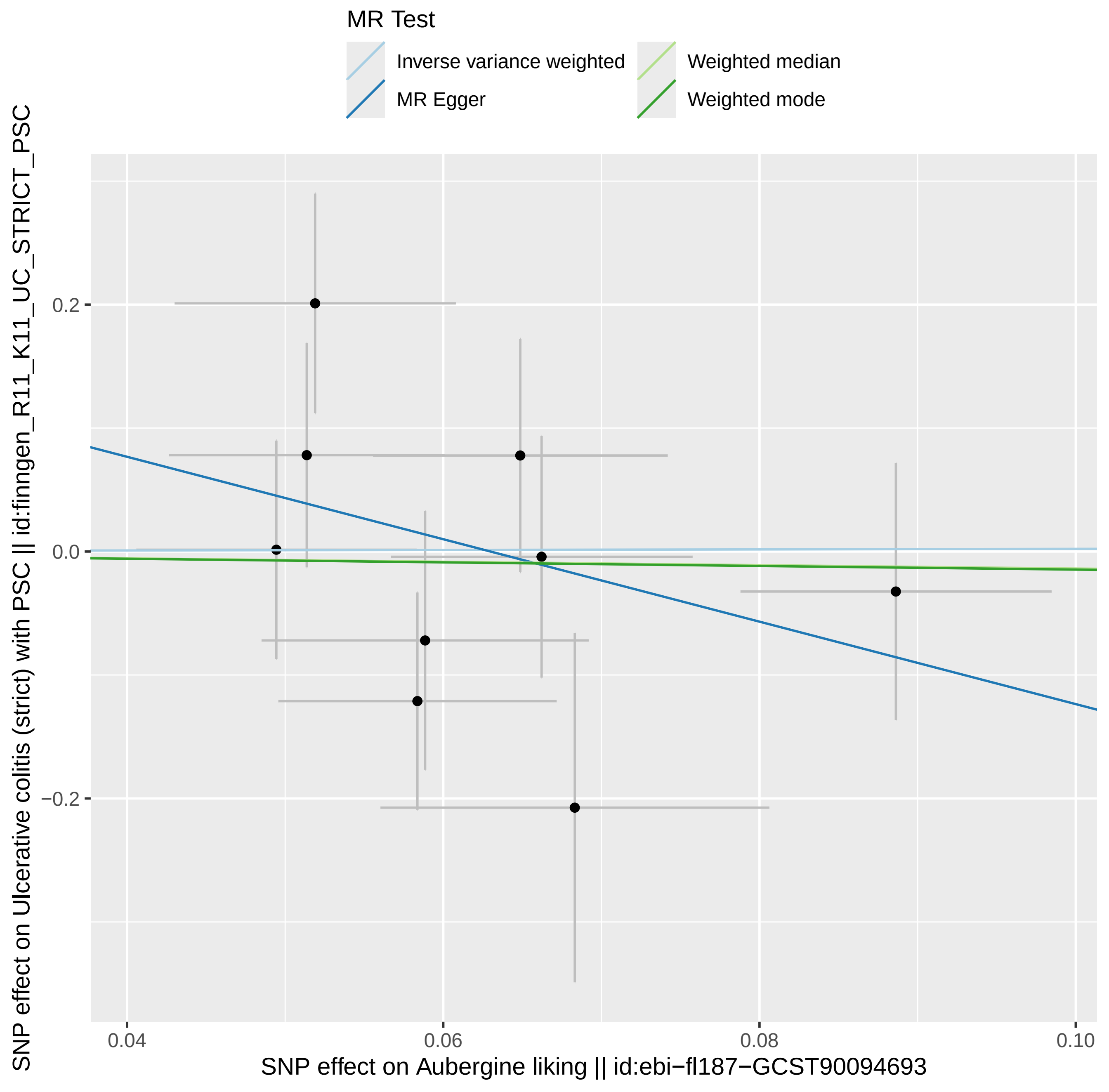

MR Test
Inverse variance weighted
MR Egger
Weighted median
Weighted mode
0.2
0.0
−0.2
0.04
0.06
0.08
0.10
SNP effect on Ulcerative colitis (strict) with PSC || id:finngen_R11_K11_UC_STRICT_PSC
SNP effect on Aubergine liking || id:ebi−fI187−GCST90094693

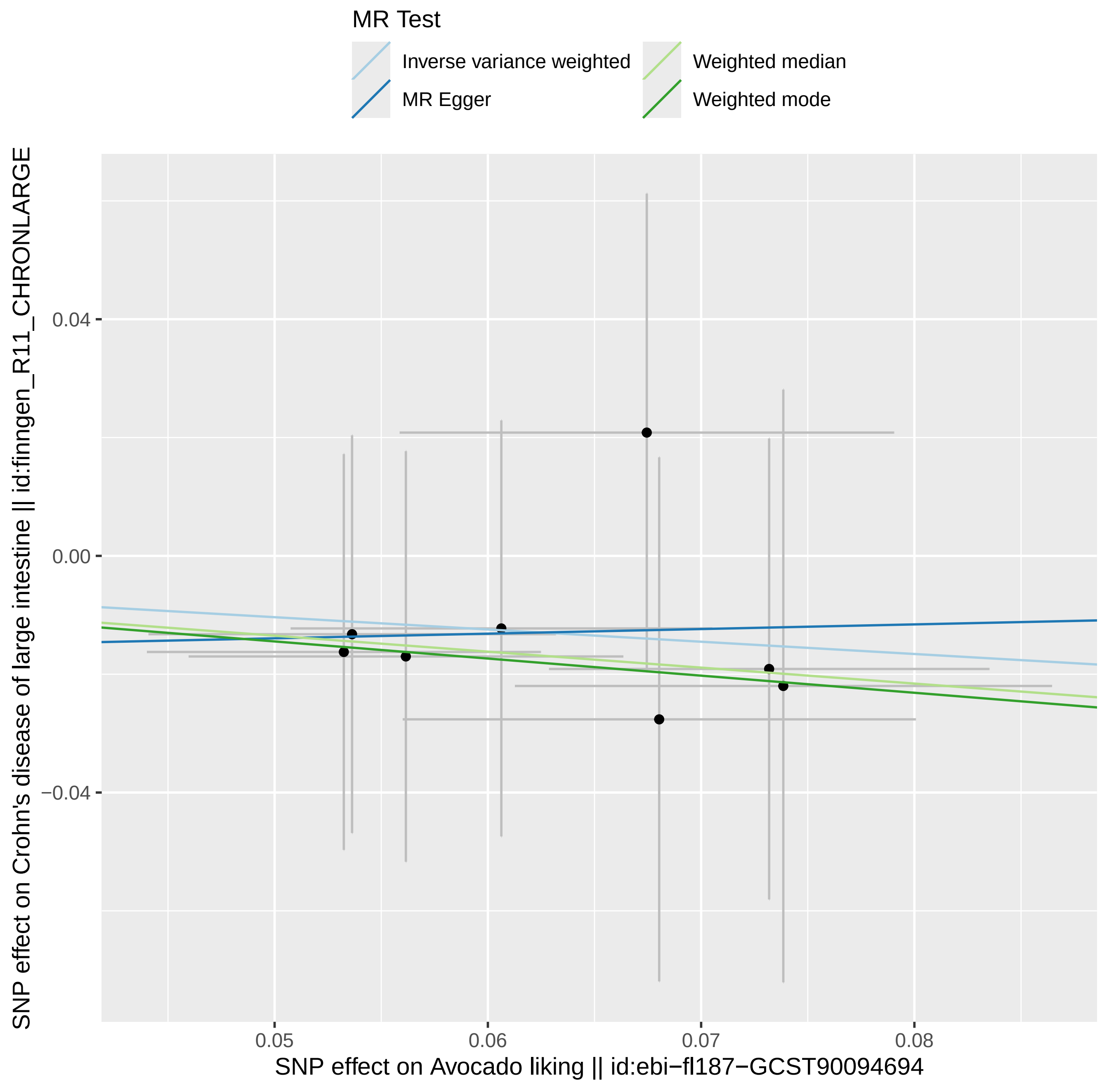
MR Test
Inverse variance weighted
MR Egger
Weighted median
Weighted mode
SNP effect on Crohn's disease of large intestine || id:finngen_R11_CHRONLARGE
0.04
0.00
−0.04
0.05
0.06
0.07
0.08
SNP effect on Avocado liking || id:ebi−fl187−GCST90094694

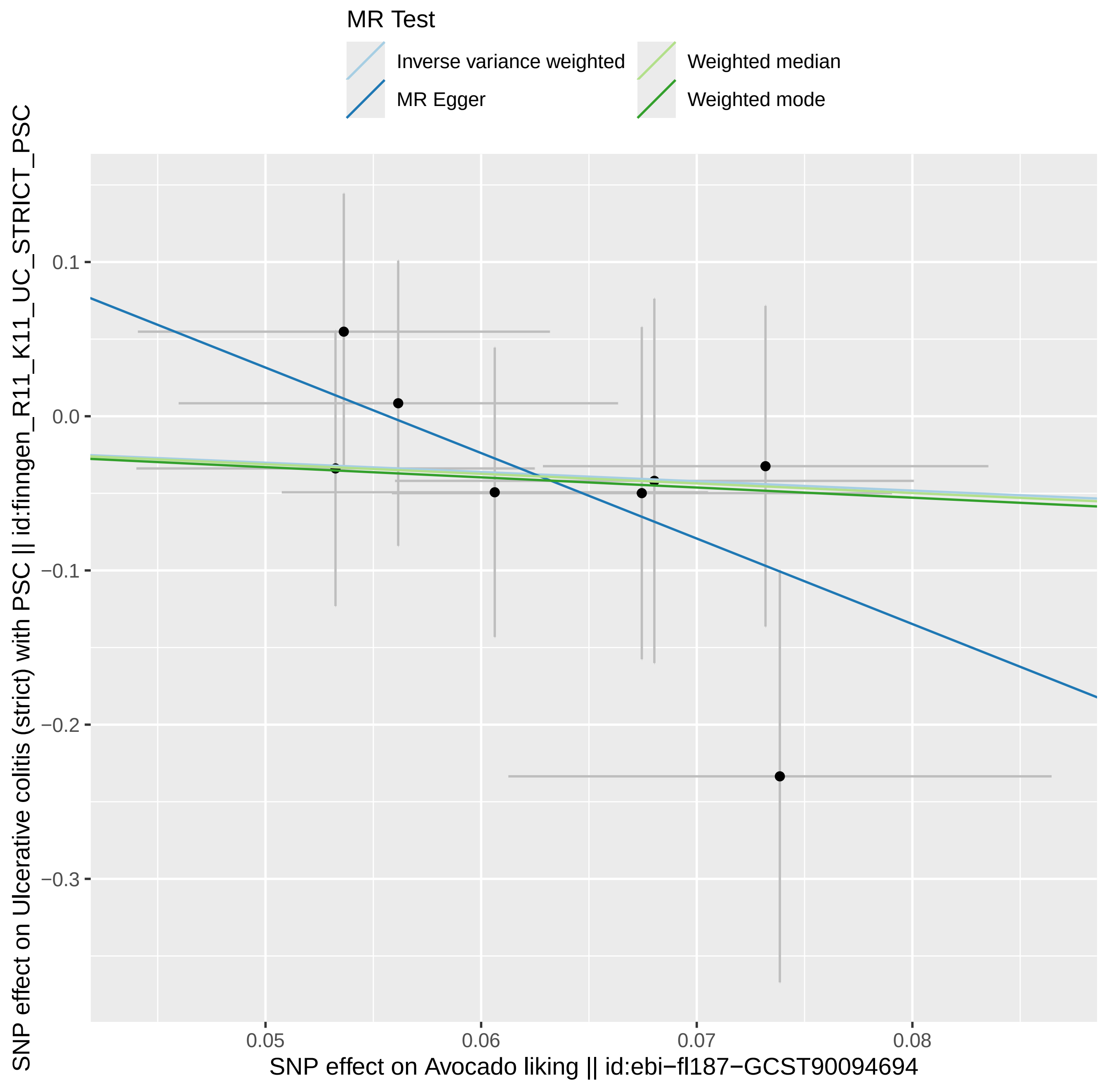
MR Test
Inverse variance weighted
MR Egger
Weighted median
Weighted mode
0.1
0.0
−0.1
−0.2
−0.3
0.05
0.06
0.07
0.08
SNP effect on Ulcerative colitis (strict) with PSC || id:finngen_R11_K11_UC_STRICT_PSC
SNP effect on Avocado liking || id:ebi−fI187−GCST90094694

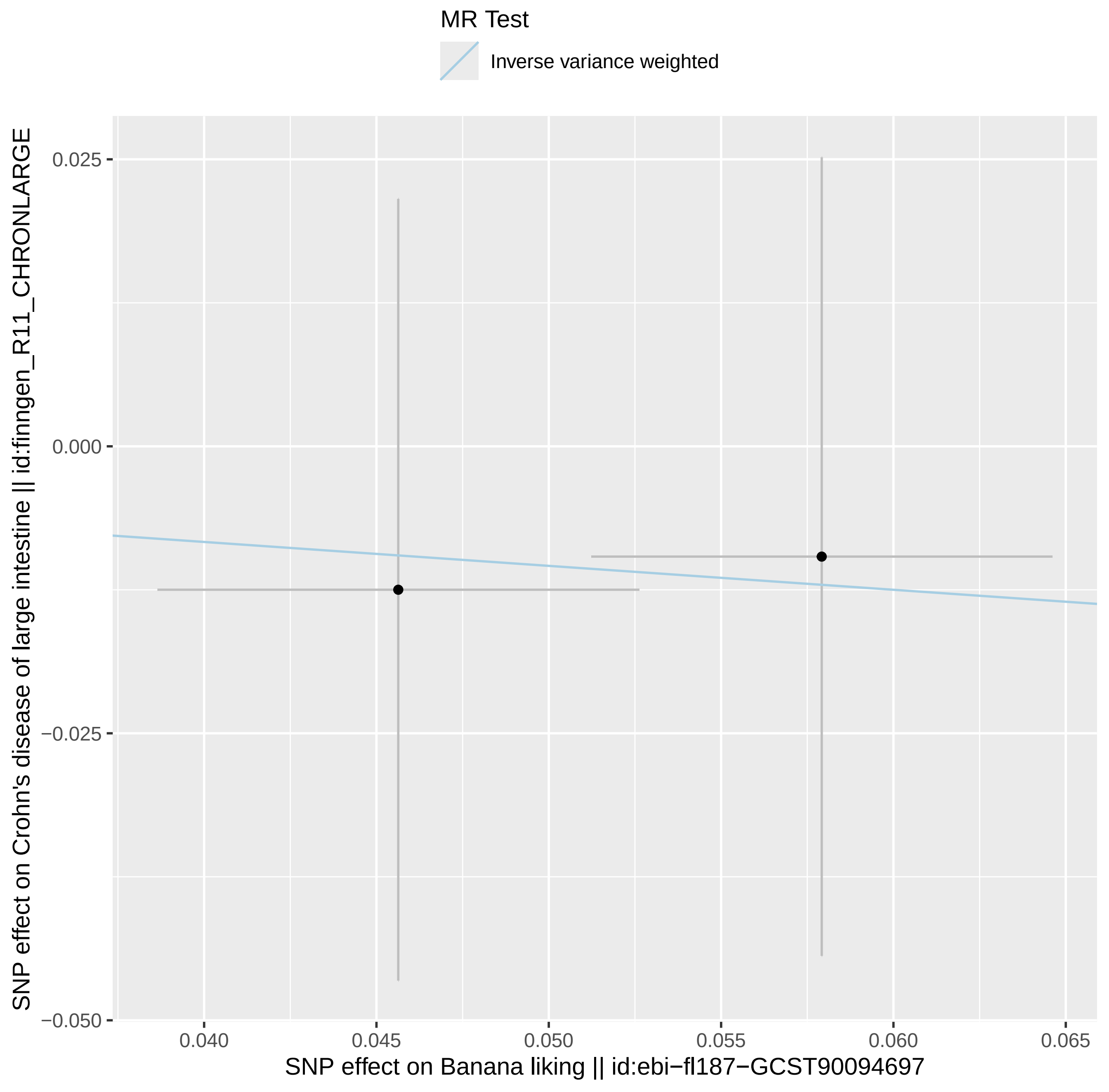
MR Test
Inverse variance weighted
SNP effect on Crohn's disease of large intestine || id:finngen_R11_CHRONLARGE
0.025
0.000
−0.025
−0.050
0.040
0.045
0.050
0.055
0.060
0.065
SNP effect on Banana liking || id:ebi−fl187−GCST90094697

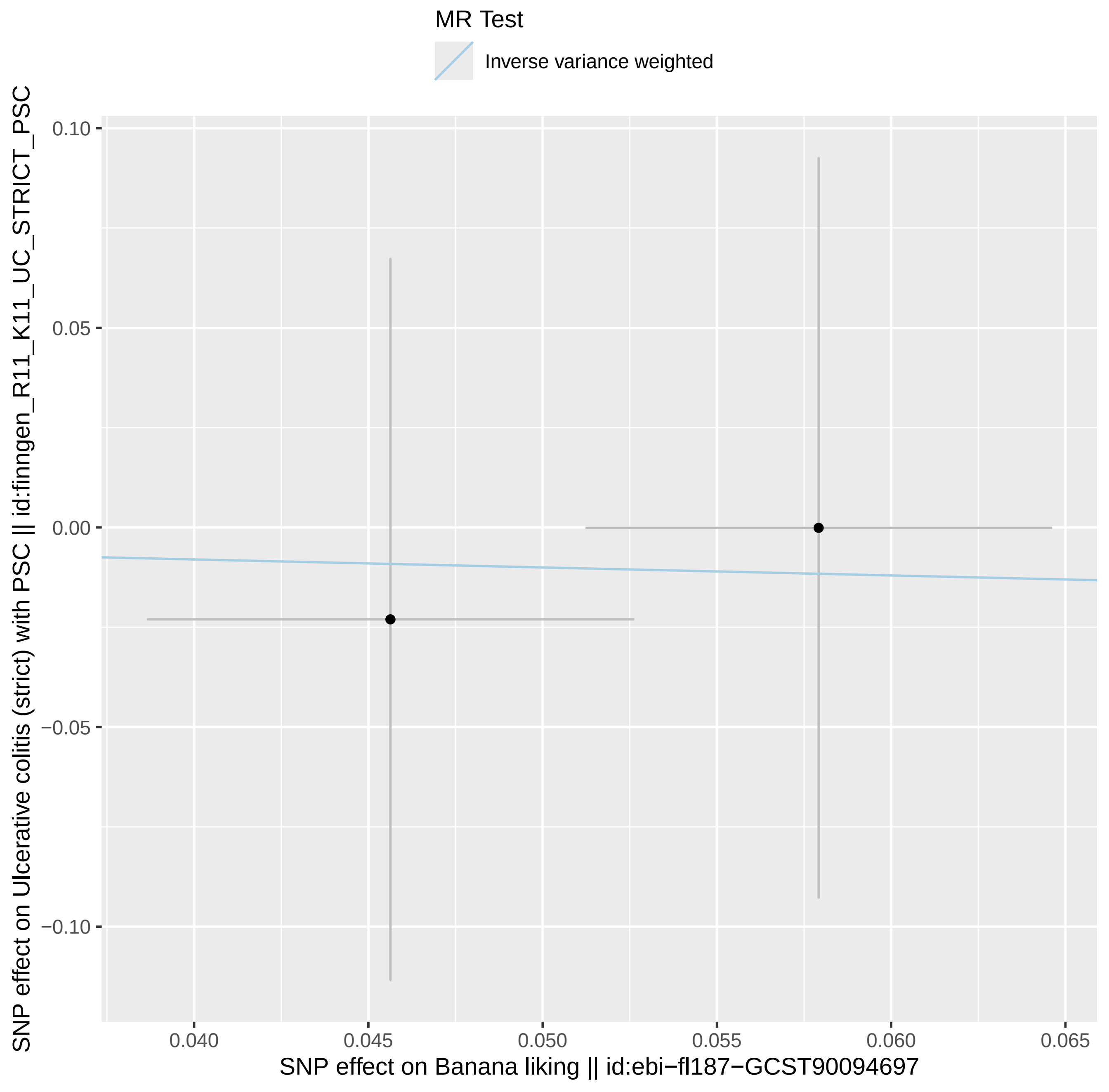
MR Test
Inverse variance weighted
SNP effect on Ulcerative colitis (strict) with PSC || id:finngen_R11_K11_UC_STRICT_PSC
0.10
0.05
0.00
−0.05
−0.10
0.040
0.045
0.050
0.055
0.060
0.065
SNP effect on Banana liking || id:ebi−fl187−GCST90094697

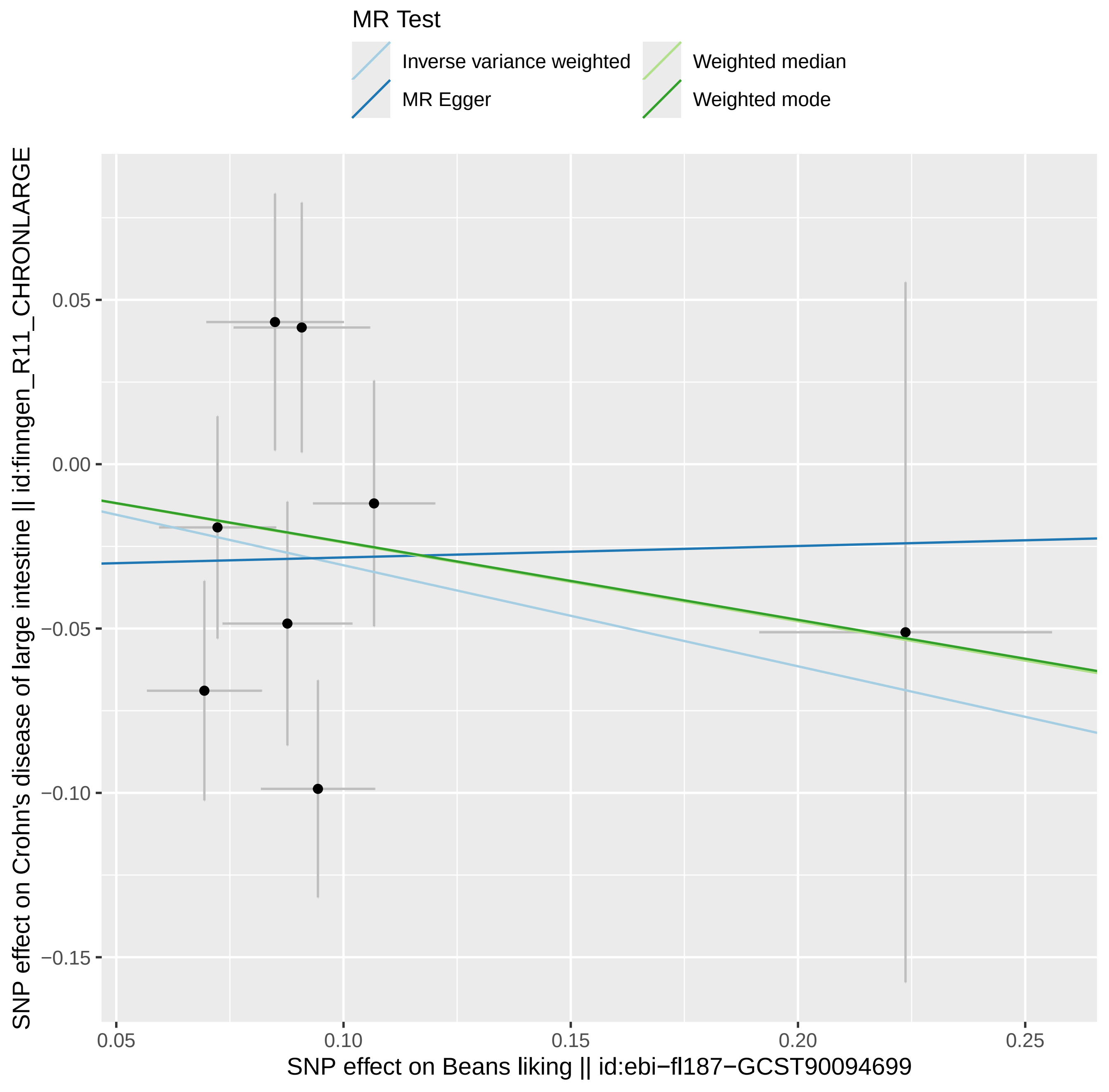
MR Test
Inverse variance weighted
MR Egger
Weighted median
Weighted mode
0.05
0.00
−0.05
−0.10
−0.15
0.05
0.10
0.15
0.20
0.25
SNP effect on Crohn's disease of large intestine || id:finngen_R11_CHRONLARGE
SNP effect on Beans liking || id:ebi−fl187−GCST90094699

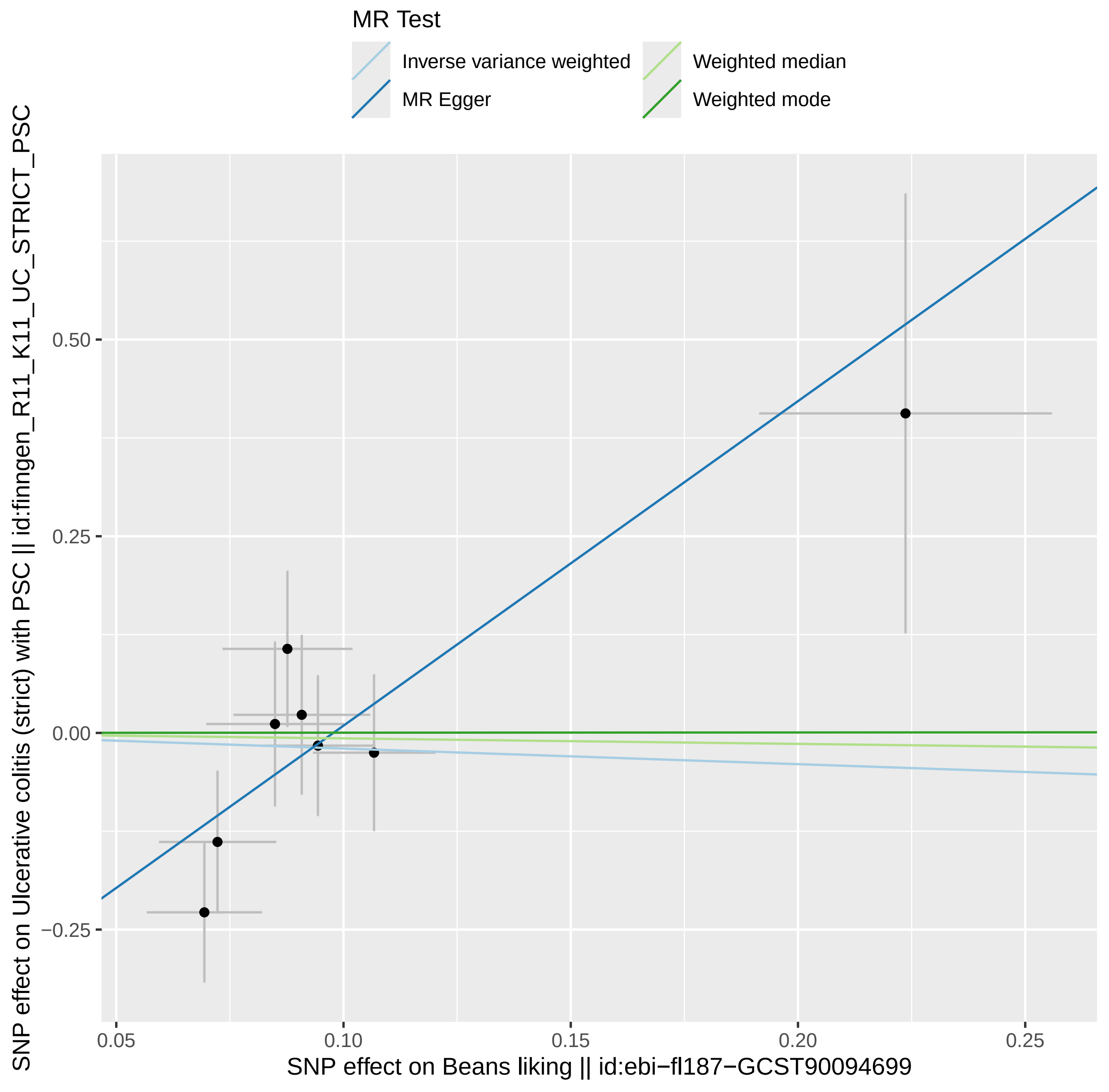
MR Test
Inverse variance weighted
MR Egger
Weighted median
Weighted mode
SNP effect on Ulcerative colitis (strict) with PSC || id:finngen_R11_K11_UC_STRICT_PSC
0.50
0.25
0.00
−0.25
0.05
0.10
0.15
0.20
0.25
SNP effect on Beans liking || id:ebi−fl187−GCST90094699

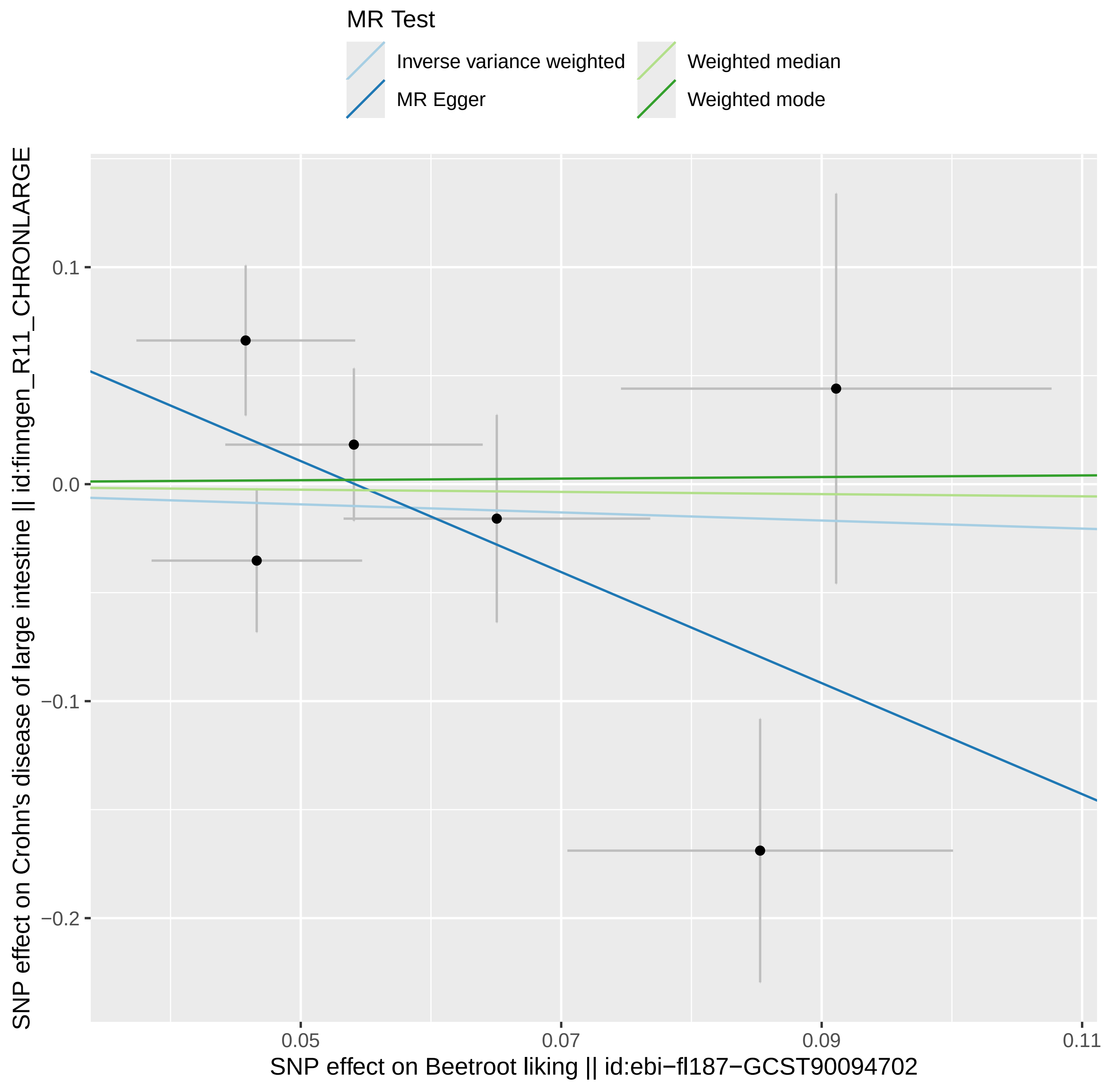
MR Test
Inverse variance weighted
MR Egger
Weighted median
Weighted mode
SNP effect on Crohn's disease of large intestine || id:finngen_R11_CHRONLARGE
0.1
0.0
−0.1
−0.2
0.05
0.07
0.09
0.11
SNP effect on Beetroot liking || id:ebi−fl187−GCST90094702

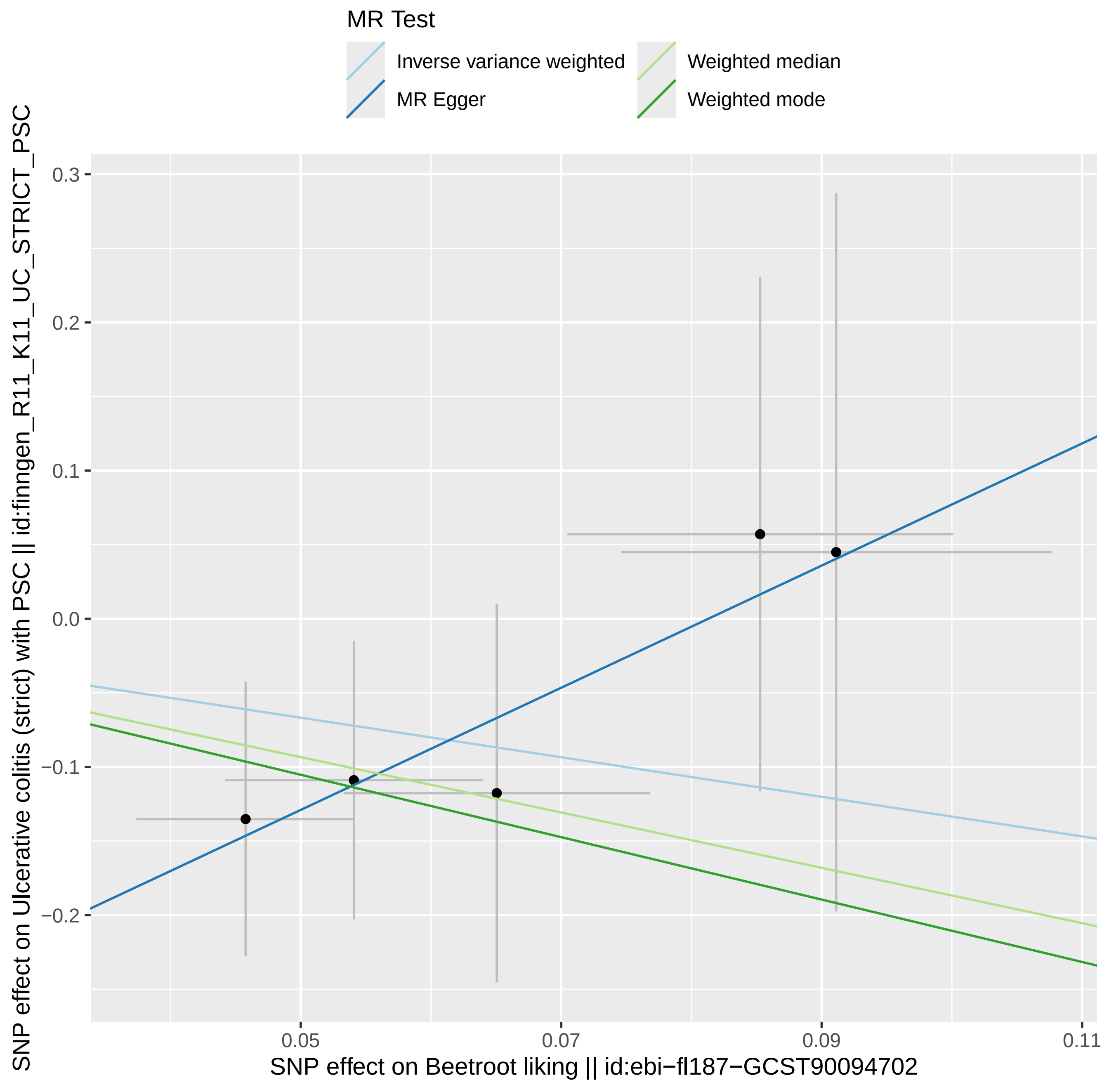
MR Test
Inverse variance weighted
MR Egger
Weighted median
Weighted mode
SNP effect on Ulcerative colitis (strict) with PSC || id:finngen_R11_K11_UC_STRICT_PSC
SNP effect on Beetroot liking || id:ebi−fl187−GCST90094702
0.3
0.2
0.1
0.0
−0.1
−0.2
0.05
0.07
0.09
0.11

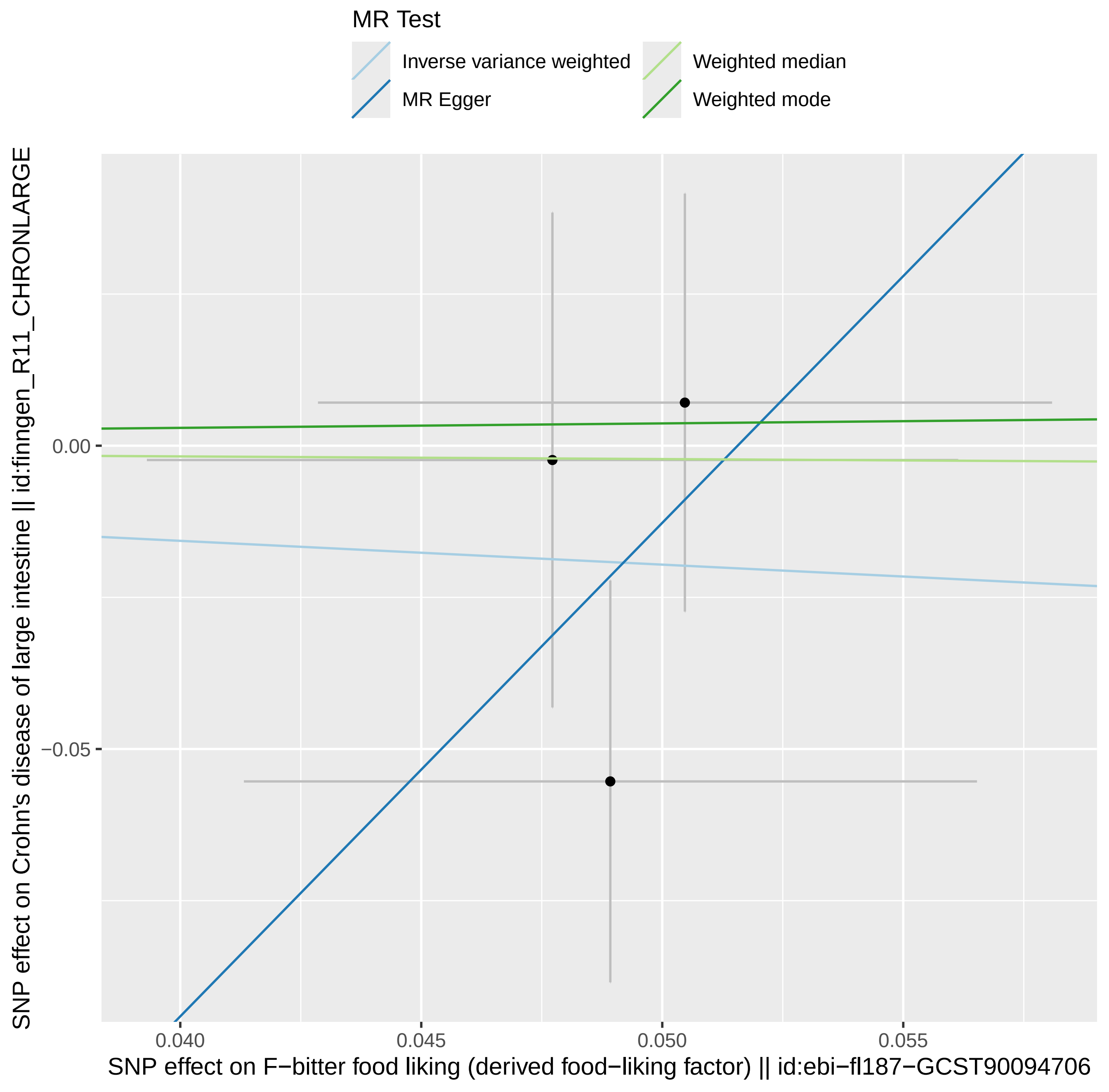

MR Test
Inverse variance weighted
MR Egger
Weighted median
Weighted mode
SNP effect on Crohn's disease of large intestine || id:finngen_R11_CHRONLARGE
0.00
−0.05
0.040
0.045
0.050
0.055
SNP effect on F−bitter food liking (derived food−liking factor) || id:ebi−fl187−GCST90094706

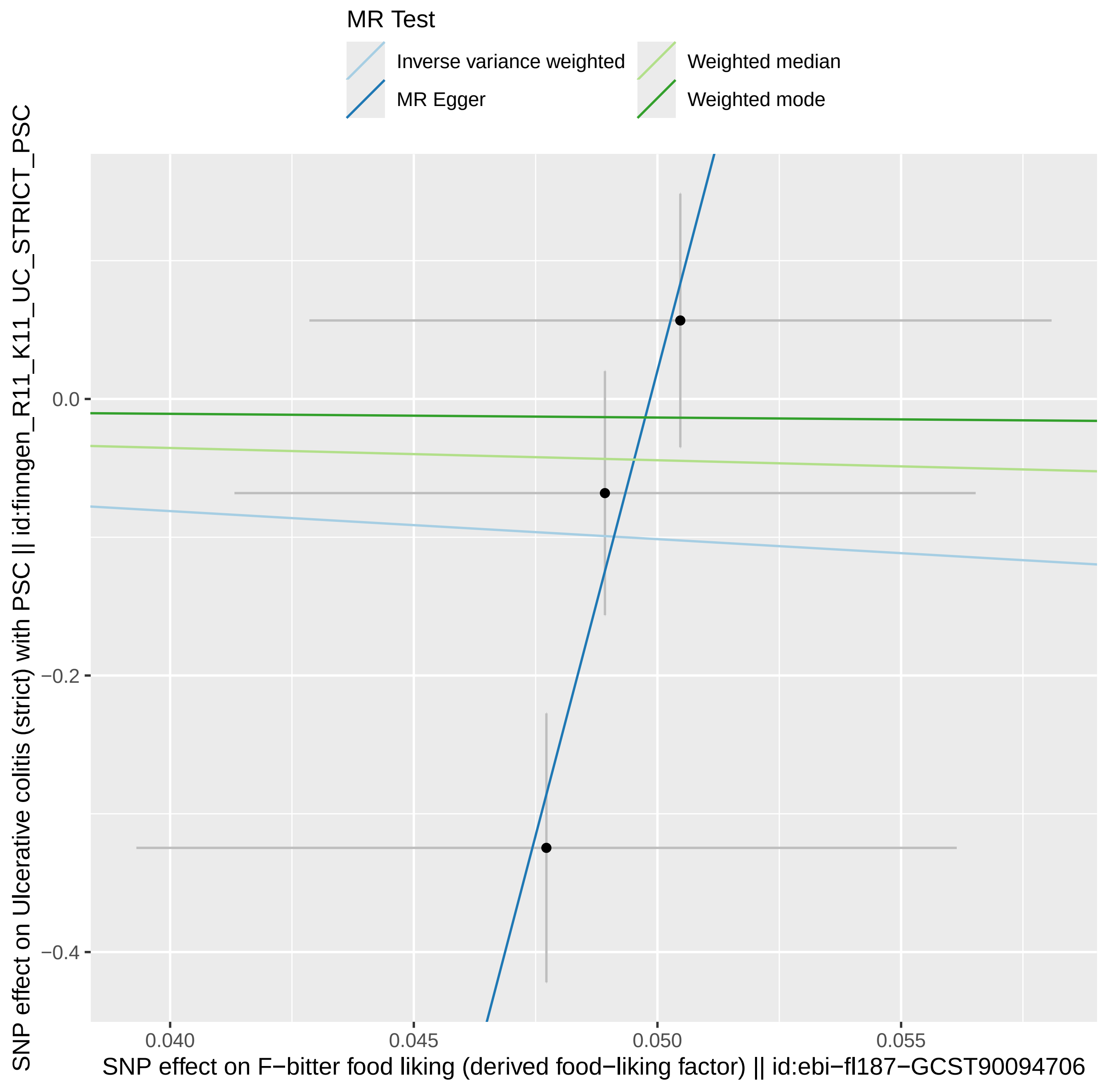

MR Test
Inverse variance weighted
MR Egger
Weighted median
Weighted mode
0.0
−0.2
−0.4
0.040
0.045
0.050
0.055
SNP effect on F−bitter food liking (derived food−liking factor) || id:ebi−fl187−GCST90094706
SNP effect on Ulcerative colitis (strict) with PSC || id:finngen_R11_K11_UC_STRICT_PSC

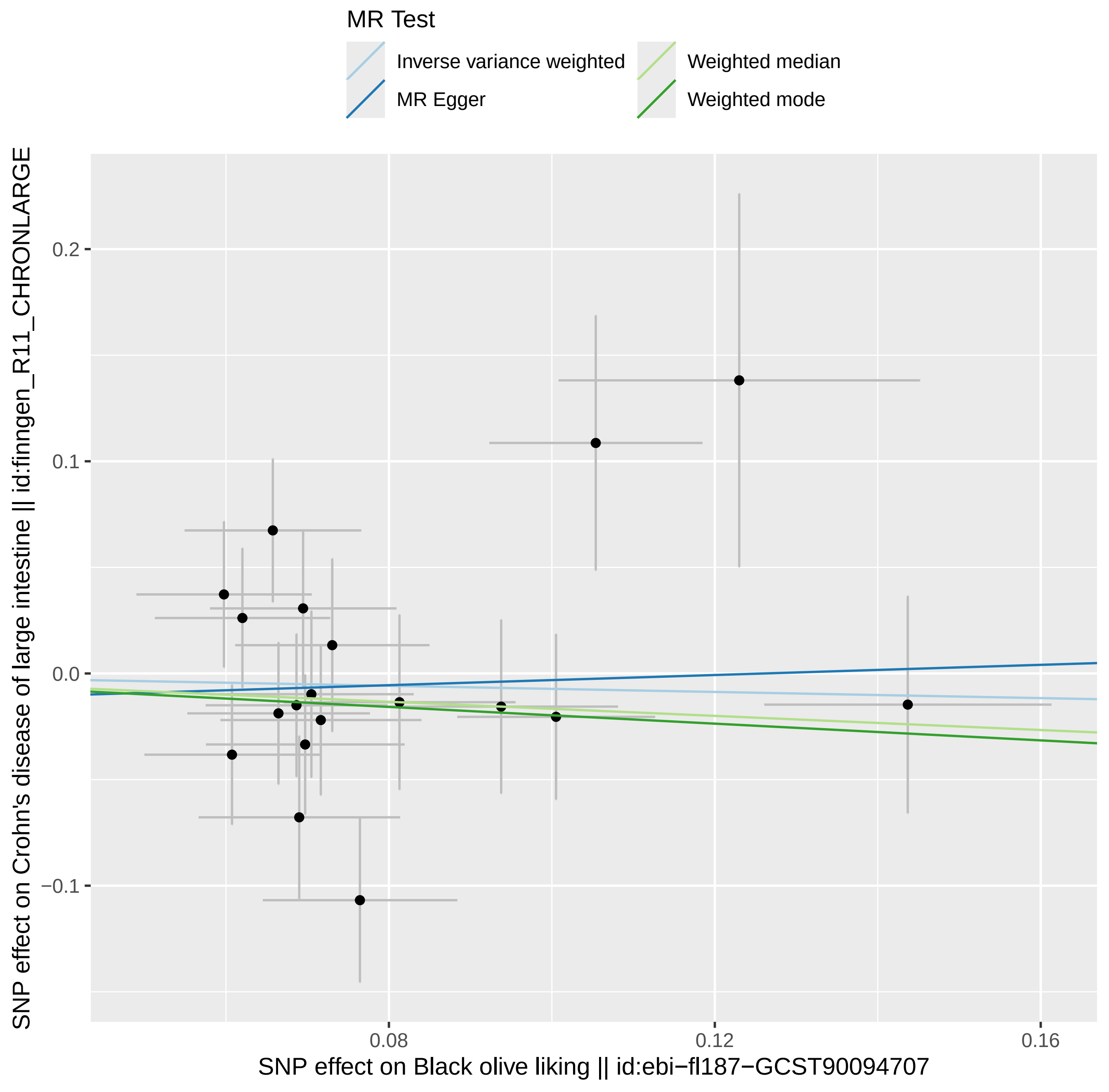

MR Test
Inverse variance weighted
MR Egger
Weighted median
Weighted mode
SNP effect on Crohn's disease of large intestine || id:finngen_R11_CHRONLARGE
0.2
0.1
0.0
−0.1
0.08
0.12
0.16
SNP effect on Black olive liking || id:ebi−fl187−GCST90094707

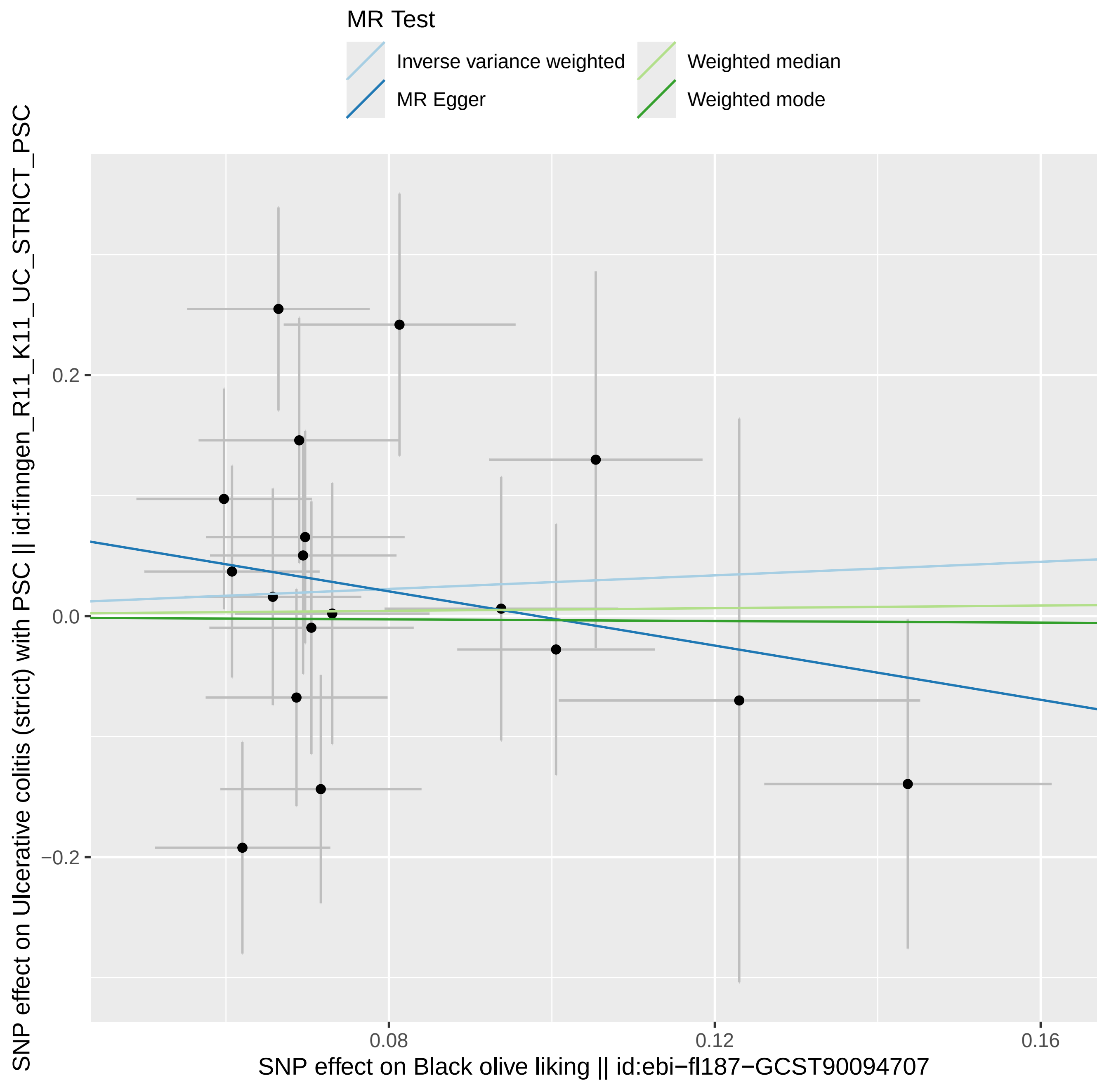
MR Test
Inverse variance weighted
MR Egger
Weighted median
Weighted mode
0.2
0.0
−0.2
0.08
0.12
0.16
SNP effect on Black olive liking || id:ebi−fl187−GCST90094707
SNP effect on Ulcerative colitis (strict) with PSC || id:finngen_R11_K11_UC_STRICT_PSC

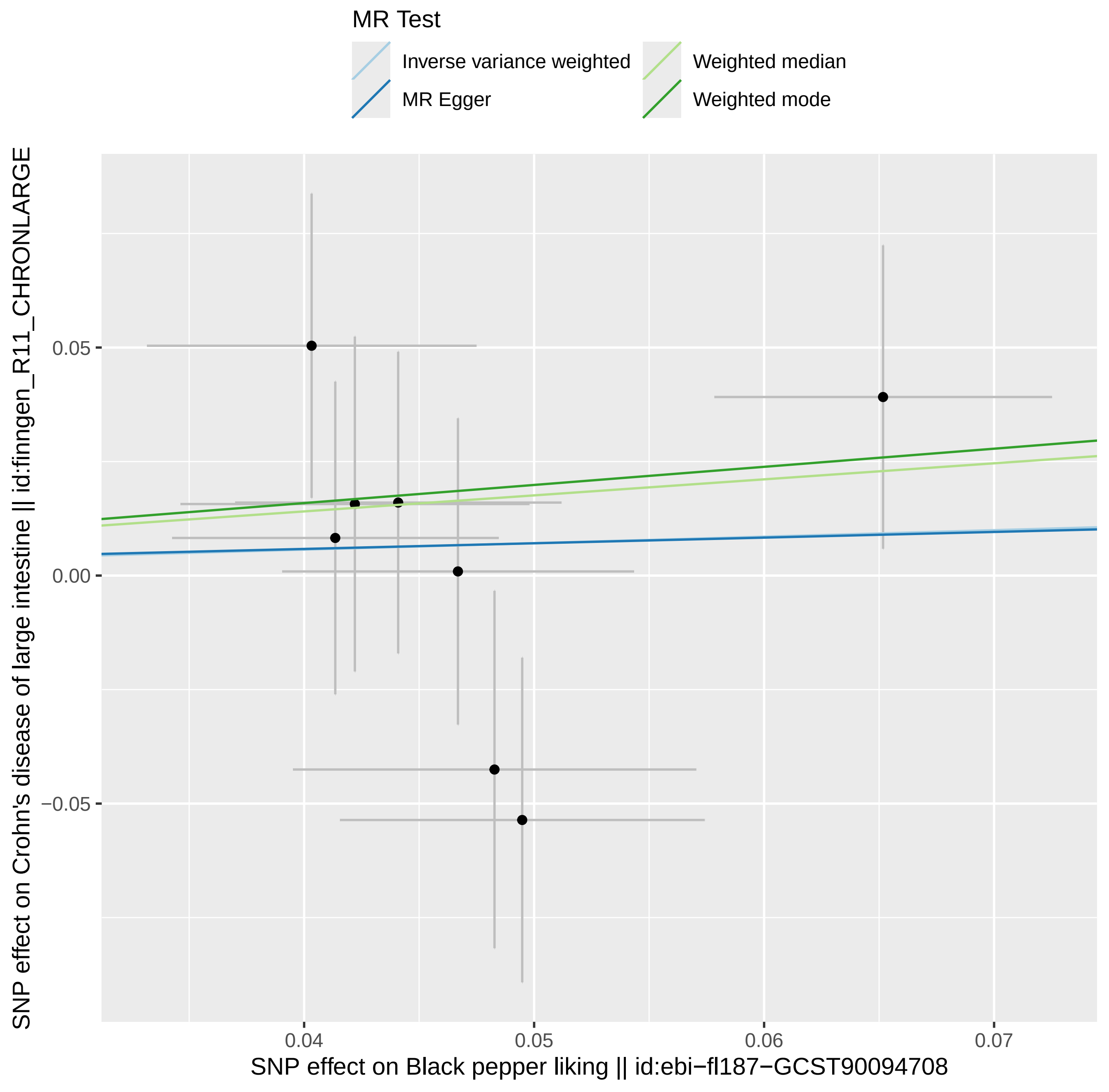
MR Test
Inverse variance weighted
MR Egger
Weighted median
Weighted mode
SNP effect on Crohn's disease of large intestine || id:finngen_R11_CHRONLARGE
0.05
0.00
−0.05
0.04
0.05
0.06
0.07
SNP effect on Black pepper liking || id:ebi−fl187−GCST90094708

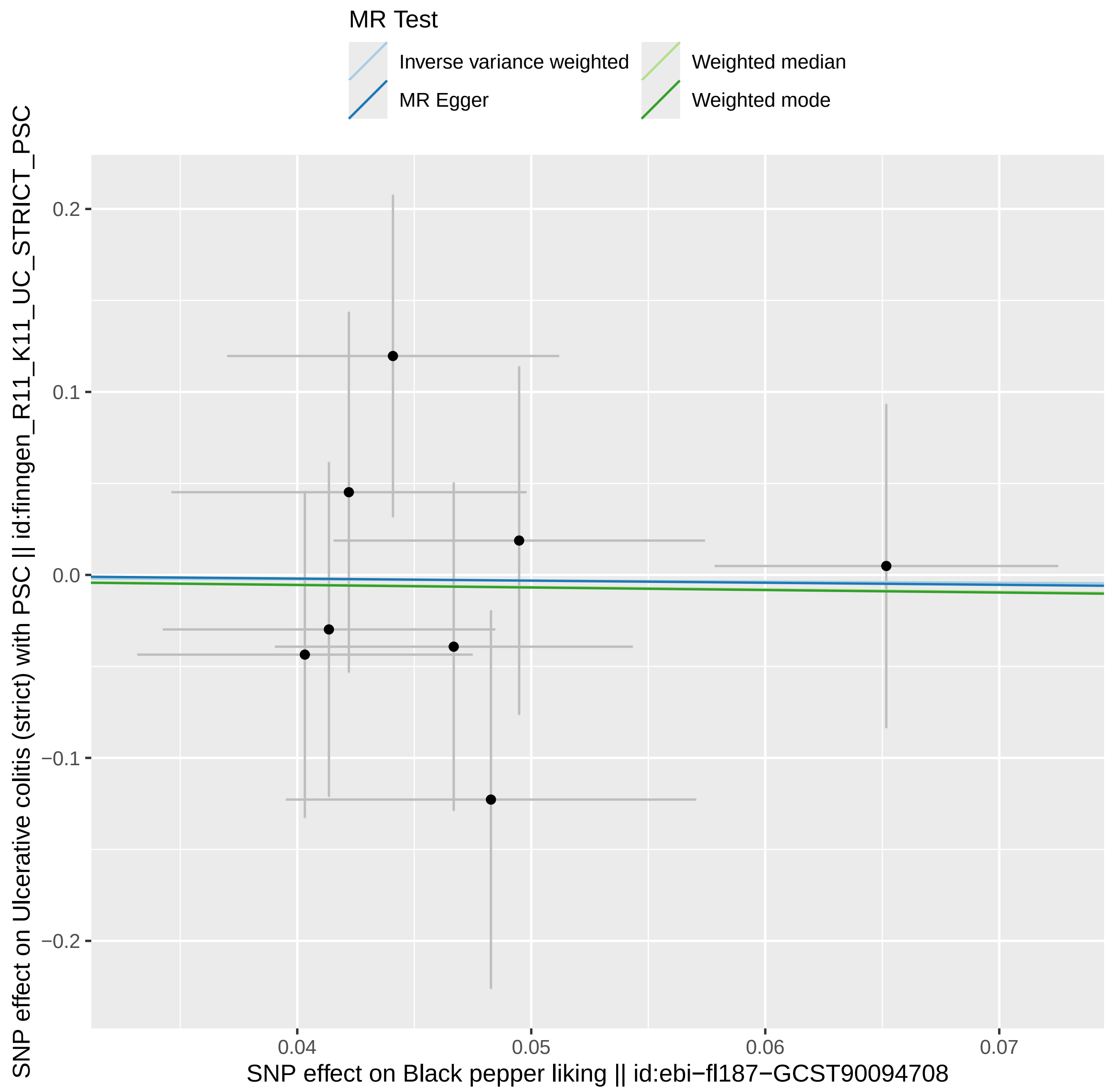
MR Test
Inverse variance weighted
MR Egger
Weighted median
Weighted mode
0.2
0.1
0.0
−0.1
−0.2
0.04
0.05
0.06
0.07
SNP effect on Ulcerative colitis (strict) with PSC || id:finngen_R11_K11_UC_STRICT_PSC
SNP effect on Black pepper liking || id:ebi−fl187−GCST90094708

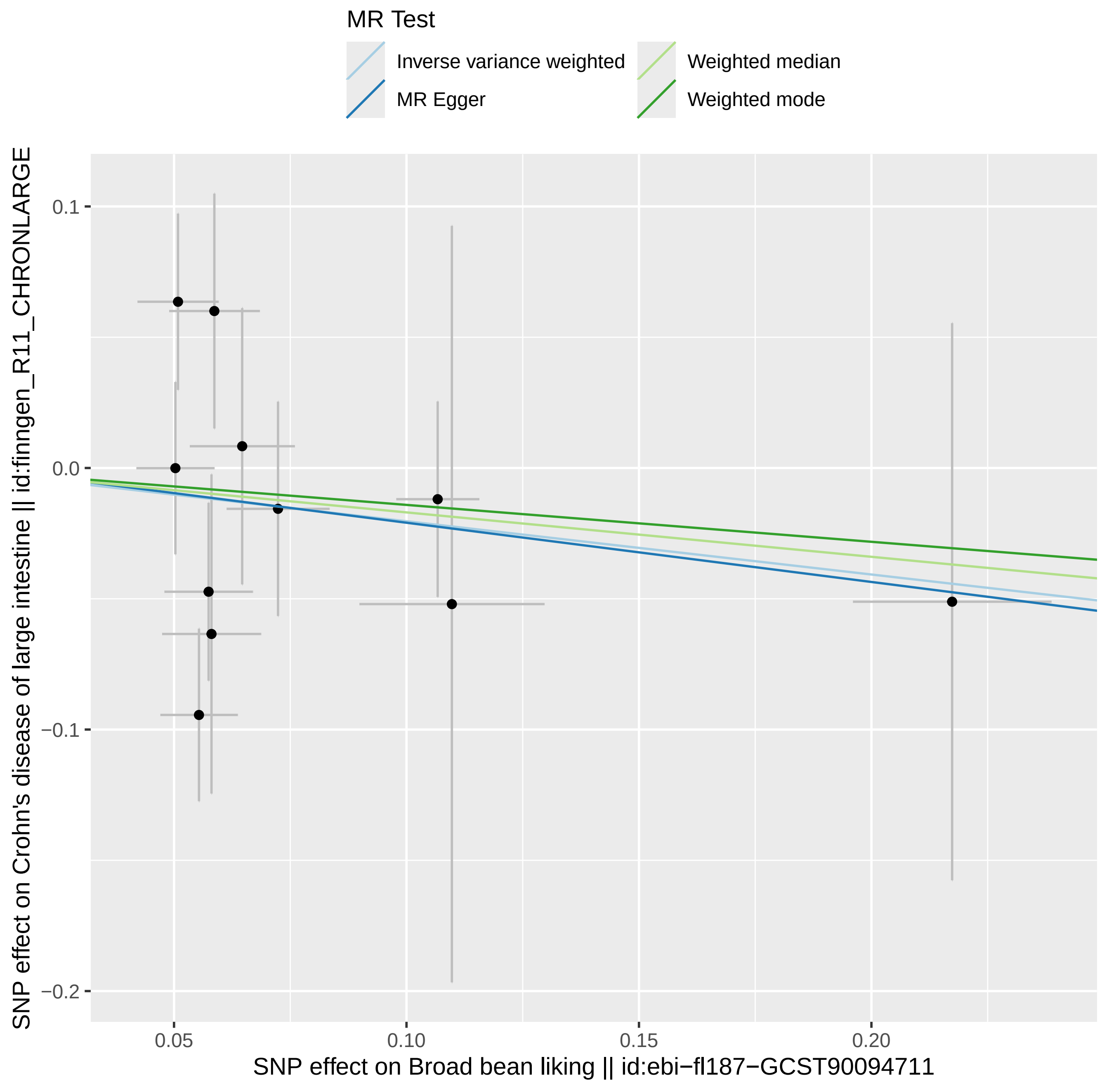

MR Test
Inverse variance weighted
MR Egger
Weighted median
Weighted mode
0.1
0.0
−0.1
−0.2
SNP effect on Crohn's disease of large intestine || id:finngen_R11_CHRONLARGE
0.05
0.10
0.15
0.20
SNP effect on Broad bean liking || id:ebi−fl187−GCST90094711

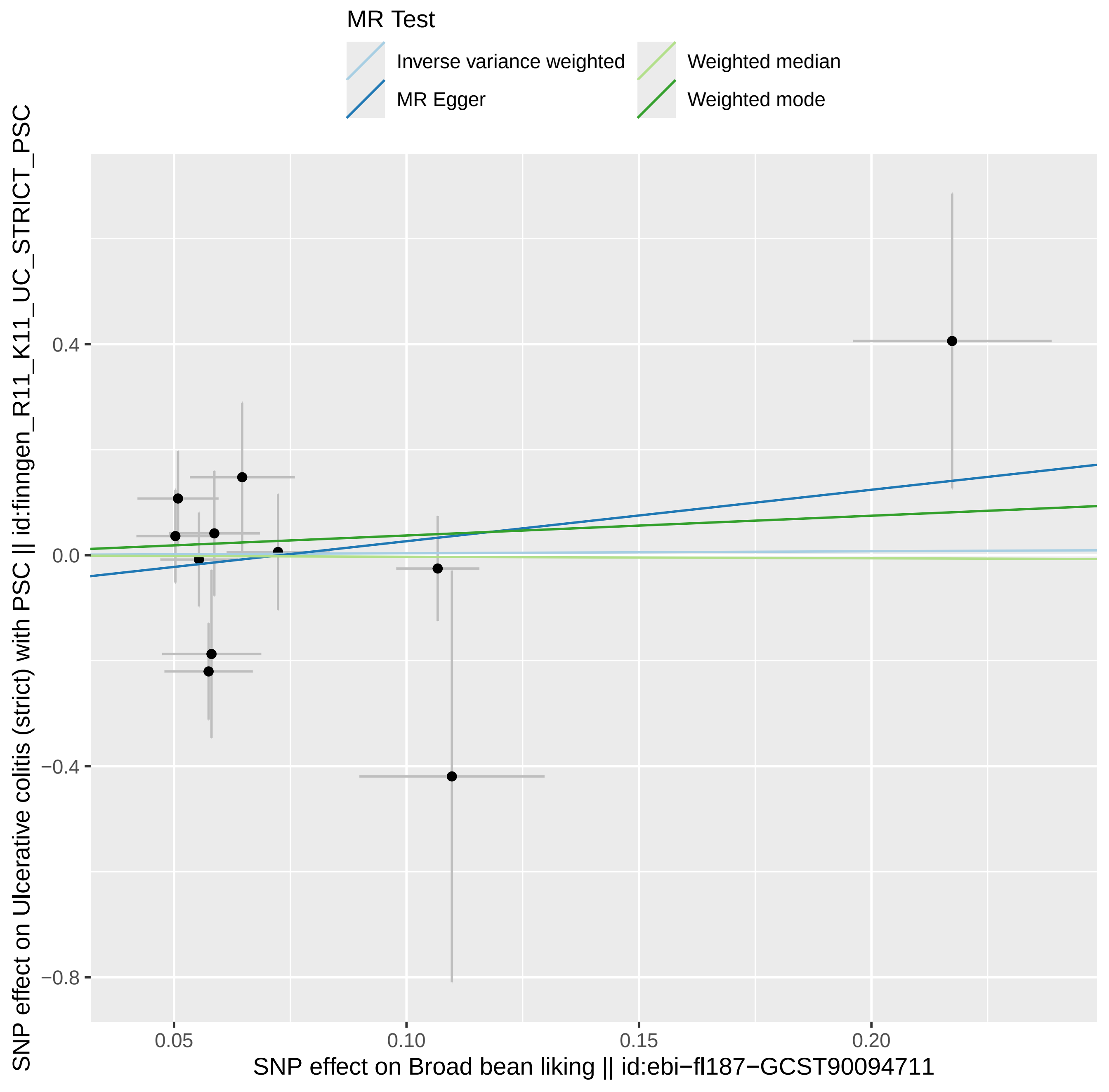
MR Test
Inverse variance weighted
MR Egger
Weighted median
Weighted mode
0.4
0.0
−0.4
−0.8
0.05
0.10
0.15
0.20
SNP effect on Broad bean liking || id:ebi−fl187−GCST90094711
SNP effect on Ulcerative colitis (strict) with PSC || id:finngen_R11_K11_UC_STRICT_PSC

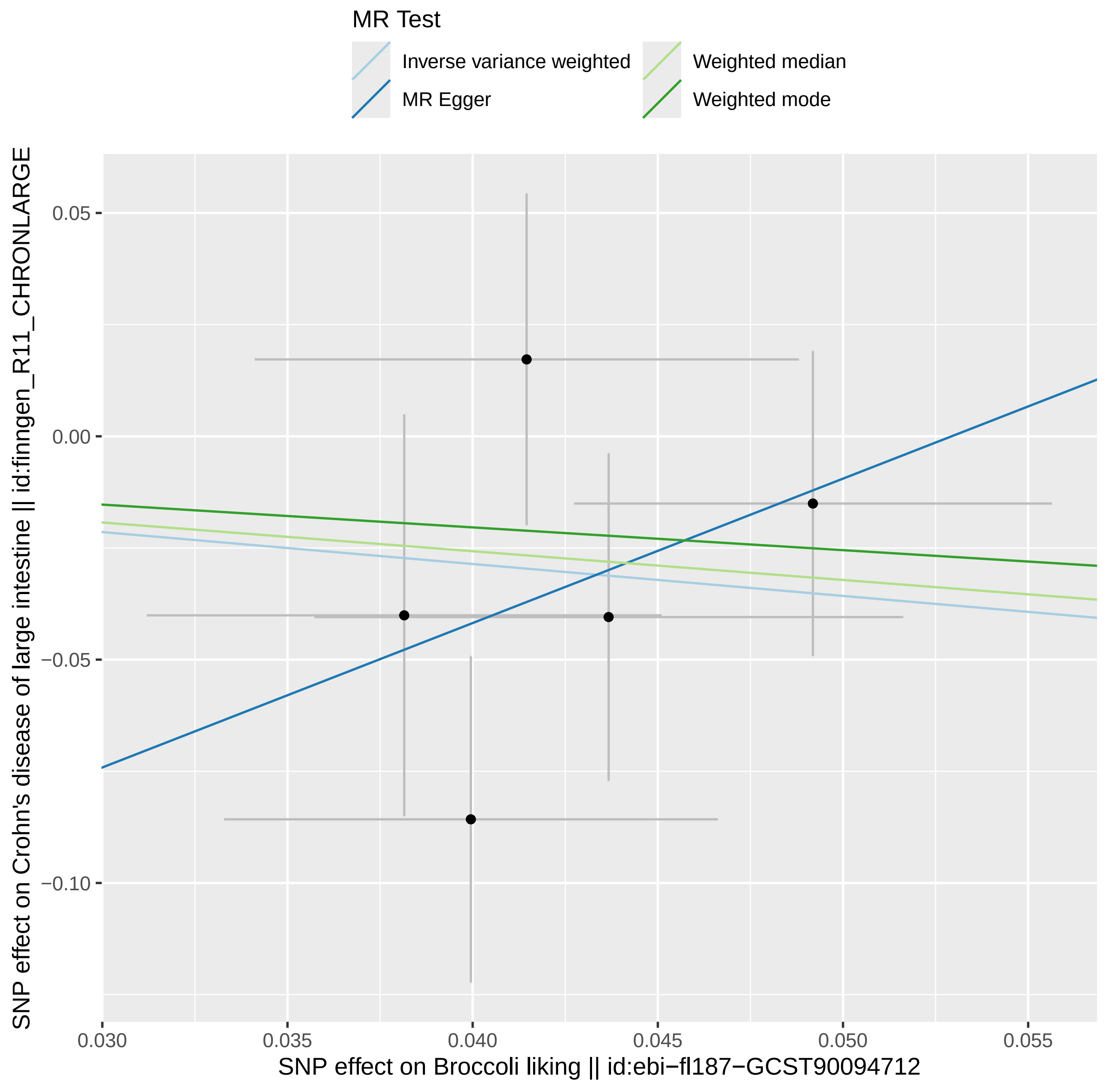

MR Test
Inverse variance weighted
MR Egger
Weighted median
Weighted mode
0.05
0.00
−0.05
−0.10
0.030
0.035
0.040
0.045
0.050
0.055
SNP effect on Broccoli liking || id:ebi−fl187−GCST90094712
SNP effect on Crohn's disease of large intestine || id:finngen_R11_CHRONLARGE

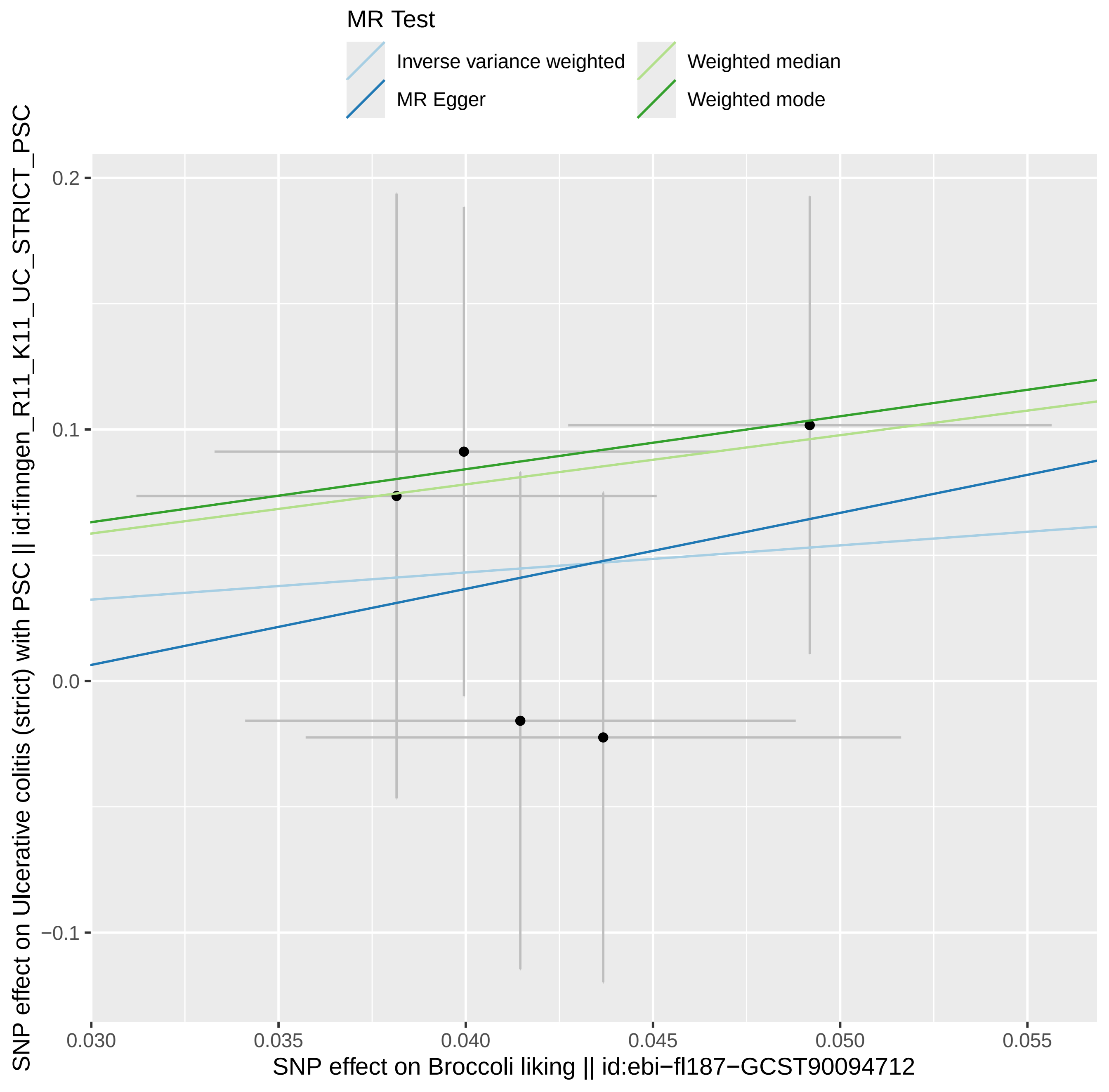
MR Test
Inverse variance weighted
MR Egger
Weighted median
Weighted mode
0.2
0.1
0.0
−0.1
0.030
0.035
0.040
0.045
0.050
0.055
SNP effect on Broccoli liking || id:ebi−fl187−GCST90094712
SNP effect on Ulcerative colitis (strict) with PSC || id:finngen_R11_K11_UC_STRICT_PSC

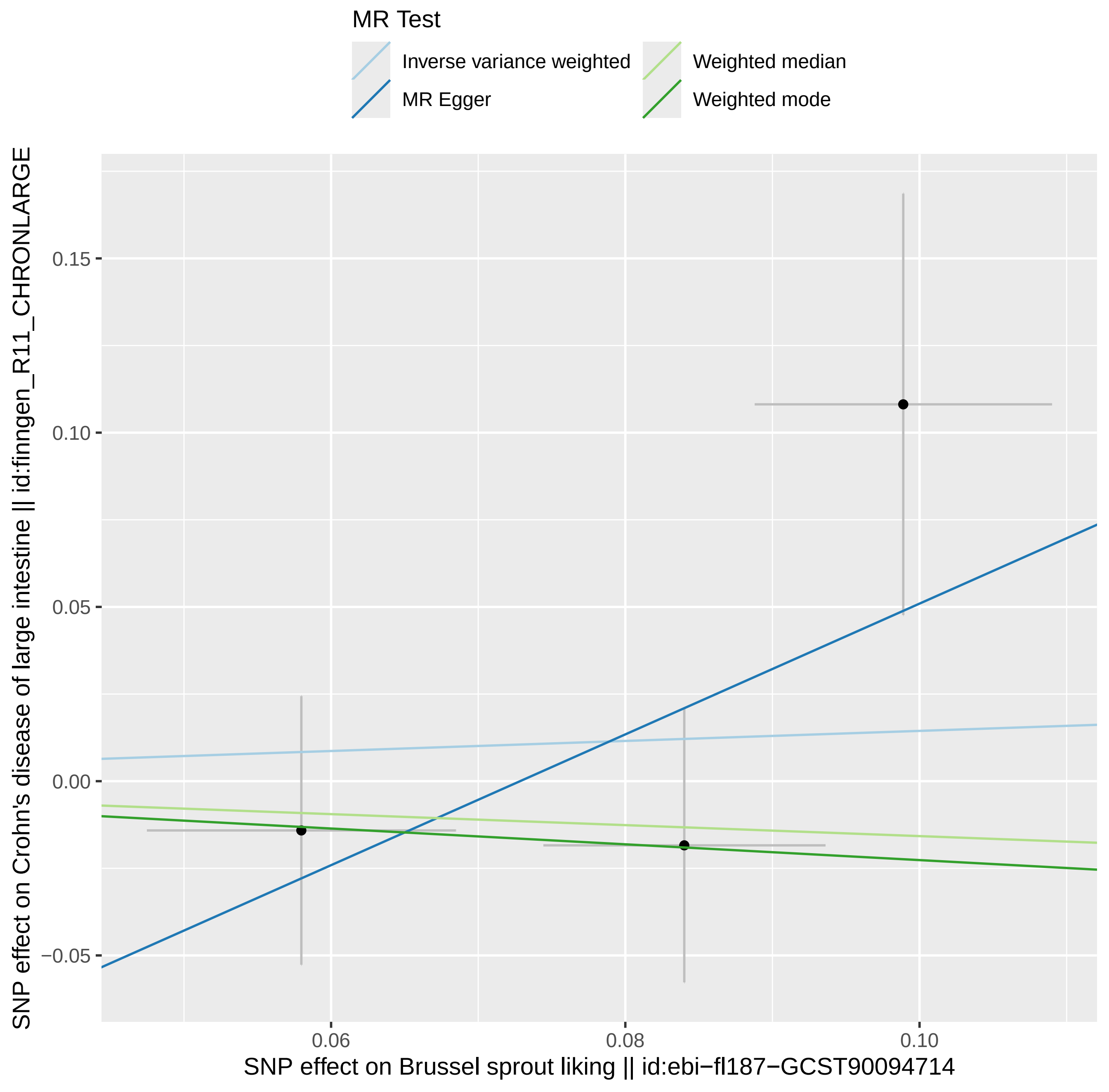

MR Test
Inverse variance weighted
MR Egger
Weighted median
Weighted mode
0.15
0.10
0.05
0.00
−0.05
0.06
0.08
0.10
SNP effect on Crohn's disease of large intestine || id:finngen_R11_CHRONLARGE
SNP effect on Brussel sprout liking || id:ebi−fl187−GCST90094714

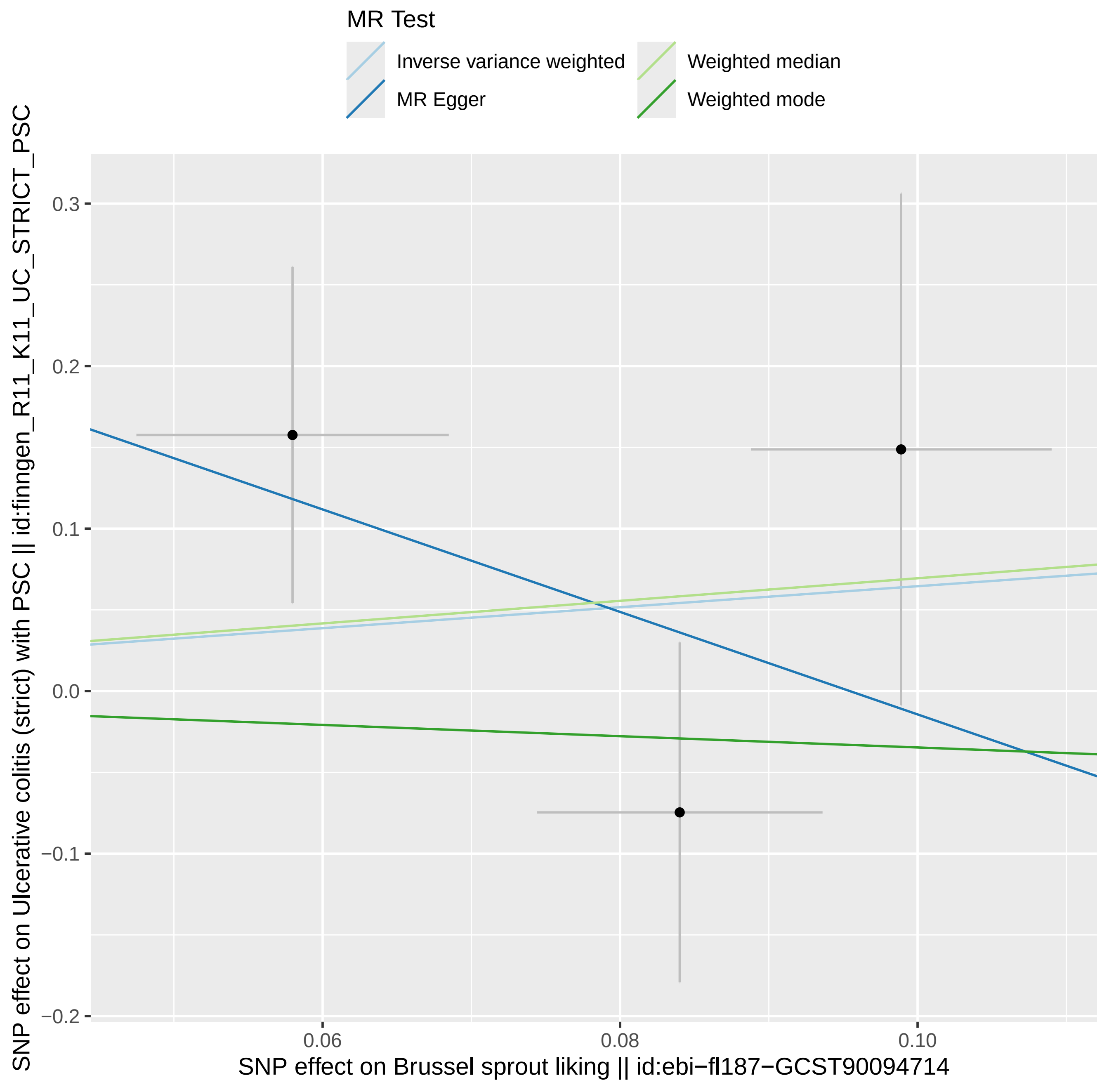
MR Test
Inverse variance weighted
MR Egger
Weighted median
Weighted mode
0.3
0.2
0.1
0.0
−0.1
−0.2
0.06
0.08
0.10
SNP effect on Ulcerative colitis (strict) with PSC || id:finngen_R11_K11_UC_STRICT_PSC
SNP effect on Brussel sprout liking || id:ebi−fl187−GCST90094714

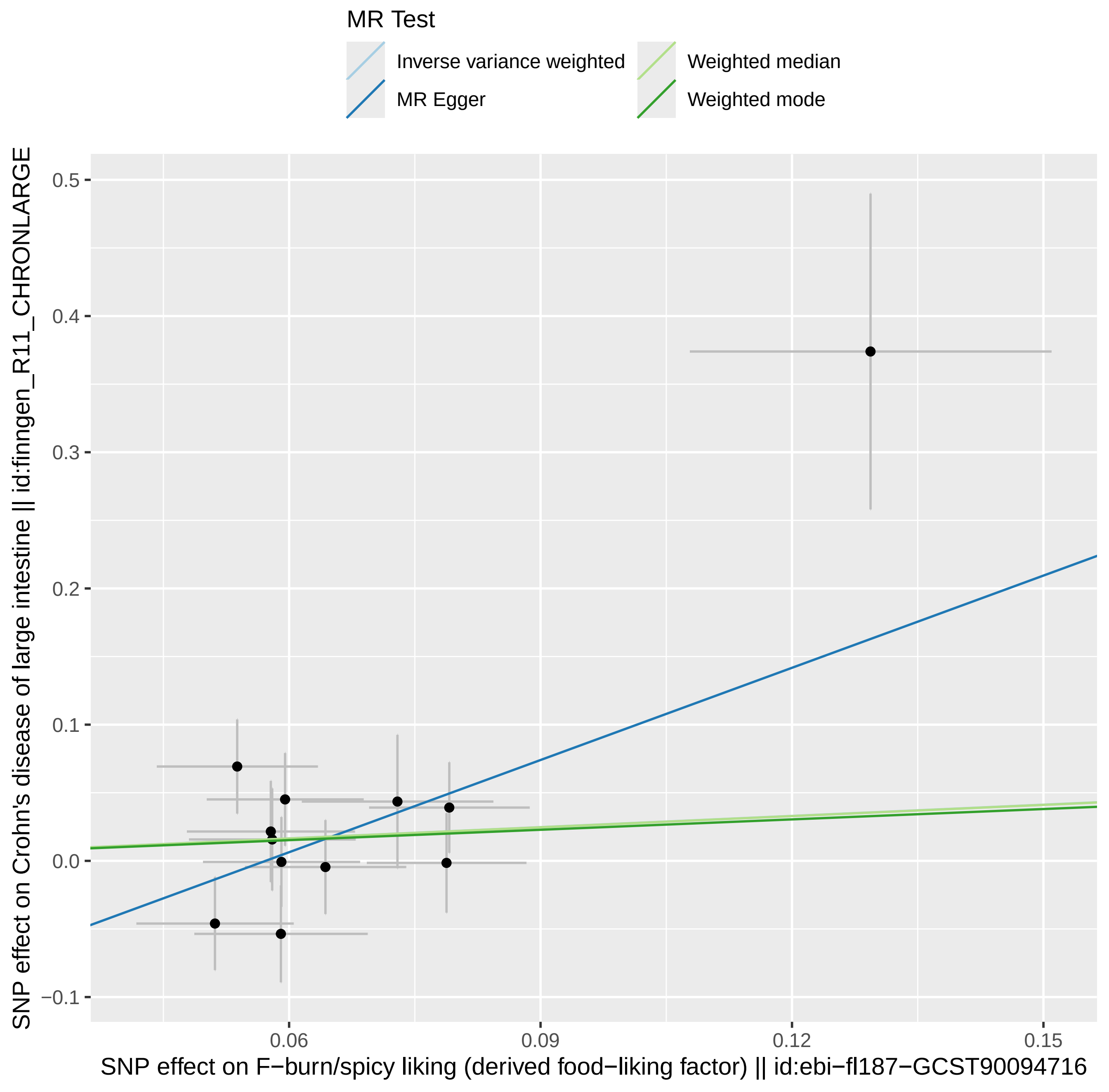
MR Test
Inverse variance weighted
MR Egger
Weighted median
Weighted mode
SNP effect on Crohn's disease of large intestine || id:finngen_R11_CHRONLARGE
0.5
0.4
0.3
0.2
0.1
0.0
−0.1
0.06
0.09
0.12
0.15
SNP effect on F−burn/spicy liking (derived food−liking factor) || id:ebi−fl187−GCST90094716

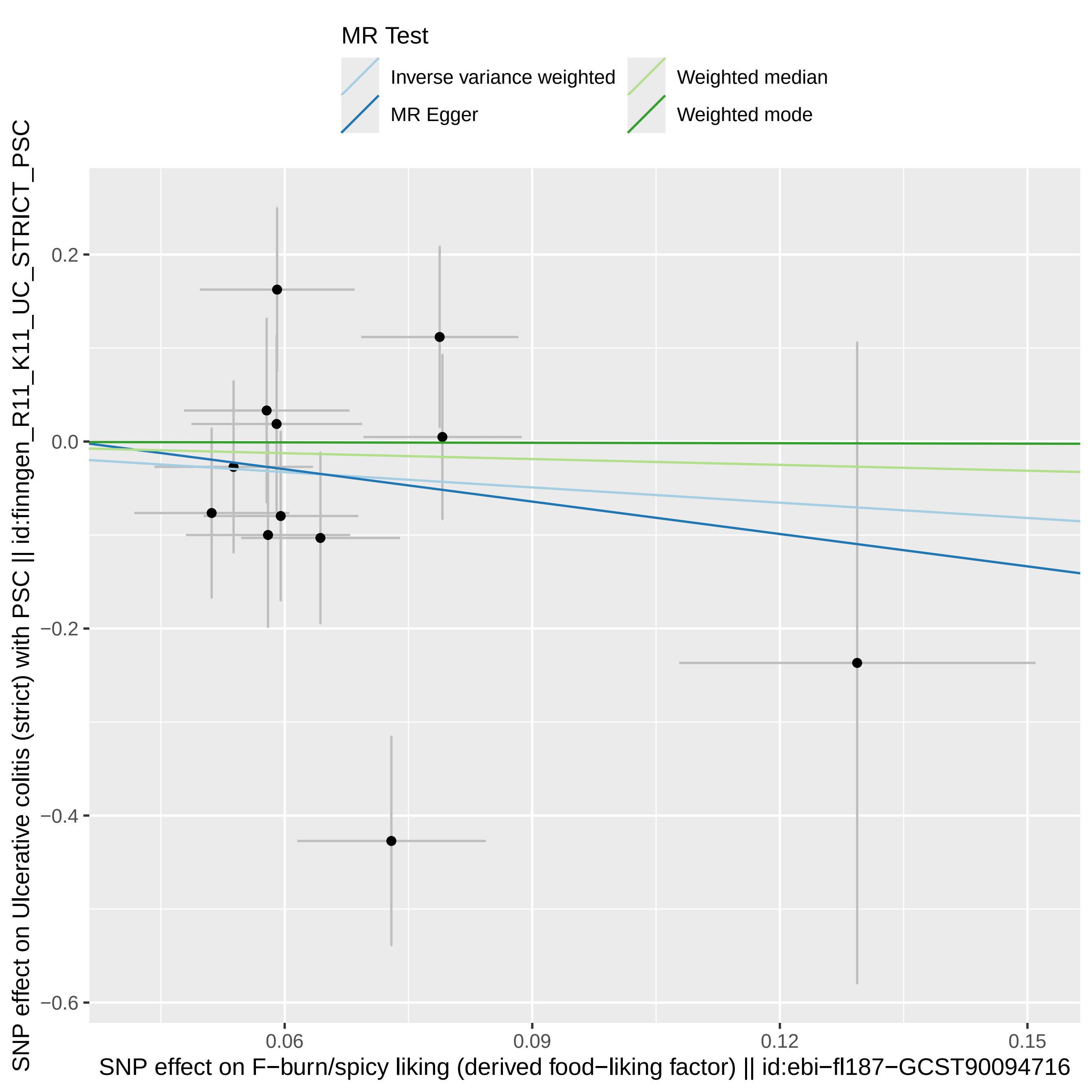
MR Test
Inverse variance weighted
MR Egger
Weighted median
Weighted mode
0.2
0.0
−0.2
−0.4
−0.6
0.06
0.09
0.12
0.15
SNP effect on Ulcerative colitis (strict) with PSC || id:finngen_R11_K11_UC_STRICT_PSC
SNP effect on F−burn/spicy liking (derived food−liking factor) || id:ebi−fl187−GCST90094716

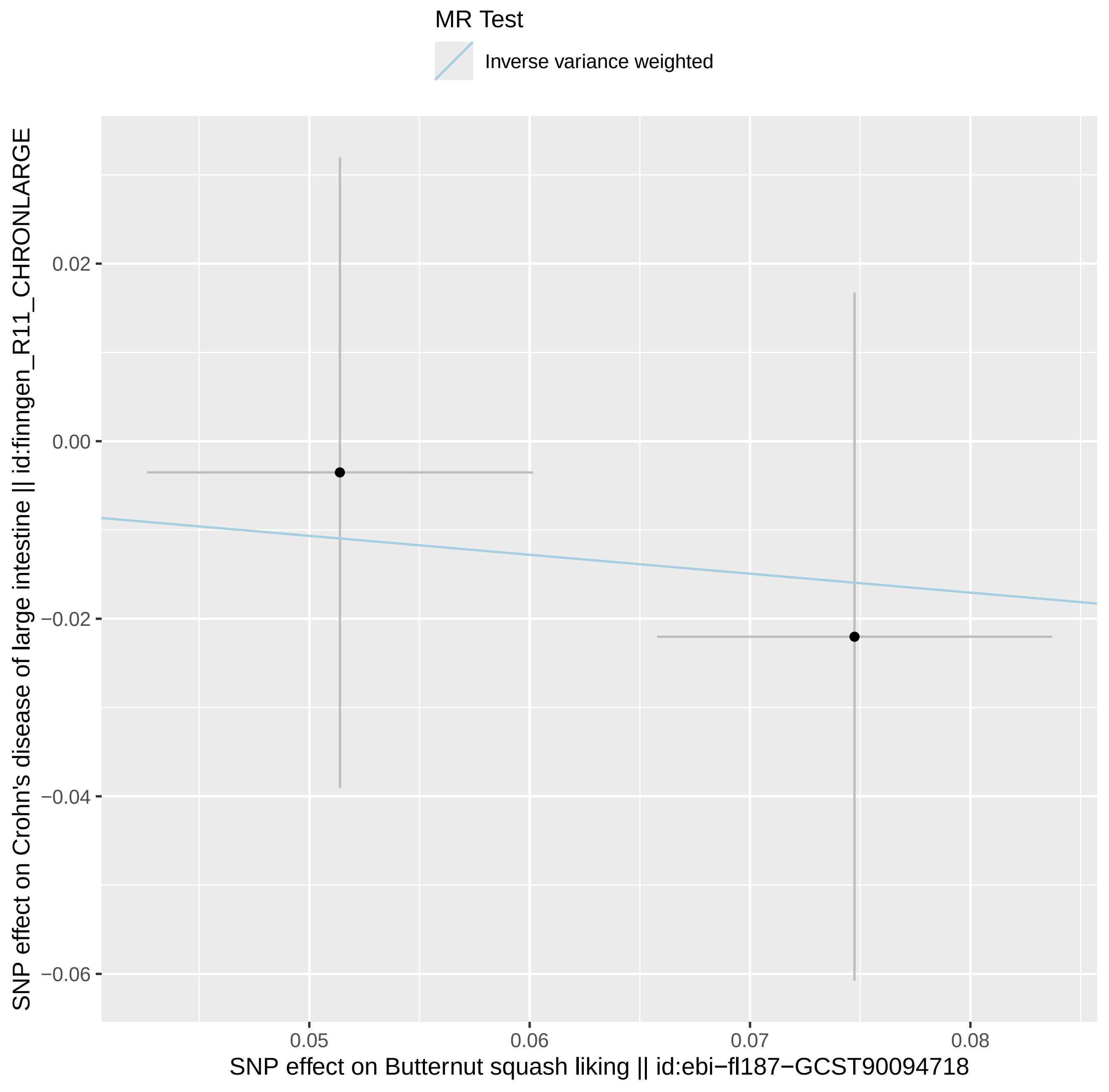
MR Test
Inverse variance weighted
0.02
0.00
−0.02
−0.04
−0.06
SNP effect on Crohn's disease of large intestine || id:finngen_R11_CHRONLARGE
0.05
0.06
0.07
0.08
SNP effect on Butternut squash liking || id:ebi−fl187−GCST90094718

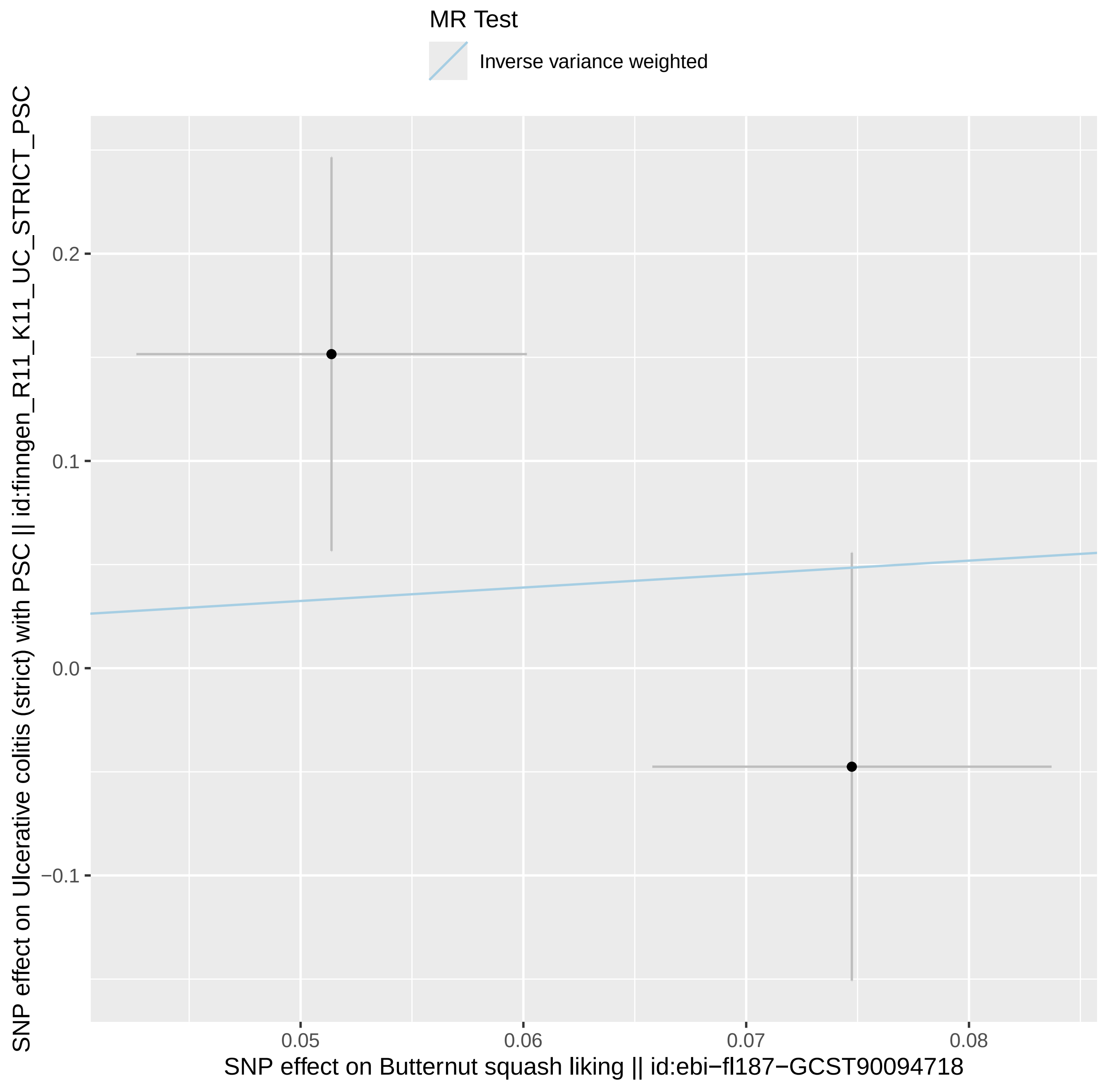

MR Test
Inverse variance weighted
SNP effect on Ulcerative colitis (strict) with PSC || id:finngen_R11_K11_UC_STRICT_PSC
0.2
0.1
0.0
−0.1
0.05
0.06
0.07
0.08
SNP effect on Butternut squash liking || id:ebi−fl187−GCST90094718

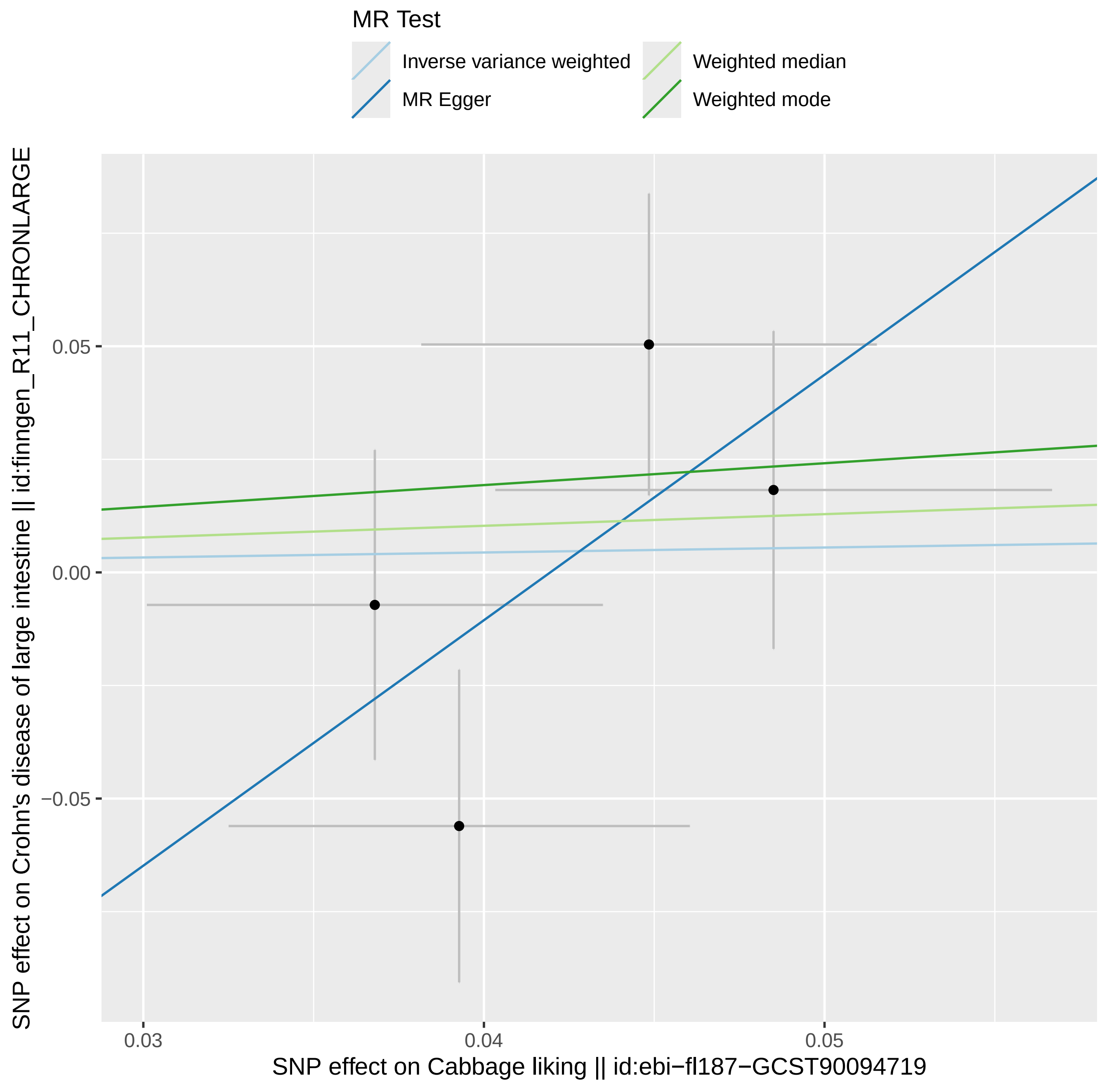
MR Test
Inverse variance weighted
MR Egger
Weighted median
Weighted mode
0.05
0.00
−0.05
0.03
0.04
0.05
SNP effect on Crohn's disease of large intestine || id:finngen_R11_CHRONLARGE
SNP effect on Cabbage liking || id:ebi−fI187−GCST90094719

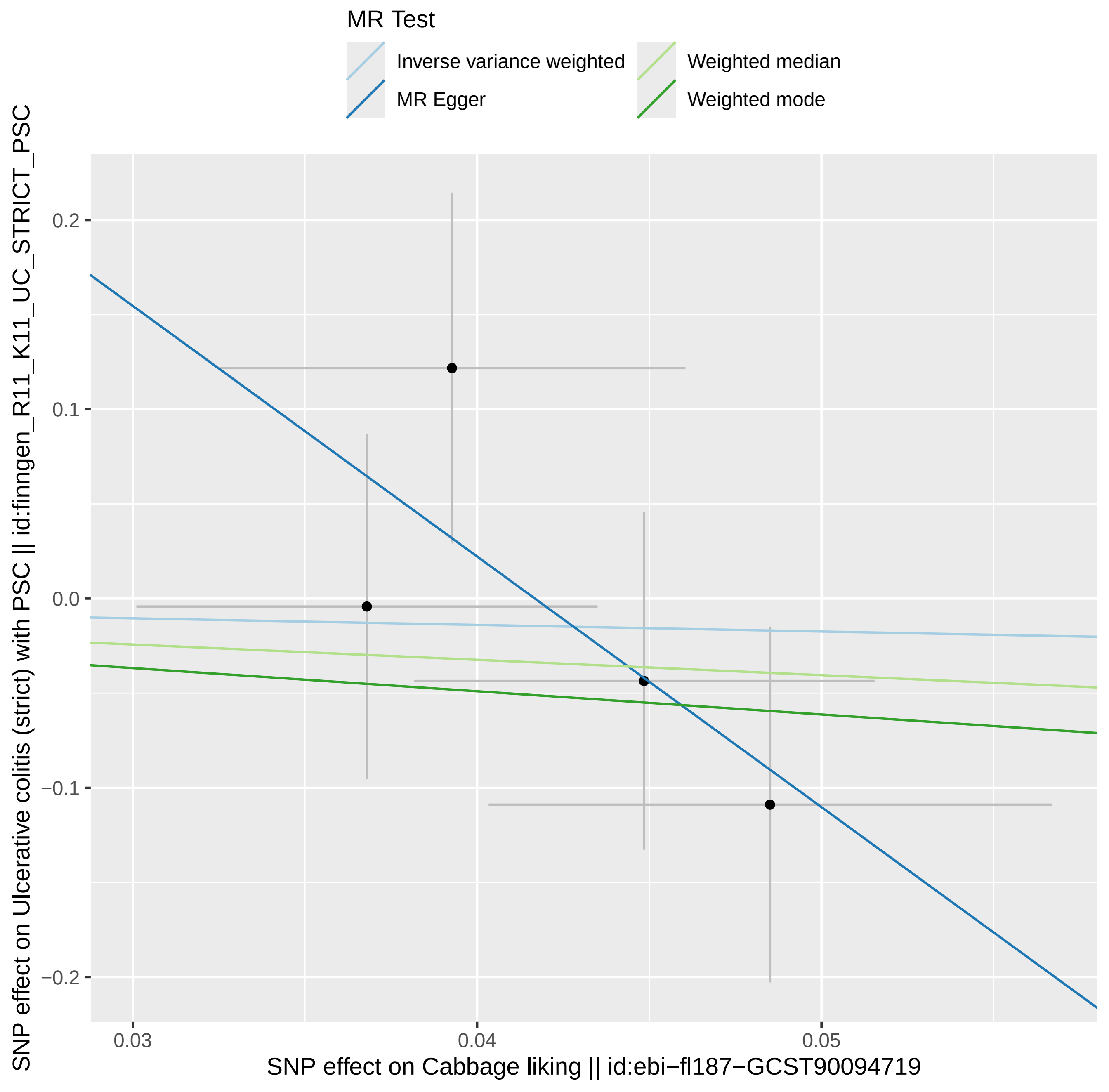

MR Test
Inverse variance weighted
MR Egger
Weighted median
Weighted mode
0.2
0.1
0.0
−0.1
−0.2
0.03
0.04
0.05
SNP effect on Ulcerative colitis (strict) with PSC || id:finngen_R11_K11_UC_STRICT_PSC
SNP effect on Cabbage liking || id:ebi−fl187−GCST90094719

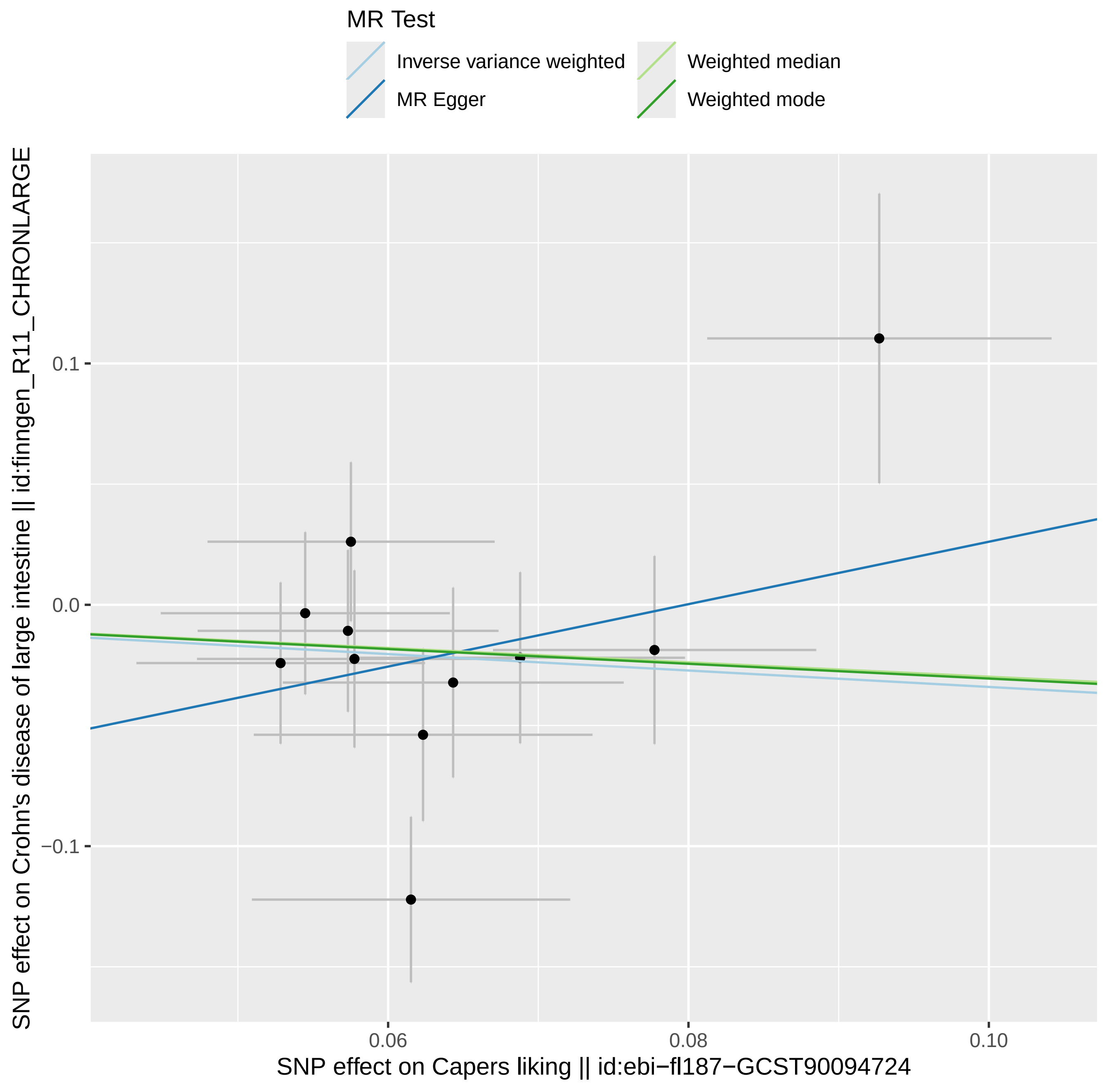
MR Test
Inverse variance weighted
MR Egger
Weighted median
Weighted mode
0.1
0.0
−0.1
0.06
0.08
0.10
SNP effect on Crohn's disease of large intestine || id:finngen_R11_CHRONLARGE
SNP effect on Capers liking || id:ebi−fl187−GCST90094724

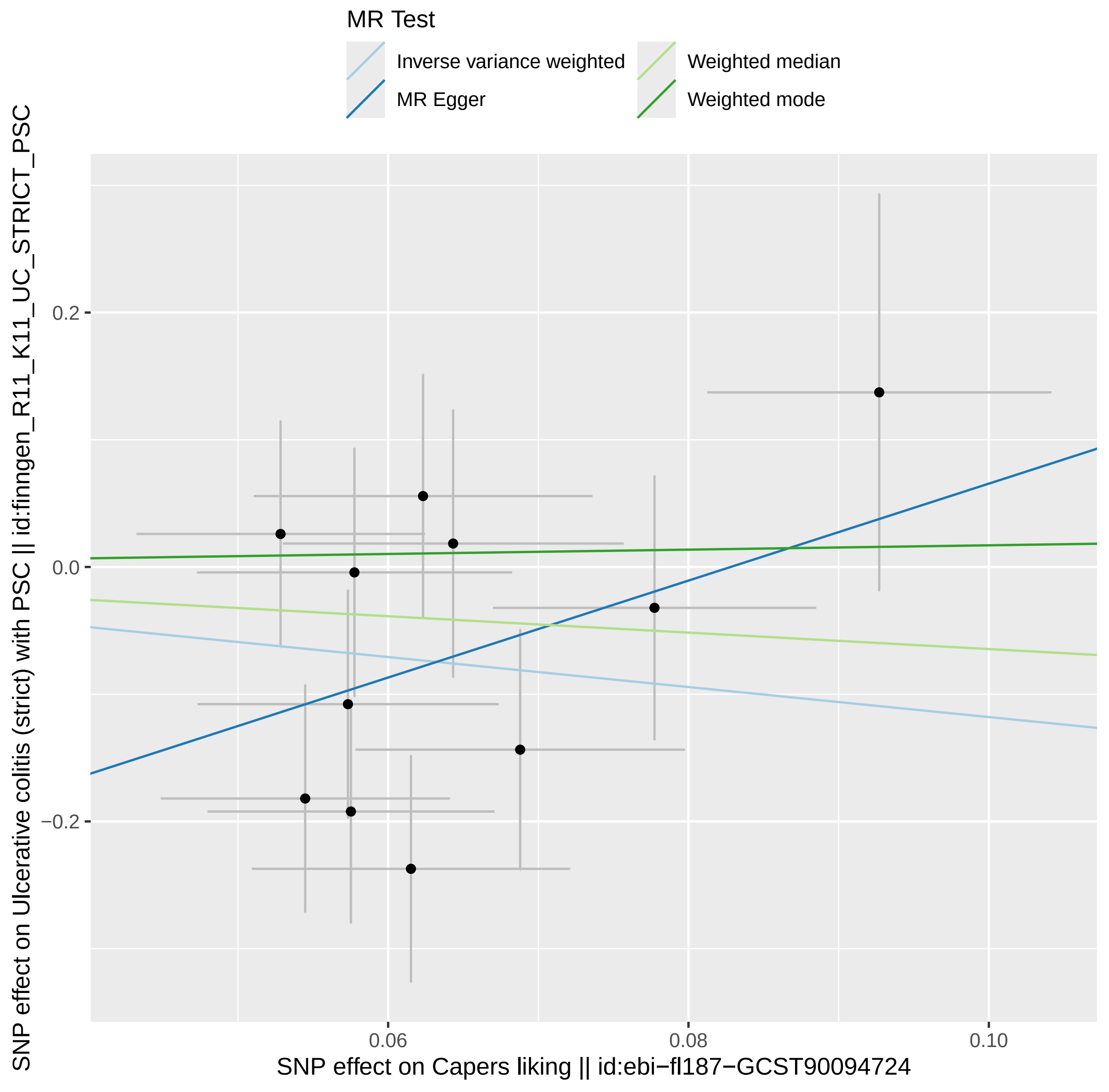

MR Test
Inverse variance weighted
MR Egger
Weighted median
Weighted mode
0.2
0.0
−0.2
0.06
0.08
0.10
SNP effect on Ulcerative colitis (strict) with PSC || id:finngen_R11_K11_UC_STRICT_PSC
SNP effect on Capers liking || id:ebi−fl187−GCST90094724

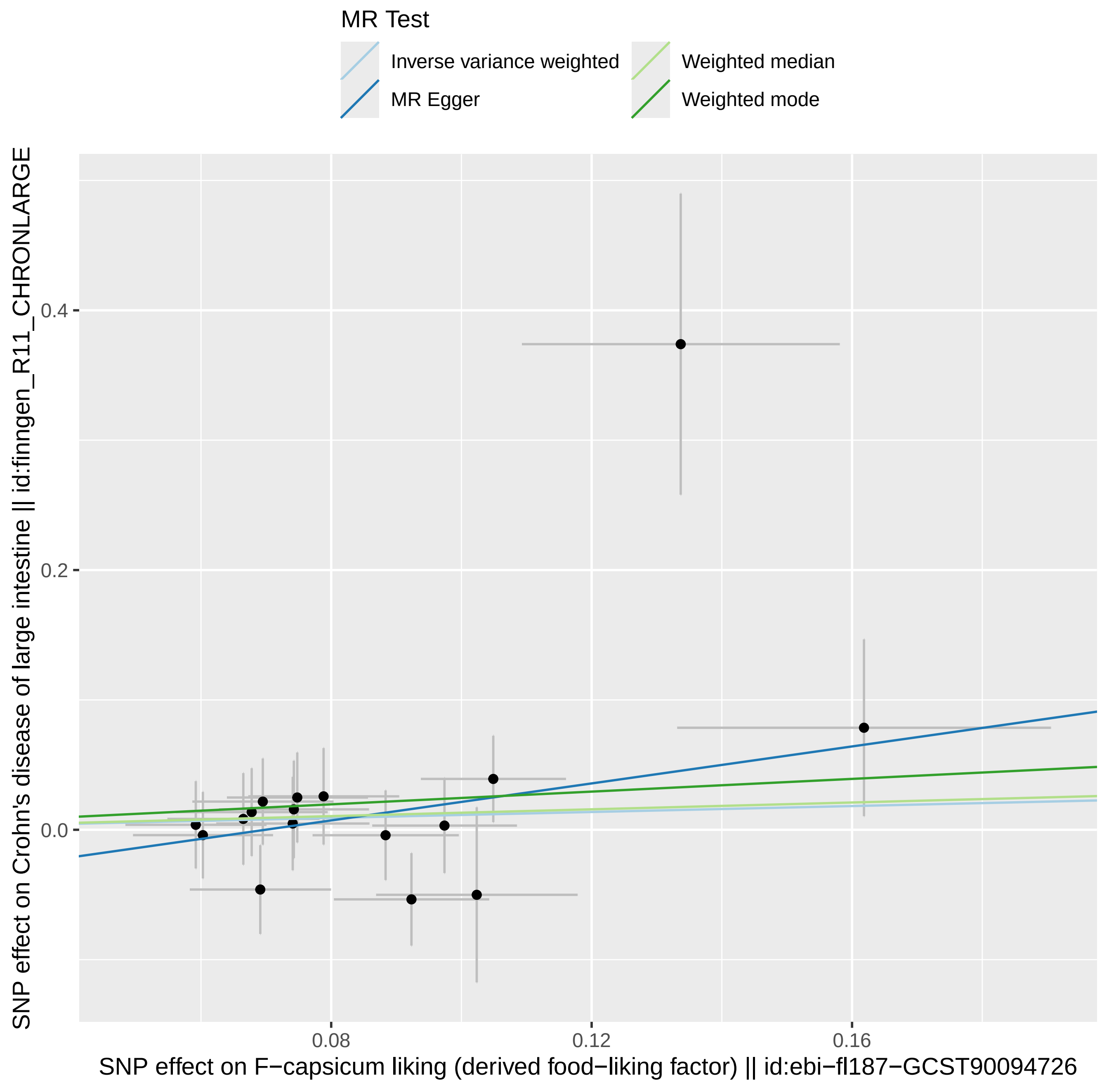

MR Test
Inverse variance weighted
MR Egger
Weighted median
Weighted mode
0.4
0.2
0.0
0.08
0.12
0.16
SNP effect on Crohn's disease of large intestine || id:finngen_R11_CHRONLARGE
SNP effect on F−capsicum liking (derived food−liking factor) || id:ebi−fl187−GCST90094726

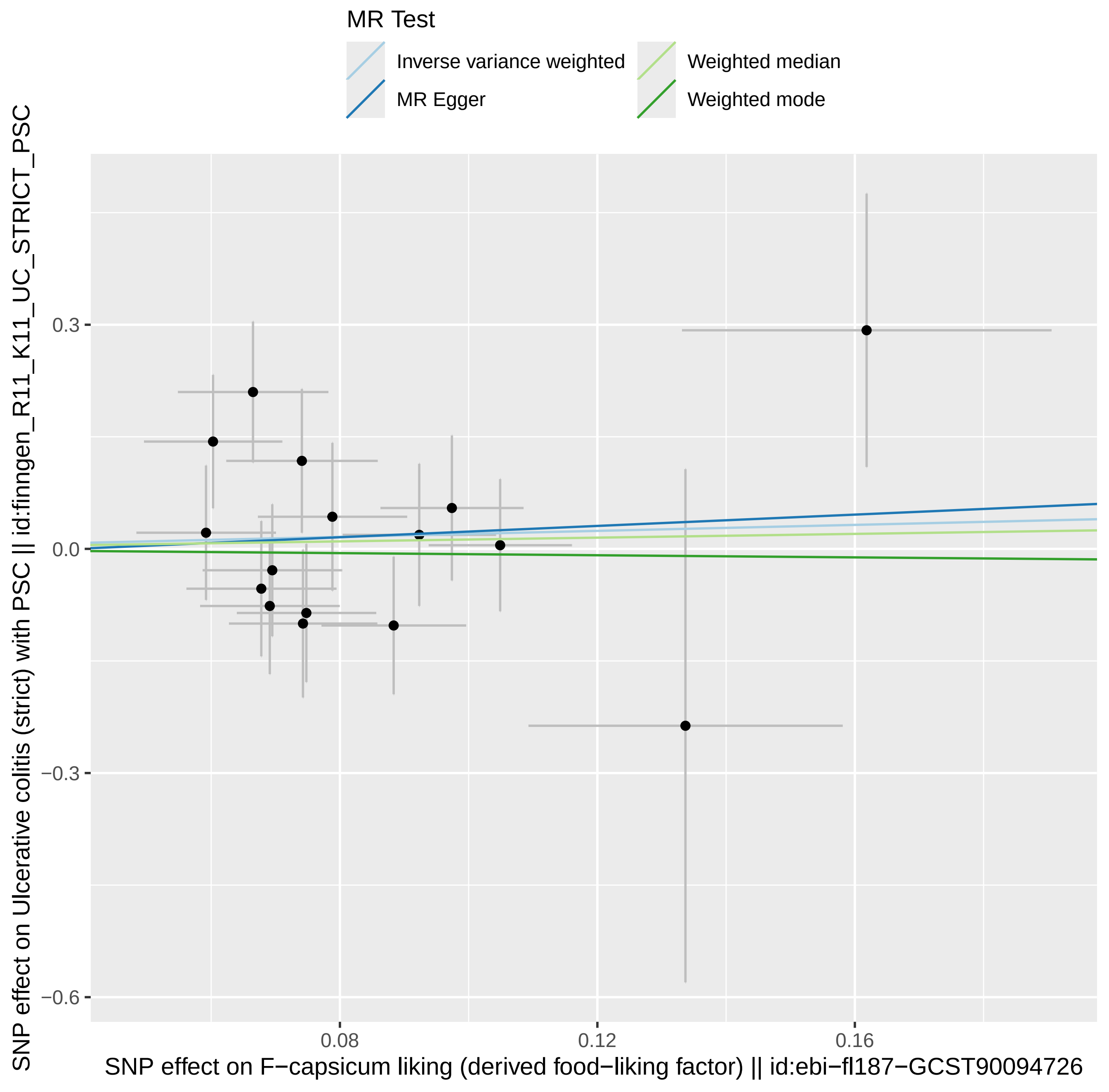
MR Test
Inverse variance weighted
MR Egger
Weighted median
Weighted mode
0.3
0.0
−0.3
−0.6
0.08
0.12
0.16
SNP effect on Ulcerative colitis (strict) with PSC || id:finngen_R11_K11_UC_STRICT_PSC
SNP effect on F−capsicum liking (derived food−liking factor) || id:ebi−fl187−GCST90094726

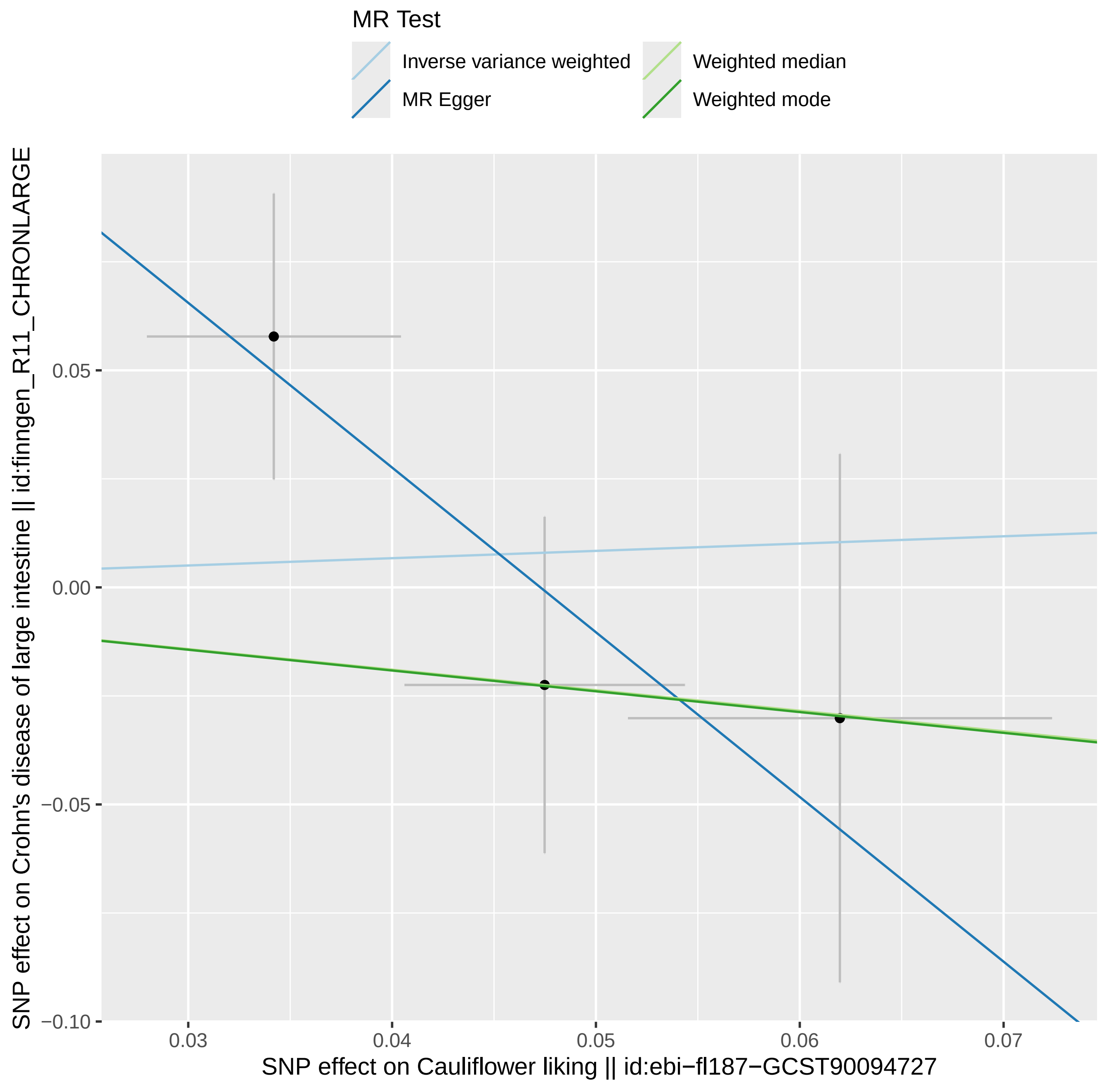
MR Test
Inverse variance weighted
MR Egger
Weighted median
Weighted mode
SNP effect on Crohn's disease of large intestine || id:finngen_R11_CHRONLARGE
0.05
0.00
−0.05
−0.10
0.03
0.04
0.05
0.06
0.07
SNP effect on Cauliflower liking || id:ebi−fl187−GCST90094727

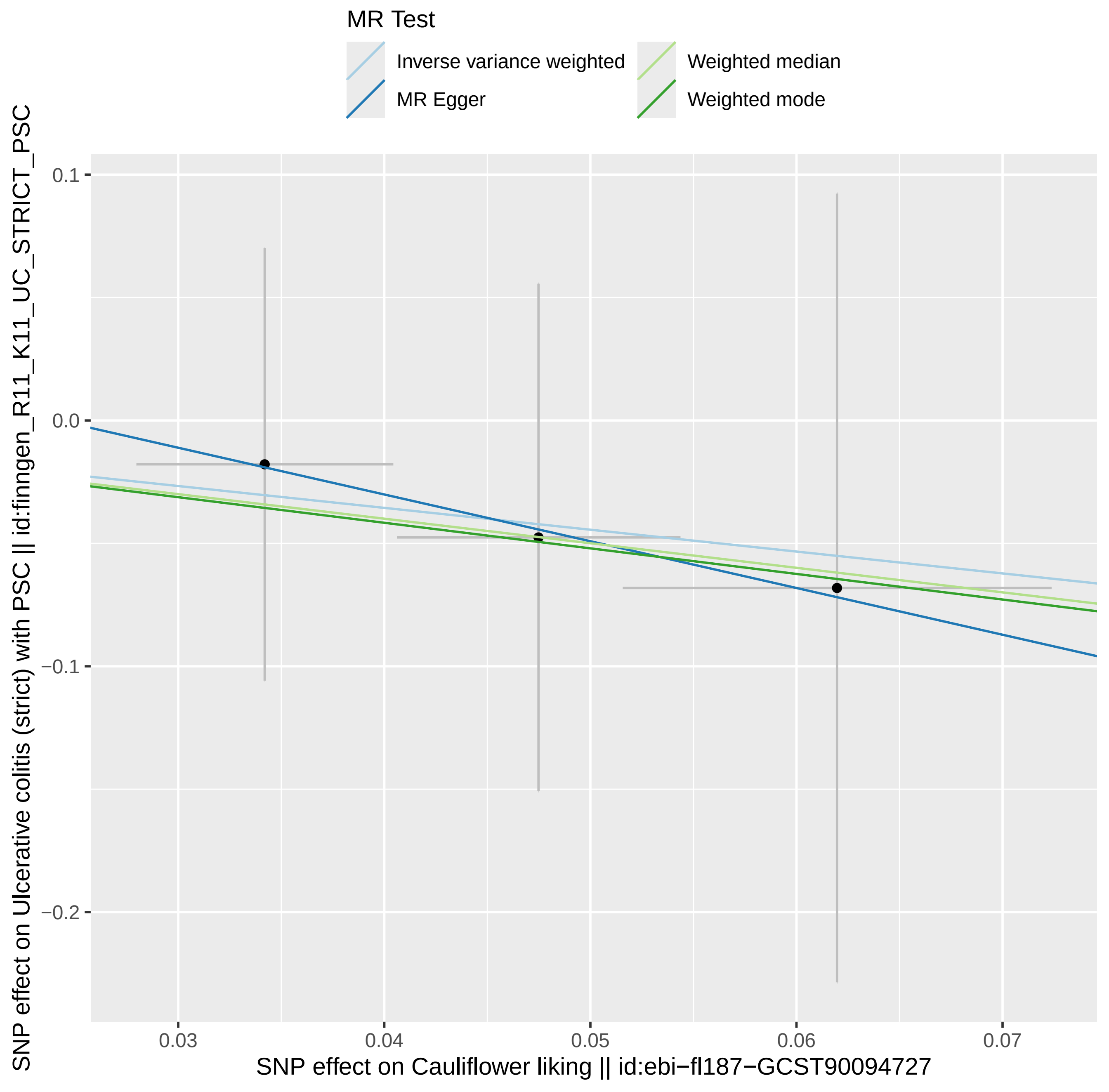
MR Test
Inverse variance weighted
MR Egger
Weighted median
Weighted mode
0.1
0.0
−0.1
−0.2
0.03
0.04
0.05
0.06
0.07
SNP effect on Ulcerative colitis (strict) with PSC || id:finngen_R11_K11_UC_STRICT_PSC
SNP effect on Cauliflower liking || id:ebi−fl187−GCST90094727

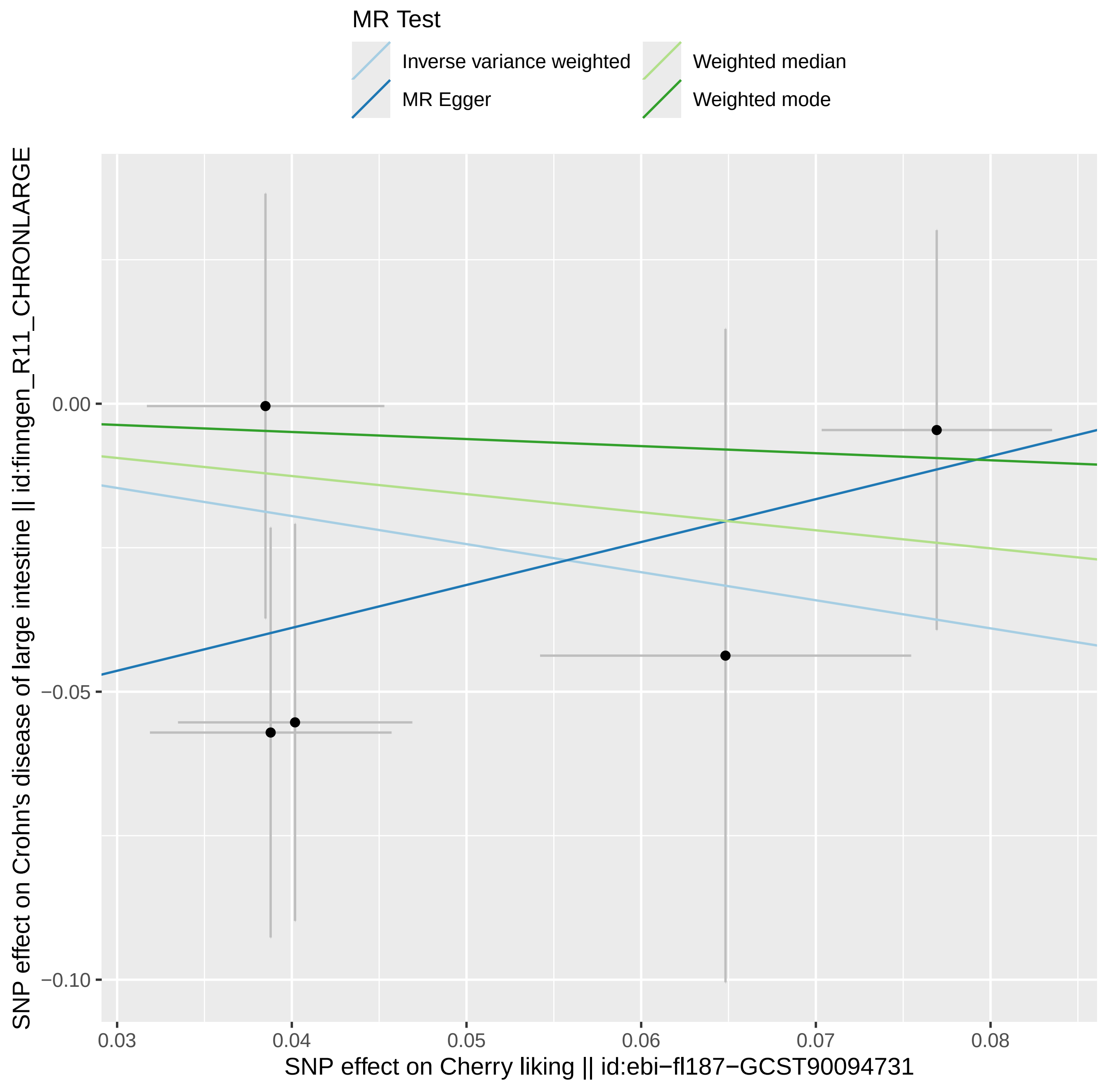

MR Test
Inverse variance weighted
MR Egger
Weighted median
Weighted mode
SNP effect on Crohn's disease of large intestine || id:finngen_R11_CHRONLARGE
0.00
−0.05
−0.10
0.03
0.04
0.05
0.06
0.07
0.08
SNP effect on Cherry liking || id:ebi−fl187−GCST90094731

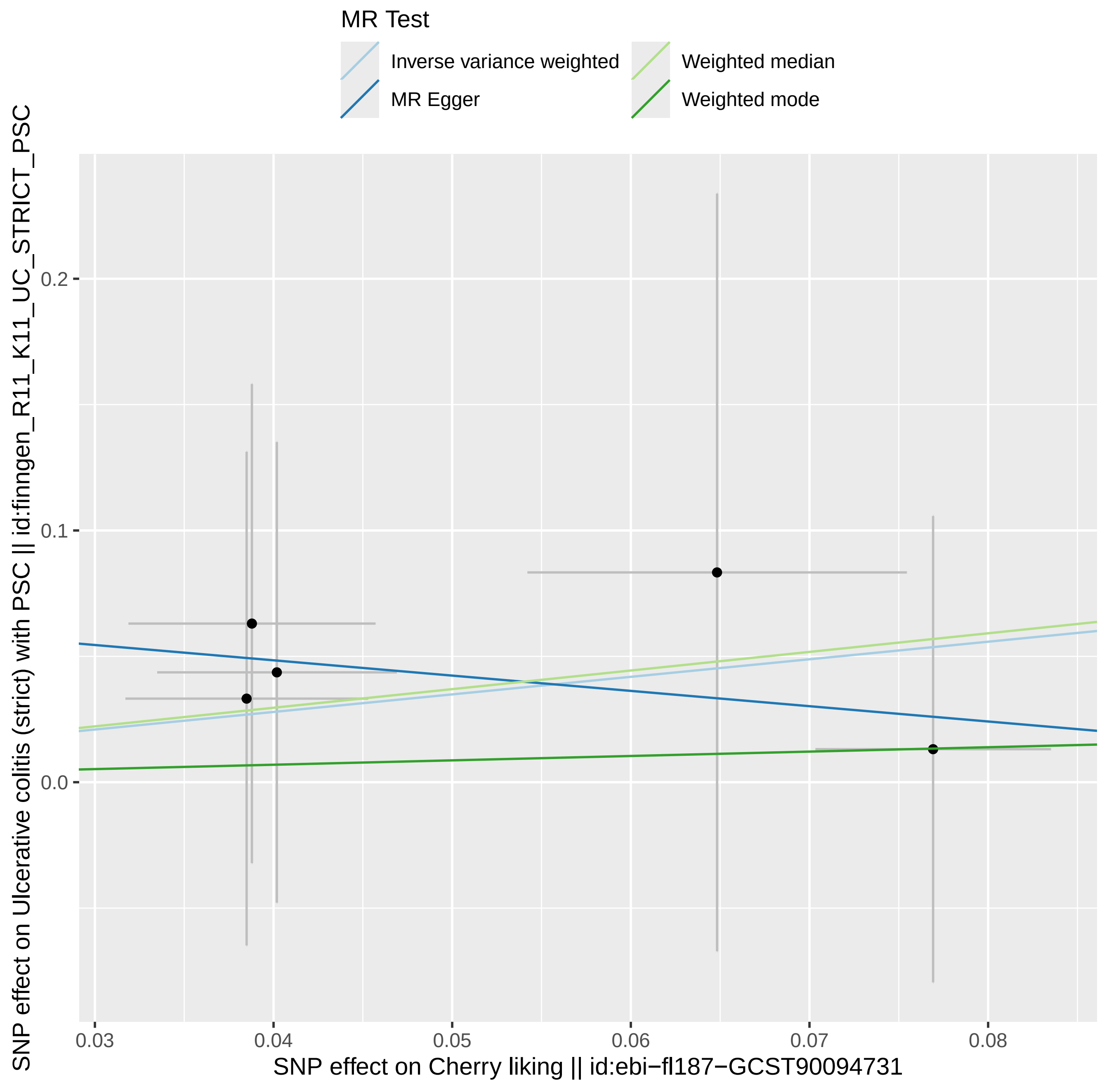
MR Test
Inverse variance weighted
MR Egger
Weighted median
Weighted mode
SNP effect on Ulcerative colitis (strict) with PSC || id:finngen_R11_K11_UC_STRICT_PSC
0.2
0.1
0.0
0.03
0.04
0.05
0.06
0.07
0.08
SNP effect on Cherry liking || id:ebi−fl187−GCST90094731

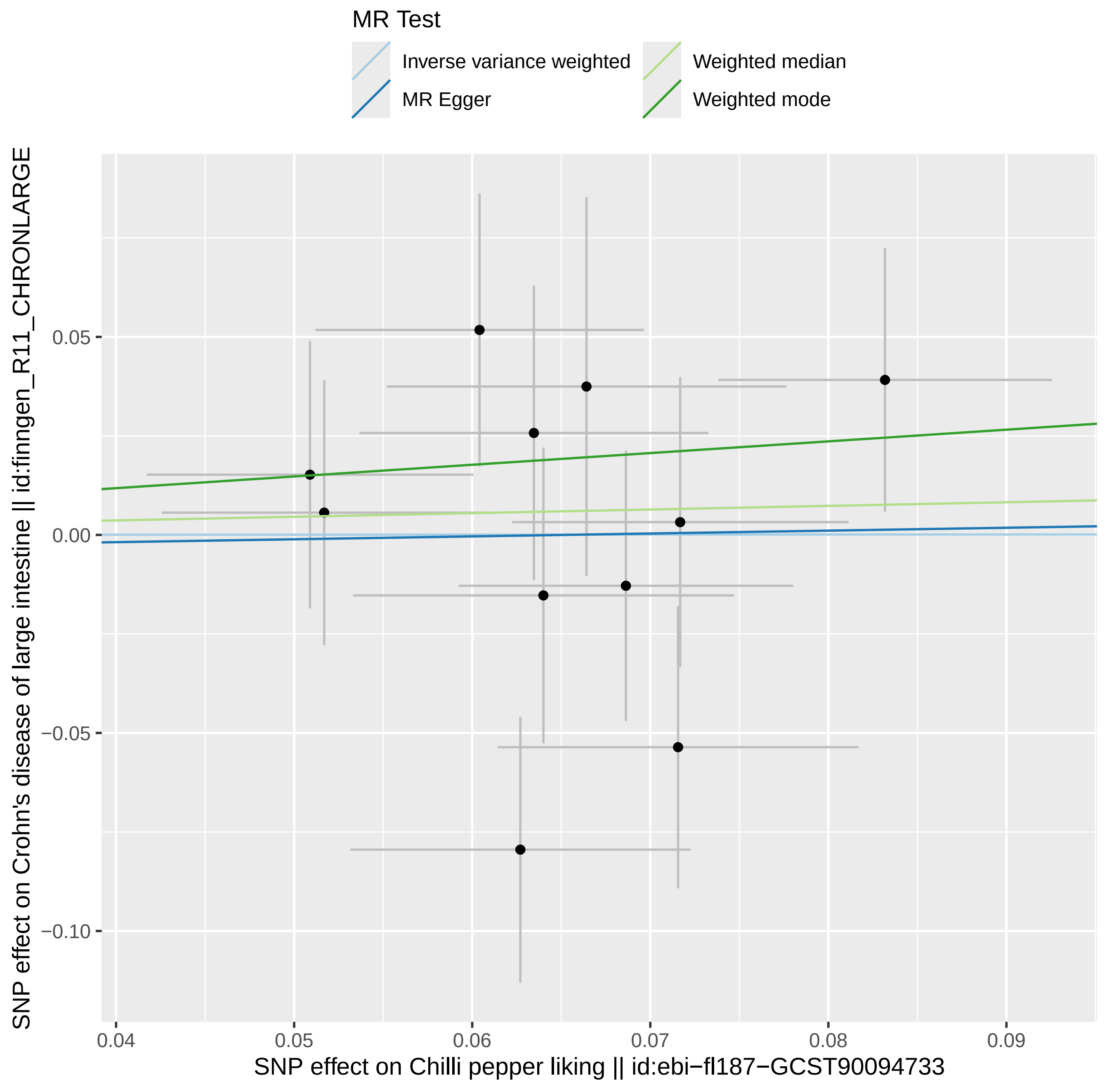
MR Test
Inverse variance weighted
MR Egger
Weighted median
Weighted mode
SNP effect on Crohn's disease of large intestine || id:finngen_R11_CHRONLARGE
0.05
0.00
−0.05
−0.10
0.04
0.05
0.06
0.07
0.08
0.09
SNP effect on Chilli pepper liking || id:ebi−fl187−GCST90094733

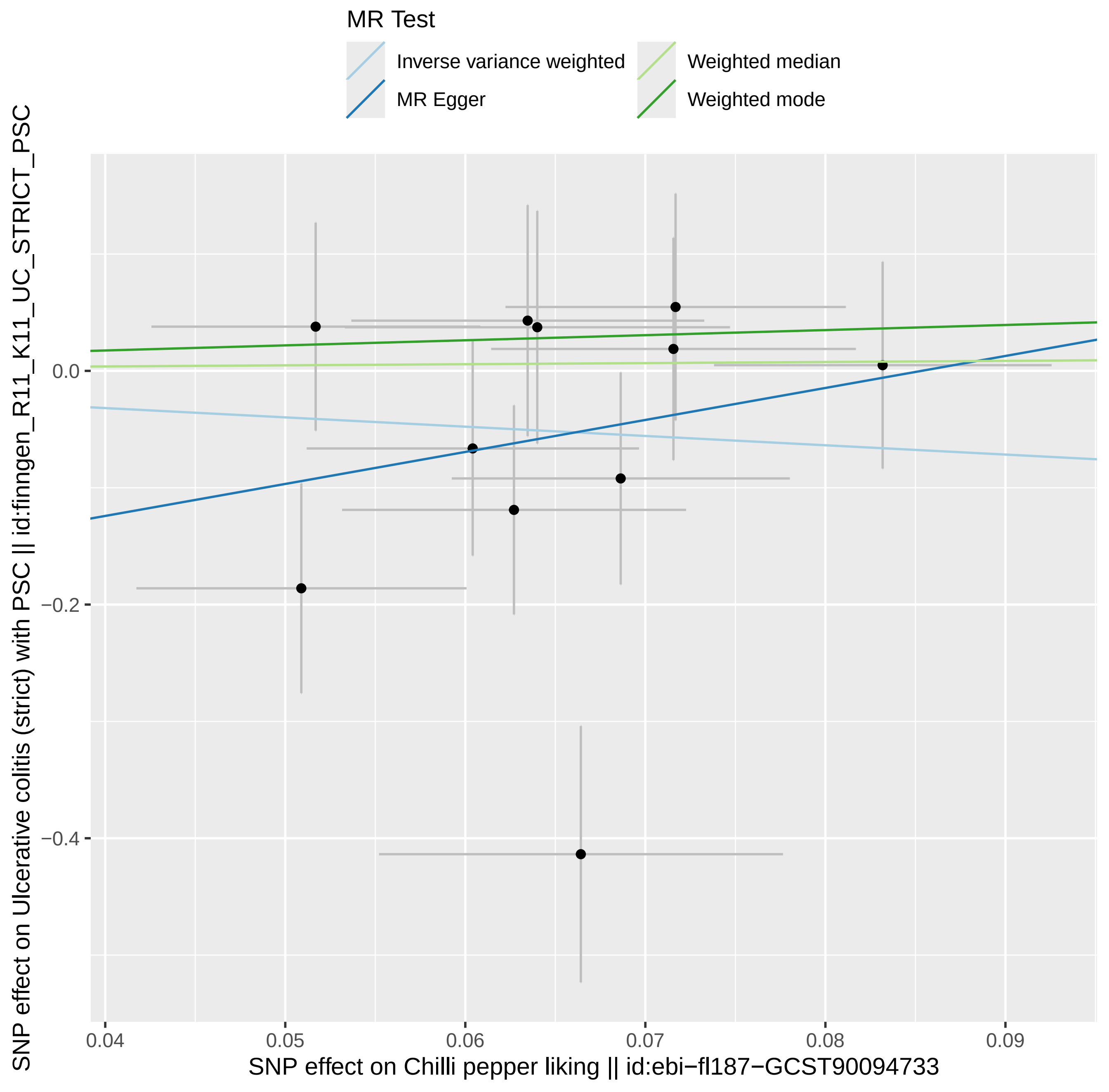

MR Test
Inverse variance weighted
MR Egger
Weighted median
Weighted mode
0.0
−0.2
−0.4
0.04
0.05
0.06
0.07
0.08
0.09
SNP effect on Chilli pepper liking || id:ebi−fl187−GCST90094733
SNP effect on Ulcerative colitis (strict) with PSC || id:finngen_R11_K11_UC_STRICT_PSC

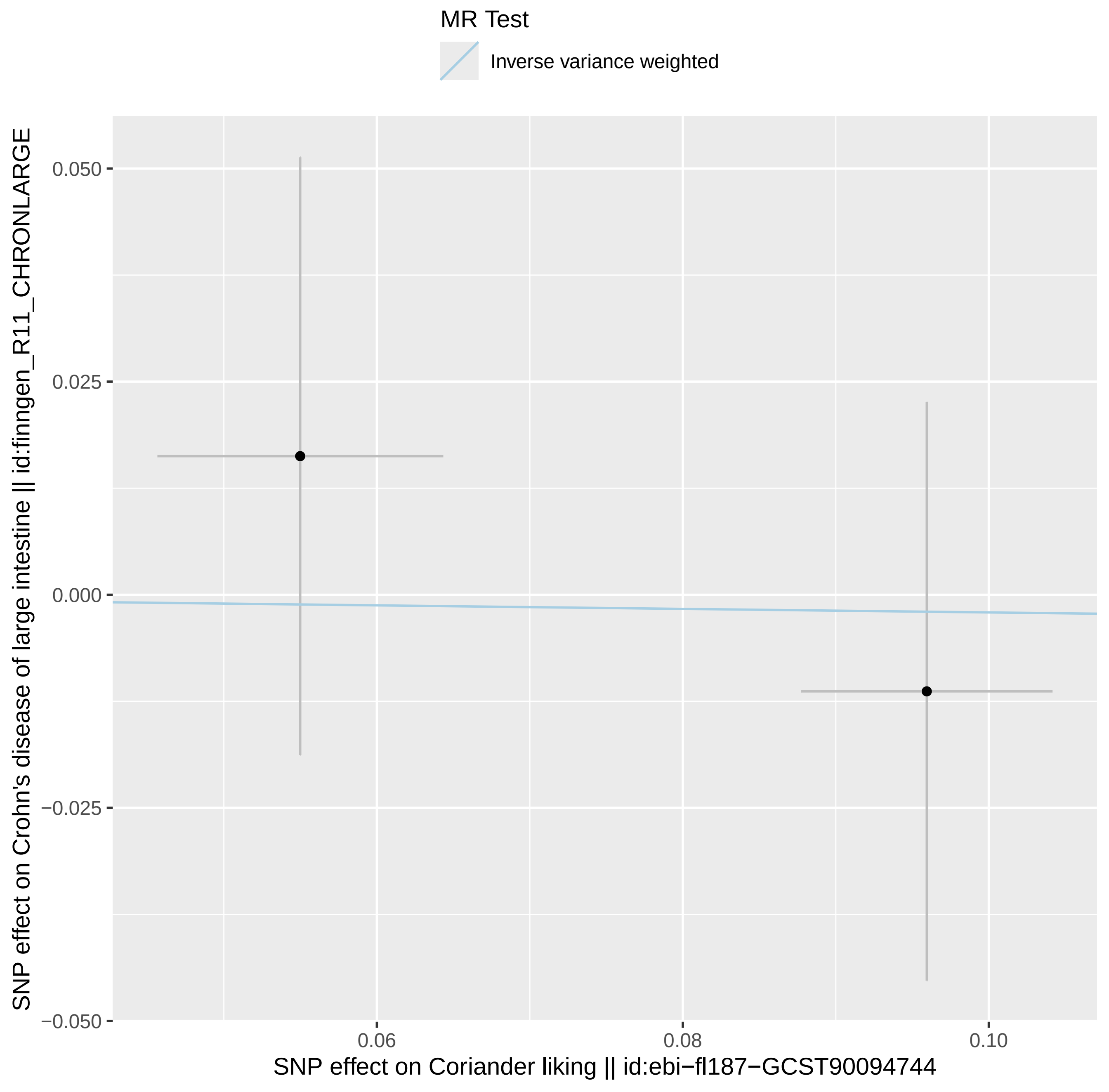
MR Test
Inverse variance weighted
SNP effect on Crohn's disease of large intestine || id:finngen_R11_CHRONLARGE
0.050
0.025
0.000
−0.025
−0.050
0.06
0.08
0.10
SNP effect on Coriander liking || id:ebi−fl187−GCST90094744

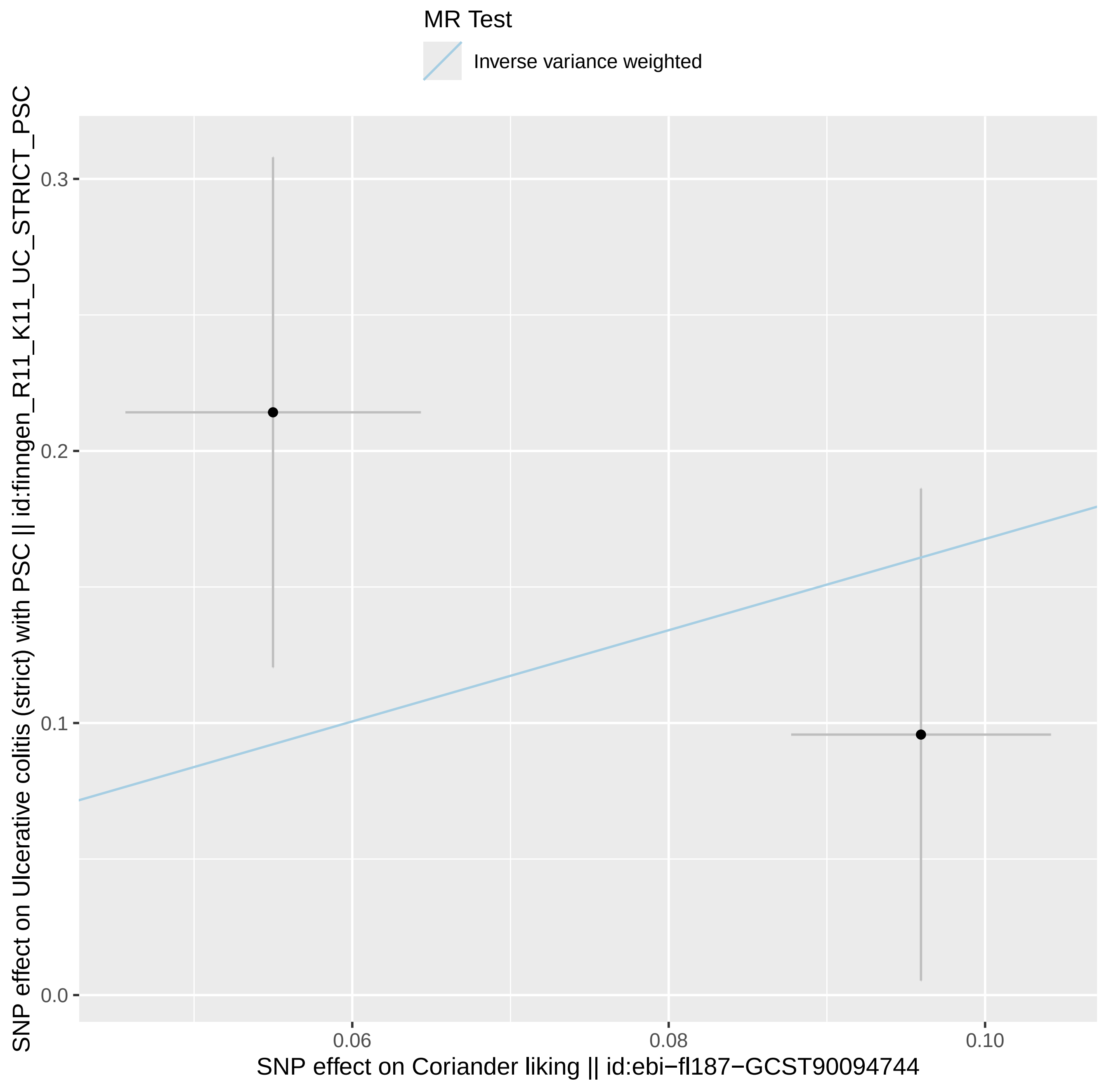
MR Test
Inverse variance weighted
SNP effect on Ulcerative colitis (strict) with PSC || id:finngen_R11_K11_UC_STRICT_PSC
0.0
0.1
0.2
0.3
0.06
0.08
0.10
SNP effect on Coriander liking || id:ebi−fl187−GCST90094744

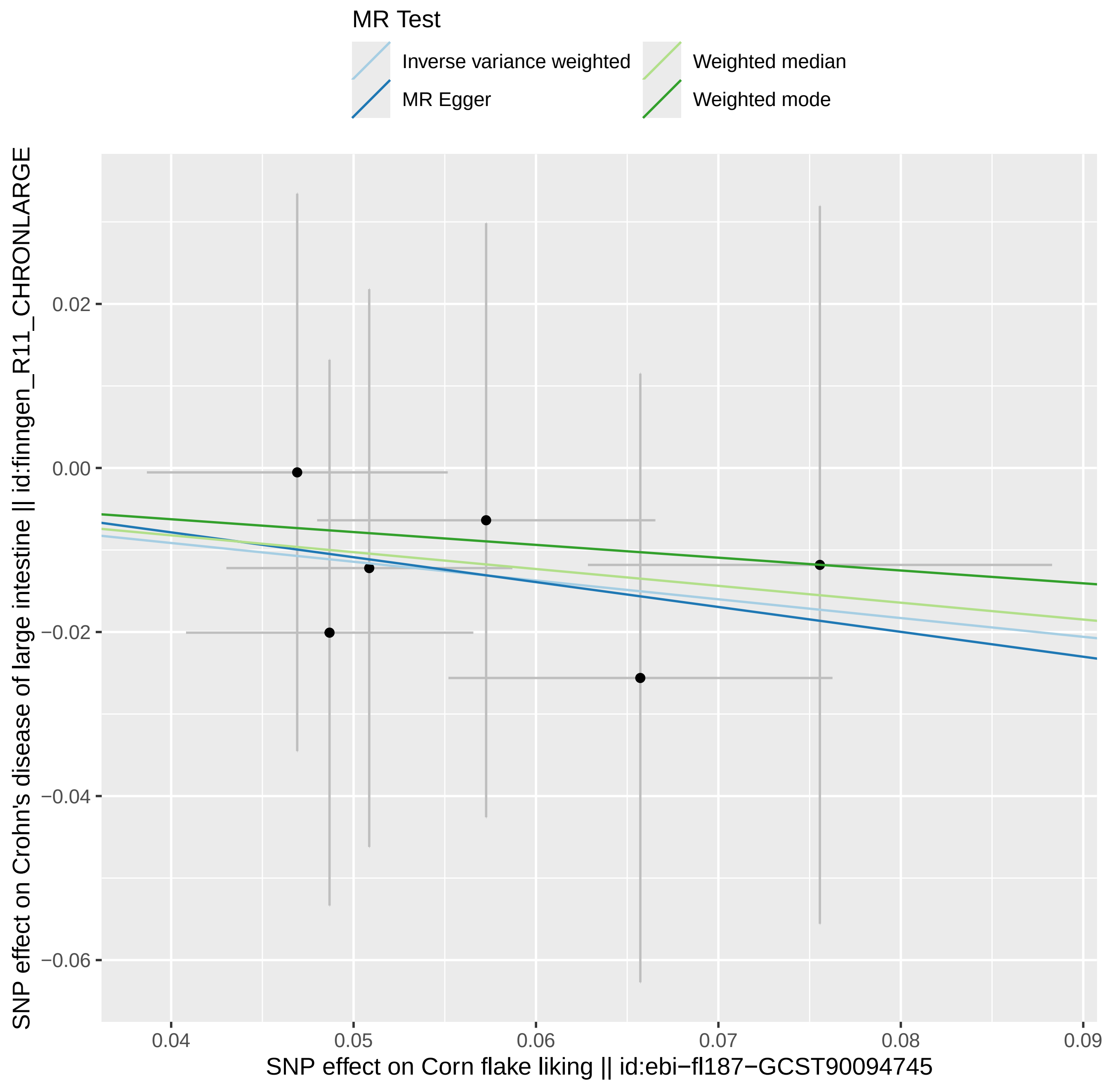
MR Test
Inverse variance weighted
MR Egger
Weighted median
Weighted mode
0.02
0.00
−0.02
−0.04
−0.06
0.04
0.05
0.06
0.07
0.08
0.09
SNP effect on Crohn's disease of large intestine || id:finngen_R11_CHRONLARGE
SNP effect on Corn flake liking || id:ebi−fl187−GCST90094745

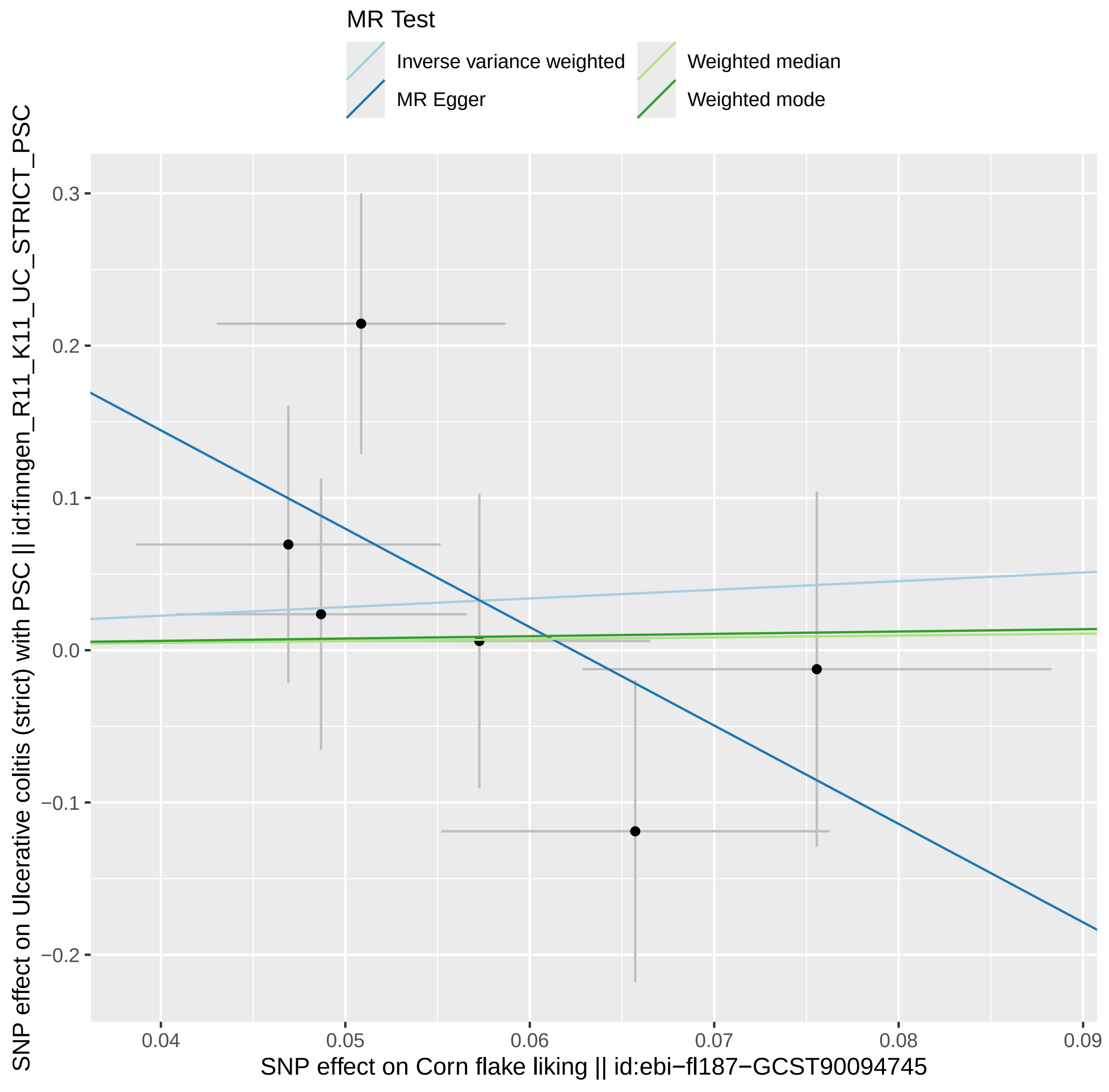

MR Test
Inverse variance weighted
MR Egger
Weighted median
Weighted mode
0.3
0.2
0.1
0.0
−0.1
−0.2
0.04
0.05
0.06
0.07
0.08
0.09
SNP effect on Ulcerative colitis (strict) with PSC || id:finngen_R11_K11_UC_STRICT_PSC
SNP effect on Corn flake liking || id:ebi−fl187−GCST90094745

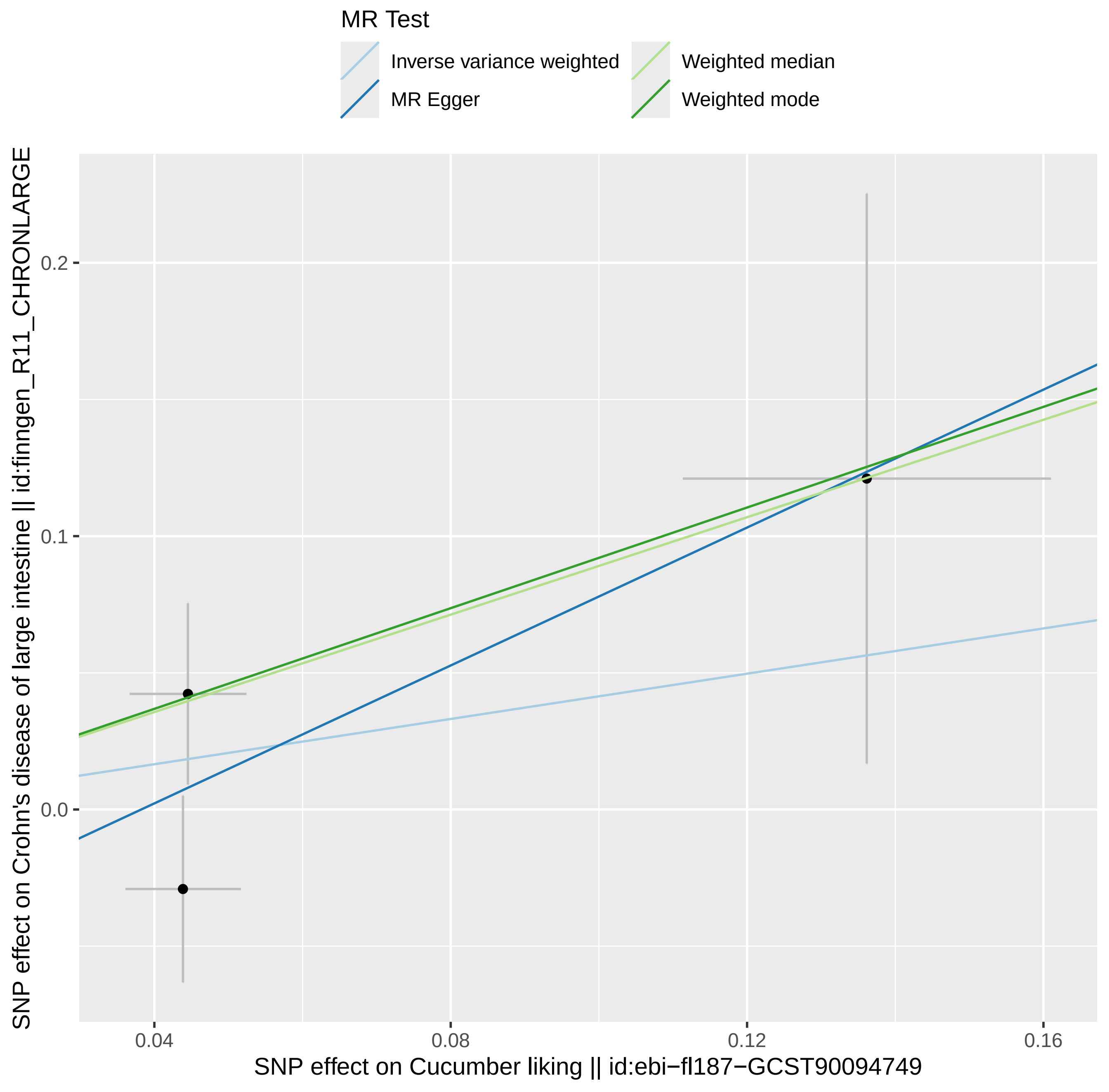
MR Test
Inverse variance weighted
MR Egger
Weighted median
Weighted mode
SNP effect on Crohn's disease of large intestine || id:finngen_R11_CHRONLARGE
0.2
0.1
0.0
0.04
0.08
0.12
0.16
SNP effect on Cucumber liking || id:ebi−fl187−GCST90094749

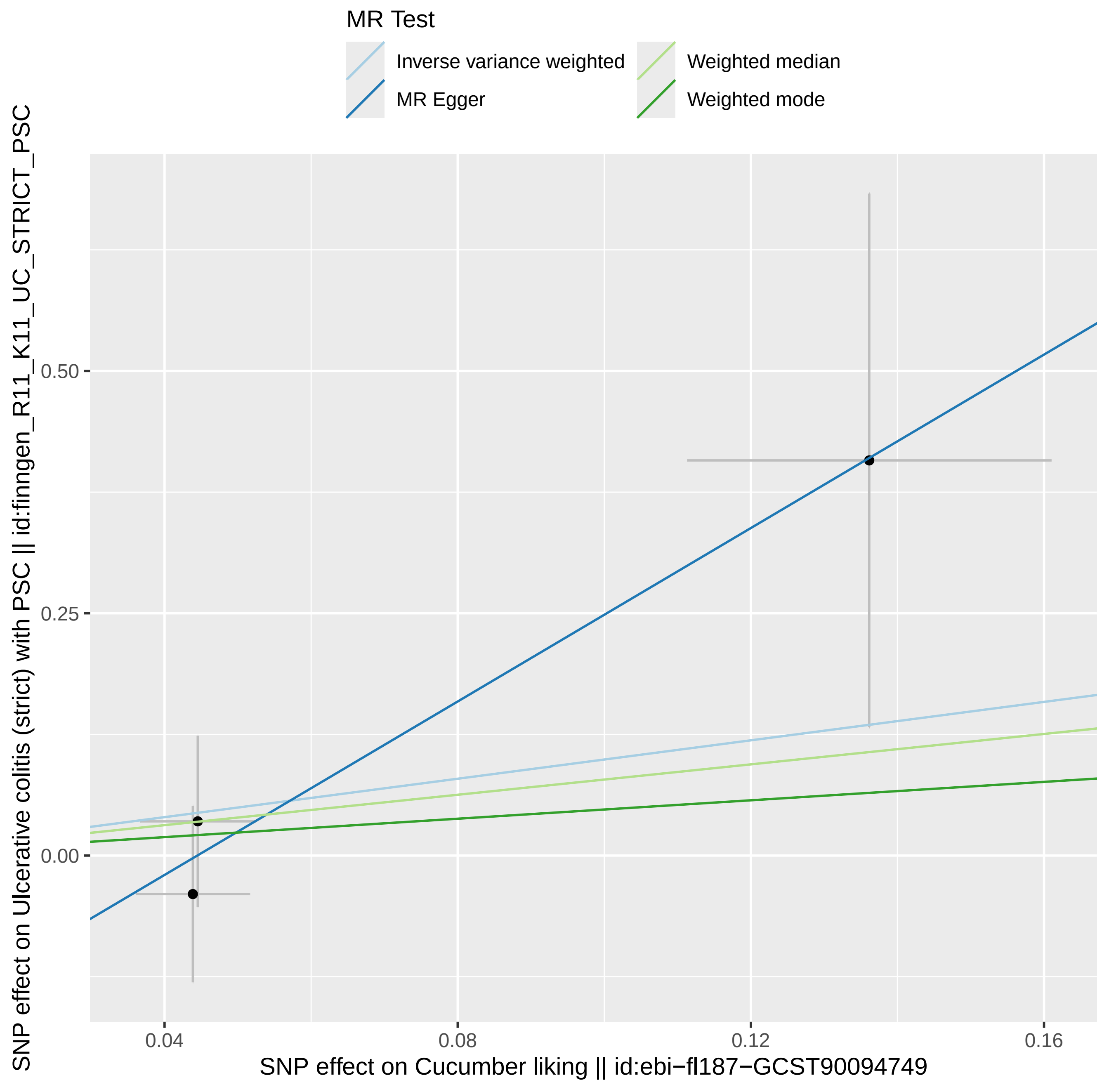
MR Test
Inverse variance weighted
MR Egger
Weighted median
Weighted mode
0.50
0.25
0.00
0.04
0.08
0.12
0.16
SNP effect on Ulcerative colitis (strict) with PSC || id:finngen_R11_K11_UC_STRICT_PSC
SNP effect on Cucumber liking || id:ebi−fl187−GCST90094749

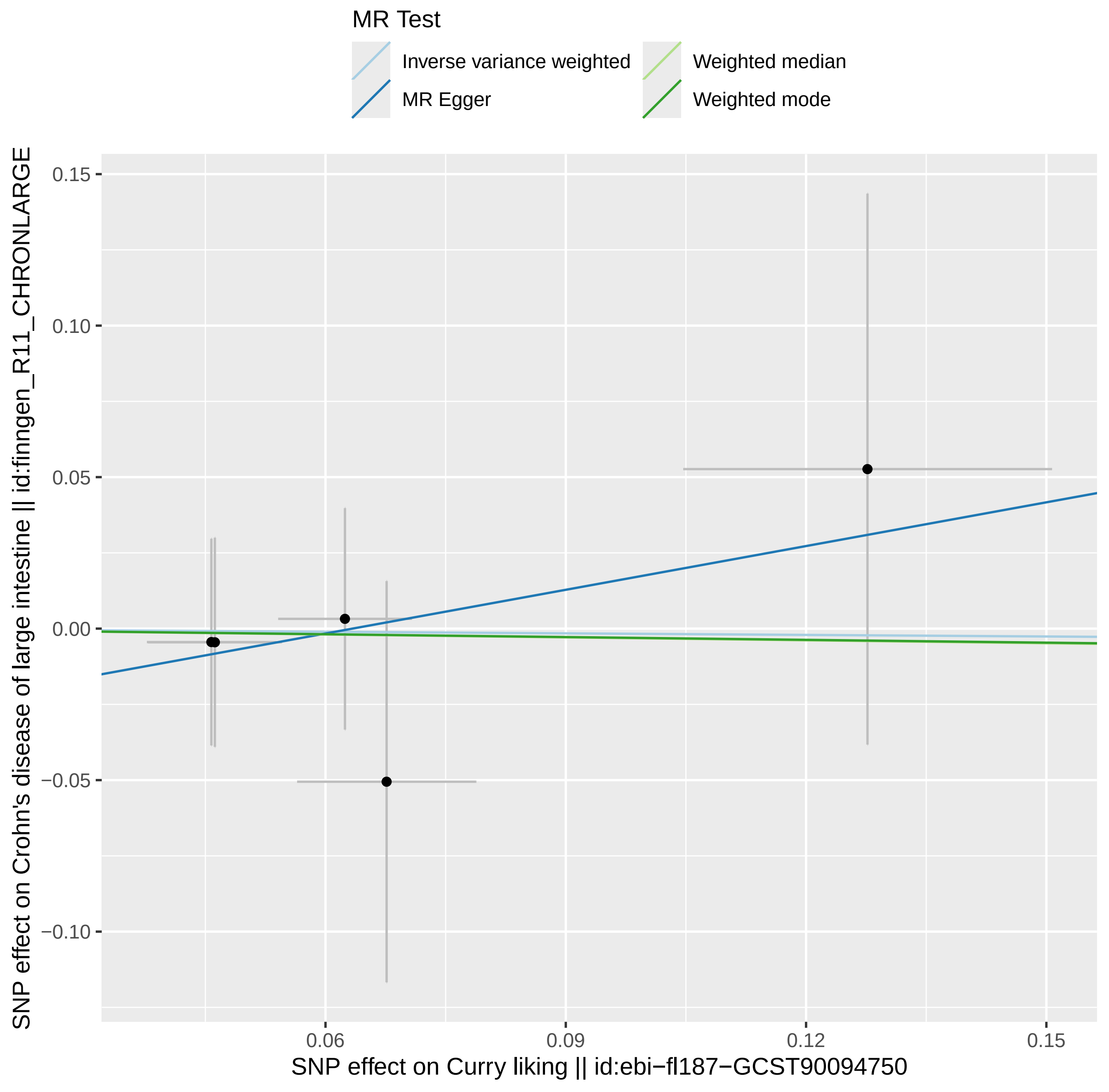
MR Test
Inverse variance weighted
MR Egger
Weighted median
Weighted mode
SNP effect on Crohn's disease of large intestine || id:finngen_R11_CHRONLARGE
0.15
0.10
0.05
0.00
−0.05
−0.10
0.06
0.09
0.12
0.15
SNP effect on Curry liking || id:ebi−fl187−GCST90094750

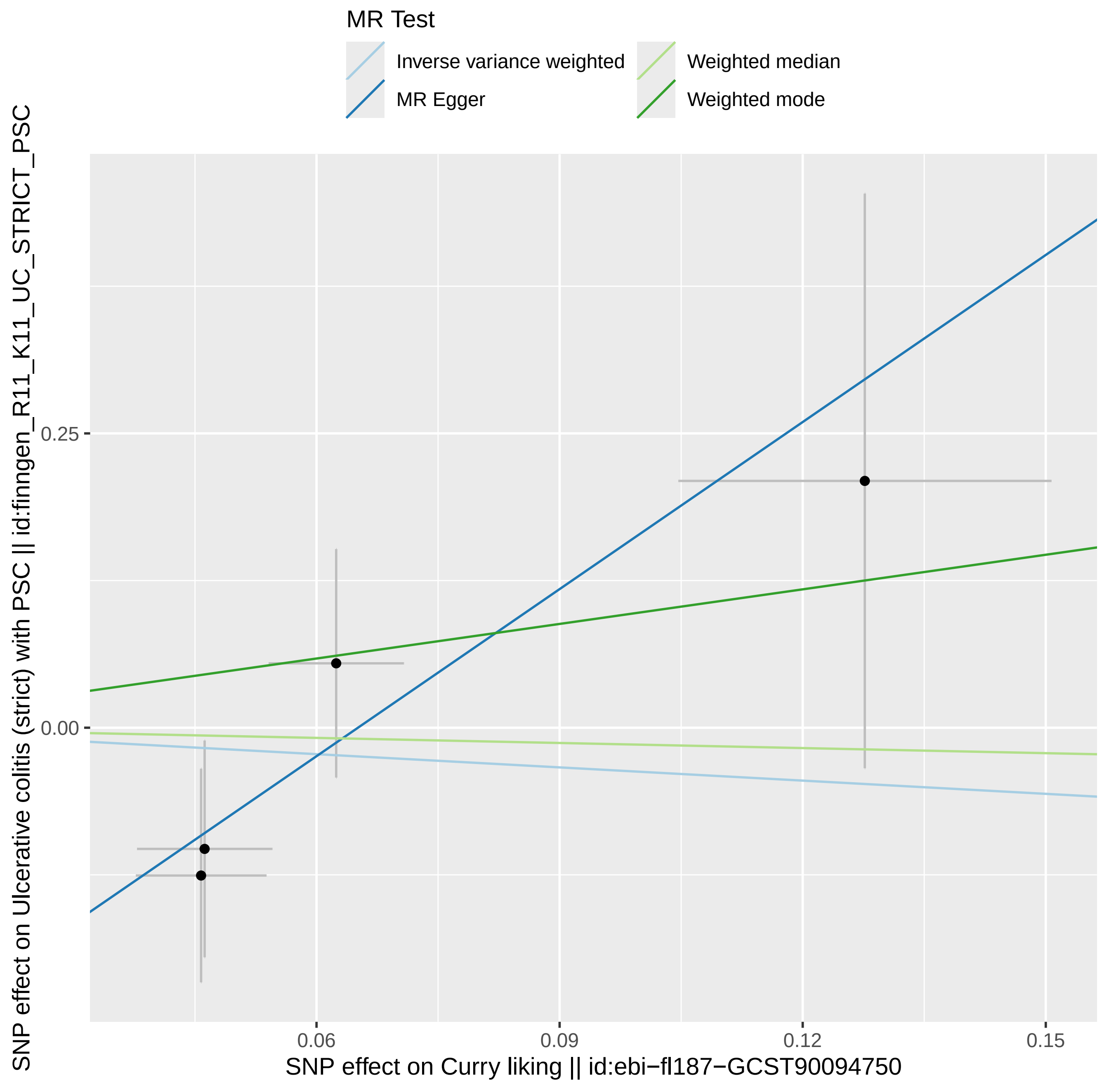

MR Test
Inverse variance weighted
MR Egger
Weighted median
Weighted mode
0.25
0.00
0.06
0.09
0.12
0.15
SNP effect on Ulcerative colitis (strict) with PSC || id:finngen_R11_K11_UC_STRICT_PSC
SNP effect on Curry liking || id:ebi−fl187−GCST90094750

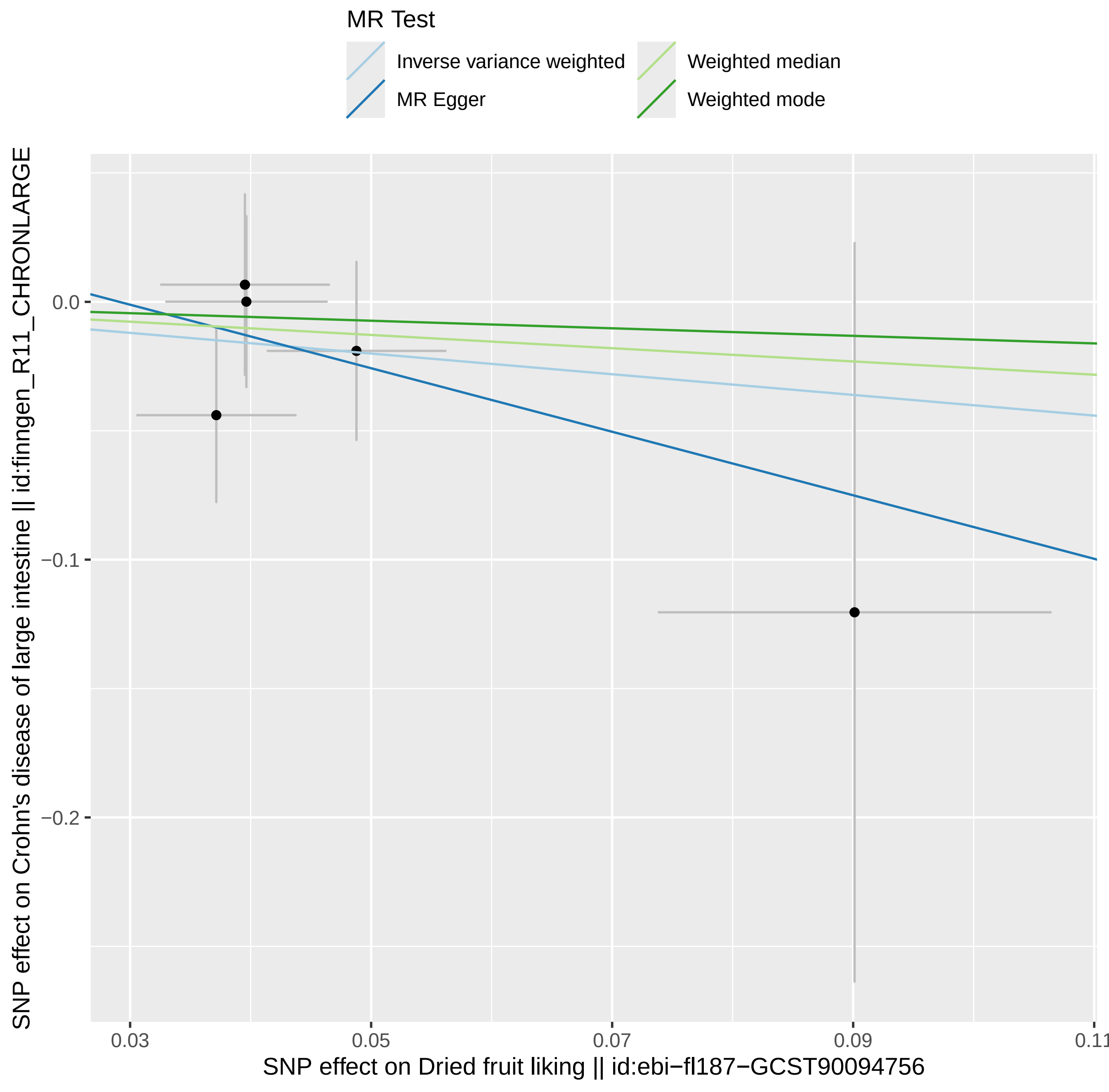
MR Test
Inverse variance weighted
MR Egger
Weighted median
Weighted mode
SNP effect on Crohn's disease of large intestine || id:finngen_R11_CHRONLARGE
0.0
−0.1
−0.2
0.03
0.05
0.07
0.09
0.11
SNP effect on Dried fruit liking || id:ebi−fl187−GCST90094756

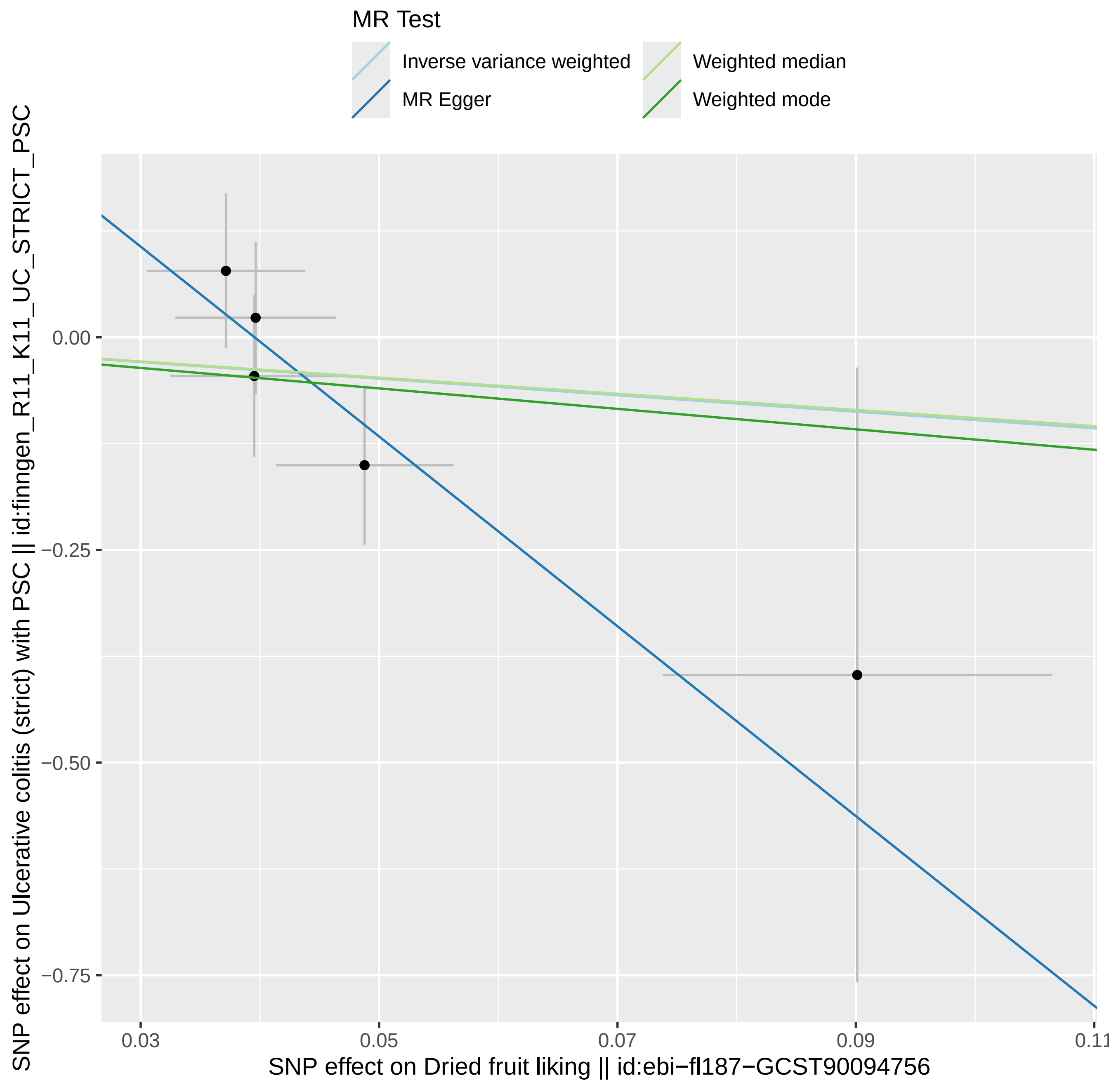

MR Test
Inverse variance weighted
MR Egger
Weighted median
Weighted mode
0.00
−0.25
−0.50
−0.75
0.03
0.05
0.07
0.09
0.11
SNP effect on Dried fruit liking || id:ebi−fl187−GCST90094756
SNP effect on Ulcerative colitis (strict) with PSC || id:finngen_R11_K11_UC_STRICT_PSC

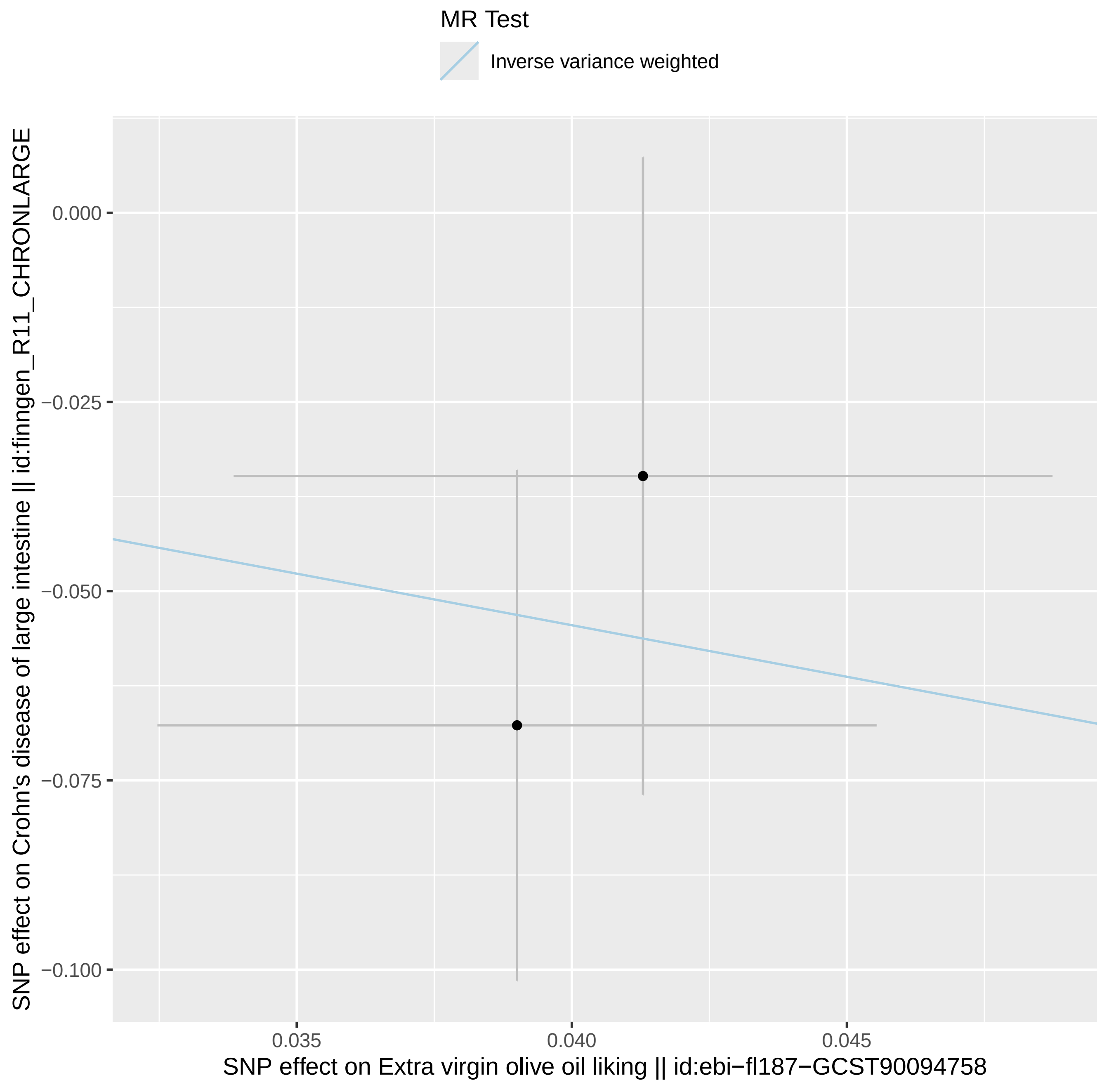
MR Test
Inverse variance weighted
SNP effect on Crohn's disease of large intestine || id:finngen_R11_CHRONLARGE
0.000
−0.025
−0.050
−0.075
−0.100
0.035
0.040
0.045
SNP effect on Extra virgin olive oil liking || id:ebi−fl187−GCST90094758

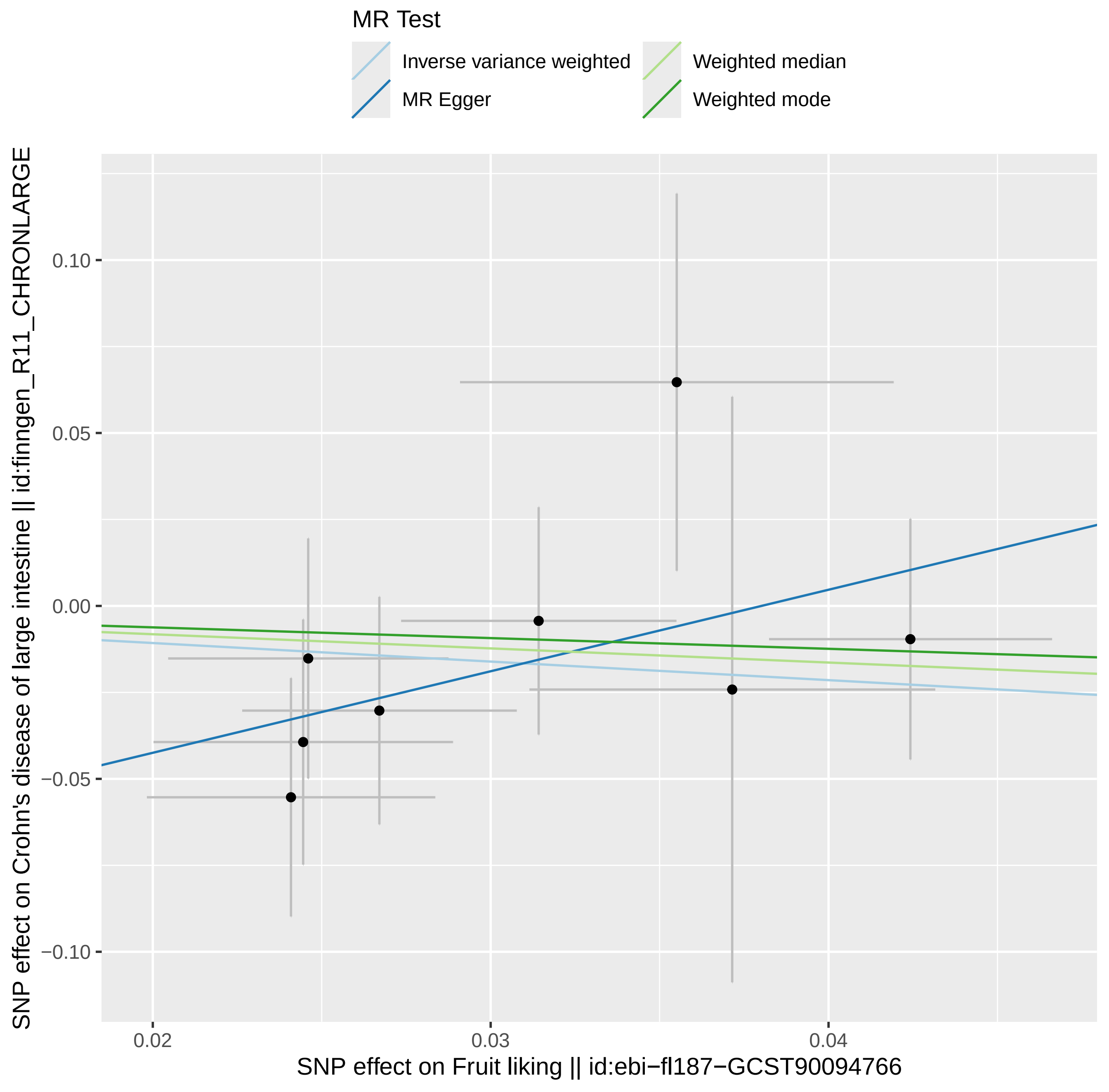

MR Test
Inverse variance weighted
MR Egger
Weighted median
Weighted mode
0.10
0.05
0.00
−0.05
−0.10
0.02
0.03
0.04
SNP effect on Crohn's disease of large intestine || id:finngen_R11_CHRONLARGE
SNP effect on Fruit liking || id:ebi−fl187−GCST90094766

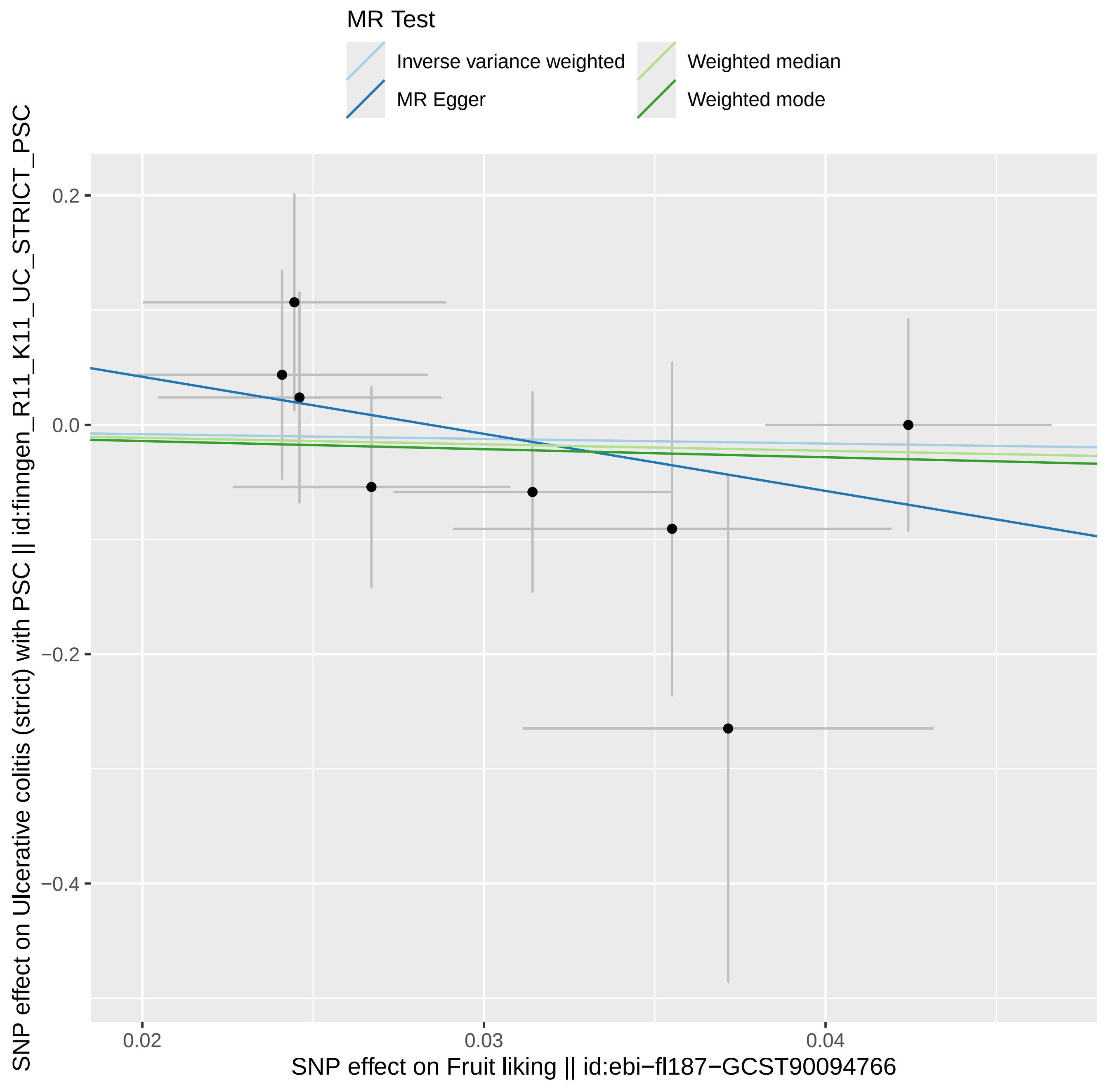

MR Test
Inverse variance weighted
MR Egger
Weighted median
Weighted mode
0.2
0.0
−0.2
−0.4
0.02
0.03
0.04
SNP effect on Ulcerative colitis (strict) with PSC || id:finngen_R11_K11_UC_STRICT_PSC
SNP effect on Fruit liking || id:ebi−fl187−GCST90094766

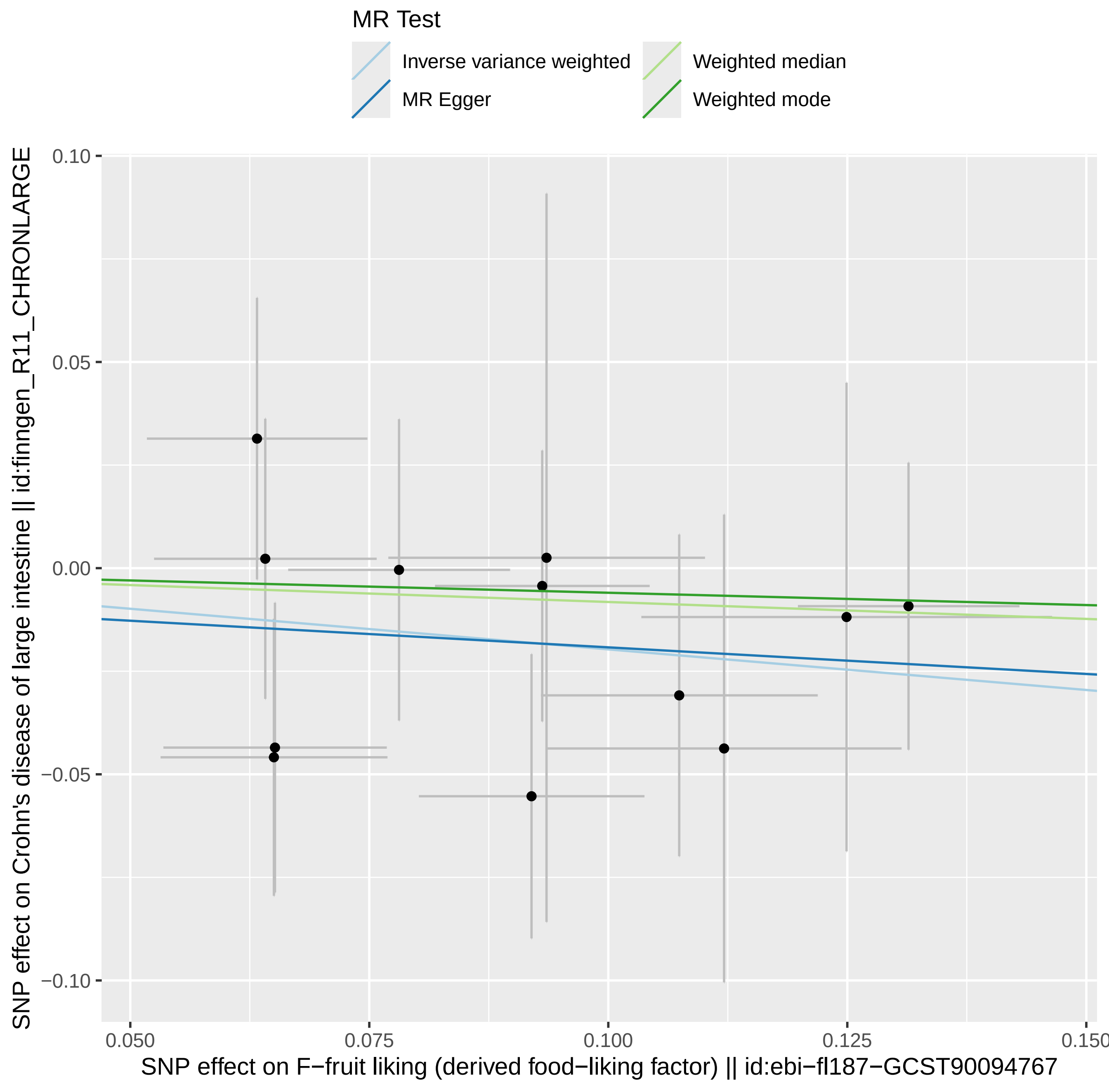

MR Test
Inverse variance weighted
MR Egger
Weighted median
Weighted mode
0.10
0.05
0.00
−0.05
−0.10
0.050
0.075
0.100
0.125
0.150
SNP effect on Crohn's disease of large intestine || id:finngen_R11_CHRONLARGE
SNP effect on F−fruit liking (derived food−liking factor) || id:ebi−fl187−GCST90094767

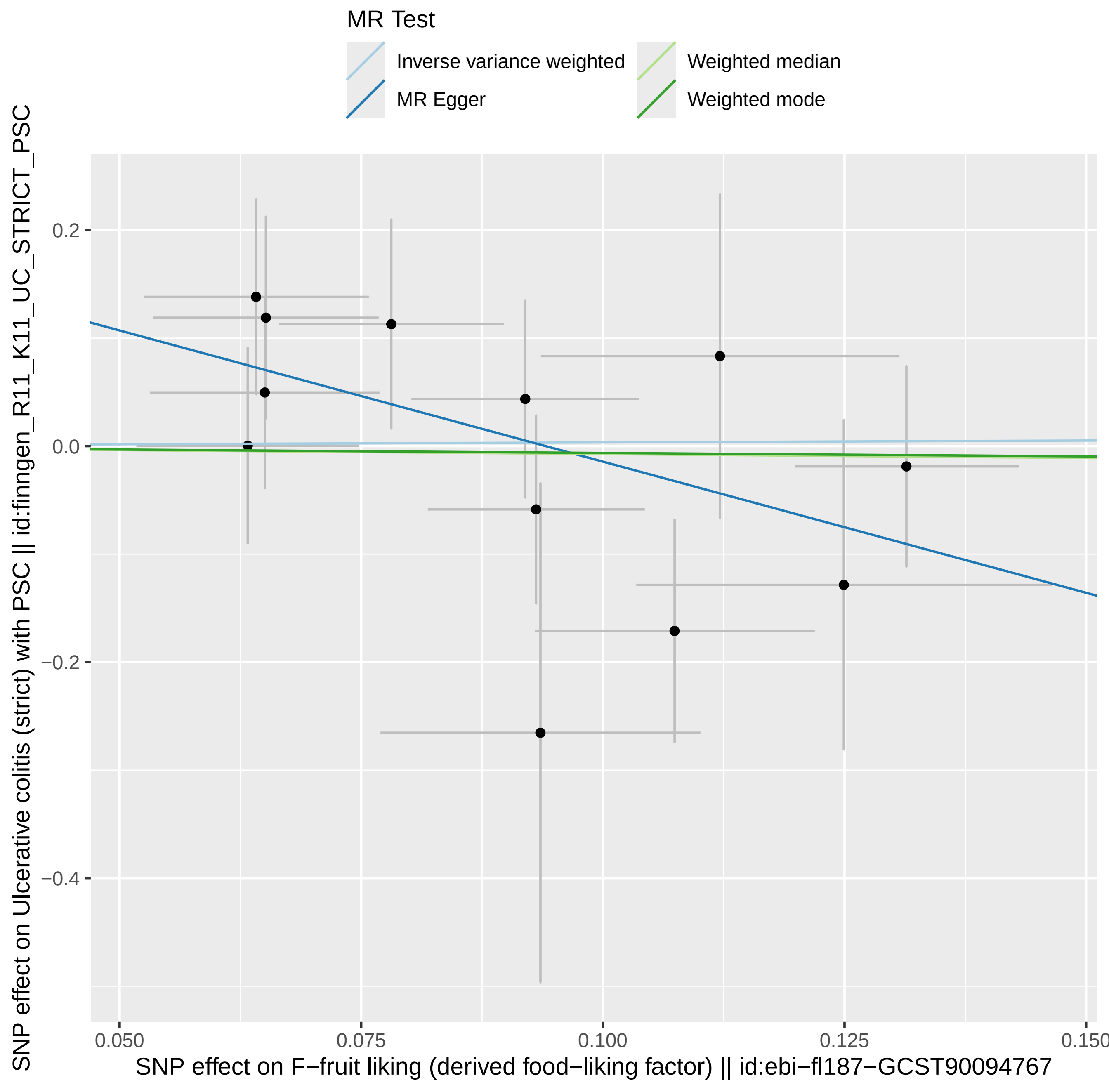

MR Test
Inverse variance weighted
MR Egger
Weighted median
Weighted mode
0.2
0.0
−0.2
−0.4
0.050
0.075
0.100
0.125
0.150
SNP effect on Ulcerative colitis (strict) with PSC || id:finngen_R11_K11_UC_STRICT_PSC
SNP effect on F−fruit liking (derived food−liking factor) || id:ebi−fl187−GCST90094767

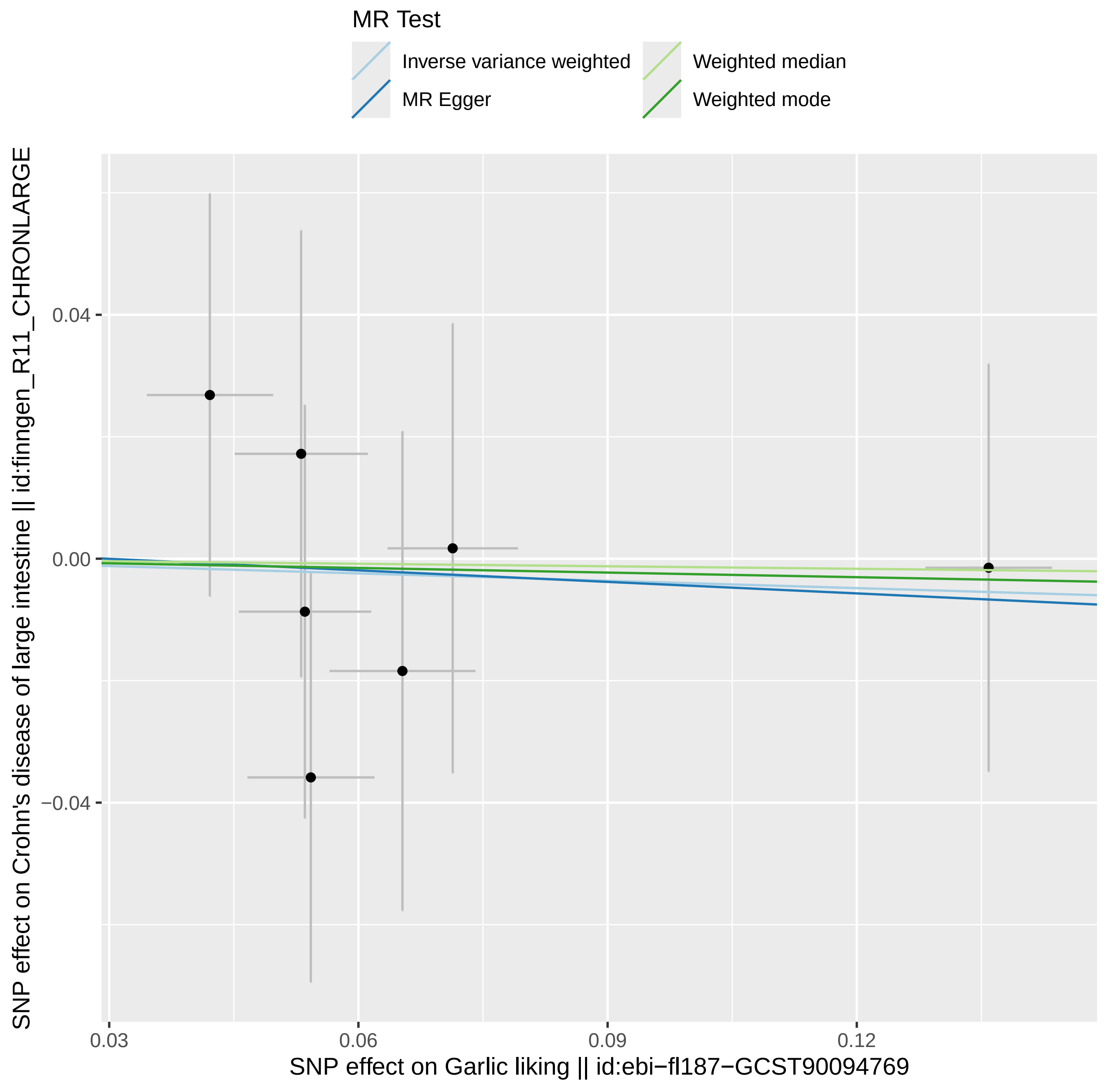
MR Test
Inverse variance weighted
MR Egger
Weighted median
Weighted mode
0.04
0.00
−0.04
0.03
0.06
0.09
0.12
SNP effect on Garlic liking || id:ebi−fl187−GCST90094769
SNP effect on Crohn's disease of large intestine || id:finngen_R11_CHRONLARGE

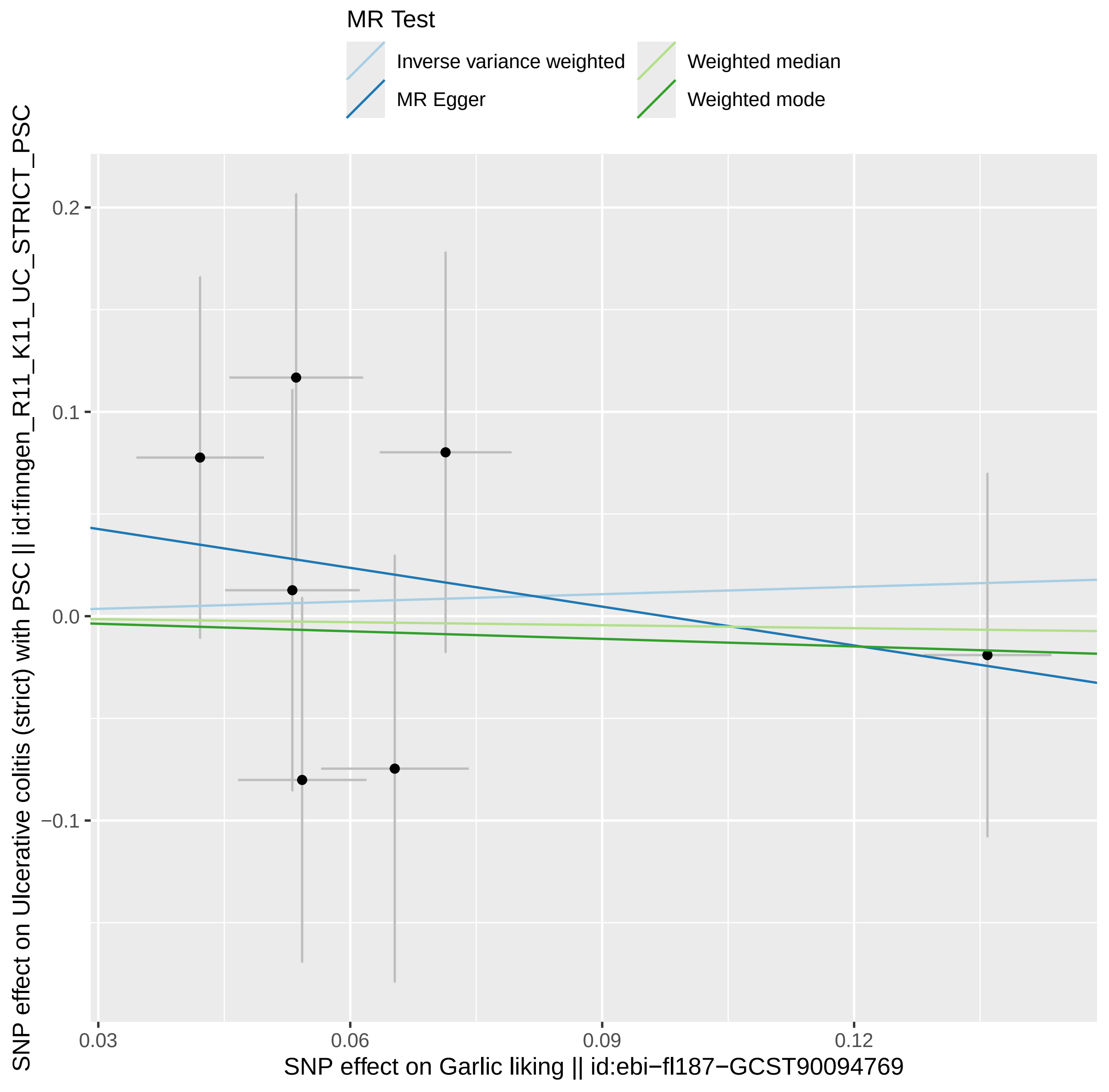

MR Test
Inverse variance weighted
MR Egger
Weighted median
Weighted mode
0.2
0.1
0.0
−0.1
0.03
0.06
0.09
0.12
SNP effect on Ulcerative colitis (strict) with PSC || id:finngen_R11_K11_UC_STRICT_PSC
SNP effect on Garlic liking || id:ebi−fl187−GCST90094769

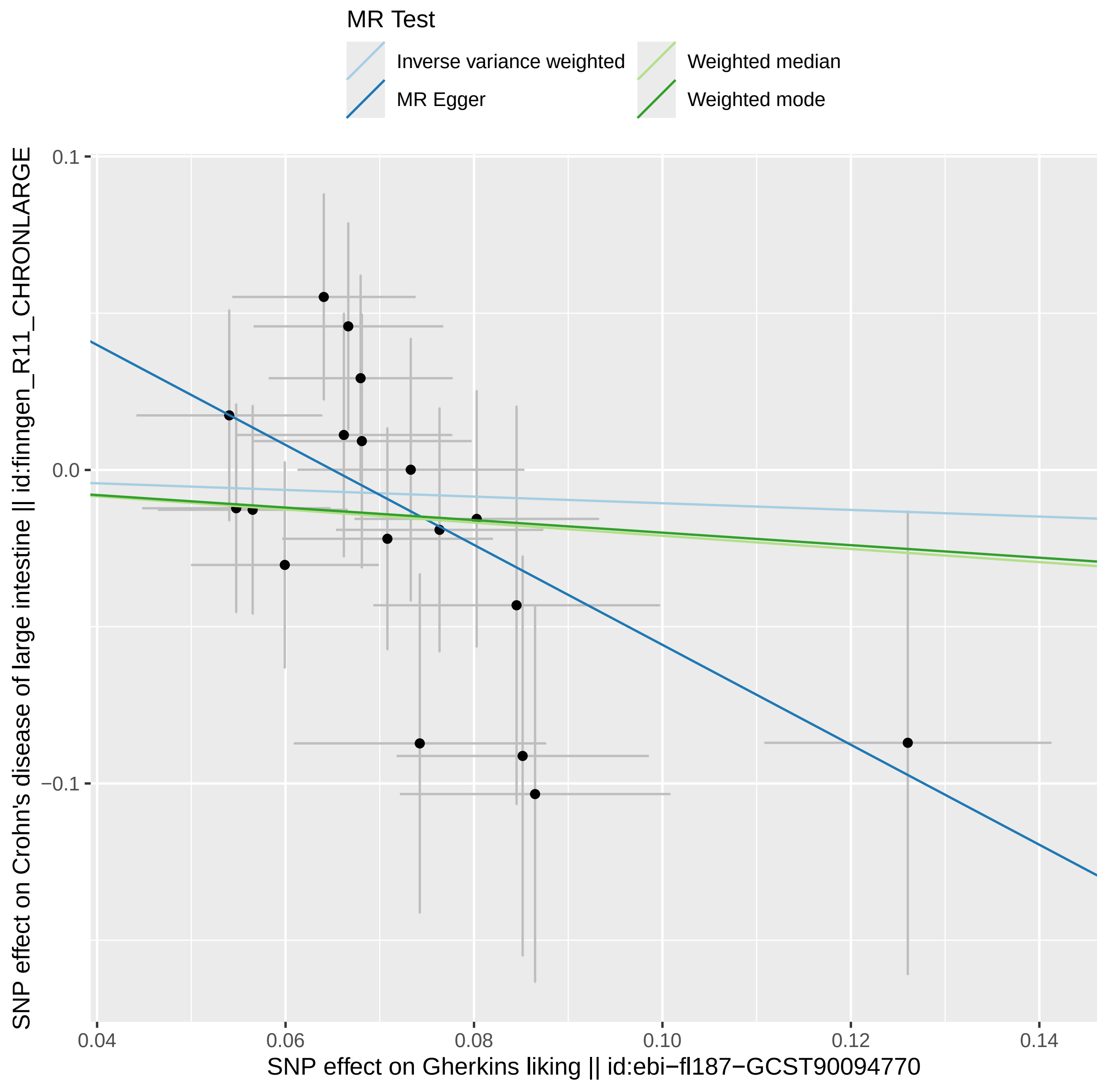
MR Test
Inverse variance weighted
MR Egger
Weighted median
Weighted mode
SNP effect on Crohn's disease of large intestine || id:finngen_R11_CHRONLARGE
SNP effect on Gherkins liking || id:ebi−fl187−GCST90094770
0.1
0.0
−0.1
0.04
0.06
0.08
0.10
0.12
0.14

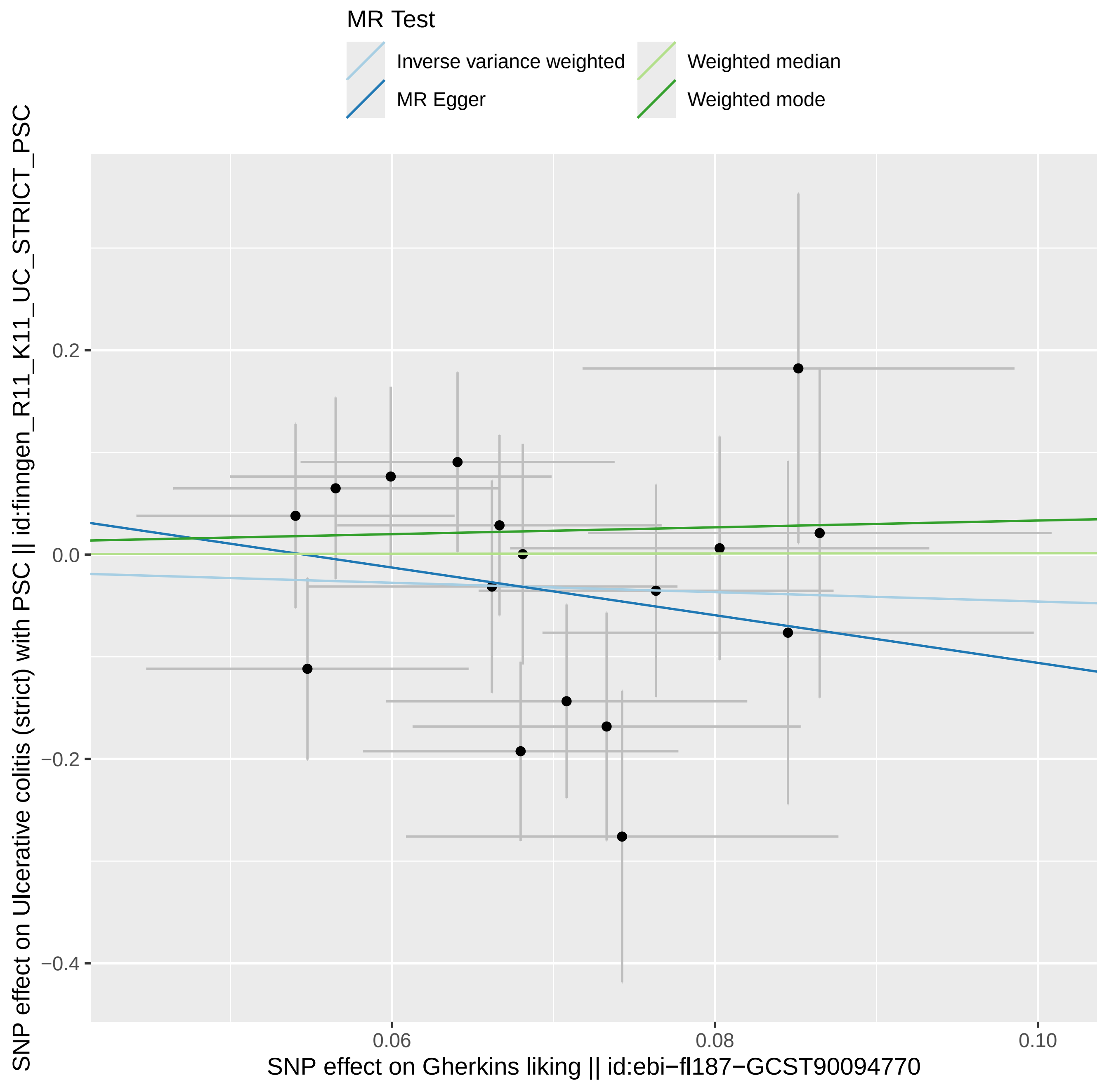

MR Test
Inverse variance weighted
MR Egger
Weighted median
Weighted mode
0.2
0.0
−0.2
−0.4
0.06
0.08
0.10
SNP effect on Ulcerative colitis (strict) with PSC || id:finngen_R11_K11_UC_STRICT_PSC
SNP effect on Gherkins liking || id:ebi−fl187−GCST90094770

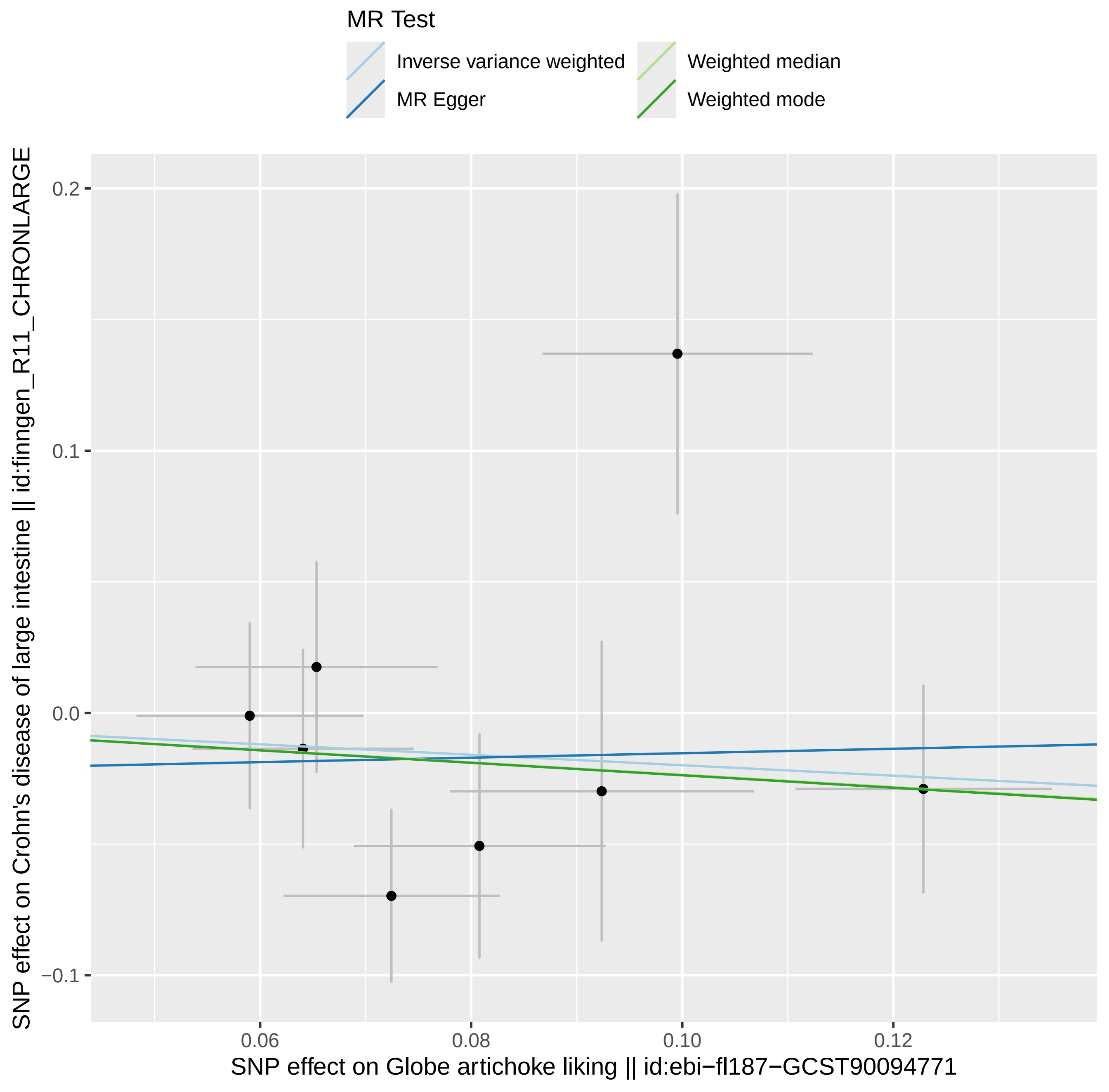

MR Test
Inverse variance weighted
MR Egger
Weighted median
Weighted mode
SNP effect on Crohn's disease of large intestine || id:finngen_R11_CHRONLARGE
0.2
0.1
0.0
−0.1
0.06
0.08
0.10
0.12
SNP effect on Globe artichoke liking || id:ebi−fI187−GCST90094771

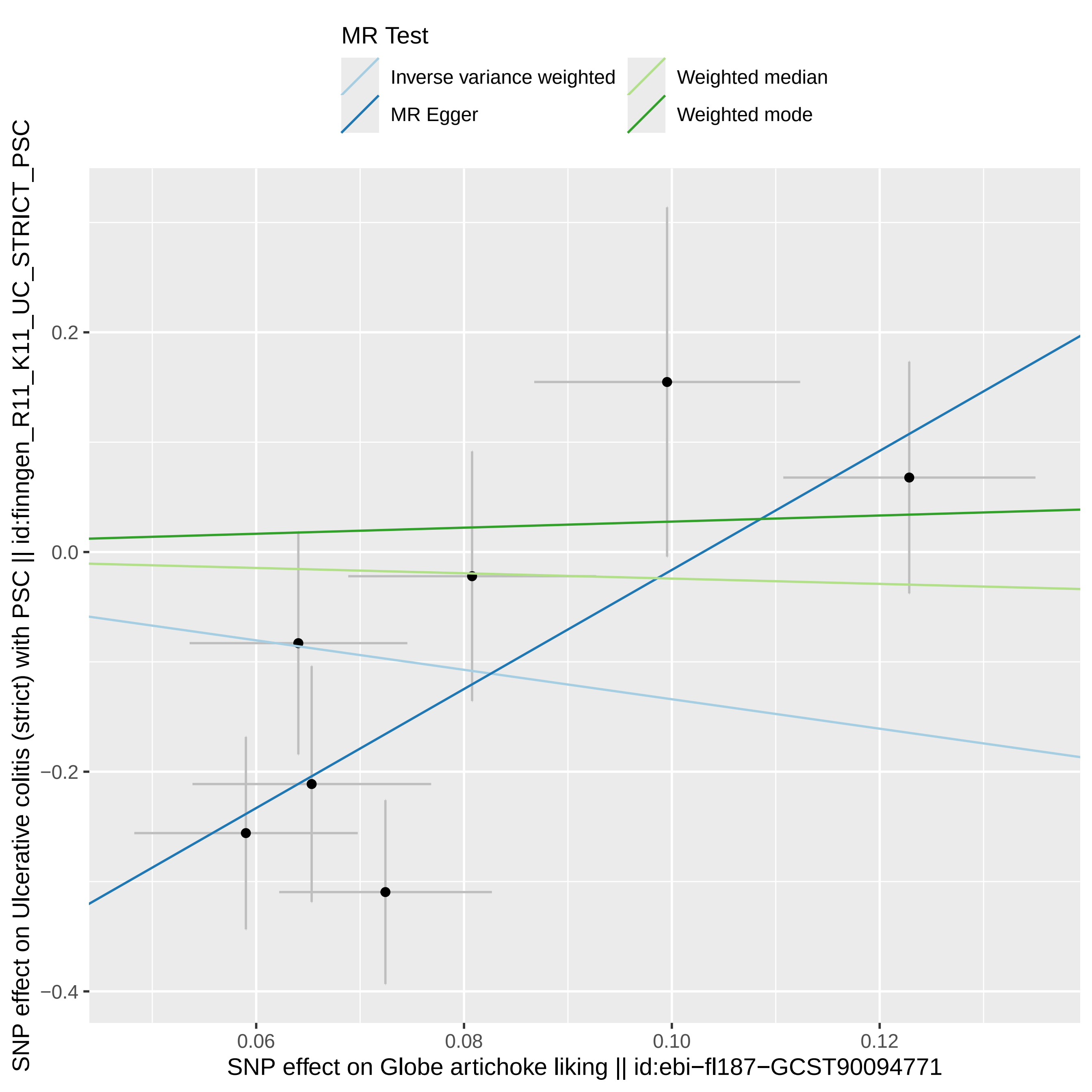
MR Test
Inverse variance weighted
MR Egger
Weighted median
Weighted mode
0.2
0.0
−0.2
−0.4
0.06
0.08
0.10
0.12
SNP effect on Ulcerative colitis (strict) with PSC || id:finngen_R11_K11_UC_STRICT_PSC
SNP effect on Globe artichoke liking || id:ebi−fl187−GCST90094771

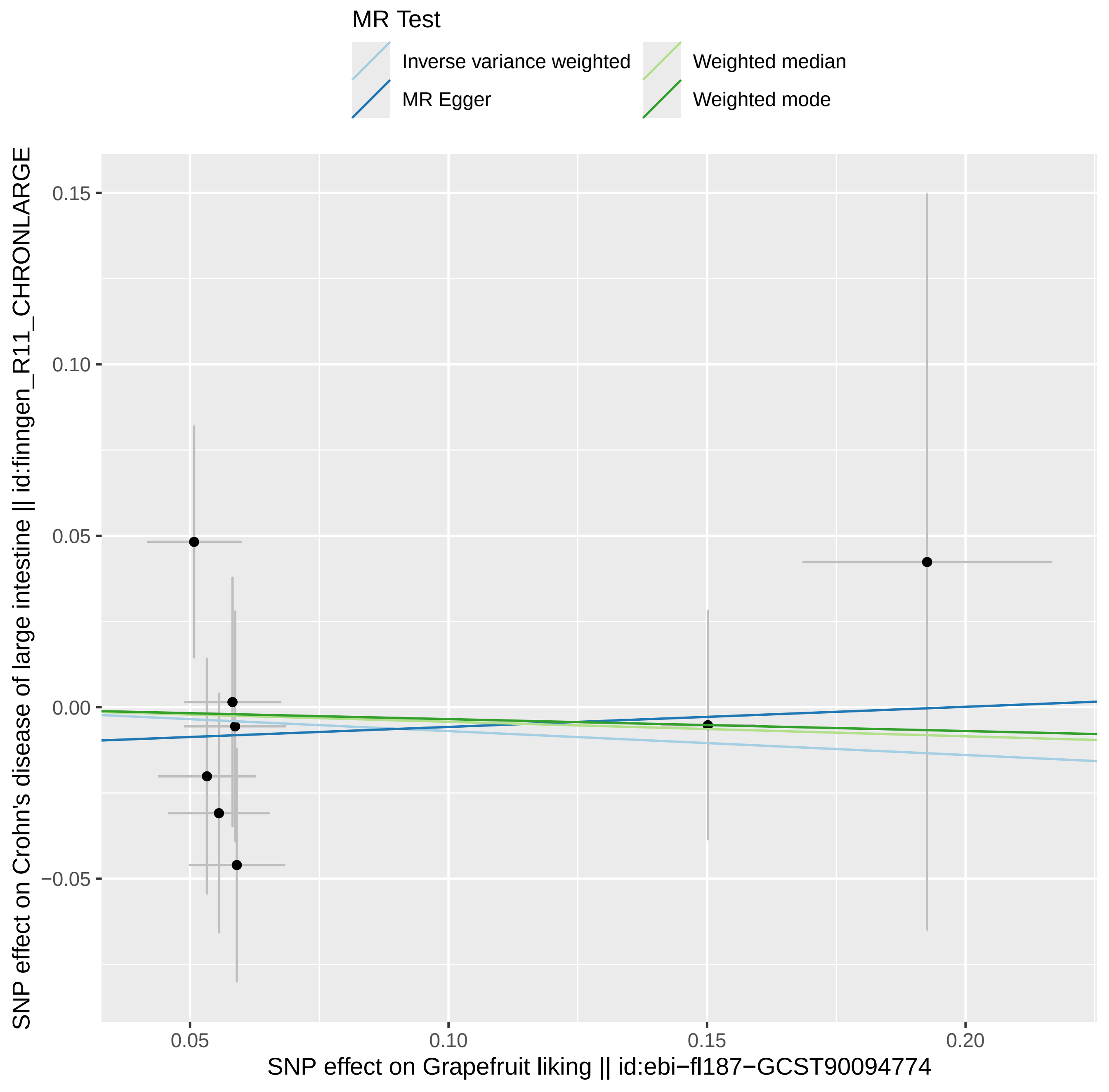
MR Test
Inverse variance weighted
MR Egger
Weighted median
Weighted mode
0.15
0.10
0.05
0.00
−0.05
0.05
0.10
0.15
0.20
SNP effect on Crohn's disease of large intestine || id:finngen_R11_CHRONLARGE
SNP effect on Grapefruit liking || id:ebi−fl187−GCST90094774

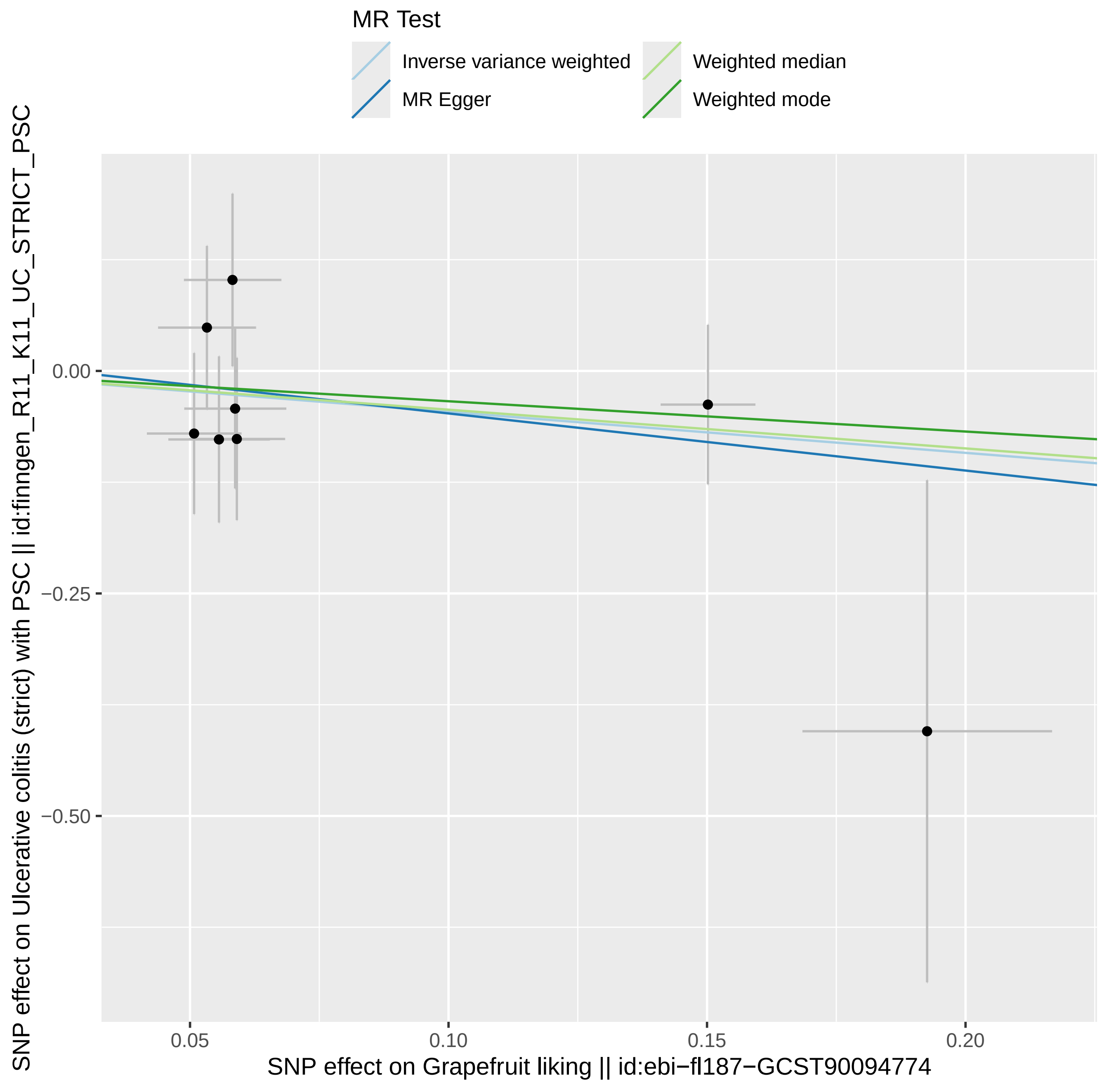
MR Test
Inverse variance weighted
MR Egger
Weighted median
Weighted mode
0.00
−0.25
−0.50
0.05
0.10
0.15
0.20
SNP effect on Ulcerative colitis (strict) with PSC || id:finngen_R11_K11_UC_STRICT_PSC
SNP effect on Grapefruit liking || id:ebi−fl187−GCST90094774

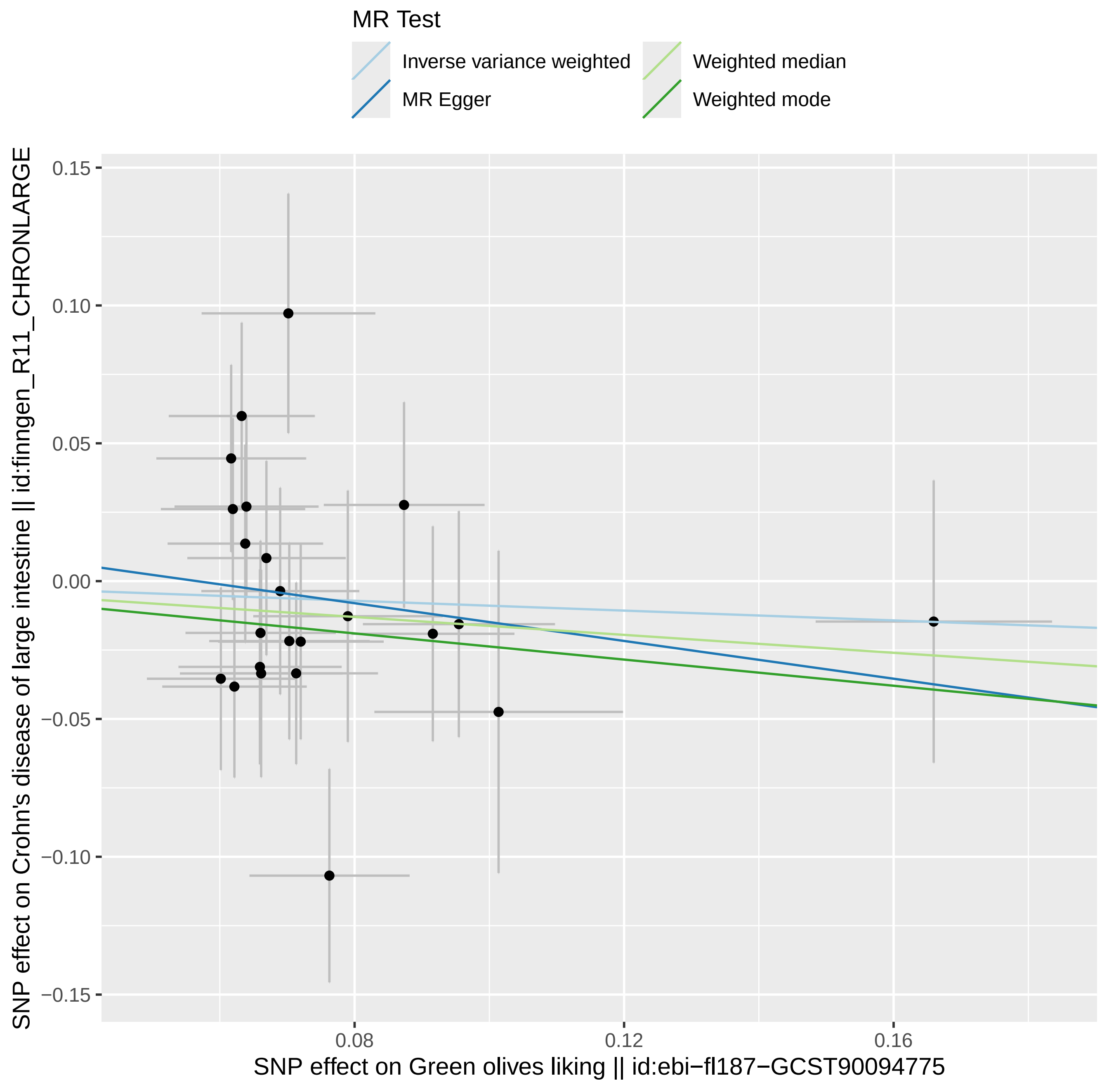
MR Test
Inverse variance weighted
MR Egger
Weighted median
Weighted mode
SNP effect on Crohn's disease of large intestine || id:finngen_R11_CHRONLARGE
0.15
0.10
0.05
0.00
−0.05
−0.10
−0.15
0.08
0.12
0.16
SNP effect on Green olives liking || id:ebi−fl187−GCST90094775

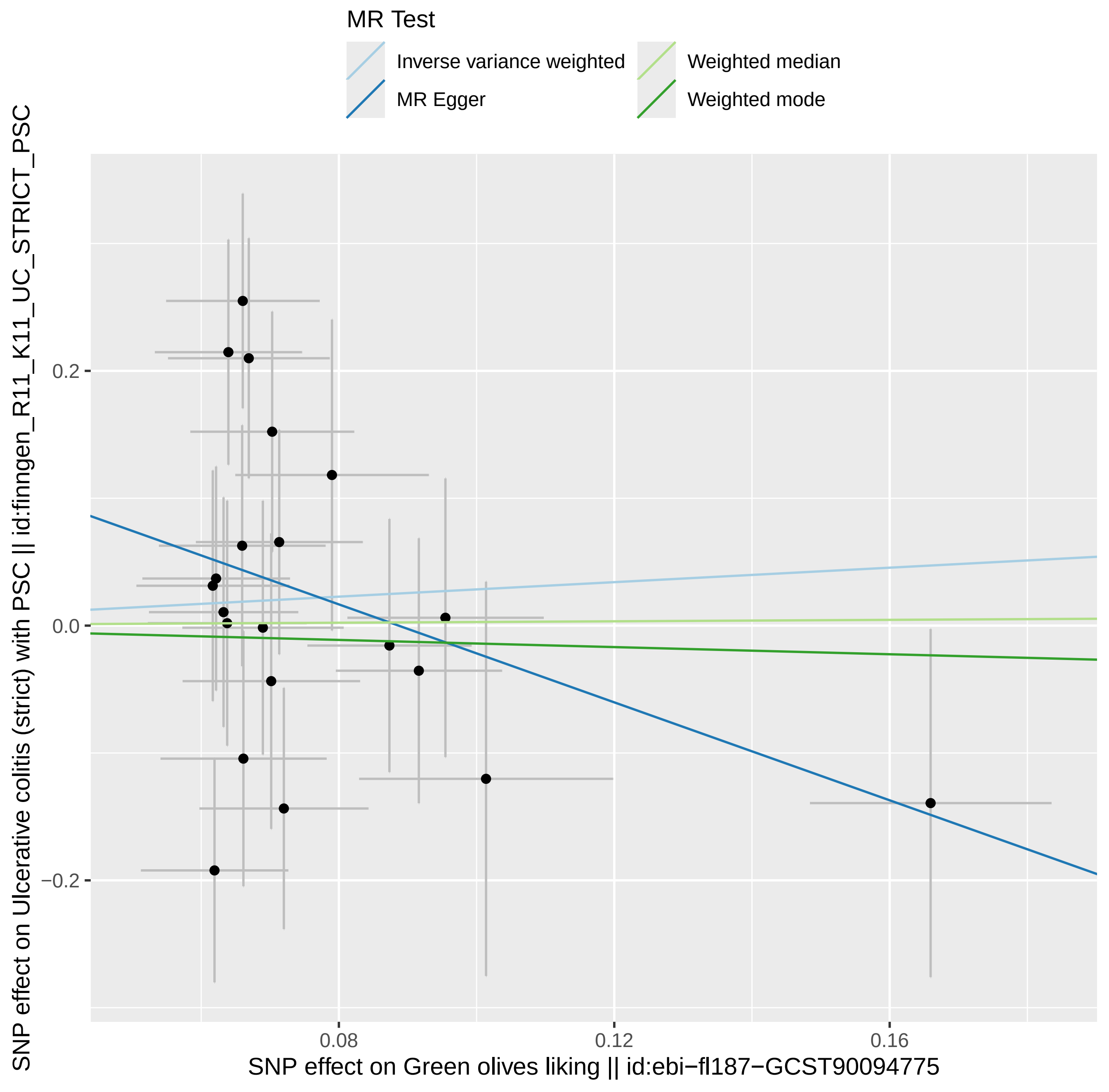

MR Test
Inverse variance weighted
MR Egger
Weighted median
Weighted mode
0.2
0.0
−0.2
0.08
0.12
0.16
SNP effect on Ulcerative colitis (strict) with PSC || id:finngen_R11_K11_UC_STRICT_PSC
SNP effect on Green olives liking || id:ebi−fl187−GCST90094775

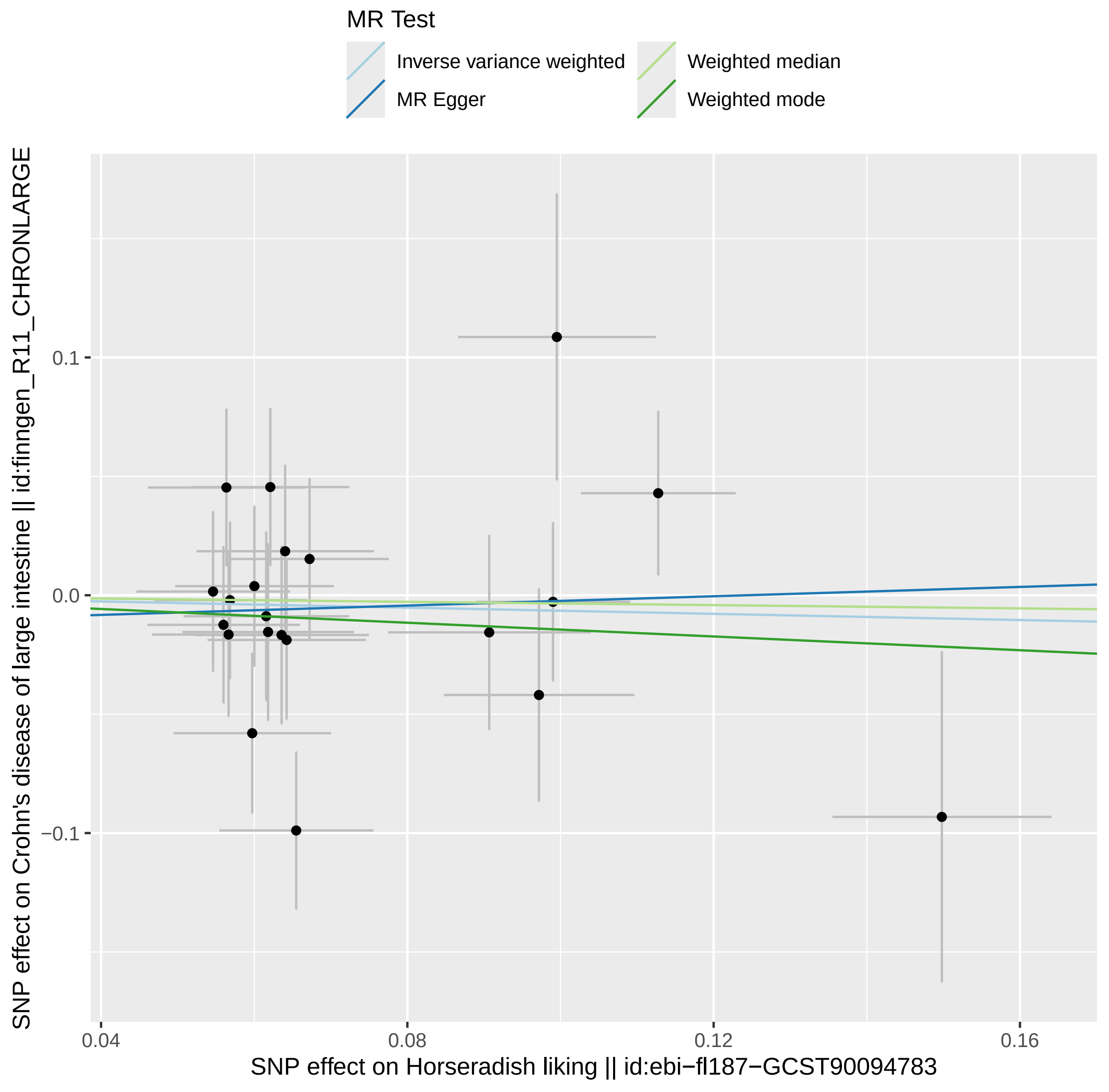

MR Test
Inverse variance weighted
MR Egger
Weighted median
Weighted mode
0.1
0.0
−0.1
0.04
0.08
0.12
0.16
SNP effect on Crohn's disease of large intestine || id:finngen_R11_CHRONLARGE
SNP effect on Horseradish liking || id:ebi−fl187−GCST90094783

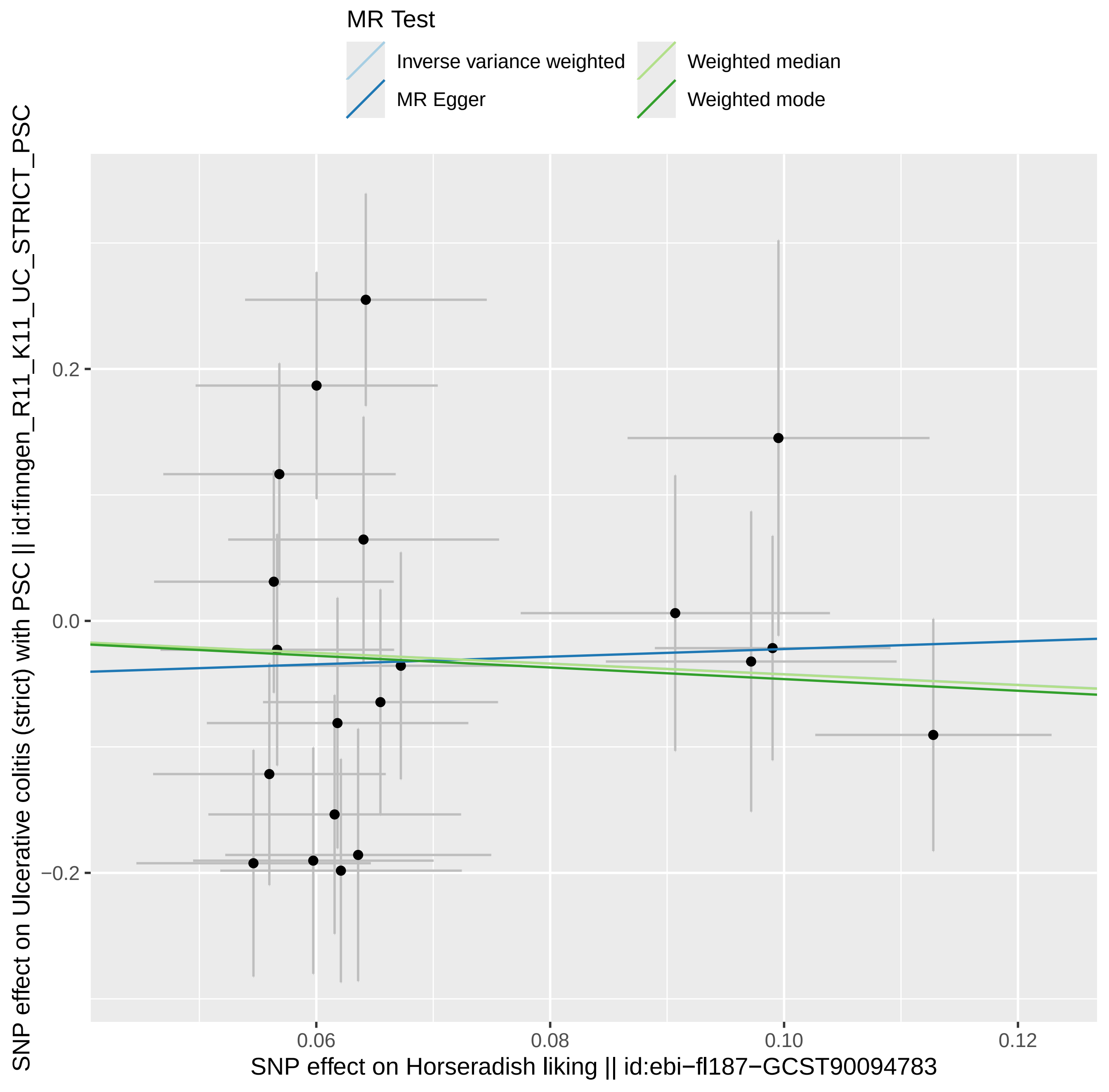

MR Test
Inverse variance weighted
MR Egger
Weighted median
Weighted mode
0.2
0.0
−0.2
0.06
0.08
0.10
0.12
SNP effect on Ulcerative colitis (strict) with PSC || id:finngen_R11_K11_UC_STRICT_PSC
SNP effect on Horseradish liking || id:ebi−fl187−GCST90094783

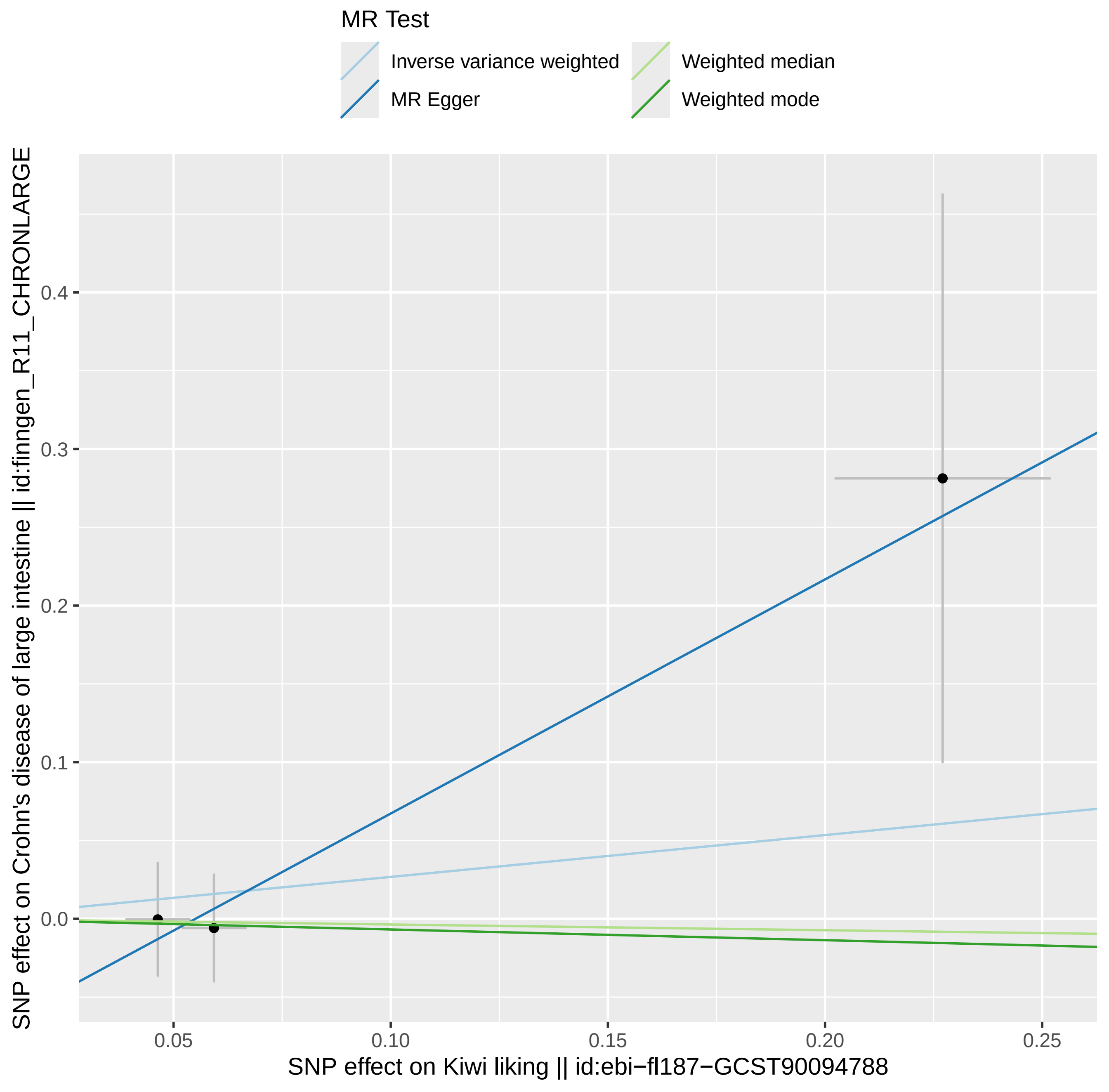
MR Test
Inverse variance weighted
MR Egger
Weighted median
Weighted mode
SNP effect on Crohn's disease of large intestine || id:finngen_R11_CHRONLARGE
0.4
0.3
0.2
0.1
0.0
0.05
0.10
0.15
0.20
0.25
SNP effect on Kiwi liking || id:ebi−fl187−GCST90094788

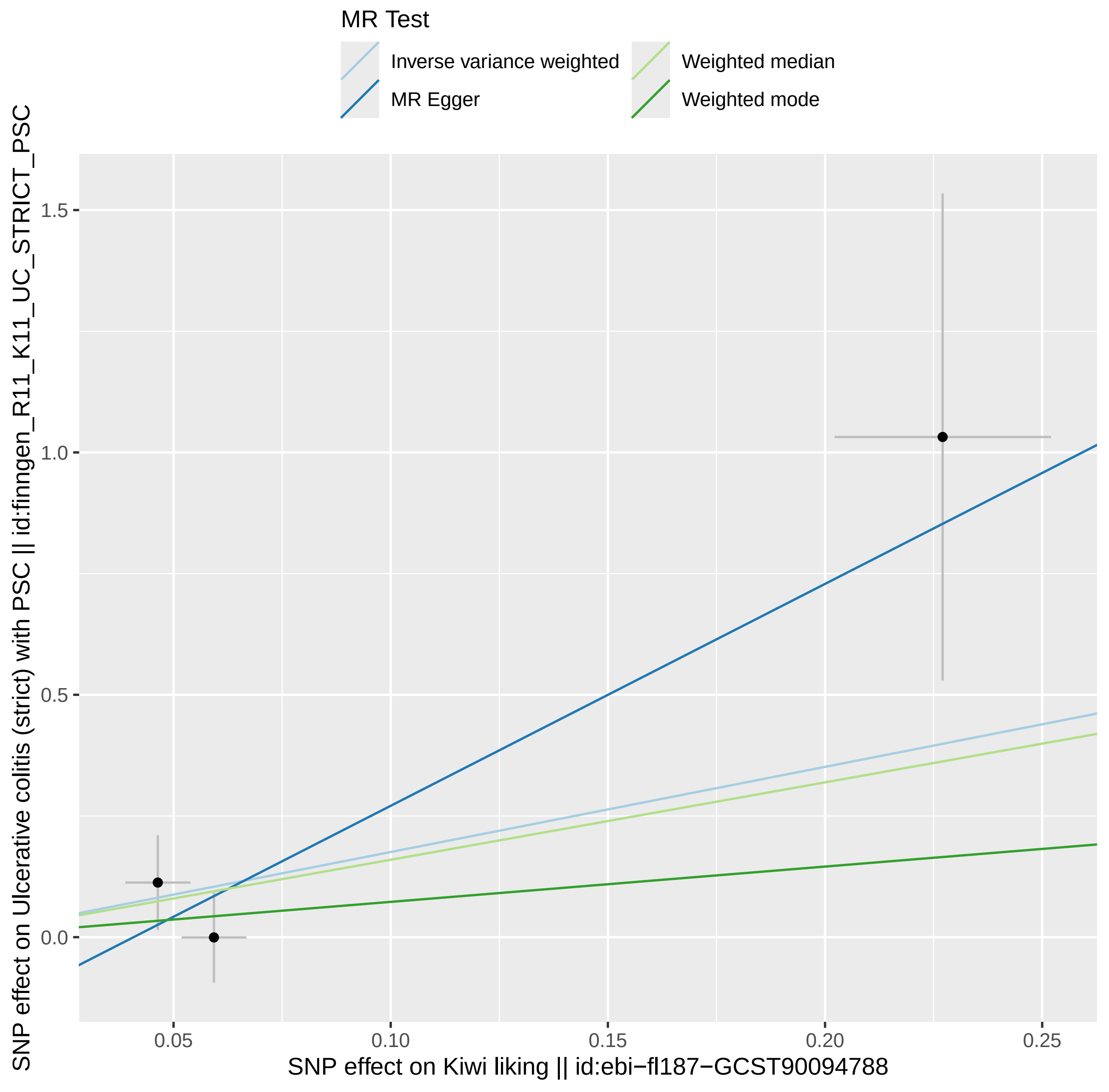
MR Test
Inverse variance weighted
MR Egger
Weighted median
Weighted mode
1.5
1.0
0.5
0.0
0.05
0.10
0.15
0.20
0.25
SNP effect on Ulcerative colitis (strict) with PSC || id:finngen_R11_K11_UC_STRICT_PSC
SNP effect on Kiwi liking || id:ebi−fl187−GCST90094788

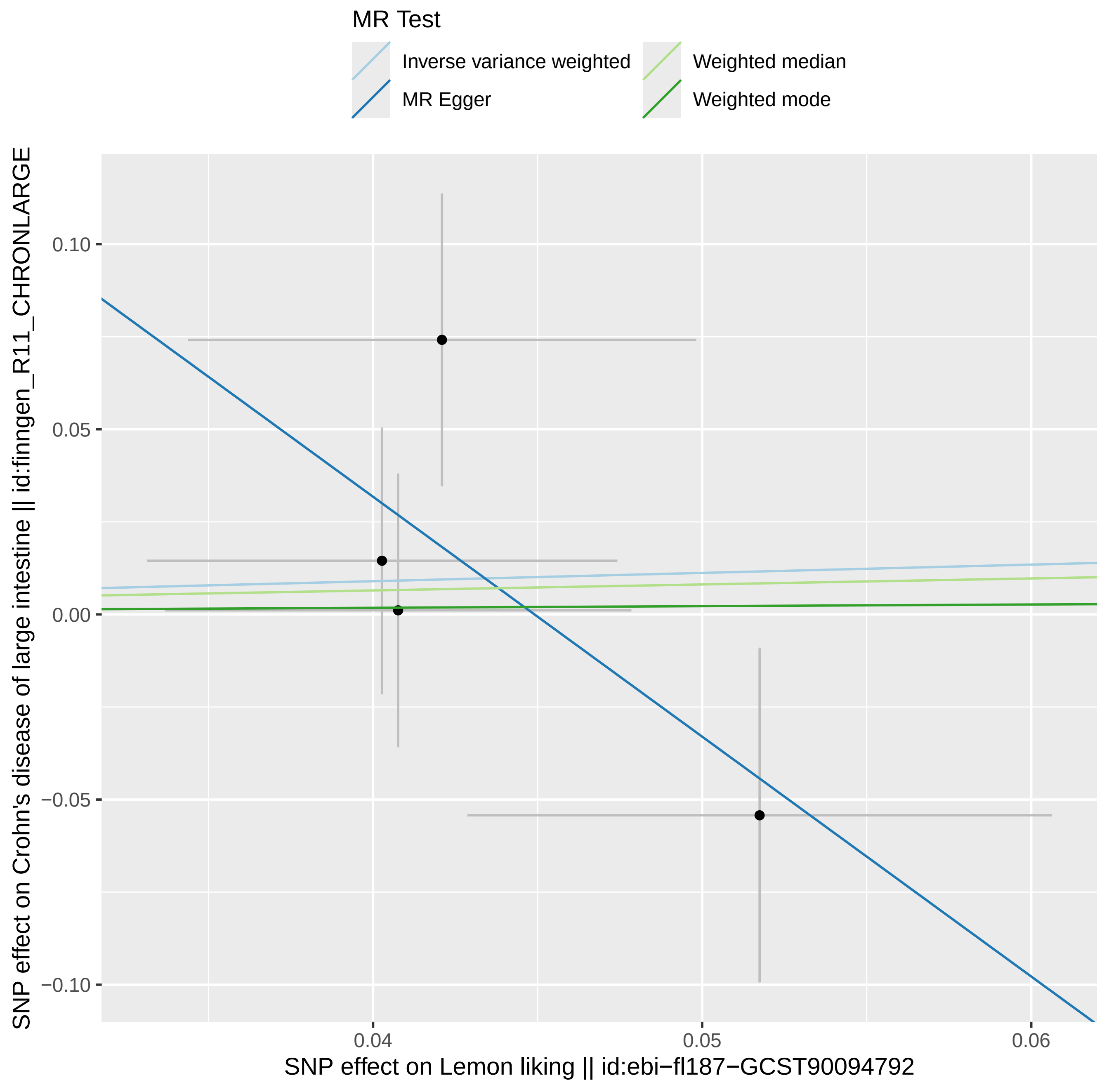
MR Test
Inverse variance weighted
MR Egger
Weighted median
Weighted mode
0.10
0.05
0.00
−0.05
−0.10
0.04
0.05
0.06
SNP effect on Lemon liking || id:ebi−fl187−GCST90094792
SNP effect on Crohn's disease of large intestine || id:finngen_R11_CHRONLARGE

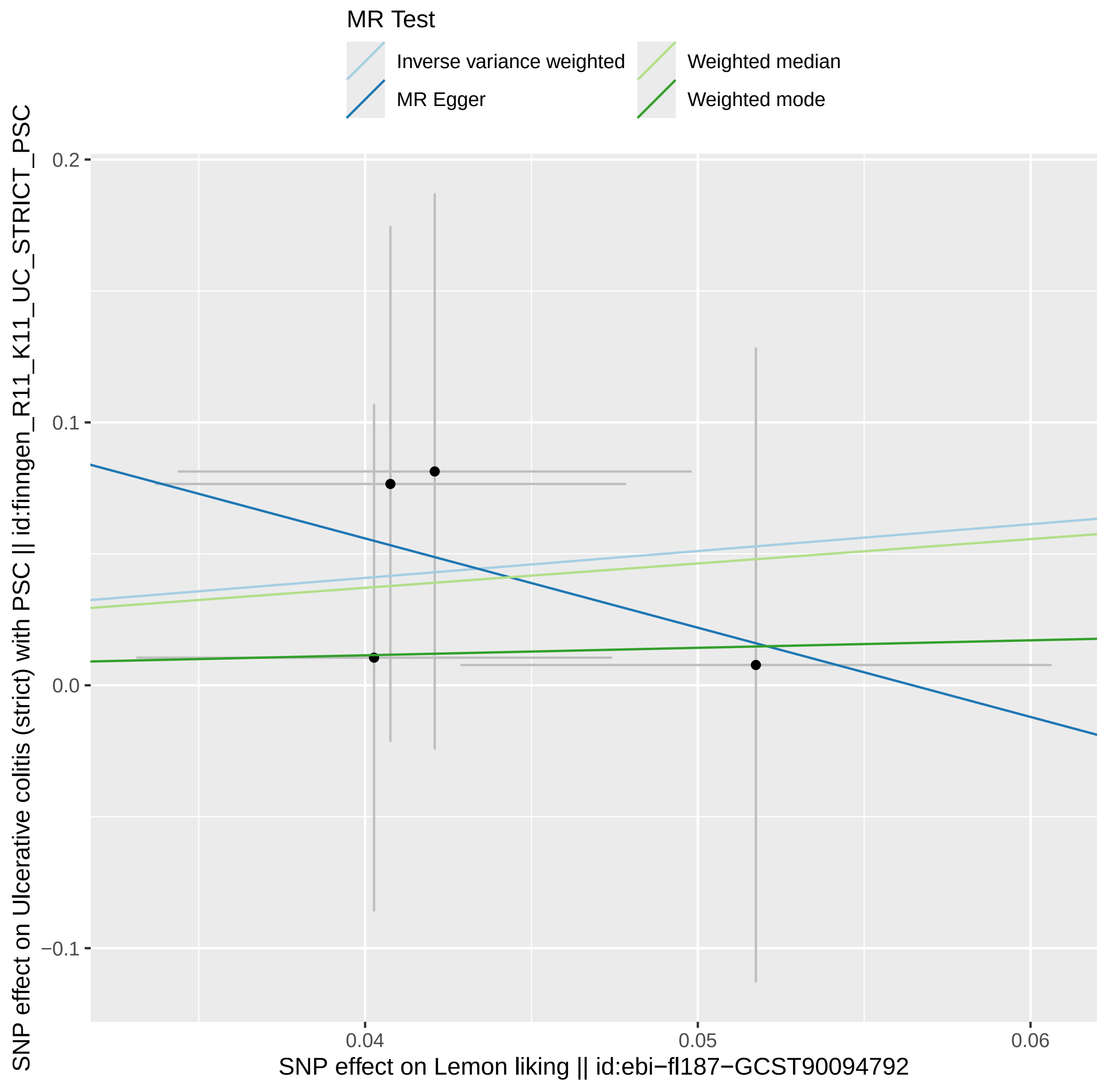

MR Test
Inverse variance weighted
Weighted median
MR Egger
Weighted mode
0.2
0.1
0.0
−0.1
0.04
0.05
0.06
SNP effect on Ulcerative colitis (strict) with PSC || id:finngen_R11_K11_UC_STRICT_PSC
SNP effect on Lemon liking || id:ebi−fl187−GCST90094792

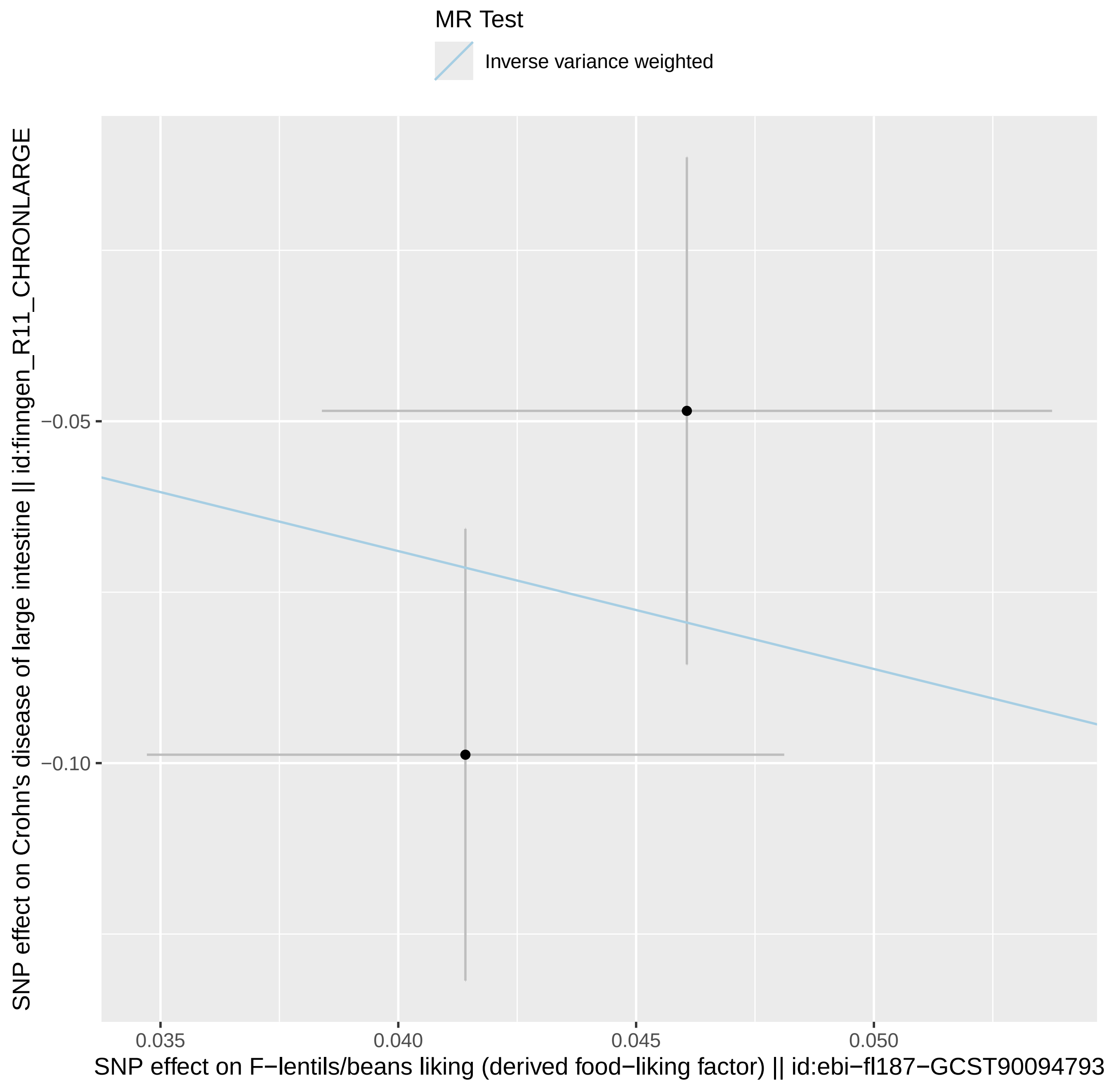
MR Test
Inverse variance weighted
SNP effect on Crohn's disease of large intestine || id:finngen_R11_CHRONLARGE
−0.05
−0.10
0.035
0.040
0.045
0.050
SNP effect on F−lentils/beans liking (derived food−liking factor) || id:ebi−fl187−GCST90094793

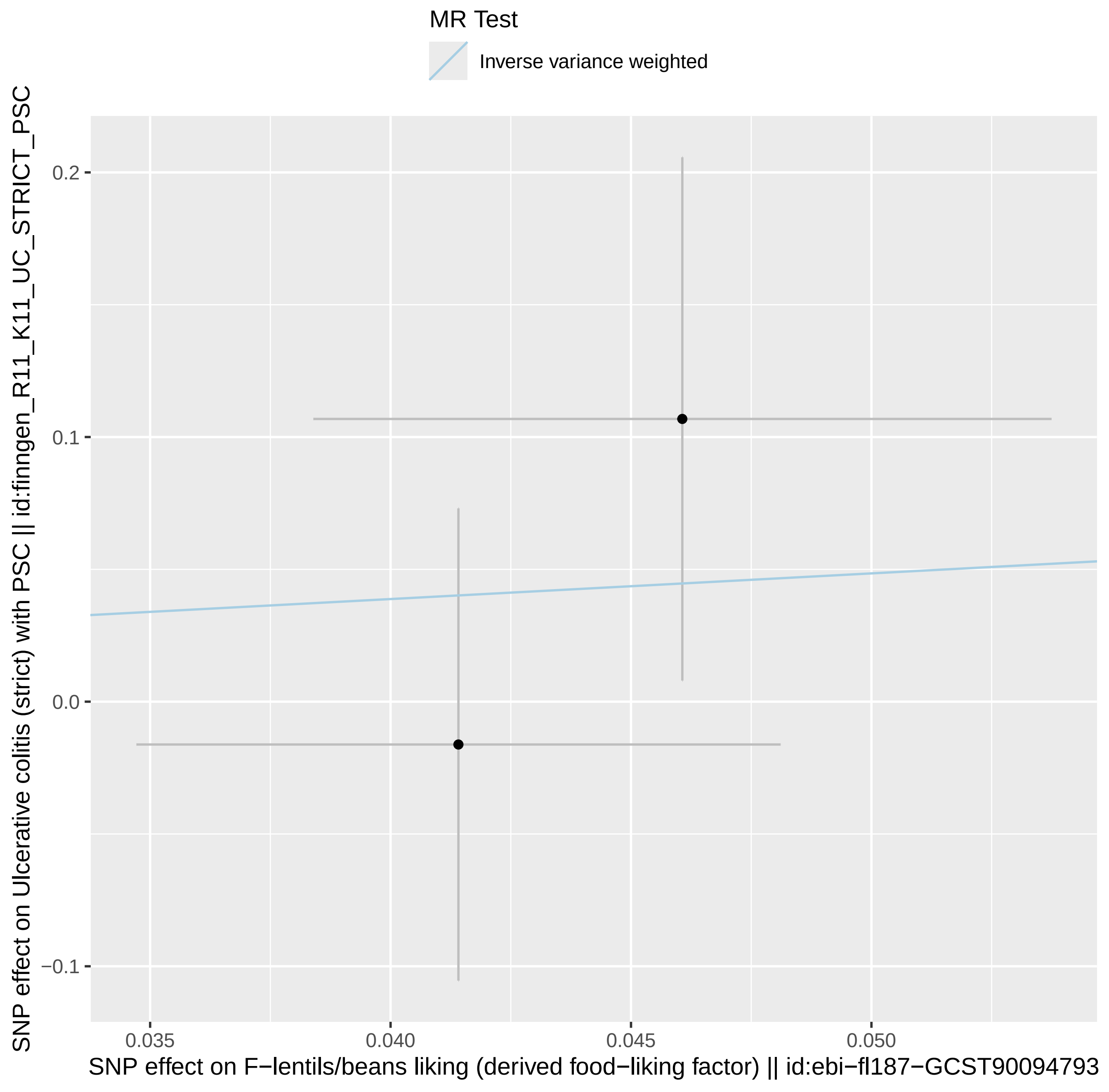
MR Test
Inverse variance weighted
0.2
0.1
0.0
−0.1
0.035
0.040
0.045
0.050
SNP effect on Ulcerative colitis (strict) with PSC || id:finngen_R11_K11_UC_STRICT_PSC
SNP effect on F−lentils/beans liking (derived food−liking factor) || id:ebi−fl187−GCST90094793

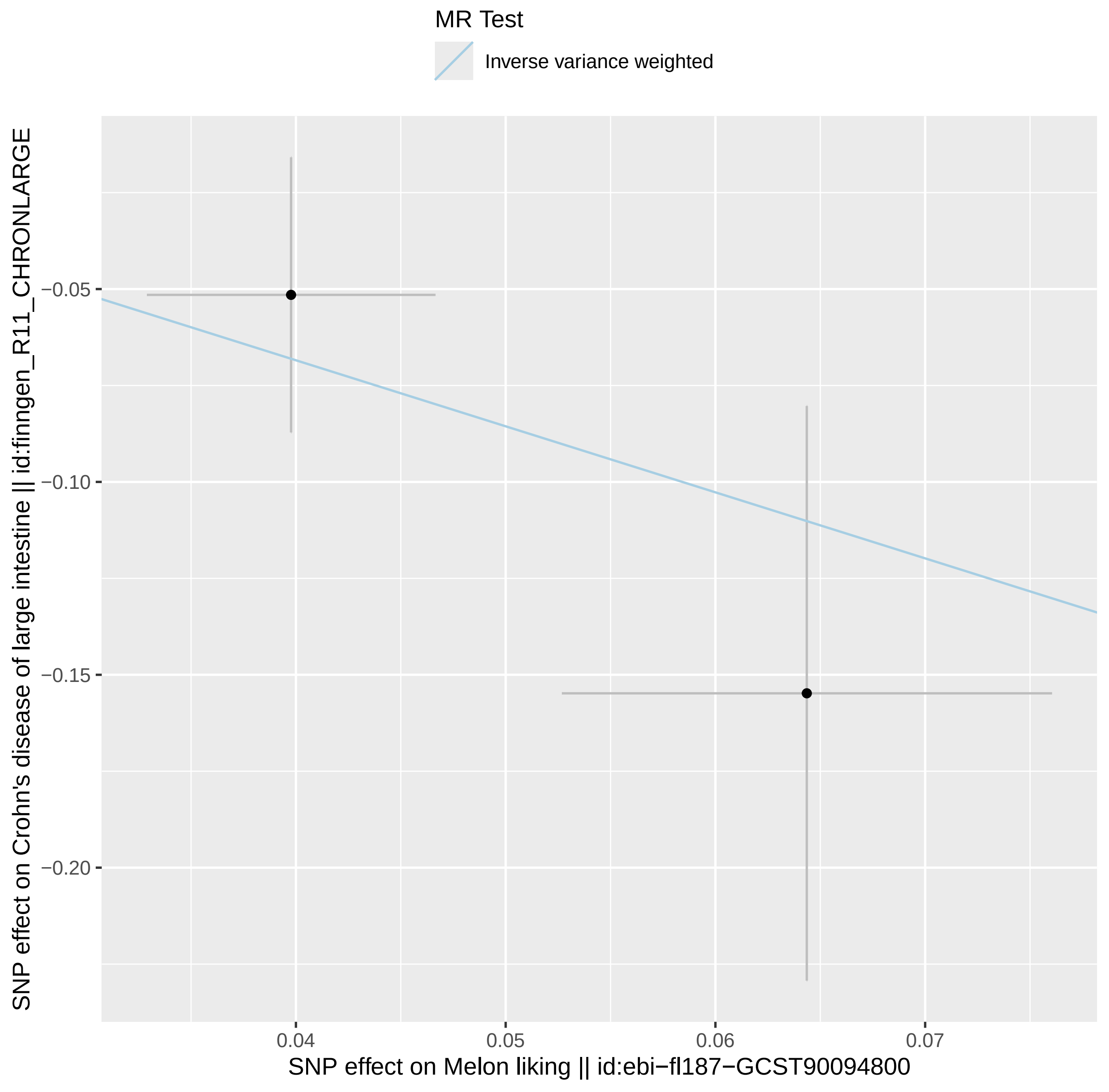
MR Test
Inverse variance weighted
SNP effect on Crohn's disease of large intestine || id:finngen_R11_CHRONLARGE
−0.05
−0.10
−0.15
−0.20
0.04
0.05
0.06
0.07
SNP effect on Melon liking || id:ebi−fl187−GCST90094800

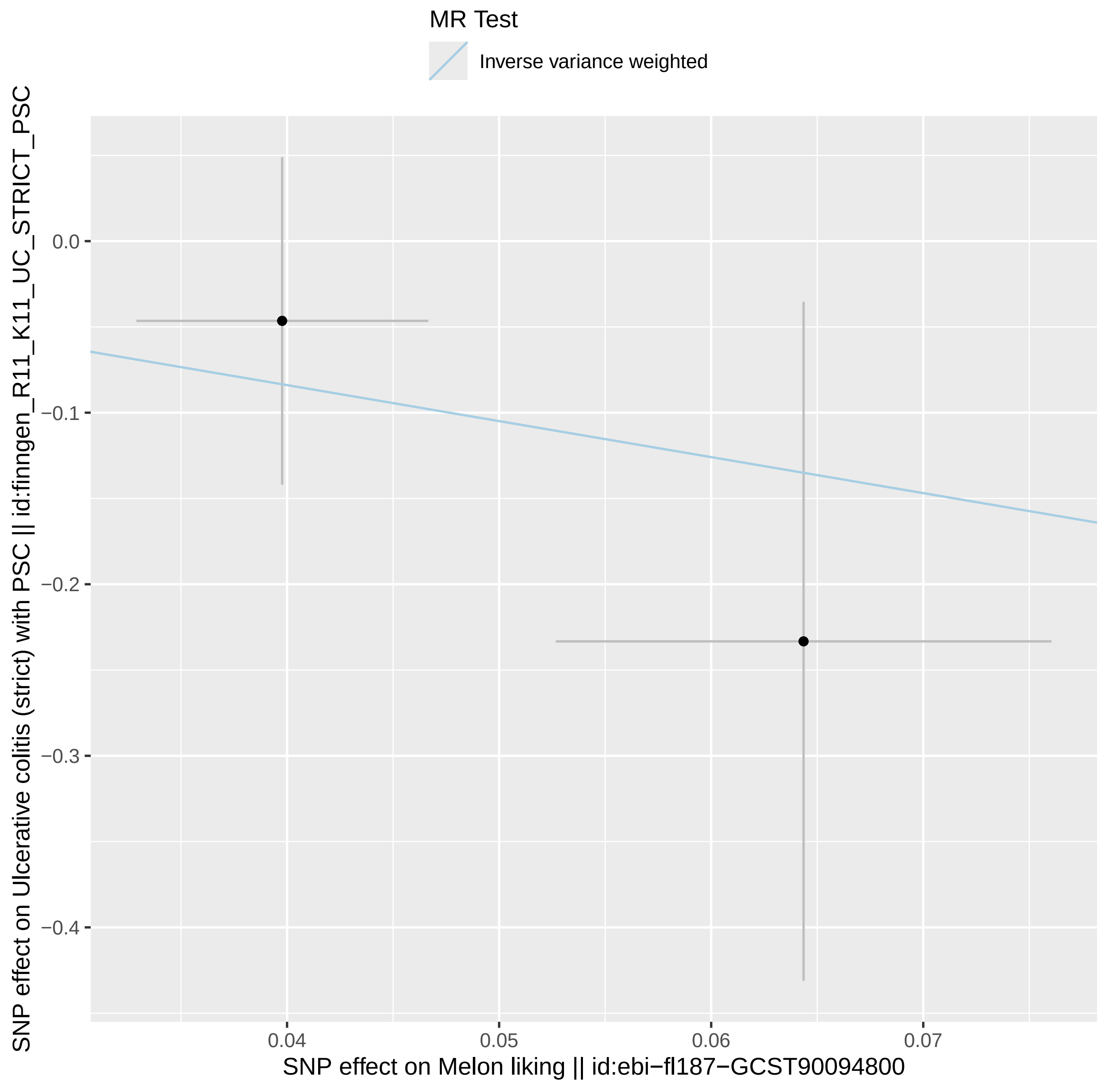

MR Test
Inverse variance weighted
0.0
−0.1
−0.2
−0.3
−0.4
0.04
0.05
0.06
0.07
SNP effect on Ulcerative colitis (strict) with PSC || id:finngen_R11_K11_UC_STRICT_PSC
SNP effect on Melon liking || id:ebi−fl187−GCST90094800

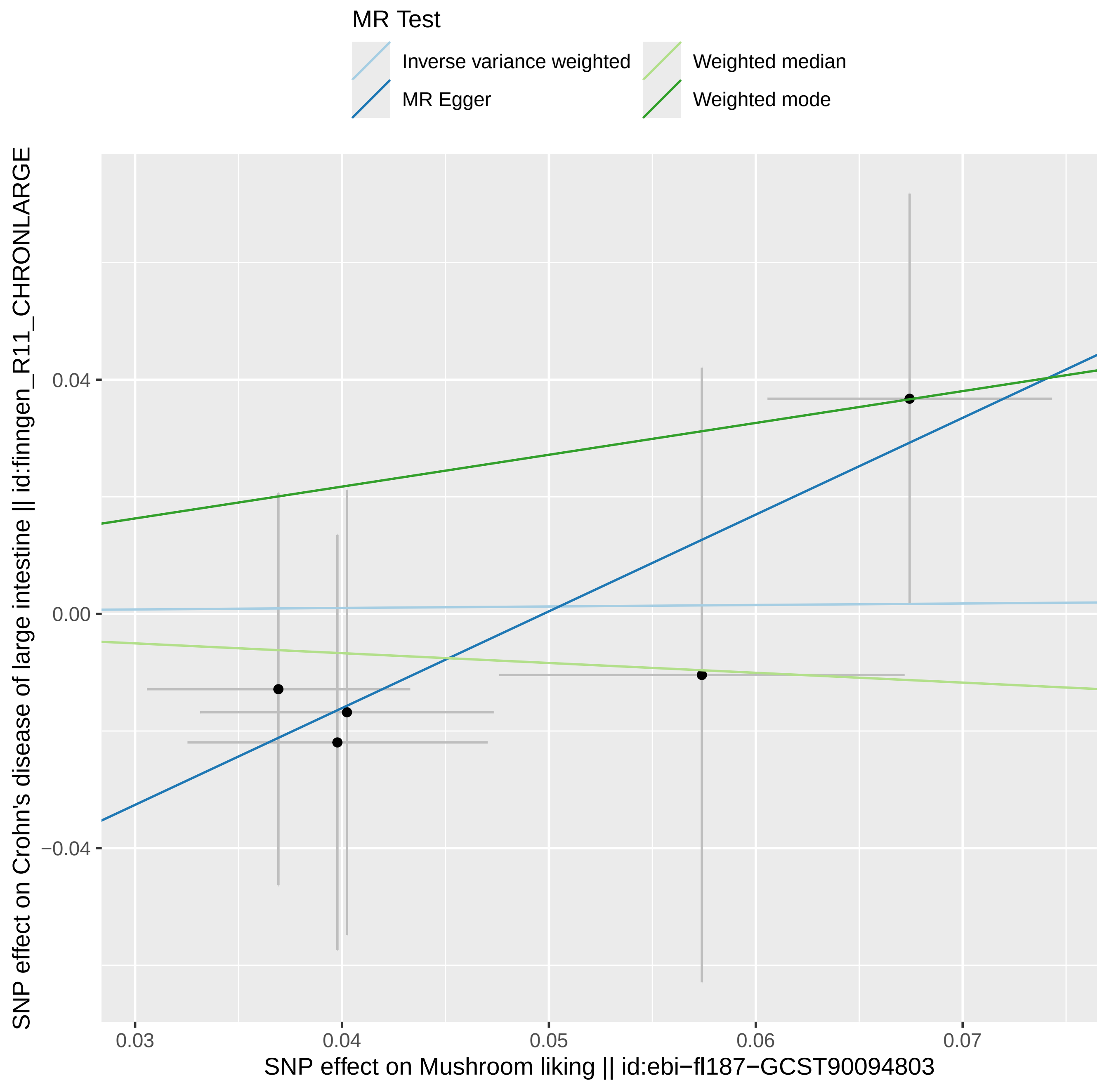
MR Test
Inverse variance weighted
MR Egger
Weighted median
Weighted mode
SNP effect on Crohn's disease of large intestine || id:finngen_R11_CHRONLARGE
0.04
0.00
−0.04
0.03
0.04
0.05
0.06
0.07
SNP effect on Mushroom liking || id:ebi−fl187−GCST90094803

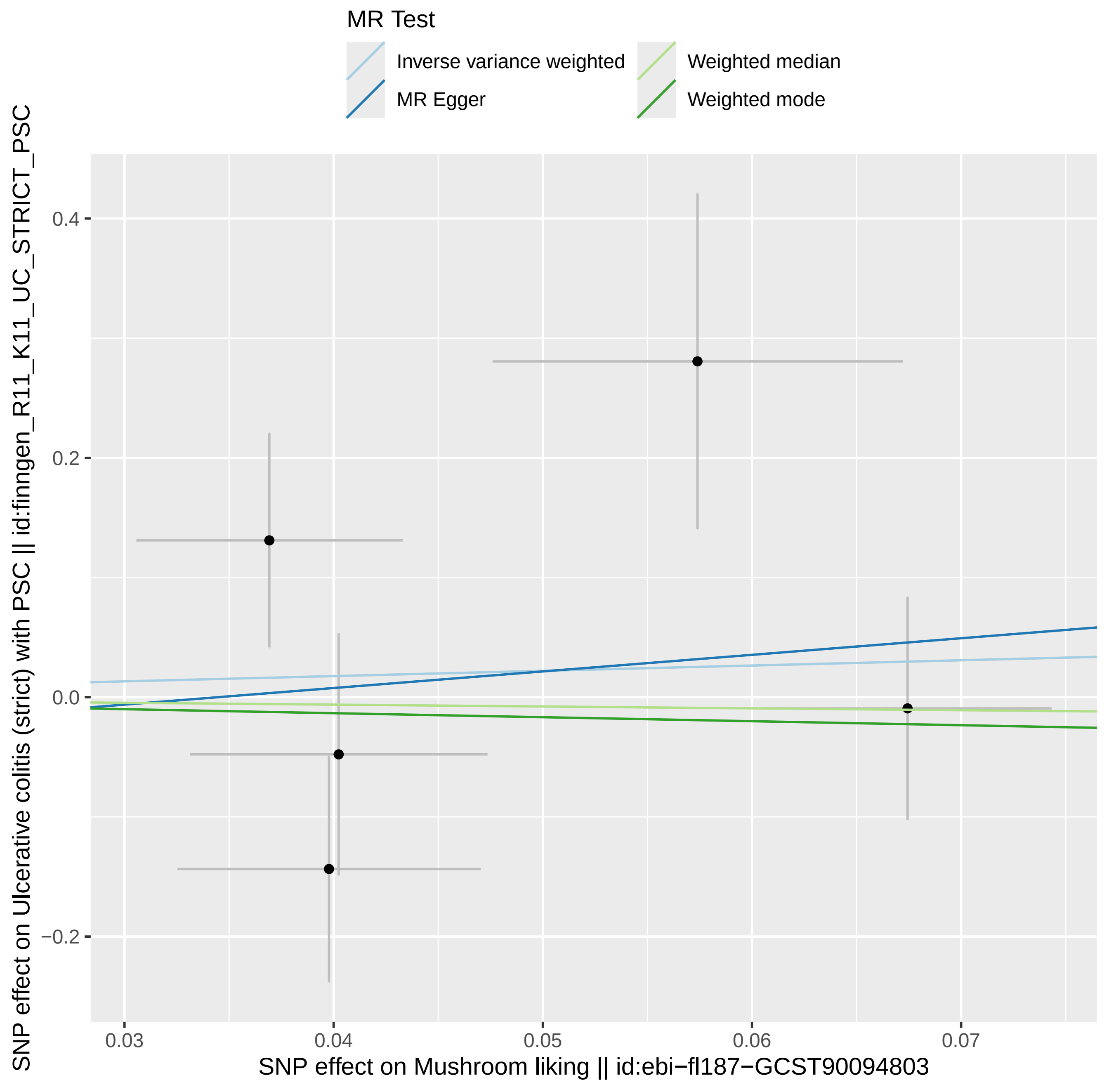
MR Test
Inverse variance weighted
MR Egger
Weighted median
Weighted mode
SNP effect on Ulcerative colitis (strict) with PSC || id:finngen_R11_K11_UC_STRICT_PSC
0.4
0.2
0.0
−0.2
0.03
0.04
0.05
0.06
0.07
SNP effect on Mushroom liking || id:ebi−fl187−GCST90094803

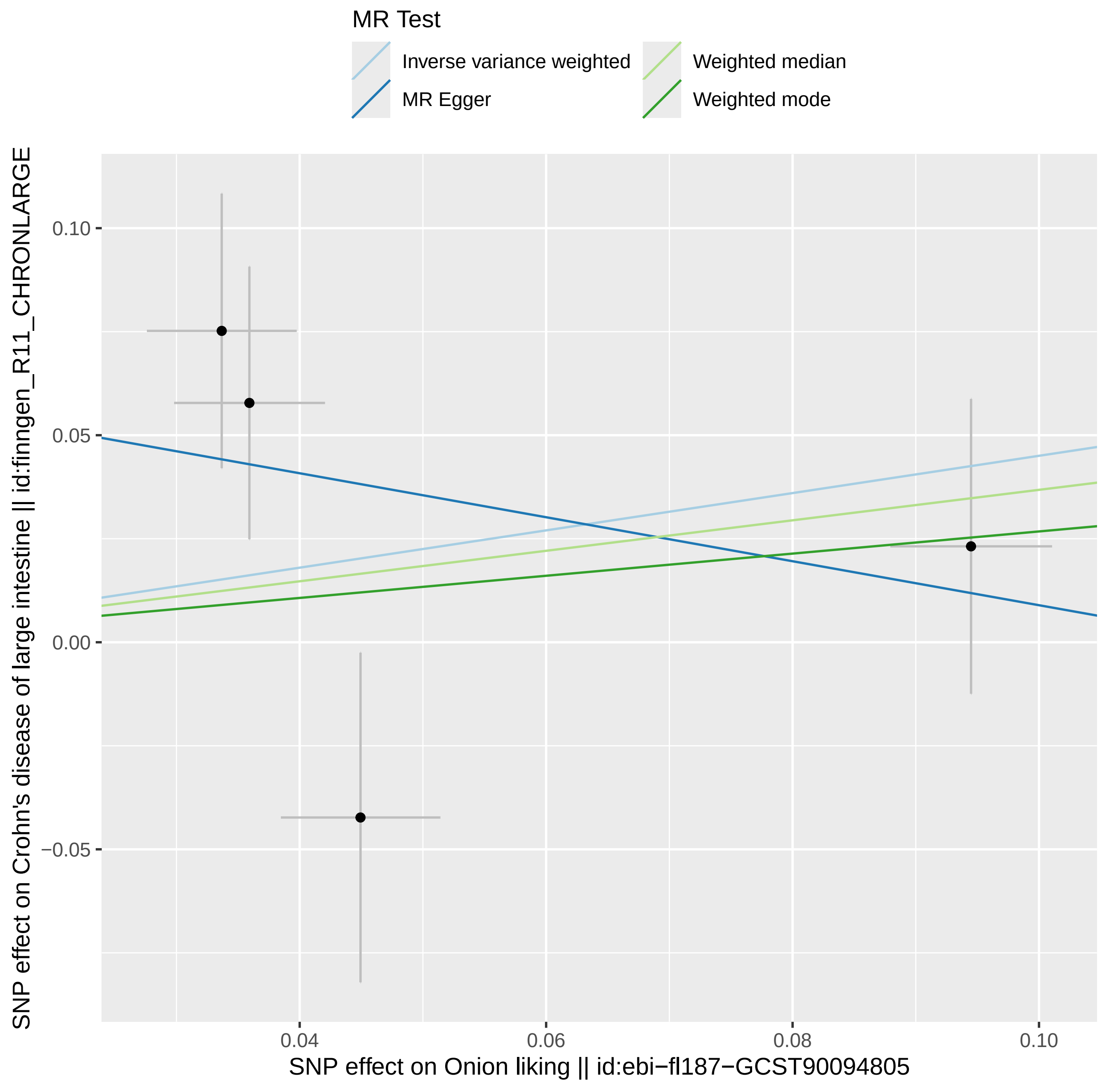
MR Test
Inverse variance weighted
MR Egger
Weighted median
Weighted mode
0.10
0.05
0.00
−0.05
0.04
0.06
0.08
0.10
SNP effect on Crohn's disease of large intestine || id:finngen_R11_CHRONLARGE
SNP effect on Onion liking || id:ebi−fl187−GCST90094805

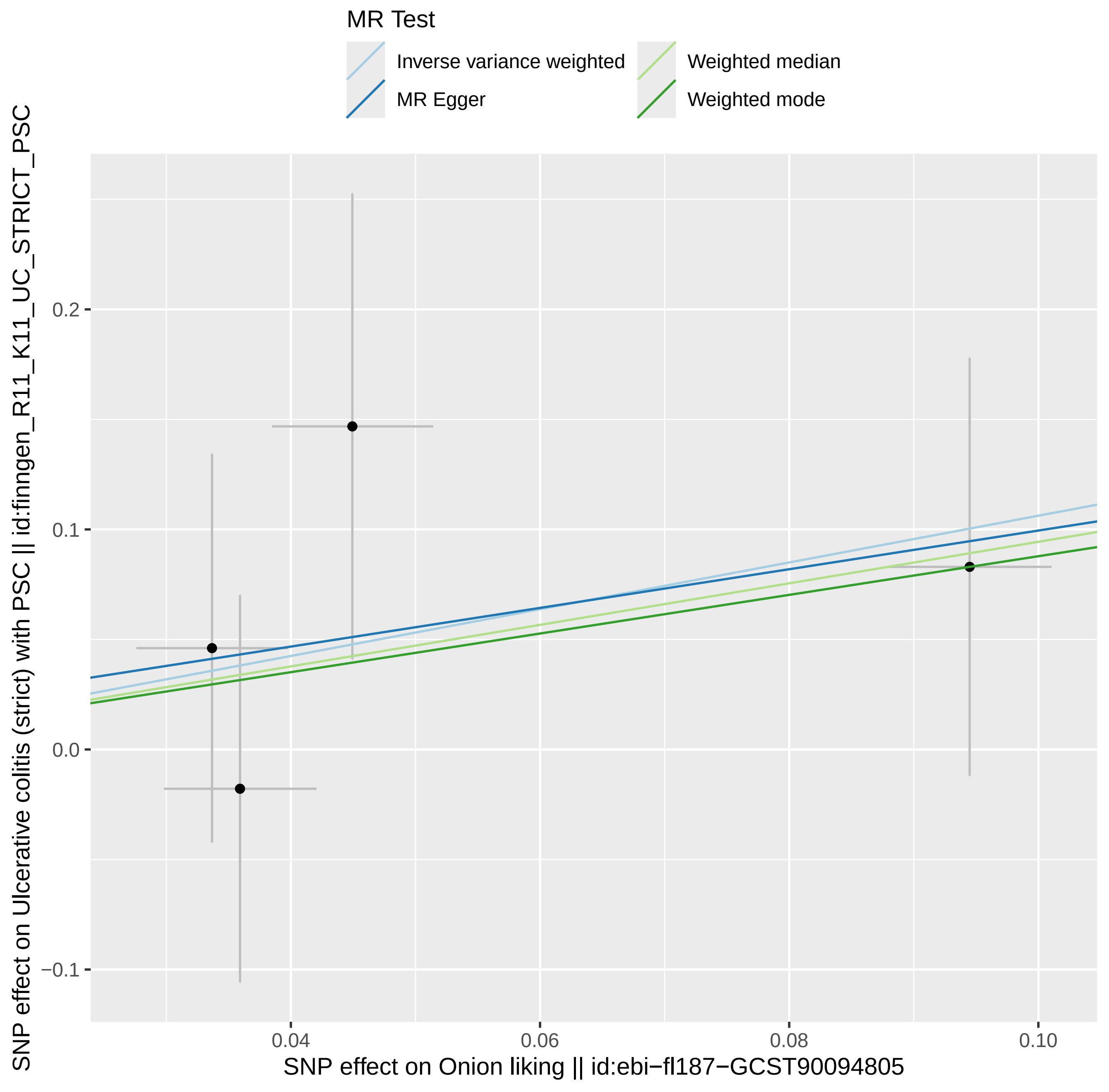
MR Test
Inverse variance weighted
MR Egger
Weighted median
Weighted mode
SNP effect on Ulcerative colitis (strict) with PSC || id:finngen_R11_K11_UC_STRICT_PSC
0.2
0.1
0.0
−0.1
0.04
0.06
0.08
0.10
SNP effect on Onion liking || id:ebi−fl187−GCST90094805

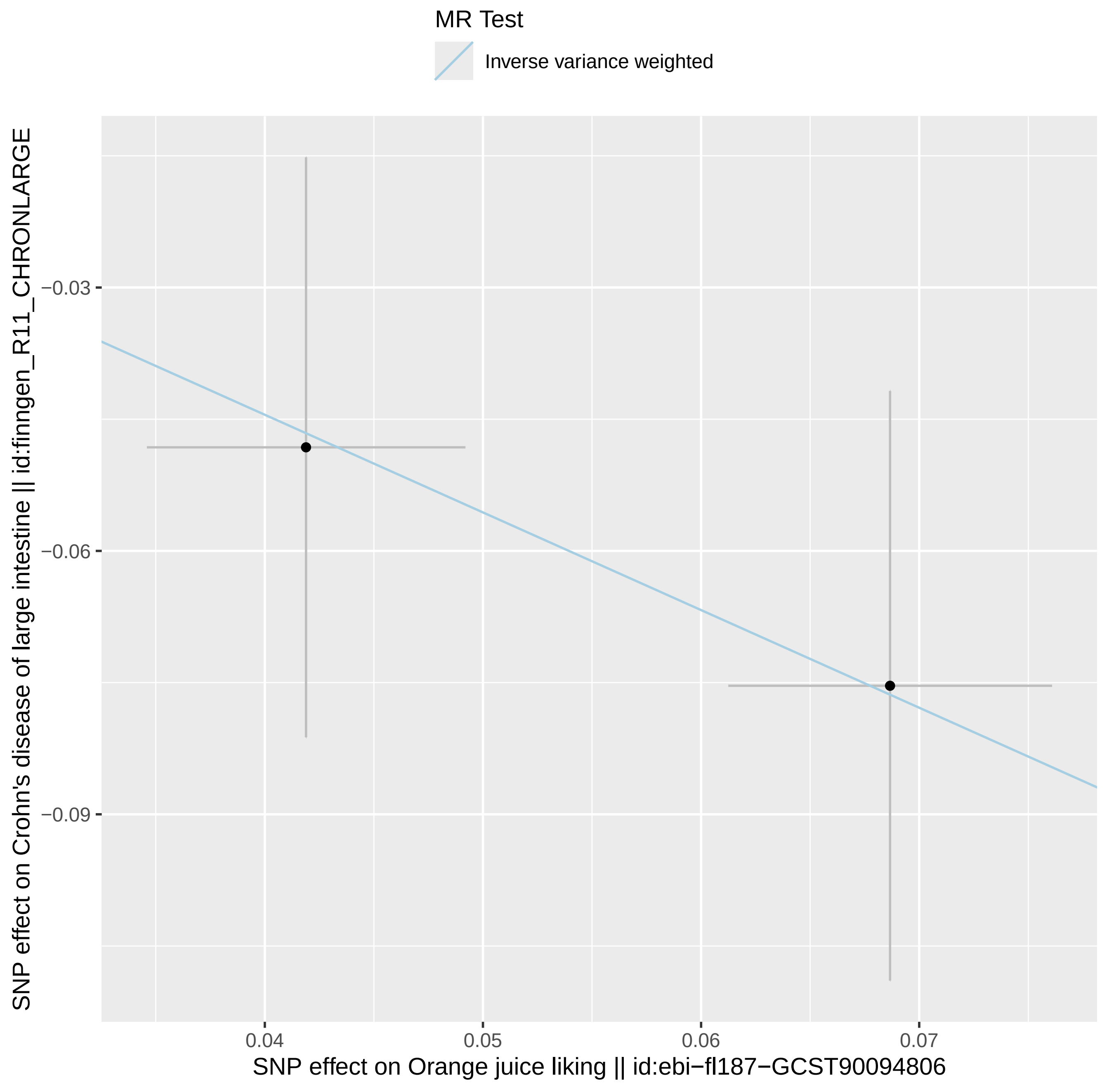
MR Test
Inverse variance weighted
−0.03
−0.06
−0.09
0.04
0.05
0.06
0.07
SNP effect on Crohn's disease of large intestine || id:finngen_R11_CHRONLARGE
SNP effect on Orange juice liking || id:ebi−fl187−GCST90094806

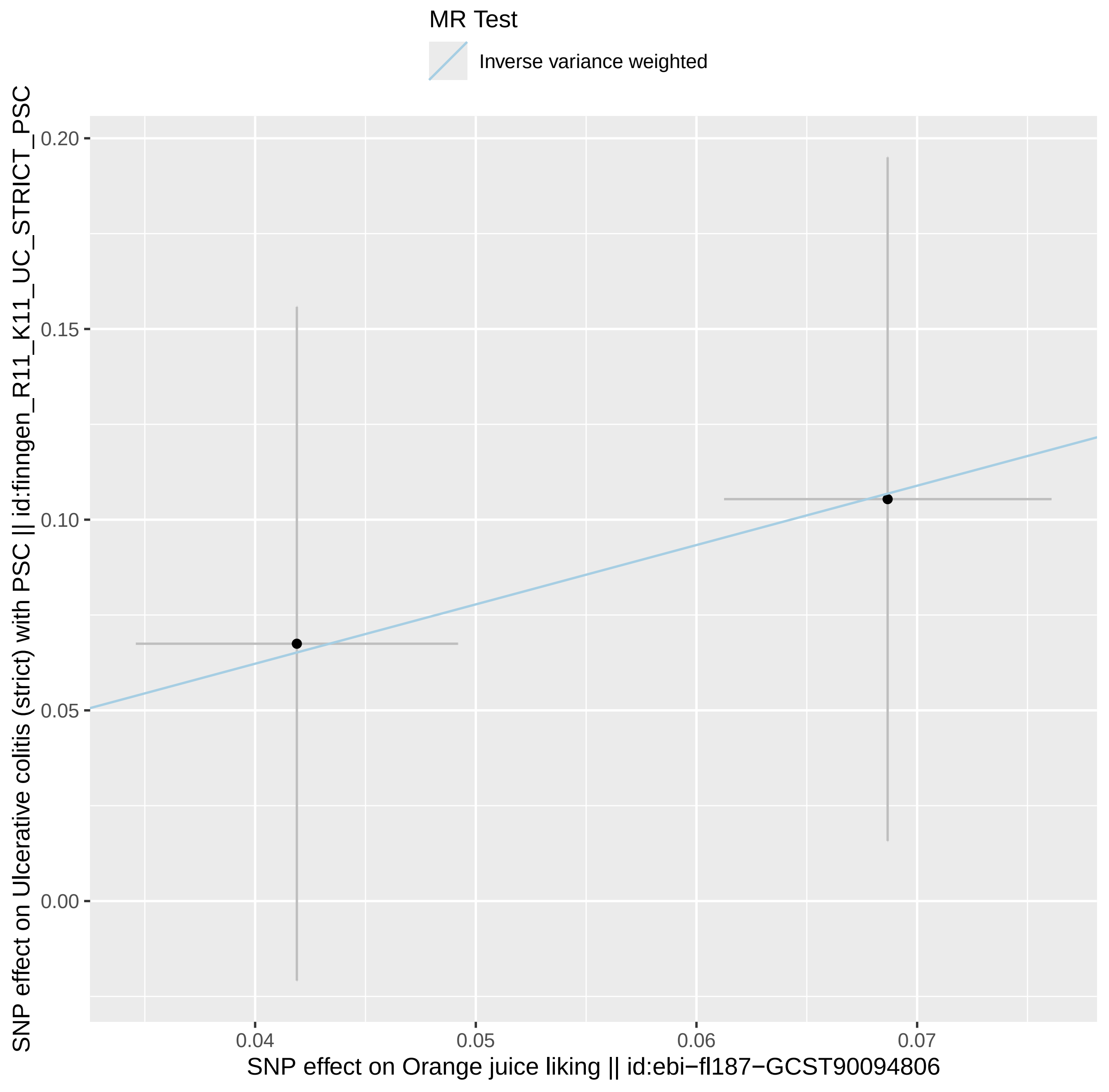

MR Test
Inverse variance weighted
0.20
0.15
0.10
0.05
0.00
0.04
0.05
0.06
0.07
SNP effect on Ulcerative colitis (strict) with PSC || id:finngen_R11_K11_UC_STRICT_PSC
SNP effect on Orange juice liking || id:ebi−fl187−GCST90094806

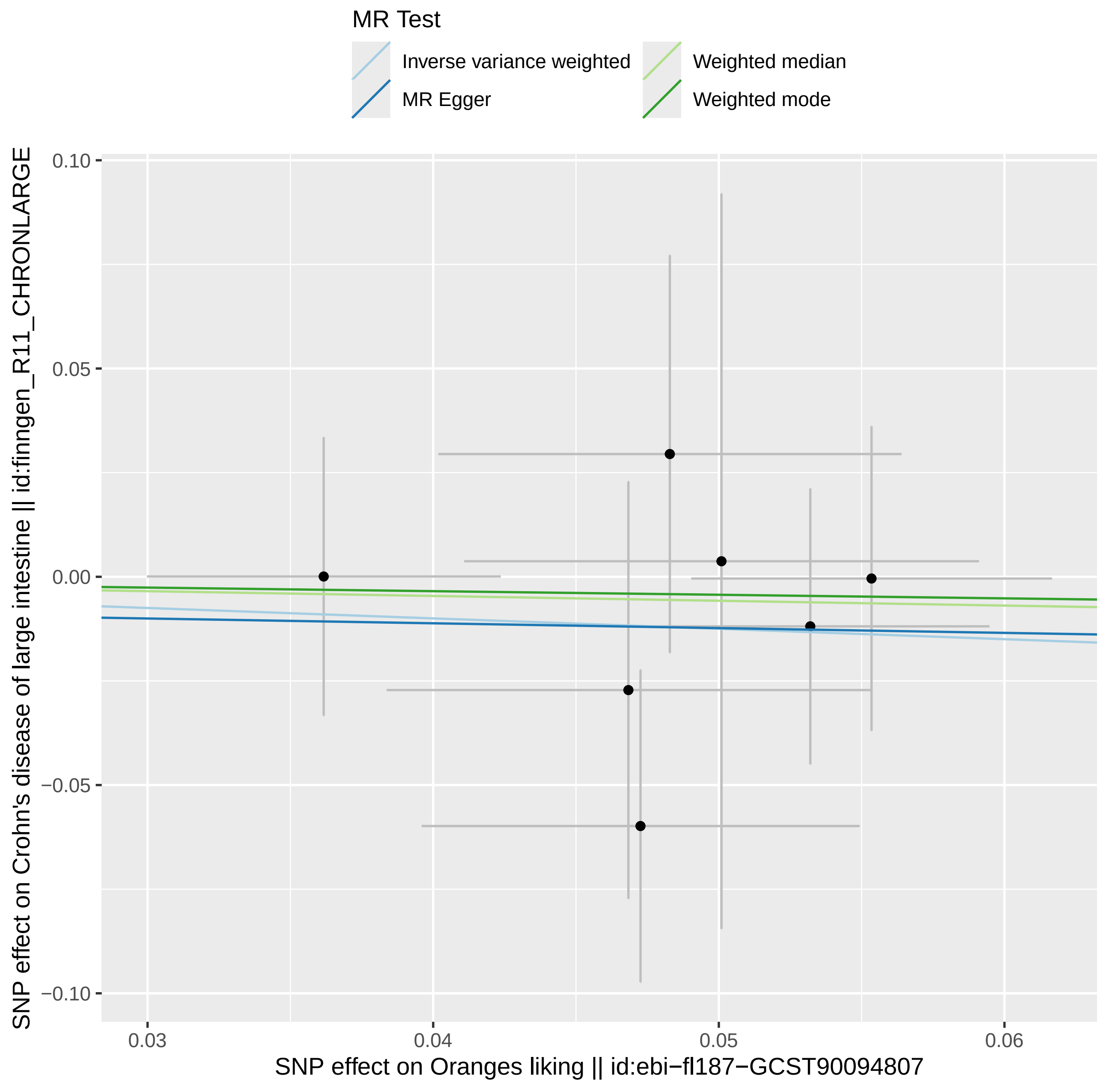
MR Test
Inverse variance weighted
MR Egger
Weighted median
Weighted mode
SNP effect on Crohn's disease of large intestine || id:finngen_R11_CHRONLARGE
0.10
0.05
0.00
−0.05
−0.10
0.03
0.04
0.05
0.06
SNP effect on Oranges liking || id:ebi−fl187−GCST90094807

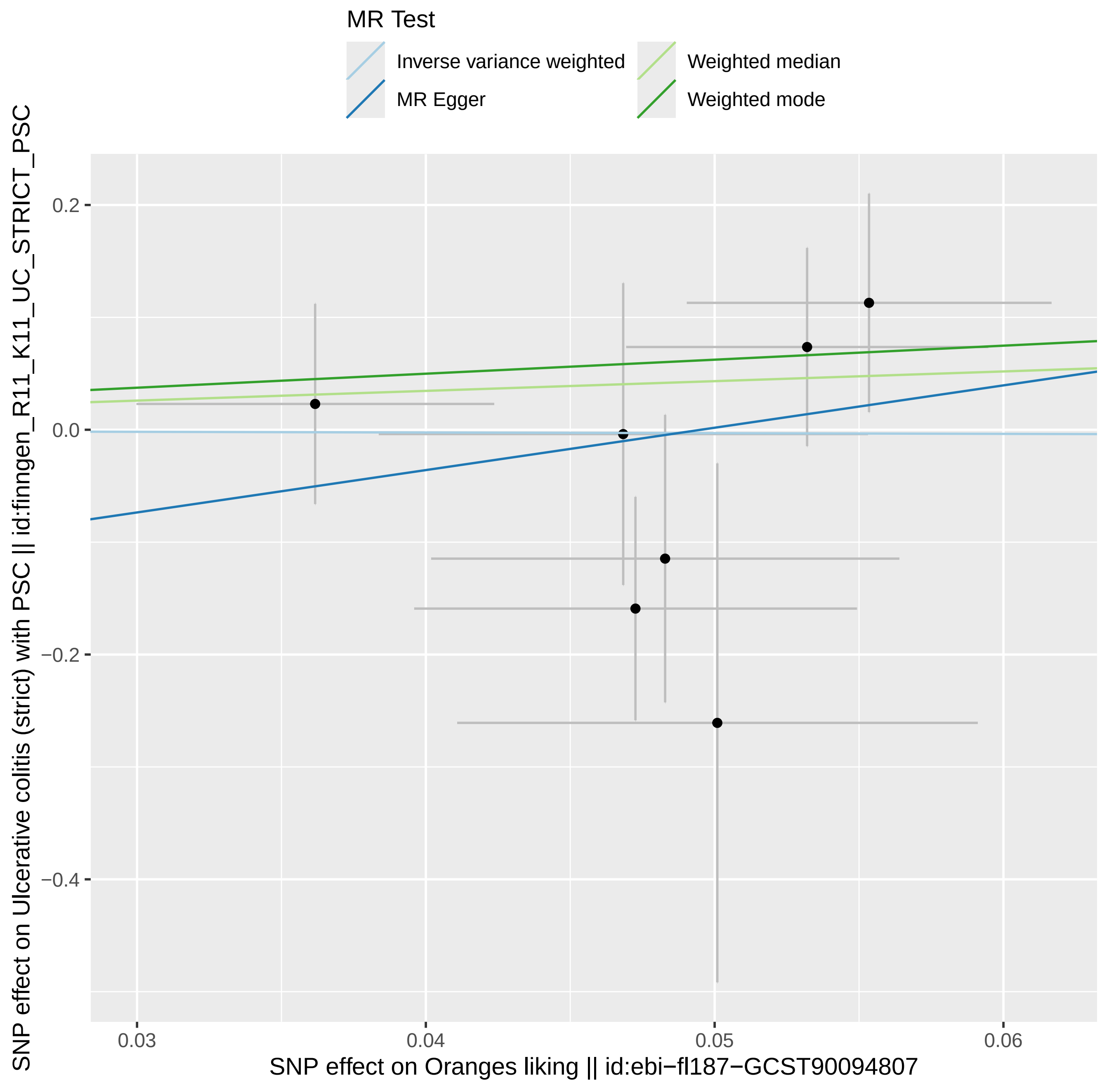

MR Test
Inverse variance weighted
MR Egger
Weighted median
Weighted mode
0.2
0.0
−0.2
−0.4
0.03
0.04
0.05
0.06
SNP effect on Ulcerative colitis (strict) with PSC || id:finngen_R11_K11_UC_STRICT_PSC
SNP effect on Oranges liking || id:ebi−fl187−GCST90094807

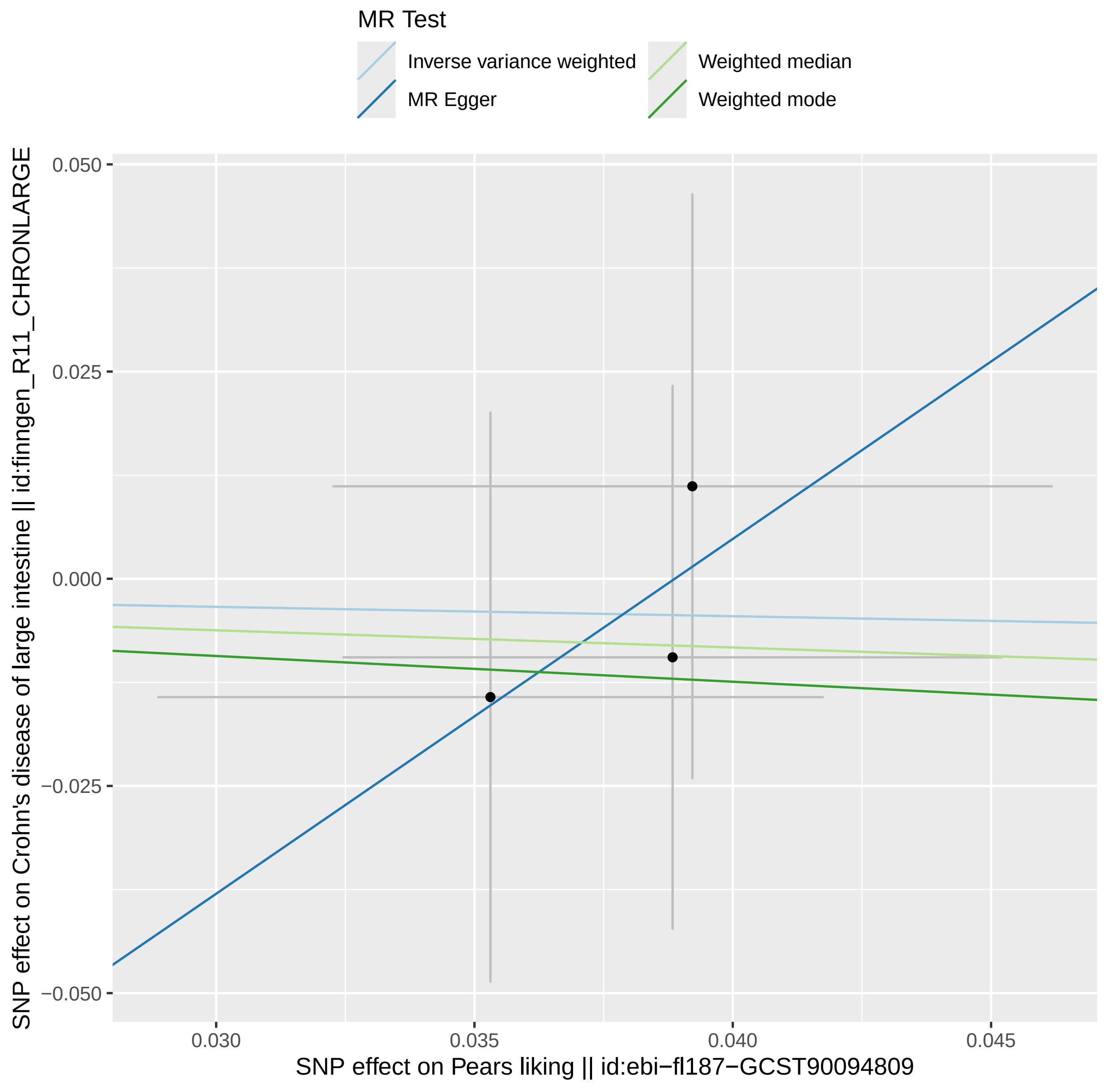

MR Test
Inverse variance weighted
MR Egger
Weighted median
Weighted mode
0.050
0.025
0.000
−0.025
−0.050
0.030
0.035
0.040
0.045
SNP effect on Crohn's disease of large intestine || id:finngen_R11_CHRONLARGE
SNP effect on Pears liking || id:ebi−fl187−GCST90094809

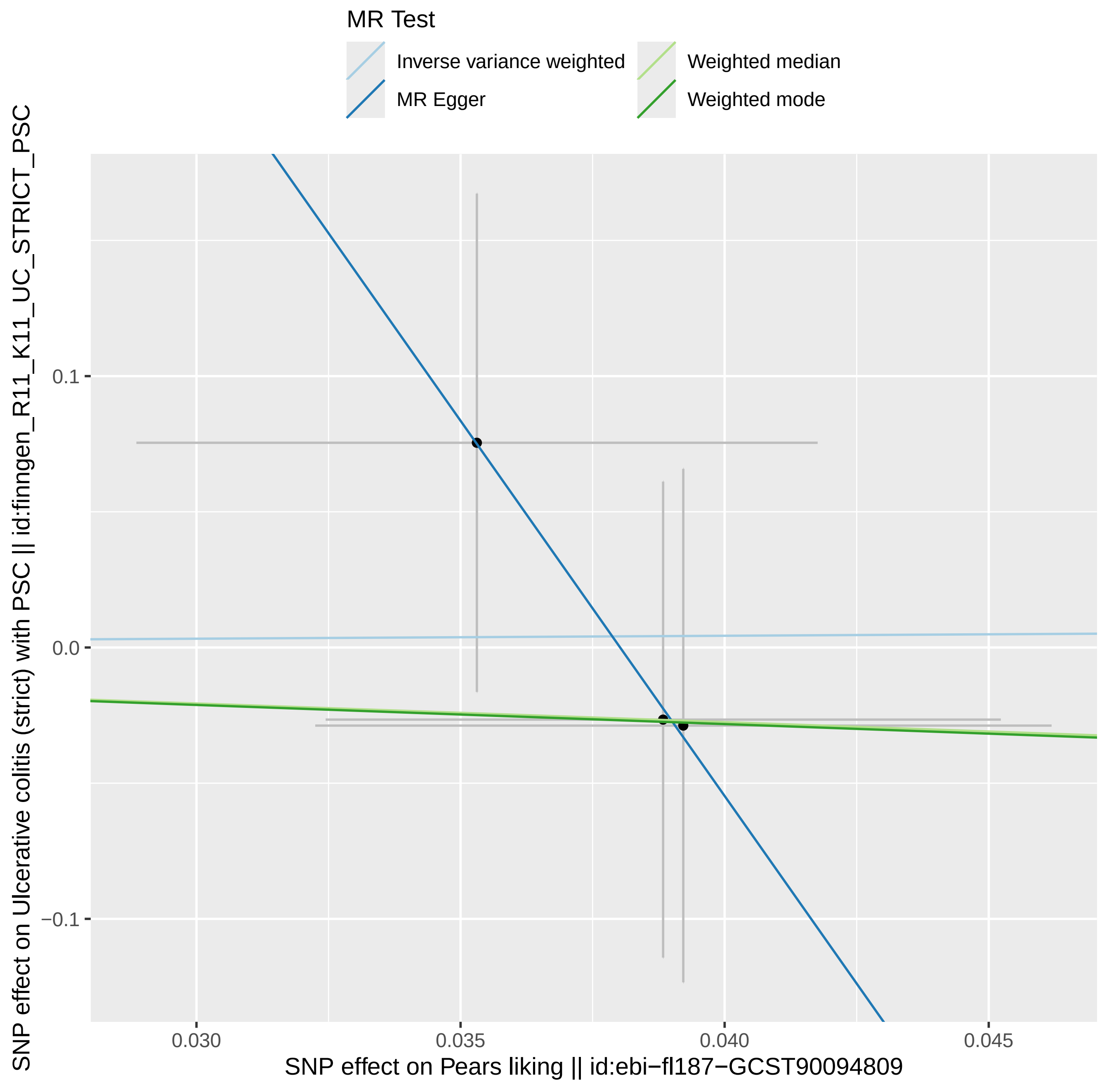

MR Test
Inverse variance weighted
MR Egger
Weighted median
Weighted mode
0.1
0.0
−0.1
0.030
0.035
0.040
0.045
SNP effect on Ulcerative colitis (strict) with PSC || id:finngen_R11_K11_UC_STRICT_PSC
SNP effect on Pears liking || id:ebi−fl187−GCST90094809

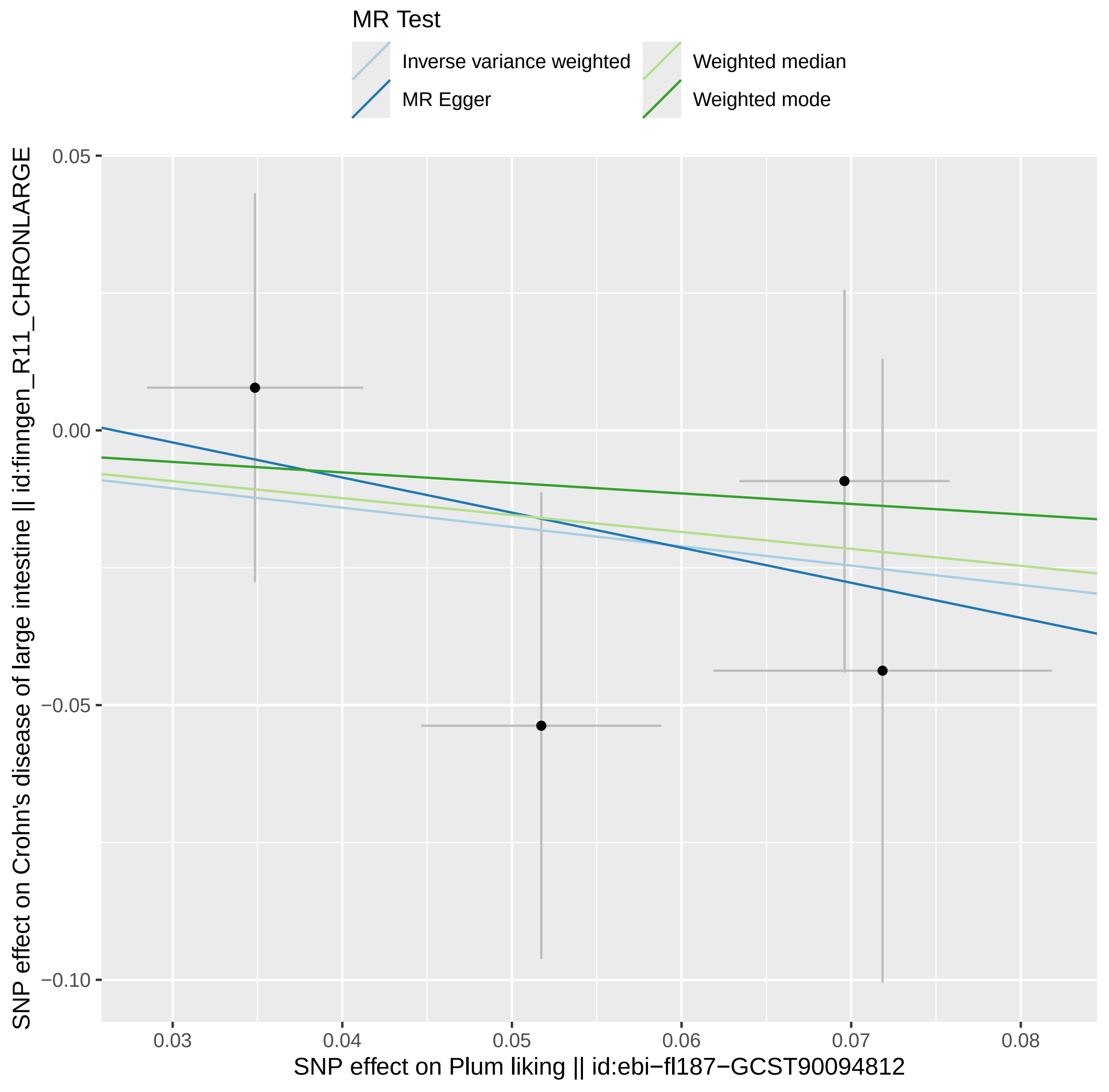
MR Test
Inverse variance weighted
MR Egger
Weighted median
Weighted mode
0.05
0.00
−0.05
−0.10
0.03
0.04
0.05
0.06
0.07
0.08
SNP effect on Crohn's disease of large intestine || id:finngen_R11_CHRONLARGE
SNP effect on Plum liking || id:ebi−fl187−GCST90094812

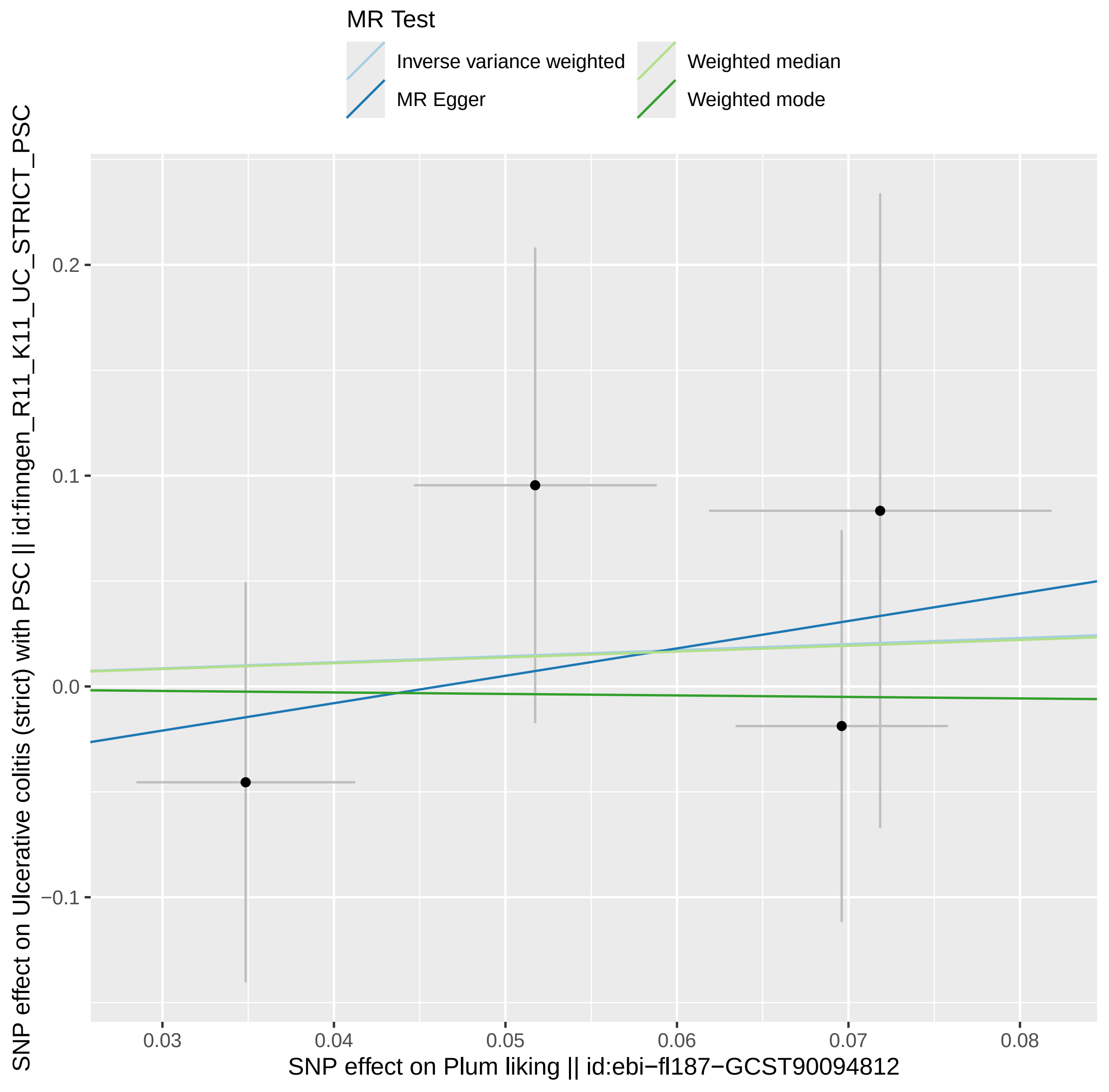

MR Test
Inverse variance weighted
Weighted median
MR Egger
Weighted mode
0.2
0.1
0.0
−0.1
0.03
0.04
0.05
0.06
0.07
0.08
SNP effect on Ulcerative colitis (strict) with PSC || id:finngen_R11_K11_UC_STRICT_PSC
SNP effect on Plum liking || id:ebi−fl187−GCST90094812

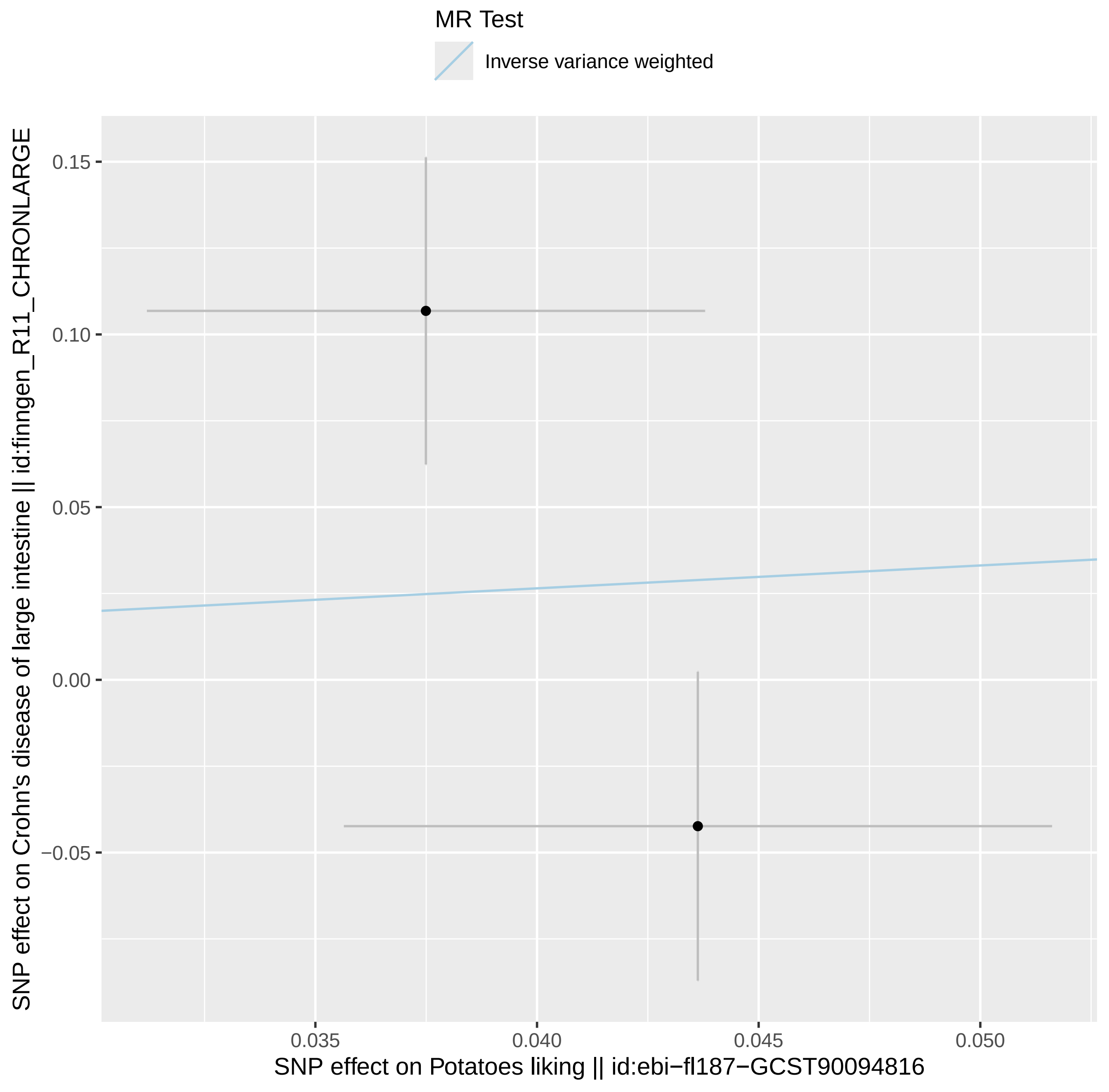
MR Test
Inverse variance weighted
SNP effect on Crohn's disease of large intestine || id:finngen_R11_CHRONLARGE
0.15
0.10
0.05
0.00
−0.05
0.035
0.040
0.045
0.050
SNP effect on Potatoes liking || id:ebi−fl187−GCST90094816

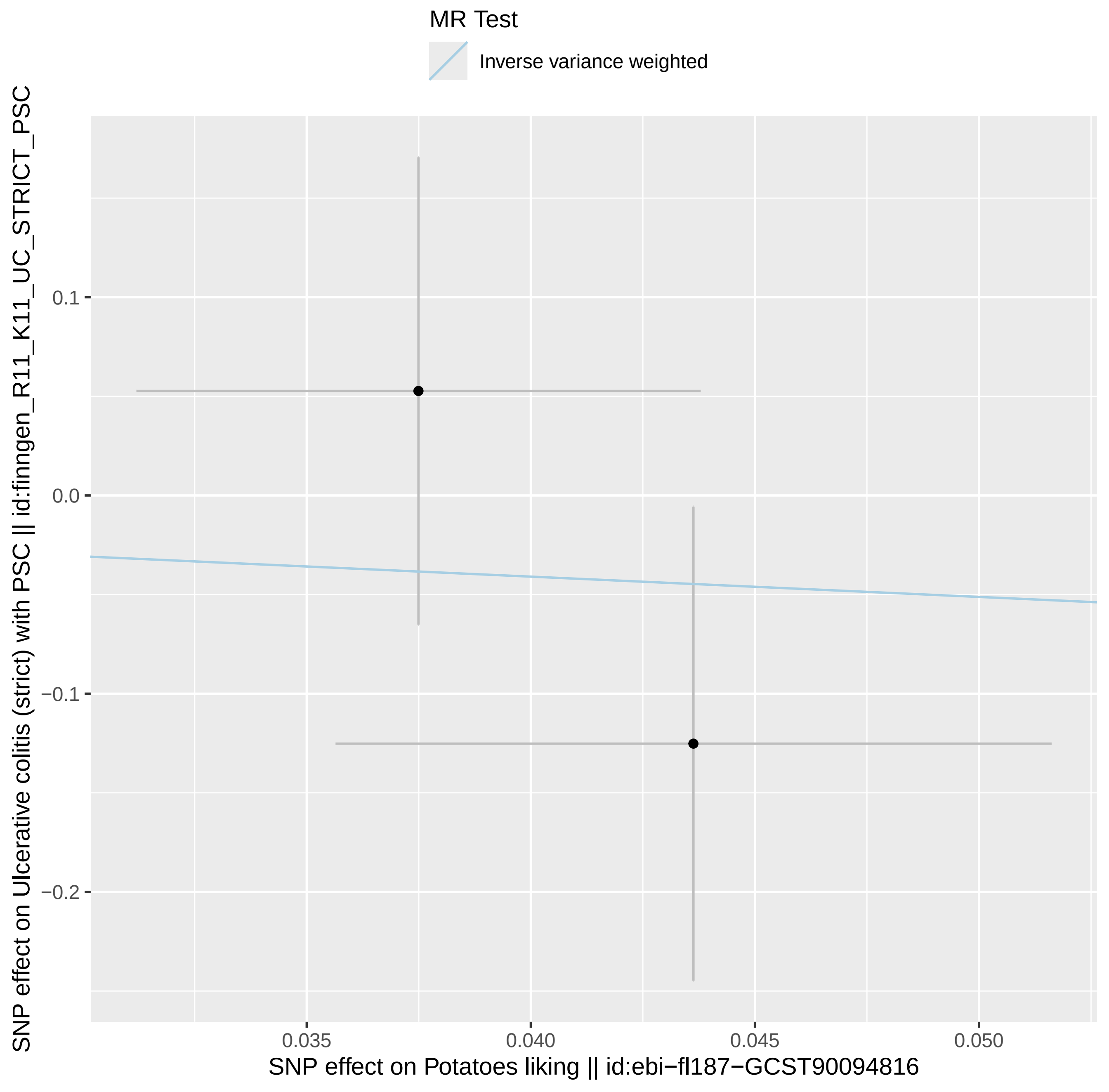
MR Test
Inverse variance weighted
SNP effect on Ulcerative colitis (strict) with PSC || id:finngen_R11_K11_UC_STRICT_PSC
0.1
0.0
−0.1
−0.2
0.035
0.040
0.045
0.050
SNP effect on Potatoes liking || id:ebi−fl187−GCST90094816

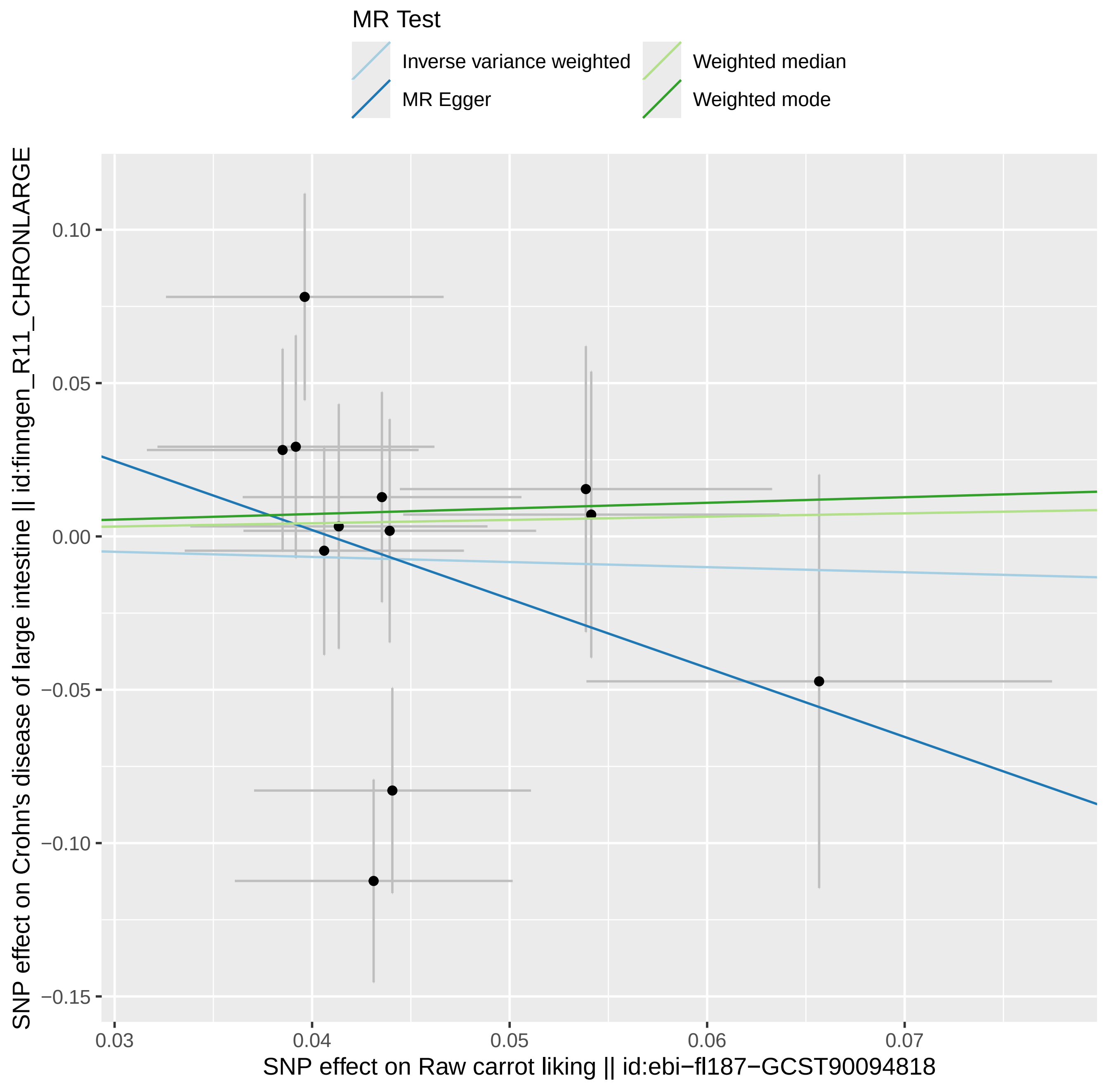

MR Test
Inverse variance weighted
MR Egger
Weighted median
Weighted mode
0.10
0.05
0.00
−0.05
−0.10
−0.15
0.03
0.04
0.05
0.06
0.07
SNP effect on Raw carrot liking || id:ebi−fl187−GCST90094818
SNP effect on Crohn's disease of large intestine || id:finngen_R11_CHRONLARGE

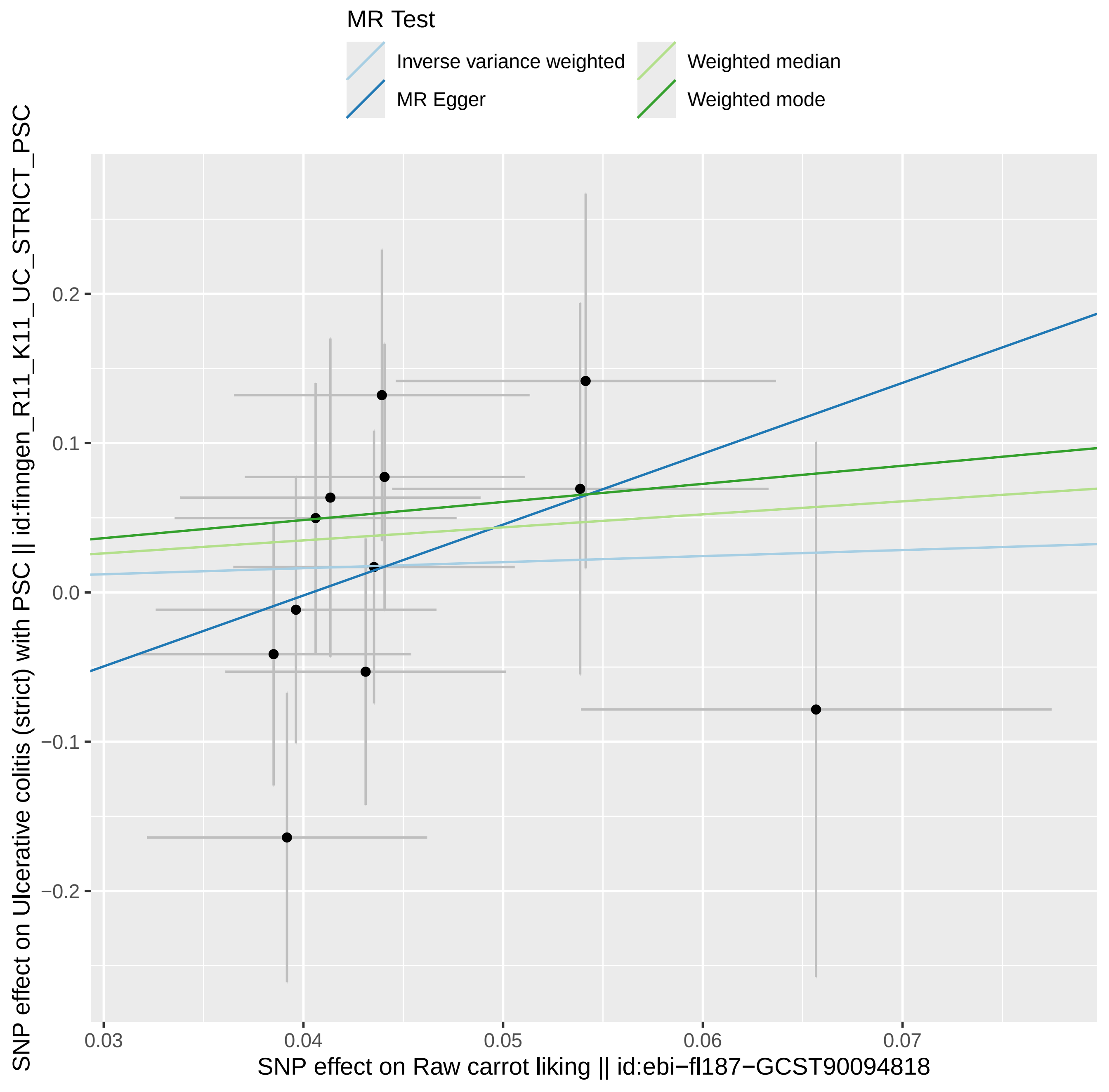
MR Test
Inverse variance weighted
MR Egger
Weighted median
Weighted mode
0.2
0.1
0.0
−0.1
−0.2
0.03
0.04
0.05
0.06
0.07
SNP effect on Ulcerative colitis (strict) with PSC || id:finngen_R11_K11_UC_STRICT_PSC
SNP effect on Raw carrot liking || id:ebi−fl187−GCST90094818

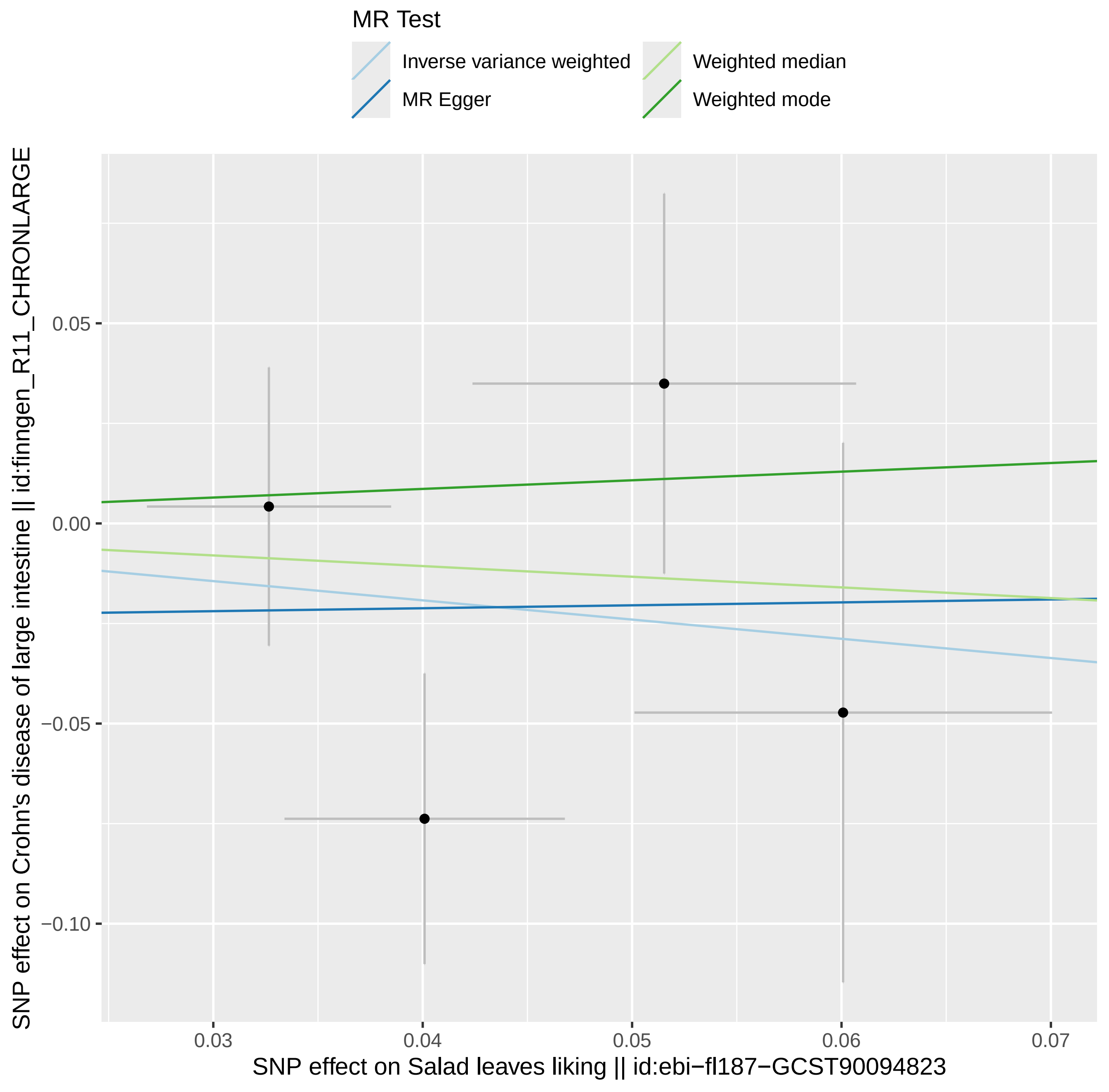
MR Test
Inverse variance weighted
MR Egger
Weighted median
Weighted mode
SNP effect on Crohn's disease of large intestine || id:finngen_R11_CHRONLARGE
0.05
0.00
−0.05
−0.10
0.03
0.04
0.05
0.06
0.07
SNP effect on Salad leaves liking || id:ebi−fl187−GCST90094823

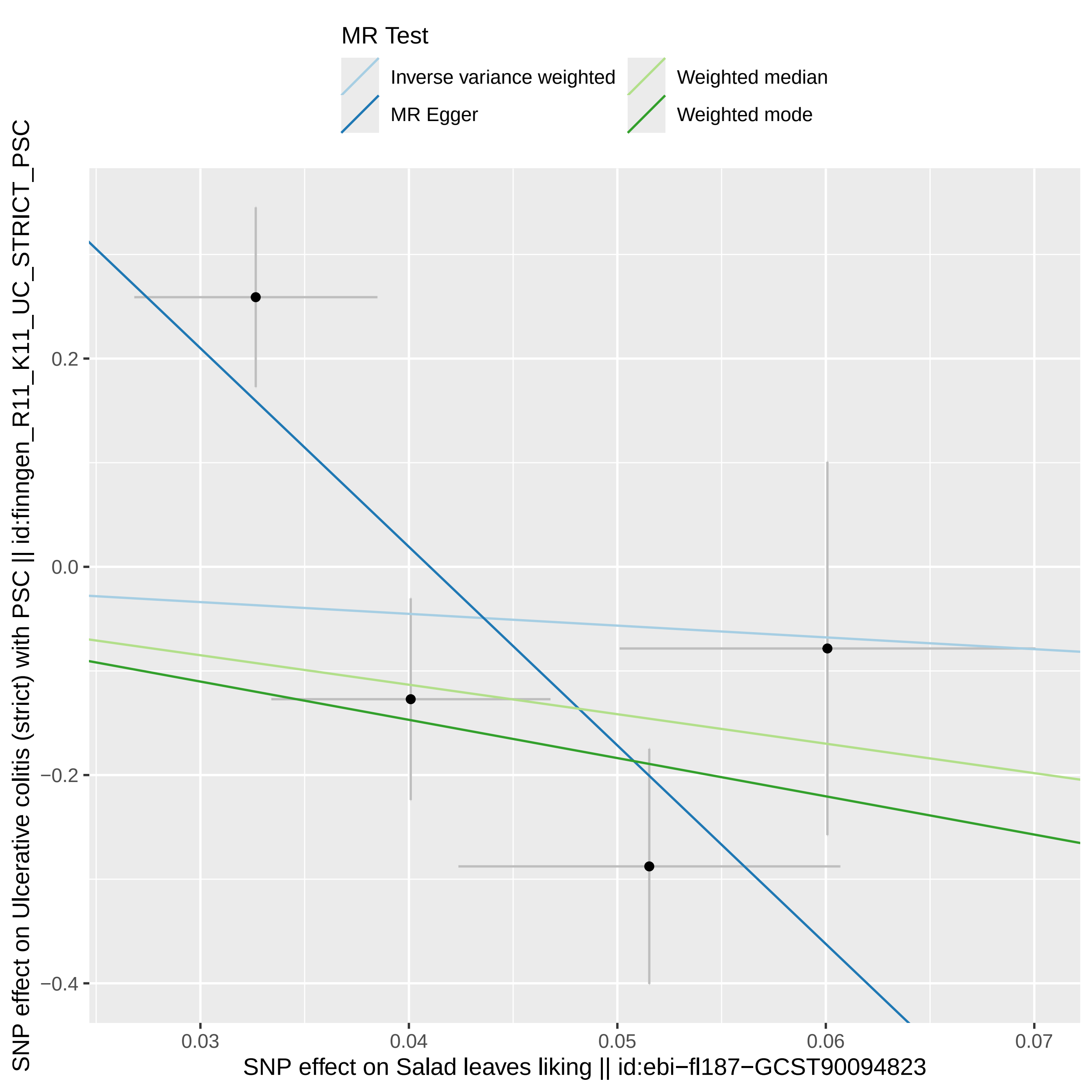
MR Test
Inverse variance weighted
MR Egger
Weighted median
Weighted mode
SNP effect on Ulcerative colitis (strict) with PSC || id:finngen_R11_K11_UC_STRICT_PSC
0.2
0.0
−0.2
−0.4
0.03
0.04
0.05
0.06
0.07
SNP effect on Salad leaves liking || id:ebi−fl187−GCST90094823

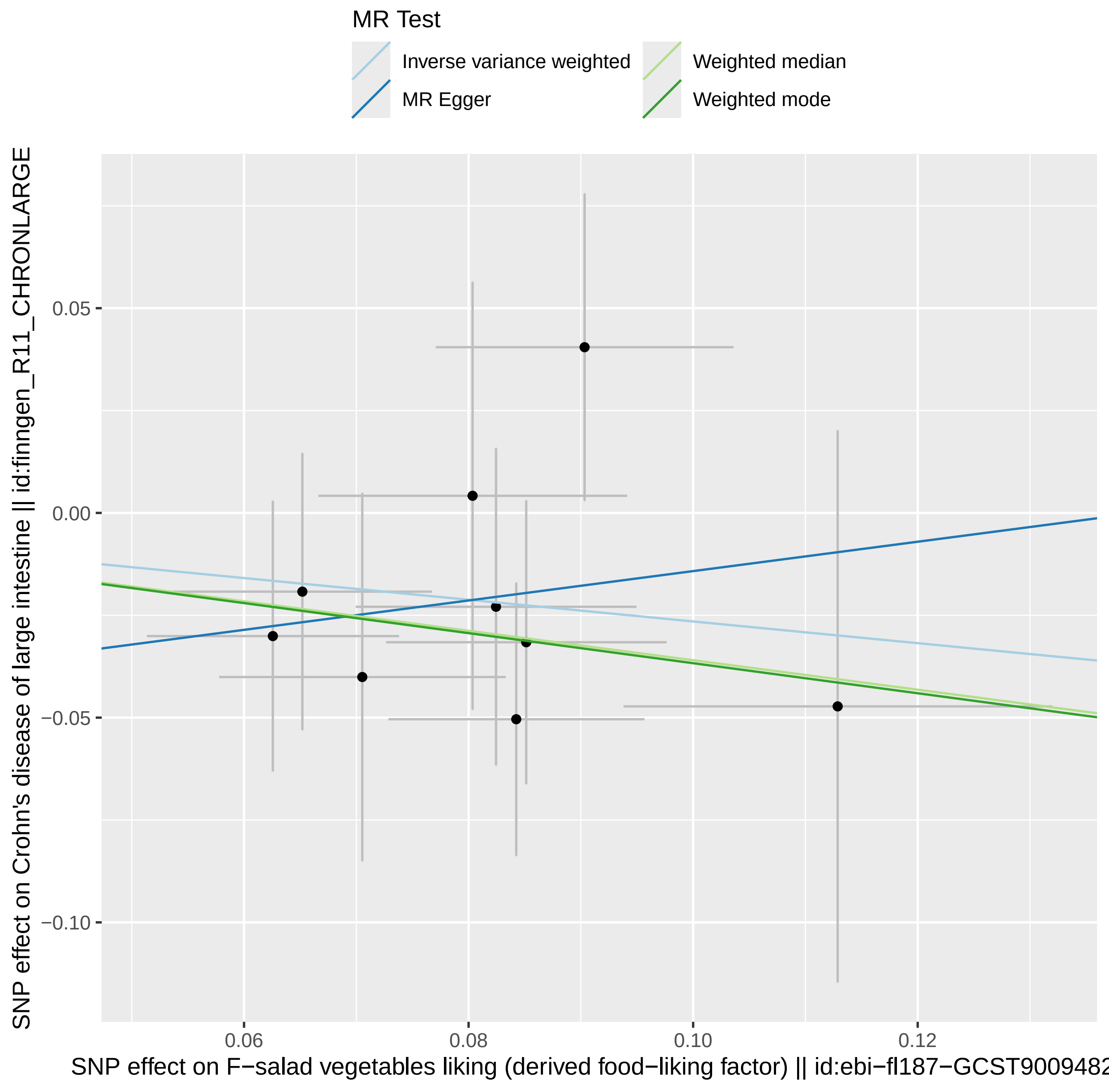

MR Test
Inverse variance weighted
MR Egger
Weighted median
Weighted mode
0.05
0.00
−0.05
−0.10
0.06
0.08
0.10
0.12
SNP effect on Crohn's disease of large intestine || id:finngen_R11_CHRONLARGE
SNP effect on F−salad vegetables liking (derived food−liking factor) || id:ebi−fl187−GCST9009482

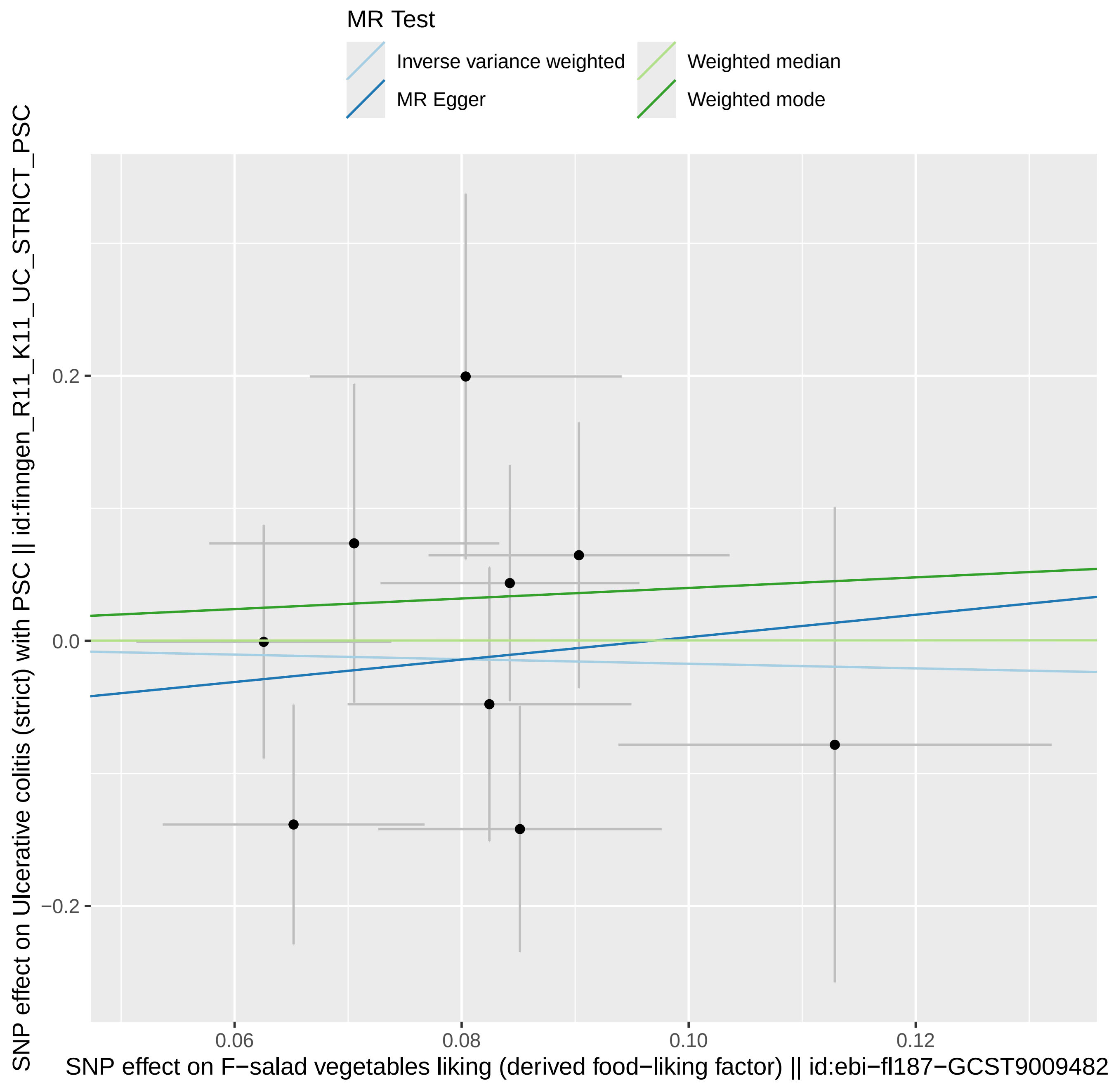
MR Test
Inverse variance weighted
MR Egger
Weighted median
Weighted mode
0.2
0.0
−0.2
0.06
0.08
0.10
0.12
SNP effect on Ulcerative colitis (strict) with PSC || id:finngen_R11_K11_UC_STRICT_PSC
SNP effect on F−salad vegetables liking (derived food−liking factor) || id:ebi−fl187−GCST9009482

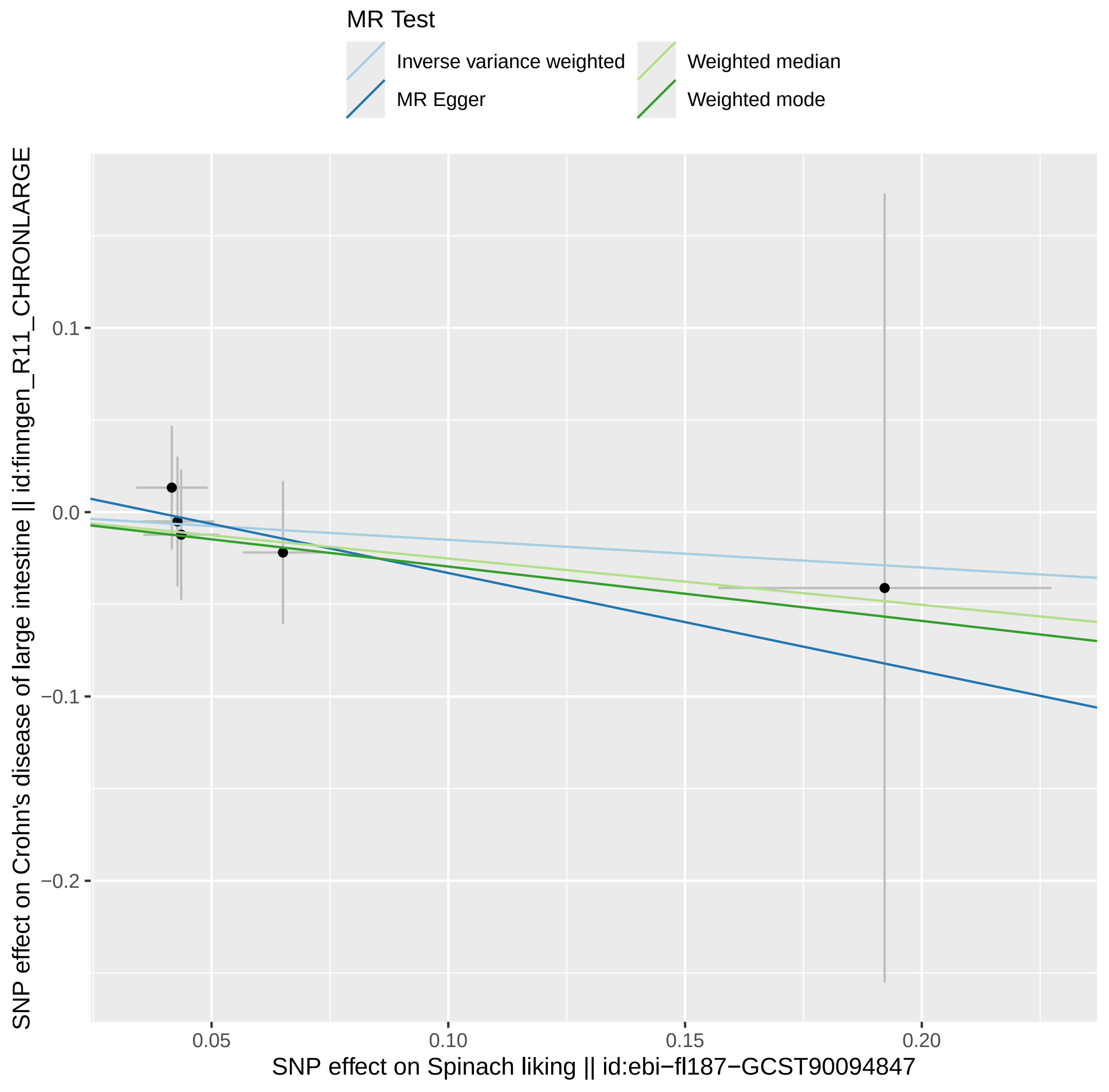
MR Test
Inverse variance weighted
MR Egger
Weighted median
Weighted mode
0.1
0.0
−0.1
−0.2
0.05
0.10
0.15
0.20
SNP effect on Crohn's disease of large intestine || id:finngen_R11_CHRONLARGE
SNP effect on Spinach liking || id:ebi−fl187−GCST90094847

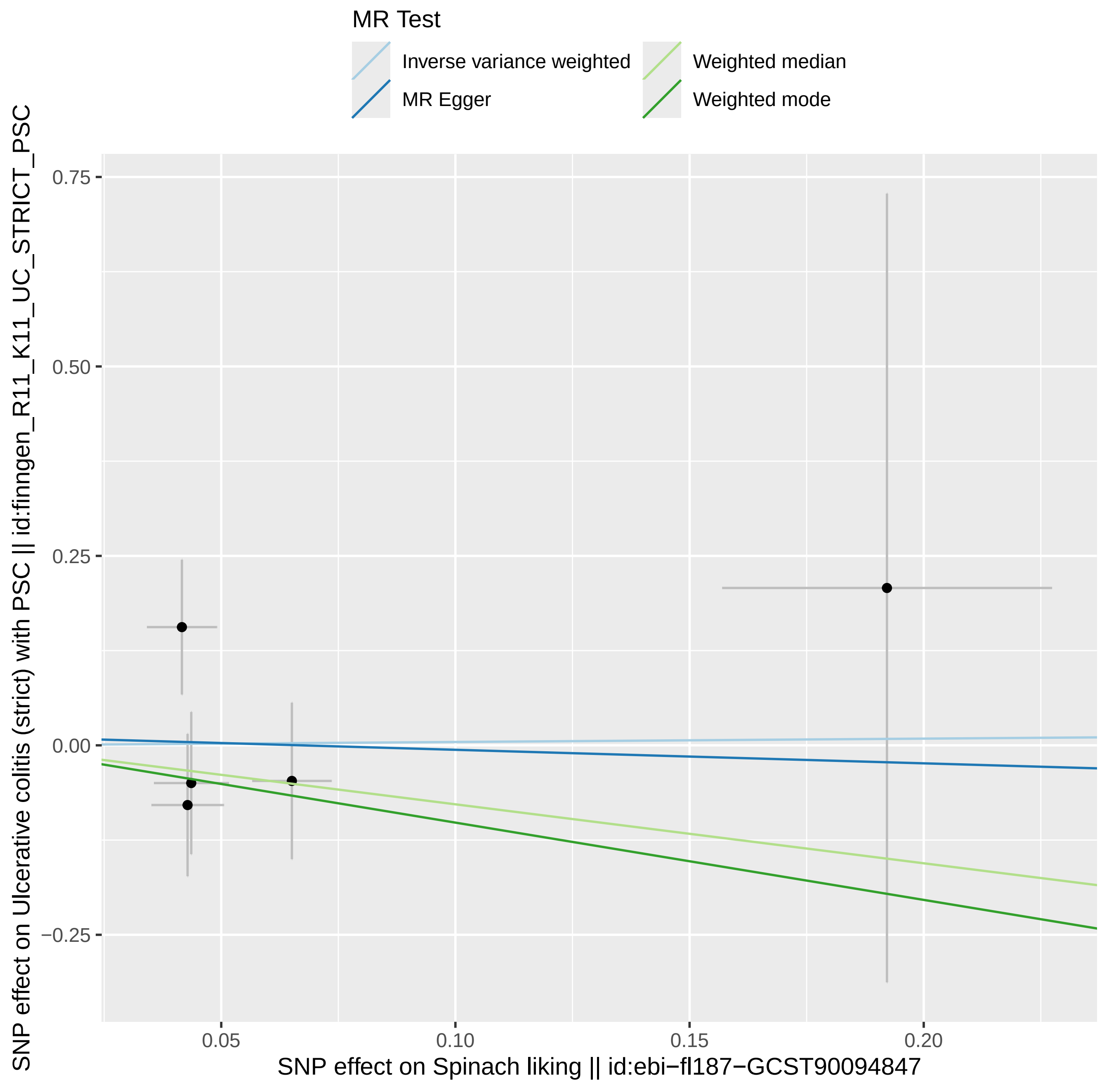
MR Test
Inverse variance weighted
MR Egger
Weighted median
Weighted mode
0.75
0.50
0.25
0.00
−0.25
0.05
0.10
0.15
0.20
SNP effect on Spinach liking || id:ebi−fl187−GCST90094847
SNP effect on Ulcerative colitis (strict) with PSC || id:finngen_R11_K11_UC_STRICT_PSC

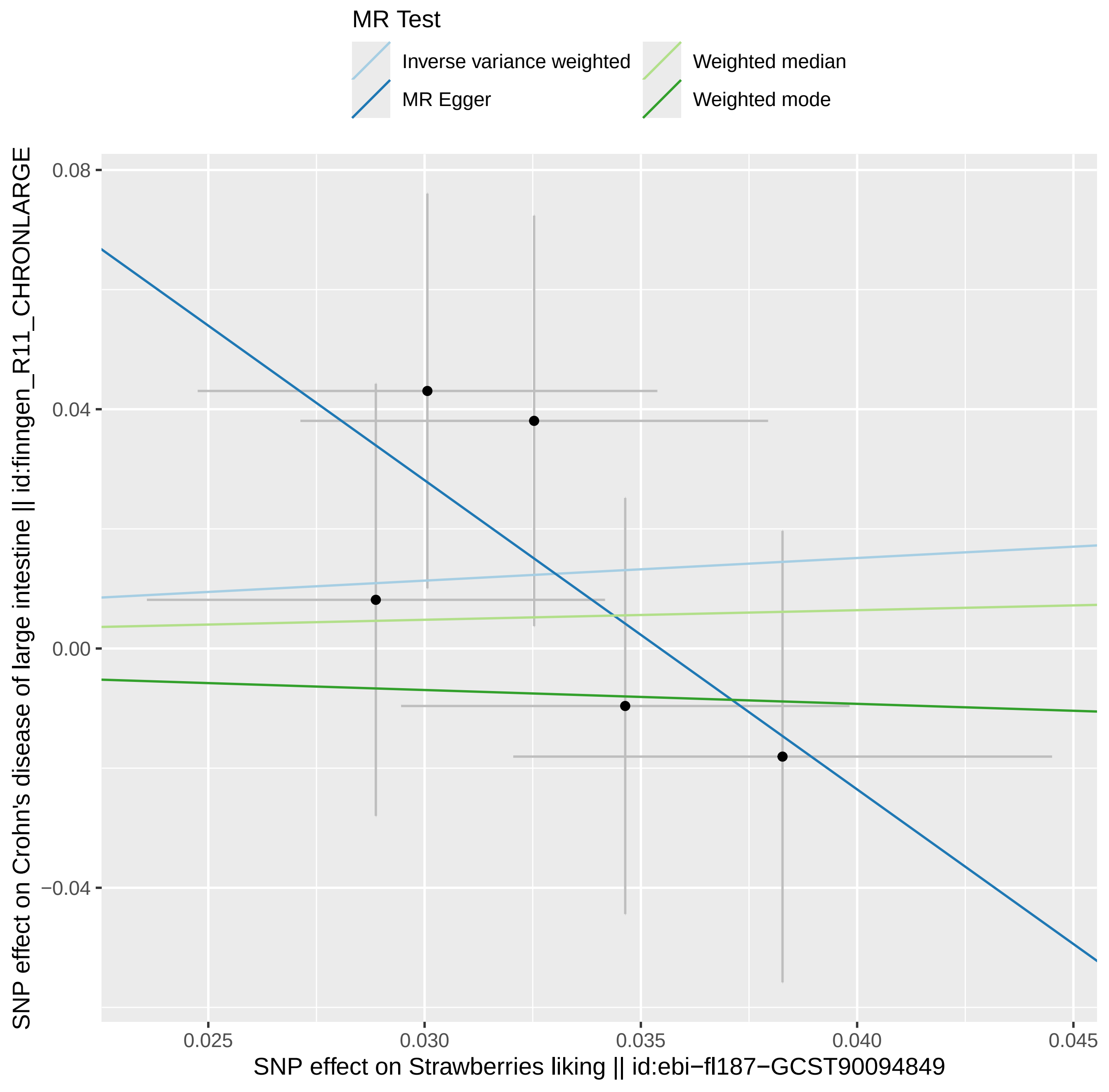

MR Test
Inverse variance weighted
MR Egger
Weighted median
Weighted mode
0.08
0.04
0.00
−0.04
0.025
0.030
0.035
0.040
0.045
SNP effect on Crohn's disease of large intestine || id:finngen_R11_CHRONLARGE
SNP effect on Strawberries liking || id:ebi−fl187−GCST90094849

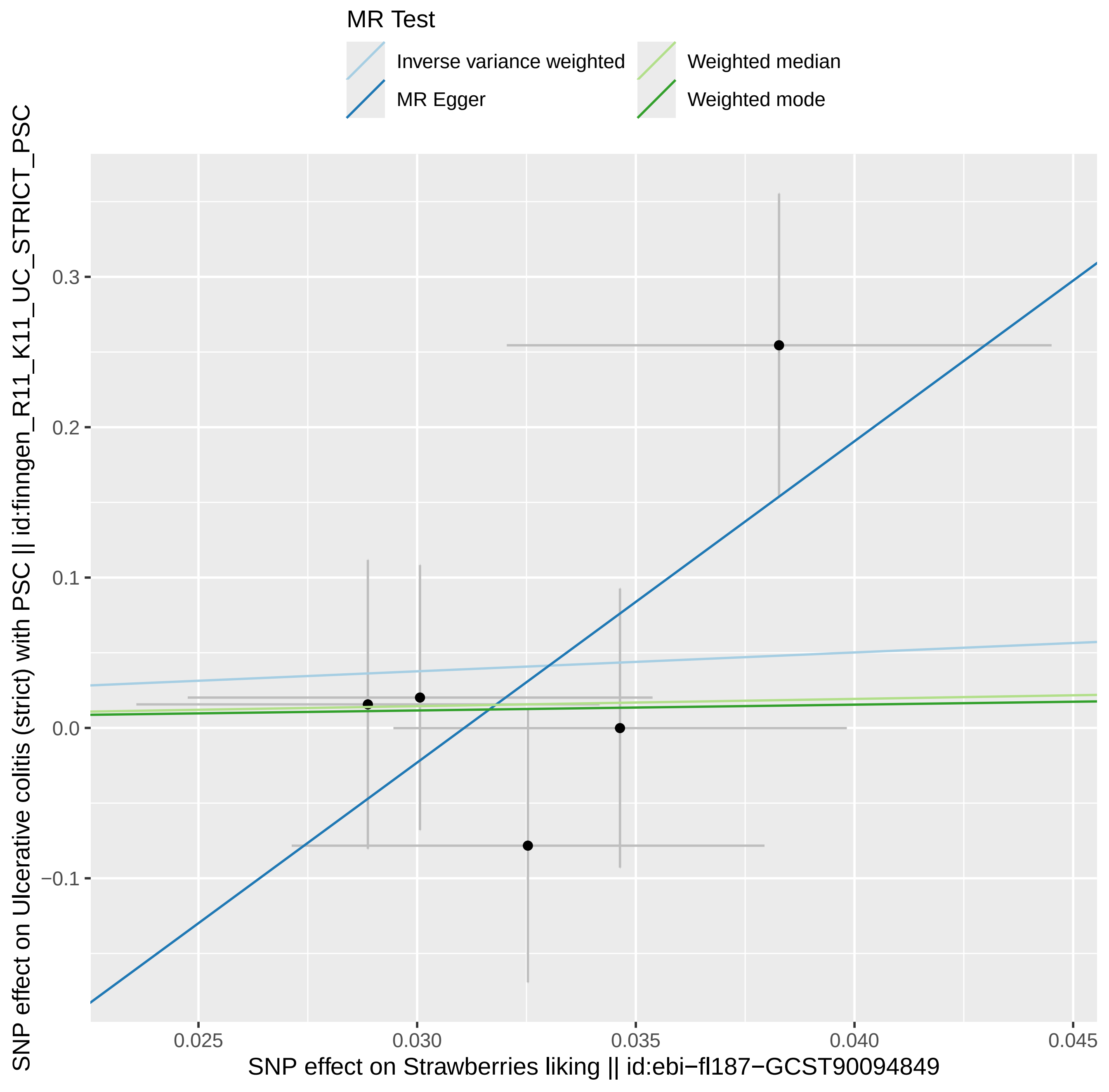

MR Test
Inverse variance weighted
MR Egger
Weighted median
Weighted mode
0.3
0.2
0.1
0.0
−0.1
0.025
0.030
0.035
0.040
0.045
SNP effect on Strawberries liking || id:ebi−fl187−GCST90094849
SNP effect on Ulcerative colitis (strict) with PSC || id:finngen_R11_K11_UC_STRICT_PSC

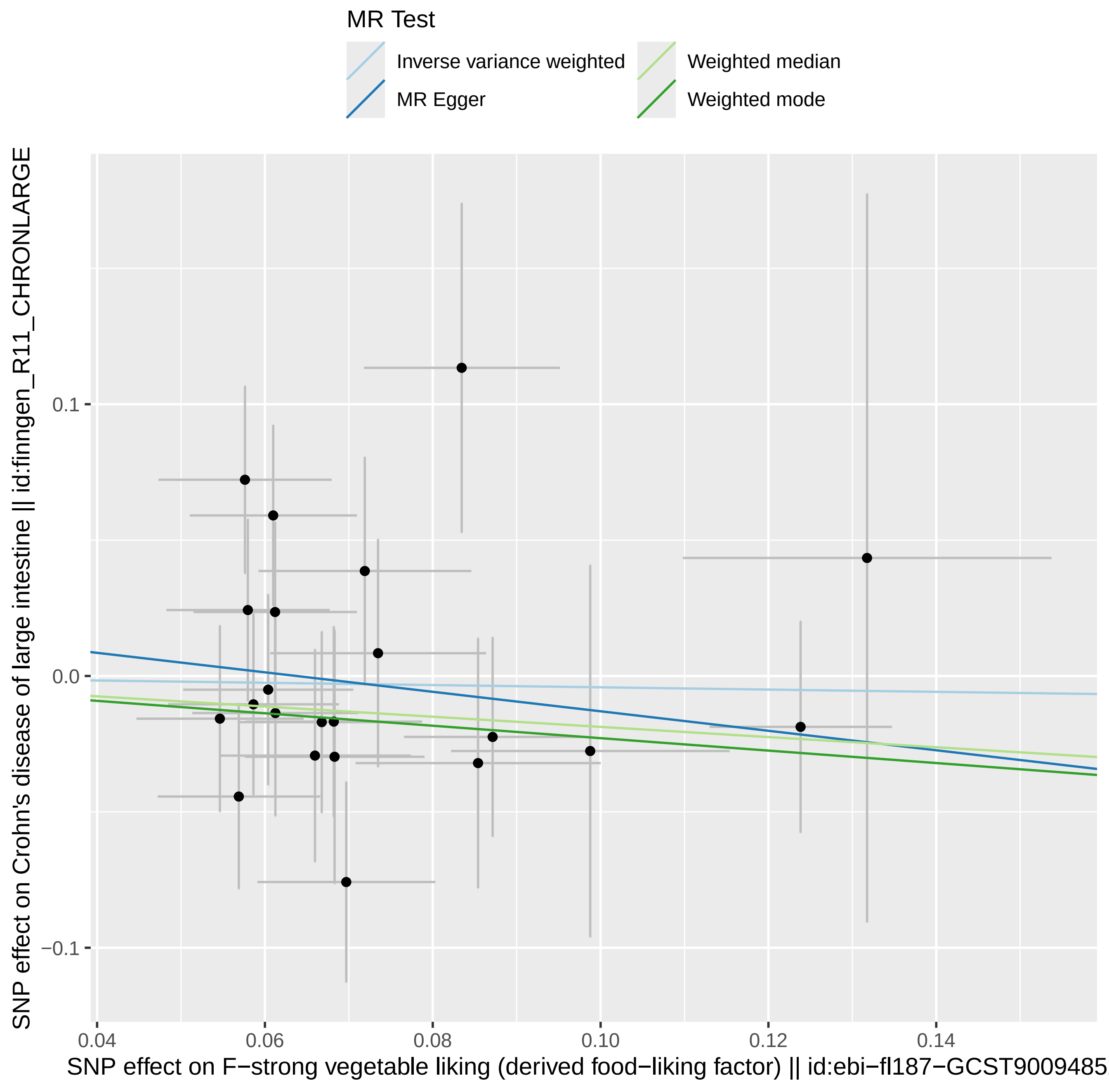

MR Test
Inverse variance weighted
MR Egger
Weighted median
Weighted mode
SNP effect on Crohn's disease of large intestine || id:finngen_R11_CHRONLARGE
0.1
0.0
−0.1
0.04
0.06
0.08
0.10
0.12
0.14
SNP effect on F−strong vegetable liking (derived food−liking factor) || id:ebi−fl187−GCST9009485

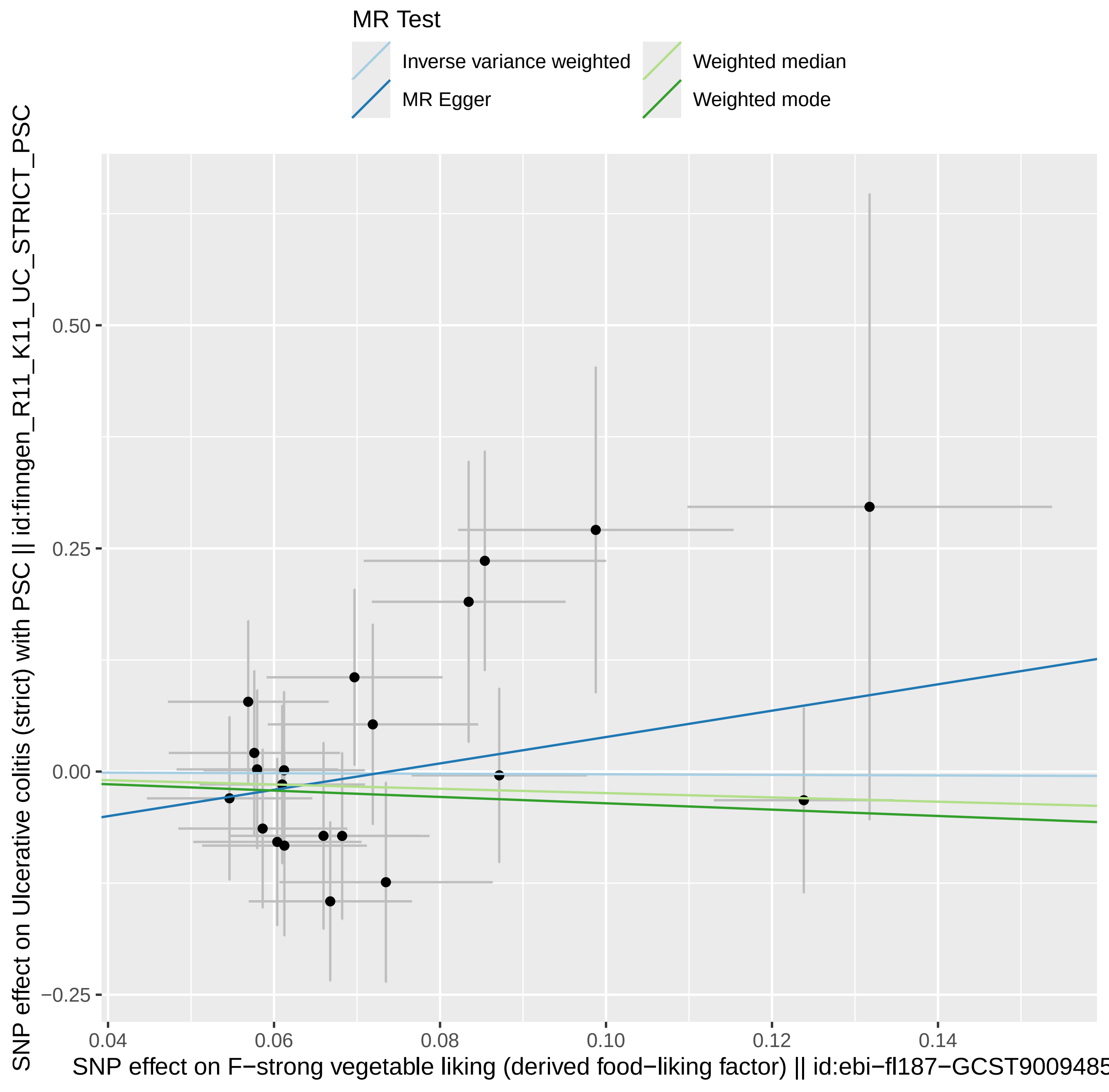

MR Test
Inverse variance weighted
MR Egger
Weighted median
Weighted mode
0.50
0.25
0.00
−0.25
0.04
0.06
0.08
0.10
0.12
0.14
SNP effect on F−strong vegetable liking (derived food−liking factor) || id:ebi−fl187−GCST9009485
SNP effect on Ulcerative colitis (strict) with PSC || id:finngen_R11_K11_UC_STRICT_PSC

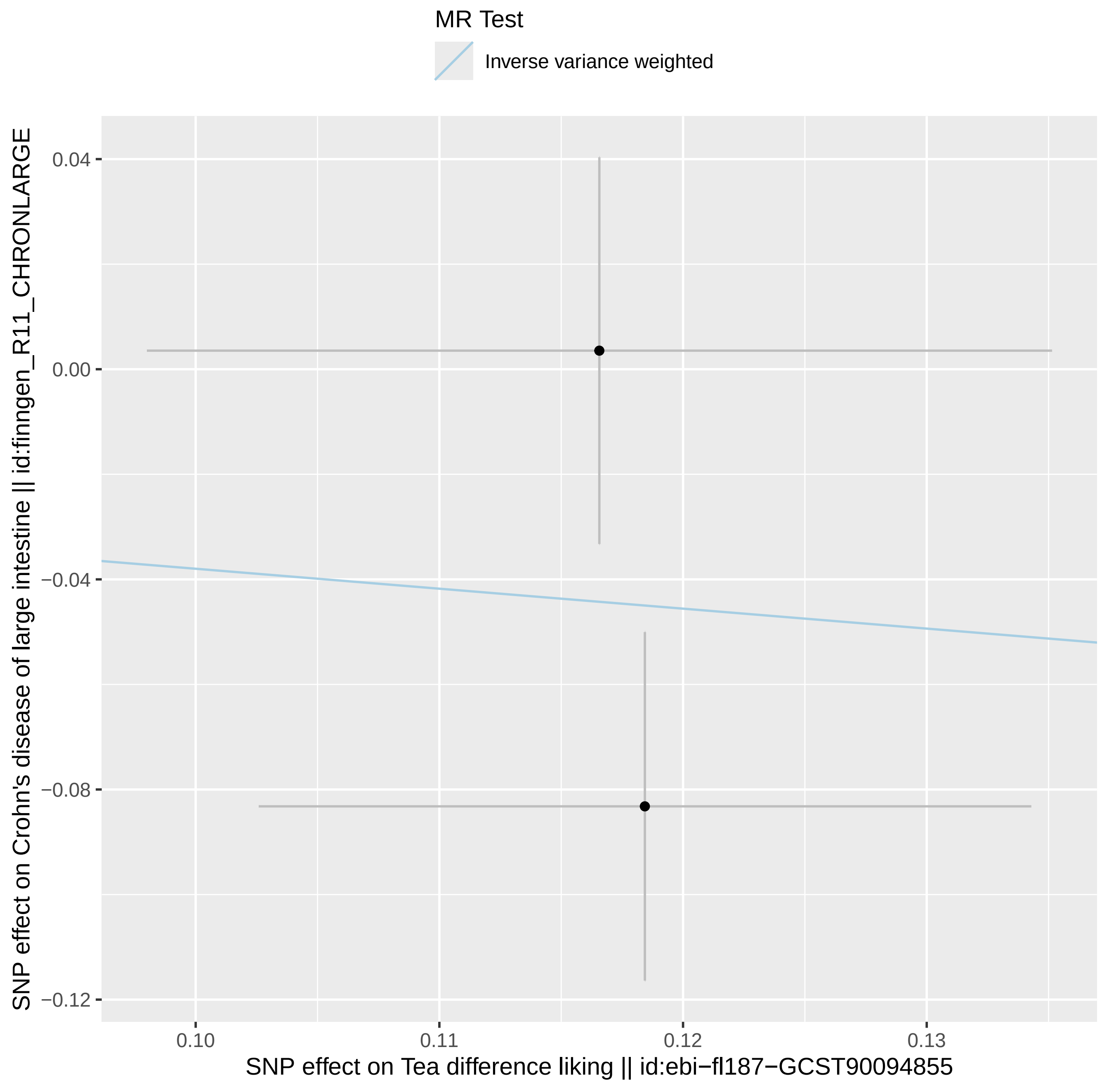

MR Test
Inverse variance weighted
0.04
0.00
−0.04
−0.08
−0.12
0.10
0.11
0.12
0.13
SNP effect on Crohn's disease of large intestine || id:finngen_R11_CHRONLARGE
SNP effect on Tea difference liking || id:ebi−fl187−GCST90094855

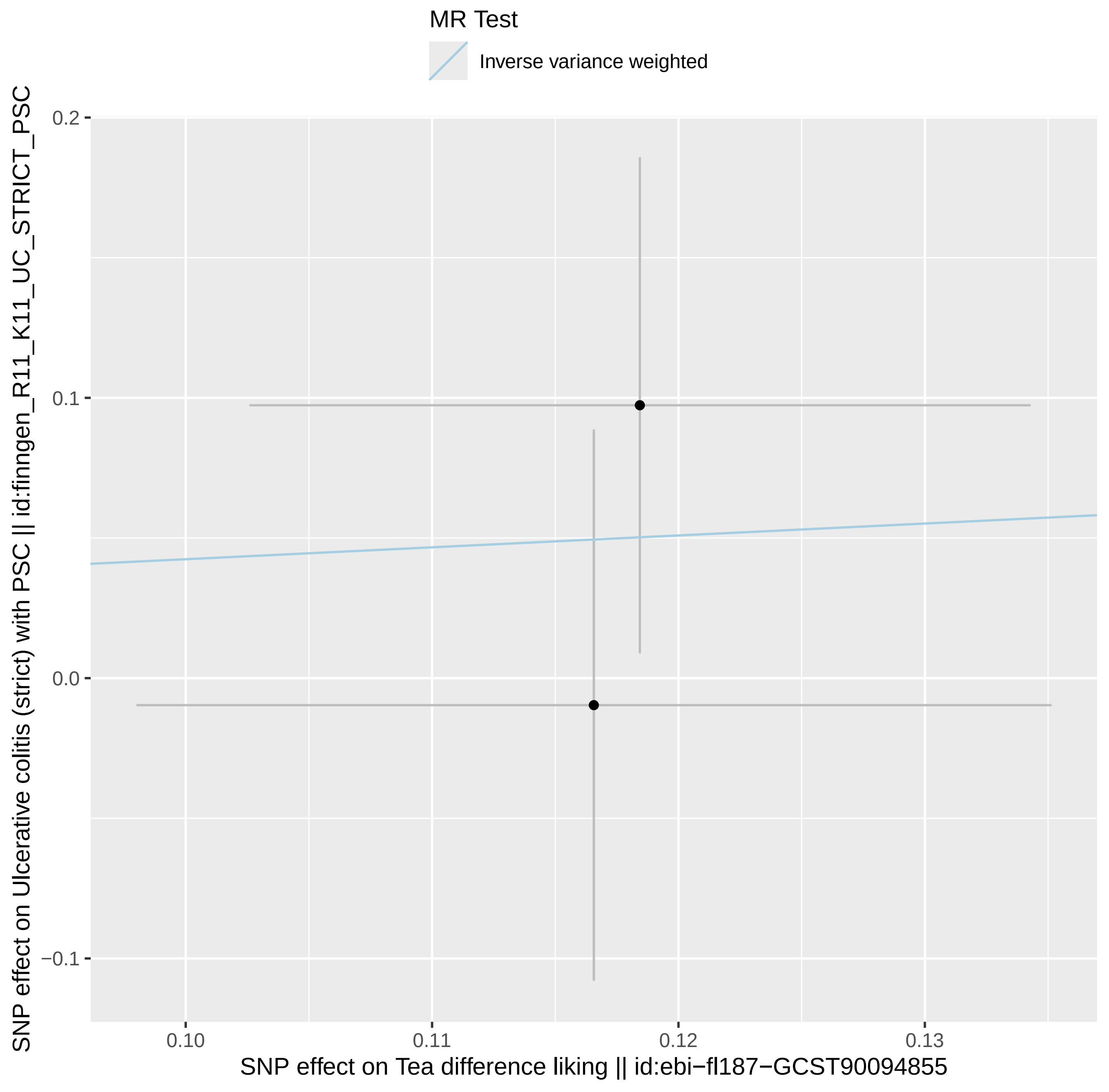
MR Test
Inverse variance weighted
0.2
0.1
0.0
−0.1
0.10
0.11
0.12
0.13
SNP effect on Ulcerative colitis (strict) with PSC || id:finngen_R11_K11_UC_STRICT_PSC
SNP effect on Tea difference liking || id:ebi−fl187−GCST90094855

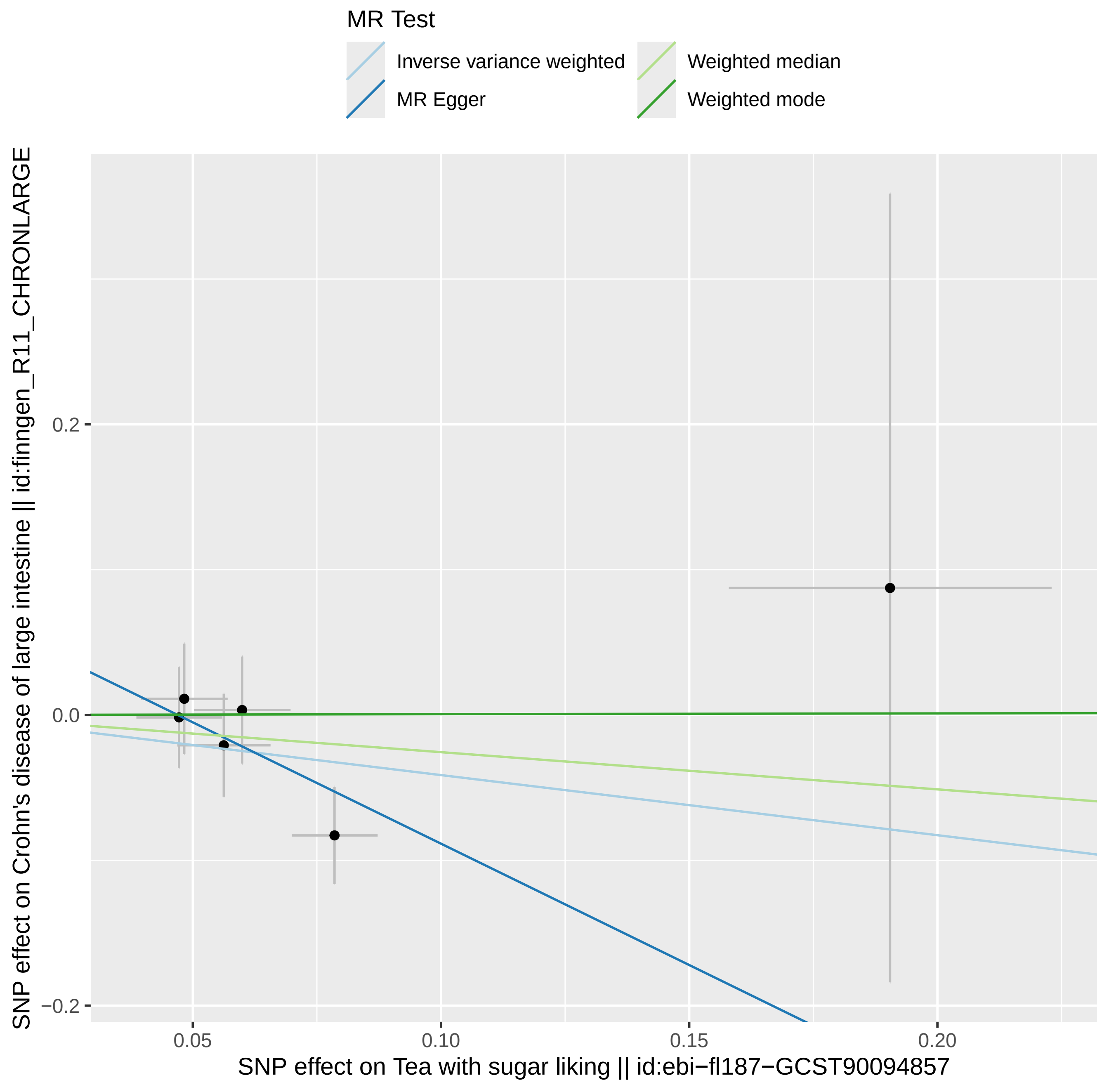
MR Test
Inverse variance weighted
MR Egger
Weighted median
Weighted mode
0.2
0.0
−0.2
0.05
0.10
0.15
0.20
SNP effect on Crohn's disease of large intestine || id:finngen_R11_CHRONLARGE
SNP effect on Tea with sugar liking || id:ebi−fl187−GCST90094857

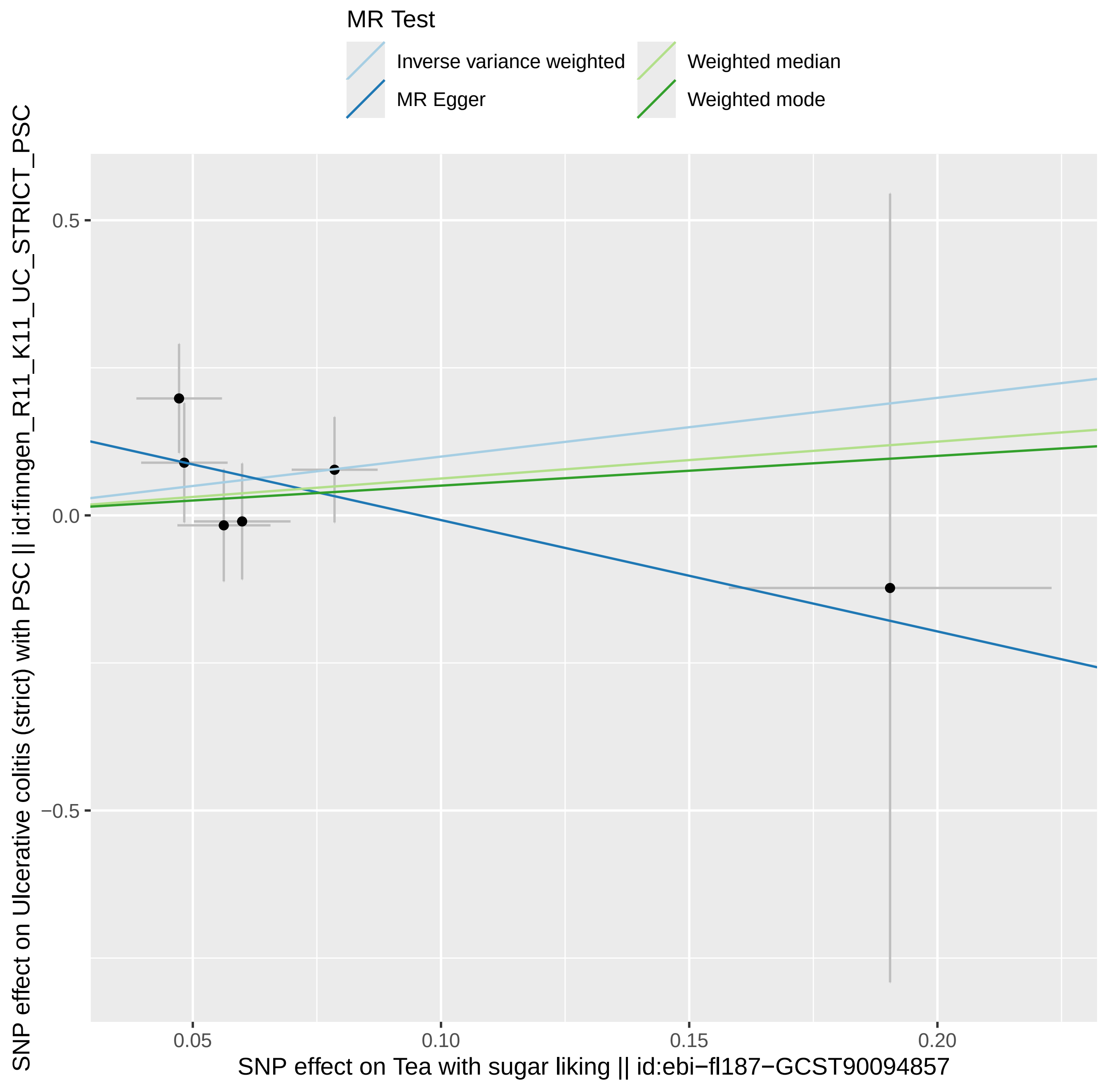
MR Test
Inverse variance weighted
MR Egger
Weighted median
Weighted mode
0.5
0.0
−0.5
0.05
0.10
0.15
0.20
SNP effect on Tea with sugar liking || id:ebi−fl187−GCST90094857
SNP effect on Ulcerative colitis (strict) with PSC || id:finngen_R11_K11_UC_STRICT_PSC

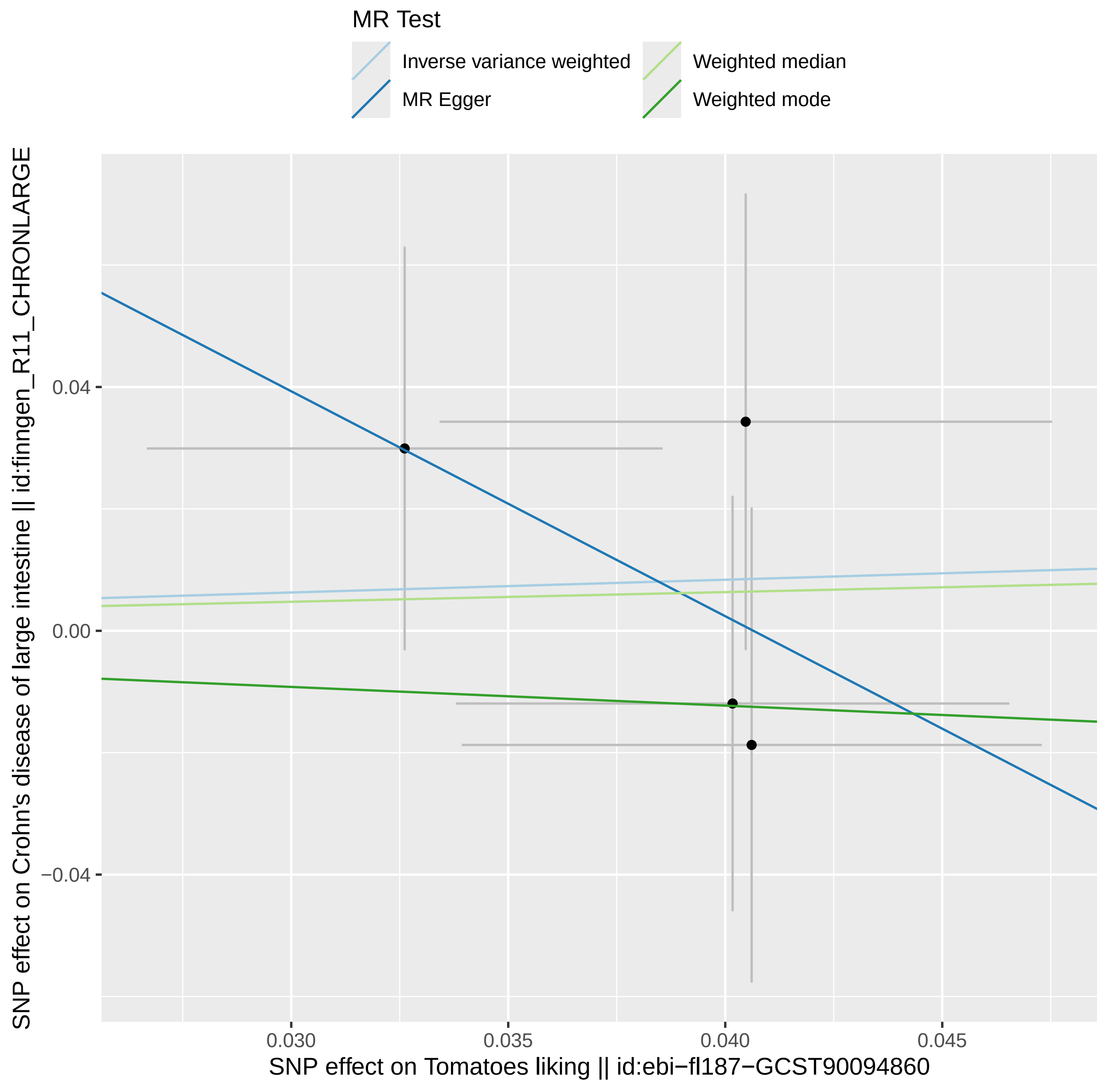
MR Test
Inverse variance weighted
MR Egger
Weighted median
Weighted mode
SNP effect on Crohn's disease of large intestine || id:finngen_R11_CHRONLARGE
0.04
0.00
−0.04
0.030
0.035
0.040
0.045
SNP effect on Tomatoes liking || id:ebi−fl187−GCST90094860

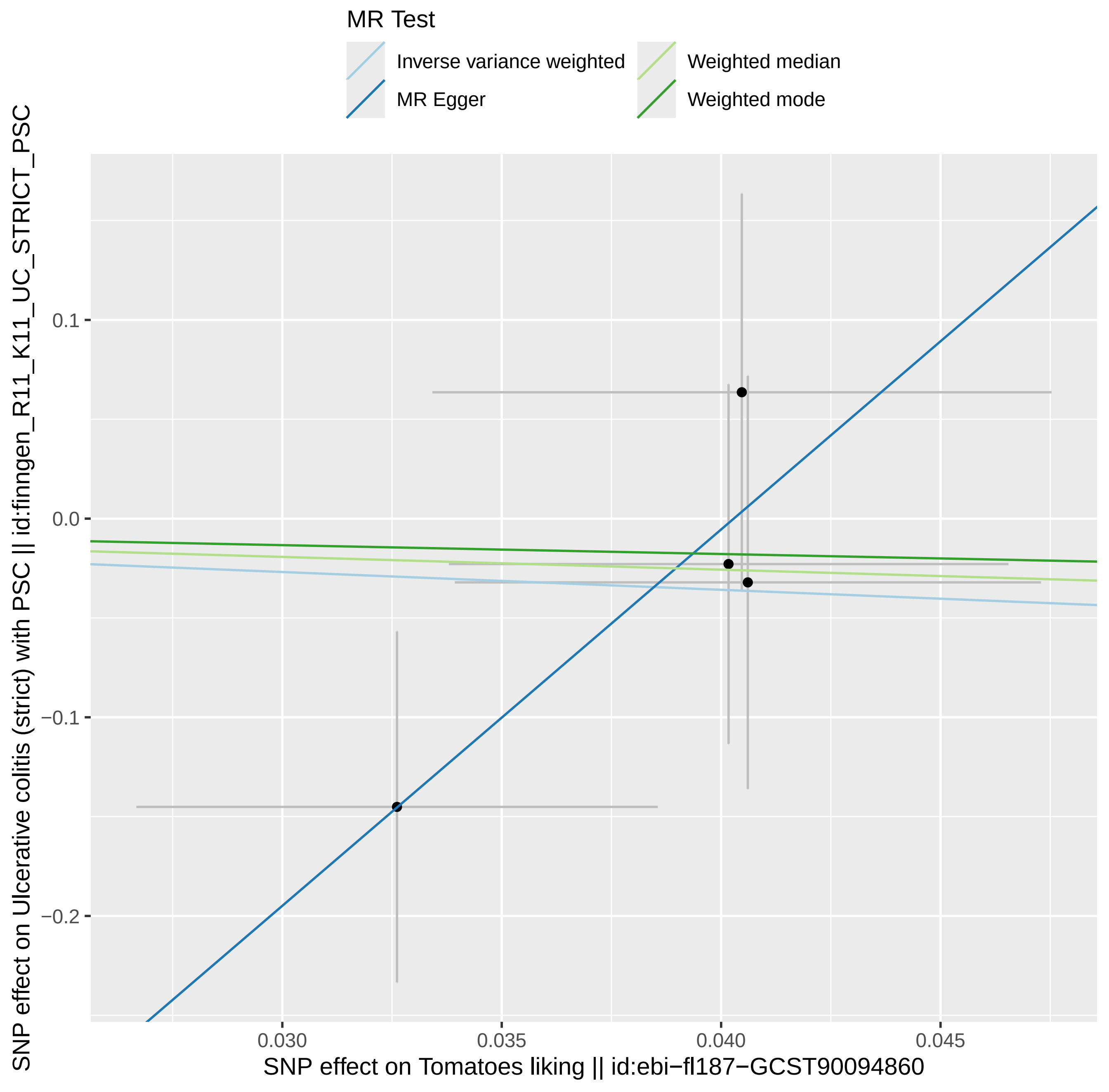
MR Test
Inverse variance weighted
MR Egger
Weighted median
Weighted mode
0.1
0.0
−0.1
−0.2
0.030
0.035
0.040
0.045
SNP effect on Tomatoes liking || id:ebi−fl187−GCST90094860
SNP effect on Ulcerative colitis (strict) with PSC || id:finngen_R11_K11_UC_STRICT_PSC

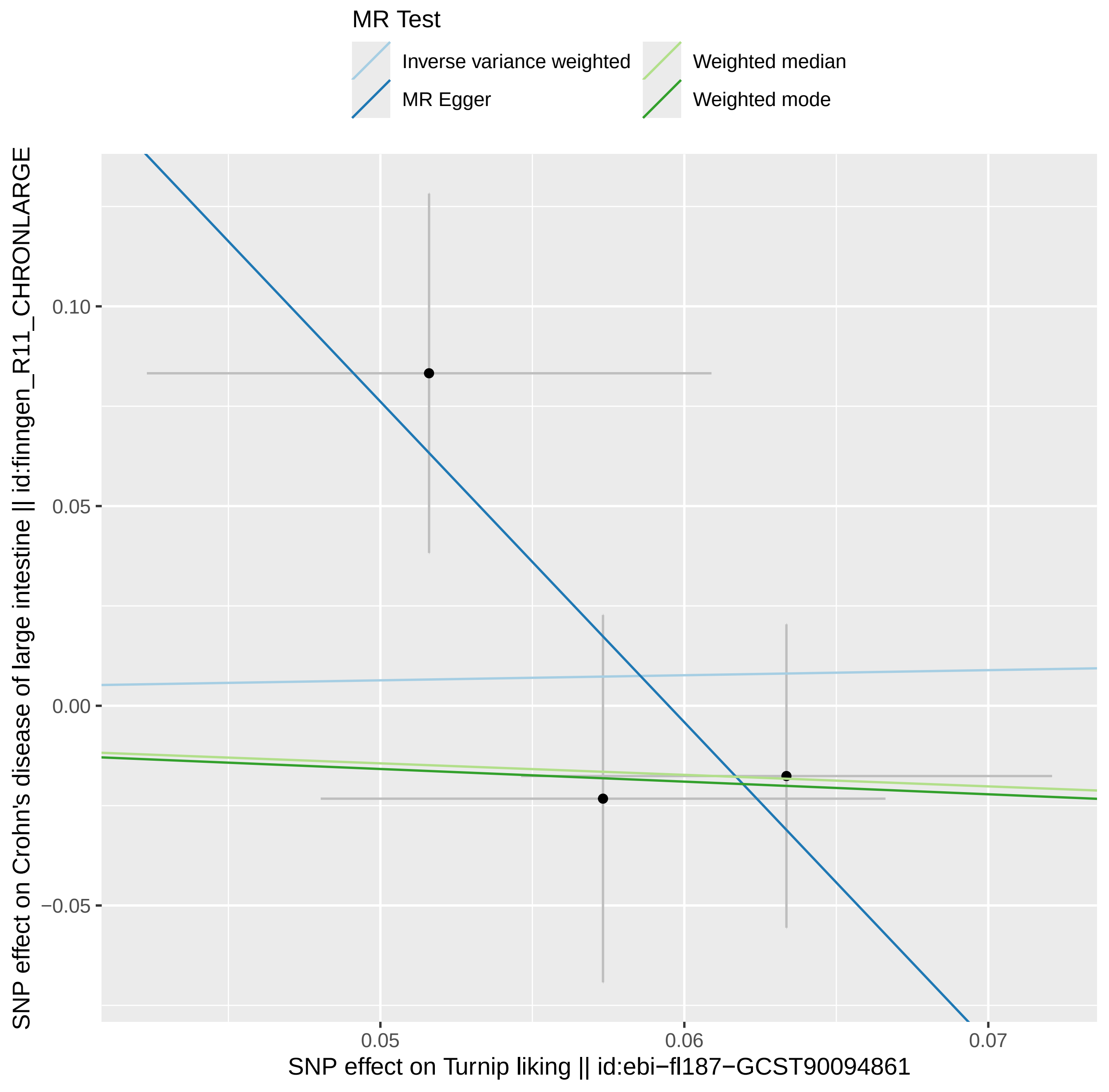
MR Test
Inverse variance weighted
MR Egger
Weighted median
Weighted mode
SNP effect on Crohn's disease of large intestine || id:finngen_R11_CHRONLARGE
0.10
0.05
0.00
−0.05
0.05
0.06
0.07
SNP effect on Turnip liking || id:ebi−fl187−GCST90094861

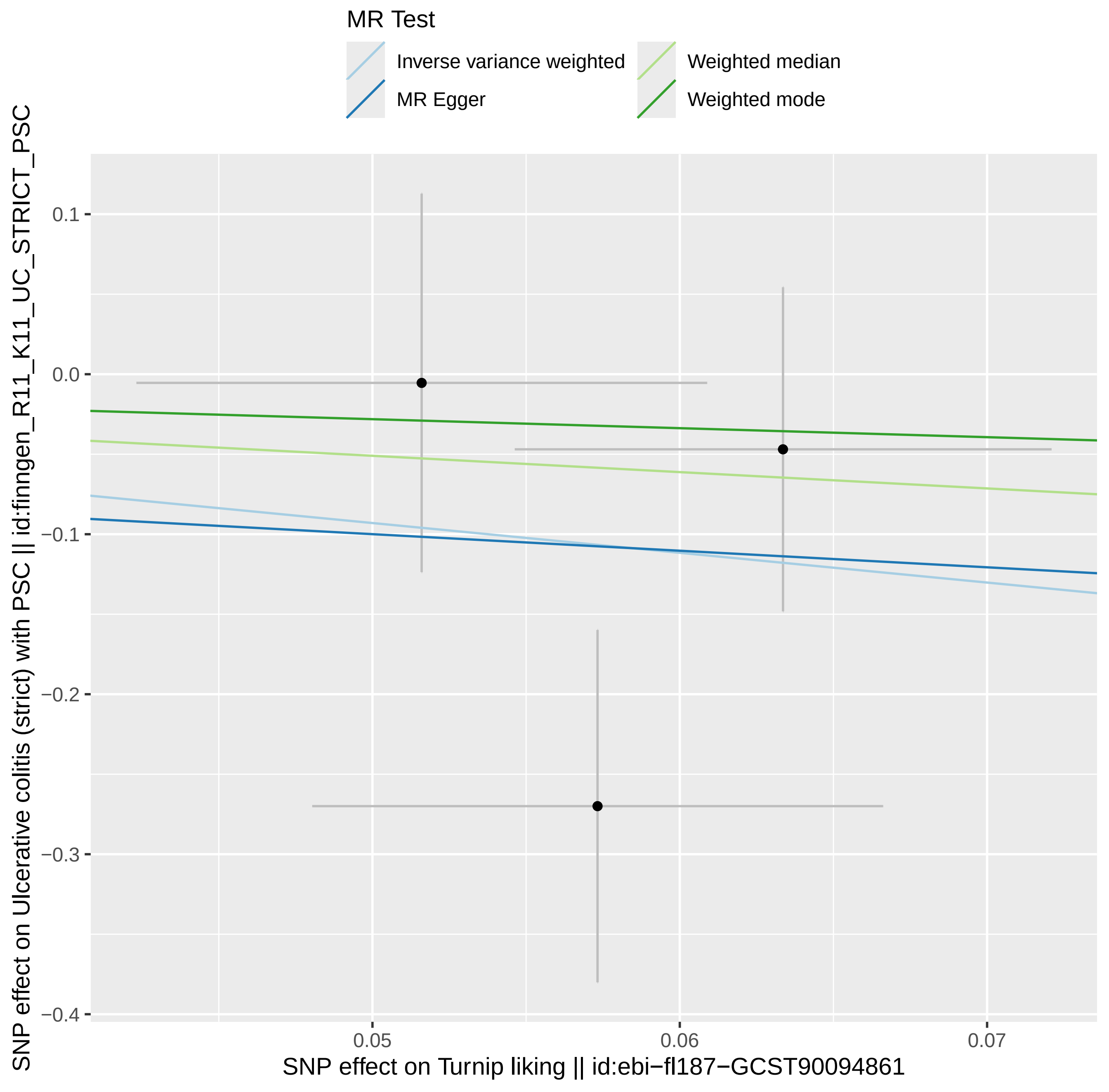
MR Test
Inverse variance weighted
MR Egger
Weighted median
Weighted mode
0.1
0.0
−0.1
−0.2
−0.3
−0.4
0.05
0.06
0.07
SNP effect on Ulcerative colitis (strict) with PSC || id:finngen_R11_K11_UC_STRICT_PSC
SNP effect on Turnip liking || id:ebi−fl187−GCST90094861

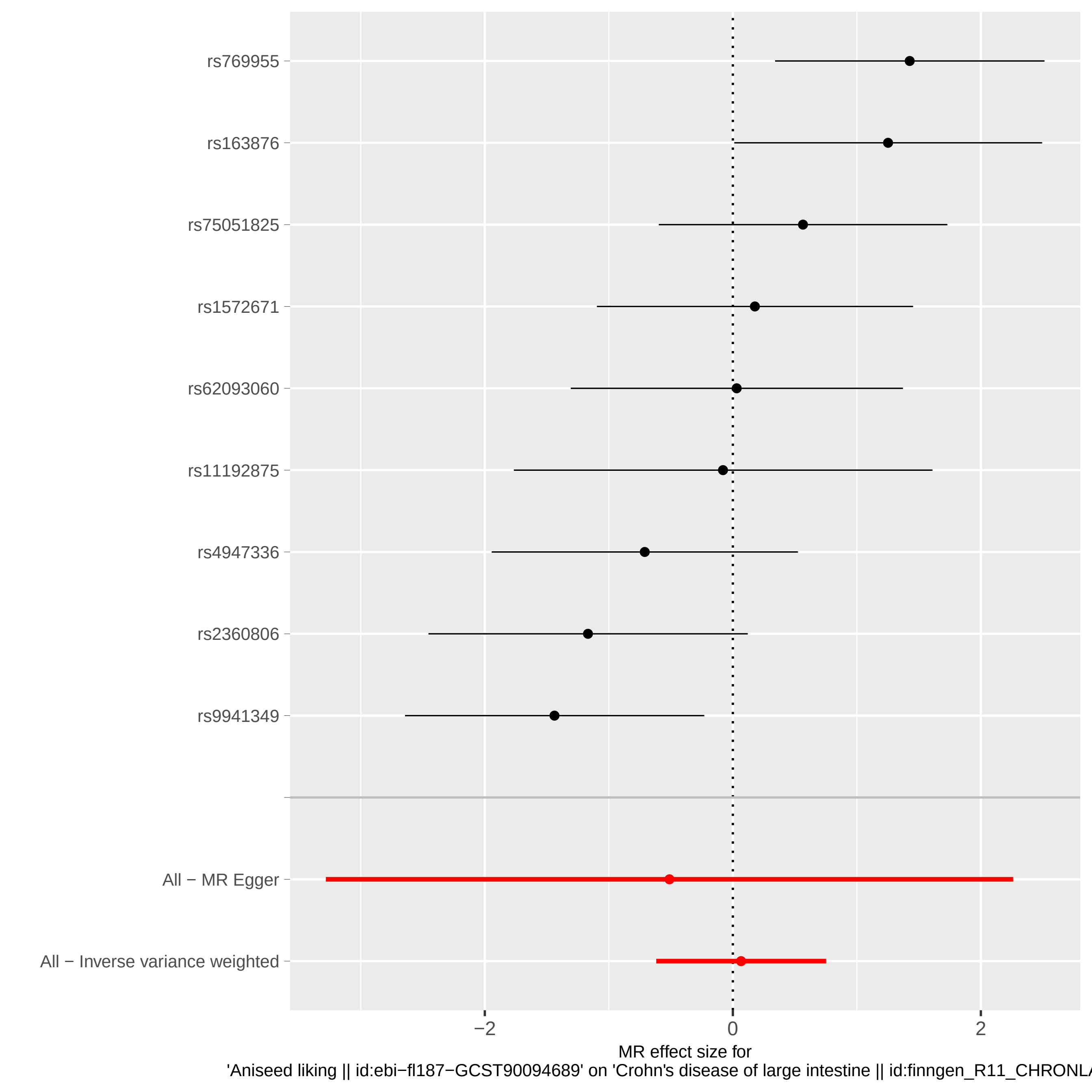
rs769955
rs163876
rs75051825
rs1572671
rs62093060
rs11192875
rs4947336
rs2360806
rs9941349
All − MR Egger
All − Inverse variance weighted
−2
0
2
MR effect size for
'Aniseed liking || id:ebi−fl187−GCST90094689' on 'Crohn's disease of large intestine || id:finngen_R11_CHRONL

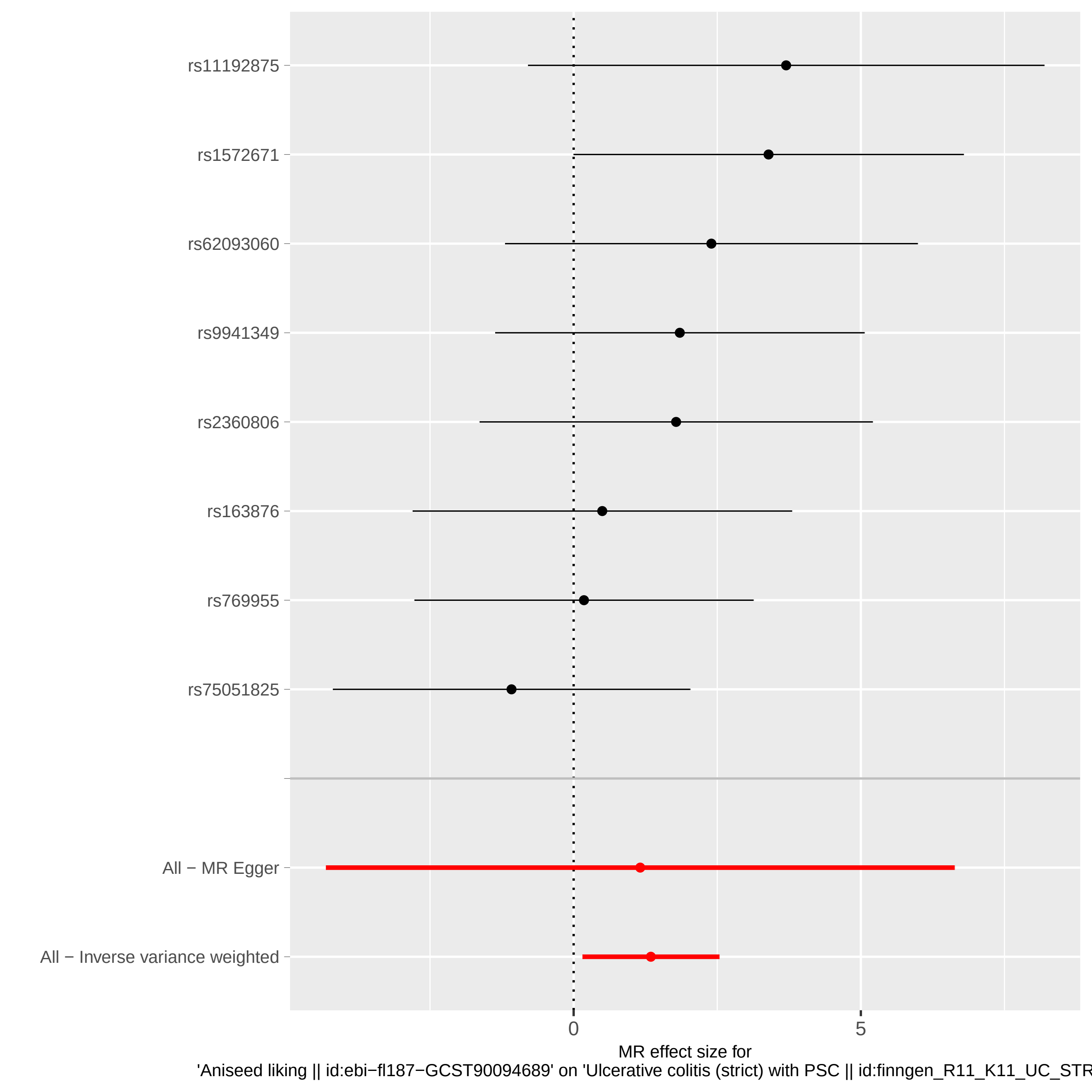
rs11192875
rs1572671
rs62093060
rs9941349
rs2360806
rs163876
rs769955
rs75051825
All − MR Egger
All − Inverse variance weighted
0
5
MR effect size for
'Aniseed liking || id:ebi−fl187−GCST90094689' on 'Ulcerative colitis (strict) with PSC || id:finngen_R11_K11_UC_STR

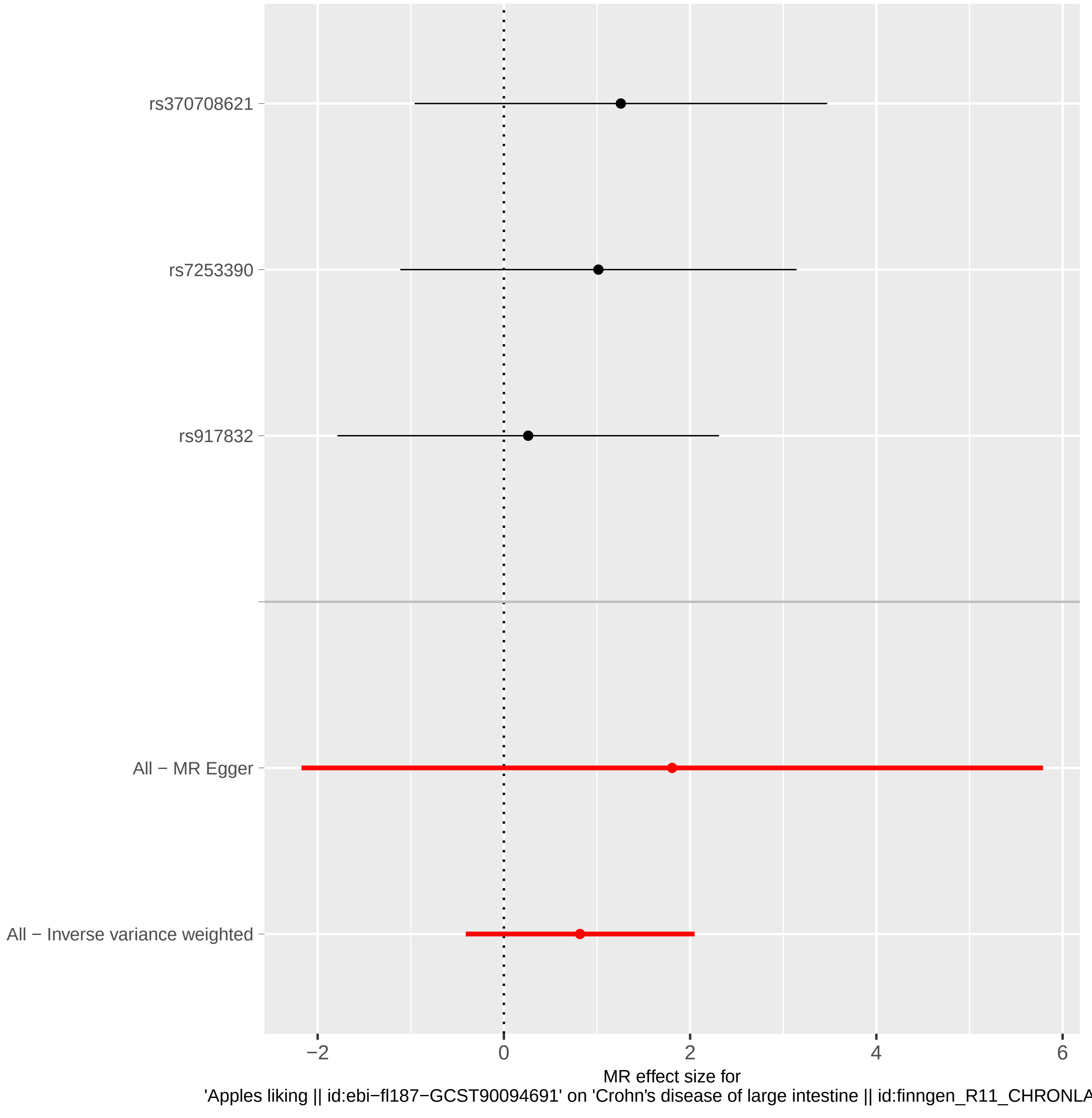
rs370708621
rs7253390
rs917832
All − MR Egger
All − Inverse variance weighted
−2
0
2
4
6
MR effect size for
'Apples liking || id:ebi−fl187−GCST90094691' on 'Crohn's disease of large intestine || id:finngen_R11_CHRONLA

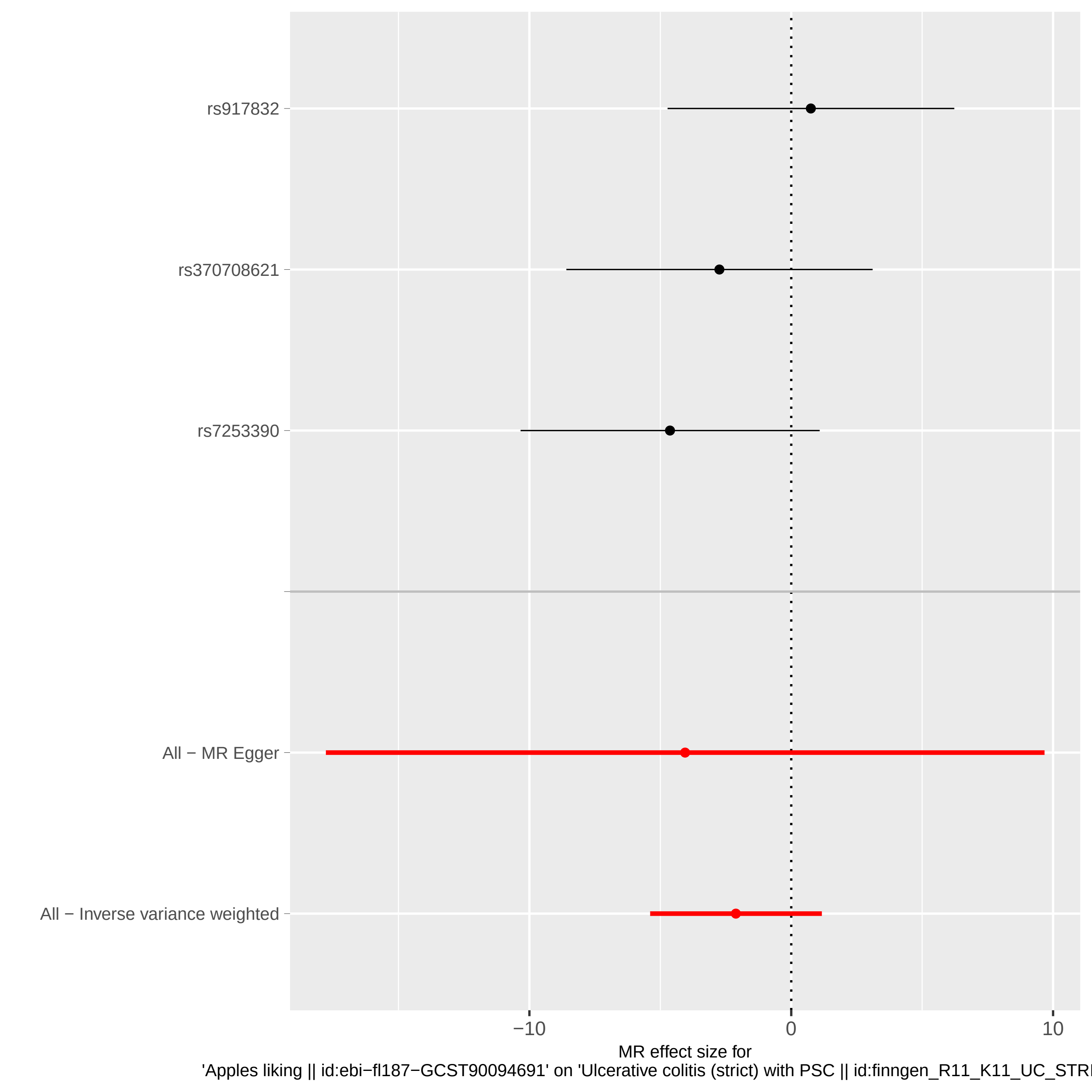

rs917832
rs370708621
rs7253390
All − MR Egger
All − Inverse variance weighted
−10
0
10
MR effect size for
'Apples liking || id:ebi−fl187−GCST90094691' on 'Ulcerative colitis (strict) with PSC || id:finngen_R11_K11_UC_STR

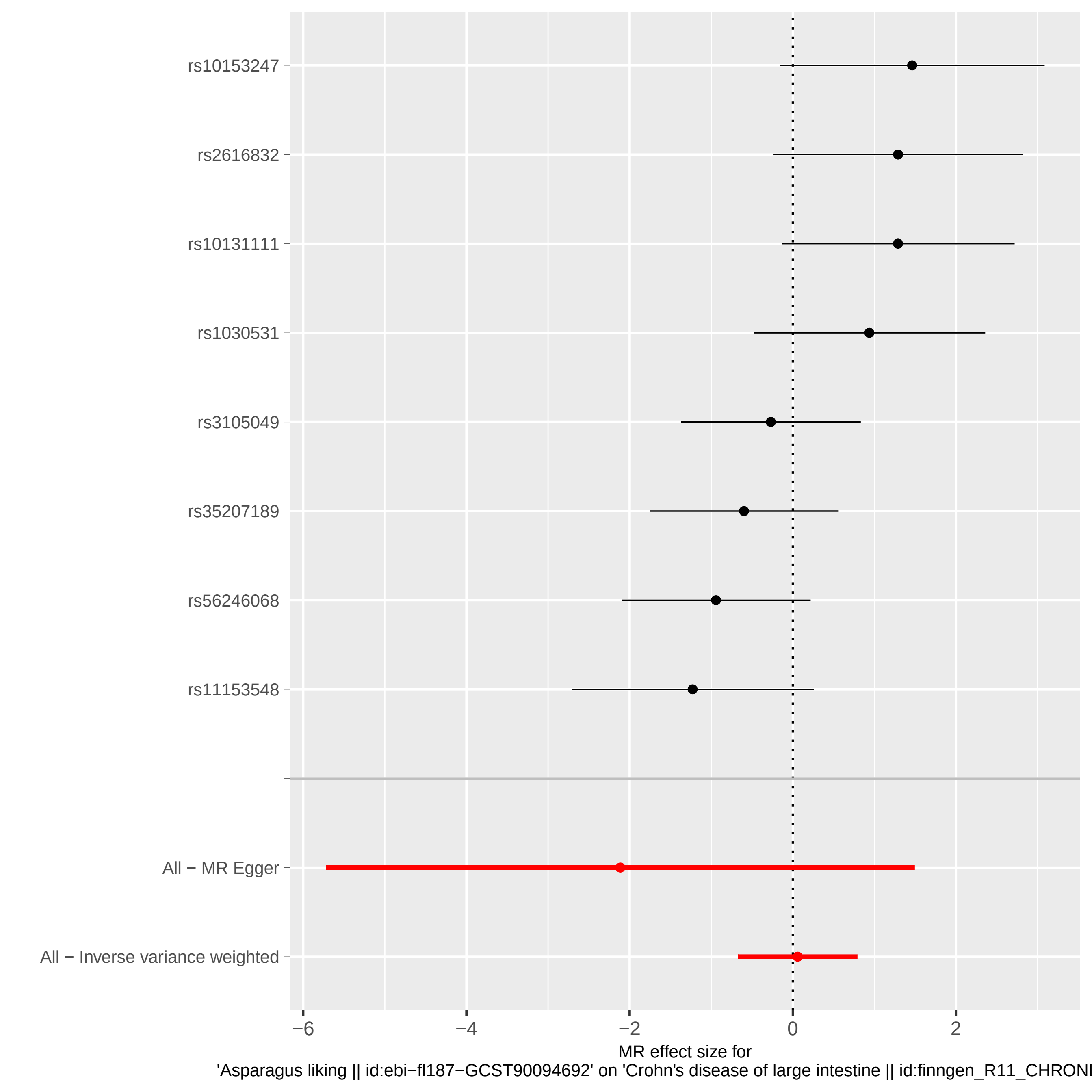

rs10153247
rs2616832
rs10131111
rs1030531
rs3105049
rs35207189
rs56246068
rs11153548
All − MR Egger
All − Inverse variance weighted
−6
−4
−2
0
2
MR effect size for
'Asparagus liking || id:ebi−fl187−GCST90094692' on 'Crohn's disease of large intestine || id:finngen_R11_CHRONI

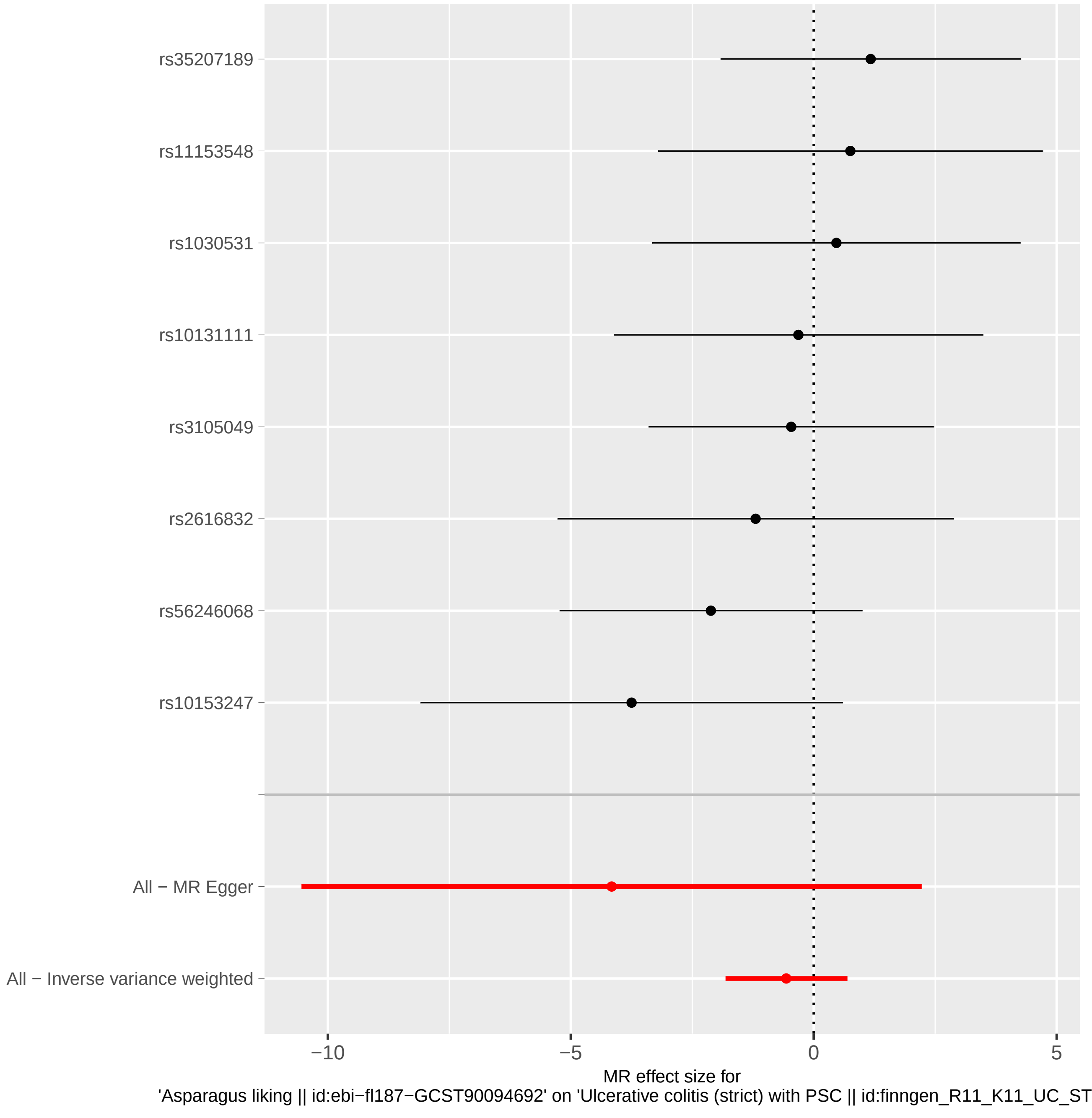
rs35207189
rs11153548
rs1030531
rs10131111
rs3105049
rs2616832
rs56246068
rs10153247
All − MR Egger
All − Inverse variance weighted
−10
−5
0
5
MR effect size for
'Asparagus liking || id:ebi−fl187−GCST90094692' on 'Ulcerative colitis (strict) with PSC || id:finngen_R11_K11_UC_ST

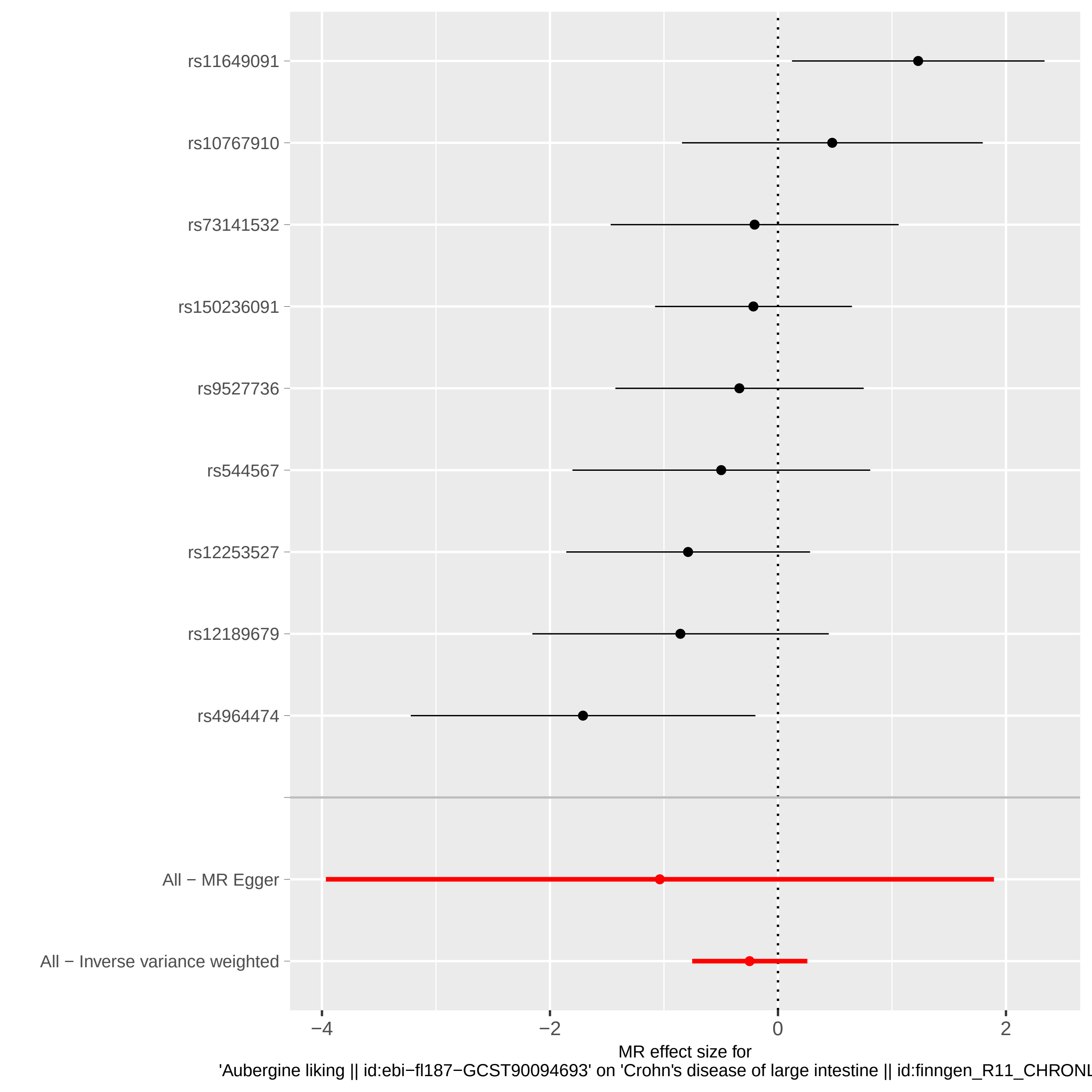

rs11649091
rs10767910
rs73141532
rs150236091
rs9527736
rs544567
rs12253527
rs12189679
rs4964474
All − MR Egger
All − Inverse variance weighted
−4
−2
0
2
MR effect size for
'Aubergine liking || id:ebi−fl187−GCST90094693' on 'Crohn's disease of large intestine || id:finngen_R11_CHRONL

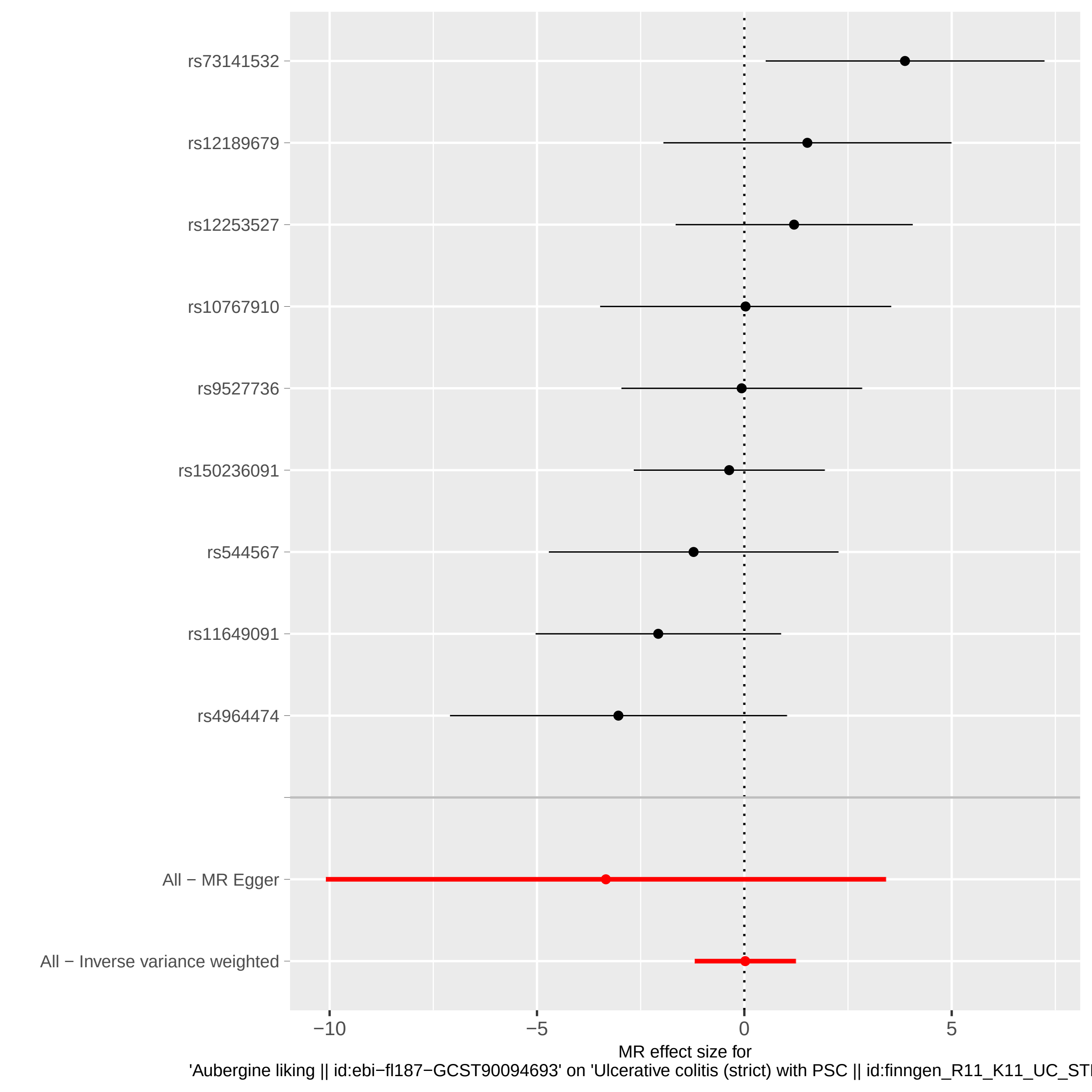
rs73141532
rs12189679
rs12253527
rs10767910
rs9527736
rs150236091
rs544567
rs11649091
rs4964474
All − MR Egger
All − Inverse variance weighted
−10
−5
0
5
MR effect size for
'Aubergine liking || id:ebi−fl187−GCST90094693' on 'Ulcerative colitis (strict) with PSC || id:finngen_R11_K11_UC_ST

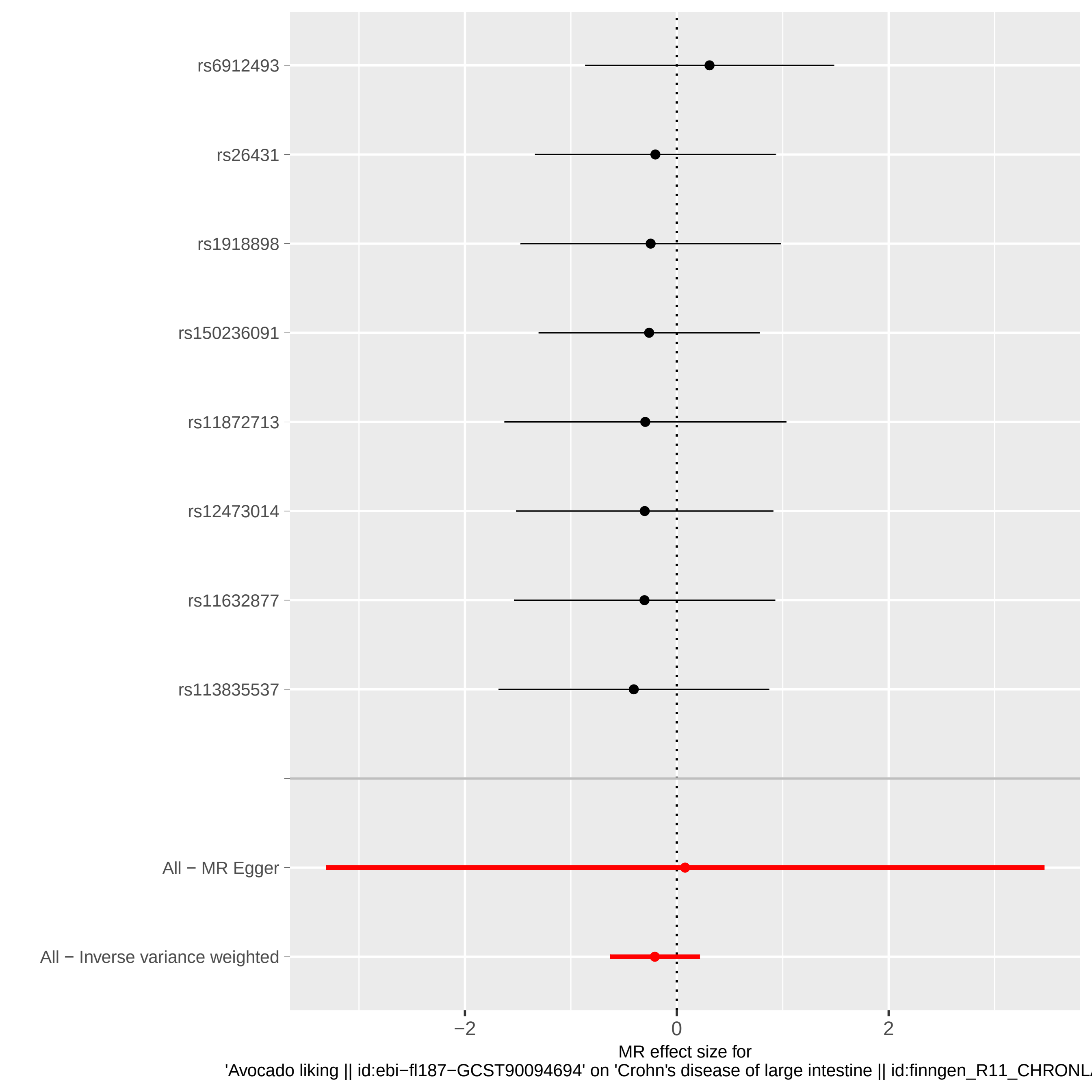
rs6912493
rs26431
rs1918898
rs150236091
rs11872713
rs12473014
rs11632877
rs113835537
All − MR Egger
All − Inverse variance weighted
−2
0
2
MR effect size for
'Avocado liking || id:ebi−fl187−GCST90094694' on 'Crohn's disease of large intestine || id:finngen_R11_CHRONL

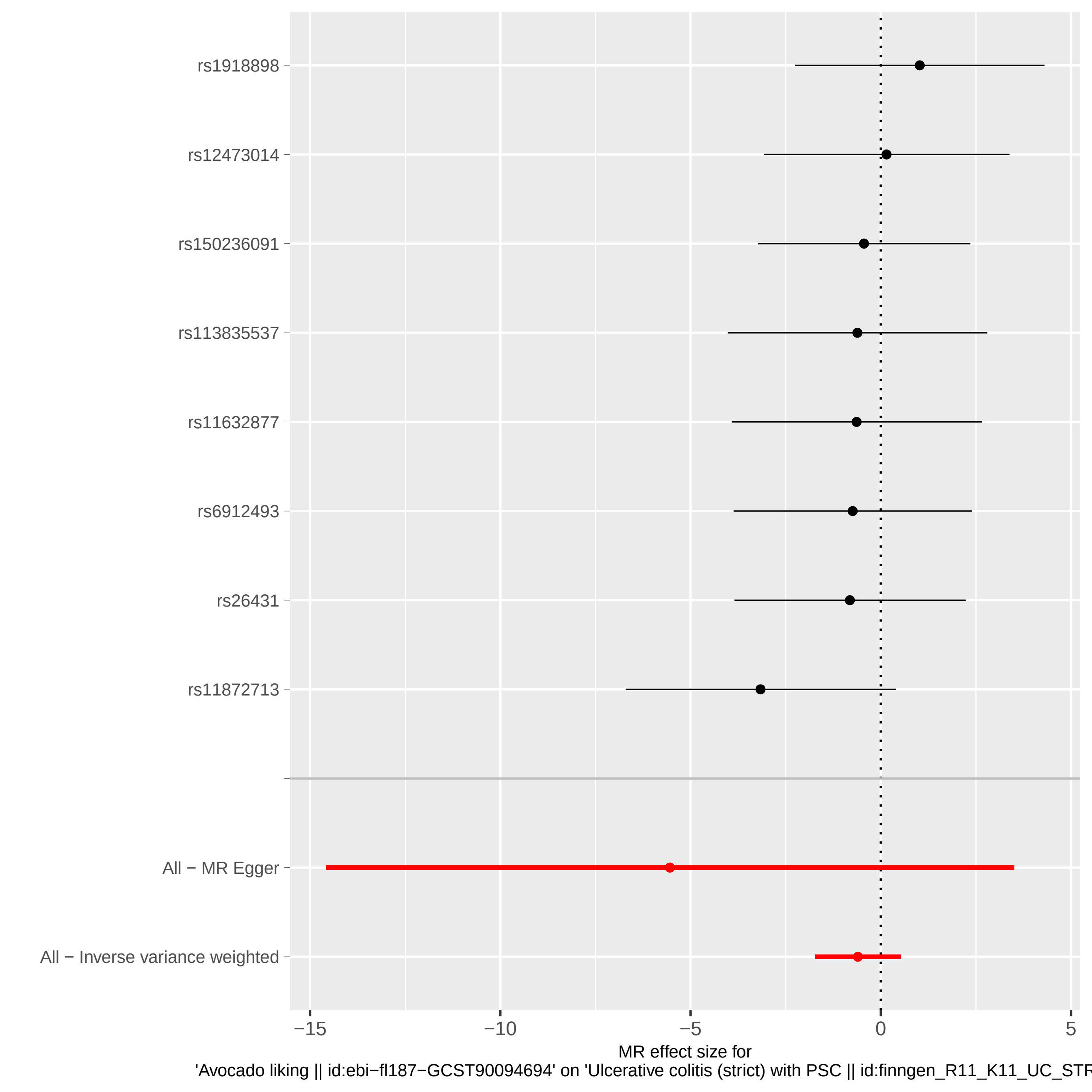
rs1918898
rs12473014
rs150236091
rs113835537
rs11632877
rs6912493
rs26431
rs11872713
All − MR Egger
All − Inverse variance weighted
−15
−10
−5
0
5
MR effect size for
'Avocado liking || id:ebi−fl187−GCST90094694' on 'Ulcerative colitis (strict) with PSC || id:finngen_R11_K11_UC_STR

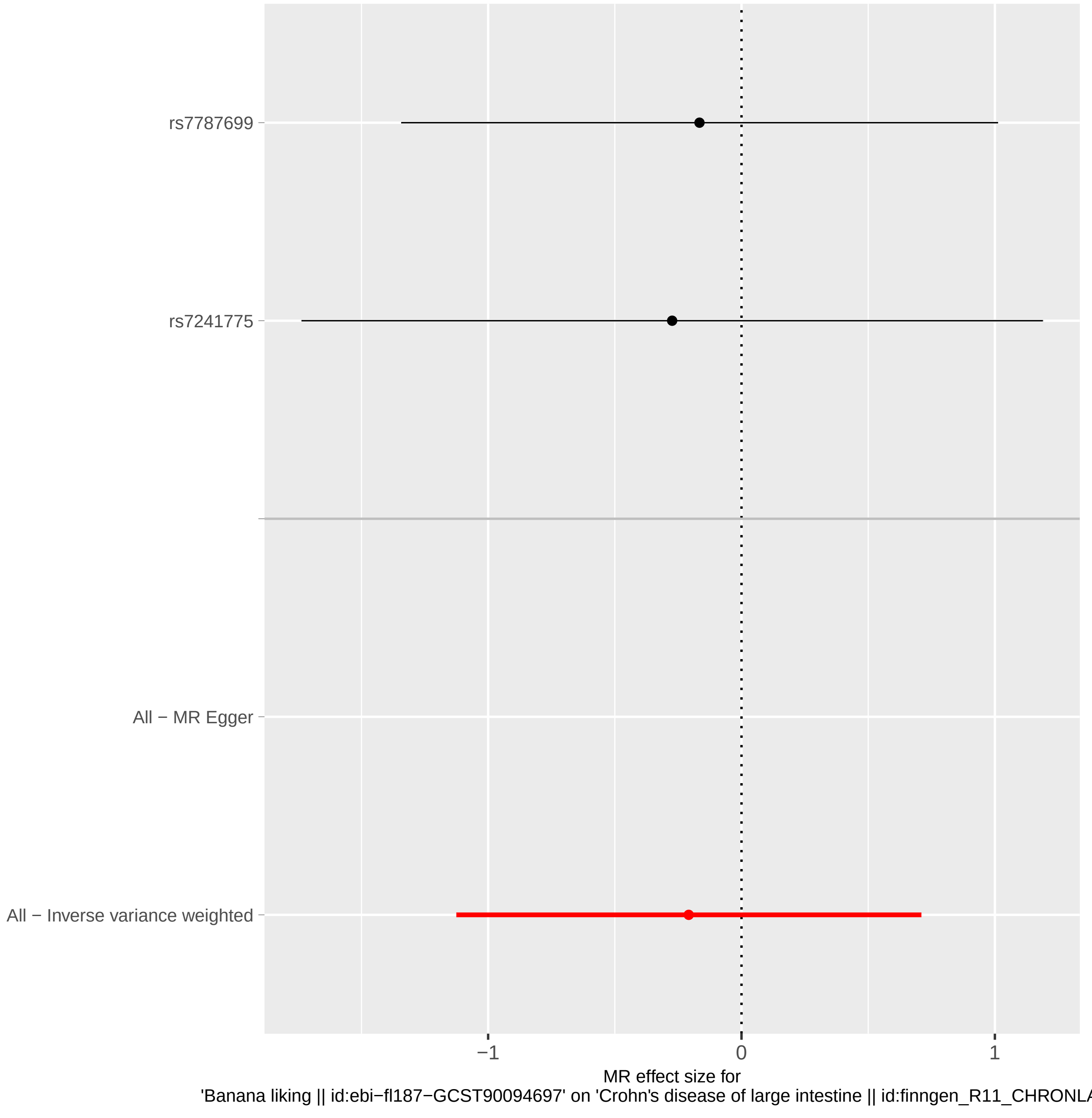

rs7787699
rs7241775
All − MR Egger
All − Inverse variance weighted
−1
0
1
MR effect size for
'Banana liking || id:ebi−fl187−GCST90094697' on 'Crohn's disease of large intestine || id:finngen_R11_CHRONLA

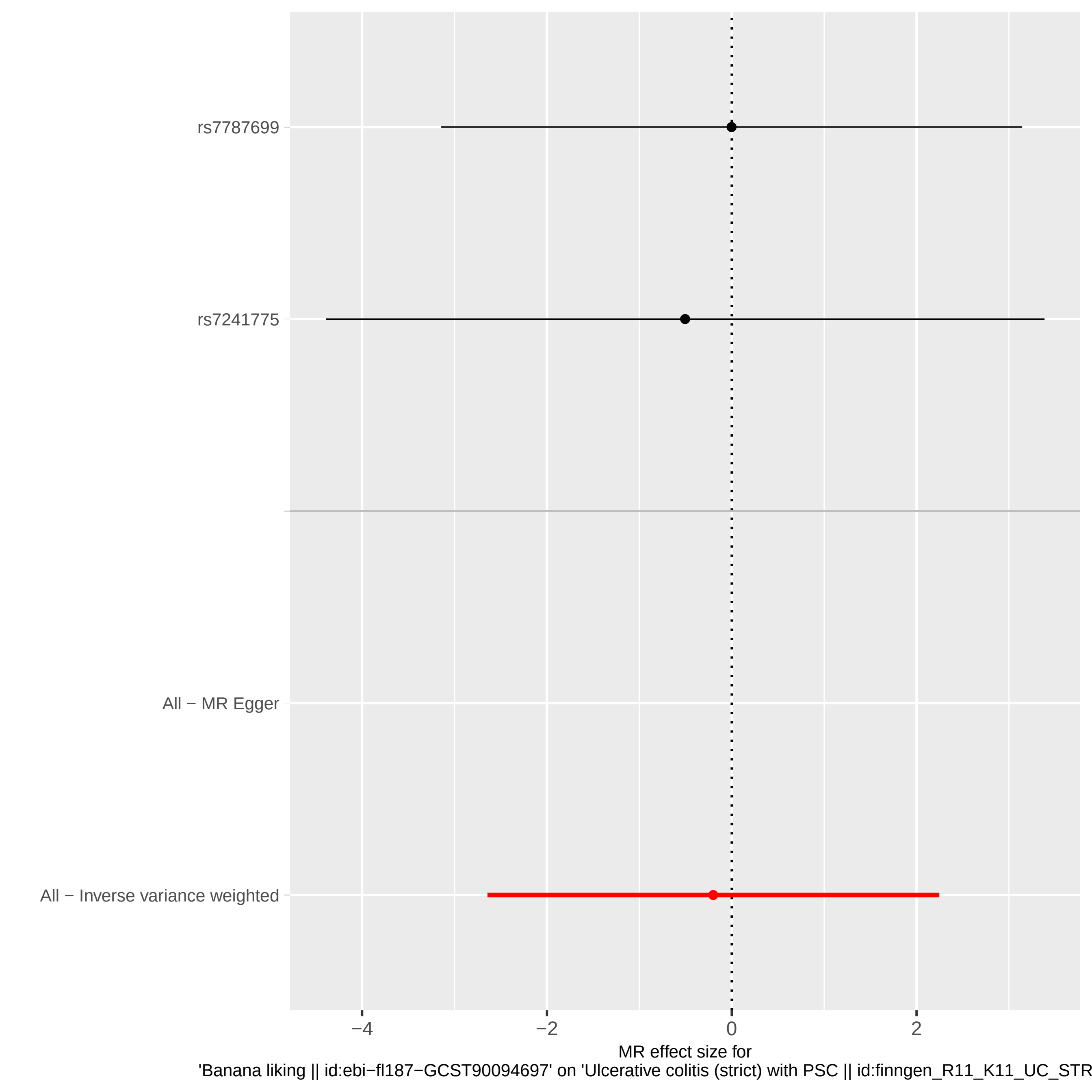
rs7787699
rs7241775
All − MR Egger
All − Inverse variance weighted
−4
−2
0
2
MR effect size for
'Banana liking || id:ebi−fl187−GCST90094697' on 'Ulcerative colitis (strict) with PSC || id:finngen_R11_K11_UC_STR

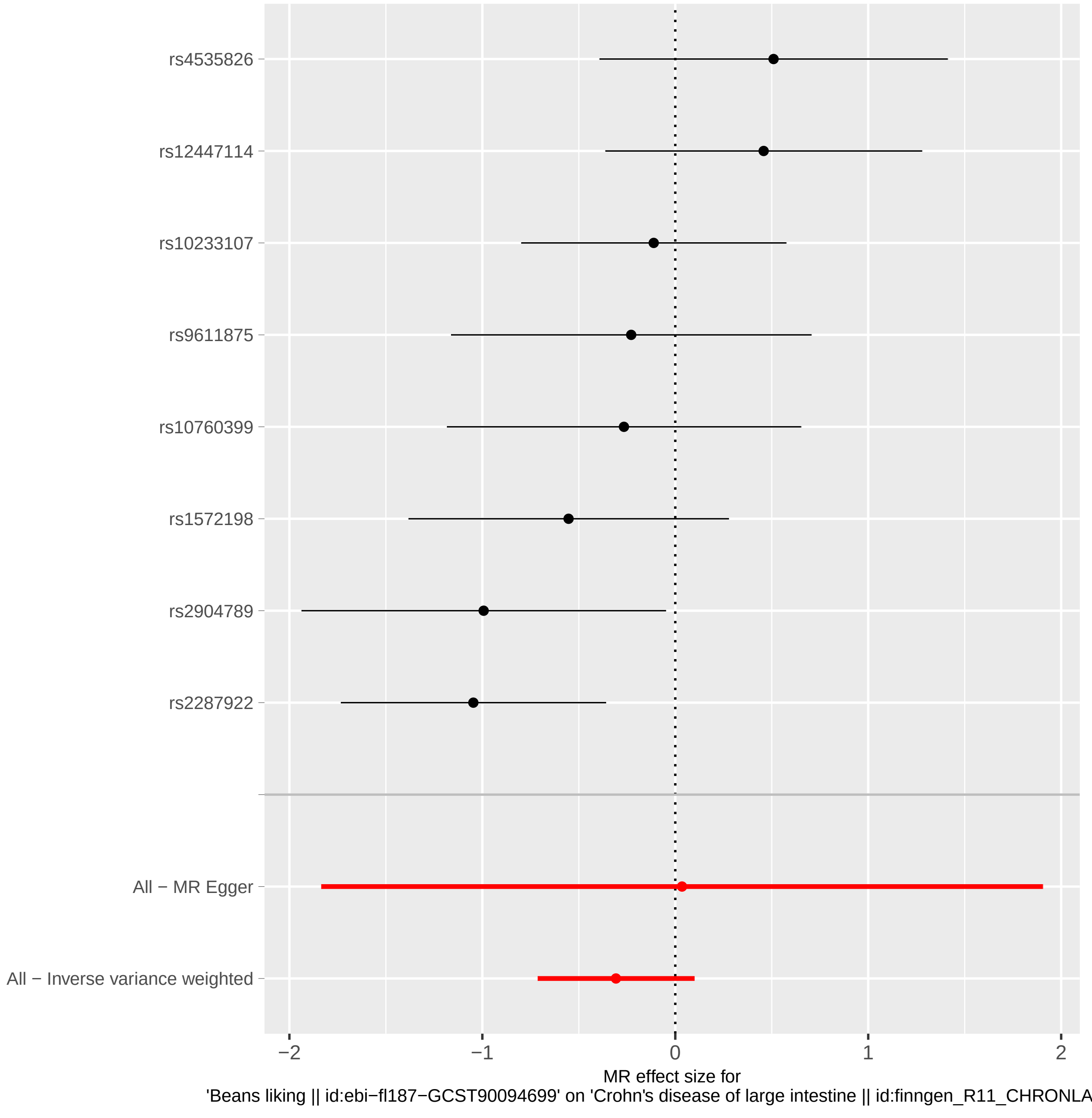
rs4535826
rs12447114
rs10233107
rs9611875
rs10760399
rs1572198
rs2904789
rs2287922
All − MR Egger
All − Inverse variance weighted
−2
−1
0
1
2
MR effect size for
'Beans liking || id:ebi−fl187−GCST90094699' on 'Crohn's disease of large intestine || id:finngen_R11_CHRONLA

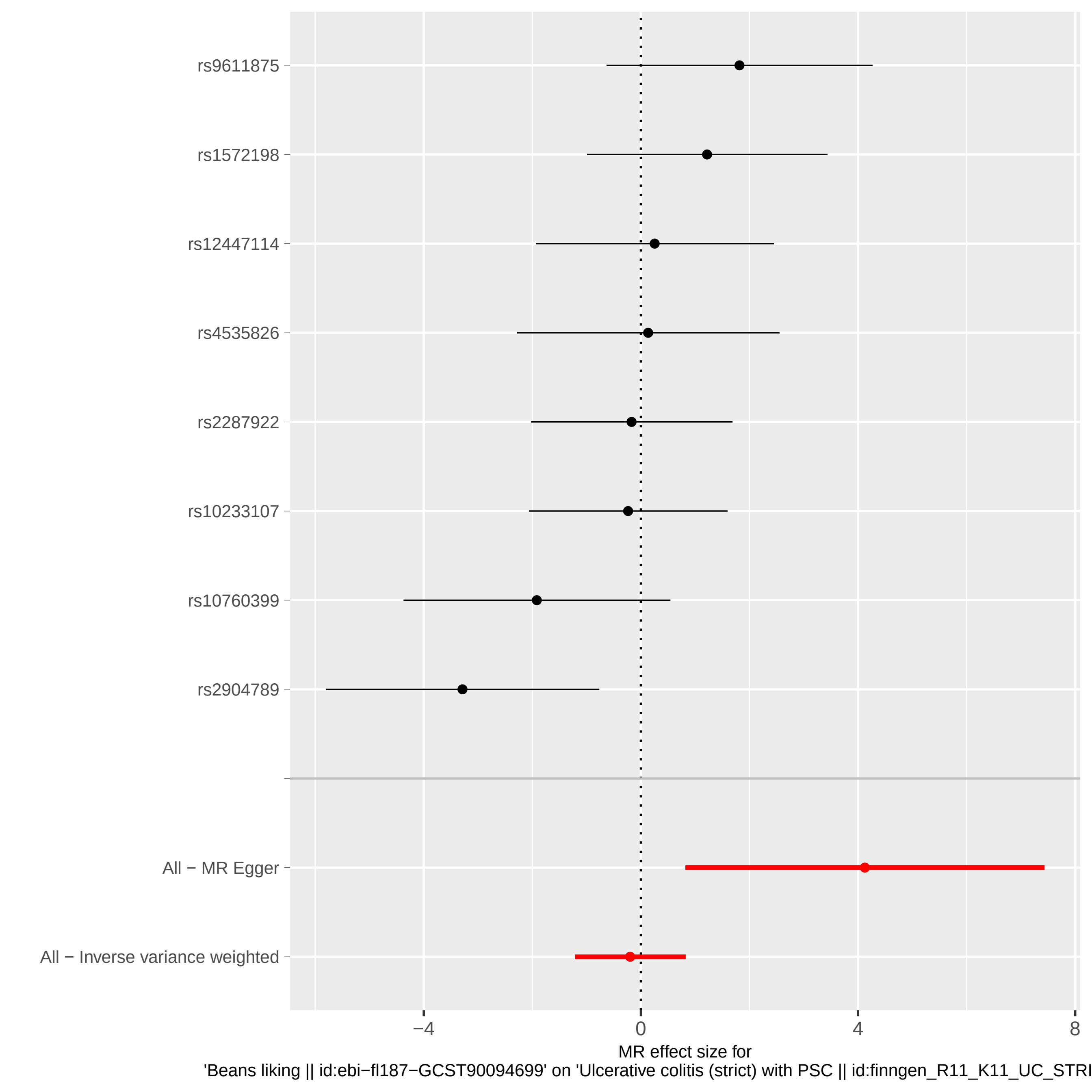
rs9611875
rs1572198
rs12447114
rs4535826
rs2287922
rs10233107
rs10760399
rs2904789
All − MR Egger
All − Inverse variance weighted
−4
0
4
8
MR effect size for
'Beans liking || id:ebi−fl187−GCST90094699' on 'Ulcerative colitis (strict) with PSC || id:finngen_R11_K11_UC_STRI

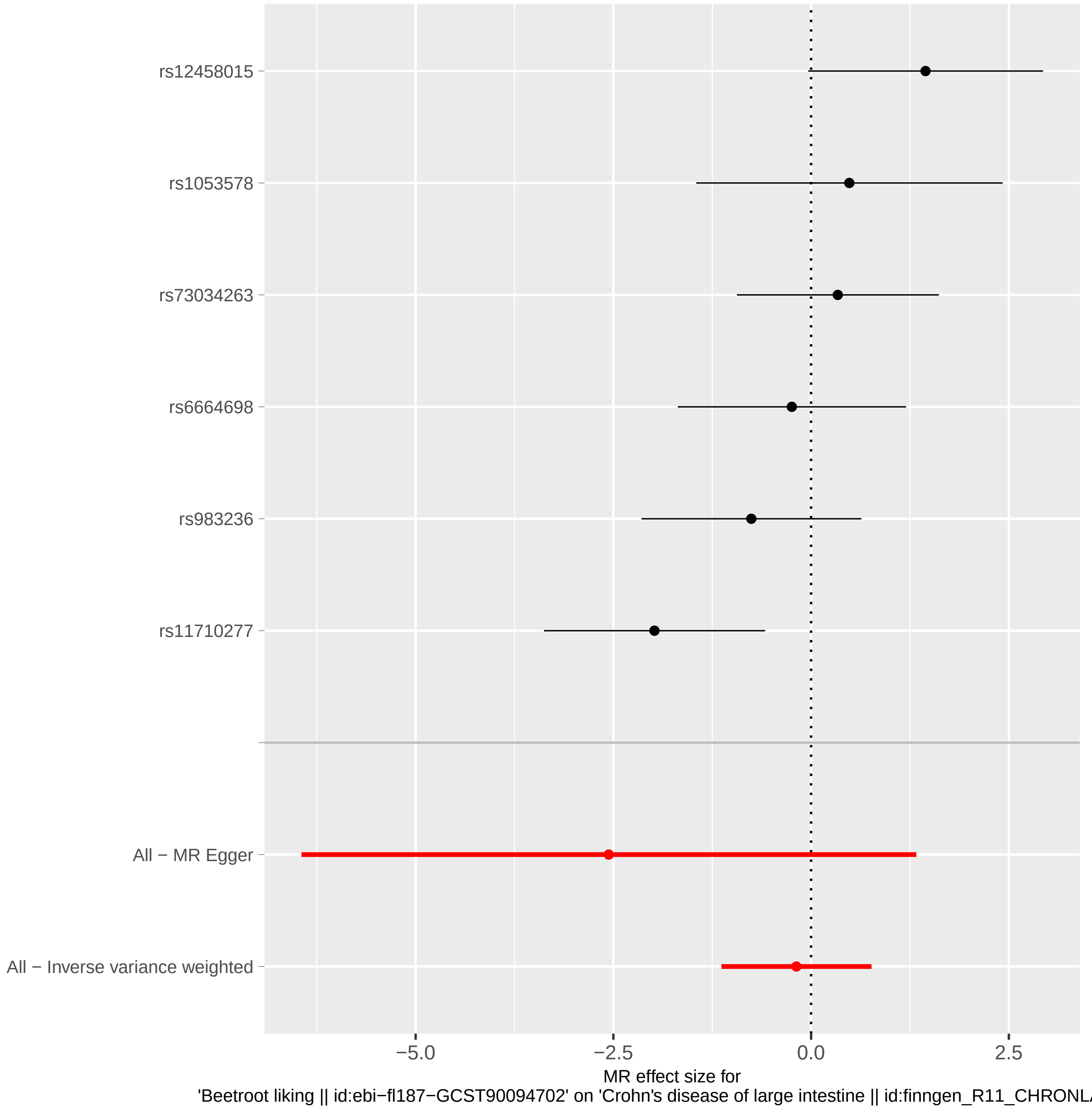
rs12458015
rs1053578
rs73034263
rs6664698
rs983236
rs11710277
All − MR Egger
All − Inverse variance weighted
−5.0
−2.5
0.0
2.5
MR effect size for
'Beetroot liking || id:ebi−fl187−GCST90094702' on 'Crohn's disease of large intestine || id:finngen_R11_CHRONL

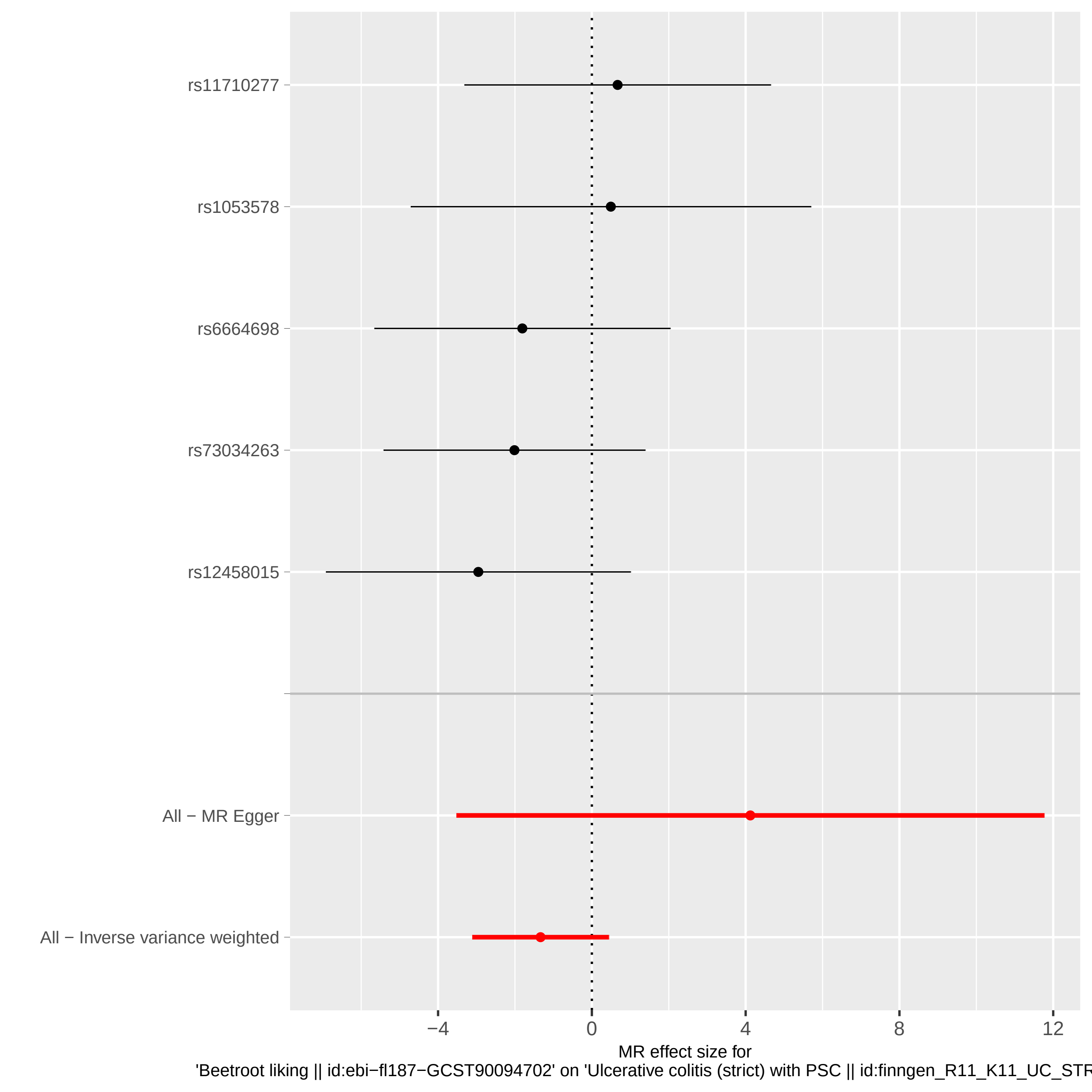
rs11710277
rs1053578
rs6664698
rs73034263
rs12458015
All − MR Egger
All − Inverse variance weighted
−4
0
4
8
12
MR effect size for
'Beetroot liking || id:ebi−fl187−GCST90094702' on 'Ulcerative colitis (strict) with PSC || id:finngen_R11_K11_UC_STR

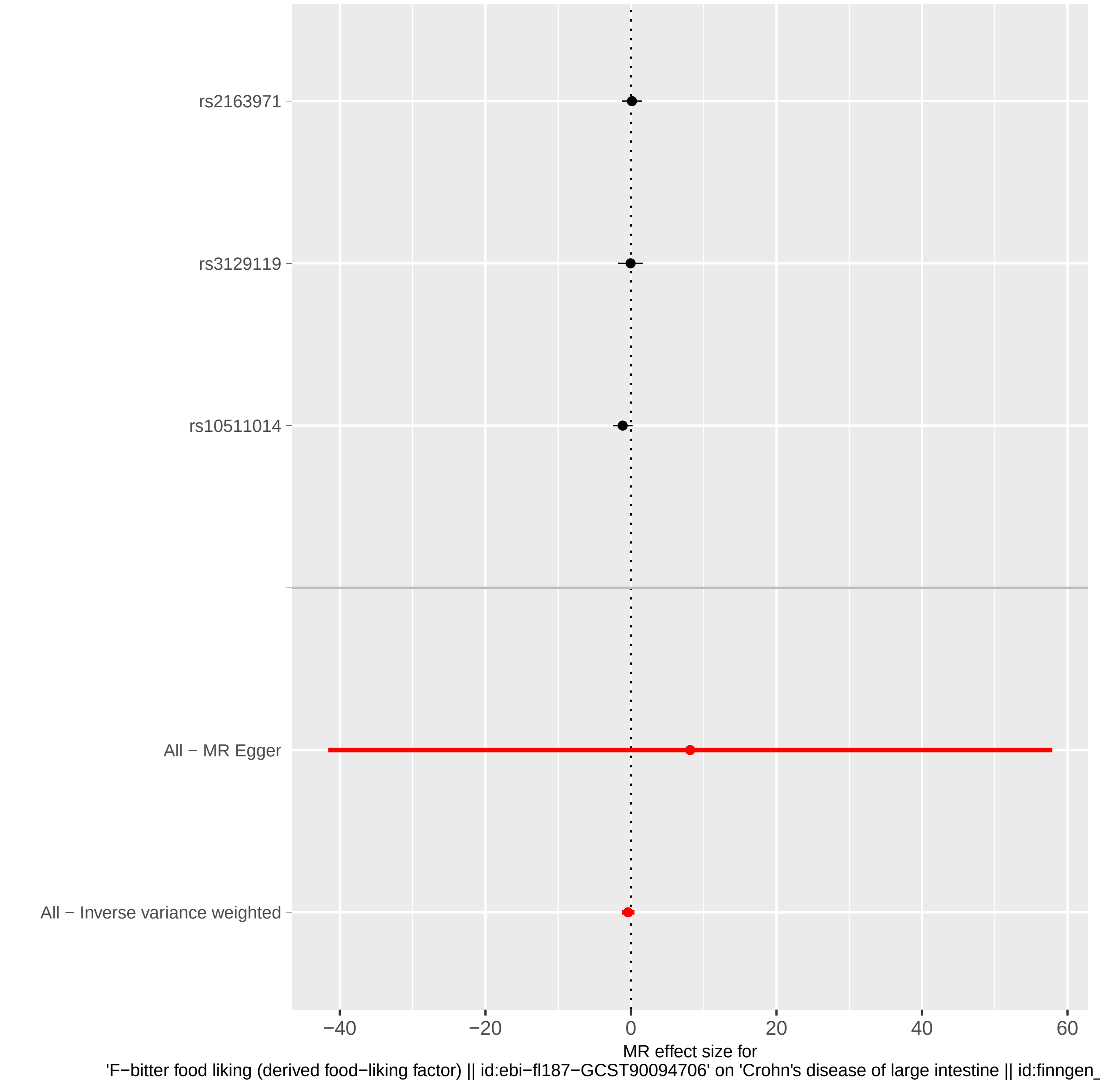
rs2163971
rs3129119
rs10511014
All − MR Egger
All − Inverse variance weighted
−40
−20
0
20
40
60
MR effect size for
'F−bitter food liking (derived food−liking factor) || id:ebi−fl187−GCST90094706' on 'Crohn's disease of large intestine || id:finngen_

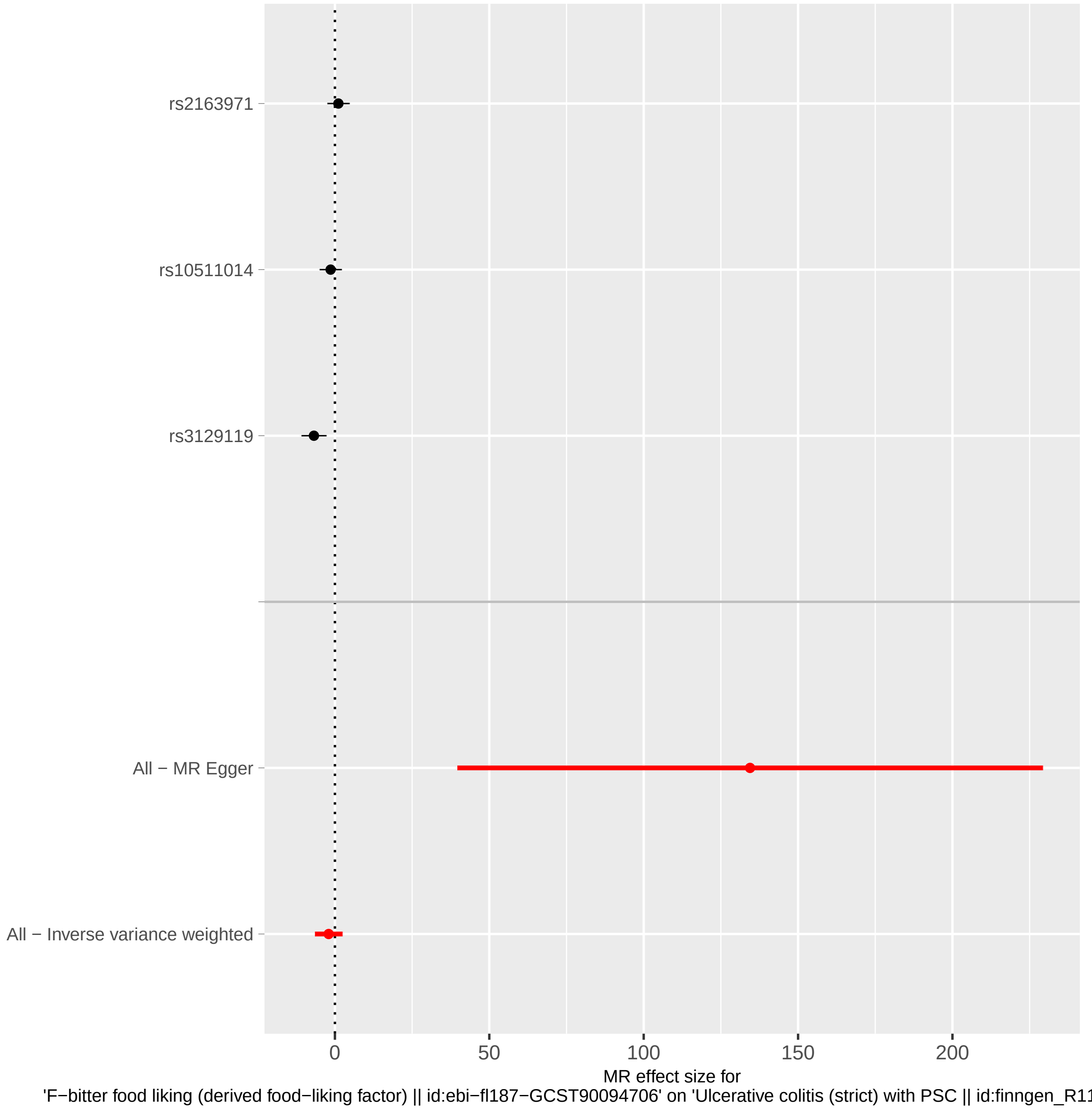
rs2163971
rs10511014
rs3129119
All − MR Egger
All − Inverse variance weighted
0
50
100
150
200
MR effect size for
'F−bitter food liking (derived food−liking factor) || id:ebi−fl187−GCST90094706' on 'Ulcerative colitis (strict) with PSC || id:finngen_R11

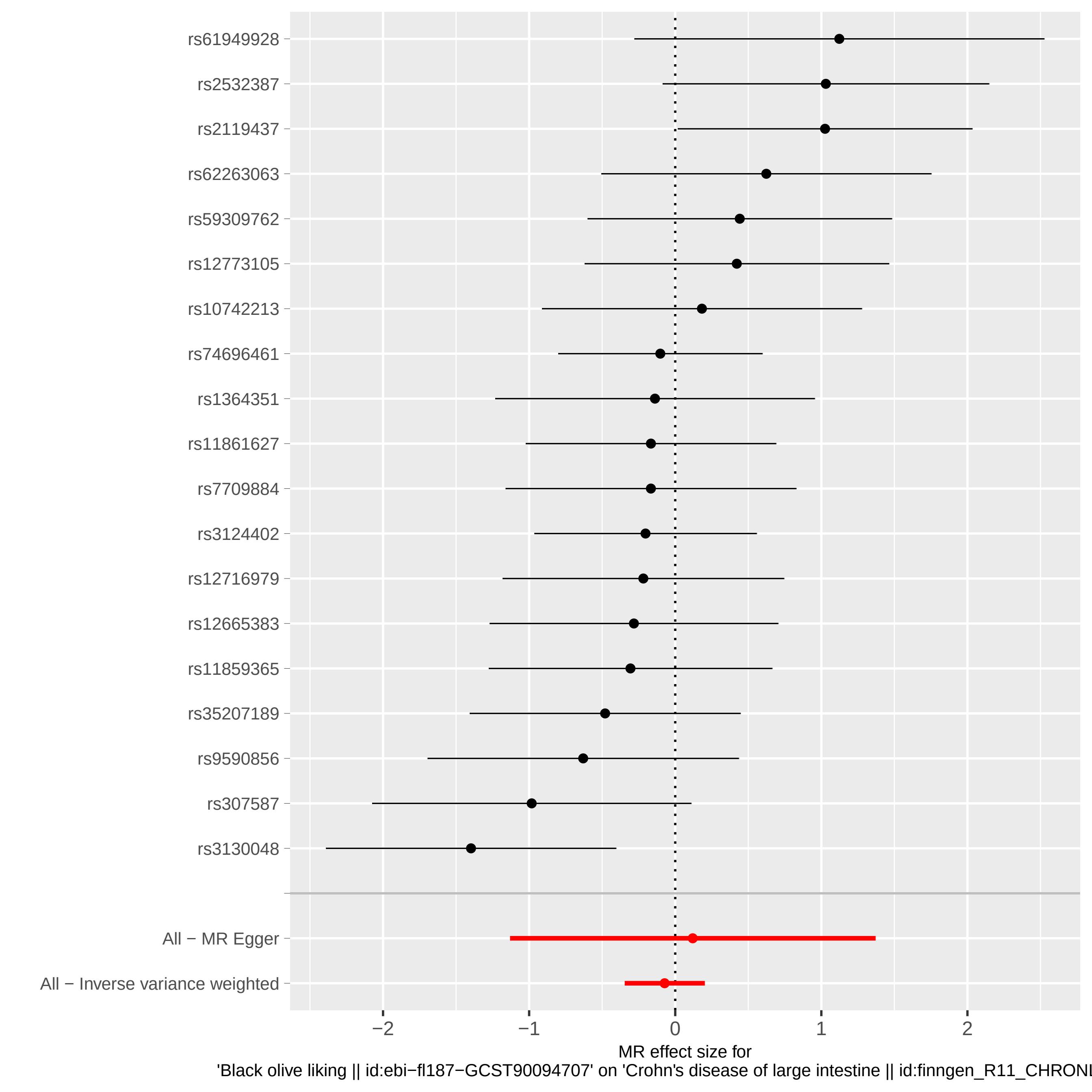
rs61949928
rs2532387
rs2119437
rs62263063
rs59309762
rs12773105
rs10742213
rs74696461
rs1364351
rs11861627
rs7709884
rs3124402
rs12716979
rs12665383
rs11859365
rs35207189
rs9590856
rs307587
rs3130048
All − MR Egger
All − Inverse variance weighted
−2
−1
0
1
2
MR effect size for
'Black olive liking || id:ebi−fl187−GCST90094707' on 'Crohn's disease of large intestine || id:finngen_R11_CHRONI

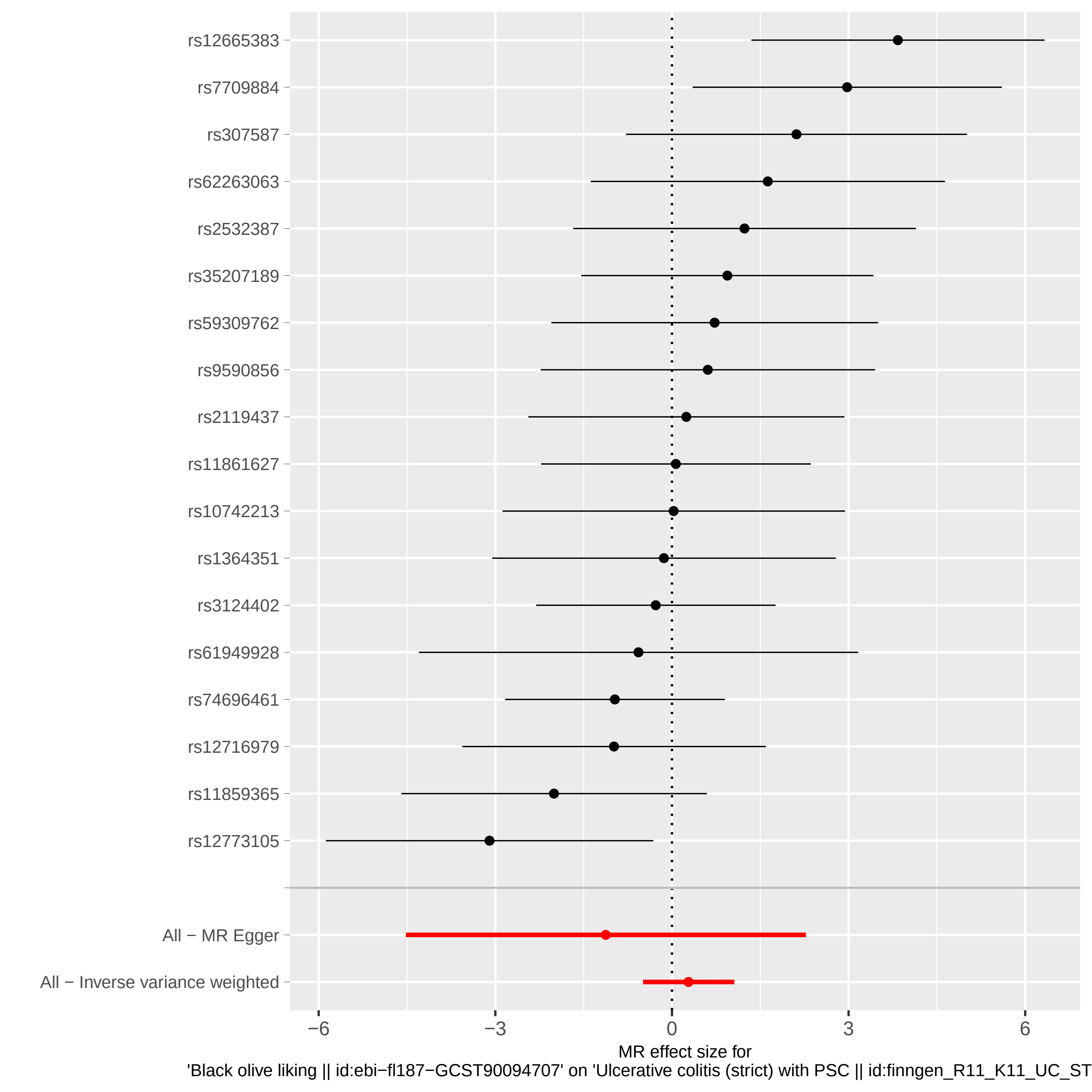
rs12665383
rs7709884
rs307587
rs62263063
rs2532387
rs35207189
rs59309762
rs9590856
rs2119437
rs11861627
rs10742213
rs1364351
rs3124402
rs61949928
rs74696461
rs12716979
rs11859365
rs12773105
All − MR Egger
All − Inverse variance weighted
−6
−3
0
3
6
MR effect size for
'Black olive liking || id:ebi−fl187−GCST90094707' on 'Ulcerative colitis (strict) with PSC || id:finngen_R11_K11_UC_ST

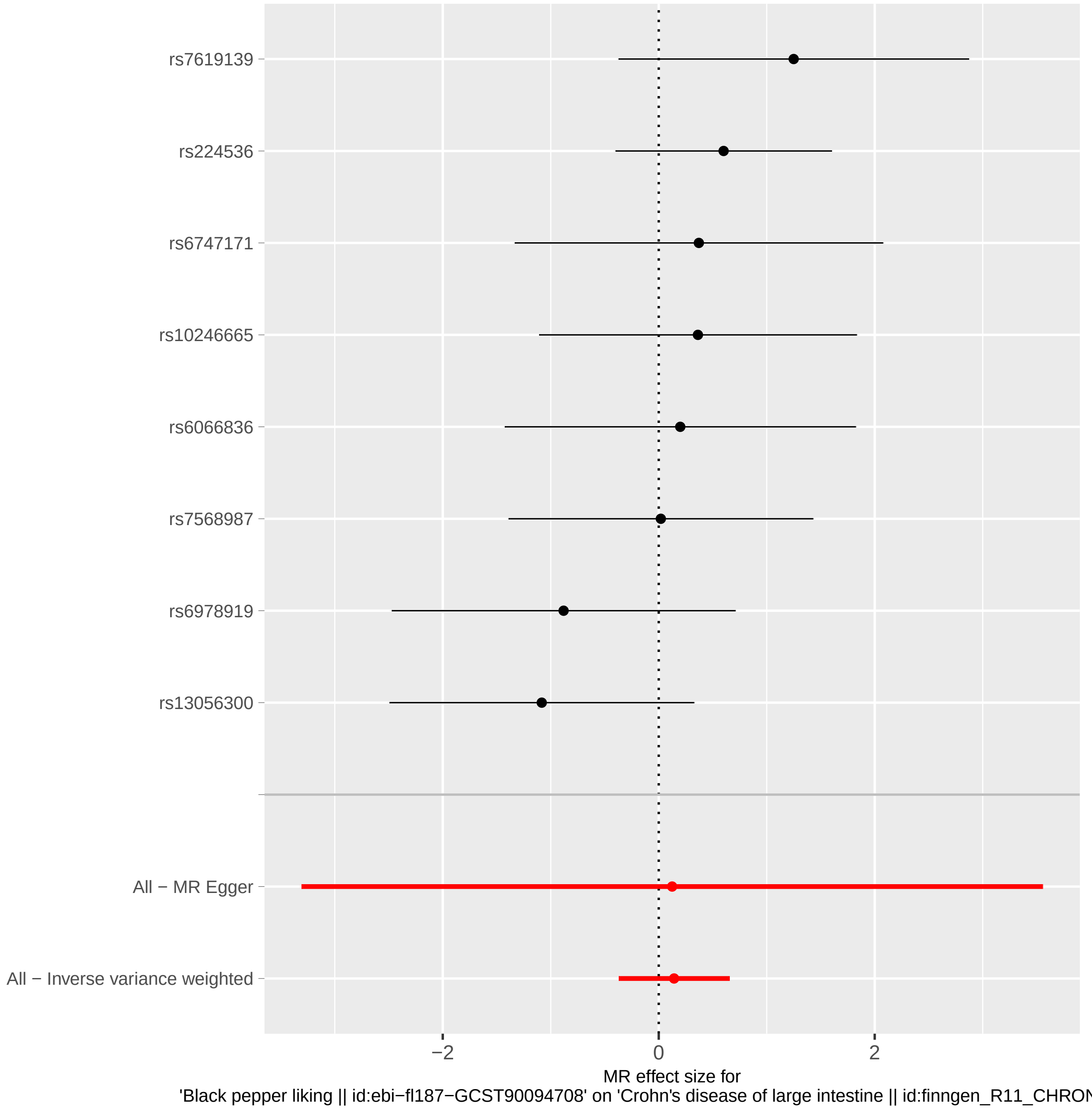
rs7619139
rs224536
rs6747171
rs10246665
rs6066836
rs7568987
rs6978919
rs13056300
All − MR Egger
All − Inverse variance weighted
−2
0
2
MR effect size for
'Black pepper liking || id:ebi−fl187−GCST90094708' on 'Crohn's disease of large intestine || id:finngen_R11_CHRON

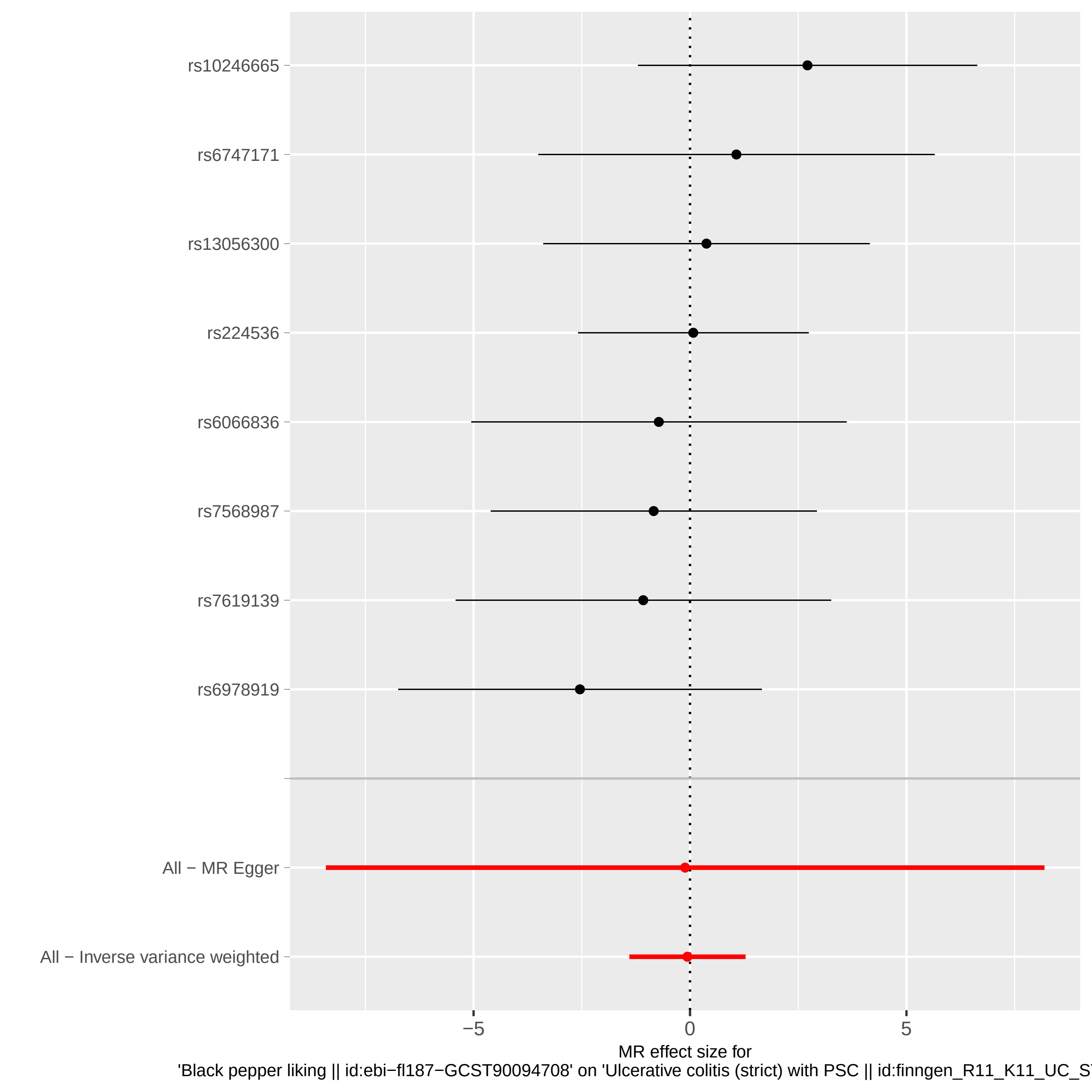
rs10246665
rs6747171
rs13056300
rs224536
rs6066836
rs7568987
rs7619139
rs6978919
All − MR Egger
All − Inverse variance weighted
−5
0
5
MR effect size for
'Black pepper liking || id:ebi−fl187−GCST90094708' on 'Ulcerative colitis (strict) with PSC || id:finngen_R11_K11_UC_S

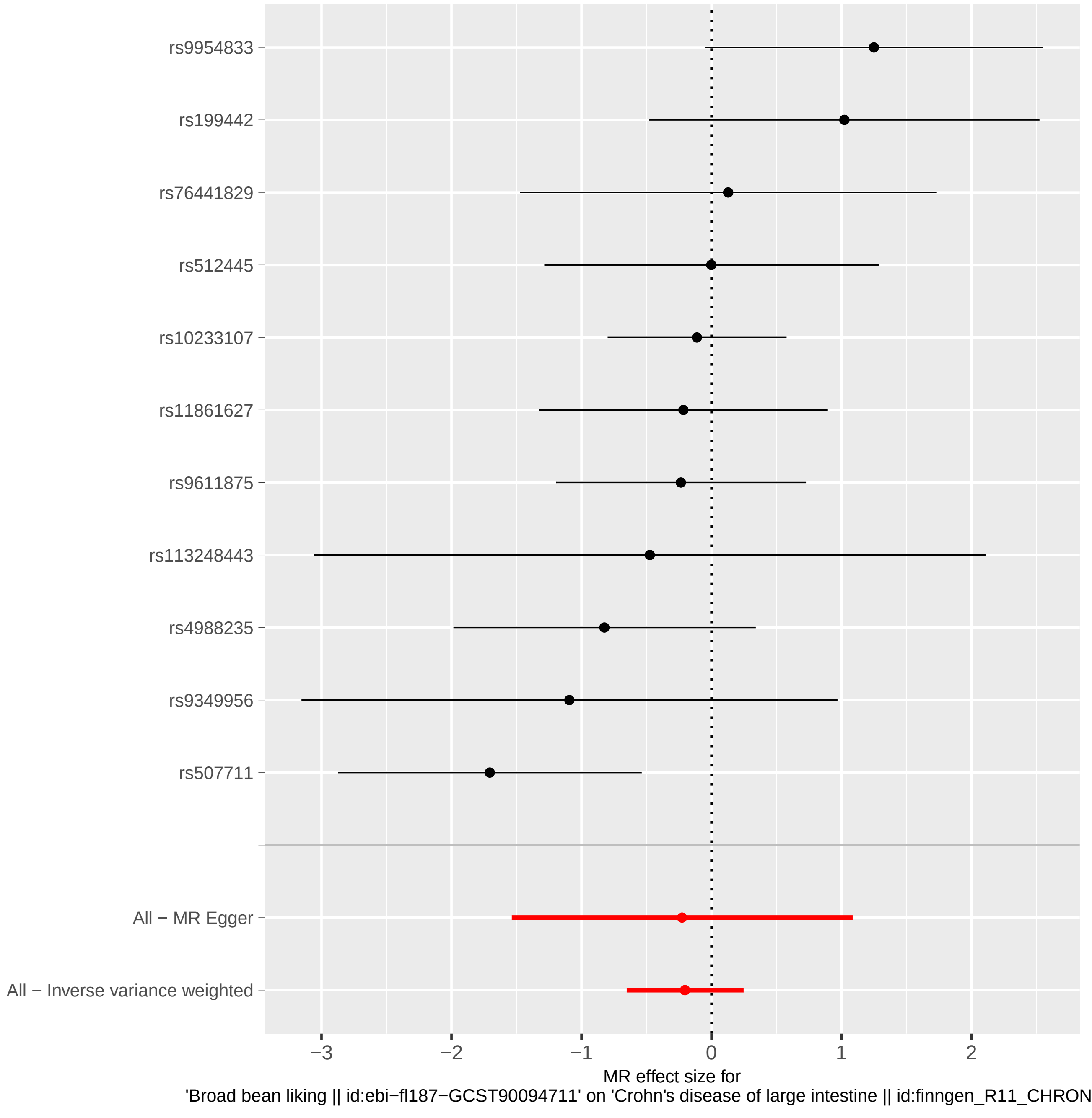
rs9954833
rs199442
rs76441829
rs512445
rs10233107
rs11861627
rs9611875
rs113248443
rs4988235
rs9349956
rs507711
All − MR Egger
All − Inverse variance weighted
−3
−2
−1
0
1
2
MR effect size for
'Broad bean liking || id:ebi−fl187−GCST90094711' on 'Crohn's disease of large intestine || id:finngen_R11_CHRON

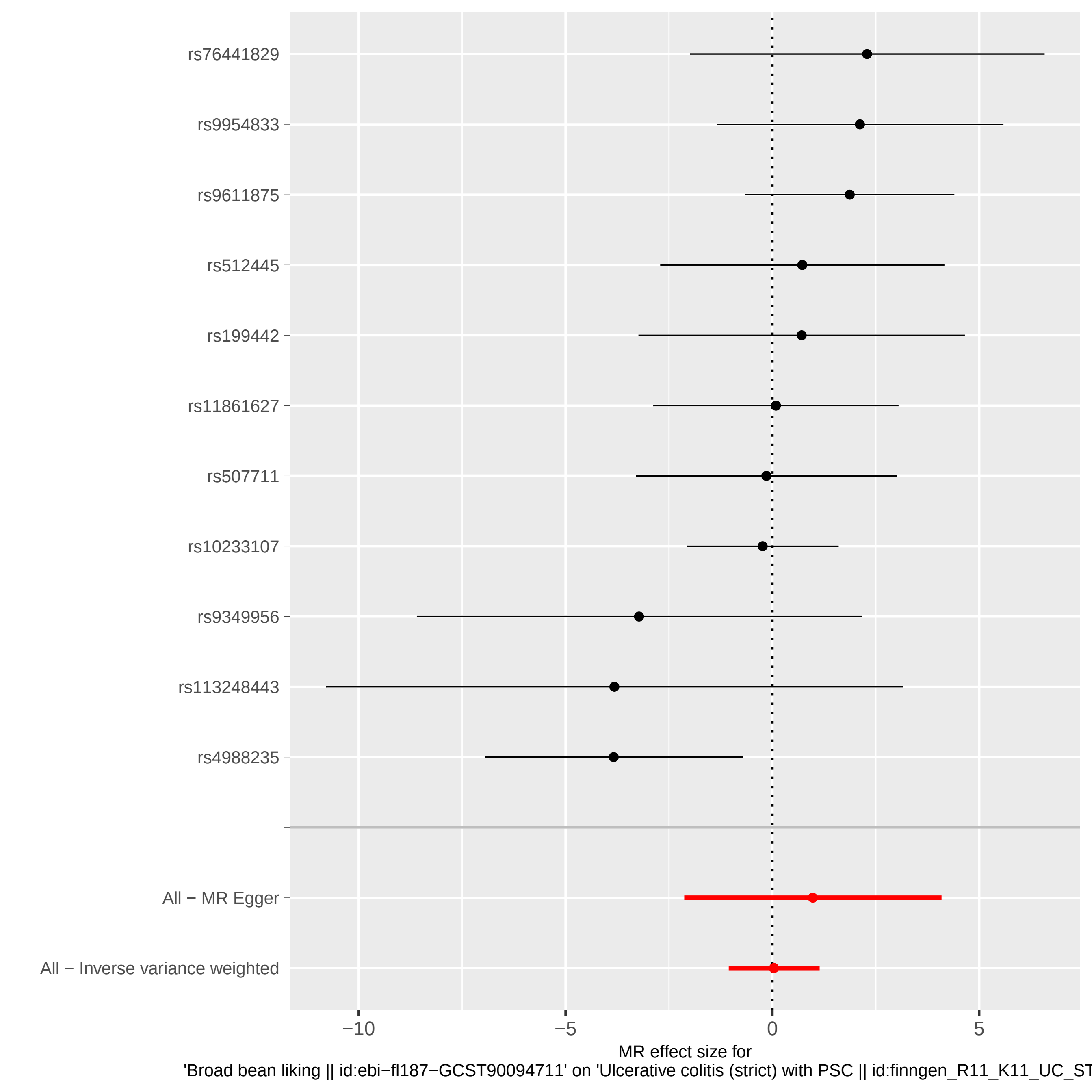
rs76441829
rs9954833
rs9611875
rs512445
rs199442
rs11861627
rs507711
rs10233107
rs9349956
rs113248443
rs4988235
All − MR Egger
All − Inverse variance weighted
−10
−5
0
5
MR effect size for
'Broad bean liking || id:ebi−fl187−GCST90094711' on 'Ulcerative colitis (strict) with PSC || id:finngen_R11_K11_UC_ST

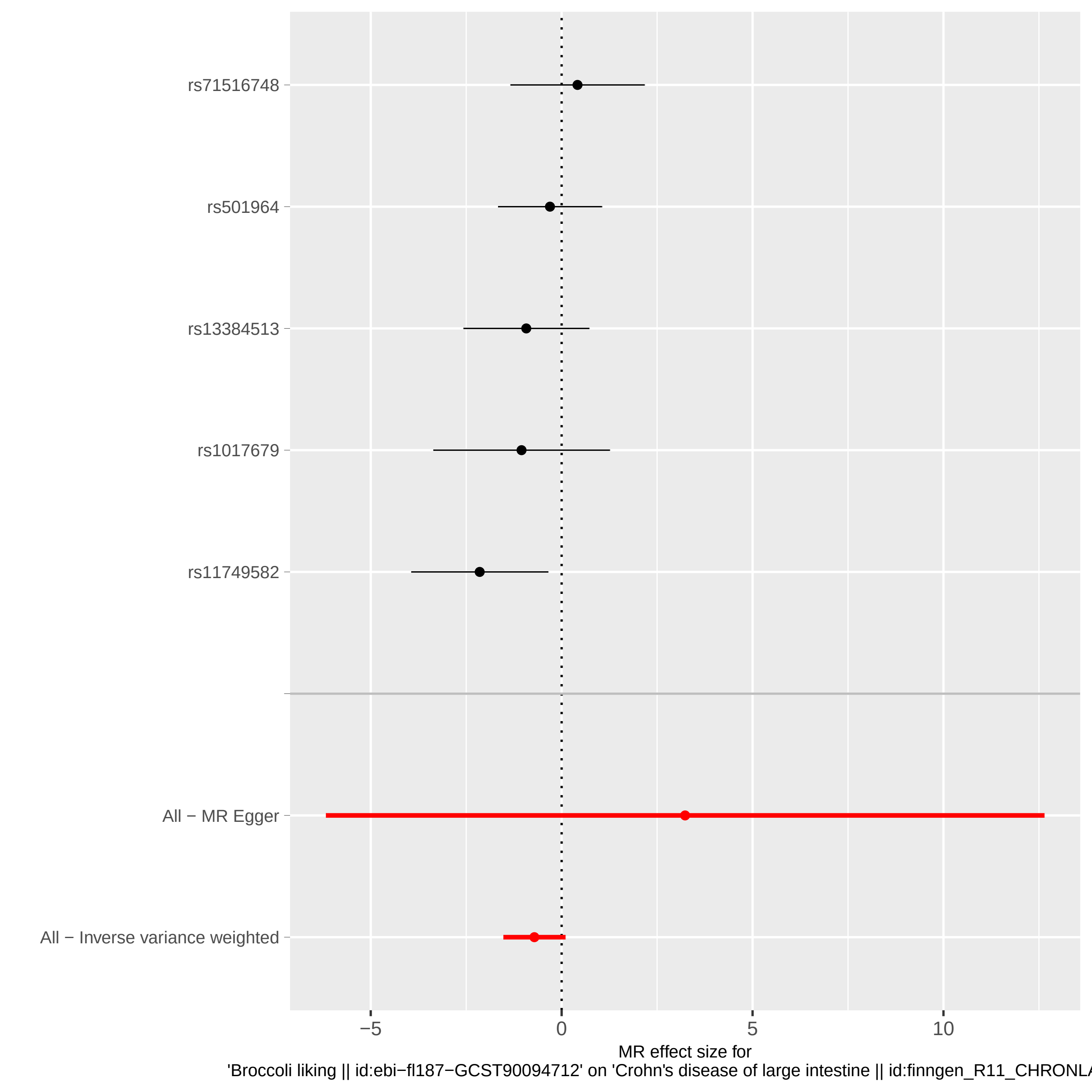
rs71516748
rs501964
rs13384513
rs1017679
rs11749582
All − MR Egger
All − Inverse variance weighted
−5
0
5
10
MR effect size for
'Broccoli liking || id:ebi−fl187−GCST90094712' on 'Crohn's disease of large intestine || id:finngen_R11_CHRONLA

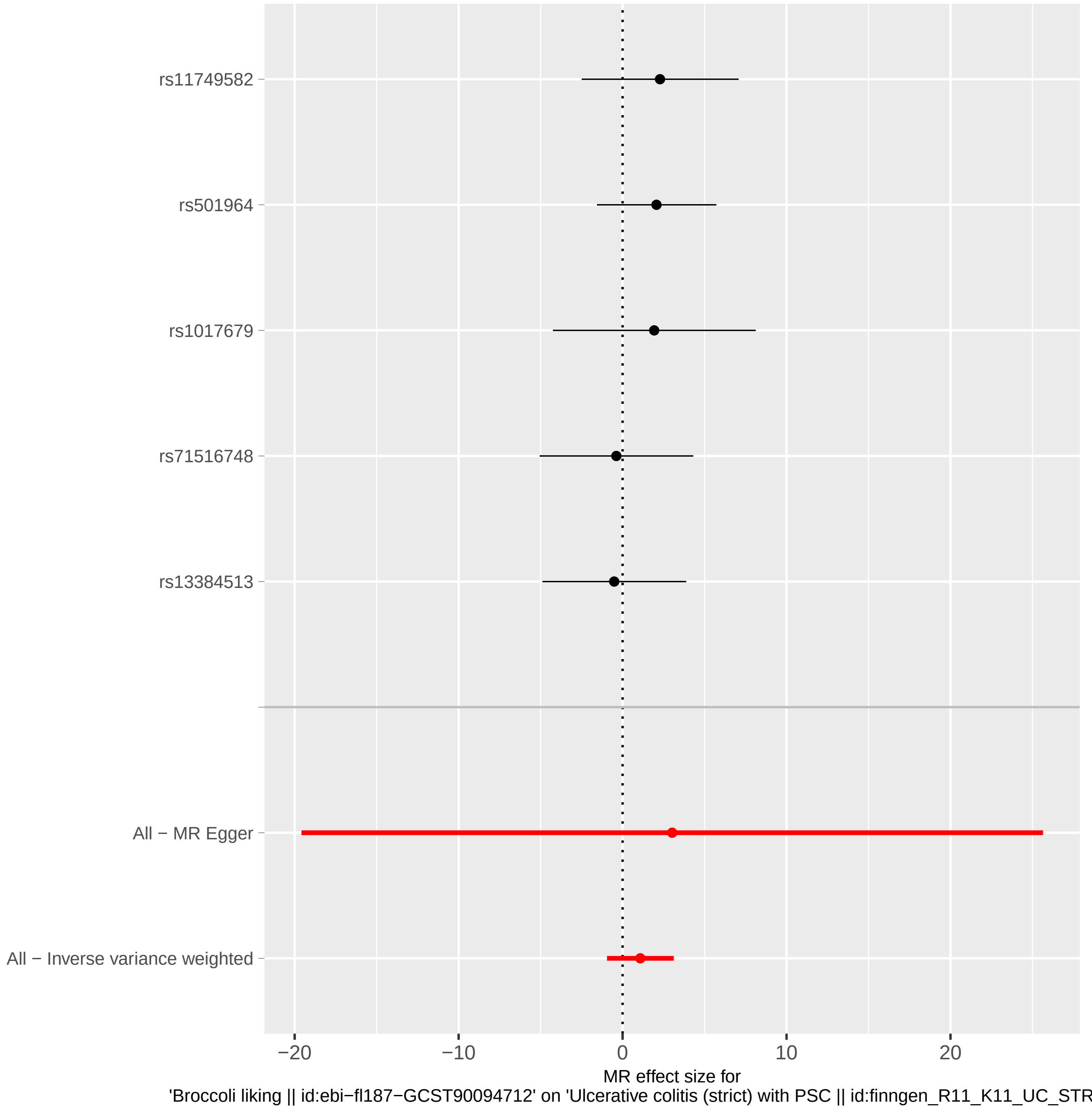

rs11749582
rs501964
rs1017679
rs71516748
rs13384513
All − MR Egger
All − Inverse variance weighted
−20
−10
0
10
20
MR effect size for
'Broccoli liking || id:ebi−fl187−GCST90094712' on 'Ulcerative colitis (strict) with PSC || id:finngen_R11_K11_UC_STR

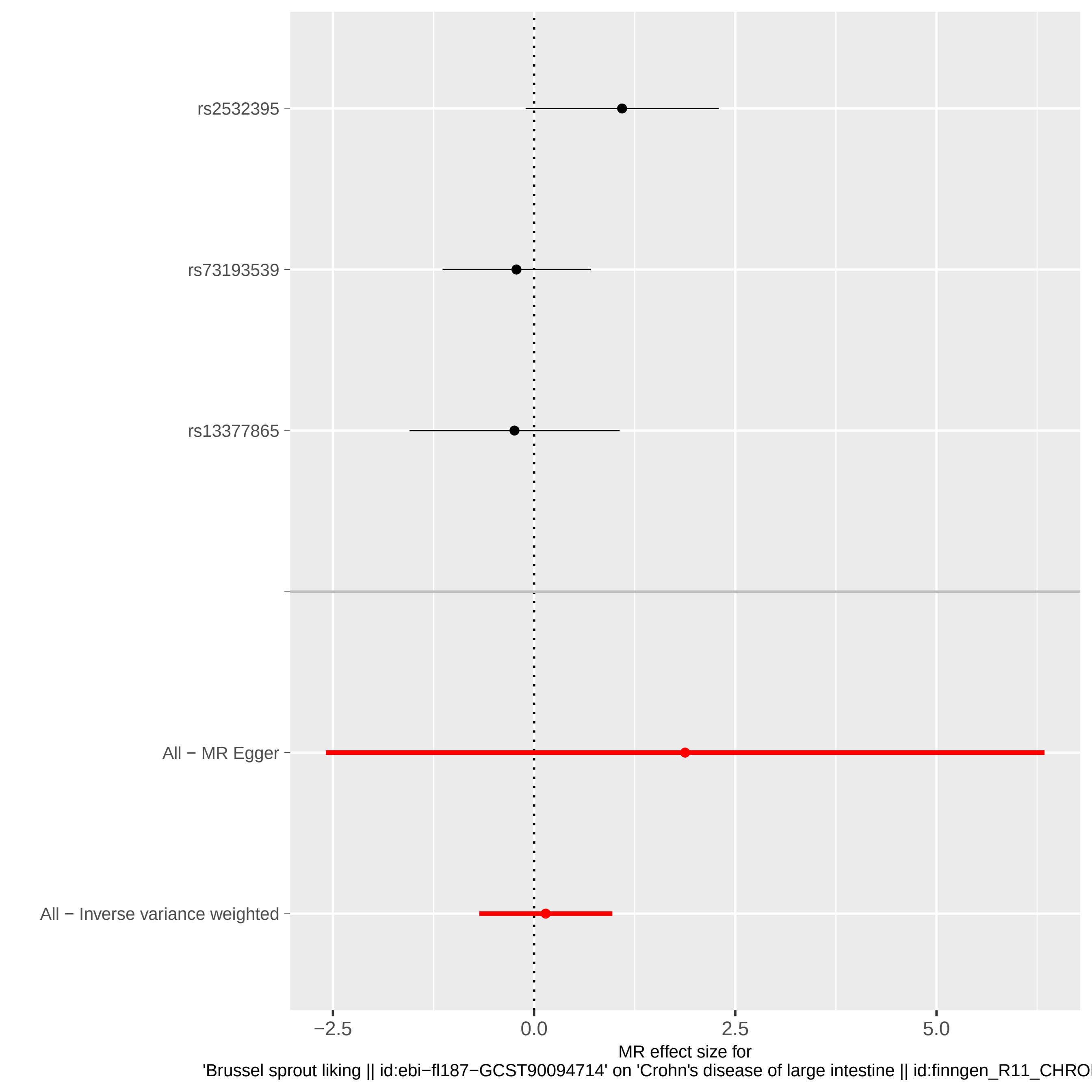
rs2532395
rs73193539
rs13377865
All − MR Egger
All − Inverse variance weighted
−2.5
0.0
2.5
5.0
MR effect size for
'Brussel sprout liking || id:ebi−fl187−GCST90094714' on 'Crohn's disease of large intestine || id:finngen_R11_CHRO

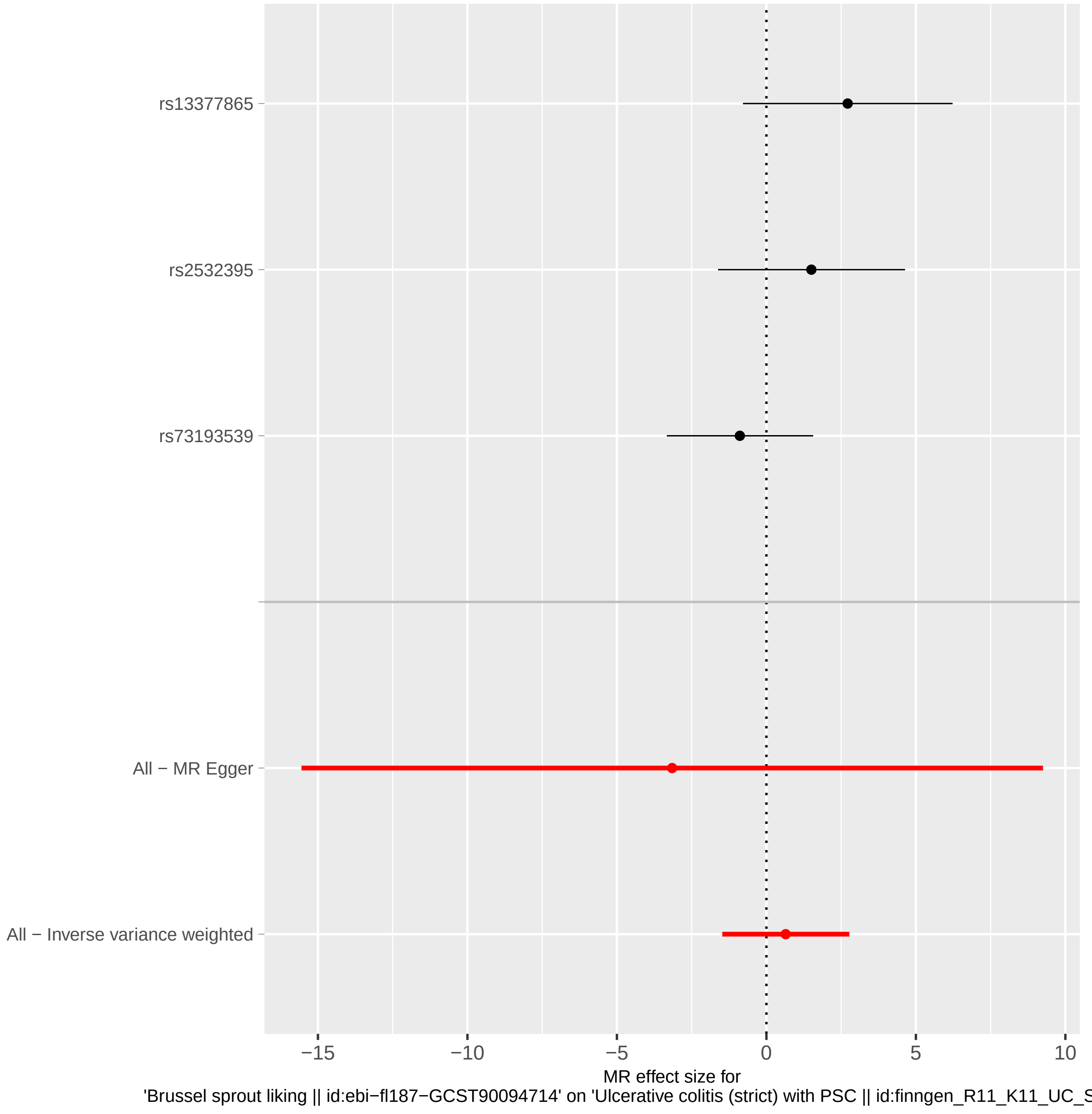

rs13377865
rs2532395
rs73193539
All − MR Egger
All − Inverse variance weighted
−15
−10
−5
0
5
10
MR effect size for
'Brussel sprout liking || id:ebi−fl187−GCST90094714' on 'Ulcerative colitis (strict) with PSC || id:finngen_R11_K11_UC_S

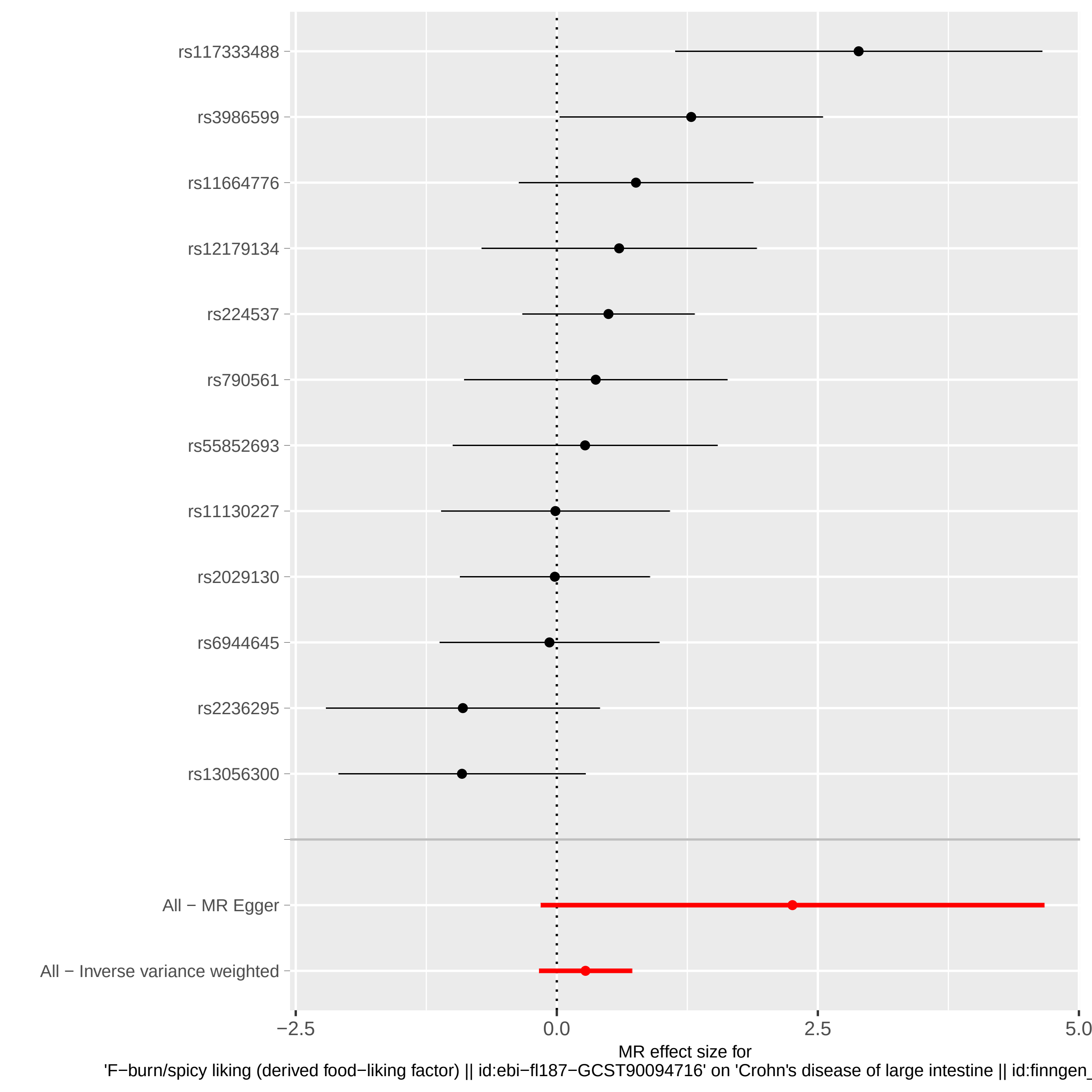

rs117333488
rs3986599
rs11664776
rs12179134
rs224537
rs790561
rs55852693
rs11130227
rs2029130
rs6944645
rs2236295
rs13056300
All − MR Egger
All − Inverse variance weighted
−2.5
0.0
2.5
5.0
MR effect size for
'F−burn/spicy liking (derived food−liking factor) || id:ebi−fl187−GCST90094716' on 'Crohn's disease of large intestine || id:finngen_

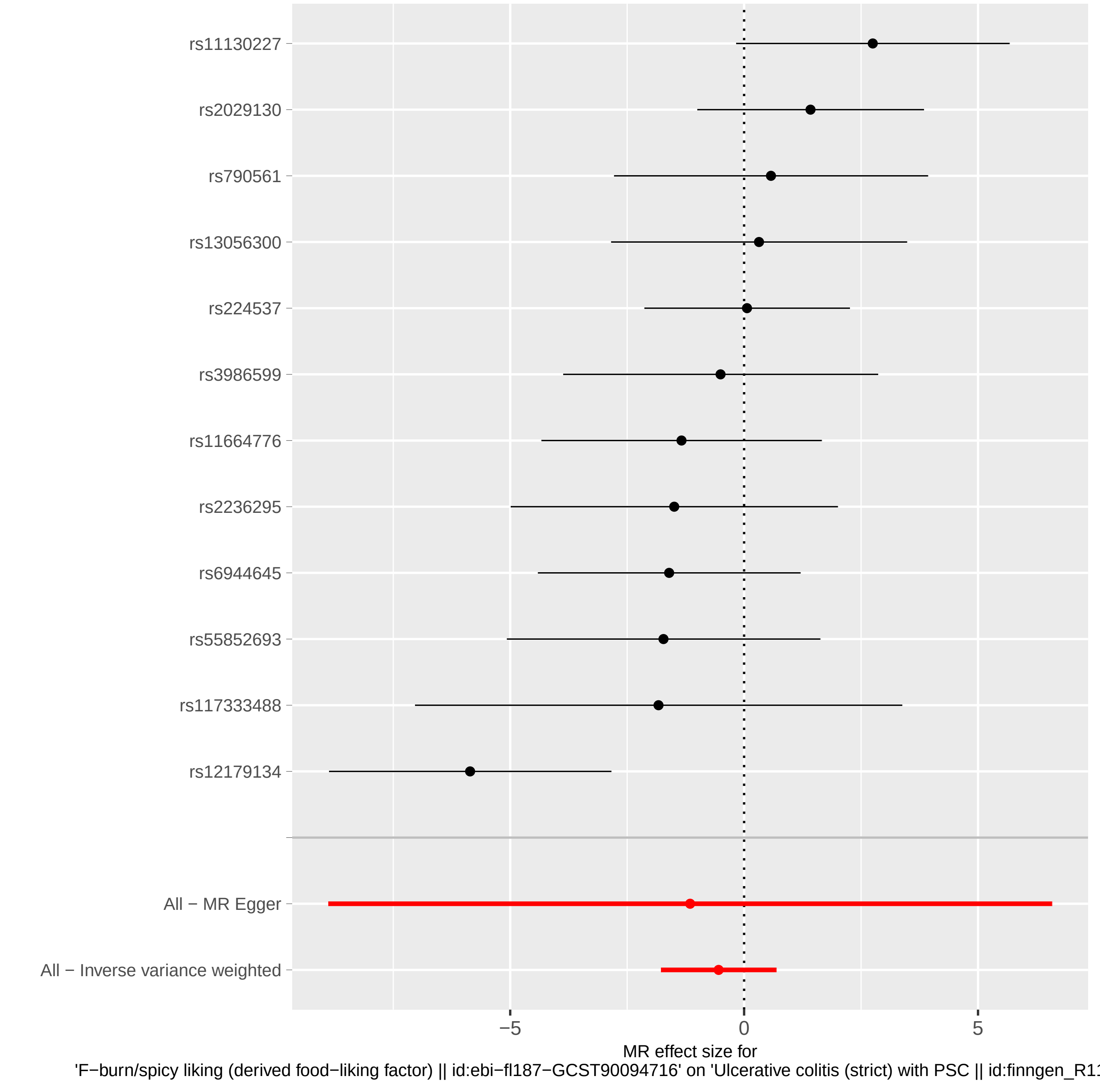

rs11130227
rs2029130
rs790561
rs13056300
rs224537
rs3986599
rs11664776
rs2236295
rs6944645
rs55852693
rs117333488
rs12179134
All − MR Egger
All − Inverse variance weighted
−5
0
5
MR effect size for
'F−burn/spicy liking (derived food−liking factor) || id:ebi−fl187−GCST90094716' on 'Ulcerative colitis (strict) with PSC || id:finngen_R1

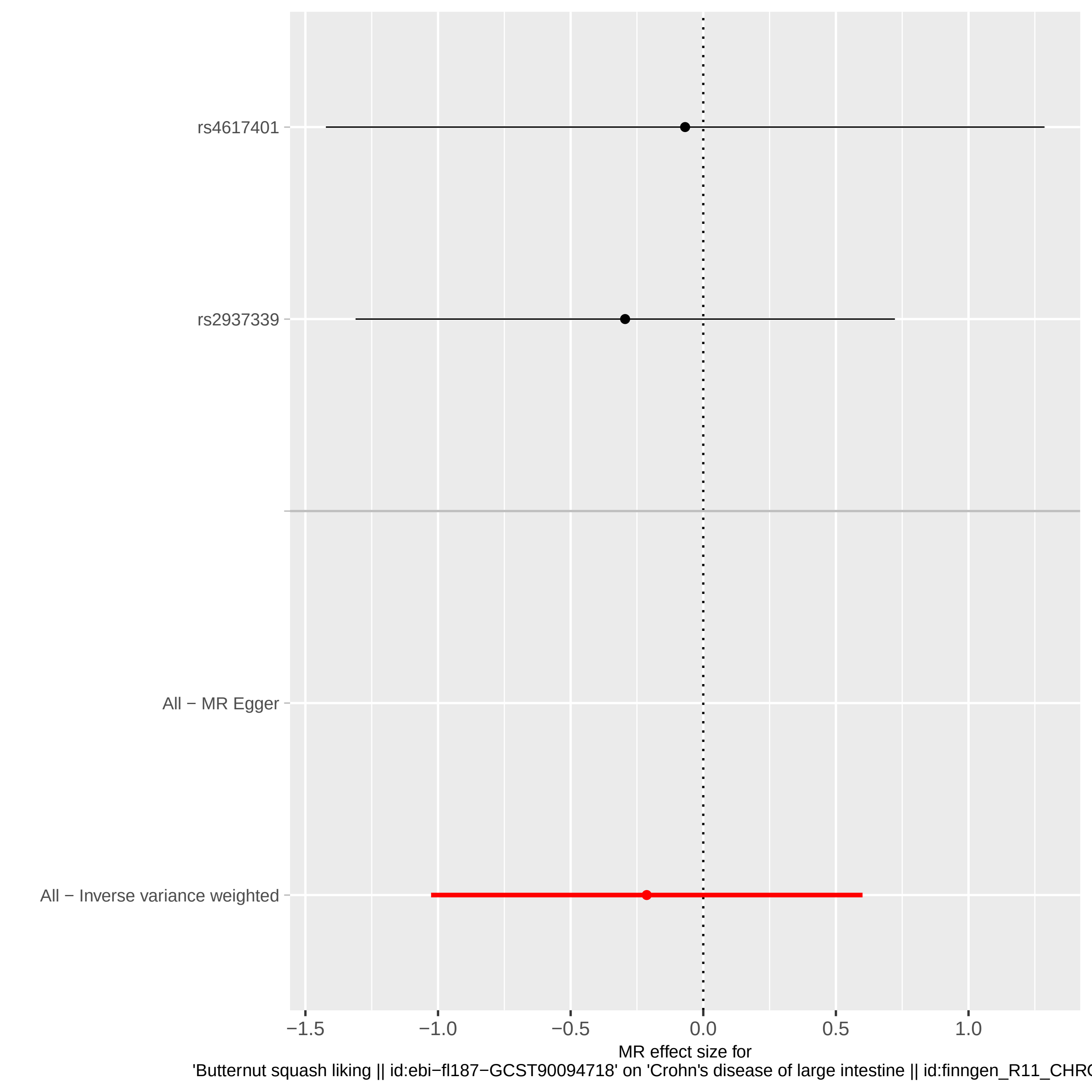
rs4617401
rs2937339
All − MR Egger
All − Inverse variance weighted
−1.5
−1.0
−0.5
0.0
0.5
1.0
MR effect size for
'Butternut squash liking || id:ebi−fl187−GCST90094718' on 'Crohn's disease of large intestine || id:finngen_R11_CHR

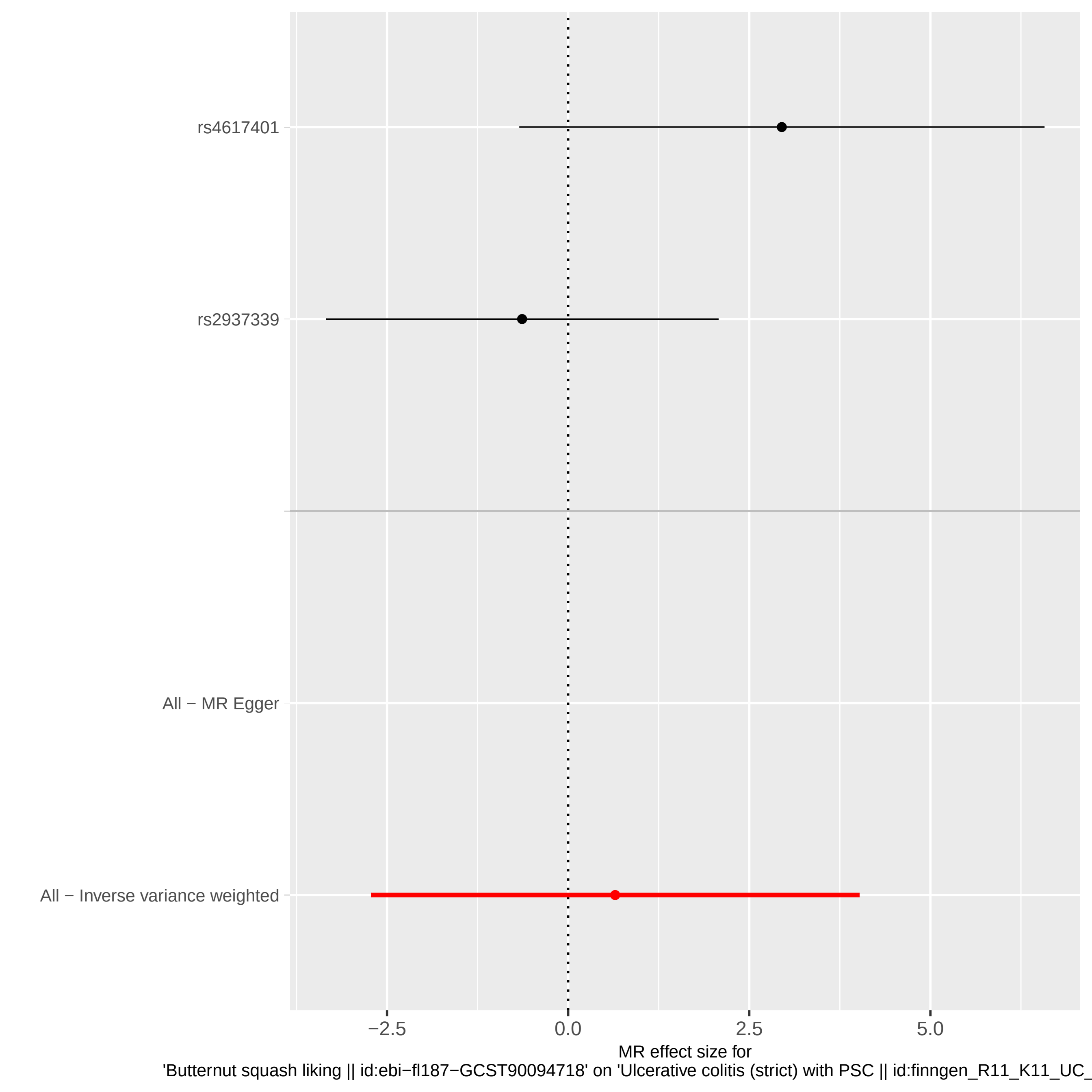
rs4617401
rs2937339
All − MR Egger
All − Inverse variance weighted
−2.5
0.0
2.5
5.0
MR effect size for
'Butternut squash liking || id:ebi−fl187−GCST90094718' on 'Ulcerative colitis (strict) with PSC || id:finngen_R11_K11_UC_

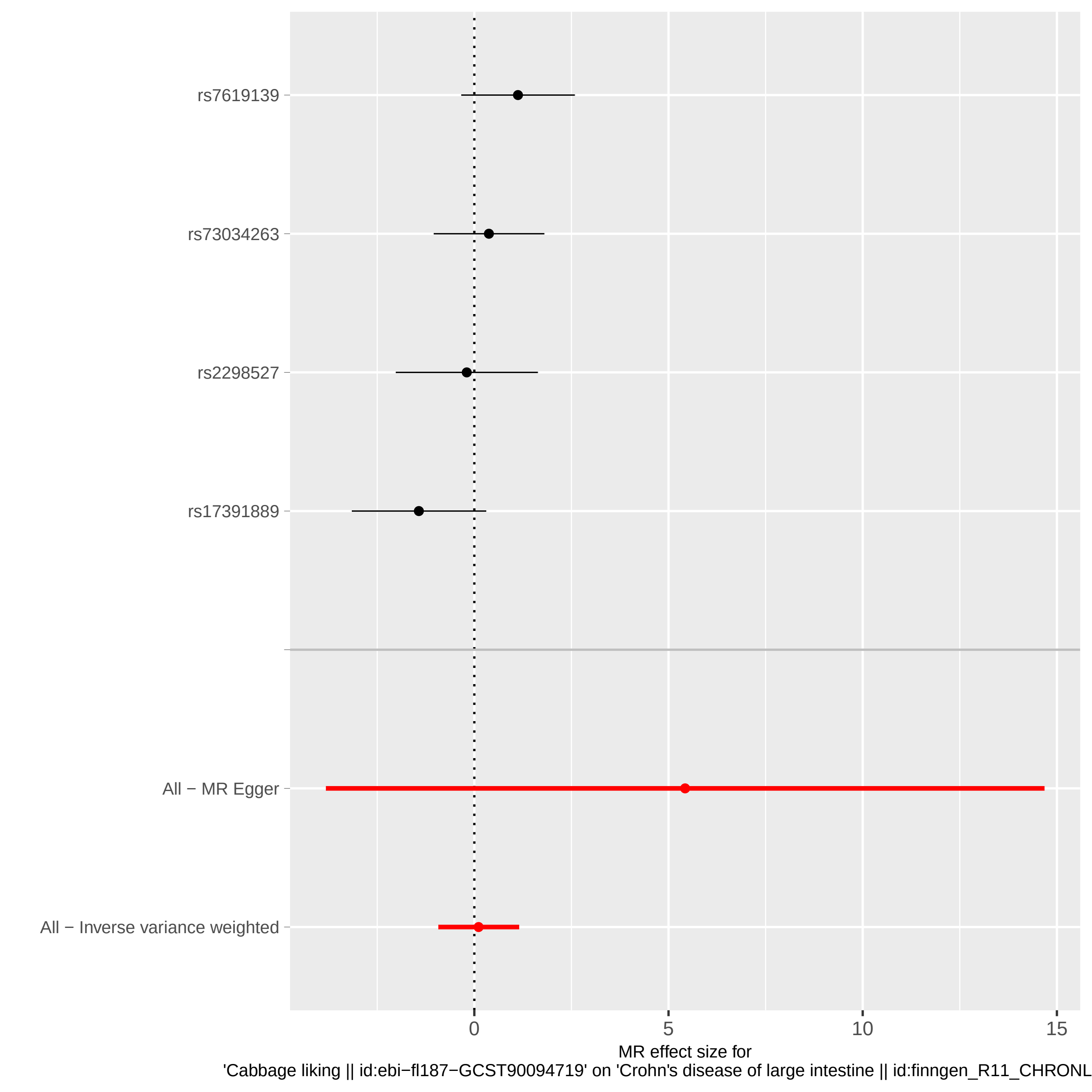

rs7619139
rs73034263
rs2298527
rs17391889
All − MR Egger
All − Inverse variance weighted
0
5
10
15
MR effect size for
'Cabbage liking || id:ebi−fl187−GCST90094719' on 'Crohn's disease of large intestine || id:finngen_R11_CHRONL

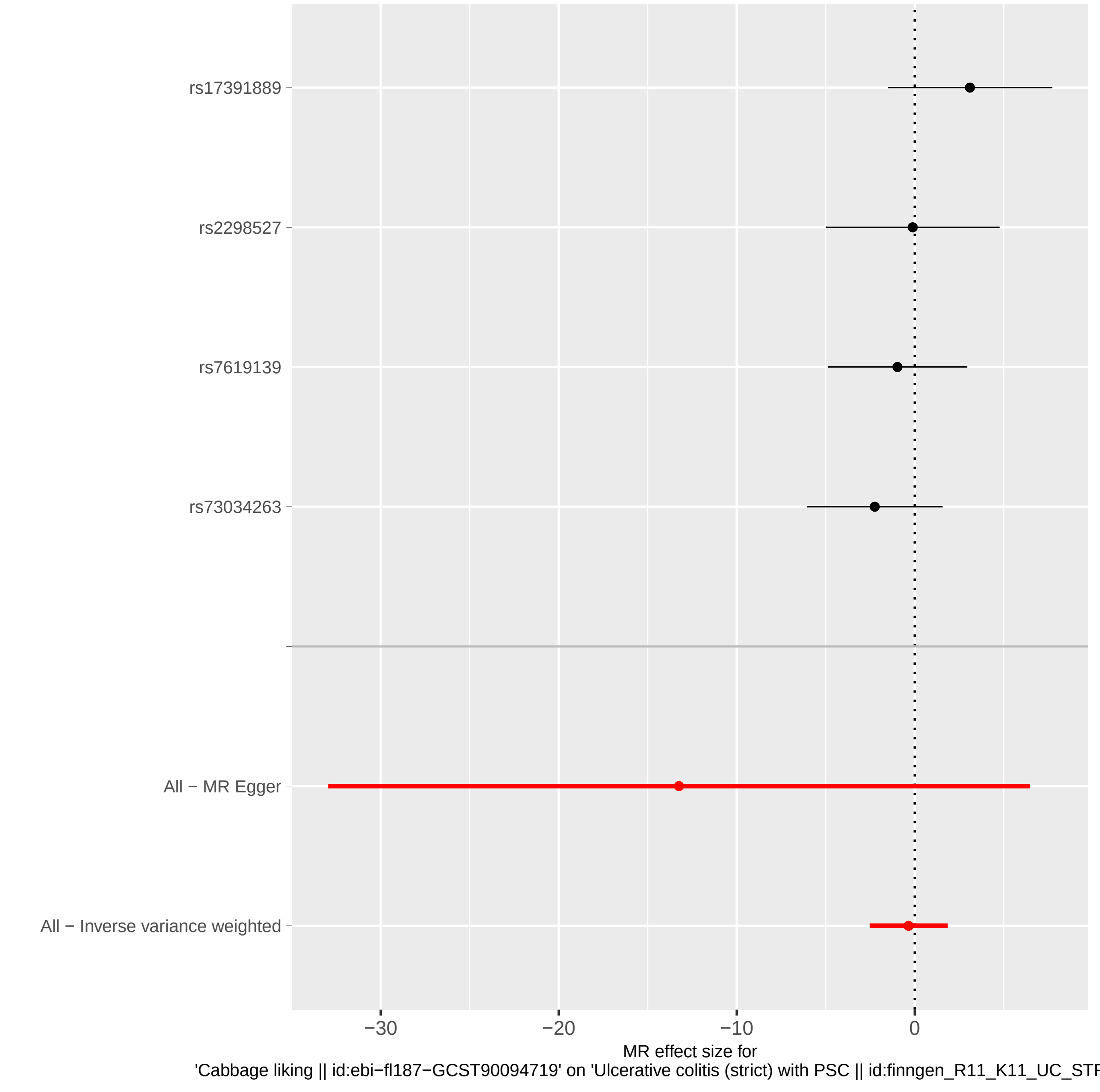

rs17391889
rs2298527
rs7619139
rs73034263
All − MR Egger
All − Inverse variance weighted
−30
−20
−10
0
MR effect size for
'Cabbage liking || id:ebi−fl187−GCST90094719' on 'Ulcerative colitis (strict) with PSC || id:finngen_R11_K11_UC_STR

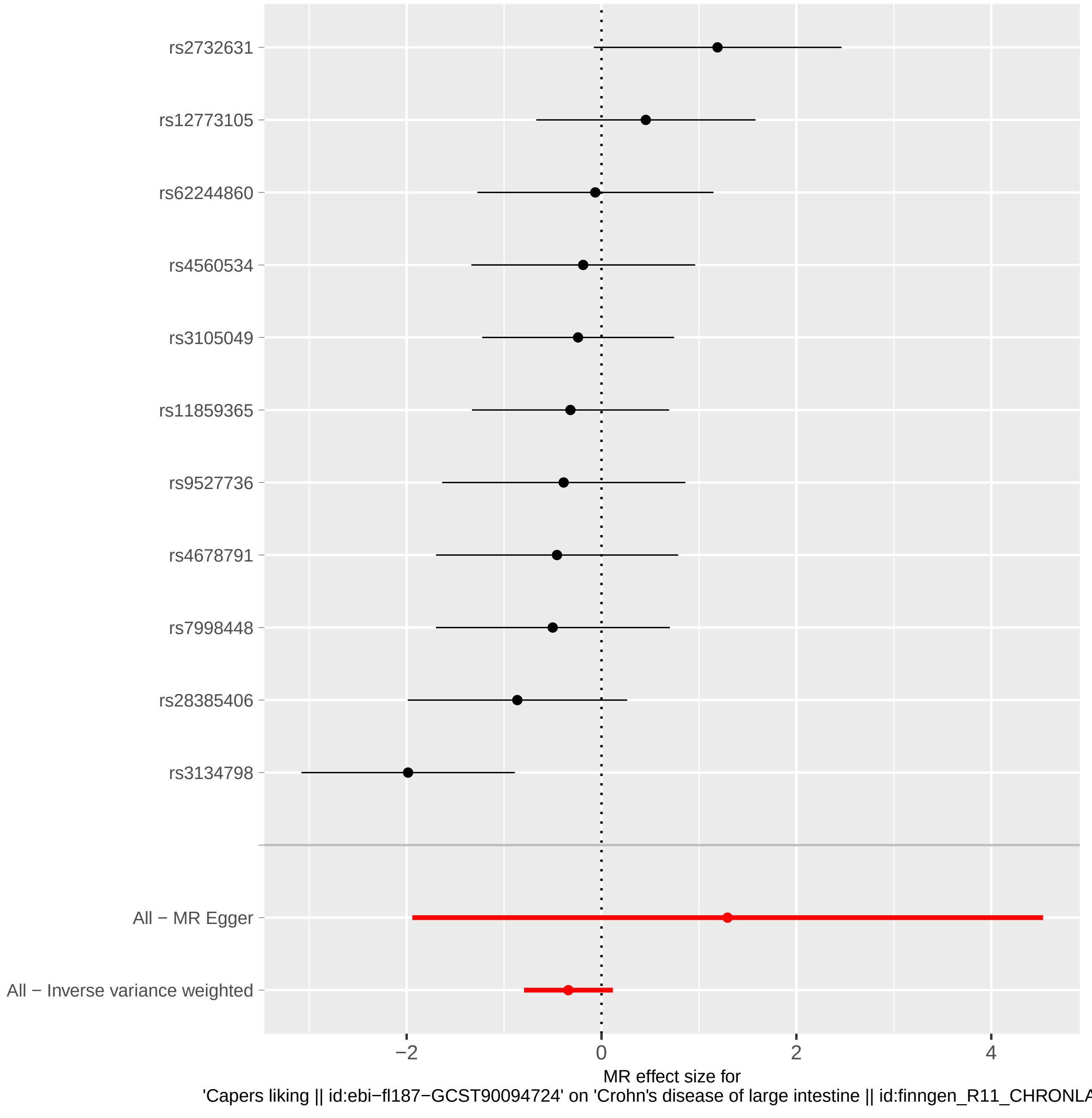
rs2732631
rs12773105
rs62244860
rs4560534
rs3105049
rs11859365
rs9527736
rs4678791
rs7998448
rs28385406
rs3134798
All − MR Egger
All − Inverse variance weighted
−2
0
2
4
MR effect size for
'Capers liking || id:ebi−fl187−GCST90094724' on 'Crohn's disease of large intestine || id:finngen_R11_CHRONLA

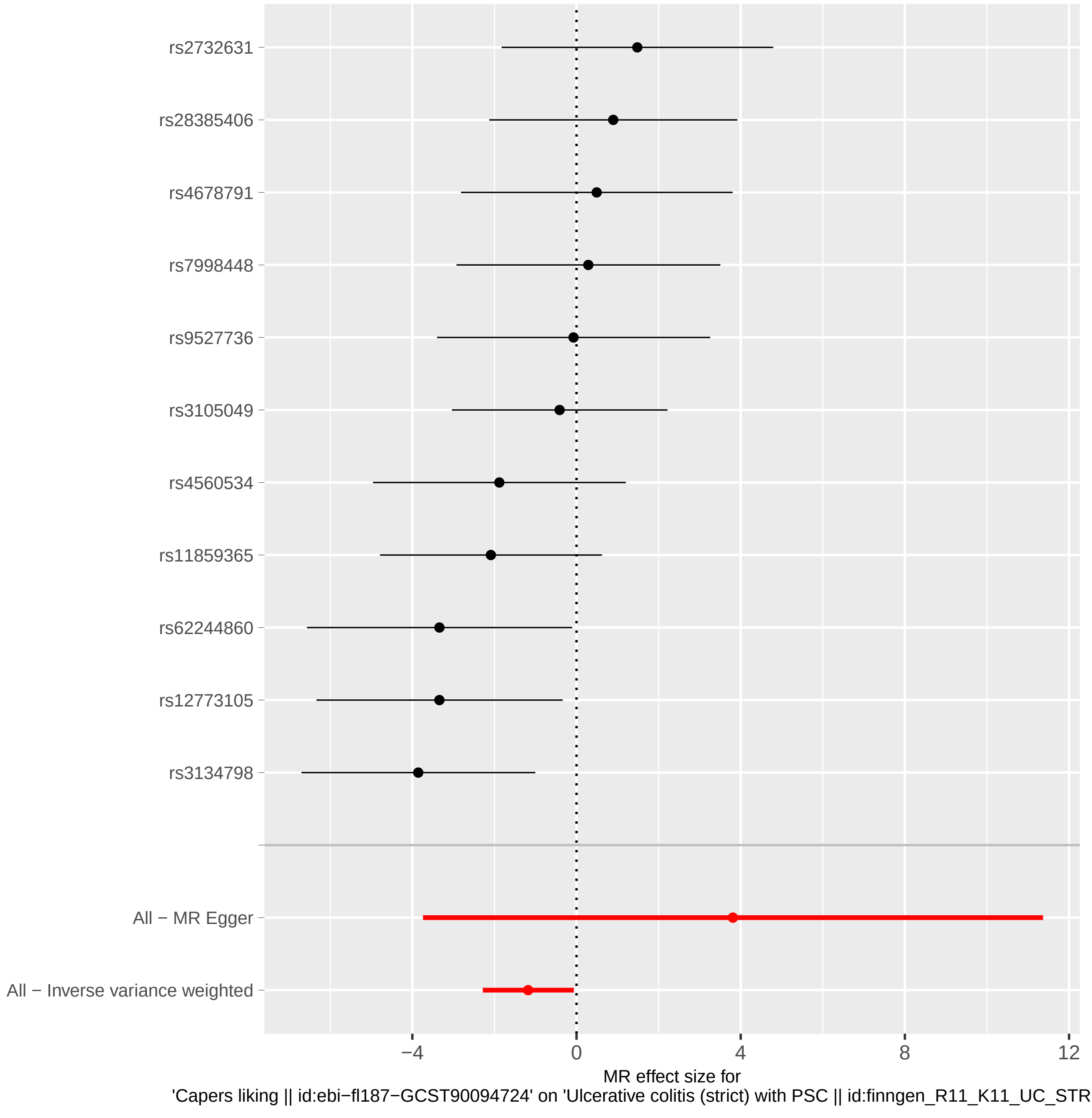
rs2732631
rs28385406
rs4678791
rs7998448
rs9527736
rs3105049
rs4560534
rs11859365
rs62244860
rs12773105
rs3134798
All − MR Egger
All − Inverse variance weighted
−4
0
4
8
12
MR effect size for
'Capers liking || id:ebi−fl187−GCST90094724' on 'Ulcerative colitis (strict) with PSC || id:finngen_R11_K11_UC_STR

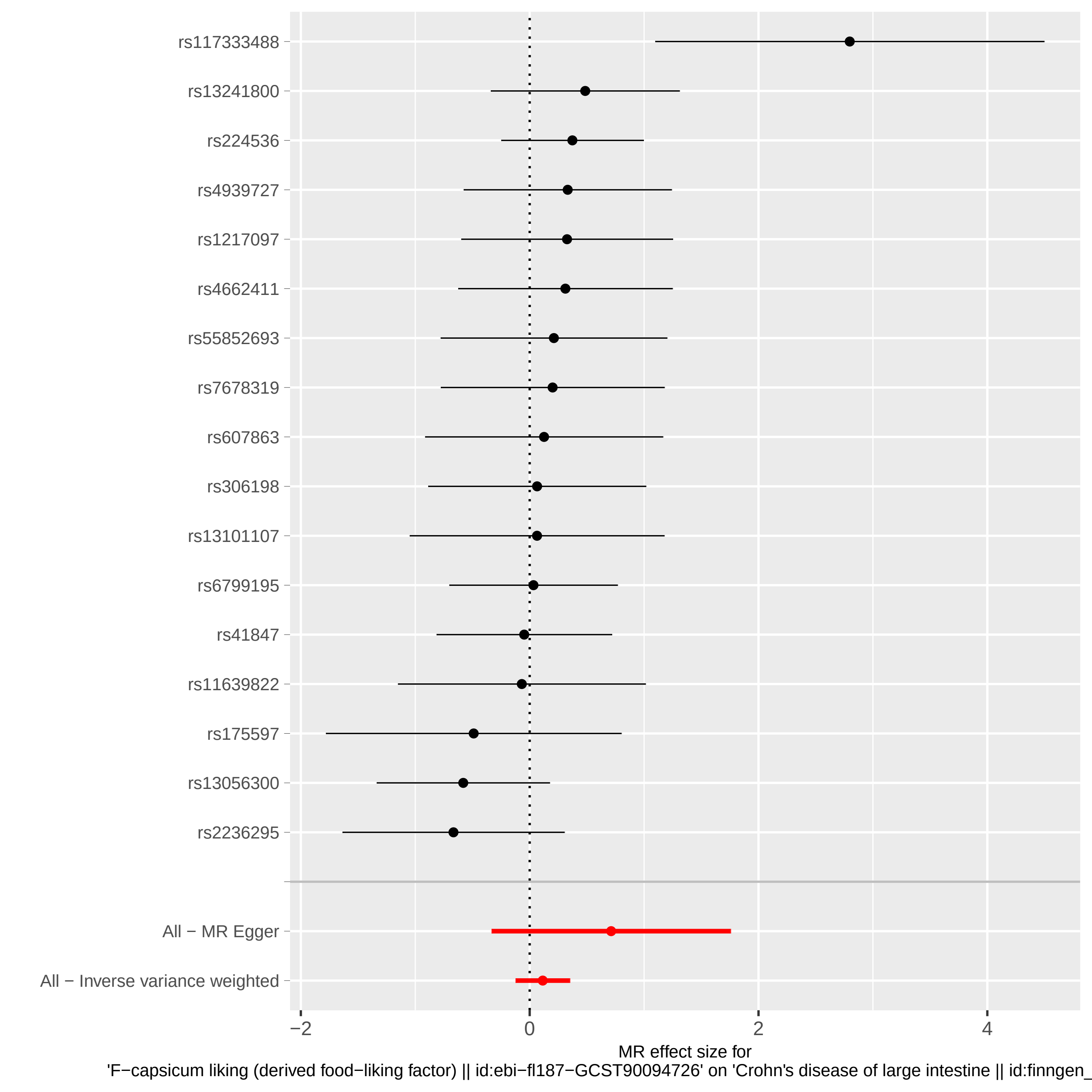

rs117333488
rs13241800
rs224536
rs4939727
rs1217097
rs4662411
rs55852693
rs7678319
rs607863
rs306198
rs13101107
rs6799195
rs41847
rs11639822
rs175597
rs13056300
rs2236295
All − MR Egger
All − Inverse variance weighted
−2
0
2
4
MR effect size for
'F−capsicum liking (derived food−liking factor) || id:ebi−fl187−GCST90094726' on 'Crohn's disease of large intestine || id:finngen_

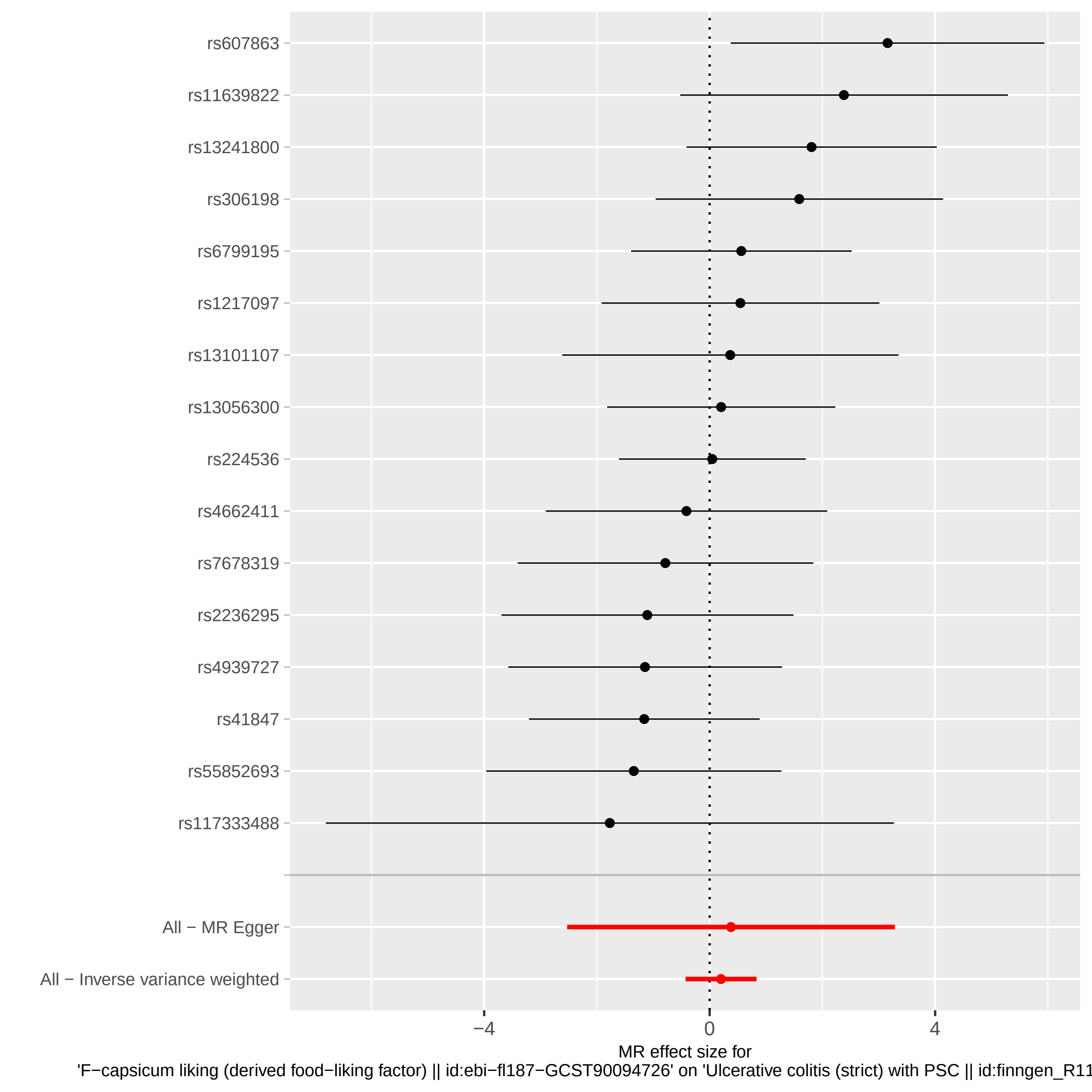
rs607863
rs11639822
rs13241800
rs306198
rs6799195
rs1217097
rs13101107
rs13056300
rs224536
rs4662411
rs7678319
rs2236295
rs4939727
rs41847
rs55852693
rs117333488
All − MR Egger
All − Inverse variance weighted
−4
0
4
MR effect size for
'F−capsicum liking (derived food−liking factor) || id:ebi−fl187−GCST90094726' on 'Ulcerative colitis (strict) with PSC || id:finngen_R11

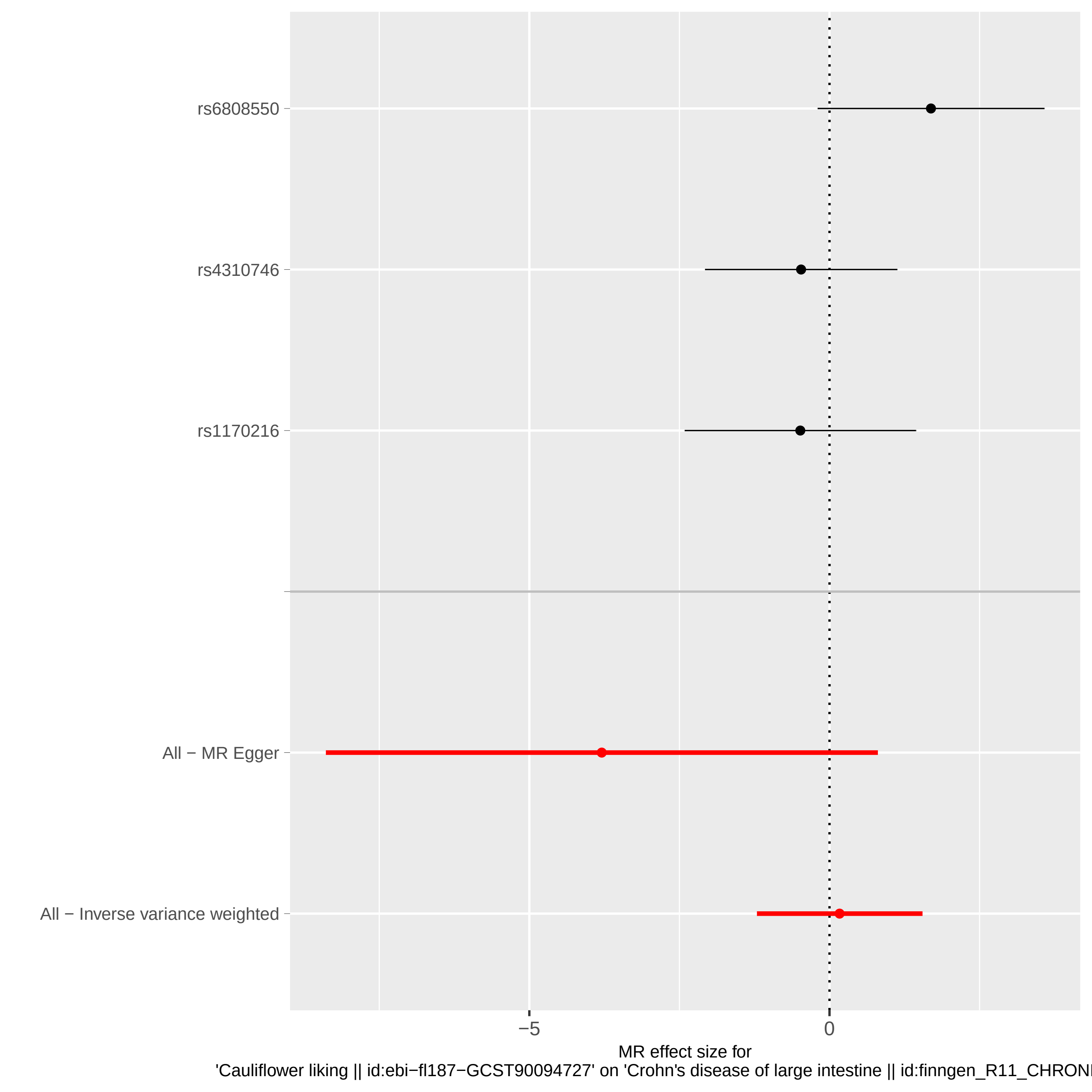
rs6808550
rs4310746
rs1170216
All − MR Egger
All − Inverse variance weighted
−5
0
MR effect size for
'Cauliflower liking || id:ebi−fl187−GCST90094727' on 'Crohn's disease of large intestine || id:finngen_R11_CHRON

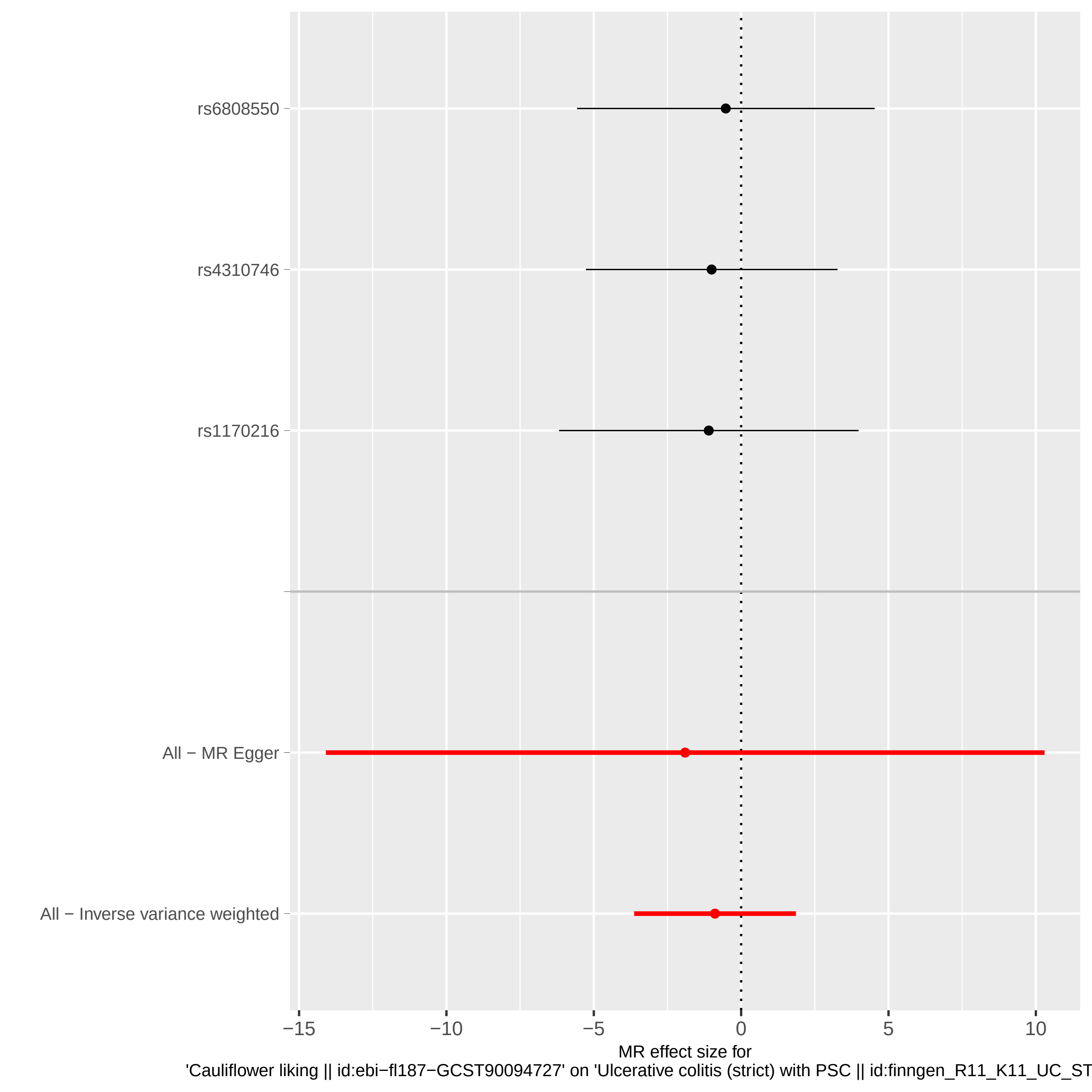
rs6808550
rs4310746
rs1170216
All − MR Egger
All − Inverse variance weighted
−15
−10
−5
0
5
10
MR effect size for
'Cauliflower liking || id:ebi−fl187−GCST90094727' on 'Ulcerative colitis (strict) with PSC || id:finngen_R11_K11_UC_ST

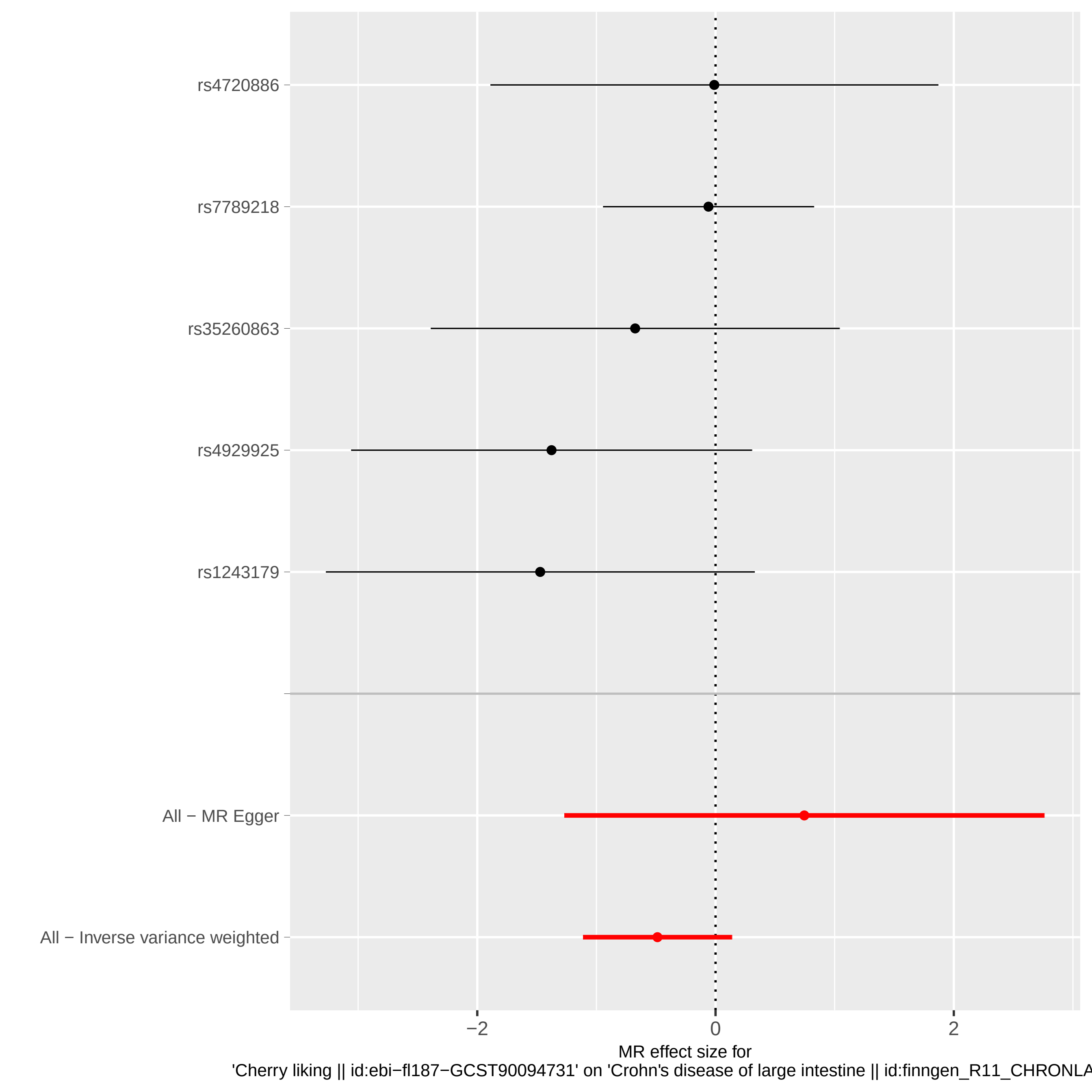
rs4720886
rs7789218
rs35260863
rs4929925
rs1243179
All − MR Egger
All − Inverse variance weighted
−2
0
2
MR effect size for
'Cherry liking || id:ebi−fl187−GCST90094731' on 'Crohn's disease of large intestine || id:finngen_R11_CHRONLA

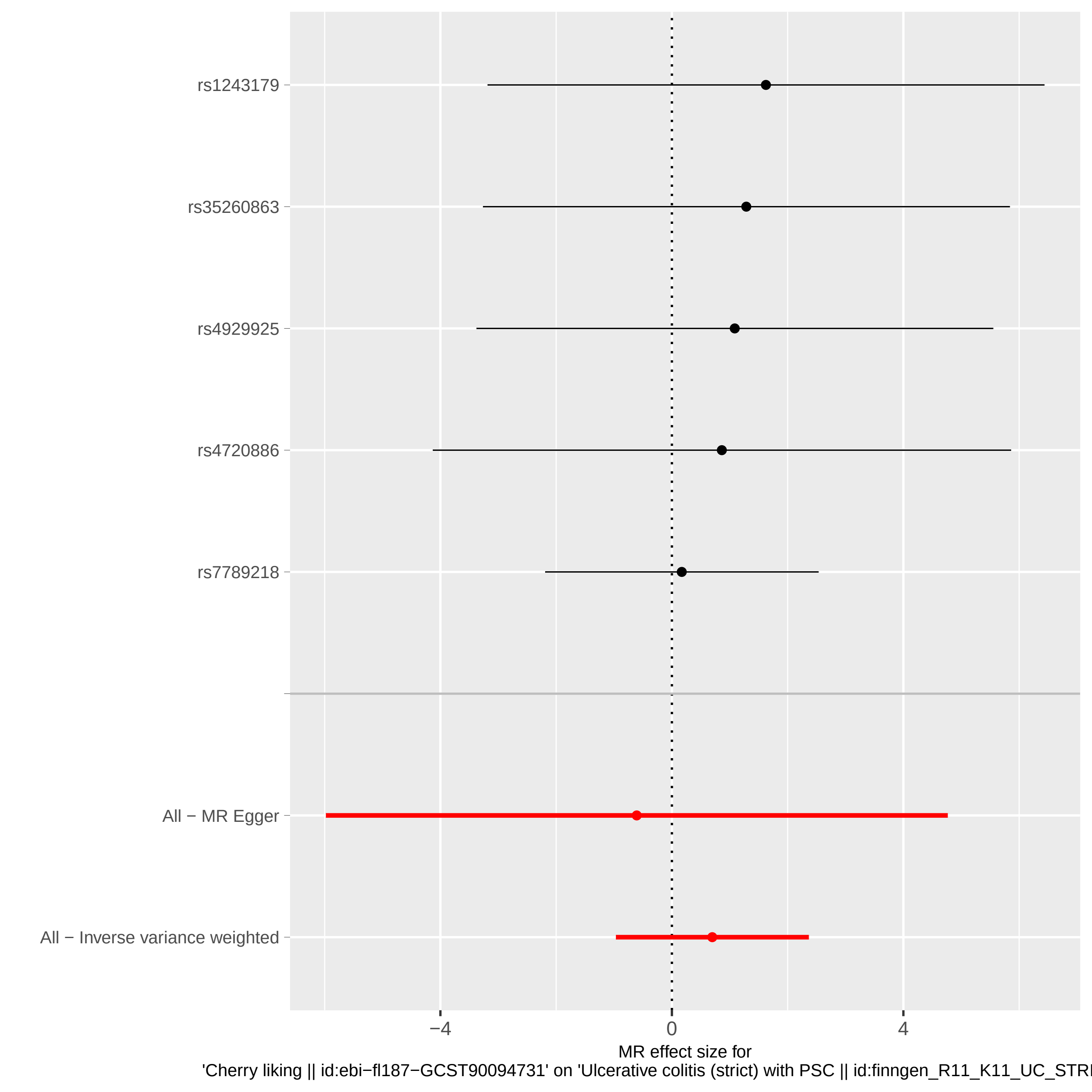

rs1243179
rs35260863
rs4929925
rs4720886
rs7789218
All − MR Egger
All − Inverse variance weighted
−4
0
4
MR effect size for
'Cherry liking || id:ebi−fl187−GCST90094731' on 'Ulcerative colitis (strict) with PSC || id:finngen_R11_K11_UC_STR

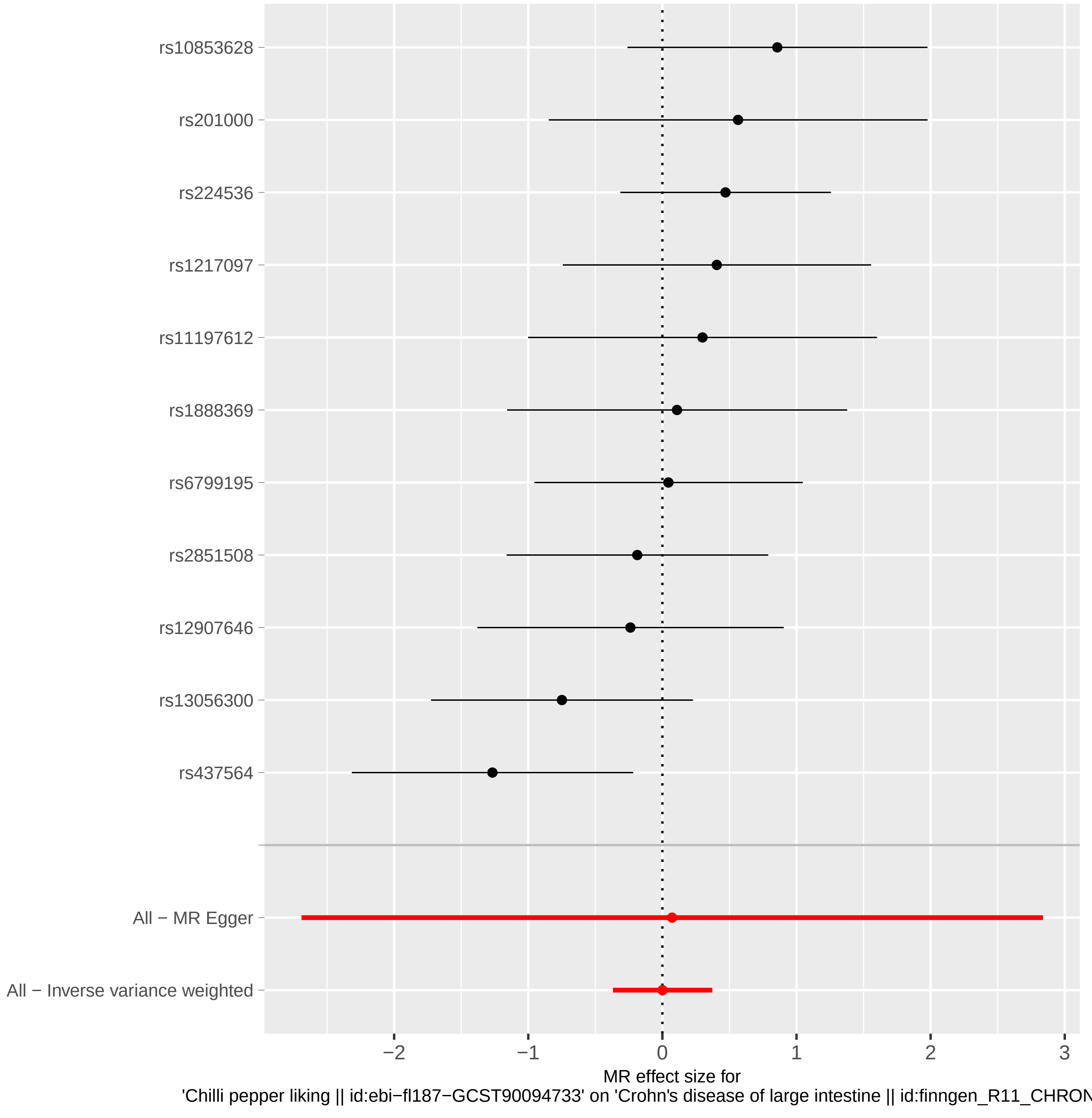
rs10853628
rs201000
rs224536
rs1217097
rs11197612
rs1888369
rs6799195
rs2851508
rs12907646
rs13056300
rs437564
All − MR Egger
All − Inverse variance weighted
−2
−1
0
1
2
3
MR effect size for
'Chilli pepper liking || id:ebi−fl187−GCST90094733' on 'Crohn's disease of large intestine || id:finngen_R11_CHRON

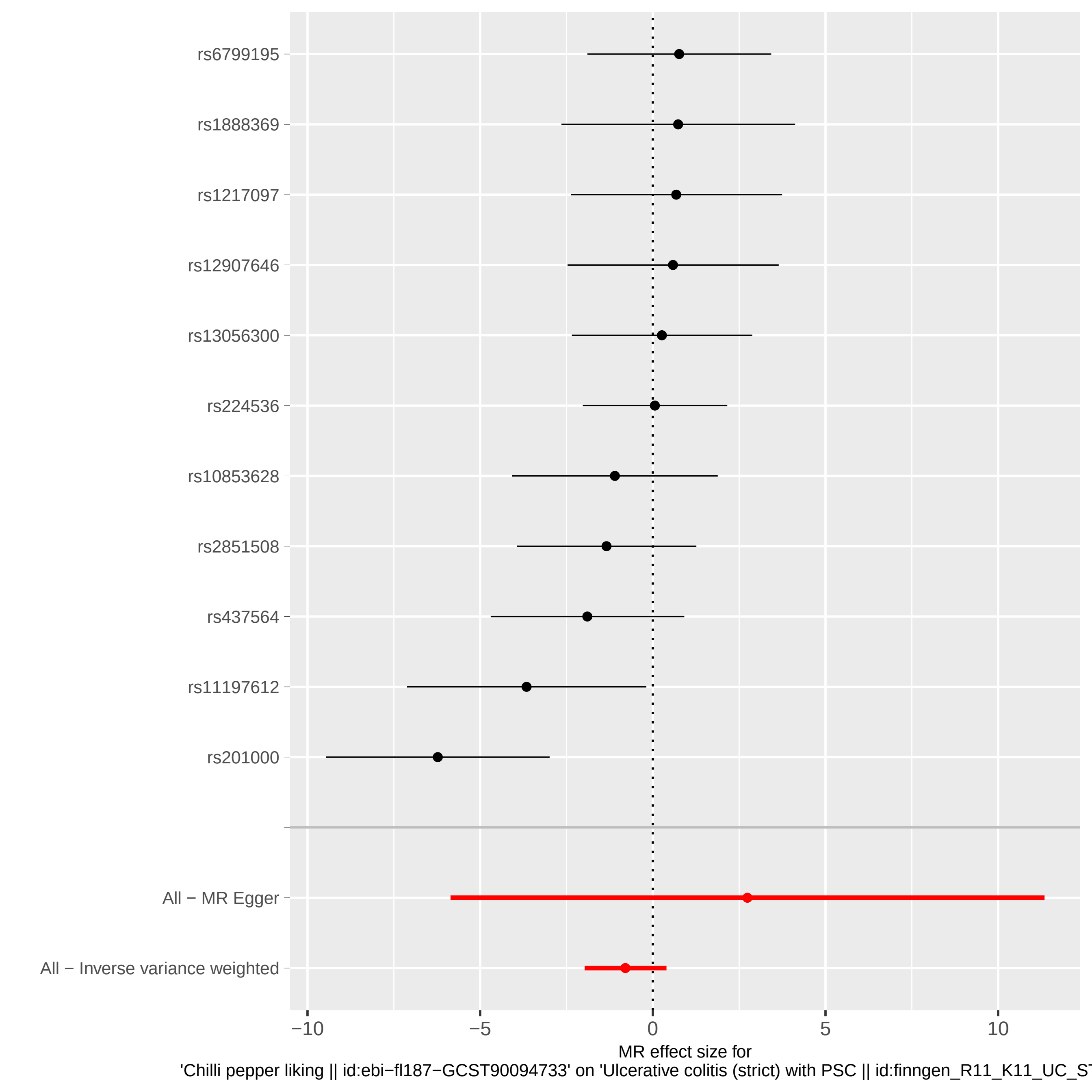
rs6799195
rs1888369
rs1217097
rs12907646
rs13056300
rs224536
rs10853628
rs2851508
rs437564
rs11197612
rs201000
All − MR Egger
All − Inverse variance weighted
−10
−5
0
5
10
MR effect size for
'Chilli pepper liking || id:ebi−fl187−GCST90094733' on 'Ulcerative colitis (strict) with PSC || id:finngen_R11_K11_UC_ST

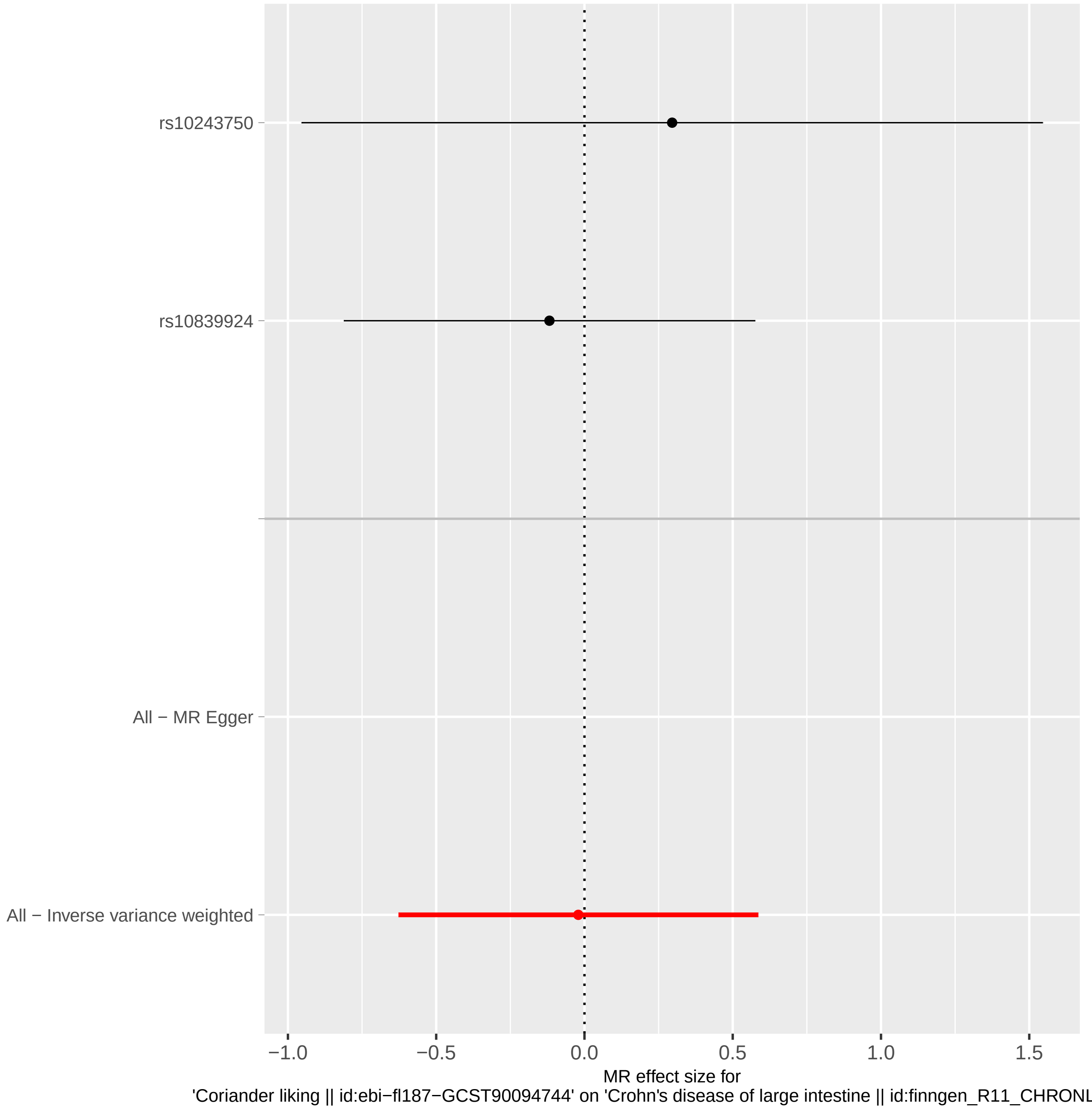
rs10243750
rs10839924
All − MR Egger
All − Inverse variance weighted
−1.0
−0.5
0.0
0.5
1.0
1.5
MR effect size for
'Coriander liking || id:ebi−fl187−GCST90094744' on 'Crohn's disease of large intestine || id:finngen_R11_CHRONL

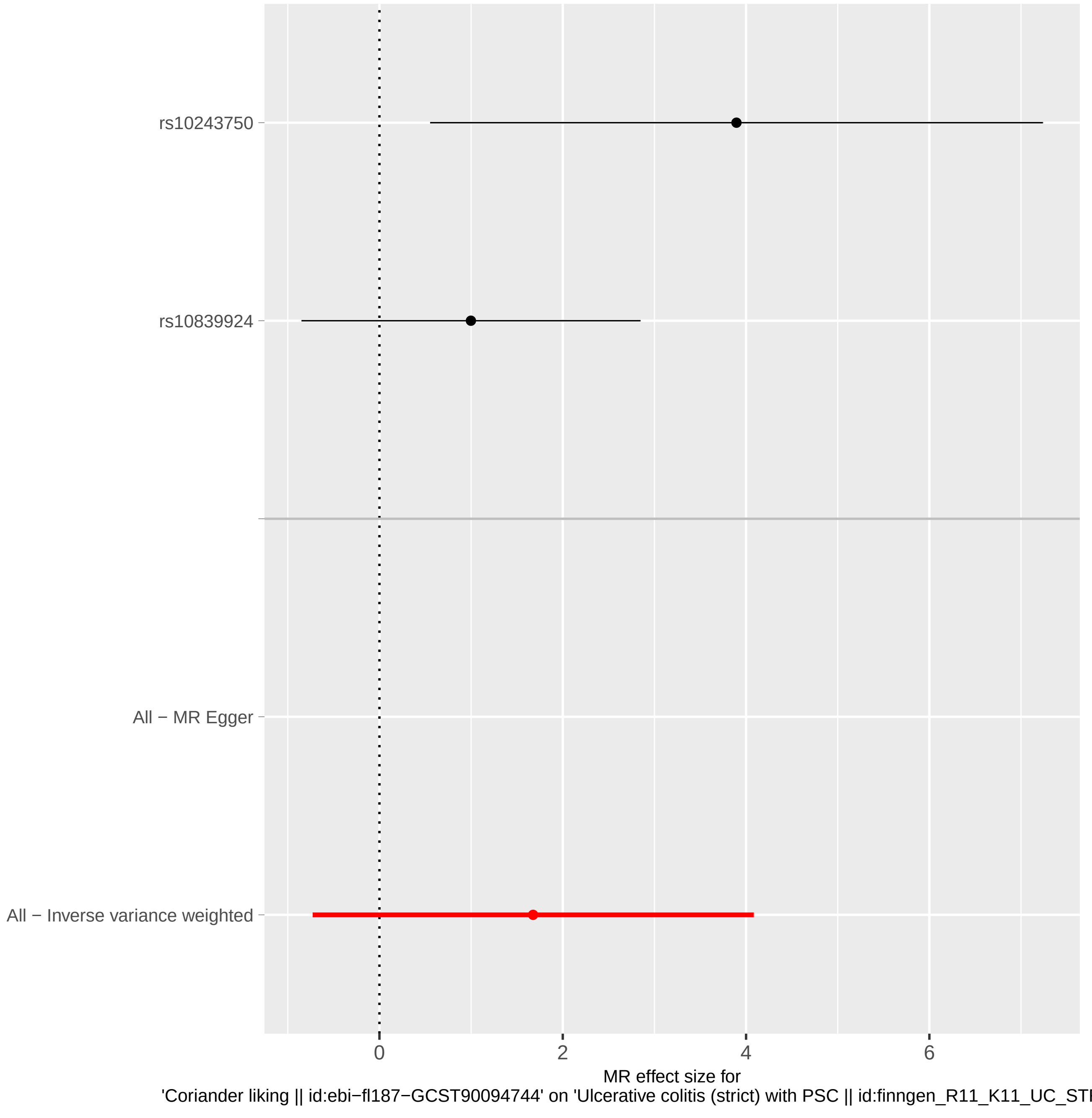
rs10243750
rs10839924
All − MR Egger
All − Inverse variance weighted
0
2
4
6
MR effect size for
'Coriander liking || id:ebi−fl187−GCST90094744' on 'Ulcerative colitis (strict) with PSC || id:finngen_R11_K11_UC_STI

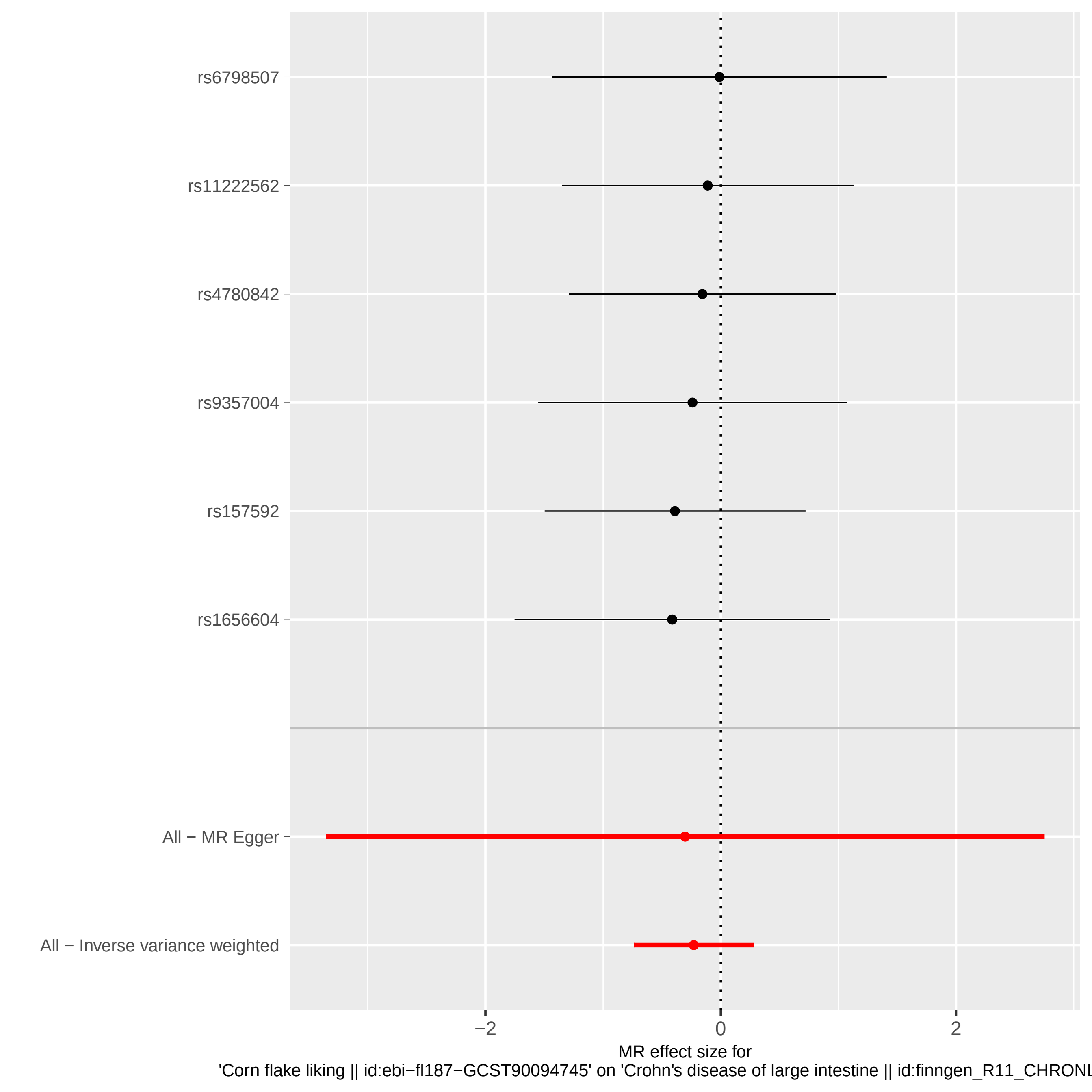
rs6798507
rs11222562
rs4780842
rs9357004
rs157592
rs1656604
All − MR Egger
All − Inverse variance weighted
−2
0
2
MR effect size for
'Corn flake liking || id:ebi−fl187−GCST90094745' on 'Crohn's disease of large intestine || id:finngen_R11_CHRONL

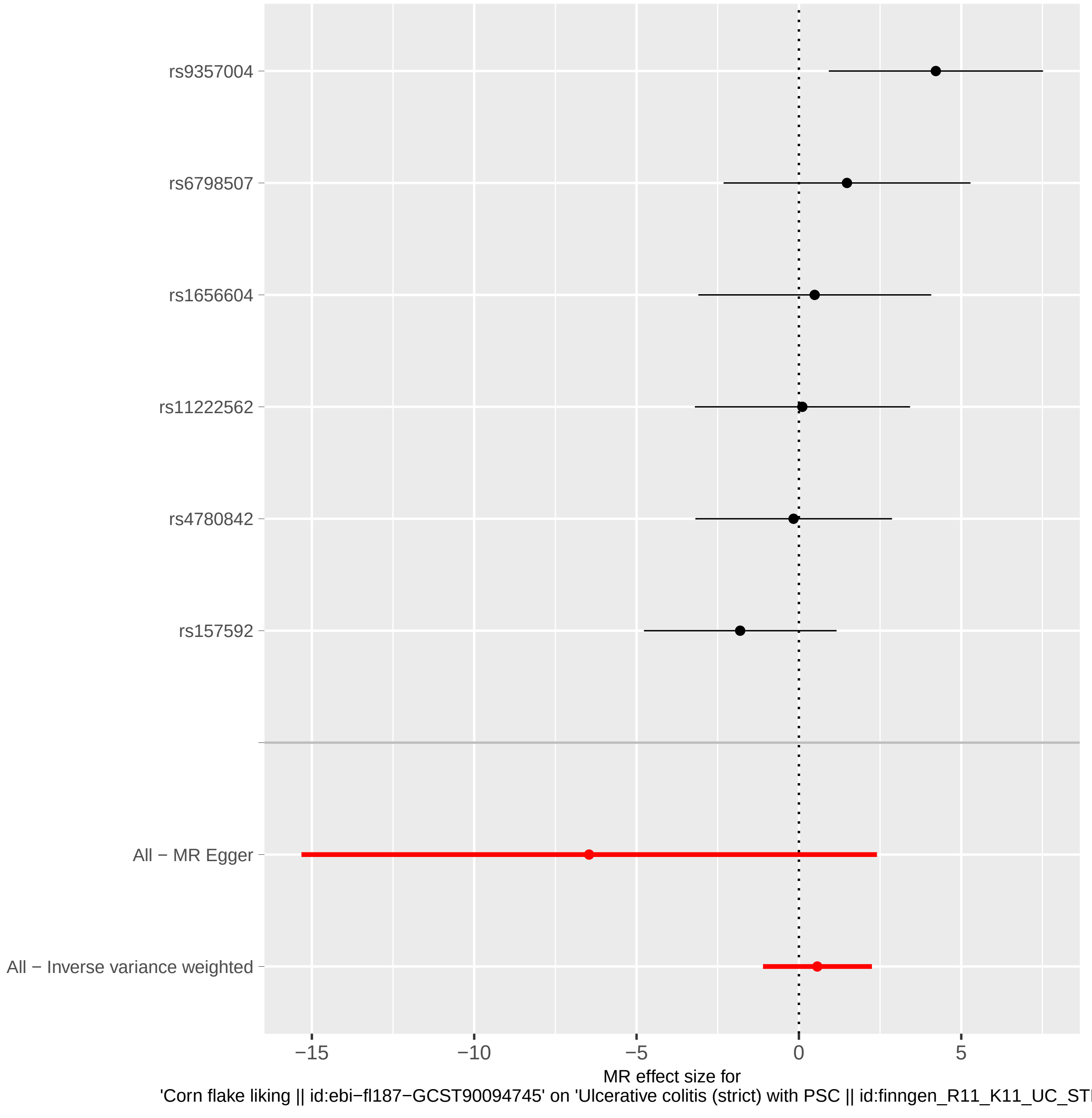

rs9357004
rs6798507
rs1656604
rs11222562
rs4780842
rs157592
All − MR Egger
All − Inverse variance weighted
−15
−10
−5
0
5
MR effect size for
'Corn flake liking || id:ebi−fl187−GCST90094745' on 'Ulcerative colitis (strict) with PSC || id:finngen_R11_K11_UC_ST

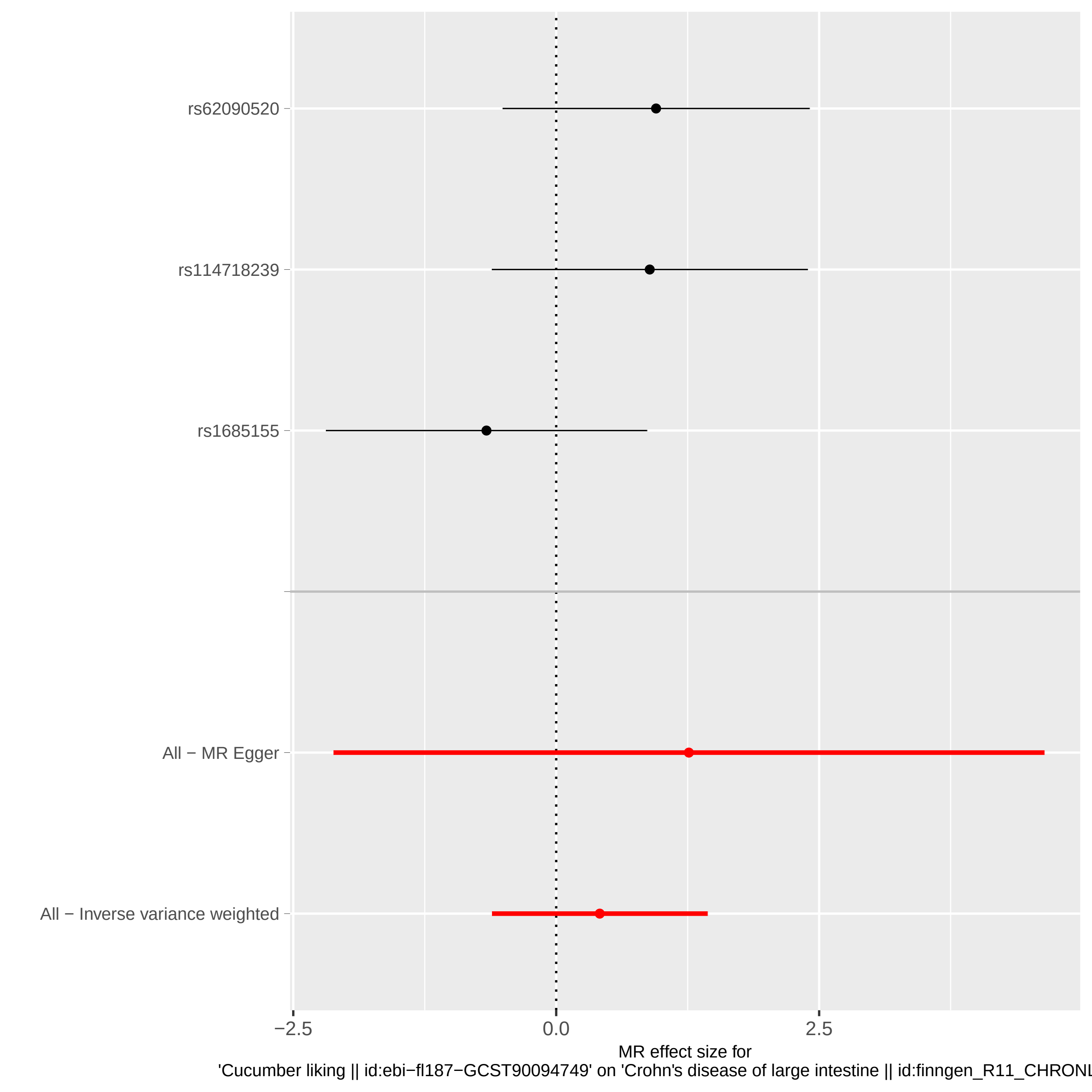
rs62090520
rs114718239
rs1685155
All − MR Egger
All − Inverse variance weighted
−2.5
0.0
2.5
MR effect size for
'Cucumber liking || id:ebi−fl187−GCST90094749' on 'Crohn's disease of large intestine || id:finngen_R11_CHRONL

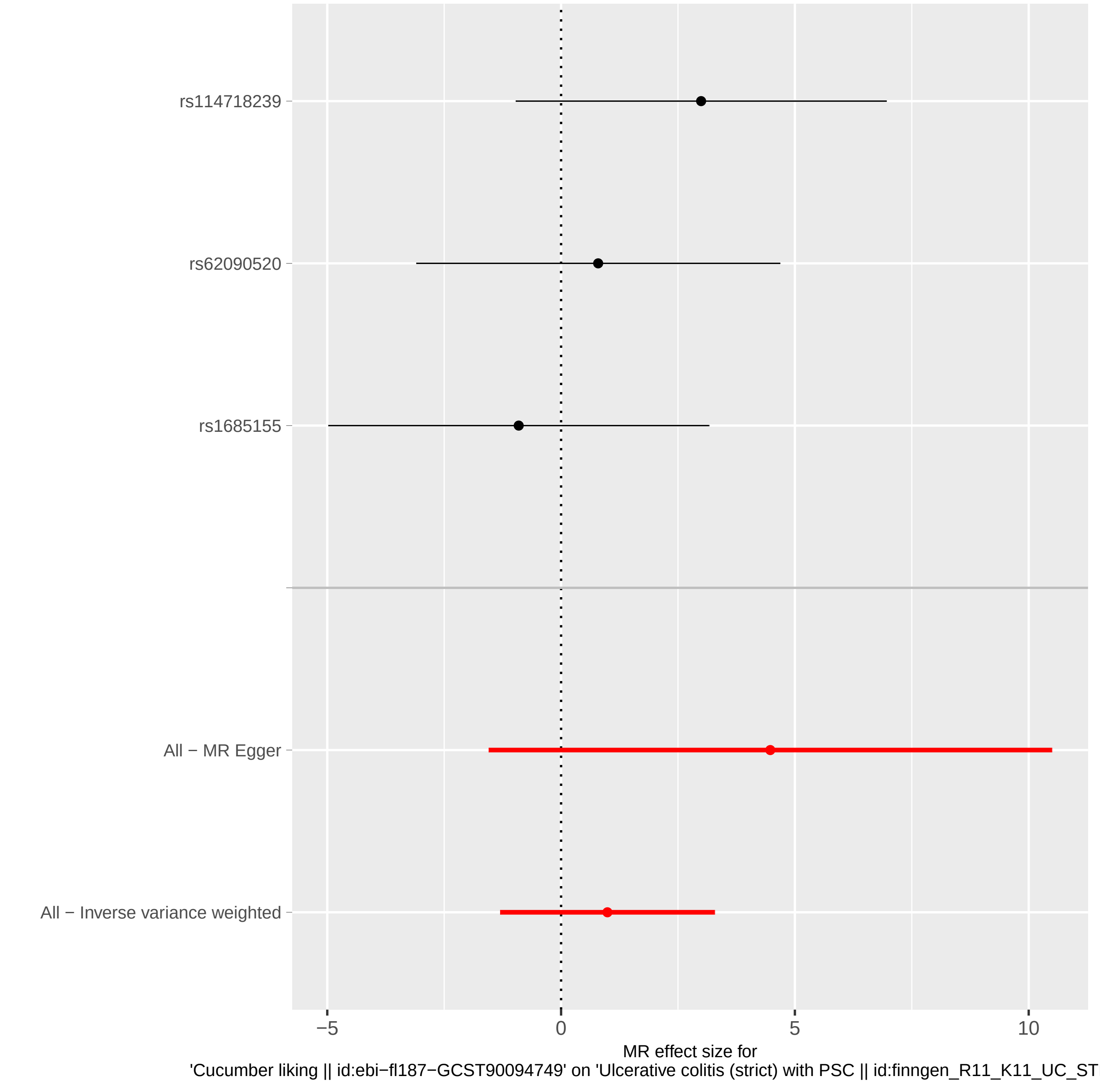
rs114718239
rs62090520
rs1685155
All − MR Egger
All − Inverse variance weighted
−5
0
5
10
MR effect size for
'Cucumber liking || id:ebi−fl187−GCST90094749' on 'Ulcerative colitis (strict) with PSC || id:finngen_R11_K11_UC_ST

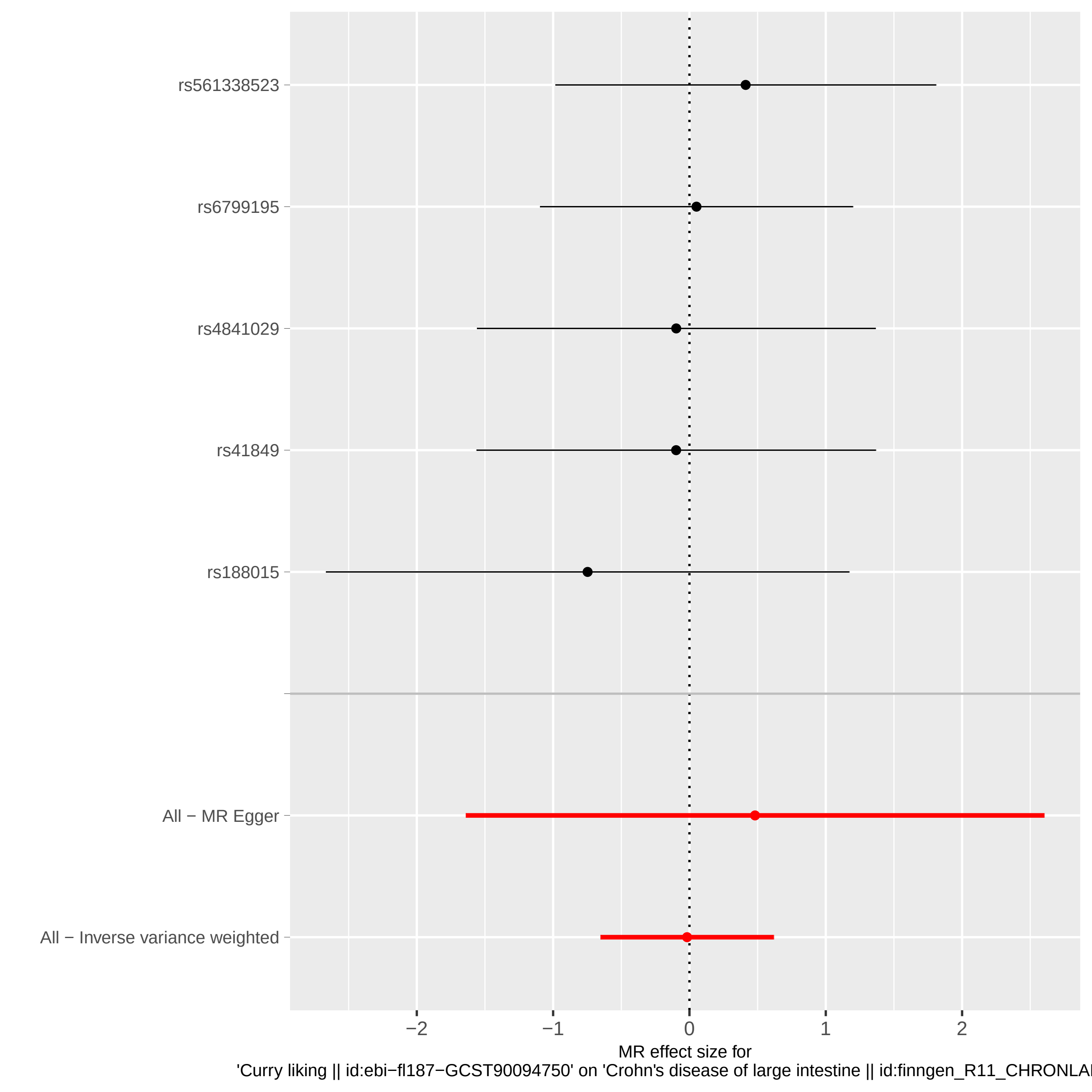

rs561338523
rs6799195
rs4841029
rs41849
rs188015
All − MR Egger
All − Inverse variance weighted
−2
−1
0
1
2
MR effect size for
'Curry liking || id:ebi−fl187−GCST90094750' on 'Crohn's disease of large intestine || id:finngen_R11_CHRONLA

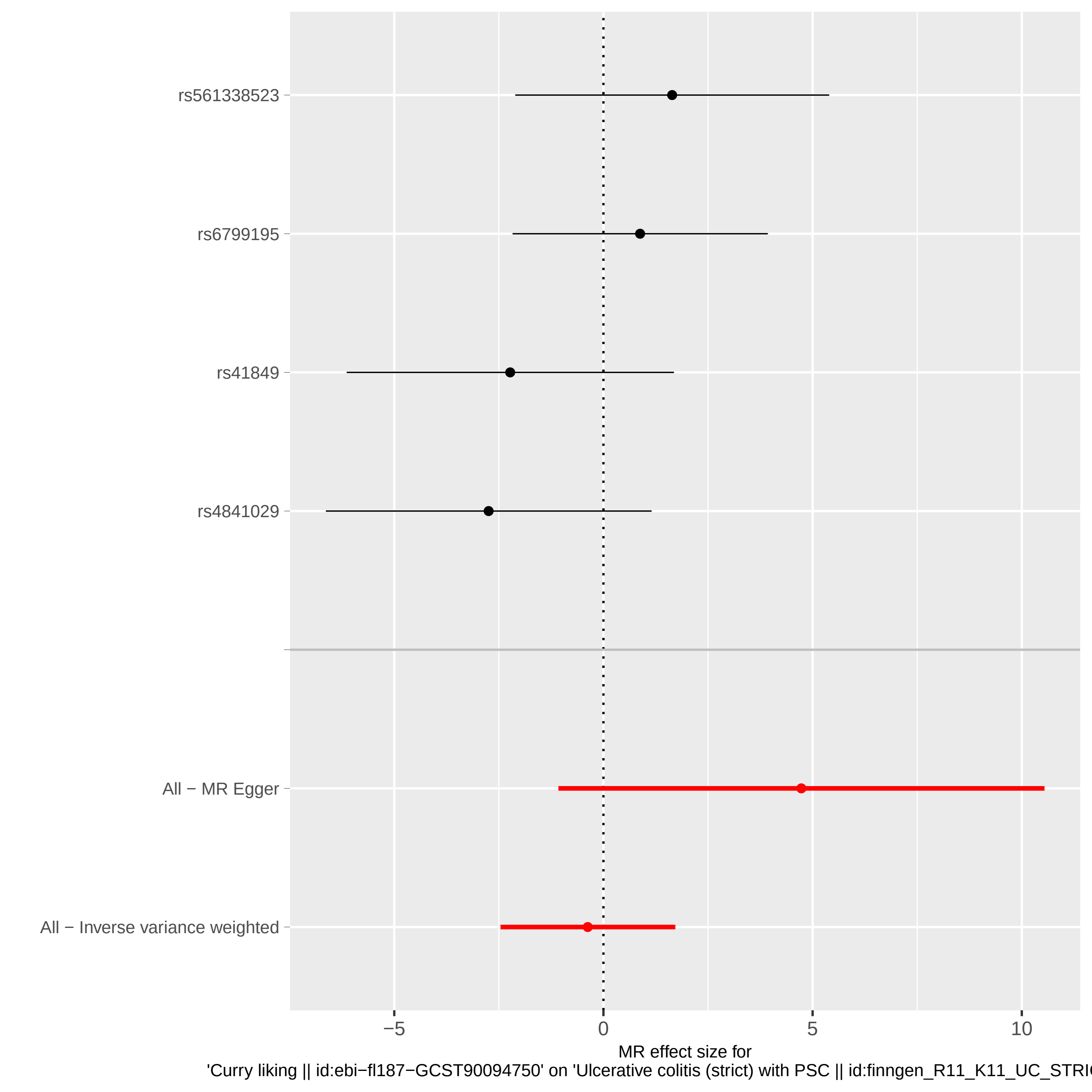
rs561338523
rs6799195
rs41849
rs4841029
All − MR Egger
All − Inverse variance weighted
−5
0
5
10
MR effect size for
'Curry liking || id:ebi−fl187−GCST90094750' on 'Ulcerative colitis (strict) with PSC || id:finngen_R11_K11_UC_STRI

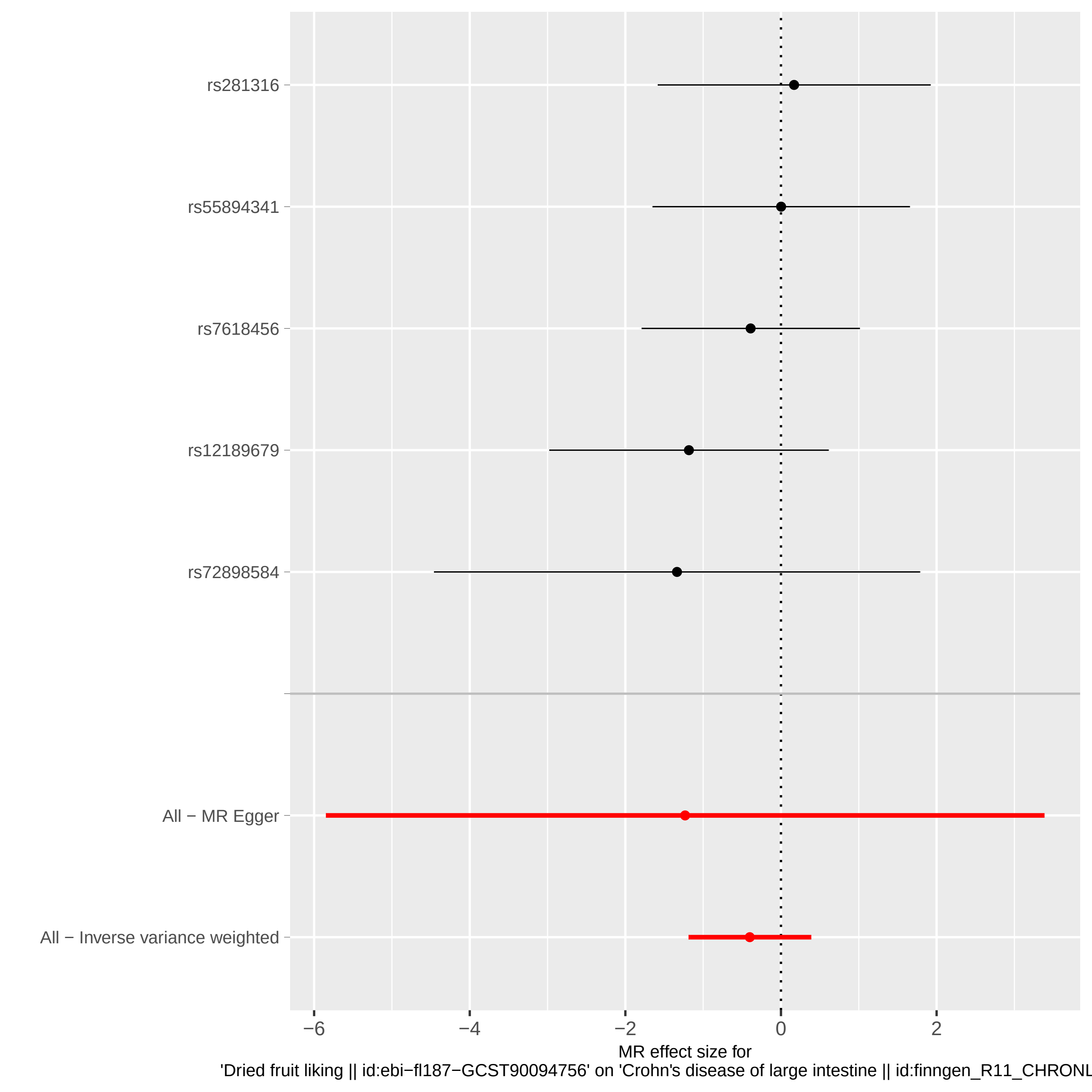

rs281316
rs55894341
rs7618456
rs12189679
rs72898584
All − MR Egger
All − Inverse variance weighted
−6
−4
−2
0
2
MR effect size for
'Dried fruit liking || id:ebi−fl187−GCST90094756' on 'Crohn's disease of large intestine || id:finngen_R11_CHRONL

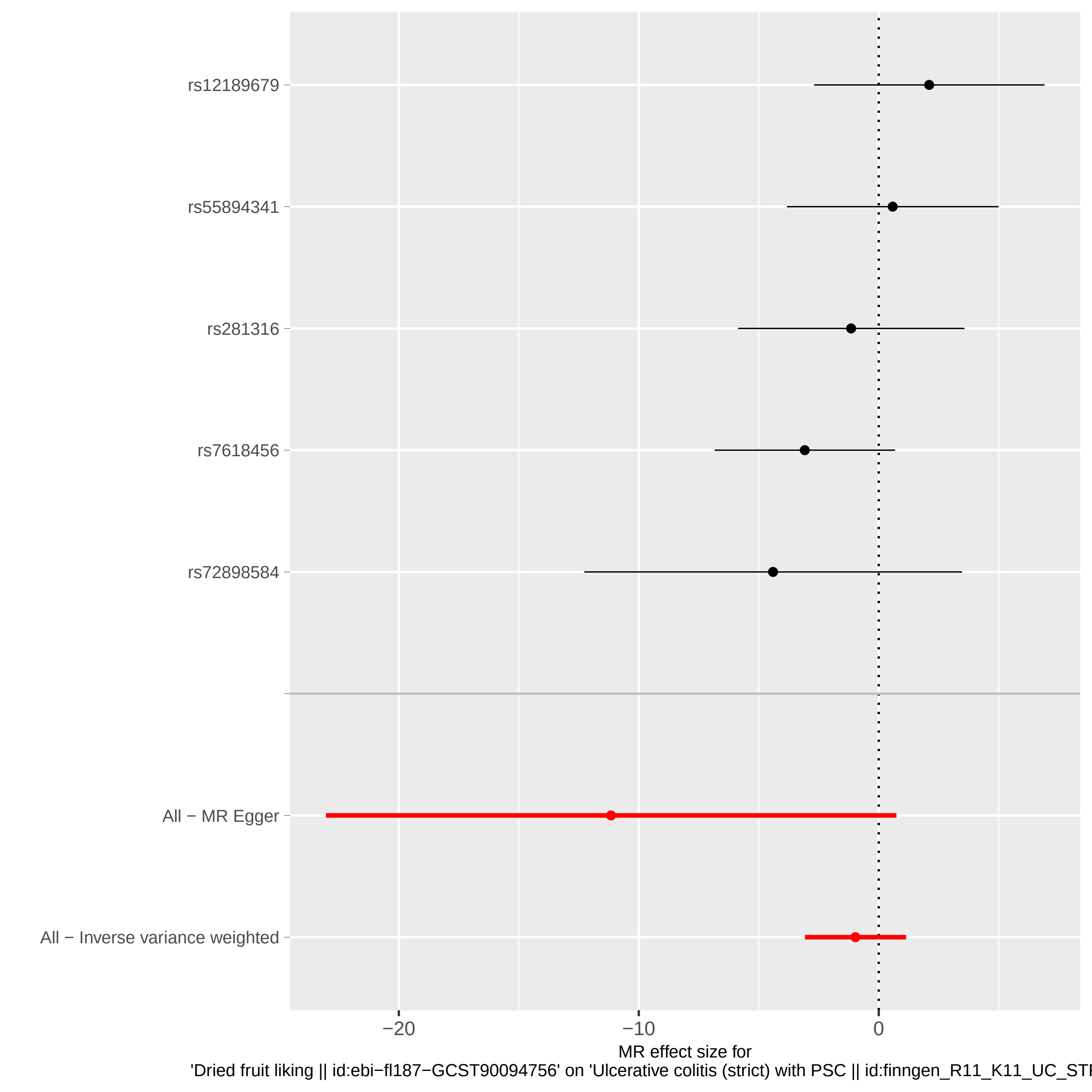
rs12189679
rs55894341
rs281316
rs7618456
rs72898584
All − MR Egger
All − Inverse variance weighted
−20
−10
0
MR effect size for
'Dried fruit liking || id:ebi−fl187−GCST90094756' on 'Ulcerative colitis (strict) with PSC || id:finngen_R11_K11_UC_STI

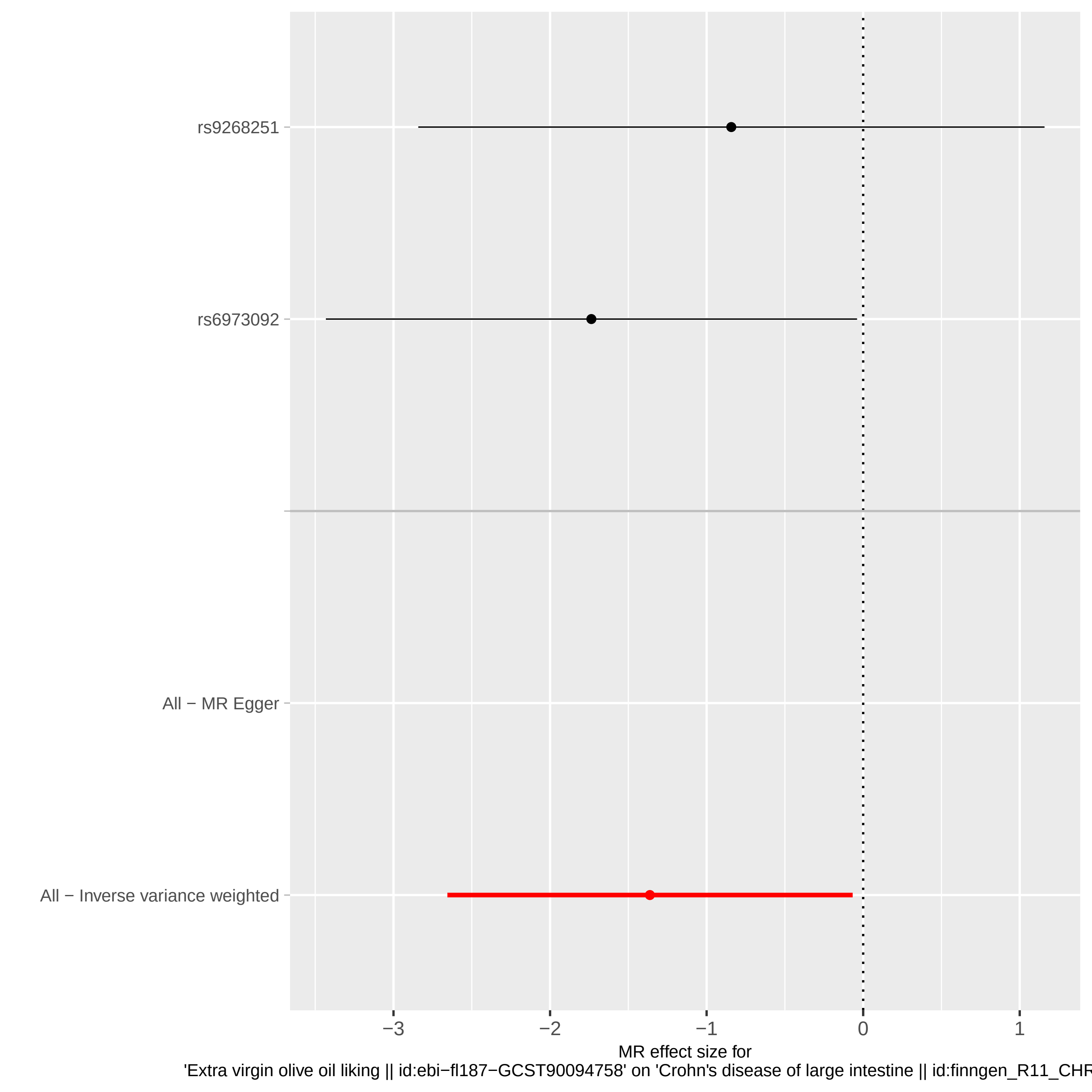
rs9268251
rs6973092
All − MR Egger
All − Inverse variance weighted
−3
−2
−1
0
1
MR effect size for
'Extra virgin olive oil liking || id:ebi−fl187−GCST90094758' on 'Crohn's disease of large intestine || id:finngen_R11_CHR

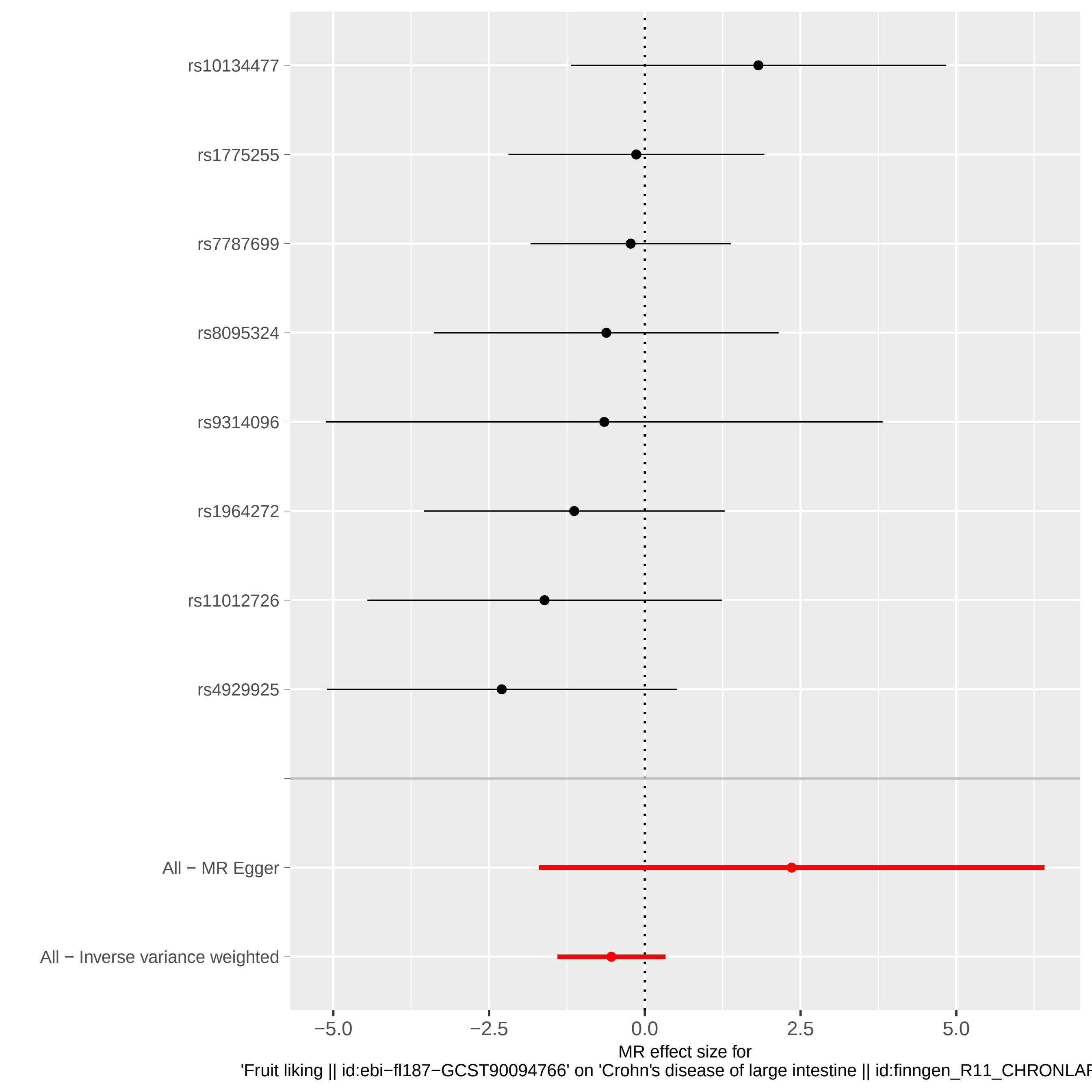
rs10134477
rs1775255
rs7787699
rs8095324
rs9314096
rs1964272
rs11012726
rs4929925
All − MR Egger
All − Inverse variance weighted
−5.0
−2.5
0.0
2.5
5.0
MR effect size for
'Fruit liking || id:ebi−fl187−GCST90094766' on 'Crohn's disease of large intestine || id:finngen_R11_CHRONLAR

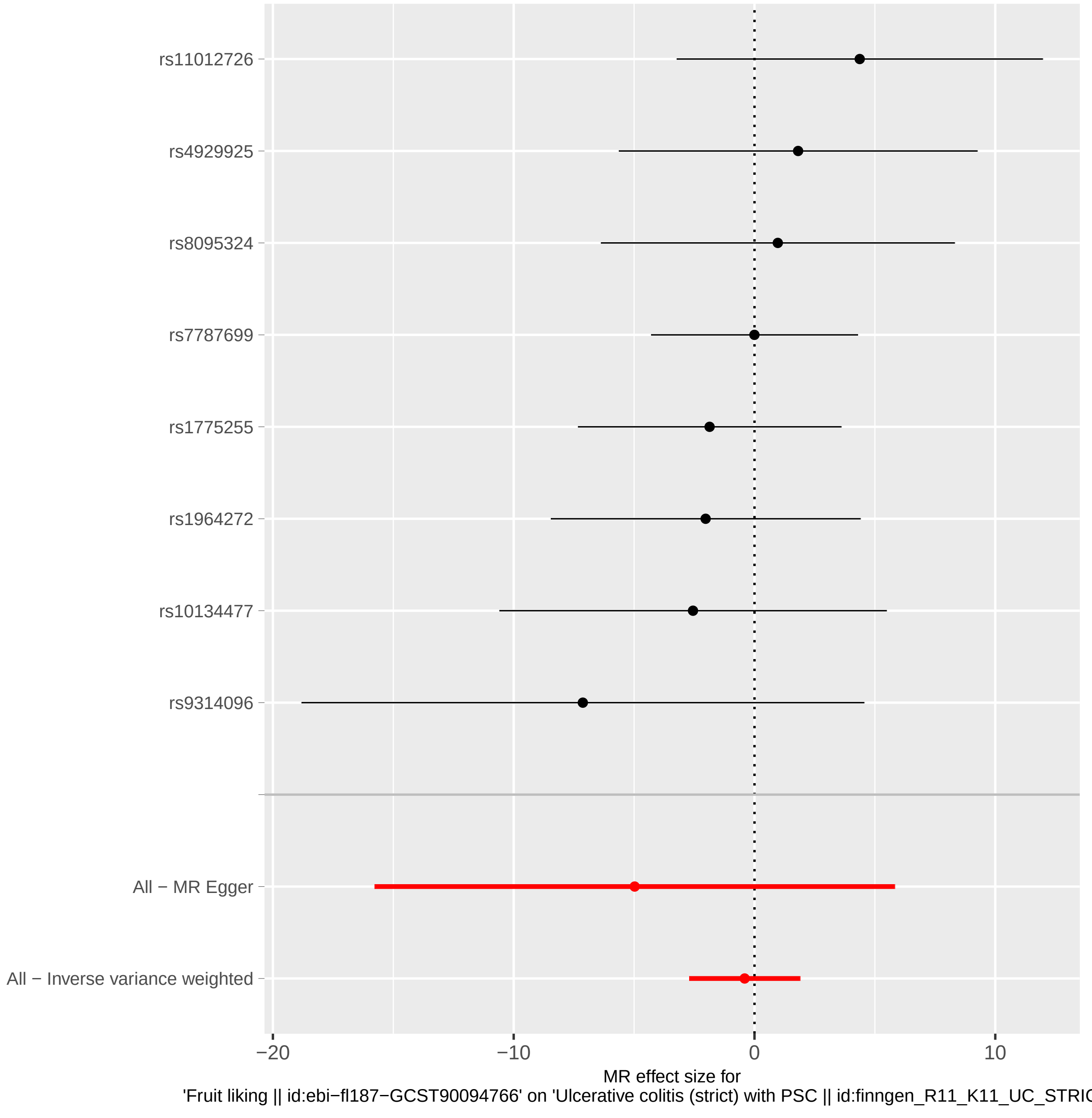
rs11012726
rs4929925
rs8095324
rs7787699
rs1775255
rs1964272
rs10134477
rs9314096
All − MR Egger
All − Inverse variance weighted
−20
−10
0
10
MR effect size for
'Fruit liking || id:ebi−fl187−GCST90094766' on 'Ulcerative colitis (strict) with PSC || id:finngen_R11_K11_UC_STRIC

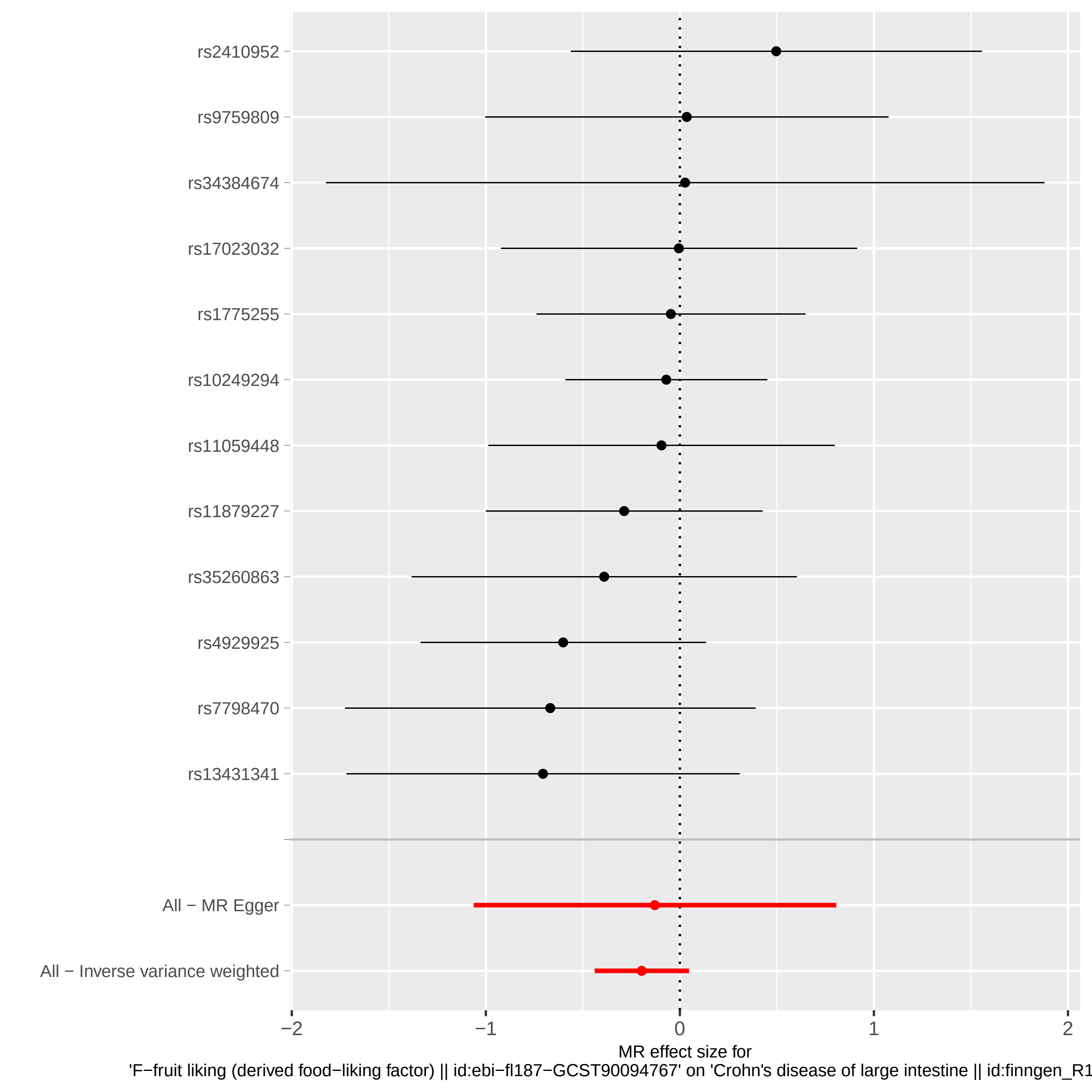
rs2410952
rs9759809
rs34384674
rs17023032
rs1775255
rs10249294
rs11059448
rs11879227
rs35260863
rs4929925
rs7798470
rs13431341
All − MR Egger
All − Inverse variance weighted
−2
−1
0
1
2
MR effect size for
'F−fruit liking (derived food−liking factor) || id:ebi−fl187−GCST90094767' on 'Crohn's disease of large intestine || id:finngen_R1

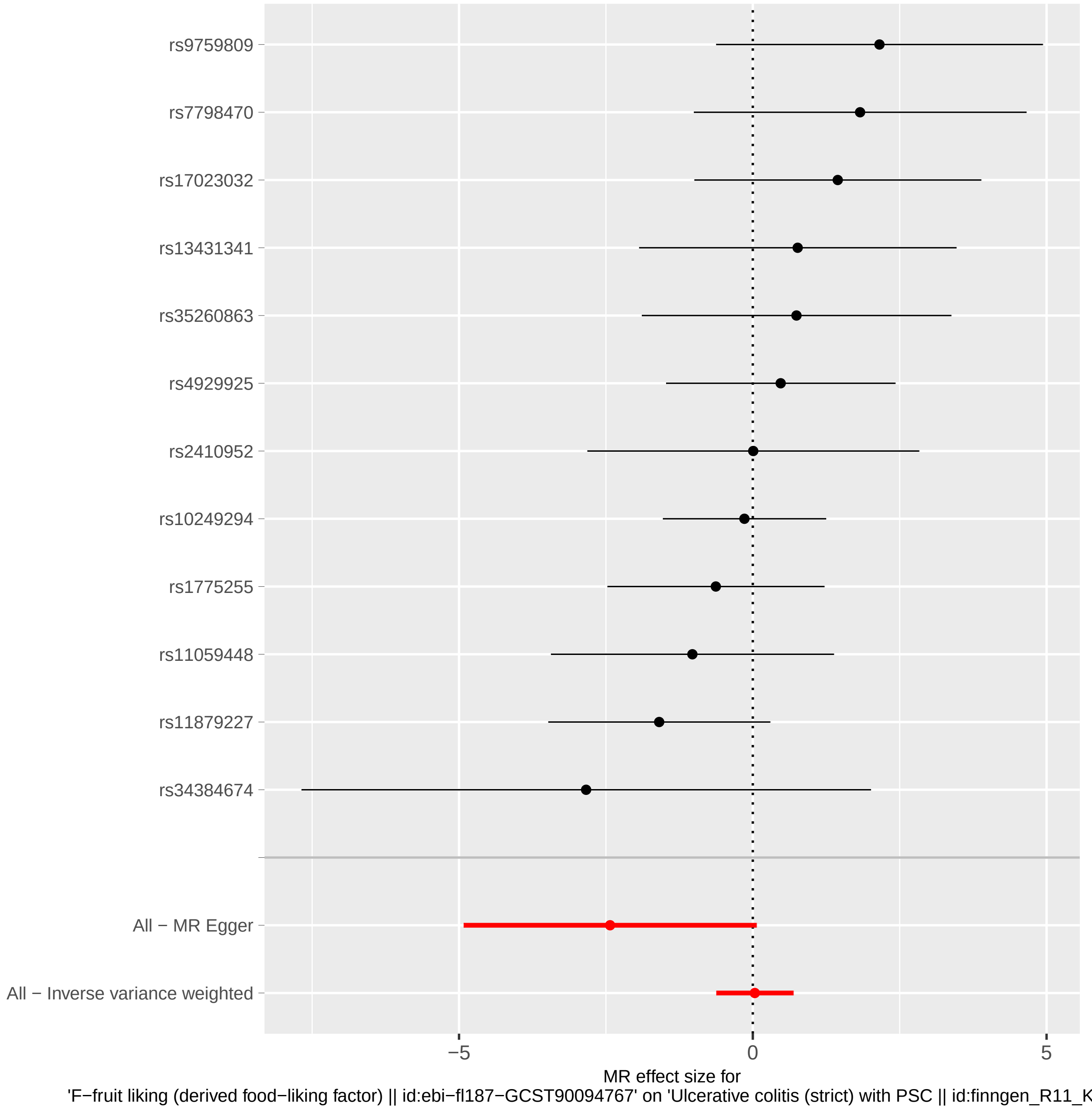
rs9759809
rs7798470
rs17023032
rs13431341
rs35260863
rs4929925
rs2410952
rs10249294
rs1775255
rs11059448
rs11879227
rs34384674
All − MR Egger
All − Inverse variance weighted
−5
0
5
MR effect size for
'F−fruit liking (derived food−liking factor) || id:ebi−fl187−GCST90094767' on 'Ulcerative colitis (strict) with PSC || id:finngen_R11_K

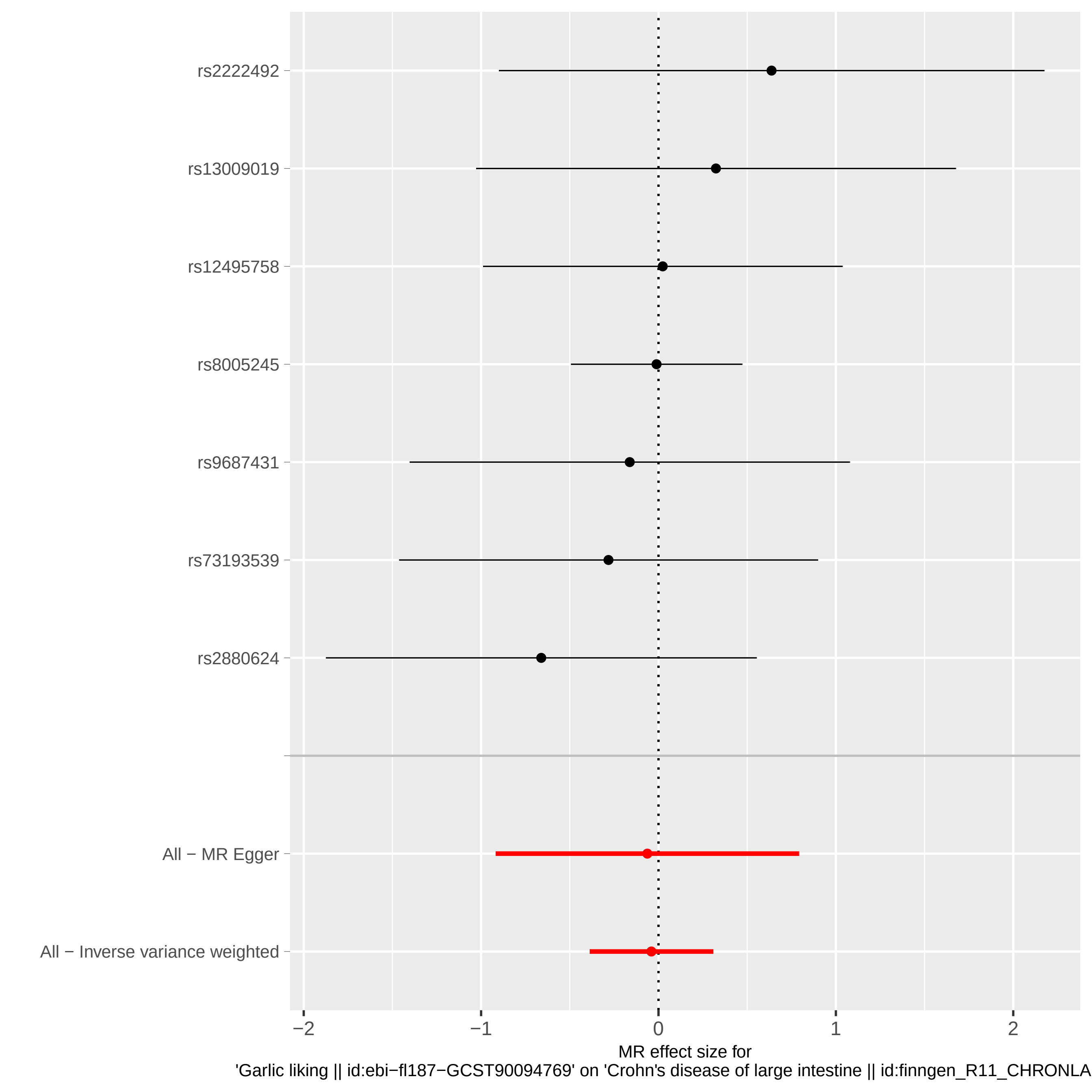
rs2222492
rs13009019
rs12495758
rs8005245
rs9687431
rs73193539
rs2880624
All − MR Egger
All − Inverse variance weighted
−2
−1
0
1
2
MR effect size for
'Garlic liking || id:ebi−fl187−GCST90094769' on 'Crohn's disease of large intestine || id:finngen_R11_CHRONLA

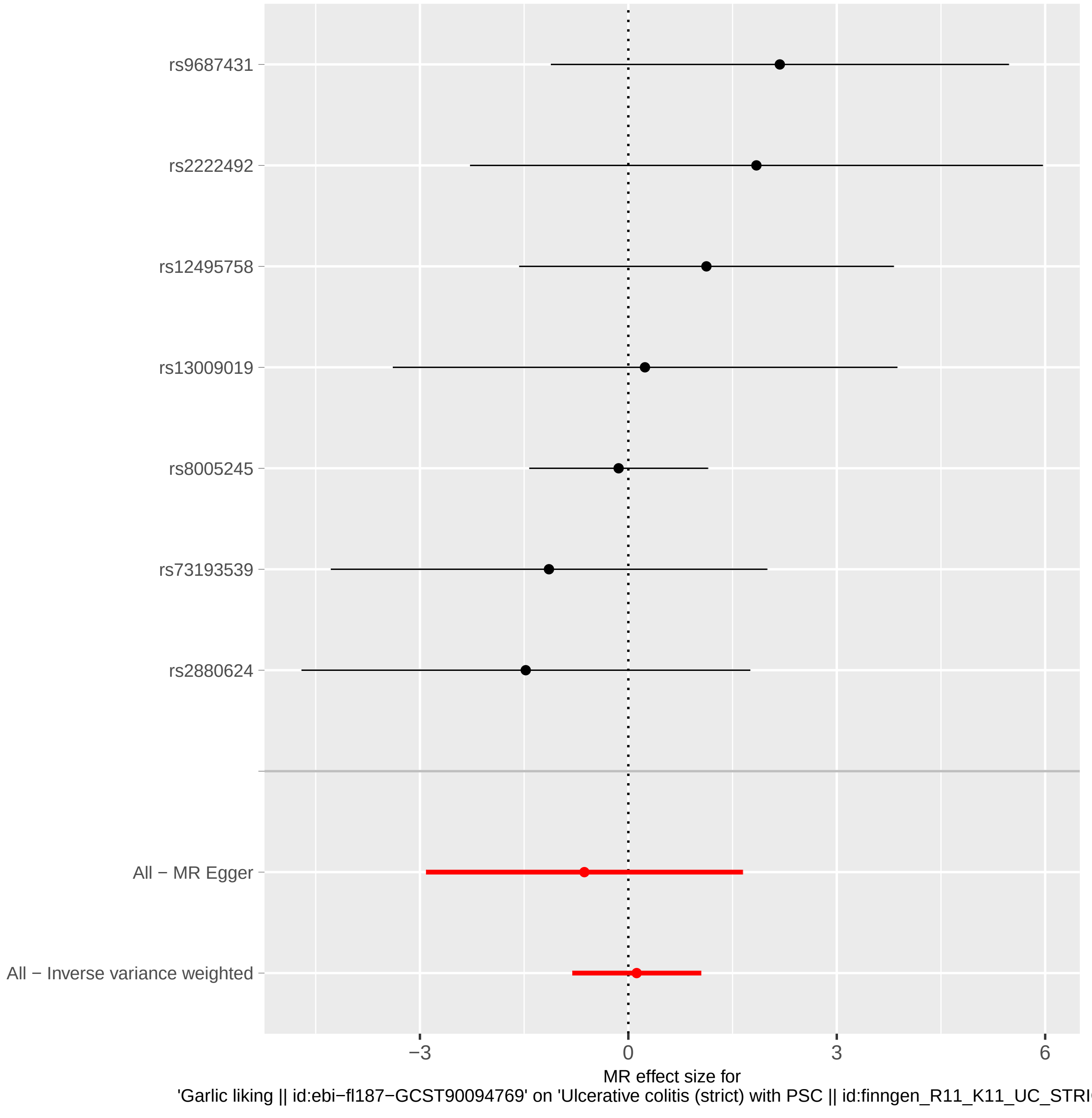
rs9687431
rs2222492
rs12495758
rs13009019
rs8005245
rs73193539
rs2880624
All − MR Egger
All − Inverse variance weighted
−3
0
3
6
MR effect size for
'Garlic liking || id:ebi−fl187−GCST90094769' on 'Ulcerative colitis (strict) with PSC || id:finngen_R11_K11_UC_STRI

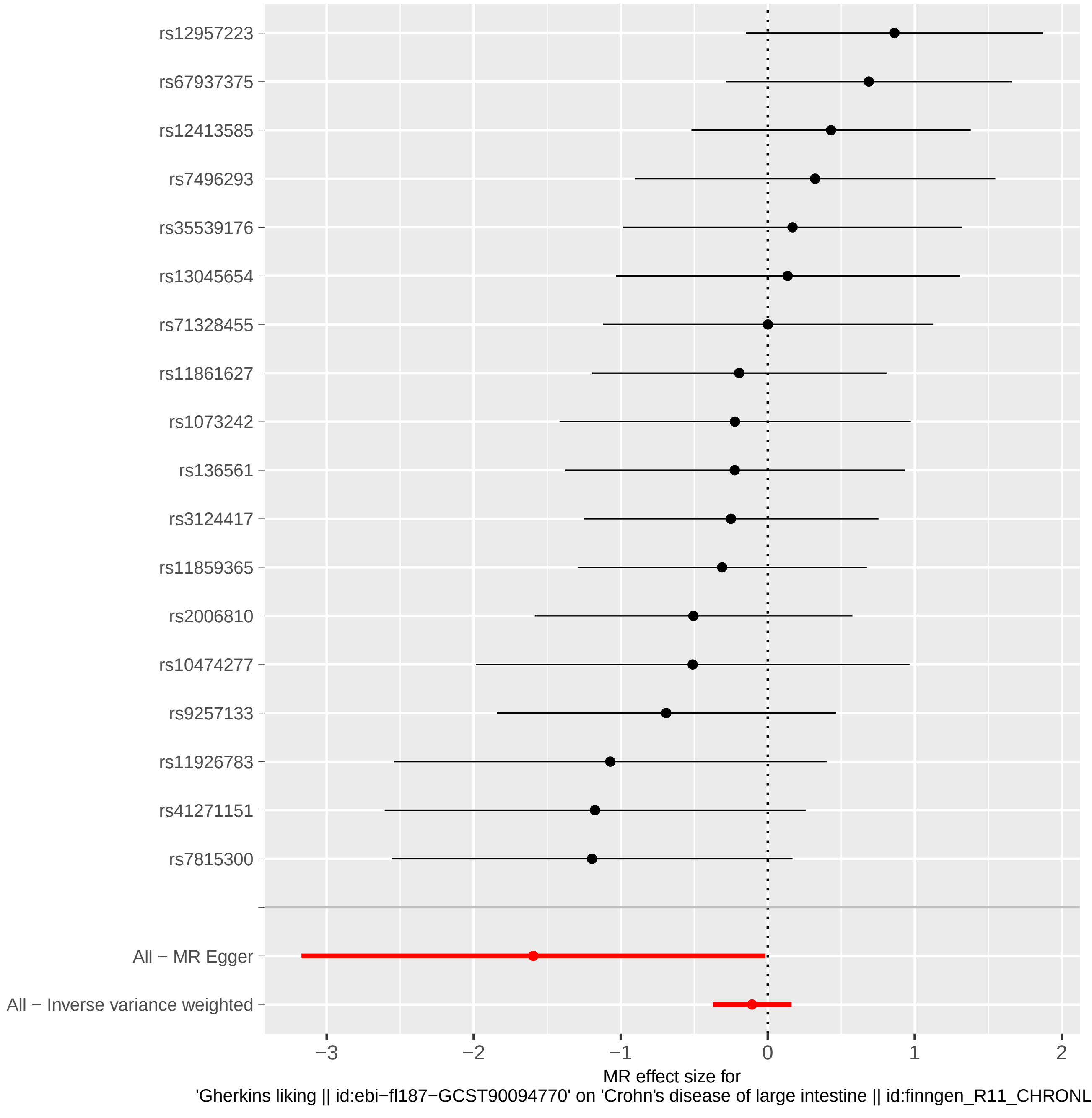
rs12957223
rs67937375
rs12413585
rs7496293
rs35539176
rs13045654
rs71328455
rs11861627
rs1073242
rs136561
rs3124417
rs11859365
rs2006810
rs10474277
rs9257133
rs11926783
rs41271151
rs7815300
All − MR Egger
All − Inverse variance weighted
−3
−2
−1
0
1
2
MR effect size for
'Gherkins liking || id:ebi−fl187−GCST90094770' on 'Crohn's disease of large intestine || id:finngen_R11_CHRONL

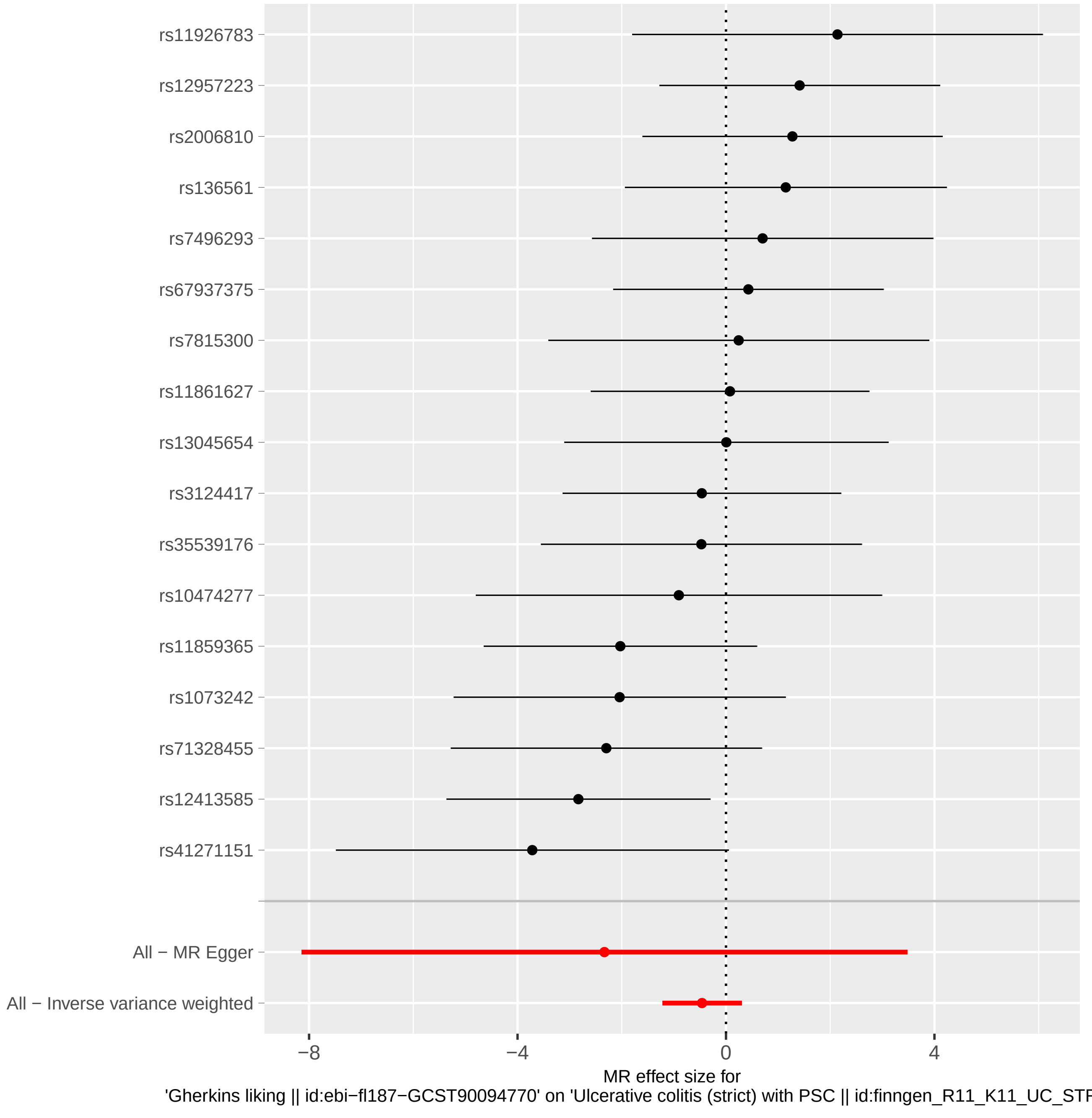

rs11926783
rs12957223
rs2006810
rs136561
rs7496293
rs67937375
rs7815300
rs11861627
rs13045654
rs3124417
rs35539176
rs10474277
rs11859365
rs1073242
rs71328455
rs12413585
rs41271151
All − MR Egger
All − Inverse variance weighted
−8
−4
0
4
MR effect size for
'Gherkins liking || id:ebi−fl187−GCST90094770' on 'Ulcerative colitis (strict) with PSC || id:finngen_R11_K11_UC_STR

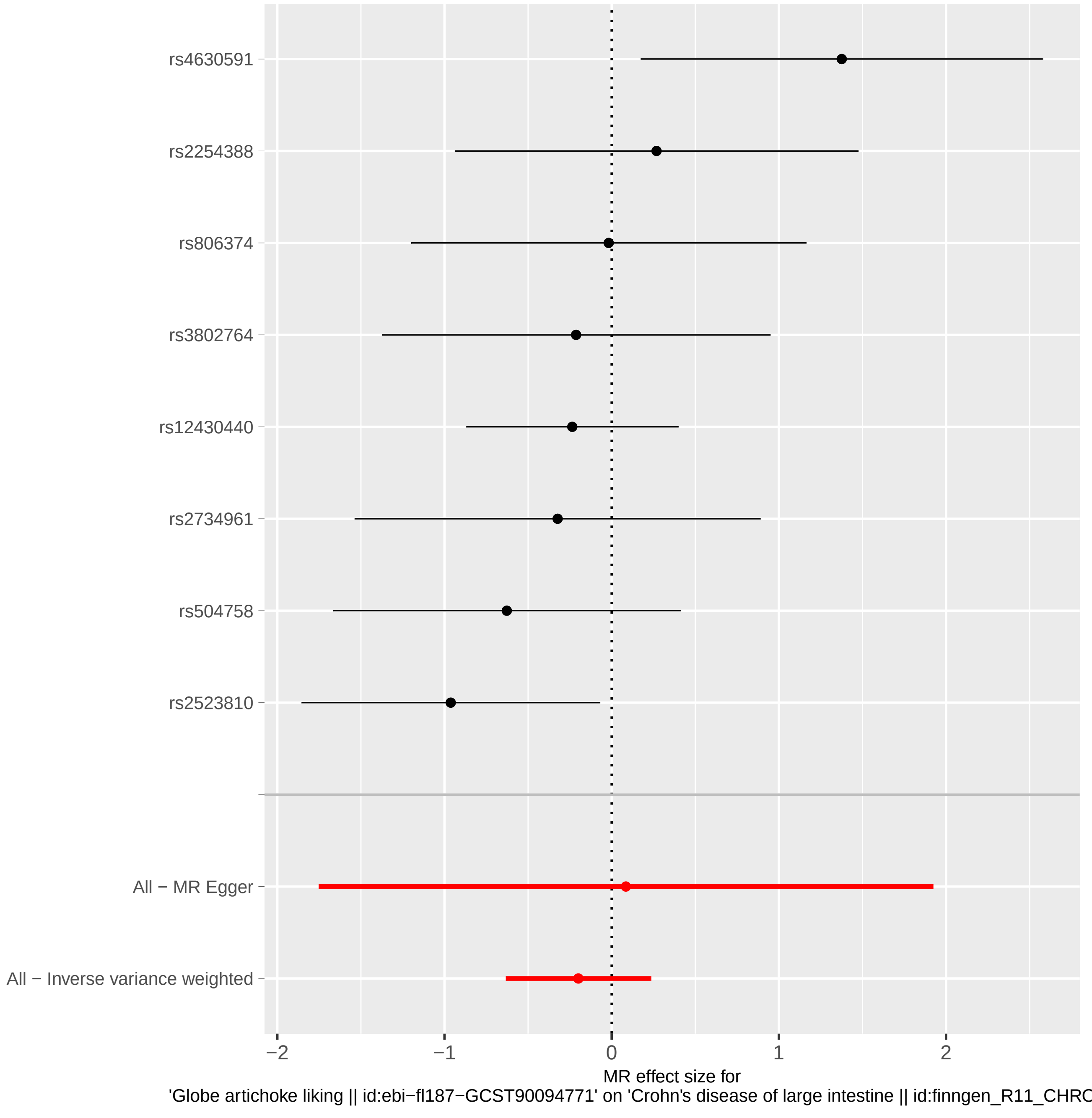

rs4630591
rs2254388
rs806374
rs3802764
rs12430440
rs2734961
rs504758
rs2523810
All − MR Egger
All − Inverse variance weighted
−2
−1
0
1
2
MR effect size for
'Globe artichoke liking || id:ebi−fl187−GCST90094771' on 'Crohn's disease of large intestine || id:finngen_R11_CHRO

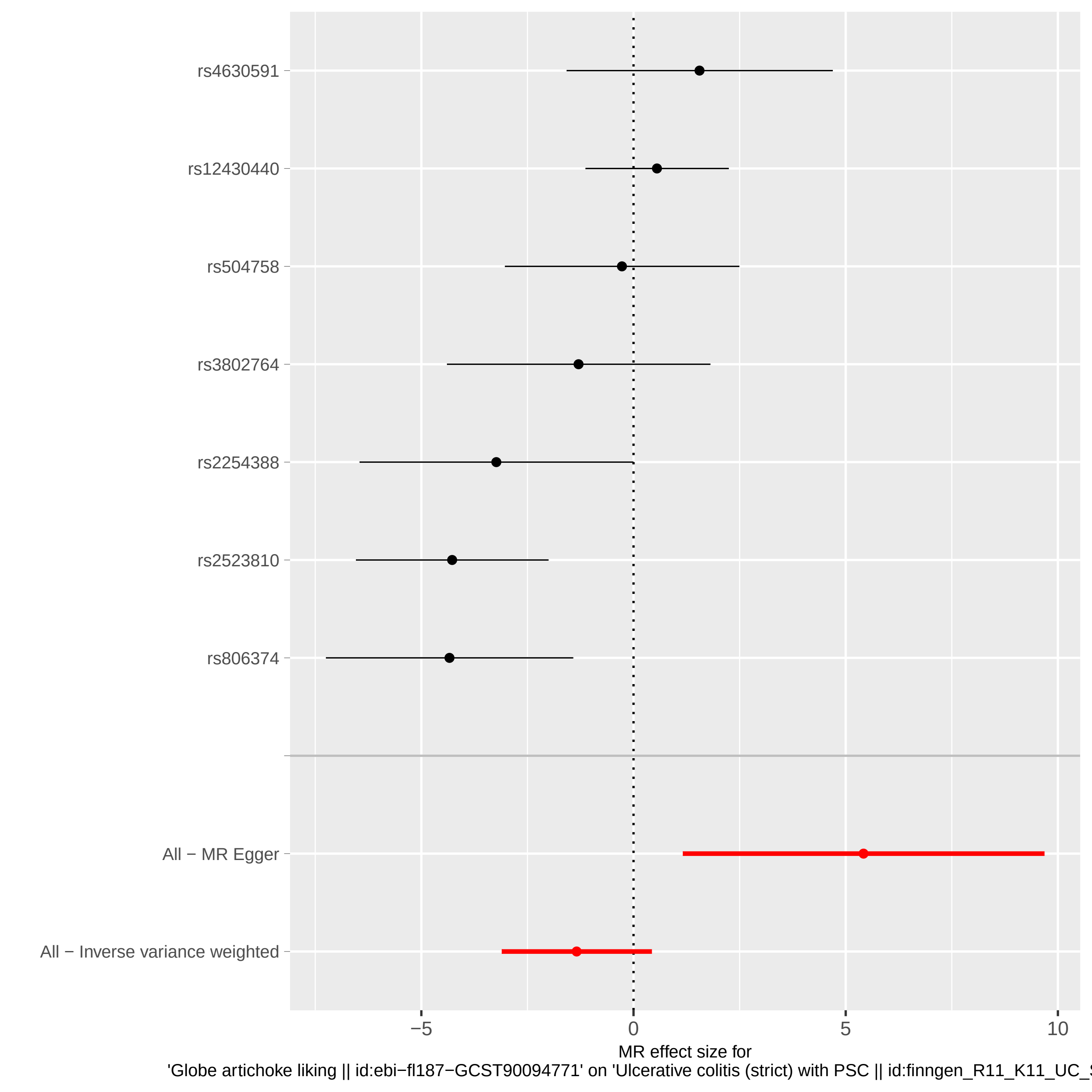
rs4630591
rs12430440
rs504758
rs3802764
rs2254388
rs2523810
rs806374
All − MR Egger
All − Inverse variance weighted
−5
0
5
10
MR effect size for
'Globe artichoke liking || id:ebi−fl187−GCST90094771' on 'Ulcerative colitis (strict) with PSC || id:finngen_R11_K11_UC_S

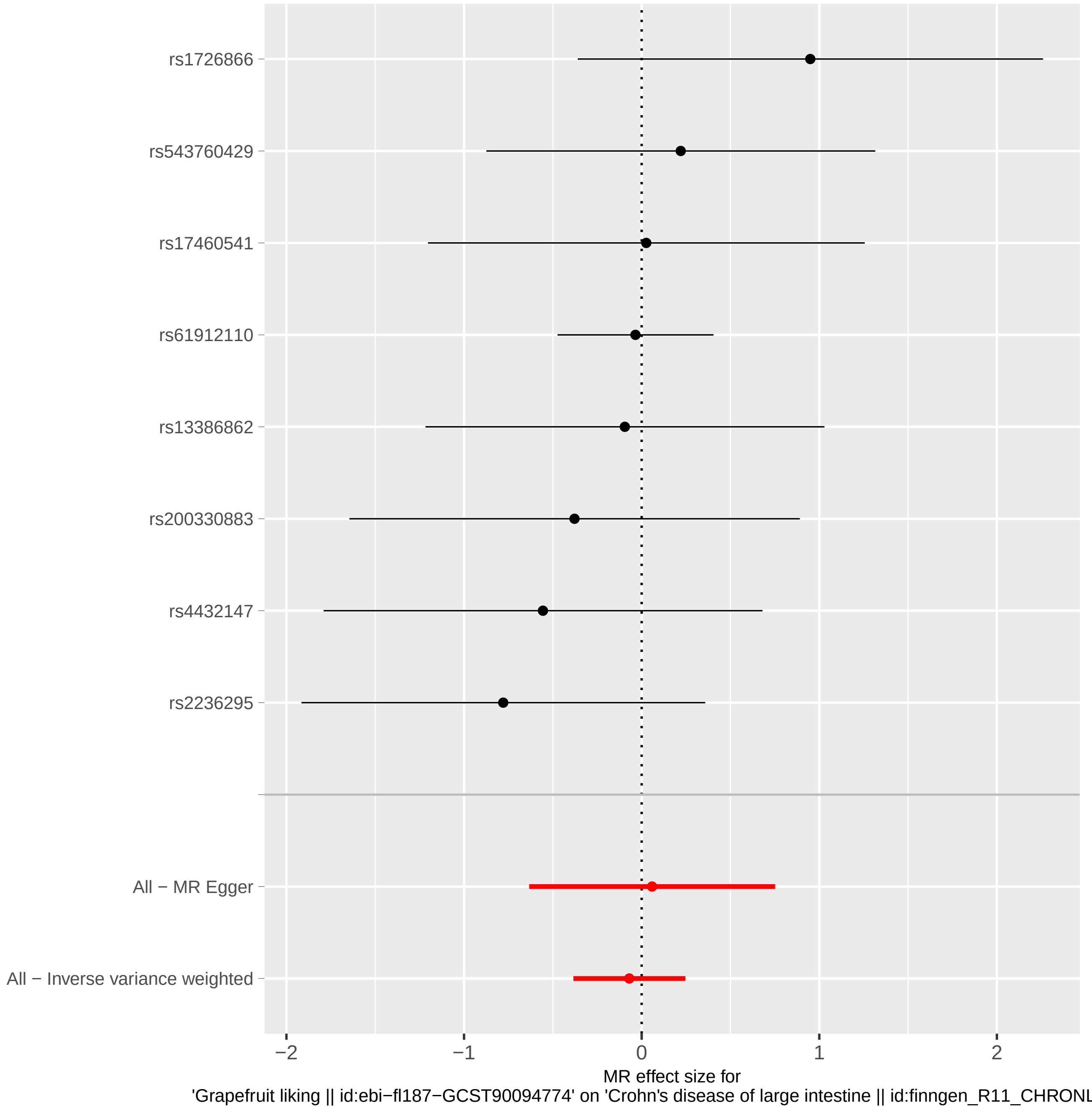

rs1726866
rs543760429
rs17460541
rs61912110
rs13386862
rs200330883
rs4432147
rs2236295
All − MR Egger
All − Inverse variance weighted
−2
−1
0
1
2
MR effect size for
'Grapefruit liking || id:ebi−fl187−GCST90094774' on 'Crohn's disease of large intestine || id:finngen_R11_CHRONL

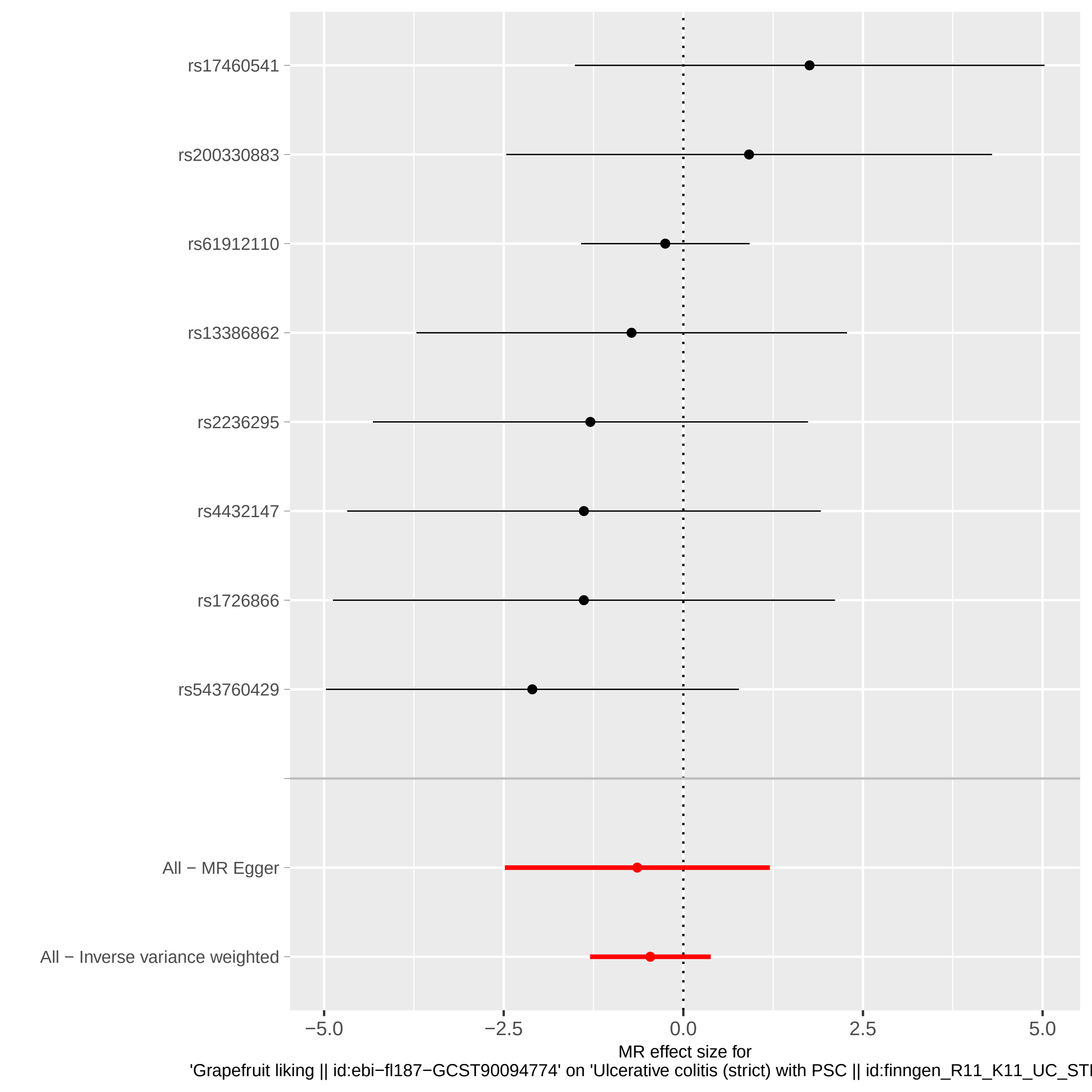

rs17460541
rs200330883
rs61912110
rs13386862
rs2236295
rs4432147
rs1726866
rs543760429
All − MR Egger
All − Inverse variance weighted
−5.0
−2.5
0.0
2.5
5.0
MR effect size for
'Grapefruit liking || id:ebi−fl187−GCST90094774' on 'Ulcerative colitis (strict) with PSC || id:finngen_R11_K11_UC_STI

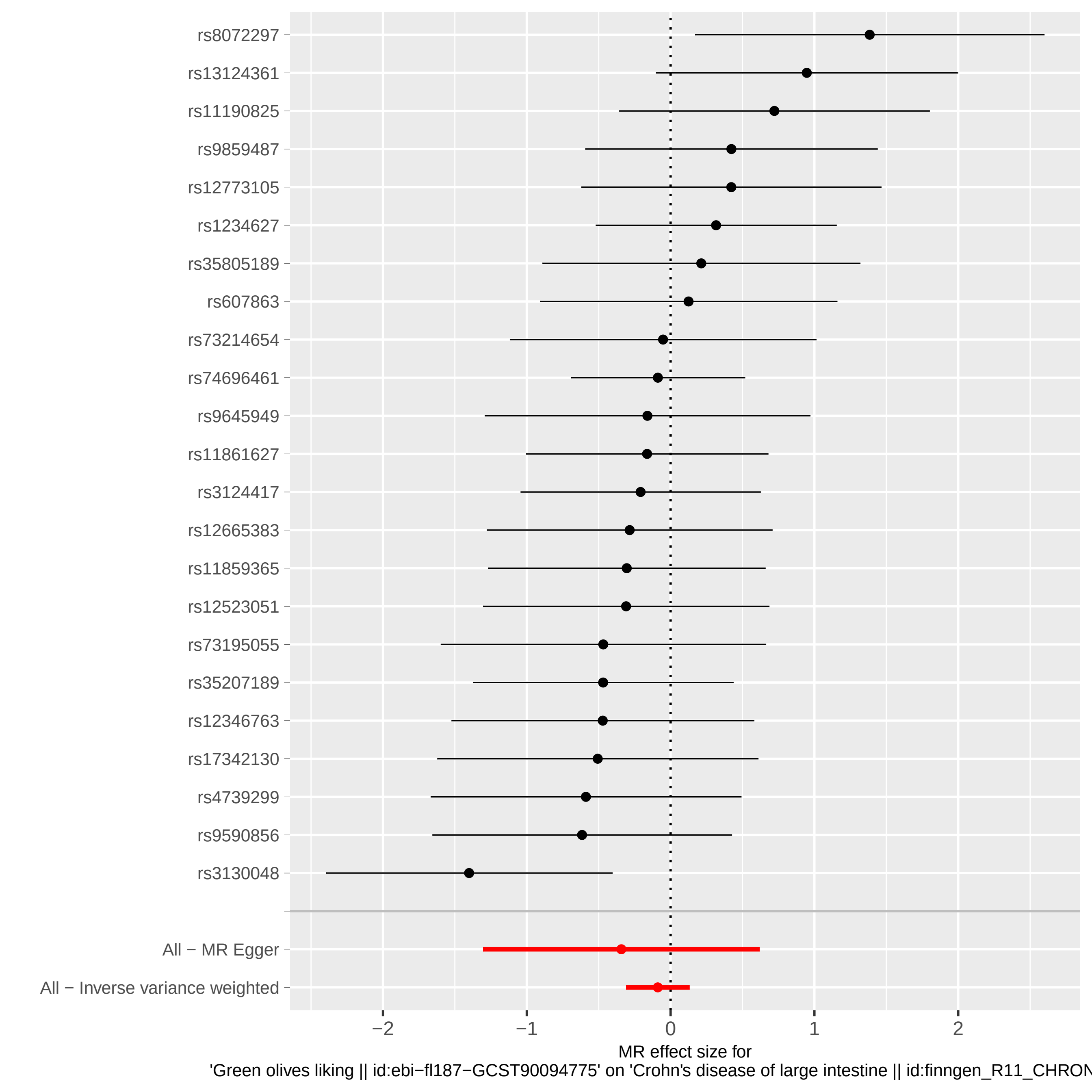
rs8072297
rs13124361
rs11190825
rs9859487
rs12773105
rs1234627
rs35805189
rs607863
rs73214654
rs74696461
rs9645949
rs11861627
rs3124417
rs12665383
rs11859365
rs12523051
rs73195055
rs35207189
rs12346763
rs17342130
rs4739299
rs9590856
rs3130048
All − MR Egger
All − Inverse variance weighted
−2
−1
0
1
2
MR effect size for
'Green olives liking || id:ebi−fl187−GCST90094775' on 'Crohn's disease of large intestine || id:finngen_R11_CHRON

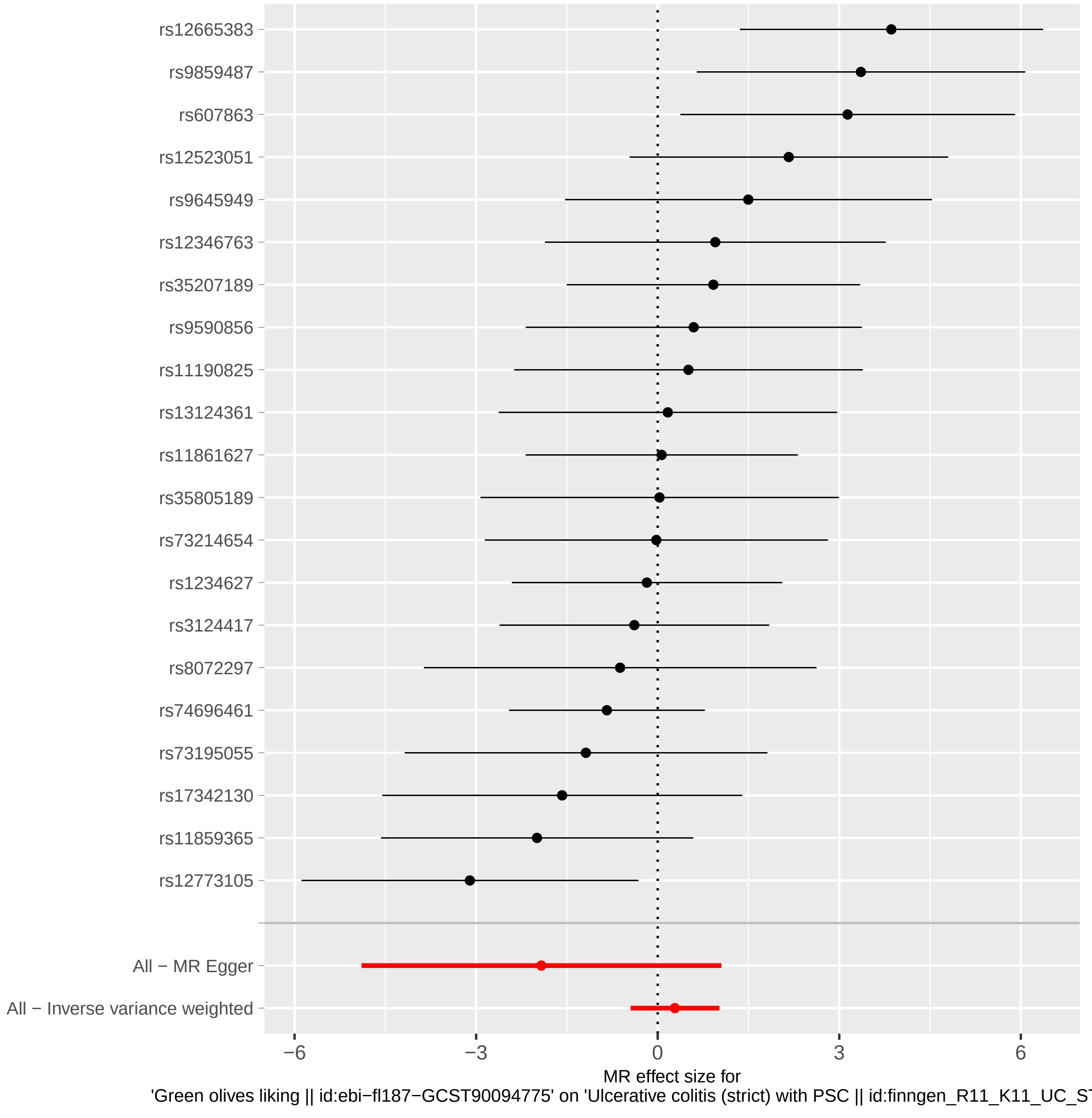
rs12665383
rs9859487
rs607863
rs12523051
rs9645949
rs12346763
rs35207189
rs9590856
rs11190825
rs13124361
rs11861627
rs35805189
rs73214654
rs1234627
rs3124417
rs8072297
rs74696461
rs73195055
rs17342130
rs11859365
rs12773105
All − MR Egger
All − Inverse variance weighted
−6
−3
0
3
6
MR effect size for
'Green olives liking || id:ebi−fl187−GCST90094775' on 'Ulcerative colitis (strict) with PSC || id:finngen_R11_K11_UC_ST

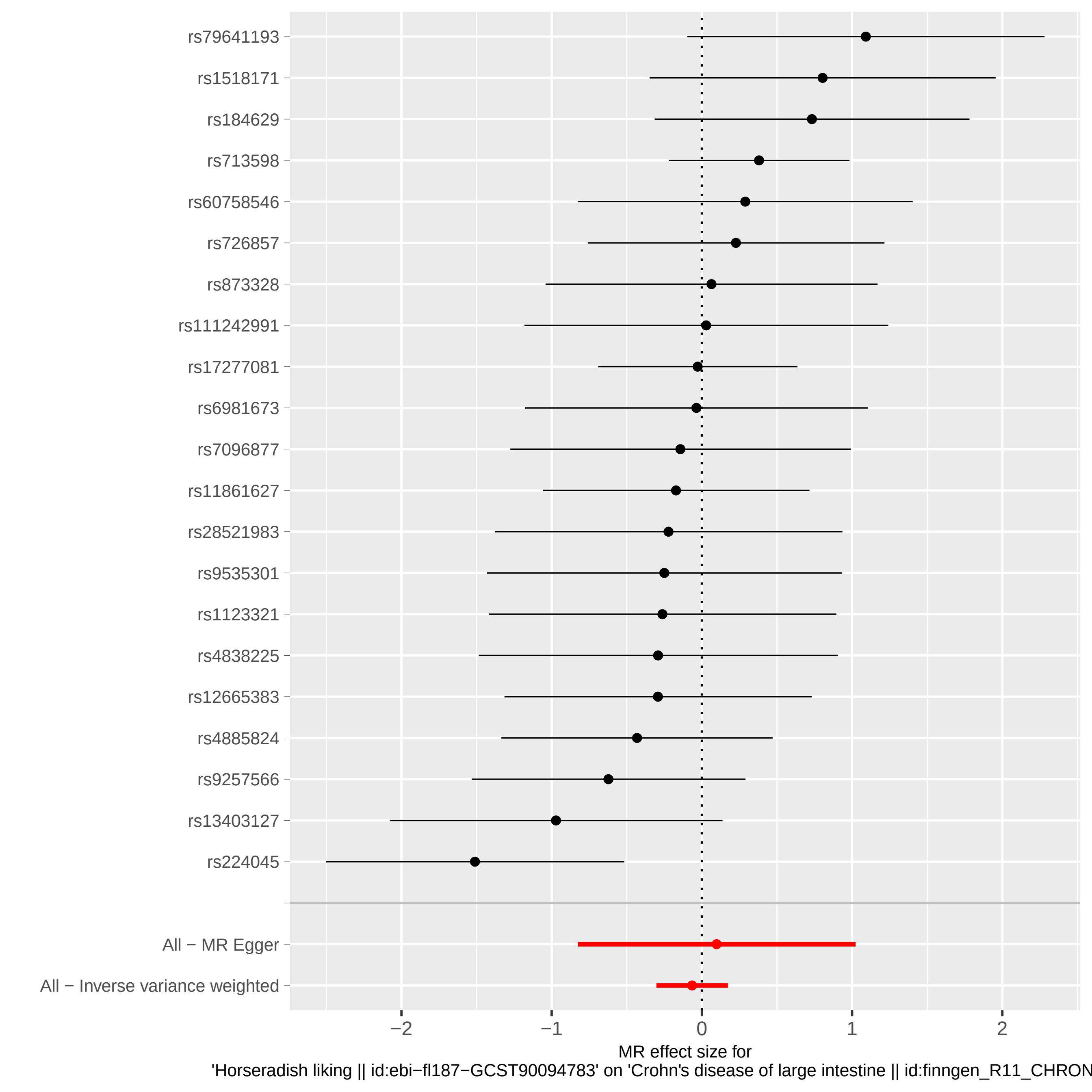

rs79641193
rs1518171
rs184629
rs713598
rs60758546
rs726857
rs873328
rs111242991
rs17277081
rs6981673
rs7096877
rs11861627
rs28521983
rs9535301
rs1123321
rs4838225
rs12665383
rs4885824
rs9257566
rs13403127
rs224045
All − MR Egger
All − Inverse variance weighted
−2
−1
0
1
2
MR effect size for
'Horseradish liking || id:ebi−fl187−GCST90094783' on 'Crohn's disease of large intestine || id:finngen_R11_CHRON

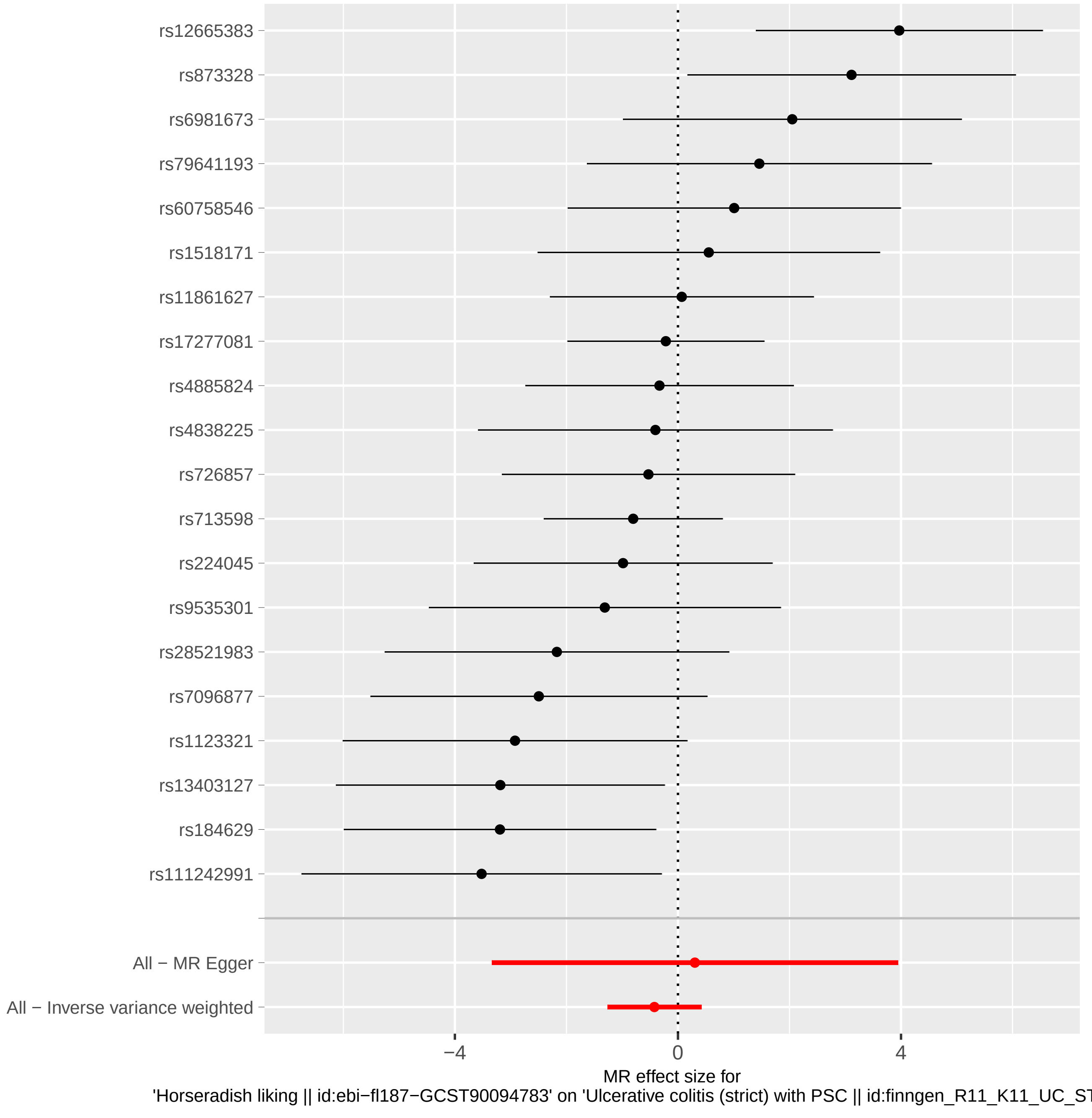
rs12665383
rs873328
rs6981673
rs79641193
rs60758546
rs1518171
rs11861627
rs17277081
rs4885824
rs4838225
rs726857
rs713598
rs224045
rs9535301
rs28521983
rs7096877
rs1123321
rs13403127
rs184629
rs111242991
All − MR Egger
All − Inverse variance weighted
−4
0
4
MR effect size for
'Horseradish liking || id:ebi−fl187−GCST90094783' on 'Ulcerative colitis (strict) with PSC || id:finngen_R11_K11_UC_ST

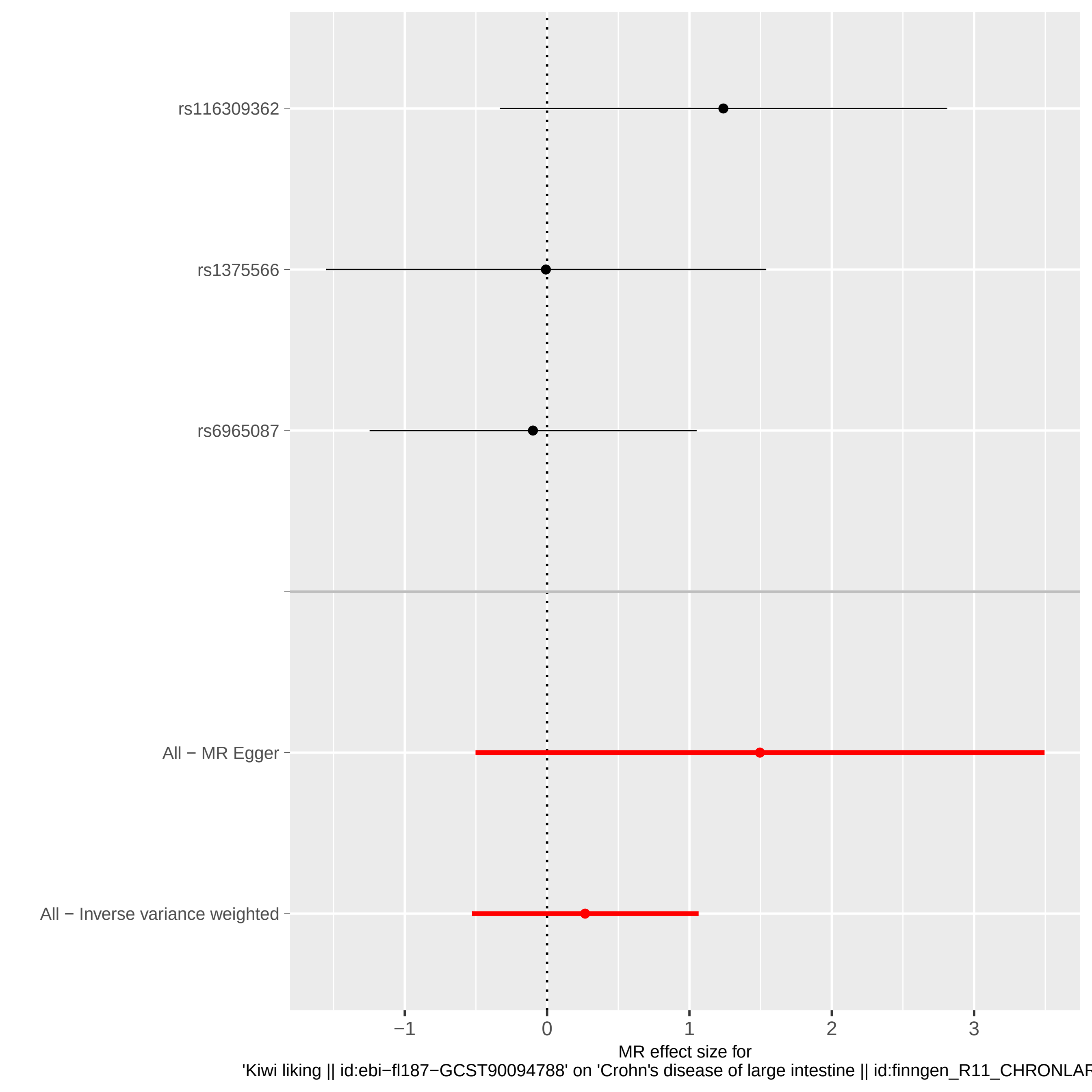

rs116309362
rs1375566
rs6965087
All − MR Egger
All − Inverse variance weighted
−1
0
1
2
3
MR effect size for
'Kiwi liking || id:ebi−fl187−GCST90094788' on 'Crohn's disease of large intestine || id:finngen_R11_CHRONLAR

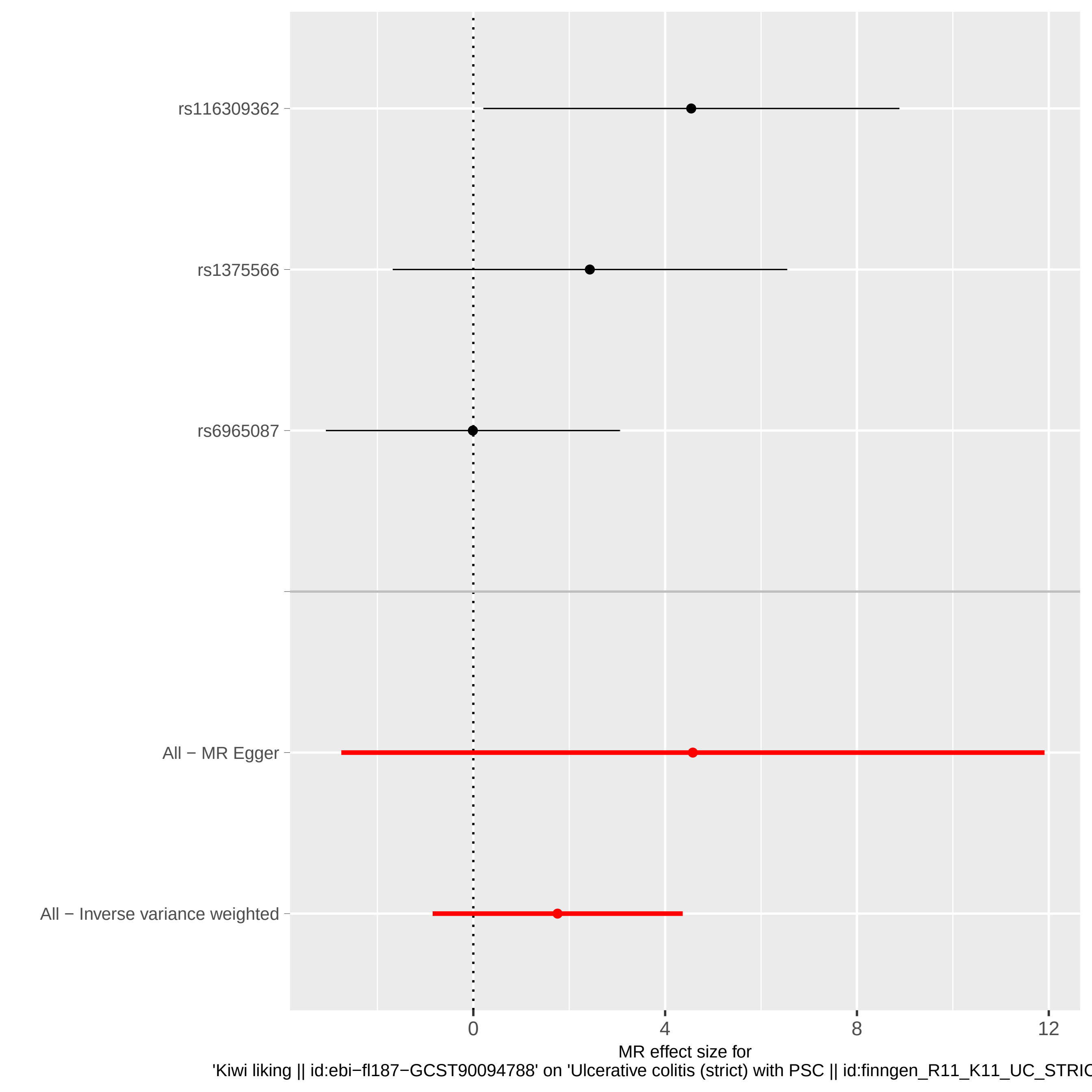
rs116309362
rs1375566
rs6965087
All − MR Egger
All − Inverse variance weighted
0
4
8
12
MR effect size for
'Kiwi liking || id:ebi−fl187−GCST90094788' on 'Ulcerative colitis (strict) with PSC || id:finngen_R11_K11_UC_STRIC

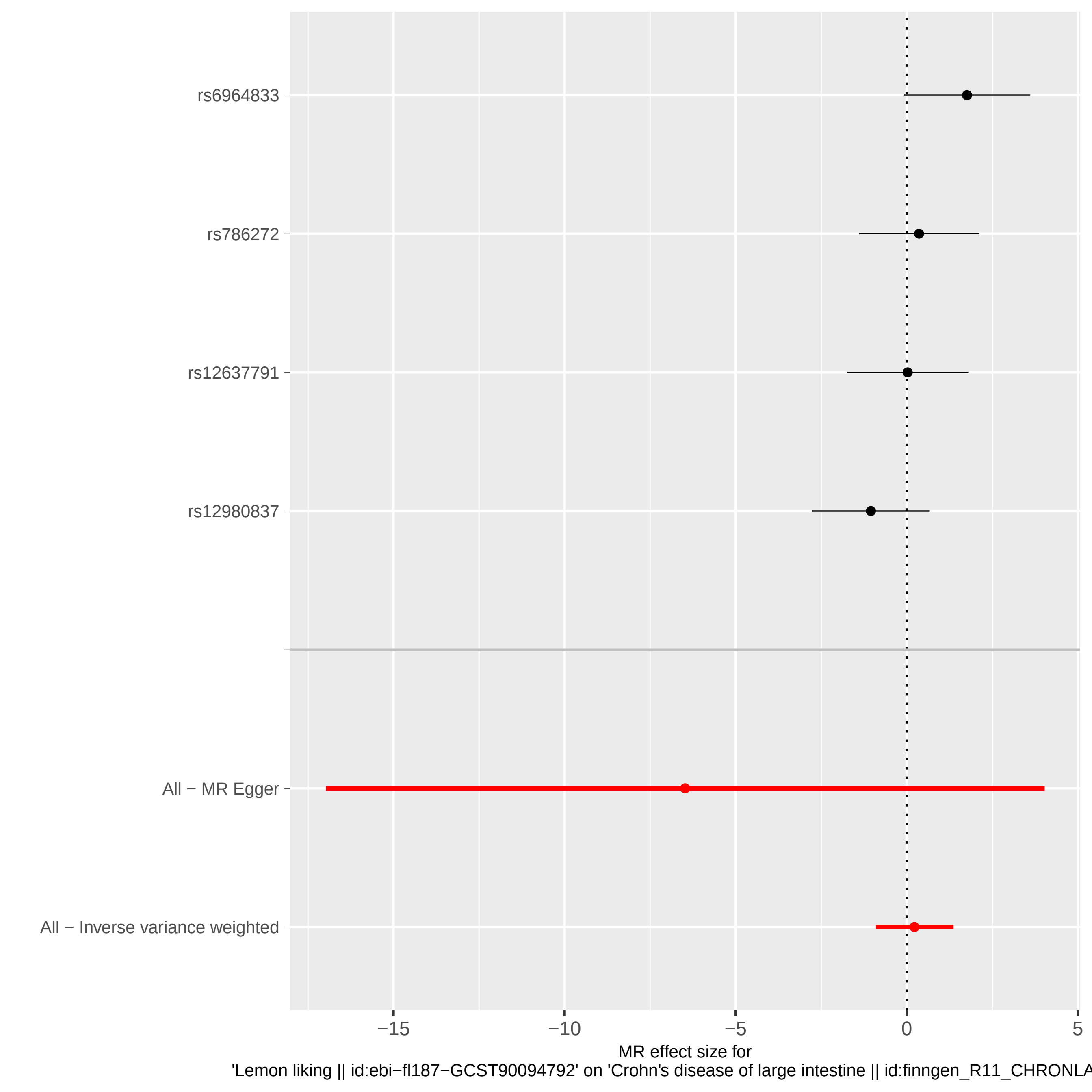

rs6964833
rs786272
rs12637791
rs12980837
All − MR Egger
All − Inverse variance weighted
−15
−10
−5
0
5
MR effect size for
'Lemon liking || id:ebi−fl187−GCST90094792' on 'Crohn's disease of large intestine || id:finngen_R11_CHRONLA

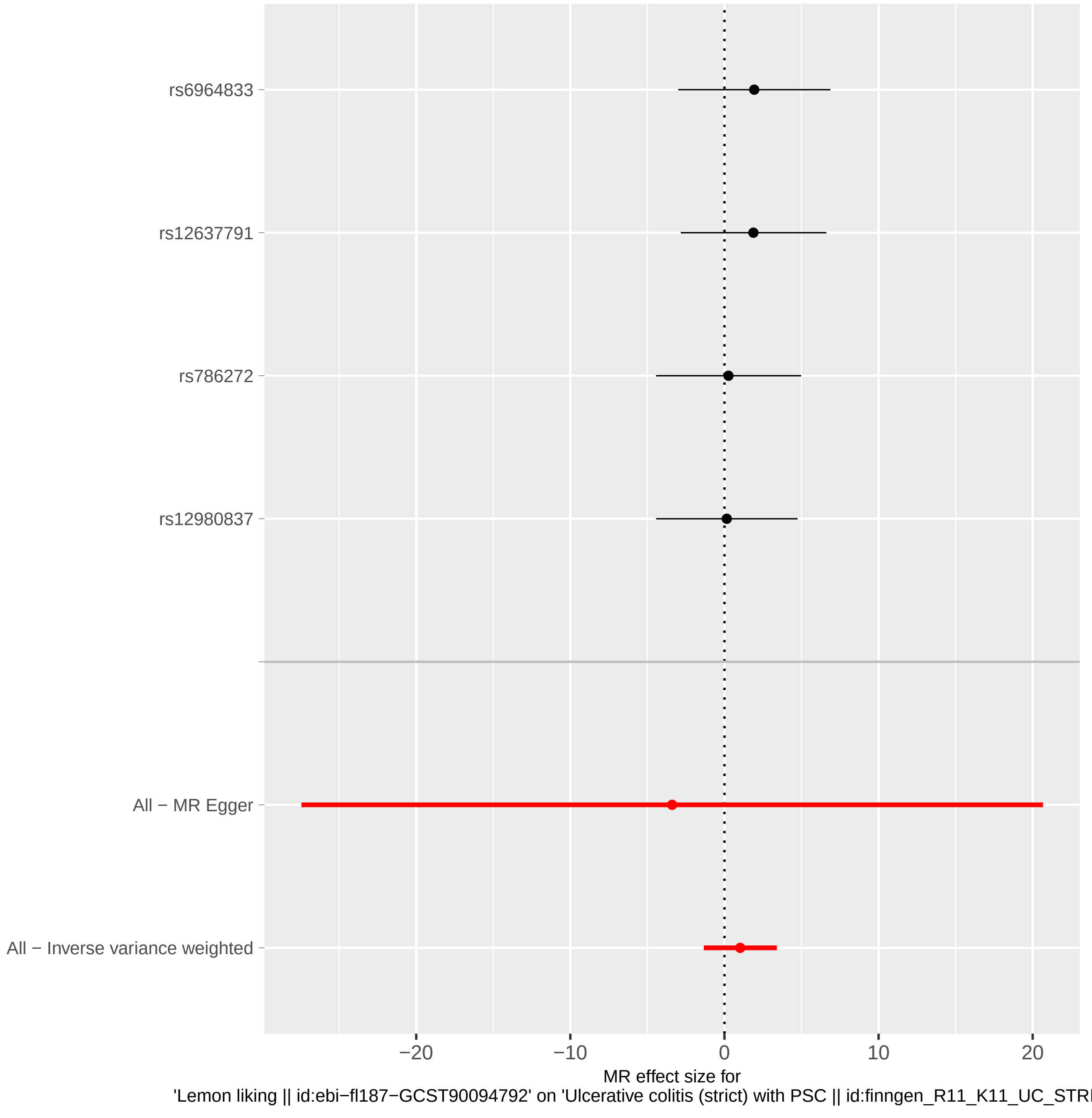

rs6964833
rs12637791
rs786272
rs12980837
All − MR Egger
All − Inverse variance weighted
−20
−10
0
10
20
MR effect size for
'Lemon liking || id:ebi−fl187−GCST90094792' on 'Ulcerative colitis (strict) with PSC || id:finngen_R11_K11_UC_STR

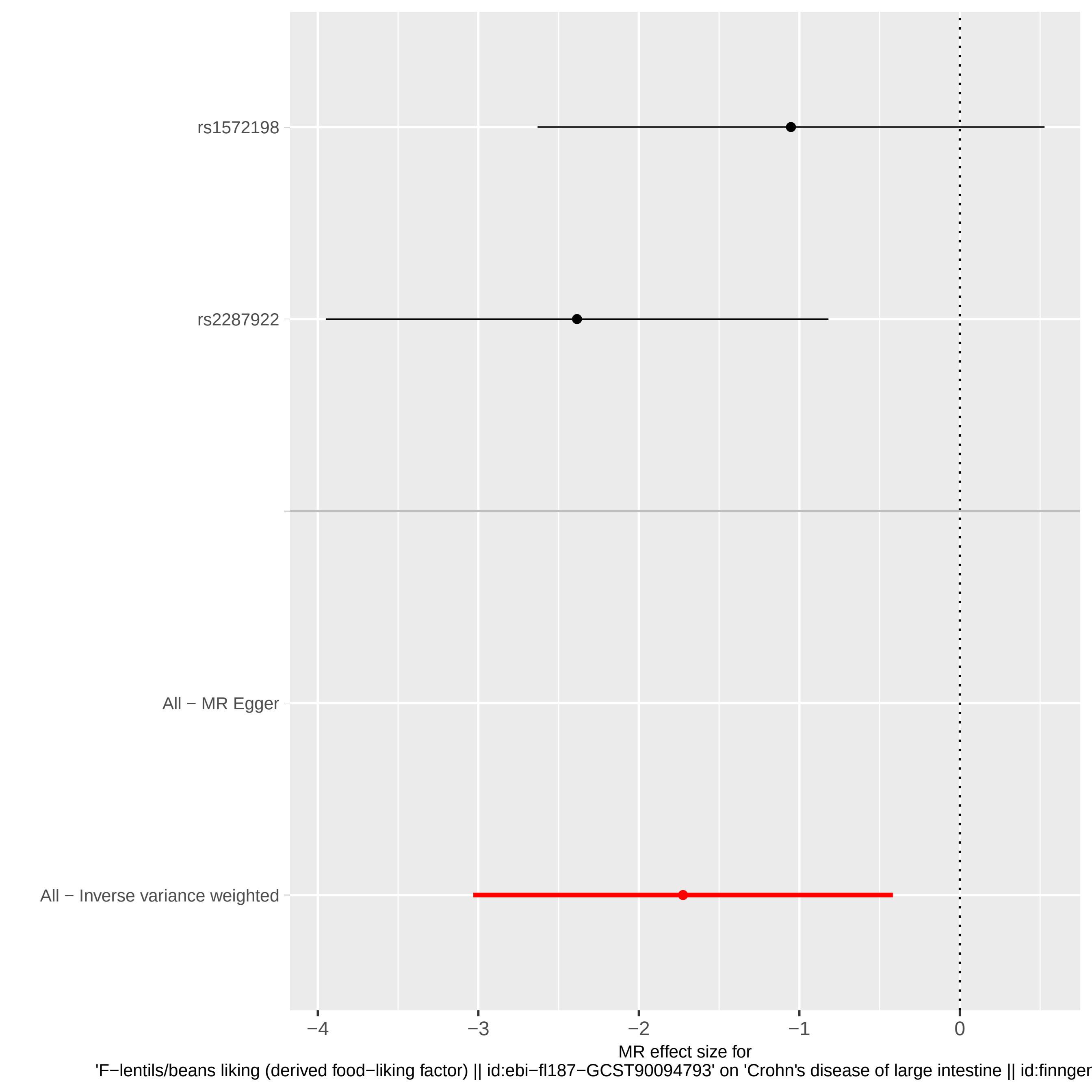

rs1572198
rs2287922
All − MR Egger
All − Inverse variance weighted
−4
−3
−2
−1
0
MR effect size for
'F−lentils/beans liking (derived food−liking factor) || id:ebi−fl187−GCST90094793' on 'Crohn's disease of large intestine || id:finnger

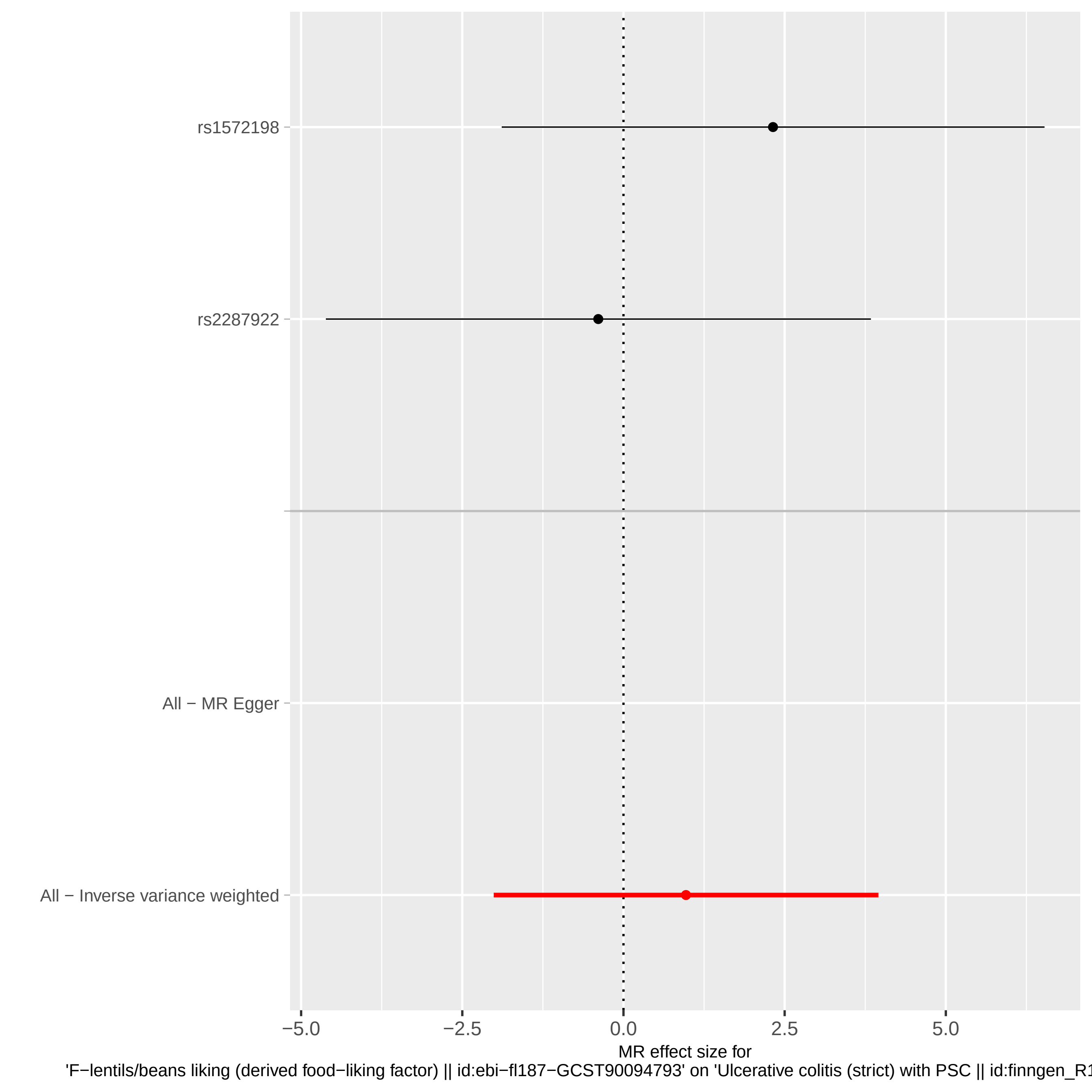
rs1572198
rs2287922
All − MR Egger
All − Inverse variance weighted
−5.0
−2.5
0.0
2.5
5.0
MR effect size for
'F−lentils/beans liking (derived food−liking factor) || id:ebi−fl187−GCST90094793' on 'Ulcerative colitis (strict) with PSC || id:finngen_R1

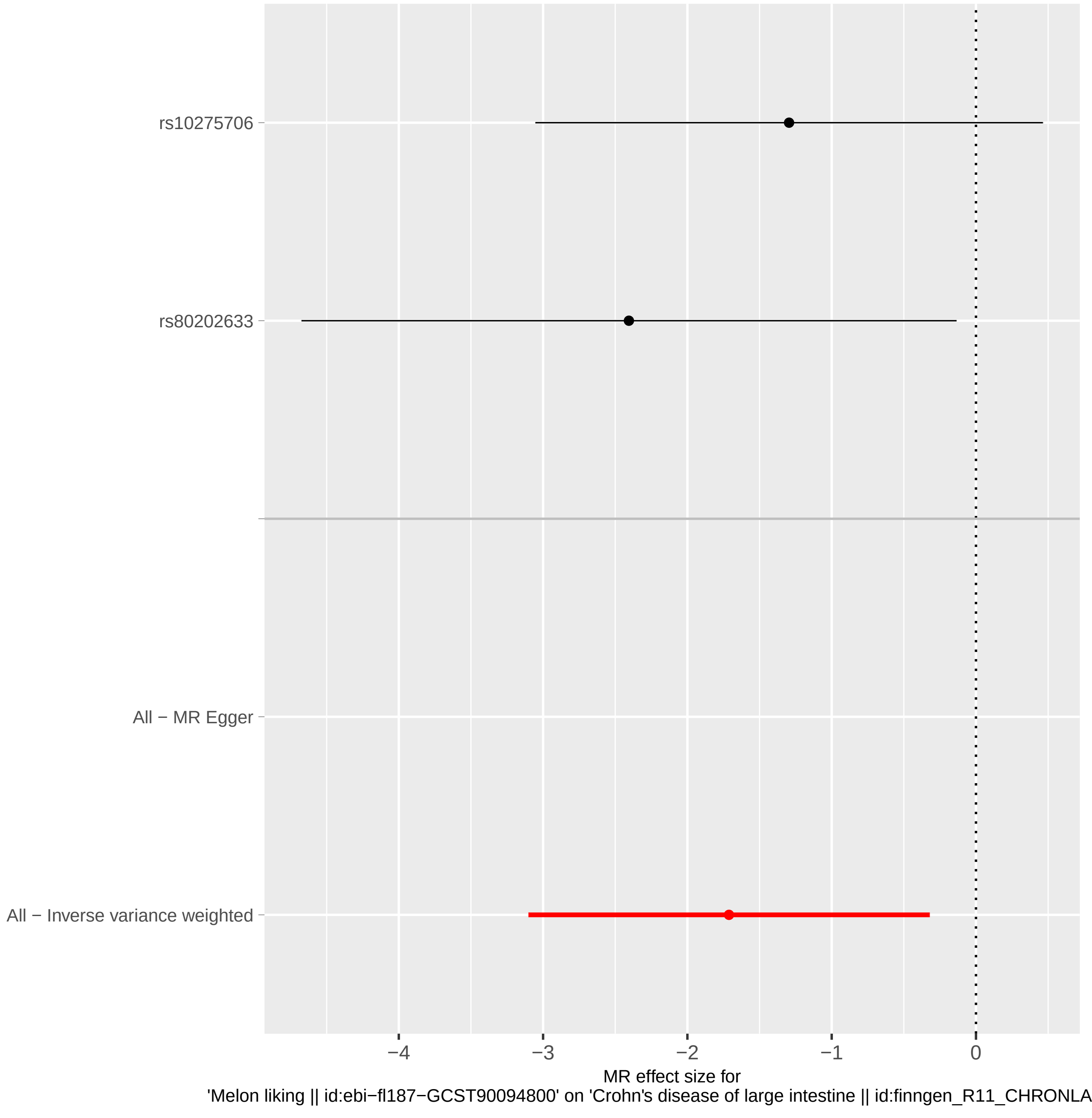
rs10275706
rs80202633
All − MR Egger
All − Inverse variance weighted
−4
−3
−2
−1
0
MR effect size for
'Melon liking || id:ebi−fl187−GCST90094800' on 'Crohn's disease of large intestine || id:finngen_R11_CHRONLA

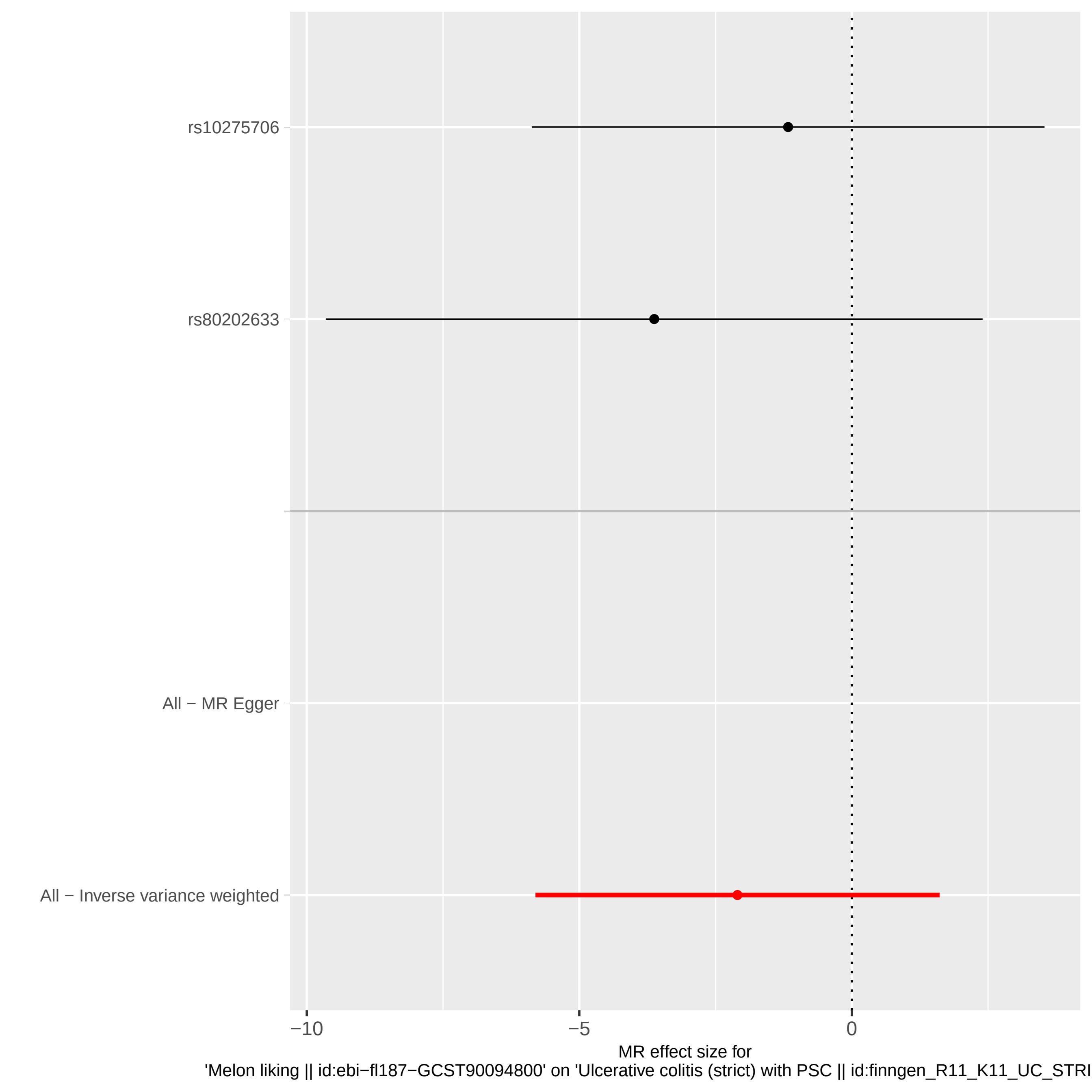
rs10275706
rs80202633
All − MR Egger
All − Inverse variance weighted
−10
−5
0
MR effect size for
'Melon liking || id:ebi−fl187−GCST90094800' on 'Ulcerative colitis (strict) with PSC || id:finngen_R11_K11_UC_STRI

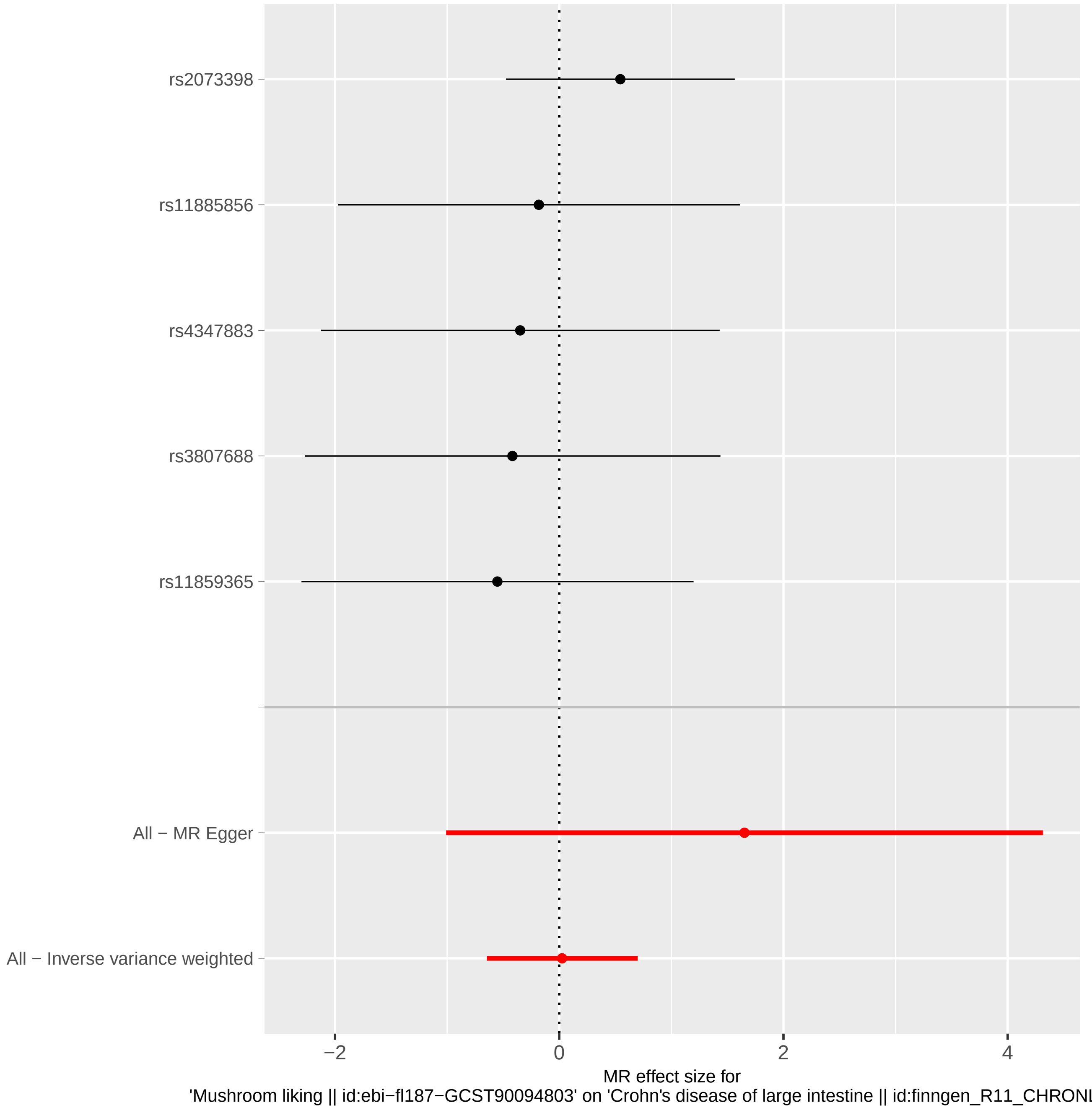
rs2073398
rs11885856
rs4347883
rs3807688
rs11859365
All − MR Egger
All − Inverse variance weighted
−2
0
2
4
MR effect size for
'Mushroom liking || id:ebi−fl187−GCST90094803' on 'Crohn's disease of large intestine || id:finngen_R11_CHRONI

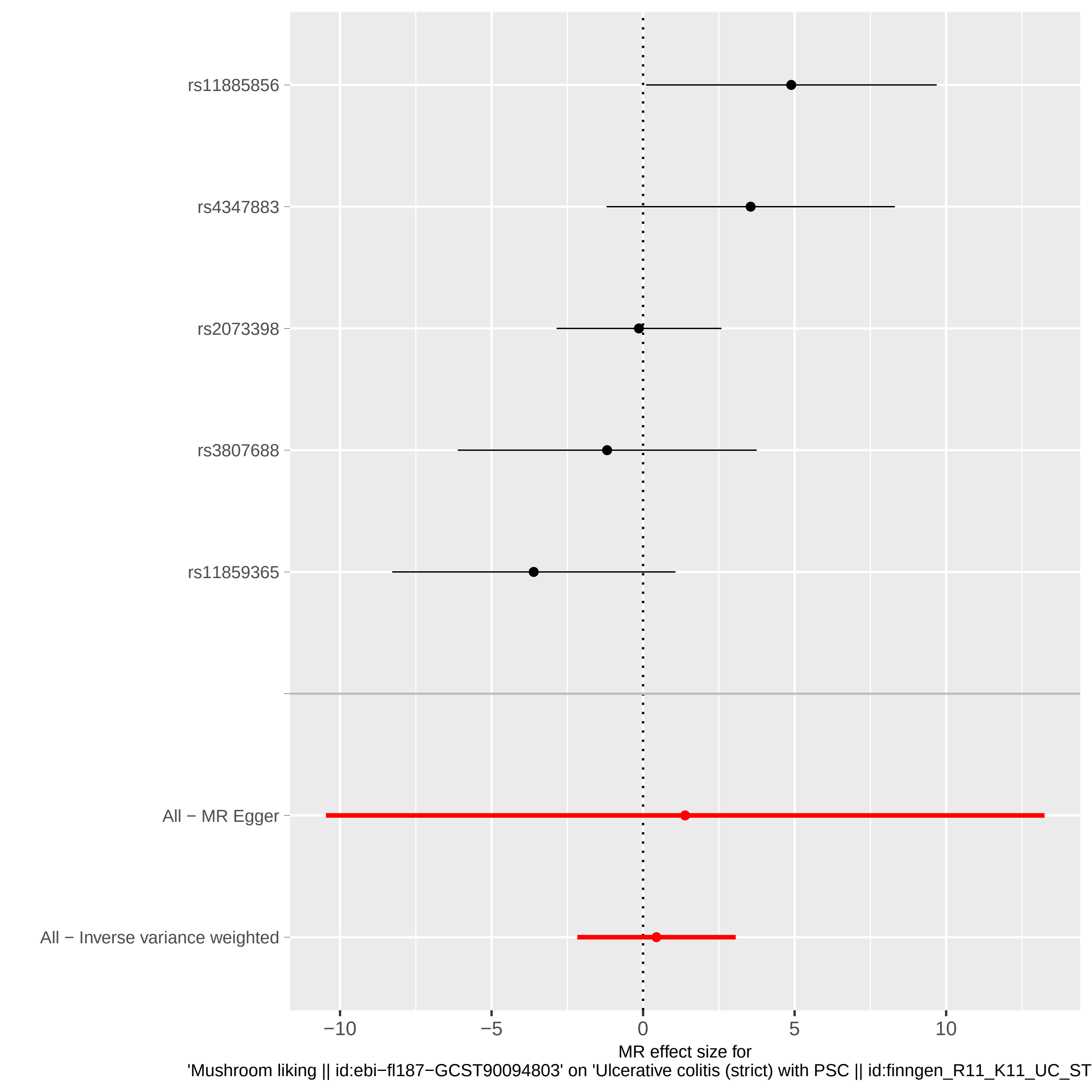

rs11885856
rs4347883
rs2073398
rs3807688
rs11859365
All − MR Egger
All − Inverse variance weighted
−10
−5
0
5
10
MR effect size for
'Mushroom liking || id:ebi−fl187−GCST90094803' on 'Ulcerative colitis (strict) with PSC || id:finngen_R11_K11_UC_ST

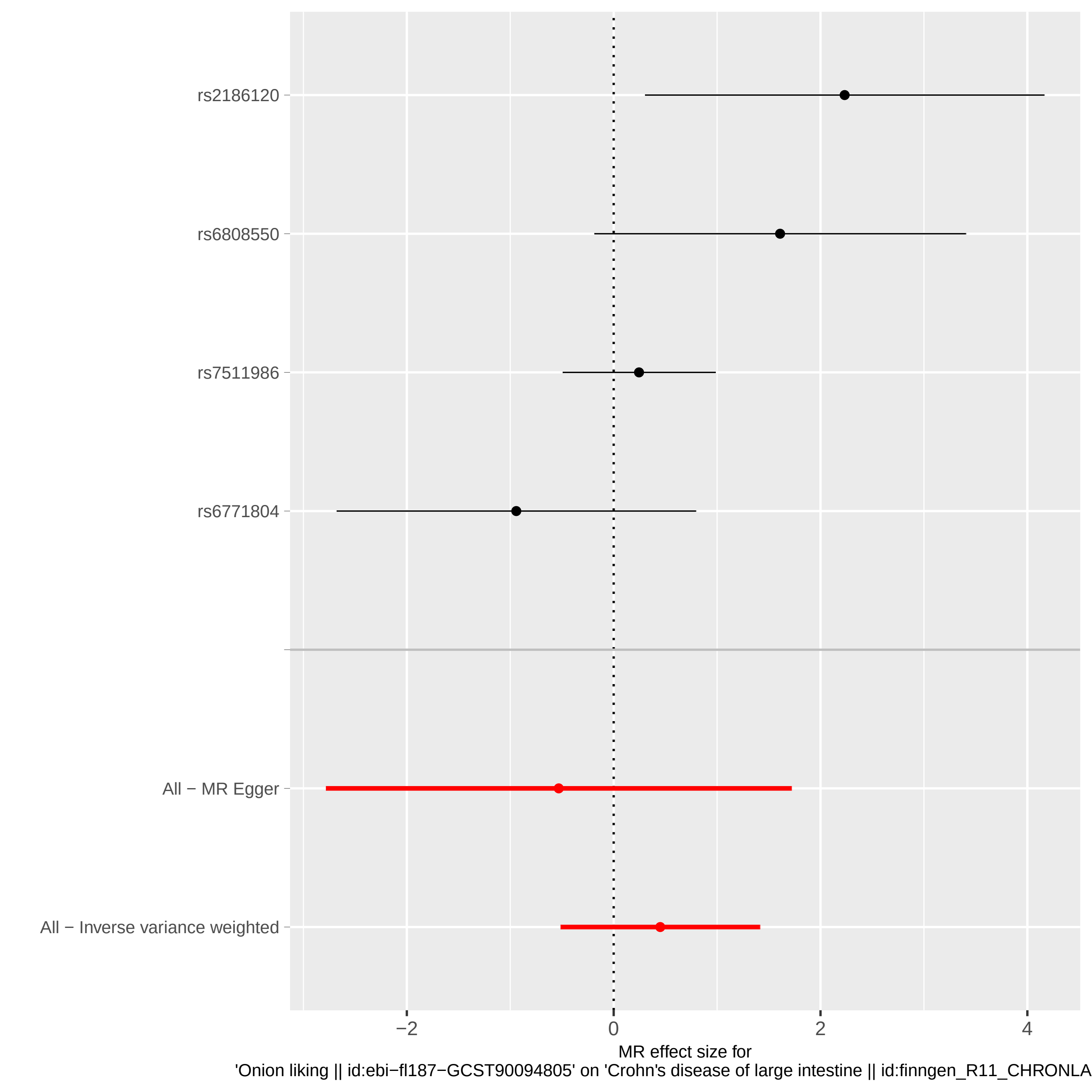
rs2186120
rs6808550
rs7511986
rs6771804
All − MR Egger
All − Inverse variance weighted
−2
0
2
4
MR effect size for
'Onion liking || id:ebi−fl187−GCST90094805' on 'Crohn's disease of large intestine || id:finngen_R11_CHRONLA

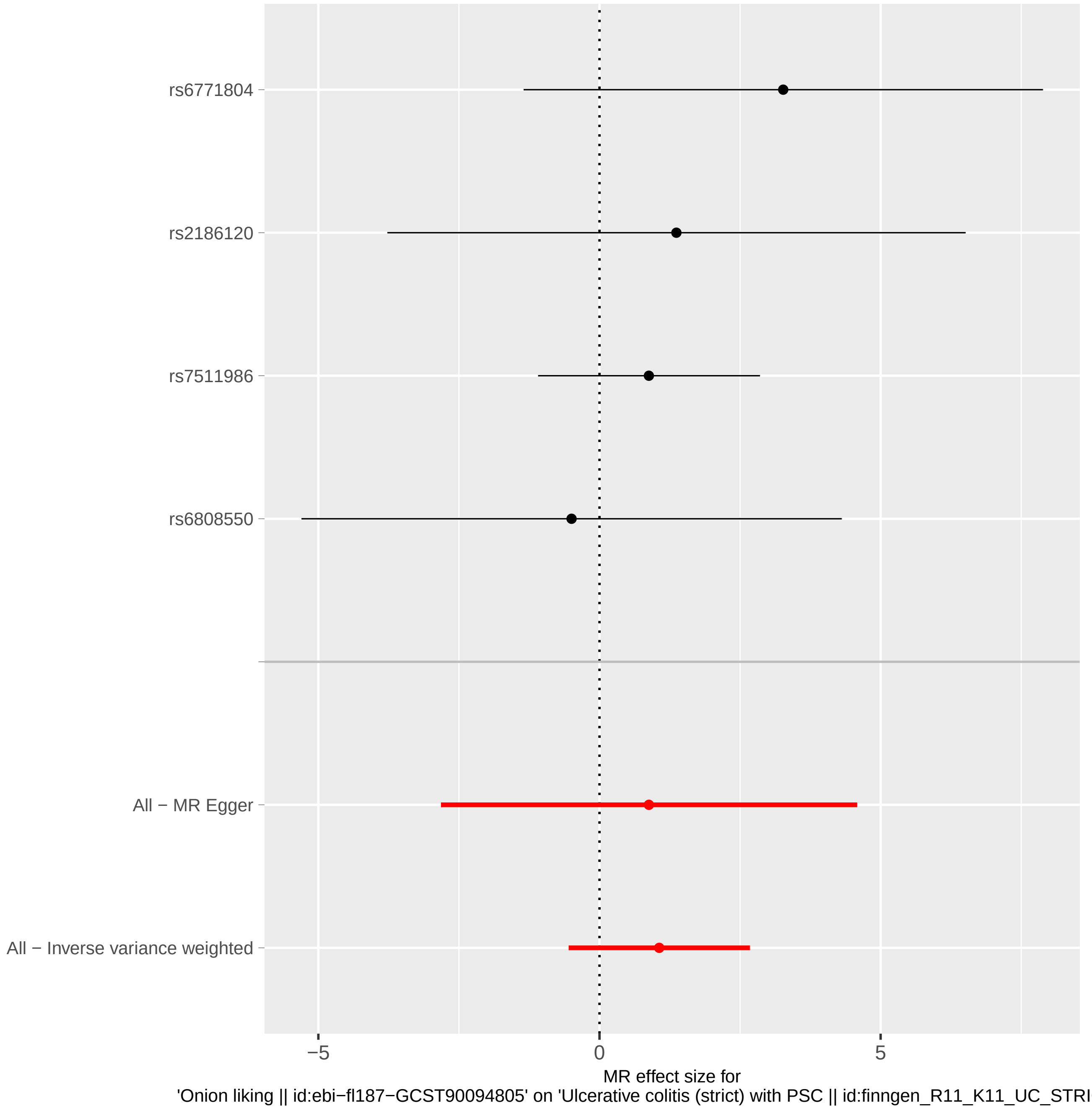
rs6771804
rs2186120
rs7511986
rs6808550
All − MR Egger
All − Inverse variance weighted
−5
0
5
MR effect size for
'Onion liking || id:ebi−fl187−GCST90094805' on 'Ulcerative colitis (strict) with PSC || id:finngen_R11_K11_UC_STRI

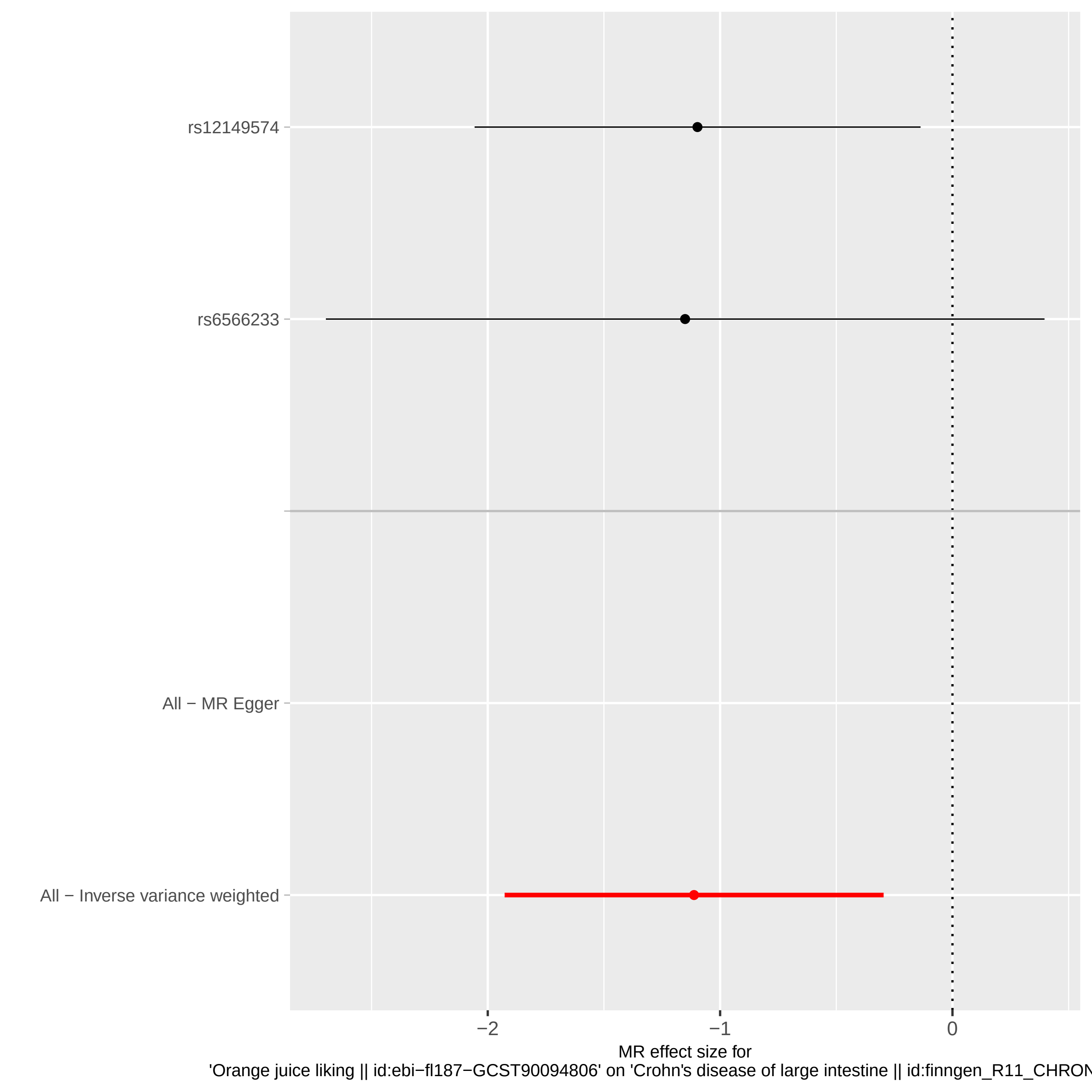
rs12149574
rs6566233
All − MR Egger
All − Inverse variance weighted
−2
−1
0
MR effect size for
'Orange juice liking || id:ebi−fl187−GCST90094806' on 'Crohn's disease of large intestine || id:finngen_R11_CHRON

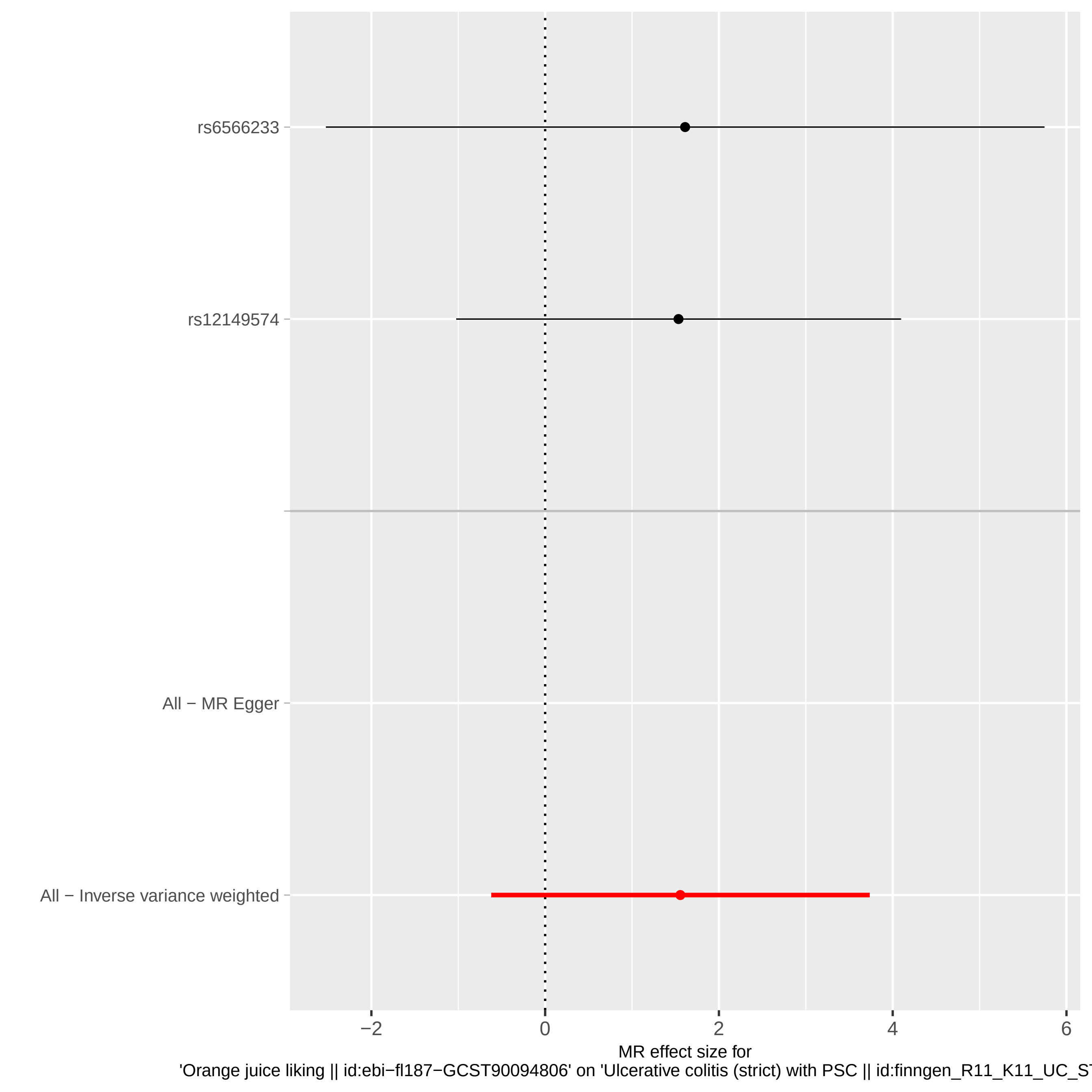
rs6566233
rs12149574
All − MR Egger
All − Inverse variance weighted
−2
0
2
4
6
MR effect size for
'Orange juice liking || id:ebi−fl187−GCST90094806' on 'Ulcerative colitis (strict) with PSC || id:finngen_R11_K11_UC_S

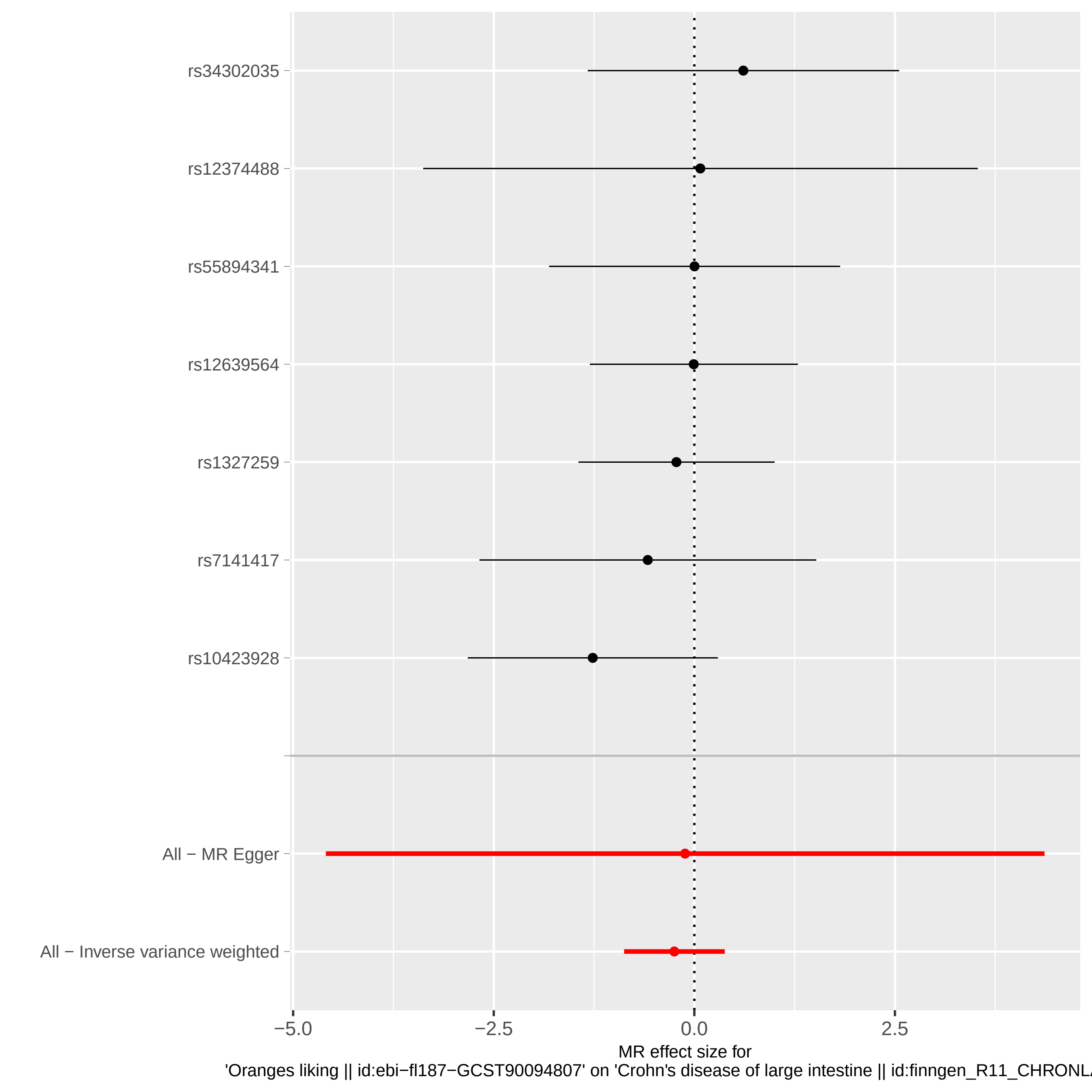
rs34302035
rs12374488
rs55894341
rs12639564
rs1327259
rs7141417
rs10423928
All − MR Egger
All − Inverse variance weighted
−5.0
−2.5
0.0
2.5
MR effect size for
'Oranges liking || id:ebi−fl187−GCST90094807' on 'Crohn's disease of large intestine || id:finngen_R11_CHRONL

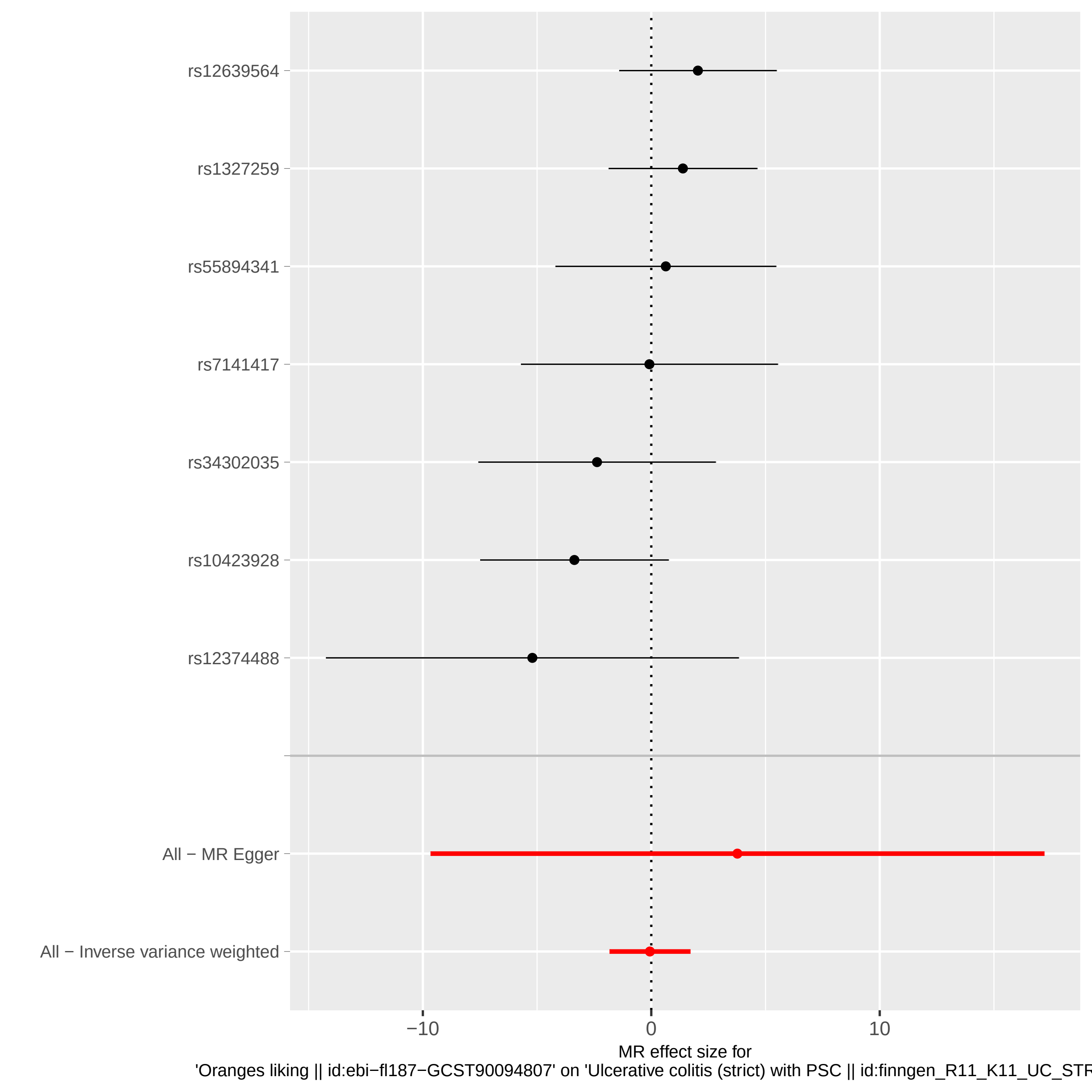

rs12639564
rs1327259
rs55894341
rs7141417
rs34302035
rs10423928
rs12374488
All − MR Egger
All − Inverse variance weighted
−10
0
10
MR effect size for
'Oranges liking || id:ebi−fl187−GCST90094807' on 'Ulcerative colitis (strict) with PSC || id:finngen_R11_K11_UC_STR

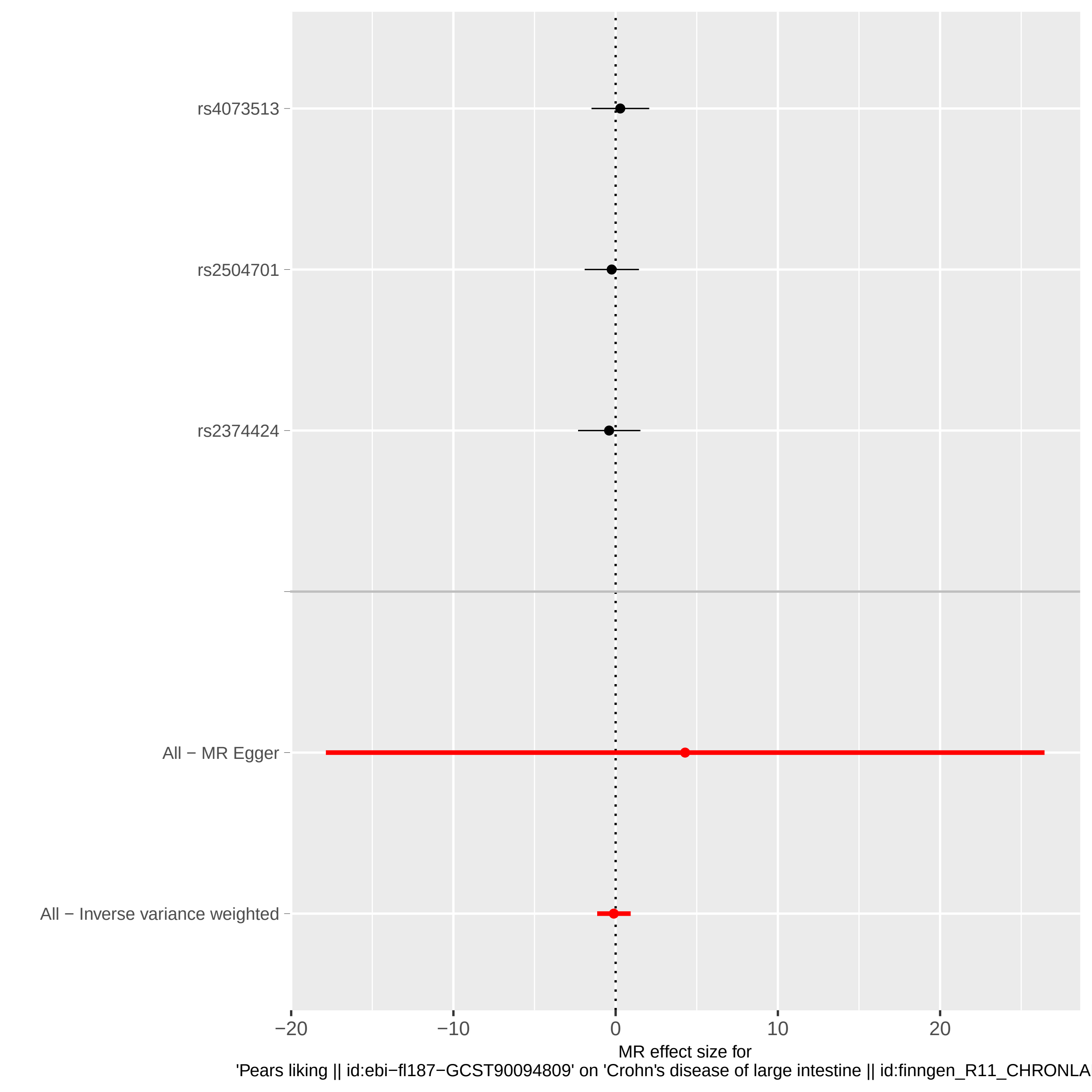
rs4073513
rs2504701
rs2374424
All − MR Egger
All − Inverse variance weighted
−20
−10
0
10
20
MR effect size for
'Pears liking || id:ebi−fl187−GCST90094809' on 'Crohn's disease of large intestine || id:finngen_R11_CHRONLA

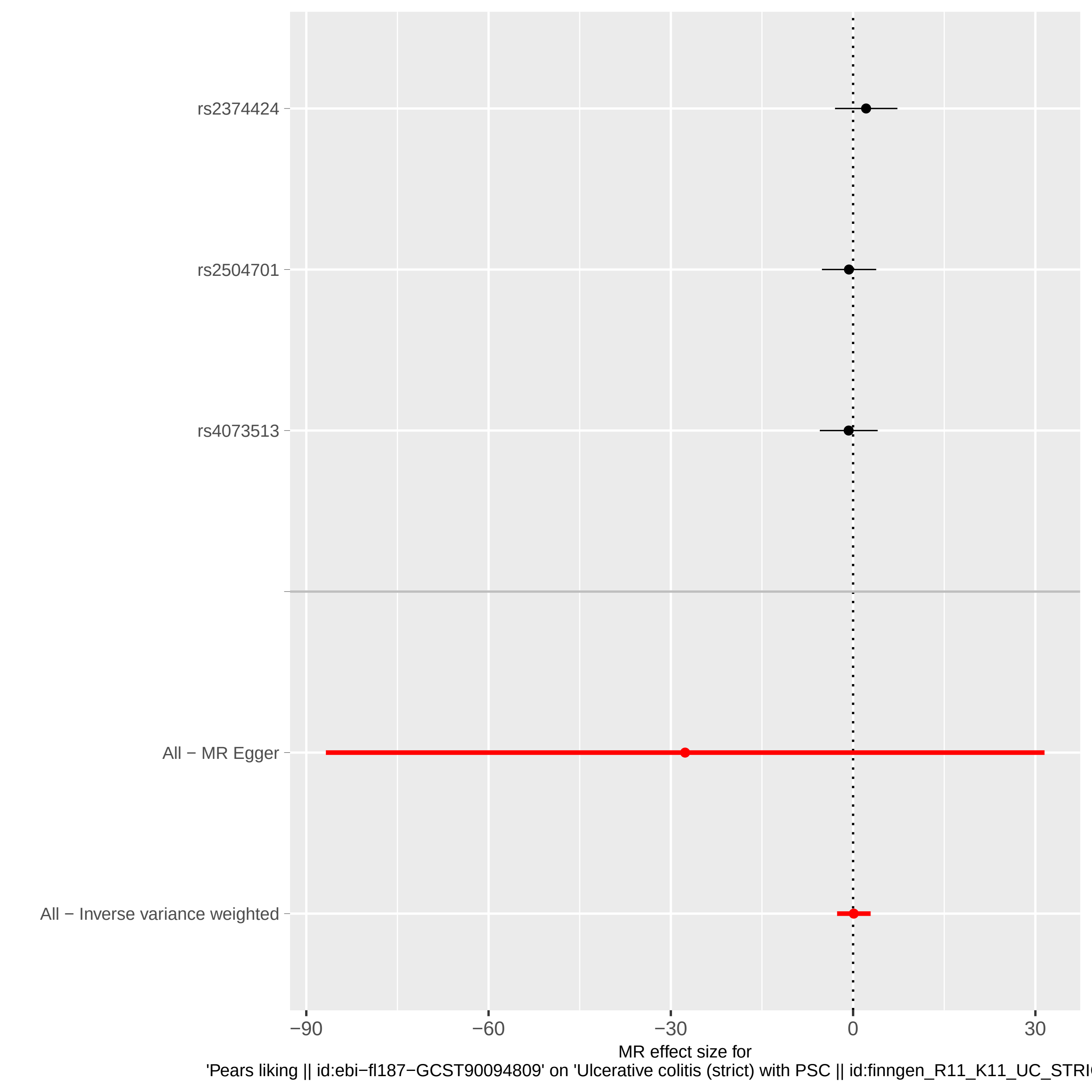

rs2374424
rs2504701
rs4073513
All − MR Egger
All − Inverse variance weighted
−90
−60
−30
0
30
MR effect size for
'Pears liking || id:ebi−fl187−GCST90094809' on 'Ulcerative colitis (strict) with PSC || id:finngen_R11_K11_UC_STRI

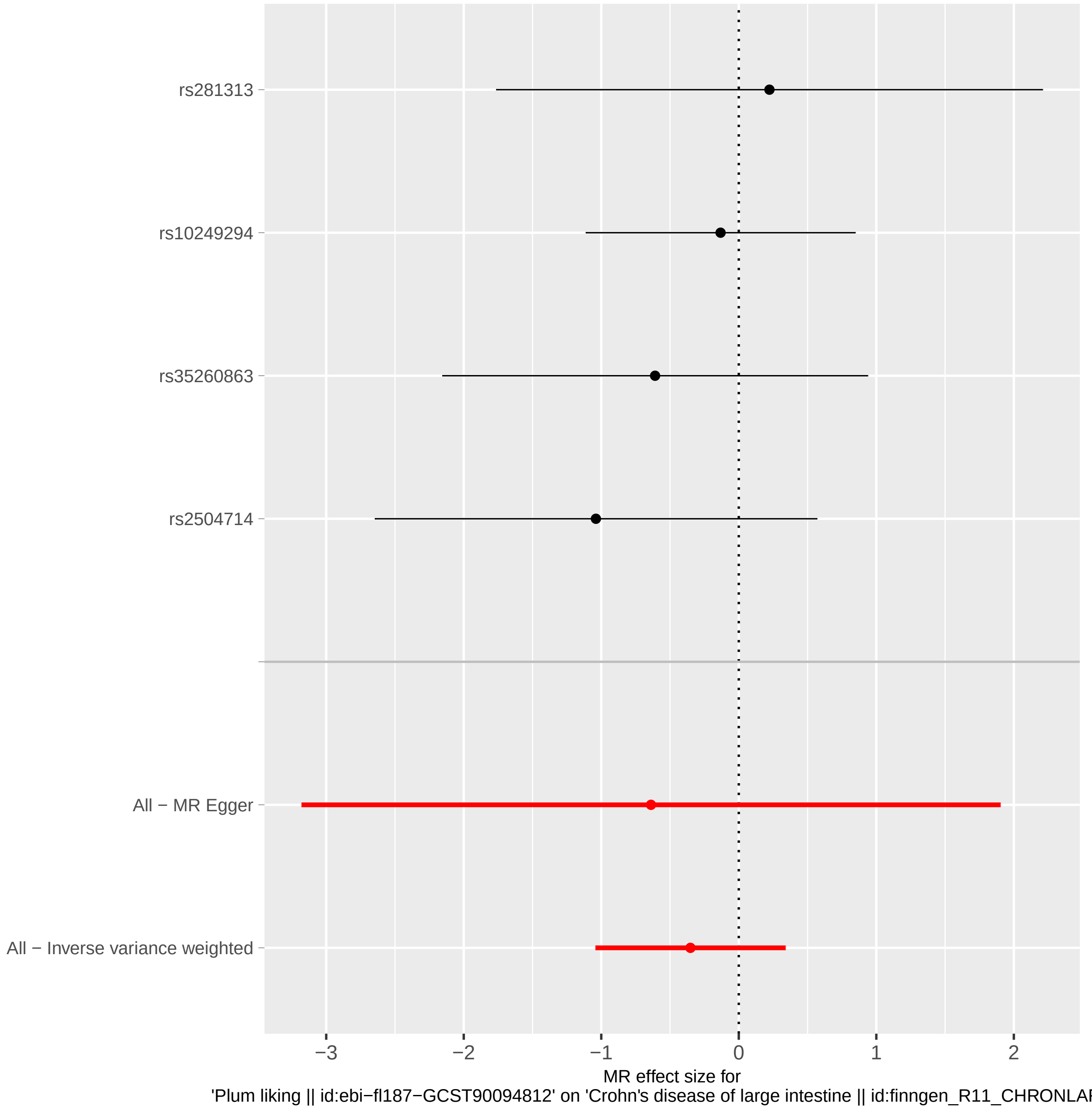

rs281313
rs10249294
rs35260863
rs2504714
All − MR Egger
All − Inverse variance weighted
−3
−2
−1
0
1
2
MR effect size for
'Plum liking || id:ebi−fl187−GCST90094812' on 'Crohn's disease of large intestine || id:finngen_R11_CHRONLAR

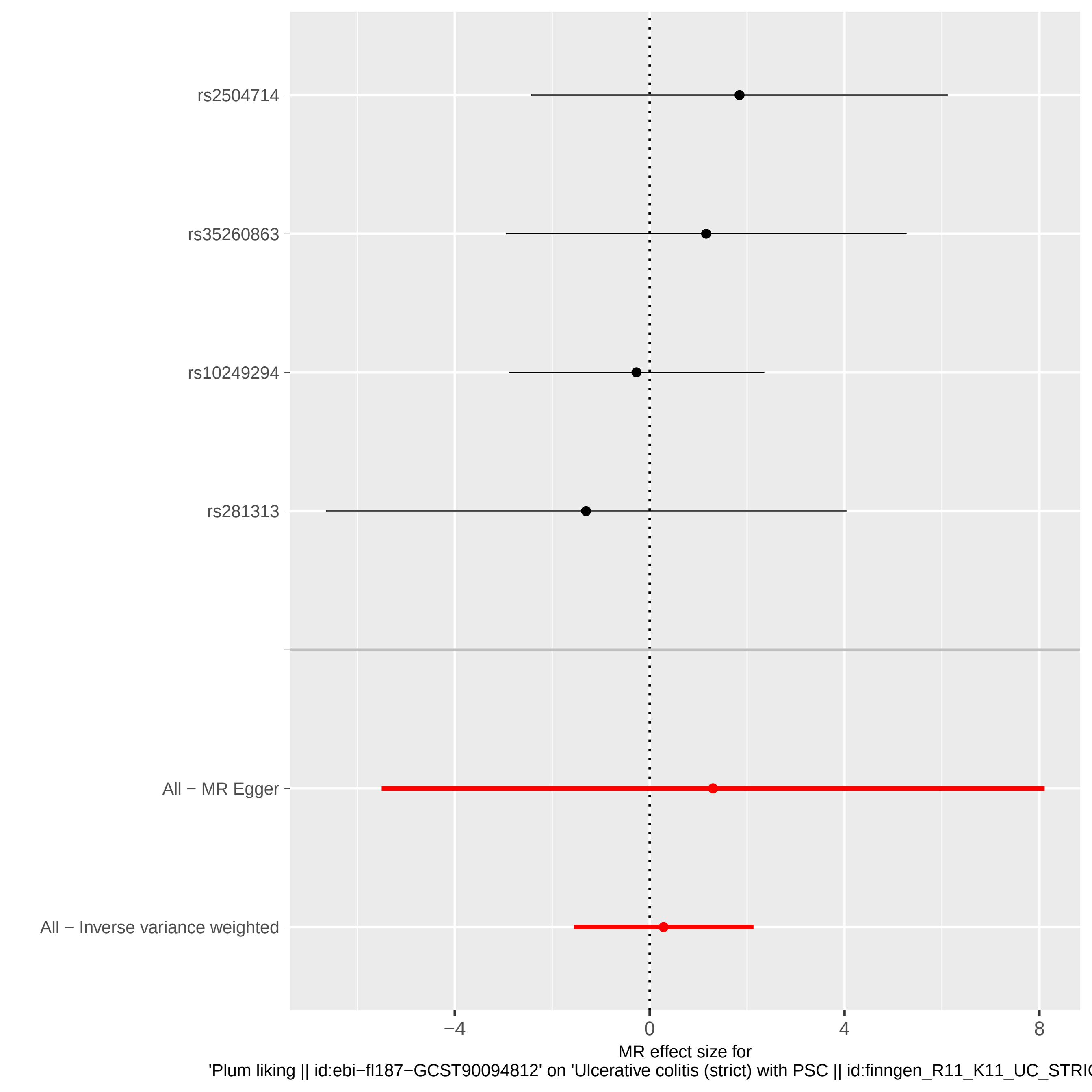
rs2504714
rs35260863
rs10249294
rs281313
All − MR Egger
All − Inverse variance weighted
−4
0
4
8
MR effect size for
'Plum liking || id:ebi−fl187−GCST90094812' on 'Ulcerative colitis (strict) with PSC || id:finngen_R11_K11_UC_STRI

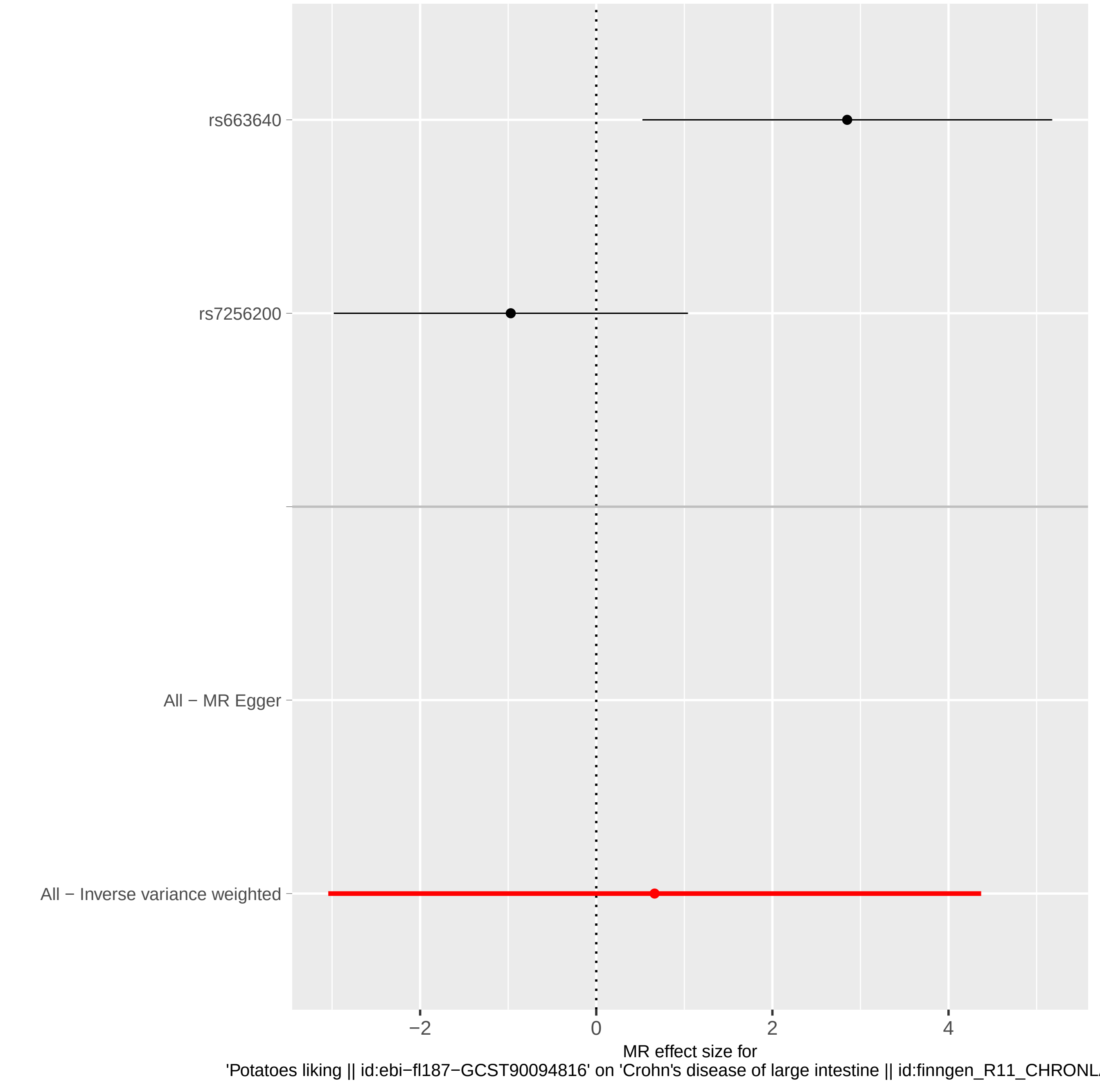
rs663640
rs7256200
All − MR Egger
All − Inverse variance weighted
−2
0
2
4
MR effect size for
'Potatoes liking || id:ebi−fl187−GCST90094816' on 'Crohn's disease of large intestine || id:finngen_R11_CHRONL

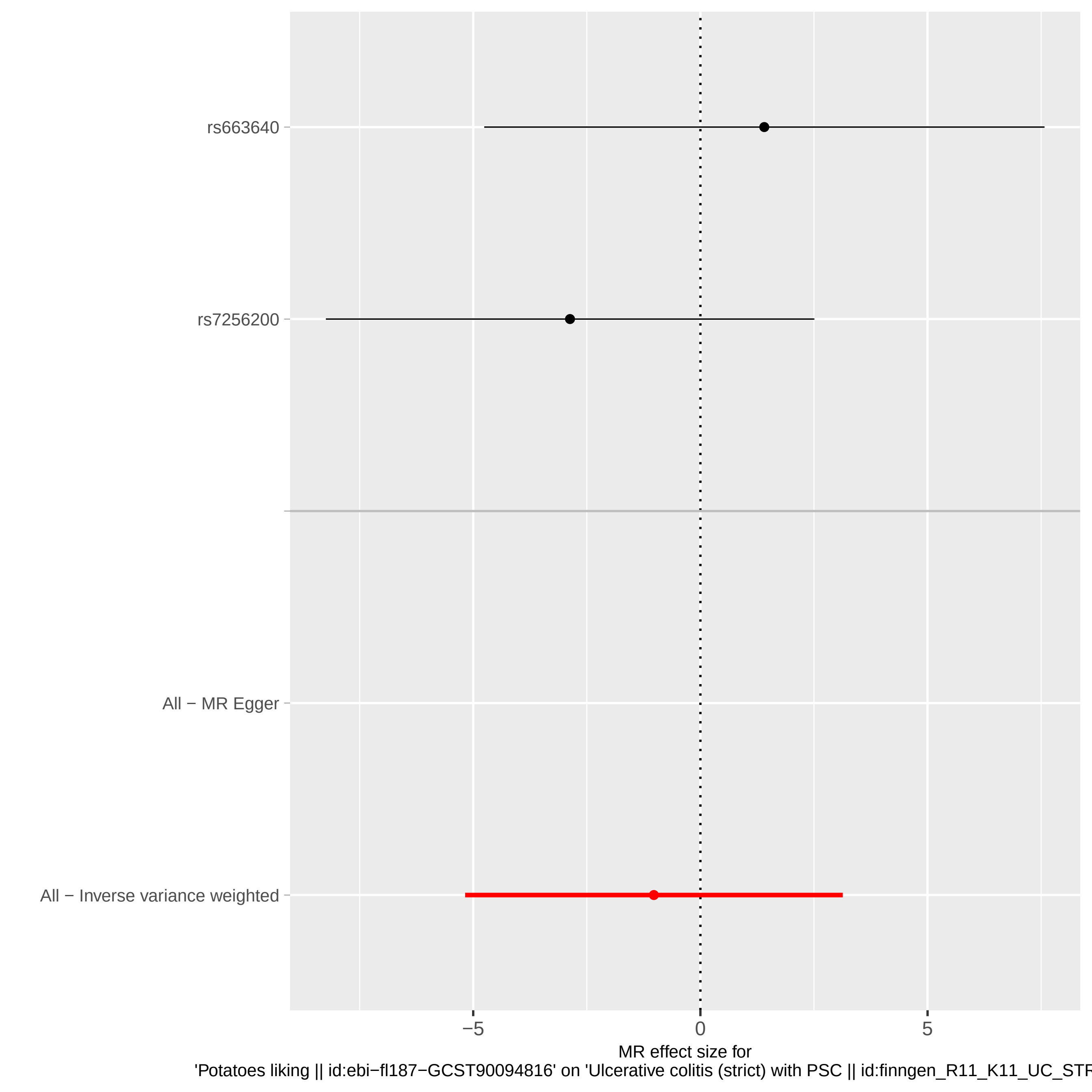
rs663640
rs7256200
All − MR Egger
All − Inverse variance weighted
−5
0
5
MR effect size for
'Potatoes liking || id:ebi−fl187−GCST90094816' on 'Ulcerative colitis (strict) with PSC || id:finngen_R11_K11_UC_STR

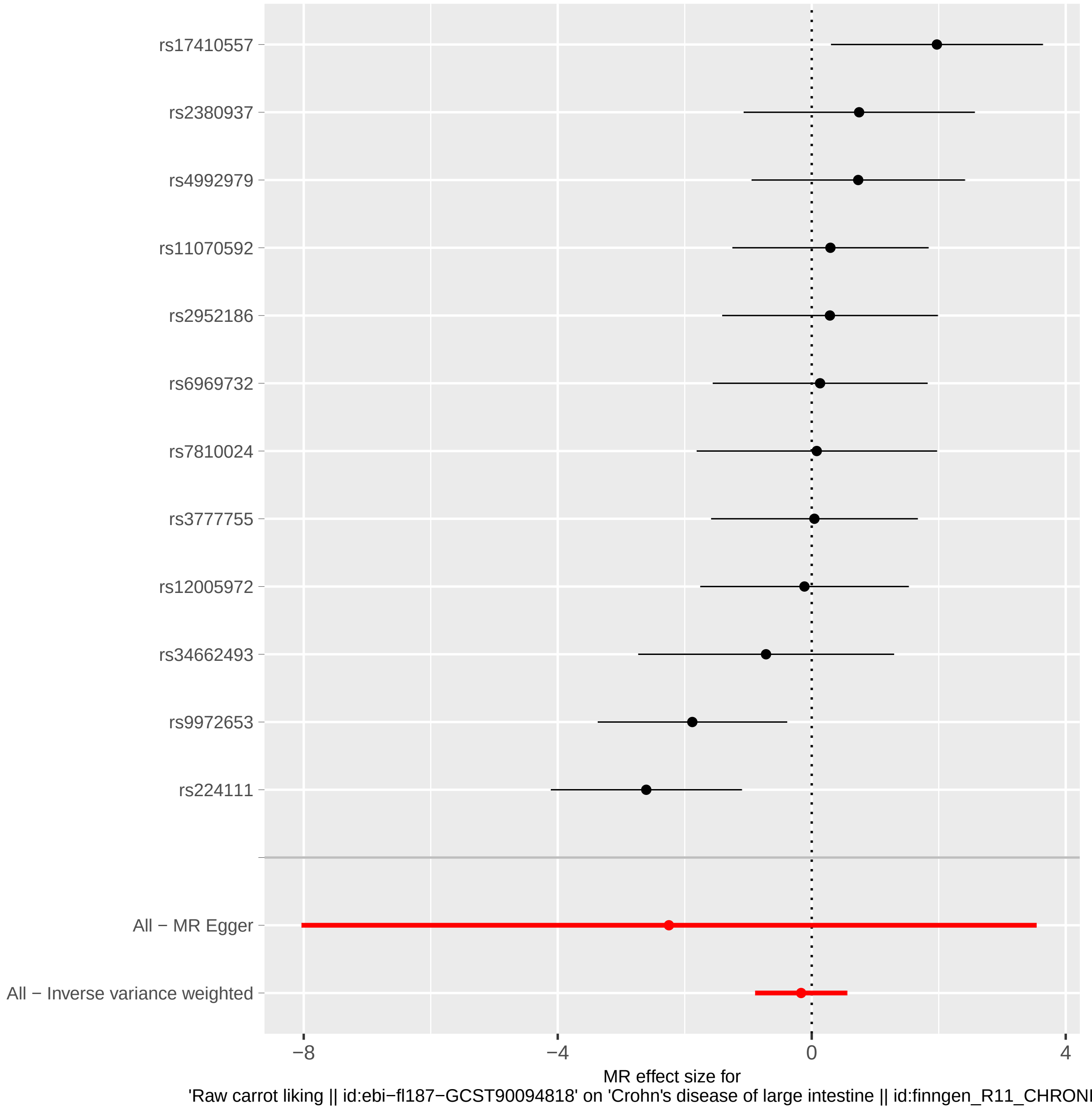
rs17410557
rs2380937
rs4992979
rs11070592
rs2952186
rs6969732
rs7810024
rs3777755
rs12005972
rs34662493
rs9972653
rs224111
All − MR Egger
All − Inverse variance weighted
−8
−4
0
4
MR effect size for
'Raw carrot liking || id:ebi−fl187−GCST90094818' on 'Crohn's disease of large intestine || id:finngen_R11_CHRONI

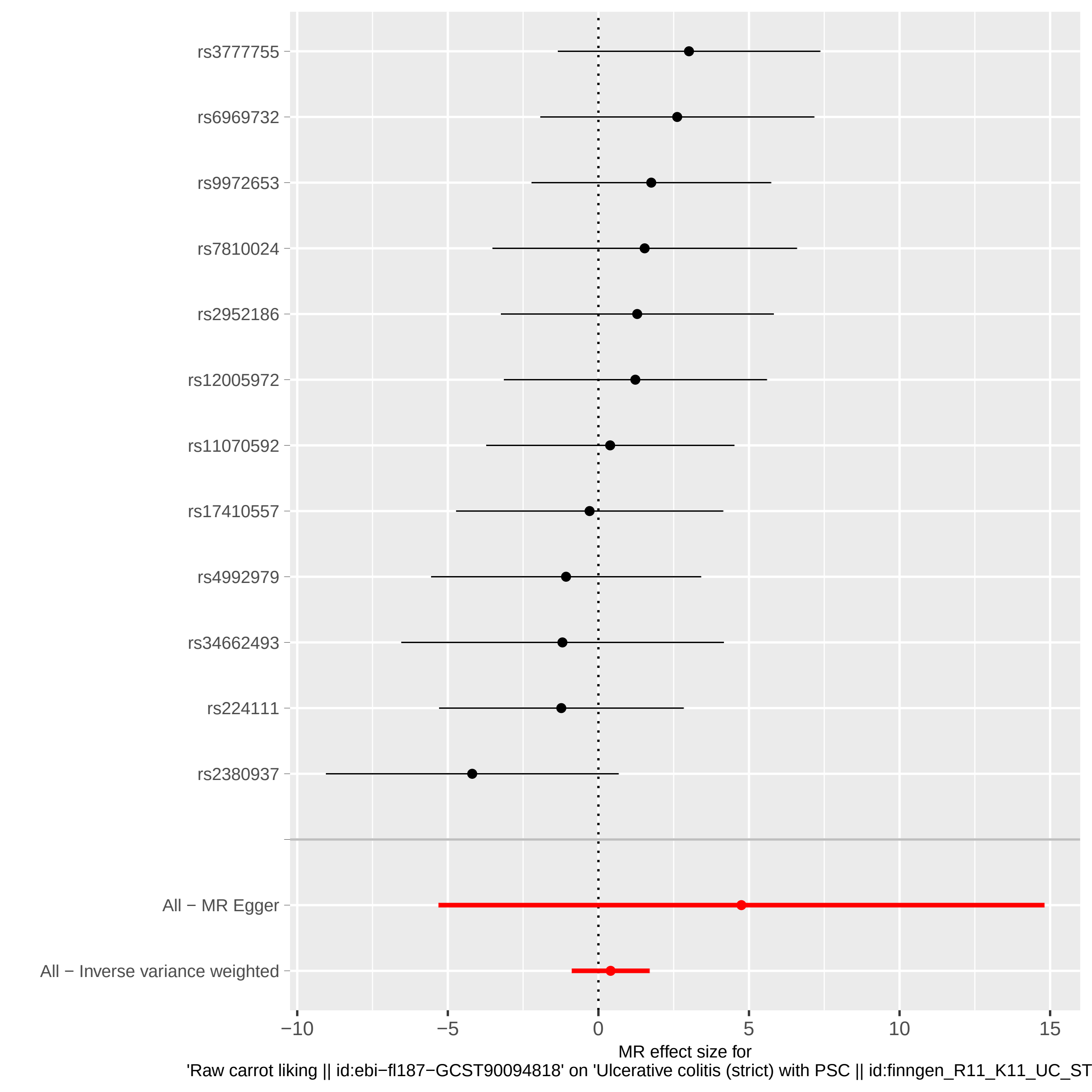
rs3777755
rs6969732
rs9972653
rs7810024
rs2952186
rs12005972
rs11070592
rs17410557
rs4992979
rs34662493
rs224111
rs2380937
All − MR Egger
All − Inverse variance weighted
−10
−5
0
5
10
15
MR effect size for
'Raw carrot liking || id:ebi−fl187−GCST90094818' on 'Ulcerative colitis (strict) with PSC || id:finngen_R11_K11_UC_ST

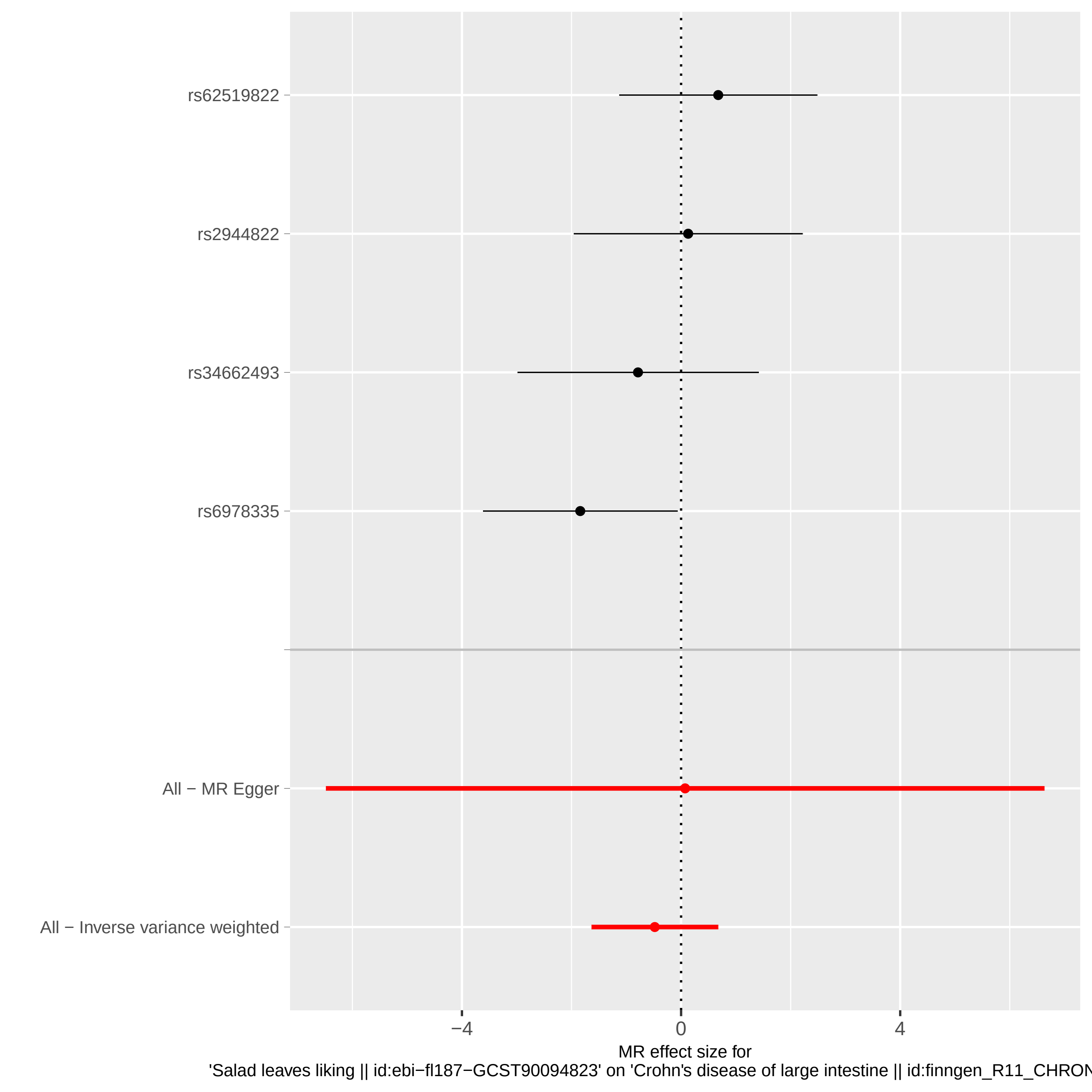
rs62519822
rs2944822
rs34662493
rs6978335
All − MR Egger
All − Inverse variance weighted
−4
0
4
MR effect size for
'Salad leaves liking || id:ebi−fl187−GCST90094823' on 'Crohn's disease of large intestine || id:finngen_R11_CHRON

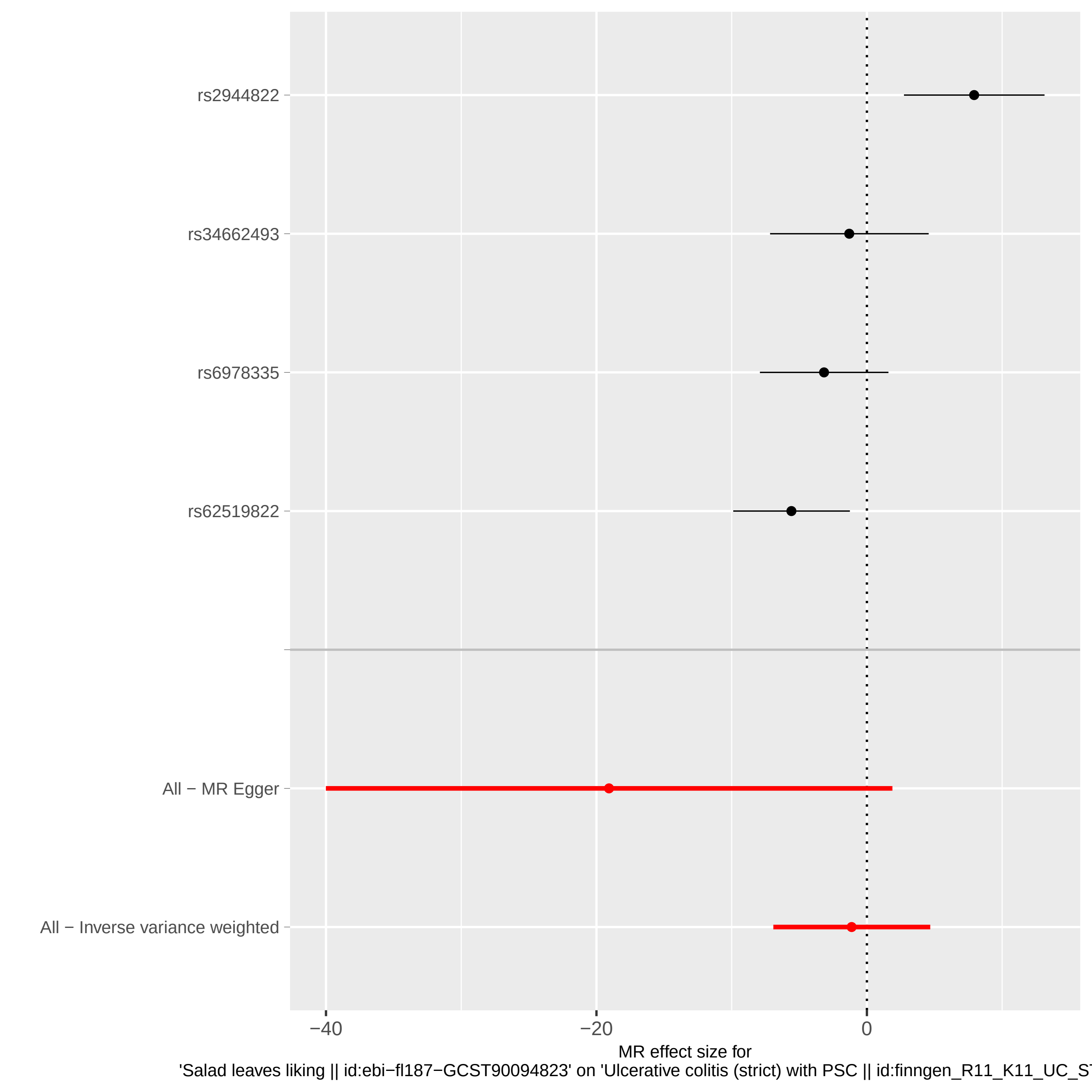
rs2944822
rs34662493
rs6978335
rs62519822
All − MR Egger
All − Inverse variance weighted
−40
−20
0
MR effect size for
'Salad leaves liking || id:ebi−fl187−GCST90094823' on 'Ulcerative colitis (strict) with PSC || id:finngen_R11_K11_UC_ST

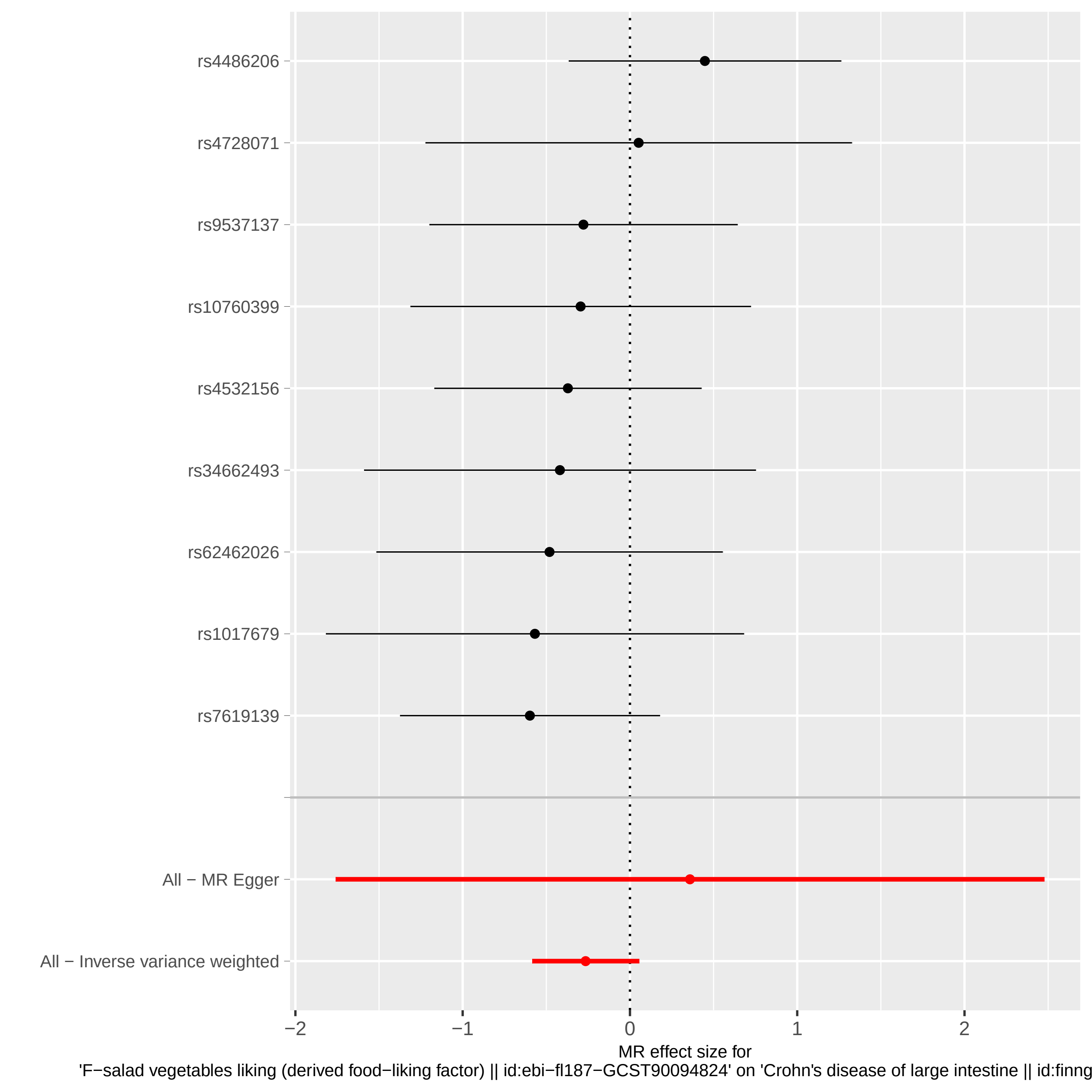
rs4486206
rs4728071
rs9537137
rs10760399
rs4532156
rs34662493
rs62462026
rs1017679
rs7619139
All − MR Egger
All − Inverse variance weighted
−2
−1
0
1
2
MR effect size for
'F−salad vegetables liking (derived food−liking factor) || id:ebi−fl187−GCST90094824' on 'Crohn's disease of large intestine || id:finng

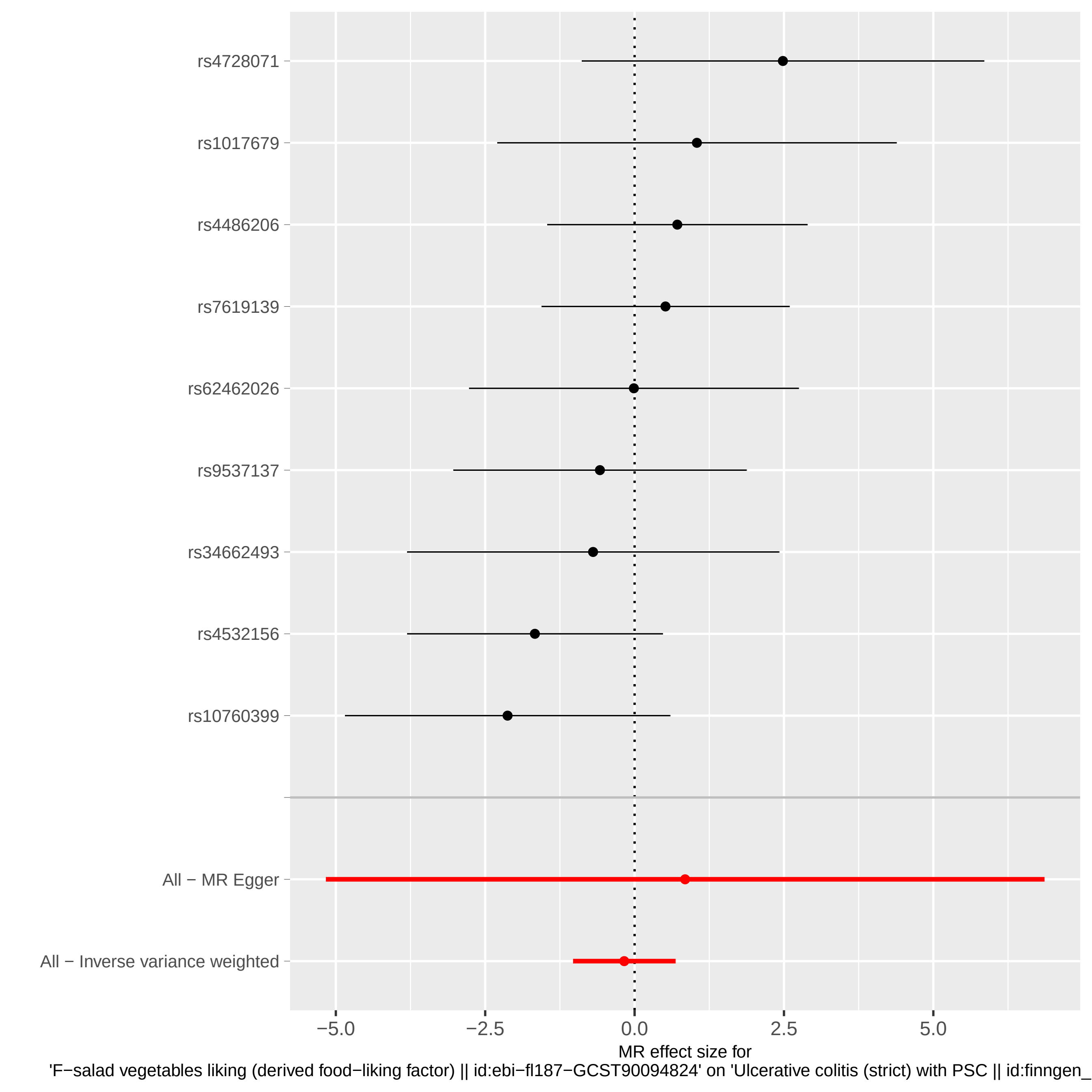
rs4728071
rs1017679
rs4486206
rs7619139
rs62462026
rs9537137
rs34662493
rs4532156
rs10760399
All − MR Egger
All − Inverse variance weighted
−5.0
−2.5
0.0
2.5
5.0
MR effect size for
'F−salad vegetables liking (derived food−liking factor) || id:ebi−fl187−GCST90094824' on 'Ulcerative colitis (strict) with PSC || id:finngen_

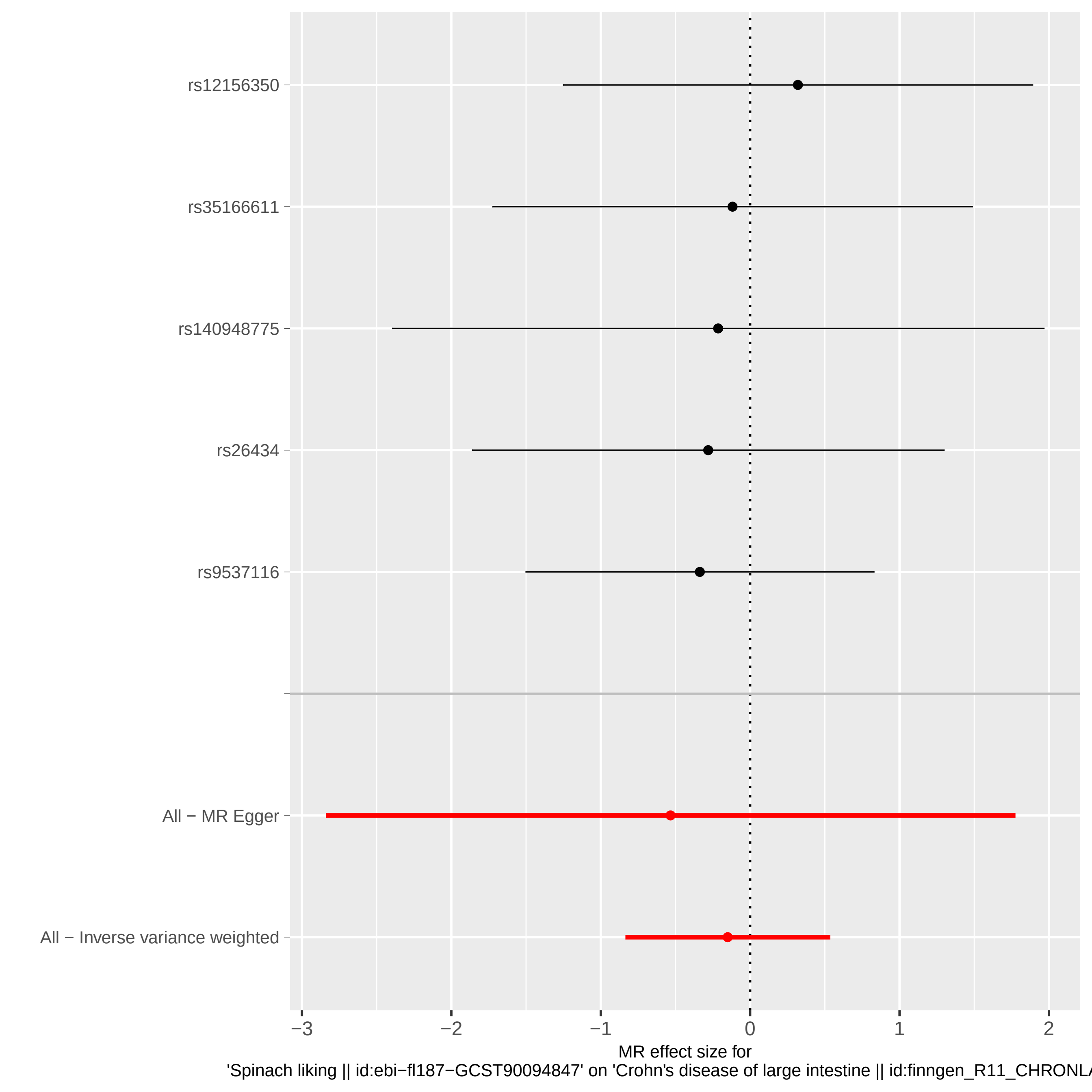
rs12156350
rs35166611
rs140948775
rs26434
rs9537116
All − MR Egger
All − Inverse variance weighted
−3
−2
−1
0
1
2
MR effect size for
'Spinach liking || id:ebi−fl187−GCST90094847' on 'Crohn's disease of large intestine || id:finngen_R11_CHRONL

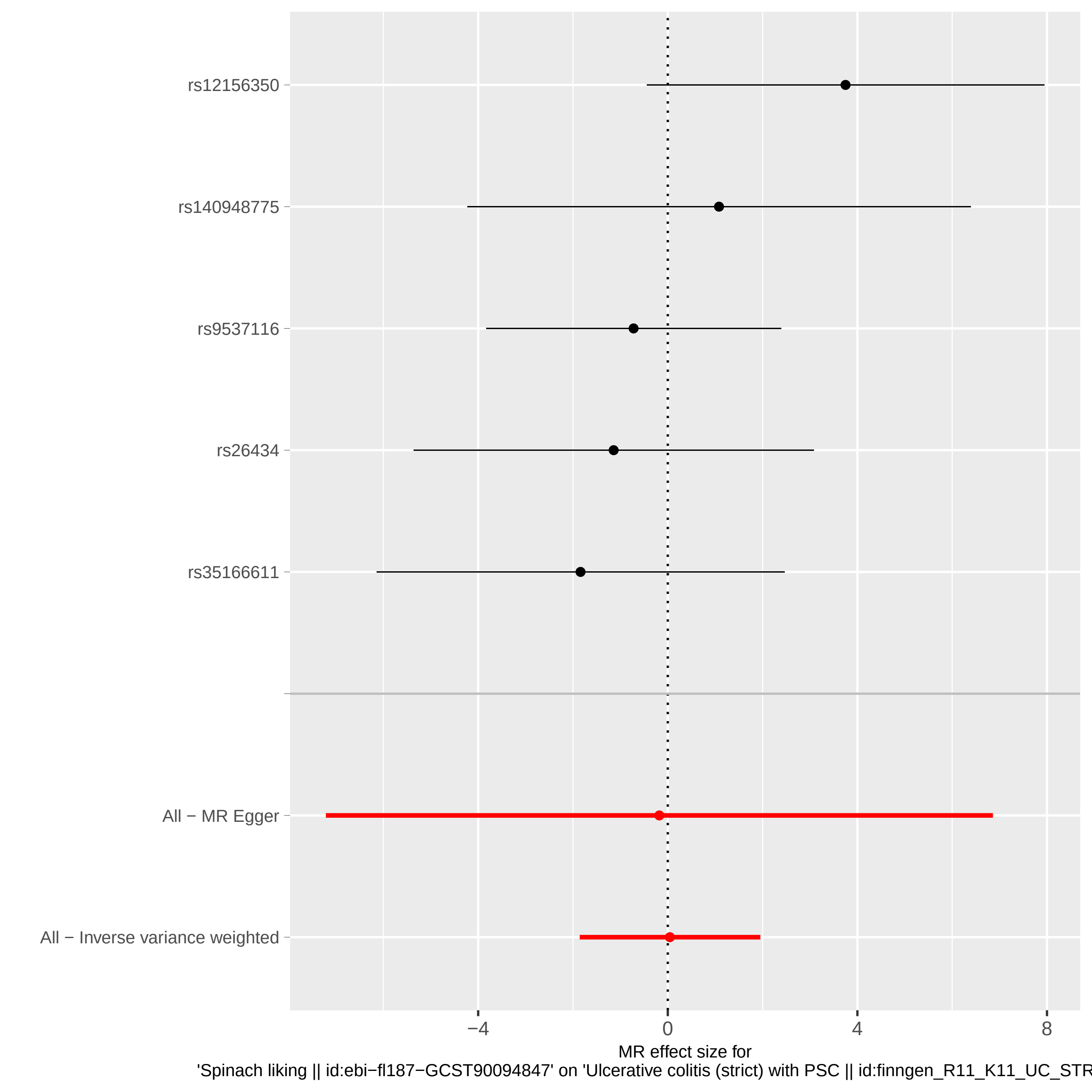
rs12156350
rs140948775
rs9537116
rs26434
rs35166611
All − MR Egger
All − Inverse variance weighted
−4
0
4
8
MR effect size for
'Spinach liking || id:ebi−fl187−GCST90094847' on 'Ulcerative colitis (strict) with PSC || id:finngen_R11_K11_UC_STR

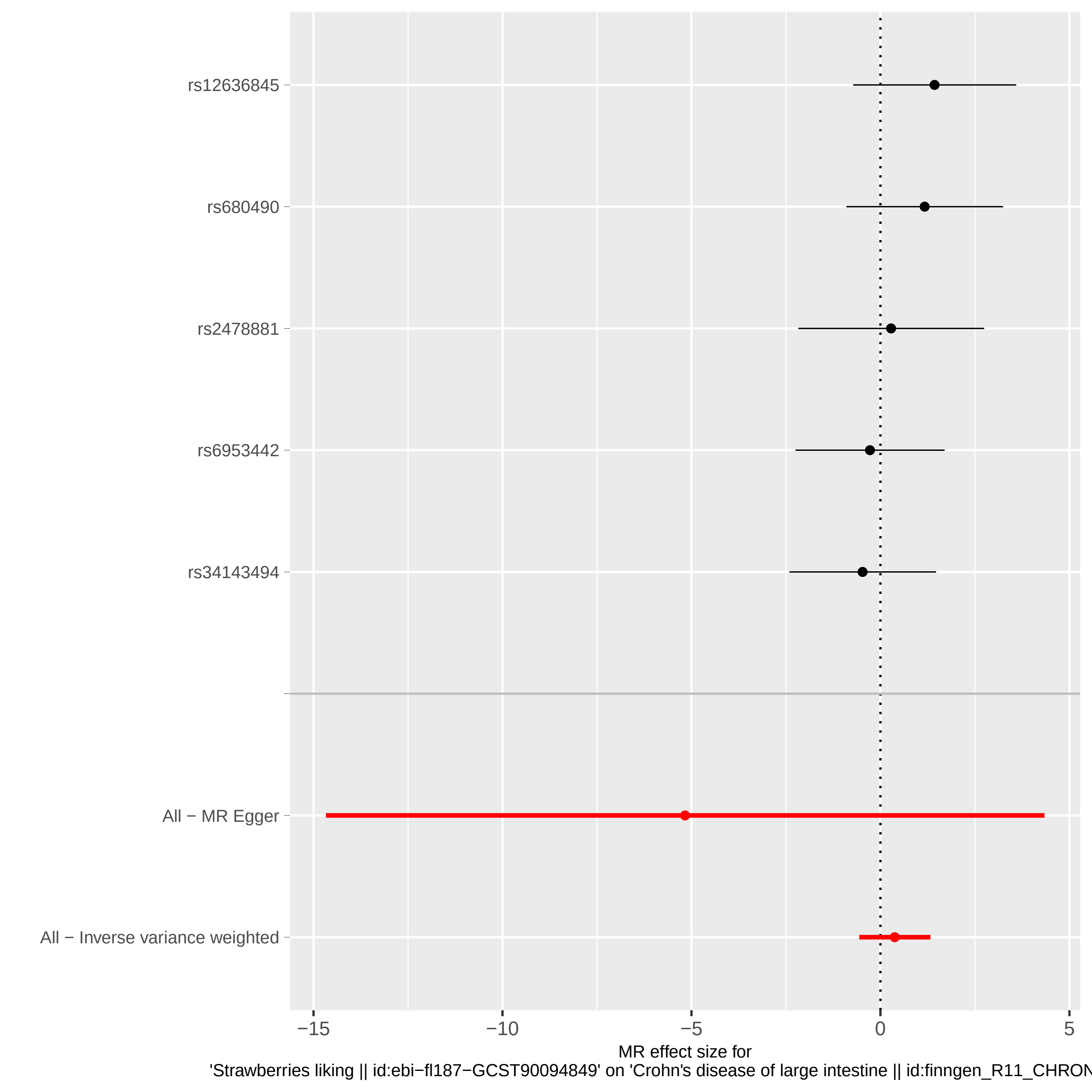
rs12636845
rs680490
rs2478881
rs6953442
rs34143494
All − MR Egger
All − Inverse variance weighted
−15
−10
−5
0
5
MR effect size for
'Strawberries liking || id:ebi−fl187−GCST90094849' on 'Crohn's disease of large intestine || id:finngen_R11_CHRON

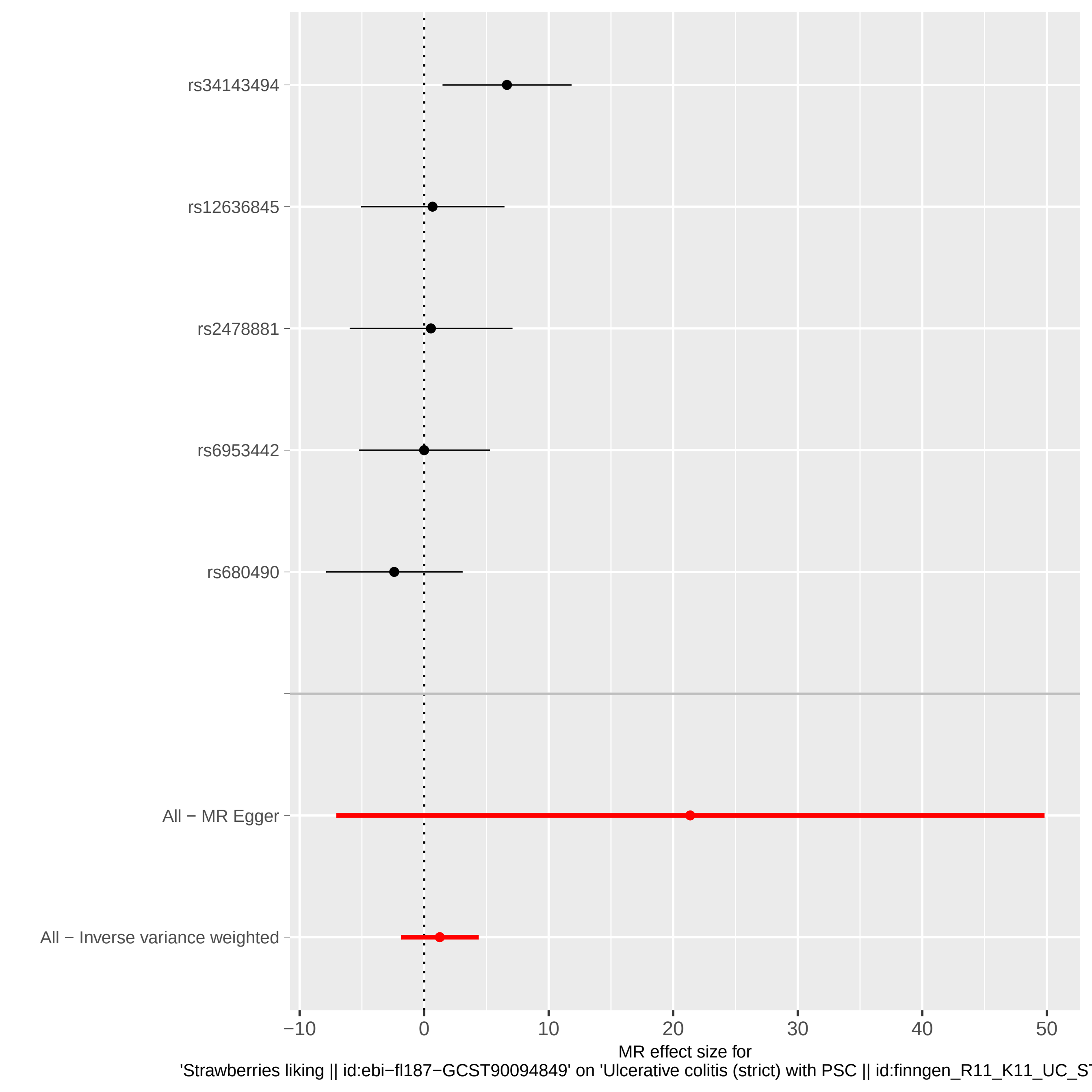
rs34143494
rs12636845
rs2478881
rs6953442
rs680490
All − MR Egger
All − Inverse variance weighted
−10
0
10
20
30
40
50
MR effect size for
'Strawberries liking || id:ebi−fl187−GCST90094849' on 'Ulcerative colitis (strict) with PSC || id:finngen_R11_K11_UC_ST

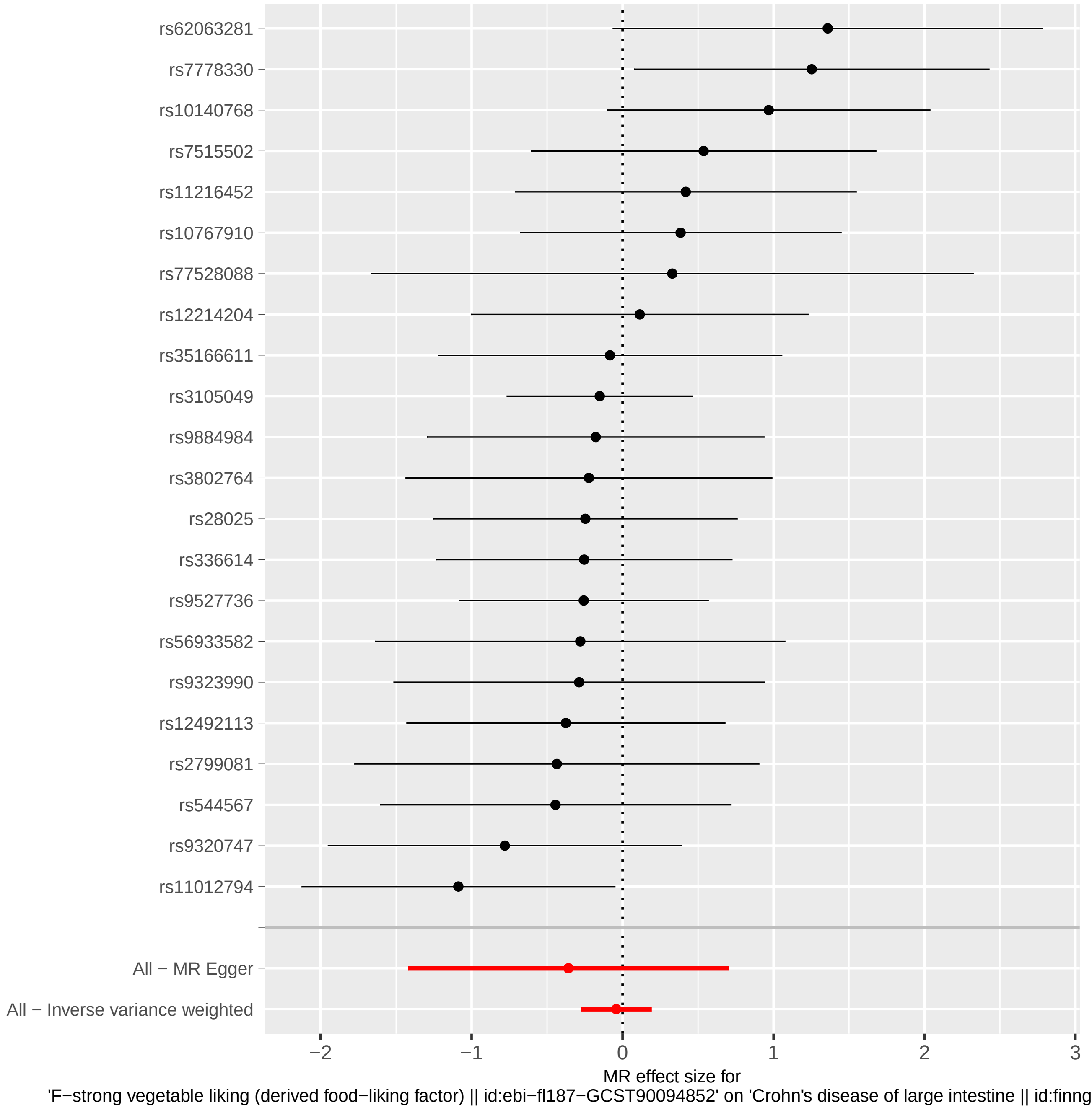
rs62063281
rs7778330
rs10140768
rs7515502
rs11216452
rs10767910
rs77528088
rs12214204
rs35166611
rs3105049
rs9884984
rs3802764
rs28025
rs336614
rs9527736
rs56933582
rs9323990
rs12492113
rs2799081
rs544567
rs9320747
rs11012794
All − MR Egger
All − Inverse variance weighted
−2
−1
0
1
2
3
MR effect size for
'F−strong vegetable liking (derived food−liking factor) || id:ebi−fl187−GCST90094852' on 'Crohn's disease of large intestine || id:finng

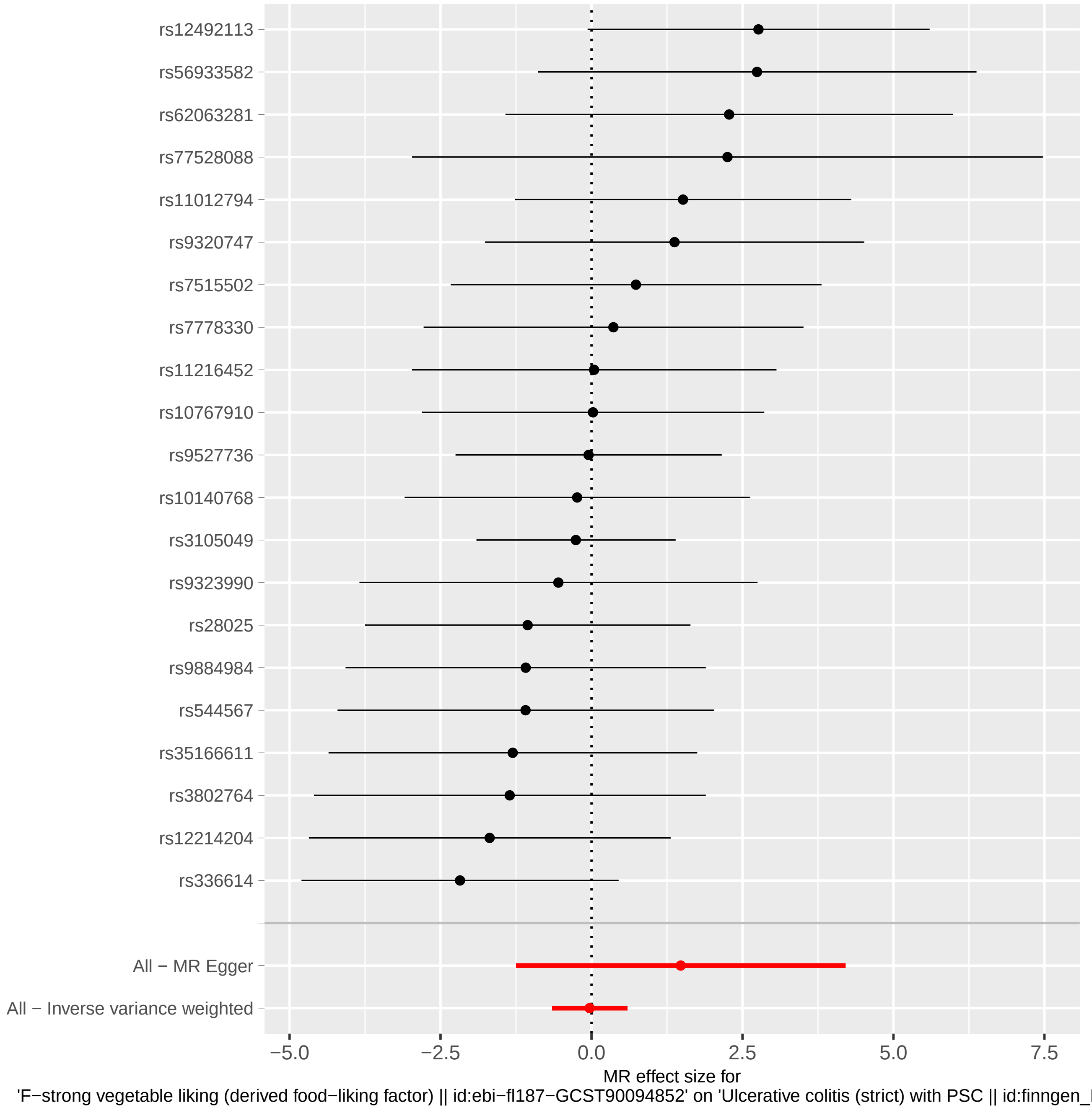
rs12492113
rs56933582
rs62063281
rs77528088
rs11012794
rs9320747
rs7515502
rs7778330
rs11216452
rs10767910
rs9527736
rs10140768
rs3105049
rs9323990
rs28025
rs9884984
rs544567
rs35166611
rs3802764
rs12214204
rs336614
All − MR Egger
All − Inverse variance weighted
−5.0
−2.5
0.0
2.5
5.0
7.5
MR effect size for
'F−strong vegetable liking (derived food−liking factor) || id:ebi−fl187−GCST90094852' on 'Ulcerative colitis (strict) with PSC || id:finngen_

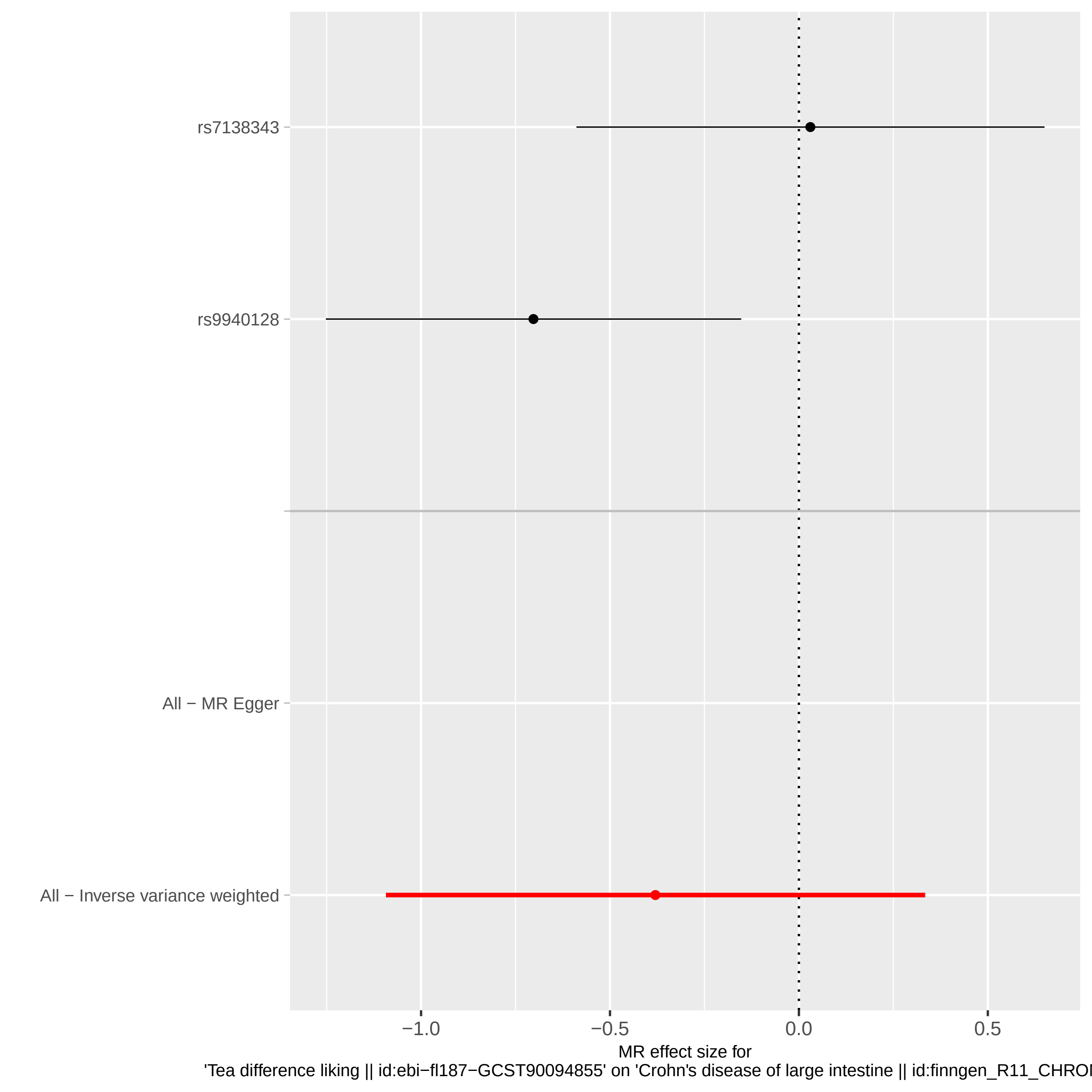
rs7138343
rs9940128
All − MR Egger
All − Inverse variance weighted
−1.0
−0.5
0.0
0.5
MR effect size for
'Tea difference liking || id:ebi−fl187−GCST90094855' on 'Crohn's disease of large intestine || id:finngen_R11_CHRO

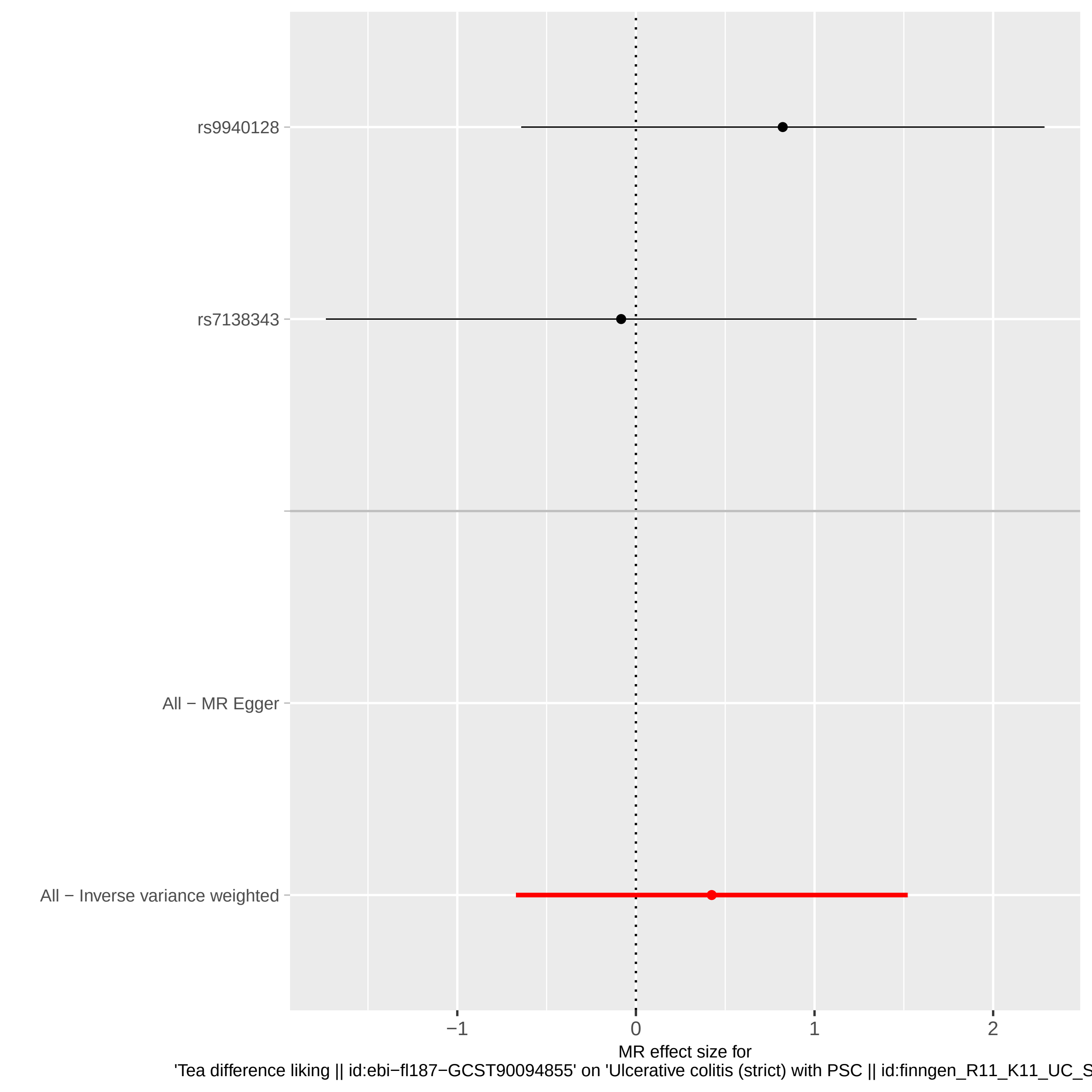
rs9940128
rs7138343
All − MR Egger
All − Inverse variance weighted
−1
0
1
2
MR effect size for
'Tea difference liking || id:ebi−fl187−GCST90094855' on 'Ulcerative colitis (strict) with PSC || id:finngen_R11_K11_UC_S

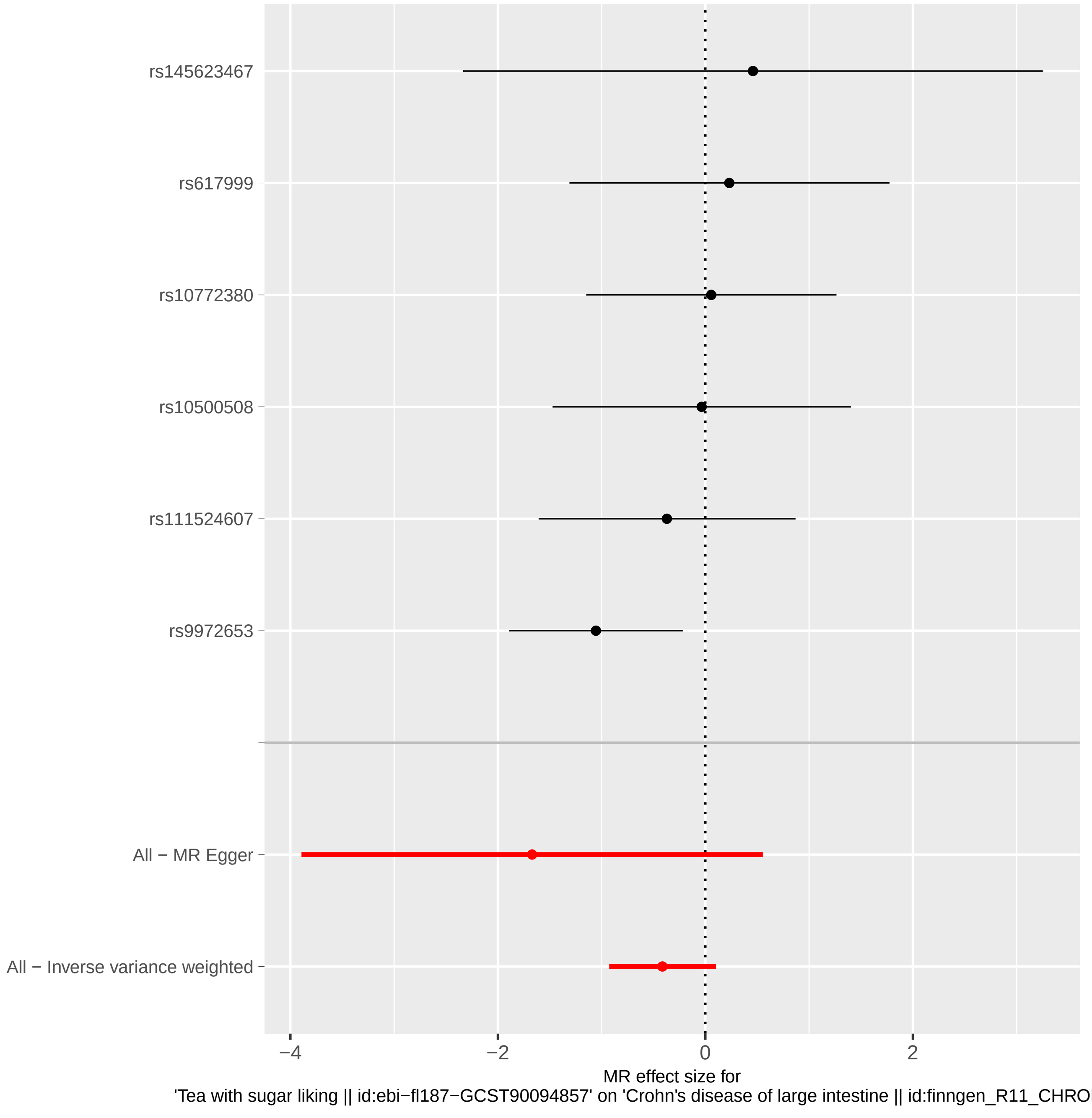
rs145623467
rs617999
rs10772380
rs10500508
rs111524607
rs9972653
All − MR Egger
All − Inverse variance weighted
−4
−2
0
2
MR effect size for
'Tea with sugar liking || id:ebi−fl187−GCST90094857' on 'Crohn's disease of large intestine || id:finngen_R11_CHRO

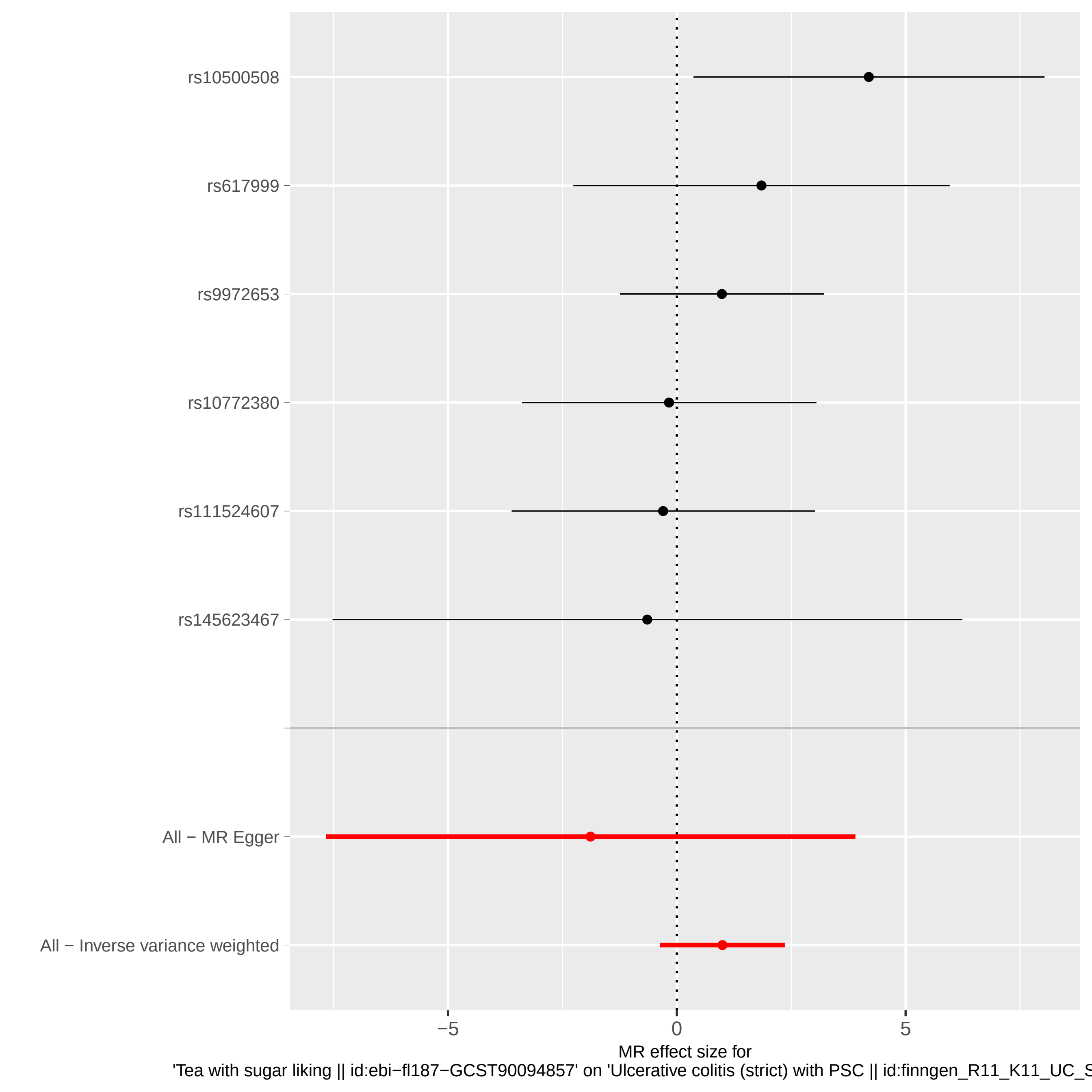
rs10500508
rs617999
rs9972653
rs10772380
rs111524607
rs145623467
All − MR Egger
All − Inverse variance weighted
−5
0
5
MR effect size for
'Tea with sugar liking || id:ebi−fl187−GCST90094857' on 'Ulcerative colitis (strict) with PSC || id:finngen_R11_K11_UC_S

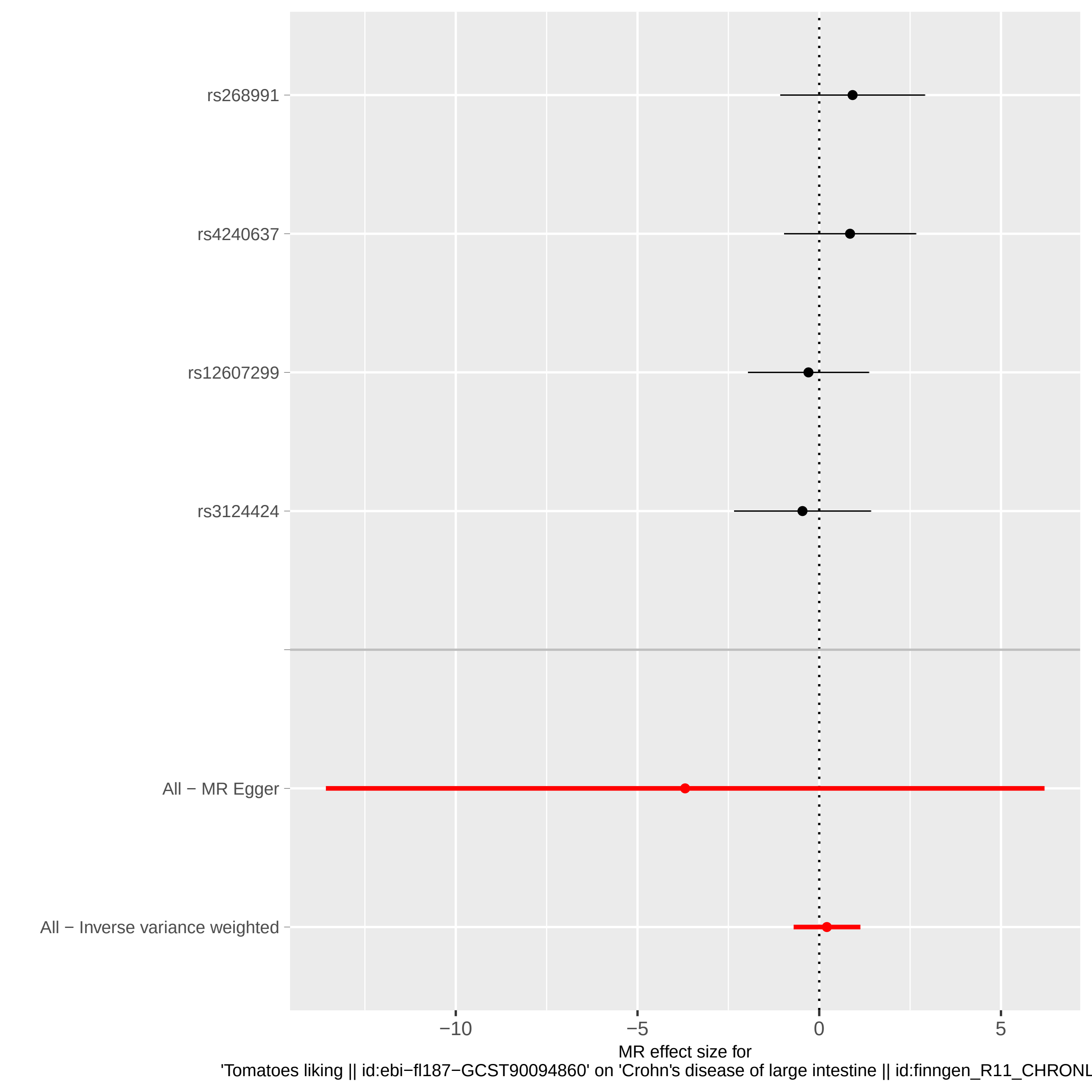

rs268991
rs4240637
rs12607299
rs3124424
All − MR Egger
All − Inverse variance weighted
−10
−5
0
5
MR effect size for
'Tomatoes liking || id:ebi−fl187−GCST90094860' on 'Crohn's disease of large intestine || id:finngen_R11_CHRONL

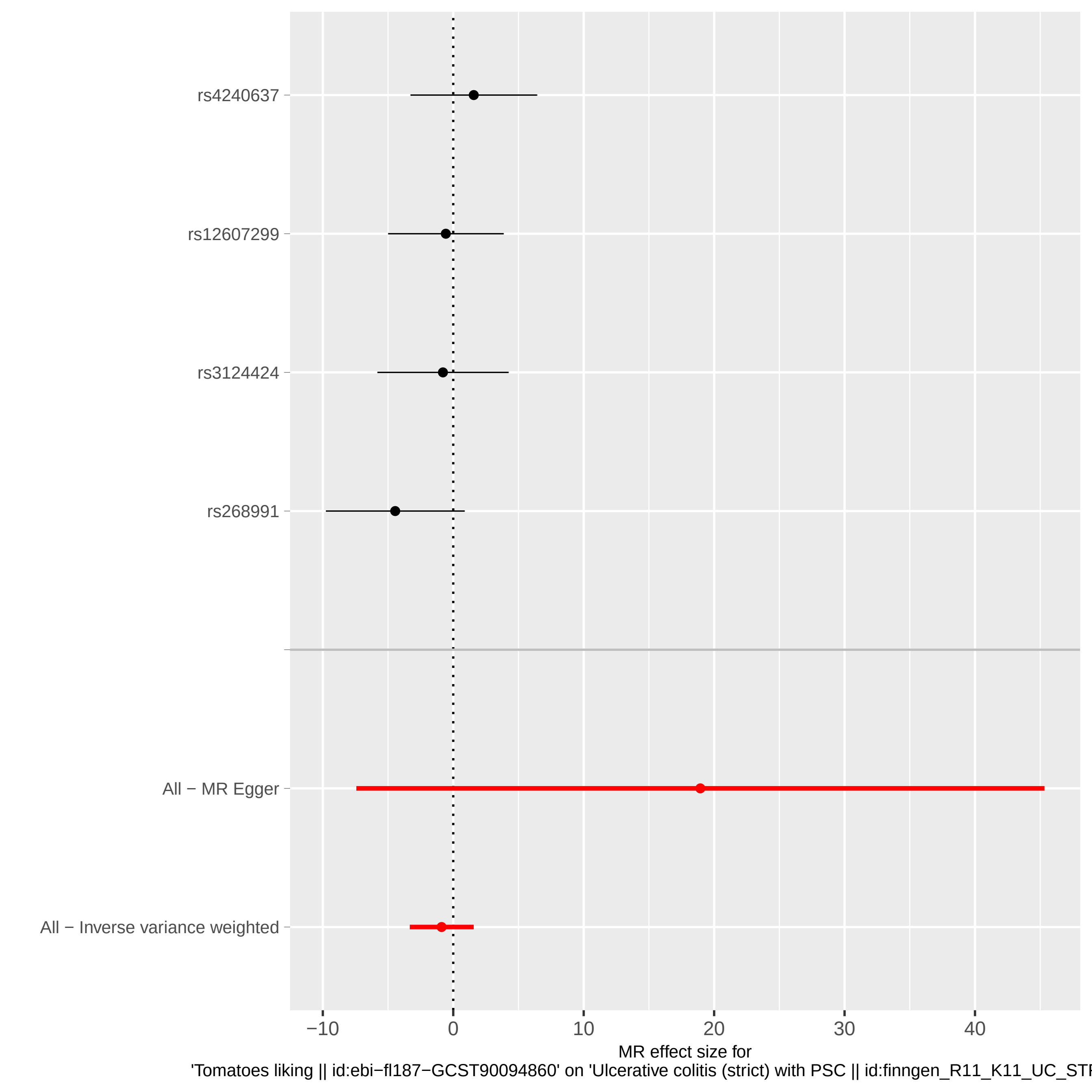
rs4240637
rs12607299
rs3124424
rs268991
All − MR Egger
All − Inverse variance weighted
−10
0
10
20
30
40
MR effect size for
'Tomatoes liking || id:ebi−fl187−GCST90094860' on 'Ulcerative colitis (strict) with PSC || id:finngen_R11_K11_UC_STR

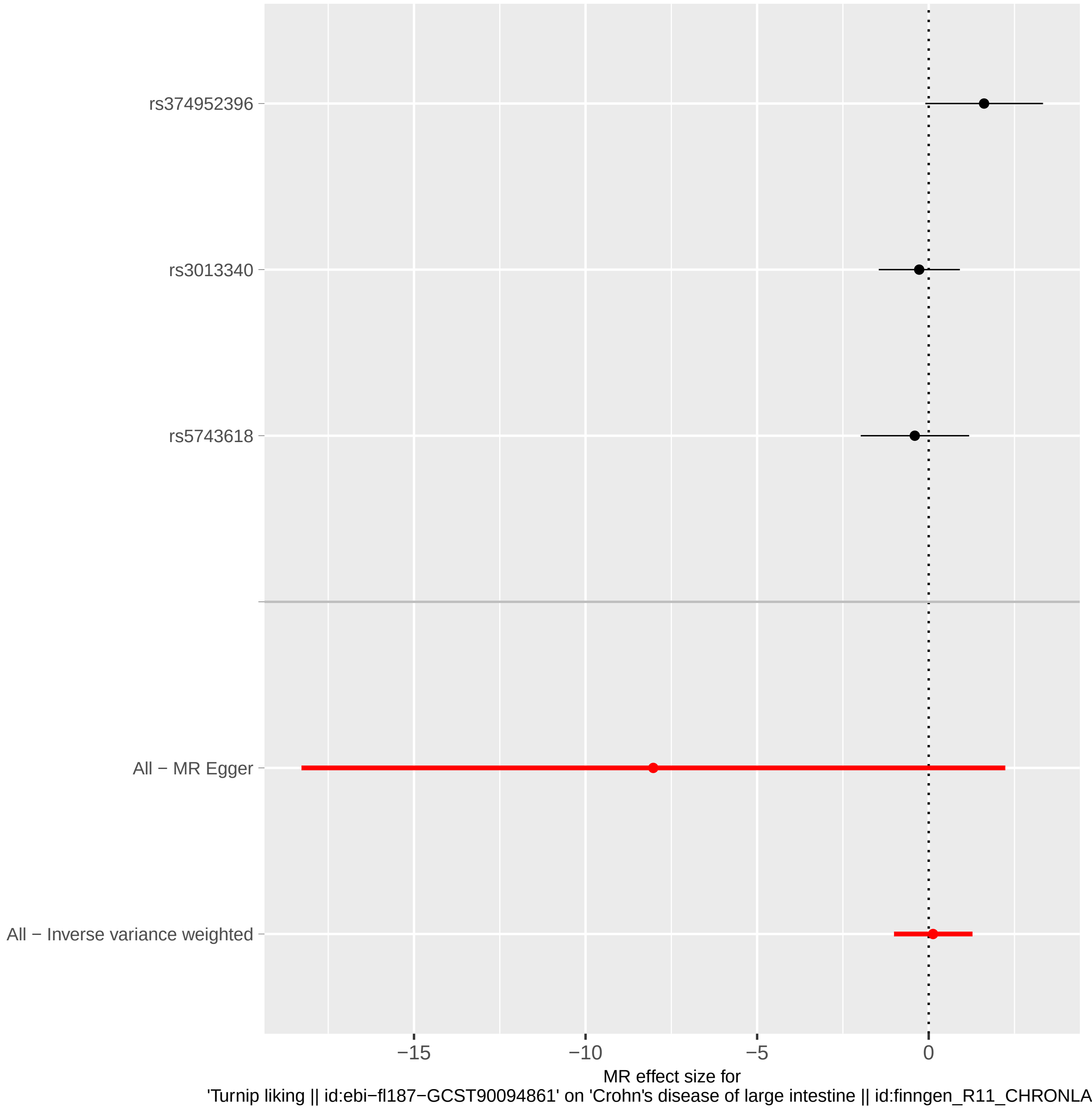

rs374952396
rs3013340
rs5743618
All − MR Egger
All − Inverse variance weighted
−15
−10
−5
0
MR effect size for
'Turnip liking || id:ebi−fl187−GCST90094861' on 'Crohn's disease of large intestine || id:finngen_R11_CHRONLA

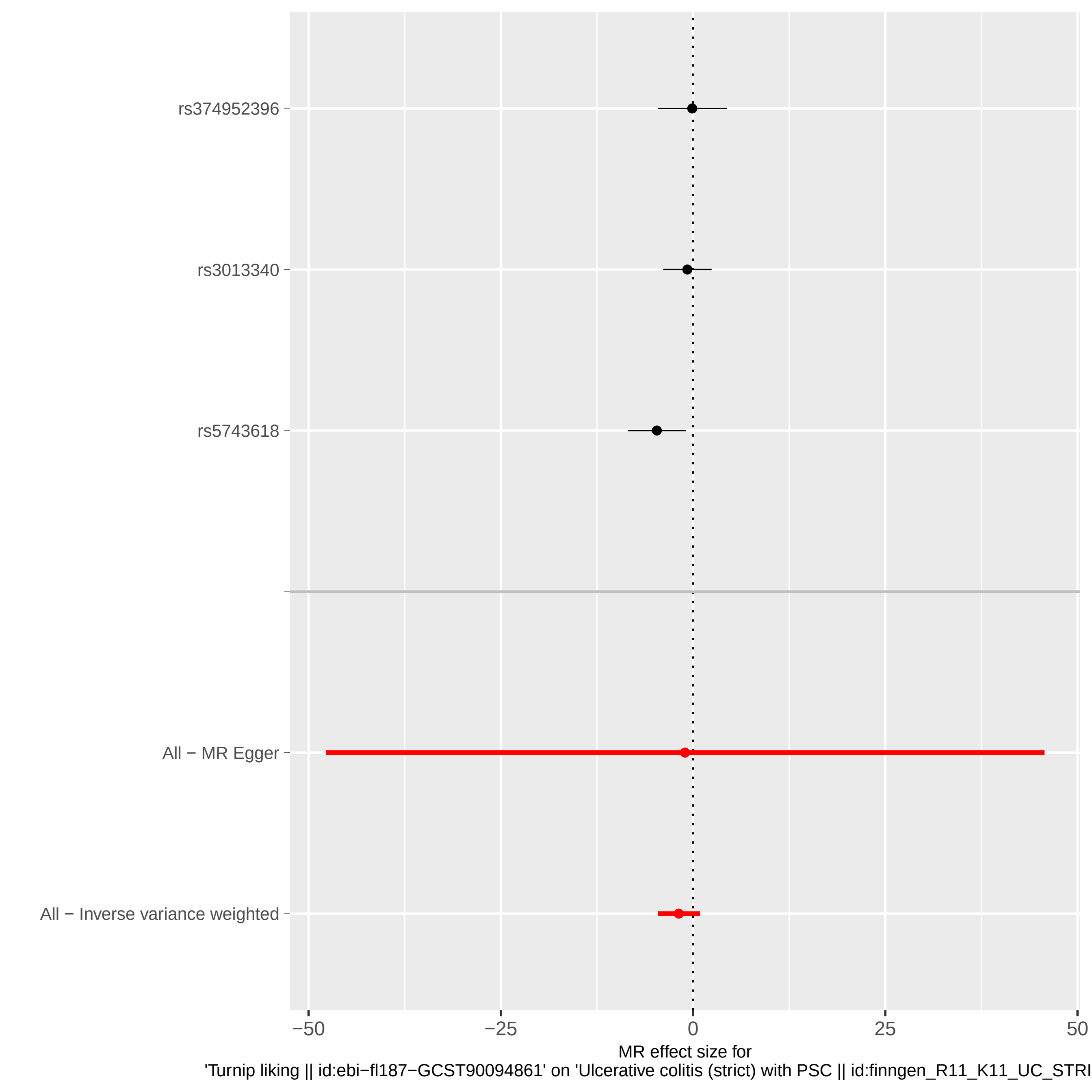

rs374952396
rs3013340
rs5743618
All − MR Egger
All − Inverse variance weighted
−50
−25
0
25
50
MR effect size for
'Turnip liking || id:ebi−fl187−GCST90094861' on 'Ulcerative colitis (strict) with PSC || id:finngen_R11_K11_UC_STRI

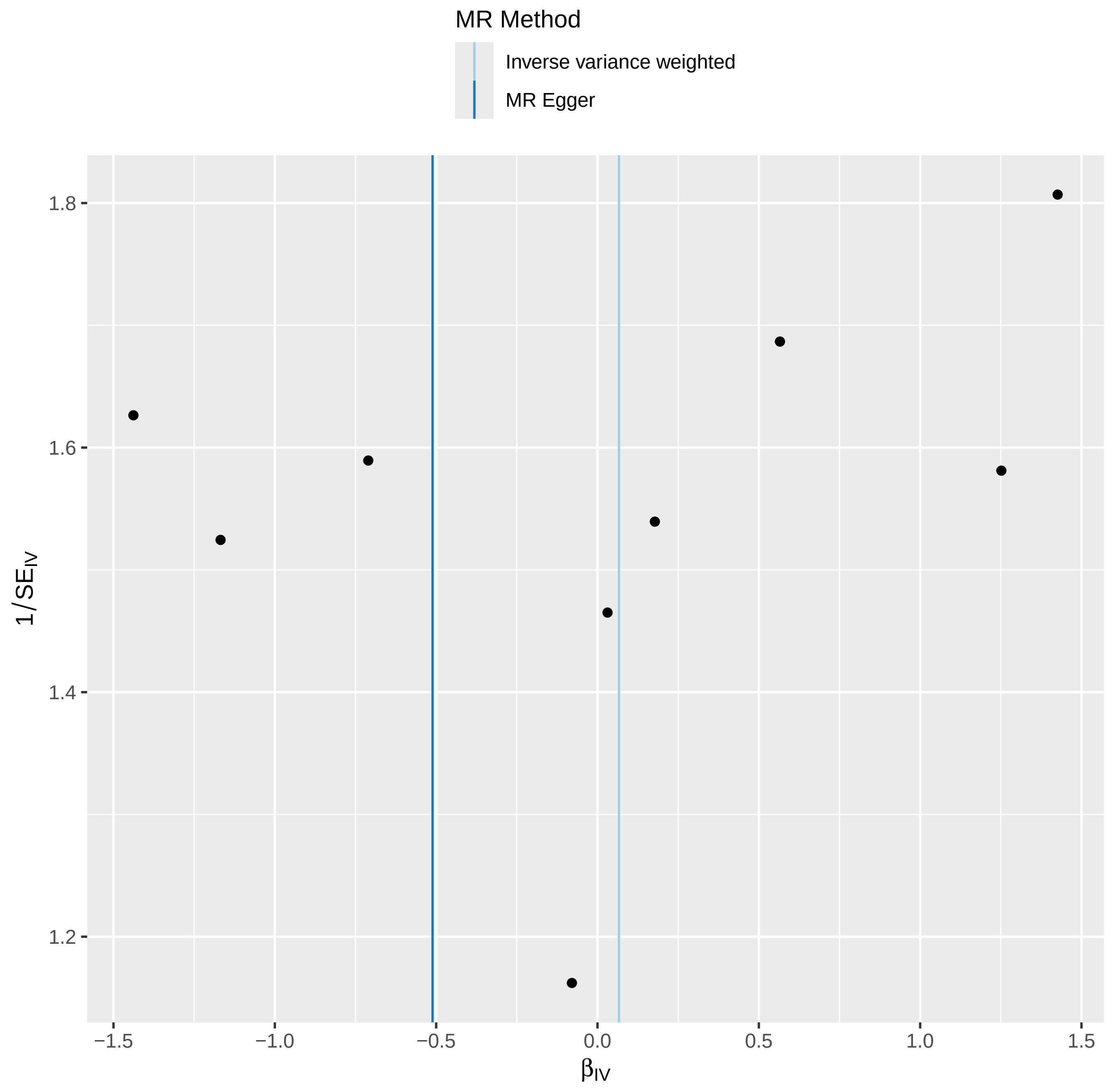
MR Method
Inverse variance weighted
MR Egger
1.8
1.6
1.4
1.2
1/SE_IV
−1.5
−1.0
−0.5
0.0
0.5
1.0
1.5
β_IV

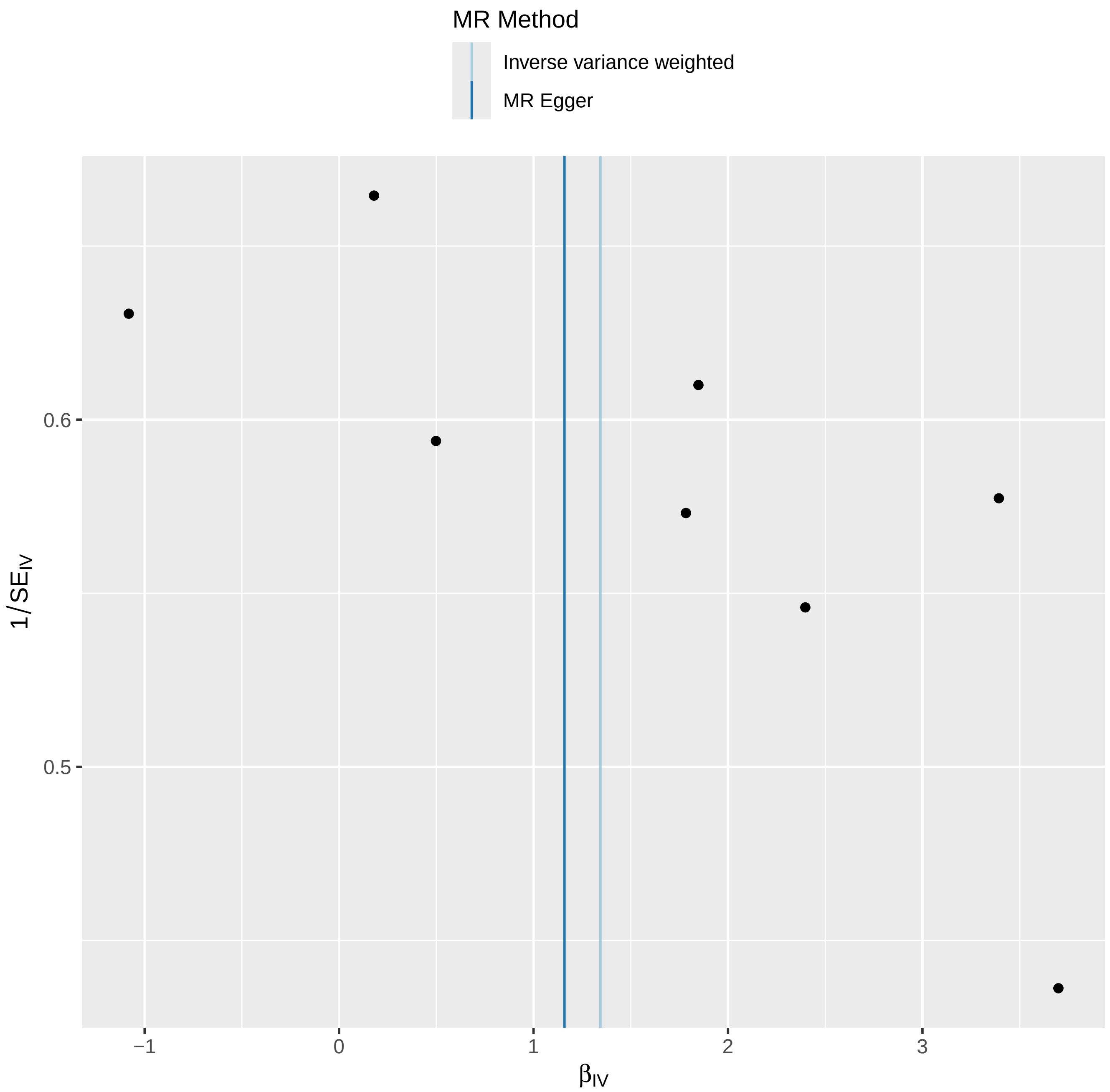

MR Method
Inverse variance weighted
MR Egger
0.6
0.5
1/SE_IV
−1
0
1
2
3
β_IV

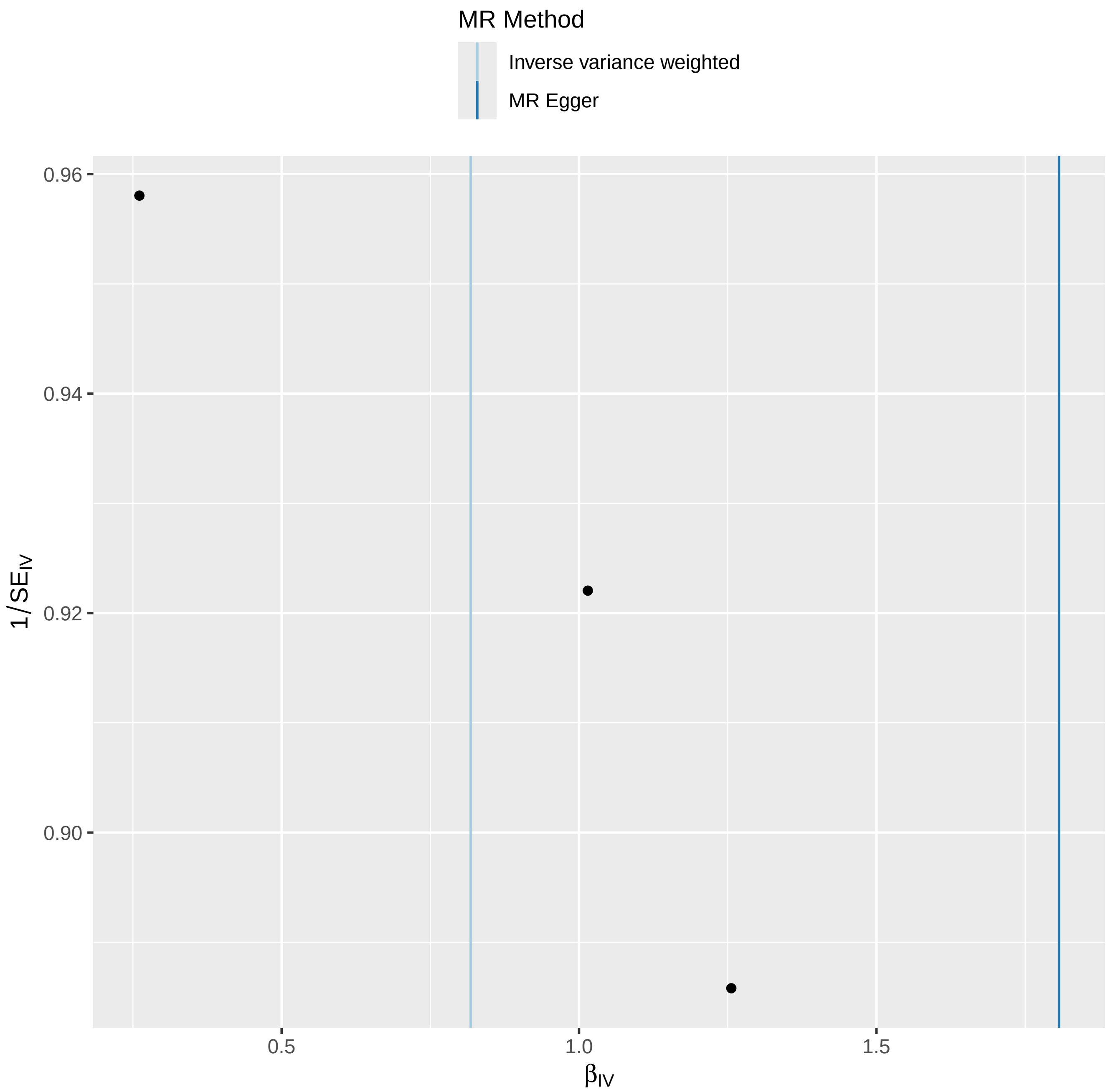
MR Method
Inverse variance weighted
MR Egger
0.96
0.94
0.92
0.90
1/SE_IV
0.5
1.0
1.5
β_IV

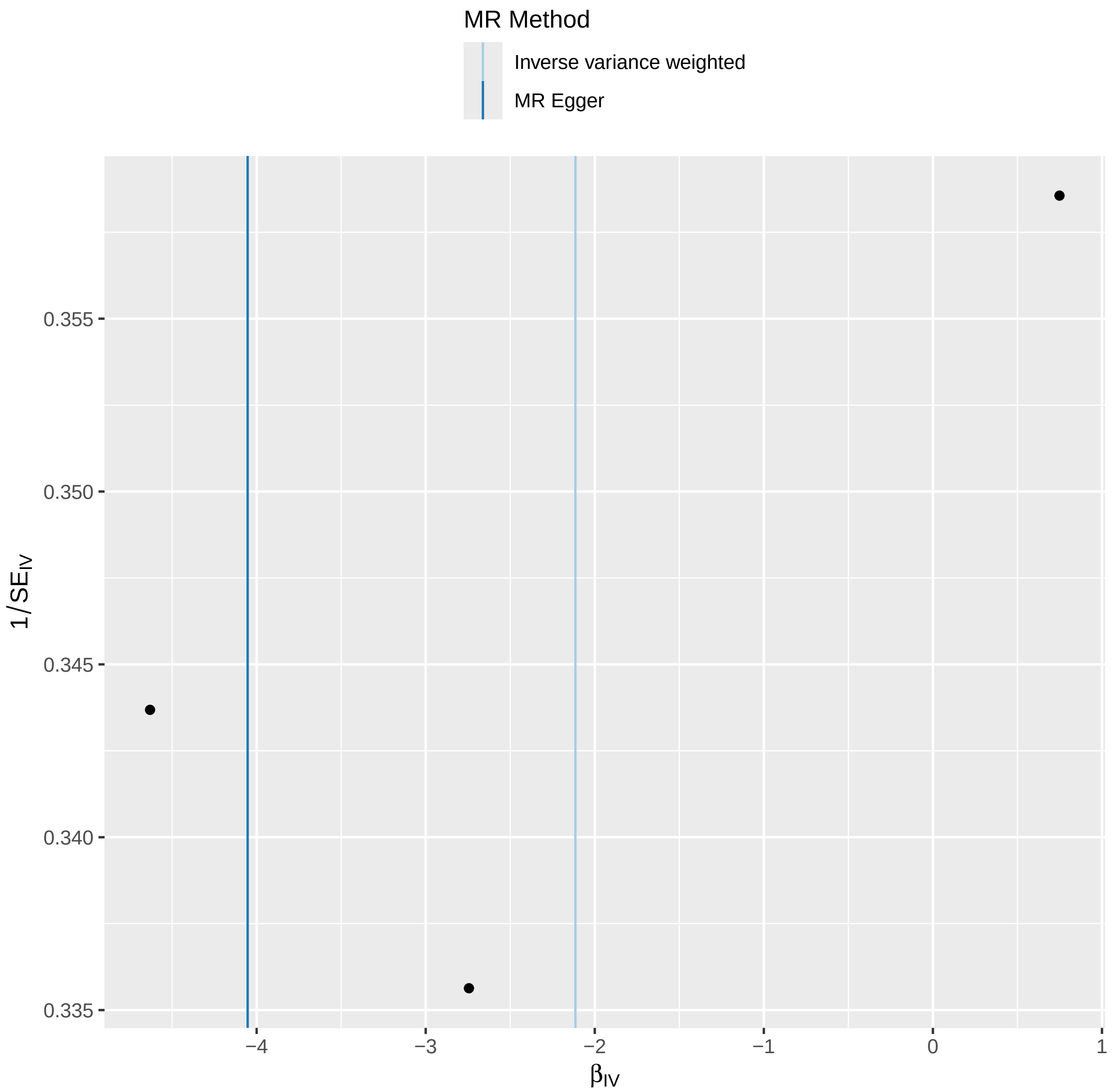
MR Method
Inverse variance weighted
MR Egger
0.355
0.350
0.345
0.340
0.335
1/SE_IV
−4
−3
−2
−1
0
1
β_IV

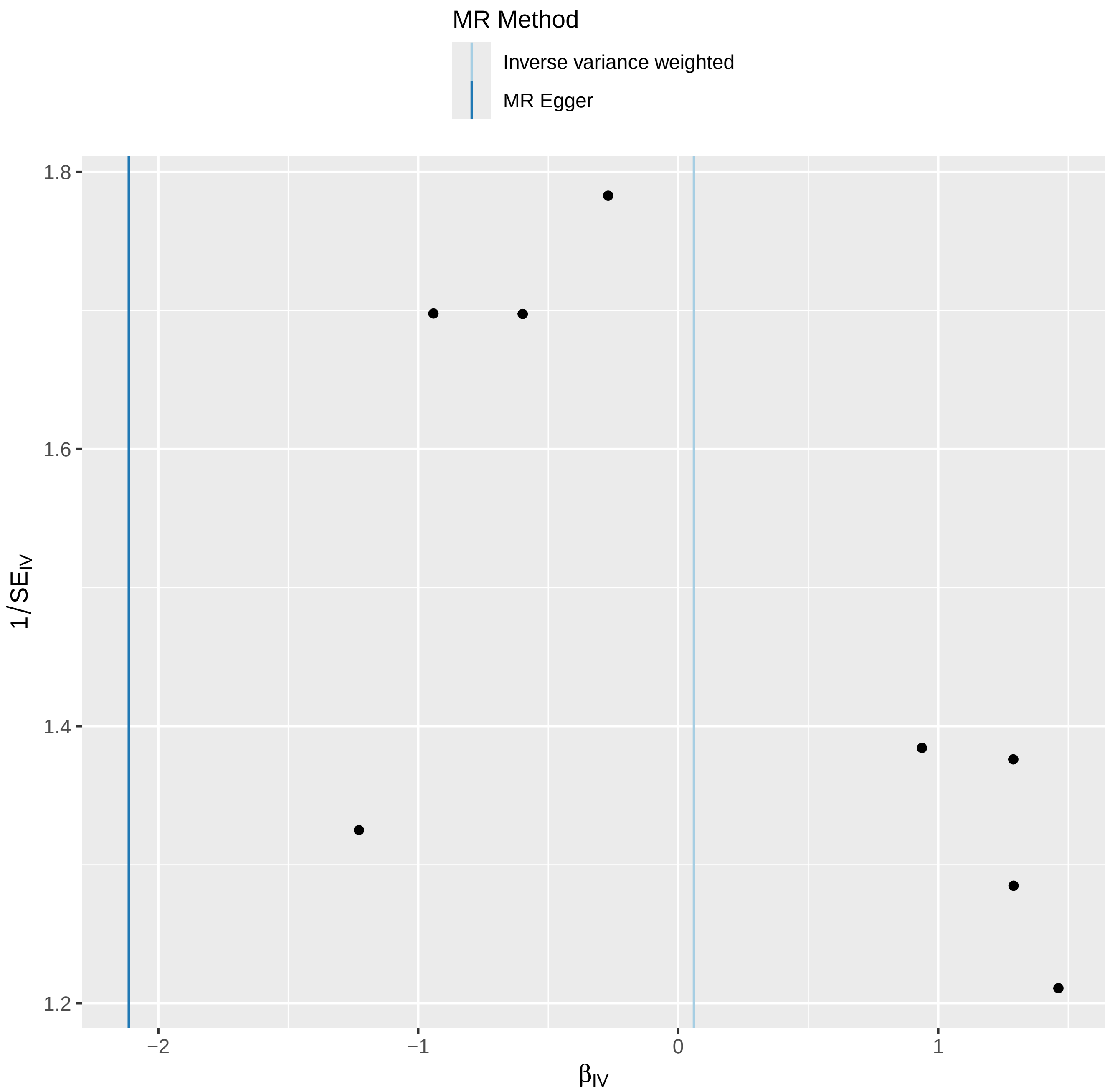

MR Method
Inverse variance weighted
MR Egger
1.8
1.6
1.4
1.2
−2
−1
0
1
$1/SE_{IV}$
β_{IV}

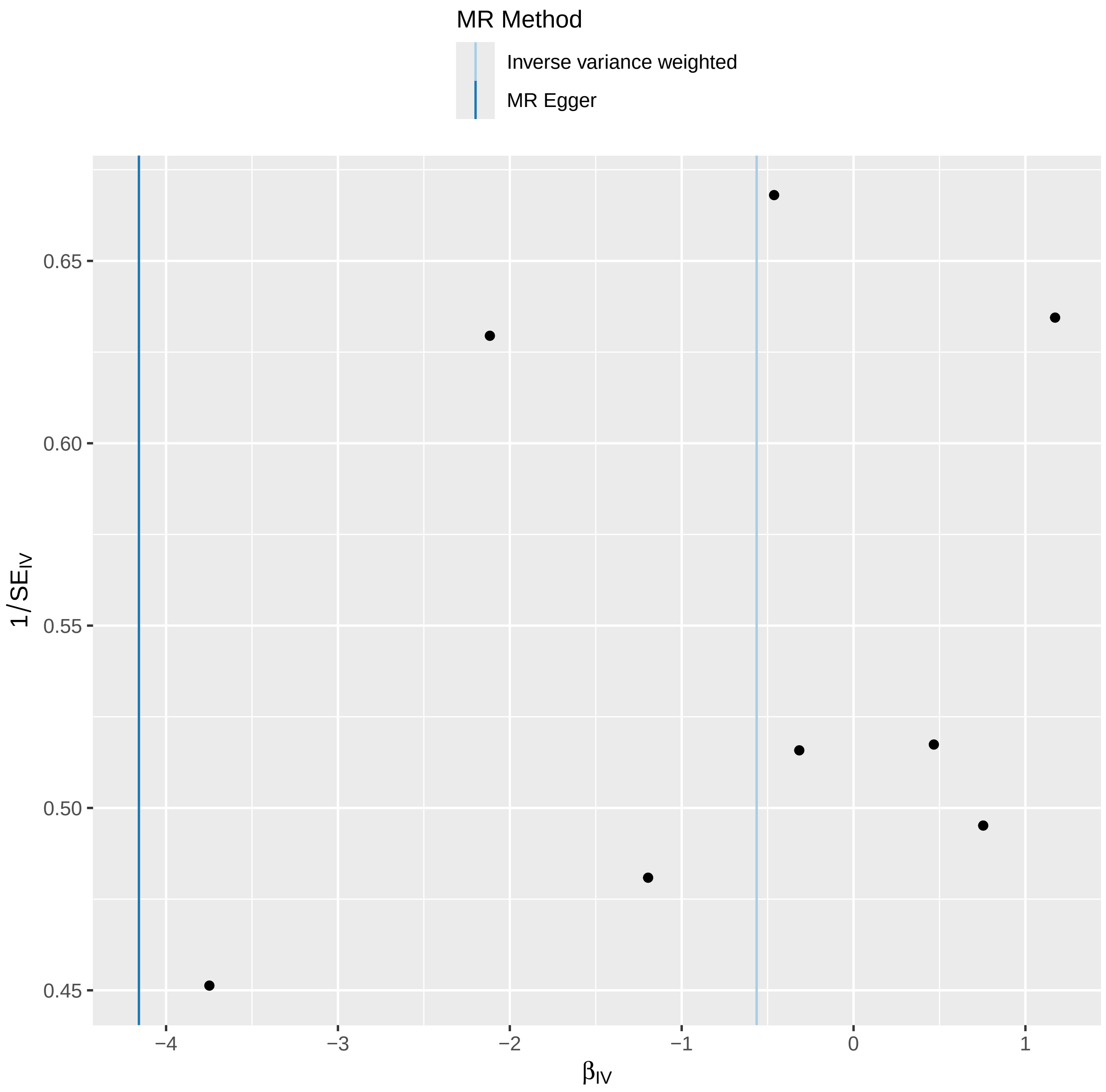
MR Method
Inverse variance weighted
MR Egger
0.65
0.60
0.55
0.50
0.45
$1/SE_{IV}$
−4
−3
−2
−1
0
1
β_{IV}

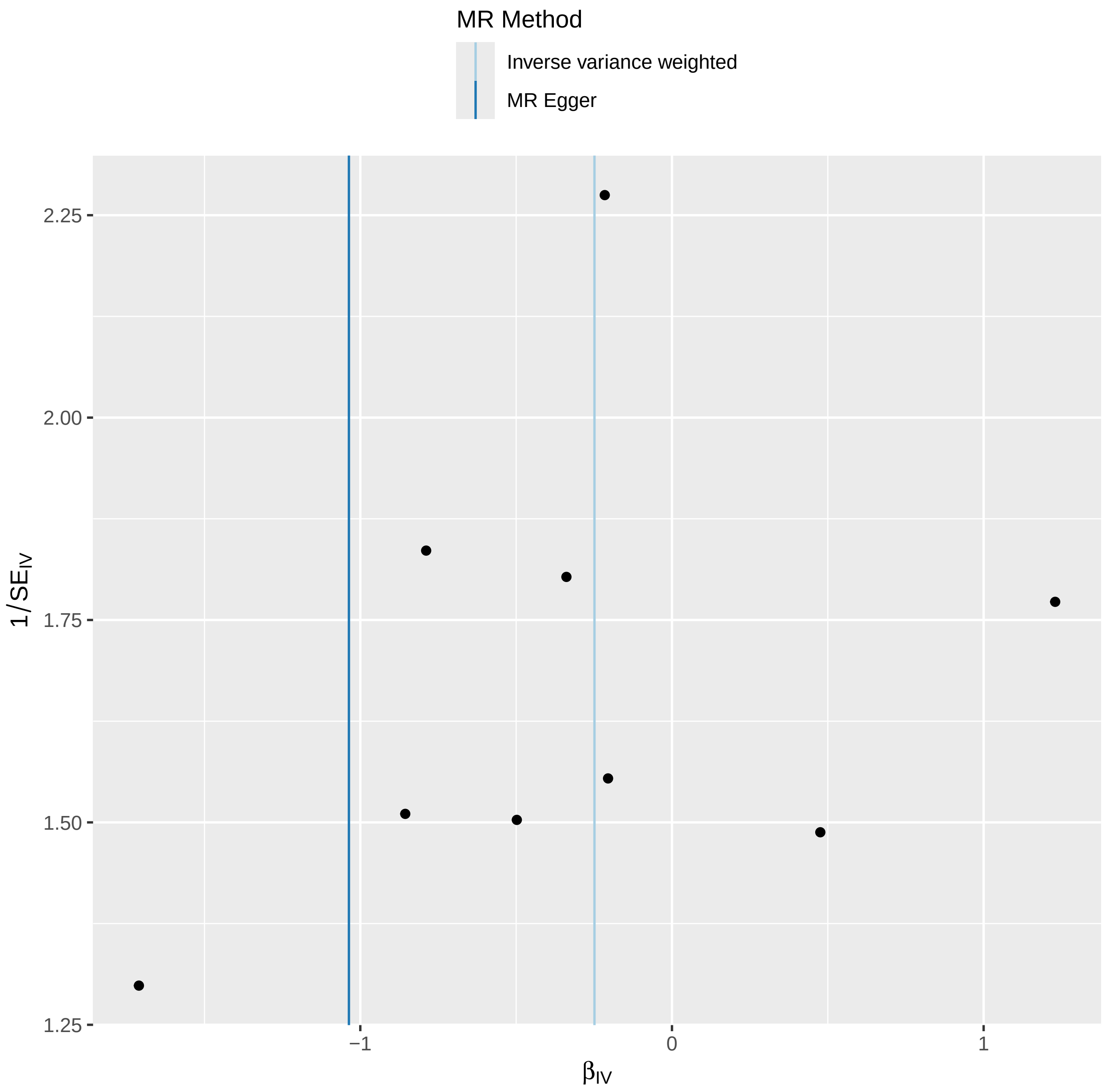

MR Method
Inverse variance weighted
MR Egger
2.25
2.00
1.75
1.50
1.25
1/SE_IV
−1
0
1
β_IV

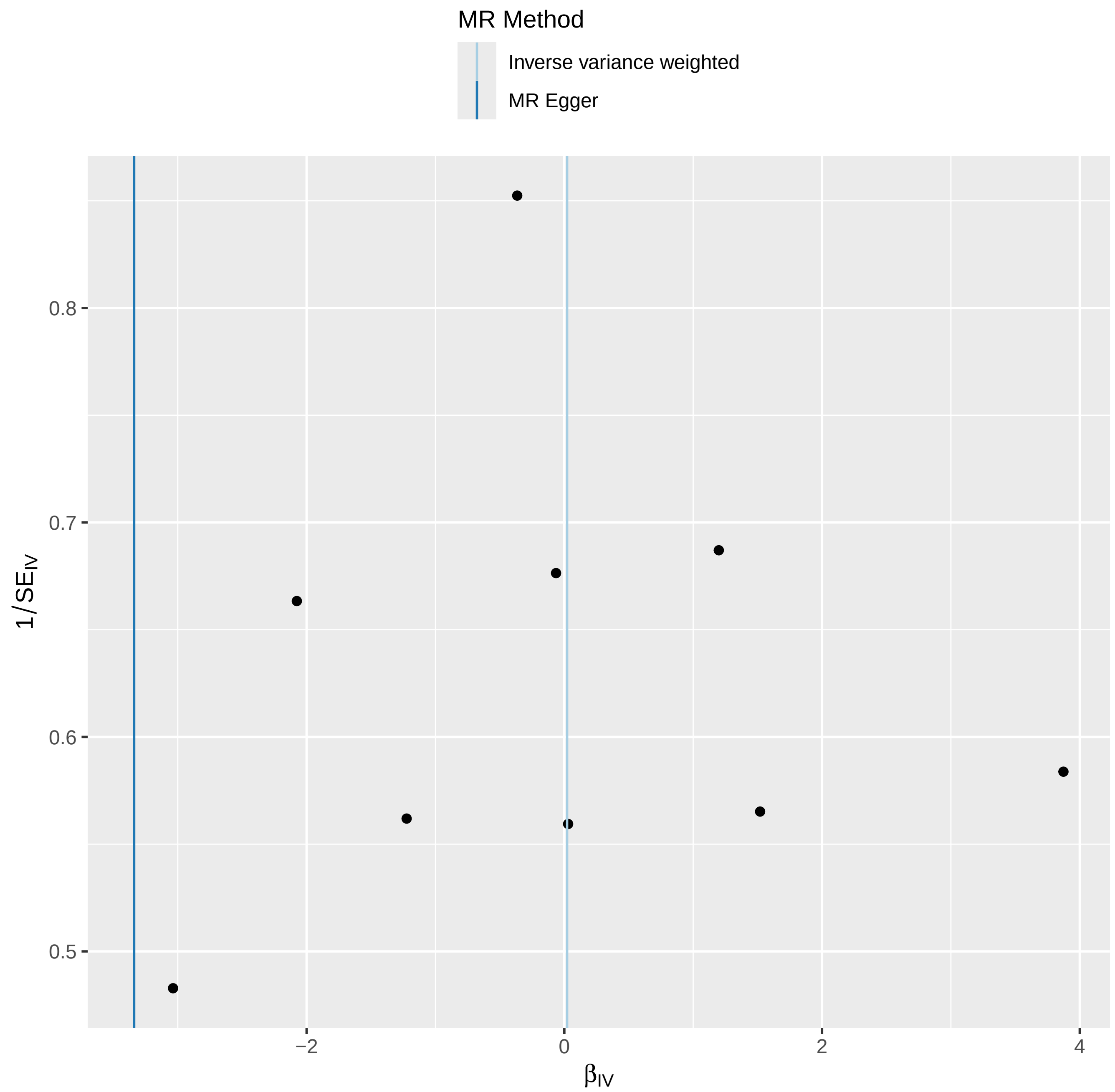
MR Method
Inverse variance weighted
MR Egger
0.8
0.7
0.6
0.5
1/SE_IV
−2
0
2
4
β_IV

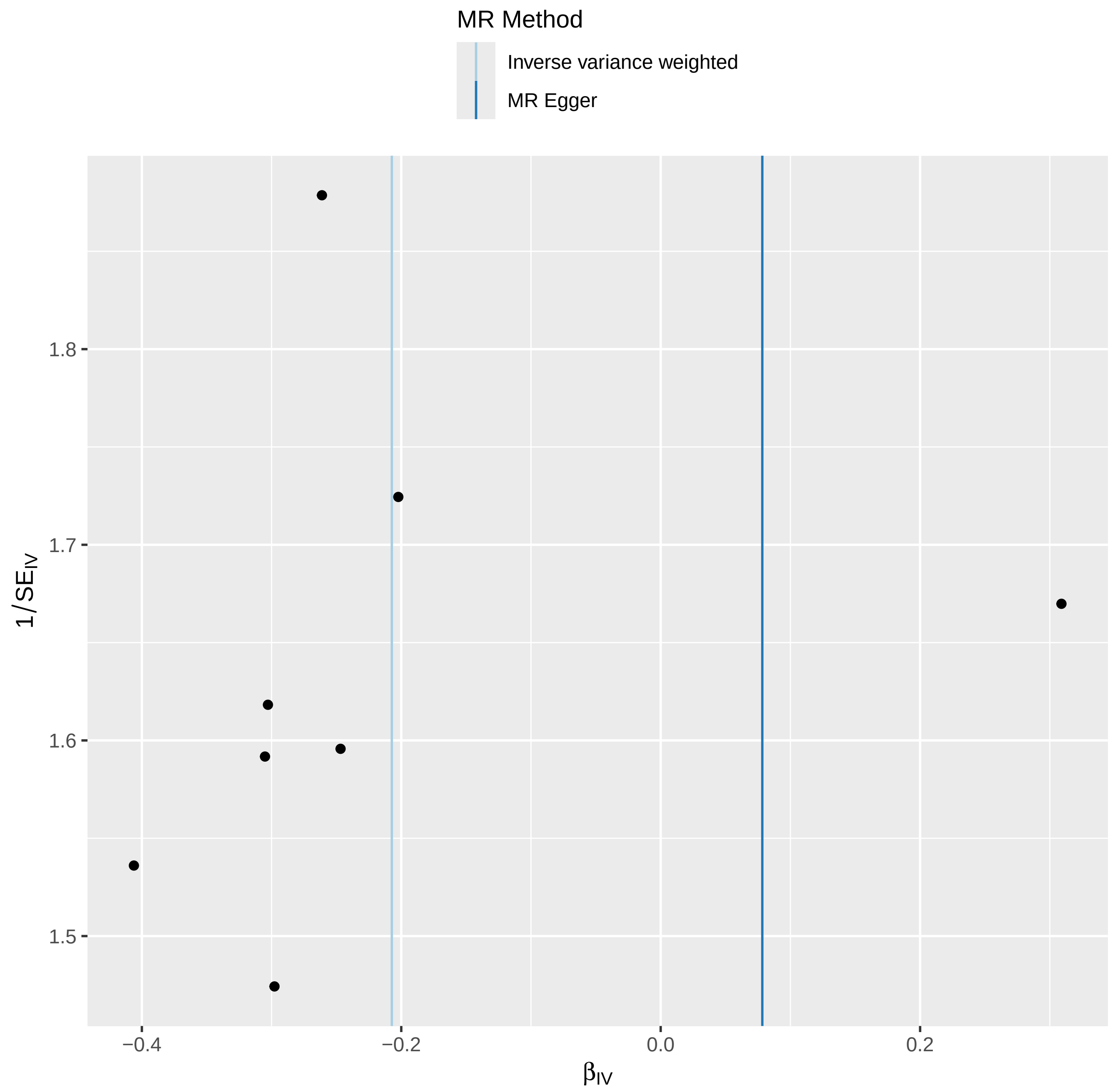
MR Method
Inverse variance weighted
MR Egger
1.8
1.7
1.6
1.5
1/SE_IV
−0.4
−0.2
0.0
0.2
β_IV

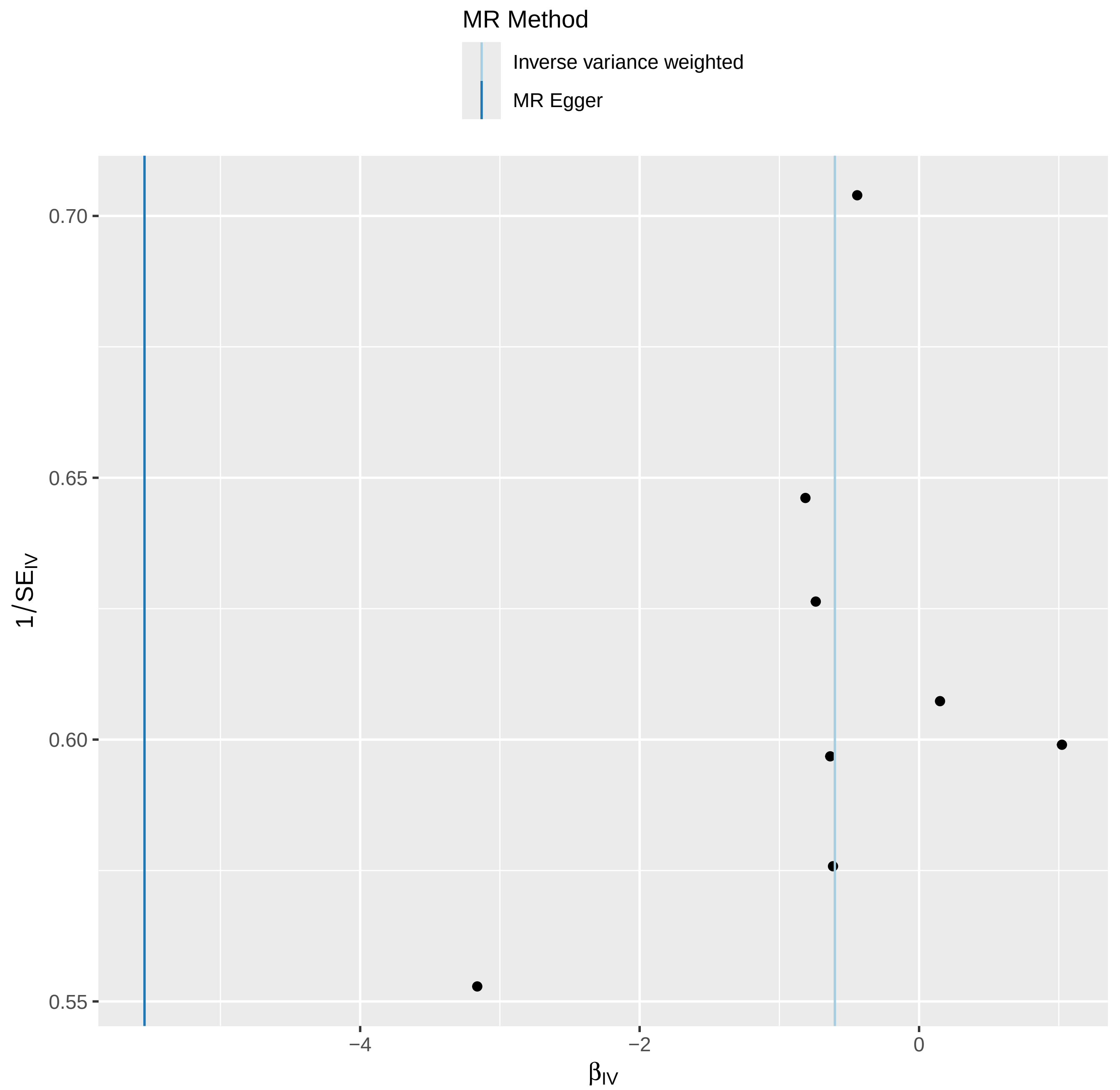
MR Method
Inverse variance weighted
MR Egger
0.70
0.65
0.60
0.55
1/SE_IV
−4
−2
0
β_IV

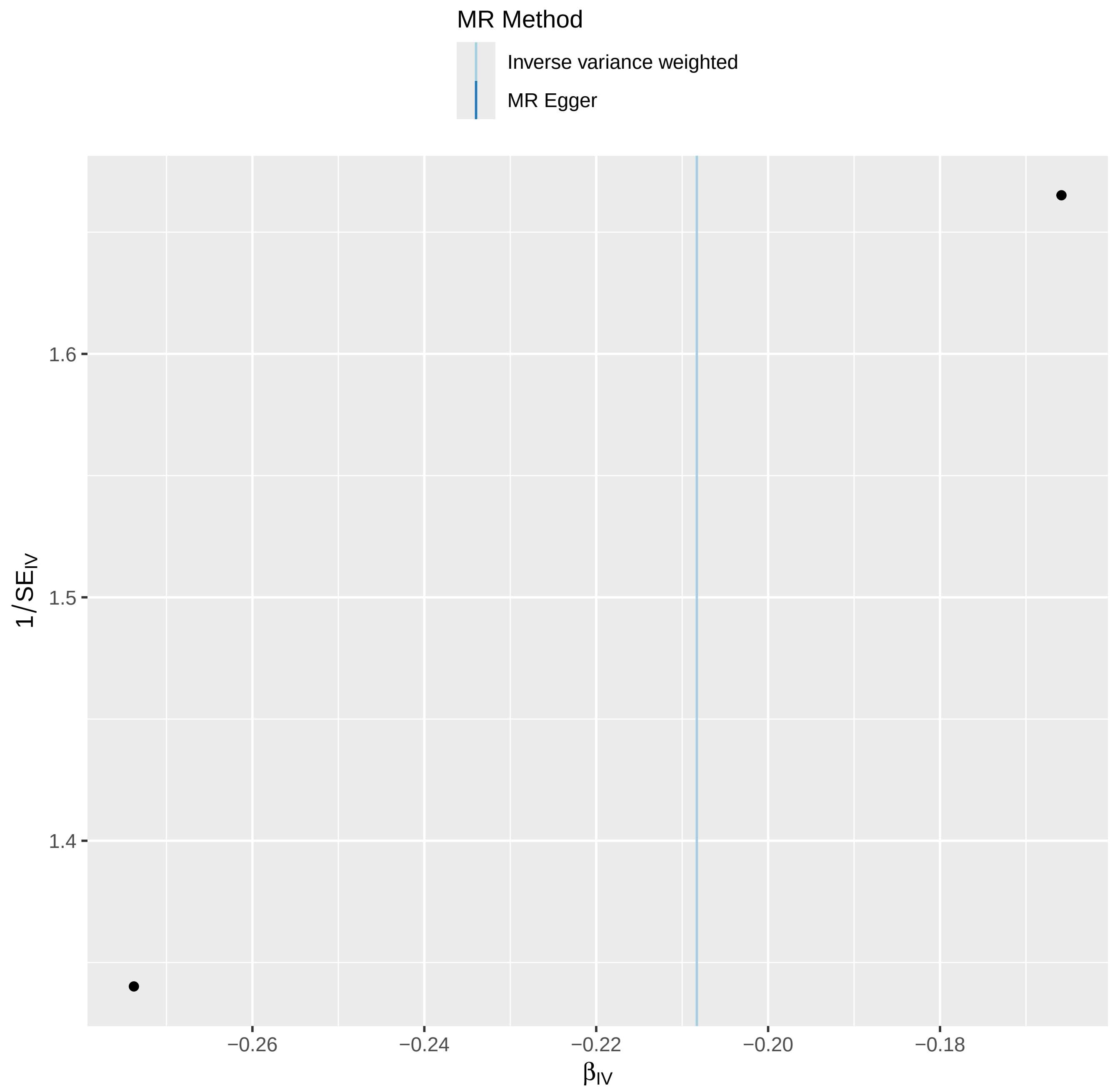
MR Method
Inverse variance weighted
MR Egger
1.6
1.5
1.4
−0.26
−0.24
−0.22
−0.20
−0.18
$1/SE_{IV}$
β_{IV}

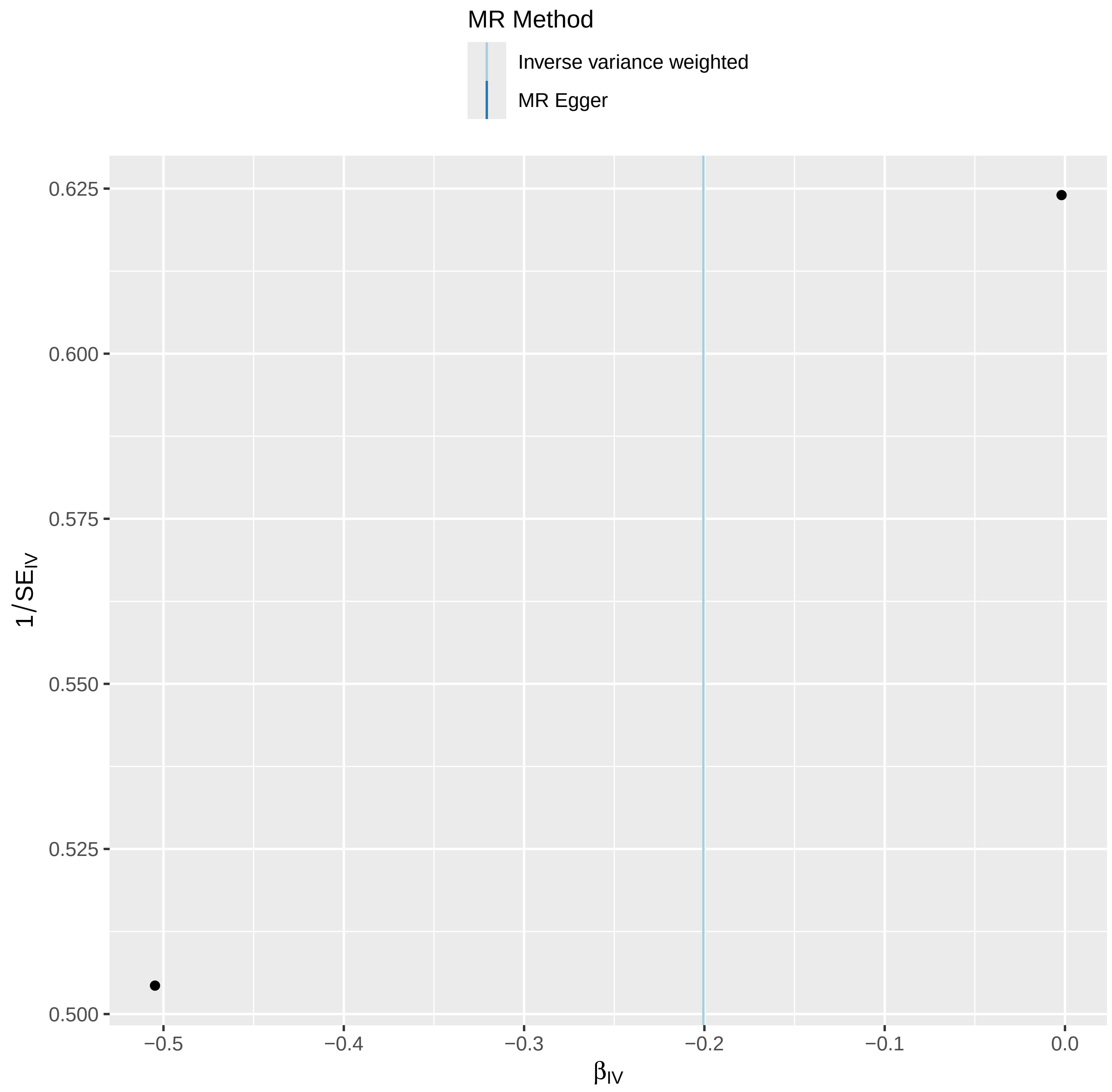
MR Method
Inverse variance weighted
MR Egger
0.625
0.600
0.575
0.550
0.525
0.500
1/SE_IV
−0.5
−0.4
−0.3
−0.2
−0.1
0.0
β_IV

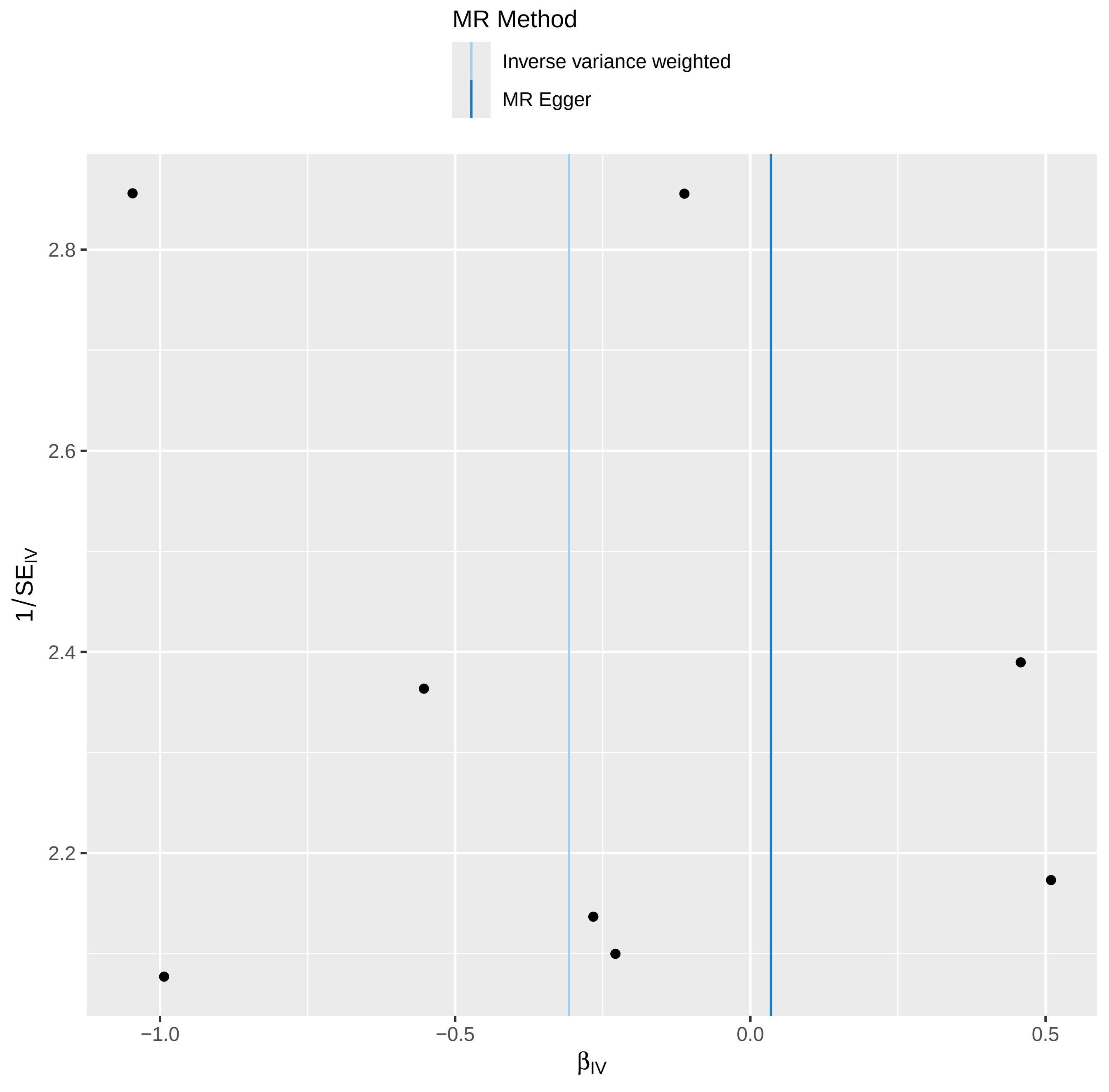

MR Method
Inverse variance weighted
MR Egger
2.8
2.6
2.4
2.2
1/SE_IV
−1.0
−0.5
0.0
0.5
β_IV

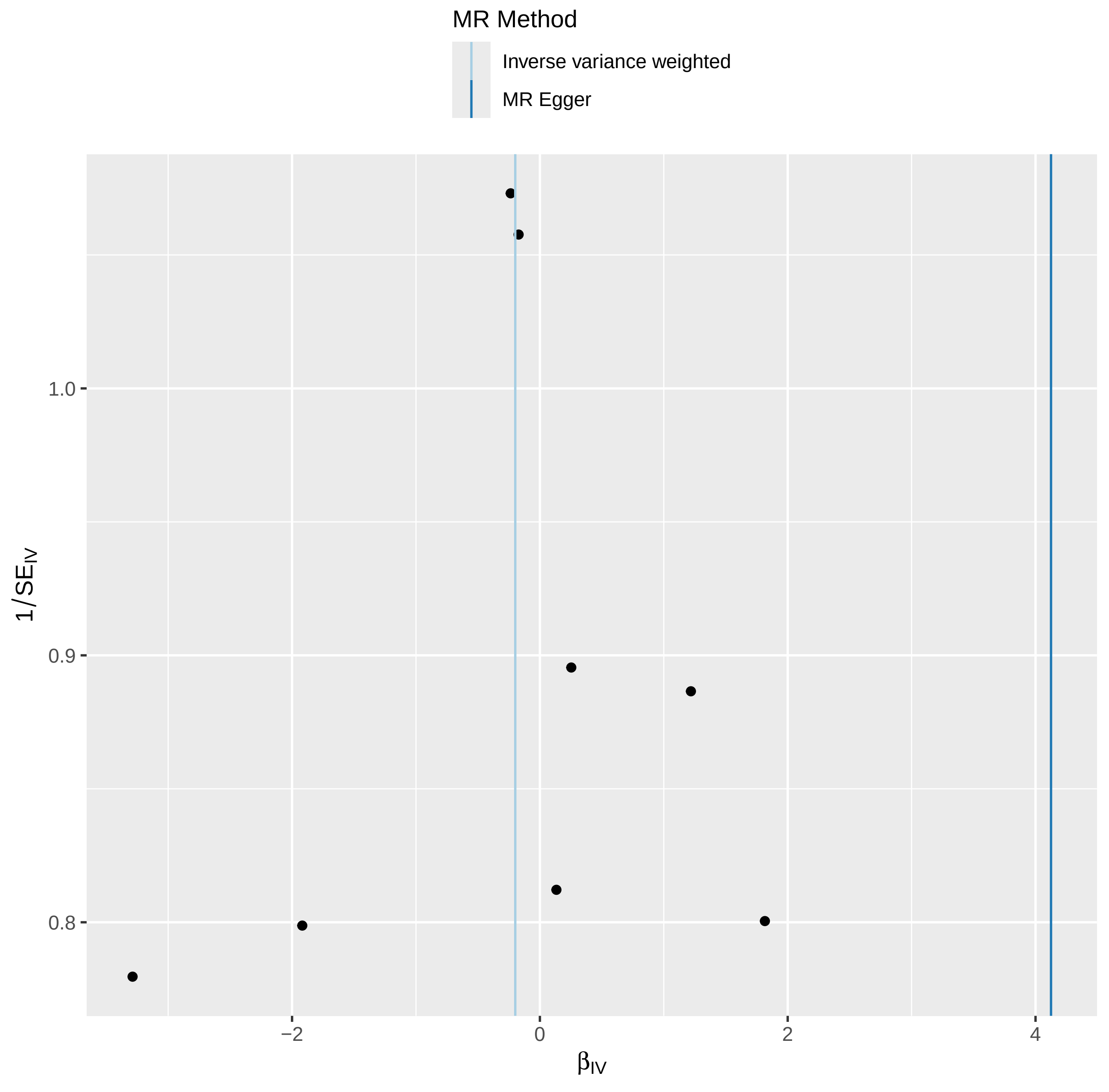

MR Method
Inverse variance weighted
MR Egger
1.0
0.9
0.8
1/SE_IV
−2
0
2
4
β_IV

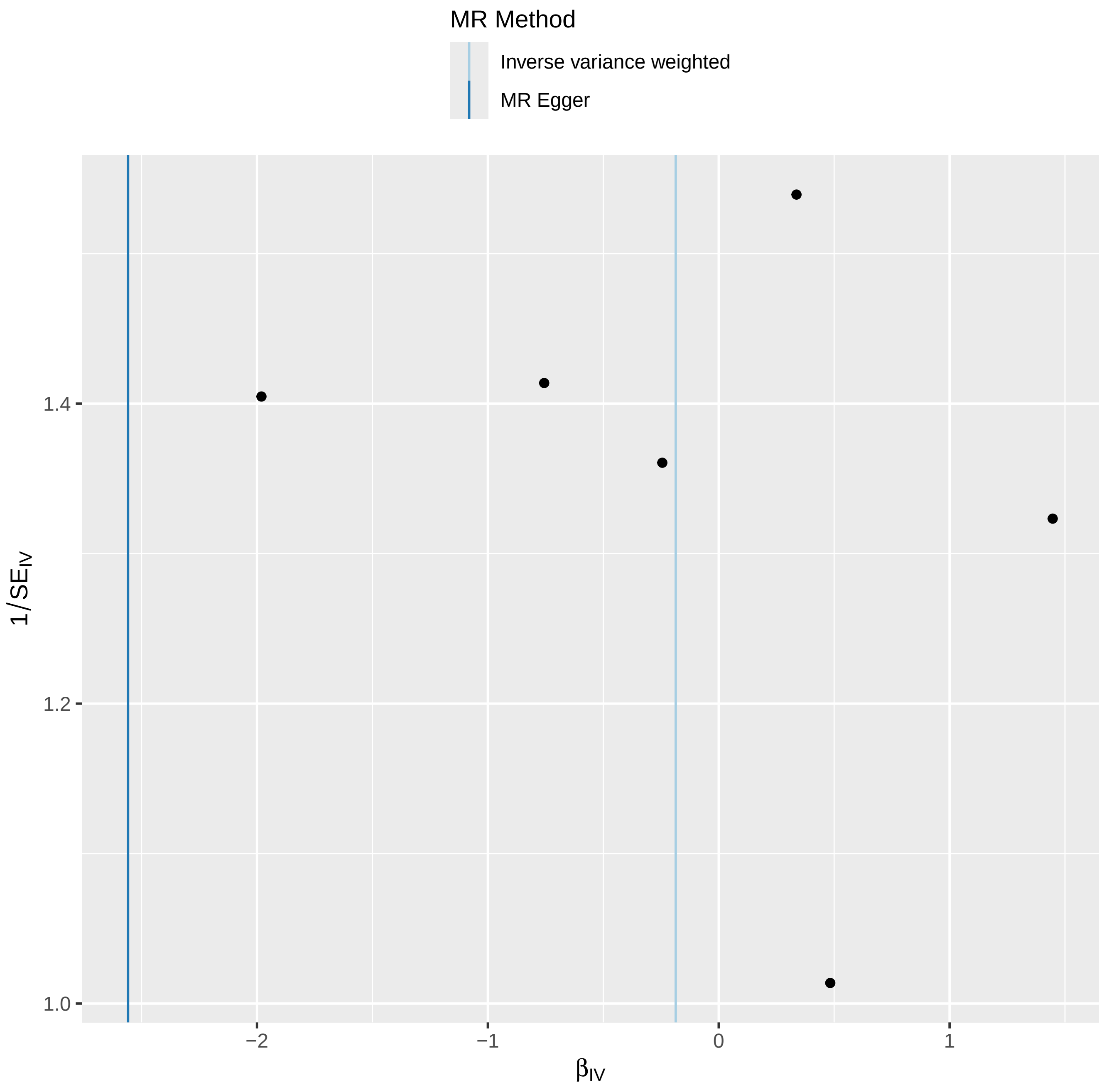

MR Method
Inverse variance weighted
MR Egger
1.4
1.2
1.0
1/SE$_{IV}$
−2
−1
0
1
β$_{IV}$

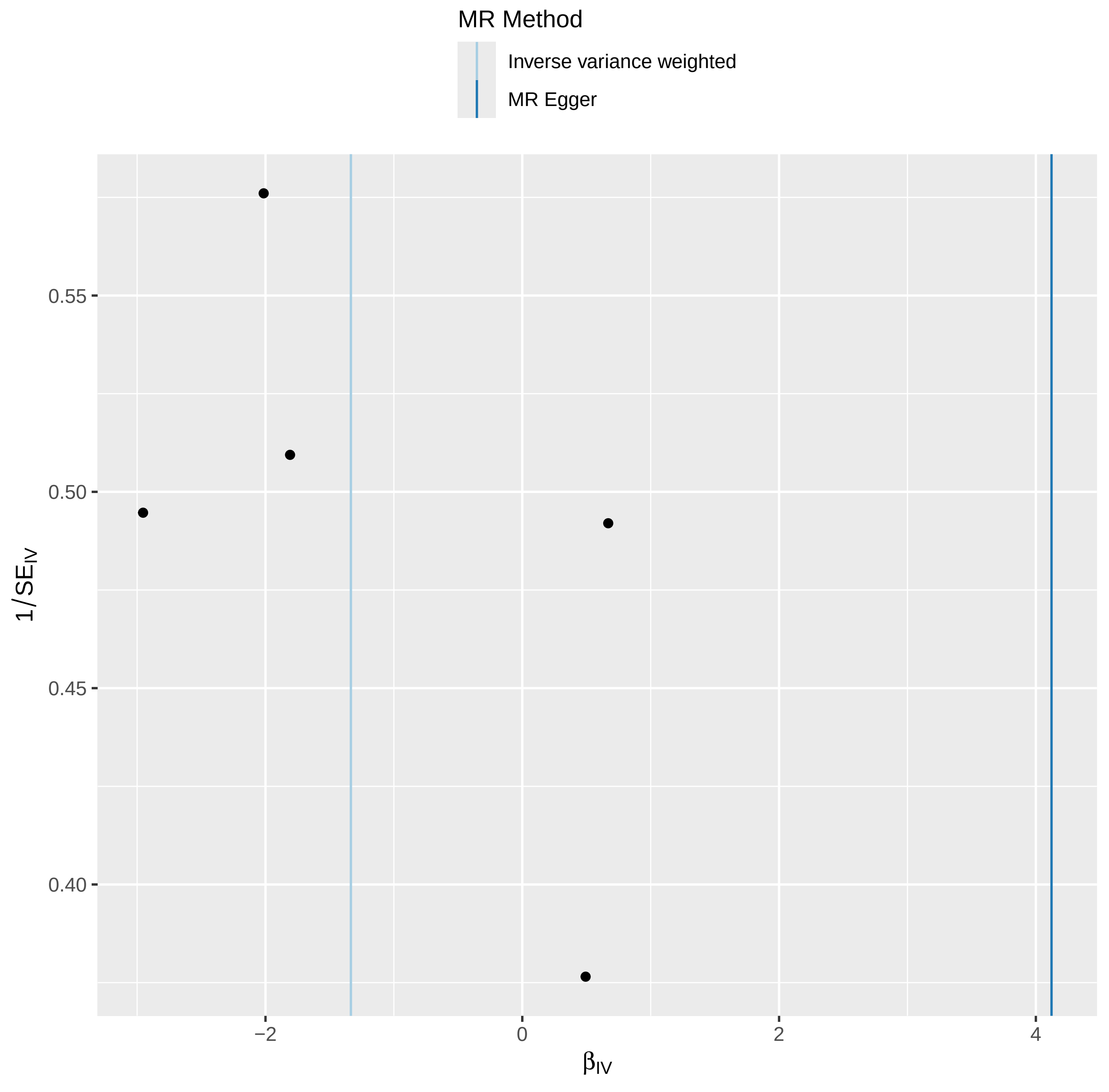
MR Method
Inverse variance weighted
MR Egger
0.55
0.50
0.45
0.40
1/SE_IV
−2
0
2
4
β_IV

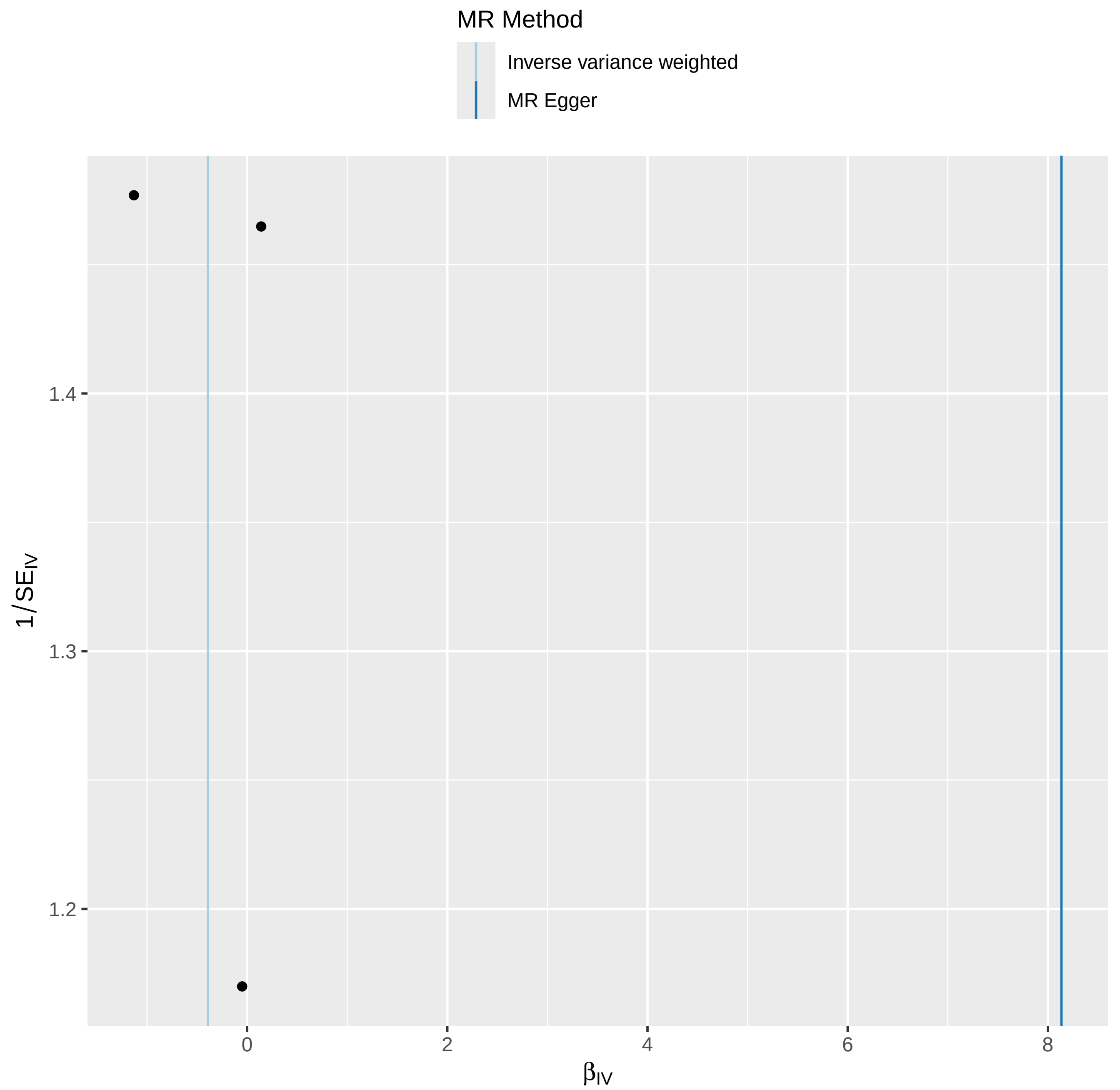
MR Method
Inverse variance weighted
MR Egger
1.4
1.3
1.2
1/SE_IV
0
2
4
6
8
β_IV

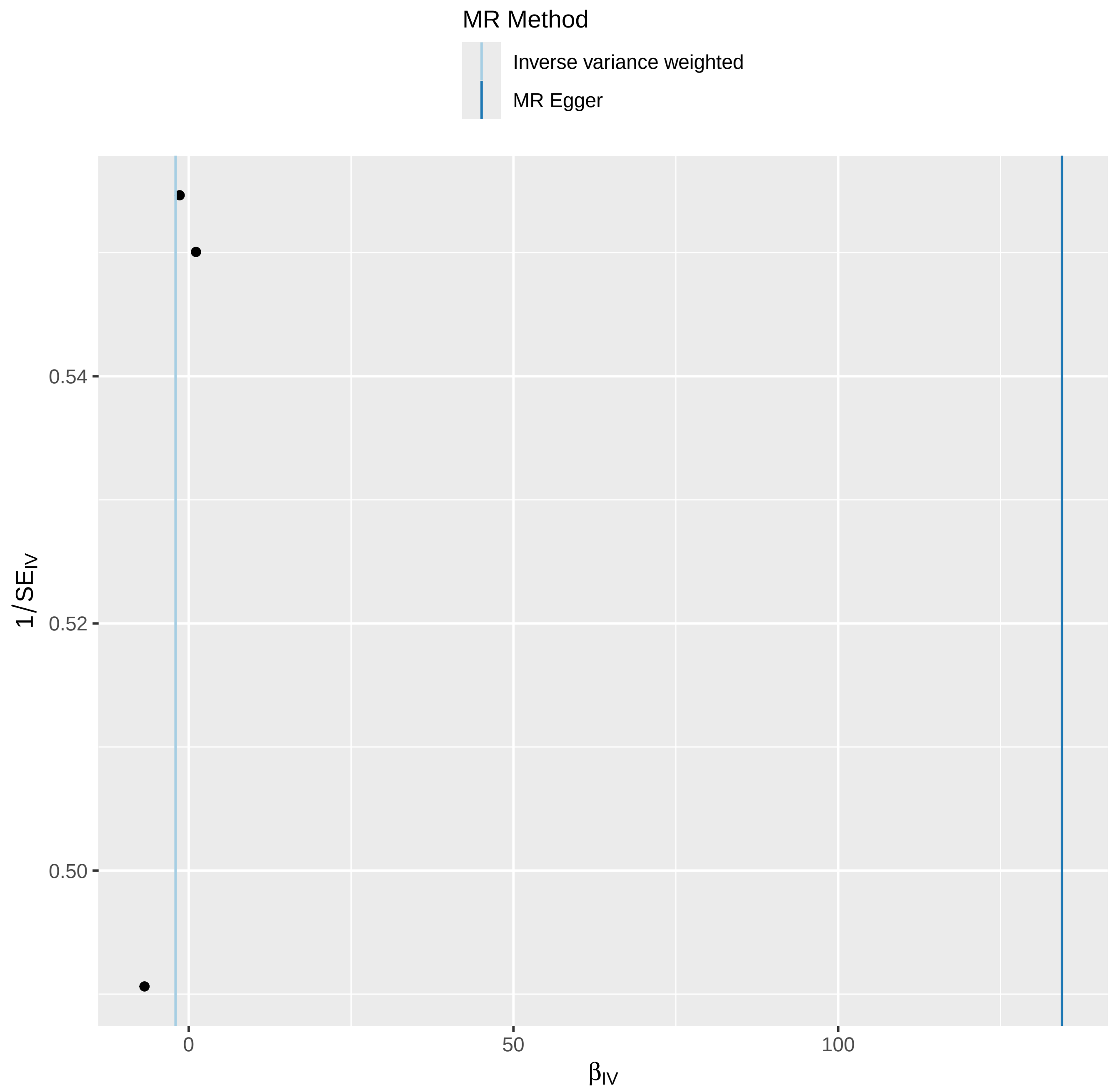

MR Method
Inverse variance weighted
MR Egger
0.54
0.52
0.50
$1/SE_{IV}$
0
50
100
β_{IV}

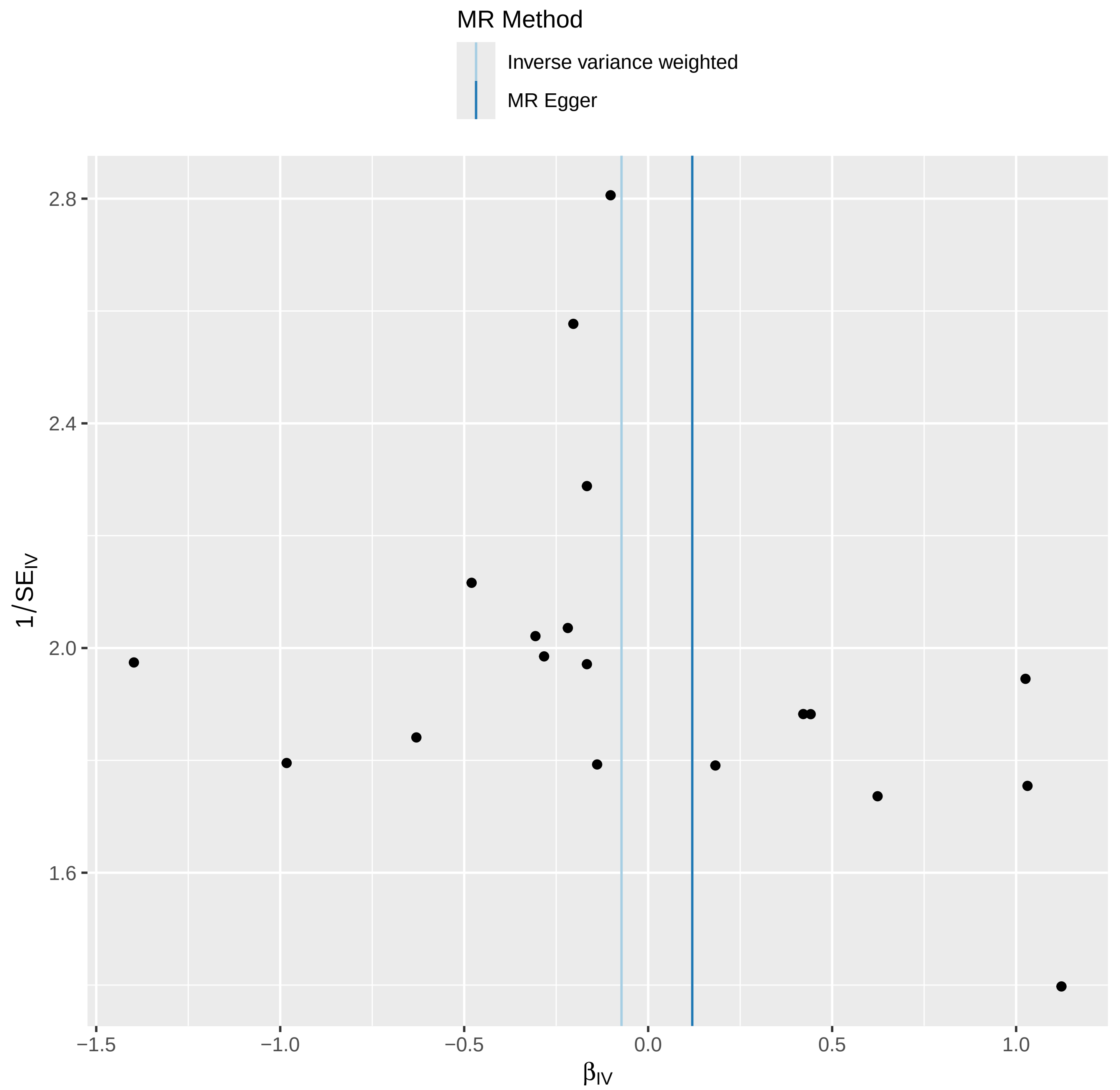

MR Method
Inverse variance weighted
MR Egger
2.8
2.4
2.0
1.6
1/SE_IV
−1.5
−1.0
−0.5
0.0
0.5
1.0
β_IV

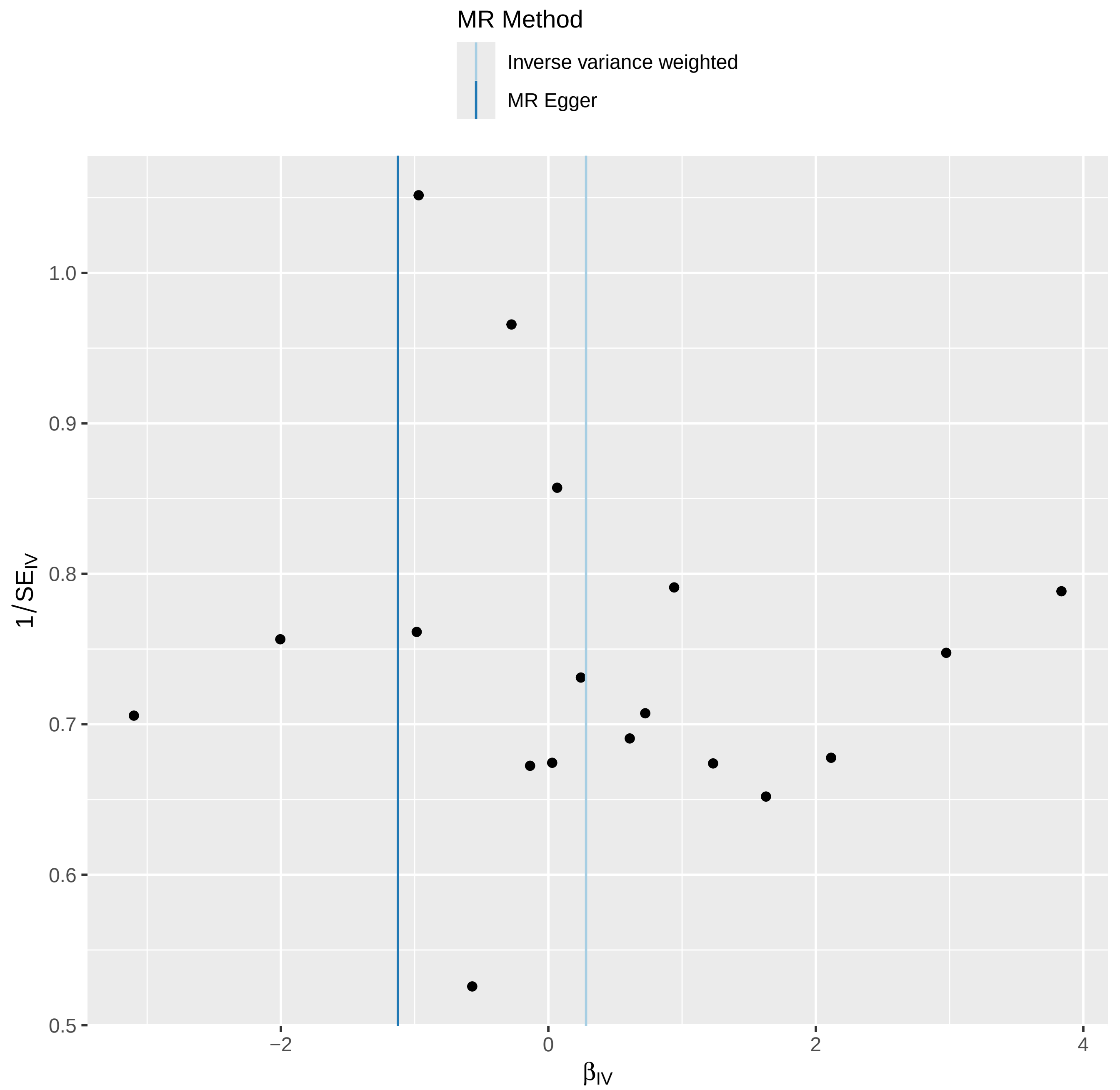
MR Method
Inverse variance weighted
MR Egger
1.0
0.9
0.8
0.7
0.6
0.5
1/SE_IV
−2
0
2
4
β_IV

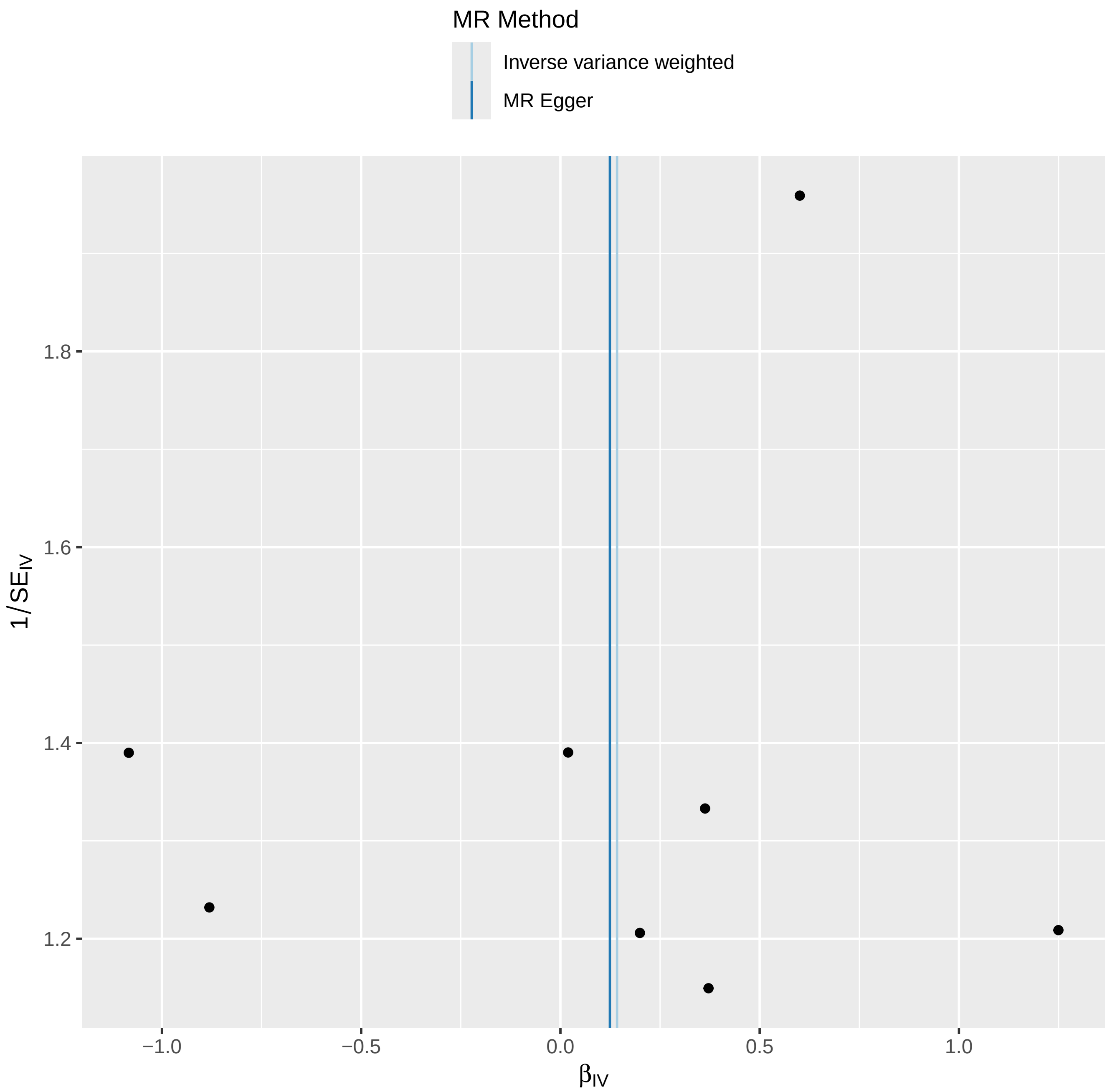
MR Method
Inverse variance weighted
MR Egger
1.8
1.6
1.4
1.2
$1/SE_{IV}$
−1.0
−0.5
0.0
0.5
1.0
β_{IV}

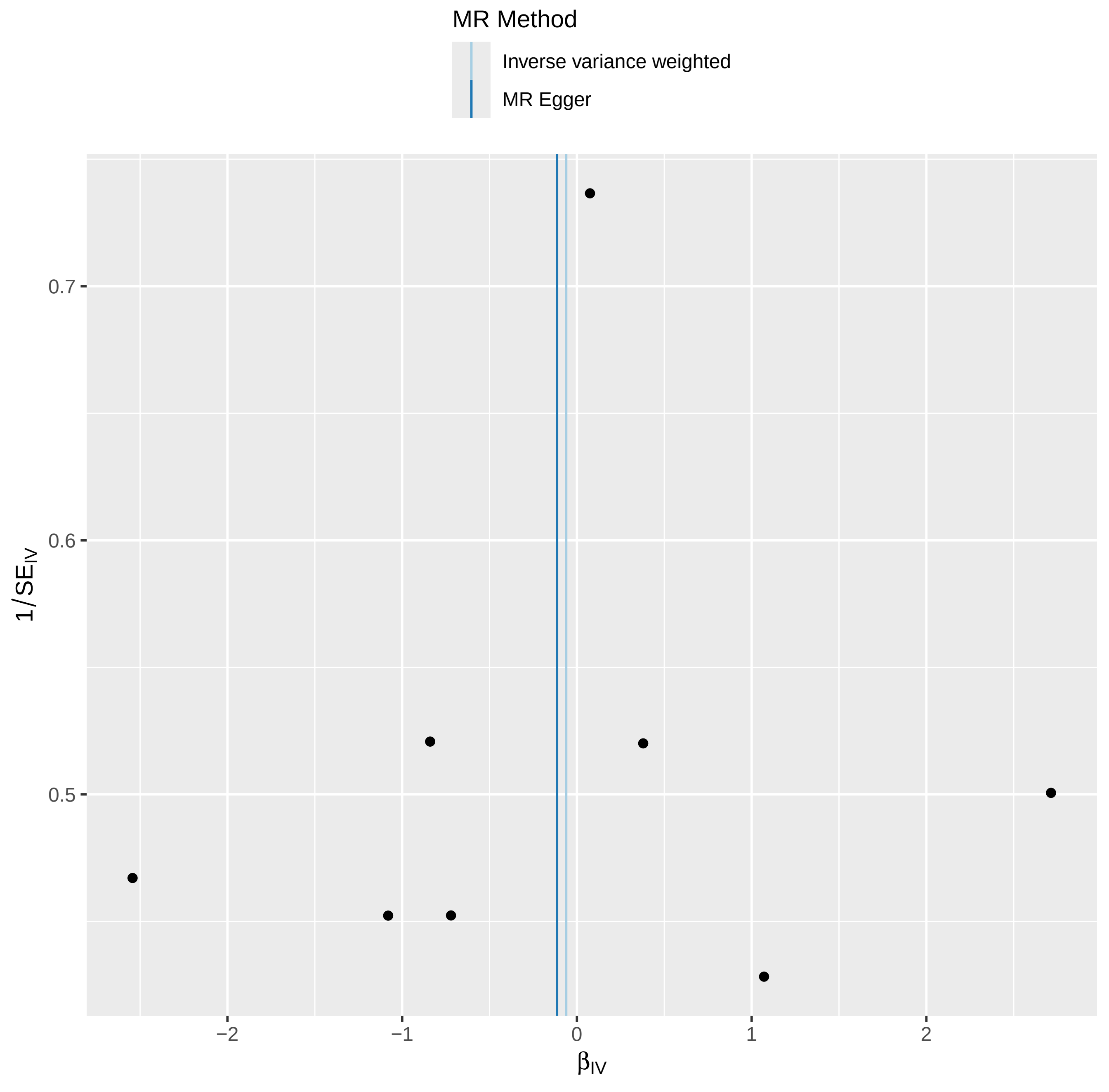

MR Method
Inverse variance weighted
MR Egger
0.7
0.6
0.5
$1/SE_{IV}$
−2
−1
0
1
2
β_{IV}

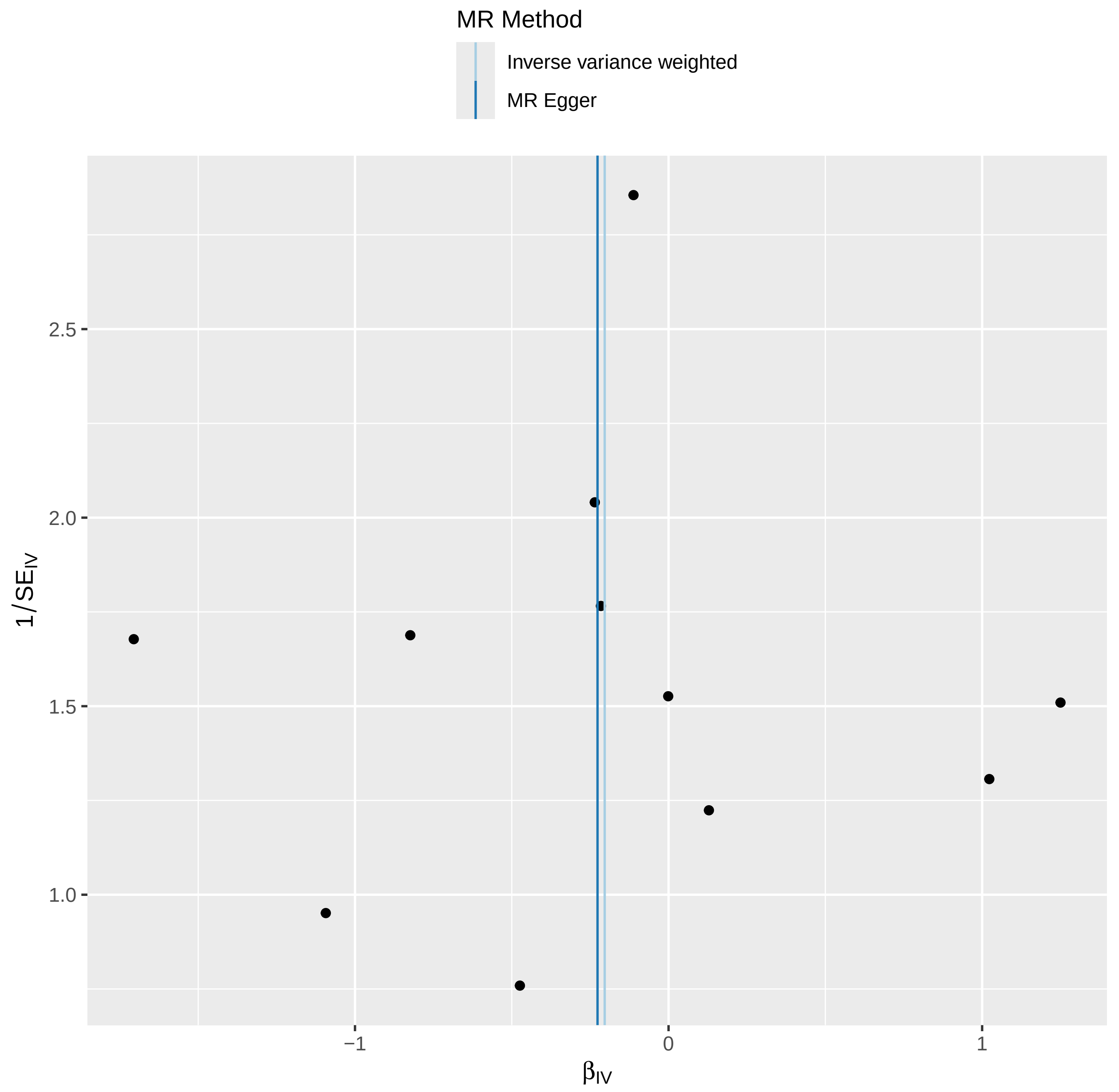
MR Method
Inverse variance weighted
MR Egger
2.5
2.0
1.5
1.0
−1
0
1
$1/SE_{IV}$
β_{IV}

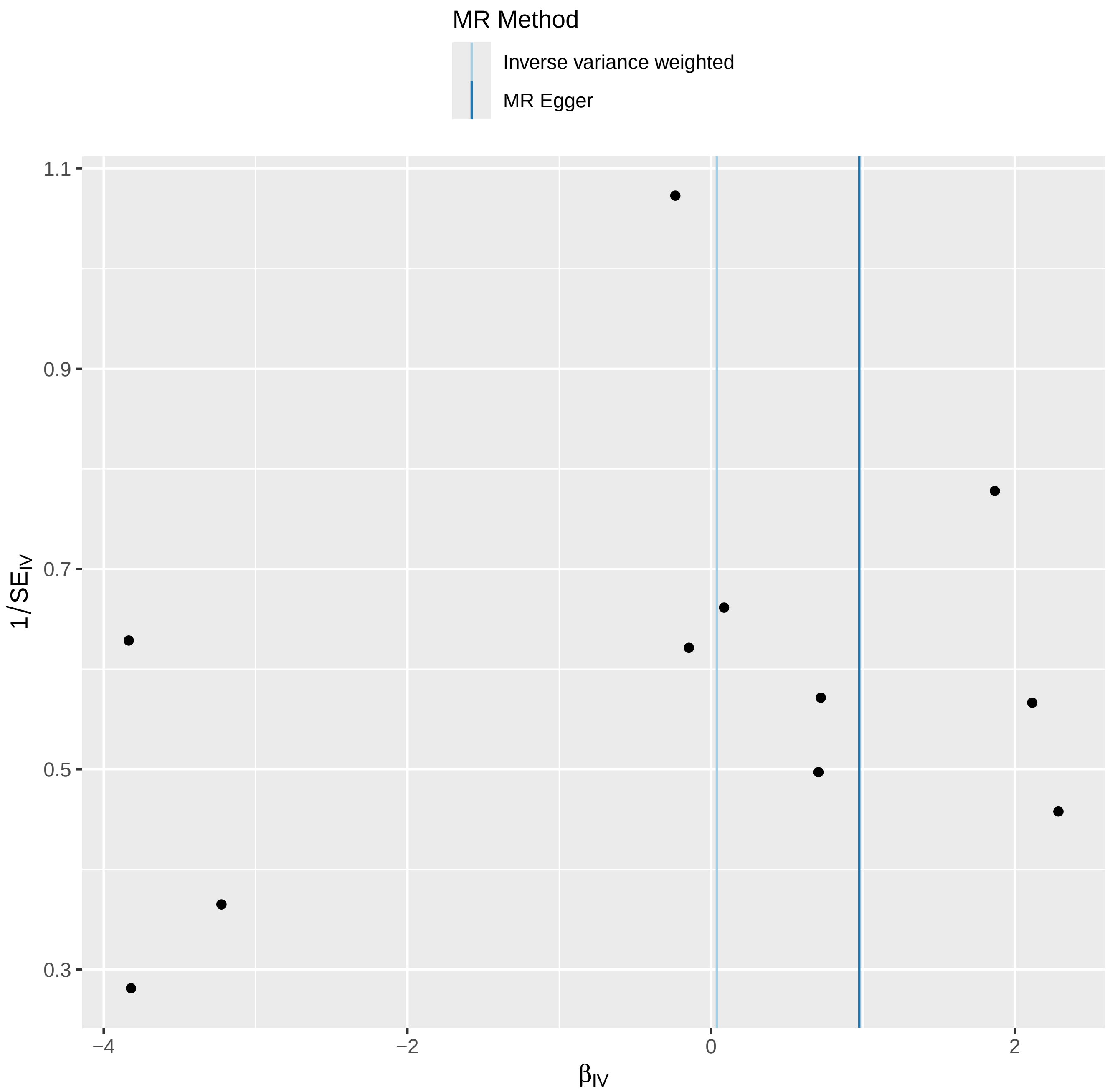
MR Method
Inverse variance weighted
MR Egger
1.1
0.9
0.7
0.5
0.3
$1/SE_{IV}$
−4
−2
0
2
β_{IV}

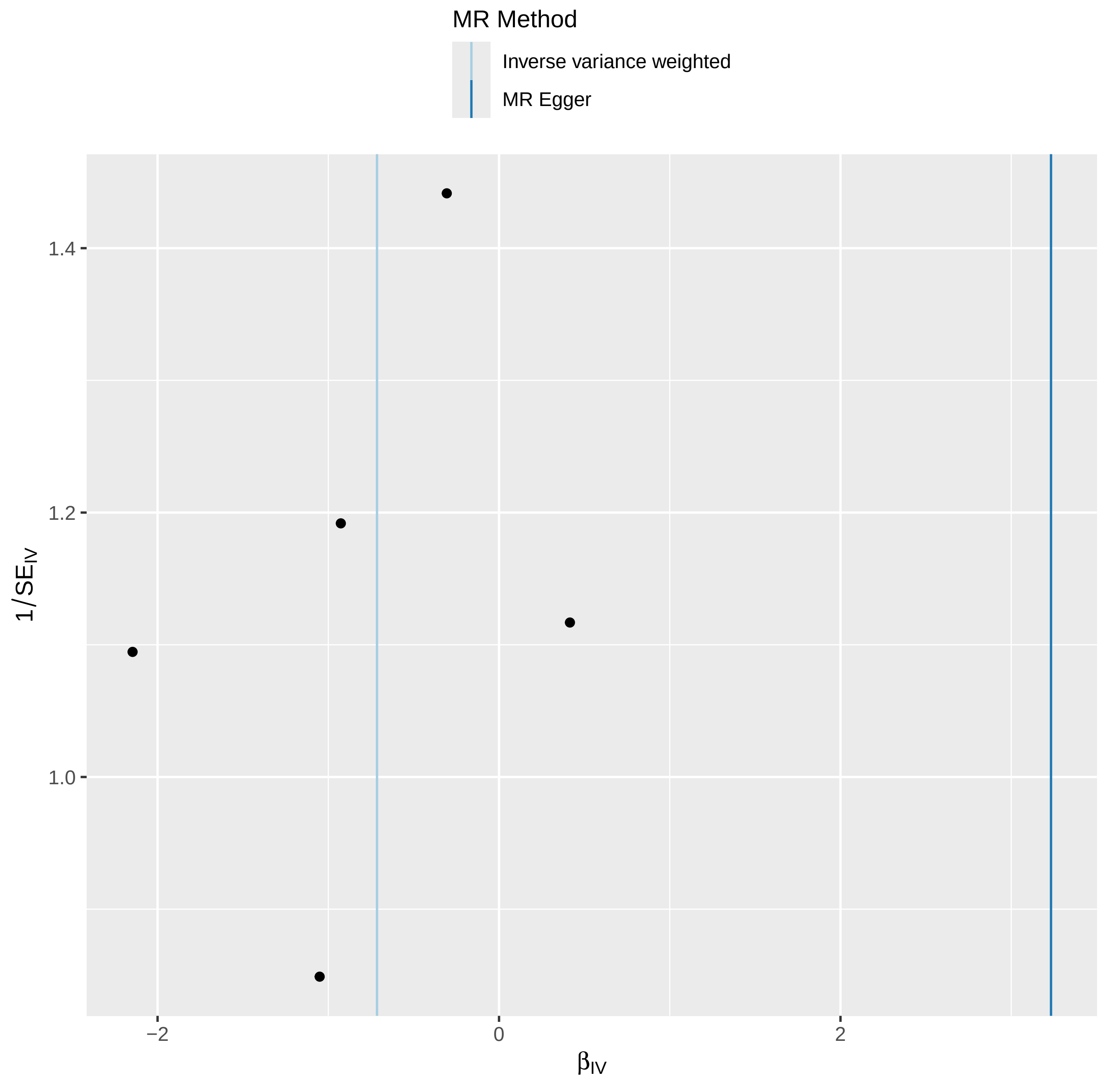
MR Method
Inverse variance weighted
MR Egger
1.4
1.2
1.0
1/SE_IV
−2
0
2
β_IV

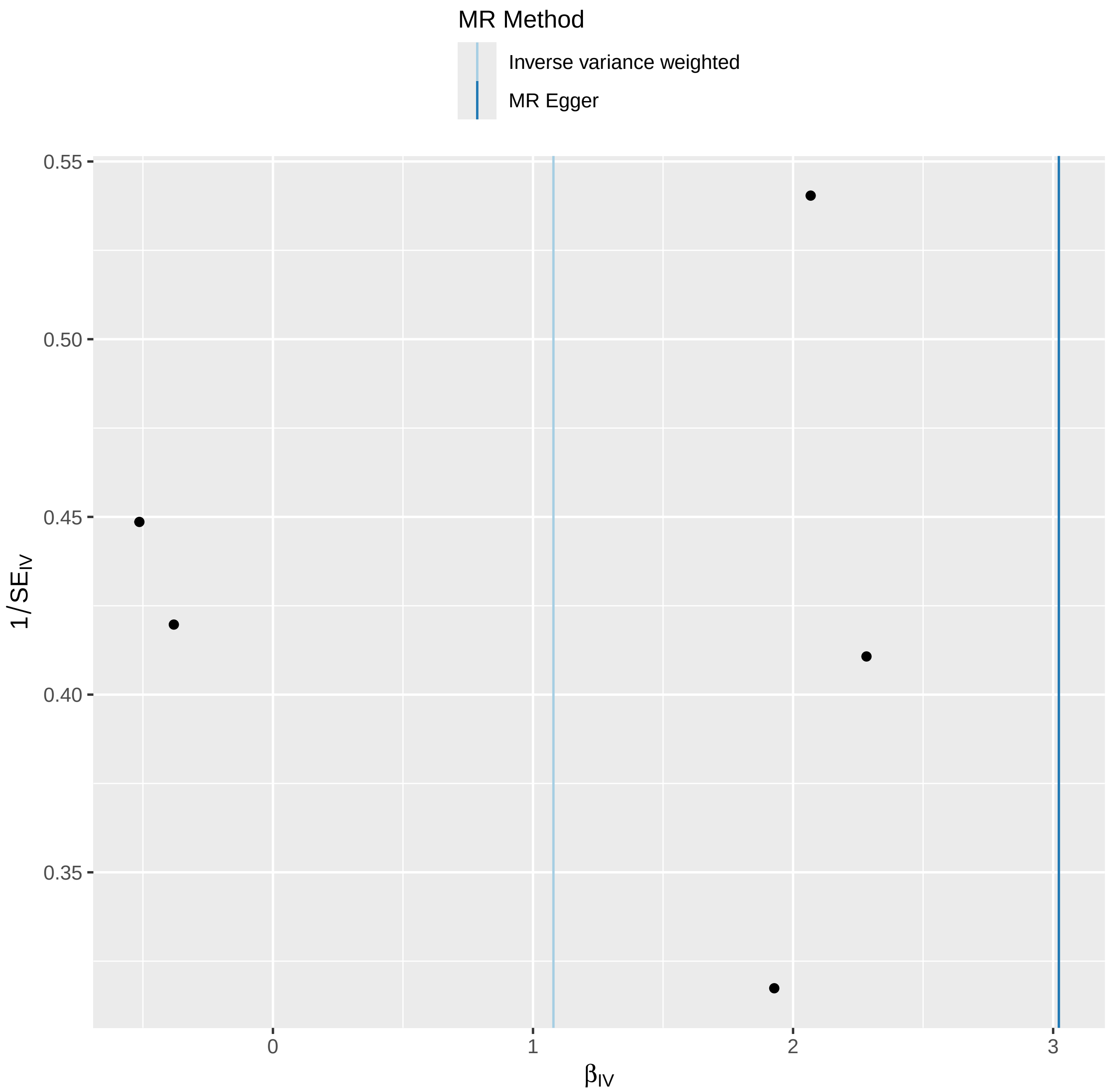
MR Method
Inverse variance weighted
MR Egger
0.55
0.50
0.45
0.40
0.35
$1/SE_{IV}$
0
1
2
3
β_{IV}

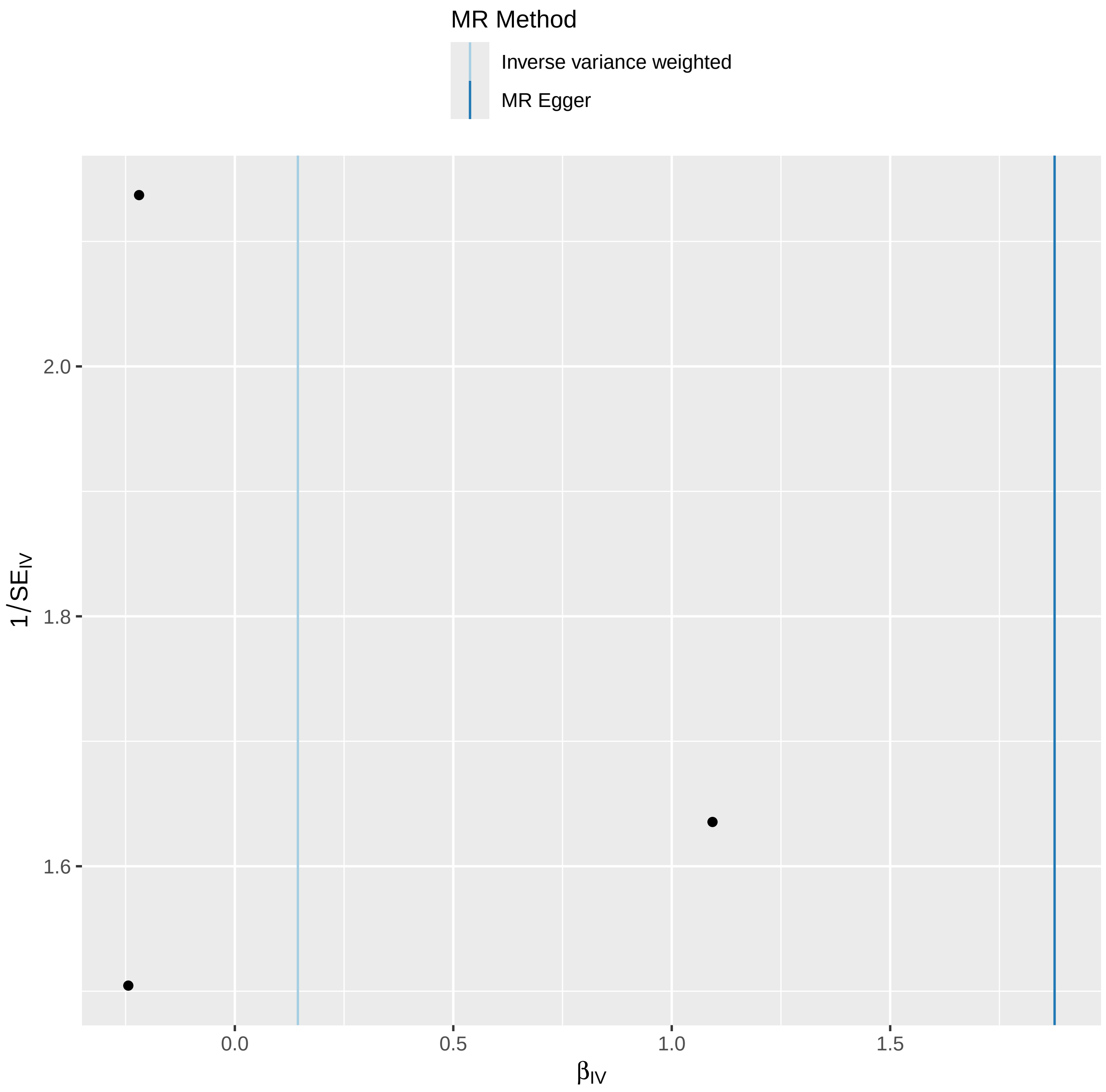

MR Method
Inverse variance weighted
MR Egger
2.0
1.8
1.6
1/SE_IV
0.0
0.5
1.0
1.5
β_IV

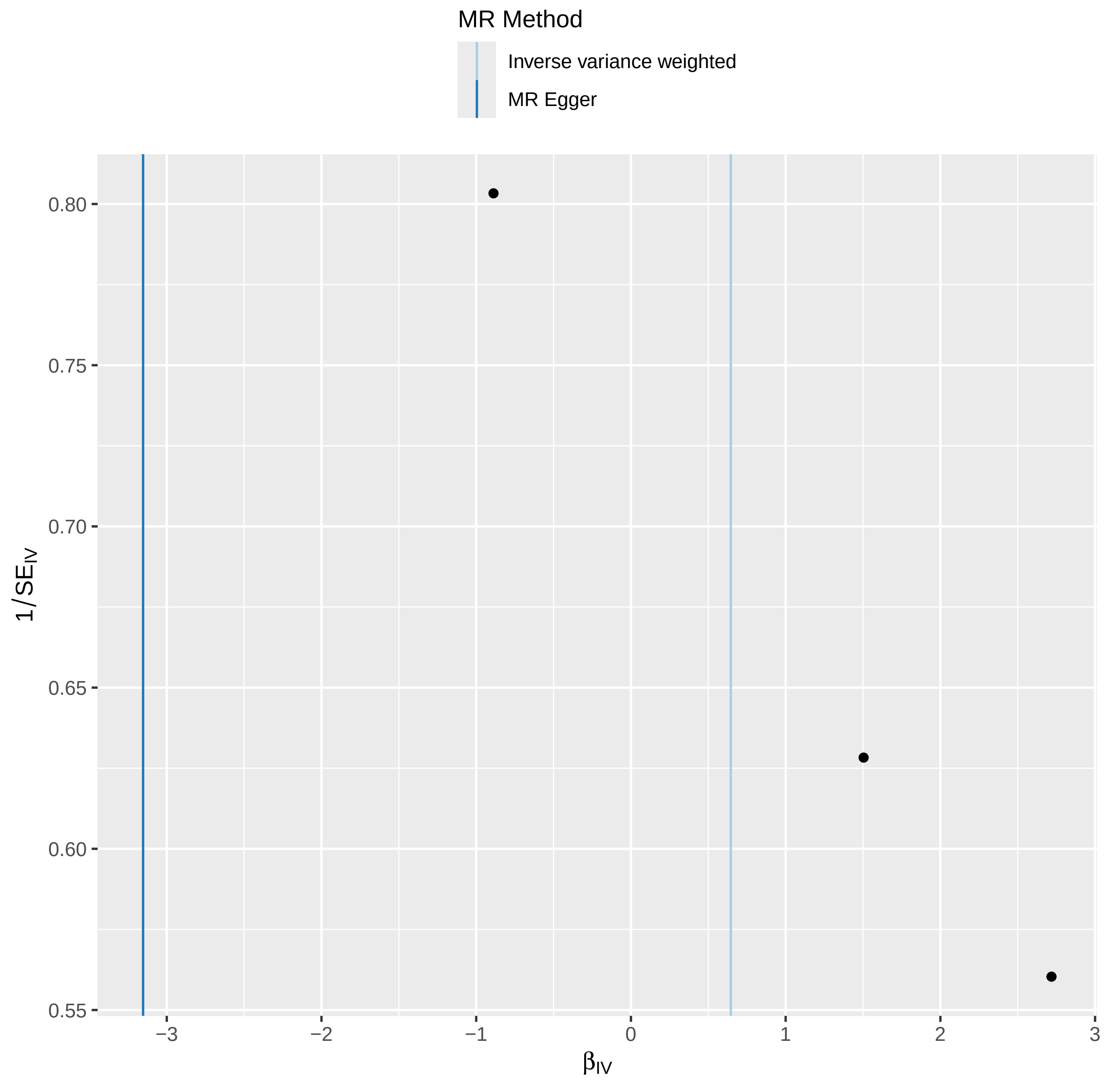
MR Method
Inverse variance weighted
MR Egger
0.80
0.75
0.70
0.65
0.60
0.55
1/SE_IV
−3
−2
−1
0
1
2
3
β_IV

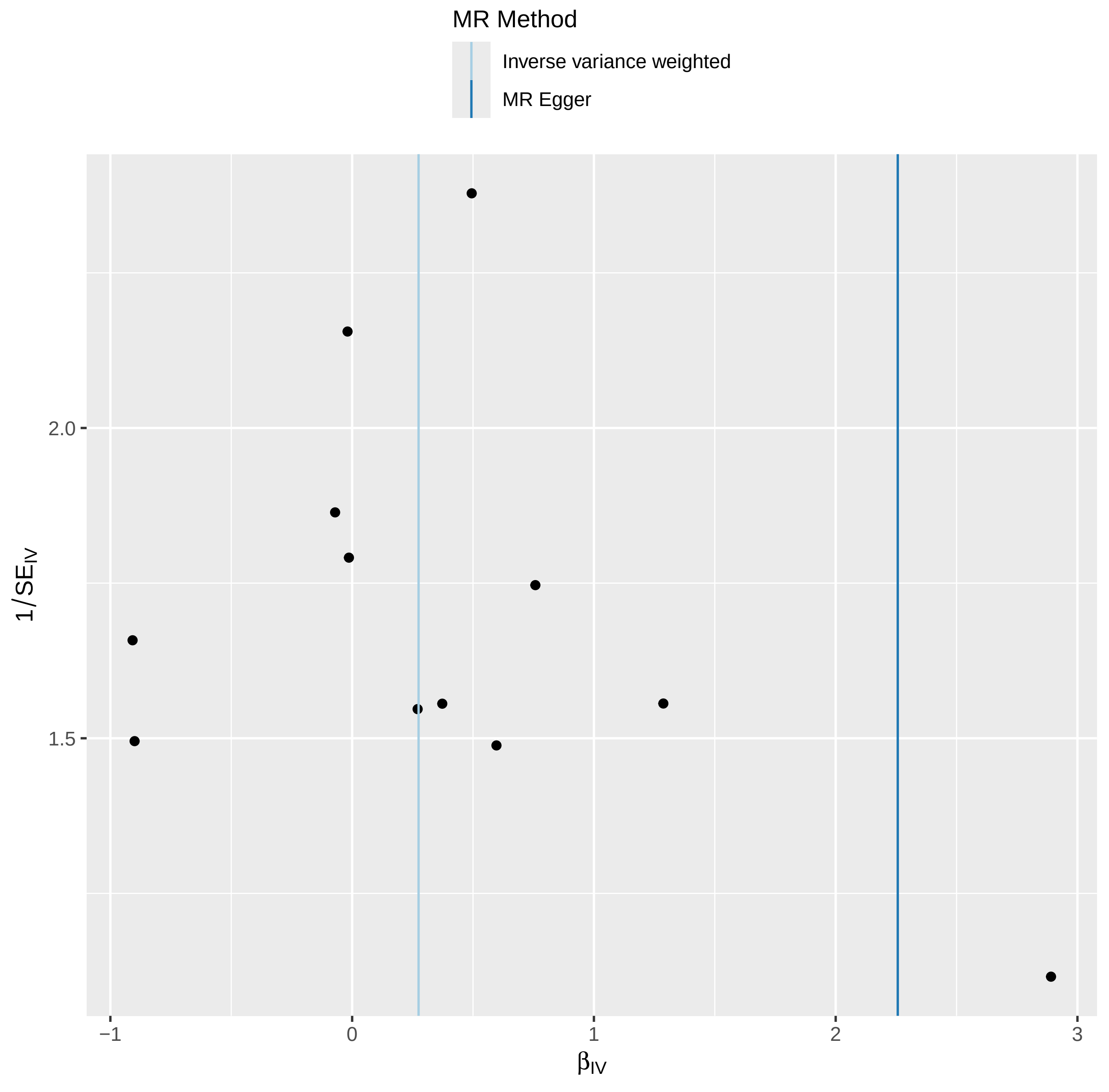

MR Method
Inverse variance weighted
MR Egger
2.0
1.5
1/SE_IV
−1
0
1
2
3
β_IV

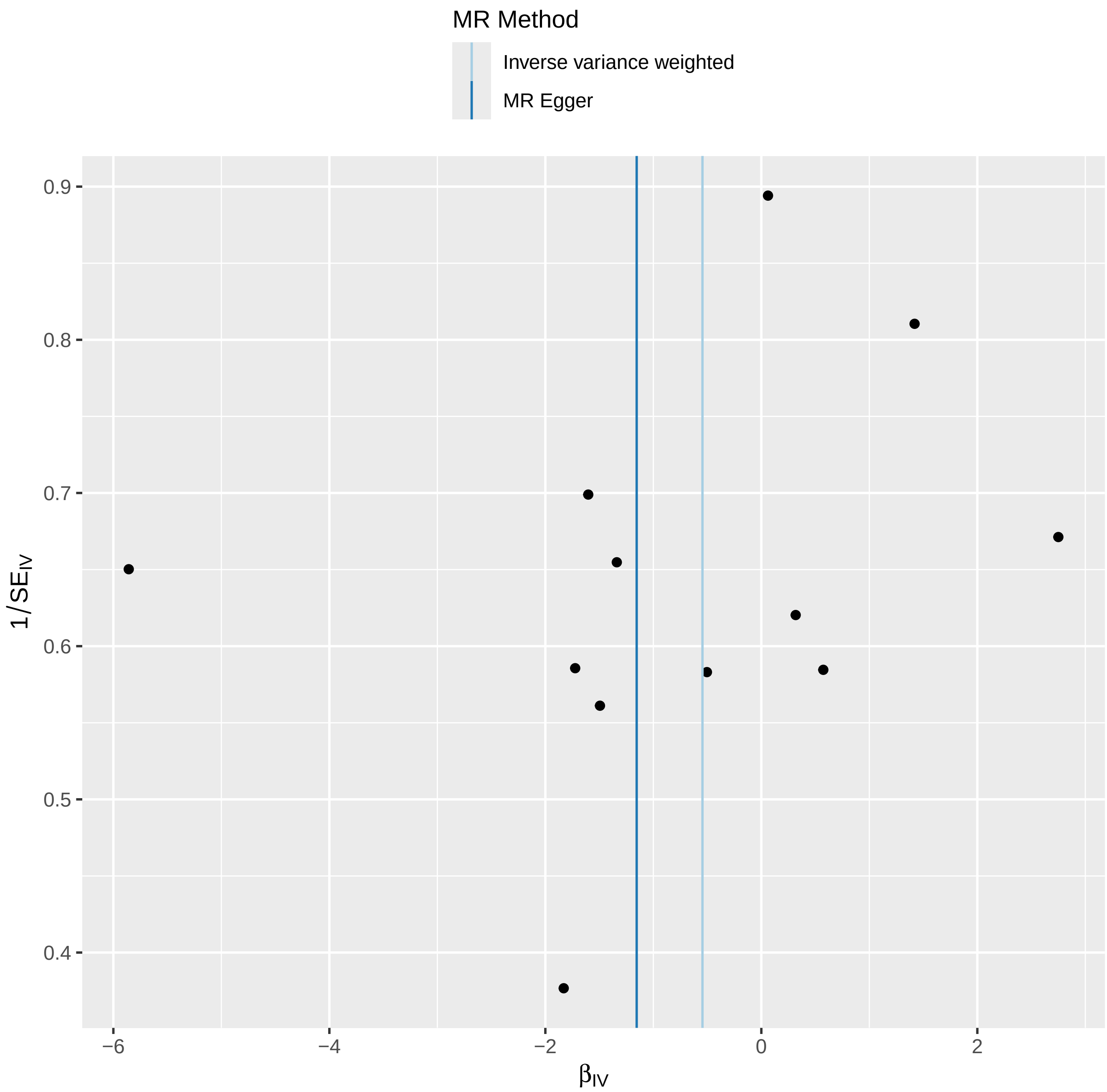
MR Method
Inverse variance weighted
MR Egger
0.9
0.8
0.7
0.6
0.5
0.4
1/SE$_{IV}$
−6
−4
−2
0
2
β$_{IV}$

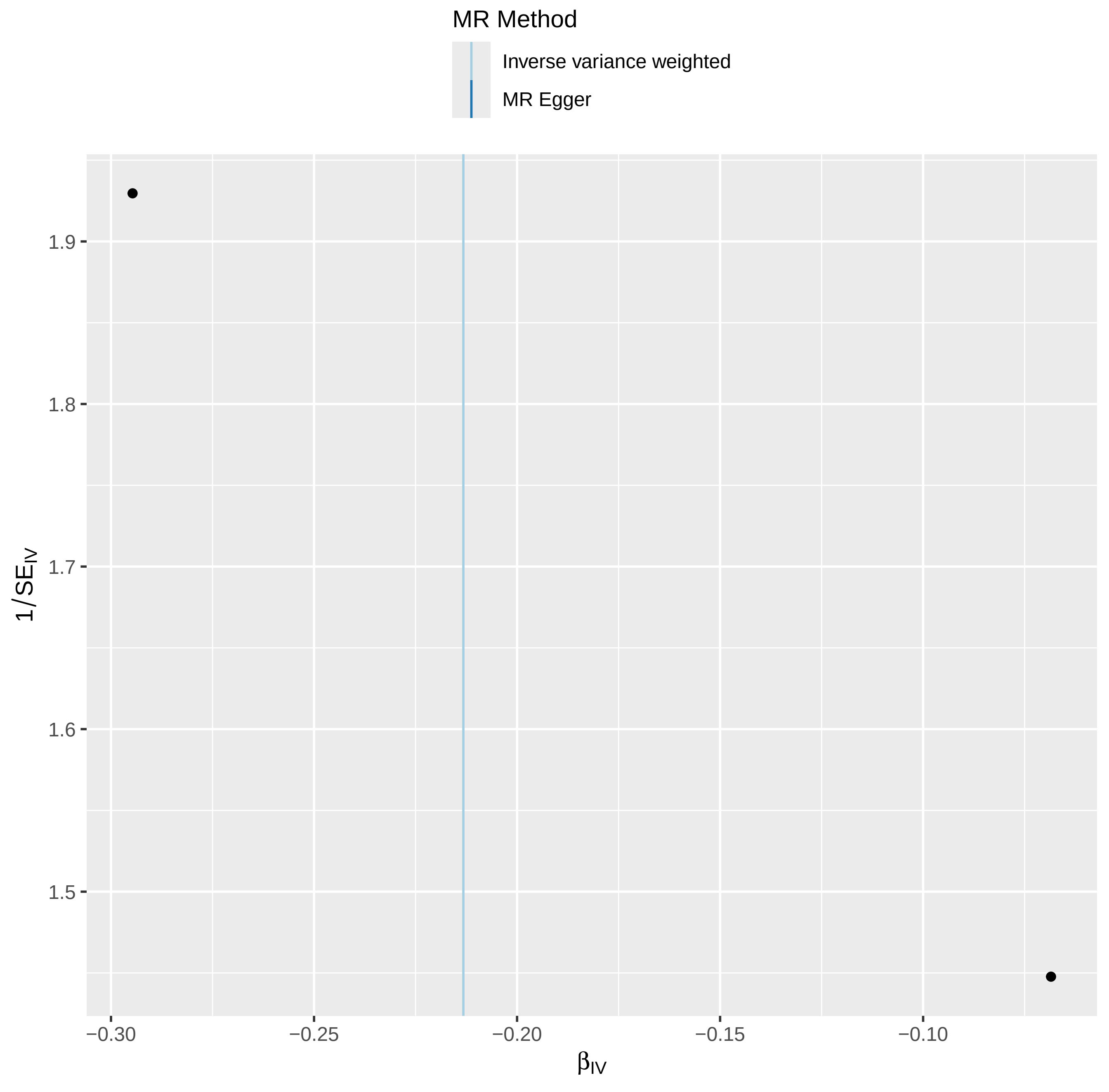

MR Method
Inverse variance weighted
MR Egger
1.9
1.8
1.7
1.6
1.5
1/SE_IV
−0.30
−0.25
−0.20
−0.15
−0.10
β_IV

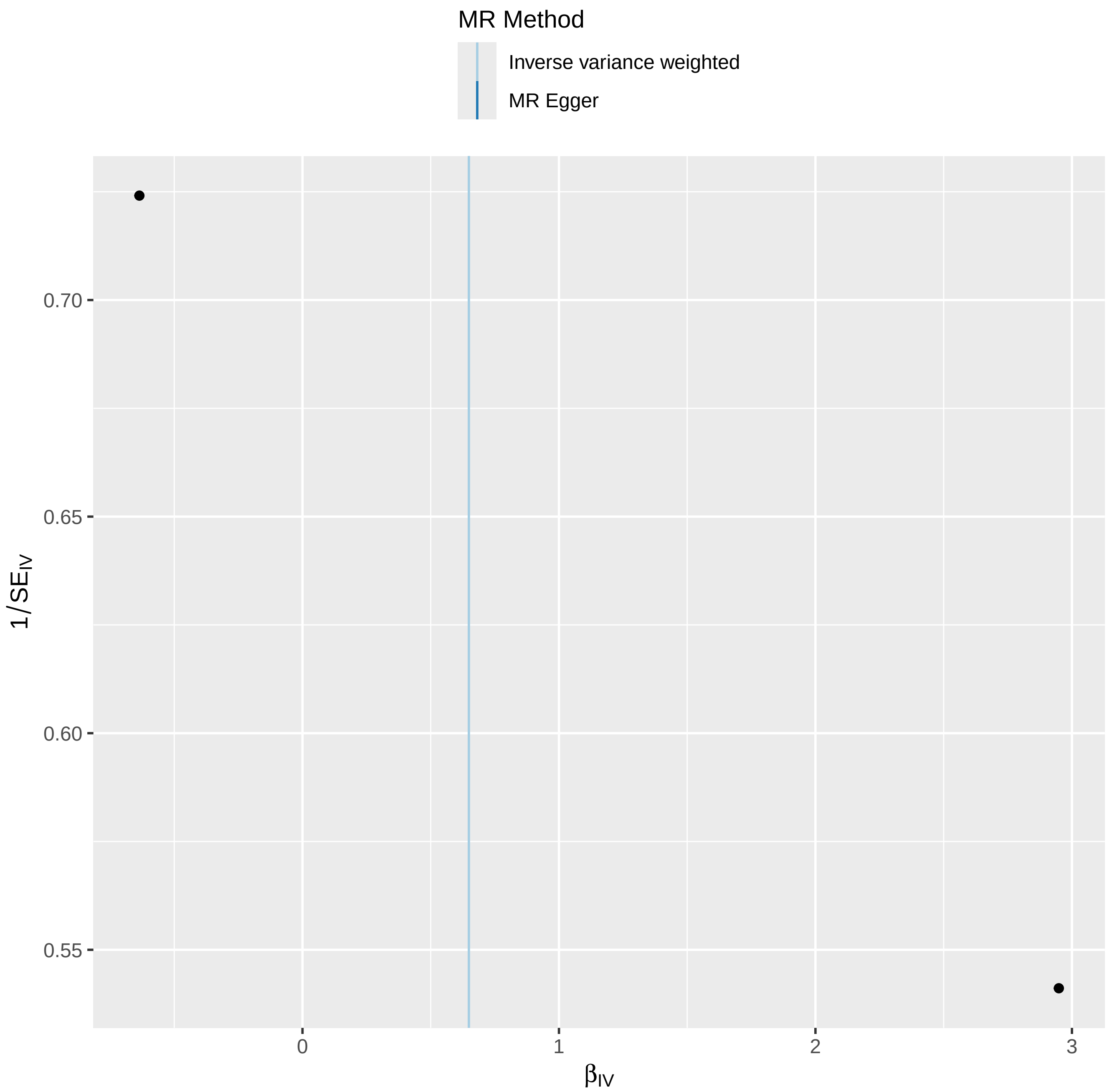
MR Method
Inverse variance weighted
MR Egger
0.70
0.65
0.60
0.55
1/SE_IV
0
1
2
3
β_IV

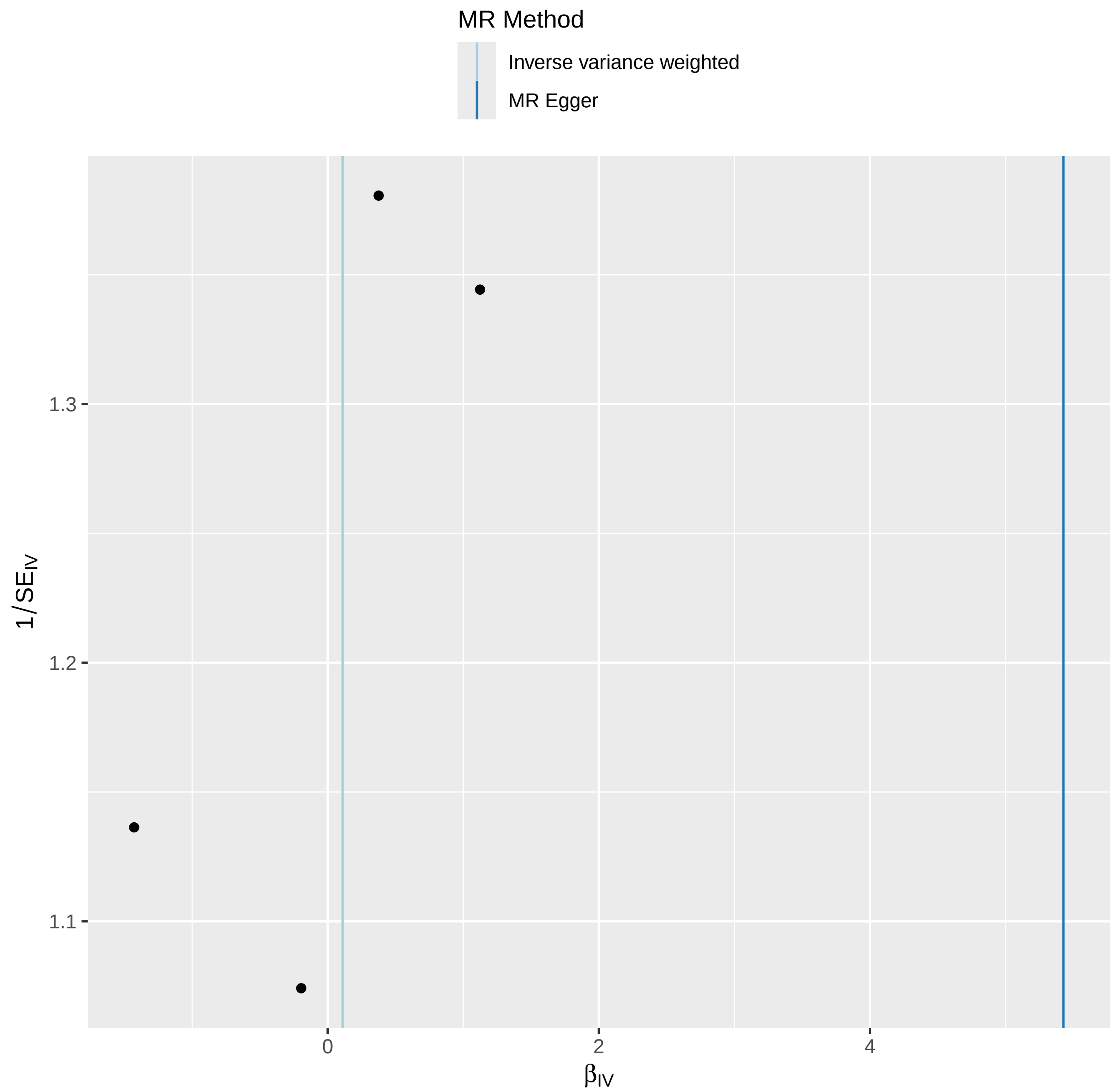

MR Method
Inverse variance weighted
MR Egger
1.3
1.2
1.1
$1/SE_{IV}$
0
2
4
β_{IV}

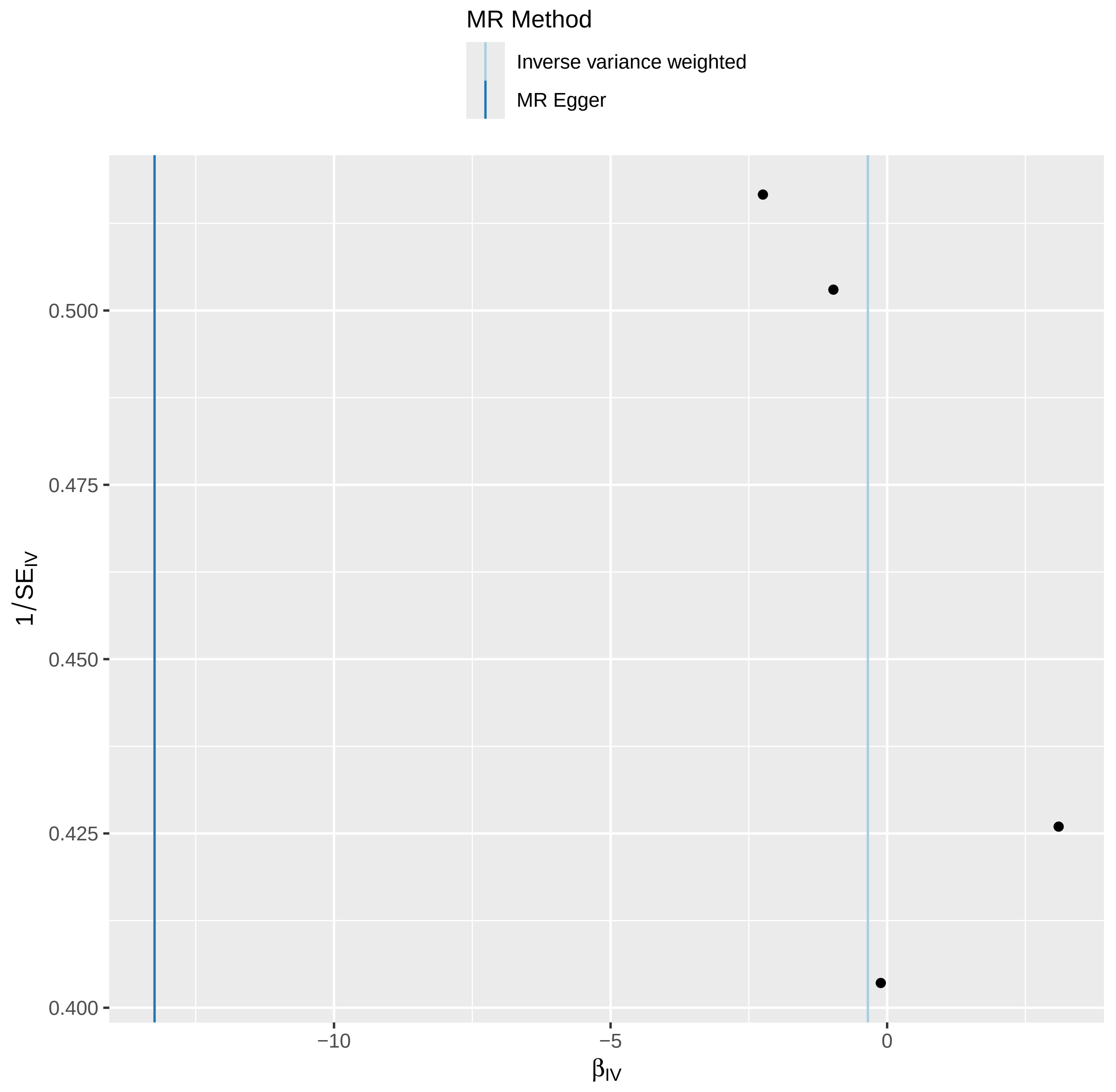

MR Method
Inverse variance weighted
MR Egger
0.500
0.475
0.450
0.425
0.400
1/SE_IV
−10
−5
0
β_IV

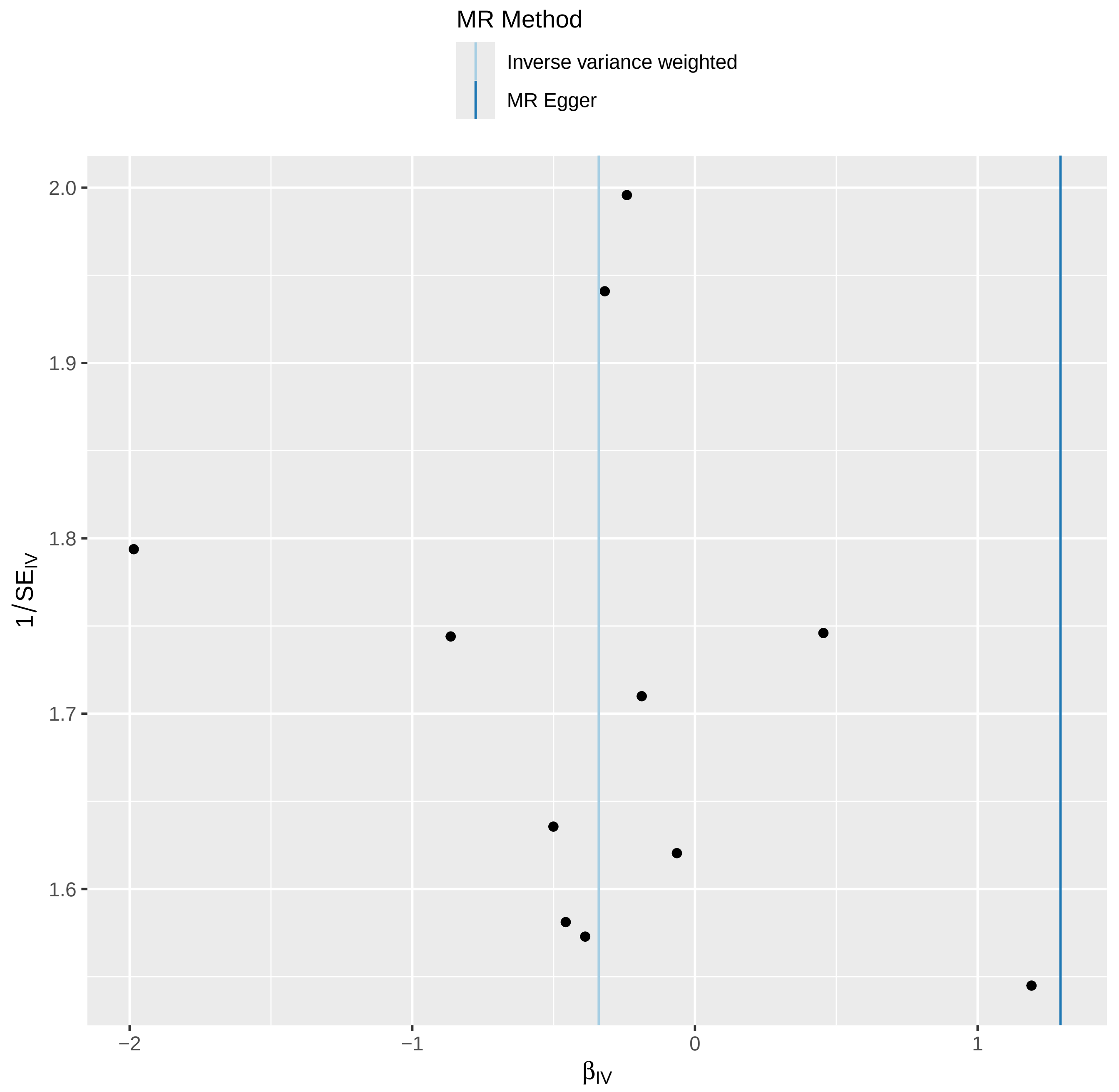

MR Method
Inverse variance weighted
MR Egger
2.0
1.9
1.8
1.7
1.6
1/SE_IV
−2
−1
0
1
β_IV

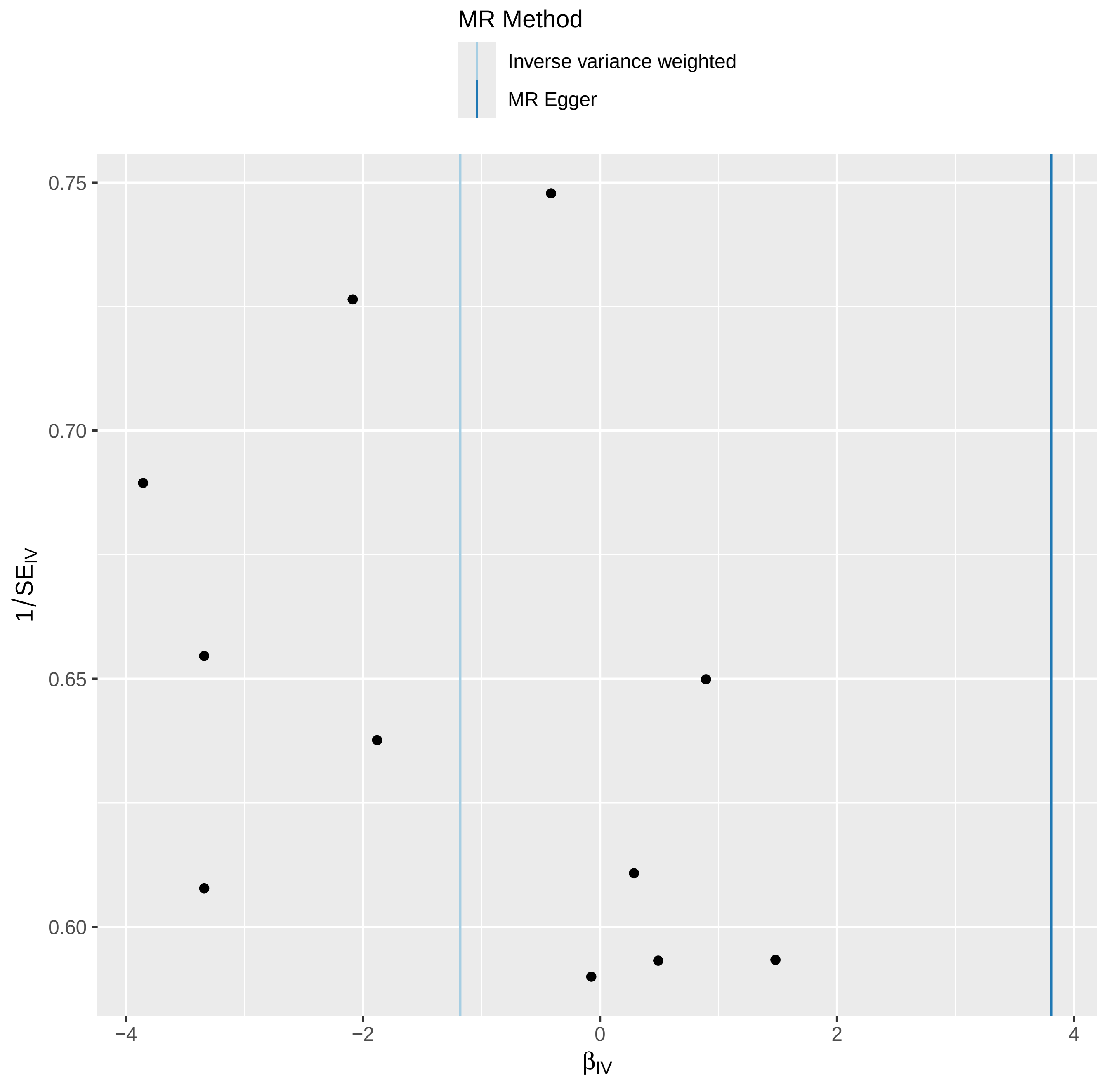
MR Method
Inverse variance weighted
MR Egger
0.75
0.70
0.65
0.60
$1/SE_{IV}$
−4
−2
0
2
4
β_{IV}

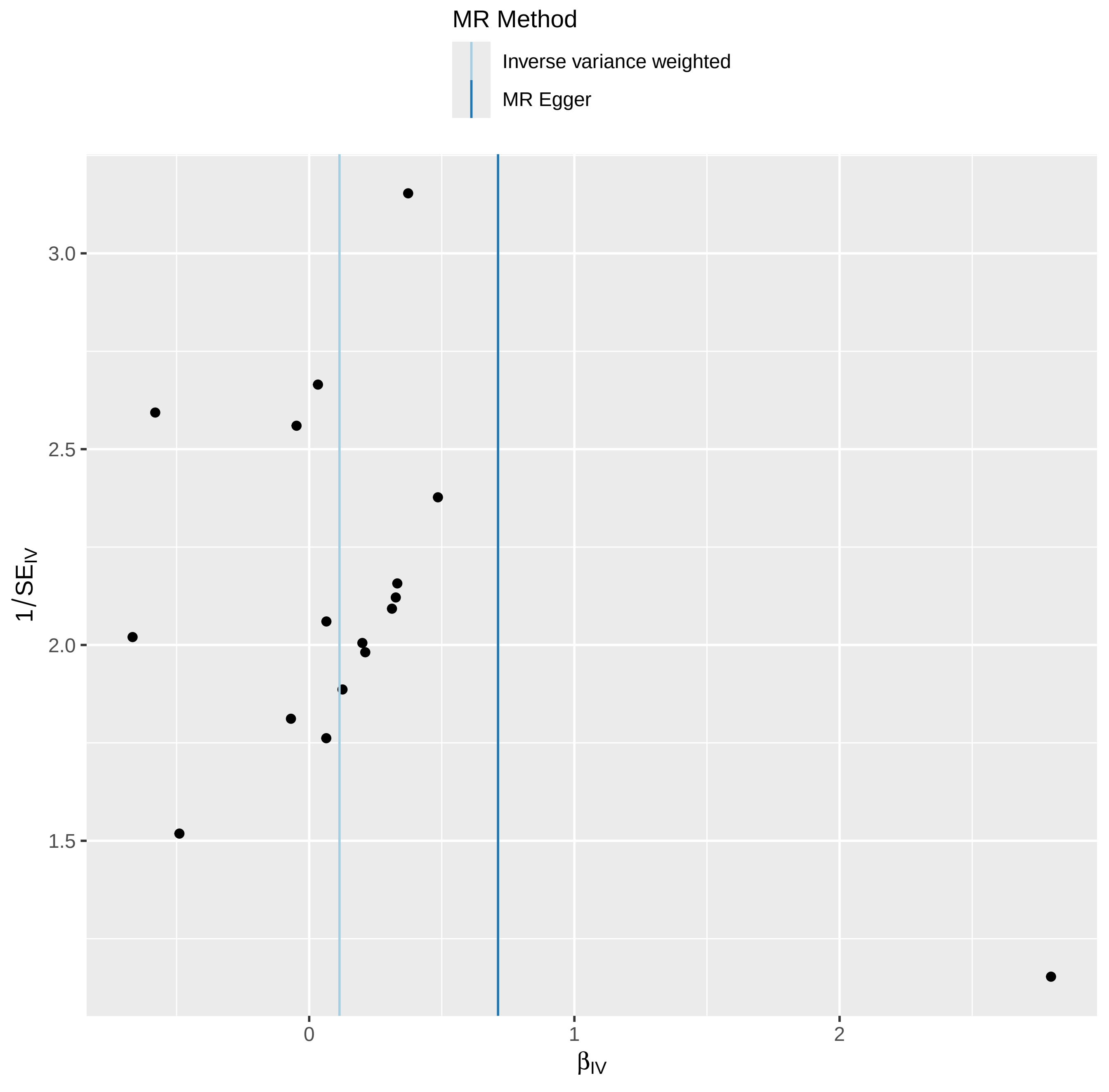
MR Method
Inverse variance weighted
MR Egger
3.0
2.5
2.0
1.5
$1/SE_{IV}$
0
1
2
β_{IV}

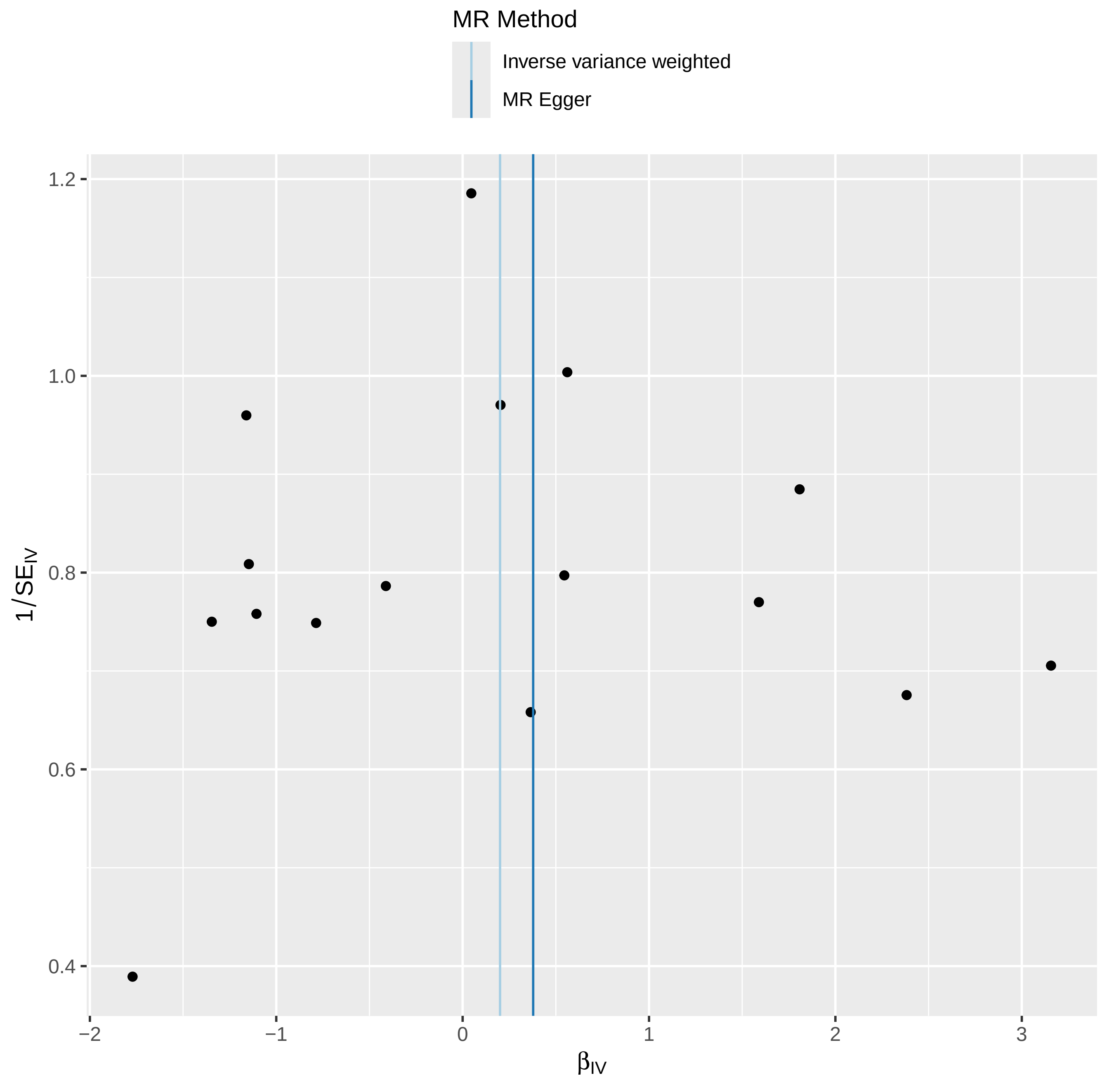
MR Method
Inverse variance weighted
MR Egger
1.2
1.0
0.8
0.6
0.4
1/SE_IV
−2
−1
0
1
2
3
β_IV

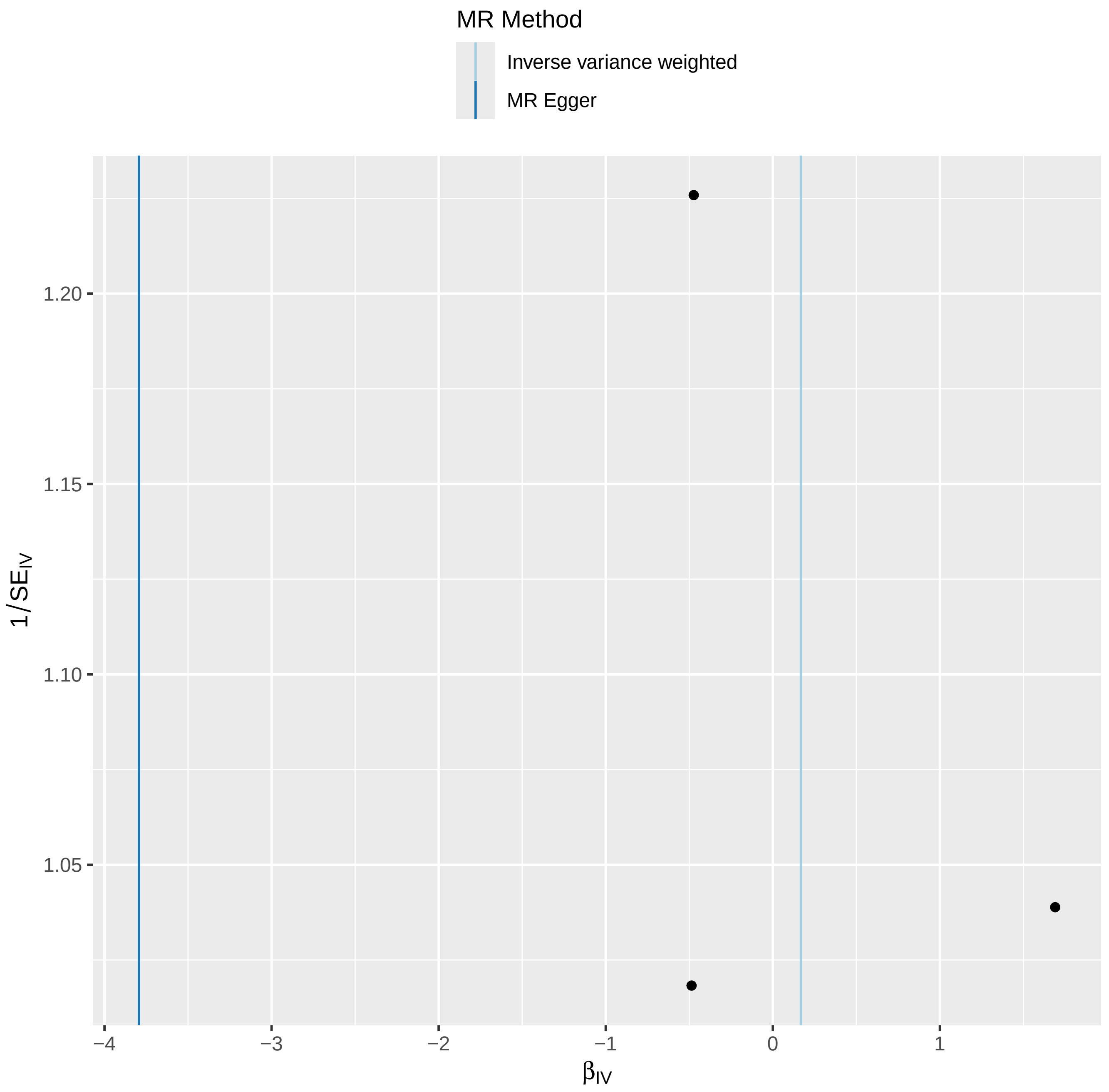
MR Method
Inverse variance weighted
MR Egger
1.20
1.15
1.10
1.05
1/SE_IV
−4
−3
−2
−1
0
1
β_IV

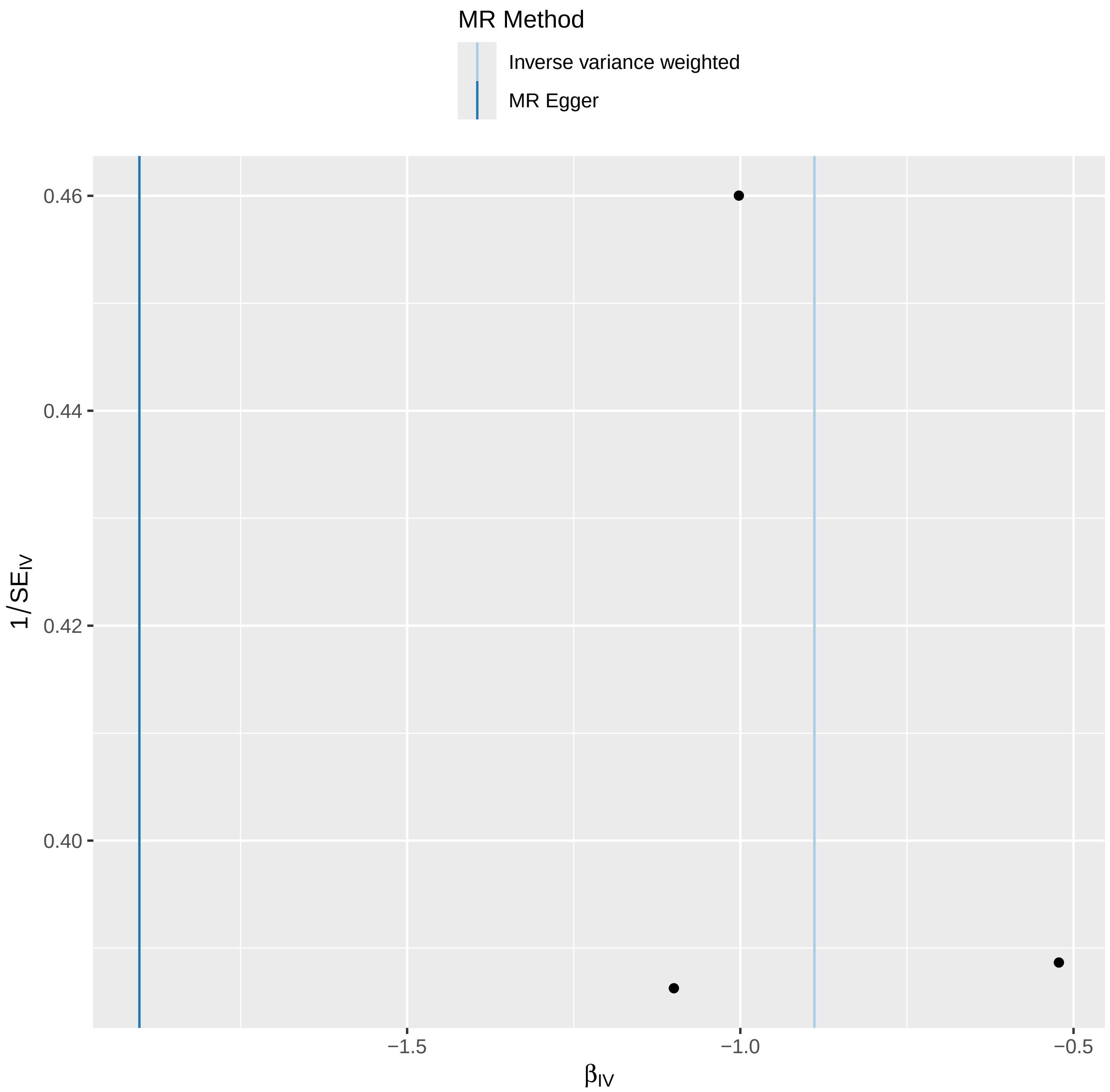
MR Method
Inverse variance weighted
MR Egger
0.46
0.44
0.42
0.40
$1/SE_{IV}$
−1.5
−1.0
−0.5
β_{IV}

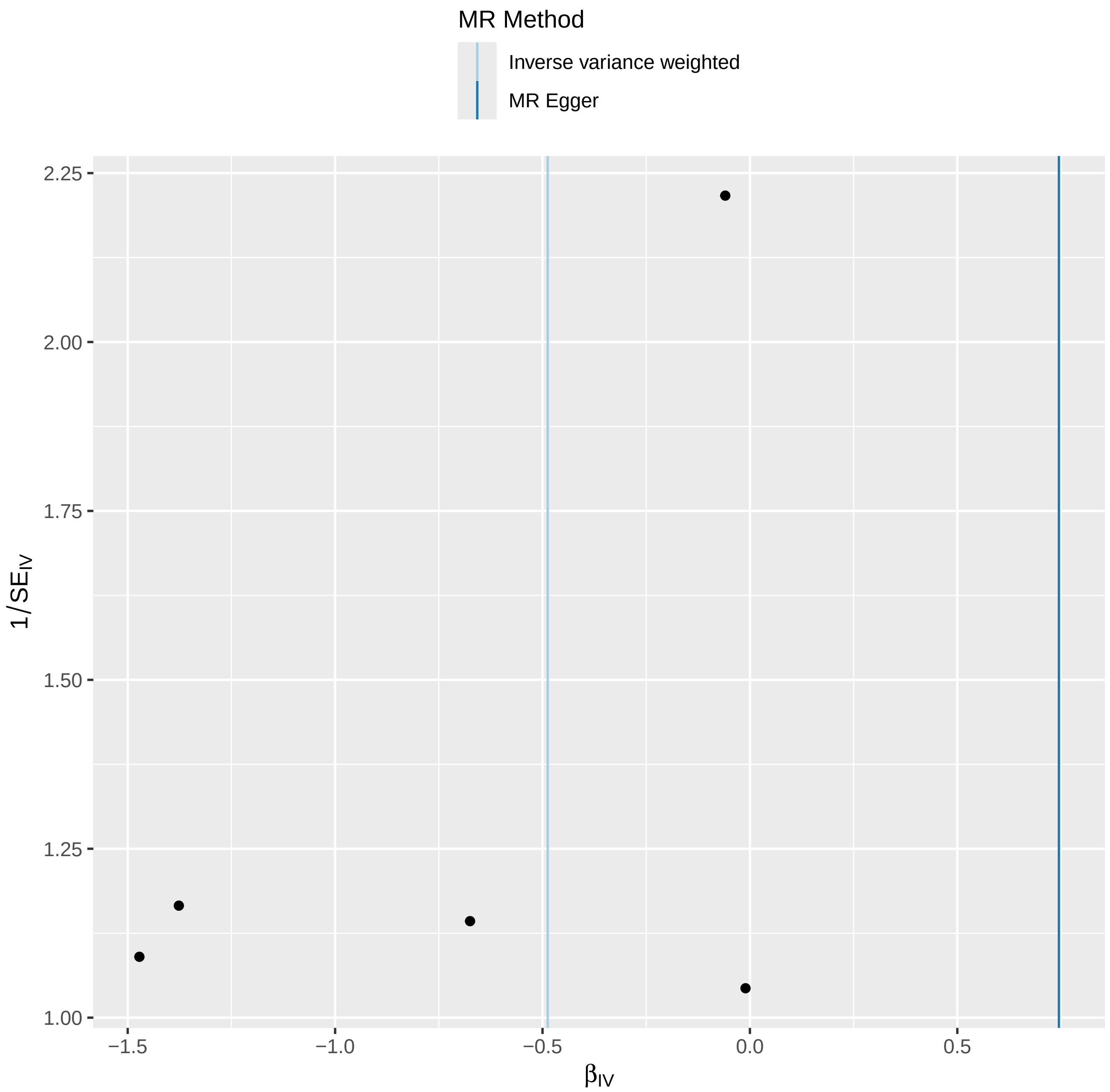

MR Method
Inverse variance weighted
MR Egger
2.25
2.00
1.75
1.50
1.25
1.00
1/SE_IV
−1.5
−1.0
−0.5
0.0
0.5
β_IV

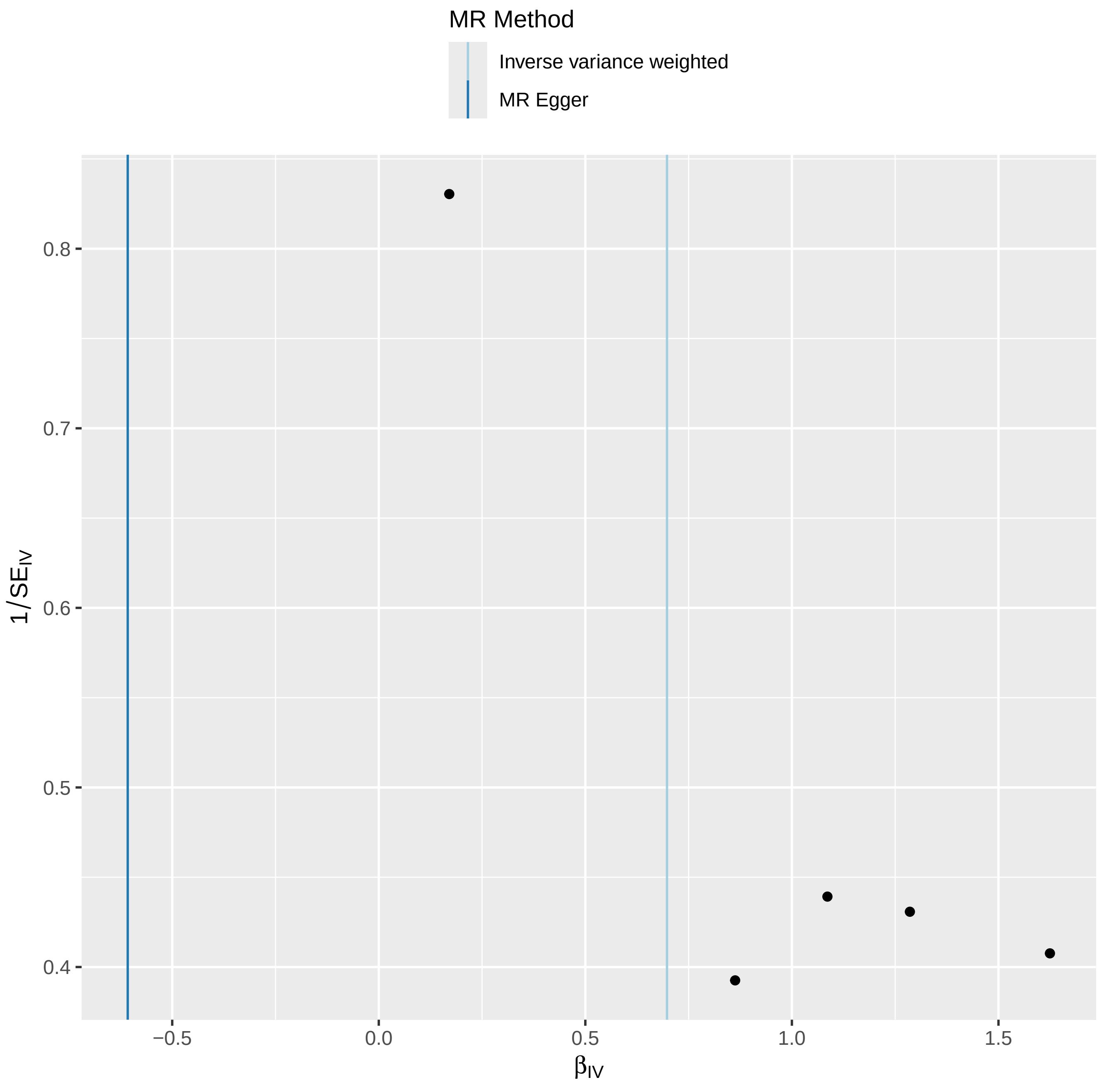
MR Method
Inverse variance weighted
MR Egger
0.8
0.7
0.6
0.5
0.4
1/SE_IV
−0.5
0.0
0.5
1.0
1.5
β_IV

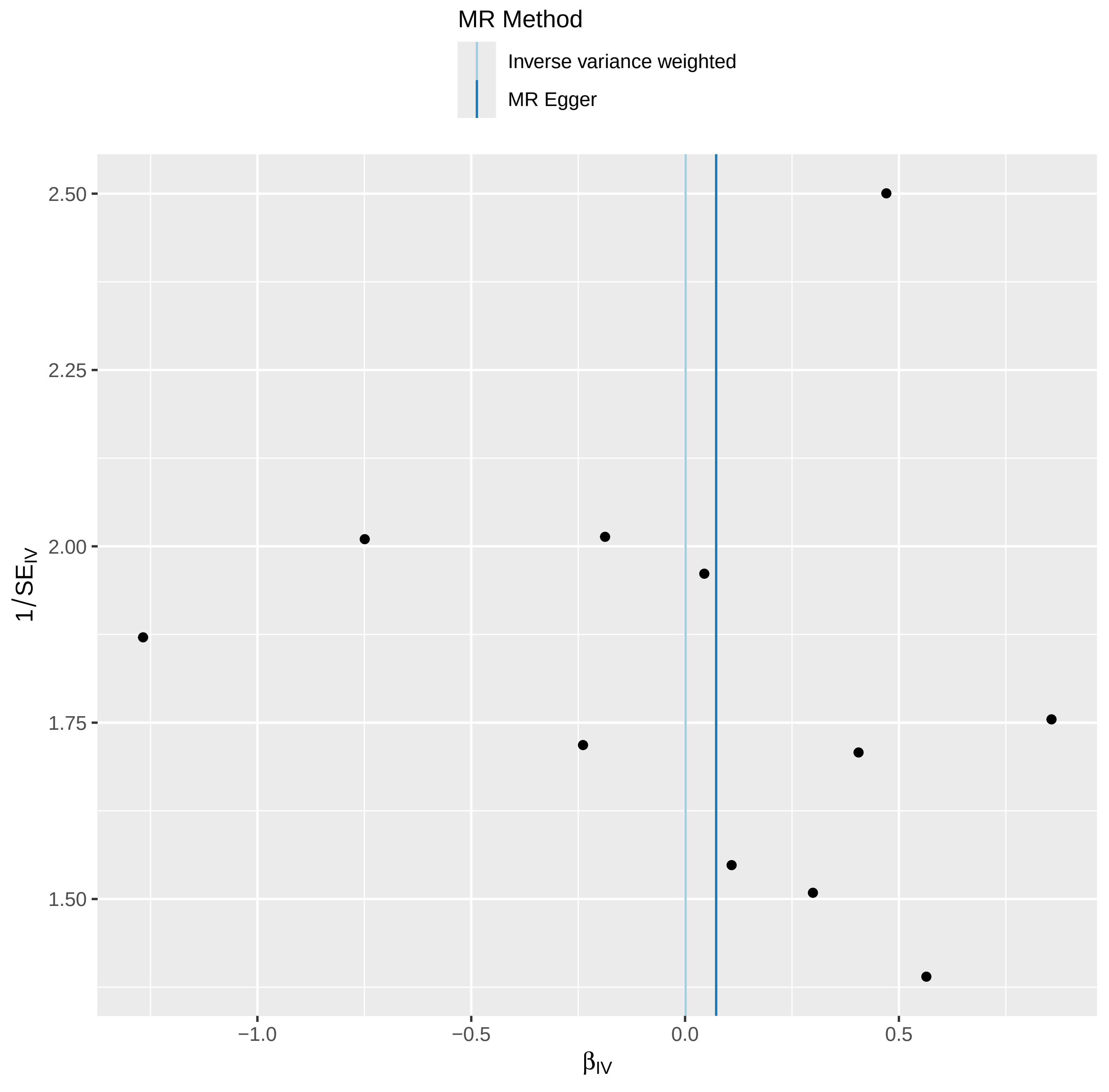
MR Method
Inverse variance weighted
MR Egger
2.50
2.25
2.00
1.75
1.50
1/SE_IV
−1.0
−0.5
0.0
0.5
β_IV

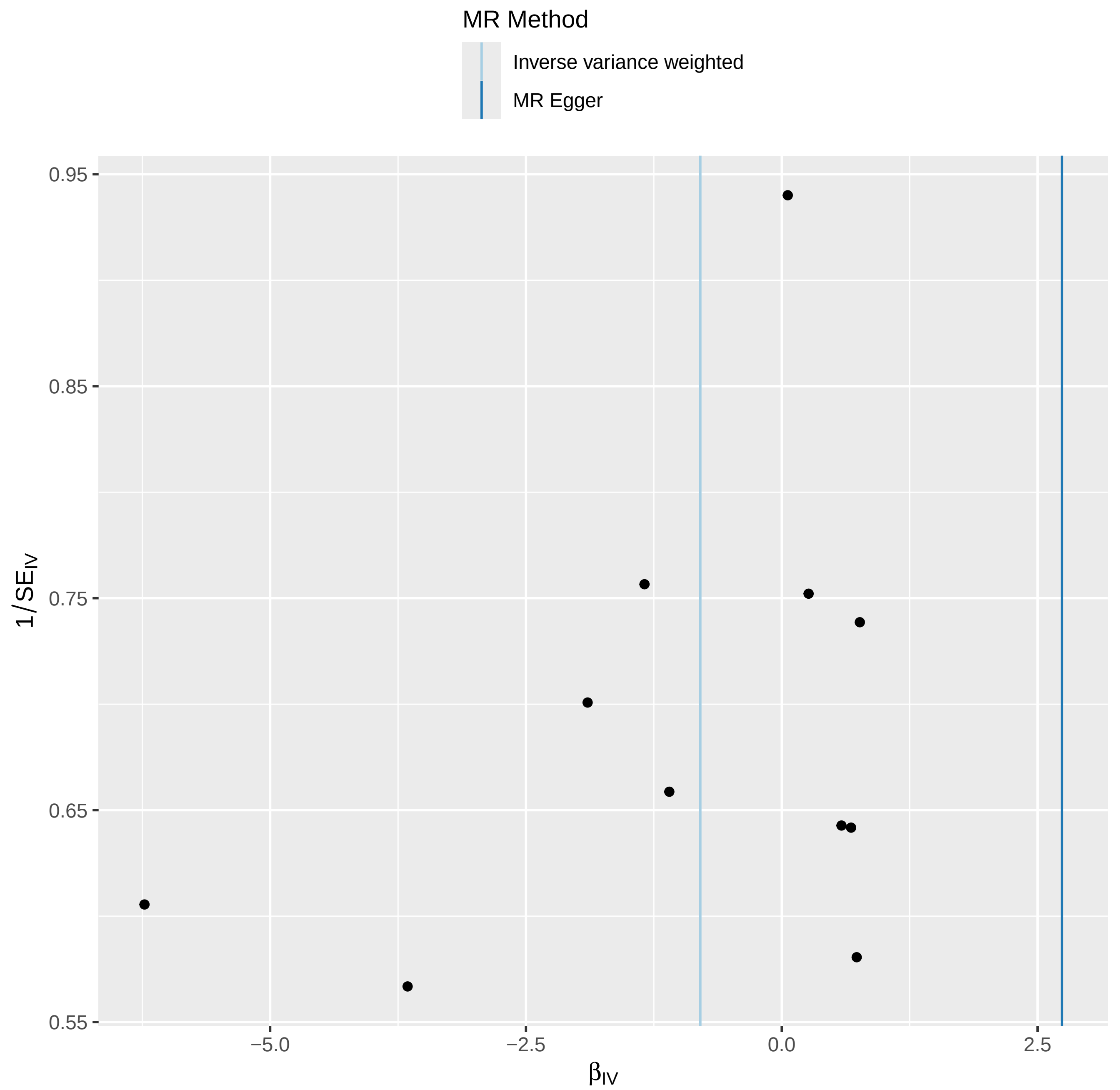

MR Method
Inverse variance weighted
MR Egger
0.95
0.85
0.75
0.65
0.55
1/SE_IV
−5.0
−2.5
0.0
2.5
β_IV

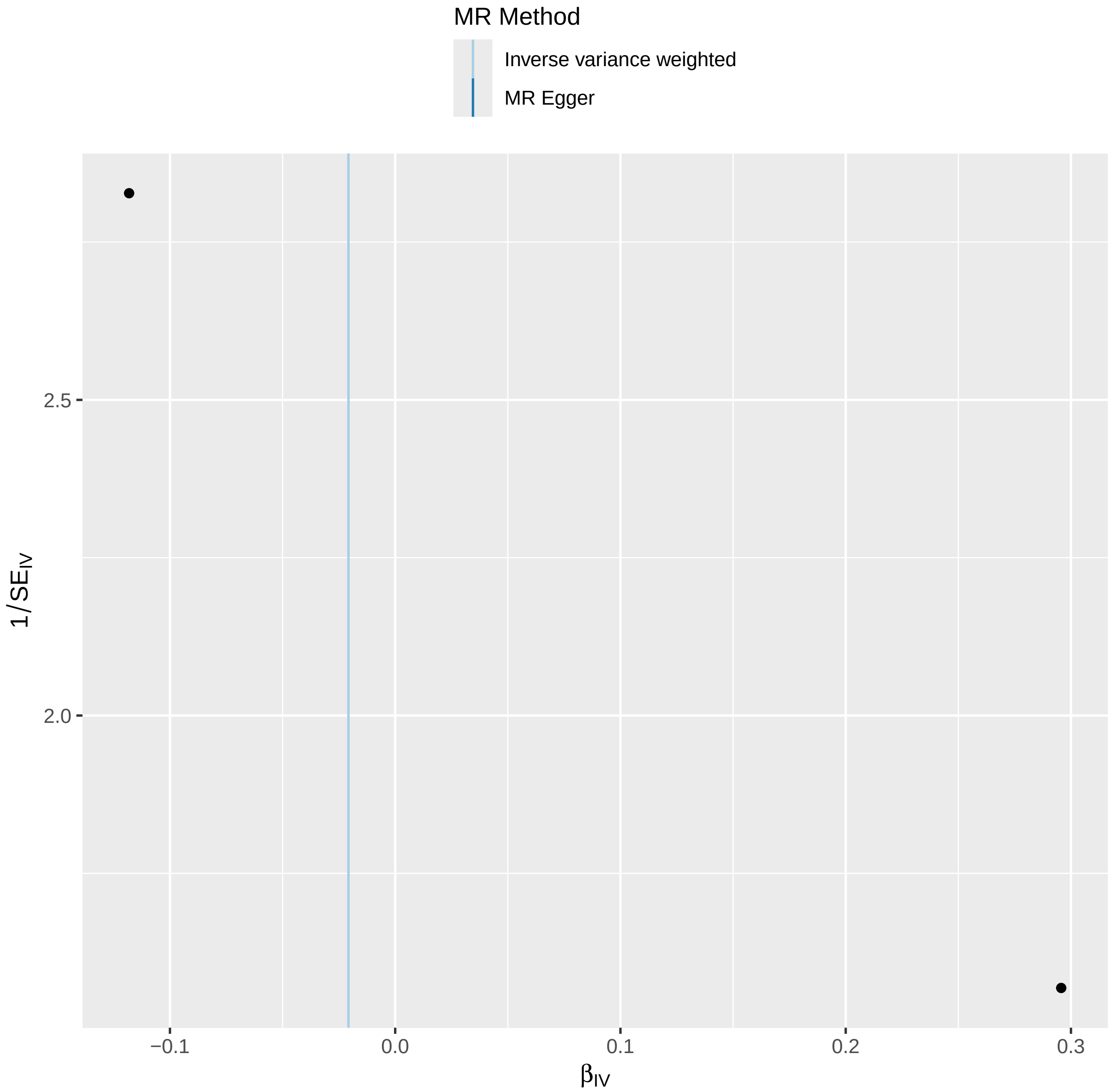
MR Method
Inverse variance weighted
MR Egger
2.5
2.0
1/SE_IV
−0.1
0.0
0.1
0.2
0.3
β_IV

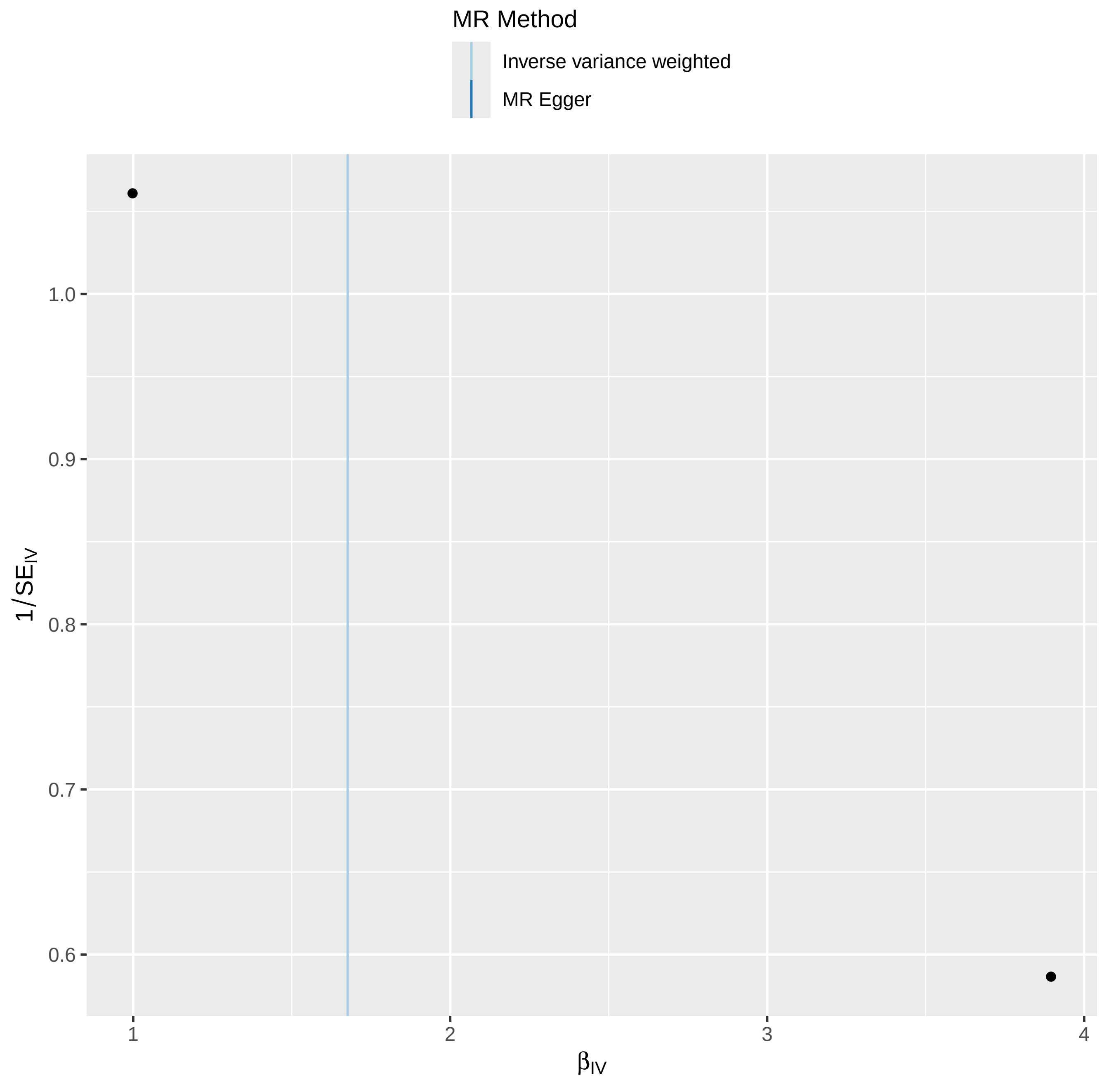
MR Method
Inverse variance weighted
MR Egger
1.0
0.9
0.8
0.7
0.6
1/SE_IV
1
2
3
4
β_IV

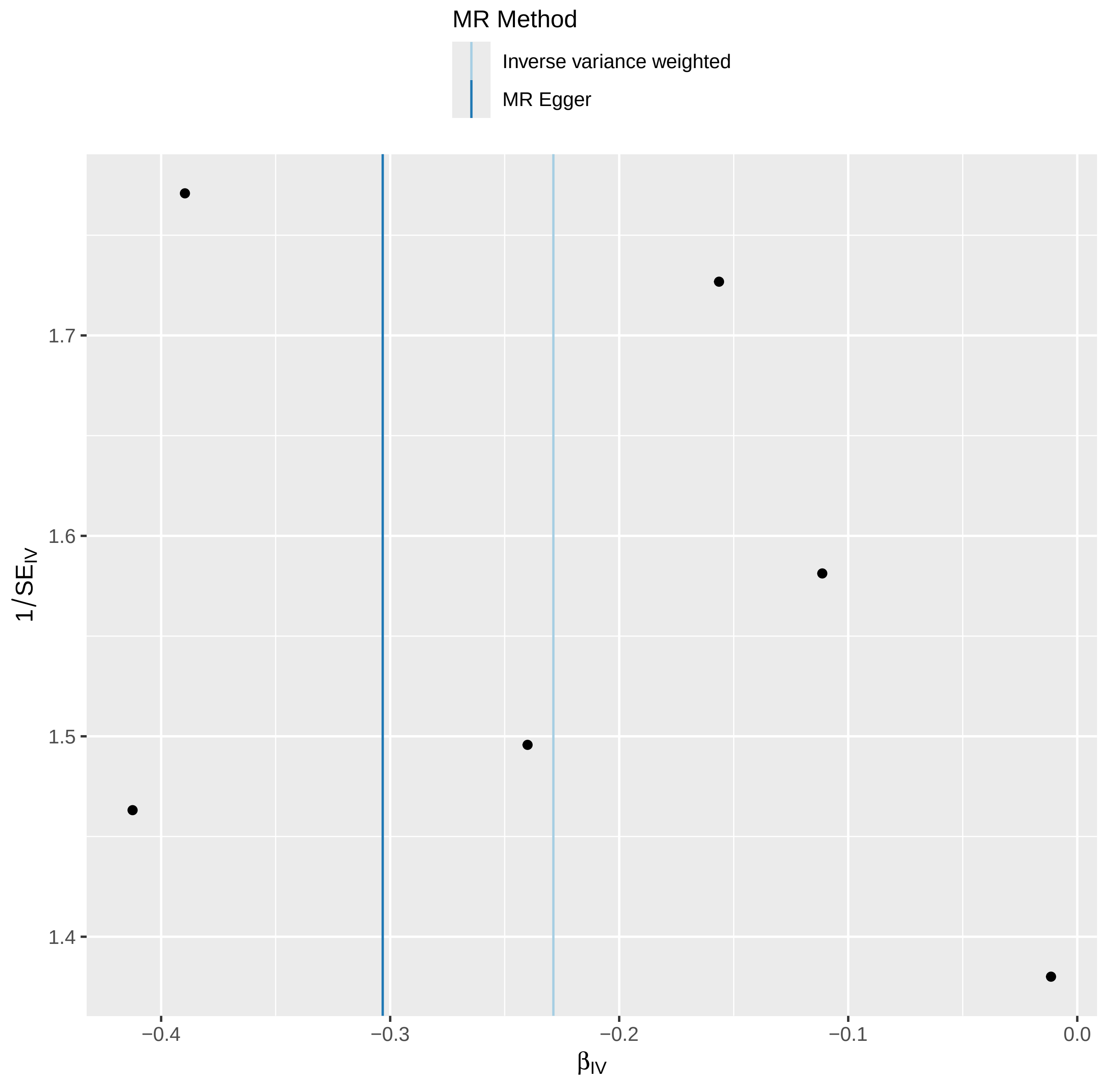

MR Method
Inverse variance weighted
MR Egger
1.7
1.6
1.5
1.4
1/SE_IV
−0.4
−0.3
−0.2
−0.1
0.0
β_IV

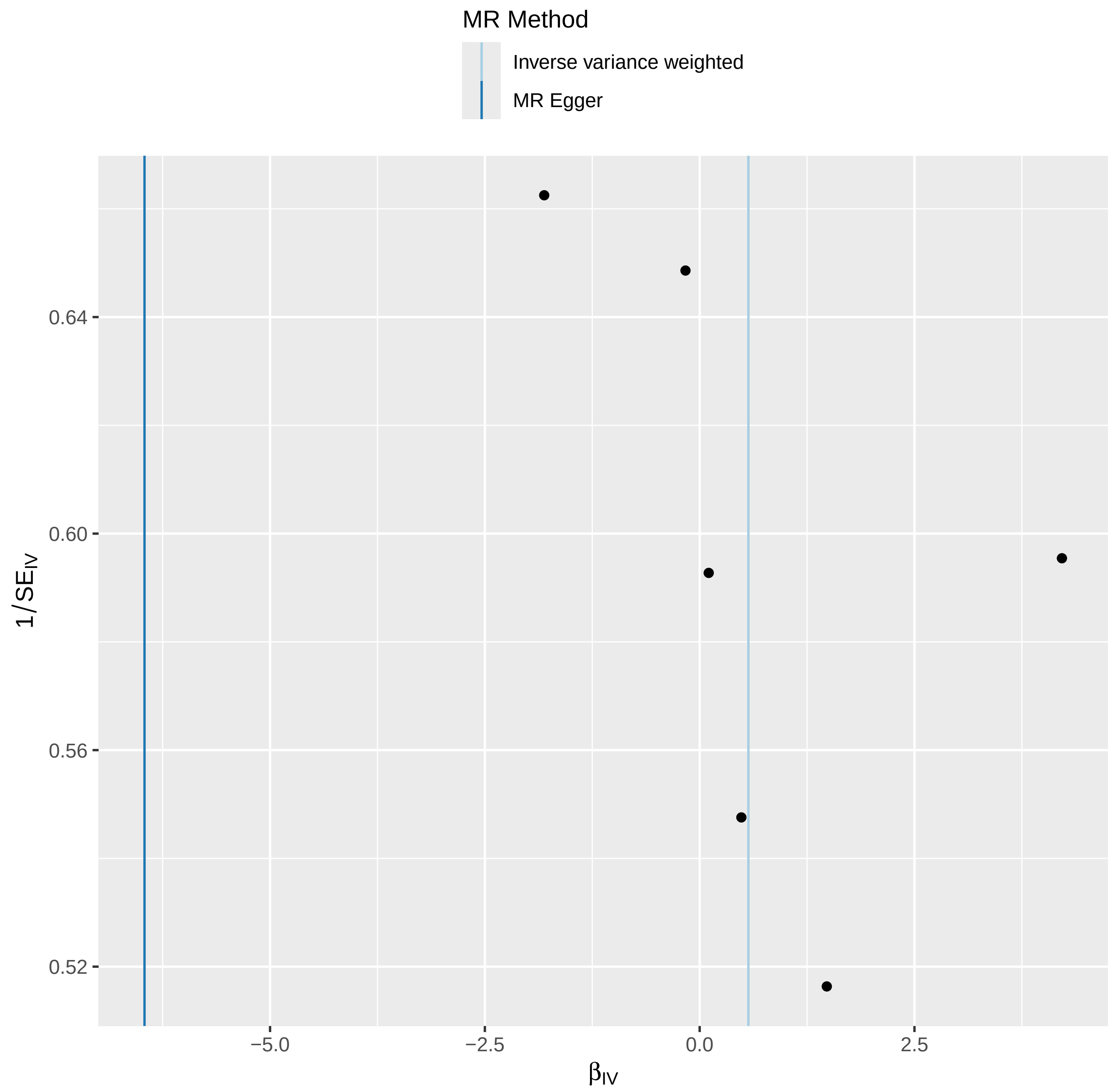
MR Method
Inverse variance weighted
MR Egger
0.64
0.60
0.56
0.52
1/SE_IV
−5.0
−2.5
0.0
2.5
β_IV

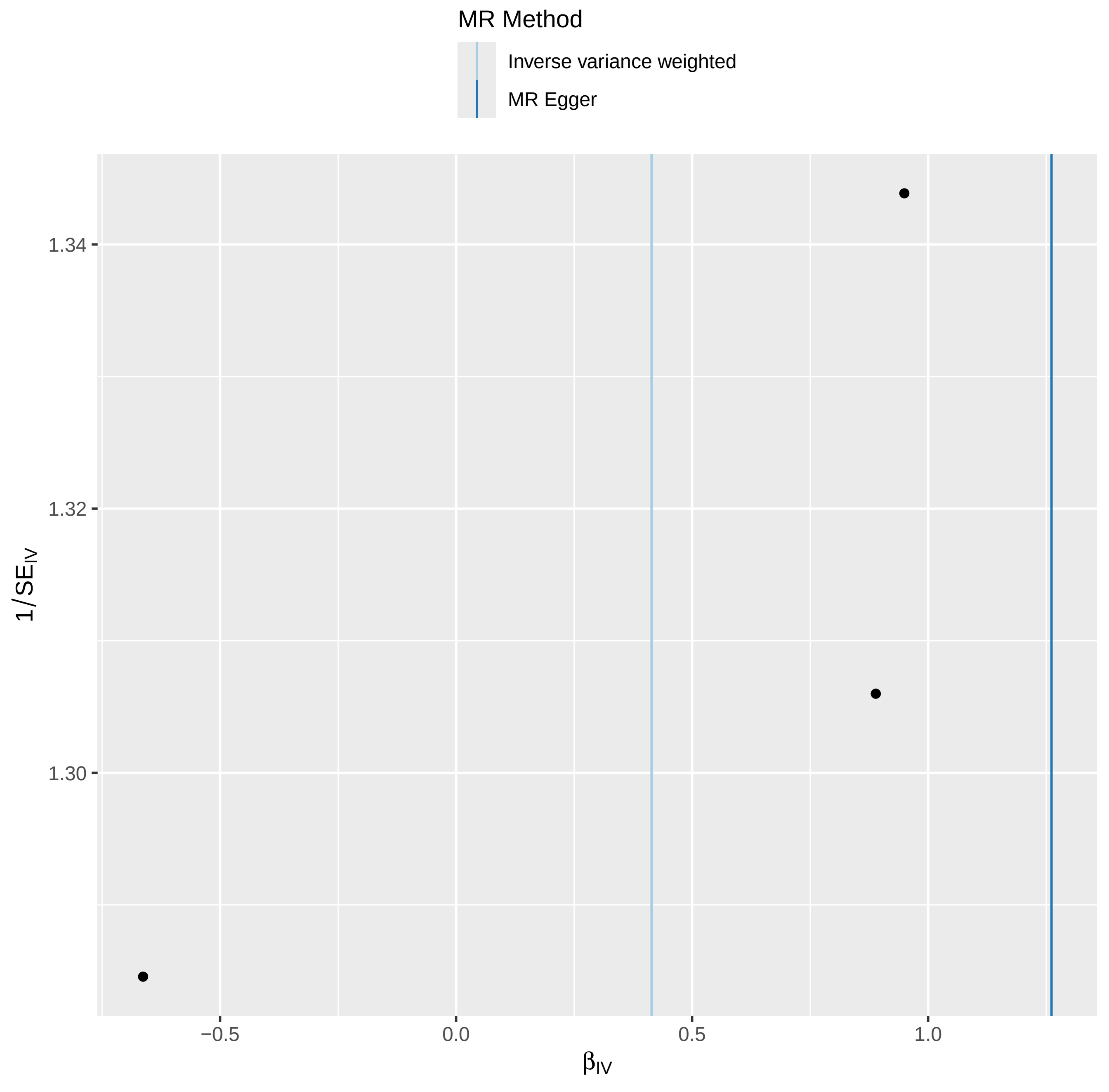
MR Method
Inverse variance weighted
MR Egger
1.34
1.32
1.30
1/SE_IV
−0.5
0.0
0.5
1.0
β_IV

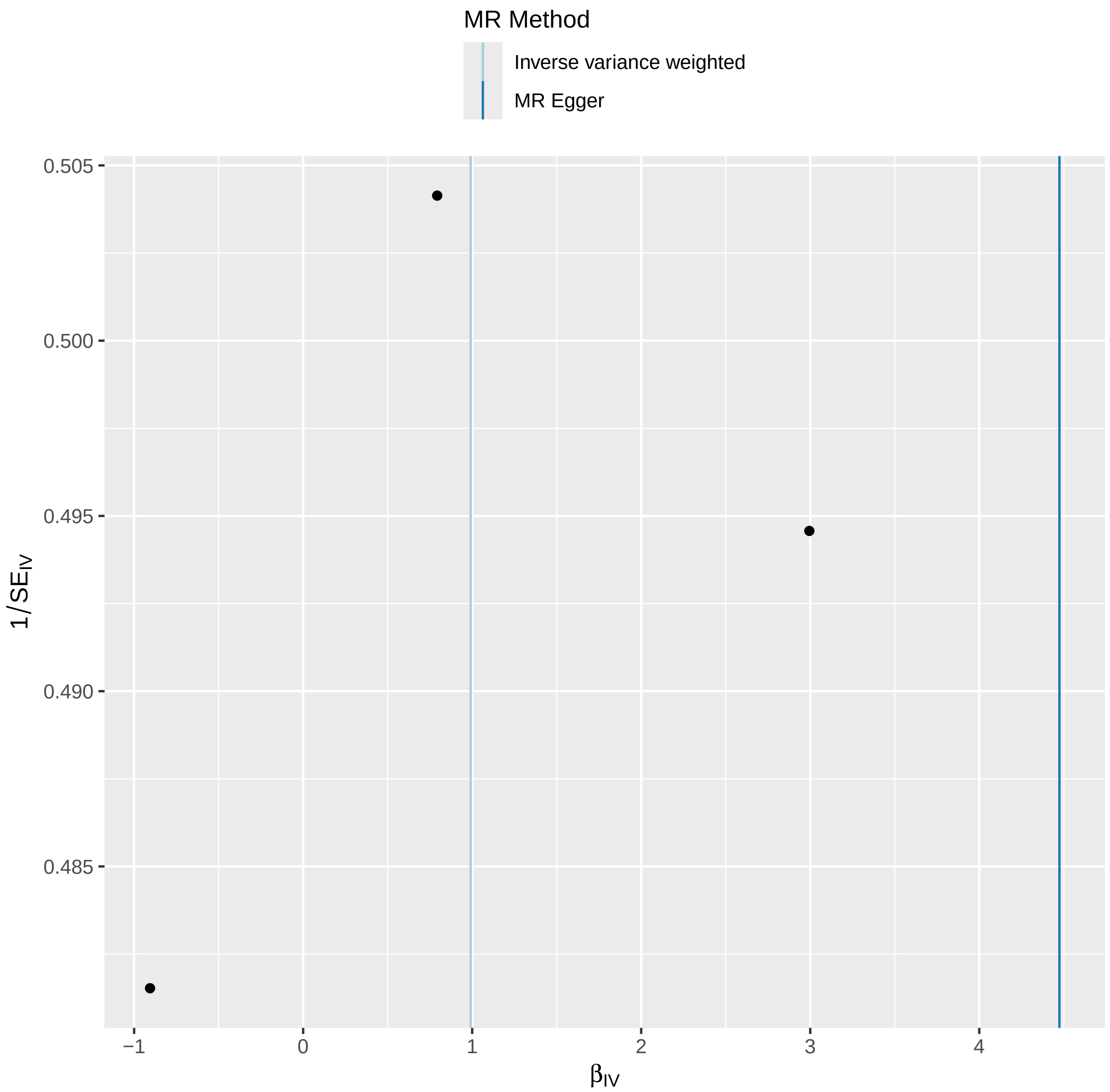

MR Method
Inverse variance weighted
MR Egger
0.505
0.500
0.495
0.490
0.485
1/SE_IV
−1
0
1
2
3
4
β_IV

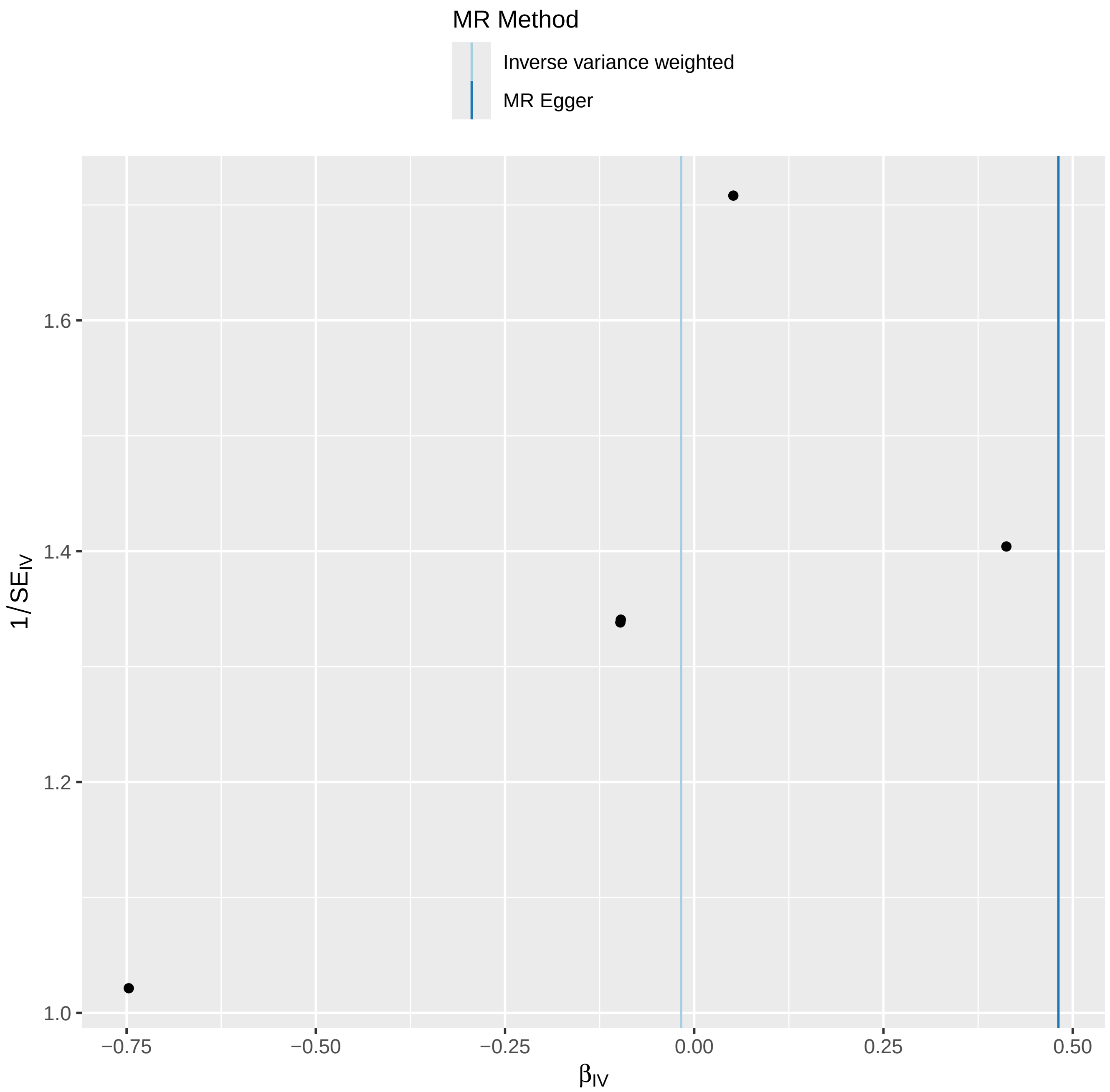
MR Method
Inverse variance weighted
MR Egger
1.6
1.4
1.2
1.0
1/SE$_{IV}$
−0.75
−0.50
−0.25
0.00
0.25
0.50
β$_{IV}$

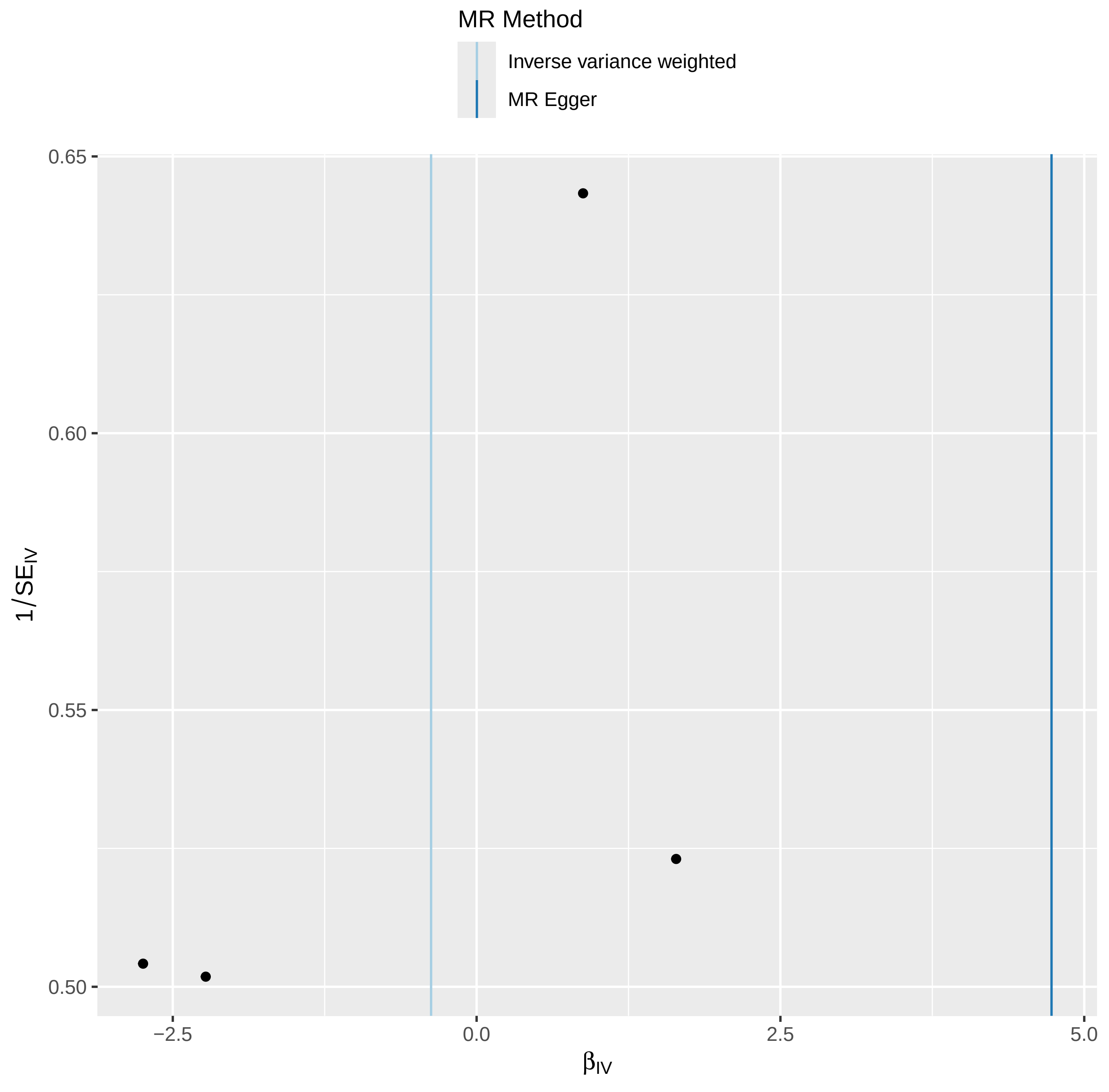
MR Method
Inverse variance weighted
MR Egger
0.65
0.60
0.55
0.50
1/SE_IV
−2.5
0.0
2.5
5.0
β_IV

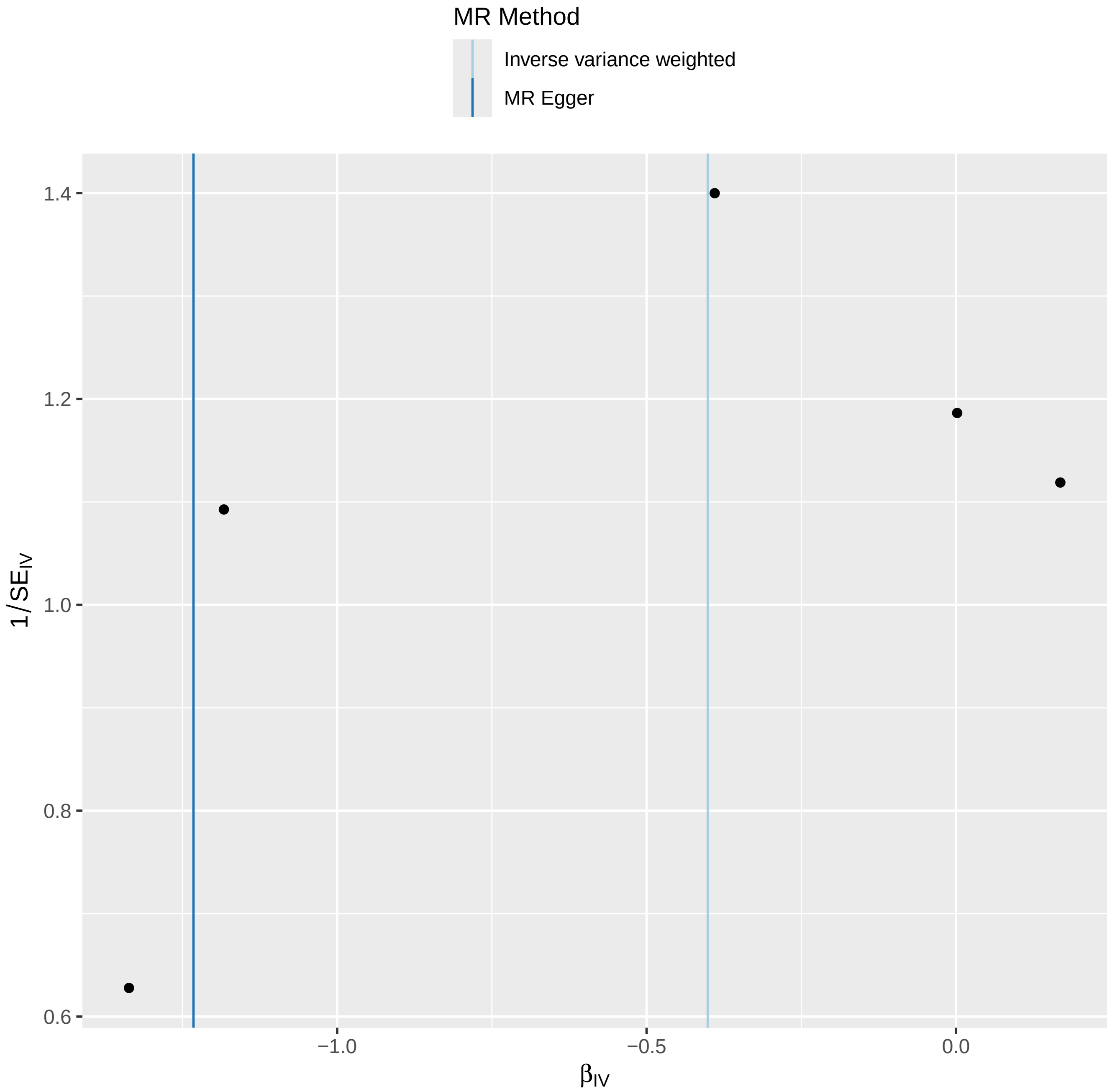
MR Method
Inverse variance weighted
MR Egger
1.4
1.2
1.0
0.8
0.6
1/SE_IV
−1.0
−0.5
0.0
β_IV

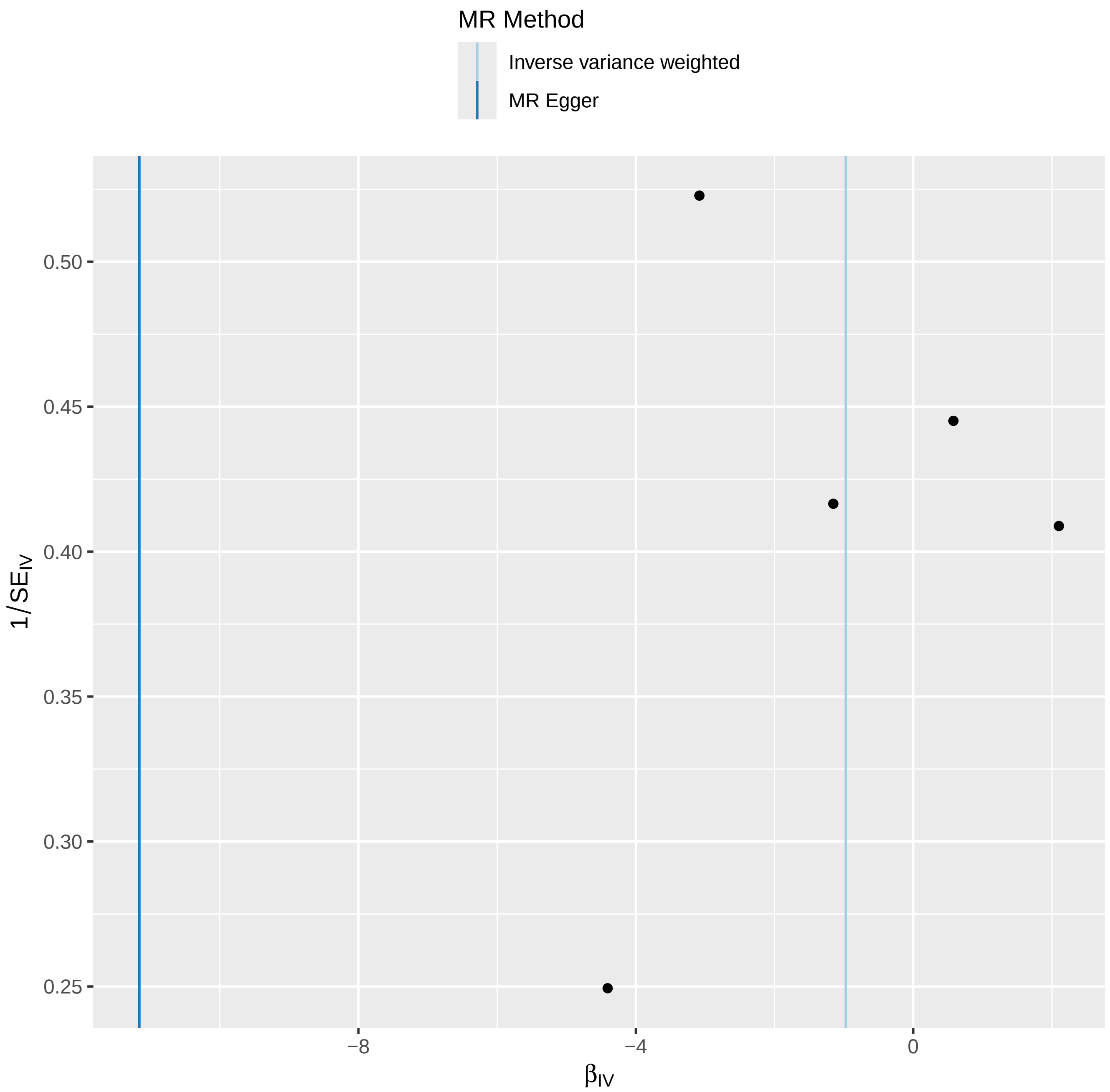
MR Method
Inverse variance weighted
MR Egger
0.50
0.45
0.40
0.35
0.30
0.25
1/SE_IV
−8
−4
0
β_IV

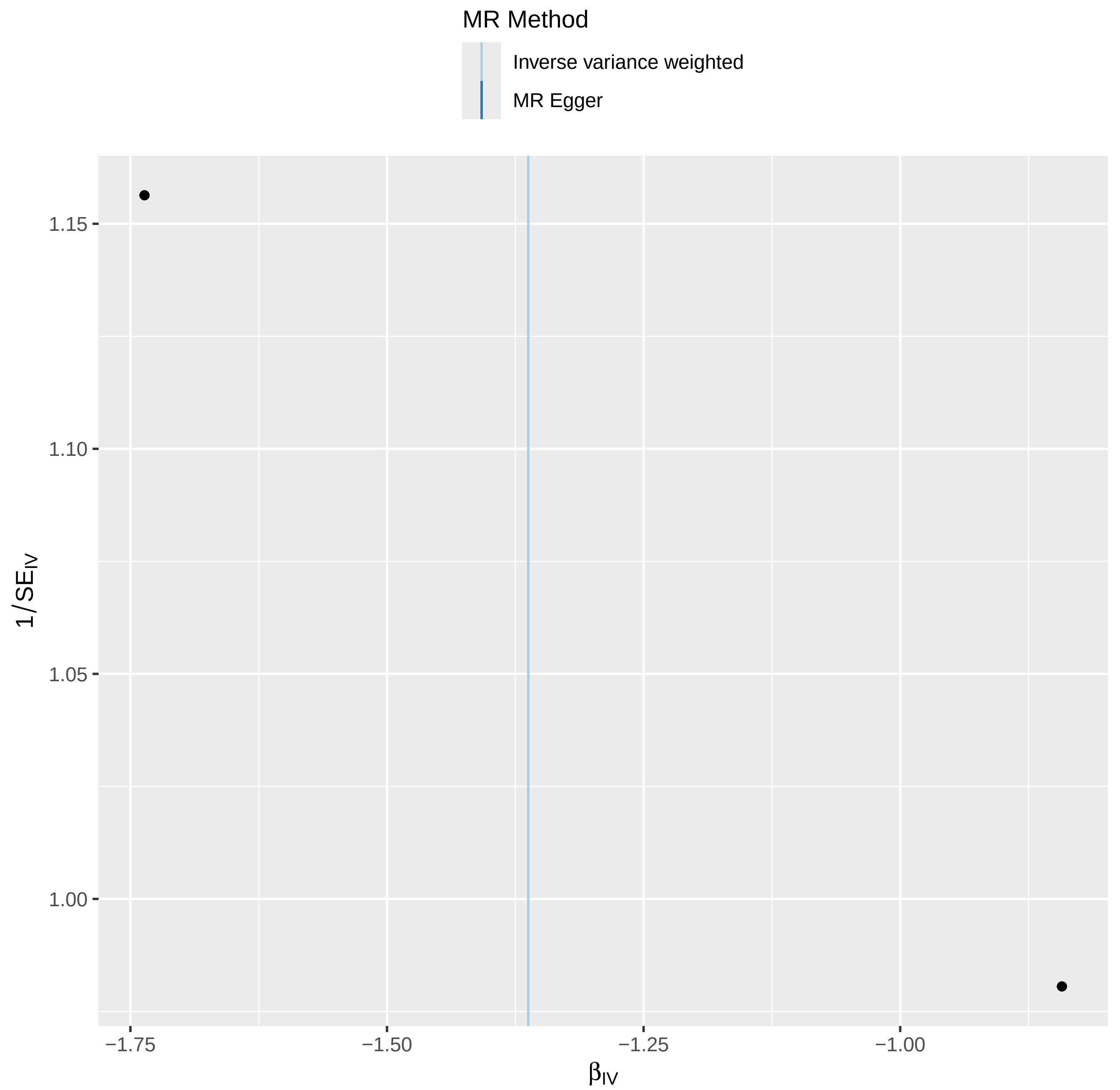

MR Method
Inverse variance weighted
MR Egger
1.15
1.10
1.05
1.00
1/SE_IV
−1.75
−1.50
−1.25
−1.00
β_IV

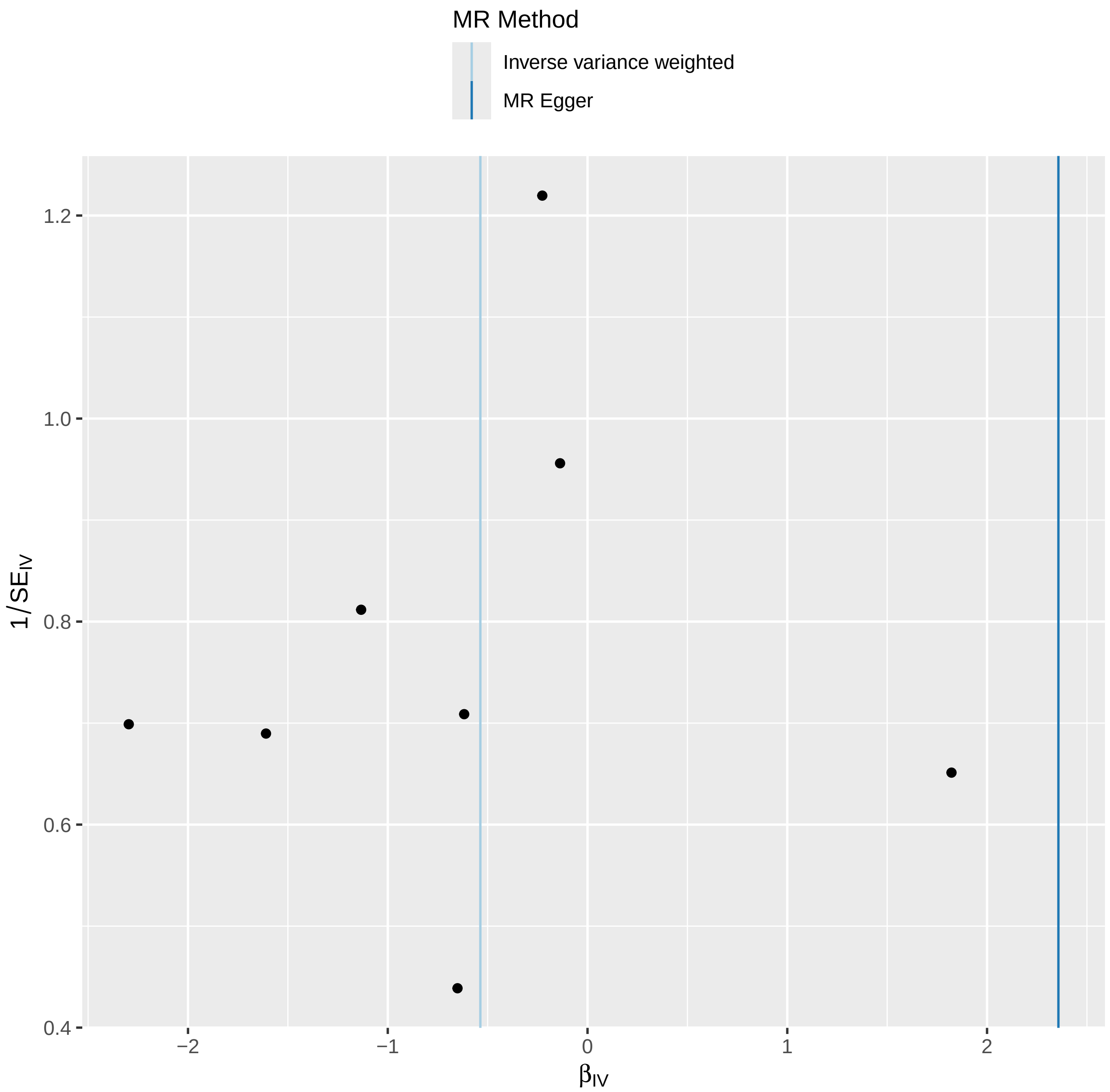
MR Method
Inverse variance weighted
MR Egger
1.2
1.0
0.8
0.6
0.4
1/SE_IV
−2
−1
0
1
2
β_IV

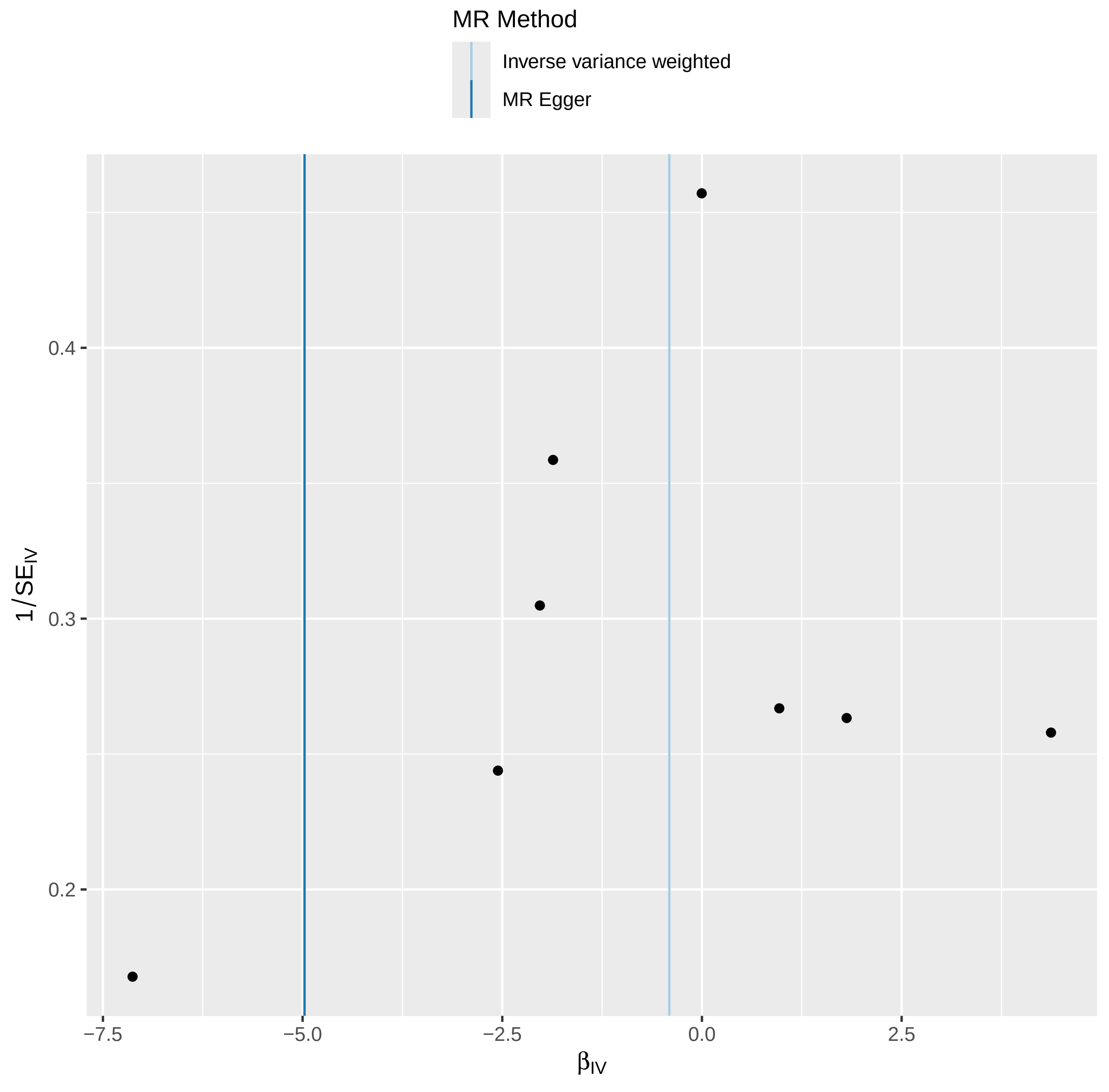

MR Method
Inverse variance weighted
MR Egger
0.4
0.3
0.2
−7.5
−5.0
−2.5
0.0
2.5
$1/SE_{IV}$
β_{IV}

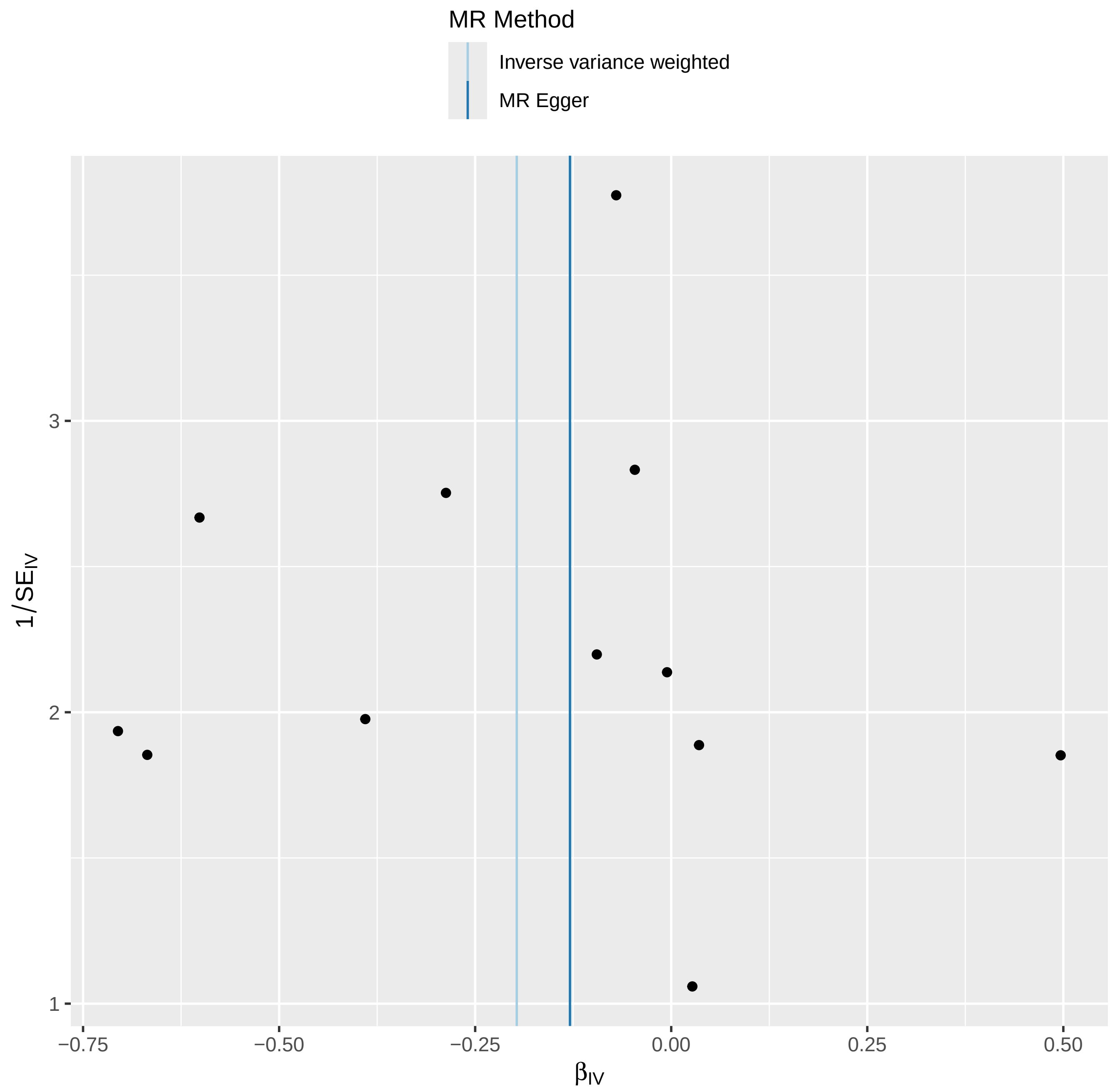

MR Method
Inverse variance weighted
MR Egger
3
2
1
$1/SE_{IV}$
−0.75
−0.50
−0.25
0.00
0.25
0.50
β_{IV}

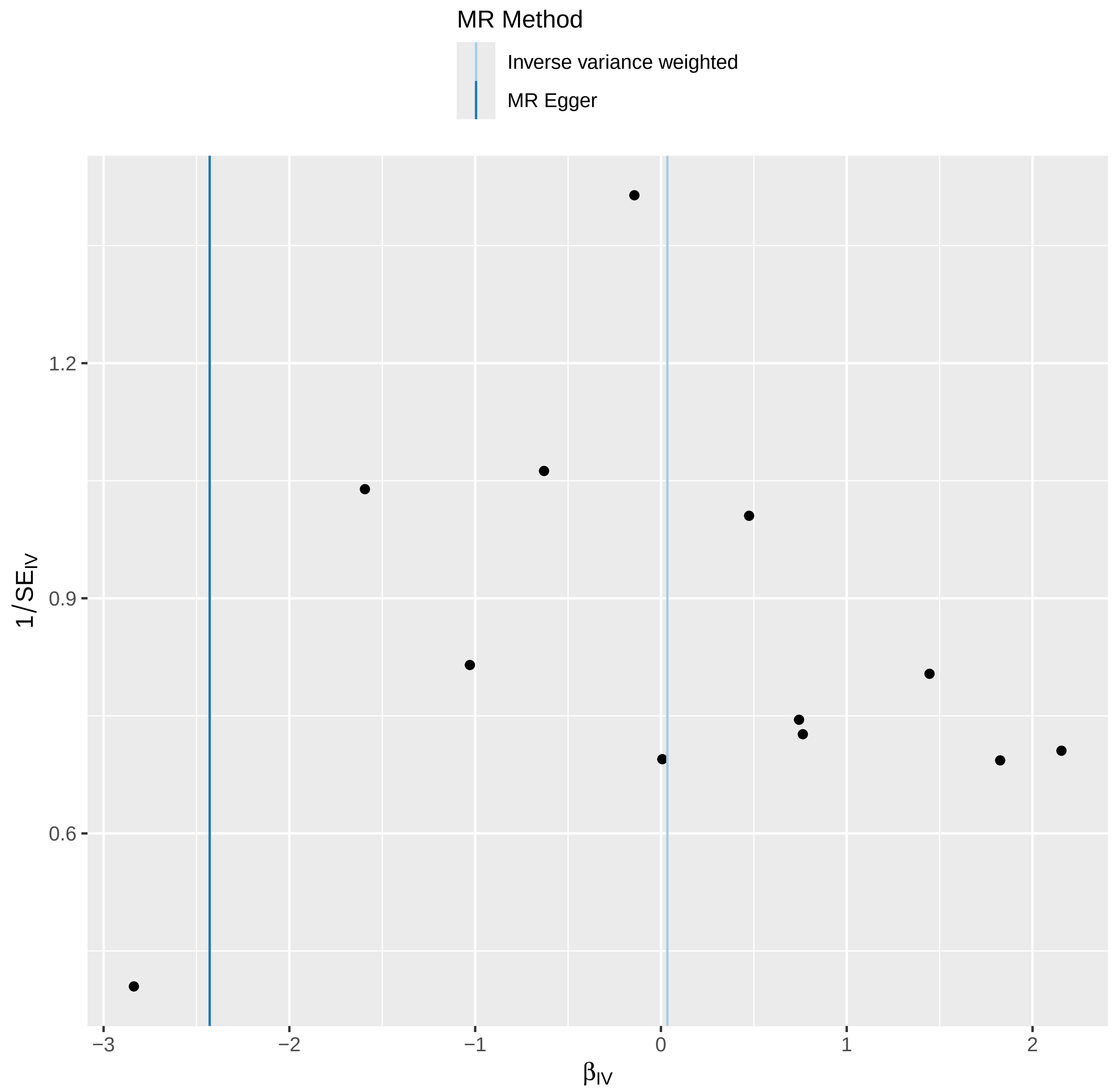
MR Method
Inverse variance weighted
MR Egger
1.2
0.9
0.6
1/SE_IV
−3
−2
−1
0
1
2
β_IV

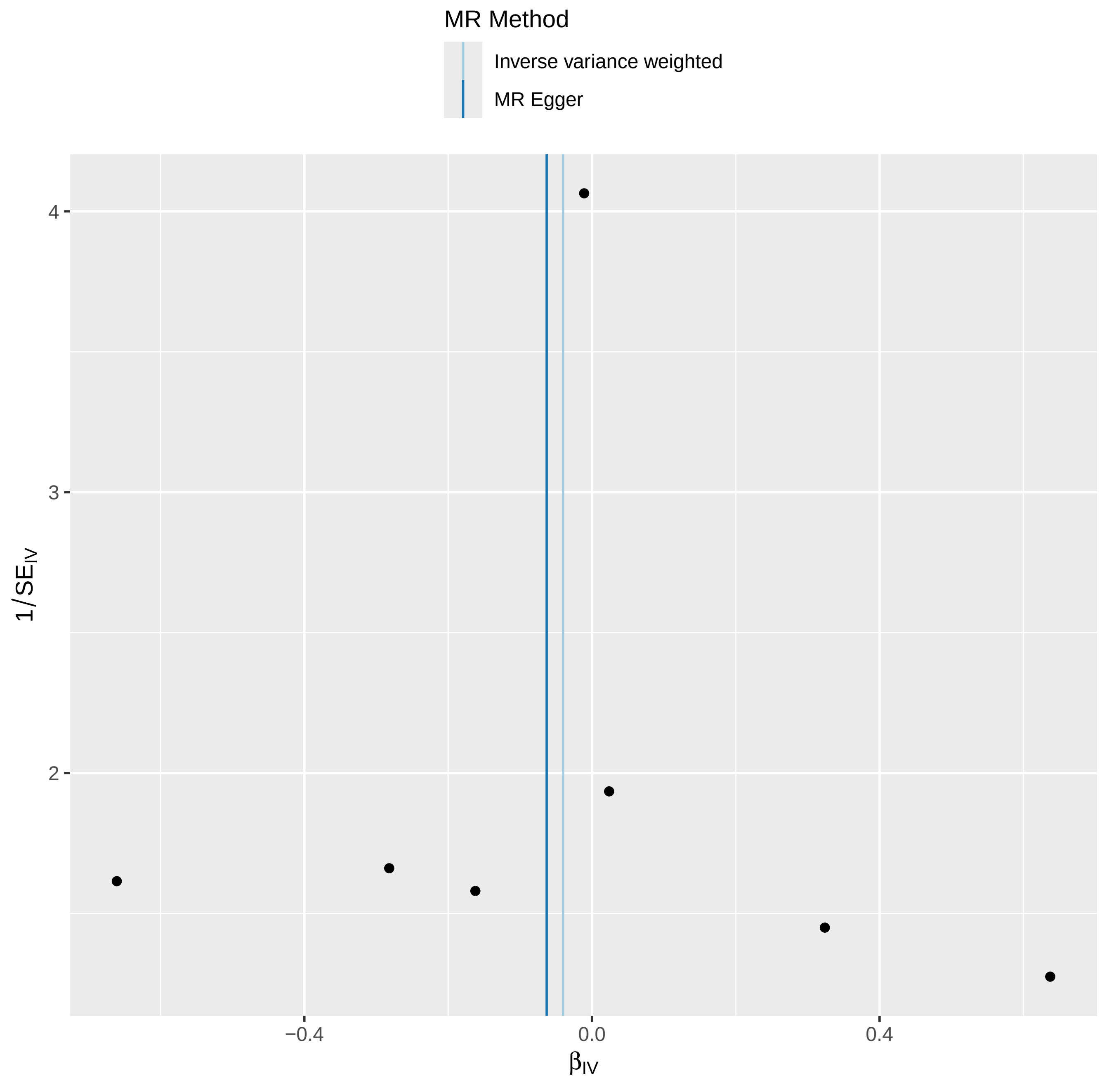
MR Method
Inverse variance weighted
MR Egger
4
3
2
1/SE_IV
−0.4
0.0
0.4
β_IV

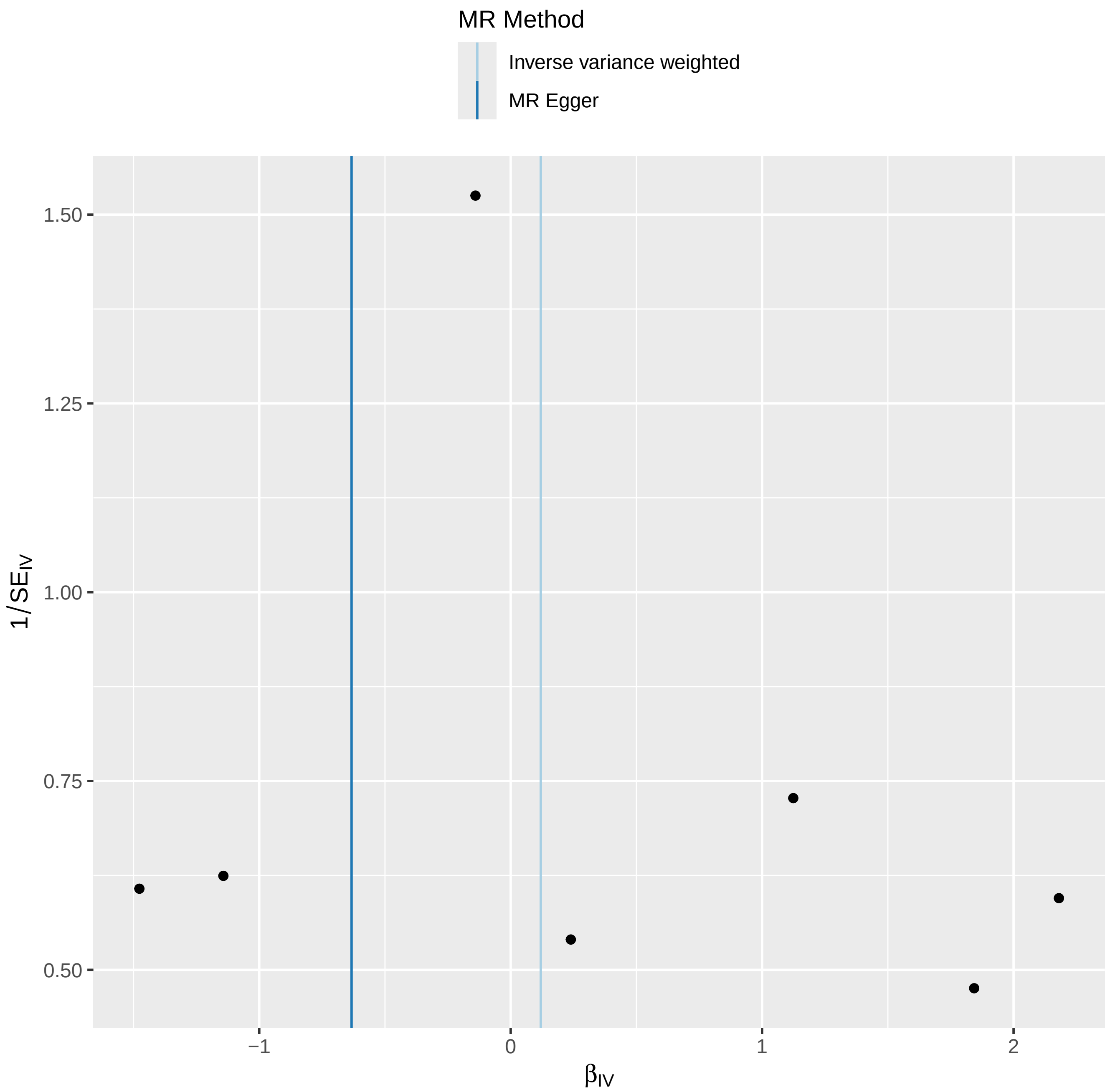

MR Method
Inverse variance weighted
MR Egger
1.50
1.25
1.00
0.75
0.50
1/SE_IV
−1
0
1
2
β_IV

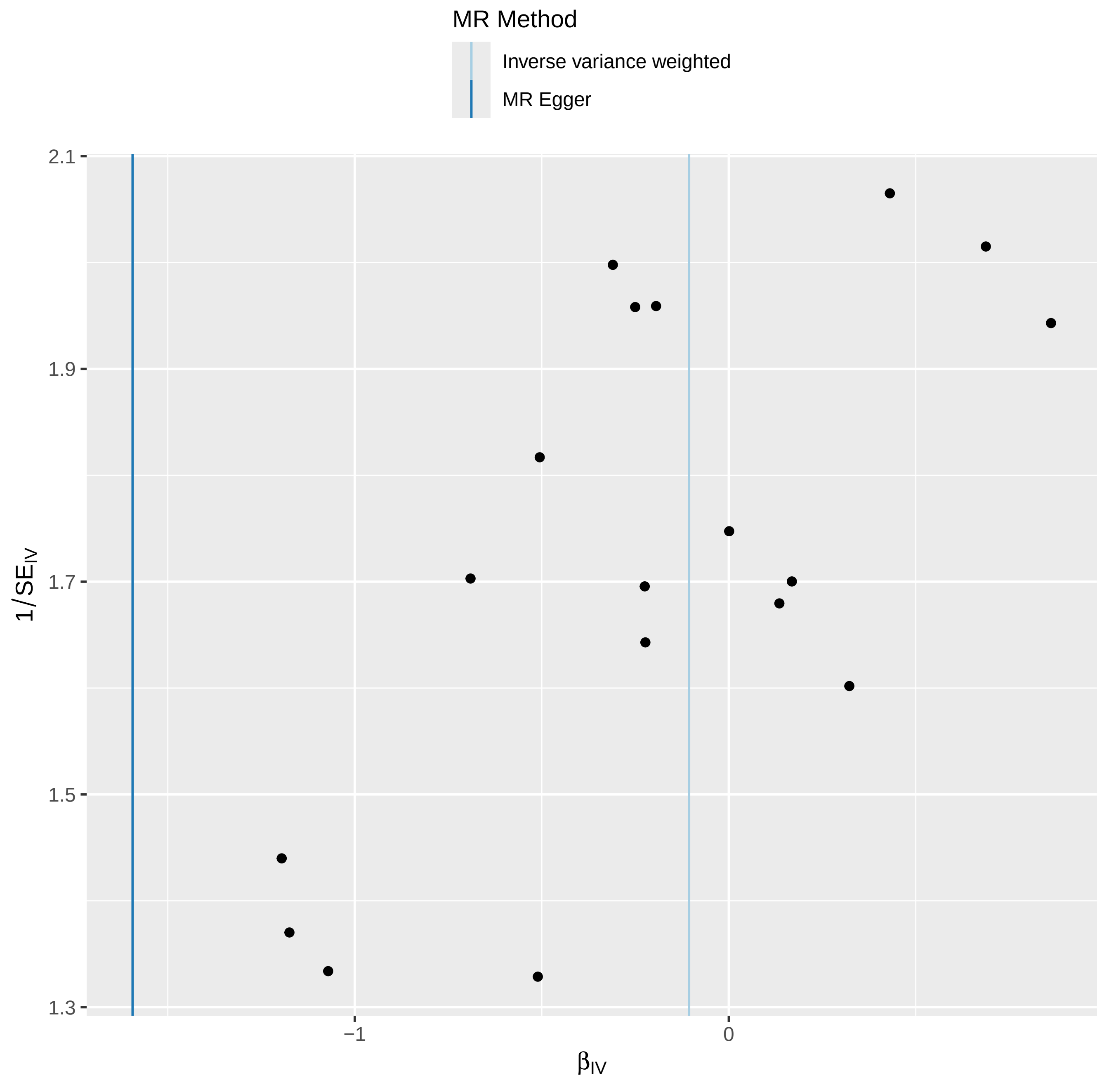
MR Method
Inverse variance weighted
MR Egger
2.1
1.9
1.7
1.5
1.3
1/SE_IV
−1
0
β_IV

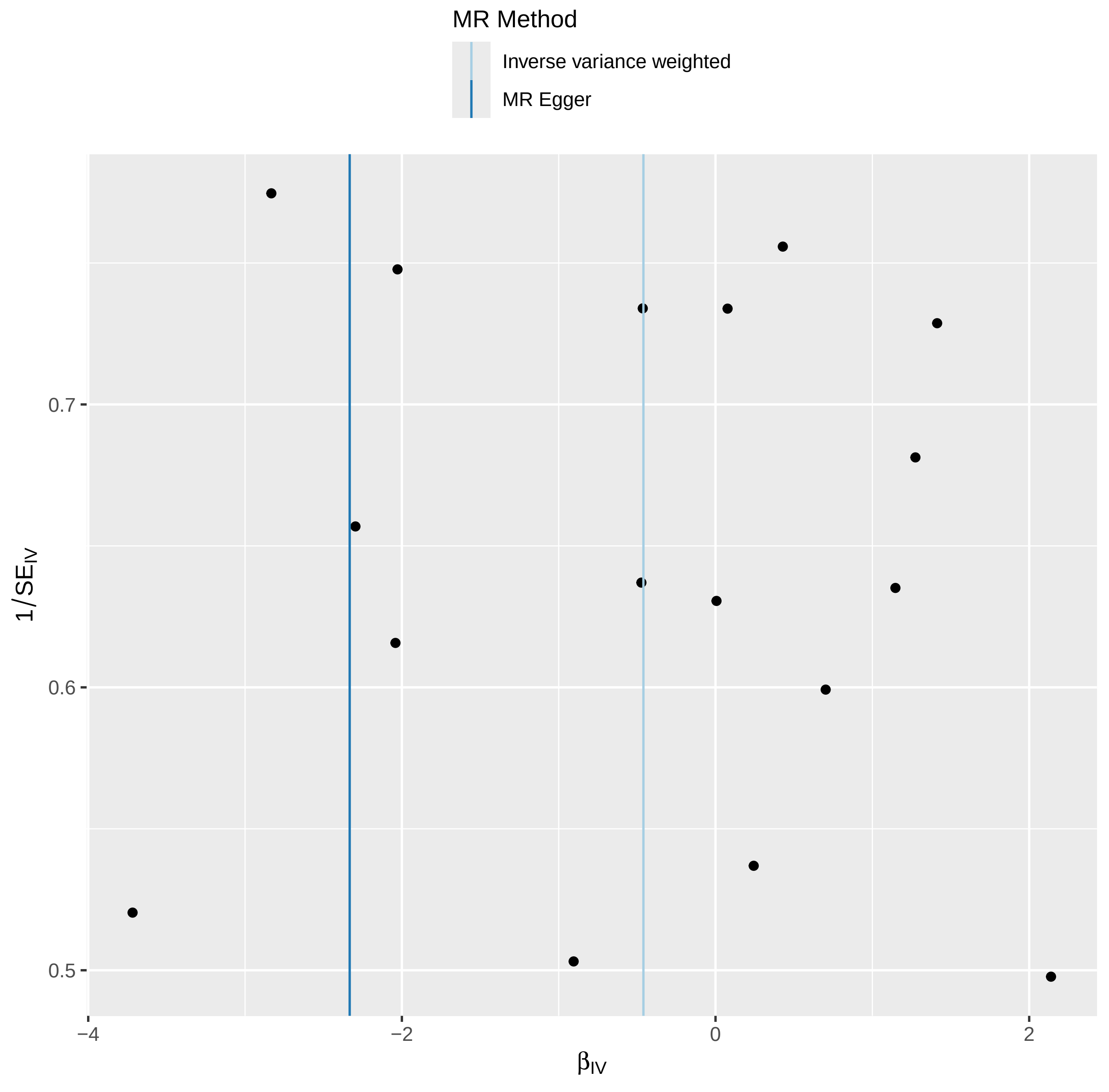
MR Method
Inverse variance weighted
MR Egger
0.7
0.6
0.5
$1/SE_{IV}$
−4
−2
0
2
β_{IV}

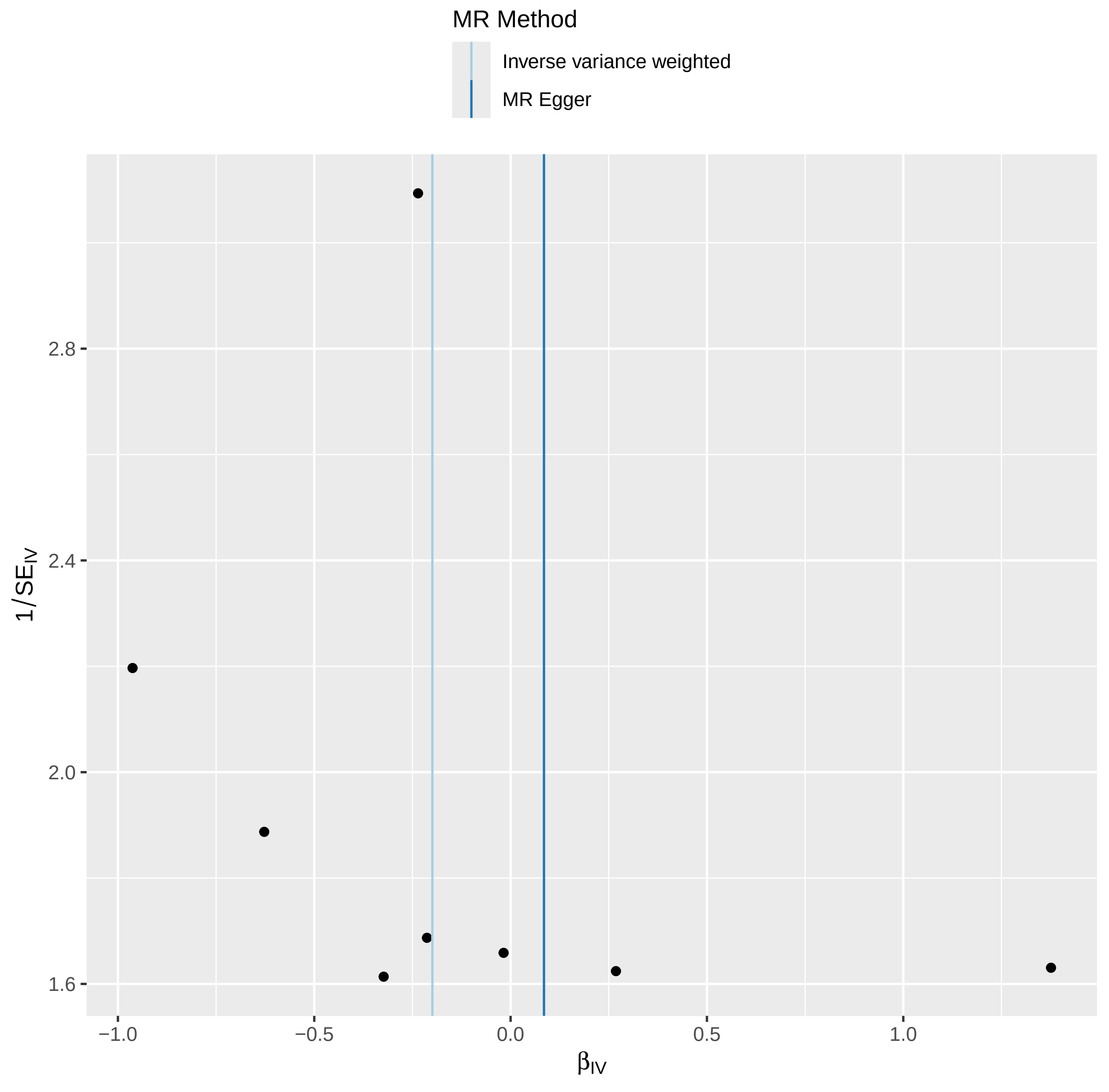
MR Method
Inverse variance weighted
MR Egger
2.8
2.4
2.0
1.6
1/SE_IV
−1.0
−0.5
0.0
0.5
1.0
β_IV

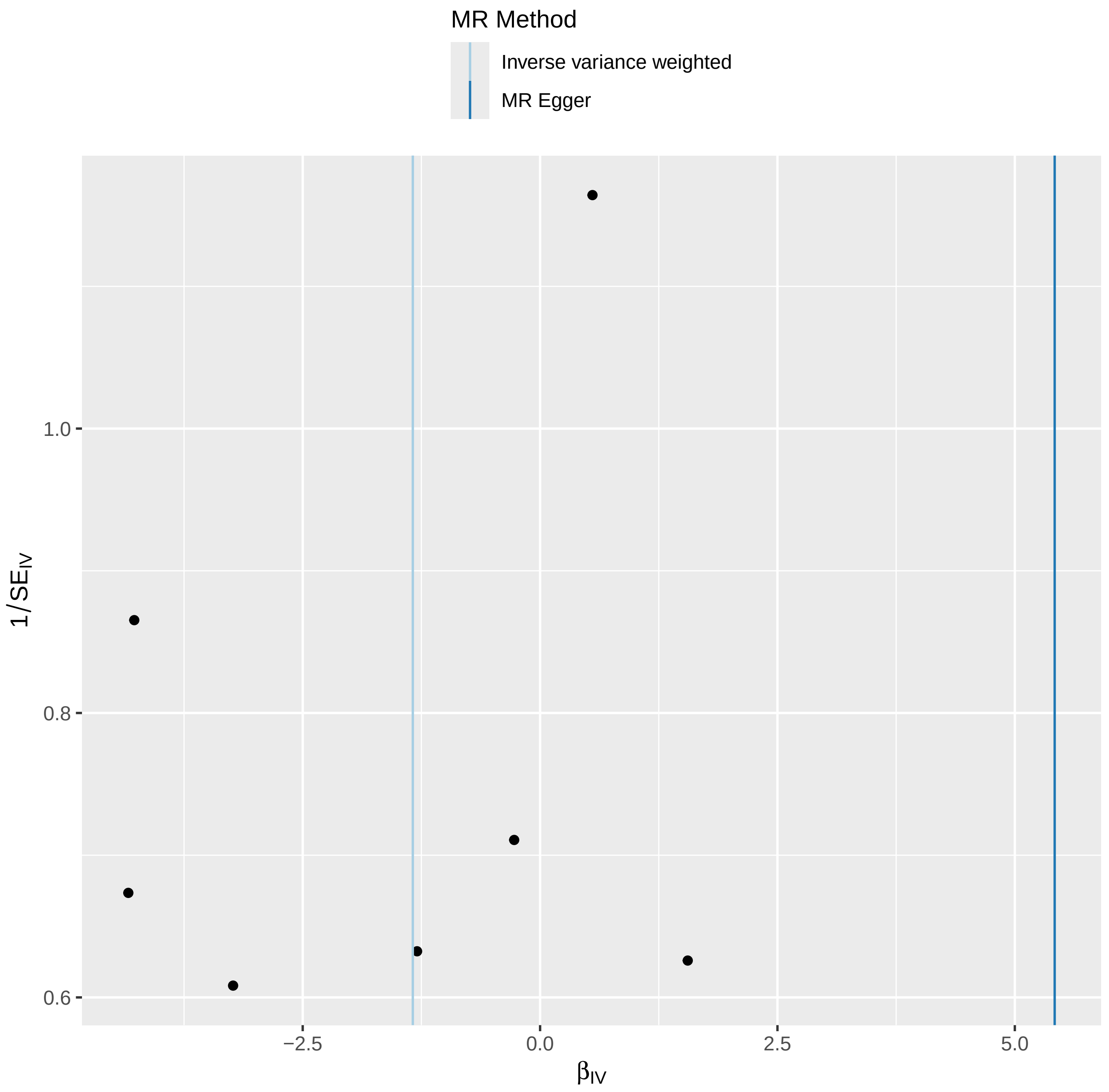
MR Method
Inverse variance weighted
MR Egger
1.0
0.8
0.6
1/SE_IV
−2.5
0.0
2.5
5.0
β_IV

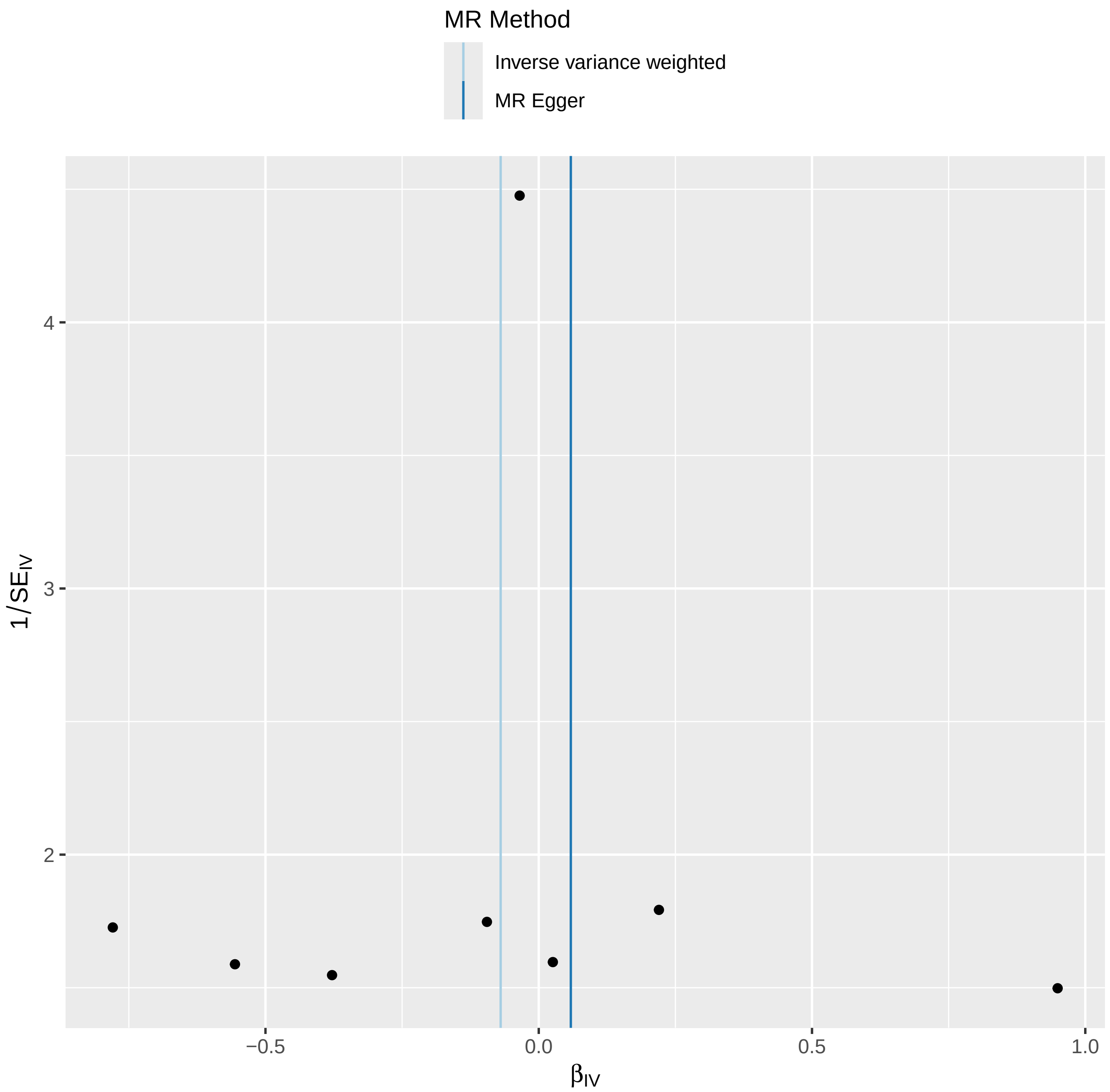

MR Method
Inverse variance weighted
MR Egger
1/SE_IV
4
3
2
−0.5
0.0
0.5
1.0
β_IV

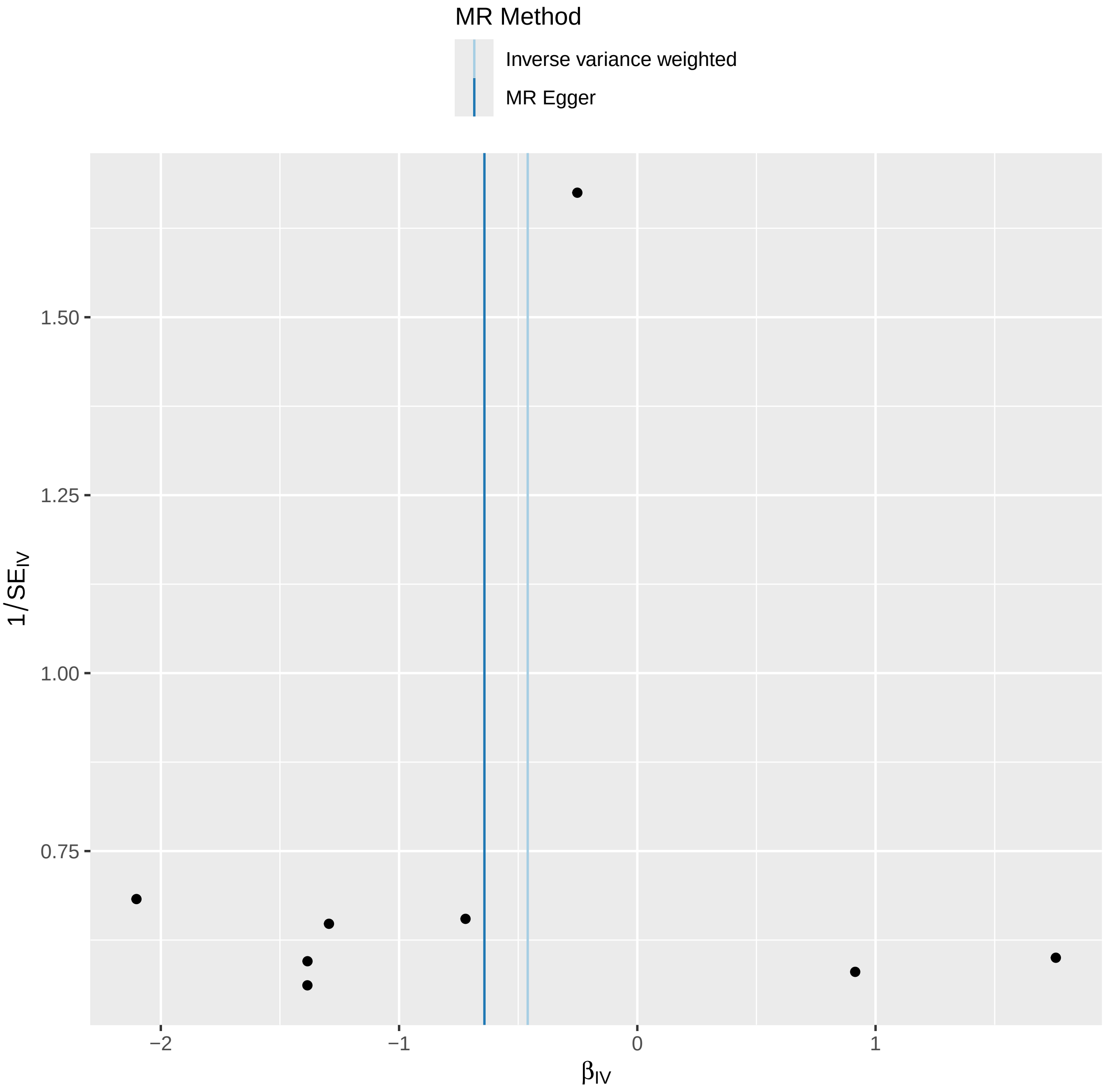

MR Method
Inverse variance weighted
MR Egger
1.50
1.25
1.00
0.75
1/SE_IV
−2
−1
0
1
β_IV

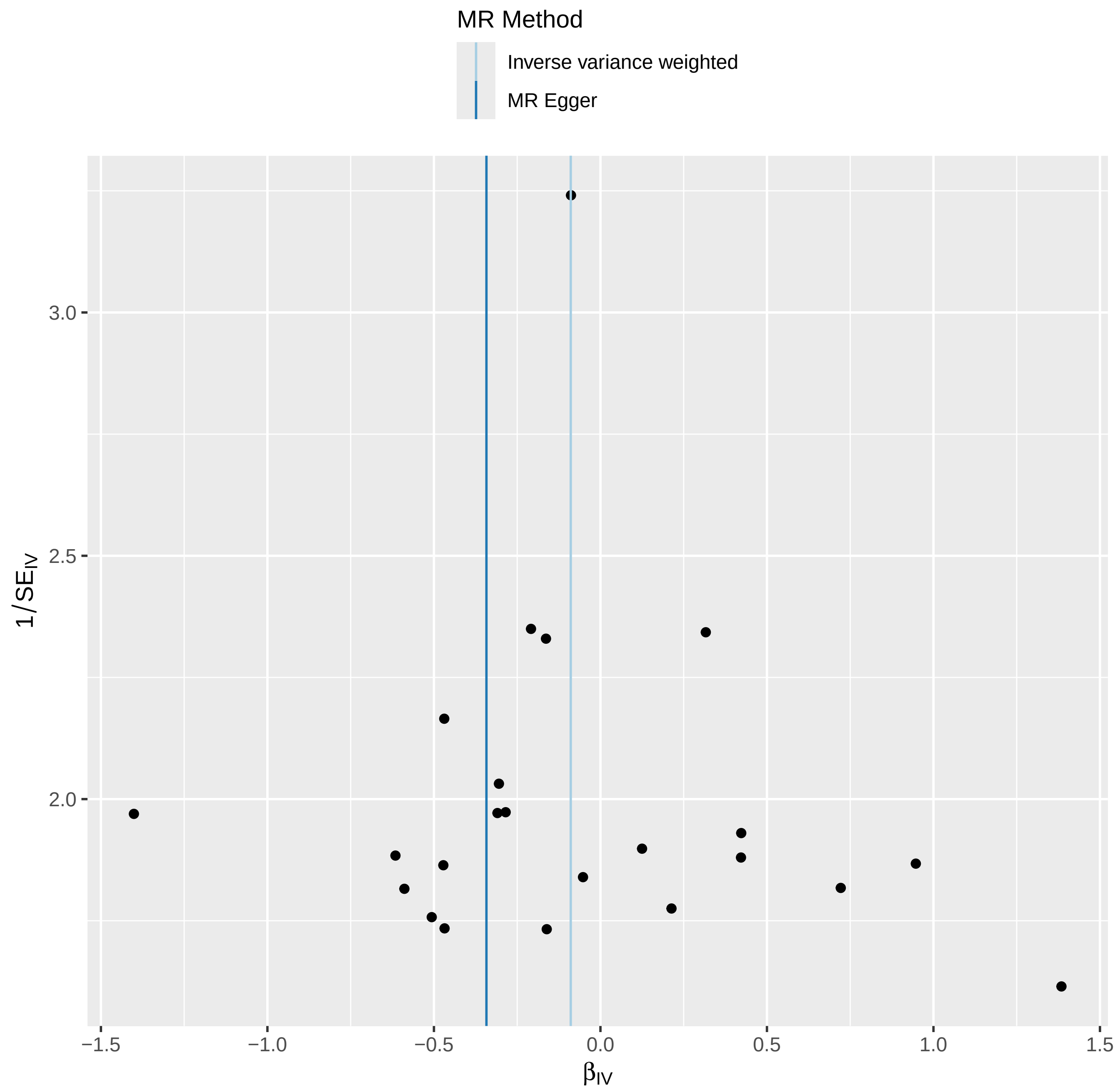

MR Method
Inverse variance weighted
MR Egger
3.0
2.5
2.0
1/SE$_{IV}$
−1.5
−1.0
−0.5
0.0
0.5
1.0
1.5
β$_{IV}$

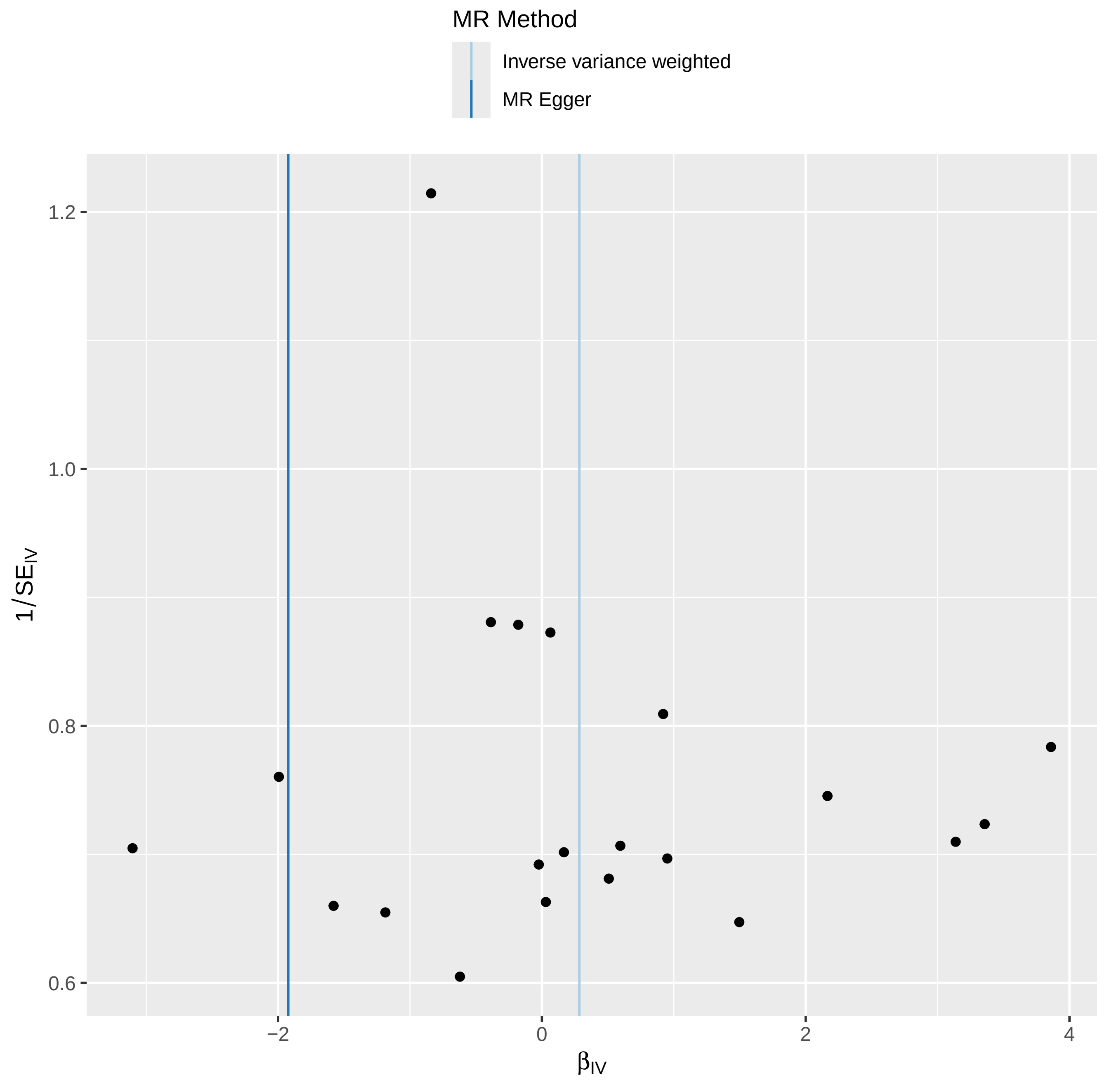

MR Method
Inverse variance weighted
MR Egger
1.2
1.0
0.8
0.6
−2
0
2
4
$1/SE_{IV}$
β_{IV}

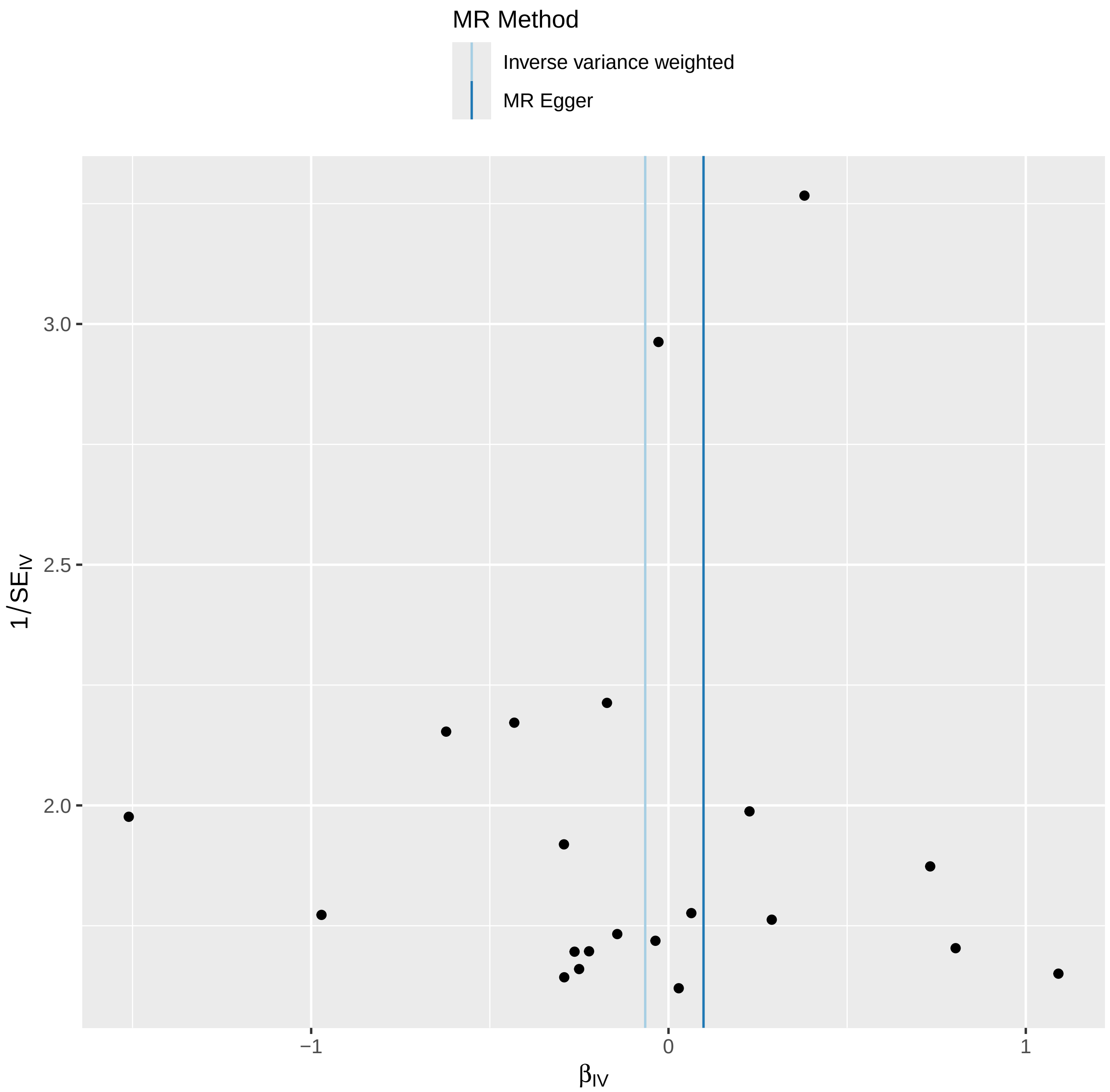

MR Method
Inverse variance weighted
MR Egger
3.0
2.5
2.0
1/SE_IV
−1
0
1
β_IV

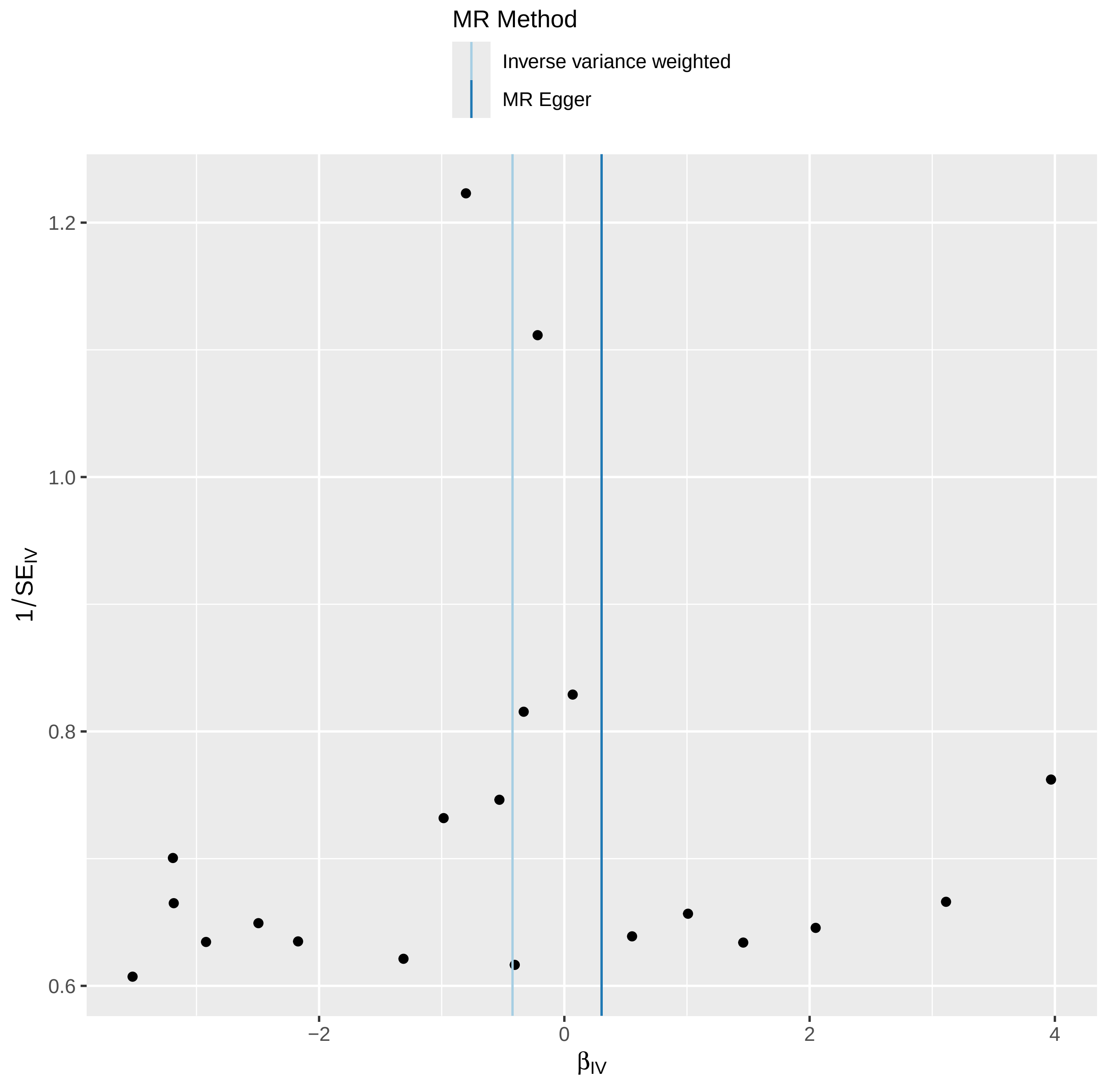
MR Method
Inverse variance weighted
MR Egger
1.2
1.0
0.8
0.6
−2
0
2
4
$1/SE_{IV}$
β_{IV}

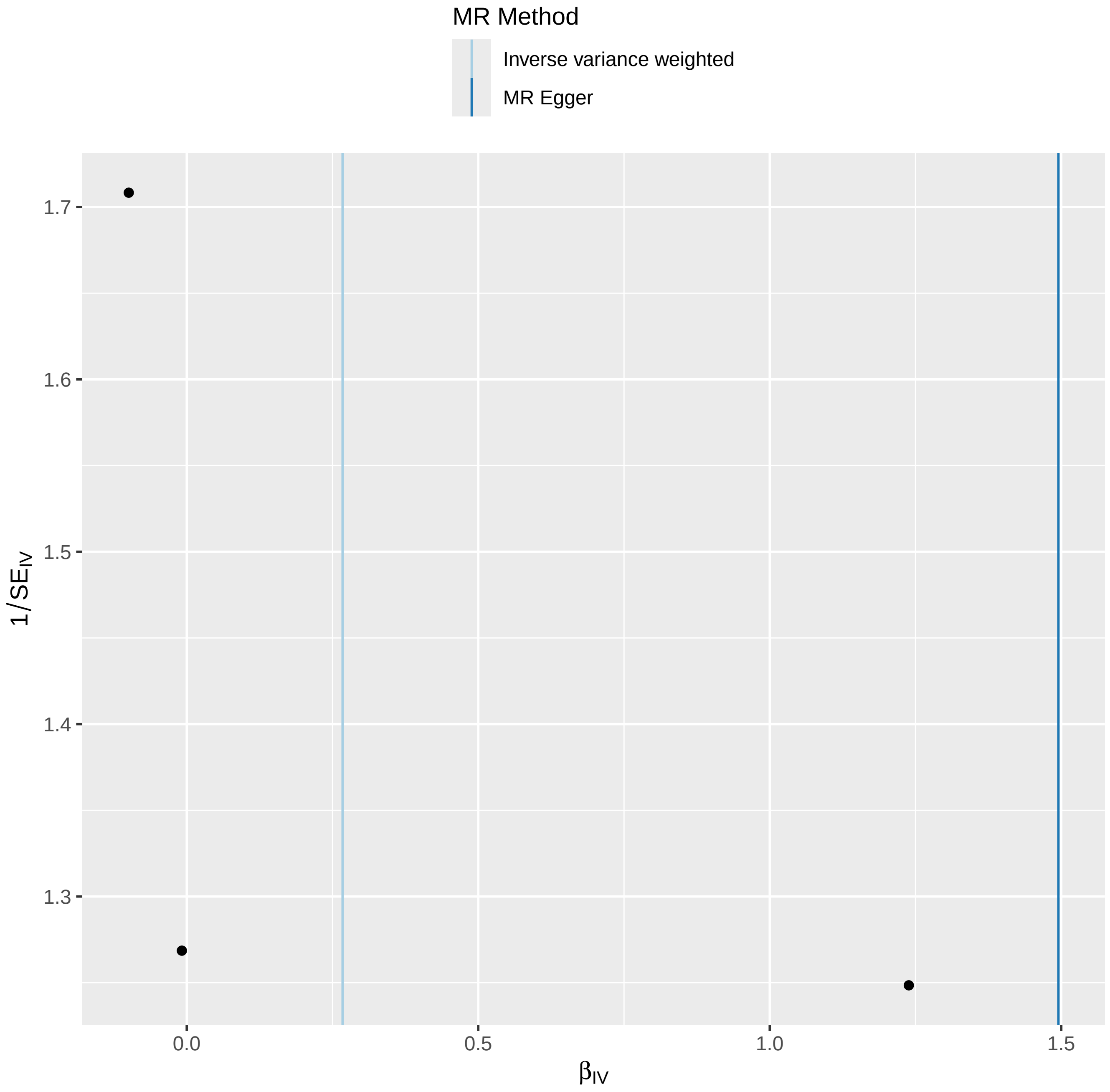
MR Method
Inverse variance weighted
MR Egger
1.7
1.6
1.5
1.4
1.3
0.0
0.5
1.0
1.5
1/SE_IV
β_IV

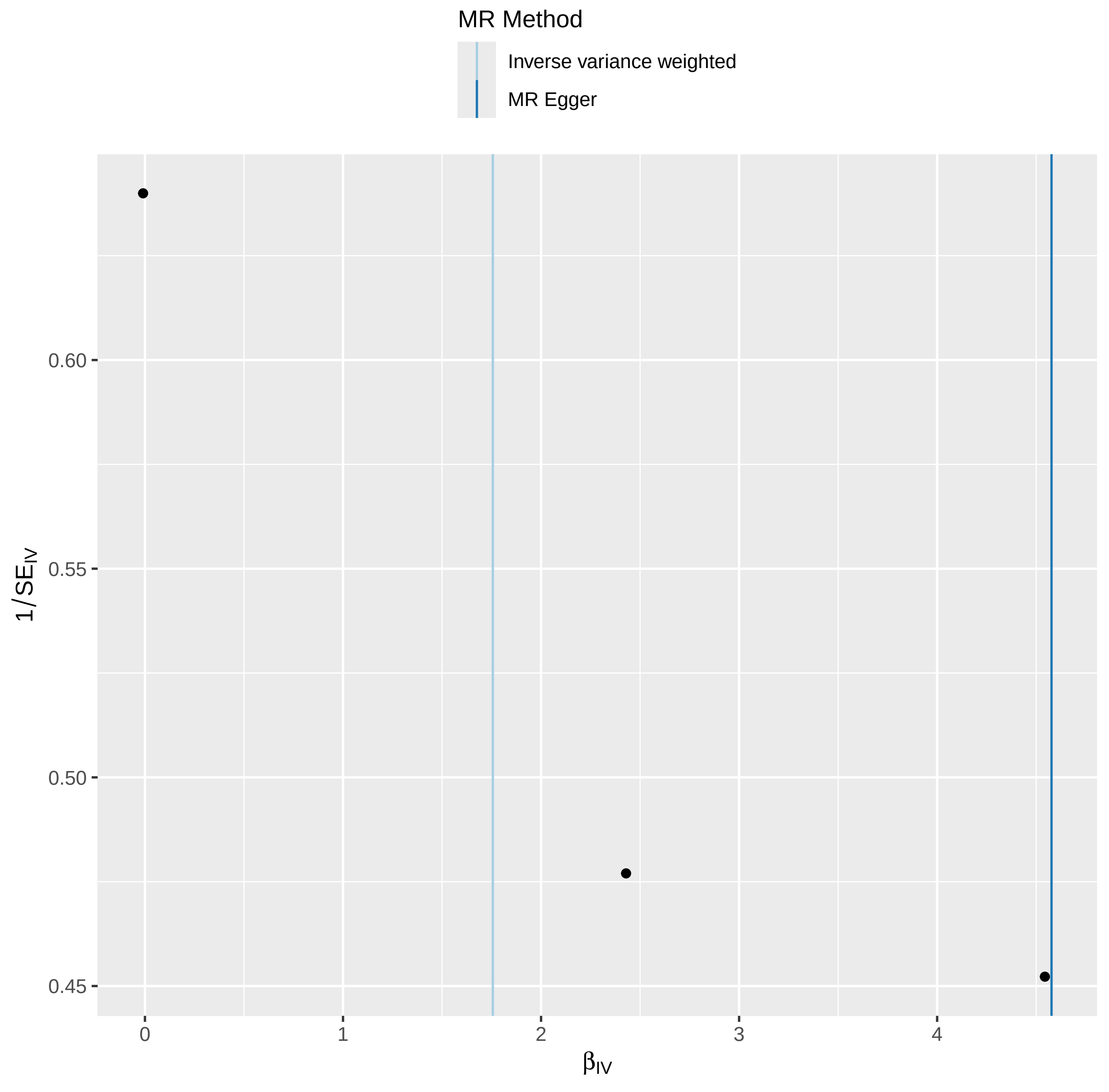
MR Method
Inverse variance weighted
MR Egger
0.60
0.55
0.50
0.45
$1/SE_{IV}$
0
1
2
3
4
β_{IV}

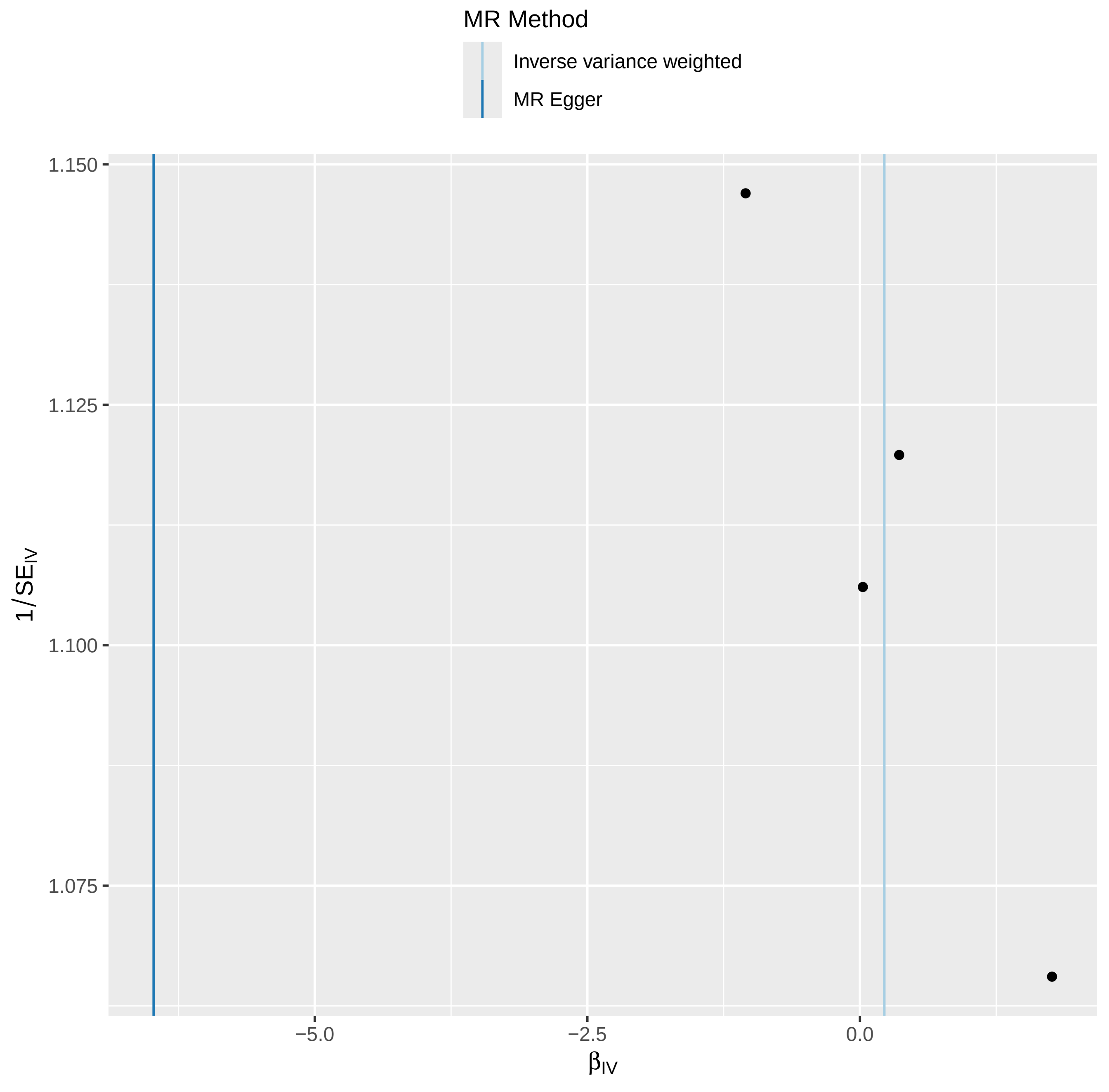
MR Method
Inverse variance weighted
MR Egger
1.150
1.125
1.100
1.075
1/SE_IV
−5.0
−2.5
0.0
β_IV

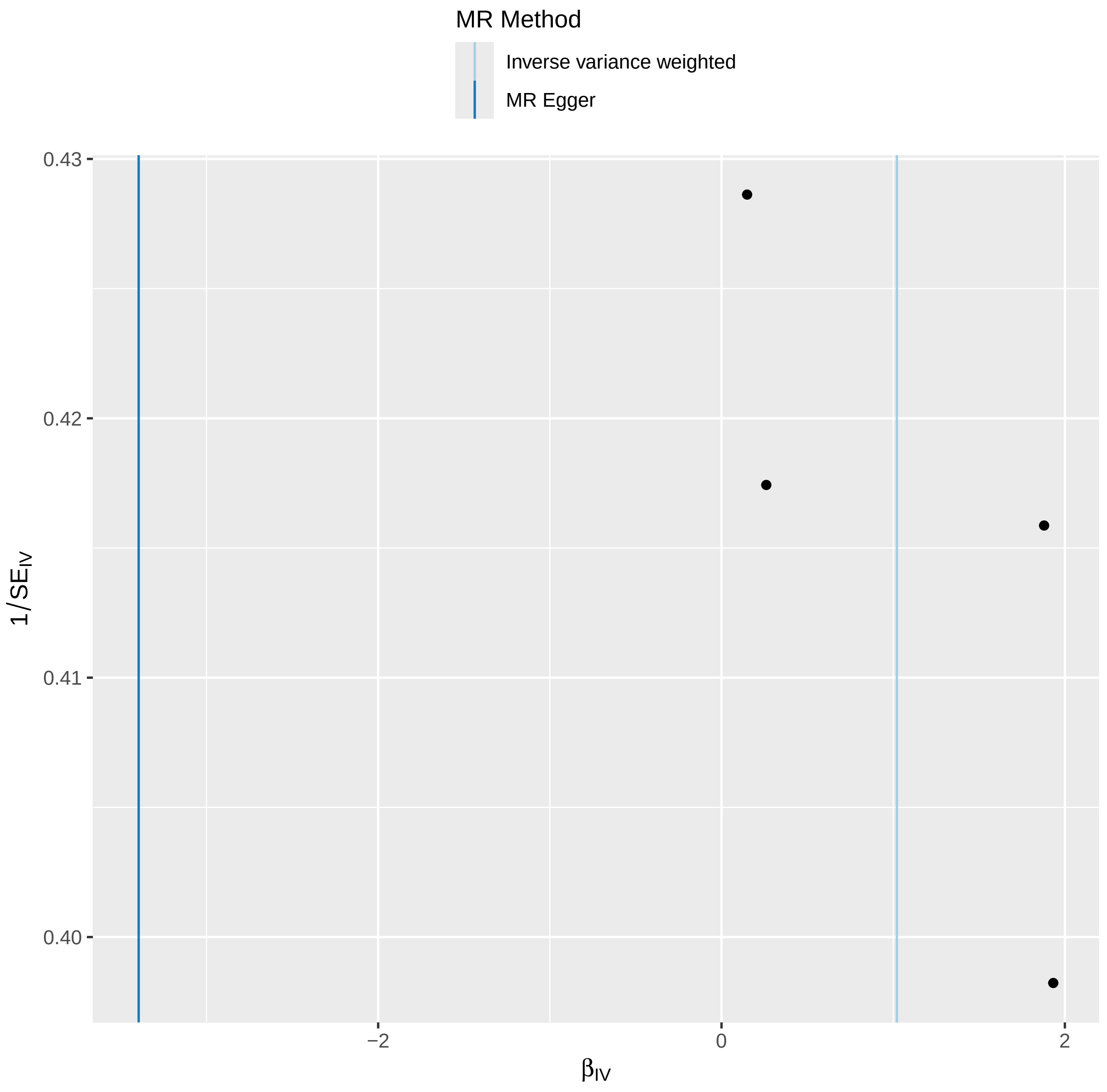

MR Method
Inverse variance weighted
MR Egger
0.43
0.42
0.41
0.40
1/SE_IV
−2
0
2
β_IV

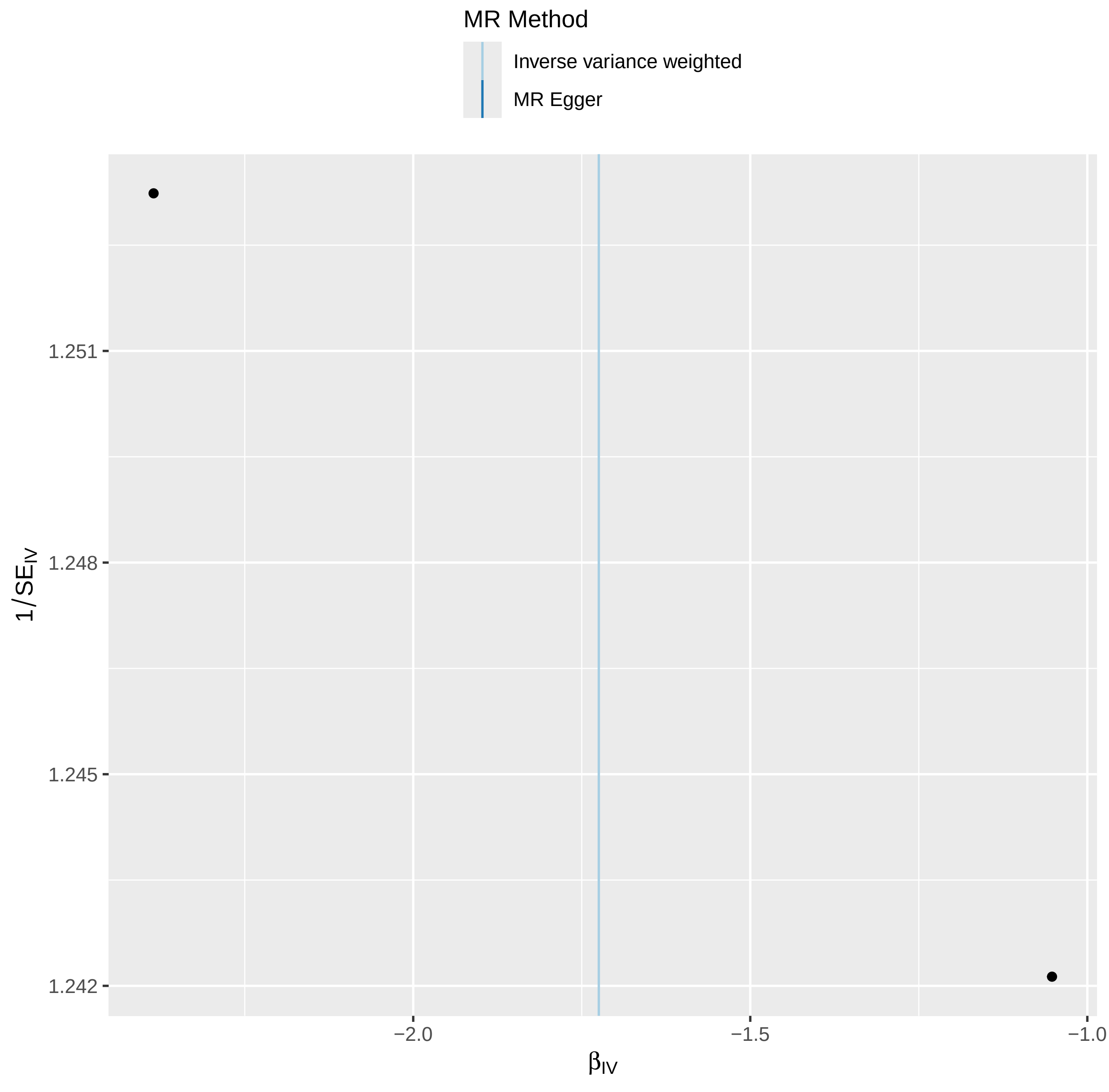

MR Method
Inverse variance weighted
MR Egger
1.251
1.248
1.245
1.242
1/SE_IV
−2.0
−1.5
−1.0
β_IV

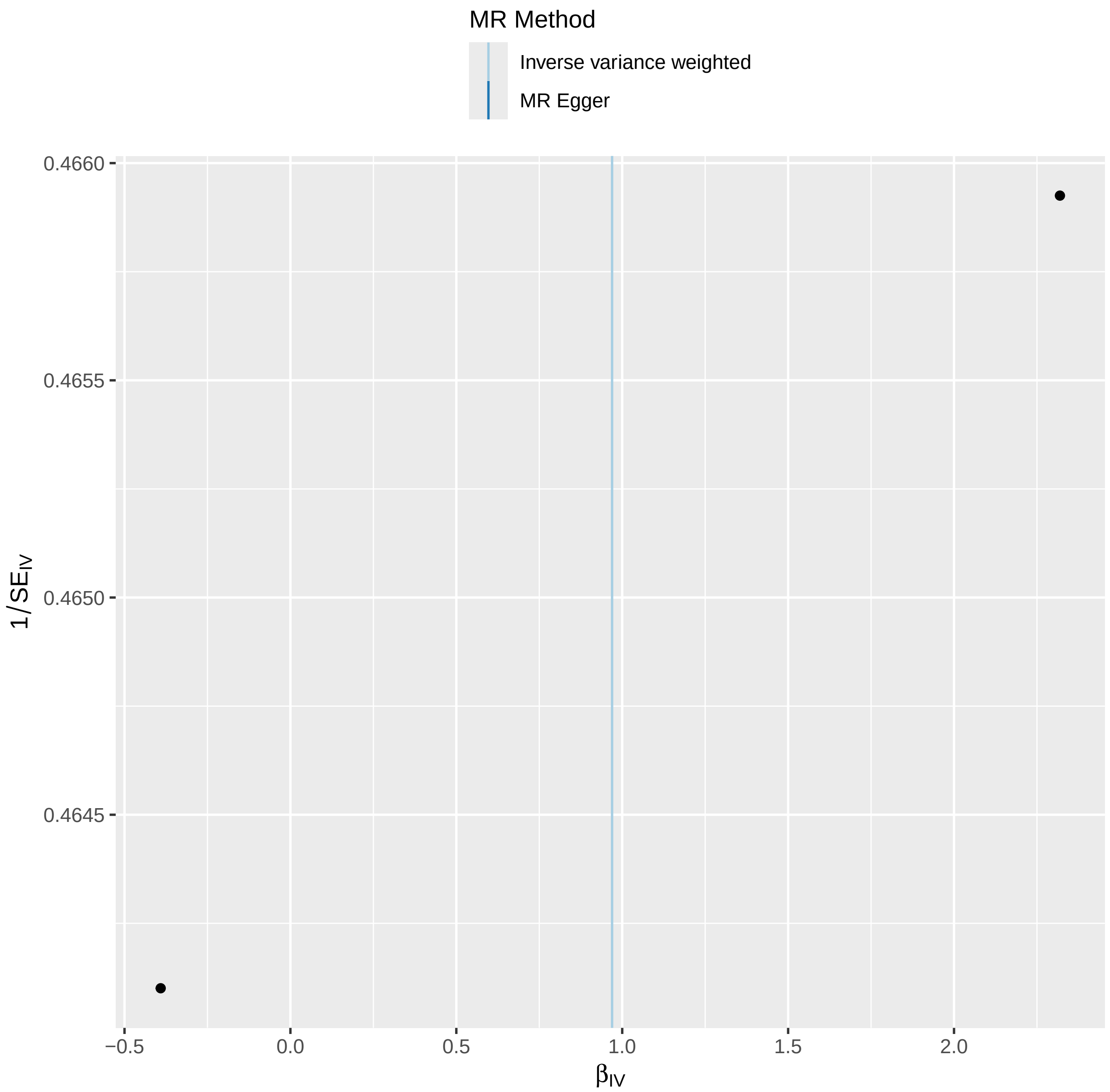
MR Method
Inverse variance weighted
MR Egger
0.4660
0.4655
0.4650
0.4645
1/SE_IV
−0.5
0.0
0.5
1.0
1.5
2.0
β_IV

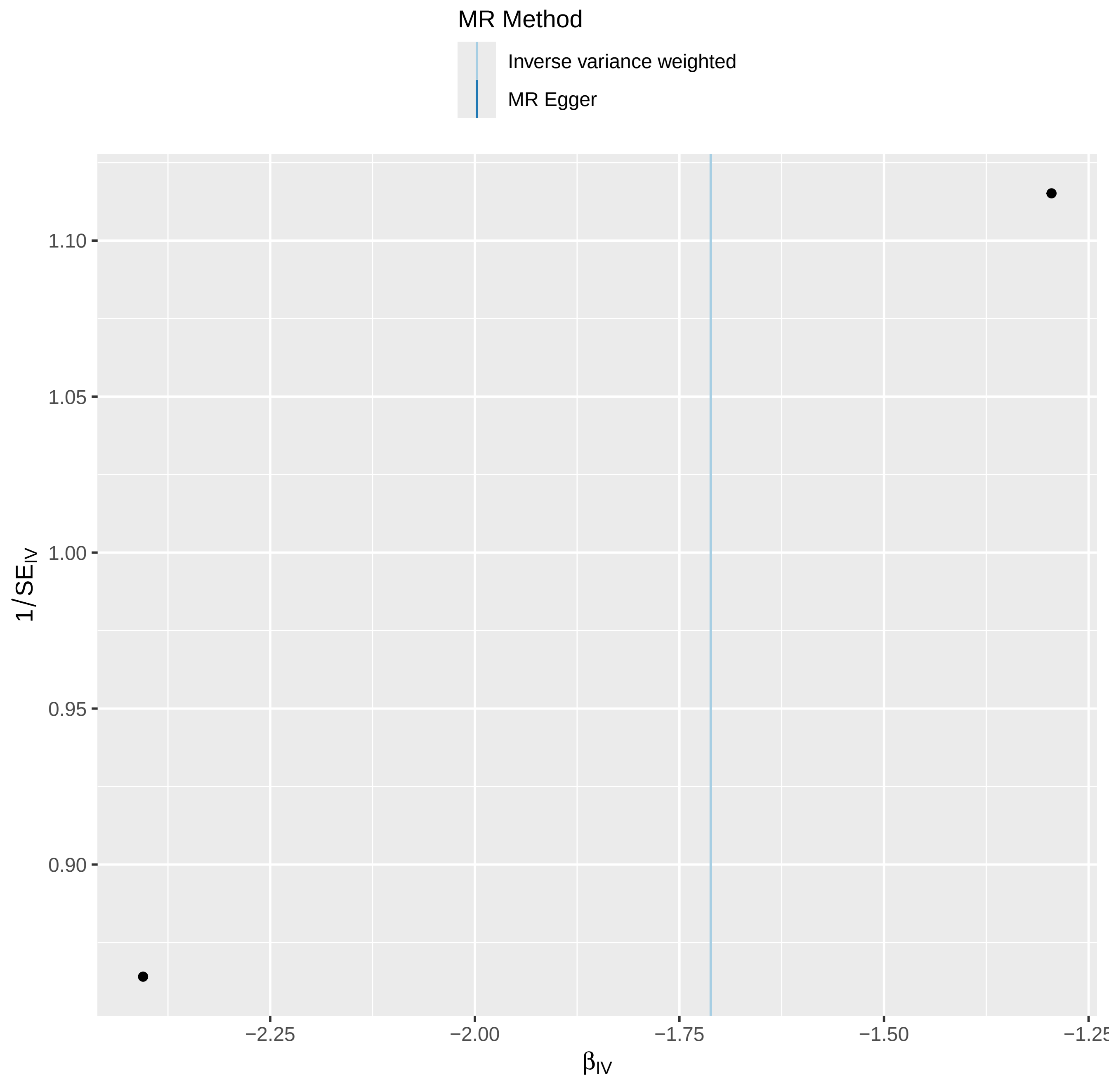
MR Method
Inverse variance weighted
MR Egger
1.10
1.05
1.00
0.95
0.90
1/SE_IV
−2.25
−2.00
−1.75
−1.50
−1.25
β_IV

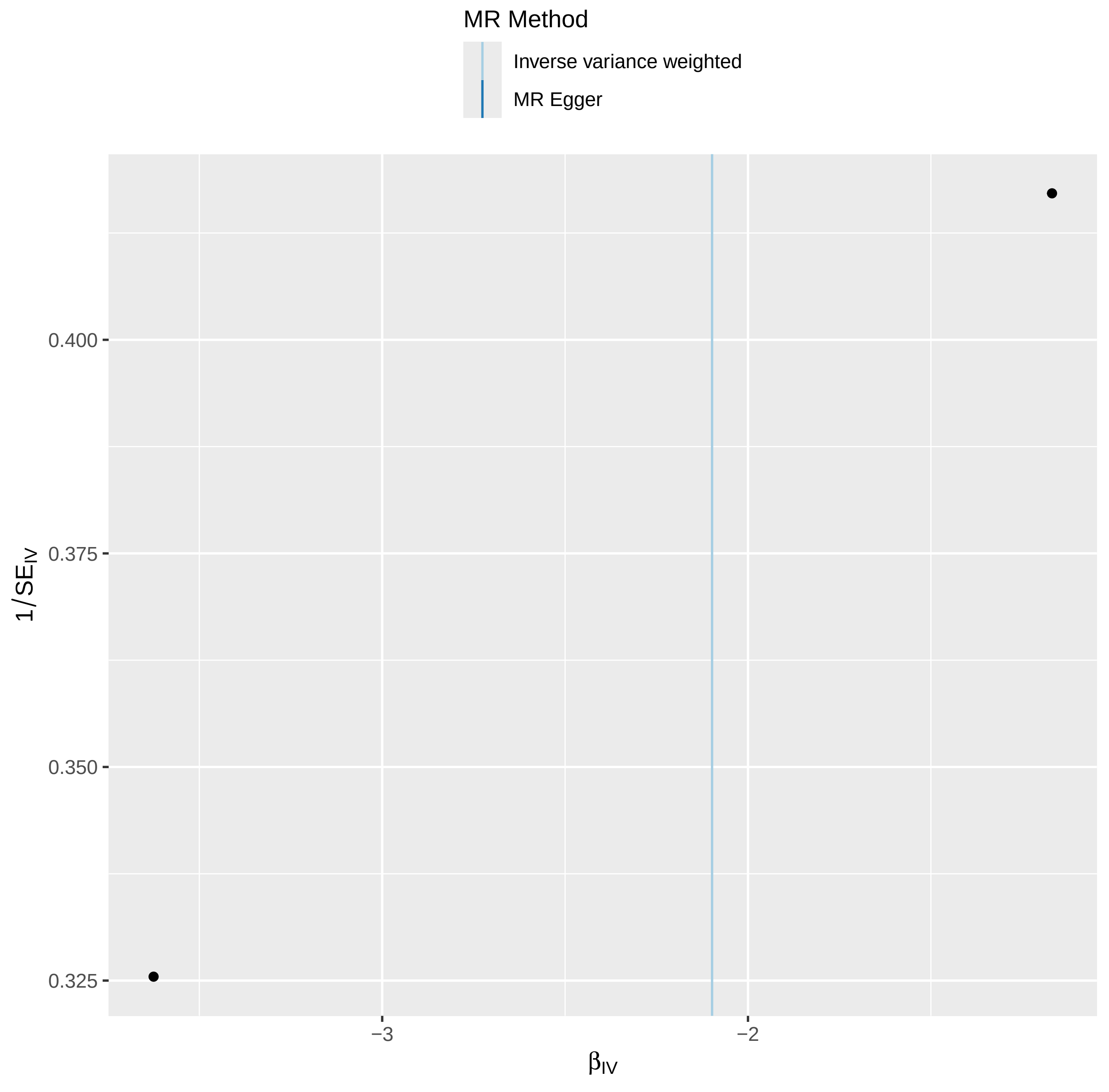
MR Method
Inverse variance weighted
MR Egger
0.400
0.375
0.350
0.325
1/SE$_{IV}$
−3
−2
β$_{IV}$

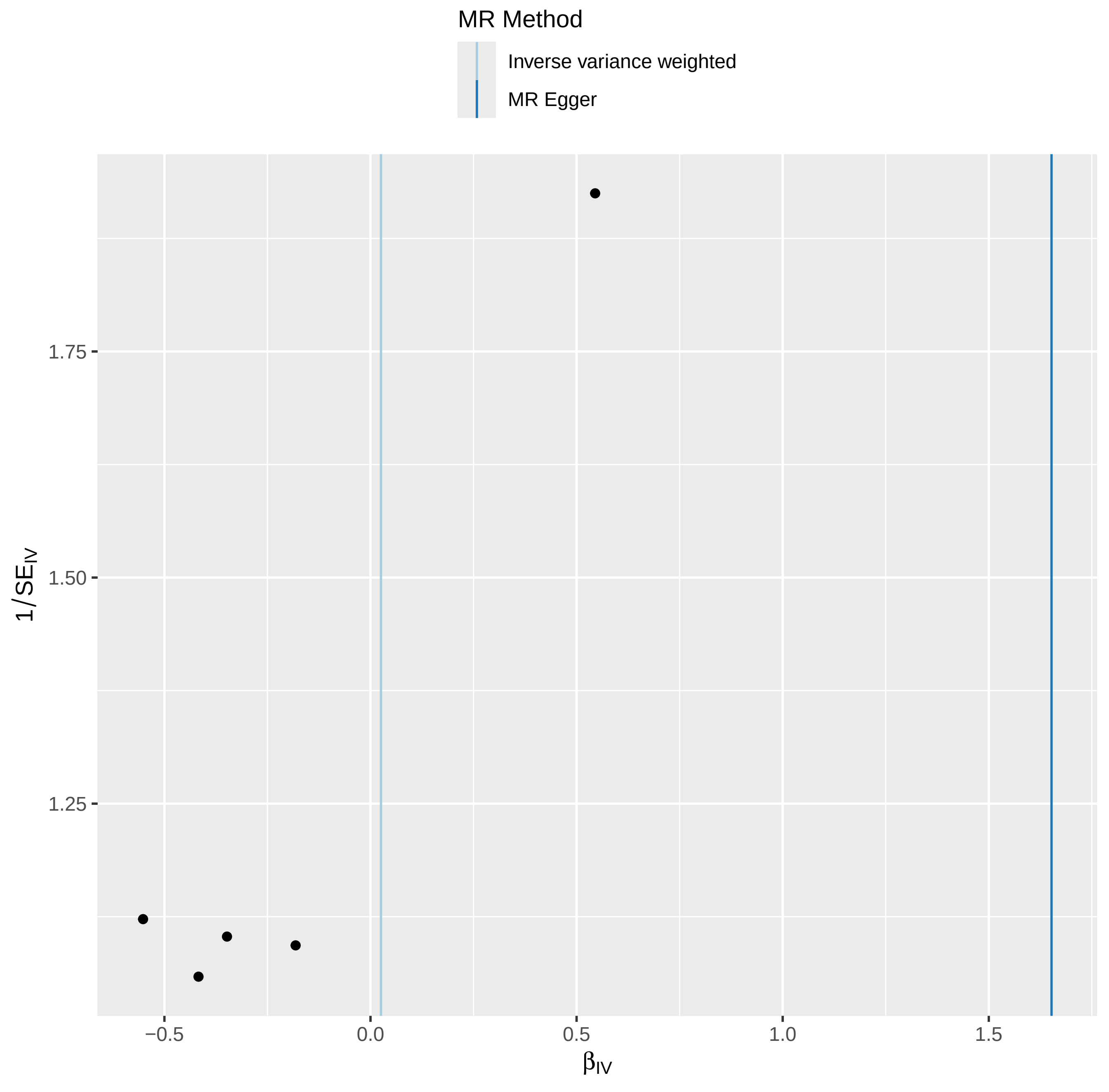

MR Method
Inverse variance weighted
MR Egger
1.75
1.50
1.25
1/SE_IV
−0.5
0.0
0.5
1.0
1.5
β_IV

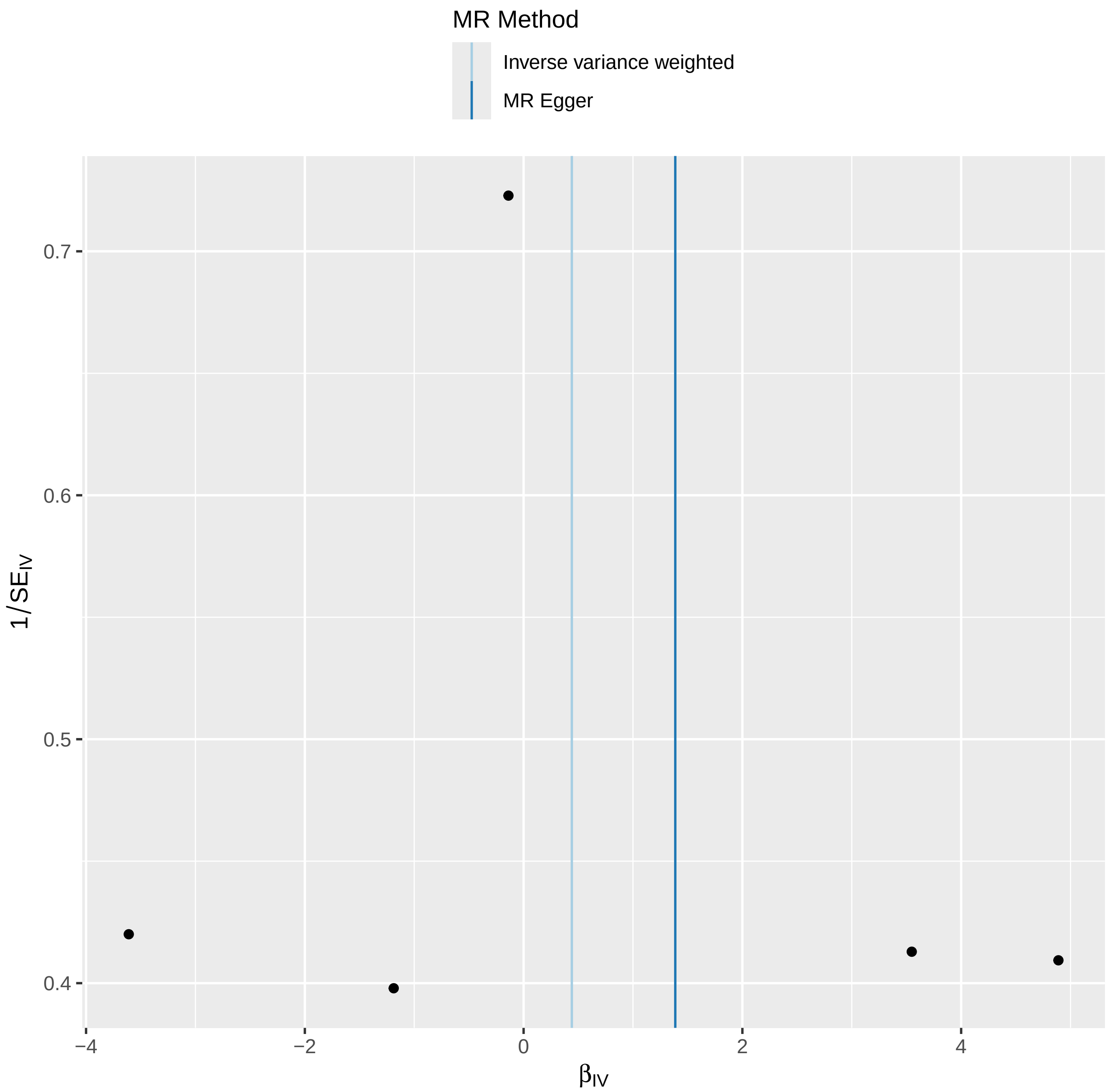
MR Method
Inverse variance weighted
MR Egger
0.7
0.6
0.5
0.4
1/SE$_{IV}$
−4
−2
0
2
4
β$_{IV}$

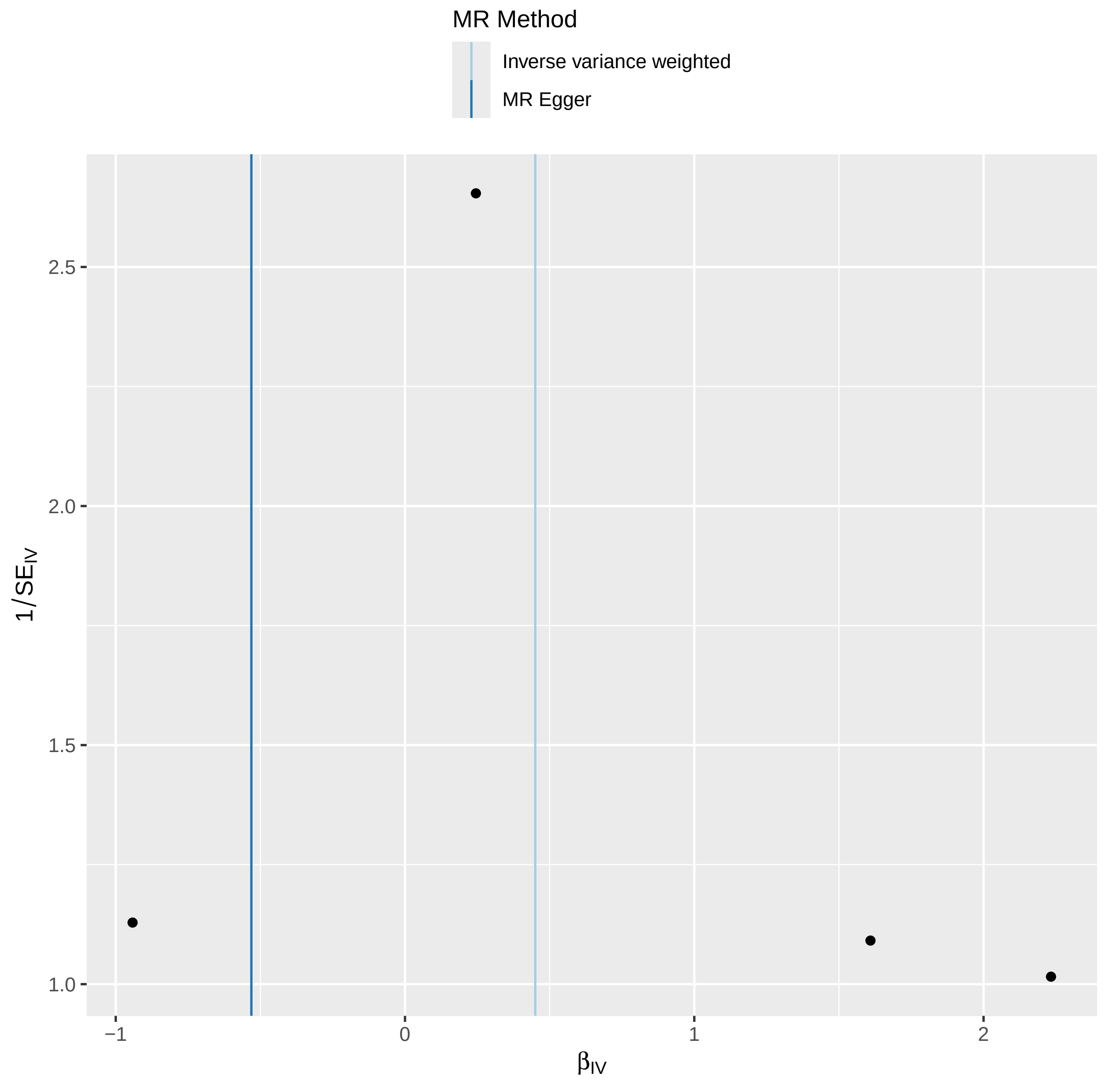
MR Method
Inverse variance weighted
MR Egger
2.5
2.0
1.5
1.0
1/SE_IV
−1
0
1
2
β_IV

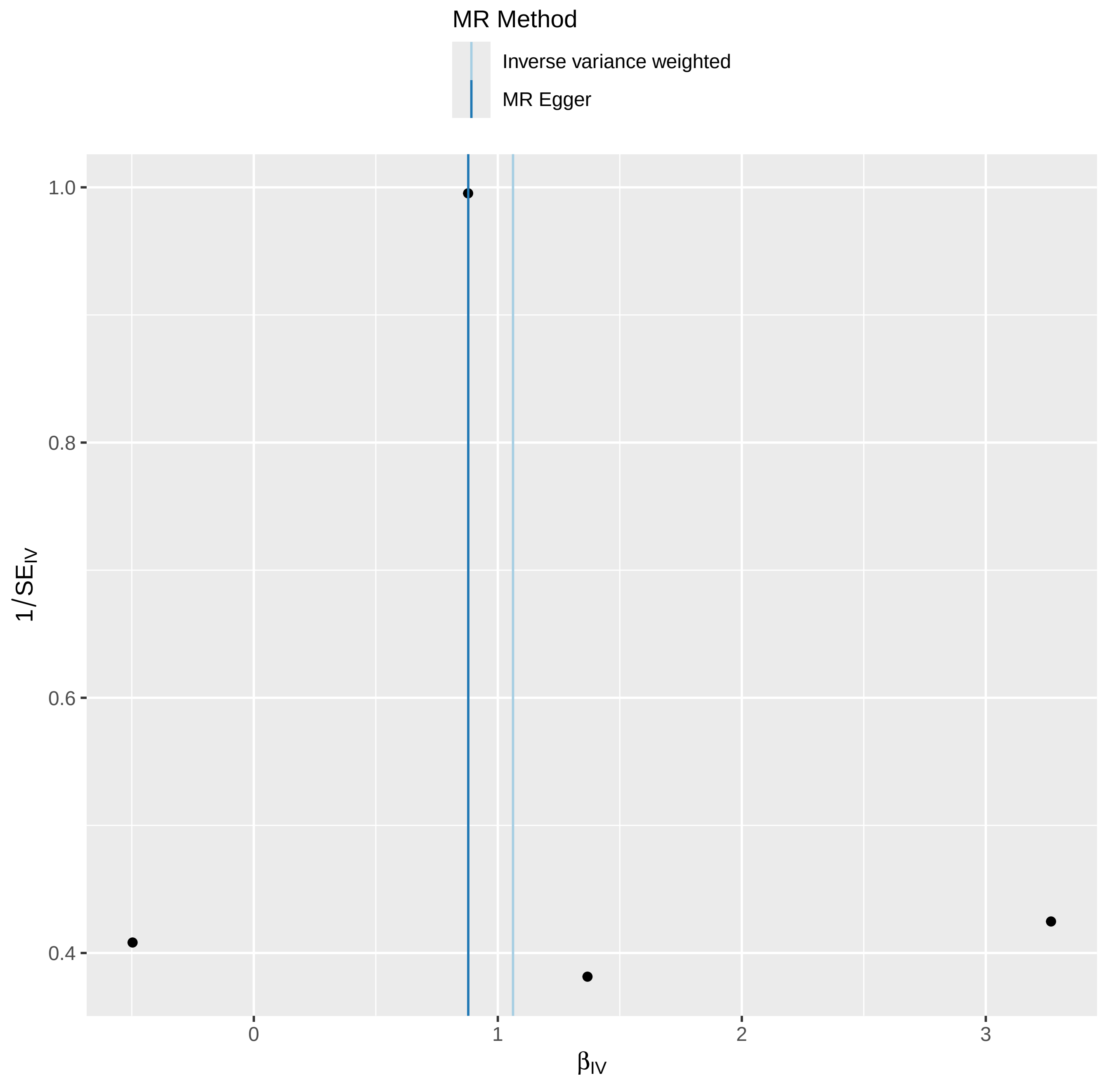
MR Method
Inverse variance weighted
MR Egger
1.0
0.8
0.6
0.4
1/SE_IV
0
1
2
3
β_IV

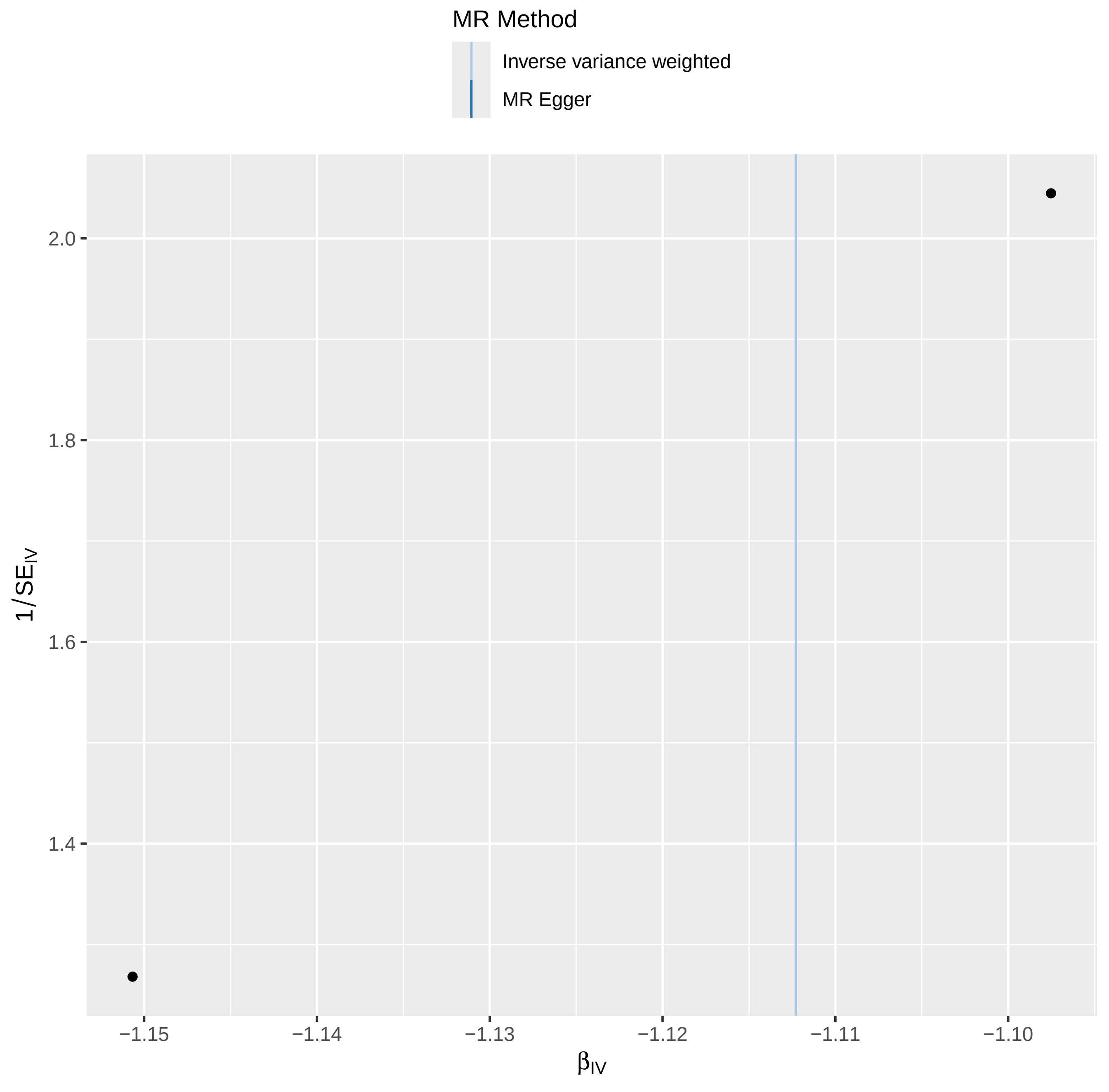
MR Method
Inverse variance weighted
MR Egger
2.0
1.8
1.6
1.4
1/SE_IV
−1.15
−1.14
−1.13
−1.12
−1.11
−1.10
β_IV

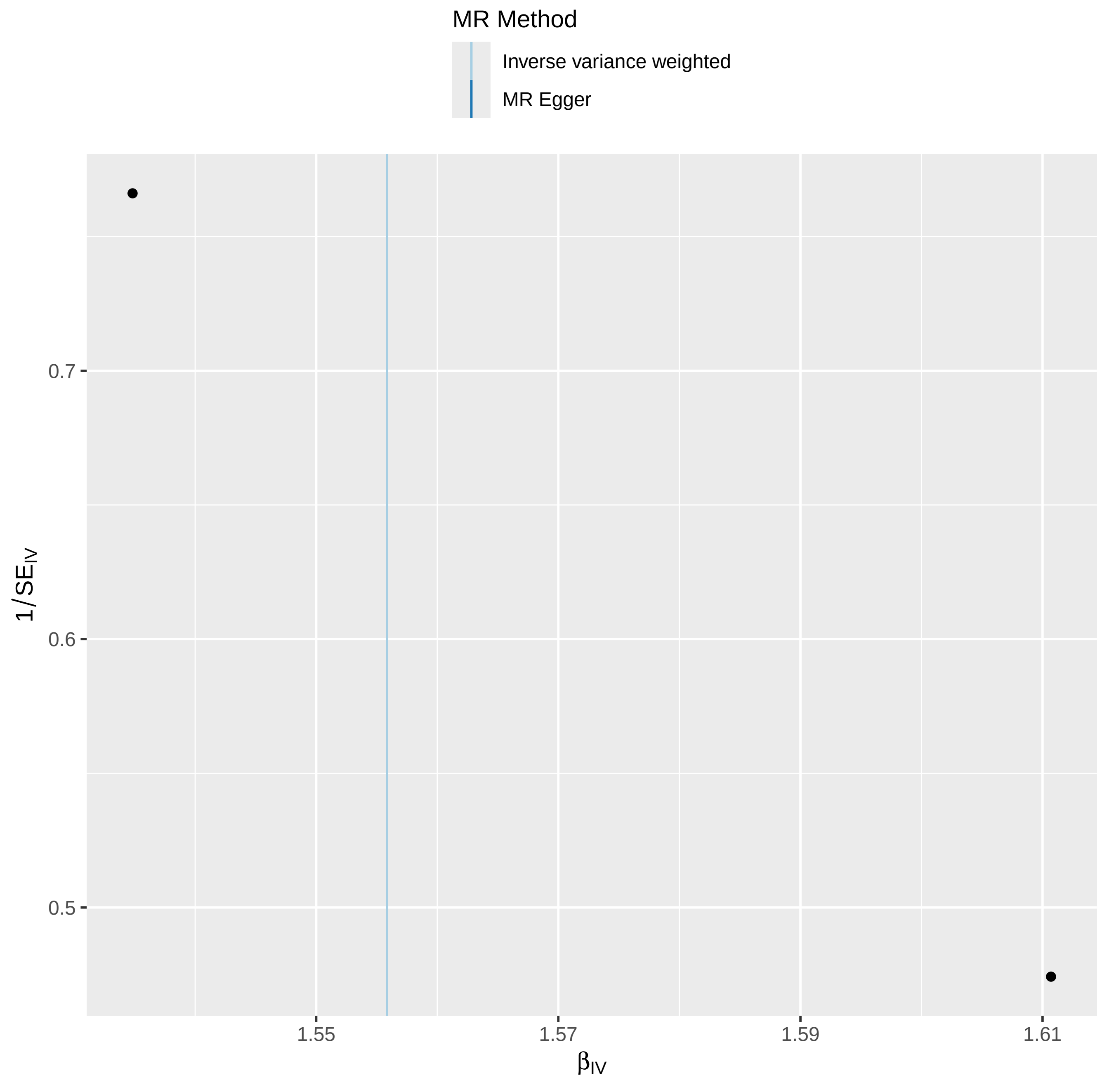

MR Method
Inverse variance weighted
MR Egger
0.7
0.6
0.5
1/SE_IV
1.55
1.57
1.59
1.61
β_IV

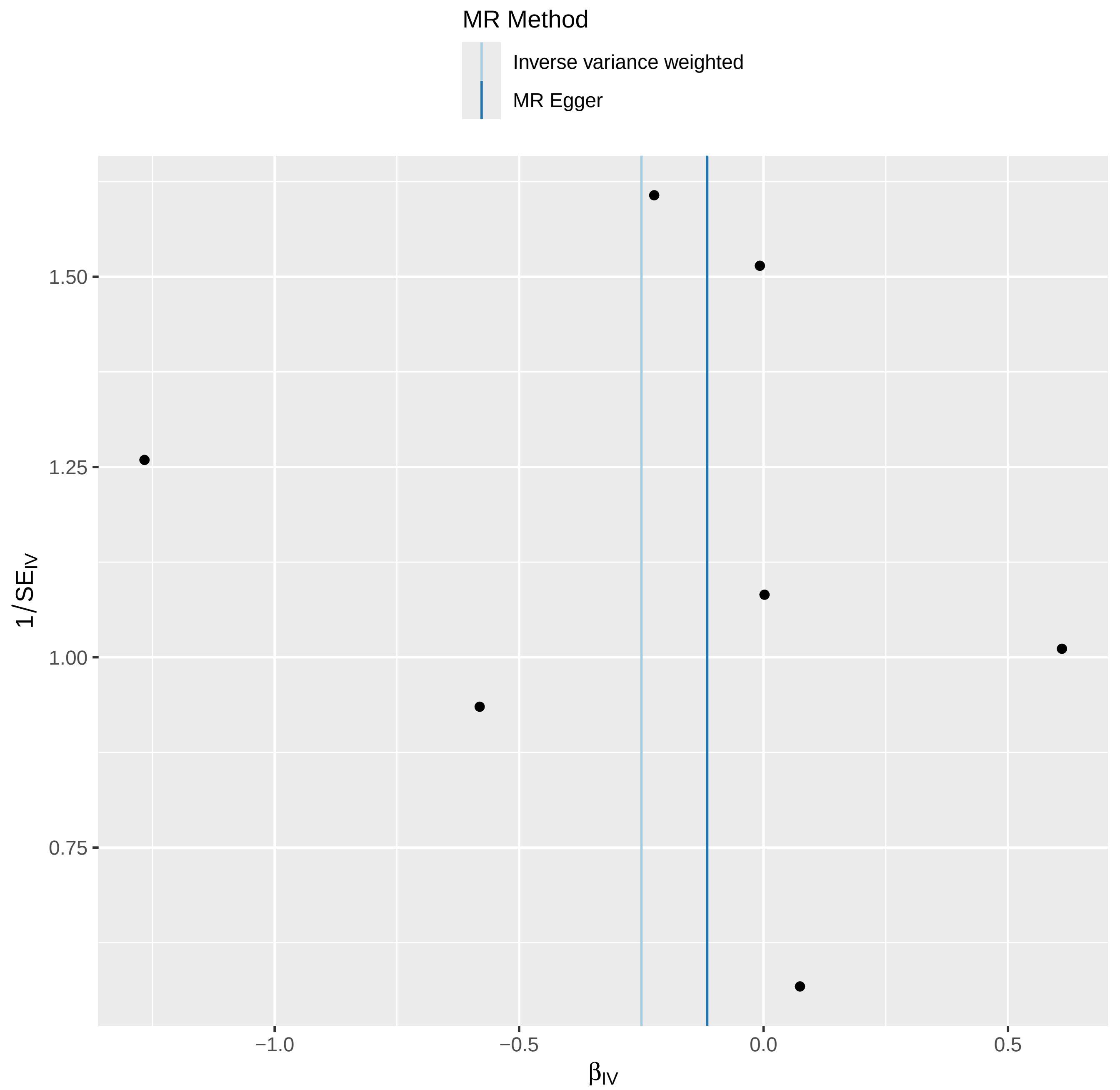

MR Method
Inverse variance weighted
MR Egger
1.50
1.25
1.00
0.75
1/SE_IV
−1.0
−0.5
0.0
0.5
β_IV

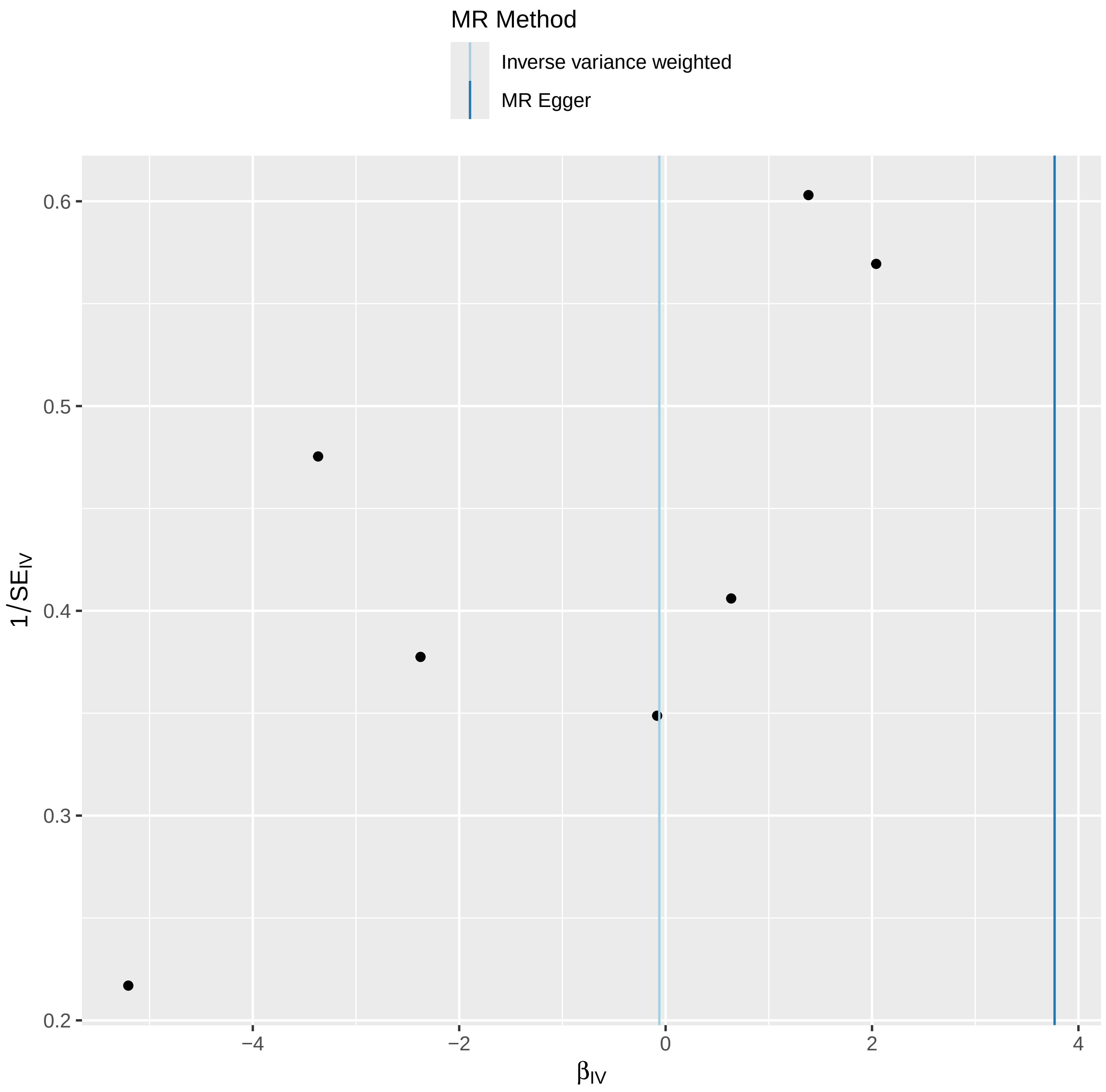

MR Method
Inverse variance weighted
MR Egger
0.6
0.5
0.4
0.3
0.2
1/SE_IV
−4
−2
0
2
4
β_IV

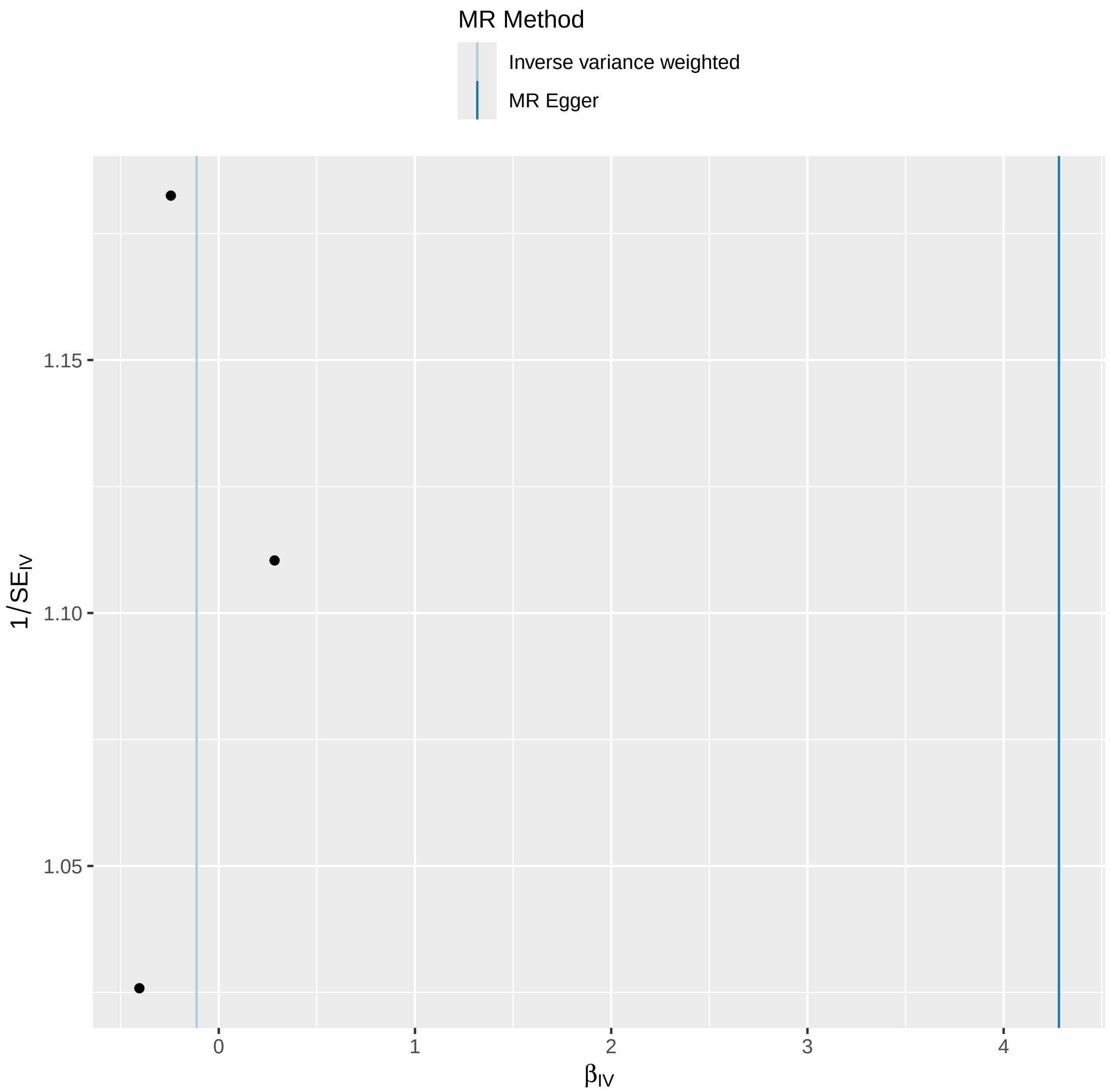
MR Method
Inverse variance weighted
MR Egger
1.15
1.10
1.05
1/SE_IV
0
1
2
3
4
β_IV

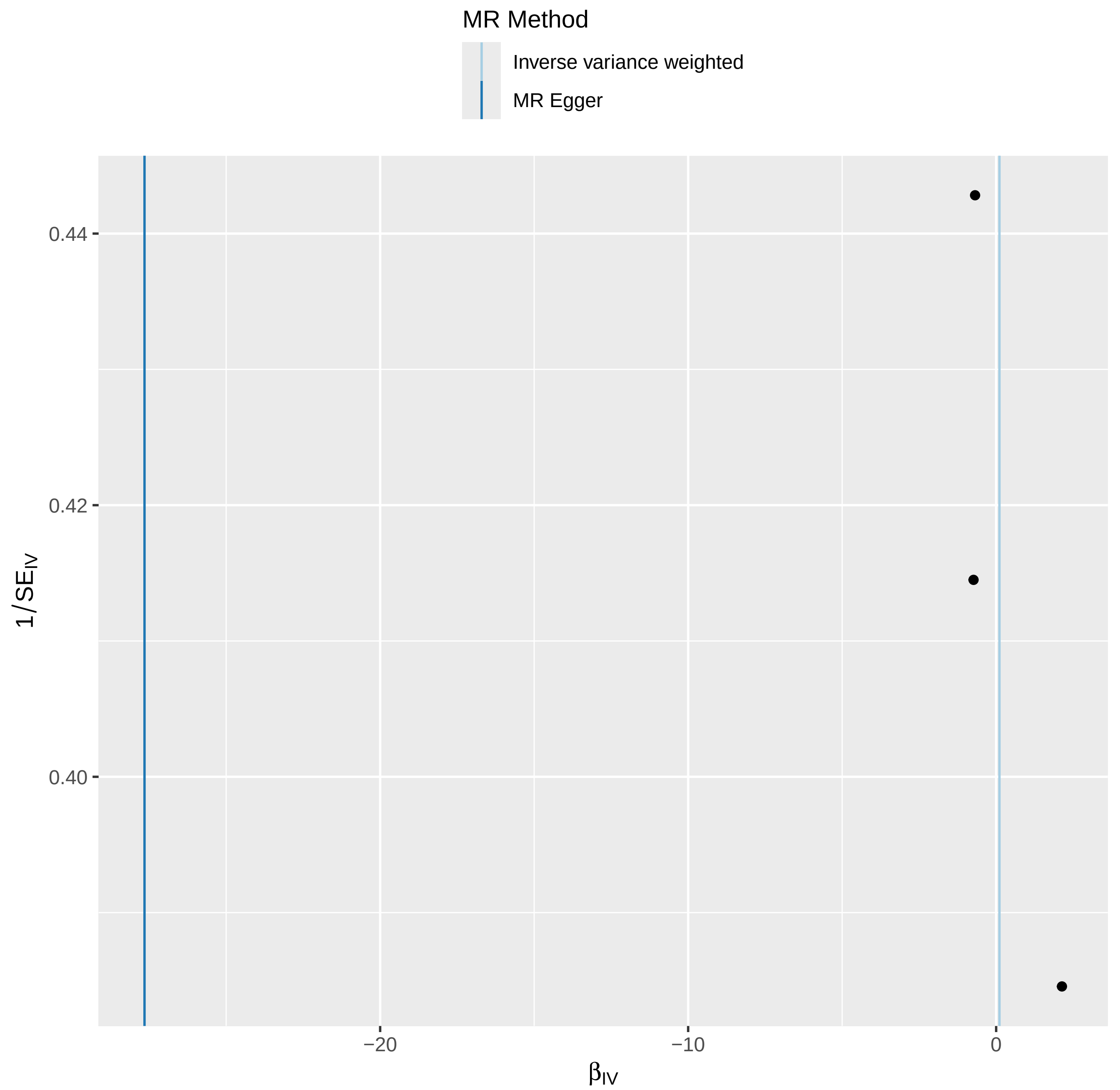

MR Method
Inverse variance weighted
MR Egger
0.44
0.42
0.40
1/SE$_{IV}$
−20
−10
0
β$_{IV}$

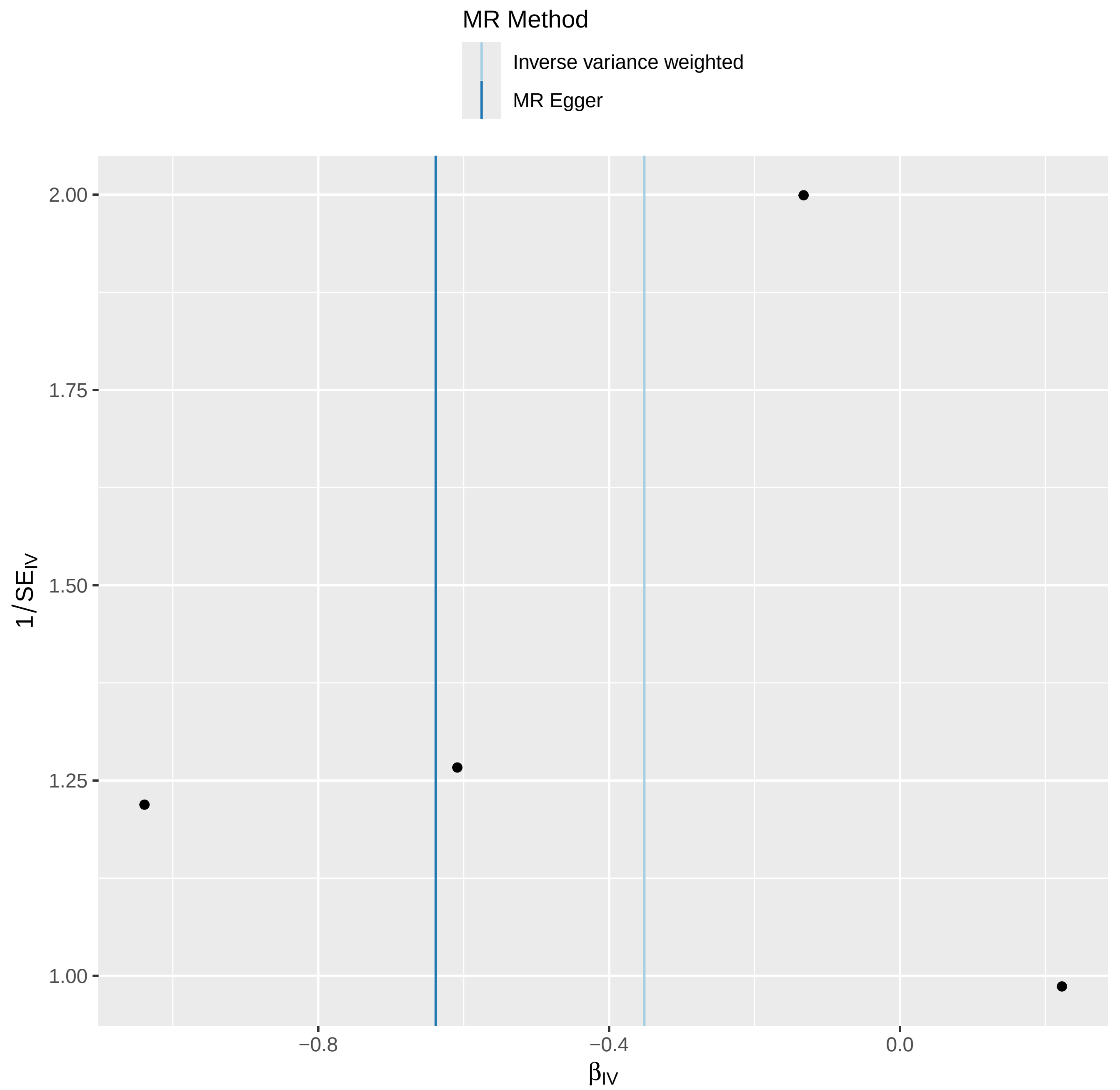

MR Method
Inverse variance weighted
MR Egger
2.00
1.75
1.50
1.25
1.00
$1/SE_{IV}$
−0.8
−0.4
0.0
β_{IV}

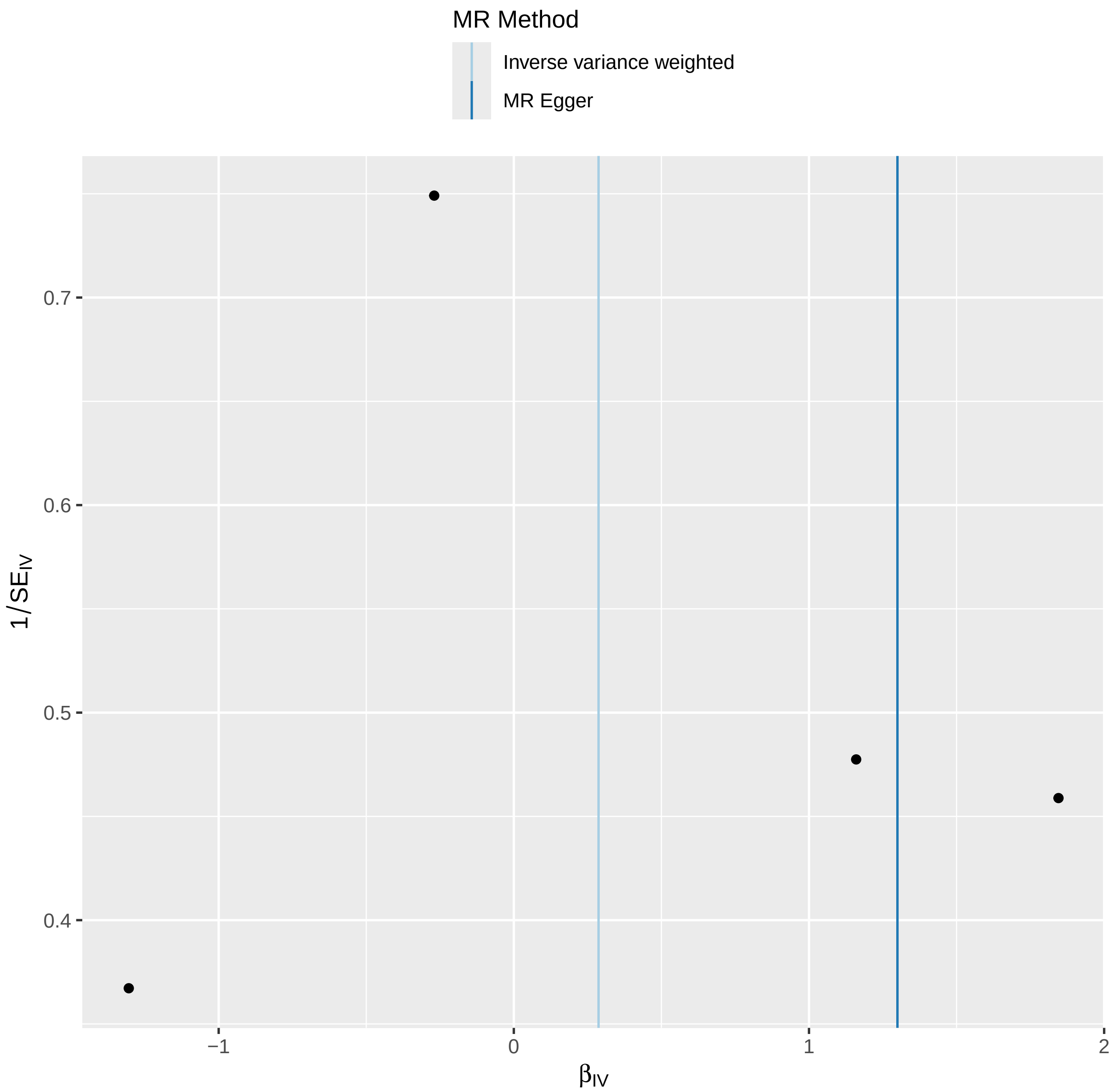

MR Method
Inverse variance weighted
MR Egger
0.7
0.6
0.5
0.4
1/SE_IV
−1
0
1
2
β_IV

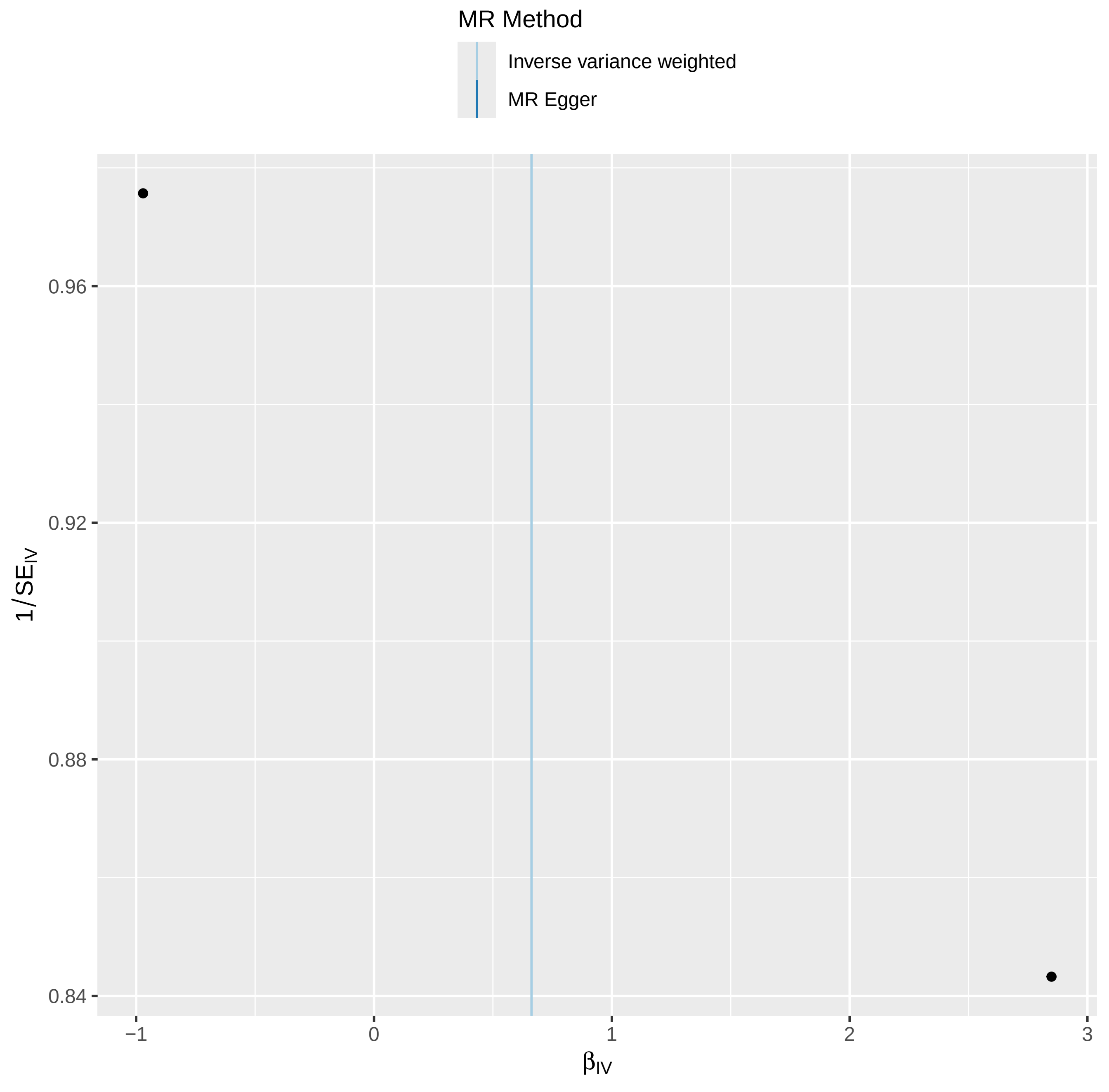
MR Method
Inverse variance weighted
MR Egger
0.96
0.92
0.88
0.84
−1
0
1
2
3
$1/SE_{IV}$
β_{IV}

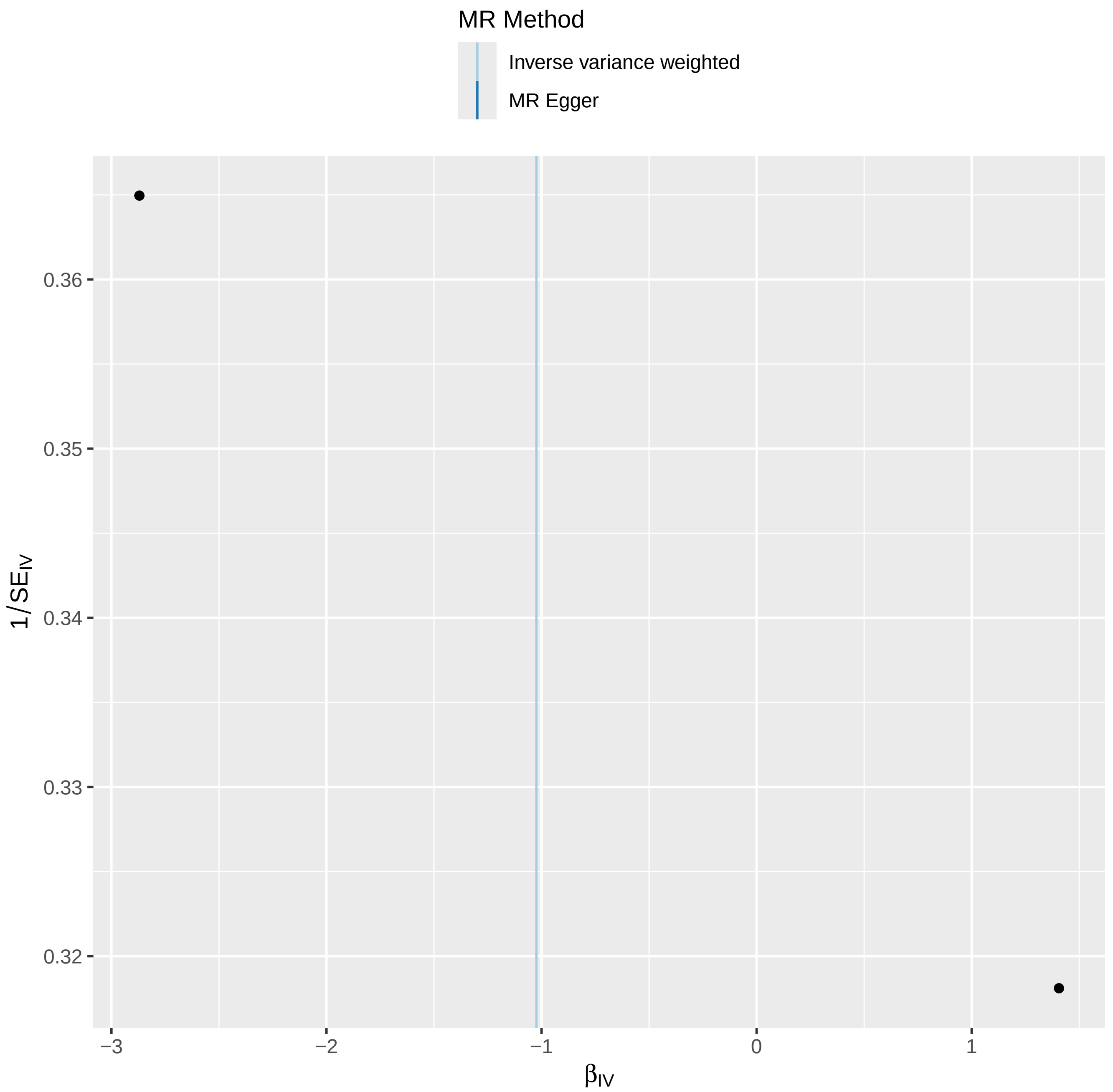

MR Method
Inverse variance weighted
MR Egger
0.36
0.35
0.34
0.33
0.32
1/SE$_{IV}$
−3
−2
−1
0
1
β$_{IV}$

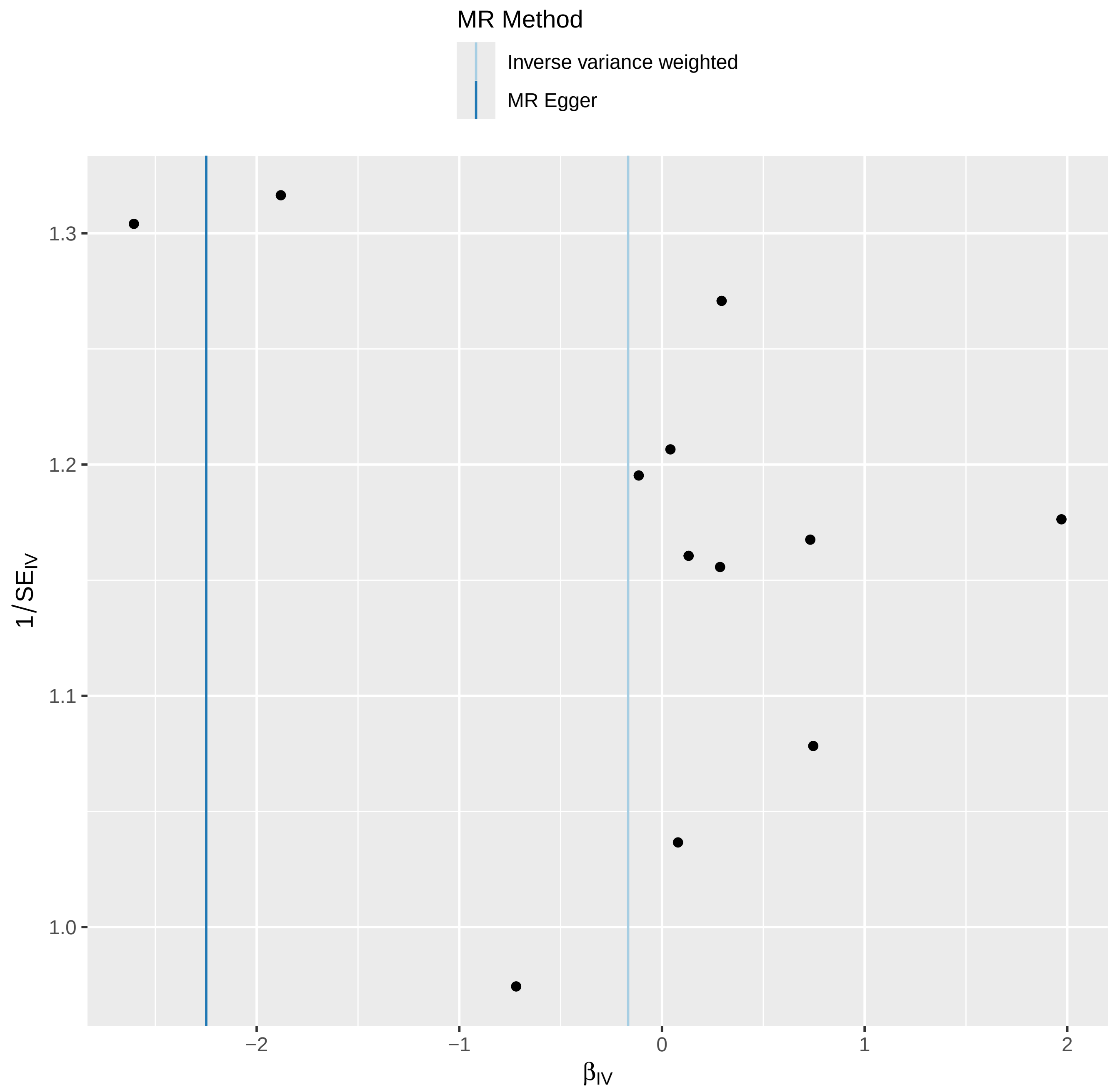
MR Method
Inverse variance weighted
MR Egger
1.3
1.2
1.1
1.0
1/SE_IV
−2
−1
0
1
2
β_IV

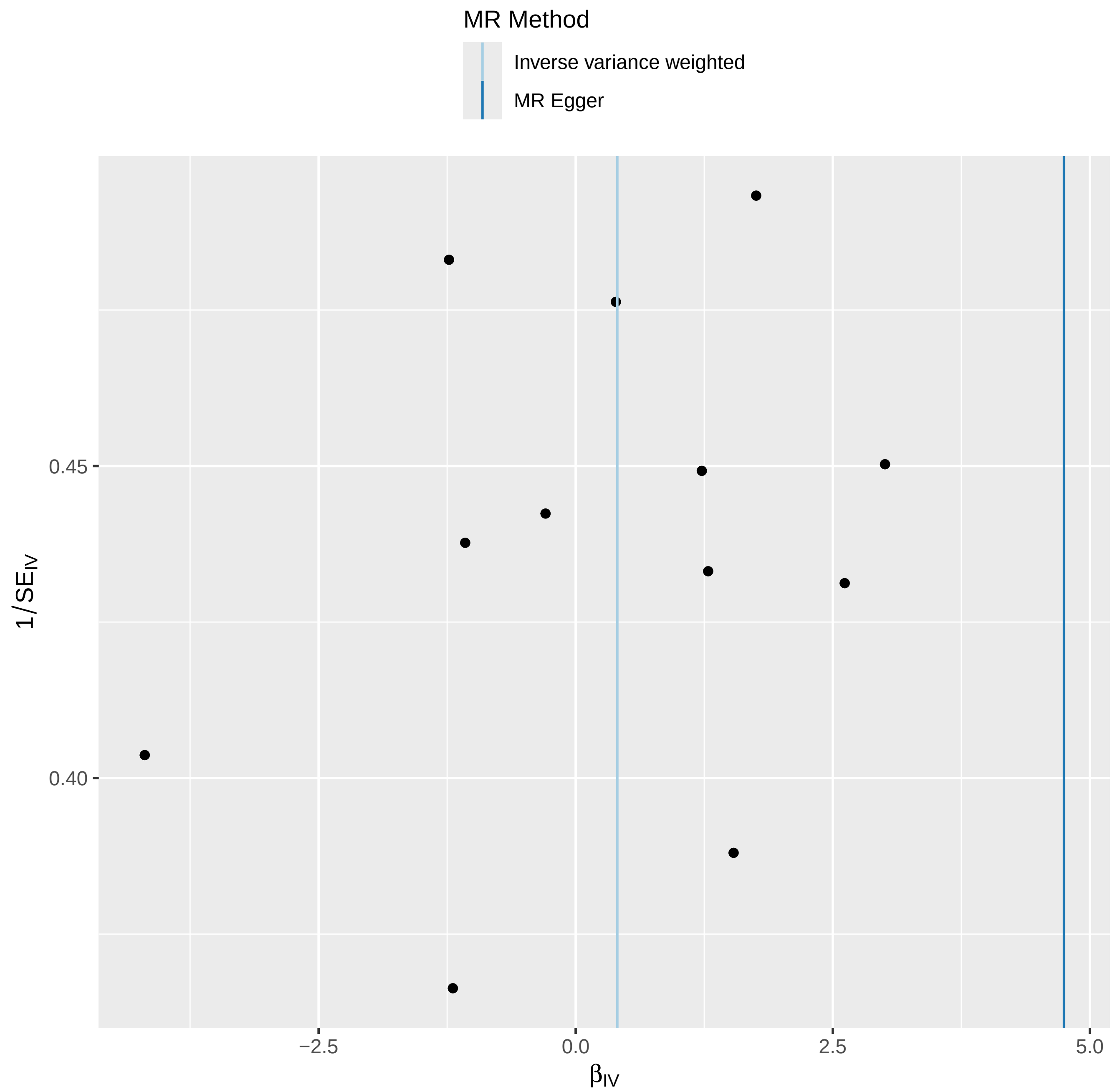
MR Method
Inverse variance weighted
MR Egger
0.45
0.40
1/SE_IV
−2.5
0.0
2.5
5.0
β_IV

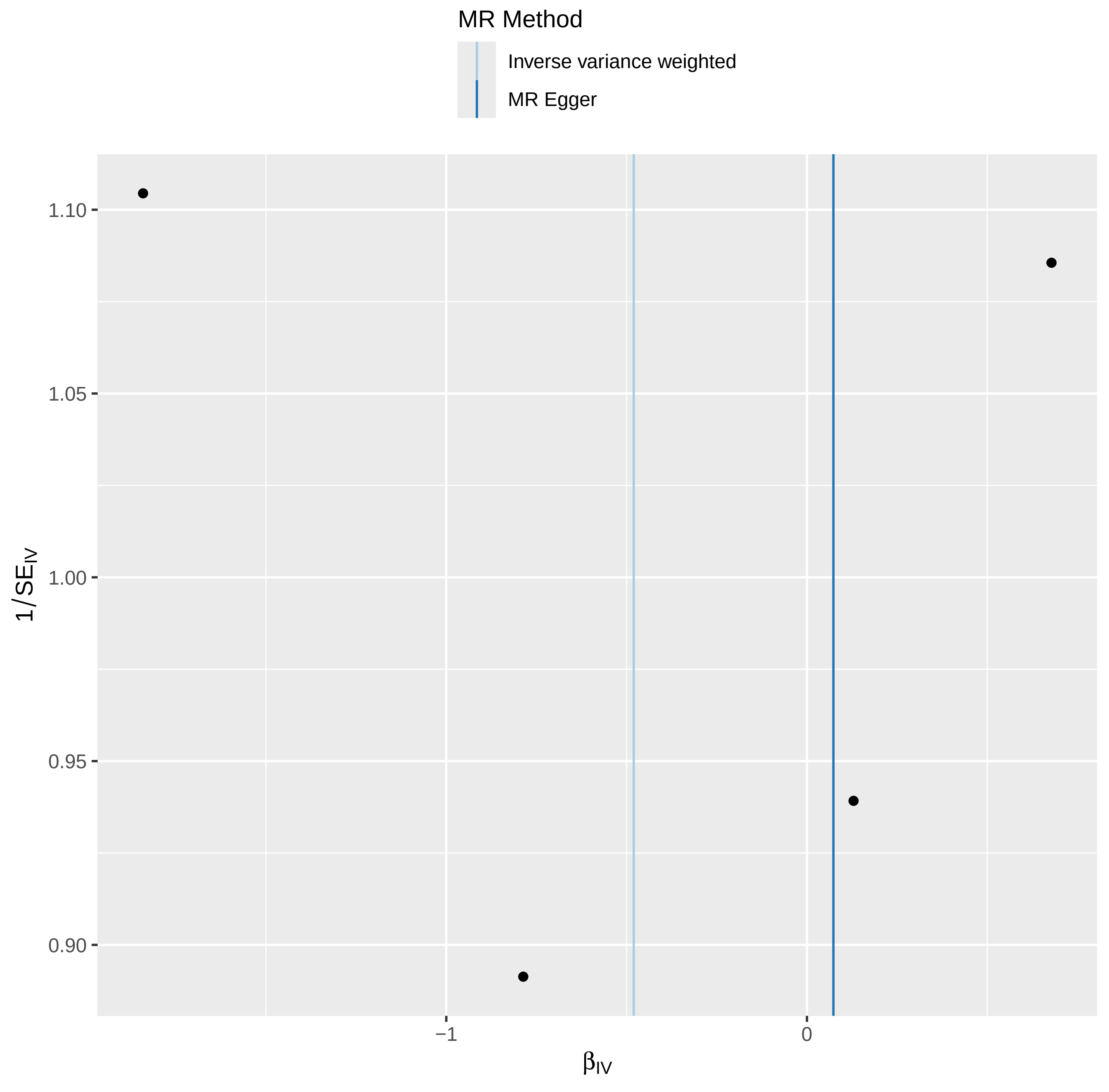
MR Method
Inverse variance weighted
MR Egger
1.10
1.05
1.00
0.95
0.90
$1/SE_{IV}$
−1
0
β_{IV}

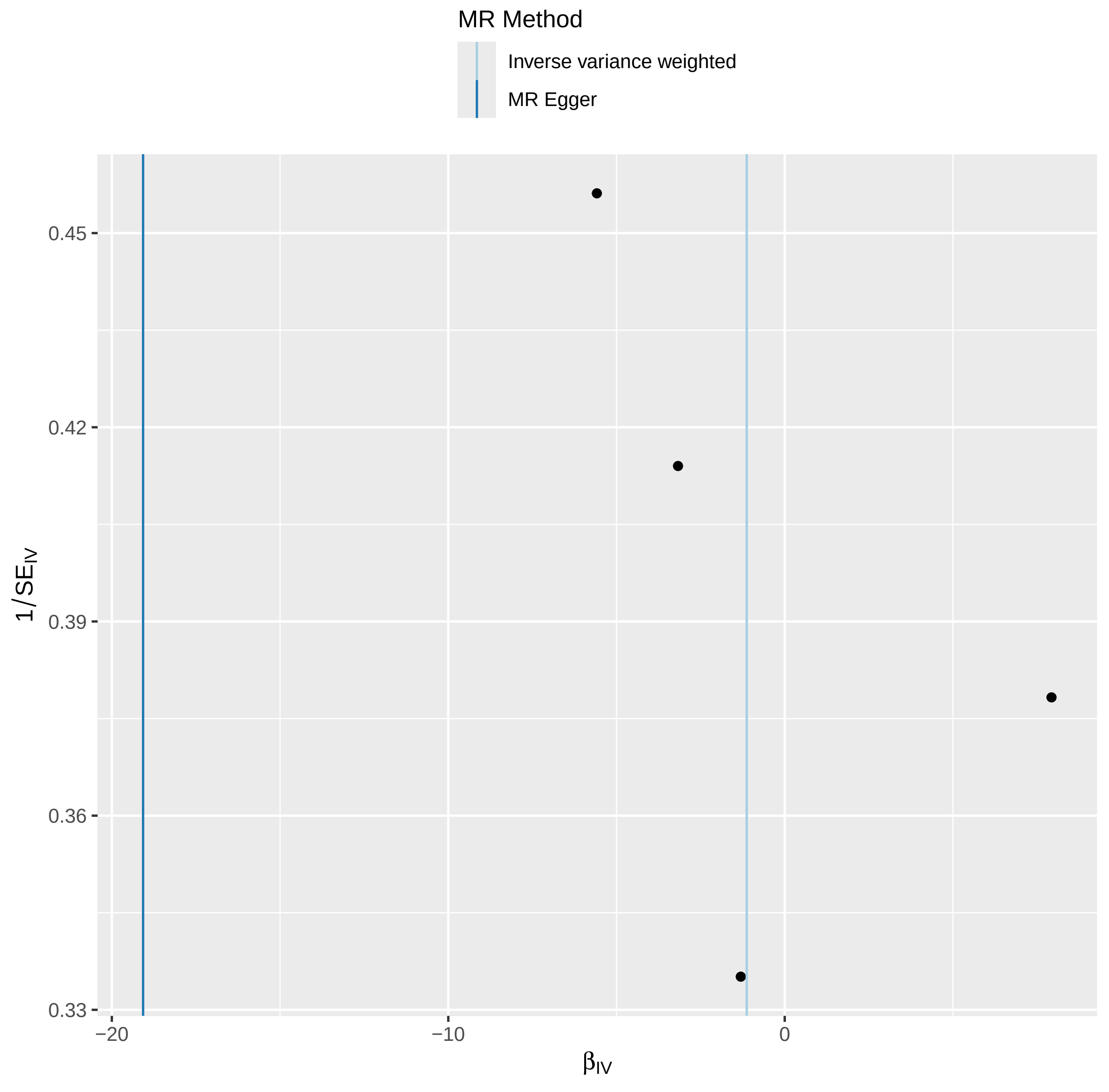
MR Method
Inverse variance weighted
MR Egger
0.45
0.42
0.39
0.36
0.33
1/SE_IV
−20
−10
0
β_IV

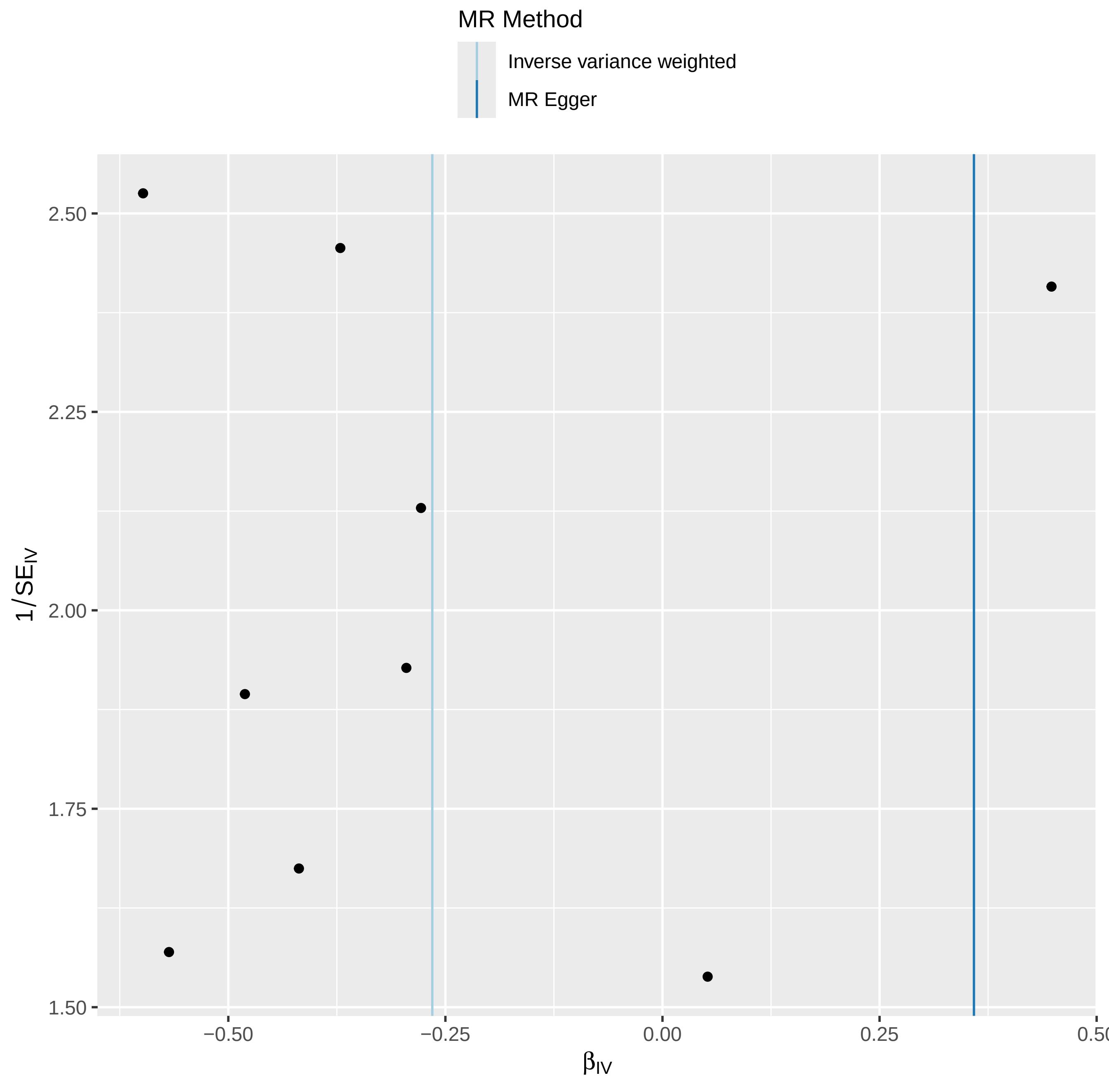
MR Method
Inverse variance weighted
MR Egger
2.50
2.25
2.00
1.75
1.50
$1/SE_{IV}$
−0.50
−0.25
0.00
0.25
0.50
β_{IV}

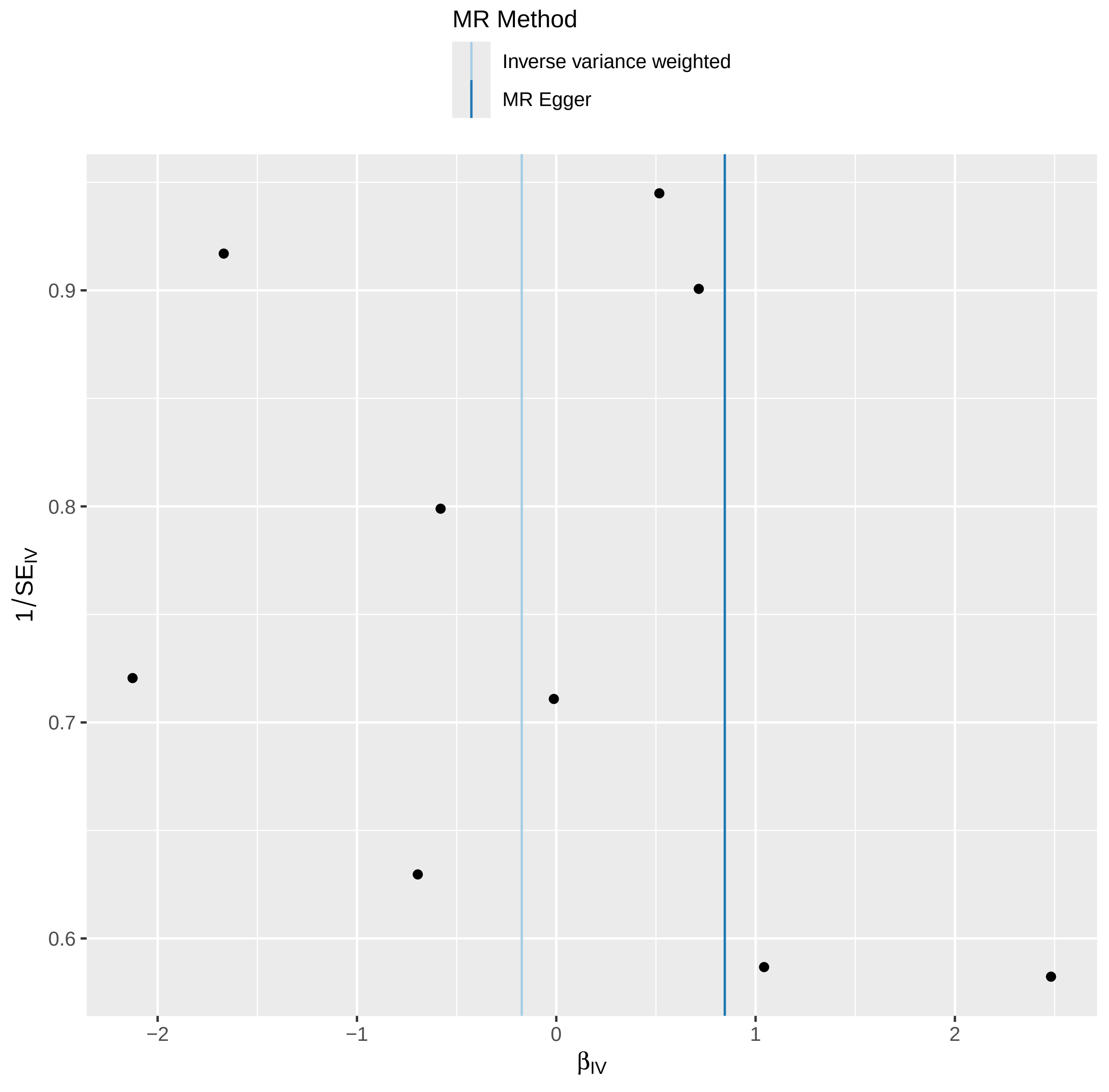

MR Method
Inverse variance weighted
MR Egger
0.9
0.8
0.7
0.6
1/SE_IV
−2
−1
0
1
2
β_IV

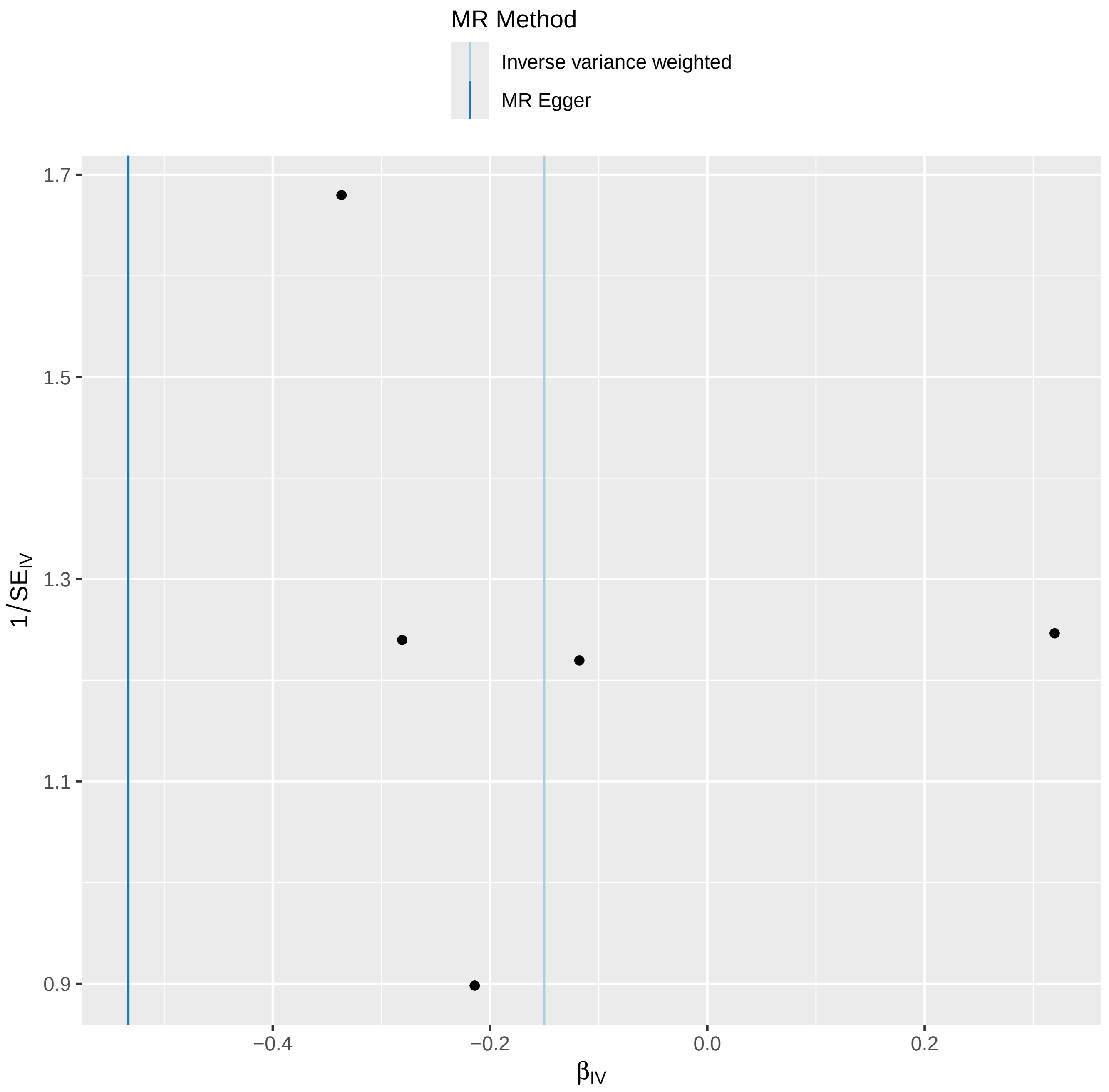

MR Method
Inverse variance weighted
MR Egger
1.7
1.5
1.3
1.1
0.9
1/SE_IV
−0.4
−0.2
0.0
0.2
β_IV

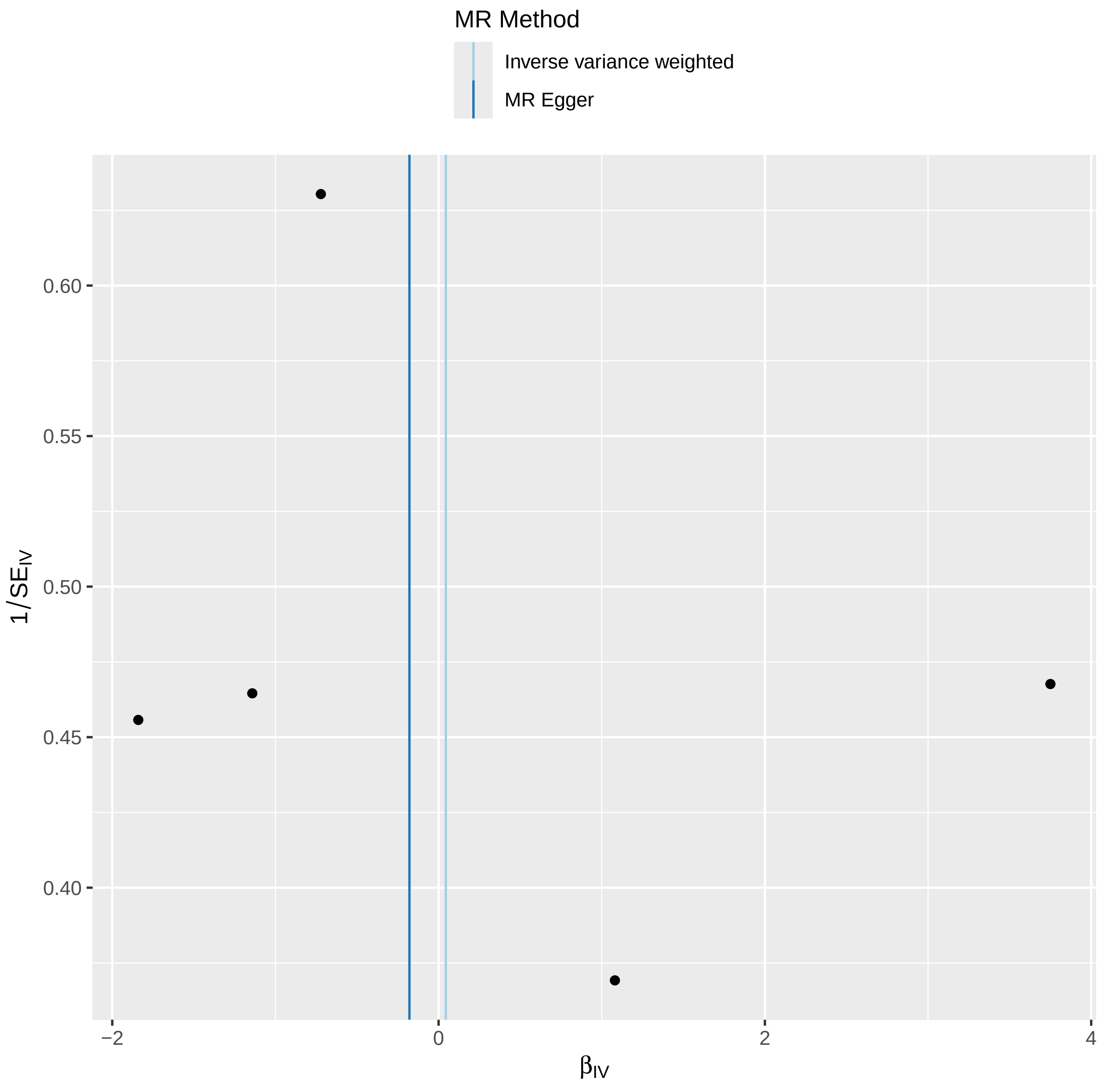

MR Method
Inverse variance weighted
MR Egger
0.60
0.55
0.50
0.45
0.40
1/SE_IV
−2
0
2
4
β_IV

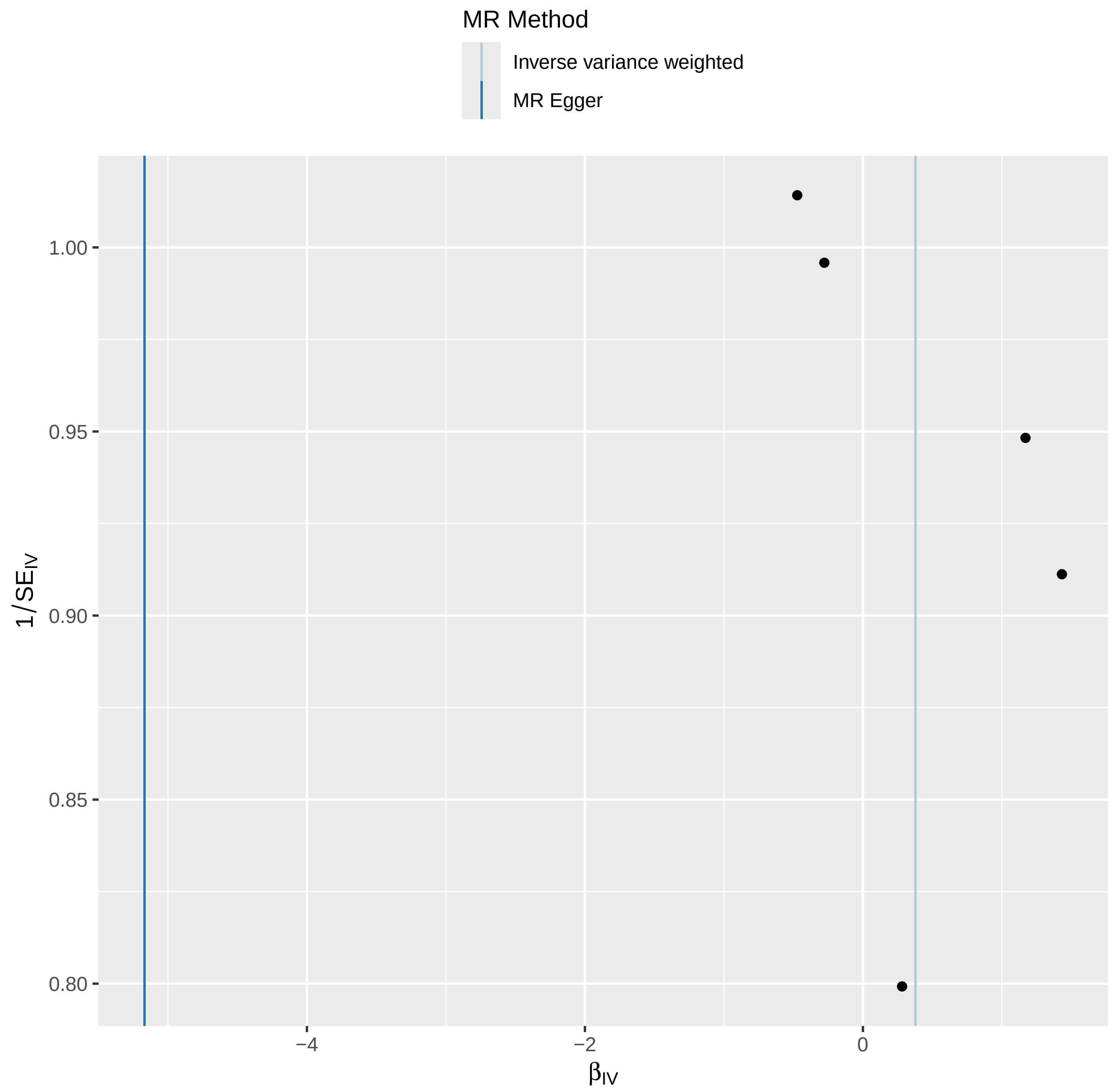
MR Method
Inverse variance weighted
MR Egger
1.00
0.95
0.90
0.85
0.80
−4
−2
0
$1/SE_{IV}$
β_{IV}

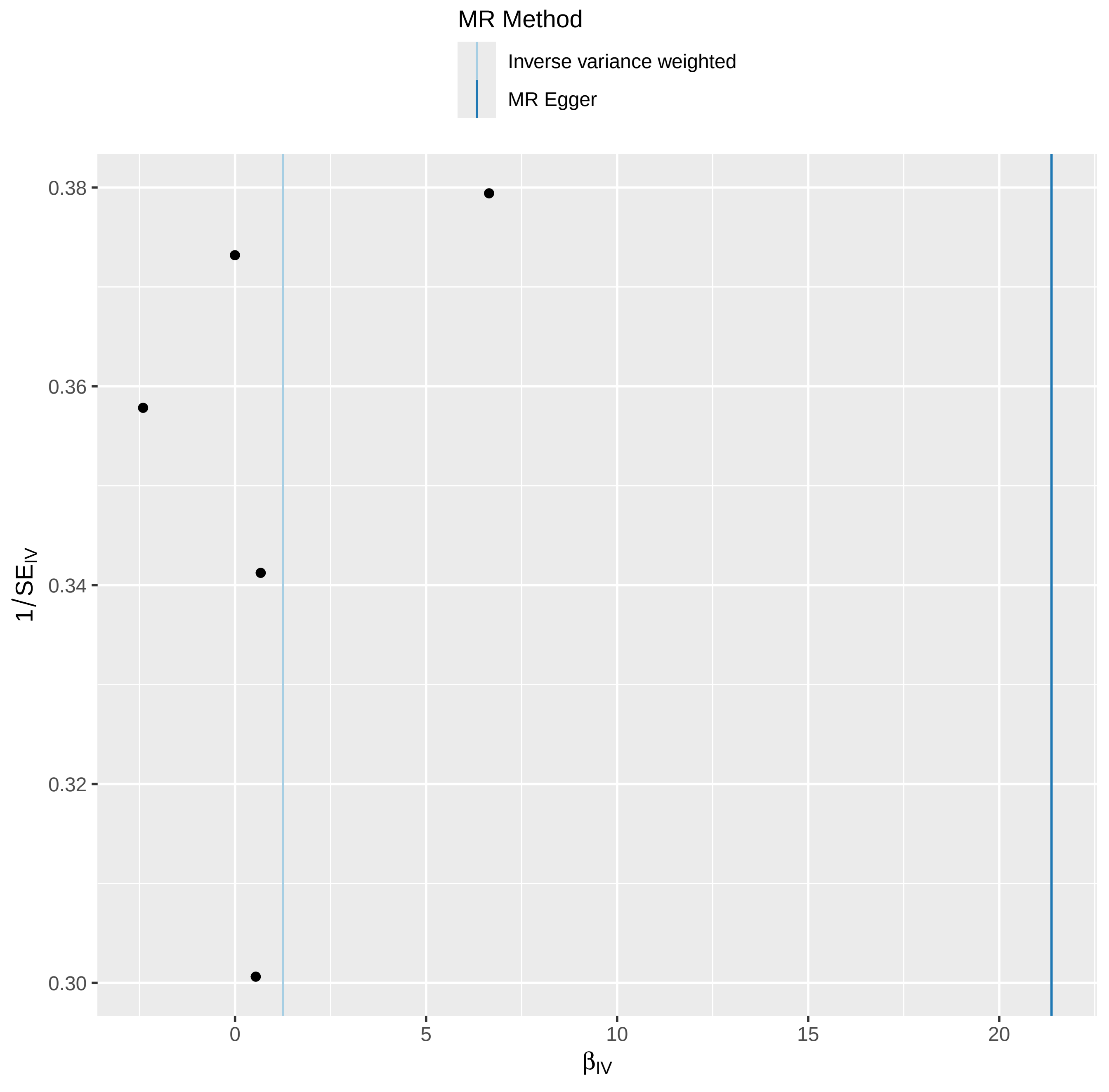
MR Method
Inverse variance weighted
MR Egger
0.38
0.36
0.34
0.32
0.30
1/SE_IV
0
5
10
15
20
β_IV

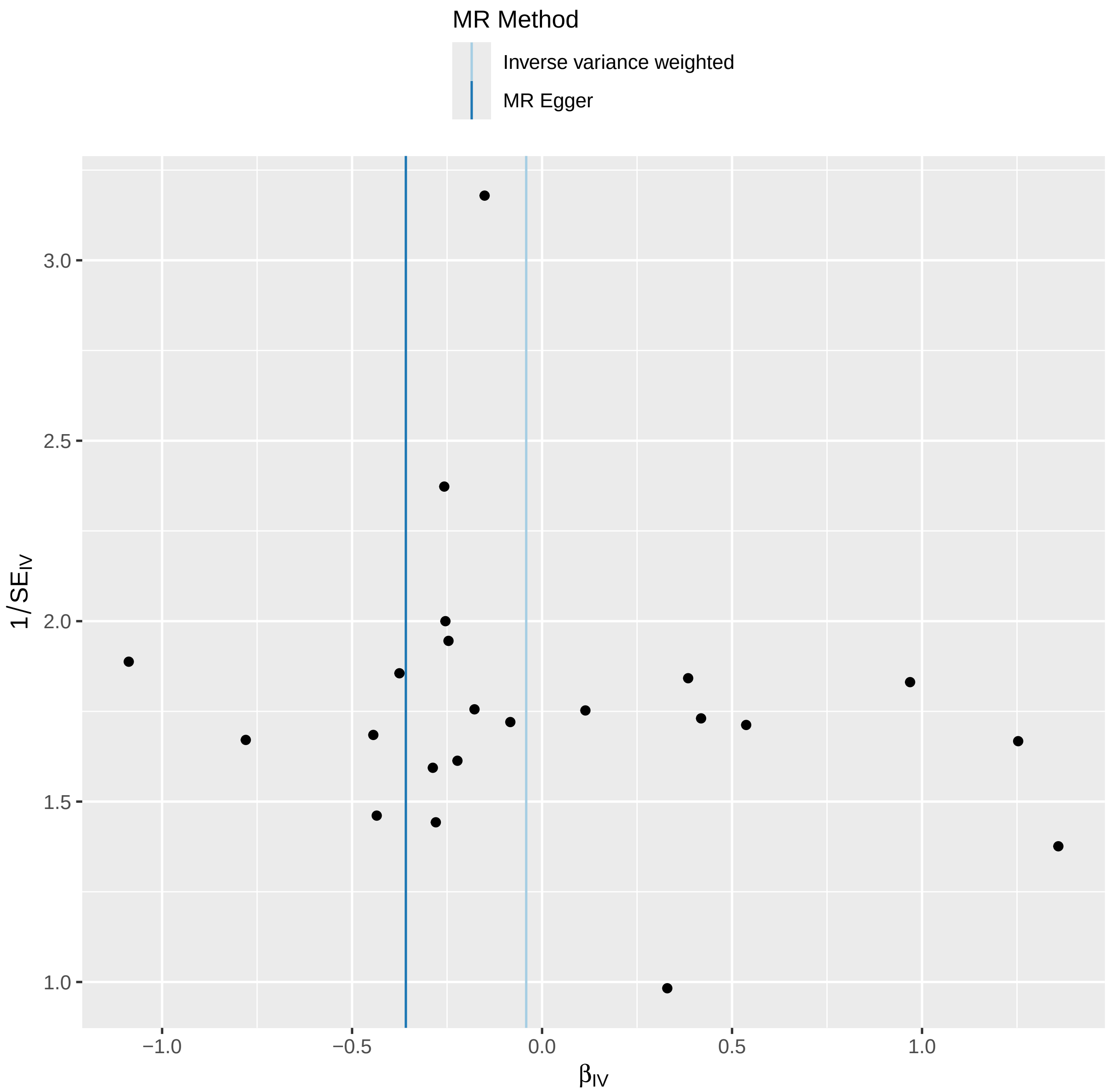
MR Method
Inverse variance weighted
MR Egger
3.0
2.5
2.0
1.5
1.0
$1/SE_{IV}$
−1.0
−0.5
0.0
0.5
1.0
β_{IV}

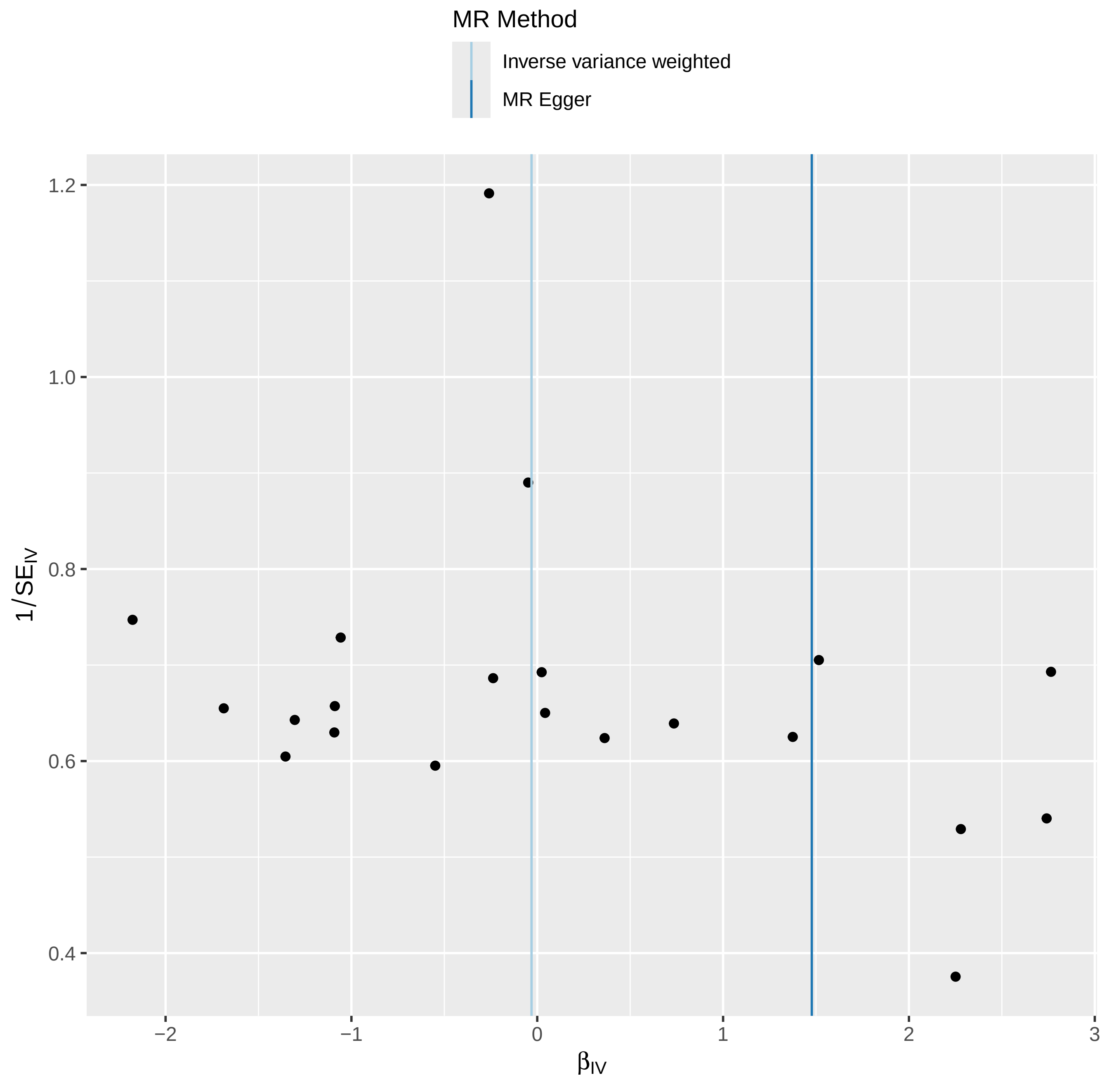
MR Method
Inverse variance weighted
MR Egger
1.2
1.0
0.8
0.6
0.4
1/SE_IV
−2
−1
0
1
2
3
β_IV

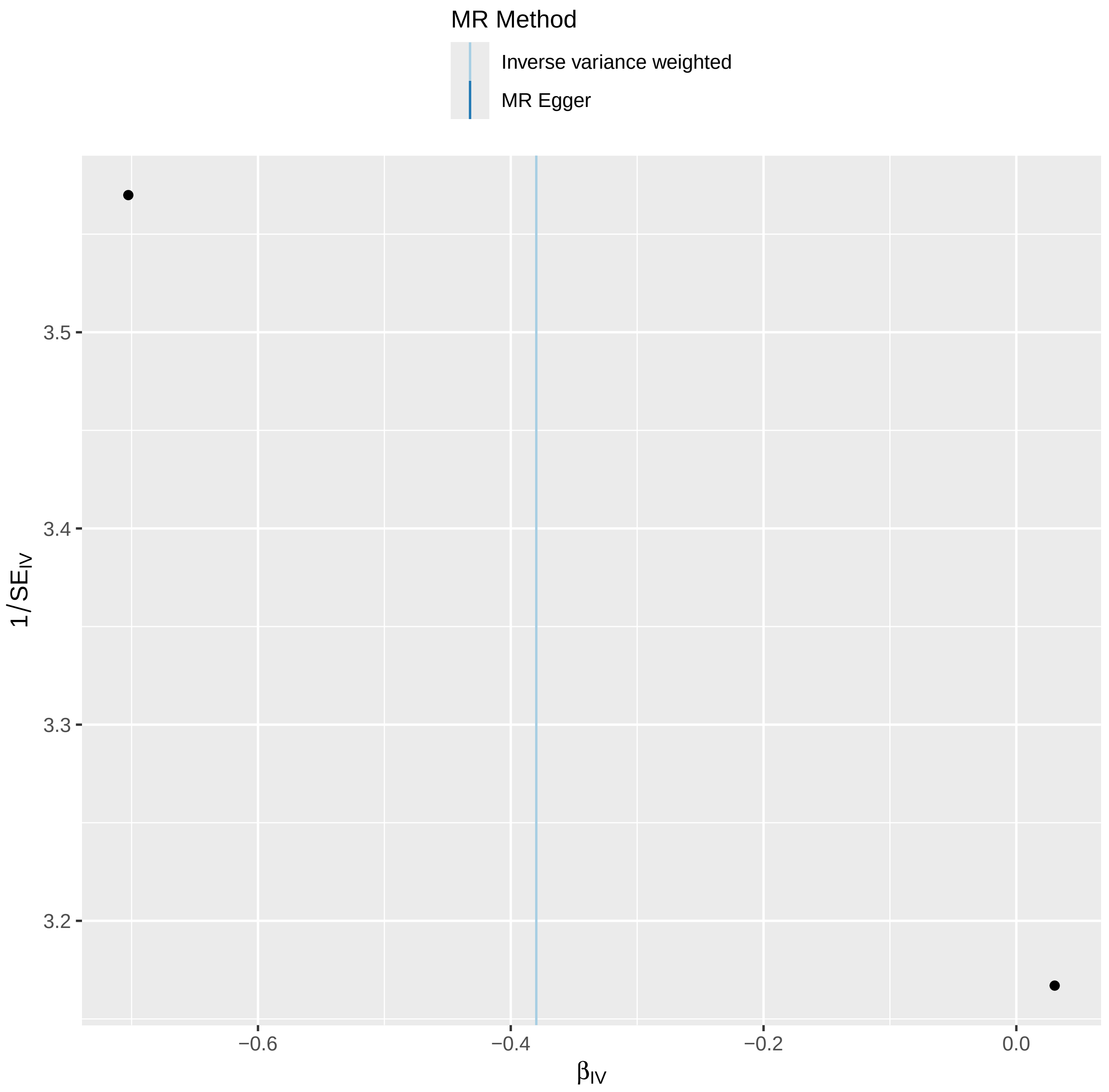
MR Method
Inverse variance weighted
MR Egger
3.5
3.4
3.3
3.2
$1/SE_{IV}$
−0.6
−0.4
−0.2
0.0
β_{IV}

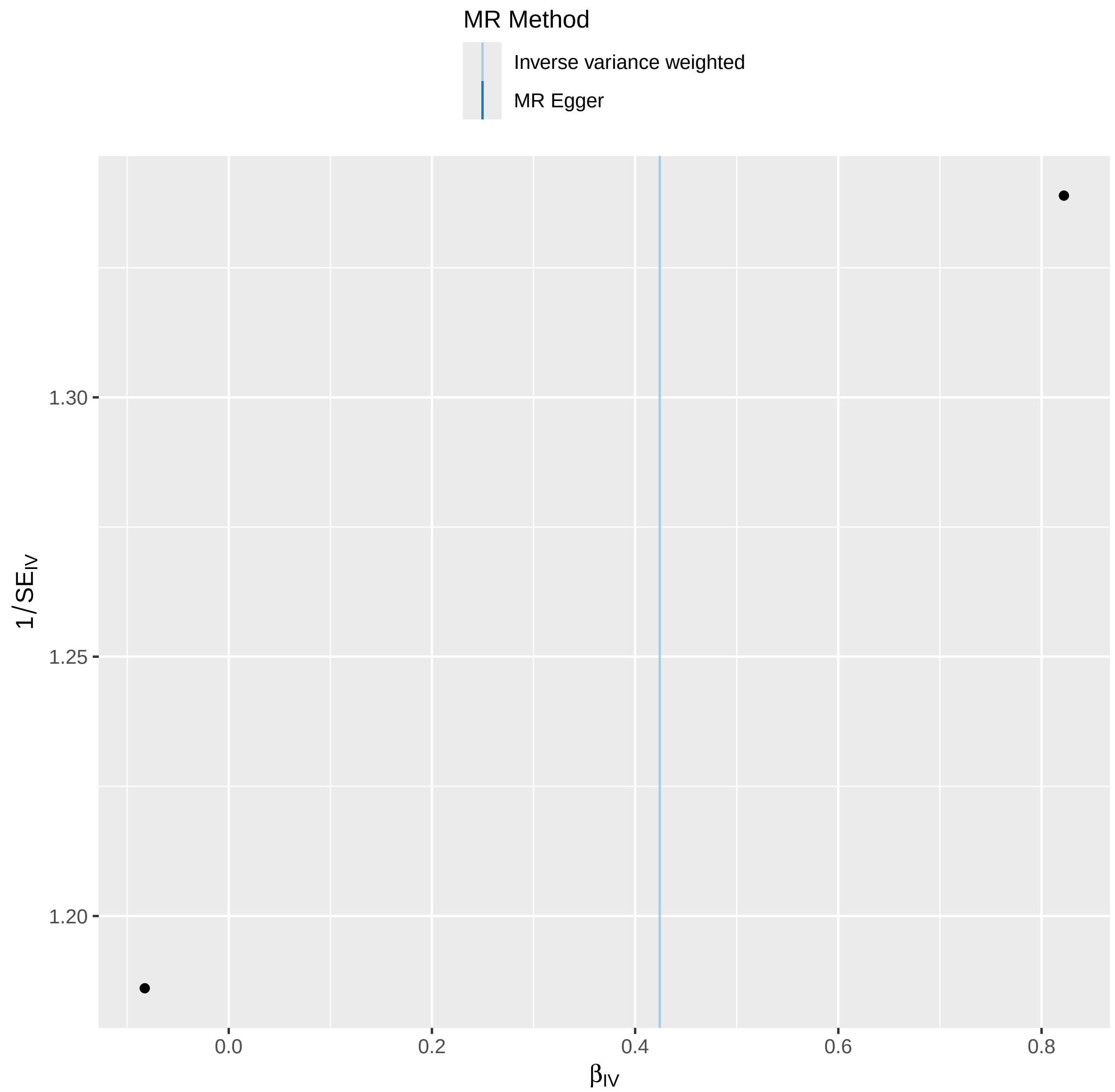
MR Method
Inverse variance weighted
MR Egger
1.30
1.25
1.20
1/SE$_{IV}$
0.0
0.2
0.4
0.6
0.8
β$_{IV}$

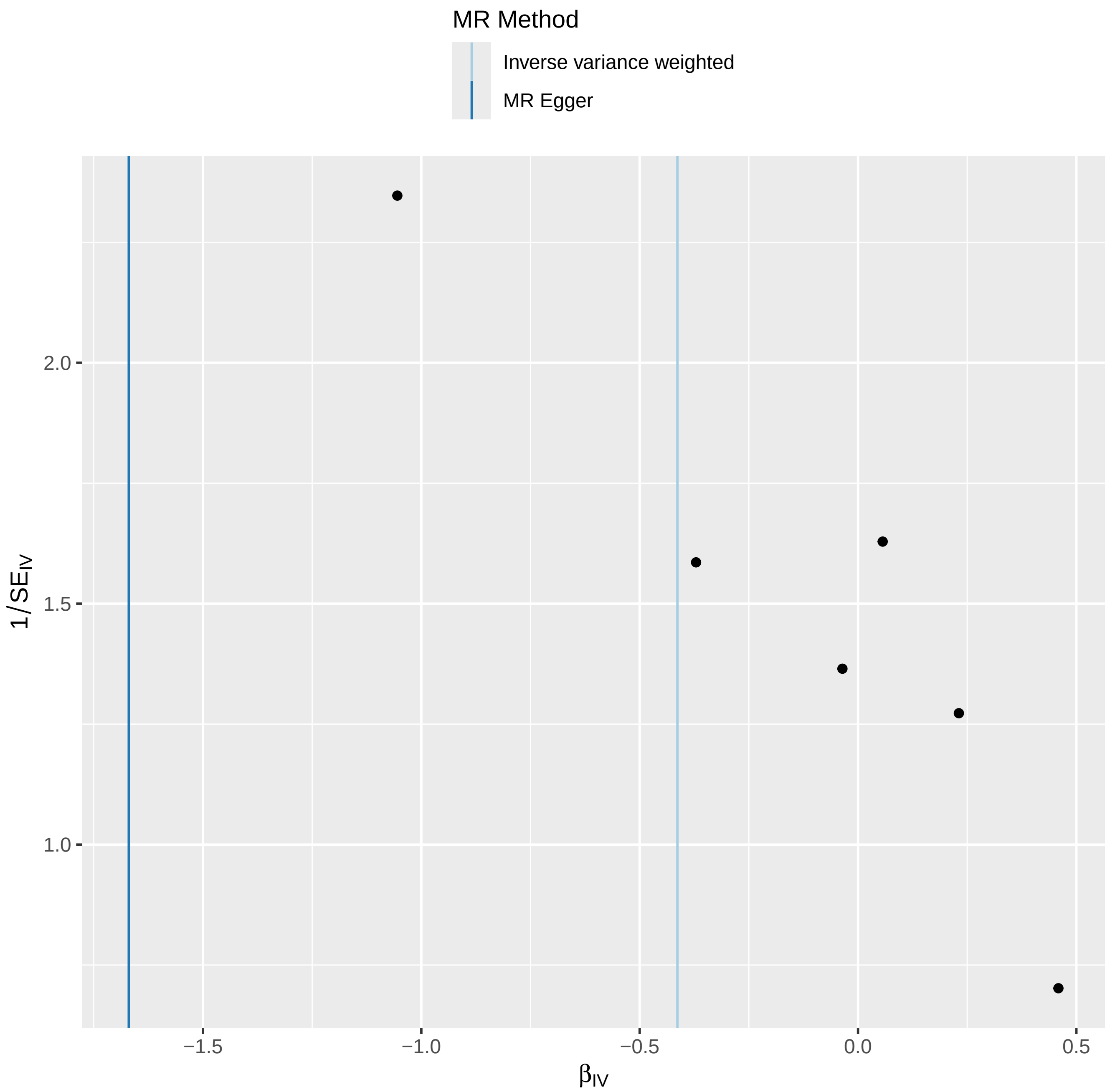
MR Method
Inverse variance weighted
MR Egger
2.0
1.5
1.0
−1.5
−1.0
−0.5
0.0
0.5
$1/SE_{IV}$
β_{IV}

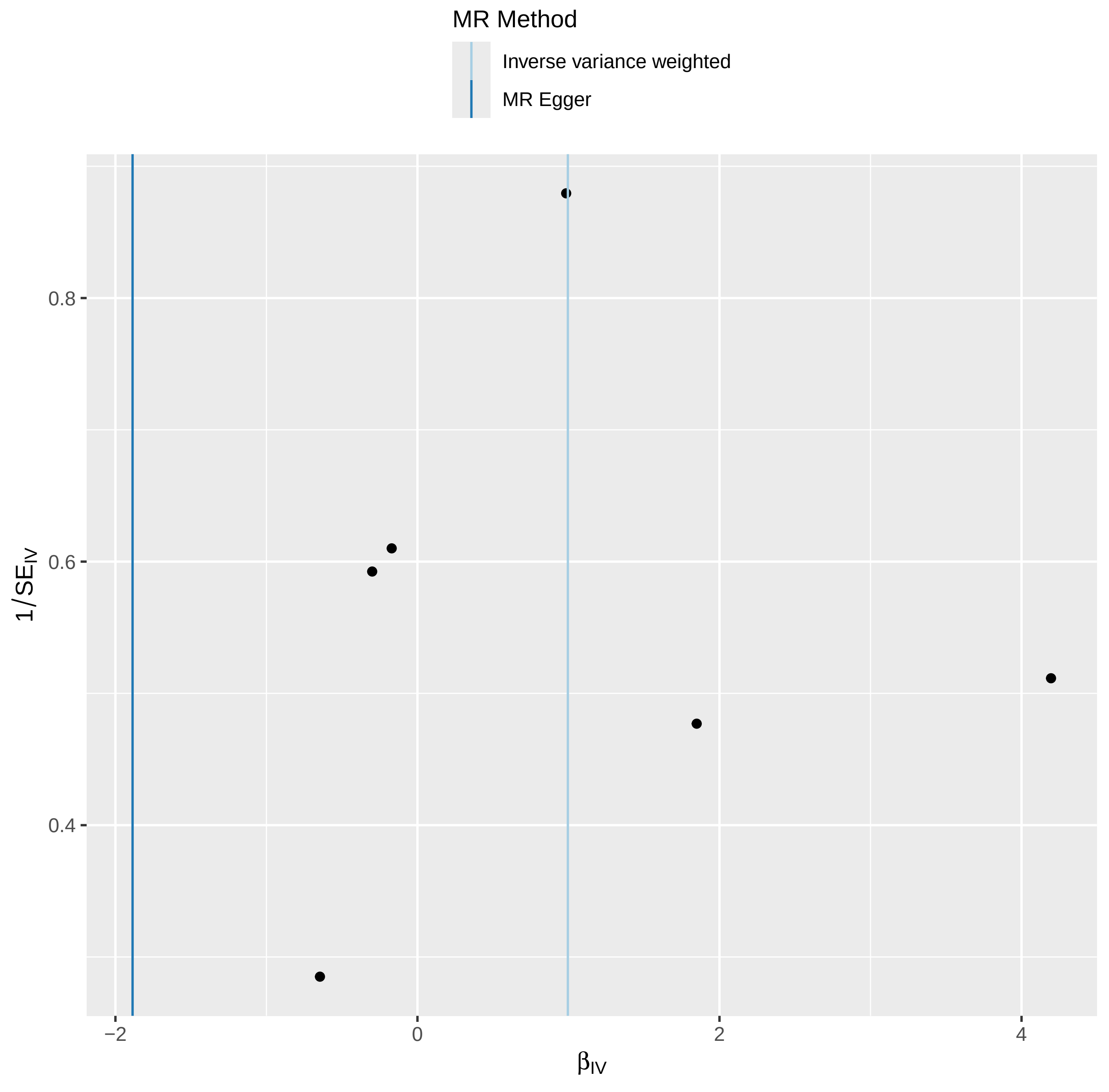
MR Method
Inverse variance weighted
MR Egger
0.8
0.6
0.4
1/SE$_{IV}$
−2
0
2
4
β$_{IV}$

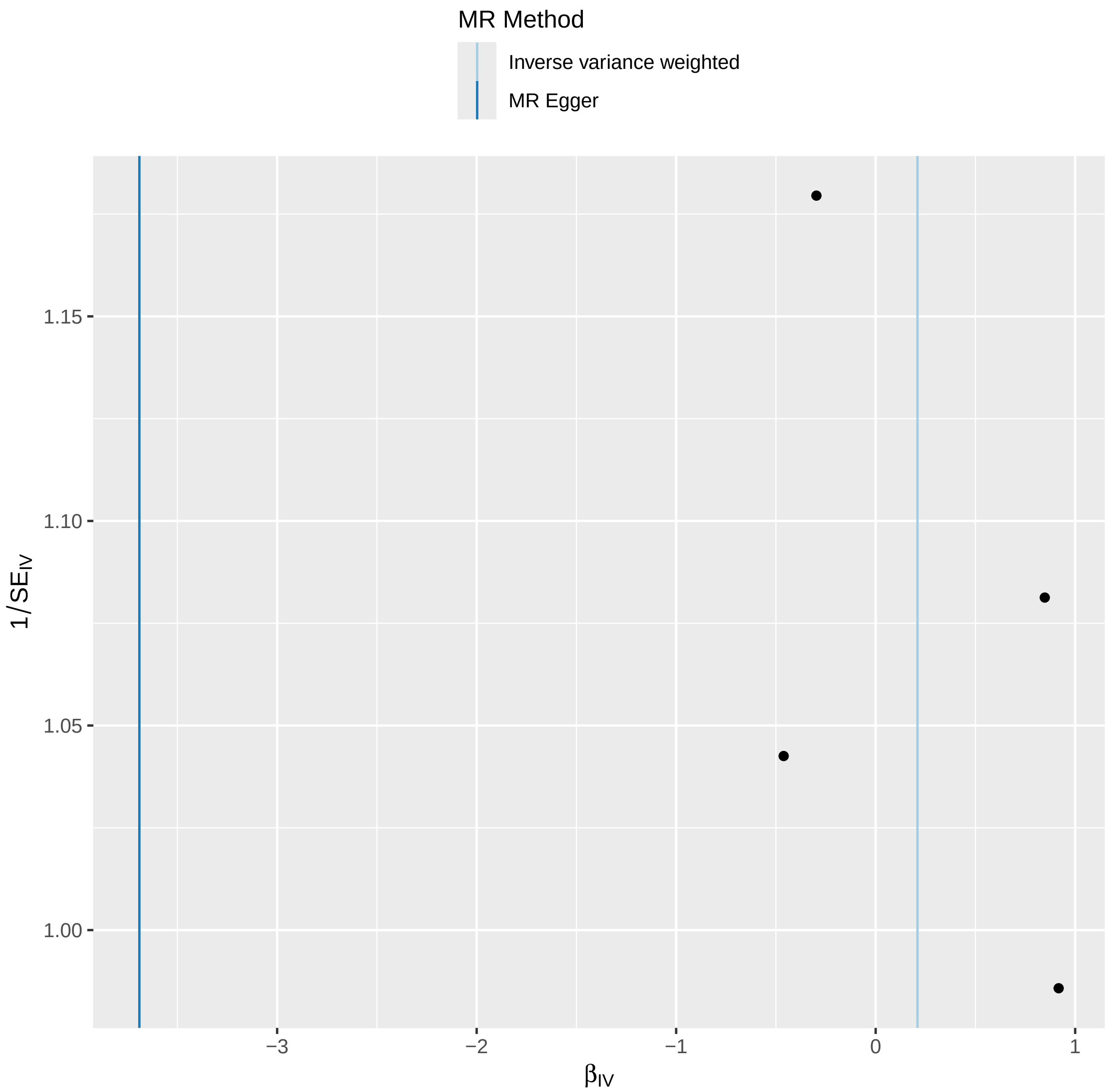
MR Method
Inverse variance weighted
MR Egger
1.15
1.10
1.05
1.00
1/SE_IV
−3
−2
−1
0
1
β_IV

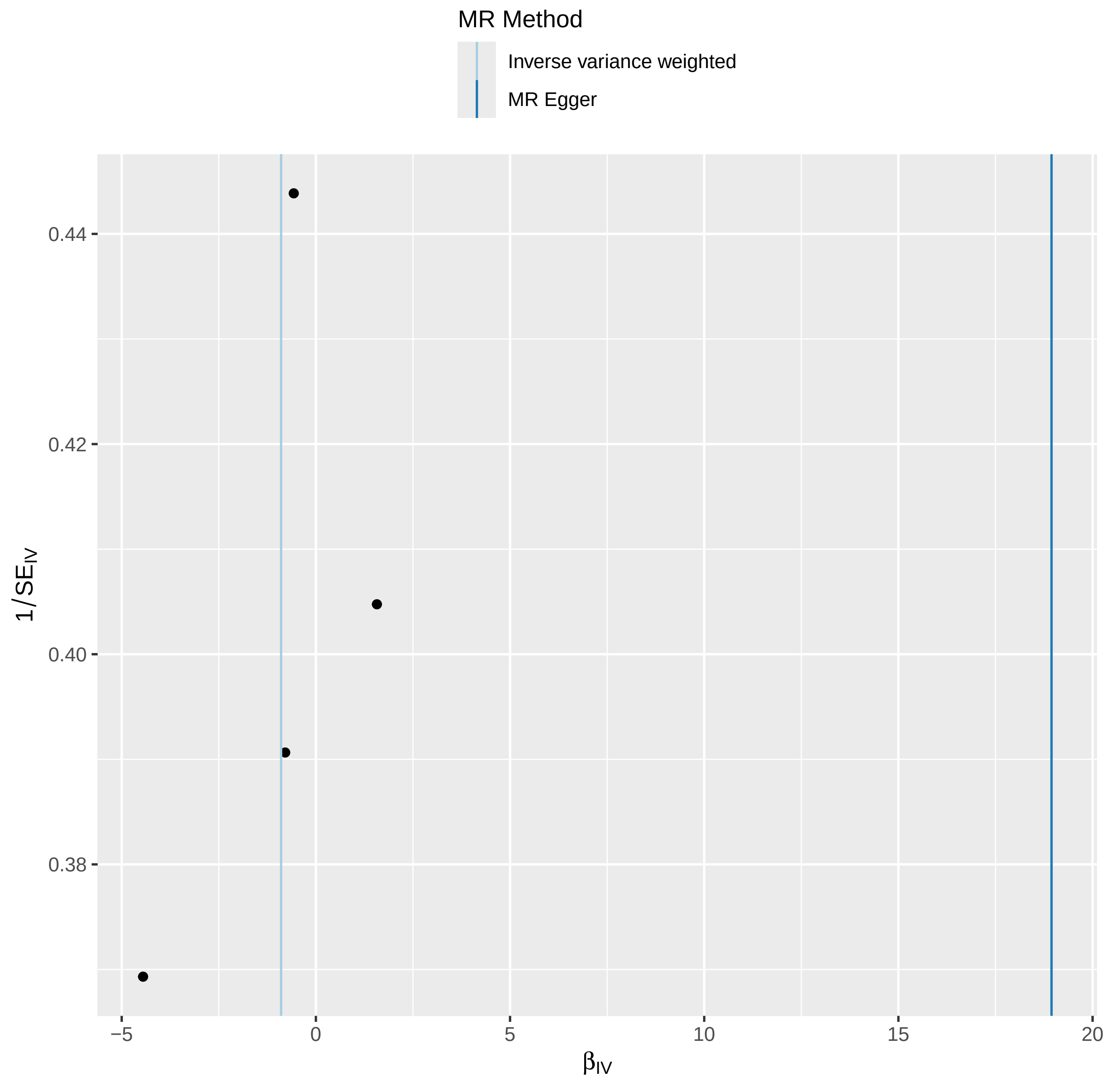
MR Method
Inverse variance weighted
MR Egger
0.44
0.42
0.40
0.38
1/SE_IV
−5
0
5
10
15
20
β_IV

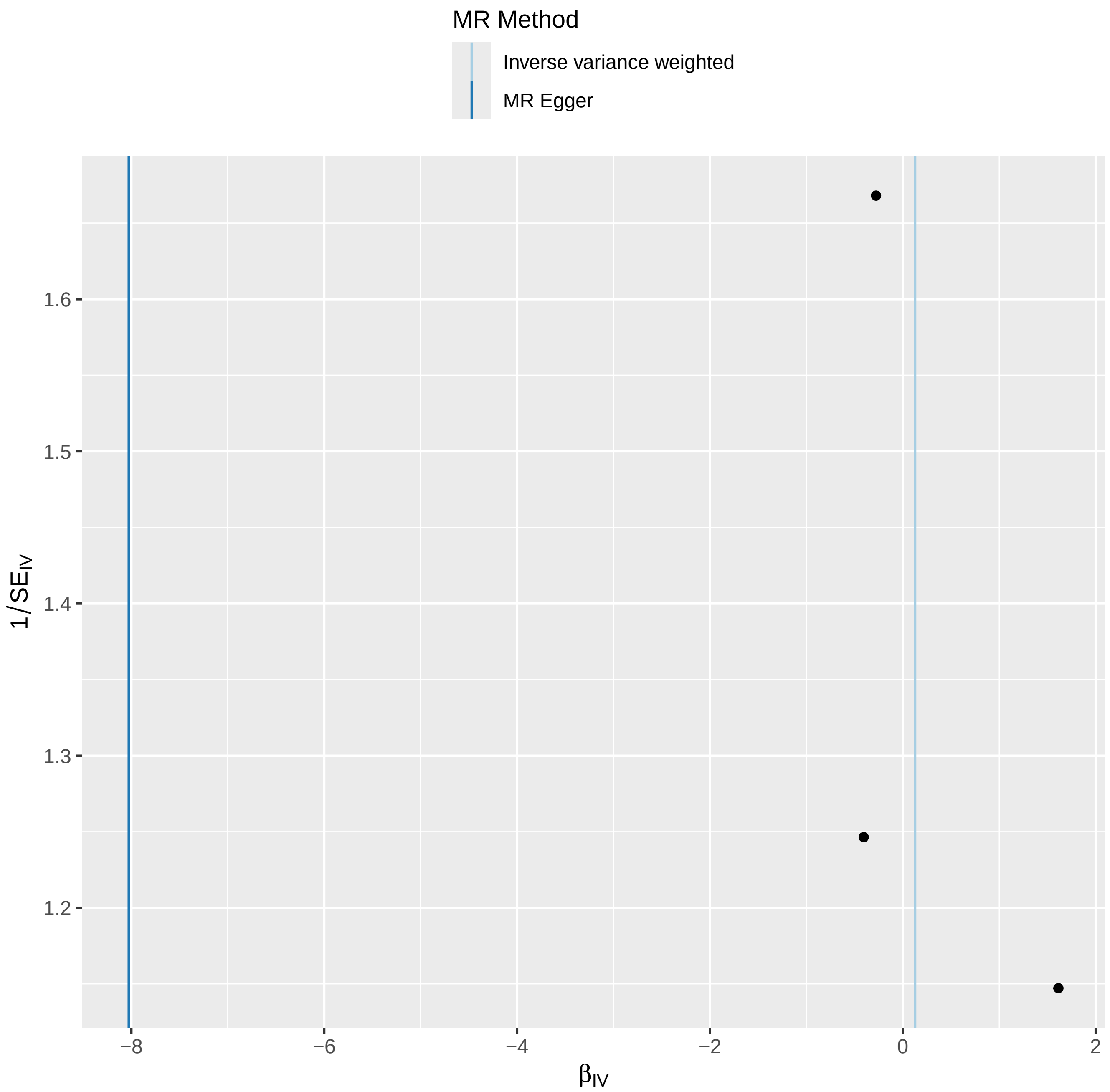
MR Method
Inverse variance weighted
MR Egger
1.6
1.5
1.4
1.3
1.2
1/SE$_{IV}$
−8
−6
−4
−2
0
2
β$_{IV}$

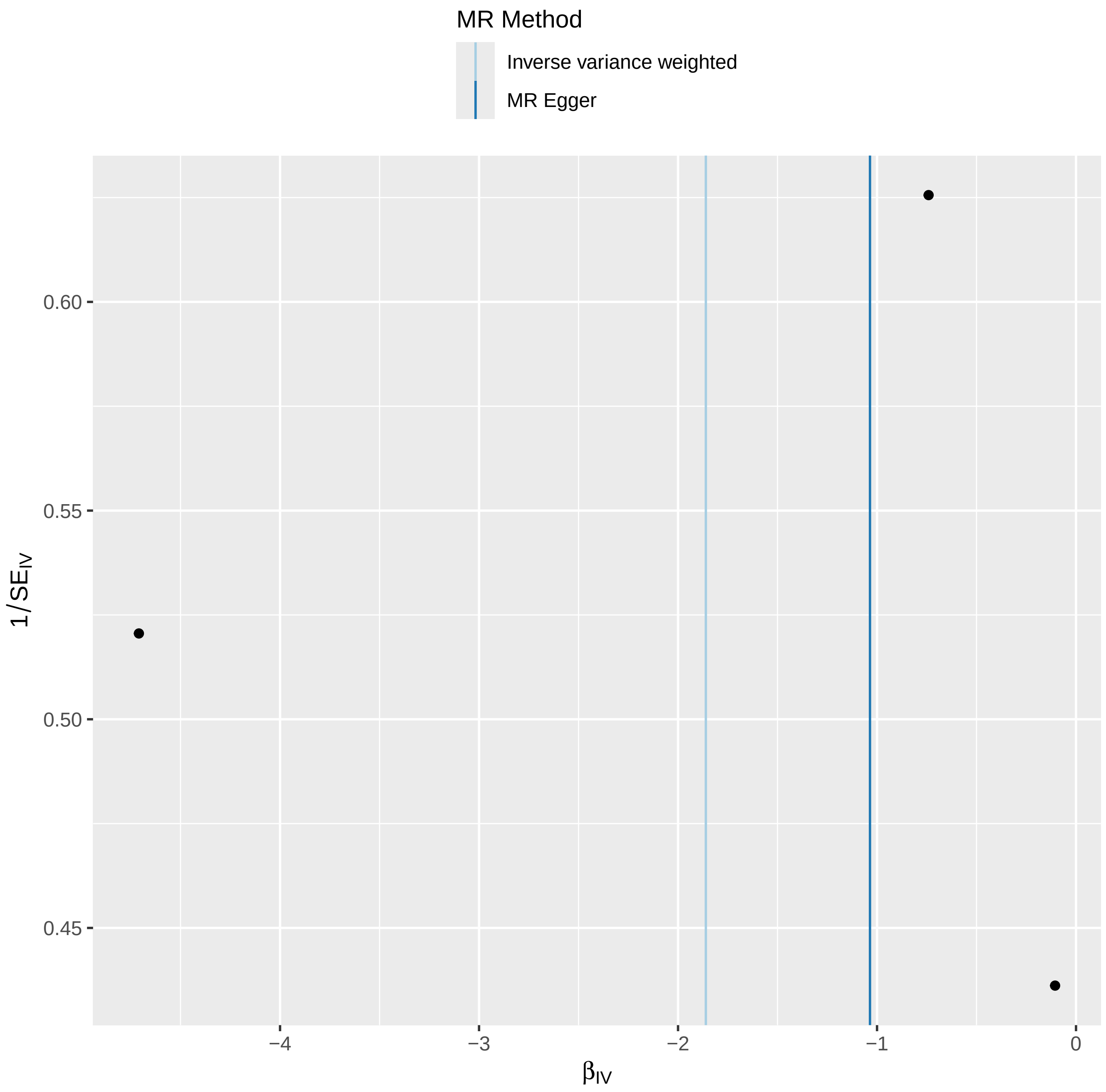
MR Method
Inverse variance weighted
MR Egger
0.60
0.55
0.50
0.45
1/SE_IV
−4
−3
−2
−1
0
β_IV
